# The rat striatum responds to nigro-striatal degeneration via the increased expression of proteins associated with growth and regeneration of neuronal circuitry

**DOI:** 10.1186/1477-5956-12-20

**Published:** 2014-04-28

**Authors:** Heidi R Fuller, Maica Llavero Hurtado, Thomas M Wishart, Monte A Gates

**Affiliations:** 1 Wolfson Centre for Inherited Neuromuscular Disease, RJAH Orthopaedic Hospital, Oswestry SY10 7AG, UK; 2 Keele University, Institute for Science and Technology in Medicine, Department of Life Sciences, Huxley Building, Keele ST5 5BG, UK; 3 Division of Neurobiology, The Roslin Institute and Royal (Dick) School of Veterinary Studies, University of Edinburgh, Easter Bush, Midlothian EH25 9RG, UK; 4 Euan MacDonald Centre for Motor Neuron Disease Research, University of Edinburgh, Edinburgh, UK

**Keywords:** Parkinson’s disease, Proteomics, Striatum, Nigro-striatal degeneration, Regeneration, iTRAQ, Guanine deaminase, GFAP, DARPP-32, Neurofilament

## Abstract

**Background:**

Idiopathic Parkinson’s disease is marked by degeneration of dopamine neurons projecting from the substantia nigra to the striatum. Although proteins expressed by the target striatum can positively affect the viability and growth of dopaminergic neurons, very little is known about the molecular response of the striatum as nigro-striatal denervation progresses. Here, iTRAQ labelling and MALDI TOF/TOF mass spectrometry have been used to quantitatively compare the striatal proteome of rats before, during, and after 6-OHDA induced dopamine denervation.

**Results:**

iTRAQ analysis revealed the differential expression of 50 proteins at 3 days, 26 proteins at 7 days, and 34 proteins at 14 days post-lesioning, compared to the unlesioned striatum. While the denervated striatum showed a reduced expression of proteins associated with the loss of dopaminergic input (e.g., TH and DARPP-32), there was an increased expression of proteins associated with regeneration and growth of neurites (e.g., GFAP). In particular, the expression of guanine deaminase (GDA, cypin) – a protein known to be involved in dendritic branching – was significantly increased in the striatum at 3, 7 and 14 days post-lesioning (a finding verified by immunohistochemistry).

**Conclusions:**

Together, these findings provide evidence to suggest that the response of the normal mammalian striatum to nigro-striatal denervation includes the increased expression of proteins that may have the capacity to facilitate repair and growth of neuronal circuitry.

## Background

Idiopathic Parkinson’s disease (PD) affects approximately 1-2% of the world’s over-60 population [1-4], making it the second most common neurodegenerative disorder after Alzheimer’s disease. The hallmark of Parkinson’s disease is progressive degeneration of dopamine neurons that project from the substantia nigra pars compacta (SNc) to the striatum (i.e., the nigro-striatal circuit) [5]. Over the past decade, great strides have been made towards detailing the abnormal molecular make up of SNc dopamine neurons, and the altered physiological properties of dopamine neurons that may contribute to their degeneration in the adult brain [6-8]. However, very little is known about the protein expression of cells in the striatum (the target of nigro-striatal dopamine neurons) during SNc degeneration. This is unfortunate due to the fact that immature SNc dopamine neurons depend on molecules produced by the striatum to grow appropriately during development, and rely on trophic support from proteins produced in the striatum to maintain their viability during and after development [9-14].

The present study was designed to use the animal model of Parkinson’s disease (i.e., the 6-OHDA medial forebrain bundle lesioned rat) to unveil how the proteome of the otherwise normal striatum changes in response to active degeneration of the nigro-striatal circuit. Here, relative levels of proteins expressed in the adult rat striatum as nigro-striatal denervation progresses have been detailed using iTRAQ labelling and MALDI TOF/TOF mass spectrometry. Quantitatively comparing the proteome of the target striatum before, during and after dopamine deafferentation has: (1) detailed the degradation of the dopaminergic afferents from the striatum; (2) highlighted a potential remedial response by glial cells in the striatum; and (3) revealed potential remedial responses in the striatum that could be directed at facilitating repair and / or viability of the nigro-striatal system. Because the 6-OHDA model of PD conveys (in the absence of other developmental events) what proteins the otherwise healthy adult striatum expresses when the survival and connectivity of nigro-striatal neurons is compromised, this has offered the unique opportunity to: unveil a proteome that may be tailored to SNc neuron viability, growth, and/or connectivity; and identify proteins whose expression may occur merely as a response to nigro-striatal degeneration itself (and, therefore, not be part of the aetiology of Parkinson’s disease).

## Results

**Figure 1:**
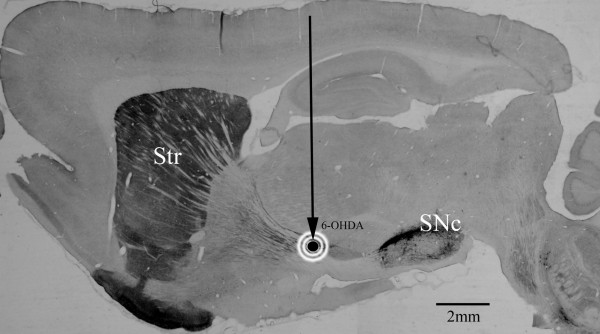
**A schematic diagram illustrating medial forebrain bundle lesioning used to detach the striatum from dopaminergic innervation.** A sagittal section from a non-lesioned rat brain, stained with an antibody to tyrosine hydroxylase (TH), highlights how dopaminergic cells residing in the substantia nigra pars compacta (SNc) extend long axonal fibers through the medial forebrain bundle on their way to innervating the striatum (Str). For lesioning, a small glass capillary (vertical arrow) was filled with 6-OHDA and lowered to a point between the SNc and Str (i.e., along the medial forebrain bundle). The 6-OHDA was slowly injected (black dot with halo surrounding) to produce a gradual degeneration of SNc dopamine neurons and their axonal innervation of the striatum. Rostral is left, dorsal is up. Scale bar = 2 mm.

The unilateral, medial forebrain bundle 6-OHDA lesion rat model of Parkinson’s disease was used to determine the relative levels of proteins expressed in the adult rat striatum before, during (3 days), and after (7 and 14 days) 6-OHDA induced dopamine denervation. Animals that were unlesioned (control), or 3 days, 7 days, or 14 days post 6-OHDA injection (lesioning), were either perfused with ice cold paraformaldehyde for histology studies (group 1: n = 4) or ice-cold sterile 0.9% sodium chloride (saline) for proteomic and western blot analysis (group 2: n = 4). A photomicrograph illustrating the location of the striatum to the unilateral medial forebrain bundle lesion and substantia nigra dopamine neurons is shown in Figure 1.

**Figure 2:**
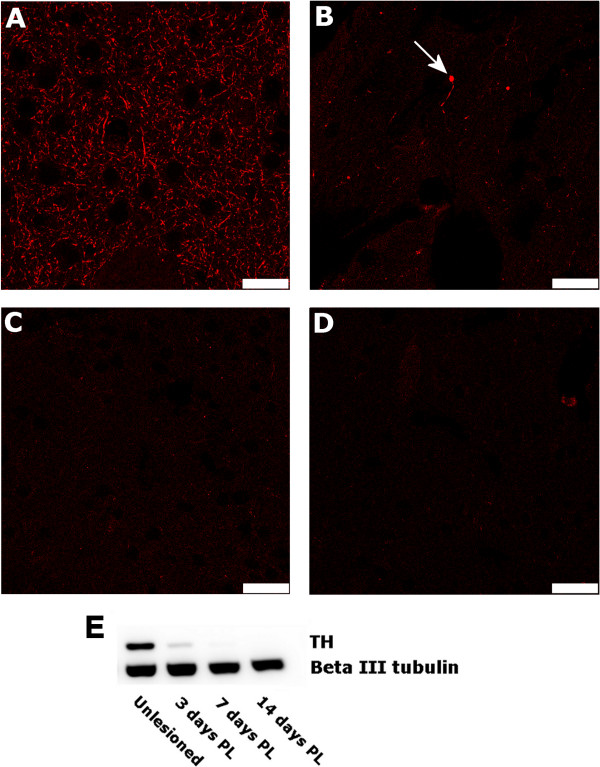
**Tyrosine hydroxylase (TH) immunoreactivity decreases by 3 days post-lesioning and is undetectable after 7 and 14 days post-lesioning.** Tyrosine hydroxylase (TH) immunoreactivity in coronal sections through the striatum of **(A)** unlesioned), **(B)** 3 day, **(C)** 7 day, and **(D)** 14 day post-lesioned animals, highlights the ongoing loss of dopaminergic input to the striatum at time points used for proteomic analysis. Though there is a noticeable staining of numerous TH + process in the striatum of the unlesioned brain **(A)**, only few swollen, varicose TH + process remain in the striatum at 3 days post 6-OHDA lesioning (arrow in **B**). As expected, there was no detectable staining of TH in the striatum after 7 days post lesioning **(C)**, and this remained unchanged at 14 days **(D)** post 6-OHDA injection to the medial forebrain bundle. Scale bars in A-D = 10 μm. Striatal protein extracts were subjected to SDS-PAGE and transferred to nitrocellulose by electroblotting blotting **(E)**. The blot was probed with an antibody against tyrosine hydroxylase – TH, with beta III tubulin as an internal loading control. PL = post 6-OHDA lesioning.

To verify and illustrate the level of deafferentation of the striatum from its dopaminergic input, we performed histological staining of coronal sections through unlesioned and 6-OHDA lesioned brains from animals prepared solely for histology analyses (group 1) using an antibody to tyrosine hydroxylase (Figure 2).

While TH staining in the unlesioned brain showed high levels of diffuse TH immunofluorescence throughout the striatum (Figure 2A), staining at 3 days post lesioning showed only sparse staining of TH + varicose fibers in the striatal grey matter (Figure 2B). By the 7^th^ day post lesioning there were no detectable TH + fibers within the striatal grey matter, indicating a complete deafferentation of the striatum from its nigral input by the end of the first week post 6-OHDA lesioning (Figure 2C). As expected, there was no change in the absence of TH staining in the striatum at 14 days post-lesioning, with the entire striatum continuing to be void of TH immunoreactivity (Figure 2D). This time course of loss of dopaminergic input to the striatum matched well the trend seen with western blot analysis of protein extracted from a second group of lesioned animals that were prepared specifically for proteomics and western blot analyses (group 2) (Figure 2E).

In order to determine which striatal proteins are changed in expression as the nuclei loses its nigral input, we performed iTRAQ labelling, followed by two-dimensional liquid chromatography and MALDI TOF/TOF mass spectrometry analysis using total protein extracted from the striatum of animals in group two at 3 days, 7 days, 14 days post-6-OHDA lesion and an unlesioned control. Using this approach to simultaneously compare the proteome of the unlesioned striatum, with the striatal proteome during nigro-striatal degeneration, a total of 1009 proteins were detected (with the stringent search settings, described in the materials and methods section; Additional file 1: Table S1 and Additional file 2: Table S2). To enable reliable quantification and robust statistical analysis of the dataset, proteins identified by fewer than 3 peptides were rejected, even though some proteins that are genuinely altered in expression may be excluded in this way. Small and low abundance proteins are more difficult to detect and if identified, are more likely to be represented by fewer peptides than larger and more abundant proteins. An example of this is tyrosine hydroxylase (TH) (hit 943, Additional file 2: Table S2), which was detected with just one peptide (despite the fact that the iTRAQ ratios of the peptide indicated substantial down-regulation at 3 days, 7 days and 14 days post-lesion, compared to the unlesioned sample), and was subsequently filtered from the dataset. The raw iTRAQ data have therefore been provided as supplementary tables to enable researchers to analyse the data as they wish, or to select other protein targets for further study.

After discarding any proteins that were identified using less than 3 peptides, 480 proteins remained in the data set. To establish which proteins were differentially expressed after lesioning, a +/− 1.25x fold change cut off was applied to the remaining 480 proteins. This level of (lesser or greater) fold change was chosen as it can be more easily validated using other biochemical / immunological methods such as quantitative western blotting [15]. This left 99 proteins at 3 days, 62 proteins at 7 days and 77 proteins at 14 days post-lesion, compared to the control unlesioned animals. Finally, statistical significance of the difference between individual peptide iTRAQ ratios for the remaining post-lesion and unlesioned proteins was determined using a Student’s one-tailed, pairwise t-test. Proteins that were significantly different in their expression levels (i.e., either up or down regulated) in the striatum among unlesioned and experimental groups (i.e. those with a p-value of less than 0.05) totaled 50 proteins at 3 days post-lesioning (Table 1), 26 proteins at 7 days post–lesioning (Table 2), and 34 proteins at 14 days post–lesioning (Table 3).

**Table 1:** Differentially expressed proteins at 3 days post-lesioning

**Protein name**	**Average iTRAQ ratio (3 days post-lesion/unlesioned)**	**p value**
**Down-regulated**		
Diacylglycerol kinase beta [9506535]	0.57 +/− 0.2 [4]	0.0192
Synaptophysin [6981622]	0.59 +/− 0.18 [5]	0.0035
Synaptic ras GTPase-activating protein p135 SynGAP [2935448]	0.62 +/− 0.1 [7]	2.92E-05
Ras-related protein Rab-3C [19424194]	0.67 +/− 0.13 [5]	0.0029
Protein NipSnap homolog 2 [62945328]	0.67 +/− 0.06 [3]	0.0066
Prohibitin [6679299]	0.67 +/− 0.26 [6]	0.0069
Succinyl-CoA synthetase alpha subunit [204356]	0.69 +/− 0.26 [5]	0.0486
RII-B-binding protein [557585]	0.69 +/− 0.12 [4]	0.0074
Type II cAMP-dependent protein kinase regulatory subunit [206671]	0.70 +/− 0.14 [16]	3.45E-08
A-kinase anchor protein 5 [19424156]	0.71 +/− 0.22 [5]	0.0276
RNA binding protein p37 AUF1 [9588102]	0.71 +/− 0.3 [6]	0.0325
Erythrocyte adducin subunit beta [10720378]	0.72 +/− 0.12 [6]	0.0010
Septin-5 [90577179]	0.72 +/− 0.09 [7]	0.0001
Reticulon-3 isoform A [57977297]	0.72 +/− 0.18 [5]	0.0151
Homer protein homolog 1 [13928988]	0.73 +/− 0.26 [6]	0.0484
Ras-related protein Rab-3C [13470090]	0.74 +/− 0.15 [5]	0.0093
V-type proton ATPase 116 kDa subunit a isoform 1 [139352]	0.75 +/− 0.22 [8]	0.0107
**Up-regulated**		
Tenascin-R precursor [6981668]	1.25 +/− 0.64 [8]	0.0545
Cytoplasmic dynein 1 light intermediate chain 1 [21955134]	1.25 +/− 0.12 [3]	0.0409
Rap1b [595280]	1.25 +/− 0.08 [4]	0.0048
G protein beta 1 subunit [984553]	1.27 +/− 0.36 [7]	0.0373
6-phosphofructokinase, muscle type [13929002]	1.27 +/− 0.47 [8]	0.0482
Transketolase [12018252]	1.27 +/− 0.32 [6]	0.0429
Guanine deaminase [148747414]	1.30 +/− 0.8 [14]	0.0453
Band 4.1-like protein 3 [16758808]	1.31 +/− 0.51 [10]	0.0145
Similar to 14-3-3 protein sigma [51260816]	1.34 +/− 0.3 [5]	0.0378
Eukaryotic initiation factor 4A-I isoform 1 [4503529]	1.34 +/− 0.25 [4]	0.0425
Reticulon-4 [13929188]	1.36 +/− 0.26 [6]	0.0080
Dihydropyrimidinase-related protein 4 [3122037]	1.38 +/− 0.35 [7]	0.0117
cGMP-dependent 3',5'-cyclic phosphodiesterase isoform 2 [13592021]	1.45 +/− 0.64 [6]	0.0544
Glutathione S-transferase P [25453420]	1.48 +/− 0.18 [4]	0.0067
Cytosol aminopeptidase [58865398]	1.53 +/− 0.4 [5]	0.0166
Glycyl-tRNA synthetase; [81889021]	1.54 +/− 0.24 [3]	0.0353
Chondroitin sulfate proteoglycan core protein 2 [21431624]	1.61 +/− 0.66 [4]	0.0585
Platelet-activating factor acetylhydrolase IB subunit alpha [7305363]	1.77 +/− 0.4 [3]	0.0409
NAD-dependent deacetylase sirtuin-2 [56605812]	1.78 +/− 0.51 [9]	0.0005
Carbonic anhydrase 2 [9506445]	1.79 +/− 0.54 [6]	0.0049
Myelin-oligodendrocyte glycoprotein precursor [158262022]	1.85 +/− 0.2 [5]	0.0005
Myelin proteolipid protein [13591880]	1.85 +/− 0.62 [17]	5.15E-06
Myelin basic protein [4454317]	1.86 +/− 0.59 [14]	1.84E-05
PMES-2C [55700833]	1.87 +/− 0.62 [4]	0.0307
Alpha-internexin [9506811]	1.87 +/− 0.54 [18]	1.5E-07
NF-M protein [205688]	1.90 +/− 0.53 [19]	8.36E-09
Myelin basic protein isoform 4 [70166270]	1.92 +/− 0.61 [15]	4.4E-06
Neurofilament light polypeptide [13929098]	1.92 +/− 0.64 [11]	4.56E-05
2',3'-cyclic-nucleotide 3'-phosphodiesterase [57977323]	1.92 +/− 0.59 [29]	5.17E-11
High molecular-weight neurofilament [2642598]	1.98 +/− 0.75 [8]	0.0006
Glycerol-3-phosphate dehydrogenase [NAD+], cytoplasmic [57527919]	1.99 +/− 0.32 [3]	0.0216
Glial fibrillary acidic protein [158186732]	3.44 +/− 2.58 [8]	0.0118

Proteins altered at 3 days post-lesion after strict filtering of the raw data. The significance of the difference between the average iTRAQ ratios for each protein at 3 days post-lesion, compared to the unlesioned control was calculated by analysing peptide ratios (see Additional file 2: Table S2) using a one-tailed, Student’s pairwise t-test. Only significant changes (i.e. those with p values of <0.05) are shown. Column headings refer to the following: protein name = given name in NCBInr database, [NCBI accession number]; Average iTRAQ ratio (3 days post-lesion/unlesioned) = average iTRAQ after data normalisation, +/− standard deviation from mean, [n] = number of peptides used for quantification.

**Table 2:** Differentially expressed proteins at 7 days post-lesioning

**Protein name**	**Average iTRAQ ratio** **(7 days post-lesion/unlesioned)**	**p value**
**Down-regulated**		
Protein phosphatase 1 regulatory subunit 1B (DARPP32) [61889054]	0.60 +/− 0.37 [6]	0.0321
Complexin-2 [5729783]	0.62 +/− 0.13 [4]	0.0057
Adh5 protein [54035294]	0.64 +/− 0.14 [3]	0.0335
cAMP and cAMP-inhibited cGMP 3',5'-cyclic phosphodiesterase 10A [13489075]	0.71 +/− 0.30 [9]	0.0018
NADH dehydrogenase [ubiquinone] iron-sulfur protein 4 [68341995]	0.73 +/− 0.14 [3]	0.0507
Microsomal Cytochrome B5 [6980893]	0.74 +/− 0.06 [3]	0.0115
Neuron-specific calcium-binding protein hippocalcin [6754240]	0.74 +/− 0.18 [8]	0.0042
**Up-regulated**		
Macrophage migration inhibitory factor [694108]	1.25 +/− 0.14 [3]	0.0537
Guanine nucleotide-binding protein G(z) subunit alpha [6980966]	1.27 +/− 0.10 [3]	0.0304
Clathrin-associated protein 17 [1809320]	1.30 +/− 0.14 [3]	0.0399
ApoE [202959]	1.32 +/− 0.20 [4]	0.0278
Brain acid soluble protein 1 [11560135]	1.33 +/− 0.71 [10]	0.0292
Gamma-enolase [26023949]	1.33 +/− 0.75 [16]	0.0165
Astrocytic phosphoprotein PEA-15 [4505705]	1.34 +/− 0.28 [4]	0.0481
Microtubule-associated protein RP/EB family member 3 [39930509]	1.39 +/− 0.65 [6]	0.0479
Platelet-activating factor acetylhydrolase IB subunit alpha [7305363]	1.39 +/− 0.09 [3]	0.0130
Beta-enolase [126723393]	1.40 +/− 0.96 [10]	0.0400
Synaptic vesicle glycoprotein 2B [17105360]	1.44 +/− 0.26 [6]	0.0042
Thiomorpholine-carboxylate dehydrogenase [16758840]	1.46 +/− 0.33 [4]	0.0312
Protein IMPACT [58866042]	1.49 +/− 0.12 [3]	0.0126
Sorting and assembly machinery component 50 homolog [51948454]	1.59 +/− 0.21 [3]	0.0233
Guanine deaminase [148747414]	1.59 +/− 1.04 [14]	0.0097
LanC-like protein 2 [62079109]	1.73 +/− 0.30 [4]	0.0094
Glial fibrillary acidic protein [158186732]	1.94 +/− 0.87 [8]	0.0047

Proteins altered at 7 days post-lesion after strict filtering of the raw data. The significance of the difference between the average iTRAQ ratios for each protein at 7 days post-lesion, compared to the unlesioned control was calculated by analysing peptide ratios (see Additional file 2: Table S2) using a one-tailed, Student’s pairwise t-test. Only significant changes (i.e. those with p values of <0.05) are shown. Column headings refer to the following: protein name = given name in NCBInr database, [NCBI accession number]; Average iTRAQ ratio (7 days post-lesion/unlesioned) = average iTRAQ after data normalisation, +/− standard deviation from mean, [n] = number of peptides used for quantification.

**Table 3:** Differentially expressed proteins at 14 days post-lesioning

**Protein name**	**Average iTRAQ ratio (14 days post-lesion/unlesioned)**	**p value**
**Down-regulated**		
Importin subunit beta-1 [8393610]	0.59 +/− 0.37 [5]	0.0407
Complexin-2 [5729783]	0.59 +/− 0.16 [4]	0.0068
Annexin A6 [130502086]	0.61 +/− 0.37 [7]	0.0263
Myelin basic protein isoform 4 [70166270]	0.67 +/− 0.21 [15]	3.27E-06
Protein phosphatase 1 regulatory subunit 1B (DARPP32) [61889054]	0.67 +/− 0.15 [6]	0.0019
Myelin basic protein [4454317]	0.68 +/− 0.24 [14]	2.76E-05
Plasma membrane calcium-transporting ATPase 3 [158138481]	0.69 +/− 0.14 [6]	0.0018
Cystatin C [227013]	0.71 +/− 0.14 [3]	0.0522
High molecular-weight neurofilament [2642598]	0.73 +/− 0.27 [8]	0.0074
Myelin-oligodendrocyte glycoprotein precursor [158262022]	0.73 +/− 0.08 [5]	0.0011
Myelin proteolipid protein [13591880]	0.74 +/− 0.24 [17]	0.0001
cAMP and cAMP-inhibited cGMP 3',5'-cyclic phosphodiesterase 10A [13489075]	0.74 +/− 0.24 [9]	0.0039
3',5'-cyclic nucleotide phosphodiesterase 1B [12083681]	0.75 +/− 0.26 [9]	0.0091
**Up-regulated**		
Mu Class Glutathione S-Transferase [442967]	1.25 +/− 0.22 [9]	0.0050
Chain A, Tetra-(5-Fluorotryptophanyl)-Glutathione Transferase [4388948]	1.25 +/− 0.22 [9]	0.0051
Chain A, Tetradeca-(3-Fluorotyrosyl)- Glutathione S-Transferase [5107744]	1.26 +/− 0.25 [7]	0.0182
NADH dehydrogenase (ubiquinone) 1 alpha subcomplex 10-like [32996721]	1.27 +/− 0.16 [4]	0.0244
V-type proton ATPase subunit B, brain isoform [17105370]	1.27 +/− 0.39 [9]	0.0250
Voltage dependent anion channel [4105605]	1.27 +/− 0.42 [10]	0.0248
Hyaluronan and proteoglycan link protein 1 [1346731]	1.27 +/− 0.32 [6]	0.0402
RNA binding protein p37 AUF1 [9588102]	1.29 +/− 0.48 [6]	0.0565
Clathryn light chain (LCA2) [203276]	1.29 +/− 0.20 [4]	0.0336
Synapsin-1 isoform b [160707907]	1.29 +/− 0.43 [18]	0.0020
Neural cell adhesion molecule long domain form [281037]	1.31 +/− 0.48 [6]	0.0516
Glutathione S-transferase Yb-3 [13592152]	1.31 +/− 0.28 [7]	0.0116
Synapsin-2 isoform 1 [77404242]	1.33 +/− 0.30 [12]	0.0007
ADP-ribosylation factor 5 [4502209]	1.36 +/− 0.16 [3]	0.0359
Guanine deaminase [148747414]	1.40 +/− 0.83 [14]	0.0126
Glial fibrillary acidic protein [158186732]	1.53 +/− 1.00 [8]	0.0321
Thiomorpholine-carboxylate dehydrogenase [16758840]	1.53 +/− 0.49 [4]	0.0592
LanC-like protein 2 [62079109]	1.54 +/− 0.43 [4]	0.0406
Synaptic vesicle glycoprotein 2B [17105360]	1.56 +/− 0.44 [6]	0.0088
Sorting and assembly machinery component 50 homolog [51948454]	1.94 +/− 0.58 [3]	0.0524

Proteins altered at 14 days post-lesion after strict filtering of the raw data. The significance of the difference between the average iTRAQ ratios for each protein at 14 days post-lesion, compared to the unlesioned control was calculated by analysing peptide ratios (see Additional file 2: Table S2) using a one-tailed, Student’s pairwise t-test. Only significant changes (i.e. those with p values of <0.05) are shown. Column headings refer to the following: protein name = given name in NCBInr database, [NCBI accession number]; Average iTRAQ ratio (14 days post-lesion/unlesioned) = average iTRAQ after data normalisation, +/− standard deviation from mean, [n] = number of peptides used for quantification.

To gain some insight into the molecular functions of the differentially expressed proteins, the filtered lists of differentially expressed proteins (Tables 1, 2 and 3) were analysed using Ingenuity Pathway Analysis software (IPA). Functional annotations that were assigned with a p value >0.05 and with fewer than three proteins were removed from the list. To highlight annotations that may reflect changes in neuronal connectivity following deafferentation from its nigral input, we further filtered the list to include only those that contained the following keywords in the annotation: axon, dendrite, dendritic, synapse, synaptic and neurite.

**Figure 3:**
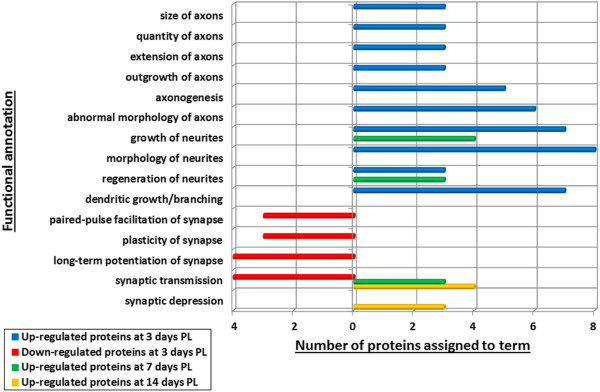
A graph illustrating the functional annotations that were assigned to the filtered lists of differentially expressed proteins using Ingenuity Pathway Analysis software (IPA).

**Figure 4:**
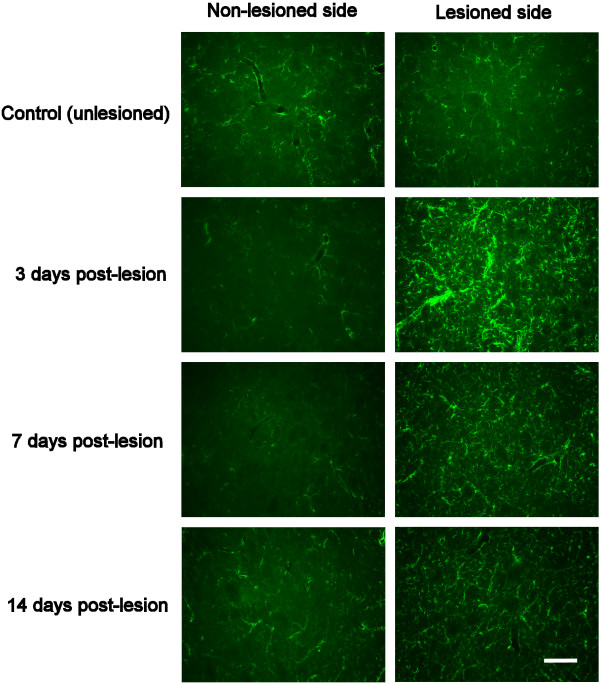
**Glial fibrillary acidic protein (GFAP) immunoreactivity is increased at 3, 7 and 14 days post-lesioning.** Glial fibrillary acidic protein (GFAP) immunoreactivity in coronal sections through the unlesioned and lesioned side of striatum from control (unlesioned), 3 days-, 7 days-, and 14 days-post-lesioned animals. At 3 days following 6-OHDA injection along the medial forebrain bundle, widespread and intense GFAP immunoreactivity can be seen in astrocytes throughout the entire striatal complex. Though this level of astrocyte reactivity had subsided somewhat by 7 days post lesioning, the level of GFAP expression in the striatum did not return to the levels seen in the unlesioned brain even after 14 days post lesioning. Scale bar for figures = 10 μm.

**Figure 5:**
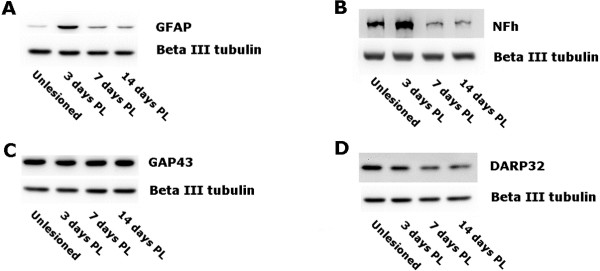
**Western blotting verifies the expression levels of several proteins that were identified by iTRAQ analysis.** Striatal protein extracts were subjected to SDS-PAGE and transferred to nitrocellulose either by electroblotting blotting **(B)**, or by diffusion blotting to produce two identical blots from one gel **(A, C,** and **D)**. Blots were probed with antibodies against **(A)** glial fibrillary acidic protein **-** GFAP, **(B)** neurofilament heavy polypeptide - NFh, **(C)** growth associated protein 43 - GAP43, or **(D)** dopamine- or cAMP- regulated neuronal phosphoprotein - DARPP-32, with beta III tubulin as an internal loading control in each case. PL = post 6-OHDA lesioning.

Several of the proteins that were up-regulated at 3 and 7 days post-lesion (almost 20% at 7 days) were assigned to the term “regeneration of neurites”, which is of potential interest when considering the healing, survival, growth of cells in the CNS (Figure 3). The most substantially increased protein in this category was glial fibrillary acidic protein (GFAP). GFAP “reactivity” among astrocytes is a widely accepted marker of a wound-healing response in the central nervous system [16]. To verify that GFAP was not increased because of a global wound-healing response to anesthetic and craniotomy, we compared GFAP expression levels in the non-lesioned side of the striatum from animals that had undergone 6-OHDA lesioning with animals that had not been subjected to operative procedures. Histological staining of coronal sections through the striatum of animals prepared for histology (group 1) confirmed that there was no significant difference in GFAP expression levels in the non-lesioned side of the 6-OHDA lesioned animals compared to those which had not been subjected to operative procedures (Figure 4). In both cases, sparse levels of GFAP expression were observed, with a majority of the immunopositive cells being perivascular astrocytes. Furthermore, a comparison of GFAP expression levels in the lesisoned and non-lesioned side of each 6-OHDA lesioned animal revealed a significant increase in GFAP expression in the lesioned side only. Intense GFAP + reactivity could be seen in astrocytes throughout the lesioned side of the striatum, with glial cells showing swollen GFAP + soma and processes. Although most evident during active nigro-striatal degeneration (i.e. at 3 days post-lesion), increased GFAP reactivity was still obvious at 7 and 14 days post-lesion, even though complete deafferentation of the striatum from its nigral input had been established for several days (Figure 2). The substantial increase in GFAP expression at 3 days post-lesioning (compared to the non-operated, control striatum), and a lessening of this reactivity in the subsequent 1–2 weeks matched the iTRAQ quantification trend well (Tables 1, 2 and 3), and was further confirmed by western blot analysis of the striatum taken from the second group of lesioned animals (group 2) (Figure 5A).

Over one fifth (22%) of the up-regulated proteins identified at 3 days post-lesion were assigned to the term “growth of neurites”. Among this category were neurofilament (NF) heavy and medium polypeptides (which were increased in comparison to controls by 1.98 x and 1.90 x, at 3 days post-lesioning respectively; Table 1). This was followed by a reduction of neurofilament heavy polypeptide to 0.73 x, compared to the control, by 14 days post lesioning (Table 3). This trend of increased neurofilament expression during ongoing nigro-striatal degeneration, followed by a reduction in its expression in the fully denervated striatum was verified by western blotting (Figure 5B). Interestingly, despite the western blot indictating a reduced expression of NF levels at 7 days post-lesioning (compared to the control), other proteins associated with “growth of neurites” were increased at this time point (Figure 3).

**Figure 6:**
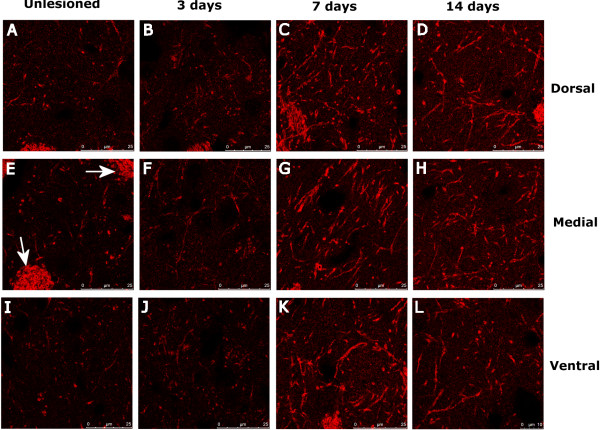
**Guanine deaminase (GDA) is increased in the striatum at 7 and 14 days post-lesioning.** Guanine deaminase (GDA) immunoreactivity in coronal sections through the striatum of **(A, E, **and **I)** unlesioned, **(B, F, and J)** 3 day, **(C, G, **and **K)** 7 day, and **(D, H, **and **L)** 14 day post-lesioned animals. High power, confocal images of the (top row) dorsal, (middle row) medial, and (bottom row) ventral portions of the striatum were taken to both see the fine staining by GDA immunoreactive fibers, and document the protein’s expression throughout the striatum. Though fiber bundles (“striations”) of the striatum were highly reactive for GDA in control and lesioned brains (arrows in **E**), there was only low levels of reactivity within the striatal grey matter of the unlesioned brain. Immunoreactivity for GDA in the brain 3 days post-lesioning**(B, F, **and **J)** suggests some change in the protein’s expression in the grey matter, however, an increase in GDA immunoreactivity was not notable until 7 days post 6-OHDA lesioning **(C, G, **and **K)**. At this stage, and at 14 days post-lesioning **(D, H, **and **L)**, numerous fibers, with long thick processes (suggestive of dendrites) were stained throughout the dorsal, medial, and ventral grey matter of the entire striatum. Scale bars for each figure are shown at the bottom right of each image.

Strikingly, all of the functional annotations that were assigned to the down-regulated proteins identified at 3 days post-lesion were related to the synapse (e.g. “synaptic transmission” and “synaptic plasticity”), even though the more general marker of the terminal end of axons, GAP43, showed little or no change in expression before, during and after nigro-striatal degeneration (Figure 5C) in western blotting. Proteins whose expression was down-regulated include synaptophysin, synGAP, NipSnap and homer 1 (Table 1). Interestingly, by 7 days post-lesioning (when deafferentation of the striatum becomes complete), this general trend of reduced expression of synaptic proteins appears to be reversed (Figure 3), when an increase in proteins related to “synaptic transmission” becomes evident. This increase is maintained at 14 days post-lesioning. An exception to this trend is the decrease in expression levels of the dopamine and cAMP regulated protein phosphatase 1, regulatory (inhibitor) subunit 1B (DARPP-32) at 7- and 14 days post-lesion (Tables 2 and 3). DARPP-32 is a protein long known to be associated with transmission at dopaminergic synapses, although its precise function is not fully understood [17,18]. The decreased expression of DARPP-32, as suggested by the iTRAQ data (Tables 2 and 3), was verified by western blotting (Figure 6D).

The increased expression of guanine deaminase (GDA; also known as cypin), at 3, 7 and 14 days post-lesion (Tables 1, 2 and 3) is of particular interest due to its known involvement in the survival of neurons and facilitation of dendritic branching [19,20]; a term also enriched in the IPA analysis at 3 days post-lesioning. Although the available anti-GDA antibody was not suitable for detection of GDA by western blotting, the trend of GDA expression in the striatum as deafferentation progressed was verified by immunohistochemical staining of sections of the striatum taken from lesioned animals processed for immunohistochemical analyses (group 1) (Figure 6). High power confocal micrographs from dorsal, medial, and ventral segments of the striatum show that while the white matter tracts of the striatum where intensely stained in both control and lesioned animals, there was a notable increase in staining intensity in fibrous processes in the grey matter of the striatum at 3 days. This increase in staining intensity was much more pronounced in sections from the striatum after 7 and 14 days post-lesioning, with large networks of fibrous GDA + staining seen throughout much of the striatal grey matter.

## Discussion

Over the past two decades, there have been attempts to take advantage of proteins (re)expressed by the striatum to improve the survival and / or growth of endogenous or transplanted SNc dopamine neurons. Studies in both rat and primate models of PD, for example, have shown that co-grafting embryonic striatal tissue (or cells) with immature ventral mesencephalic (VM) cells significantly increases the survival and growth of transplanted dopamine neurons [11,21,22]. Perhaps more interestingly are observations from the rodent model of PD showing that the denervated adult striatum is itself capable of promoting the growth of immature dopaminergic axons *in vivo*[23]. Although such studies highlight the (therapeutic) potential of both developmentally regulated and adult striatal proteins to enhance the growth and viability of nigro-striatal dopamine neurons, very little is known about the proteome of the striatum in both the normal and lesioned brain.

Here, the rodent model of Parkinson’s disease (i.e., 6-OHDA lesioning of adult rats) has been used to identify and quantify proteins expressed in the otherwise normal mammalian striatum during ongoing detachment from SNc dopaminergic neurons. Comparing the proteome of the striatum before, during, and after dopamine denervation has detailed not only proteins related to the ongoing degeneration of nigro-striatal input, but also potential regenerative responses by cells in the striatum indicative of wound healing, and growth / plasticity. Detailing the proteome in this way has not only identified the molecular response of the otherwise normal adult brain to the challenge of preserving the viability and connectivity of the nigro-striatal circuit, but also offers a base line for comparison (in future studies) with the Parkinsonian brain to determine if the target striatum in humans responds similarly to this same challenge.

### Changes in protein expression related to the degeneration of nigro-striatal dopamine neurons

Immunohistochemistry for TH, revealed the loss of dopaminergic input to the striatum at 3, 7, and 14 days post 6-OHDA lesioning (Figures 1 and 2). Accompanying this, iTRAQ analysis of the striatal proteome at 3 days post lesioning suggests that the expression of certain synaptic proteins (e.g., synaptophysin, synaptic ras GTPase-activating protein) may also be affected by this denervation (Table 1; Figure 3). Western blot analysis of DARPP-32, a protein long known to be associated with transmission at dopaminergic synapses [17,18] verified iTRAQ analysis showing that when cells within the striatum are completely detached from their nigro-striatal input (i.e., at 7 and 14 days post 6-OHDA lesioning), there is a corresponding reduction in dopamine synapse-related proteins. These alterations in the proteome might be expected when considering that as destruction of afferent SNc dopamine neurons proceeds, there is a concomitant loss of proteins related to dopaminergic connectivity (i.e., synapses) within the striatum. Such findings support previous assertions that nigro-striatal denervation negatively affects DARPP-32 expression [24], though this contrasts with other (indirect) estimations suggesting that denervation may not affect striatal DARPP-32 levels [25]. It will be important to clarify the effect of dopamine denervation on DARPP-32 expression in the striatum of human PD sufferers, as inactivation of DARPP-32 in mice can significantly reduce the possibility of l-dopa induced dyskinesias [26].

Interestingly, there appears to be a significant increase in proteins associated with axonal reorganization (e.g. neurofilament polypeptides) during ongoing dopamine denervation of the striatum (i.e., at 3 days post lesioning; Table 1 and Figure 3), followed by a reduction (in comparison to controls) after nigro-striatal degeneration is complete at 7 and 14 days post 6-OHDA injection. This seems confounding when considering there is a substantial loss of dopaminergic fibres from the SNc at 3 days post lesioning. An initial increase in cytoskeletal proteins could represent either swelling of nigro-striatal axons during anterior-grade degeneration (as seen with the varicose TH staining at 3 days post lesioning, Figure 2), or an up-regulation in the cytoskeleton of other intact afferent axons (e.g., cortico-striatal axons). It’s unlikely that this initial increase in cytoskeletal proteins is related to swelling of degenerating nigro-striatal afferents at 3 days post lesioning, as there is a near complete loss of dopaminergic fibers by this stage of degeneration. Because the expression profile of neurofilament proteins is first lightly reduced at 7 days, then more significantly reduced at 14 days (in comparison to the intact striatum; see Additional file 1: Table S1-Additional file 2: Table S2, Table 3, and Figure 5B), this seems to indicate that if initial increases in the cytoskeletal proteins in the striatum is related to plasticity or new growth, then this is a very rapid process. This seems possible when considering that the remaining striatal afferents (e.g., cortico-striatal projections) will have synapses on the same cells that have lost their dopaminergic afferent input.

### Changes in proteins related to growth and regeneration of neurites

The possibility that there is a heightened period of plasticity / wound healing / regeneration in the striatum is supported by IPA bioinformatics analysis which indicates that several proteins found to be up-regulated in response to lesioning (e.g., neural cell adhesion molecule NCAM; Table 3) are categorised by “regeneration of neurites”. In fact, the response of cells in the striatum to the loss of dopaminergic input appears to be very similar to that seen with wound healing after traumatic injury [16]. This response is important to clarify as such proteins in the adult brain can have a dramatic effect on cell survival and the potential for neurons (including transplanted cells) to regenerate into a site where these proteins are expressed [27]. Though the striatum receives no direct physical trauma during 6-OHDA lesioning of the medial forebrain bundle (Figure 1), astrocytes become highly reactive by 3 days post lesioning, increasing their level of GFAP expression at a time when dopamine denervation is ongoing (Figure 4). This reactivity in the striatum subsides somewhat by the 7^th^ day post lesioning (when dopamine denervation is complete), but levels of GFAP expression remain higher than that seen in the intact striatum. This astrocyte reactivity appears to be accompanied by an increase in the expression of other astrocyte-related proteins (e.g., tenascin, CSPG, and hyaluronan; Tables 1, 2 and 3) that are known to be associated with wound healing in the brain [16]. Such proteins are commonly expressed as part of the extracellular matrix that helps stabilise perineuronal nets [28], and facilitate cell and neurite adhesion [29,30]. Because astrocytes are known to play a major role in buffering the environment of neuronal cells (including; modulating the presence of neurotransmitters and potassium, providing a reservoir of glycogen for increased neuronal activity, and the potential to perform the dual role of helping in the formation and elimination of synapses) [31], it will be important to determine their level of response in Parkinson’s disease. It may well be that astrocytes can provide a means by which the viability of endogenous nigro-striatal neurons could be increased, or (through the expression of extracellular matrix) be useful in providing a milieu that increases the possibility to reform nigro-striatal connectivity (via cell transplantation) in the adult brain.

### Changes in proteins related to dendritic branching

Here we have focused on the increased expression of the enzyme guanine deaminase (GDA, also known as cypin) throughout all phases of dopamine denervation of the striatum, due to its expression profile and the fact that the protein’s expression has been shown to dramatically increase dendritic arborisation in the CNS [20,32-35]. Also, guanine deaminase (an enzyme that converts guanine to xanthine) is known to be involved in purine metabolism, the end product of which is urate. Urate, itself, is thought to be neuroprotective in the ageing brain, as an increased expression of the protein is suggestive of a lower risk of Parkinson’s disease [36]. In addition to this, there is evidence to suggest that purines, released by dying or injured cells, may induce brain repair via astrocytic activity [19].

Here, immunohistochemistry revealed a substantial increase in GDA expression during and after striatal denervation. Such an increase in protein expression could represent an effort by projection neurons within the striatum to expand their dendritic arbors to facilitate the formation of new connections from other afferents or intra-striatal input. With the expression of such proteins (that may promote the growth of axons and dendrites) in the striatum after denervation on the rise, and myelin related proteins being reduced (which may negatively affect neuronal growth/regeneration) [37], there remains the real possibility that the denervated striatum is primed for plasticity. Though this signaling may benefit efforts at restoring connectivity with the striatum (e.g., through cell transplantation), it may greatly complicate the balance of excitatory and inhibitory input to the direct and indirect pathway (respectively) that dopaminergic innervation once modulated.

### Changes in synaptic functions

The iTRAQ analysis here, indicates that the normal striatum responds to denervation from nigro-striatal neurons by firstly decreasing the expression of synaptic proteins (Table 1 and Figure 3) followed by an increase in synapse-related proteins at 7 and 14 days (Tables 2 and 3 and Figure 3). In particular, synaptic vesicle protein 2B, and synapsin 1 and 2 appear to up-regulated in response to striatal denervation at 7 and 14 days post lesioning (Tables 2 and 3). Synapsins have long been known to regulate synaptic depression and to increase transmission at synaptic sites [38], and synaptic vesicle protein 2B (which is associated with high rates of neurotransmitter release at the synapse) is known to be absent from dopaminergic neurons [39]. Such up-regulation of synapse related proteins is surprising when considering that a vast network would have been abolished by lesioning of dopaminergic input, and indicates altered activity among other striatal afferent projections.

### Changes in proteins implicated in Parkinson’s disease

A useful aspect of looking at the proteome of the otherwise normal mammalian striatum as it is artificially detached from its dopaminergic input (i.e., via 6-OHDA lesioning of laboratory rats), is that it allows for the identification of proteins that may be a reaction to the denervation event alone, and, hence, not part of the aetiology of Parkinson’s disease. For example, iTRAQ analysis here suggest that NAD-dependent deacetylase siturin-2 rises (at 3 days post 6-OHDA lesioning) in response to dopamine denervation of the striatum (Table 1). This is interesting when considering that SIRT1 and SIRT2 have both been implicated in the protection of neurons against age related disease [40], and that SIRT2 may enhance alph-synuclein mediated toxicity [41]. In addition, NADH dehydrogenase (ubiquinone) – an enzyme catalyzing the first step in the mitochondrial electron transport chain – also appears to be decreased early on in the striatum in response to 6-OHDA induced deafferentation from its dopaminergic input (at 7 days post lesioning; Table 2). Though mitochondrial dysfunction has long been linked to oxidative stress and cell death in PD [42], the data presented here suggests reduced expression of NADH dehydrogenase in the striatum is likely to be a response to SNc dopamine neuron destruction, and not part of the primary cause of Parkinson’s disease. However, an increase in NADH dehydrogenase (ubiquinone) expression after more prolonged deafferentation of the striatum (as is suggested here from the striatal proteome at 14 days post 6-OHDA lesioning; Table 3) could be of interest due to the fact that its increased expression may represent a dramatic rise in the generation of ATP (energy) by cells within the denervated striatum. This could be important if there is a process of extensive wound healing and plasticity ongoing in the striatum (as mentioned above). Finally, the iTRAQ analysis here suggests that there is an increase in the expression of the voltage-dependent anion channel 1 (VDAC-1) – a protein identified as linking parkin to defective mitochondria in PD [43,44] – after complete dopamine deafferentation of the striatum at 14 days post 6-OHDA lesioning (Table 3). Similarly, the expression of SEPT5 – which is linked to parkin and proteosomal degradation [45] - is reduced in the striatum at 3 days post-6-OHDA lesioning (Table 1). Such findings imply that some of the altered protein expression in the striatum may be reactionary and not part of the underlying cause/aetiology of dopamine neuron degeneration seen in PD.

## Conclusions

Here, we have detailed changes in the proteome of the rat striatum as it becomes detached from its dopaminergic input via 6-OHDA lesioning of the medial forebrain bundle. This analysis of changing protein expression in the striatum was conducted over a period of time when it is known that the striatum loses innervation from dopaminergic input following 6-OHDA lesioning [46]. This analysis not only provides a detailed description of the changing proteome of the striatum directly related to the destruction of nigro-striatal neurons themselves (e.g., reduced TH and neurofilament expression), but has also revealed a potential regenerative growth response (e.g., increased GFAP expression and GDA/cypin expression) by cells within the target nuclei. In the future, it will be important to detail the changing protein profile of the striata of human PD sufferers, as the time course for degeneration of the circuitry is more drawn out in the case of human PD and may offer more details of compensatory changes not seen in the rodent model. However, comparing the responses of the rat striatum in the 6-OHDA model of PD with the protein expression seen in human Parkinson’s disease may allow for the discovery of proteins that the human striata fails to express when challenged with the loss of nigro-striatal dopamine neurons, or detail proteins that are not part of the aetiology of the disease itself (and merely a response to, or representation of, dopamine cell degeneration). In addition, identifying the function of molecules expressed by the otherwise normal striatum to a denervating event may offer clues to proteins or cellular pathways that could be manipulated to enhance the survival and/or connectivity of immature nigro-striatal dopamine neurons transplanted to Parkinson’s sufferers as a part of a cell replacement strategy.

## Methods

All in vivo procedures were approved by the Animal Welfare & Ethical Review Body (AWERB) at Keele University, and were carried out under the licensed authority of the UK Home Office (PPL40/3556). All adult Sprague Dawley rats were housed in a 12-12 h light–dark environment, and given free access to food and water throughout the study.

### Medial forebrain bundle 6-OHDA lesioning

Adult, female rats (~230 g in weight) were anesthetised using isoflurane gas, and subsequently secured in a stereotaxic frame. With the incisor bar set at −4.5 mm from horizontal, a midline incision was made along the scalp and a single small hole drilled in the skull (on the right side only) 4.0 mm anterior to the bregma suture, and 1.3 mm lateral to the midline. A fine glass capillary, filled with a 30 mM 6-OHDA (Sigma)/0.03% ascorbic acid solution, was lowered 7.0 mm deep to the cortical dura, and 3 μl of the solution injected (using a Nanoject injector; Drummond) into the right medial forebrain bundle (MFB) over a 3 minute period of time. The needle was left in situ for an additional 2 minutes, and then subsequently removed, and the animal sutured and placed in a warmed cage.

Animals that were either unlesioned (control), or 3 days, 7 days, or 14 days post 6-OHDA injection (lesioning), were given an overdose of pentobarbitone anesthetic (via i.p. injection) and cardiac perfused with either 4.0% solution of paraformaldehyde (PFA) in Tris-buffered saline (TBS) for histology studies (group 1: n = 4) or ice-cold sterile 0.9% sodium chloride (saline) for proteomic and western blot analysis (group 2: n = 4).

### Tissue / protein extraction

A portion of the striatum (i.e., the central core of the striatum, cut from a ~500 μm coronal slice through the basal ganglia at a point where the anterior commissure lay just ventro-lateral to the lateral ventricles) was dissected and placed in fresh sterile saline. Subsequently, the saline was aspirated off and the striatal samples homogenised in 4 volumes (w/v) of 6 M Urea, 2 M thiourea, 2% CHAPS and 0.5% SDS using a pellet pestle (30 strokes with the pestle, left on ice for 10 minutes, followed by another 30 strokes with the pestle). The extracts were sonicated briefly and left on ice for 10 minutes, followed by centrifugation at 13,000 x g for 10 minutes at 4°C to pellet any insoluble material. For mass spectrometry analysis, an aliquot of the extracted proteins from each rat was precipitated in 6 volumes of ice-cold acetone overnight at −20°C. The remaining extracts were stored at −80°C for validation studies. The acetone precipitates were pelleted by centrifugation at 13,000 x g for 10 minutes at 4°C and the supernatant was carefully removed and discarded. The pellets were resuspended in 6 M Urea in 50 mM TEAB. The protein concentration in each sample was determined using a Bradford protein assay.

### Sample preparation for mass spectrometry analysis

Reduction, alkylation and digestion steps were performed using the reagents and according to the recommendations detailed in the iTRAQ labelling kit (AB Sciex). The extracts were diluted with 50 mM TEAB (so that the urea concentration was less than 1 M) before the addition of trypsin and overnight incubation at 37°C. The digests were then dried down in a vacuum centrifuge and iTRAQ labelling carried out according to instructions outlined in the iTRAQ labelling kit. The iTRAQ tags were assigned to samples as follows: 114-control, 115–3 days, 116–7 days and 117–14 days post lesion. Each tag was incubated with 85 μg of total protein (as determined by a Bradford protein assay).

iTRAQ-labeled peptides were pooled and made up to a total volume of 2.4 mls in SCX buffer A (10 mM phosphate, pH 3.0 in 20% acetonitrile (Romil, UK)). The pooled peptides (2.4 mls) were then separated by strong cation-exchange chromatography (SCX) using a polysulfoethyl A column, 300A, 5 uM (PolyLC)) at a flow rate of 400 ul/minute. Following sample injection, the column was washed with SCX buffer A until the baseline returned. The gradient was run as follows: 50% SCX buffer B (10 mM phosphate, 1 M NaCl, pH 3.0 in 20% acetonitrile) over 25 minutes followed by a ramp up from 50% to 100% SCX buffer B over 5 minutes. The column was then washed in 100% SCX buffer B for 5 minutes before equilibrating for 10 minutes with SCX buffer A. Fractions were collected (400 ul) during the elution period and dried down completely in a vacuum centrifuge.

### Protein identification and quantification by mass spectrometry

The iTRAQ tryptic peptide fractions were each resuspended in 35 μl of RP buffer A (2% acetonitrile, 0.05% TFA in water (Sigma Chromasolv plus). Prior to mass spectrometry analysis, fractions were first separated by liquid chromatography (Dionex Ultimate 3000) on a Pepmap C18 column, 75 μm x 15 cm (LC Packings) at a flow rate of 0.356 μl/minute. Fractions were injected by full-loop injection (20 μl) and the order of loading was randomized to minimise effects from carry-over. The eluants used were: A. (0.5 TFA in 2% ACN in water) and B. (0.05% TFA in 90% acetonitrile in water). The gradient was run as follows: 10 minutes isocratic pre-run at 100% A, followed by a linear gradient from 0-30% B over 100 minutes, followed by another linear gradient from 30%-60% over 35 minutes. The column was then washed in 100% B for a further 10 minutes, before a final equilibration step in 100% A for 10 minutes. During the elution gradient, fractions were spotted at 10 second intervals using a Probot (LC Packings) with α-cyano-4-hydroxycinnamic acid (CHCA) at 3 mg/ml (70% MeCN, 0.1% TFA) at a flow rate of 1.2 μl/min.

Both MS and MS/MS analysis was performed on the fractionated peptides using an Applied Biosystems 4800 MALDI TOF/TOF mass spectrometer. The mass spectrometer was operated under control of 4000 Series Explorer v3.5.2 software (Applied Biosystems). A total of 1000 shots per MS spectrum (no stop conditions) and 2500 shots per MS/MS spectrum (no stop conditions) were acquired. The following MS/MS acquisition settings were used: 2KV operating mode with CID on and precursor mass window resolution set to 300.00 (FWHM). Peak lists of MS and MS/MS spectra were generated using 4000 Series Explorer v3.5.2 software and the following parameters were used after selective labelling of monoisotopic mass peaks: MS peak lists: S/N threshold 10, Savitzky Golay smoothing (3 points across peak (FWHM)), no baseline correction, MS/MS peak lists: S/N threshold 14; smoothing algorithm: Savitzky Golay, smoothing (7 points across peak (FWHM)).

An automated database search was run using GPS Explorer v3.6 (AB Sciex). MASCOT was used as the search engine to search the NCBI non-redundant database using the following search parameters: precursor ion mass tolerance of 150 ppm, MS/MS fragment ion mass tolerance of 0.3 Da, iTRAQ fragment ion mass tolerance of 0.2 Da, the taxonomy was selected as rats, oxidation of methionine residues were allowed as variable modifications and N-term (iTRAQ), lysine (iTRAQ) and MMTS modification of cysteine residues were set as fixed modifications. The version of the NCBI database used was: Tue Oct 04, 2011, and the number of sequences in the rat subset was: 19185. Quantification of the iTRAQ peptides was performed using the GPS Explorer v3.6 software. The most stringent search settings were used for protein identification (peptide rank 1 and total ion score confidence intervals of at least 95%). iTRAQ Ratios were normalized using the following formula: iTRAQ Ratio = Ratio/(median iTRAQ Ratio of all found pairs) that was applied in GPS Explorer software.

In order to determine which striatal proteins are changed in expression levels following 6-OHDA lesioning, all proteins detected were subjected to strict filtering. Proteins identified from fewer than 3 peptides were deemed unreliable for quantification, and were excluded from analysis. Those with an average post-lesion/control ratio of less than 1.25 or greater than 0.75 were also removed from the list. Lastly, a statistical analysis of potential significant difference between individual peptide iTRAQ ratios for the remaining 3, 7 and 14 days post-lesion and unlesioned proteins was determined using a Student’s pairwise t-test.

### In silico protein network analysis

To obtain further insight into potential cellular pathways that may be modified as a result of protein changes identified in our experiments, the Ingenuity Pathways Analysis (IPA) application (Ingenuity Systems) was used as previously described [47]. IPA dynamically generates networks of gene, protein, small molecule, drug, and disease associations on the basis of “hand-curated” data held in a proprietary database. More than 90% of the information in this database is “expert-curated” and is drawn from the full text of peer reviewed journals. Less than 10% of interactions have been identified by techniques such as natural language processing. In the current analysis candidate interactions were limited to experimentally observed interactions only, but could be drawn from any source in the IPA database. Networks generated by IPA were limited in this study to ten networks comprising a maximum of 35 members per network. To enhance the explorative interpretation of data, networks are ranked according to a score calculated via a right tailed Fisher’s exact test. This test outputs a value that takes into account the original input gene or proteins of interest and the size of the network generated. Further information on the computational methods implemented in IPA can be obtained from Ingenuity Systems (http://www.ingenuity.com/).

### SDS-polyacrylamide gel electrophoresis and western blotting

Protein extracts were prepared by boiling in SDS loading buffer (2% sodium dodecyl sulphate- SDS, 5% 2-mercaptoethanol, 62.5 mM Tris–HCl, pH 6.8), for 2 min. Proteins were subjected to SDS-PAGE using 12% polyacrylamide gels and transferred to nitrocellulose membranes by western blotting. After blocking non-specific sites with 4% powdered milk solution, membranes were incubated with primary antibodies: 1:3000 mouse anti-TH (tyrosine hydroxylase; Milipore); 1:2000 mouse anti-NFh (neurofilament heavy chain; Sigma); 1:2000 rabbit anti-GFAP (glial fibrillary acidic protein; Dakko); 1:300 rabbit anti-GAP43 (growth associated protein 43; Abcam); 1:20000 mouse anti-DARP-32 (Milipore) 1:1000 mouse anti-beta III tubulin (Covance), diluted in dilution buffer (PBS, 1% fetal bovine serum, 1% horse serum and 0.1% BSA). Antibody reacting bands were visualized by development with either peroxidase-labeled goat anti-mouse Ig, or peroxidise-labeled swine anti-rabbit Ig (1 μg/ml in dilution buffer) and a chemiluminescent detection system (West Pico, Pierce).

### Histology

After removal of the brain specimens, and incubation in PFA overnight, specimens were transferred to a 30% sucrose solution in TBS, and allowed to fully sink in the solution at room temperature (RT). Subsequently, specimens were placed on a sliding, freezing microtome, and 40 μm coronal sections cut through the entire striatum. Sections were stored in 24-well plates containing TBS with sodium azide. For immunohistochemistry, sections were rinsed 3 times in TBS, and incubated in a blocking solution containing 3% goat serum/TBS for 1 hour at RT. The blocking solution was subsequently replaced with one of the following primary antibodies in TBS containing 1% goat serum, and left to incubate overnight at RT: a 1:1000 dilution of mouse anti-TH antibody (tyrosine hydroxylase; Chemicon), 1:500 rabbit anti-GFAP (glial fibrillary acidic protein; Dakko), or 1:100 rabbit anti GDA (guanine deaminase; Tebu-Bio). The following day, sections were rinsed 3 times in TBS followed by a 2 hour incubation in a 1:200 dilution of an appropriate fluorescent secondary (Molecular Probes). Subsequently, sections were rinsed 3 times in TBS, mounted onto slides, and coversliped using Vectashield Hardmount mounting media (Vector Labs).

### Microscopy

Low power images were obtained via the use of a Hammamatsu digital camera attached to a Nikkon Eclipse 80i fluorescence microscope. For detail of fine neuritic processes, high power images were obtained using a Leica SP5 confocal microscope imaging system via a 63x oil emersion objective.

## Abbreviations

iTRAQ: Isobaric tags for relative and absolute quantification; MALDI TOF/TOF: Matrix-assisted laser desorption ionisation time-of-flight mass spectrometry; 6-OHDA: 6-hydroxydopamine; TH: Tyrosine hydroxylase; DARPP-32: Dopamine and cAMP regulated protein phosphatase 1, regulatory (inhibitor) subunit 1B; GFAP: Glial fibrillary acidic protein; GDA: Guanine deaminase; PD: Parkinson’s disease; SNc: Substantia nigra pars compacta; IPA: Ingenuity pathway analysis software; CNS: Central nervous system; NF: Neurofilament polypeptide.

## Competing interests

The authors declare that they have no competing interests.

## Authors’ contributions

HRF participated in the study design, conducted the proteomics and western blotting experiments, and coordinated and helped to draft the manuscript. MLH and TMW conducted bioinformatics analysis and helped to draft the manuscript. MAG conceived of the study, participated in its design, conducted tissue collection and immunohistochemistry experiments, and coordinated and helped to draft the manuscript. All authors read and approved the final manuscript.

## Supplementary Material

Additional files 1: Table S1 A list of all the proteins that were identified by MALDI TOF/TOF mass spectrometry with total ion score confidence intervals of greater than 95% and peptide rank of 1. For each protein identified, the corresponding NCBInr Accession number is shown, together with the total ion score confidence interval (C.I).% and the number of peptides used for the identification. iTRAQ ratios, iTRAQ ratio standard deviation and number of peptides used for the iTRAQ-quantification are also shown for each protein. Proteins highlighted in bold font are those that met the significance criteria for inclusion into the tables in the manuscript (i.e. more than 2 peptides, +/- 1.25x fold change and a p-vaule of less than 0.05. The iTRAQ tags were assigned as follows: 114 control (unlesioned), 115 - 3 days-post lesion, 116 - 7 days post-lesion, 117 - 14 days post-lesion. Click here for file

Additional files 2: Table S2 A protein – peptide summary of all the proteins that were identified by MALDI TOF/TOF mass spectrometry with total ion score confidence intervals of greater than 95% and peptide rank of 1. For each protein identified (Table S1), the individual peptide sequences, individual peptide ion score confidence intervals (C.I).% and individual peptide iTRAQ ratios are given. The iTRAQ tags were assigned as follows: 114 control (unlesioned), 115 - 3 days-post lesion, 116 - 7 days post-lesion, 117 - 14 days post-lesion. Click here for file
