# Supplementary material for: The rat striatum responds to nigro-striatal degeneration via the increased expression of proteins associated with growth and regeneration of neuronal circuitry
Source: Proteome Sci. 2014 Apr 28;12:20. doi: 10.1186/1477-5956-12-20 (PMC4021461; doi:10.1186/1477-5956-12-20)
Supplement: Additional files 2: Table S2 — A protein – peptide summary of all the proteins that were identified by MALDI TOF/TOF mass spectrometry with total ion score confidence intervals of greater than 95% and peptide rank of 1. For each protein identified (Table S1), the individual peptide sequences, individual peptide ion score confidence intervals (C.I).% and individual peptide iTRAQ ratios are given. The iTRAQ tags were assigned as follows: 114 control (unlesioned), 115 - 3 days-post lesion, 116 - 7 days post-lesion, 117 - 14 days post-lesion. [file 1477-5956-12-20-S2.pdf]

Applied Biosystems

Analysis Information

Report Type	Protein-Peptide Summary by Sample Set	Analysis Type	MS/MS
Sample Set Name	Striatum iTRAQ Sept12	Database	NewNCBInr
Analysis Name	Rat	Creation Date	09/18/2012 11:08:39
Reported By	09/24/2012 13:30:33 - admin	Last Modified	09/18/2012 15:53:22

MS Acq. : Proc. Methods	(Unspecified) : (Unspecified)
Interpretation Method	(Unspecified)

Rank	Protein Name	Accession No.	Protein MW	Pep. Count	Total Ion Score	Avg. iTRAQ Ratio 115/114*	Avg. iTRAQ Ratio 116/114*	Avg. iTRAQ Ratio 117/114*	iTRAQ SDev. 115/114*	iTRAQ SDev. 116/114*	iTRAQ SDev. 117/114*	iTRAQ Peptides 115/114	iTRAQ Peptides 116/114	iTRAQ Peptides 117/114	Total Ion C. I. %
1	RecName: Full=Spectrin alpha chain, brain; AltName: Full=Alpha-II spectrin; AltName: Full=Fodrin al	gi|17380501	313782.4	84	5153	1.042	0.979	0.983	0.369	0.317	0.356	97	97	97	100

Peptide Information

Calc. Mass	Obsrv. Mass	± da	± ppm	Start Seq.	End Seq.	Sequence	Ion Score	C. I. %	Modification	Plate [#] Name	Gel Idx/Pos [4700 Sample Name]	iTRAQ Ratio 115/114*	iTRAQ Ratio 116/114*	iTRAQ Ratio 117/114*	Rank	Result Type
1016.5438	1016.5273	-0.0165	-16	46	51	FQFFQR	28	96.356	(N-term)_iTRAQ[0]	[7] F5 120912	263/255	0.909	0.985	0.911	1	Mascot
1134.6392	1134.5739	-0.0653	-58	251	259	LFGAAEVQR	28	96.455	(N-term)_iTRAQ[0]	[5] F4	153/145	1.179	1.185	0.920	1	Mascot
1217.661	1217.5813	-0.0797	-65	1217	1225	SLQQLAEER	29	97.024	(N-term)_iTRAQ[0]	[5] F4	145/137	0.888	0.871	0.611	1	Mascot
1229.7338	1229.6974	-0.0364	-30	286	295	DLASVQALLR	46	99.94	(N-term)_iTRAQ[0]	[1] F3 030912	354/346	0.846	1.391	0.749	1	Mascot
1244.6871	1244.7332	0.0461	37	2079	2087	LLEAQSHFR	29	96.646	(N-term)_iTRAQ[0]	[3] F6 and F9	1180/1172	1.344	0.895	1.208	1	Mascot
1282.6763	1282.6219	-0.0544	-42	1785	1794	LLVSSEDYGR	29	96.767	(N-term)_iTRAQ[0]	[1] F3 030912	219/211	0.565	0.681	0.606	1	Mascot
1309.6873	1309.6072	-0.0801	-61	2427	2435	EELYQNLTR	53	99.986	(N-term)_iTRAQ[0]	[5] F4	150/142	1.612	1.416	0.916	1	Mascot
1317.7134	1317.6434	-0.07	-53	1370	1380	DVTGAEALLER	47	99.955	(N-term)_iTRAQ[0]	[1] F3 030912	261/253	0.753	0.931	0.777	1	Mascot
1346.793	1346.7758	-0.0172	-13	130	137	QWELLLEK	45	99.913	(N-term)_iTRAQ[0], Lysine(K)_iTRAQ[8]	[7] F5 120912	356/348	0.806	0.892	1.284	1	Mascot
1357.7964	1357.7609	-0.0355	-26	450	459	AALLELWELR	48	99.965	(N-term)_iTRAQ[0]	[1] F3 030912	501/493	0.680	0.898	0.534	1	Mascot
1359.7644	1359.7129	-0.0515	-38	1132	1138	KFDDFQK	36	99.348	(N-term)_iTRAQ[0], Lysine(K)_iTRAQ[1,7]	[2] F12 040912	199/191	1.453	1.424	1.574	1	Mascot
1359.7644	1359.8247	0.0603	44	1132	1138	KFDDFQK	33	98.76	(N-term)_iTRAQ[0], Lysine(K)_iTRAQ[1,7]	[4] F7 and F10+11	1075/1067	1.016	0.847	1.019	1	Mascot
1360.7834	1360.8546	0.0712	52	638	646	LIDVNHYAK	46	99.941	(N-term)_iTRAQ[0], Lysine(K)_iTRAQ[9]	[4] F7 and F10+11	1110/1102	1.118	1.130	1.529	1	Mascot
1367.7933	1367.7679	-0.0254	-19	1013	1022	QGFVPAAYVK	57	99.995	(N-term)_iTRAQ[0], Lysine(K)_iTRAQ[10]	[7] F5 120912	214/206	1.138	1.164	1.043	1	Mascot
1407.7842	1407.7512	-0.033	-23	205	214	VNEVNQFAAK	67	100	(N-term)_iTRAQ[0], Lysine(K)_iTRAQ[10]	[7] F5 120912	157/149	1.072	1.050	1.083	1	Mascot
1407.7916	1407.7772	-0.0144	-10	653	661	MNEVISLWK	36	99.318	(N-term)_iTRAQ[0], Lysine(K)_iTRAQ[9]	[7] F5 120912	359/351	1.172	1.071	0.913	1	Mascot
1435.6825	1435.6041	-0.0784	-55	792	801	DVEDEETWIR	63	99.999	(N-term)_iTRAQ[0]	[1] F3 030912	241/233	0.974	0.786	0.705	1	Mascot
1445.8151	1445.8721	0.057	39	1845	1853	LAQFVEHWK	69	100	(N-term)_iTRAQ[0], Lysine(K)_iTRAQ[9]	[4] F7 and F10+11	1255/1247	0.950	0.958	0.911	1	Mascot
1446.756	1446.6876	-0.0684	-47	8	18	VLETAEDIQER	54	99.989	(N-term)_iTRAQ[0]	[1] F3 030912	222/214	1.190	1.105	1.070	1	Mascot
1460.8319	1460.7939	-0.038	-26	709	718	DLTNVQNLQK	53	99.989	(N-term)_iTRAQ[0], Lysine(K)_iTRAQ[10]	[7] F5 120912	157/149	1.113	1.162	0.954	1	Mascot
1463.7992	1463.7296	-0.0696	-48	263	272	DVDETIGWIK	40	99.746	(N-term)_iTRAQ[0], Lysine(K)_iTRAQ[10]	[5] F4	225/217	1.182	1.374	0.980	1	Mascot
1463.7992	1463.7505	-0.0487	-33	263	272	DVDETIGWIK	46	99.944	(N-term)_iTRAQ[0], Lysine(K)_iTRAQ[10]	[7] F5 120912	283/275	1.315	1.184	1.326	1	Mascot
1468.7992	1468.7928	-0.0064	-4	1226	1237	SQLLGSAHEVQR	30	97.444	(N-term)_iTRAQ[0]	[4] F7 and F10+11	165/157	3.906	2.573	1.510	1	Mascot
1497.7869	1497.7147	-0.0722	-48	372	381	DLTSWVTEMK	63	99.999	(N-term)_iTRAQ[0], Lysine(K)_iTRAQ[10]	[5] F4	280/272	1.125	0.743	0.969	1	Mascot
1497.7869	1497.7594	-0.0275	-18	372	381	DLTSWVTEMK	37	99.533	(N-term)_iTRAQ[0], Lysine(K)_iTRAQ[10]	[7] F5 120912	351/343	1.049	0.810	0.733	1	Mascot
1513.8975	1513.8357	-0.0618	-41	450	460	AALLELWELRR	49	99.971	(N-term)_iTRAQ[0]	[6] F8 110912	447/439	1.243	0.642	0.866	1	Mascot
1520.8206	1520.7815	-0.0391	-26	1985	1995	ADVVESWIGEK	51	99.98	(N-term)_iTRAQ[0], Lysine(K)_iTRAQ[11]	[7] F5 120912	298/290	0.845	1.116	0.544	1	Mascot
1537.8094	1537.736	-0.0734	-48	1547	1558	LGESQTLQQFSR	30	97.69	(N-term)_iTRAQ[0]	[5] F4	166/158	1.100	0.979	0.738	1	Mascot
1554.8737	1554.9392	0.0655	42	1784	1794	KLLVSSEDYGR	34	98.971	(N-term)_iTRAQ[0], Lysine(K)_iTRAQ[1]	[4] F7 and F10+11	1092/1084	1.424	1.390	1.409	1	Mascot
1574.8298	1574.7783	-0.0515	-33	337	347	EELITNWEQIR	39	99.718	(N-term)_iTRAQ[0]	[1] F3 030912	340/332	0.948	0.629	0.573	1	Mascot

1594.8872	1594.7958	-0.0914	-57	766	776	YEALKEPMVAR	27	95.36	(N-term)_iTRAQ[0], Lysine(K)_iTRAQ[5]	[6] F8 110912	235/227	1.171	1.252	1.176	1	Mascot
1594.8872	1594.8605	-0.0267	-17	766	776	YEALKEPMVAR	32	98.372	(N-term)_iTRAQ[0], Lysine(K)_iTRAQ[5]	[4] F7 and F10+11	228/220	0.680	0.746	0.900	1	Mascot
1613.6611	1613.6017	-0.0594	-37	1453	1464	DCEQAENWMAAR	49	99.966	(N-term)_iTRAQ[0], MMTS (C)[2]	[1] F3 030912	288/280	0.886	0.887	0.798	1	Mascot
1615.8776	1615.8124	-0.0652	-40	1033	1045	ENLLEEQGSIALR	71	100	(N-term)_iTRAQ[0]	[1] F3 030912	296/288	1.799	1.375	1.093	1	Mascot
1639.8458	1639.7642	-0.0816	-50	2304	2315	MQHNLEQQIQAR	87	100	(N-term)_iTRAQ[0]	[6] F8 110912	201/193	0.842	0.866	1.010	1	Mascot
1640.7836	1640.7211	-0.0625	-38	475	485	DTEQVDNWMSK	54	99.989	(N-term)_iTRAQ[0], Lysine(K)_iTRAQ[11]	[7] F5 120912	195/187	1.083	0.998	1.041	1	Mascot
1689.8793	1689.9681	0.0888	53	720	733	HALLEADVAAHQDR	57	99.996	(N-term)_iTRAQ[0]	[4] F7 and F10+11	1083/1075	0.840	0.970	0.924	1	Mascot
1700.8953	1700.8412	-0.0541	-32	826	839	HQALQAEIAGHEPR	54	99.99	(N-term)_iTRAQ[0]	[2] F12 040912	204/196	0.976	0.941	1.058	1	Mascot
1711.9966	1712.0667	0.0701	41	2415	2426	ALSSEGKPYVTK	50	99.977	(N-term)_iTRAQ[0], Lysine(K)_iTRAQ[7,12]	[4] F7 and F10+11	1064/1056	0.857	0.916	0.808	1	Mascot
1721.8844	1721.8232	-0.0612	-36	1559	1570	DVDEIEAWISEK	67	99.999	(N-term)_iTRAQ[0], Lysine(K)_iTRAQ[12]	[5] F4	315/307	1.232	0.862	1.175	1	Mascot
1730.0911	1730.1655	0.0744	43	813	824	GKDLIGVQNLLK	89	100	(N-term)_iTRAQ[0], Lysine(K)_iTRAQ[2,12]	[4] F7 and F10+11	1212/1204	0.993	0.913	0.926	1	Mascot
1730.9309	1731.0026	0.0717	41	336	347	REELITNWEQIR	35	99.259	(N-term)_iTRAQ[0]	[3] F6 and F9	1273/1265	1.023	1.249	1.159	1	Mascot
1731.8534	1731.7552	-0.0982	-57	614	627	HQAFEAELSANQSR	47	99.952	(N-term)_iTRAQ[0]	[6] F8 110912	193/185	1.273	1.075	1.181	1	Mascot
1743.0427	1743.0693	0.0266	15	2088	2098	KVEDLFLTFAK	84	100	(N-term)_iTRAQ[0], Lysine(K)_iTRAQ[1,11]	[3] F6 and F9	1362/1354	0.857	0.733	0.761	1	Mascot
1743.0427	1743.1091	0.0664	38	2088	2098	KVEDLFLTFAK	72	100	(N-term)_iTRAQ[0], Lysine(K)_iTRAQ[1,11]	[4] F7 and F10+11	1302/1294	0.966	1.051	0.992	1	Mascot
1747.9337	1748.0013	0.0676	39	228	239	TKQEEVNAAWQR	64	99.999	(N-term)_iTRAQ[0], Lysine(K)_iTRAQ[2]	[4] F7 and F10+11	1068/1060	0.967	0.878	1.161	1	Mascot
1748.8854	1748.8329	-0.0525	-30	1912	1923	HEAFETDFTVHK	42	99.858	(N-term)_iTRAQ[0], Lysine(K)_iTRAQ[12]	[2] F12 040912	255/247	1.881	1.533	1.531	1	Mascot
1748.9979	1748.9946	-0.0033	-2	1197	1209	LMVHTVATFNSIK	75	100	(N-term)_iTRAQ[0], Lysine(K)_iTRAQ[13]	[4] F7 and F10+11	326/318	0.775	0.468	0.772	1	Mascot
1752.8538	1752.7878	-0.066	-38	1592	1605	HQAFEAELHANADR	63	99.999	(N-term)_iTRAQ[0]	[2] F12 040912	198/190	0.778	0.850	0.891	1	Mascot
1752.8538	1752.9523	0.0985	56	1592	1605	HQAFEAELHANADR	63	99.999	(N-term)_iTRAQ[0]	[8] F13-15 and F1+2	1555/1547	0.701	0.810	0.714	1	Mascot
1777.8953	1777.9542	0.0589	33	1700	1713	HQLLEADISAHEDR	35	99.217	(N-term)_iTRAQ[0]	[4] F7 and F10+11	1100/1092	1.450	1.109	1.408	1	Mascot
1784.8903	1784.8116	-0.0787	-44	1314	1326	CTELNQAWTSLGK	55	99.991	(N-term)_iTRAQ[0], Lysine(K)_iTRAQ[13], MMTS (C)[1]	[5] F4	280/272	1.383	1.754	1.142	1	Mascot
1789.933	1789.9196	-0.0134	-7	1099	1110	EANELQQWINEK	49	99.971	(N-term)_iTRAQ[0], Lysine(K)_iTRAQ[12]	[7] F5 120912	316/308	0.979	1.039	1.080	1	Mascot
1813.9	1813.8058	-0.0942	-52	273	285	EKEQLMASDDFGR	86	100	(N-term)_iTRAQ[0], Lysine(K)_iTRAQ[2]	[6] F8 110912	220/212	0.878	0.728	0.792	1	Mascot
1813.9	1813.9512	0.0512	28	273	285	EKEQLMASDDFGR	87	100	(N-term)_iTRAQ[0], Lysine(K)_iTRAQ[2]	[3] F6 and F9	1179/1171	2.153	1.873	2.137	1	Mascot
1830.7939	1830.8125	0.0186	10	157	168	ECEDVMDWINDK	47	99.956	(N-term)_iTRAQ[0], Lysine(K)_iTRAQ[12], MMTS (C)[2]	[7] F5 120912	421/413	0.831	0.855	0.303	1	Mascot
1849.113	1849.1886	0.0756	41	990	1002	KGDILTLLNSTNK	82	100	(N-term)_iTRAQ[0], Lysine(K)_iTRAQ[1,13]	[4] F7 and F10+11	1224/1216	1.160	1.334	0.633	1	Mascot
1851.9626	1851.9777	0.0151	8	567	580	AQLADSFHLQQFFR	91	100	(N-term)_iTRAQ[0]	[4] F7 and F10+11	430/422	1.337	1.198	1.051	1	Mascot
1865.879	1865.8339	-0.0451	-24	191	204	FEEFQTDLAAHEER	53	99.988	(N-term)_iTRAQ[0]	[3] F6 and F9	296/288	0.623	1.132	0.396	1	Mascot
1889.9325	1889.8335	-0.099	-52	1830	1844	LSDDNTIGQEEIQQR	87	100	(N-term)_iTRAQ[0]	[1] F3 030912	200/192	0.909	0.860	0.694	1	Mascot
1894.0531	1894.0034	-0.0497	-26	215	227	LIQEQHPEEELIK	46	99.944	(N-term)_iTRAQ[0], Lysine(K)_iTRAQ[13]	[3] F6 and F9	256/248	0.777	0.970	1.004	1	Mascot
1894.0531	1894.0327	-0.0204	-11	215	227	LIQEQHPEEELIK	66	99.999	(N-term)_iTRAQ[0], Lysine(K)_iTRAQ[13]	[4] F7 and F10+11	254/246	1.302	1.061	1.135	1	Mascot
1894.0531	1894.0823	0.0292	15	215	227	LIQEQHPEEELIK	64	99.999	(N-term)_iTRAQ[0], Lysine(K)_iTRAQ[13]	[4] F7 and F10+11	260/252	2.271	1.255	0.957	1	Mascot
1899.9851	1899.9469	-0.0382	-20	1972	1984	LDENSAFLQFNWK	39	99.68	(N-term)_iTRAQ[0], Lysine(K)_iTRAQ[13]	[7] F5 120912	393/385	1.406	1.072	1.041	1	Mascot
1907.9497	1907.8375	-0.1122	-59	743	756	QFQDAGHFDAENIK	101	100	(N-term)_iTRAQ[0], Lysine(K)_iTRAQ[14]	[6] F8 110912	237/229	1.048	1.225	1.164	1	Mascot
1918.9565	1918.8901	-0.0664	-35	533	547	LIQNNHYAMEDVATR	68	100	(N-term)_iTRAQ[0]	[3] F6 and F9	242/234	0.964	0.749	0.904	1	Mascot
1919.1	1919.0251	-0.0749	-39	1633	1646	LAALADQWQFLVQK	79	100	(N-term)_iTRAQ[0], Lysine(K)_iTRAQ[14]	[5] F4	378/370	1.161	1.092	0.940	1	Mascot
1919.1	1919.0345	-0.0655	-34	1633	1646	LAALADQWQFLVQK	59	99.997	(N-term)_iTRAQ[0], Lysine(K)_iTRAQ[14]	[1] F3 030912	488/480	0.624	0.441	0.652	1	Mascot
1924.9875	1925.0732	0.0857	45	1241	1252	DADETKEWIEEK	58	99.996	(N-term)_iTRAQ[0], Lysine(K)_iTRAQ[6,12]	[4] F7 and F10+11	1129/1121	0.702	0.980	1.034	1	Mascot
1975.948	1976.0106	0.0626	32	1439	1452	MMLDHCLELQLFHR	72	100	(N-term)_iTRAQ[0], MMTS (C)[6]	[4] F7 and F10+11	1333/1325	1.053	0.688	1.046	1	Mascot
1979.1284	1979.1339	0.0055	3	321	335	LQQSHPLSANQIQVK	93	100	(N-term)_iTRAQ[0], Lysine(K)_iTRAQ[15]	[4] F7 and F10+11	212/204	1.027	0.807	1.044	1	Mascot
1991.9742	1991.9474	-0.0268	-13	847	862	GNAMVEEGHFAAEDVK	110	100	(N-term)_iTRAQ[0], Lysine(K)_iTRAQ[16]	[4] F7 and F10+11	230/222	0.834	0.873	0.772	1	Mascot

2007.9691	2007.9219	-0.0472	-24	847	862	GNAMVEEGHFAAEDVK	47	99.946	(N-term)_iTRAQ[0], Lysine(K)_iTRAQ[16], Oxidation (M)[4]	[4] F7 and F10+11	188/180	1.146	1.023	1.297	1 Mascot
2066.1267	2066.0769	-0.0498	-24	1068	1082	QVEELYQSLLELGEK	86	100	(N-term)_iTRAQ[0], Lysine(K)_iTRAQ[15]	[1] F3 030912	512/504	0.897	0.908	0.851	1 Mascot
2087.113	2086.9897	-0.1233	-59	85	101	HQAFEAEVQANSGAIVK	133	100	(N-term)_iTRAQ[0], Lysine(K)_iTRAQ[17]	[6] F8 110912	250/242	1.159	0.986	1.194	1 Mascot
2087.113	2087.0706	-0.0424	-20	85	101	HQAFEAEVQANSGAIVK	97	100	(N-term)_iTRAQ[0], Lysine(K)_iTRAQ[17]	[4] F7 and F10+11	246/238	1.175	0.968	1.426	1 Mascot
2133.1357	2133.0706	-0.0651	-31	2265	2281	IEDLGAAMEEALILDNK	89	100	(N-term)_iTRAQ[0], Lysine(K)_iTRAQ[17]	[1] F3 030912	496/488	2.028	1.642	2.436	1 Mascot
2138.0764	2138.1724	0.096	45	190	204	KFEEFQTDLAAHEER	83	100	(N-term)_iTRAQ[0], Lysine(K)_iTRAQ[1]	[4] F7 and F10+11	1169/1161	0.894	0.899	1.173	1 Mascot
2156.1934	2156.2861	0.0927	43	1299	1313	LIQSHPESAEDLKEK	89	100	(N-term)_iTRAQ[0], Lysine(K)_iTRAQ[13, 15]	[4] F7 and F10+11	1088/1080	1.158	0.741	1.095	1 Mascot
2162.1299	2161.9651	-0.1648	-76	1829	1844	KLSDDNTIGQEEIQQR	71	100	(N-term)_iTRAQ[0], Lysine(K)_iTRAQ[1]	[6] F8 110912	188/180	0.865	0.808	0.951	1 Mascot
2170.1362	2170.0073	-0.1289	-59	595	610	TATDEAYKDPSNLQGK	102	100	(N-term)_iTRAQ[0], Lysine(K)_iTRAQ[8,1 6]	[6] F8 110912	162/154	0.822	0.762	0.877	1 Mascot
2180.1472	2180.0791	-0.0681	-31	743	757	QFQDAGHFDAENIKK	91	100	(N-term)_iTRAQ[0], Lysine(K)_iTRAQ[14, 15]	[2] F12 040912	233/225	1.303	1.072	1.274	1 Mascot
2180.1472	2180.252	0.1048	48	743	757	QFQDAGHFDAENIKK	81	100	(N-term)_iTRAQ[0], Lysine(K)_iTRAQ[14, 15]	[4] F7 and F10+11	1106/1098	0.888	0.712	0.903	1 Mascot
2291.1023	2291.0374	-0.0649	-28	419	439	SADESGQALLAAGHYAS DEVR	84	100	(N-term)_iTRAQ[0]	[7] F5 120912	255/247	1.495	1.303	1.223	1 Mascot
2346.3066	2346.2673	-0.0393	-17	1501	1519	IAALQAFADQLIAVDHYAK	59	99.997	(N-term)_iTRAQ[0], Lysine(K)_iTRAQ[19]	[7] F5 120912	507/499	0.929	1.113	1.299	1 Mascot
2364.1626	2364.0911	-0.0715	-30	102	121	LDETGNLMISEGHFASET IR	42	99.837	(N-term)_iTRAQ[0]	[7] F5 120912	333/325	0.783	0.714	1.034	1 Mascot
2385.1609	2385.2307	0.0698	29	2208	2225	QEFAQHANAFHQWIQET R	70	100	(N-term)_iTRAQ[0]	[4] F7 and F10+11	1217/1209	1.002	0.941	0.888	1 Mascot
2405.3333	2405.3679	0.0346	14	2264	2281	KIEDLGAAMEEALILDNK	62	99.998	(N-term)_iTRAQ[0], Lysine(K)_iTRAQ[1,1 8]	[4] F7 and F10+11	475/467	1.177	1.037	1.127	1 Mascot
2442.1333	2442.0791	-0.0542	-22	2100	2119	ASAFNSWFENAEEDLTD PVR	82	100	(N-term)_iTRAQ[0]	[1] F3 030912	517/509	0.857	0.834	0.732	1 Mascot
2465.3069	2465.5017	0.1948	79	931	950	KHEALMSDLSAYGSSIQA LR	89	100	(N-term)_iTRAQ[0], Lysine(K)_iTRAQ[1]	[4] F7 and F10+11	1260/1252	0.781	1.031	1.077	1 Mascot
2472.2351	2472.1663	-0.0688	-28	1253	1273	NQALNTDNYGHDLASVQ ALQR	58	99.996	(N-term)_iTRAQ[0]	[7] F5 120912	255/247	0.765	0.892	1.052	1 Mascot
2475.1775	2475.1404	-0.0371	-15	2162	2180	VASNPYTWFTMEALEET WR	32	98.536	(N-term)_iTRAQ[0]	[1] F3 030912	554/546	0.612	0.374	1.211	1 Mascot
2497.3469	2497.2737	-0.0732	-29	1890	1910	MTLVASEDYGDTLAAIQG LLK	53	99.987	(N-term)_iTRAQ[0], Lysine(K)_iTRAQ[21]	[1] F3 030912	539/531	0.980	0.919	1.456	1 Mascot
2533.2339	2533.1152	-0.1187	-47	1716	1736	DLNSQADSLMTSSAFDT SQVK	96	100	(N-term)_iTRAQ[0], Lysine(K)_iTRAQ[21]	[1] F3 030912	316/308	1.179	1.207	1.117	1 Mascot
2707.4287	2707.3738	-0.0549	-20	486	507	QEAFLLNEDLGDSLDSVE ALLK	106	100	(N-term)_iTRAQ[0], Lysine(K)_iTRAQ[22]	[1] F3 030912	554/546	0.735	1.031	0.899	1 Mascot
2858.5635	2858.636	0.0725	25	2415	2435	ALSSEGKPYVTKEELYQN LTR	28	96.472	(N-term)_iTRAQ[0], Lysine(K)_iTRAQ[7,1 2]	[4] F7 and F10+11	1221/1213	1.447	1.060	1.169	1 Mascot
3214.5676	3214.4517	-0.1159	-36	1866	1889	LEESLEYQQFVANVEEEE AWINEK	74	100	(N-term)_iTRAQ[0], Lysine(K)_iTRAQ[24]	[1] F3 030912	522/514	1.071	1.062	1.695	1 Mascot

2	spectrin beta chain, brain 1 [Rattus norvegicus]	gi|61557085	299802.2	54	3021	1.140	0.988	0.982	0.476	0.401	0.514	65	65	65	100

Peptide Information

Calc. Mass	Obsrv. Mass	± da	± ppm	Start Seq.	End Seq.	Sequence	Ion Score	C. I. %	Modification	Plate [#] Name	Gel Idx/Pos [4700 Sample Name]	iTRAQ Ratio 115/114*	iTRAQ Ratio 116/114*	iTRAQ Ratio 117/114*	Rank Result Type
986.592	986.5673	-0.0247	-25	2351	2356	FSLFGK	32	98.3	(N-term)_iTRAQ[0], Lysine(K)_iTRAQ[6]	[3] F6 and F9	315/307	1.629	0.892	1.131	1 Mascot
1056.6649	1056.5992	-0.0657	-62	478	486	VQAVVAVAR	41	99.816	(N-term)_iTRAQ[0]	[1] F3 030912	196/188	1.627	1.264	1.659	1 Mascot
1113.6864	1113.605	-0.0814	-73	905	913	VAVVNQIAR	36	99.395	(N-term)_iTRAQ[0]	[5] F4	127/119	1.542	1.237	2.338	1 Mascot
1285.6886	1285.6348	-0.0538	-42	660	668	ILSSDDYGK	46	99.938	(N-term)_iTRAQ[0], Lysine(K)_iTRAQ[9]	[3] F6 and F9	195/187	1.435	1.055	0.982	1 Mascot
1285.6886	1285.655	-0.0336	-26	660	668	ILSSDDYGK	50	99.978	(N-term)_iTRAQ[0], Lysine(K)_iTRAQ[9]	[7] F5 120912	157/149	1.066	0.790	0.802	1 Mascot
1330.8066	1330.7794	-0.0272	-20	2074	2083	LTTLELLEVR	34	98.98	(N-term)_iTRAQ[0]	[1] F3 030912	400/392	1.250	1.080	1.340	1 Mascot
1385.8025	1385.8049	0.0024	2	2010	2019	LILEVHQFSR	36	99.323	(N-term)_iTRAQ[0]	[4] F7 and F10+11	309/301	0.661	0.841	0.558	1 Mascot
1396.8046	1396.845	0.0404	29	831	839	YKEVAELTR	27	95.486	(N-term)_iTRAQ[0], Lysine(K)_iTRAQ[2]	[3] F6 and F9	1172/1164	1.138	0.774	0.965	1 Mascot
1404.7206	1404.7928	0.0722	51	110	118	IHCLENVDK	31	98.123	(N-term)_iTRAQ[0], Lysine(K)_iTRAQ[9], MMTS (C)[3]	[4] F7 and F10+11	1137/1129	0.916	0.913	1.142	1 Mascot
1405.7573	1405.7129	-0.0444	-32	1881	1889	ENEVLEAWK	50	99.978	(N-term)_iTRAQ[0], Lysine(K)_iTRAQ[9]	[7] F5 120912	250/242	1.184	0.983	0.921	1 Mascot
1423.8407	1423.7781	-0.0626	-44	549	558	VLLLSQDYGK	53	99.988	(N-term)_iTRAQ[0], Lysine(K)_iTRAQ[10]	[5] F4	207/199	1.075	0.820	0.806	1 Mascot

1423.8407	1423.8071	-0.0336	-24	549	558	VLLLSQDYGK	58	99.996	(N-term)_iTRAQ[0], Lysine(K)_iTRAQ[10]	[7] F5 120912	256/248	1.229	1.196	1.310	1	Mascot
1492.8746	1492.9303	0.0557	37	1328	1335	EWLDKIEK	41	99.783	(N-term)_iTRAQ[0], Lysine(K)_iTRAQ[5,8]	[4] F7 and F10+11	1166/1158	1.227	1.012	1.098	1	Mascot
1497.851	1497.8684	0.0174	12	2172	2185	SALPAQSAATLPAR	36	99.384	(N-term)_iTRAQ[0]	[8] F13-15 and F1+2	580/572	1.055	0.838	0.787	1	Mascot
1497.851	1497.9017	0.0507	34	2172	2185	SALPAQSAATLPAR	42	99.86	(N-term)_iTRAQ[0]	[8] F13-15 and F1+2	583/575	3.440	3.400	3.800	1	Mascot
1520.8054	1520.7421	-0.0633	-42	2039	2049	EIGQSVDEVEK	45	99.929	(N-term)_iTRAQ[0], Lysine(K)_iTRAQ[11]	[7] F5 120912	150/142	1.195	0.863	1.094	1	Mascot
1525.7407	1525.7076	-0.0331	-22	1070	1080	DLDDFQSWLSR	46	99.941	(N-term)_iTRAQ[0]	[1] F3 030912	400/392	1.227	0.948	1.124	1	Mascot
1534.8059	1534.8301	0.0242	16	1462	1472	FMELLEPLNER	43	99.879	(N-term)_iTRAQ[0]	[8] F13-15 and F1+2	386/378	1.062	0.868	1.025	1	Mascot
1543.8479	1543.9353	0.0874	57	215	224	HRPDLIDFDK	28	95.649	(N-term)_iTRAQ[0], Lysine(K)_iTRAQ[10]	[8] F13-15 and F1+2	1505/1497	1.391	1.034	1.085	1	Mascot
1543.8479	1543.9387	0.0908	59	215	224	HRPDLIDFDK	36	99.331	(N-term)_iTRAQ[0], Lysine(K)_iTRAQ[10]	[8] F13-15 and F1+2	1171/1163	1.318	1.175	1.061	1	Mascot
1554.8074	1554.7385	-0.0689	-44	177	186	DALLLWCQMK	31	98.178	(N-term)_iTRAQ[0], Lysine(K)_iTRAQ[10], MMTS (C)[7]	[5] F4	394/386	1.364	0.935	1.012	1	Mascot
1554.8074	1554.7823	-0.0251	-16	177	186	DALLLWCQMK	39	99.696	(N-term)_iTRAQ[0], Lysine(K)_iTRAQ[10], MMTS (C)[7]	[7] F5 120912	482/474	2.711	3.327	4.912	1	Mascot
1564.7988	1564.774	-0.0248	-16	1917	1927	DLMLWMEDVIR	30	97.774	(N-term)_iTRAQ[0]	[5] F4	422/414	0.853	0.465	0.429	1	Mascot
1564.7988	1564.7742	-0.0246	-16	1917	1927	DLMLWMEDVIR	57	99.995	(N-term)_iTRAQ[0]	[1] F3 030912	554/546	0.846	1.060	0.814	1	Mascot
1603.8088	1603.7562	-0.0526	-33	1488	1499	DVEDEILWVGER	47	99.953	(N-term)_iTRAQ[0]	[1] F3 030912	370/362	1.277	1.041	0.782	1	Mascot
1603.8088	1603.808	-0.0008	0	1488	1499	DVEDEILWVGER	41	99.816	(N-term)_iTRAQ[0]	[8] F13-15 and F1+2	408/400	0.631	0.457	0.399	1	Mascot
1603.8088	1603.8186	0.0098	6	1488	1499	DVEDEILWVGER	52	99.984	(N-term)_iTRAQ[0]	[8] F13-15 and F1+2	401/393	0.636	0.647	0.498	1	Mascot
1603.8088	1603.8192	0.0104	6	1488	1499	DVEDEILWVGER	57	99.995	(N-term)_iTRAQ[0]	[8] F13-15 and F1+2	404/396	0.865	0.784	0.831	1	Mascot
1621.8319	1621.7416	-0.0903	-56	487	497	ELEAESYHDIK	57	99.995	(N-term)_iTRAQ[0], Lysine(K)_iTRAQ[11]	[6] F8 110912	199/191	0.721	0.681	0.448	1	Mascot
1646.8146	1646.7734	-0.0412	-25	1701	1712	EVDDLEQWIAER	68	100	(N-term)_iTRAQ[0]	[1] F3 030912	402/394	0.710	0.684	0.790	1	Mascot
1672.8792	1672.8226	-0.0566	-34	2273	2284	LSDGNEYLFQAK	43	99.881	(N-term)_iTRAQ[0], Lysine(K)_iTRAQ[12]	[7] F5 120912	246/238	2.041	1.188	1.399	1	Mascot
1674.7588	1674.6687	-0.0901	-54	1295	1307	MLTAQDMSYDEAR	60	99.998	(N-term)_iTRAQ[0]	[1] F3 030912	233/225	0.806	0.939	0.880	1	Mascot
1701.9044	1701.8894	-0.015	-9	724	736	EQWANLEQLSAIR	50	99.974	(N-term)_iTRAQ[0]	[1] F3 030912	373/365	1.539	1.087	1.091	1	Mascot
1737.8979	1737.813	-0.0849	-49	1037	1048	LAEISDVWEEMK	50	99.975	(N-term)_iTRAQ[0], Lysine(K)_iTRAQ[12]	[5] F4	335/327	1.172	0.959	0.955	1	Mascot
1738.9745	1739.0365	0.062	36	1616	1627	AKDEQSAVSMLK	77	100	(N-term)_iTRAQ[0], Lysine(K)_iTRAQ[2,12]	[4] F7 and F10+11	1098/1090	1.114	1.036	0.902	1	Mascot
1740.9088	1740.8516	-0.0572	-33	1361	1372	MWEVLESTTQTK	66	99.999	(N-term)_iTRAQ[0], Lysine(K)_iTRAQ[12]	[5] F4	263/255	0.966	0.792	1.066	1	Mascot
1750.036	1749.9863	-0.0497	-28	360	372	GNLEVLLFTIQSK	37	99.51	(N-term)_iTRAQ[0], Lysine(K)_iTRAQ[13]	[5] F4	371/363	1.539	1.196	1.165	1	Mascot
1774.8583	1774.8059	-0.0524	-30	1382	1394	AELFTQSCADLDK	64	99.999	(N-term)_iTRAQ[0], Lysine(K)_iTRAQ[13], MMTS (C)[8]	[5] F4	269/261	1.238	1.343	1.082	1	Mascot
1777.933	1777.8538	-0.0792	-45	487	498	ELEAESYHDIKR	44	99.896	(N-term)_iTRAQ[0], Lysine(K)_iTRAQ[11]	[2] F12 040912	191/183	1.241	0.680	0.959	1	Mascot
1795.8405	1795.7512	-0.0893	-50	891	904	FESLEPEMNNQASR	37	99.55	(N-term)_iTRAQ[0]	[1] F3 030912	238/230	1.716	1.173	1.746	1	Mascot
1847.8545	1847.7628	-0.0917	-50	1109	1120	NEIDNYEEDYQK	72	100	(N-term)_iTRAQ[0], Lysine(K)_iTRAQ[12]	[7] F5 120912	165/157	0.921	0.776	0.811	1	Mascot
1852.7859	1852.7164	-0.0695	-38	25	38	WDVDDWDNENSSAR	57	99.995	(N-term)_iTRAQ[0]	[1] F3 030912	273/265	1.260	0.843	0.742	1	Mascot
1873.8704	1873.8013	-0.0691	-37	643	655	FFWEMAEEEGWIR	30	97.255	(N-term)_iTRAQ[0]	[5] F4	410/402	0.999	1.022	1.022	1	Mascot
1903.0898	1903.0244	-0.0654	-34	1567	1579	QLWGLLIEETEKR	44	99.905	(N-term)_iTRAQ[0], Lysine(K)_iTRAQ[12]	[6] F8 110912	469/461	1.199	1.037	1.016	1	Mascot
1911.9182	1911.8557	-0.0625	-33	2104	2117	VSEEAESQQWDTSK	81	100	(N-term)_iTRAQ[0], Lysine(K)_iTRAQ[14]	[7] F5 120912	150/142	1.469	1.466	0.853	1	Mascot
1963.0216	1963.0377	0.0161	8	1543	1559	SQNIITDSSSLNAEAIR	43	99.885	(N-term)_iTRAQ[0]	[8] F13-15 and F1+2	499/491	1.722	1.224	1.391	1	Mascot
1995.9215	1995.8149	-0.1066	-53	700	714	EGEDMIAEEHFGSEK	48	99.965	(N-term)_iTRAQ[0], Lysine(K)_iTRAQ[15]	[6] F8 110912	234/226	0.964	1.468	1.070	1	Mascot
1995.9215	1995.8894	-0.0321	-16	700	714	EGEDMIAEEHFGSEK	77	100	(N-term)_iTRAQ[0], Lysine(K)_iTRAQ[15]	[4] F7 and F10+11	228/220	1.290	1.291	0.757	1	Mascot
2039.1145	2039.0043	-0.1102	-54	1865	1879	LQAAYAGDKADDIQK	96	100	(N-term)_iTRAQ[0], Lysine(K)_iTRAQ[9,15]	[6] F8 110912	204/196	1.100	0.749	1.083	1	Mascot
2063.0654	2063.0068	-0.0586	-28	463	477	KHEAIETDIAAYEER	59	99.997	(N-term)_iTRAQ[0], Lysine(K)_iTRAQ[1]	[2] F12 040912	268/260	0.891	1.210	0.832	1	Mascot
2063.0654	2063.1697	0.1043	51	463	477	KHEAIETDIAAYEER	65	99.999	(N-term)_iTRAQ[0], Lysine(K)_iTRAQ[1]	[4] F7 and F10+11	1140/1132	0.551	0.892	0.865	1	Mascot
2072.1055	2072.0425	-0.063	-30	1937	1952	DVSSVELLMNNHQGIK	28	96.131	(N-term)_iTRAQ[0], Lysine(K)_iTRAQ[16]	[3] F6 and F9	312/304	0.699	0.671	0.867	1	Mascot
2088.0732	2088.0344	-0.0388	-19	1776	1792	DGLNEAWADLLELIDTR	38	99.6	(N-term)_iTRAQ[0]	[1] F3 030912	587/579	0.511	1.014	0.517	1	Mascot
2088.0894	2088.0349	-0.0545	-26	660	675	ILSSDDYGKDLTSVMR	40	99.777	(N-term)_iTRAQ[0], Lysine(K)_iTRAQ[9]	[4] F7 and F10+11	344/336	0.663	0.482	0.721	1	Mascot
2122.0591	2122.0635	0.0044	2	187	203	TAGYPNVNIHNFTTSWR	46	99.942	(N-term)_iTRAQ[0]	[4] F7 and F10+11	356/348	1.654	1.420	1.374	1	Mascot

2128.073	2128.1406	0.0676	32	1840	1856	MHTTFEHDIQALGTQVR	65	99.999	(N-term)_iTRAQ[0]	[4] F7 and F10+11	1220/1212	0.893	0.958	1.302	1 Mascot
2164.092	2163.9937	-0.0983	-45	1395	1410	WLHGLESQIQSDDYGK	122	100	(N-term)_iTRAQ[0], Lysine(K)_iTRAQ[16]	[6] F8 110912	375/367	1.037	0.944	0.728	1 Mascot
2191.2068	2191.1042	-0.1026	-47	2320	2338	AQTLPTSVVTITSESSPGK	87	100	(N-term)_iTRAQ[0], Lysine(K)_iTRAQ[19]	[1] F3 030912	274/266	1.405	0.678	0.580	1 Mascot
2249.0801	2249.1707	0.0906	40	1210	1224	KQEDFMTTMDANEEK	109	100	(N-term)_iTRAQ[0], Lysine(K)_iTRAQ[1,15]	[4] F7 and F10+11	1116/1108	1.139	1.164	1.016	1 Mascot
2310.2227	2310.1401	-0.0826	-36	444	462	LVSQDNFGFDLPAVEAATK	114	100	(N-term)_iTRAQ[0], Lysine(K)_iTRAQ[19]	[1] F3 030912	427/419	1.309	1.283	1.194	1 Mascot
2338.1694	2338.0417	-0.1277	-55	1081	1099	TQTAIASEDMPNTLTEAEK	96	100	(N-term)_iTRAQ[0], Lysine(K)_iTRAQ[19]	[1] F3 030912	246/238	1.021	0.766	1.064	1 Mascot
2355.2415	2355.1921	-0.0494	-21	157	172	FQIQDISVETEDNKEK	37	99.536	(N-term)_iTRAQ[0], Lysine(K)_iTRAQ[14,16]	[4] F7 and F10+11	274/266	1.009	1.152	1.032	1 Mascot
2439.2725	2439.3708	0.0983	40	1018	1036	EAEKLESEHPDQAQAILSR	71	100	(N-term)_iTRAQ[0], Lysine(K)_iTRAQ[4]	[4] F7 and F10+11	1124/1116	1.625	1.311	0.992	1 Mascot
2528.2073	2528.1521	-0.0552	-22	2285	2304	DDDEMNTWIQAITSAISSDK	91	100	(N-term)_iTRAQ[0], Lysine(K)_iTRAQ[20]	[1] F3 030912	555/547	1.023	0.855	0.527	1 Mascot
2626.3723	2626.4419	0.0696	27	1629	1648	HQILEQAVEDYAETVHQLSK	68	100	(N-term)_iTRAQ[0], Lysine(K)_iTRAQ[20]	[4] F7 and F10+11	1334/1326	1.914	1.482	1.450	1 Mascot

3	clathrin heavy chain 1 [Rattus norvegicus]	gi|9506497	206978.2	40	2326	0.909	1.053	1.110	0.389	0.359	0.334	46	46	46	100

Peptide Information

Calc. Mass	Obsrv. Mass	± da	± ppm	Start Seq.	End Seq.	Sequence	Ion Score	C. I. %	Modification	Plate [#] Name	Gel Idx/Pos [4700 Sample Name]	iTRAQ Ratio 115/114*	iTRAQ Ratio 116/114*	iTRAQ Ratio 117/114*	Rank Result Type
1116.6299	1116.5845	-0.0454	-41	1435	1441	AVNYFSK	47	99.955	(N-term)_iTRAQ[0], Lysine(K)_iTRAQ[7]	[3] F6 and F9	201/193	1.107	1.074	1.066	1 Mascot
1130.5929	1130.5599	-0.033	-29	731	737	YIQAACK	41	99.808	(N-term)_iTRAQ[0], Lysine(K)_iTRAQ[7], MMTS (C)[6]	[7] F5 120912	194/186	1.058	0.961	0.776	1 Mascot
1254.7694	1254.7344	-0.035	-28	857	865	LLLPWLEAR	35	99.228	(N-term)_iTRAQ[0]	[1] F3 030912	485/477	0.733	0.829	0.823	1 Mascot
1343.7821	1343.7211	-0.061	-45	799	806	YIEIYVQK	41	99.821	(N-term)_iTRAQ[0], Lysine(K)_iTRAQ[8]	[5] F4	209/201	0.825	0.784	0.883	1 Mascot
1346.7151	1346.7882	0.0731	54	611	619	AHIAQLCEK	43	99.872	(N-term)_iTRAQ[0], Lysine(K)_iTRAQ[9], MMTS (C)[7]	[4] F7 and F10+11	1140/1132	0.606	0.767	0.911	1 Mascot
1359.8246	1359.865	0.0404	30	270	278	HDVVFLITK	62	99.998	(N-term)_iTRAQ[0], Lysine(K)_iTRAQ[9]	[3] F6 and F9	1257/1249	0.864	0.959	1.100	1 Mascot
1360.7134	1360.7269	0.0135	10	1334	1342	EHLELFWSR	48	99.962	(N-term)_iTRAQ[0]	[3] F6 and F9	1293/1285	0.667	0.893	0.907	1 Mascot
1360.7134	1360.7627	0.0493	36	1334	1342	EHLELFWSR	45	99.915	(N-term)_iTRAQ[0]	[4] F7 and F10+11	1233/1225	0.798	1.093	0.915	1 Mascot
1386.7878	1386.7261	-0.0617	-44	1398	1406	VANVELYYK	48	99.958	(N-term)_iTRAQ[0], Lysine(K)_iTRAQ[9]	[7] F5 120912	219/211	1.019	0.948	1.384	1 Mascot
1440.772	1440.7178	-0.0542	-38	1216	1226	LLYNNVSNFGR	46	99.943	(N-term)_iTRAQ[0]	[5] F4	212/204	0.704	0.973	1.020	1 Mascot
1448.7617	1448.7045	-0.0572	-39	355	366	NNLAGAEELFAR	65	99.999	(N-term)_iTRAQ[0]	[1] F3 030912	313/305	1.021	1.129	1.290	1 Mascot
1450.641	1450.6282	-0.0128	-9	1255	1264	EVCFACVDGK	46	99.933	(N-term)_iTRAQ[0], Lysine(K)_iTRAQ[10], MMTS (C)[3,6]	[7] F5 120912	341/333	2.406	2.563	1.845	1 Mascot
1463.6876	1463.6755	-0.0121	-8	913	923	DPHLACVAYER	38	99.652	(N-term)_iTRAQ[0], MMTS (C)[6]	[4] F7 and F10+11	254/246	0.631	0.899	0.904	1 Mascot
1478.736	1478.6433	-0.0927	-63	882	892	IYIDSNNNPER	42	99.859	(N-term)_iTRAQ[0]	[5] F4	117/109	1.093	0.931	0.937	1 Mascot
1514.9668	1514.8855	-0.0813	-54	1444	1453	QLPLVKPYLR	36	99.348	(N-term)_iTRAQ[0], Lysine(K)_iTRAQ[6]	[6] F8 110912	295/287	0.783	0.737	0.918	1 Mascot
1520.853	1520.9012	0.0482	32	1610	1620	VDKLDASESLR	58	99.996	(N-term)_iTRAQ[0], Lysine(K)_iTRAQ[3]	[3] F6 and F9	1153/1145	0.646	0.704	0.903	1 Mascot
1524.8706	1524.7911	-0.0795	-52	87	96	TLQIFNIEMK	59	99.997	(N-term)_iTRAQ[0], Lysine(K)_iTRAQ[10]	[5] F4	298/290	1.437	1.434	1.507	1 Mascot
1559.8302	1559.7946	-0.0356	-23	1011	1022	IVLDNSVFSEHR	31	98.066	(N-term)_iTRAQ[0]	[4] F7 and F10+11	290/282	2.355	2.844	1.618	1 Mascot
1601.926	1601.8865	-0.0395	-25	1499	1508	LEKHELIEFR	43	99.889	(N-term)_iTRAQ[0], Lysine(K)_iTRAQ[3]	[2] F12 040912	285/277	0.786	0.913	0.768	1 Mascot
1601.926	1602.0145	0.0885	55	1499	1508	LEKHELIEFR	38	99.609	(N-term)_iTRAQ[0], Lysine(K)_iTRAQ[3]	[8] F13-15 and F1+2	1155/1147	0.614	0.972	1.053	1 Mascot
1623.9019	1623.8423	-0.0596	-37	508	519	VGYTPDWIFLLR	40	99.751	(N-term)_iTRAQ[0]	[1] F3 030912	527/519	1.225	1.179	1.415	1 Mascot
1642.0513	1642	-0.0513	-31	1023	1034	NLQNLLILTAIK	58	99.997	(N-term)_iTRAQ[0], Lysine(K)_iTRAQ[12]	[5] F4	368/360	0.850	0.998	1.399	1 Mascot
1642.0513	1642.0133	-0.038	-23	1023	1034	NLQNLLILTAIK	73	100	(N-term)_iTRAQ[0], Lysine(K)_iTRAQ[12]	[1] F3 030912	468/460	0.799	0.836	1.088	1 Mascot
1679.8477	1679.7715	-0.0762	-45	1102	1113	CNEPAVWSQLAK	54	99.99	(N-term)_iTRAQ[0], Lysine(K)_iTRAQ[12], MMTS (C)[1]	[5] F4	250/242	1.047	1.225	1.298	1 Mascot
1688.9181	1688.9155	-0.0026	-2	768	780	LTDQLPLIIVCDR	64	99.999	(N-term)_iTRAQ[0], MMTS (C)[11]	[8] F13-15 and F1+2	286/278	1.029	1.331	1.535	1 Mascot
1739.8673	1739.8962	0.0289	17	101	112	AHTMTDDVTFWK	90	100	(N-term)_iTRAQ[0], Lysine(K)_iTRAQ[12]	[3] F6 and F9	1255/1247	0.589	1.017	1.114	1 Mascot
1739.8673	1739.9355	0.0682	39	101	112	AHTMTDDVTFWK	70	100	(N-term)_iTRAQ[0], Lysine(K)_iTRAQ[12]	[4] F7 and F10+11	1195/1187	0.863	1.360	1.547	1 Mascot
1752.9418	1752.8821	-0.0597	-34	626	637	ALEHFTDLYDIK	64	99.999	(N-term)_iTRAQ[0], Lysine(K)_iTRAQ[12]	[6] F8 110912	363/355	1.131	0.943	0.901	1 Mascot

Calc. Mass	Obsrv. Mass	± da	± ppm	Start Seq.	End Seq.	Sequence	Ion Score	C. I. %	Modification	Plate [#] Name	Gel Idx/Pos [4700 Sample Name]	iTRAQ Ratio 115/114*	iTRAQ Ratio 116/114*	iTRAQ Ratio 117/114*	Rank	Result Type
1752.9418	1752.9512	0.0094	5	626	637	ALEHFTDLYDIK	67	100	(N-term)_iTRAQ[0], Lysine(K)_iTRAQ[12]	[4] F7 and F10+11	364/356	0.779	0.729	0.834	1	Mascot
1755.8622	1755.9255	0.0633	36	101	112	AHTMTDDVTFWK	46	99.943	(N-term)_iTRAQ[0], Lysine(K)_iTRAQ[12], Oxidation (M)[4]	[3] F6 and F9	1217/1209	0.973	0.935	1.285	1	Mascot
1784.9265	1784.8331	-0.0934	-52	488	500	VIQCFAETGQVQK	80	100	(N-term)_iTRAQ[0], Lysine(K)_iTRAQ[13], MMTS (C)[4]	[5] F4	238/230	1.307	1.287	1.337	1	Mascot
1839.9586	1839.8589	-0.0997	-54	838	851	GQFSTDELVAEVEK	73	100	(N-term)_iTRAQ[0], Lysine(K)_iTRAQ[14]	[5] F4	241/233	0.815	0.960	0.805	1	Mascot
1846.0103	1845.9502	-0.0601	-33	64	78	RPISADSAIMNPASK	37	99.532	(N-term)_iTRAQ[0], Lysine(K)_iTRAQ[15]	[6] F8 110912	208/200	0.834	0.913	1.038	1	Mascot
1896.0994	1896.0414	-0.058	-31	507	519	KVGYTPDWIFLLR	28	96.528	(N-term)_iTRAQ[0], Lysine(K)_iTRAQ[1]	[6] F8 110912	486/478	0.891	1.043	1.286	1	Mascot
1996.0597	1996.0786	0.0189	9	838	852	GQFSTDELVAEVEKR	78	100	(N-term)_iTRAQ[0], Lysine(K)_iTRAQ[14]	[4] F7 and F10+11	338/330	0.951	0.957	0.831	1	Mascot
2000.0594	2000.0343	-0.0251	-13	1312	1326	AHMGMFTELAILYSK	50	99.975	(N-term)_iTRAQ[0], Lysine(K)_iTRAQ[15]	[6] F8 110912	484/476	0.601	1.103	0.889	1	Mascot
2054.0027	2054.0996	0.0969	47	866	881	IHEGCEEPATHNALAK	104	100	(N-term)_iTRAQ[0], Lysine(K)_iTRAQ[16], MMTS (C)[5]	[4] F7 and F10+11	1084/1076	0.366	1.128	1.246	1	Mascot
2087.0166	2087.0208	0.0042	2	1482	1498	TSIDAYDNFDNISLAQR	32	98.284	(N-term)_iTRAQ[0]	[8] F13-15 and F1+2	446/438	0.822	1.031	0.940	1	Mascot
2185.1084	2185.032	-0.0764	-35	454	468	EDKLECSEELGDLVK	68	100	(N-term)_iTRAQ[0], Lysine(K)_iTRAQ[3,15], MMTS (C)[6]	[6] F8 110912	370/362	0.814	1.034	1.047	1	Mascot
2191.1882	2191.2729	0.0847	39	367	382	KFNALFAQGNYSEAAK	87	100	(N-term)_iTRAQ[0], Lysine(K)_iTRAQ[1,16]	[4] F7 and F10+11	1181/1173	0.922	0.781	1.073	1	Mascot
2235.2192	2235.1763	-0.0429	-19	994	1010	AFMTADLPNELIELLEK	102	100	(N-term)_iTRAQ[0], Lysine(K)_iTRAQ[17]	[1] F3 030912	567/559	1.020	0.931	1.072	1	Mascot
2273.1531	2273.196	0.0429	19	1589	1604	HNIMDFAMPYFIQVMK	104	100	(N-term)_iTRAQ[0], Lysine(K)_iTRAQ[16]	[4] F7 and F10+11	532/524	0.700	1.052	1.061	1	Mascot
2277.2727	2277.3601	0.0874	38	189	205	KVSQPIEGHAASFAQFK	101	100	(N-term)_iTRAQ[0], Lysine(K)_iTRAQ[1,17]	[4] F7 and F10+11	1147/1139	2.524	2.623	3.139	1	Mascot
2291.228	2291.238	0.01	4	951	967	KDPELWGSVLLESNPYR	47	99.951	(N-term)_iTRAQ[0], Lysine(K)_iTRAQ[1]	[4] F7 and F10+11	422/414	1.024	1.070	1.152	1	Mascot
2419.1338	2419.0542	-0.0796	-33	924	941	GQCDLELINVCNENSLFK	90	100	(N-term)_iTRAQ[0], Lysine(K)_iTRAQ[18], MMTS (C)[3,11]	[1] F3 030912	523/515	1.143	1.176	1.129	1	Mascot
2499.2488	2499.2798	0.031	12	1462	1481	SVNESLNNLFITEEDYQALR	28	96.348	(N-term)_iTRAQ[0]	[8] F13-15 and F1+2	386/378	0.759	0.992	1.144	1	Mascot

4	spectrin beta chain, brain 2 [Rattus norvegicus]	gi|9507135	289505.2	38	1932	0.881	0.976	1.010	0.332	0.390	0.484	39	39	39	100	

Protein Group

RecName: Full=Spectrin beta chain, brain 2; AltName: Full=Beta SpIII sigma 1; AltName: Full=Beta-II gi|17367415 289633.2

Peptide Information

Calc. Mass	Obsrv. Mass	± da	± ppm	Start Seq.	End Seq.	Sequence	Ion Score	C. I. %	Modification	Plate [#] Name	Gel Idx/Pos [4700 Sample Name]	iTRAQ Ratio 115/114*	iTRAQ Ratio 116/114*	iTRAQ Ratio 117/114*	Rank	Result Type
1091.6346	1091.5948	-0.0398	-36	1292	1297	LWIDEK	38	99.597	(N-term)_iTRAQ[0], Lysine(K)_iTRAQ[6]	[3] F6 and F9	269/261	0.702	0.925	0.957	1	Mascot
1244.6719	1244.6061	-0.0658	-53	1831	1840	DLNAAEALQR	54	99.99	(N-term)_iTRAQ[0]	[1] F3 030912	201/193	0.771	0.922	1.052	1	Mascot
1345.6985	1345.6135	-0.085	-63	722	731	AAELQAQWER	39	99.662	(N-term)_iTRAQ[0]	[5] F4	146/138	0.647	1.075	1.217	1	Mascot
1367.6385	1367.5881	-0.0504	-37	2312	2321	DEAEMSSWLR	41	99.787	(N-term)_iTRAQ[0]	[5] F4	215/207	0.844	1.006	0.603	1	Mascot
1372.7556	1372.6873	-0.0683	-50	884	893	LEDLEVVQQR	35	99.293	(N-term)_iTRAQ[0]	[1] F3 030912	245/237	0.652	0.919	0.690	1	Mascot
1399.7777	1399.7037	-0.074	-53	1263	1273	NQEAVQQLLGR	40	99.759	(N-term)_iTRAQ[0]	[1] F3 030912	247/239	0.510	0.729	0.917	1	Mascot
1404.7206	1404.7928	0.0722	51	113	121	IHCLENVDK	31	98.123	(N-term)_iTRAQ[0], Lysine(K)_iTRAQ[9], MMTS (C)[3]	[4] F7 and F10+11	1137/1129	0.916	0.913	1.142	1	Mascot
1443.804	1443.7115	-0.0925	-64	929	939	IIGTQEQLNQR	29	96.941	(N-term)_iTRAQ[0]	[1] F3 030912	187/179	0.862	0.716	1.100	1	Mascot
1451.7404	1451.6868	-0.0536	-37	1073	1083	SLDDFQAWLGR	47	99.955	(N-term)_iTRAQ[0]	[5] F4	317/309	1.561	1.707	1.774	1	Mascot
1462.8138	1462.7765	-0.0373	-25	1794	1805	GQVLAAAYELQR	32	98.42	(N-term)_iTRAQ[0]	[1] F3 030912	278/270	0.860	0.892	0.782	1	Mascot
1546.7986	1546.7391	-0.0595	-38	1040	1051	LGEVQTGWEDLR	31	98.17	(N-term)_iTRAQ[0]	[1] F3 030912	318/310	1.003	1.597	1.328	1	Mascot
1554.8074	1554.7385	-0.0689	-44	180	189	DALLLWCQMK	31	98.178	(N-term)_iTRAQ[0], Lysine(K)_iTRAQ[10], MMTS (C)[7]	[5] F4	394/386	1.364	0.935	1.012	1	Mascot
1554.8074	1554.7823	-0.0251	-16	180	189	DALLLWCQMK	39	99.696	(N-term)_iTRAQ[0], Lysine(K)_iTRAQ[10], MMTS (C)[7]	[7] F5 120912	482/474	2.711	3.327	4.912	1	Mascot
1556.8478	1556.9214	0.0736	47	2228	2236	KQEMEAFNK	35	99.254	(N-term)_iTRAQ[0], Lysine(K)_iTRAQ[1,9]	[4] F7 and F10+11	1058/1050	0.995	0.711	0.906	1	Mascot
1567.8325	1567.8855	0.053	34	1869	1880	AYAGDKAEEIGR	33	98.731	(N-term)_iTRAQ[0], Lysine(K)_iTRAQ[6]	[4] F7 and F10+11	1048/1040	1.005	1.011	1.327	1	Mascot
1583.8262	1583.8007	-0.0255	-16	659	671	EQQHLLASAETGR	68	100	(N-term)_iTRAQ[0]	[4] F7 and F10+11	173/165	0.810	0.941	0.850	1	Mascot
1608.8479	1608.8247	-0.0232	-14	2118	2129	VLDTAWDGTQSK	42	99.827	(N-term)_iTRAQ[0],	[7] F5 120912	206/198	0.491	0.757	0.848	1	Mascot

Calc. Mass	Obsrv. Mass	± da	± ppm	Start Seq.	End Seq.	Sequence	Ion Score	C. I. %	Modification	Plate [#] Name	Gel Idx/Pos [4700 Sample Name]	iTRAQ Ratio 115/114*	iTRAQ Ratio 116/114*	iTRAQ Ratio 117/114*	Rank	Result Type
									Lysine(K)_iTRAQ[12]							
1609.8319	1609.7544	-0.0775	-48	1365	1375	WDELETTTQAK	63	99.999	(N-term)_iTRAQ[0], Lysine(K)_iTRAQ[11]	[5] F4	157/149	0.969	0.941	0.856	1	Mascot
1631.9955	1632.0403	0.0448	27	1342	1351	ELTLEKPELK	55	99.992	(N-term)_iTRAQ[0], Lysine(K)_iTRAQ[6,10]	[3] F6 and F9	1208/1200	0.816	1.089	1.133	1	Mascot
1642.7867	1642.7131	-0.0736	-45	1298	1310	MLTAQDVSYDEAR	76	100	(N-term)_iTRAQ[0]	[1] F3 030912	224/216	0.854	0.804	0.784	1	Mascot
1674.9888	1674.9033	-0.0855	-51	908	920	VTAVSDIAEQLLK	37	99.553	(N-term)_iTRAQ[0], Lysine(K)_iTRAQ[13]	[5] F4	335/327	1.426	0.502	0.531	1	Mascot
1720.9817	1721.0658	0.0841	49	1617	1628	AKDELSAQAEVK	81	100	(N-term)_iTRAQ[0], Lysine(K)_iTRAQ[2,12]	[4] F7 and F10+11	1050/1042	0.862	1.405	0.972	1	Mascot
1735.8927	1735.8452	-0.0475	-27	646	658	FLWEVGEAEAWVR	51	99.98	(N-term)_iTRAQ[0]	[1] F3 030912	505/497	0.915	0.867	0.871	1	Mascot
1750.036	1749.9863	-0.0497	-28	363	375	GNLEVLLFTIQSK	37	99.51	(N-term)_iTRAQ[0], Lysine(K)_iTRAQ[13]	[5] F4	371/363	1.539	1.196	1.165	1	Mascot
1757.9796	1757.9419	-0.0377	-21	218	229	HRPDLLDFESLK	33	98.637	(N-term)_iTRAQ[0], Lysine(K)_iTRAQ[12]	[2] F12 040912	359/351	0.732	1.170	0.834	1	Mascot
1776.0238	1776.0948	0.071	40	1436	1447	EKEVEAIQAQAK	85	100	(N-term)_iTRAQ[0], Lysine(K)_iTRAQ[2,12]	[4] F7 and F10+11	1065/1057	0.724	0.932	1.092	1	Mascot
1977.0651	1977.1527	0.0876	44	466	480	KHEAIETDIVAYSGR	37	99.472	(N-term)_iTRAQ[0], Lysine(K)_iTRAQ[1]	[4] F7 and F10+11	1134/1126	0.787	0.881	0.639	1	Mascot
2009.8597	2009.7887	-0.071	-35	26	41	WDLPDSDWDNDSSSAR	54	99.99	(N-term)_iTRAQ[0]	[1] F3 030912	305/297	0.703	0.797	0.676	1	Mascot
2027.0267	2026.9677	-0.059	-29	2038	2053	SAELGCTVDEVESLIK	48	99.959	(N-term)_iTRAQ[0], Lysine(K)_iTRAQ[16], MMTS (C)[6]	[1] F3 030912	486/478	0.539	0.801	1.085	1	Mascot
2057.2593	2057.2551	-0.0042	-2	94	108	LLEVLSGETLPKPTK	61	99.998	(N-term)_iTRAQ[0], Lysine(K)_iTRAQ[12,15]	[4] F7 and F10+11	387/379	0.827	0.861	1.101	1	Mascot
2108.0435	2108.0183	-0.0252	-12	190	206	TAGYPNVNVHNFTTSWR	36	99.317	(N-term)_iTRAQ[0]	[4] F7 and F10+11	329/321	0.939	1.017	0.804	1	Mascot
2116.1272	2116.0242	-0.103	-49	808	826	EQAAALPPALSHTPEVQGR	58	99.996	(N-term)_iTRAQ[0]	[5] F4	158/150	0.704	1.022	0.867	1	Mascot
2116.1648	2116.1877	0.0229	11	1630	1645	HQVLEQALADYAQTIK	66	99.999	(N-term)_iTRAQ[0], Lysine(K)_iTRAQ[16]	[4] F7 and F10+11	411/403	0.828	1.082	1.195	1	Mascot
2156.0791	2156.0635	-0.0156	-7	1881	1899	HMQAVAEAWAQLQGSSAAR	89	100	(N-term)_iTRAQ[0]	[4] F7 and F10+11	381/373	0.818	0.993	1.208	1	Mascot
2304.2156	2304.1553	-0.0603	-26	1553	1571	TLGTAAAGPELAELQEMWK	88	100	(N-term)_iTRAQ[0], Lysine(K)_iTRAQ[19]	[1] F3 030912	468/460	0.872	0.838	0.797	1	Mascot
2355.2415	2355.1921	-0.0494	-21	160	175	FQIQDISVETEDNKEK	37	99.536	(N-term)_iTRAQ[0], Lysine(K)_iTRAQ[14,16]	[4] F7 and F10+11	274/266	1.009	1.152	1.032	1	Mascot
2388.3623	2388.4641	0.1018	43	1629	1645	KHQVLEQALADYAQTIK	75	100	(N-term)_iTRAQ[0], Lysine(K)_iTRAQ[1,17]	[4] F7 and F10+11	1263/1255	0.643	0.817	0.692	1	Mascot
2644.3926	2644.2859	-0.1067	-40	1015	1039	VGELTQEANALAAGHPAQAPAINTR	45	99.924	(N-term)_iTRAQ[0]	[1] F3 030912	318/310	0.990	0.457	1.041	1	Mascot
2989.5715	2989.4666	-0.1049	-35	1084	1111	TQTAVASEEGPATLPEAEALLAQHAALR	102	100	(N-term)_iTRAQ[0]	[1] F3 030912	400/392	1.227	1.939	2.831	1	Mascot

5	sodium/potassium-transporting ATPase subunit alpha-3 [Rattus norvegicus]	gi|6978547	121125.6	30	1783	1.143	0.977	0.951	0.461	0.326	0.333	44	44	44	100

Protein Group

RecName: Full=Sodium/potassium-transporting ATPase subunit alpha-3; Short=Na(+)/K(+) ATPase alpha-3 gi|19855078 121127.5

Peptide Information

Calc. Mass	Obsrv. Mass	± da	± ppm	Start Seq.	End Seq.	Sequence	Ion Score	C. I. %	Modification	Plate [#] Name	Gel Idx/Pos [4700 Sample Name]	iTRAQ Ratio 115/114*	iTRAQ Ratio 116/114*	iTRAQ Ratio 117/114*	Rank	Result Type
1115.667	1115.6121	-0.0549	-49	587	595	AAVPDAVGK	61	99.998	(N-term)_iTRAQ[0], Lysine(K)_iTRAQ[9]	[7] F5 120912	113/105	0.699	0.645	0.635	1	Mascot
1150.7081	1150.6692	-0.0389	-34	757	763	LIFDNLK	36	99.428	(N-term)_iTRAQ[0], Lysine(K)_iTRAQ[7]	[7] F5 120912	289/281	1.128	0.944	0.789	1	Mascot
1174.6466	1174.6395	-0.0071	-6	1002	1009	NPGGWVEK	40	99.736	(N-term)_iTRAQ[0], Lysine(K)_iTRAQ[8]	[3] F6 and F9	188/180	0.886	0.885	1.161	1	Mascot
1220.6597	1220.5994	-0.0603	-49	689	697	LIIVEGCQR	28	96.28	(N-term)_iTRAQ[0], MMTS (C)[7]	[5] F4	234/226	0.711	1.038	0.564	1	Mascot
1220.6597	1220.6172	-0.0425	-35	689	697	LIIVEGCQR	48	99.965	(N-term)_iTRAQ[0], MMTS (C)[7]	[1] F3 030912	310/302	0.968	0.922	0.948	1	Mascot
1328.7783	1328.7262	-0.0521	-39	425	434	GGQDNIPVLK	57	99.995	(N-term)_iTRAQ[0], Lysine(K)_iTRAQ[10]	[7] F5 120912	170/162	1.170	0.964	1.122	1	Mascot
1356.7094	1356.6833	-0.0261	-19	449	458	CIELSSGSVK	34	98.91	(N-term)_iTRAQ[0], Lysine(K)_iTRAQ[10], MMTS (C)[1]	[7] F5 120912	208/200	0.880	0.954	1.005	1	Mascot
1380.8083	1380.7411	-0.0672	-49	638	648	LNIPVSQVNPR	42	99.834	(N-term)_iTRAQ[0]	[1] F3 030912	238/230	1.087	0.795	0.948	1	Mascot
1390.7413	1390.781	0.0397	29	652	661	ACVIHGTDLK	71	100	(N-term)_iTRAQ[0], Lysine(K)_iTRAQ[10], MMTS (C)[2]	[3] F6 and F9	1190/1182	0.941	0.796	0.780	1	Mascot
1390.7413	1390.8002	0.0589	42	652	661	ACVIHGTDLK	72	100	(N-term)_iTRAQ[0], Lysine(K)_iTRAQ[10], MMTS (C)[2]	[4] F7 and F10+11	1119/1111	1.806	1.006	1.672	1	Mascot

1412.8168	1412.7659	-0.0509	-36	153	163	NMVPQQALVIR	30	97.372	(N-term)_iTRAQ[0]	[1] F3 030912	265/257	1.559	1.149	1.196	1	Mascot
1412.8168	1412.8419	0.0251	18	153	163	NMVPQQALVIR	55	99.992	(N-term)_iTRAQ[0]	[8] F13-15 and F1+2	499/491	1.096	1.031	1.016	1	Mascot
1412.8168	1412.8593	0.0425	30	153	163	NMVPQQALVIR	39	99.68	(N-term)_iTRAQ[0]	[8] F13-15 and F1+2	555/547	1.131	0.931	0.923	1	Mascot
1422.9055	1422.95	0.0445	31	757	764	LIFDNLKK	43	99.87	(N-term)_iTRAQ[0], Lysine(K)_iTRAQ[7,8]	[4] F7 and F10+11	1176/1168	1.407	1.125	1.304	1	Mascot
1507.8367	1507.8013	-0.0354	-23	467	477	VAEIPFNSTNK	63	99.999	(N-term)_iTRAQ[0], Lysine(K)_iTRAQ[11]	[7] F5 120912	191/183	1.239	1.096	1.129	1	Mascot
1563.8475	1563.7438	-0.1037	-66	436	448	DVAGDASESALLK	81	100	(N-term)_iTRAQ[0], Lysine(K)_iTRAQ[13]	[5] F4	140/132	1.142	0.960	0.848	1	Mascot
1669.9557	1669.916	-0.0397	-24	603	615	VIMVTGDHPITAK	78	100	(N-term)_iTRAQ[0], Lysine(K)_iTRAQ[13]	[3] F6 and F9	232/224	0.670	0.709	0.702	1	Mascot
1669.9557	1669.9308	-0.0249	-15	603	615	VIMVTGDHPITAK	86	100	(N-term)_iTRAQ[0], Lysine(K)_iTRAQ[13]	[4] F7 and F10+11	234/226	1.185	1.115	1.045	1	Mascot
1674.7317	1674.7208	-0.0109	-7	218	230	SPDCTHDNPLETR	49	99.97	(N-term)_iTRAQ[0], MMTS (C)[4]	[4] F7 and F10+11	201/193	1.479	1.445	0.931	1	Mascot
1719.9486	1720.0229	0.0743	43	435	448	RDVAGDASESALLK	37	99.482	(N-term)_iTRAQ[0], Lysine(K)_iTRAQ[14]	[3] F6 and F9	1159/1151	1.020	0.723	0.680	1	Mascot
1763.8531	1763.7506	-0.1025	-58	203	217	VDNSSLTGESEPQTR	77	100	(N-term)_iTRAQ[0]	[1] F3 030912	136/128	1.082	1.168	1.150	1	Mascot
1780.0341	1780.1221	0.088	49	466	477	KVAEIPFNSTNK	42	99.837	(N-term)_iTRAQ[0], Lysine(K)_iTRAQ[1,12]	[4] F7 and F10+11	1104/1096	2.171	1.822	1.084	1	Mascot
1849.8678	1849.8837	0.0159	9	231	245	NITFFSTNCVEGTAR	31	98.131	(N-term)_iTRAQ[0], MMTS (C)[9]	[8] F13-15 and F1+2	401/393	1.353	1.014	1.538	1	Mascot
1849.8678	1849.8857	0.0179	10	231	245	NITFFSTNCVEGTAR	37	99.502	(N-term)_iTRAQ[0], MMTS (C)[9]	[8] F13-15 and F1+2	404/396	1.217	0.986	0.888	1	Mascot
1974.0264	1974.0244	-0.002	-1	620	637	GVGIISEGNETVEDIAAR	48	99.96	(N-term)_iTRAQ[0]	[8] F13-15 and F1+2	454/446	1.135	0.930	0.924	1	Mascot
1974.0264	1974.0256	-0.0008	0	620	637	GVGIISEGNETVEDIAAR	52	99.984	(N-term)_iTRAQ[0]	[8] F13-15 and F1+2	446/438	1.254	0.827	1.022	1	Mascot
1974.0264	1974.0314	0.005	3	620	637	GVGIISEGNETVEDIAAR	79	100	(N-term)_iTRAQ[0]	[8] F13-15 and F1+2	450/442	1.181	0.894	1.002	1	Mascot
1974.0264	1974.0327	0.0063	3	620	637	GVGIISEGNETVEDIAAR	31	98.048	(N-term)_iTRAQ[0]	[8] F13-15 and F1+2	503/495	1.407	1.001	1.440	1	Mascot
1974.0264	1974.0538	0.0274	14	620	637	GVGIISEGNETVEDIAAR	40	99.731	(N-term)_iTRAQ[0]	[8] F13-15 and F1+2	555/547	0.853	0.908	0.967	1	Mascot
1974.9277	1974.8658	-0.0619	-31	478	492	YQLSIHETEDPNDNR	70	100	(N-term)_iTRAQ[0]	[3] F6 and F9	201/193	1.244	0.964	1.048	1	Mascot
1974.9277	1975.0115	0.0838	42	478	492	YQLSIHETEDPNDNR	57	99.996	(N-term)_iTRAQ[0]	[4] F7 and F10+11	1063/1055	1.152	0.874	0.920	1	Mascot
2042.0165	2042.0784	0.0619	30	404	420	SSHTWVALSHIAGLCNR	99	100	(N-term)_iTRAQ[0], MMTS (C)[15]	[4] F7 and F10+11	1306/1298	0.879	0.991	0.815	1	Mascot
2051.1543	2051.0442	-0.1101	-54	717	733	KADIGVAMGIAGSDVSK	32	98.599	(N-term)_iTRAQ[0], Lysine(K)_iTRAQ[1,17]	[6] F8 110912	275/267	0.542	0.634	0.426	1	Mascot
2072.062	2071.9893	-0.0727	-35	44	57	KYNTDCVQGLTHSK	86	100	(N-term)_iTRAQ[0], Lysine(K)_iTRAQ[1,14], MMTS (C)[6]	[2] F12 040912	247/239	1.053	0.872	1.069	1	Mascot
2100.1545	2100.0515	-0.103	-49	698	716	QGAIVAVTGDGVNDSPALK	112	100	(N-term)_iTRAQ[0], Lysine(K)_iTRAQ[19]	[1] F3 030912	255/247	1.065	0.886	0.737	1	Mascot
2108.1274	2108.0452	-0.0822	-39	65	81	DGPNALTPPPTTPEWVK	57	99.995	(N-term)_iTRAQ[0], Lysine(K)_iTRAQ[17]	[1] F3 030912	289/281	1.020	0.625	0.647	1	Mascot
2160.1831	2160.116	-0.0671	-31	168	184	MQVNAEEVVVGDLVEIK	66	99.999	(N-term)_iTRAQ[0], Lysine(K)_iTRAQ[17]	[1] F3 030912	466/458	1.074	0.810	0.851	1	Mascot
2160.1831	2160.1345	-0.0486	-22	168	184	MQVNAEEVVVGDLVEIK	52	99.985	(N-term)_iTRAQ[0], Lysine(K)_iTRAQ[17]	[2] F12 040912	470/462	1.098	1.428	0.421	1	Mascot
2160.1831	2160.1375	-0.0456	-21	168	184	MQVNAEEVVVGDLVEIK	37	99.515	(N-term)_iTRAQ[0], Lysine(K)_iTRAQ[17]	[1] F3 030912	589/581	1.134	0.659	1.544	1	Mascot
2160.1831	2160.1609	-0.0222	-10	168	184	MQVNAEEVVVGDLVEIK	29	97.236	(N-term)_iTRAQ[0], Lysine(K)_iTRAQ[17]	[1] F3 030912	657/649	1.040	2.087	1.216	1	Mascot
2202.0859	2202.0366	-0.0493	-22	350	367	NLEAVETLGSTSTICSDK	99	100	(N-term)_iTRAQ[0], Lysine(K)_iTRAQ[18], MMTS (C)[15]	[1] F3 030912	363/355	1.008	0.712	0.912	1	Mascot
2372.3521	2372.2949	-0.0572	-24	698	717	QGAIVAVTGDGVNDSPALKK	30	97.508	(N-term)_iTRAQ[0], Lysine(K)_iTRAQ[19,20]	[4] F7 and F10+11	246/238	0.887	0.802	0.929	1	Mascot
2376.0864	2375.9856	-0.1008	-42	884	901	TVNDLEDSYGQQWTYEQR	39	99.707	(N-term)_iTRAQ[0]	[1] F3 030912	293/285	3.619	1.464	1.061	1	Mascot
2608.3049	2608.2537	-0.0512	-20	734	756	QAADMILLDDNFASIVTGVEEGR	55	99.992	(N-term)_iTRAQ[0]	[1] F3 030912	548/540	3.111	2.449	1.643	1	Mascot

6 cytoplasmic dynein 1 heavy chain 1 [Rattus norvegicus] gi|148491097 576717.4 35 1725 1.040 0.926 0.931 0.326 0.310 0.301 38 38 38 100

Peptide Information

Calc. Mass	Obsrv. Mass	± da	± ppm	Start Seq.	End Seq.	Sequence	Ion Score	C. I. %	Modification	Plate [#] Name	Gel Idx/Pos [4700 Sample Name]	iTRAQ Ratio 115/114*	iTRAQ Ratio 116/114*	iTRAQ Ratio 117/114*	Rank	Result Type
1233.6382	1233.576	-0.0622	-50	3428	3436	ANEVEQMIR	28	96.288	(N-term)_iTRAQ[0]	[5] F4	155/147	0.935	1.099	0.772	1	Mascot
1282.6677	1282.6555	-0.0122	-10	1487	1494	GWDDLFNK	36	99.348	(N-term)_iTRAQ[0], Lysine(K)_iTRAQ[8]	[3] F6 and F9	331/323	1.251	0.795	1.253	1	Mascot
1322.7189	1322.6381	-0.0808	-61	601	609	EYQTQLIQR	43	99.879	(N-term)_iTRAQ[0]	[1] F3 030912	185/177	0.968	0.708	0.943	1	Mascot
1327.6589	1327.5944	-0.0645	-49	1833	1841	SFEWLSQMR	42	99.836	(N-term)_iTRAQ[0]	[5] F4	282/274	1.396	0.886	1.015	1	Mascot
1369.7349	1369.6476	-0.0873	-64	1346	1355	EQPWVSVQPR	56	99.994	(N-term)_iTRAQ[0]	[5] F4	151/143	0.882	0.870	1.089	1	Mascot

Calc. Mass	Obsrv. Mass	± da	± ppm	Start Seq.	End Seq.	Sequence	Ion Score	C. I. %	Modification	Plate [#] Name	Gel Idx/Pos [4700 Sample Name]	iTRAQ Ratio 115/114*	iTRAQ Ratio 116/114*	iTRAQ Ratio 117/114*	Rank	Result Type
1369.7349	1369.6693	-0.0656	-48	1346	1355	EQPWVSVQPR	40	99.76	(N-term)_iTRAQ[0]	[1] F3 030912	221/213	0.635	0.578	0.678	1	Mascot
1412.8499	1412.8789	0.029	21	353	363	QALVAIFTHLR	44	99.896	(N-term)_iTRAQ[0]	[4] F7 and F10+11	449/441	0.950	0.846	0.855	1	Mascot
1434.7137	1434.6248	-0.0889	-62	1377	1386	QYASYEFVQR	36	99.381	(N-term)_iTRAQ[0]	[5] F4	152/144	0.615	0.957	0.599	1	Mascot
1464.7766	1464.7222	-0.0544	-37	1337	1345	VWEQIDQMK	52	99.986	(N-term)_iTRAQ[0], Lysine(K)_iTRAQ[9]	[7] F5 120912	273/265	1.000	0.853	1.513	1	Mascot
1485.7421	1485.6907	-0.0514	-35	623	632	VQYPQSQACK	55	99.993	(N-term)_iTRAQ[0], Lysine(K)_iTRAQ[10], MMTS (C)[9]	[7] F5 120912	152/144	1.162	0.910	0.948	1	Mascot
1503.6724	1503.5947	-0.0777	-52	2965	2975	YTGEDFDEDLR	28	95.768	(N-term)_iTRAQ[0]	[1] F3 030912	235/227	1.172	1.600	1.337	1	Mascot
1510.7457	1510.7277	-0.018	-12	539	549	EGTEAWEAAMK	37	99.543	(N-term)_iTRAQ[0], Lysine(K)_iTRAQ[11]	[7] F5 120912	208/200	1.078	1.223	1.114	1	Mascot
1520.7717	1520.7822	0.0105	7	2878	2888	DYIPVDQEELR	69	100	(N-term)_iTRAQ[0]	[8] F13-15 and F1+2	516/508	1.171	0.856	0.664	1	Mascot
1520.7717	1520.7966	0.0249	16	2878	2888	DYIPVDQEELR	46	99.932	(N-term)_iTRAQ[0]	[8] F13-15 and F1+2	565/557	1.495	1.342	0.823	1	Mascot
1531.8002	1531.8832	0.083	54	4202	4211	KYEFGESDLR	42	99.836	(N-term)_iTRAQ[0], Lysine(K)_iTRAQ[1]	[4] F7 and F10+11	1105/1097	1.147	0.989	1.092	1	Mascot
1543.9054	1543.8843	-0.0211	-14	1359	1369	QNLDGLLNQLK	37	99.543	(N-term)_iTRAQ[0], Lysine(K)_iTRAQ[11]	[7] F5 120912	341/333	0.790	0.564	0.542	1	Mascot
1602.7731	1602.6831	-0.09	-56	2382	2394	SIPLDEGEDEAQR	41	99.801	(N-term)_iTRAQ[0]	[1] F3 030912	184/176	1.103	0.696	1.558	1	Mascot
1614.8934	1614.8022	-0.0912	-56	3922	3935	VQGLTVEQAEAVAR	28	96.005	(N-term)_iTRAQ[0]	[1] F3 030912	254/246	0.743	0.778	0.958	1	Mascot
1617.851	1617.8638	0.0128	8	2272	2283	EWTDGLFTHVLR	30	97.502	(N-term)_iTRAQ[0]	[4] F7 and F10+11	407/399	0.985	1.113	0.647	1	Mascot
1651.8201	1651.7542	-0.0659	-40	691	702	LNTQEIFDDWAR	44	99.911	(N-term)_iTRAQ[0]	[5] F4	316/308	0.711	0.750	0.827	1	Mascot
1683.8662	1683.931	0.0648	38	399	409	LMHVAYEEFEK	71	100	(N-term)_iTRAQ[0], Lysine(K)_iTRAQ[11]	[3] F6 and F9	1271/1263	1.677	1.179	0.727	1	Mascot
1722.8982	1722.8431	-0.0551	-32	2843	2854	WTDENIDMVALK	50	99.973	(N-term)_iTRAQ[0], Lysine(K)_iTRAQ[12]	[5] F4	260/252	1.838	1.566	2.022	1	Mascot
1755.8687	1755.792	-0.0767	-44	1513	1524	VFEEDALSWEDK	55	99.991	(N-term)_iTRAQ[0], Lysine(K)_iTRAQ[12]	[5] F4	253/245	0.696	0.575	0.599	1	Mascot
1758.8743	1758.8462	-0.0281	-16	2382	2395	SIPLDEGEDEAQRR	40	99.76	(N-term)_iTRAQ[0]	[4] F7 and F10+11	178/170	1.163	0.702	0.821	1	Mascot
1802.9972	1802.9464	-0.0508	-28	1566	1578	FQSISTEFLALMK	60	99.997	(N-term)_iTRAQ[0], Lysine(K)_iTRAQ[13]	[1] F3 030912	521/513	0.783	1.272	0.940	1	Mascot
1896.9365	1896.8912	-0.0453	-24	2782	2795	FTQDTQPHYIYSPR	35	99.146	(N-term)_iTRAQ[0]	[3] F6 and F9	229/221	0.982	0.758	0.795	1	Mascot
1924.0724	1923.9906	-0.0818	-43	4096	4109	NVHLAPGWLMQLEK	59	99.997	(N-term)_iTRAQ[0], Lysine(K)_iTRAQ[14]	[6] F8 110912	415/407	1.378	1.215	0.907	1	Mascot
1924.0724	1924.0879	0.0155	8	4096	4109	NVHLAPGWLMQLEK	64	99.999	(N-term)_iTRAQ[0], Lysine(K)_iTRAQ[14]	[4] F7 and F10+11	411/403	1.010	1.254	0.909	1	Mascot
1926.9828	1926.9832	0.0004	0	3190	3204	SELEEQQMHLNVGLR	42	99.834	(N-term)_iTRAQ[0]	[3] F6 and F9	281/273	1.314	0.924	1.358	1	Mascot
1956.0636	1956.0809	0.0173	9	398	409	KLMHVAYEEFEK	73	100	(N-term)_iTRAQ[0], Lysine(K)_iTRAQ[1,12]	[2] F12 040912	322/314	1.299	1.057	1.185	1	Mascot
1974.9016	1974.8224	-0.0792	-40	4212	4226	SACDTVDTWLDDTAK	48	99.956	(N-term)_iTRAQ[0], Lysine(K)_iTRAQ[15], MMTS (C)[3]	[5] F4	313/305	1.423	0.969	0.898	1	Mascot
1979.0583	1979.0092	-0.0491	-25	336	350	DFPLNDLLSATELDK	83	100	(N-term)_iTRAQ[0], Lysine(K)_iTRAQ[15]	[1] F3 030912	468/460	0.605	0.872	0.734	1	Mascot
2005.1315	2005.052	-0.0795	-40	3758	3772	ILDDDTIITTLENLK	78	100	(N-term)_iTRAQ[0], Lysine(K)_iTRAQ[15]	[1] F3 030912	528/520	1.295	1.519	1.196	1	Mascot
2012.0222	2011.9768	-0.0454	-23	1002	1015	YQVGVHYELTEEEK	52	99.983	(N-term)_iTRAQ[0], Lysine(K)_iTRAQ[14]	[4] F7 and F10+11	246/238	1.262	0.860	0.838	1	Mascot
2139.0967	2139.0757	-0.021	-10	1513	1527	VFEEDALSWEDKLNR	55	99.992	(N-term)_iTRAQ[0], Lysine(K)_iTRAQ[12]	[4] F7 and F10+11	401/393	1.152	0.445	1.017	1	Mascot
2247.1794	2247.123	-0.0564	-25	1627	1643	FYFVGDEDLLEIIGNSK	71	100	(N-term)_iTRAQ[0], Lysine(K)_iTRAQ[17]	[1] F3 030912	533/525	1.001	1.140	0.823	1	Mascot
2276.1438	2276.1138	-0.03	-13	2398	2415	GKEDEGEEAASPMLQIQR	62	99.998	(N-term)_iTRAQ[0], Lysine(K)_iTRAQ[2]	[4] F7 and F10+11	225/217	1.166	1.094	1.021	1	Mascot
2287.292	2287.3037	0.0117	5	3445	3460	YKEEYAVLISEAQAIK	56	99.994	(N-term)_iTRAQ[0], Lysine(K)_iTRAQ[2,16]	[4] F7 and F10+11	430/422	0.743	0.840	0.861	1	Mascot

7	microtubule-associated protein 2 [Rattus norvegicus]	gi|6981182	220959.3	28	1521	0.920	1.067	1.089	0.343	0.305	0.392	32	32	32	100

Peptide Information

Calc. Mass	Obsrv. Mass	± da	± ppm	Start Seq.	End Seq.	Sequence	Ion Score	C. I. %	Modification	Plate [#] Name	Gel Idx/Pos [4700 Sample Name]	iTRAQ Ratio 115/114*	iTRAQ Ratio 116/114*	iTRAQ Ratio 117/114*	Rank	Result Type
1246.6929	1246.6686	-0.0243	-19	568	575	YFETSALK	31	97.965	(N-term)_iTRAQ[0], Lysine(K)_iTRAQ[8]	[7] F5 120912	211/203	0.626	0.857	1.100	1	Mascot
1324.6816	1324.62	-0.0616	-47	155	162	MEFPEQQK	49	99.968	(N-term)_iTRAQ[0], Lysine(K)_iTRAQ[8]	[3] F6 and F9	193/185	1.156	1.019	1.775	1	Mascot
1416.7244	1416.6552	-0.0692	-49	1314	1324	FAAPVQPEEER	50	99.973	(N-term)_iTRAQ[0]	[1] F3 030912	194/186	0.819	1.183	1.087	1	Mascot
1416.7244	1416.7628	0.0384	27	1314	1324	FAAPVQPEEER	34	99.015	(N-term)_iTRAQ[0]	[8] F13-15 and F1+2	586/578	0.943	1.166	0.636	1	Mascot
1416.7244	1416.7806	0.0562	40	1314	1324	FAAPVQPEEER	29	96.804	(N-term)_iTRAQ[0]	[8] F13-15 and F1+2	583/575	0.825	1.074	1.061	1	Mascot
1498.8489	1498.9106	0.0617	41	924	932	VKDEFTAEK	54	99.99	(N-term)_iTRAQ[0], Lysine(K)_iTRAQ[2,9]	[4] F7 and F10+11	1063/1055	0.678	1.115	1.049	1	Mascot
1501.8472	1501.9095	0.0623	41	290	299	EKDVLEDIPR	70	100	(N-term)_iTRAQ[0], Lysine(K)_iTRAQ[2]	[3] F6 and F9	1220/1212	1.080	1.000	0.964	1	Mascot

1590.9075	1590.9686	0.0611	38	1043	1052	KDDQSPLDIK	42	99.837	(N-term)_iTRAQ[0], Lysine(K)_iTRAQ[1,10]	[4] F7 and F10+11	1072/1064	0.771	0.917	1.348	1	Mascot
1600.1387	1600.0826	-0.0561	-35	1486	1494	KLILKPAIK	41	99.824	(N-term)_iTRAQ[0], Lysine(K)_iTRAQ[1,5,9]	[2] F12 040912	247/239	1.242	1.480	1.415	1	Mascot
1619.885	1619.7651	-0.1199	-74	516	527	VTSEPEAVSEKR	33	98.689	(N-term)_iTRAQ[0], Lysine(K)_iTRAQ[11]	[6] F8 110912	130/122	1.451	1.109	2.089	1	Mascot
1633.8643	1633.7664	-0.0979	-60	596	608	GNAQESLDTVSPK	73	100	(N-term)_iTRAQ[0], Lysine(K)_iTRAQ[13]	[5] F4	104/96	0.634	0.736	0.645	1	Mascot
1633.8643	1633.8213	-0.043	-26	596	608	GNAQESLDTVSPK	65	99.999	(N-term)_iTRAQ[0], Lysine(K)_iTRAQ[13]	[7] F5 120912	136/128	2.001	1.410	1.278	1	Mascot
1653.868	1653.8567	-0.0113	-7	1770	1783	VDHGAEIITQSPSR	36	99.318	(N-term)_iTRAQ[0]	[4] F7 and F10+11	169/161	0.852	0.986	0.902	1	Mascot
1668.8553	1668.7684	-0.0869	-52	260	270	DWFIEMPVESK	63	99.999	(N-term)_iTRAQ[0], Lysine(K)_iTRAQ[11]	[5] F4	298/290	0.717	0.865	0.797	1	Mascot
1720.9553	1720.8921	-0.0632	-37	1193	1204	AEVQMEFIQLPK	36	99.317	(N-term)_iTRAQ[0], Lysine(K)_iTRAQ[12]	[5] F4	274/266	0.971	0.780	0.599	1	Mascot
1728.088	1728.0371	-0.0509	-29	94	107	IVQVVTAEAVAVLK	75	100	(N-term)_iTRAQ[0], Lysine(K)_iTRAQ[14]	[1] F3 030912	523/515	1.434	0.841	1.187	1	Mascot
1738.8007	1738.696	-0.1047	-60	1270	1281	EEFVETCPGEHK	53	99.987	(N-term)_iTRAQ[0], Lysine(K)_iTRAQ[12], MMTS (C)[7]	[6] F8 110912	229/221	1.257	1.192	1.411	1	Mascot
1794.9371	1794.8988	-0.0383	-21	528	540	EIQGLFEEDIADK	50	99.975	(N-term)_iTRAQ[0], Lysine(K)_iTRAQ[13]	[5] F4	266/258	0.978	0.760	0.819	1	Mascot
1841.9491	1841.8363	-0.1128	-61	1508	1523	TTATSGESAQAPSAFK	90	100	(N-term)_iTRAQ[0], Lysine(K)_iTRAQ[16]	[5] F4	104/96	1.009	1.291	1.284	1	Mascot
1846.9796	1846.9247	-0.0549	-30	568	580	YFETSALKEDVTR	63	99.999	(N-term)_iTRAQ[0], Lysine(K)_iTRAQ[8]	[4] F7 and F10+11	287/279	0.898	0.925	1.322	1	Mascot
1914.8701	1914.7356	-0.1345	-70	1237	1250	GEEEEIEAEGEYDK	66	99.999	(N-term)_iTRAQ[0], Lysine(K)_iTRAQ[14]	[5] F4	123/115	1.109	1.099	1.156	1	Mascot
1935.0532	1934.9753	-0.0779	-40	1017	1032	GLSSVPEVAEVETTTK	48	99.965	(N-term)_iTRAQ[0], Lysine(K)_iTRAQ[16]	[1] F3 030912	275/267	0.948	1.454	1.430	1	Mascot
1974.951	1974.8635	-0.0875	-44	1053	1069	VSDFGQMASGMSVDAGK	56	99.994	(N-term)_iTRAQ[0], Lysine(K)_iTRAQ[17]	[5] F4	207/199	1.123	1.329	1.270	1	Mascot
2050.104	2049.9949	-0.1091	-53	478	491	EDQKGEEQTIEALK	42	99.847	(N-term)_iTRAQ[0], Lysine(K)_iTRAQ[4,14]	[6] F8 110912	220/212	0.556	0.531	0.640	1	Mascot
2050.104	2050.1531	0.0491	24	478	491	EDQKGEEQTIEALK	39	99.707	(N-term)_iTRAQ[0], Lysine(K)_iTRAQ[4,14]	[3] F6 and F9	1179/1171	1.212	1.483	1.200	1	Mascot
2114.1465	2114.0139	-0.1326	-63	1507	1523	KTTATSGESAQAPSAFK	32	98.303	(N-term)_iTRAQ[0], Lysine(K)_iTRAQ[1,17]	[6] F8 110912	151/143	1.012	1.731	1.432	1	Mascot
2211.1543	2211.0891	-0.0652	-29	217	234	SPASPFAQTFGTNLEDIK	50	99.975	(N-term)_iTRAQ[0], Lysine(K)_iTRAQ[18]	[1] F3 030912	380/372	0.590	1.544	0.648	1	Mascot
2339.1978	2339.0884	-0.1094	-47	774	793	VTGGQTTQVETSSESPFPAK	120	100	(N-term)_iTRAQ[0], Lysine(K)_iTRAQ[20]	[1] F3 030912	213/205	0.998	1.104	1.474	1	Mascot
2399.2717	2399.2705	-0.0012	-1	163	179	LPSSFAEPLDKEETEFK	35	99.244	(N-term)_iTRAQ[0], Lysine(K)_iTRAQ[11,17]	[4] F7 and F10+11	356/348	0.898	0.930	1.077	1	Mascot
2411.2373	2411.167	-0.0703	-29	492	511	QDSFPISLEQAVTDAAMATK	50	99.976	(N-term)_iTRAQ[0], Lysine(K)_iTRAQ[20]	[1] F3 030912	508/500	0.387	1.309	1.305	1	Mascot
2644.3188	2644.2927	-0.0261	-10	11	32	APHWTSASLTEAAAHPHSPEMK	57	99.995	(N-term)_iTRAQ[0], Lysine(K)_iTRAQ[22]	[2] F12 040912	277/269	0.746	1.105	0.855	1	Mascot
3661.7754	3661.635	-0.1404	-38	874	904	YTVPLPSPVQDSENLSGESGSFYEGTDDKVR	60	99.998	(N-term)_iTRAQ[0], Lysine(K)_iTRAQ[29]	[1] F3 030912	352/344	1.016	0.877	1.213	1	Mascot

8 heat shock cognate 71 kDa protein [Rattus norvegicus] gi|13242237 78936.8 21 1488 0.996 0.990 1.029 0.238 0.287 0.224 33 33 33 100

Protein Group

dnaK-type molecular chaperone hsp72-ps1 - rat gi|347019 78993.8

Peptide Information

Calc. Mass	Obsrv. Mass	± da	± ppm	Start Seq.	End Seq.	Sequence	Ion Score	C. I. %	Modification	Plate [#] Name	Gel Idx/Pos [4700 Sample Name]	iTRAQ Ratio 115/114*	iTRAQ Ratio 116/114*	iTRAQ Ratio 117/114*	Rank	Result Type
1221.6926	1221.6371	-0.0555	-45	602	609	VCNPIITK	41	99.803	(N-term)_iTRAQ[0], Lysine(K)_iTRAQ[8], MMTS (C)[2]	[7] F5 120912	219/211	1.014	0.867	0.909	1	Mascot
1281.73	1281.7069	-0.0231	-18	129	137	EIAEAYLGK	52	99.985	(N-term)_iTRAQ[0], Lysine(K)_iTRAQ[9]	[7] F5 120912	201/193	1.069	1.178	0.996	1	Mascot
1343.7767	1343.7209	-0.0558	-42	160	171	DAGTIAGLNVLR	63	99.999	(N-term)_iTRAQ[0]	[1] F3 030912	296/288	0.895	0.802	0.915	1	Mascot
1397.7185	1397.6718	-0.0467	-33	302	311	FEELNADLFR	64	99.999	(N-term)_iTRAQ[0]	[1] F3 030912	370/362	1.021	1.082	0.872	1	Mascot
1523.829	1523.8643	0.0353	23	237	246	MVNHFIAEFK	69	100	(N-term)_iTRAQ[0], Lysine(K)_iTRAQ[10]	[3] F6 and F9	1309/1301	0.732	0.665	0.739	1	Mascot
1523.829	1523.8849	0.0559	37	237	246	MVNHFIAEFK	70	100	(N-term)_iTRAQ[0], Lysine(K)_iTRAQ[10]	[4] F7 and F10+11	1248/1240	1.347	1.224	1.262	1	Mascot
1539.824	1539.8489	0.0249	16	237	246	MVNHFIAEFK	35	99.235	(N-term)_iTRAQ[0], Lysine(K)_iTRAQ[10], Oxidation (M)[1]	[3] F6 and F9	1255/1247	0.690	0.717	0.974	1	Mascot
1539.824	1539.884	0.06	39	237	246	MVNHFIAEFK	44	99.9	(N-term)_iTRAQ[0], Lysine(K)_iTRAQ[10], Oxidation (M)[1]	[4] F7 and F10+11	1193/1185	0.760	0.803	1.101	1	Mascot

1542.772	1542.691	-0.081	-53	78	88	FDDAVVQSDMK	84	100	(N-term)_iTRAQ[0], Lysine(K)_iTRAQ[11]	[5] F4	153/145	1.443	1.094	1.135	1	Mascot
1554.7887	1554.7954	0.0067	4	574	583	CNEIISWLDK	37	99.531	(N-term)_iTRAQ[0], Lysine(K)_iTRAQ[10], MMTS (C)[1]	[7] F5 120912	453/445	0.854	0.800	1.052	1	Mascot
1554.7887	1554.8456	0.0569	37	574	583	CNEIISWLDK	50	99.977	(N-term)_iTRAQ[0], Lysine(K)_iTRAQ[10], MMTS (C)[1]	[7] F5 120912	468/460	0.946	0.764	1.016	1	Mascot
1591.8036	1591.7517	-0.0519	-33	540	550	NSLESYAFNMK	52	99.983	(N-term)_iTRAQ[0], Lysine(K)_iTRAQ[11]	[7] F5 120912	284/276	1.198	1.171	1.330	1	Mascot
1612.9281	1613.0043	0.0762	47	103	112	VQVEYKGETK	71	100	(N-term)_iTRAQ[0], Lysine(K)_iTRAQ[6,10]	[4] F7 and F10+11	1045/1037	1.098	0.853	1.241	1	Mascot
1625.9095	1625.8794	-0.0301	-19	329	342	SQIHDIVLVGGSTR	83	100	(N-term)_iTRAQ[0]	[4] F7 and F10+11	238/230	1.040	1.206	0.908	1	Mascot
1631.8037	1631.7163	-0.0874	-54	37	49	TTPSYVAFTDTER	55	99.993	(N-term)_iTRAQ[0]	[1] F3 030912	233/225	0.967	1.012	1.106	1	Mascot
1631.8037	1631.8102	0.0065	4	37	49	TTPSYVAFTDTER	64	99.999	(N-term)_iTRAQ[0]	[8] F13-15 and F1+2	527/519	0.721	0.876	0.799	1	Mascot
1684.9679	1685.0229	0.055	33	127	137	MKEIAEAYLGK	65	99.999	(N-term)_iTRAQ[0], Lysine(K)_iTRAQ[2,11]	[4] F7 and F10+11	1220/1212	1.123	0.903	1.145	1	Mascot
1698.873	1699.0149	0.1419	84	77	88	RFDDAVVQSDMK	63	99.999	(N-term)_iTRAQ[0], Lysine(K)_iTRAQ[12]	[4] F7 and F10+11	1102/1094	1.062	0.884	1.057	1	Mascot
1835.828	1835.823	-0.005	-3	221	236	STAGDTHLGGEDFDNR	88	100	(N-term)_iTRAQ[0]	[4] F7 and F10+11	165/157	1.319	1.055	1.153	1	Mascot
1904.9926	1904.9669	-0.0257	-13	113	126	SFYPEEVSSMVLTK	85	100	(N-term)_iTRAQ[0], Lysine(K)_iTRAQ[14]	[1] F3 030912	373/365	1.165	1.208	1.128	1	Mascot
1938.0001	1937.8787	-0.1214	-63	57	71	NQVAMNPTNTVFDAK	72	100	(N-term)_iTRAQ[0], Lysine(K)_iTRAQ[15]	[5] F4	175/167	0.803	1.042	0.942	1	Mascot
1938.0001	1937.8979	-0.1022	-53	57	71	NQVAMNPTNTVFDAK	78	100	(N-term)_iTRAQ[0], Lysine(K)_iTRAQ[15]	[1] F3 030912	246/238	0.952	0.786	0.826	1	Mascot
1942.0367	1942.0227	-0.014	-7	89	102	HWPFMVVNDAGRPK	65	99.999	(N-term)_iTRAQ[0], Lysine(K)_iTRAQ[14]	[2] F12 040912	338/330	1.149	1.020	1.103	1	Mascot
1942.0367	1942.1321	0.0954	49	89	102	HWPFMVVNDAGRPK	39	99.678	(N-term)_iTRAQ[0], Lysine(K)_iTRAQ[14]	[4] F7 and F10+11	1206/1198	0.972	0.945	1.247	1	Mascot
1958.0316	1957.9885	-0.0431	-22	89	102	HWPFMVVNDAGRPK	28	96.077	(N-term)_iTRAQ[0], Lysine(K)_iTRAQ[14], Oxidation (M)[5]	[2] F12 040912	301/293	0.928	0.695	0.862	1	Mascot
2126.1001	2126.0967	-0.0034	-2	138	155	TVTNAVVTVPAYFNDSQR	49	99.969	(N-term)_iTRAQ[0]	[8] F13-15 and F1+2	454/446	0.813	0.838	0.765	1	Mascot
2126.1001	2126.1035	0.0034	2	138	155	TVTNAVVTVPAYFNDSQR	35	99.233	(N-term)_iTRAQ[0]	[8] F13-15 and F1+2	450/442	1.258	1.121	1.131	1	Mascot
2126.1001	2126.1104	0.0103	5	138	155	TVTNAVVTVPAYFNDSQR	41	99.811	(N-term)_iTRAQ[0]	[8] F13-15 and F1+2	503/495	0.787	2.212	1.625	1	Mascot
2126.1001	2126.1316	0.0315	15	138	155	TVTNAVVTVPAYFNDSQR	38	99.644	(N-term)_iTRAQ[0]	[8] F13-15 and F1+2	555/547	0.702	0.817	0.598	1	Mascot
2178.1162	2178.0076	-0.1086	-50	584	597	NQTAEKEEFEHQQK	69	100	(N-term)_iTRAQ[0], Lysine(K)_iTRAQ[6,14]	[2] F12 040912	136/128	1.085	1.149	1.141	1	Mascot
2178.1162	2178.1909	0.0747	34	584	597	NQTAEKEEFEHQQK	55	99.992	(N-term)_iTRAQ[0], Lysine(K)_iTRAQ[6,14]	[2] F12 040912	150/142	0.992	1.464	1.357	1	Mascot
2220.2974	2220.2842	-0.0132	-6	172	188	IINEPTAAAIAYGLDKK	106	100	(N-term)_iTRAQ[0], Lysine(K)_iTRAQ[16,17]	[4] F7 and F10+11	369/361	1.010	0.896	1.002	1	Mascot
2548.3503	2548.2507	-0.0996	-39	362	384	SINPDEAVAYGAAVQAAILSGDK	129	100	(N-term)_iTRAQ[0], Lysine(K)_iTRAQ[23]	[1] F3 030912	488/480	1.747	1.699	1.159	1	Mascot

9	microtubule-associated protein 1A [Rattus norvegicus]	gi|13591886	328211.6	27	1479	1.186	0.968	1.032	0.421	0.342	0.486	29	29	29	100

Peptide Information

Calc. Mass	Obsrv. Mass	± da	± ppm	Start Seq.	End Seq.	Sequence	Ion Score	C. I. %	Modification	Plate [#] Name	Gel Idx/Pos [4700 Sample Name]	iTRAQ Ratio 115/114*	iTRAQ Ratio 116/114*	iTRAQ Ratio 117/114*	Rank	Result Type
1260.6458	1260.6028	-0.043	-34	1611	1619	EQDVVQGWR	55	99.992	(N-term)_iTRAQ[0]	[7] F5 120912	170/162	1.041	1.108	0.970	1	Mascot
1300.7147	1300.6643	-0.0504	-39	1634	1642	EPVPAWEGK	38	99.62	(N-term)_iTRAQ[0], Lysine(K)_iTRAQ[9]	[7] F5 120912	170/162	1.336	1.552	1.560	1	Mascot
1303.7494	1303.6945	-0.0549	-42	169	178	LGIQAEPLYR	38	99.576	(N-term)_iTRAQ[0]	[1] F3 030912	265/257	1.332	0.840	0.962	1	Mascot
1342.7109	1342.6799	-0.031	-23	211	218	EMQFLMQK	44	99.892	(N-term)_iTRAQ[0], Lysine(K)_iTRAQ[8]	[7] F5 120912	255/247	1.859	1.113	1.163	1	Mascot
1575.8475	1575.7338	-0.1137	-72	1588	1600	ALGLEESPAEGSK	62	99.998	(N-term)_iTRAQ[0], Lysine(K)_iTRAQ[13]	[5] F4	120/112	1.214	0.821	1.200	1	Mascot
1629.7643	1629.6859	-0.0784	-48	1017	1027	DFQEDSWGETK	61	99.998	(N-term)_iTRAQ[0], Lysine(K)_iTRAQ[11]	[7] F5 120912	174/166	1.170	1.010	0.913	1	Mascot
1721.8579	1721.76	-0.0979	-57	1474	1487	APEHSIPEPTQTDR	33	98.871	(N-term)_iTRAQ[0]	[3] F6 and F9	145/137	0.625	0.802	0.664	1	Mascot
1721.8579	1721.833	-0.0249	-14	1474	1487	APEHSIPEPTQTDR	52	99.983	(N-term)_iTRAQ[0]	[4] F7 and F10+11	145/137	1.700	1.161	1.300	1	Mascot
1748.8912	1748.7815	-0.1097	-63	1572	1585	ADSVEQQDGAALEK	102	100	(N-term)_iTRAQ[0], Lysine(K)_iTRAQ[14]	[5] F4	101/93	1.391	1.128	1.172	1	Mascot
1780.0579	1780.0029	-0.055	-31	1346	1358	VAVVEQDLIIHQK	48	99.962	(N-term)_iTRAQ[0], Lysine(K)_iTRAQ[13]	[4] F7 and F10+11	292/284	1.676	1.105	1.117	1	Mascot
1816.915	1816.8994	-0.0156	-9	155	168	SIEEACLTLQHLNR	43	99.865	(N-term)_iTRAQ[0], MMTS (C)[6]	[3] F6 and F9	431/423	0.842	0.611	0.915	1	Mascot
1823.916	1823.8181	-0.0979	-54	1653	1665	DRDITLQQDAYWR	43	99.875	(N-term)_iTRAQ[0]	[6] F8 110912	268/260	1.141	1.120	1.268	1	Mascot

1867.892	1867.7646	-0.1274	-68	1883	1899	EGEGGAGAPDSSSFSPK	127	100	(N-term)_iTRAQ[0], Lysine(K)_iTRAQ[17]	[5] F4	97/89	1.214	1.012	0.712	1	Mascot
1876.856	1876.9257	0.0697	37	2068	2081	GHWDDGTNDSDLEK	83	100	(N-term)_iTRAQ[0], Lysine(K)_iTRAQ[14]	[4] F7 and F10+11	1060/1052	1.158	0.908	1.488	1	Mascot
1886.0997	1886.0616	-0.0381	-20	179	192	VVSNTIEPLTLFHK	28	95.958	(N-term)_iTRAQ[0], Lysine(K)_iTRAQ[14]	[3] F6 and F9	375/367	1.200	0.647	0.907	1	Mascot
1929.1154	1929.0237	-0.0917	-48	365	376	EPSEKPPEKPSK	44	99.908	(N-term)_iTRAQ[0], Lysine(K)_iTRAQ[5,9,12]	[2] F12 040912	102/94	1.420	1.185	0.869	1	Mascot
1945.875	1945.7621	-0.1129	-58	907	921	SPPCEDFSVTGESEK	57	99.995	(N-term)_iTRAQ[0], Lysine(K)_iTRAQ[15], MMTS (C)[4]	[5] F4	193/185	0.836	0.906	1.008	1	Mascot
2059.2288	2059.2915	0.0627	30	459	472	LSKPDLKPFTPEVR	35	99.161	(N-term)_iTRAQ[0], Lysine(K)_iTRAQ[3,7]	[4] F7 and F10+11	1174/1166	1.050	1.152	0.970	1	Mascot
2121.092	2121.0349	-0.0571	-27	1112	1127	TSTEEATEPQKDEVLR	43	99.875	(N-term)_iTRAQ[0], Lysine(K)_iTRAQ[11]	[3] F6 and F9	174/166	0.952	0.658	0.779	1	Mascot
2121.092	2121.0737	-0.0183	-9	1112	1127	TSTEEATEPQKDEVLR	79	100	(N-term)_iTRAQ[0], Lysine(K)_iTRAQ[11]	[4] F7 and F10+11	169/161	1.420	1.302	1.392	1	Mascot
2133.1074	2133.0542	-0.0532	-25	2244	2262	FPPGLEAAEQSAEGLGSGK	54	99.99	(N-term)_iTRAQ[0], Lysine(K)_iTRAQ[19]	[1] F3 030912	351/343	0.941	0.878	0.913	1	Mascot
2197.1987	2197.0818	-0.1169	-53	1626	1642	GEPVGGQKEPVPAWEGK	37	99.459	(N-term)_iTRAQ[0], Lysine(K)_iTRAQ[8,17]	[6] F8 110912	217/209	0.986	0.875	0.994	1	Mascot
2308.2004	2308.0508	-0.1496	-65	1568	1585	SAEKADSVEQQDGAALEK	78	100	(N-term)_iTRAQ[0], Lysine(K)_iTRAQ[4,18]	[6] F8 110912	166/158	2.348	1.305	2.079	1	Mascot
2350.2693	2350.186	-0.0833	-35	1797	1814	NEPTTPSWLAEIPPWVPK	43	99.888	(N-term)_iTRAQ[0], Lysine(K)_iTRAQ[18]	[1] F3 030912	488/480	1.541	1.298	1.941	1	Mascot
2450.2007	2450.1072	-0.0935	-38	1301	1320	SPESLSSPAMEDLAVEWEGK	78	100	(N-term)_iTRAQ[0], Lysine(K)_iTRAQ[20]	[1] F3 030912	415/407	1.065	1.113	0.968	1	Mascot
2461.124	2460.9531	-0.1709	-69	1911	1929	DTEQTEPEQREPTPYPDER	33	98.799	(N-term)_iTRAQ[0]	[5] F4	92/84	0.780	0.721	1.125	1	Mascot
2535.2732	2535.3923	0.1191	47	2620	2639	STPSQVTSAEEKDGHSPMSK	28	96.014	(N-term)_iTRAQ[0], Lysine(K)_iTRAQ[12,20]	[4] F7 and F10+11	1050/1042	2.109	0.967	1.751	1	Mascot
2988.467	2988.3281	-0.1389	-46	1249	1277	ATVSPSTDETPAGTLPGGSFSHSALSVDR	47	99.949	(N-term)_iTRAQ[0]	[1] F3 030912	282/274	0.912	1.594	0.612	1	Mascot
3093.5764	3093.4714	-0.105	-34	949	977	LSGQYAAVFGAPGHTLPPGEPALGEVEER	48	99.963	(N-term)_iTRAQ[0]	[1] F3 030912	380/372	0.796	0.362	0.270	1	Mascot

10	brain-specific alpha actinin 1 isoform [Rattus norvegicus]	gi|38018016	113980.2	25	1419	0.858	0.864	0.898	0.302	0.337	0.284	30	30	30	100

Protein Group

Actn1 protein [Rattus norvegicus]	gi|49256643	110932.8
alpha-actinin-1 [Rattus norvegicus]	gi|13591902	111137

Peptide Information

Calc. Mass	Obsrv. Mass	± da	± ppm	Start Seq.	End Seq.	Sequence	Ion Score	C. I. %	Modification	Plate [#] Name	Gel Idx/Pos [4700 Sample Name]	iTRAQ Ratio 115/114*	iTRAQ Ratio 116/114*	iTRAQ Ratio 117/114*	Rank	Result Type
1172.6548	1172.5901	-0.0647	-55	293	300	TIPWLENR	35	99.171	(N-term)_iTRAQ[0]	[5] F4	227/219	0.832	0.451	0.835	1	Mascot
1307.6705	1307.6019	-0.0686	-52	148	156	EGLLLWCQR	29	97.236	(N-term)_iTRAQ[0], MMTS (C)[7]	[5] F4	340/332	0.867	1.008	0.539	1	Mascot
1359.7756	1359.7264	-0.0492	-36	282	291	LASDLLEWIR	58	99.996	(N-term)_iTRAQ[0]	[5] F4	402/394	0.825	0.958	0.928	1	Mascot
1391.7029	1391.6445	-0.0584	-42	175	185	DGLGFCALIHR	38	99.608	(N-term)_iTRAQ[0], MMTS (C)[6]	[6] F8 110912	424/416	1.055	1.179	1.018	1	Mascot
1391.7029	1391.7189	0.016	11	175	185	DGLGFCALIHR	37	99.471	(N-term)_iTRAQ[0], MMTS (C)[6]	[4] F7 and F10+11	422/414	1.234	1.032	0.727	1	Mascot
1482.7131	1482.6206	-0.0925	-62	742	752	GISQEQMNEFR	64	99.999	(N-term)_iTRAQ[0]	[5] F4	145/137	0.828	0.946	0.939	1	Mascot
1502.7849	1502.8262	0.0413	27	403	413	ASIHEAWTDGK	28	96.059	(N-term)_iTRAQ[0], Lysine(K)_iTRAQ[11]	[4] F7 and F10+11	1077/1069	0.845	1.008	0.809	1	Mascot
1516.8893	1516.8516	-0.0377	-25	65	76	LMLLLEVISGER	39	99.72	(N-term)_iTRAQ[0]	[1] F3 030912	575/567	0.938	0.971	1.122	1	Mascot
1525.7145	1525.7584	0.0439	29	36	46	TFTAWCNSHLR	59	99.997	(N-term)_iTRAQ[0], MMTS (C)[6]	[3] F6 and F9	1321/1313	0.531	0.626	0.709	1	Mascot
1530.8765	1530.8425	-0.034	-22	715	726	VGWEQLLTTIAR	63	99.999	(N-term)_iTRAQ[0]	[1] F3 030912	508/500	0.920	1.048	0.749	1	Mascot
1557.8258	1557.7272	-0.0986	-63	421	431	DYETATLSEIK	64	99.999	(N-term)_iTRAQ[0], Lysine(K)_iTRAQ[11]	[5] F4	171/163	0.899	0.725	1.230	1	Mascot
1565.8083	1565.7439	-0.0644	-41	377	387	GYEEWLLNEIR	50	99.973	(N-term)_iTRAQ[0]	[5] F4	348/340	1.039	0.929	0.835	1	Mascot
1573.8669	1573.8112	-0.0557	-35	727	738	TINEVENQILTR	45	99.925	(N-term)_iTRAQ[0]	[1] F3 030912	305/297	0.714	0.414	0.613	1	Mascot
1692.8658	1692.7502	-0.1156	-68	301	312	VPENTMQAMQQK	70	100	(N-term)_iTRAQ[0], Lysine(K)_iTRAQ[12]	[5] F4	128/120	0.749	0.949	0.763	1	Mascot
1692.8658	1692.7804	-0.0854	-50	301	312	VPENTMQAMQQK	43	99.882	(N-term)_iTRAQ[0], Lysine(K)_iTRAQ[12]	[7] F5 120912	165/157	2.122	1.607	1.832	1	Mascot
1694.7983	1694.8353	0.037	22	873	885	ELPPDQAEYCIAR	54	99.991	(N-term)_iTRAQ[0], MMTS (C)[10]	[8] F13-15 and F1+2	535/527	0.761	0.697	0.658	1	Mascot
1694.7983	1694.8413	0.043	25	873	885	ELPPDQAEYCIAR	54	99.991	(N-term)_iTRAQ[0], MMTS (C)[10]	[8] F13-15 and F1+2	486/478	0.676	0.789	0.813	1	Mascot
1787.9802	1788.0089	0.0287	16	163	174	NVNIQNFHISWK	52	99.985	(N-term)_iTRAQ[0],	[3] F6 and F9	1301/1293	0.501	0.402	0.590	1	Mascot

Calc. Mass	Obsrv. Mass	± da	± ppm	Start Seq.	End Seq.	Sequence	Ion Score	C. I. %	Modification	Plate [#] Name	Gel Idx/Pos [4700 Sample Name]	iTRAQ Ratio 115/114*	iTRAQ Ratio 116/114*	iTRAQ Ratio 117/114*	Rank	Result Type
									Lysine(K)_iTRAQ[12]							
1825.9794	1825.9082	-0.0712	-39	134	147	FAIQDISVEETSAK	85	100	(N-term)_iTRAQ[0], Lysine(K)_iTRAQ[14]	[1] F3 030912	292/284	0.703	1.212	0.911	1	Mascot
1938.0178	1938.0767	0.0589	30	47	60	KAGTQIENIEEDFR	42	99.86	(N-term)_iTRAQ[0], Lysine(K)_iTRAQ[1]	[3] F6 and F9	1257/1249	1.201	1.201	1.258	1	Mascot
1938.9645	1938.8958	-0.0687	-35	479	492	ICDQWDNLGALTQK	48	99.961	(N-term)_iTRAQ[0], Lysine(K)_iTRAQ[14], MMTS (C)[2]	[5] F4	317/309	1.071	1.054	1.421	1	Mascot
1958.0818	1958.1106	0.0288	15	419	431	QKDYETATLSEIK	52	99.985	(N-term)_iTRAQ[0], Lysine(K)_iTRAQ[2,13]	[3] F6 and F9	1195/1187	0.438	0.535	0.733	1	Mascot
1958.0818	1958.1659	0.0841	43	419	431	QKDYETATLSEIK	50	99.973	(N-term)_iTRAQ[0], Lysine(K)_iTRAQ[2,13]	[4] F7 and F10+11	1128/1120	0.890	0.707	1.203	1	Mascot
2000.1201	2000.076	-0.0441	-22	503	516	LLETIDQLYLEYAK	46	99.943	(N-term)_iTRAQ[0], Lysine(K)_iTRAQ[14]	[5] F4	422/414	1.060	0.997	1.037	1	Mascot
2000.1201	2000.0837	-0.0364	-18	503	516	LLETIDQLYLEYAK	62	99.999	(N-term)_iTRAQ[0], Lysine(K)_iTRAQ[14]	[1] F3 030912	554/546	0.813	0.730	0.796	1	Mascot
2017.9634	2017.8835	-0.0799	-40	841	856	ETADTDTADQVMASFK	93	100	(N-term)_iTRAQ[0], Lysine(K)_iTRAQ[16]	[1] F3 030912	297/289	0.839	0.940	0.915	1	Mascot
2207.1553	2207.1702	0.0149	7	198	214	KDDPLTNLNTAFDVAER	107	100	(N-term)_iTRAQ[0], Lysine(K)_iTRAQ[1]	[4] F7 and F10+11	365/357	0.730	0.889	0.830	1	Mascot
2223.1501	2223.147	-0.0031	-1	48	64	AGTQIENIEEDFRDGLK	66	99.999	(N-term)_iTRAQ[0], Lysine(K)_iTRAQ[17]	[4] F7 and F10+11	401/393	1.030	0.995	0.784	1	Mascot
2242.0532	2241.9604	-0.0928	-41	360	376	MVSDINNAWGCLEQAEK	32	98.563	(N-term)_iTRAQ[0], Lysine(K)_iTRAQ[17], MMTS (C)[11]	[5] F4	348/340	1.265	1.441	1.369	1	Mascot
2340.1638	2340.0747	-0.0891	-38	265	281	VLAVNQENEQLMEDYEK	81	100	(N-term)_iTRAQ[0], Lysine(K)_iTRAQ[17]	[1] F3 030912	305/297	0.606	0.807	1.067	1	Mascot

11	ATPase alpha1,Na/K	gi|358959	122092.3	23	1403	1.039	0.972	0.948	0.362	0.285	0.293	31	31	31	100

Protein Group

sodium/potassium-transporting ATPase subunit alpha-1 precursor [Rattus norvegicus] gi|6978543 122253.4

Peptide Information

Calc. Mass	Obsrv. Mass	± da	± ppm	Start Seq.	End Seq.	Sequence	Ion Score	C. I. %	Modification	Plate [#] Name	Gel Idx/Pos [4700 Sample Name]	iTRAQ Ratio 115/114*	iTRAQ Ratio 116/114*	iTRAQ Ratio 117/114*	Rank	Result Type
1115.667	1115.6121	-0.0549	-49	597	605	AAVPDAVGK	61	99.998	(N-term)_iTRAQ[0], Lysine(K)_iTRAQ[9]	[7] F5 120912	113/105	0.699	0.645	0.635	1	Mascot
1126.634	1126.651	0.017	15	46	53	LSLDELHR	28	96.297	(N-term)_iTRAQ[0]	[3] F6 and F9	1195/1187	0.868	0.944	0.634	1	Mascot
1150.7081	1150.6692	-0.0389	-34	767	773	LIFDNLK	36	99.428	(N-term)_iTRAQ[0], Lysine(K)_iTRAQ[7]	[7] F5 120912	289/281	1.128	0.944	0.789	1	Mascot
1220.6597	1220.5994	-0.0603	-49	699	707	LIIVEGCQR	28	96.28	(N-term)_iTRAQ[0], MMTS (C)[7]	[5] F4	234/226	0.711	1.038	0.564	1	Mascot
1220.6597	1220.6172	-0.0425	-35	699	707	LIIVEGCQR	48	99.965	(N-term)_iTRAQ[0], MMTS (C)[7]	[1] F3 030912	310/302	0.968	0.922	0.948	1	Mascot
1279.6919	1279.6124	-0.0795	-62	684	692	YHTEIVFAR	52	99.985	(N-term)_iTRAQ[0]	[6] F8 110912	243/235	0.555	0.840	0.849	1	Mascot
1388.752	1388.7264	-0.0256	-18	527	535	EQPLDEELK	40	99.724	(N-term)_iTRAQ[0], Lysine(K)_iTRAQ[9]	[7] F5 120912	159/151	0.874	0.883	0.895	1	Mascot
1407.8192	1407.749	-0.0702	-50	648	658	LNIPVNQVNPR	48	99.964	(N-term)_iTRAQ[0]	[1] F3 030912	232/224	0.999	1.012	0.880	1	Mascot
1412.8168	1412.7659	-0.0509	-36	163	173	NMVPQQALVIR	30	97.372	(N-term)_iTRAQ[0]	[1] F3 030912	265/257	1.559	1.149	1.196	1	Mascot
1412.8168	1412.8419	0.0251	18	163	173	NMVPQQALVIR	55	99.992	(N-term)_iTRAQ[0]	[8] F13-15 and F1+2	499/491	1.096	1.031	1.016	1	Mascot
1412.8168	1412.8593	0.0425	30	163	173	NMVPQQALVIR	39	99.68	(N-term)_iTRAQ[0]	[8] F13-15 and F1+2	555/547	1.131	0.931	0.923	1	Mascot
1422.9055	1422.95	0.0445	31	767	774	LIFDNLKK	43	99.87	(N-term)_iTRAQ[0], Lysine(K)_iTRAQ[7,8]	[4] F7 and F10+11	1176/1168	1.407	1.125	1.304	1	Mascot
1549.8835	1549.8198	-0.0637	-41	477	487	IVEIPFNSTNK	72	100	(N-term)_iTRAQ[0], Lysine(K)_iTRAQ[11]	[5] F4	207/199	0.891	0.967	0.887	1	Mascot
1580.7598	1580.7791	0.0193	12	672	683	DMTSEELDDILR	28	96.297	(N-term)_iTRAQ[0]	[8] F13-15 and F1+2	530/522	0.862	1.586	0.681	1	Mascot
1663.8048	1663.7162	-0.0886	-53	228	240	SPDFTNENPLETR	59	99.997	(N-term)_iTRAQ[0]	[1] F3 030912	235/227	1.159	1.049	1.195	1	Mascot
1669.9557	1669.916	-0.0397	-24	613	625	VIMVTGDHPITAK	78	100	(N-term)_iTRAQ[0], Lysine(K)_iTRAQ[13]	[3] F6 and F9	232/224	0.670	0.709	0.702	1	Mascot
1669.9557	1669.9308	-0.0249	-15	613	625	VIMVTGDHPITAK	86	100	(N-term)_iTRAQ[0], Lysine(K)_iTRAQ[13]	[4] F7 and F10+11	234/226	1.185	1.115	1.045	1	Mascot
1763.8531	1763.7506	-0.1025	-58	213	227	VDNSSLTGESEPQTR	77	100	(N-term)_iTRAQ[0]	[1] F3 030912	136/128	1.082	1.168	1.150	1	Mascot
1862.9984	1862.9014	-0.097	-52	10	22	YEPAAVSEHGDKK	63	99.999	(N-term)_iTRAQ[0], Lysine(K)_iTRAQ[12,13]	[2] F12 040912	133/125	0.913	0.857	1.104	1	Mascot
1873.0792	1872.9906	-0.0886	-47	431	444	AVFQANQENLPILK	81	100	(N-term)_iTRAQ[0], Lysine(K)_iTRAQ[14]	[5] F4	247/239	1.107	1.401	1.756	1	Mascot
1974.0264	1974.0244	-0.002	-1	630	647	GVGIISEGNETVEDIAAR	48	99.96	(N-term)_iTRAQ[0]	[8] F13-15 and F1+2	454/446	1.135	0.930	0.924	1	Mascot
1974.0264	1974.0256	-0.0008	0	630	647	GVGIISEGNETVEDIAAR	52	99.984	(N-term)_iTRAQ[0]	[8] F13-15 and F1+2	446/438	1.254	0.827	1.022	1	Mascot
1974.0264	1974.0314	0.005	3	630	647	GVGIISEGNETVEDIAAR	79	100	(N-term)_iTRAQ[0]	[8] F13-15 and F1+2	450/442	1.181	0.894	1.002	1	Mascot
1974.0264	1974.0327	0.0063	3	630	647	GVGIISEGNETVEDIAAR	31	98.048	(N-term)_iTRAQ[0]	[8] F13-15 and F1+2	503/495	1.407	1.001	1.440	1	Mascot
1974.0264	1974.0538	0.0274	14	630	647	GVGIISEGNETVEDIAAR	40	99.731	(N-term)_iTRAQ[0]	[8] F13-15 and F1+2	555/547	0.853	0.908	0.967	1	Mascot

Calc. Mass	Obsrv. Mass	± da	± ppm	Start Seq.	End Seq.	Sequence	Ion Score	C. I. %	Modification	Plate [#] Name	Gel Idx/Pos [4700 Sample Name]	iTRAQ Ratio 115/114*	iTRAQ Ratio 116/114*	iTRAQ Ratio 117/114*	Rank	Result Type
2100.1545	2100.0515	-0.103	-49	708	726	QGAIVAVTGDGVNDSPA LK	112	100	(N-term)_iTRAQ[0], Lysine(K)_iTRAQ[19]	[1] F3 030912	255/247	1.065	0.886	0.737	1	Mascot
2105.1409	2105.0635	-0.0774	-37	178	194	MSINAEDVVVGDLVEVK	95	100	(N-term)_iTRAQ[0], Lysine(K)_iTRAQ[17]	[1] F3 030912	427/419	1.132	0.923	1.123	1	Mascot
2108.1274	2108.0452	-0.0822	-39	75	91	DGPNALTPPPTTPEWVK	57	99.995	(N-term)_iTRAQ[0], Lysine(K)_iTRAQ[17]	[1] F3 030912	289/281	1.020	0.625	0.647	1	Mascot
2202.0859	2202.0366	-0.0493	-22	360	377	NLEAVETLGSTSTICSDK	99	100	(N-term)_iTRAQ[0], Lysine(K)_iTRAQ[18], MMTS (C)[15]	[1] F3 030912	363/355	1.008	0.712	0.912	1	Mascot
2372.3521	2372.2949	-0.0572	-24	708	727	QGAIVAVTGDGVNDSPA LKK	30	97.508	(N-term)_iTRAQ[0], Lysine(K)_iTRAQ[19, 20]	[4] F7 and F10+11	246/238	0.887	0.802	0.929	1	Mascot
2608.3049	2608.2537	-0.0512	-20	744	766	QAADMILLDDNFASIVTG VEEGR	55	99.992	(N-term)_iTRAQ[0]	[1] F3 030912	548/540	3.111	2.449	1.643	1	Mascot

12	dihydropyrimidinase-related protein 2 [Mus musculus]	gi|40254595	67315.9	21	1401	1.195	1.093	1.115	0.676	0.421	0.530	27	27	27	100

Peptide Information

Calc. Mass	Obsrv. Mass	± da	± ppm	Start Seq.	End Seq.	Sequence	Ion Score	C. I. %	Modification	Plate [#] Name	Gel Idx/Pos [4700 Sample Name]	iTRAQ Ratio 115/114*	iTRAQ Ratio 116/114*	iTRAQ Ratio 117/114*	Rank	Result Type
1159.6555	1159.5724	-0.0831	-72	259	268	SAAEVIAQAR	47	99.948	(N-term)_iTRAQ[0]	[5] F4	121/113	2.037	1.493	1.145	1	Mascot
1159.6555	1159.5841	-0.0714	-62	259	268	SAAEVIAQAR	46	99.933	(N-term)_iTRAQ[0]	[1] F3 030912	191/183	0.898	0.325	1.010	1	Mascot
1166.6489	1166.6199	-0.029	-25	362	368	MSVIWDK	38	99.583	(N-term)_iTRAQ[0], Lysine(K)_iTRAQ[7]	[3] F6 and F9	289/281	1.472	0.900	1.205	1	Mascot
1372.8409	1372.7793	-0.0616	-45	441	451	GSPLVVISQGK	56	99.994	(N-term)_iTRAQ[0], Lysine(K)_iTRAQ[11]	[5] F4	162/154	0.957	0.982	0.908	1	Mascot
1438.796	1438.7744	-0.0216	-15	64	75	MVIPGGIDVHTR	63	99.999	(N-term)_iTRAQ[0]	[4] F7 and F10+11	265/257	1.009	1.040	1.087	1	Mascot
1534.8396	1534.781	-0.0586	-38	147	157	GIQEEMEALVK	83	100	(N-term)_iTRAQ[0], Lysine(K)_iTRAQ[11]	[5] F4	273/265	1.319	1.072	1.087	1	Mascot
1572.9161	1572.9777	0.0616	39	472	480	KPFPDFVYK	44	99.908	(N-term)_iTRAQ[0], Lysine(K)_iTRAQ[1,9]	[4] F7 and F10+11	1190/1182	1.116	1.002	1.047	1	Mascot
1611.9679	1611.8875	-0.0804	-50	44	56	QIGENLIVPGGVK	71	100	(N-term)_iTRAQ[0], Lysine(K)_iTRAQ[13]	[5] F4	202/194	0.895	1.286	0.968	1	Mascot
1826.9731	1826.9469	-0.0262	-14	452	467	IVLEDGTLHVTEGSGR	84	100	(N-term)_iTRAQ[0]	[3] F6 and F9	292/284	1.495	1.172	1.277	1	Mascot
1897.963	1897.9196	-0.0434	-23	497	511	GLYDGPVCEVSVTPK	73	100	(N-term)_iTRAQ[0], Lysine(K)_iTRAQ[15], MMTS (C)[8]	[1] F3 030912	332/324	1.335	1.032	1.061	1	Mascot
1915.9985	1915.9392	-0.0593	-31	158	171	DHGVNSFLVYMAFK	118	100	(N-term)_iTRAQ[0], Lysine(K)_iTRAQ[14]	[6] F8 110912	454/446	0.981	0.958	1.218	1	Mascot
1936.9373	1936.964	0.0267	14	346	361	DNFTLIPEGTNGTEER	30	97.774	(N-term)_iTRAQ[0]	[8] F13-15 and F1+2	492/484	1.097	0.961	0.842	1	Mascot
2014.0161	2013.9055	-0.1106	-55	375	390	MDENQFVAVTSTNAAK	82	100	(N-term)_iTRAQ[0], Lysine(K)_iTRAQ[16]	[5] F4	165/157	1.268	1.136	1.117	1	Mascot
2055.1248	2055.0757	-0.0491	-24	174	189	FQLTDSQIYEVLSVIR	63	99.999	(N-term)_iTRAQ[0]	[1] F3 030912	544/536	1.243	1.167	0.861	1	Mascot
2055.1248	2055.1033	-0.0215	-10	174	189	FQLTDSQIYEVLSVIR	36	99.345	(N-term)_iTRAQ[0]	[8] F13-15 and F1+2	248/240	1.271	0.965	1.104	1	Mascot
2100.106	2100.0376	-0.0684	-33	239	254	SITIANQTNCPLYVTK	83	100	(N-term)_iTRAQ[0], Lysine(K)_iTRAQ[16], MMTS (C)[10]	[1] F3 030912	337/329	1.342	1.113	1.129	1	Mascot
2203.9675	2203.9097	-0.0578	-26	424	440	THNSALEYNIFEGMECR	38	99.592	(N-term)_iTRAQ[0], MMTS (C)[16]	[2] F12 040912	407/399	0.575	1.311	0.829	1	Mascot
2203.9675	2203.9817	0.0142	6	424	440	THNSALEYNIFEGMECR	55	99.992	(N-term)_iTRAQ[0], MMTS (C)[16]	[4] F7 and F10+11	422/414	1.225	1.853	1.457	1	Mascot
2203.9675	2204.0886	0.1211	55	424	440	THNSALEYNIFEGMECR	49	99.971	(N-term)_iTRAQ[0], MMTS (C)[16]	[4] F7 and F10+11	1273/1265	0.874	1.193	1.164	1	Mascot
2204.1697	2204.104	-0.0657	-30	401	418	ISVGSDADLVIWDPDSVK	79	100	(N-term)_iTRAQ[0], Lysine(K)_iTRAQ[18]	[1] F3 030912	404/396	1.254	1.124	0.796	1	Mascot
2313.1707	2313.093	-0.0777	-34	533	552	NLHQSGFSLSGAQIDDNI PR	53	99.988	(N-term)_iTRAQ[0]	[7] F5 120912	275/267	0.446	0.879	1.024	1	Mascot
2313.1707	2313.2561	0.0854	37	533	552	NLHQSGFSLSGAQIDDNI PR	95	100	(N-term)_iTRAQ[0]	[4] F7 and F10+11	1181/1173	0.895	1.068	0.725	1	Mascot
2439.1206	2439.0537	-0.0669	-27	76	94	FQMPDQGMTSADDFFQ GTK	101	100	(N-term)_iTRAQ[0], Lysine(K)_iTRAQ[19]	[1] F3 030912	376/368	1.054	1.008	1.147	1	Mascot
2455.1155	2455.0278	-0.0877	-36	76	94	FQMPDQGMTSADDFFQ GTK	71	100	(N-term)_iTRAQ[0], Lysine(K)_iTRAQ[19], Oxidation (M)[8]	[1] F3 030912	331/323	1.783	1.535	1.046	1	Mascot
2521.2766	2521.1526	-0.124	-49	190	211	DIGAIAQVHAENGDIIAEE QQR	72	100	(N-term)_iTRAQ[0]	[1] F3 030912	281/273	0.959	0.754	1.110	1	Mascot
2985.5693	2985.655	0.0857	29	270	293	KGTVVYGEPITASLGTDG SHYWSK	31	98.02	(N-term)_iTRAQ[0], Lysine(K)_iTRAQ[1,2 4]	[4] F7 and F10+11	1214/1206	1.832	1.561	0.985	1	Mascot
3281.7112	3281.614	-0.0972	-30	95	124	AALAGGTTMIIDHVVPEP GTSLLAAFDQWR	36	99.432	(N-term)_iTRAQ[0]	[1] F3 030912	553/545	6.035	2.048	6.934	1	Mascot

13	RecName: Full=Microtubule-associated protein 1B; Short=MAP-1B; AltName: Full=Neuraxin; Contains: Re	gi|19856246	303587.6	22	1343	1.242	1.028	1.015	0.431	0.298	0.266	24	24	24	100

Peptide Information

Calc. Mass	Obsrv. Mass	± da	± ppm	Start Seq.	End Seq.	Sequence	Ion Score	C. I. %	Modification	Plate [#] Name	Gel Idx/Pos [4700 Sample Name]	iTRAQ Ratio 115/114*	iTRAQ Ratio 116/114*	iTRAQ Ratio 117/114*	Rank	Result Type

Calc. Mass	Obsrv. Mass	± da	± ppm	Start Seq.	End Seq.	Sequence	Ion Score	C. I. %	Modification	Plate [#] Name	Gel Idx/Pos [4700 Sample Name]	iTRAQ Ratio 115/114*	iTRAQ Ratio 116/114*	iTRAQ Ratio 117/114*	Rank	Result Type
1199.7231	1199.6721	-0.051	-43	55	64	AIGNIELGIR	30	97.384	(N-term)_iTRAQ[0]	[5] F4	213/205	0.846	0.633	0.902	1	Mascot
1276.7147	1276.6882	-0.0265	-21	90	98	FSPEVPGQK	56	99.994	(N-term)_iTRAQ[0], Lysine(K)_iTRAQ[9]	[7] F5 120912	155/147	0.960	0.903	0.893	1	Mascot
1285.7037	1285.7019	-0.0018	-1	2362	2369	NVDVEFFK	46	99.935	(N-term)_iTRAQ[0], Lysine(K)_iTRAQ[8]	[7] F5 120912	259/251	1.199	1.003	1.005	1	Mascot
1529.8685	1529.9176	0.0491	32	204	213	HNLQDFINIK	47	99.945	(N-term)_iTRAQ[0], Lysine(K)_iTRAQ[10]	[4] F7 and F10+11	1196/1188	1.054	0.880	0.972	1	Mascot
1535.7219	1535.7031	-0.0188	-12	2188	2199	HMDPPPAPMQDR	35	99.232	(N-term)_iTRAQ[0]	[4] F7 and F10+11	155/147	1.023	0.823	0.878	1	Mascot
1537.7533	1537.816	0.0627	41	2422	2430	EWYQETHEK	35	99.253	(N-term)_iTRAQ[0], Lysine(K)_iTRAQ[9]	[4] F7 and F10+11	1068/1060	2.318	1.681	1.630	1	Mascot
1561.7996	1561.7019	-0.0977	-63	1957	1967	TPEVSGYTYEK	78	100	(N-term)_iTRAQ[0], Lysine(K)_iTRAQ[11]	[5] F4	121/113	1.141	0.820	0.724	1	Mascot
1610.8271	1610.8761	0.049	30	1217	1227	DAYRPEETDVK	54	99.99	(N-term)_iTRAQ[0], Lysine(K)_iTRAQ[11]	[3] F6 and F9	1101/1093	1.632	1.366	1.252	1	Mascot
1636.9155	1636.8379	-0.0776	-47	1267	1278	SVNFSLTPNEIK	57	99.995	(N-term)_iTRAQ[0], Lysine(K)_iTRAQ[12]	[5] F4	209/201	1.524	1.048	1.011	1	Mascot
1710.9524	1710.8707	-0.0817	-48	1247	1260	SPSLSPSPPSPIEK	49	99.97	(N-term)_iTRAQ[0], Lysine(K)_iTRAQ[14]	[1] F3 030912	214/206	1.655	1.227	1.509	1	Mascot
1749.8052	1749.7159	-0.0893	-51	2042	2055	TPQASTYSYETSDR	32	98.276	(N-term)_iTRAQ[0]	[1] F3 030912	157/149	1.173	0.897	0.885	1	Mascot
1789.0078	1789.0199	0.0121	7	605	616	EVPSKEEQSPVK	65	99.999	(N-term)_iTRAQ[0], Lysine(K)_iTRAQ[5,12]	[3] F6 and F9	1092/1084	0.678	0.711	0.799	1	Mascot
1789.0078	1789.0848	0.077	43	605	616	EVPSKEEQSPVK	58	99.996	(N-term)_iTRAQ[0], Lysine(K)_iTRAQ[5,12]	[4] F7 and F10+11	1026/1018	1.250	0.979	1.041	1	Mascot
1862.8379	1862.7587	-0.0792	-43	1923	1936	TPEDGGYSCEITEK	51	99.98	(N-term)_iTRAQ[0], Lysine(K)_iTRAQ[14], MMTS (C)[9]	[5] F4	164/156	1.350	0.977	1.314	1	Mascot
1876.9062	1876.7725	-0.1337	-71	1940	1953	TPEEGGYSYEISEK	87	100	(N-term)_iTRAQ[0], Lysine(K)_iTRAQ[14]	[5] F4	134/126	1.008	1.087	1.004	1	Mascot
1912.8535	1912.7468	-0.1067	-56	1906	1919	SPCDSGYSYETIEK	59	99.997	(N-term)_iTRAQ[0], Lysine(K)_iTRAQ[14], MMTS (C)[3]	[5] F4	186/178	1.913	1.181	1.109	1	Mascot
1915.1837	1915.0929	-0.0908	-47	2256	2269	SKPSAASPKPGALK	39	99.668	(N-term)_iTRAQ[0], Lysine(K)_iTRAQ[2,9,14]	[2] F12 040912	127/119	1.497	1.070	1.189	1	Mascot
1931.9749	1931.8773	-0.0976	-51	1886	1899	TIQAHDVGGYYYEK	93	100	(N-term)_iTRAQ[0], Lysine(K)_iTRAQ[14]	[6] F8 110912	217/209	0.964	0.917	1.173	1	Mascot
1931.9749	1931.9758	0.0009	0	1886	1899	TIQAHDVGGYYYEK	44	99.906	(N-term)_iTRAQ[0], Lysine(K)_iTRAQ[14]	[4] F7 and F10+11	218/210	0.713	0.719	0.551	1	Mascot
1958.8413	1958.7219	-0.1194	-61	2025	2038	SPDTSAYCYETMEK	48	99.959	(N-term)_iTRAQ[0], Lysine(K)_iTRAQ[14], MMTS (C)[8]	[5] F4	188/180	1.035	0.905	0.763	1	Mascot
2018.0072	2018.0895	0.0823	41	1825	1840	SSMLFDTMQHHLALSR	59	99.997	(N-term)_iTRAQ[0]	[4] F7 and F10+11	1239/1231	1.488	1.155	1.007	1	Mascot
2071.9692	2072.0024	0.0332	16	2373	2390	SSYYVVSGNDPAAEEPSR	28	95.649	(N-term)_iTRAQ[0]	[8] F13-15 and F1+2	569/561	1.738	1.590	1.042	1	Mascot
2258.0962	2257.988	-0.1082	-48	2005	2021	ITSFPESESYSYETTTK	104	100	(N-term)_iTRAQ[0], Lysine(K)_iTRAQ[17]	[1] F3 030912	262/254	1.507	1.267	1.267	1	Mascot
2697.1743	2696.9875	-0.1868	-69	938	960	AETEEAEEPEEDGEDNVSGSASK	199	100	(N-term)_iTRAQ[0], Lysine(K)_iTRAQ[23]	[1] F3 030912	137/129	1.472	1.670	1.084	1	Mascot

14	tubulin beta-5 chain [Mus musculus]	gi|7106439	52312.5	20	1323	1.148	1.014	1.117	0.760	0.512	0.587	54	54	54	100	

Peptide Information

Calc. Mass	Obsrv. Mass	± da	± ppm	Start Seq.	End Seq.	Sequence	Ion Score	C. I. %	Modification	Plate [#] Name	Gel Idx/Pos [4700 Sample Name]	iTRAQ Ratio 115/114*	iTRAQ Ratio 116/114*	iTRAQ Ratio 117/114*	Rank	Result Type
1161.5862	1161.538	-0.0482	-41	351	359	TAVCDIPPR	34	99.07	(N-term)_iTRAQ[0], MMTS (C)[4]	[1] F3 030912	224/216	0.853	0.869	0.811	1	Mascot
1183.6959	1183.6681	-0.0278	-23	310	318	YLTVAAVFR	51	99.981	(N-term)_iTRAQ[0]	[1] F3 030912	375/367	1.511	1.264	1.071	1	Mascot
1198.4943	1198.4203	-0.074	-62	298	306	NMMAACDPR	42	99.828	(N-term)_iTRAQ[0], MMTS (C)[6]	[5] F4	175/167	1.069	1.113	1.167	1	Mascot
1221.6348	1221.6912	0.0564	46	155	162	IREEYPDR	27	95.568	(N-term)_iTRAQ[0]	[4] F7 and F10+11	1026/1018	0.939	1.088	1.289	1	Mascot
1274.6978	1274.6228	-0.075	-59	242	251	FPGQLNADLR	51	99.981	(N-term)_iTRAQ[0]	[5] F4	190/182	1.149	0.984	1.021	1	Mascot
1274.6978	1274.6488	-0.049	-38	242	251	FPGQLNADLR	43	99.868	(N-term)_iTRAQ[0]	[1] F3 030912	265/257	0.873	0.845	0.884	1	Mascot
1287.7367	1287.6992	-0.0375	-29	253	262	LAVNMVPFPR	35	99.138	(N-term)_iTRAQ[0]	[1] F3 030912	349/341	0.961	0.841	0.953	1	Mascot
1287.7367	1287.7393	0.0026	2	253	262	LAVNMVPFPR	32	98.442	(N-term)_iTRAQ[0]	[1] F3 030912	716/708	0.788	0.478	0.836	1	Mascot
1373.7008	1373.6394	-0.0614	-45	381	390	ISEQFTAMFR	62	99.999	(N-term)_iTRAQ[0]	[5] F4	284/276	0.941	0.970	1.014	1	Mascot
1373.7008	1373.7096	0.0088	6	381	390	ISEQFTAMFR	27	95.141	(N-term)_iTRAQ[0]	[5] F4	591/583	0.831	1.259	1.157	1	Mascot
1546.8951	1546.9545	0.0594	38	242	252	FPGQLNADLRK	46	99.945	(N-term)_iTRAQ[0], Lysine(K)_iTRAQ[11]	[3] F6 and F9	1213/1205	1.144	0.783	1.344	1	Mascot
1559.9342	1559.8873	-0.0469	-30	252	262	KLAVNMVPFPR	33	98.897	(N-term)_iTRAQ[0], Lysine(K)_iTRAQ[1]	[6] F8 110912	367/359	0.926	0.879	0.841	1	Mascot
1559.9342	1560.0081	0.0739	47	252	262	KLAVNMVPFPR	45	99.924	(N-term)_iTRAQ[0], Lysine(K)_iTRAQ[1]	[3] F6 and F9	1277/1269	1.868	1.166	1.510	1	Mascot
1589.842	1589.8147	-0.0273	-17	47	58	ISVYYNEATGGK	75	100	(N-term)_iTRAQ[0], Lysine(K)_iTRAQ[12]	[7] F5 120912	206/198	1.270	1.197	1.253	1	Mascot

1607.9077	1607.8265	-0.0812	-51	163	174	IMNTFSVVPSPK	64	99.999	(N-term)_iTRAQ[0], Lysine(K)_iTRAQ[12]	[5] F4	234/226	0.954	0.876	1.059	1	Mascot
1734.8942	1734.7839	-0.1103	-64	325	336	EVDEQMLNVQNK	63	99.999	(N-term)_iTRAQ[0], Lysine(K)_iTRAQ[12]	[5] F4	140/132	0.980	0.919	0.865	1	Mascot
1734.8942	1734.8132	-0.081	-47	325	336	EVDEQMLNVQNK	82	100	(N-term)_iTRAQ[0], Lysine(K)_iTRAQ[12]	[7] F5 120912	177/169	1.326	1.321	1.178	1	Mascot
1734.8942	1734.9392	0.045	26	325	336	EVDEQMLNVQNK	31	98.248	(N-term)_iTRAQ[0], Lysine(K)_iTRAQ[12]	[5] F4	591/583	0.461	0.361	0.692	1	Mascot
1750.8892	1750.7924	-0.0968	-55	325	336	EVDEQMLNVQNK	77	100	(N-term)_iTRAQ[0], Lysine(K)_iTRAQ[12], Oxidation (M)[6]	[5] F4	105/97	1.124	1.154	1.249	1	Mascot
1759.9384	1759.9335	-0.0049	-3	63	77	AILVDLEPGTMDSVR	75	100	(N-term)_iTRAQ[0]	[8] F13-15 and F1+2	408/400	1.012	1.229	0.951	1	Mascot
1759.9384	1759.9364	-0.002	-1	63	77	AILVDLEPGTMDSVR	64	99.999	(N-term)_iTRAQ[0]	[8] F13-15 and F1+2	420/412	1.115	1.414	1.398	1	Mascot
1759.9384	1759.9373	-0.0011	-1	63	77	AILVDLEPGTMDSVR	52	99.984	(N-term)_iTRAQ[0]	[8] F13-15 and F1+2	461/453	1.241	1.771	1.343	1	Mascot
1759.9384	1759.9391	0.0007	0	63	77	AILVDLEPGTMDSVR	73	100	(N-term)_iTRAQ[0]	[8] F13-15 and F1+2	466/458	1.176	0.994	0.979	1	Mascot
1759.9384	1759.9392	0.0008	0	63	77	AILVDLEPGTMDSVR	30	97.57	(N-term)_iTRAQ[0]	[8] F13-15 and F1+2	516/508	1.051	1.080	0.935	1	Mascot
1759.9384	1759.946	0.0076	4	63	77	AILVDLEPGTMDSVR	93	100	(N-term)_iTRAQ[0]	[8] F13-15 and F1+2	412/404	1.164	1.244	1.086	1	Mascot
1759.9384	1759.9512	0.0128	7	63	77	AILVDLEPGTMDSVR	33	98.686	(N-term)_iTRAQ[0]	[8] F13-15 and F1+2	512/504	1.092	1.100	1.180	1	Mascot
1759.9384	1759.9528	0.0144	8	63	77	AILVDLEPGTMDSVR	68	100	(N-term)_iTRAQ[0]	[8] F13-15 and F1+2	404/396	0.658	0.704	0.767	1	Mascot
1759.9384	1759.954	0.0156	9	63	77	AILVDLEPGTMDSVR	66	99.999	(N-term)_iTRAQ[0]	[8] F13-15 and F1+2	417/409	0.952	1.015	0.884	1	Mascot
1759.9384	1759.9597	0.0213	12	63	77	AILVDLEPGTMDSVR	28	95.748	(N-term)_iTRAQ[0]	[8] F13-15 and F1+2	523/515	0.828	0.208	0.738	1	Mascot
1764.938	1764.9121	-0.0259	-15	263	276	LHFFMPGFAPLTSR	61	99.998	(N-term)_iTRAQ[0]	[3] F6 and F9	456/448	0.729	1.073	1.164	1	Mascot
1764.938	1764.9736	0.0356	20	263	276	LHFFMPGFAPLTSR	47	99.95	(N-term)_iTRAQ[0]	[4] F7 and F10+11	470/462	0.960	1.090	1.164	1	Mascot
1764.938	1765.0078	0.0698	40	263	276	LHFFMPGFAPLTSR	38	99.601	(N-term)_iTRAQ[0]	[4] F7 and F10+11	531/523	0.891	1.360	1.548	1	Mascot
1948.1001	1948.048	-0.0521	-27	283	297	ALTVPELTQQVFDAK	72	100	(N-term)_iTRAQ[0], Lysine(K)_iTRAQ[15]	[1] F3 030912	408/400	1.125	0.931	1.111	1	Mascot
1948.1001	1948.052	-0.0481	-25	283	297	ALTVPELTQQVFDAK	37	99.533	(N-term)_iTRAQ[0], Lysine(K)_iTRAQ[15]	[2] F12 040912	441/433	1.245	0.737	0.882	1	Mascot
1985.0378	1984.9299	-0.1079	-54	337	350	NSSYFVEWIPNNVK	39	99.708	(N-term)_iTRAQ[0], Lysine(K)_iTRAQ[14]	[5] F4	298/290	1.071	1.010	1.098	1	Mascot
1985.0378	1984.957	-0.0808	-41	337	350	NSSYFVEWIPNNVK	43	99.868	(N-term)_iTRAQ[0], Lysine(K)_iTRAQ[14]	[6] F8 110912	369/361	0.630	0.821	0.824	1	Mascot
1985.0378	1984.9778	-0.06	-30	337	350	NSSYFVEWIPNNVK	32	98.529	(N-term)_iTRAQ[0], Lysine(K)_iTRAQ[14]	[6] F8 110912	363/355	1.422	0.707	0.524	1	Mascot
1985.0378	1984.988	-0.0498	-25	337	350	NSSYFVEWIPNNVK	30	97.598	(N-term)_iTRAQ[0], Lysine(K)_iTRAQ[14]	[5] F4	339/331	1.470	1.187	0.924	1	Mascot
2100.092	2099.9648	-0.1272	-61	3	19	EIVHIQAGQCGNQIGAK	66	99.999	(N-term)_iTRAQ[0], Lysine(K)_iTRAQ[17], MMTS (C)[10]	[6] F8 110912	261/253	0.727	0.775	0.614	1	Mascot
2100.092	2100.0605	-0.0315	-15	3	19	EIVHIQAGQCGNQIGAK	109	100	(N-term)_iTRAQ[0], Lysine(K)_iTRAQ[17], MMTS (C)[10]	[4] F7 and F10+11	265/257	1.064	0.910	1.094	1	Mascot
2100.092	2100.1162	0.0242	12	3	19	EIVHIQAGQCGNQIGAK	89	100	(N-term)_iTRAQ[0], Lysine(K)_iTRAQ[17], MMTS (C)[10]	[4] F7 and F10+11	379/371	1.194	0.967	0.685	1	Mascot
2100.092	2100.1257	0.0337	16	3	19	EIVHIQAGQCGNQIGAK	41	99.786	(N-term)_iTRAQ[0], Lysine(K)_iTRAQ[17], MMTS (C)[10]	[4] F7 and F10+11	631/623	1.893	1.942	1.642	1	Mascot
2100.092	2100.1853	0.0933	44	3	19	EIVHIQAGQCGNQIGAK	37	99.482	(N-term)_iTRAQ[0], Lysine(K)_iTRAQ[17], MMTS (C)[10]	[4] F7 and F10+11	558/550	1.799	1.038	2.313	1	Mascot
2103.0842	2103.0032	-0.081	-39	104	121	GHYTEGAELVDSVLDVV R	37	99.506	(N-term)_iTRAQ[0]	[6] F8 110912	488/480	1.671	0.987	0.997	1	Mascot
2103.0842	2103.0552	-0.029	-14	104	121	GHYTEGAELVDSVLDVV R	37	99.468	(N-term)_iTRAQ[0]	[3] F6 and F9	722/714	3.032	1.634	1.575	1	Mascot
2103.0842	2103.0581	-0.0261	-12	104	121	GHYTEGAELVDSVLDVV R	53	99.987	(N-term)_iTRAQ[0]	[2] F12 040912	496/488	1.368	1.498	3.600	1	Mascot
2103.0842	2103.0779	-0.0063	-3	104	121	GHYTEGAELVDSVLDVV R	54	99.991	(N-term)_iTRAQ[0]	[7] F5 120912	465/457	0.957	0.667	1.064	1	Mascot
2103.0842	2103.0789	-0.0053	-3	104	121	GHYTEGAELVDSVLDVV R	48	99.96	(N-term)_iTRAQ[0]	[3] F6 and F9	481/473	1.228	1.113	1.179	1	Mascot
2103.0842	2103.1221	0.0379	18	104	121	GHYTEGAELVDSVLDVV R	111	100	(N-term)_iTRAQ[0]	[4] F7 and F10+11	489/481	1.151	0.950	0.918	1	Mascot
2103.0842	2103.1589	0.0747	36	104	121	GHYTEGAELVDSVLDVV R	119	100	(N-term)_iTRAQ[0]	[4] F7 and F10+11	1342/1334	0.615	0.767	0.660	1	Mascot
2103.0842	2103.1692	0.085	40	104	121	GHYTEGAELVDSVLDVV R	36	99.343	(N-term)_iTRAQ[0]	[4] F7 and F10+11	1318/1310	14.597	2.288	6.391	1	Mascot
2158.1829	2158.116	-0.0669	-31	363	379	MAVTFIGNSTAIQELFK	99	100	(N-term)_iTRAQ[0], Lysine(K)_iTRAQ[17]	[1] F3 030912	505/497	0.863	1.101	1.125	1	Mascot
2841.405	2841.3303	-0.0747	-26	217	241	LTTPTYGDLNHLVSATMS GVTTCLR	61	99.998	(N-term)_iTRAQ[0], MMTS (C)[23]	[1] F3 030912	521/513	4.790	3.531	3.426	1	Mascot
2841.405	2841.3484	-0.0566	-20	217	241	LTTPTYGDLNHLVSATMS GVTTCLR	115	100	(N-term)_iTRAQ[0], MMTS (C)[23]	[1] F3 030912	548/540	0.897	1.100	1.024	1	Mascot

15	tubulin T beta15	gi|224839	52578.5	20	1319	1.163	1.046	1.120	0.749	0.542	0.657	60	60	60	100

Peptide Information

Calc. Mass	Obsrv. Mass	± da	± ppm	Start	End	Sequence	Ion	C. I. %	Modification	Plate [#] Name	Gel Idx/Pos [4700	iTRAQ	iTRAQ	iTRAQ	Rank	Result Type

				Seq.	Seq.		Score				Sample Name]	Ratio 115/114*	Ratio 116/114*	Ratio 117/114*		
1161.5862	1161.538	-0.0482	-41	351	359	TAVCDIPPR	34	99.07	(N-term)_iTRAQ[0], MMTS (C)[4]	[1] F3 030912	224/216	0.853	0.869	0.811	1	Mascot
1197.7115	1197.6688	-0.0427	-36	310	318	YLTVAAIFR	50	99.974	(N-term)_iTRAQ[0]	[1] F3 030912	419/411	1.133	1.104	1.346	1	Mascot
1198.4943	1198.4203	-0.074	-62	298	306	NMMAACDPR	42	99.828	(N-term)_iTRAQ[0], MMTS (C)[6]	[5] F4	175/167	1.069	1.113	1.167	1	Mascot
1221.6348	1221.6912	0.0564	46	155	162	IREEYPDR	27	95.568	(N-term)_iTRAQ[0]	[4] F7 and F10+11	1026/1018	0.939	1.088	1.289	1	Mascot
1274.6978	1274.6228	-0.075	-59	242	251	FPGQLNADLR	51	99.981	(N-term)_iTRAQ[0]	[5] F4	190/182	1.149	0.984	1.021	1	Mascot
1274.6978	1274.6488	-0.049	-38	242	251	FPGQLNADLR	43	99.868	(N-term)_iTRAQ[0]	[1] F3 030912	265/257	0.873	0.845	0.884	1	Mascot
1287.7367	1287.6992	-0.0375	-29	253	262	LAVNMVPFPR	35	99.138	(N-term)_iTRAQ[0]	[1] F3 030912	349/341	0.961	0.841	0.953	1	Mascot
1287.7367	1287.7393	0.0026	2	253	262	LAVNMVPFPR	32	98.442	(N-term)_iTRAQ[0]	[1] F3 030912	716/708	0.788	0.478	0.836	1	Mascot
1373.7008	1373.6394	-0.0614	-45	381	390	ISEQFTAMFR	62	99.999	(N-term)_iTRAQ[0]	[5] F4	284/276	0.941	0.970	1.014	1	Mascot
1373.7008	1373.7096	0.0088	6	381	390	ISEQFTAMFR	27	95.141	(N-term)_iTRAQ[0]	[5] F4	591/583	0.831	1.259	1.157	1	Mascot
1546.8951	1546.9545	0.0594	38	242	252	FPGQLNADLRK	46	99.945	(N-term)_iTRAQ[0], Lysine(K)_iTRAQ[11]	[3] F6 and F9	1213/1205	1.144	0.783	1.344	1	Mascot
1559.9342	1559.8873	-0.0469	-30	252	262	KLAVNMVPFPR	33	98.897	(N-term)_iTRAQ[0], Lysine(K)_iTRAQ[1]	[6] F8 110912	367/359	0.926	0.879	0.841	1	Mascot
1559.9342	1560.0081	0.0739	47	252	262	KLAVNMVPFPR	45	99.924	(N-term)_iTRAQ[0], Lysine(K)_iTRAQ[1]	[3] F6 and F9	1277/1269	1.868	1.166	1.510	1	Mascot
1639.8798	1639.7889	-0.0909	-55	163	174	IMNTFSVMPSPK	58	99.996	(N-term)_iTRAQ[0], Lysine(K)_iTRAQ[12]	[5] F4	242/234	1.063	1.290	0.925	1	Mascot
1643.8639	1643.7812	-0.0827	-50	47	58	INVYYNEAAGNK	66	99.999	(N-term)_iTRAQ[0], Lysine(K)_iTRAQ[12]	[7] F5 120912	182/174	1.156	1.205	1.297	1	Mascot
1643.8639	1643.8152	-0.0487	-30	47	58	INVYYNEAAGNK	50	99.978	(N-term)_iTRAQ[0], Lysine(K)_iTRAQ[12]	[3] F6 and F9	223/215	2.360	2.748	1.721	1	Mascot
1643.8639	1643.9257	0.0618	38	47	58	INVYYNEAAGNK	48	99.958	(N-term)_iTRAQ[0], Lysine(K)_iTRAQ[12]	[3] F6 and F9	1133/1125	0.924	0.748	0.778	1	Mascot
1734.8942	1734.7839	-0.1103	-64	325	336	EVDEQMLNVQNK	63	99.999	(N-term)_iTRAQ[0], Lysine(K)_iTRAQ[12]	[5] F4	140/132	0.980	0.919	0.865	1	Mascot
1734.8942	1734.8132	-0.081	-47	325	336	EVDEQMLNVQNK	82	100	(N-term)_iTRAQ[0], Lysine(K)_iTRAQ[12]	[7] F5 120912	177/169	1.326	1.321	1.178	1	Mascot
1734.8942	1734.9392	0.045	26	325	336	EVDEQMLNVQNK	31	98.248	(N-term)_iTRAQ[0], Lysine(K)_iTRAQ[12]	[5] F4	591/583	0.461	0.361	0.692	1	Mascot
1750.8892	1750.7924	-0.0968	-55	325	336	EVDEQMLNVQNK	77	100	(N-term)_iTRAQ[0], Lysine(K)_iTRAQ[12], Oxidation (M)[6]	[5] F4	105/97	1.124	1.154	1.249	1	Mascot
1759.9384	1759.9335	-0.0049	-3	63	77	AILVDLEPGTMDSVR	75	100	(N-term)_iTRAQ[0]	[8] F13-15 and F1+2	408/400	1.012	1.229	0.951	1	Mascot
1759.9384	1759.9364	-0.002	-1	63	77	AILVDLEPGTMDSVR	64	99.999	(N-term)_iTRAQ[0]	[8] F13-15 and F1+2	420/412	1.115	1.414	1.398	1	Mascot
1759.9384	1759.9373	-0.0011	-1	63	77	AILVDLEPGTMDSVR	52	99.984	(N-term)_iTRAQ[0]	[8] F13-15 and F1+2	461/453	1.241	1.771	1.343	1	Mascot
1759.9384	1759.9391	0.0007	0	63	77	AILVDLEPGTMDSVR	73	100	(N-term)_iTRAQ[0]	[8] F13-15 and F1+2	466/458	1.176	0.994	0.979	1	Mascot
1759.9384	1759.9392	0.0008	0	63	77	AILVDLEPGTMDSVR	30	97.57	(N-term)_iTRAQ[0]	[8] F13-15 and F1+2	516/508	1.051	1.080	0.935	1	Mascot
1759.9384	1759.946	0.0076	4	63	77	AILVDLEPGTMDSVR	93	100	(N-term)_iTRAQ[0]	[8] F13-15 and F1+2	412/404	1.164	1.244	1.086	1	Mascot
1759.9384	1759.9512	0.0128	7	63	77	AILVDLEPGTMDSVR	33	98.686	(N-term)_iTRAQ[0]	[8] F13-15 and F1+2	512/504	1.092	1.100	1.180	1	Mascot
1759.9384	1759.9528	0.0144	8	63	77	AILVDLEPGTMDSVR	68	100	(N-term)_iTRAQ[0]	[8] F13-15 and F1+2	404/396	0.658	0.704	0.767	1	Mascot
1759.9384	1759.954	0.0156	9	63	77	AILVDLEPGTMDSVR	66	99.999	(N-term)_iTRAQ[0]	[8] F13-15 and F1+2	417/409	0.952	1.015	0.884	1	Mascot
1759.9384	1759.9597	0.0213	12	63	77	AILVDLEPGTMDSVR	28	95.748	(N-term)_iTRAQ[0]	[8] F13-15 and F1+2	523/515	0.828	0.208	0.738	1	Mascot
1764.938	1764.9121	-0.0259	-15	263	276	LHFFMPGFAPLTSR	61	99.998	(N-term)_iTRAQ[0]	[3] F6 and F9	456/448	0.729	1.073	1.164	1	Mascot
1764.938	1764.9736	0.0356	20	263	276	LHFFMPGFAPLTSR	47	99.95	(N-term)_iTRAQ[0]	[4] F7 and F10+11	470/462	0.960	1.090	1.164	1	Mascot
1764.938	1765.0078	0.0698	40	263	276	LHFFMPGFAPLTSR	38	99.601	(N-term)_iTRAQ[0]	[4] F7 and F10+11	531/523	0.891	1.360	1.548	1	Mascot
1985.0378	1984.9299	-0.1079	-54	337	350	NSSYFVEWIPNNVK	39	99.708	(N-term)_iTRAQ[0], Lysine(K)_iTRAQ[14]	[5] F4	298/290	1.071	1.010	1.098	1	Mascot
1985.0378	1984.957	-0.0808	-41	337	350	NSSYFVEWIPNNVK	43	99.868	(N-term)_iTRAQ[0], Lysine(K)_iTRAQ[14]	[6] F8 110912	369/361	0.630	0.821	0.824	1	Mascot
1985.0378	1984.9778	-0.06	-30	337	350	NSSYFVEWIPNNVK	32	98.529	(N-term)_iTRAQ[0], Lysine(K)_iTRAQ[14]	[6] F8 110912	363/355	1.422	0.707	0.524	1	Mascot
1985.0378	1984.988	-0.0498	-25	337	350	NSSYFVEWIPNNVK	30	97.598	(N-term)_iTRAQ[0], Lysine(K)_iTRAQ[14]	[5] F4	339/331	1.470	1.187	0.924	1	Mascot
1996.0671	1996.0134	-0.0537	-27	283	297	ALTVPELTQQMFDSK	73	100	(N-term)_iTRAQ[0], Lysine(K)_iTRAQ[15]	[1] F3 030912	408/400	1.193	1.015	1.075	1	Mascot
1996.0671	1996.0165	-0.0506	-25	283	297	ALTVPELTQQMFDSK	50	99.973	(N-term)_iTRAQ[0], Lysine(K)_iTRAQ[15]	[2] F12 040912	441/433	1.145	1.136	0.998	1	Mascot
1996.0671	1996.0317	-0.0354	-18	283	297	ALTVPELTQQMFDSK	33	98.889	(N-term)_iTRAQ[0], Lysine(K)_iTRAQ[15]	[5] F4	321/313	1.425	1.261	1.308	1	Mascot
1996.0671	1996.0551	-0.012	-6	283	297	ALTVPELTQQMFDSK	34	99.099	(N-term)_iTRAQ[0], Lysine(K)_iTRAQ[15]	[1] F3 030912	665/657	1.874	1.259	2.678	1	Mascot
2100.092	2099.9648	-0.1272	-61	3	19	EIVHIQAGQCGNQIGAK	66	99.999	(N-term)_iTRAQ[0], Lysine(K)_iTRAQ[17], MMTS (C)[10]	[6] F8 110912	261/253	0.727	0.775	0.614	1	Mascot
2100.092	2100.0605	-0.0315	-15	3	19	EIVHIQAGQCGNQIGAK	109	100	(N-term)_iTRAQ[0], Lysine(K)_iTRAQ[17], MMTS (C)[10]	[4] F7 and F10+11	265/257	1.064	0.910	1.094	1	Mascot

2100.092	2100.1162	0.0242	12	3	19	EIVHIQAGQCGNQIGAK	89	100	(N-term)_iTRAQ[0], Lysine(K)_iTRAQ[17], MMTS (C)[10]	[4] F7 and F10+11	379/371	1.194	0.967	0.685	1	Mascot
2100.092	2100.1257	0.0337	16	3	19	EIVHIQAGQCGNQIGAK	41	99.786	(N-term)_iTRAQ[0], Lysine(K)_iTRAQ[17], MMTS (C)[10]	[4] F7 and F10+11	631/623	1.893	1.942	1.642	1	Mascot
2100.092	2100.1853	0.0933	44	3	19	EIVHIQAGQCGNQIGAK	37	99.482	(N-term)_iTRAQ[0], Lysine(K)_iTRAQ[17], MMTS (C)[10]	[4] F7 and F10+11	558/550	1.799	1.038	2.313	1	Mascot
2103.0842	2103.0032	-0.081	-39	104	121	GHYTEGAELVDSVLDVVR	37	99.506	(N-term)_iTRAQ[0]	[6] F8 110912	488/480	1.671	0.987	0.997	1	Mascot
2103.0842	2103.0552	-0.029	-14	104	121	GHYTEGAELVDSVLDVVR	37	99.468	(N-term)_iTRAQ[0]	[3] F6 and F9	722/714	3.032	1.634	1.575	1	Mascot
2103.0842	2103.0581	-0.0261	-12	104	121	GHYTEGAELVDSVLDVVR	53	99.987	(N-term)_iTRAQ[0]	[2] F12 040912	496/488	1.368	1.498	3.600	1	Mascot
2103.0842	2103.0779	-0.0063	-3	104	121	GHYTEGAELVDSVLDVVR	54	99.991	(N-term)_iTRAQ[0]	[7] F5 120912	465/457	0.957	0.667	1.064	1	Mascot
2103.0842	2103.0789	-0.0053	-3	104	121	GHYTEGAELVDSVLDVVR	48	99.96	(N-term)_iTRAQ[0]	[3] F6 and F9	481/473	1.228	1.113	1.179	1	Mascot
2103.0842	2103.1221	0.0379	18	104	121	GHYTEGAELVDSVLDVVR	111	100	(N-term)_iTRAQ[0]	[4] F7 and F10+11	489/481	1.151	0.950	0.918	1	Mascot
2103.0842	2103.1589	0.0747	36	104	121	GHYTEGAELVDSVLDVVR	119	100	(N-term)_iTRAQ[0]	[4] F7 and F10+11	1342/1334	0.615	0.767	0.660	1	Mascot
2103.0842	2103.1692	0.085	40	104	121	GHYTEGAELVDSVLDVVR	36	99.343	(N-term)_iTRAQ[0]	[4] F7 and F10+11	1318/1310	14.597	2.288	6.391	1	Mascot
2146.1465	2146.0693	-0.0772	-36	363	379	MSATFIGNSTAIQELFK	109	100	(N-term)_iTRAQ[0], Lysine(K)_iTRAQ[17]	[1] F3 030912	473/465	0.984	0.914	1.090	1	Mascot
2146.1465	2146.0789	-0.0676	-31	363	379	MSATFIGNSTAIQELFK	36	99.392	(N-term)_iTRAQ[0], Lysine(K)_iTRAQ[17]	[5] F4	370/362	1.391	1.691	1.438	1	Mascot
2146.1465	2146.1436	-0.0029	-1	363	379	MSATFIGNSTAIQELFK	40	99.78	(N-term)_iTRAQ[0], Lysine(K)_iTRAQ[17]	[1] F3 030912	616/608	0.754	0.699	0.278	1	Mascot
2841.405	2841.3303	-0.0747	-26	217	241	LTTPTYGDLNHLVSATMSGVTTCLR	61	99.998	(N-term)_iTRAQ[0], MMTS (C)[23]	[1] F3 030912	521/513	4.790	3.531	3.426	1	Mascot
2841.405	2841.3484	-0.0566	-20	217	241	LTTPTYGDLNHLVSATMSGVTTCLR	115	100	(N-term)_iTRAQ[0], MMTS (C)[23]	[1] F3 030912	548/540	0.897	1.100	1.024	1	Mascot

16	sodium/potassium-transporting ATPase subunit alpha-2 gi|6978545 precursor [Rattus norvegicus]	121272.9	23	1314	1.162	0.987	0.992	0.411	0.388	0.312	32	32	32	100

Peptide Information

Calc. Mass	Obsrv. Mass	± da	± ppm	Start Seq.	End Seq.	Sequence	Ion Score	C. I. %	Modification	Plate [#] Name	Gel Idx/Pos [4700 Sample Name]	iTRAQ Ratio 115/114*	iTRAQ Ratio 116/114*	iTRAQ Ratio 117/114*	Rank	Result Type
1115.667	1115.6121	-0.0549	-49	594	602	AAVPDAVGK	61	99.998	(N-term)_iTRAQ[0], Lysine(K)_iTRAQ[9]	[7] F5 120912	113/105	0.699	0.645	0.635	1	Mascot
1150.7081	1150.6692	-0.0389	-34	764	770	LIFDNLK	36	99.428	(N-term)_iTRAQ[0], Lysine(K)_iTRAQ[7]	[7] F5 120912	289/281	1.128	0.944	0.789	1	Mascot
1220.6597	1220.5994	-0.0603	-49	696	704	LIIVEGCQR	28	96.28	(N-term)_iTRAQ[0], MMTS (C)[7]	[5] F4	234/226	0.711	1.038	0.564	1	Mascot
1220.6597	1220.6172	-0.0425	-35	696	704	LIIVEGCQR	48	99.965	(N-term)_iTRAQ[0], MMTS (C)[7]	[1] F3 030912	310/302	0.968	0.922	0.948	1	Mascot
1231.6555	1231.5898	-0.0657	-53	681	689	DHTEIVFAR	42	99.837	(N-term)_iTRAQ[0]	[6] F8 110912	200/192	0.798	0.921	0.829	1	Mascot
1320.7369	1320.6796	-0.0573	-43	433	442	AGQENISVSK	35	99.163	(N-term)_iTRAQ[0], Lysine(K)_iTRAQ[10]	[3] F6 and F9	119/111	2.198	2.937	2.007	1	Mascot
1379.7681	1379.8309	0.0628	46	1008	1016	RYPGGWVEK	29	96.826	(N-term)_iTRAQ[0], Lysine(K)_iTRAQ[9]	[4] F7 and F10+11	1096/1088	1.249	1.103	1.218	1	Mascot
1380.8083	1380.7411	-0.0672	-49	645	655	LNIPVSQVNPR	42	99.834	(N-term)_iTRAQ[0]	[1] F3 030912	238/230	1.087	0.795	0.948	1	Mascot
1412.8168	1412.7659	-0.0509	-36	161	171	NMVPQQALVIR	30	97.372	(N-term)_iTRAQ[0]	[1] F3 030912	265/257	1.559	1.149	1.196	1	Mascot
1412.8168	1412.8419	0.0251	18	161	171	NMVPQQALVIR	55	99.992	(N-term)_iTRAQ[0]	[8] F13-15 and F1+2	499/491	1.096	1.031	1.016	1	Mascot
1412.8168	1412.8593	0.0425	30	161	171	NMVPQQALVIR	39	99.68	(N-term)_iTRAQ[0]	[8] F13-15 and F1+2	555/547	1.131	0.931	0.923	1	Mascot
1412.8485	1412.8945	0.046	33	52	59	KYQVDLSK	46	99.94	(N-term)_iTRAQ[0], Lysine(K)_iTRAQ[1,8]	[4] F7 and F10+11	1066/1058	1.141	0.981	1.007	1	Mascot
1422.9055	1422.95	0.0445	31	764	771	LIFDNLKK	43	99.87	(N-term)_iTRAQ[0], Lysine(K)_iTRAQ[7,8]	[4] F7 and F10+11	1176/1168	1.407	1.125	1.304	1	Mascot
1507.8367	1507.8013	-0.0354	-23	475	485	VAEIPFNSTNK	63	99.999	(N-term)_iTRAQ[0], Lysine(K)_iTRAQ[11]	[7] F5 120912	191/183	1.239	1.096	1.129	1	Mascot
1657.8829	1657.7828	-0.1001	-60	494	505	EDSPQSHVLVMK	57	99.995	(N-term)_iTRAQ[0], Lysine(K)_iTRAQ[12]	[6] F8 110912	199/191	1.668	1.229	1.599	1	Mascot
1657.8829	1657.8474	-0.0355	-21	494	505	EDSPQSHVLVMK	77	100	(N-term)_iTRAQ[0], Lysine(K)_iTRAQ[12]	[4] F7 and F10+11	196/188	1.316	1.037	0.982	1	Mascot
1669.9557	1669.916	-0.0397	-24	610	622	VIMVTGDHPITAK	78	100	(N-term)_iTRAQ[0], Lysine(K)_iTRAQ[13]	[3] F6 and F9	232/224	0.670	0.709	0.702	1	Mascot
1669.9557	1669.9308	-0.0249	-15	610	622	VIMVTGDHPITAK	86	100	(N-term)_iTRAQ[0], Lysine(K)_iTRAQ[13]	[4] F7 and F10+11	234/226	1.185	1.115	1.045	1	Mascot
1700.8364	1700.8555	0.0191	11	226	238	SPEFTHENPLETR	47	99.948	(N-term)_iTRAQ[0]	[4] F7 and F10+11	220/212	1.354	1.041	0.809	1	Mascot
1709.9309	1709.8496	-0.0813	-48	549	561	VLGFCQLNLPSGK	36	99.399	(N-term)_iTRAQ[0], Lysine(K)_iTRAQ[13], MMTS (C)[5]	[5] F4	343/335	1.220	0.480	1.074	1	Mascot
1743.8687	1743.7974	-0.0713	-41	568	579	FDTDELNFPTEK	63	99.999	(N-term)_iTRAQ[0], Lysine(K)_iTRAQ[12]	[5] F4	220/212	1.287	1.235	0.895	1	Mascot
1763.8531	1763.7506	-0.1025	-58	211	225	VDNSSLTGESEPQTR	77	100	(N-term)_iTRAQ[0]	[1] F3 030912	136/128	1.082	1.168	1.150	1	Mascot

1974.0264	1974.0244	-0.002	-1	627	644	GVGIISEGNETVEDIAAR	48	99.96	(N-term)_iTRAQ[0]	[8] F13-15 and F1+2	454/446	1.135	0.930	0.924	1	Mascot
1974.0264	1974.0256	-0.0008	0	627	644	GVGIISEGNETVEDIAAR	52	99.984	(N-term)_iTRAQ[0]	[8] F13-15 and F1+2	446/438	1.254	0.827	1.022	1	Mascot
1974.0264	1974.0314	0.005	3	627	644	GVGIISEGNETVEDIAAR	79	100	(N-term)_iTRAQ[0]	[8] F13-15 and F1+2	450/442	1.181	0.894	1.002	1	Mascot
1974.0264	1974.0327	0.0063	3	627	644	GVGIISEGNETVEDIAAR	31	98.048	(N-term)_iTRAQ[0]	[8] F13-15 and F1+2	503/495	1.407	1.001	1.440	1	Mascot
1974.0264	1974.0538	0.0274	14	627	644	GVGIISEGNETVEDIAAR	40	99.731	(N-term)_iTRAQ[0]	[8] F13-15 and F1+2	555/547	0.853	0.908	0.967	1	Mascot
2100.1545	2100.0515	-0.103	-49	705	723	QGAIVAVTGDGVNDSPALK	112	100	(N-term)_iTRAQ[0], Lysine(K)_iTRAQ[19]	[1] F3 030912	255/247	1.065	0.886	0.737	1	Mascot
2108.1274	2108.0452	-0.0822	-39	73	89	DGPNALTPPPTTPEWVK	57	99.995	(N-term)_iTRAQ[0], Lysine(K)_iTRAQ[17]	[1] F3 030912	289/281	1.020	0.625	0.647	1	Mascot
2202.0859	2202.0366	-0.0493	-22	358	375	NLEAVETLGSTSTICSDK	99	100	(N-term)_iTRAQ[0], Lysine(K)_iTRAQ[18], MMTS (C)[15]	[1] F3 030912	363/355	1.008	0.712	0.912	1	Mascot
2372.3521	2372.2949	-0.0572	-24	705	724	QGAIVAVTGDGVNDSPALKK	30	97.508	(N-term)_iTRAQ[0], Lysine(K)_iTRAQ[19, 20]	[4] F7 and F10+11	246/238	0.887	0.802	0.929	1	Mascot
2608.3049	2608.2537	-0.0512	-20	741	763	QAADMILLDDNFASIVTGVEEGR	55	99.992	(N-term)_iTRAQ[0]	[1] F3 030912	548/540	3.111	2.449	1.643	1	Mascot

17	aconitate hydratase, mitochondrial precursor [Rattus norvegicus]	gi|40538860	93759.3	21	1280	0.790	0.930	1.005	0.295	0.243	0.251	26	26	26	100

Peptide Information

Calc. Mass	Obsrv. Mass	± da	± ppm	Start Seq.	End Seq.	Sequence	Ion Score	C. I. %	Modification	Plate [#] Name	Gel Idx/Pos [4700 Sample Name]	iTRAQ Ratio 115/114*	iTRAQ Ratio 116/114*	iTRAQ Ratio 117/114*	Rank	Result Type
1060.6976	1060.6705	-0.0271	-26	724	730	LTIQGLK	38	99.628	(N-term)_iTRAQ[0], Lysine(K)_iTRAQ[7]	[7] F5 120912	214/206	0.666	0.644	1.033	1	Mascot
1079.5969	1079.5262	-0.0707	-65	430	437	DGYAQILR	30	97.68	(N-term)_iTRAQ[0]	[5] F4	151/143	0.491	0.938	0.564	1	Mascot
1129.6127	1129.5725	-0.0402	-36	371	378	EGWPLDIR	30	97.664	(N-term)_iTRAQ[0]	[7] F5 120912	287/279	1.132	1.825	1.027	1	Mascot
1508.8206	1508.787	-0.0336	-22	313	323	ADIANLAEEFK	59	99.997	(N-term)_iTRAQ[0], Lysine(K)_iTRAQ[11]	[7] F5 120912	340/332	0.926	0.785	1.019	1	Mascot
1556.8207	1556.7473	-0.0734	-47	507	517	FNPETDFLTGK	66	99.999	(N-term)_iTRAQ[0], Lysine(K)_iTRAQ[11]	[5] F4	232/224	0.565	0.908	1.313	1	Mascot
1556.8207	1556.7748	-0.0459	-29	507	517	FNPETDFLTGK	45	99.913	(N-term)_iTRAQ[0], Lysine(K)_iTRAQ[11]	[7] F5 120912	291/283	0.546	0.782	1.148	1	Mascot
1607.8513	1607.7717	-0.0796	-50	412	424	SQFTITPGSEQIR	61	99.998	(N-term)_iTRAQ[0]	[1] F3 030912	230/222	0.865	0.954	1.034	1	Mascot
1709.8441	1709.8309	-0.0132	-8	32	44	VAMSHFEPSEYIR	67	100	(N-term)_iTRAQ[0]	[4] F7 and F10+11	305/297	0.876	0.788	1.166	1	Mascot
1745.8943	1745.9364	0.0421	24	634	648	NAVTQEFGPVPDTAR	81	100	(N-term)_iTRAQ[0]	[8] F13-15 and F1+2	532/524	0.929	0.969	1.109	1	Mascot
1788.9741	1788.9293	-0.0448	-25	522	534	FKLEAPDADELPR	69	100	(N-term)_iTRAQ[0], Lysine(K)_iTRAQ[2]	[4] F7 and F10+11	295/287	0.727	0.811	0.908	1	Mascot
1811.8684	1811.7925	-0.0759	-42	657	671	WVVIGDENYGEGSSR	90	100	(N-term)_iTRAQ[0]	[1] F3 030912	286/278	1.121	1.022	1.192	1	Mascot
1811.8684	1811.9004	0.032	18	657	671	WVVIGDENYGEGSSR	71	100	(N-term)_iTRAQ[0]	[8] F13-15 and F1+2	482/474	0.870	0.912	0.955	1	Mascot
1833.9219	1833.9116	-0.0103	-6	592	605	CTTDHISAAGPWLK	78	100	(N-term)_iTRAQ[0], Lysine(K)_iTRAQ[14], MMTS (C)[1]	[4] F7 and F10+11	307/299	0.782	0.670	0.942	1	Mascot
1894.0647	1894.0699	0.0052	3	731	743	DFAPGKPLNCIIK	49	99.966	(N-term)_iTRAQ[0], Lysine(K)_iTRAQ[6,13], MMTS (C)[10]	[4] F7 and F10+11	356/348	0.595	0.662	0.983	1	Mascot
1978.8773	1978.9031	0.0258	13	379	395	VGLIGSCTNSSYEDMGR	28	96.288	(N-term)_iTRAQ[0], MMTS (C)[7]	[8] F13-15 and F1+2	442/434	0.743	0.901	0.798	1	Mascot
2013.0161	2012.9655	-0.0506	-25	69	84	IVYGHLDDPANQEIER	38	99.633	(N-term)_iTRAQ[0]	[3] F6 and F9	256/248	1.215	1.396	1.127	1	Mascot
2013.0161	2013.0858	0.0697	35	69	84	IVYGHLDDPANQEIER	32	98.519	(N-term)_iTRAQ[0]	[4] F7 and F10+11	1098/1090	0.937	1.000	0.693	1	Mascot
2019.9658	2019.8405	-0.1253	-62	535	549	SDFDPGQDTYQHPPK	76	100	(N-term)_iTRAQ[0], Lysine(K)_iTRAQ[15]	[6] F8 110912	182/174	1.132	0.880	1.138	1	Mascot
2019.9658	2019.9231	-0.0427	-21	535	549	SDFDPGQDTYQHPPK	69	100	(N-term)_iTRAQ[0], Lysine(K)_iTRAQ[15]	[4] F7 and F10+11	182/174	0.818	0.910	0.954	1	Mascot
2019.9658	2020.0353	0.0695	34	535	549	SDFDPGQDTYQHPPK	42	99.86	(N-term)_iTRAQ[0], Lysine(K)_iTRAQ[15]	[4] F7 and F10+11	1071/1063	0.339	0.798	1.027	1	Mascot
2021.1443	2021.0381	-0.1062	-53	565	577	LQLLEPFDKWDGK	73	100	(N-term)_iTRAQ[0], Lysine(K)_iTRAQ[9,13]	[6] F8 110912	424/416	0.940	0.977	1.072	1	Mascot
2042.0804	2041.9954	-0.085	-42	145	160	DINQEVYNFLATAGAK	52	99.983	(N-term)_iTRAQ[0], Lysine(K)_iTRAQ[16]	[5] F4	360/352	0.837	1.496	1.132	1	Mascot
2118.2183	2118.1335	-0.0848	-40	574	587	WDGKDLEDLQILIK	73	100	(N-term)_iTRAQ[0], Lysine(K)_iTRAQ[4,14]	[6] F8 110912	443/435	0.512	0.946	0.552	1	Mascot
2339.2983	2339.3086	0.0103	4	701	717	KQGLLPLTFADPSDYNK	31	98.186	(N-term)_iTRAQ[0], Lysine(K)_iTRAQ[1,17]	[4] F7 and F10+11	364/356	0.655	0.929	1.206	1	Mascot
2385.3149	2385.217	-0.0979	-41	143	160	AKDINQEVYNFLATAGAK	86	100	(N-term)_iTRAQ[0], Lysine(K)_iTRAQ[2,18]	[6] F8 110912	444/436	0.878	0.862	1.212	1	Mascot
2626.3652	2626.2888	-0.0764	-29	96	117	VAMQDATAQMAMLQFISSGLPK	105	100	(N-term)_iTRAQ[0], Lysine(K)_iTRAQ[22]	[1] F3 030912	534/526	1.443	1.142	1.422	1	Mascot

18	tubulin beta-2C chain [Rattus norvegicus]	gi|40018568	52442.5	19	1221	1.190	1.031	1.095	0.801	0.419	0.672	49	49	49	100

Peptide Information

Calc. Mass	Obsrv. Mass	± da	± ppm	Start Seq.	End Seq.	Sequence	Ion Score	C. I. %	Modification	Plate [#] Name	Gel Idx/Pos [4700 Sample Name]	iTRAQ Ratio 115/114*	iTRAQ Ratio 116/114*	iTRAQ Ratio 117/114*	Rank	Result Type
1161.5862	1161.538	-0.0482	-41	351	359	TAVCDIPPR	34	99.07	(N-term)_iTRAQ[0], MMTS (C)[4]	[1] F3 030912	224/216	0.853	0.869	0.811	1	Mascot
1183.6959	1183.6681	-0.0278	-23	310	318	YLTVAAVFR	51	99.981	(N-term)_iTRAQ[0]	[1] F3 030912	375/367	1.511	1.264	1.071	1	Mascot
1198.4943	1198.4203	-0.074	-62	298	306	NMMAACDPR	42	99.828	(N-term)_iTRAQ[0], MMTS (C)[6]	[5] F4	175/167	1.069	1.113	1.167	1	Mascot
1221.6348	1221.6912	0.0564	46	155	162	IREEYPDR	27	95.568	(N-term)_iTRAQ[0]	[4] F7 and F10+11	1026/1018	0.939	1.088	1.289	1	Mascot
1274.6978	1274.6228	-0.075	-59	242	251	FPGQLNADLR	51	99.981	(N-term)_iTRAQ[0]	[5] F4	190/182	1.149	0.984	1.021	1	Mascot
1274.6978	1274.6488	-0.049	-38	242	251	FPGQLNADLR	43	99.868	(N-term)_iTRAQ[0]	[1] F3 030912	265/257	0.873	0.845	0.884	1	Mascot
1287.7367	1287.6992	-0.0375	-29	253	262	LAVNMVPFPR	35	99.138	(N-term)_iTRAQ[0]	[1] F3 030912	349/341	0.961	0.841	0.953	1	Mascot
1287.7367	1287.7393	0.0026	2	253	262	LAVNMVPFPR	32	98.442	(N-term)_iTRAQ[0]	[1] F3 030912	716/708	0.788	0.478	0.836	1	Mascot
1373.7008	1373.6394	-0.0614	-45	381	390	ISEQFTAMFR	62	99.999	(N-term)_iTRAQ[0]	[5] F4	284/276	0.941	0.970	1.014	1	Mascot
1373.7008	1373.7096	0.0088	6	381	390	ISEQFTAMFR	27	95.141	(N-term)_iTRAQ[0]	[5] F4	591/583	0.831	1.259	1.157	1	Mascot
1546.8951	1546.9545	0.0594	38	242	252	FPGQLNADLRK	46	99.945	(N-term)_iTRAQ[0], Lysine(K)_iTRAQ[11]	[3] F6 and F9	1213/1205	1.144	0.783	1.344	1	Mascot
1559.9342	1559.8873	-0.0469	-30	252	262	KLAVNMVPFPR	33	98.897	(N-term)_iTRAQ[0], Lysine(K)_iTRAQ[1]	[6] F8 110912	367/359	0.926	0.879	0.841	1	Mascot
1559.9342	1560.0081	0.0739	47	252	262	KLAVNMVPFPR	45	99.924	(N-term)_iTRAQ[0], Lysine(K)_iTRAQ[1]	[3] F6 and F9	1277/1269	1.868	1.166	1.510	1	Mascot
1607.9077	1607.8265	-0.0812	-51	163	174	IMNTFSVVPSPK	64	99.999	(N-term)_iTRAQ[0], Lysine(K)_iTRAQ[12]	[5] F4	234/226	0.954	0.876	1.059	1	Mascot
1616.8529	1616.7706	-0.0823	-51	47	58	INVYYNEATGGK	27	95.548	(N-term)_iTRAQ[0], Lysine(K)_iTRAQ[12]	[7] F5 120912	165/157	2.345	2.320	1.894	1	Mascot
1616.8529	1616.8282	-0.0247	-15	47	58	INVYYNEATGGK	87	100	(N-term)_iTRAQ[0], Lysine(K)_iTRAQ[12]	[7] F5 120912	201/193	1.062	0.906	0.966	1	Mascot
1734.8942	1734.7839	-0.1103	-64	325	336	EVDEQMLNVQNK	63	99.999	(N-term)_iTRAQ[0], Lysine(K)_iTRAQ[12]	[5] F4	140/132	0.980	0.919	0.865	1	Mascot
1734.8942	1734.8132	-0.081	-47	325	336	EVDEQMLNVQNK	82	100	(N-term)_iTRAQ[0], Lysine(K)_iTRAQ[12]	[7] F5 120912	177/169	1.326	1.321	1.178	1	Mascot
1734.8942	1734.9392	0.045	26	325	336	EVDEQMLNVQNK	31	98.248	(N-term)_iTRAQ[0], Lysine(K)_iTRAQ[12]	[5] F4	591/583	0.461	0.361	0.692	1	Mascot
1745.9227	1745.9333	0.0106	6	63	77	AVLVDLEPGTMDSVR	82	100	(N-term)_iTRAQ[0]	[8] F13-15 and F1+2	438/430	1.231	1.027	1.048	1	Mascot
1745.9227	1745.9403	0.0176	10	63	77	AVLVDLEPGTMDSVR	63	99.999	(N-term)_iTRAQ[0]	[8] F13-15 and F1+2	442/434	1.364	1.066	0.943	1	Mascot
1745.9227	1745.9579	0.0352	20	63	77	AVLVDLEPGTMDSVR	52	99.983	(N-term)_iTRAQ[0]	[8] F13-15 and F1+2	547/539	2.229	1.285	1.043	1	Mascot
1745.9227	1745.9629	0.0402	23	63	77	AVLVDLEPGTMDSVR	57	99.995	(N-term)_iTRAQ[0]	[8] F13-15 and F1+2	594/586	0.844	0.888	0.557	1	Mascot
1750.8892	1750.7924	-0.0968	-55	325	336	EVDEQMLNVQNK	77	100	(N-term)_iTRAQ[0], Lysine(K)_iTRAQ[12], Oxidation (M)[6]	[5] F4	105/97	1.124	1.154	1.249	1	Mascot
1764.938	1764.9121	-0.0259	-15	263	276	LHFFMPGFAPLTSR	61	99.998	(N-term)_iTRAQ[0]	[3] F6 and F9	456/448	0.729	1.073	1.164	1	Mascot
1764.938	1764.9736	0.0356	20	263	276	LHFFMPGFAPLTSR	47	99.95	(N-term)_iTRAQ[0]	[4] F7 and F10+11	470/462	0.960	1.090	1.164	1	Mascot
1764.938	1765.0078	0.0698	40	263	276	LHFFMPGFAPLTSR	38	99.601	(N-term)_iTRAQ[0]	[4] F7 and F10+11	531/523	0.891	1.360	1.548	1	Mascot
1980.0721	1980.0026	-0.0695	-35	283	297	ALTVPELTQQMFDAK	72	100	(N-term)_iTRAQ[0], Lysine(K)_iTRAQ[15]	[1] F3 030912	419/411	1.201	0.990	0.970	1	Mascot
1980.0721	1980.0112	-0.0609	-31	283	297	ALTVPELTQQMFDAK	59	99.997	(N-term)_iTRAQ[0], Lysine(K)_iTRAQ[15]	[2] F12 040912	448/440	2.288	1.835	2.450	1	Mascot
1985.0378	1984.9299	-0.1079	-54	337	350	NSSYFVEWIPNNVK	39	99.708	(N-term)_iTRAQ[0], Lysine(K)_iTRAQ[14]	[5] F4	298/290	1.071	1.010	1.098	1	Mascot
1985.0378	1984.957	-0.0808	-41	337	350	NSSYFVEWIPNNVK	43	99.868	(N-term)_iTRAQ[0], Lysine(K)_iTRAQ[14]	[6] F8 110912	369/361	0.630	0.821	0.824	1	Mascot
1985.0378	1984.9778	-0.06	-30	337	350	NSSYFVEWIPNNVK	32	98.529	(N-term)_iTRAQ[0], Lysine(K)_iTRAQ[14]	[6] F8 110912	363/355	1.422	0.707	0.524	1	Mascot
1985.0378	1984.988	-0.0498	-25	337	350	NSSYFVEWIPNNVK	30	97.598	(N-term)_iTRAQ[0], Lysine(K)_iTRAQ[14]	[5] F4	339/331	1.470	1.187	0.924	1	Mascot
2100.092	2099.9648	-0.1272	-61	3	19	EIVHLQAGQCGNQIGAK	66	99.999	(N-term)_iTRAQ[0], Lysine(K)_iTRAQ[17], MMTS (C)[10]	[6] F8 110912	261/253	0.727	0.775	0.614	1	Mascot
2100.092	2100.0605	-0.0315	-15	3	19	EIVHLQAGQCGNQIGAK	109	100	(N-term)_iTRAQ[0], Lysine(K)_iTRAQ[17], MMTS (C)[10]	[4] F7 and F10+11	265/257	1.064	0.910	1.094	1	Mascot
2100.092	2100.1162	0.0242	12	3	19	EIVHLQAGQCGNQIGAK	89	100	(N-term)_iTRAQ[0], Lysine(K)_iTRAQ[17], MMTS (C)[10]	[4] F7 and F10+11	379/371	1.194	0.967	0.685	1	Mascot
2100.092	2100.1257	0.0337	16	3	19	EIVHLQAGQCGNQIGAK	41	99.786	(N-term)_iTRAQ[0], Lysine(K)_iTRAQ[17], MMTS (C)[10]	[4] F7 and F10+11	631/623	1.893	1.942	1.642	1	Mascot
2100.092	2100.1853	0.0933	44	3	19	EIVHLQAGQCGNQIGAK	37	99.482	(N-term)_iTRAQ[0], Lysine(K)_iTRAQ[17], MMTS (C)[10]	[4] F7 and F10+11	558/550	1.799	1.038	2.313	1	Mascot
2103.0842	2103.0032	-0.081	-39	104	121	GHYTEGAELVDSVLDVVR	37	99.506	(N-term)_iTRAQ[0]	[6] F8 110912	488/480	1.671	0.987	0.997	1	Mascot
2103.0842	2103.0552	-0.029	-14	104	121	GHYTEGAELVDSVLDVVR	37	99.468	(N-term)_iTRAQ[0]	[3] F6 and F9	722/714	3.032	1.634	1.575	1	Mascot

2103.0842	2103.0581	-0.0261	-12	104	121	GHYTEGAELVDSVLDVVR	53	99.987	(N-term)_iTRAQ[0]	[2] F12 040912	496/488	1.368	1.498	3.600	1	Mascot
2103.0842	2103.0779	-0.0063	-3	104	121	GHYTEGAELVDSVLDVVR	54	99.991	(N-term)_iTRAQ[0]	[7] F5 120912	465/457	0.957	0.667	1.064	1	Mascot
2103.0842	2103.0789	-0.0053	-3	104	121	GHYTEGAELVDSVLDVVR	48	99.96	(N-term)_iTRAQ[0]	[3] F6 and F9	481/473	1.228	1.113	1.179	1	Mascot
2103.0842	2103.1221	0.0379	18	104	121	GHYTEGAELVDSVLDVVR	111	100	(N-term)_iTRAQ[0]	[4] F7 and F10+11	489/481	1.151	0.950	0.918	1	Mascot
2103.0842	2103.1589	0.0747	36	104	121	GHYTEGAELVDSVLDVVR	119	100	(N-term)_iTRAQ[0]	[4] F7 and F10+11	1342/1334	0.615	0.767	0.660	1	Mascot
2103.0842	2103.1692	0.085	40	104	121	GHYTEGAELVDSVLDVVR	36	99.343	(N-term)_iTRAQ[0]	[4] F7 and F10+11	1318/1310	14.597	2.288	6.391	1	Mascot
2146.1465	2146.0693	-0.0772	-36	363	379	MSATFIGNSTAIQELFK	109	100	(N-term)_iTRAQ[0], Lysine(K)_iTRAQ[17]	[1] F3 030912	473/465	0.984	0.914	1.090	1	Mascot
2146.1465	2146.0789	-0.0676	-31	363	379	MSATFIGNSTAIQELFK	36	99.392	(N-term)_iTRAQ[0], Lysine(K)_iTRAQ[17]	[5] F4	370/362	1.391	1.691	1.438	1	Mascot
2146.1465	2146.1436	-0.0029	-1	363	379	MSATFIGNSTAIQELFK	40	99.78	(N-term)_iTRAQ[0], Lysine(K)_iTRAQ[17]	[1] F3 030912	616/608	0.754	0.699	0.278	1	Mascot

19	2',3'-cyclic-nucleotide 3'-phosphodiesterase [Rattus norvegicus]	gi|57977323	53756.9	22	1171	1.923	0.905	0.852	0.590	0.384	0.246	29	29	29	100

Peptide Information

Calc. Mass	Obsrv. Mass	± da	± ppm	Start Seq.	End Seq.	Sequence	Ion Score	C. I. %	Modification	Plate [#] Name	Gel Idx/Pos [4700 Sample Name]	iTRAQ Ratio 115/114*	iTRAQ Ratio 116/114*	iTRAQ Ratio 117/114*	Rank	Result Type
1066.4928	1066.4633	-0.0295	-28	255	260	FCDYGK	31	98.186	(N-term)_iTRAQ[0], Lysine(K)_iTRAQ[6], MMTS (C)[2]	[3] F6 and F9	234/226	1.582	0.725	0.670	1	Mascot
1066.4928	1066.476	-0.0168	-16	255	260	FCDYGK	31	97.814	(N-term)_iTRAQ[0], Lysine(K)_iTRAQ[6], MMTS (C)[2]	[4] F7 and F10+11	234/226	2.690	1.424	1.156	1	Mascot
1124.6035	1124.5641	-0.0394	-35	155	161	LDCAQLK	41	99.798	(N-term)_iTRAQ[0], Lysine(K)_iTRAQ[7], MMTS (C)[3]	[3] F6 and F9	269/261	2.131	1.098	0.858	1	Mascot
1136.6748	1136.6591	-0.0157	-14	379	385	WMLSLAK	44	99.899	(N-term)_iTRAQ[0], Lysine(K)_iTRAQ[7]	[7] F5 120912	325/317	1.643	0.979	0.837	1	Mascot
1172.6232	1172.5817	-0.0415	-35	80	87	MVSADAYK	39	99.683	(N-term)_iTRAQ[0], Lysine(K)_iTRAQ[8]	[3] F6 and F9	170/162	3.463	2.095	2.047	1	Mascot
1276.6307	1276.5841	-0.0466	-37	94	101	ADFSEEYK	40	99.75	(N-term)_iTRAQ[0], Lysine(K)_iTRAQ[8]	[3] F6 and F9	178/170	2.885	1.247	1.260	1	Mascot
1307.7245	1307.6655	-0.059	-45	391	399	AIFTGYYGK	56	99.994	(N-term)_iTRAQ[0], Lysine(K)_iTRAQ[9]	[7] F5 120912	222/214	1.804	0.989	0.867	1	Mascot
1329.7664	1329.7661	-0.0003	0	235	243	LDLVSYFGK	60	99.998	(N-term)_iTRAQ[0], Lysine(K)_iTRAQ[9]	[7] F5 120912	369/361	2.019	0.759	0.832	1	Mascot
1329.7664	1329.8137	0.0473	36	235	243	LDLVSYFGK	34	99.021	(N-term)_iTRAQ[0], Lysine(K)_iTRAQ[9]	[8] F13-15 and F1+2	883/875	2.193	0.811	0.797	1	Mascot
1344.6029	1344.5643	-0.0386	-29	103	112	LDEDLAGYCR	46	99.943	(N-term)_iTRAQ[0], MMTS (C)[9]	[5] F4	257/249	2.175	1.283	1.023	1	Mascot
1432.7318	1432.8036	0.0718	50	94	102	ADFSEEYKR	30	97.536	(N-term)_iTRAQ[0], Lysine(K)_iTRAQ[8]	[4] F7 and F10+11	1050/1042	1.454	0.805	0.959	1	Mascot
1458.7308	1458.6704	-0.0604	-41	356	368	GGSQGEEVGELPR	29	97.065	(N-term)_iTRAQ[0]	[1] F3 030912	167/159	1.670	0.609	0.665	1	Mascot
1500.704	1500.765	0.061	41	102	112	RLDEDLAGYCR	55	99.993	(N-term)_iTRAQ[0], MMTS (C)[10]	[3] F6 and F9	1270/1262	1.796	0.815	0.537	1	Mascot
1500.704	1500.7739	0.0699	47	102	112	RLDEDLAGYCR	42	99.842	(N-term)_iTRAQ[0], MMTS (C)[10]	[4] F7 and F10+11	1207/1199	2.731	1.274	0.832	1	Mascot
1542.8475	1542.9379	0.0904	59	244	254	RPPGVLHCTTK	27	95.558	(N-term)_iTRAQ[0], Lysine(K)_iTRAQ[11], MMTS (C)[8]	[8] F13-15 and F1+2	1205/1197	2.201	0.984	0.873	1	Mascot
1647.8953	1647.8571	-0.0382	-23	164	174	NQWQLSLDDLK	62	99.998	(N-term)_iTRAQ[0], Lysine(K)_iTRAQ[11]	[7] F5 120912	345/337	2.235	1.150	0.861	1	Mascot
1680.8313	1680.7365	-0.0948	-56	261	274	ATGAEEYAQQDVVR	64	99.999	(N-term)_iTRAQ[0]	[1] F3 030912	189/181	1.833	1.032	0.713	1	Mascot
1680.8313	1680.7369	-0.0944	-56	261	274	ATGAEEYAQQDVVR	59	99.997	(N-term)_iTRAQ[0]	[1] F3 030912	164/156	2.204	0.927	0.714	1	Mascot
1680.8313	1680.7593	-0.072	-43	261	274	ATGAEEYAQQDVVR	31	98.07	(N-term)_iTRAQ[0]	[1] F3 030912	217/209	1.114	0.611	0.806	1	Mascot
1729.9482	1729.891	-0.0572	-33	204	216	AGQVFLEELGNHK	53	99.989	(N-term)_iTRAQ[0], Lysine(K)_iTRAQ[13]	[6] F8 110912	362/354	1.710	0.631	1.005	1	Mascot
1731.0063	1731.0734	0.0671	39	233	243	EKLDLVSYFGK	76	100	(N-term)_iTRAQ[0], Lysine(K)_iTRAQ[2,11]	[4] F7 and F10+11	1241/1233	2.352	1.135	0.945	1	Mascot
1836.9324	1836.9021	-0.0303	-16	261	275	ATGAEEYAQQDVVRR	33	98.9	(N-term)_iTRAQ[0]	[4] F7 and F10+11	178/170	1.353	0.553	0.866	1	Mascot
1920.0927	1920.1495	0.0568	30	164	175	NQWQLSLDDLKK	88	100	(N-term)_iTRAQ[0], Lysine(K)_iTRAQ[11, 12]	[4] F7 and F10+11	1220/1212	2.338	1.222	0.955	1	Mascot
1935.0667	1934.9886	-0.0781	-40	183	195	DFLPLYFGWFLTK	57	99.995	(N-term)_iTRAQ[0], Lysine(K)_iTRAQ[13]	[5] F4	439/431	1.844	0.702	0.775	1	Mascot
1935.0667	1935.0671	0.0004	0	183	195	DFLPLYFGWFLTK	53	99.988	(N-term)_iTRAQ[0], Lysine(K)_iTRAQ[13]	[5] F4	445/437	1.029	0.308	0.625	1	Mascot
2002.1456	2002.1371	-0.0085	-4	203	216	KAGQVFLEELGNHK	77	100	(N-term)_iTRAQ[0], Lysine(K)_iTRAQ[1,14]	[2] F12 040912	328/320	1.908	0.983	0.908	1	Mascot
2002.1456	2002.2092	0.0636	32	203	216	KAGQVFLEELGNHK	53	99.987	(N-term)_iTRAQ[0], Lysine(K)_iTRAQ[1,14]	[4] F7 and F10+11	1196/1188	1.580	0.871	0.583	1	Mascot

Calc. Mass	Obsrv. Mass	± da	± ppm	Start Seq.	End Seq.	Sequence	Ion Score	C. I. %	Modification	Plate [#] Name	Gel Idx/Pos [4700 Sample Name]	iTRAQ Ratio 115/114*	iTRAQ Ratio 116/114*	iTRAQ Ratio 117/114*	Rank	Result Type
2049.1353	2049.1924	0.0571	28	162	174	EKNQWQLSLDDLK	75	100	(N-term)_iTRAQ[0], Lysine(K)_iTRAQ[2,13]	[4] F7 and F10+11	1228/1220	2.336	1.081	0.975	1	Mascot
3924.0237	3923.8484	-0.1753	-45	294	327	TAGAQVVLNEQELQLWPSDLDKPSSSESLPPGSR	88	100	(N-term)_iTRAQ[0], Lysine(K)_iTRAQ[22]	[1] F3 030912	398/390	1.479	0.762	0.654	1	Mascot

20	heat shock protein HSP 90-alpha [Rattus norvegicus]	gi|28467005	96756	21	1163	1.096	1.112	0.919	0.283	0.393	0.343	25	25	25	100

Peptide Information

Calc. Mass	Obsrv. Mass	± da	± ppm	Start Seq.	End Seq.	Sequence	Ion Score	C. I. %	Modification	Plate [#] Name	Gel Idx/Pos [4700 Sample Name]	iTRAQ Ratio 115/114*	iTRAQ Ratio 116/114*	iTRAQ Ratio 117/114*	Rank	Result Type
1010.6971	1010.7395	0.0424	42	186	191	VILHLK	34	98.902	(N-term)_iTRAQ[0], Lysine(K)_iTRAQ[6]	[4] F7 and F10+11	1127/1119	0.913	0.913	1.330	1	Mascot
1236.651	1236.65	-0.001	-1	438	444	FYEQFSK	42	99.841	(N-term)_iTRAQ[0], Lysine(K)_iTRAQ[7]	[3] F6 and F9	240/232	1.217	1.053	1.472	1	Mascot
1379.7039	1379.6249	-0.079	-57	501	511	DQVANSAFVER	54	99.99	(N-term)_iTRAQ[0]	[1] F3 030912	179/171	1.256	1.013	0.687	1	Mascot
1408.7457	1408.8113	0.0656	47	347	356	RAPFDLFENR	30	97.68	(N-term)_iTRAQ[0]	[4] F7 and F10+11	1210/1202	1.108	1.087	1.163	1	Mascot
1439.7628	1439.7272	-0.0356	-25	285	293	YIDQEELNK	61	99.998	(N-term)_iTRAQ[0], Lysine(K)_iTRAQ[9]	[7] F5 120912	164/156	1.102	0.998	1.156	1	Mascot
1455.6724	1455.5995	-0.0729	-50	192	201	EDQTEYLEER	31	98.007	(N-term)_iTRAQ[0]	[5] F4	108/100	2.030	1.640	1.012	1	Mascot
1476.7073	1476.6628	-0.0445	-30	622	632	DNSTMGYMAAK	38	99.616	(N-term)_iTRAQ[0], Lysine(K)_iTRAQ[11]	[7] F5 120912	149/141	0.913	1.029	0.580	1	Mascot
1492.7668	1492.7969	0.0301	20	329	339	HFSVEGQLEFR	46	99.936	(N-term)_iTRAQ[0]	[3] F6 and F9	1244/1236	0.888	0.850	0.885	1	Mascot
1496.8359	1496.8894	0.0535	36	491	500	HIYFITGETK	80	100	(N-term)_iTRAQ[0], Lysine(K)_iTRAQ[10]	[3] F6 and F9	1211/1203	0.914	0.834	0.833	1	Mascot
1496.8359	1496.9026	0.0667	45	491	500	HIYFITGETK	71	100	(N-term)_iTRAQ[0], Lysine(K)_iTRAQ[10]	[4] F7 and F10+11	1144/1136	1.191	1.277	1.039	1	Mascot
1657.8881	1657.9039	0.0158	10	388	401	GVVDSEDLPLNISR	66	99.999	(N-term)_iTRAQ[0]	[8] F13-15 and F1+2	477/469	1.222	1.183	0.785	1	Mascot
1657.8881	1657.9056	0.0175	11	388	401	GVVDSEDLPLNISR	39	99.693	(N-term)_iTRAQ[0]	[8] F13-15 and F1+2	578/570	1.457	2.128	1.058	1	Mascot
1704.8425	1704.7418	-0.1007	-59	548	559	EGLELPEDEEEK	58	99.996	(N-term)_iTRAQ[0], Lysine(K)_iTRAQ[12]	[5] F4	137/129	0.828	1.143	0.648	1	Mascot
1838.9092	1838.7948	-0.1144	-62	466	479	YYTSASGDEMVSLK	68	100	(N-term)_iTRAQ[0], Lysine(K)_iTRAQ[14]	[5] F4	185/177	0.886	1.035	0.996	1	Mascot
1838.9092	1838.8215	-0.0877	-48	466	479	YYTSASGDEMVSLK	80	100	(N-term)_iTRAQ[0], Lysine(K)_iTRAQ[14]	[1] F3 030912	257/249	0.824	1.047	1.363	1	Mascot
1841.0028	1841.0774	0.0746	41	283	293	EKYIDQEELNK	46	99.932	(N-term)_iTRAQ[0], Lysine(K)_iTRAQ[2,11]	[4] F7 and F10+11	1077/1069	1.411	0.917	1.137	1	Mascot
1878.0807	1878.1661	0.0854	45	75	87	ELHINLIPNKQDR	72	100	(N-term)_iTRAQ[0], Lysine(K)_iTRAQ[10]	[4] F7 and F10+11	1159/1151	0.948	0.751	0.832	1	Mascot
1931.0471	1931.1305	0.0834	43	634	648	HLEINPDHSIIETLR	82	100	(N-term)_iTRAQ[0]	[4] F7 and F10+11	1210/1202	0.794	0.647	0.529	1	Mascot
1972.061	1971.9427	-0.1183	-60	61	74	YESLTDPSKLDSGK	50	99.977	(N-term)_iTRAQ[0], Lysine(K)_iTRAQ[9,14]	[6] F8 110912	224/216	1.290	1.198	1.409	1	Mascot
1977.0399	1976.955	-0.0849	-43	548	560	EGLELPEDEEEKK	72	100	(N-term)_iTRAQ[0], Lysine(K)_iTRAQ[12,13]	[6] F8 110912	205/197	0.978	1.051	0.552	1	Mascot
1977.0399	1977.1086	0.0687	35	548	560	EGLELPEDEEEKK	57	99.995	(N-term)_iTRAQ[0], Lysine(K)_iTRAQ[12,13]	[3] F6 and F9	1164/1156	1.567	2.875	0.617	1	Mascot
2067.1523	2067.1631	0.0108	5	210	224	HSQFIGYPITLFVEK	89	100	(N-term)_iTRAQ[0], Lysine(K)_iTRAQ[15]	[4] F7 and F10+11	454/446	1.159	0.951	0.905	1	Mascot
2121.9863	2121.8503	-0.136	-64	301	315	NPDDITNEEYGEFYK	48	99.959	(N-term)_iTRAQ[0], Lysine(K)_iTRAQ[15]	[5] F4	190/182	1.322	1.322	1.086	1	Mascot
2303.2493	2303.2961	0.0468	20	186	201	VILHLKEDQTEYLEER	40	99.751	(N-term)_iTRAQ[0], Lysine(K)_iTRAQ[6]	[4] F7 and F10+11	1203/1195	1.100	1.109	1.442	1	Mascot
2400.0613	2400.0706	0.0093	4	154	173	HNDDEQYAWESSAGGSFTVR	45	99.921	(N-term)_iTRAQ[0]	[4] F7 and F10+11	284/276	0.841	1.248	0.594	1	Mascot

21	Eno1 protein [Rattus norvegicus]	gi|38649320	56968.9	19	1149	1.030	1.115	1.076	0.592	0.605	0.530	25	25	25	100

Protein Group

Enolase 1, (alpha) [Rattus norvegicus]	gi|59808815	52736
alpha-enolase isoform 1 [Rattus norvegicus]	gi|158186649	52705.9
unnamed protein product [Rattus norvegicus]	gi|56107	52693.9

Peptide Information

Calc. Mass	Obsrv. Mass	± da	± ppm	Start Seq.	End Seq.	Sequence	Ion Score	C. I. %	Modification	Plate [#] Name	Gel Idx/Pos [4700 Sample Name]	iTRAQ Ratio 115/114*	iTRAQ Ratio 116/114*	iTRAQ Ratio 117/114*	Rank	Result Type
1088.5874	1088.5542	-0.0332	-30	295	300	YDLDFK	29	97.132	(N-term)_iTRAQ[0], Lysine(K)_iTRAQ[6]	[3] F6 and F9	256/248	1.076	0.966	1.088	1	Mascot
1098.6517	1098.6979	0.0462	42	103	109	AVEHINK	40	99.78	(N-term)_iTRAQ[0], Lysine(K)_iTRAQ[7]	[4] F7 and F10+11	989/981	0.558	1.078	0.387	1	Mascot
1103.6921	1103.6464	-0.0457	-41	260	266	EALELLK	34	99.074	(N-term)_iTRAQ[0], Lysine(K)_iTRAQ[7]	[7] F5 120912	241/233	0.768	0.856	0.785	1	Mascot

1187.761	1187.6798	-0.0812	-68	110	118	TIAPALVSK	51	99.98	(N-term)_iTRAQ[0], Lysine(K)_iTRAQ[9]	[5] F4	137/129	0.783	0.829	1.037	1	Mascot
1192.6671	1192.623	-0.0441	-37	451	458	IEEELGSK	38	99.63	(N-term)_iTRAQ[0], Lysine(K)_iTRAQ[8]	[7] F5 120912	152/144	0.568	0.716	0.891	1	Mascot
1192.6671	1192.6613	-0.0058	-5	451	458	IEEELGSK	58	99.996	(N-term)_iTRAQ[0], Lysine(K)_iTRAQ[8]	[3] F6 and F9	188/180	1.025	1.181	1.257	1	Mascot
1246.7252	1246.6826	-0.0426	-34	120	127	LNVVEQEK	47	99.949	(N-term)_iTRAQ[0], Lysine(K)_iTRAQ[8]	[7] F5 120912	150/142	0.989	1.010	1.059	1	Mascot
1431.8206	1431.8364	0.0158	11	222	231	IGAEVYHNLK	48	99.96	(N-term)_iTRAQ[0], Lysine(K)_iTRAQ[10]	[3] F6 and F9	1204/1196	0.733	0.691	0.649	1	Mascot
1431.8206	1431.8899	0.0693	48	222	231	IGAEVYHNLK	57	99.995	(N-term)_iTRAQ[0], Lysine(K)_iTRAQ[10]	[4] F7 and F10+11	1135/1127	0.746	0.850	0.866	1	Mascot
1488.8434	1488.896	0.0526	35	292	300	AGKYDLDFK	57	99.995	(N-term)_iTRAQ[0], Lysine(K)_iTRAQ[3,9]	[4] F7 and F10+11	1119/1111	1.350	1.899	1.149	1	Mascot
1518.9226	1518.9938	0.0712	47	119	127	KLNVVEQEK	62	99.999	(N-term)_iTRAQ[0], Lysine(K)_iTRAQ[1,9]	[4] F7 and F10+11	1056/1048	0.968	0.962	0.974	1	Mascot
1694.921	1694.8099	-0.1111	-66	54	66	GNPTVEVDLYTAK	80	100	(N-term)_iTRAQ[0], Lysine(K)_iTRAQ[13]	[5] F4	177/169	1.053	1.020	0.964	1	Mascot
1727.9465	1727.8671	-0.0794	-46	308	319	YITPDQLADLYK	47	99.949	(N-term)_iTRAQ[0], Lysine(K)_iTRAQ[12]	[5] F4	284/276	0.956	1.152	1.243	1	Mascot
1727.9465	1727.9161	-0.0304	-18	308	319	YITPDQLADLYK	52	99.985	(N-term)_iTRAQ[0], Lysine(K)_iTRAQ[12]	[1] F3 030912	373/365	0.720	0.676	0.735	1	Mascot
1796.9993	1796.9619	-0.0374	-21	144	158	FGANAILGVSLAVCK	56	99.994	(N-term)_iTRAQ[0], Lysine(K)_iTRAQ[15], MMTS (C)[14]	[2] F12 040912	523/515	3.175	3.392	3.289	1	Mascot
1796.9993	1796.9692	-0.0301	-17	144	158	FGANAILGVSLAVCK	90	100	(N-term)_iTRAQ[0], Lysine(K)_iTRAQ[15], MMTS (C)[14]	[1] F3 030912	517/509	0.897	1.051	0.925	1	Mascot
1796.9993	1796.983	-0.0163	-9	144	158	FGANAILGVSLAVCK	50	99.973	(N-term)_iTRAQ[0], Lysine(K)_iTRAQ[15], MMTS (C)[14]	[1] F3 030912	589/581	2.528	2.618	1.762	1	Mascot
1816.8964	1816.786	-0.1104	-61	295	307	YDLDFKSPDDASR	38	99.651	(N-term)_iTRAQ[0], Lysine(K)_iTRAQ[6]	[6] F8 110912	241/233	0.579	0.710	1.261	1	Mascot
1910.9906	1910.9333	-0.0573	-30	382	396	VNQIGSVTESLQACK	72	100	(N-term)_iTRAQ[0], Lysine(K)_iTRAQ[15], MMTS (C)[14]	[5] F4	258/250	1.070	1.182	1.188	1	Mascot
1924.9606	1924.8956	-0.065	-34	128	141	IDQLMIEMDGTENK	68	100	(N-term)_iTRAQ[0], Lysine(K)_iTRAQ[14]	[5] F4	258/250	1.370	1.162	1.290	1	Mascot
1949.0464	1949.0646	0.0182	9	71	88	AAVPSGASTGIYEALELR	43	99.878	(N-term)_iTRAQ[0]	[8] F13-15 and F1+2	397/389	1.044	1.100	0.973	1	Mascot
1949.0464	1949.1187	0.0723	37	71	88	AAVPSGASTGIYEALELR	47	99.954	(N-term)_iTRAQ[0]	[8] F13-15 and F1+2	1089/1081	3.036	3.154	2.483	1	Mascot
2041.0735	2041.0812	0.0077	4	201	217	LAMQEFMILPVGASSFR	56	99.993	(N-term)_iTRAQ[0]	[8] F13-15 and F1+2	281/273	0.734	1.084	1.111	1	Mascot
2249.1294	2249.0332	-0.0962	-43	241	259	DATNVGDEGGFAPNILEN K	105	100	(N-term)_iTRAQ[0], Lysine(K)_iTRAQ[19]	[1] F3 030912	294/286	1.218	0.726	1.071	1	Mascot
2856.325	2856.2114	-0.1136	-40	324	344	DYPVVSIEDPFDQDDWD AWQK	106	100	(N-term)_iTRAQ[0], Lysine(K)_iTRAQ[21]	[1] F3 030912	480/472	1.127	1.130	0.929	1	Mascot

22	neuron-specific class III beta-tubulin [Rattus norvegicus] gi|20799322	53072.7	16	1149	1.207	0.999	1.098	0.775	0.466	0.635	44	44	44	100

Peptide Information

Calc. Mass	Obsrv. Mass	± da	± ppm	Start Seq.	End Seq.	Sequence	Ion Score	C. I. %	Modification	Plate [#] Name	Gel Idx/Pos [4700 Sample Name]	iTRAQ Ratio 115/114*	iTRAQ Ratio 116/114*	iTRAQ Ratio 117/114*	Rank	Result Type
1198.4943	1198.4203	-0.074	-62	298	306	NMMAACDPR	42	99.828	(N-term)_iTRAQ[0], MMTS (C)[6]	[5] F4	175/167	1.069	1.113	1.167	1	Mascot
1213.7065	1213.6716	-0.0349	-29	310	318	YLTVATVFR	43	99.88	(N-term)_iTRAQ[0]	[1] F3 030912	357/349	0.906	0.822	1.113	1	Mascot
1287.7367	1287.6992	-0.0375	-29	253	262	LAVNMVPFPR	35	99.138	(N-term)_iTRAQ[0]	[1] F3 030912	349/341	0.961	0.841	0.953	1	Mascot
1287.7367	1287.7393	0.0026	2	253	262	LAVNMVPFPR	32	98.442	(N-term)_iTRAQ[0]	[1] F3 030912	716/708	0.788	0.478	0.836	1	Mascot
1559.9342	1559.8873	-0.0469	-30	252	262	KLAVNMVPFPR	33	98.897	(N-term)_iTRAQ[0], Lysine(K)_iTRAQ[1]	[6] F8 110912	367/359	0.926	0.879	0.841	1	Mascot
1559.9342	1560.0081	0.0739	47	252	262	KLAVNMVPFPR	45	99.924	(N-term)_iTRAQ[0], Lysine(K)_iTRAQ[1]	[3] F6 and F9	1277/1269	1.868	1.166	1.510	1	Mascot
1607.9077	1607.8265	-0.0812	-51	163	174	IMNTFSVVPSPK	64	99.999	(N-term)_iTRAQ[0], Lysine(K)_iTRAQ[12]	[5] F4	234/226	0.954	0.876	1.059	1	Mascot
1678.8932	1678.7491	-0.1441	-86	325	336	EVDEQMLAIQSK	27	95.599	(N-term)_iTRAQ[0], Lysine(K)_iTRAQ[12]	[5] F4	142/134	1.167	0.829	0.795	1	Mascot
1678.8932	1678.8002	-0.093	-55	325	336	EVDEQMLAIQSK	68	100	(N-term)_iTRAQ[0], Lysine(K)_iTRAQ[12]	[5] F4	167/159	1.156	1.179	1.147	1	Mascot
1685.8744	1685.7914	-0.083	-49	47	58	ISVYYNEASSHK	64	99.999	(N-term)_iTRAQ[0], Lysine(K)_iTRAQ[12]	[6] F8 110912	217/209	1.394	0.940	1.421	1	Mascot
1685.8744	1685.9323	0.0579	34	47	58	ISVYYNEASSHK	91	100	(N-term)_iTRAQ[0], Lysine(K)_iTRAQ[12]	[3] F6 and F9	1176/1168	1.408	1.099	1.223	1	Mascot
1748.943	1748.9092	-0.0338	-19	263	276	LHFFMPGFAPLTAR	55	99.993	(N-term)_iTRAQ[0]	[3] F6 and F9	468/460	0.837	0.961	1.127	1	Mascot
1759.9384	1759.9335	-0.0049	-3	63	77	AILVDLEPGTMDSVR	75	100	(N-term)_iTRAQ[0]	[8] F13-15 and F1+2	408/400	1.012	1.229	0.951	1	Mascot
1759.9384	1759.9364	-0.002	-1	63	77	AILVDLEPGTMDSVR	64	99.999	(N-term)_iTRAQ[0]	[8] F13-15 and F1+2	420/412	1.115	1.414	1.398	1	Mascot
1759.9384	1759.9373	-0.0011	-1	63	77	AILVDLEPGTMDSVR	52	99.984	(N-term)_iTRAQ[0]	[8] F13-15 and F1+2	461/453	1.241	1.771	1.343	1	Mascot
1759.9384	1759.9391	0.0007	0	63	77	AILVDLEPGTMDSVR	73	100	(N-term)_iTRAQ[0]	[8] F13-15 and F1+2	466/458	1.176	0.994	0.979	1	Mascot
1759.9384	1759.9392	0.0008	0	63	77	AILVDLEPGTMDSVR	30	97.57	(N-term)_iTRAQ[0]	[8] F13-15 and F1+2	516/508	1.051	1.080	0.935	1	Mascot

1759.9384	1759.946	0.0076	4	63	77	AILVDLEPGTMDSVR	93	100	(N-term)_iTRAQ[0]	[8] F13-15 and F1+2	412/404	1.164	1.244	1.086	1	Mascot
1759.9384	1759.9512	0.0128	7	63	77	AILVDLEPGTMDSVR	33	98.686	(N-term)_iTRAQ[0]	[8] F13-15 and F1+2	512/504	1.092	1.100	1.180	1	Mascot
1759.9384	1759.9528	0.0144	8	63	77	AILVDLEPGTMDSVR	68	100	(N-term)_iTRAQ[0]	[8] F13-15 and F1+2	404/396	0.658	0.704	0.767	1	Mascot
1759.9384	1759.954	0.0156	9	63	77	AILVDLEPGTMDSVR	66	99.999	(N-term)_iTRAQ[0]	[8] F13-15 and F1+2	417/409	0.952	1.015	0.884	1	Mascot
1759.9384	1759.9597	0.0213	12	63	77	AILVDLEPGTMDSVR	28	95.748	(N-term)_iTRAQ[0]	[8] F13-15 and F1+2	523/515	0.828	0.208	0.738	1	Mascot
1980.0721	1980.0026	-0.0695	-35	283	297	ALTVPELTQQMFDAK	72	100	(N-term)_iTRAQ[0], Lysine(K)_iTRAQ[15]	[1] F3 030912	419/411	1.201	0.990	0.970	1	Mascot
1980.0721	1980.0112	-0.0609	-31	283	297	ALTVPELTQQMFDAK	59	99.997	(N-term)_iTRAQ[0], Lysine(K)_iTRAQ[15]	[2] F12 040912	448/440	2.288	1.835	2.450	1	Mascot
1985.0378	1984.9299	-0.1079	-54	337	350	NSSYFVEWIPNNVK	39	99.708	(N-term)_iTRAQ[0], Lysine(K)_iTRAQ[14]	[5] F4	298/290	1.071	1.010	1.098	1	Mascot
1985.0378	1984.957	-0.0808	-41	337	350	NSSYFVEWIPNNVK	43	99.868	(N-term)_iTRAQ[0], Lysine(K)_iTRAQ[14]	[6] F8 110912	369/361	0.630	0.821	0.824	1	Mascot
1985.0378	1984.9778	-0.06	-30	337	350	NSSYFVEWIPNNVK	32	98.529	(N-term)_iTRAQ[0], Lysine(K)_iTRAQ[14]	[6] F8 110912	363/355	1.422	0.707	0.524	1	Mascot
1985.0378	1984.988	-0.0498	-25	337	350	NSSYFVEWIPNNVK	30	97.598	(N-term)_iTRAQ[0], Lysine(K)_iTRAQ[14]	[5] F4	339/331	1.470	1.187	0.924	1	Mascot
2100.092	2099.9648	-0.1272	-61	3	19	EIVHIQAGQCGNQIGAK	66	99.999	(N-term)_iTRAQ[0], Lysine(K)_iTRAQ[17], MMTS (C)[10]	[6] F8 110912	261/253	0.727	0.775	0.614	1	Mascot
2100.092	2100.0605	-0.0315	-15	3	19	EIVHIQAGQCGNQIGAK	109	100	(N-term)_iTRAQ[0], Lysine(K)_iTRAQ[17], MMTS (C)[10]	[4] F7 and F10+11	265/257	1.064	0.910	1.094	1	Mascot
2100.092	2100.1162	0.0242	12	3	19	EIVHIQAGQCGNQIGAK	89	100	(N-term)_iTRAQ[0], Lysine(K)_iTRAQ[17], MMTS (C)[10]	[4] F7 and F10+11	379/371	1.194	0.967	0.685	1	Mascot
2100.092	2100.1257	0.0337	16	3	19	EIVHIQAGQCGNQIGAK	41	99.786	(N-term)_iTRAQ[0], Lysine(K)_iTRAQ[17], MMTS (C)[10]	[4] F7 and F10+11	631/623	1.893	1.942	1.642	1	Mascot
2100.092	2100.1853	0.0933	44	3	19	EIVHIQAGQCGNQIGAK	37	99.482	(N-term)_iTRAQ[0], Lysine(K)_iTRAQ[17], MMTS (C)[10]	[4] F7 and F10+11	558/550	1.799	1.038	2.313	1	Mascot
2103.0842	2103.0032	-0.081	-39	104	121	GHYTEGAELVDSVLDVV R	37	99.506	(N-term)_iTRAQ[0]	[6] F8 110912	488/480	1.671	0.987	0.997	1	Mascot
2103.0842	2103.0552	-0.029	-14	104	121	GHYTEGAELVDSVLDVV R	37	99.468	(N-term)_iTRAQ[0]	[3] F6 and F9	722/714	3.032	1.634	1.575	1	Mascot
2103.0842	2103.0581	-0.0261	-12	104	121	GHYTEGAELVDSVLDVV R	53	99.987	(N-term)_iTRAQ[0]	[2] F12 040912	496/488	1.368	1.498	3.600	1	Mascot
2103.0842	2103.0779	-0.0063	-3	104	121	GHYTEGAELVDSVLDVV R	54	99.991	(N-term)_iTRAQ[0]	[7] F5 120912	465/457	0.957	0.667	1.064	1	Mascot
2103.0842	2103.0789	-0.0053	-3	104	121	GHYTEGAELVDSVLDVV R	48	99.96	(N-term)_iTRAQ[0]	[3] F6 and F9	481/473	1.228	1.113	1.179	1	Mascot
2103.0842	2103.1221	0.0379	18	104	121	GHYTEGAELVDSVLDVV R	111	100	(N-term)_iTRAQ[0]	[4] F7 and F10+11	489/481	1.151	0.950	0.918	1	Mascot
2103.0842	2103.1589	0.0747	36	104	121	GHYTEGAELVDSVLDVV R	119	100	(N-term)_iTRAQ[0]	[4] F7 and F10+11	1342/1334	0.615	0.767	0.660	1	Mascot
2103.0842	2103.1692	0.085	40	104	121	GHYTEGAELVDSVLDVV R	36	99.343	(N-term)_iTRAQ[0]	[4] F7 and F10+11	1318/1310	14.597	2.288	6.391	1	Mascot
2162.1414	2162.0635	-0.0779	-36	363	379	MSSTFIGNSTAIQELFK	31	97.884	(N-term)_iTRAQ[0], Lysine(K)_iTRAQ[17]	[5] F4	363/355	0.867	0.713	0.650	1	Mascot
2749.4314	2749.353	-0.0784	-29	217	241	LATPTYGDLNHLVSATMS GVTTSLR	153	100	(N-term)_iTRAQ[0]	[1] F3 030912	508/500	1.218	1.146	1.230	1	Mascot
3221.5146	3221.3904	-0.1242	-39	20	46	FWEVISDEHGIDPSGNYV GDSDLQLER	87	100	(N-term)_iTRAQ[0]	[1] F3 030912	430/422	1.206	1.038	0.596	1	Mascot

23 syntaxin-binding protein 1 [Rattus norvegicus] gi|6981602 75485.2 18 1125 1.073 1.081 1.037 0.345 0.337 0.384 19 19 19 100

Protein Group

syntaxin-binding protein 1 isoform a [Homo sapiens] gi|4507297 76219.3

Peptide Information

Calc. Mass	Obsrv. Mass	± da	± ppm	Start Seq.	End Seq.	Sequence	Ion Score	C. I. %	Modification	Plate [#] Name	Gel Idx/Pos [4700 Sample Name]	iTRAQ Ratio 115/114*	iTRAQ Ratio 116/114*	iTRAQ Ratio 117/114*	Rank	Result Type
1101.6752	1101.6143	-0.0609	-55	228	235	SQLLILDR	27	95.538	(N-term)_iTRAQ[0]	[5] F4	200/192	2.086	2.231	2.388	1	Mascot
1143.6805	1143.7294	0.0489	43	14	20	IMHDVIK	36	99.364	(N-term)_iTRAQ[0], Lysine(K)_iTRAQ[7]	[4] F7 and F10+11	1096/1088	1.826	1.436	1.482	1	Mascot
1303.7528	1303.7002	-0.0526	-40	30	39	VLVVDQLSMR	60	99.998	(N-term)_iTRAQ[0]	[1] F3 030912	308/300	0.941	1.232	0.838	1	Mascot
1368.7244	1368.6335	-0.0909	-66	468	477	ISEQTYQLSR	52	99.984	(N-term)_iTRAQ[0]	[5] F4	124/116	1.129	1.012	1.169	1	Mascot
1412.7618	1412.6857	-0.0761	-54	295	305	HIAEVSQEVTR	47	99.947	(N-term)_iTRAQ[0]	[6] F8 110912	164/156	1.056	0.982	0.994	1	Mascot
1415.7251	1415.6484	-0.0767	-54	506	518	SSASFSTTAVSAR	49	99.97	(N-term)_iTRAQ[0]	[1] F3 030912	164/156	0.898	0.930	0.870	1	Mascot
1520.7148	1520.6622	-0.0526	-35	214	225	ADDPTMGEGPDK	28	95.835	(N-term)_iTRAQ[0], Lysine(K)_iTRAQ[12]	[7] F5 120912	87/79	0.652	0.857	0.471	1	Mascot
1629.9421	1629.8933	-0.0488	-30	197	208	DNALLAQLIQDK	69	100	(N-term)_iTRAQ[0], Lysine(K)_iTRAQ[12]	[5] F4	307/299	0.825	0.947	0.801	1	Mascot
1708.9568	1708.9242	-0.0326	-19	172	184	LAEQIATLCATLK	101	100	(N-term)_iTRAQ[0], Lysine(K)_iTRAQ[13], MMTS (C)[9]	[5] F4	422/414	0.929	0.803	1.172	1	Mascot

1880.9156	1880.7946	-0.121	-64	368	382	VEQDLAMGTDAEGEK	115	100	(N-term)_iTRAQ[0], Lysine(K)_iTRAQ[15]	[5] F4	140/132	1.067	1.176	1.182	1	Mascot
2020.1576	2020.0676	-0.09	-45	389	404	AIVPILLDANVSTYDK	91	100	(N-term)_iTRAQ[0], Lysine(K)_iTRAQ[16]	[1] F3 030912	418/410	0.737	1.125	0.817	1	Mascot
2098.0962	2098.0706	-0.0256	-12	484	497	DIMEDTIEDKLDTK	105	100	(N-term)_iTRAQ[0], Lysine(K)_iTRAQ[10, 14]	[4] F7 and F10+11	341/333	0.948	0.697	0.864	1	Mascot
2174.2192	2174.2617	0.0425	20	83	98	SVHSLISDFKDPPTAK	71	100	(N-term)_iTRAQ[0], Lysine(K)_iTRAQ[10, 16]	[4] F7 and F10+11	1203/1195	0.877	0.913	0.989	1	Mascot
2211.1499	2211.1074	-0.0425	-19	47	63	MTDIMTEGITIVEDINK	70	100	(N-term)_iTRAQ[0], Lysine(K)_iTRAQ[17]	[1] F3 030912	534/526	1.341	1.427	1.086	1	Mascot
2251.2668	2251.1565	-0.1103	-49	193	208	GEYKDNALLAQLIQDK	96	100	(N-term)_iTRAQ[0], Lysine(K)_iTRAQ[4,16]	[6] F8 110912	402/394	1.220	1.072	1.078	1	Mascot
2330.2854	2330.3098	0.0244	10	276	292	VKEVLLDEDDDLWIALR	32	98.579	(N-term)_iTRAQ[0], Lysine(K)_iTRAQ[2]	[4] F7 and F10+11	489/481	1.070	1.237	0.830	1	Mascot
2364.3511	2364.3943	0.0432	18	197	213	DNALLAQLIQDKLDAYK	44	99.899	(N-term)_iTRAQ[0], Lysine(K)_iTRAQ[12, 17]	[4] F7 and F10+11	504/496	0.996	0.702	1.309	1	Mascot
2367.251	2367.2935	0.0425	18	47	64	MTDIMTEGITIVEDINKR	35	99.293	(N-term)_iTRAQ[0], Lysine(K)_iTRAQ[17]	[4] F7 and F10+11	532/524	1.273	1.389	1.229	1	Mascot
2367.251	2367.3813	0.1303	55	47	64	MTDIMTEGITIVEDINKR	32	98.519	(N-term)_iTRAQ[0], Lysine(K)_iTRAQ[17]	[4] F7 and F10+11	1362/1354	1.374	1.182	1.167	1	Mascot

24	Heat shock protein 90kDa alpha (cytosolic), class B member 1 [Rattus norvegicus]	gi|51859516	94516.9	22	1109	1.155	1.162	0.924	0.585	0.805	0.501	25	25	25	100

Protein Group

heat shock protein HSP 90-beta [Mus musculus] gi|40556608 94456.8

Peptide Information

Calc. Mass	Obsrv. Mass	± da	± ppm	Start Seq.	End Seq.	Sequence	Ion Score	C. I. %	Modification	Plate [#] Name	Gel Idx/Pos [4700 Sample Name]	iTRAQ Ratio 115/114*	iTRAQ Ratio 116/114*	iTRAQ Ratio 117/114*	Rank	Result Type
973.6318	973.6001	-0.0317	-33	331	337	ALLFIPR	31	98.161	(N-term)_iTRAQ[0]	[5] F4	257/249	4.647	4.618	2.531	1	Mascot
1010.6971	1010.7395	0.0424	42	181	186	VILHLK	34	98.902	(N-term)_iTRAQ[0], Lysine(K)_iTRAQ[6]	[4] F7 and F10+11	1127/1119	0.913	0.913	1.330	1	Mascot
1179.6295	1179.5936	-0.0359	-30	429	435	FYEAFSK	34	99.093	(N-term)_iTRAQ[0], Lysine(K)_iTRAQ[7]	[3] F6 and F9	266/258	0.873	0.923	1.033	1	Mascot
1338.7501	1338.6855	-0.0646	-48	73	82	IDIIPNPQER	34	98.973	(N-term)_iTRAQ[0]	[1] F3 030912	252/244	0.892	0.988	0.713	1	Mascot
1393.7196	1393.6388	-0.0808	-58	492	502	EQVANSAFVER	74	100	(N-term)_iTRAQ[0]	[1] F3 030912	179/171	0.929	0.807	0.661	1	Mascot
1439.7628	1439.7272	-0.0356	-25	276	284	YIDQEELNK	61	99.998	(N-term)_iTRAQ[0], Lysine(K)_iTRAQ[9]	[7] F5 120912	164/156	1.102	0.998	1.156	1	Mascot
1455.6724	1455.5995	-0.0729	-50	187	196	EDQTEYLEER	31	98.007	(N-term)_iTRAQ[0]	[5] F4	108/100	2.030	1.640	1.012	1	Mascot
1492.7668	1492.7969	0.0301	20	320	330	HFSVEGQLEFR	46	99.936	(N-term)_iTRAQ[0]	[3] F6 and F9	1244/1236	0.888	0.850	0.885	1	Mascot
1524.842	1524.9113	0.0693	45	338	347	RAPFDLFENK	40	99.765	(N-term)_iTRAQ[0], Lysine(K)_iTRAQ[10]	[4] F7 and F10+11	1207/1199	0.986	1.113	1.124	1	Mascot
1536.7106	1536.6569	-0.0537	-35	613	623	DNSTMGYMMAK	42	99.837	(N-term)_iTRAQ[0], Lysine(K)_iTRAQ[11]	[7] F5 120912	180/172	2.347	2.985	2.105	1	Mascot
1563.8475	1563.792	-0.0555	-35	42	53	ELISNASDALDK	49	99.97	(N-term)_iTRAQ[0], Lysine(K)_iTRAQ[12]	[5] F4	164/156	0.906	0.999	0.864	1	Mascot
1657.8881	1657.9039	0.0158	10	379	392	GVVDSEDLPLNISR	66	99.999	(N-term)_iTRAQ[0]	[8] F13-15 and F1+2	477/469	1.222	1.183	0.785	1	Mascot
1657.8881	1657.9056	0.0175	11	379	392	GVVDSEDLPLNISR	39	99.693	(N-term)_iTRAQ[0]	[8] F13-15 and F1+2	578/570	1.457	2.128	1.058	1	Mascot
1704.8425	1704.7418	-0.1007	-59	539	550	EGLELPEDEEEK	58	99.996	(N-term)_iTRAQ[0], Lysine(K)_iTRAQ[12]	[5] F4	137/129	0.828	1.143	0.648	1	Mascot
1841.0028	1841.0774	0.0746	41	274	284	EKYIDQEELNK	46	99.932	(N-term)_iTRAQ[0], Lysine(K)_iTRAQ[2,11]	[4] F7 and F10+11	1077/1069	1.411	0.917	1.137	1	Mascot
1927.0521	1927.0808	0.0287	15	625	639	HLEINPDHPIVETLR	47	99.951	(N-term)_iTRAQ[0]	[3] F6 and F9	1242/1234	0.949	0.920	0.917	1	Mascot
1972.061	1971.9427	-0.1183	-60	56	69	YESLTDPSKLDSGK	50	99.977	(N-term)_iTRAQ[0], Lysine(K)_iTRAQ[9,14]	[6] F8 110912	224/216	1.290	1.198	1.409	1	Mascot
1977.0399	1976.955	-0.0849	-43	539	551	EGLELPEDEEEKK	72	100	(N-term)_iTRAQ[0], Lysine(K)_iTRAQ[12, 13]	[6] F8 110912	205/197	0.978	1.051	0.552	1	Mascot
1977.0399	1977.1086	0.0687	35	539	551	EGLELPEDEEEKK	57	99.995	(N-term)_iTRAQ[0], Lysine(K)_iTRAQ[12, 13]	[3] F6 and F9	1164/1156	1.567	2.875	0.617	1	Mascot
2097.1631	2097.1604	-0.0027	-1	205	219	HSQFIGYPITLYLEK	88	100	(N-term)_iTRAQ[0], Lysine(K)_iTRAQ[15]	[4] F7 and F10+11	440/432	0.944	1.007	0.786	1	Mascot
2136.002	2135.8914	-0.1106	-52	292	306	NPDDITQEEYGEFYK	39	99.659	(N-term)_iTRAQ[0], Lysine(K)_iTRAQ[15]	[5] F4	197/189	1.095	1.191	1.141	1	Mascot
2136.002	2135.9236	-0.0784	-37	292	306	NPDDITQEEYGEFYK	53	99.987	(N-term)_iTRAQ[0], Lysine(K)_iTRAQ[15]	[1] F3 030912	271/263	0.572	0.277	0.324	1	Mascot
2303.2493	2303.2961	0.0468	20	181	196	VILHLKEDQTEYLEER	40	99.751	(N-term)_iTRAQ[0], Lysine(K)_iTRAQ[6]	[4] F7 and F10+11	1203/1195	1.100	1.109	1.442	1	Mascot
2400.0613	2400.0706	0.0093	4	149	168	HNDDEQYAWESSAGGS FTVR	45	99.921	(N-term)_iTRAQ[0]	[4] F7 and F10+11	284/276	0.841	1.248	0.594	1	Mascot

Calc. Mass	Obsrv. Mass	± da	± ppm	Start Seq.	End Seq.	Sequence	Ion Score	C. I. %	Modification	Plate [#] Name	Gel Idx/Pos [4700 Sample Name]	iTRAQ Ratio 115/114*	iTRAQ Ratio 116/114*	iTRAQ Ratio 117/114*	Rank	Result Type
2725.3154	2725.2676	-0.0478	-18	507	526	GFEVVYMTEPIDEYCVQ QLK	70	100	(N-term)_iTRAQ[0], Lysine(K)_iTRAQ[20], MMTS (C)[15]	[1] F3 030912	529/521	1.338	0.734	0.602	1	Mascot

25	ATP synthase subunit beta, mitochondrial precursor [Rattus norvegicus]	gi|54792127	59776.9	18	1100	0.928	0.986	1.056	0.491	0.214	0.456	25	25	25	100

Protein Group

ATP synthase beta subunit [Rattus norvegicus]	gi|1374715	54485
Chain B, Rat Liver F1-Atpase	gi|6729935	54779.2

Peptide Information

Calc. Mass	Obsrv. Mass	± da	± ppm	Start Seq.	End Seq.	Sequence	Ion Score	C. I. %	Modification	Plate [#] Name	Gel Idx/Pos [4700 Sample Name]	iTRAQ Ratio 115/114*	iTRAQ Ratio 116/114*	iTRAQ Ratio 117/114*	Rank	Result Type
1067.6346	1067.5879	-0.0467	-44	427	432	ILQDYK	45	99.929	(N-term)_iTRAQ[0], Lysine(K)_iTRAQ[6]	[3] F6 and F9	207/199	1.033	1.089	1.288	1	Mascot
1182.6967	1182.6327	-0.064	-54	134	143	IPVGPETLGR	34	99.101	(N-term)_iTRAQ[0]	[1] F3 030912	235/227	0.980	1.307	1.098	1	Mascot
1263.7671	1263.7345	-0.0326	-26	202	212	IGLFGGAGVGK	100	100	(N-term)_iTRAQ[0], Lysine(K)_iTRAQ[11]	[7] F5 120912	255/247	0.879	1.092	0.852	1	Mascot
1376.84	1376.7938	-0.0462	-34	189	198	VVDLLAPYAK	55	99.992	(N-term)_iTRAQ[0], Lysine(K)_iTRAQ[10]	[5] F4	263/255	1.047	1.279	1.534	1	Mascot
1406.7434	1406.6686	-0.0748	-53	110	121	TIAMDGTEGLVR	51	99.982	(N-term)_iTRAQ[0]	[1] F3 030912	240/232	0.779	0.845	0.918	1	Mascot
1529.8118	1529.7588	-0.053	-35	144	155	IMNVIGEPIDER	49	99.967	(N-term)_iTRAQ[0]	[1] F3 030912	310/302	0.901	0.976	1.228	1	Mascot
1550.7836	1550.6982	-0.0854	-55	226	239	AHGGYSVFAGVGER	69	100	(N-term)_iTRAQ[0]	[6] F8 110912	245/237	0.557	0.727	0.820	1	Mascot
1550.7836	1550.8077	0.0241	16	226	239	AHGGYSVFAGVGER	64	99.999	(N-term)_iTRAQ[0]	[3] F6 and F9	1201/1193	0.845	1.286	1.298	1	Mascot
1550.7836	1550.8883	0.1047	68	226	239	AHGGYSVFAGVGER	37	99.488	(N-term)_iTRAQ[0]	[8] F13-15 and F1+2	1526/1518	1.855	0.744	0.664	1	Mascot
1579.8563	1579.8696	0.0133	8	311	324	FTQAGSEVSALLGR	74	100	(N-term)_iTRAQ[0]	[8] F13-15 and F1+2	404/396	0.851	0.989	0.926	1	Mascot
1579.8563	1579.8706	0.0143	9	311	324	FTQAGSEVSALLGR	58	99.996	(N-term)_iTRAQ[0]	[8] F13-15 and F1+2	507/499	1.125	1.049	1.198	1	Mascot
1579.8563	1579.8755	0.0192	12	311	324	FTQAGSEVSALLGR	36	99.326	(N-term)_iTRAQ[0]	[8] F13-15 and F1+2	561/553	0.422	0.755	0.861	1	Mascot
1583.8917	1583.9119	0.0202	13	282	294	VALTGLTVAEYFR	60	99.998	(N-term)_iTRAQ[0]	[8] F13-15 and F1+2	378/370	0.775	1.028	1.054	1	Mascot
1745.9128	1745.8387	-0.0741	-42	265	279	VALVYGQMNEPPGAR	50	99.978	(N-term)_iTRAQ[0]	[1] F3 030912	259/251	1.955	0.733	1.491	1	Mascot
1745.9128	1745.9324	0.0196	11	265	279	VALVYGQMNEPPGAR	53	99.988	(N-term)_iTRAQ[0]	[8] F13-15 and F1+2	507/499	0.872	1.045	1.010	1	Mascot
1746.0444	1745.9807	-0.0637	-36	213	225	TVLIMELINNVAK	70	100	(N-term)_iTRAQ[0], Lysine(K)_iTRAQ[13]	[5] F4	405/397	1.170	1.049	2.299	1	Mascot
1746.0444	1745.9817	-0.0627	-36	213	225	TVLIMELINNVAK	72	100	(N-term)_iTRAQ[0], Lysine(K)_iTRAQ[13]	[1] F3 030912	527/519	0.775	1.112	1.234	1	Mascot
1795.0198	1794.9919	-0.0279	-16	95	109	LVLEVAQHLGESTVR	45	99.93	(N-term)_iTRAQ[0]	[7] F5 120912	382/374	2.156	1.353	2.087	1	Mascot
1959.9718	1959.9629	-0.0089	-5	407	422	IMDPNIVGSEHYDVAR	69	100	(N-term)_iTRAQ[0]	[3] F6 and F9	283/275	0.963	0.919	0.951	1	Mascot
2066.0679	2066.0156	-0.0523	-25	295	310	DQEGQDVLLFIDNIFR	89	100	(N-term)_iTRAQ[0]	[1] F3 030912	577/569	0.957	1.081	1.001	1	Mascot
2349.2007	2349.2263	0.0256	11	242	259	EGNDLYHEMIESGVINLK	75	100	(N-term)_iTRAQ[0], Lysine(K)_iTRAQ[18]	[4] F7 and F10+11	414/406	1.244	1.327	0.972	1	Mascot
2349.2007	2349.2673	0.0666	28	242	259	EGNDLYHEMIESGVINLK	88	100	(N-term)_iTRAQ[0], Lysine(K)_iTRAQ[18]	[4] F7 and F10+11	1284/1276	0.832	0.751	0.564	1	Mascot
2407.2524	2407.1753	-0.0771	-32	433	451	SLQDIIAILGMDELSEEDK	59	99.997	(N-term)_iTRAQ[0], Lysine(K)_iTRAQ[19]	[1] F3 030912	550/542	0.270	0.818	0.918	1	Mascot
2606.3494	2606.4473	0.0979	38	240	259	TREGNDLYHEMIESGVIN LK	38	99.596	(N-term)_iTRAQ[0], Lysine(K)_iTRAQ[20]	[4] F7 and F10+11	1266/1258	1.010	0.944	1.441	1	Mascot
3624.8293	3624.7119	-0.1174	-32	490	519	GFQQILAGDYDHLPEQAF YMVGPIEEAVAK	52	99.985	(N-term)_iTRAQ[0], Lysine(K)_iTRAQ[30]	[1] F3 030912	539/531	1.003	0.833	0.429	1	Mascot

26	tubulin alpha-1B chain [Mus musculus]	gi|34740335	53553.5	19	1092	1.202	1.171	1.117	0.442	0.403	0.582	36	36	36	100

Peptide Information

Calc. Mass	Obsrv. Mass	± da	± ppm	Start Seq.	End Seq.	Sequence	Ion Score	C. I. %	Modification	Plate [#] Name	Gel Idx/Pos [4700 Sample Name]	iTRAQ Ratio 115/114*	iTRAQ Ratio 116/114*	iTRAQ Ratio 117/114*	Rank	Result Type
1063.6146	1063.6521	0.0375	35	106	112	GHYTIGK	40	99.748	(N-term)_iTRAQ[0], Lysine(K)_iTRAQ[7]	[4] F7 and F10+11	1016/1008	0.862	0.680	0.880	1	Mascot
1069.6139	1069.5664	-0.0475	-44	157	163	LSVDYGK	50	99.977	(N-term)_iTRAQ[0], Lysine(K)_iTRAQ[7]	[3] F6 and F9	186/178	1.105	1.103	0.965	1	Mascot
1069.6139	1069.588	-0.0259	-24	157	163	LSVDYGK	35	99.254	(N-term)_iTRAQ[0], Lysine(K)_iTRAQ[7]	[4] F7 and F10+11	184/176	2.054	1.109	1.285	1	Mascot
1175.6381	1175.603	-0.0351	-30	395	401	FDLMYAK	41	99.785	(N-term)_iTRAQ[0], Lysine(K)_iTRAQ[7]	[7] F5 120912	255/247	1.048	1.116	0.986	1	Mascot
1229.7225	1229.6846	-0.0379	-31	113	121	EIIDLVLDR	35	99.261	(N-term)_iTRAQ[0]	[1] F3 030912	412/404	0.821	1.183	1.148	1	Mascot
1303.7831	1303.7196	-0.0635	-49	327	336	DVNAAIATIK	72	100	(N-term)_iTRAQ[0], Lysine(K)_iTRAQ[10]	[5] F4	160/152	1.082	1.109	1.010	1	Mascot
1303.7831	1303.7577	-0.0254	-19	327	336	DVNAAIATIK	49	99.971	(N-term)_iTRAQ[0], Lysine(K)_iTRAQ[10]	[5] F4	597/589	1.385	1.345	1.031	1	Mascot
1341.8113	1341.8744	0.0631	47	157	164	LSVDYGKK	47	99.946	(N-term)_iTRAQ[0], Lysine(K)_iTRAQ[7,8]	[4] F7 and F10+11	1058/1050	0.812	0.883	1.166	1	Mascot

1371.5837	1371.552	-0.0317	-23	312	320	YMACCLLYR	32	98.334	(N-term)_iTRAQ[0], MMTS (C)[4,5]	[1] F3 030912	501/493	1.766	1.780	1.455	1	Mascot
1631.9816	1631.9253	-0.0563	-34	230	243	LISQIVSSITASLR	73	100	(N-term)_iTRAQ[0]	[1] F3 030912	580/572	1.272	1.138	1.282	1	Mascot
1631.9816	1631.9677	-0.0139	-9	230	243	LISQIVSSITASLR	52	99.985	(N-term)_iTRAQ[0]	[8] F13-15 and F1+2	211/203	1.352	1.231	1.211	1	Mascot
1698.9789	1698.9282	-0.0507	-30	85	96	QLFHPEQLITGK	73	100	(N-term)_iTRAQ[0], Lysine(K)_iTRAQ[12]	[6] F8 110912	370/362	1.300	1.240	0.957	1	Mascot
1698.9789	1698.9305	-0.0484	-28	85	96	QLFHPEQLITGK	38	99.641	(N-term)_iTRAQ[0], Lysine(K)_iTRAQ[12]	[7] F5 120912	275/267	1.165	1.747	1.318	1	Mascot
1698.9789	1698.9484	-0.0305	-18	85	96	QLFHPEQLITGK	48	99.962	(N-term)_iTRAQ[0], Lysine(K)_iTRAQ[12]	[6] F8 110912	587/579	2.345	2.048	3.682	1	Mascot
1698.9789	1698.9783	-0.0006	0	85	96	QLFHPEQLITGK	81	100	(N-term)_iTRAQ[0], Lysine(K)_iTRAQ[12]	[4] F7 and F10+11	311/303	0.826	0.760	0.890	1	Mascot
1698.9789	1699.0499	0.071	42	85	96	QLFHPEQLITGK	32	98.505	(N-term)_iTRAQ[0], Lysine(K)_iTRAQ[12]	[3] F6 and F9	1265/1257	1.091	1.050	1.033	1	Mascot
1813.0054	1812.9636	-0.0418	-23	391	401	LDHKFDLMYAK	38	99.609	(N-term)_iTRAQ[0], Lysine(K)_iTRAQ[4,11]	[2] F12 040912	317/309	0.885	0.665	1.291	1	Mascot
1829.0002	1828.9752	-0.025	-14	391	401	LDHKFDLMYAK	38	99.613	(N-term)_iTRAQ[0], Lysine(K)_iTRAQ[4,11], Oxidation (M)[8]	[2] F12 040912	276/268	0.839	0.682	1.068	1	Mascot
1846.0082	1845.9462	-0.062	-34	65	79	AVFVDLEPTVIDEVR	63	99.999	(N-term)_iTRAQ[0]	[1] F3 030912	427/419	0.819	1.071	1.252	1	Mascot
1846.0082	1845.97	-0.0382	-21	65	79	AVFVDLEPTVIDEVR	62	99.998	(N-term)_iTRAQ[0]	[8] F13-15 and F1+2	216/208	1.793	1.969	1.936	1	Mascot
1846.0082	1846.0332	0.025	14	65	79	AVFVDLEPTVIDEVR	49	99.967	(N-term)_iTRAQ[0]	[8] F13-15 and F1+2	386/378	1.231	1.311	1.173	1	Mascot
1861.9208	1861.8561	-0.0647	-35	340	352	SIQFVDWCPTGFK	31	97.854	(N-term)_iTRAQ[0], Lysine(K)_iTRAQ[13], MMTS (C)[8]	[6] F8 110912	488/480	0.875	1.523	0.343	1	Mascot
1861.9208	1861.8618	-0.059	-32	340	352	SIQFVDWCPTGFK	32	98.315	(N-term)_iTRAQ[0], Lysine(K)_iTRAQ[13], MMTS (C)[8]	[1] F3 030912	478/470	2.663	0.986	2.990	1	Mascot
1861.9208	1861.8782	-0.0426	-23	340	352	SIQFVDWCPTGFK	58	99.996	(N-term)_iTRAQ[0], Lysine(K)_iTRAQ[13], MMTS (C)[8]	[5] F4	373/365	1.178	1.009	1.062	1	Mascot
1862.9845	1862.9556	-0.0289	-16	216	229	NLDIERPTYTNLNR	30	97.732	(N-term)_iTRAQ[0]	[4] F7 and F10+11	238/230	1.553	1.246	1.030	1	Mascot
1997.9712	1997.916	-0.0552	-28	374	390	AVCMLSNTTAIAEAWAR	29	96.623	(N-term)_iTRAQ[0], MMTS (C)[3]	[1] F3 030912	492/484	2.182	2.439	1.956	1	Mascot
1997.9712	1997.9668	-0.0044	-2	374	390	AVCMLSNTTAIAEAWAR	38	99.619	(N-term)_iTRAQ[0], MMTS (C)[3]	[8] F13-15 and F1+2	310/302	1.165	1.072	0.786	1	Mascot
1997.9712	1997.9895	0.0183	9	374	390	AVCMLSNTTAIAEAWAR	39	99.666	(N-term)_iTRAQ[0], MMTS (C)[3]	[4] F7 and F10+11	495/487	1.117	0.977	0.445	1	Mascot
1997.9712	1998.0536	0.0824	41	374	390	AVCMLSNTTAIAEAWAR	53	99.986	(N-term)_iTRAQ[0], MMTS (C)[3]	[4] F7 and F10+11	1343/1335	1.505	1.793	0.689	1	Mascot
2045.1681	2045.1163	-0.0518	-25	265	280	IHFPLATYAPVISAEK	33	98.745	(N-term)_iTRAQ[0], Lysine(K)_iTRAQ[16]	[3] F6 and F9	399/391	0.894	1.154	1.043	1	Mascot
2045.1681	2045.1881	0.02	10	265	280	IHFPLATYAPVISAEK	66	99.999	(N-term)_iTRAQ[0], Lysine(K)_iTRAQ[16]	[4] F7 and F10+11	414/406	0.821	1.282	1.061	1	Mascot
2113.1902	2113.1169	-0.0733	-35	353	370	VGINYQPPTVVPGGDLAK	107	100	(N-term)_iTRAQ[0], Lysine(K)_iTRAQ[18]	[1] F3 030912	309/301	1.136	1.140	0.923	1	Mascot
2296.0979	2296.031	-0.0669	-29	41	60	TIGGGDDSFNTFFSETGAGK	123	100	(N-term)_iTRAQ[0], Lysine(K)_iTRAQ[20]	[1] F3 030912	351/343	1.229	1.113	1.269	1	Mascot
2474.1206	2473.991	-0.1296	-52	403	422	AFVHWYVGEGMEEGEFSEAR	44	99.905	(N-term)_iTRAQ[0]	[6] F8 110912	399/391	1.267	1.191	1.075	1	Mascot
2474.1206	2474.1038	-0.0168	-7	403	422	AFVHWYVGEGMEEGEFSEAR	54	99.989	(N-term)_iTRAQ[0]	[4] F7 and F10+11	397/389	1.024	1.067	0.778	1	Mascot
2703.4099	2703.511	0.1011	37	85	105	QLFHPEQLITGKEDAANNYAR	31	97.893	(N-term)_iTRAQ[0], Lysine(K)_iTRAQ[12]	[4] F7 and F10+11	1207/1199	1.179	0.877	1.646	1	Mascot

27	tubulin alpha-1A chain [Mus musculus]	gi|6755901	53537.5	19	1072	1.130	1.143	1.084	0.440	0.440	0.561	35	35	35	100

Protein Group

Tubulin, alpha 1A [Rattus norvegicus]	gi|38328248	53421.4
tubulin alpha	gi|223556	53643.5

Peptide Information

Calc. Mass	Obsrv. Mass	± da	± ppm	Start Seq.	End Seq.	Sequence	Ion Score	C. I. %	Modification	Plate [#] Name	Gel Idx/Pos [4700 Sample Name]	iTRAQ Ratio 115/114*	iTRAQ Ratio 116/114*	iTRAQ Ratio 117/114*	Rank	Result Type
1063.6146	1063.6521	0.0375	35	106	112	GHYTIGK	40	99.748	(N-term)_iTRAQ[0], Lysine(K)_iTRAQ[7]	[4] F7 and F10+11	1016/1008	0.862	0.680	0.880	1	Mascot
1069.6139	1069.5664	-0.0475	-44	157	163	LSVDYGK	50	99.977	(N-term)_iTRAQ[0], Lysine(K)_iTRAQ[7]	[3] F6 and F9	186/178	1.105	1.103	0.965	1	Mascot
1069.6139	1069.588	-0.0259	-24	157	163	LSVDYGK	35	99.254	(N-term)_iTRAQ[0], Lysine(K)_iTRAQ[7]	[4] F7 and F10+11	184/176	2.054	1.109	1.285	1	Mascot
1175.6381	1175.603	-0.0351	-30	395	401	FDLMYAK	41	99.785	(N-term)_iTRAQ[0], Lysine(K)_iTRAQ[7]	[7] F5 120912	255/247	1.048	1.116	0.986	1	Mascot
1229.7225	1229.6846	-0.0379	-31	113	121	EIIDLVLDR	35	99.261	(N-term)_iTRAQ[0]	[1] F3 030912	412/404	0.821	1.183	1.148	1	Mascot
1303.7831	1303.7196	-0.0635	-49	327	336	DVNAAIATIK	72	100	(N-term)_iTRAQ[0], Lysine(K)_iTRAQ[10]	[5] F4	160/152	1.082	1.109	1.010	1	Mascot
1303.7831	1303.7577	-0.0254	-19	327	336	DVNAAIATIK	49	99.971	(N-term)_iTRAQ[0], Lysine(K)_iTRAQ[10]	[5] F4	597/589	1.385	1.345	1.031	1	Mascot

Calc. Mass	Obsrv. Mass	± da	± ppm	Start Seq.	End Seq.	Sequence	Ion Score	C. I. %	Modification	Plate [#] Name	Gel Idx/Pos [4700 Sample Name]	iTRAQ Ratio 115/114*	iTRAQ Ratio 116/114*	iTRAQ Ratio 117/114*	Rank	Result Type
1341.8113	1341.8744	0.0631	47	157	164	LSVDYGKK	47	99.946	(N-term)_iTRAQ[0], Lysine(K)_iTRAQ[7,8]	[4] F7 and F10+11	1058/1050	0.812	0.883	1.166	1	Mascot
1371.5837	1371.552	-0.0317	-23	312	320	YMACCLLYR	32	98.334	(N-term)_iTRAQ[0], MMTS (C)[4,5]	[1] F3 030912	501/493	1.766	1.780	1.455	1	Mascot
1601.9711	1601.9298	-0.0413	-26	230	243	LIGQIVSSITASLR	52	99.984	(N-term)_iTRAQ[0]	[1] F3 030912	575/567	1.151	1.342	1.449	1	Mascot
1601.9711	1601.9503	-0.0208	-13	230	243	LIGQIVSSITASLR	50	99.974	(N-term)_iTRAQ[0]	[8] F13-15 and F1+2	220/212	0.931	1.153	1.311	1	Mascot
1698.9789	1698.9282	-0.0507	-30	85	96	QLFHPEQLITGK	73	100	(N-term)_iTRAQ[0], Lysine(K)_iTRAQ[12]	[6] F8 110912	370/362	1.300	1.240	0.957	1	Mascot
1698.9789	1698.9305	-0.0484	-28	85	96	QLFHPEQLITGK	38	99.641	(N-term)_iTRAQ[0], Lysine(K)_iTRAQ[12]	[7] F5 120912	275/267	1.165	1.747	1.318	1	Mascot
1698.9789	1698.9484	-0.0305	-18	85	96	QLFHPEQLITGK	48	99.962	(N-term)_iTRAQ[0], Lysine(K)_iTRAQ[12]	[6] F8 110912	587/579	2.345	2.048	3.682	1	Mascot
1698.9789	1698.9783	-0.0006	0	85	96	QLFHPEQLITGK	81	100	(N-term)_iTRAQ[0], Lysine(K)_iTRAQ[12]	[4] F7 and F10+11	311/303	0.826	0.760	0.890	1	Mascot
1698.9789	1699.0499	0.071	42	85	96	QLFHPEQLITGK	32	98.505	(N-term)_iTRAQ[0], Lysine(K)_iTRAQ[12]	[3] F6 and F9	1265/1257	1.091	1.050	1.033	1	Mascot
1813.0054	1812.9636	-0.0418	-23	391	401	LDHKFDLMYAK	38	99.609	(N-term)_iTRAQ[0], Lysine(K)_iTRAQ[4,11]	[2] F12 040912	317/309	0.885	0.665	1.291	1	Mascot
1829.0002	1828.9752	-0.025	-14	391	401	LDHKFDLMYAK	38	99.613	(N-term)_iTRAQ[0], Lysine(K)_iTRAQ[4,11], Oxidation (M)[8]	[2] F12 040912	276/268	0.839	0.682	1.068	1	Mascot
1846.0082	1845.9462	-0.062	-34	65	79	AVFVDLEPTVIDEVR	63	99.999	(N-term)_iTRAQ[0]	[1] F3 030912	427/419	0.819	1.071	1.252	1	Mascot
1846.0082	1845.97	-0.0382	-21	65	79	AVFVDLEPTVIDEVR	62	99.998	(N-term)_iTRAQ[0]	[8] F13-15 and F1+2	216/208	1.793	1.969	1.936	1	Mascot
1846.0082	1846.0332	0.025	14	65	79	AVFVDLEPTVIDEVR	49	99.967	(N-term)_iTRAQ[0]	[8] F13-15 and F1+2	386/378	1.231	1.311	1.173	1	Mascot
1862.9845	1862.9556	-0.0289	-16	216	229	NLDIERPTYTNLNR	30	97.732	(N-term)_iTRAQ[0]	[4] F7 and F10+11	238/230	1.553	1.246	1.030	1	Mascot
1875.9364	1875.8833	-0.0531	-28	340	352	TIQFVDWCPTGFK	56	99.993	(N-term)_iTRAQ[0], Lysine(K)_iTRAQ[13], MMTS (C)[8]	[1] F3 030912	486/478	0.440	0.524	0.264	1	Mascot
1875.9364	1875.886	-0.0504	-27	340	352	TIQFVDWCPTGFK	59	99.997	(N-term)_iTRAQ[0], Lysine(K)_iTRAQ[13], MMTS (C)[8]	[5] F4	378/370	0.939	0.969	1.057	1	Mascot
1997.9712	1997.916	-0.0552	-28	374	390	AVCMLSNTTAIAEAWAR	29	96.623	(N-term)_iTRAQ[0], MMTS (C)[3]	[1] F3 030912	492/484	2.182	2.439	1.956	1	Mascot
1997.9712	1997.9668	-0.0044	-2	374	390	AVCMLSNTTAIAEAWAR	38	99.619	(N-term)_iTRAQ[0], MMTS (C)[3]	[8] F13-15 and F1+2	310/302	1.165	1.072	0.786	1	Mascot
1997.9712	1997.9895	0.0183	9	374	390	AVCMLSNTTAIAEAWAR	39	99.666	(N-term)_iTRAQ[0], MMTS (C)[3]	[4] F7 and F10+11	495/487	1.117	0.977	0.445	1	Mascot
1997.9712	1998.0536	0.0824	41	374	390	AVCMLSNTTAIAEAWAR	53	99.986	(N-term)_iTRAQ[0], MMTS (C)[3]	[4] F7 and F10+11	1343/1335	1.505	1.793	0.689	1	Mascot
2045.1681	2045.1163	-0.0518	-25	265	280	IHFPLATYAPVISAEK	33	98.745	(N-term)_iTRAQ[0], Lysine(K)_iTRAQ[16]	[3] F6 and F9	399/391	0.894	1.154	1.043	1	Mascot
2045.1681	2045.1881	0.02	10	265	280	IHFPLATYAPVISAEK	66	99.999	(N-term)_iTRAQ[0], Lysine(K)_iTRAQ[16]	[4] F7 and F10+11	414/406	0.821	1.282	1.061	1	Mascot
2113.1902	2113.1169	-0.0733	-35	353	370	VGINYQPPTVVPGGDLAK	107	100	(N-term)_iTRAQ[0], Lysine(K)_iTRAQ[18]	[1] F3 030912	309/301	1.136	1.140	0.923	1	Mascot
2296.0979	2296.031	-0.0669	-29	41	60	TIGGGDDSFNTFFSETGAGK	123	100	(N-term)_iTRAQ[0], Lysine(K)_iTRAQ[20]	[1] F3 030912	351/343	1.229	1.113	1.269	1	Mascot
2474.1206	2473.991	-0.1296	-52	403	422	AFVHWYVGEGMEEGEFSEAR	44	99.905	(N-term)_iTRAQ[0]	[6] F8 110912	399/391	1.267	1.191	1.075	1	Mascot
2474.1206	2474.1038	-0.0168	-7	403	422	AFVHWYVGEGMEEGEFSEAR	54	99.989	(N-term)_iTRAQ[0]	[4] F7 and F10+11	397/389	1.024	1.067	0.778	1	Mascot
2703.4099	2703.511	0.1011	37	85	105	QLFHPEQLITGKEDAANNYAR	31	97.893	(N-term)_iTRAQ[0], Lysine(K)_iTRAQ[12]	[4] F7 and F10+11	1207/1199	1.179	0.877	1.646	1	Mascot

28	AP-2 complex subunit beta [Rattus norvegicus]	gi|18034787	115294.7	21	1022	0.847	0.975	0.961	0.312	0.383	0.279	26	26	26	100

Protein Group

AP-2 complex subunit beta isoform b [Homo sapiens] gi|4557469 114156.2

Peptide Information

Calc. Mass	Obsrv. Mass	± da	± ppm	Start Seq.	End Seq.	Sequence	Ion Score	C. I. %	Modification	Plate [#] Name	Gel Idx/Pos [4700 Sample Name]	iTRAQ Ratio 115/114*	iTRAQ Ratio 116/114*	iTRAQ Ratio 117/114*	Rank	Result Type
927.5913	927.5652	-0.0261	-28	323	327	VFFVK	30	97.642	(N-term)_iTRAQ[0], Lysine(K)_iTRAQ[5]	[3] F6 and F9	276/268	0.689	1.146	1.283	1	Mascot
1001.5328	1001.515	-0.0178	-18	523	528	GYIYWR	32	98.338	(N-term)_iTRAQ[0]	[3] F6 and F9	278/270	0.599	0.615	0.615	1	Mascot
1171.7449	1171.7028	-0.0421	-36	726	733	AVWLPAVK	50	99.975	(N-term)_iTRAQ[0], Lysine(K)_iTRAQ[8]	[7] F5 120912	275/267	0.855	0.622	1.273	1	Mascot
1229.7827	1229.7479	-0.0348	-28	305	312	NINLIVQK	43	99.886	(N-term)_iTRAQ[0], Lysine(K)_iTRAQ[8]	[7] F5 120912	198/190	0.659	0.823	1.064	1	Mascot
1299.7195	1299.6555	-0.064	-49	328	335	YNDPIYVK	33	98.627	(N-term)_iTRAQ[0], Lysine(K)_iTRAQ[8]	[7] F5 120912	179/171	0.982	1.190	1.066	1	Mascot
1361.6295	1361.5603	-0.0692	-51	94	103	DCEDPNPLIR	28	96.439	(N-term)_iTRAQ[0], MMTS (C)[2]	[1] F3 030912	235/227	0.620	1.163	1.095	1	Mascot
1361.7297	1361.6544	-0.0753	-55	736	746	GLEISGTFTHR	38	99.623	(N-term)_iTRAQ[0]	[6] F8 110912	240/232	0.435	0.537	0.507	1	Mascot
1395.8583	1395.9244	0.0661	47	12	19	KGEIFELK	43	99.887	(N-term)_iTRAQ[0], Lysine(K)_iTRAQ[1,8]	[4] F7 and F10+11	1144/1136	0.907	1.168	0.745	1	Mascot
1571.7788	1571.8212	0.0424	27	129	138	CLKDEDPYVR	30	97.576	(N-term)_iTRAQ[0], Lysine(K)_iTRAQ[3],	[3] F6 and F9	1187/1179	0.684	0.880	0.781	1	Mascot

Calc. Mass	Obsrv. Mass	± da	± ppm	Start Seq.	End Seq.	Sequence	Ion Score	C. I. %	Modification	Plate [#] Name	Gel Idx/Pos [4700 Sample Name]	iTRAQ Ratio 115/114*	iTRAQ Ratio 116/114*	iTRAQ Ratio 117/114*	Rank	Result Type
									MMTS (C)[1]							
1586.7822	1586.7245	-0.0577	-36	360	371	EYATEVDVDFVR	50	99.973	(N-term)_iTRAQ[0]	[1] F3 030912	296/288	1.273	1.076	1.201	1	Mascot
1586.7822	1586.7748	-0.0074	-5	360	371	EYATEVDVDFVR	76	100	(N-term)_iTRAQ[0]	[8] F13-15 and F1+2	474/466	1.126	1.045	0.787	1	Mascot
1586.7822	1586.797	0.0148	9	360	371	EYATEVDVDFVR	49	99.972	(N-term)_iTRAQ[0]	[8] F13-15 and F1+2	477/469	0.870	1.026	0.845	1	Mascot
1643.9401	1643.871	-0.0691	-42	811	822	MEPLNNLQVAVK	42	99.851	(N-term)_iTRAQ[0], Lysine(K)_iTRAQ[12]	[5] F4	216/208	1.030	1.115	0.959	1	Mascot
1732.9844	1732.8806	-0.1038	-60	919	931	IQPGNPNYTLSLK	47	99.95	(N-term)_iTRAQ[0], Lysine(K)_iTRAQ[13]	[5] F4	188/180	0.979	0.810	0.977	1	Mascot
1812.9412	1812.8472	-0.094	-52	894	906	NVEGQDMLYQSLK	59	99.997	(N-term)_iTRAQ[0], Lysine(K)_iTRAQ[13]	[5] F4	200/192	0.466	1.344	0.950	1	Mascot
1876.0062	1875.9169	-0.0893	-48	857	869	DIPNENELQFQIK	67	99.999	(N-term)_iTRAQ[0], Lysine(K)_iTRAQ[13]	[5] F4	225/217	1.214	1.270	1.100	1	Mascot
1876.0062	1875.9373	-0.0689	-37	857	869	DIPNENELQFQIK	35	99.167	(N-term)_iTRAQ[0], Lysine(K)_iTRAQ[13]	[1] F3 030912	302/294	0.695	0.483	0.726	1	Mascot
1900.9296	1900.986	0.0564	30	747	759	QGHIYMEMNFTNK	62	99.998	(N-term)_iTRAQ[0], Lysine(K)_iTRAQ[13]	[3] F6 and F9	1232/1224	0.620	1.123	0.882	1	Mascot
1913.0103	1913.0256	0.0153	8	115	127	VDKITEYLCEPLR	48	99.965	(N-term)_iTRAQ[0], Lysine(K)_iTRAQ[3], MMTS (C)[9]	[4] F7 and F10+11	450/442	1.102	1.414	1.346	1	Mascot
1926.0074	1925.9187	-0.0887	-46	79	93	SQPDMAIMAVNSFVK	37	99.541	(N-term)_iTRAQ[0], Lysine(K)_iTRAQ[15]	[1] F3 030912	392/384	0.834	0.916	0.903	1	Mascot
1952.031	1951.9601	-0.0709	-36	760	773	ALQHMTDFAIQFNK	97	100	(N-term)_iTRAQ[0], Lysine(K)_iTRAQ[14]	[6] F8 110912	372/364	0.804	0.928	1.186	1	Mascot
1952.031	1952.0179	-0.0131	-7	760	773	ALQHMTDFAIQFNK	88	100	(N-term)_iTRAQ[0], Lysine(K)_iTRAQ[14]	[4] F7 and F10+11	373/365	0.896	0.708	1.191	1	Mascot
1952.031	1952.1044	0.0734	38	760	773	ALQHMTDFAIQFNK	29	96.722	(N-term)_iTRAQ[0], Lysine(K)_iTRAQ[14]	[4] F7 and F10+11	1195/1187	0.975	0.630	0.650	1	Mascot
2304.2373	2304.1807	-0.0566	-25	934	950	APEVSQYIYQVYDSILK	84	100	(N-term)_iTRAQ[0], Lysine(K)_iTRAQ[17]	[1] F3 030912	534/526	1.454	1.479	1.455	1	Mascot
2345.168	2345.1128	-0.0552	-24	148	166	LHDINAQMVEDQGFLDSLR	48	99.96	(N-term)_iTRAQ[0]	[7] F5 120912	388/380	0.764	1.262	1.021	1	Mascot
3199.6692	3199.5825	-0.0867	-27	495	520	KPSETQELVQQVLSLATQDSDNPDLR	31	97.908	(N-term)_iTRAQ[0], Lysine(K)_iTRAQ[1]	[7] F5 120912	493/485	1.580	1.943	1.146	1	Mascot

29	RecName: Full=Plasma membrane calcium-transporting ATPase 1; Short=PMCA1; AltName: Full=Plasma memb	gi|14286099	152382.9	15	1008	0.887	0.789	0.787	0.224	0.259	0.269	17	17	17	100

Peptide Information

Calc. Mass	Obsrv. Mass	± da	± ppm	Start Seq.	End Seq.	Sequence	Ion Score	C. I. %	Modification	Plate [#] Name	Gel Idx/Pos [4700 Sample Name]	iTRAQ Ratio 115/114*	iTRAQ Ratio 116/114*	iTRAQ Ratio 117/114*	Rank	Result Type
1063.6173	1063.5909	-0.0264	-25	1103	1109	GQILWFR	30	97.593	(N-term)_iTRAQ[0]	[7] F5 120912	330/322	0.913	0.784	0.531	1	Mascot
1348.7192	1348.6387	-0.0805	-60	779	789	GIIDSTVSEQR	59	99.997	(N-term)_iTRAQ[0]	[1] F3 030912	185/177	1.107	1.048	0.948	1	Mascot
1446.7747	1446.7833	0.0086	6	635	646	TVIEPMASEGLR	31	98.174	(N-term)_iTRAQ[0]	[8] F13-15 and F1+2	524/516	0.780	0.674	0.585	1	Mascot
1446.7747	1446.7932	0.0185	13	635	646	TVIEPMASEGLR	50	99.974	(N-term)_iTRAQ[0]	[8] F13-15 and F1+2	573/565	0.666	0.620	0.702	1	Mascot
1493.8097	1493.7333	-0.0764	-51	565	574	NEIPEEALYK	57	99.995	(N-term)_iTRAQ[0], Lysine(K)_iTRAQ[10]	[5] F4	157/149	0.977	1.123	0.786	1	Mascot
1583.8713	1583.7921	-0.0792	-50	485	495	MTVVQAYINEK	60	99.998	(N-term)_iTRAQ[0], Lysine(K)_iTRAQ[11]	[5] F4	222/214	0.967	1.107	0.658	1	Mascot
1677.855	1677.7393	-0.1157	-69	325	337	AQDGAAMEMQPLK	64	99.999	(N-term)_iTRAQ[0], Lysine(K)_iTRAQ[13]	[5] F4	138/130	0.732	0.672	0.865	1	Mascot
1746.8506	1746.8075	-0.0431	-25	1177	1189	SSIHNFMTHPEFR	35	99.249	(N-term)_iTRAQ[0]	[2] F12 040912	290/282	1.270	1.084	1.358	1	Mascot
1800.8848	1800.7812	-0.1036	-58	65	80	TSPNEGLSGNPADLER	62	99.998	(N-term)_iTRAQ[0]	[1] F3 030912	197/189	1.388	1.028	1.575	1	Mascot
1800.8848	1800.9136	0.0288	16	65	80	TSPNEGLSGNPADLER	27	95.327	(N-term)_iTRAQ[0]	[8] F13-15 and F1+2	575/567	0.820	0.475	0.459	1	Mascot
1901.9478	1901.9454	-0.0024	-1	20	35	EANHDGDFGITLAELR	102	100	(N-term)_iTRAQ[0]	[4] F7 and F10+11	350/342	1.196	0.910	0.858	1	Mascot
1909.9266	1909.8694	-0.0572	-30	49	62	IQESYGDVYGICTK	59	99.997	(N-term)_iTRAQ[0], Lysine(K)_iTRAQ[14], MMTS (C)[12]	[5] F4	263/255	0.710	0.689	0.767	1	Mascot
1930.0491	1929.9447	-0.1044	-54	790	806	QVVAVTGDGTNDGPALK	139	100	(N-term)_iTRAQ[0], Lysine(K)_iTRAQ[17]	[1] F3 030912	207/199	0.811	0.816	0.750	1	Mascot
2169.1536	2169.0759	-0.0777	-36	824	840	EASDIILTDDNFTSIVK	94	100	(N-term)_iTRAQ[0], Lysine(K)_iTRAQ[17]	[1] F3 030912	385/377	0.721	0.657	0.864	1	Mascot
2202.2466	2202.2207	-0.0259	-12	790	807	QVVAVTGDGTNDGPALKK	45	99.929	(N-term)_iTRAQ[0], Lysine(K)_iTRAQ[17, 18]	[4] F7 and F10+11	194/186	0.780	0.419	0.852	1	Mascot
2203.2219	2203.1421	-0.0798	-36	220	237	YGDLLPADGILIQGNDLK	59	99.997	(N-term)_iTRAQ[0], Lysine(K)_iTRAQ[18]	[1] F3 030912	419/411	0.990	0.919	0.809	1	Mascot
2259.9749	2259.9724	-0.0025	-1	459	476	HLDACETMGNATAICSDK	96	100	(N-term)_iTRAQ[0], Lysine(K)_iTRAQ[18], MMTS (C)[5,15]	[4] F7 and F10+11	378/370	0.641	0.897	0.639	1	Mascot

30	vesicle-fusing ATPase [Rattus norvegicus]	gi|13489067	91561.9	18	1003	0.999	1.080	0.963	0.211	0.231	0.263	22	22	22	100

Peptide Information

Calc. Mass	Obsrv. Mass	± da	± ppm	Start Seq.	End Seq.	Sequence	Ion Score	C. I. %	Modification	Plate [#] Name	Gel Idx/Pos [4700 Sample Name]	iTRAQ Ratio 115/114*	iTRAQ Ratio 116/114*	iTRAQ Ratio 117/114*	Rank	Result Type

Calc. Mass	Obsrv. Mass	± da	± ppm	Start Seq.	End Seq.	Sequence	Ion Score	C. I. %	Modification	Plate [#] Name	Gel Idx/Pos [4700 Sample Name]	iTRAQ Ratio 115/114*	iTRAQ Ratio 116/114*	iTRAQ Ratio 117/114*	Rank	Result Type
1003.655	1003.6295	-0.0255	-25	703	708	VWIGIK	34	99.044	(N-term)_iTRAQ[0], Lysine(K)_iTRAQ[6]	[3] F6 and F9	292/284	0.895	0.998	0.939	1	Mascot
1271.6915	1271.6417	-0.0498	-39	573	581	MIGFSETAK	32	98.595	(N-term)_iTRAQ[0], Lysine(K)_iTRAQ[9]	[7] F5 120912	186/178	1.117	0.833	1.105	1	Mascot
1281.6559	1281.5822	-0.0737	-58	294	303	YVGESEANIR	31	98.165	(N-term)_iTRAQ[0]	[1] F3 030912	174/166	0.862	0.962	0.543	1	Mascot
1281.6559	1281.5917	-0.0642	-50	294	303	YVGESEANIR	51	99.98	(N-term)_iTRAQ[0]	[5] F4	112/104	0.996	1.016	1.119	1	Mascot
1345.8188	1345.8025	-0.0163	-12	404	413	LQILHIHTAR	39	99.705	(N-term)_iTRAQ[0]	[2] F12 040912	276/268	0.828	1.164	0.923	1	Mascot
1345.8188	1345.8931	0.0743	55	404	413	LQILHIHTAR	30	97.354	(N-term)_iTRAQ[0]	[8] F13-15 and F1+2	1500/1492	1.718	1.063	1.221	1	Mascot
1435.7665	1435.7201	-0.0464	-32	435	446	NFSGAELEGLVR	52	99.986	(N-term)_iTRAQ[0]	[1] F3 030912	323/315	1.027	1.239	1.264	1	Mascot
1463.7185	1463.6215	-0.097	-66	28	38	DYQSGQHVMVR	65	99.999	(N-term)_iTRAQ[0]	[6] F8 110912	134/126	0.878	1.129	0.840	1	Mascot
1507.7625	1507.6764	-0.0861	-57	305	315	LFADAEEEQRR	49	99.972	(N-term)_iTRAQ[0]	[6] F8 110912	207/199	0.915	0.781	0.858	1	Mascot
1508.8196	1508.8047	-0.0149	-10	255	266	GILLYGPPGCGK	27	95.24	(N-term)_iTRAQ[0], Lysine(K)_iTRAQ[12], MMTS (C)[10]	[5] F4	263/255	1.364	1.276	1.385	1	Mascot
1519.8287	1519.7621	-0.0666	-44	151	161	DIEAMDPSILK	67	100	(N-term)_iTRAQ[0], Lysine(K)_iTRAQ[11]	[5] F4	216/208	1.089	1.069	1.121	1	Mascot
1582.8727	1582.8232	-0.0495	-31	556	566	IAEESNFPFIK	57	99.995	(N-term)_iTRAQ[0], Lysine(K)_iTRAQ[11]	[5] F4	257/249	0.842	0.733	0.699	1	Mascot
1583.9003	1583.7938	-0.1065	-67	416	427	GHQLLSADVDIK	81	100	(N-term)_iTRAQ[0], Lysine(K)_iTRAQ[12]	[6] F8 110912	250/242	0.967	1.527	1.094	1	Mascot
1623.8588	1623.9399	0.0811	50	304	314	KLFADAEEEQR	65	99.999	(N-term)_iTRAQ[0], Lysine(K)_iTRAQ[1]	[4] F7 and F10+11	1088/1080	0.878	1.098	0.785	1	Mascot
1652.8088	1652.7605	-0.0483	-29	595	607	SQLSCVVVDDIER	31	97.884	(N-term)_iTRAQ[0], MMTS (C)[5]	[1] F3 030912	367/359	1.237	1.252	1.042	1	Mascot
1745.9895	1745.9041	-0.0854	-49	517	529	VLDDGELLVQQTK	77	100	(N-term)_iTRAQ[0], Lysine(K)_iTRAQ[13]	[5] F4	229/221	0.860	0.938	1.143	1	Mascot
1778.9324	1778.9021	-0.0303	-17	206	217	QSIINPDWNFEK	63	99.999	(N-term)_iTRAQ[0], Lysine(K)_iTRAQ[12]	[7] F5 120912	307/299	0.924	1.148	1.112	1	Mascot
1810.9132	1810.8508	-0.0624	-34	239	251	VFPPEIVEQMGCK	52	99.985	(N-term)_iTRAQ[0], Lysine(K)_iTRAQ[13], MMTS (C)[12]	[1] F3 030912	419/411	0.980	1.617	1.139	1	Mascot
1973.0411	1972.9625	-0.0786	-40	218	232	MGIGGLDKEFSDIFR	69	100	(N-term)_iTRAQ[0], Lysine(K)_iTRAQ[8]	[6] F8 110912	476/468	1.028	1.308	0.789	1	Mascot
1973.0411	1973.0702	0.0291	15	218	232	MGIGGLDKEFSDIFR	63	99.999	(N-term)_iTRAQ[0], Lysine(K)_iTRAQ[8]	[4] F7 and F10+11	475/467	0.801	0.913	0.588	1	Mascot
1989.0361	1988.9487	-0.0874	-44	218	232	MGIGGLDKEFSDIFR	51	99.981	(N-term)_iTRAQ[0], Lysine(K)_iTRAQ[8], Oxidation (M)[1]	[6] F8 110912	476/468	0.833	1.208	0.865	1	Mascot
2202.0122	2201.9519	-0.0603	-27	11	27	CPTDELSLSNCAVVNEK	91	100	(N-term)_iTRAQ[0], Lysine(K)_iTRAQ[17], MMTS (C)[1,11]	[1] F3 030912	383/375	1.373	0.941	1.203	1	Mascot

31	pyruvate kinase isozymes M1/M2 [Rattus norvegicus]	gi|16757994	63526.5	15	996	1.080	1.072	1.056	0.430	0.409	0.413	21	21	21	100

Peptide Information

Calc. Mass	Obsrv. Mass	± da	± ppm	Start Seq.	End Seq.	Sequence	Ion Score	C. I. %	Modification	Plate [#] Name	Gel Idx/Pos [4700 Sample Name]	iTRAQ Ratio 115/114*	iTRAQ Ratio 116/114*	iTRAQ Ratio 117/114*	Rank	Result Type
1134.6643	1134.6005	-0.0638	-56	393	400	LLFEELAR	39	99.67	(N-term)_iTRAQ[0]	[5] F4	300/292	2.415	1.695	2.181	1	Mascot
1134.6643	1134.6421	-0.0222	-20	393	400	LLFEELAR	43	99.888	(N-term)_iTRAQ[0]	[1] F3 030912	389/381	0.838	0.832	0.644	1	Mascot
1173.6136	1173.5406	-0.073	-62	384	392	EAEAAVFHR	39	99.707	(N-term)_iTRAQ[0]	[6] F8 110912	164/156	0.875	1.169	1.145	1	Mascot
1173.6136	1173.6998	0.0862	73	384	392	EAEAAVFHR	47	99.948	(N-term)_iTRAQ[0]	[3] F6 and F9	1128/1120	0.995	0.870	1.075	1	Mascot
1210.6919	1210.6653	-0.0266	-22	468	475	GIFPVLCK	40	99.755	(N-term)_iTRAQ[0], Lysine(K)_iTRAQ[8], MMTS (C)[7]	[7] F5 120912	386/378	1.466	1.205	1.343	1	Mascot
1233.7235	1233.681	-0.0425	-34	490	498	VNLAMNVGK	57	99.995	(N-term)_iTRAQ[0], Lysine(K)_iTRAQ[9]	[7] F5 120912	186/178	0.846	0.905	0.853	1	Mascot
1233.7235	1233.6936	-0.0299	-24	490	498	VNLAMNVGK	44	99.909	(N-term)_iTRAQ[0], Lysine(K)_iTRAQ[9]	[3] F6 and F9	226/218	1.646	0.998	1.695	1	Mascot
1315.7343	1315.661	-0.0733	-56	33	43	LDIDSAPITAR	58	99.996	(N-term)_iTRAQ[0]	[1] F3 030912	241/233	0.909	0.727	0.669	1	Mascot
1429.8148	1429.72	-0.0948	-66	295	305	GDLGIEIPAEK	73	100	(N-term)_iTRAQ[0], Lysine(K)_iTRAQ[11]	[5] F4	182/174	1.134	1.000	1.073	1	Mascot
1485.7869	1485.6895	-0.0974	-66	142	151	ITLDNAYMEK	63	99.999	(N-term)_iTRAQ[0], Lysine(K)_iTRAQ[10]	[5] F4	185/177	0.659	0.605	0.622	1	Mascot
1485.7869	1485.7252	-0.0617	-42	142	151	ITLDNAYMEK	82	100	(N-term)_iTRAQ[0], Lysine(K)_iTRAQ[10]	[7] F5 120912	232/224	1.032	1.165	1.232	1	Mascot
1492.7717	1492.767	-0.0047	-3	44	56	NTGIICTIGPASR	39	99.682	(N-term)_iTRAQ[0], MMTS (C)[6]	[8] F13-15 and F1+2	474/466	1.455	1.360	1.022	1	Mascot
1492.7717	1492.7815	0.0098	7	44	56	NTGIICTIGPASR	46	99.944	(N-term)_iTRAQ[0], MMTS (C)[6]	[8] F13-15 and F1+2	470/462	0.794	0.808	1.006	1	Mascot
1745.8469	1745.7858	-0.0611	-35	152	162	CDENILWLDYK	61	99.998	(N-term)_iTRAQ[0], Lysine(K)_iTRAQ[11], MMTS (C)[1]	[5] F4	317/309	0.626	0.823	0.975	1	Mascot
1745.8469	1745.8217	-0.0252	-14	152	162	CDENILWLDYK	34	99.015	(N-term)_iTRAQ[0], Lysine(K)_iTRAQ[11], MMTS (C)[1]	[7] F5 120912	396/388	2.039	2.904	2.178	1	Mascot
1751.02	1750.9913	-0.0287	-16	174	186	IYVDDGLISLQVK	98	100	(N-term)_iTRAQ[0], Lysine(K)_iTRAQ[13]	[1] F3 030912	389/381	0.811	1.040	1.070	1	Mascot

Calc. Mass	Obsrv. Mass	± da	± ppm	Start Seq.	End Seq.	Sequence	Ion Score	C. I. %	Modification	Plate [#] Name	Gel Idx/Pos [4700 Sample Name]	iTRAQ Ratio 115/114*	iTRAQ Ratio 116/114*	iTRAQ Ratio 117/114*	Rank	Result Type
1925.0953	1925.0188	-0.0765	-40	208	224	GVNLPGAAVDLPAVSEK	92	100	(N-term)_iTRAQ[0], Lysine(K)_iTRAQ[17]	[1] F3 030912	320/312	1.114	0.978	0.862	1	Mascot
1966.0188	1966.0433	0.0245	12	279	294	RFDEILEASDGIMVAR	59	99.997	(N-term)_iTRAQ[0]	[4] F7 and F10+11	407/399	1.116	0.981	1.094	1	Mascot
2004.0021	2003.9596	-0.0425	-21	231	246	FGVEQDVDMVFASFIR	92	100	(N-term)_iTRAQ[0]	[1] F3 030912	569/561	1.066	1.188	1.071	1	Mascot
2197.2927	2197.2874	-0.0053	-2	207	224	KGVNLPGAAVDLPAVSE K	76	100	(N-term)_iTRAQ[0], Lysine(K)_iTRAQ[1,1 8]	[4] F7 and F10+11	300/292	1.221	1.445	0.844	1	Mascot
2298.241	2298.2778	0.0368	16	320	336	AGKPVICATQMLESMIK	73	100	(N-term)_iTRAQ[0], Lysine(K)_iTRAQ[3,1 7], MMTS (C)[7]	[4] F7 and F10+11	524/516	1.033	1.196	0.844	1	Mascot

32	tubulin alpha-1C chain [Rattus norvegicus]	gi|58865558	53195.3	18	986	1.145	1.140	1.083	0.454	0.444	0.564	34	34	34	100

Peptide Information

Calc. Mass	Obsrv. Mass	± da	± ppm	Start Seq.	End Seq.	Sequence	Ion Score	C. I. %	Modification	Plate [#] Name	Gel Idx/Pos [4700 Sample Name]	iTRAQ Ratio 115/114*	iTRAQ Ratio 116/114*	iTRAQ Ratio 117/114*	Rank	Result Type
1063.6146	1063.6521	0.0375	35	106	112	GHYTIGK	40	99.748	(N-term)_iTRAQ[0], Lysine(K)_iTRAQ[7]	[4] F7 and F10+11	1016/1008	0.862	0.680	0.880	1	Mascot
1069.6139	1069.5664	-0.0475	-44	157	163	LSVDYGK	50	99.977	(N-term)_iTRAQ[0], Lysine(K)_iTRAQ[7]	[3] F6 and F9	186/178	1.105	1.103	0.965	1	Mascot
1069.6139	1069.588	-0.0259	-24	157	163	LSVDYGK	35	99.254	(N-term)_iTRAQ[0], Lysine(K)_iTRAQ[7]	[4] F7 and F10+11	184/176	2.054	1.109	1.285	1	Mascot
1175.6381	1175.603	-0.0351	-30	395	401	FDLMYAK	41	99.785	(N-term)_iTRAQ[0], Lysine(K)_iTRAQ[7]	[7] F5 120912	255/247	1.048	1.116	0.986	1	Mascot
1229.7225	1229.6846	-0.0379	-31	113	121	EIIDLVLDR	35	99.261	(N-term)_iTRAQ[0]	[1] F3 030912	412/404	0.821	1.183	1.148	1	Mascot
1303.7831	1303.7196	-0.0635	-49	327	336	DVNAAIATIK	72	100	(N-term)_iTRAQ[0], Lysine(K)_iTRAQ[10]	[5] F4	160/152	1.082	1.109	1.010	1	Mascot
1303.7831	1303.7577	-0.0254	-19	327	336	DVNAAIATIK	49	99.971	(N-term)_iTRAQ[0], Lysine(K)_iTRAQ[10]	[5] F4	597/589	1.385	1.345	1.031	1	Mascot
1341.8113	1341.8744	0.0631	47	157	164	LSVDYGKK	47	99.946	(N-term)_iTRAQ[0], Lysine(K)_iTRAQ[7,8]	[4] F7 and F10+11	1058/1050	0.812	0.883	1.166	1	Mascot
1371.5837	1371.552	-0.0317	-23	312	320	YMACCLLYR	32	98.334	(N-term)_iTRAQ[0], MMTS (C)[4,5]	[1] F3 030912	501/493	1.766	1.780	1.455	1	Mascot
1631.9816	1631.9253	-0.0563	-34	230	243	LISQIVSSITASLR	73	100	(N-term)_iTRAQ[0]	[1] F3 030912	580/572	1.272	1.138	1.282	1	Mascot
1631.9816	1631.9677	-0.0139	-9	230	243	LISQIVSSITASLR	52	99.985	(N-term)_iTRAQ[0]	[8] F13-15 and F1+2	211/203	1.352	1.231	1.211	1	Mascot
1698.9789	1698.9282	-0.0507	-30	85	96	QLFHPEQLITGK	73	100	(N-term)_iTRAQ[0], Lysine(K)_iTRAQ[12]	[6] F8 110912	370/362	1.300	1.240	0.957	1	Mascot
1698.9789	1698.9305	-0.0484	-28	85	96	QLFHPEQLITGK	38	99.641	(N-term)_iTRAQ[0], Lysine(K)_iTRAQ[12]	[7] F5 120912	275/267	1.165	1.747	1.318	1	Mascot
1698.9789	1698.9484	-0.0305	-18	85	96	QLFHPEQLITGK	48	99.962	(N-term)_iTRAQ[0], Lysine(K)_iTRAQ[12]	[6] F8 110912	587/579	2.345	2.048	3.682	1	Mascot
1698.9789	1698.9783	-0.0006	0	85	96	QLFHPEQLITGK	81	100	(N-term)_iTRAQ[0], Lysine(K)_iTRAQ[12]	[4] F7 and F10+11	311/303	0.826	0.760	0.890	1	Mascot
1698.9789	1699.0499	0.071	42	85	96	QLFHPEQLITGK	32	98.505	(N-term)_iTRAQ[0], Lysine(K)_iTRAQ[12]	[3] F6 and F9	1265/1257	1.091	1.050	1.033	1	Mascot
1813.0054	1812.9636	-0.0418	-23	391	401	LDHKFDLMYAK	38	99.609	(N-term)_iTRAQ[0], Lysine(K)_iTRAQ[4,1 1]	[2] F12 040912	317/309	0.885	0.665	1.291	1	Mascot
1829.0002	1828.9752	-0.025	-14	391	401	LDHKFDLMYAK	38	99.613	(N-term)_iTRAQ[0], Lysine(K)_iTRAQ[4,1 1], Oxidation (M)[8]	[2] F12 040912	276/268	0.839	0.682	1.068	1	Mascot
1846.0082	1845.9462	-0.062	-34	65	79	AVFVDLEPTVIDEVR	63	99.999	(N-term)_iTRAQ[0]	[1] F3 030912	427/419	0.819	1.071	1.252	1	Mascot
1846.0082	1845.97	-0.0382	-21	65	79	AVFVDLEPTVIDEVR	62	99.998	(N-term)_iTRAQ[0]	[8] F13-15 and F1+2	216/208	1.793	1.969	1.936	1	Mascot
1846.0082	1846.0332	0.025	14	65	79	AVFVDLEPTVIDEVR	49	99.967	(N-term)_iTRAQ[0]	[8] F13-15 and F1+2	386/378	1.231	1.311	1.173	1	Mascot
1862.9845	1862.9556	-0.0289	-16	216	229	NLDIERPTYTNLNR	30	97.732	(N-term)_iTRAQ[0]	[4] F7 and F10+11	238/230	1.553	1.246	1.030	1	Mascot
1875.9364	1875.8833	-0.0531	-28	340	352	TIQFVDWCPTGFK	56	99.993	(N-term)_iTRAQ[0], Lysine(K)_iTRAQ[13], MMTS (C)[8]	[1] F3 030912	486/478	0.440	0.524	0.264	1	Mascot
1875.9364	1875.886	-0.0504	-27	340	352	TIQFVDWCPTGFK	59	99.997	(N-term)_iTRAQ[0], Lysine(K)_iTRAQ[13], MMTS (C)[8]	[5] F4	378/370	0.939	0.969	1.057	1	Mascot
1997.9712	1997.916	-0.0552	-28	374	390	AVCMLSNTTAIAEAWAR	29	96.623	(N-term)_iTRAQ[0], MMTS (C)[3]	[1] F3 030912	492/484	2.182	2.439	1.956	1	Mascot
1997.9712	1997.9668	-0.0044	-2	374	390	AVCMLSNTTAIAEAWAR	38	99.619	(N-term)_iTRAQ[0], MMTS (C)[3]	[8] F13-15 and F1+2	310/302	1.165	1.072	0.786	1	Mascot
1997.9712	1997.9895	0.0183	9	374	390	AVCMLSNTTAIAEAWAR	39	99.666	(N-term)_iTRAQ[0], MMTS (C)[3]	[4] F7 and F10+11	495/487	1.117	0.977	0.445	1	Mascot
1997.9712	1998.0536	0.0824	41	374	390	AVCMLSNTTAIAEAWAR	53	99.986	(N-term)_iTRAQ[0], MMTS (C)[3]	[4] F7 and F10+11	1343/1335	1.505	1.793	0.689	1	Mascot
2045.1681	2045.1163	-0.0518	-25	265	280	IHFPLATYAPVISAEK	33	98.745	(N-term)_iTRAQ[0], Lysine(K)_iTRAQ[16]	[3] F6 and F9	399/391	0.894	1.154	1.043	1	Mascot
2045.1681	2045.1881	0.02	10	265	280	IHFPLATYAPVISAEK	66	99.999	(N-term)_iTRAQ[0], Lysine(K)_iTRAQ[16]	[4] F7 and F10+11	414/406	0.821	1.282	1.061	1	Mascot
2296.0979	2296.031	-0.0669	-29	41	60	TIGGGDDSFNTFFSETGA GK	123	100	(N-term)_iTRAQ[0], Lysine(K)_iTRAQ[20]	[1] F3 030912	351/343	1.229	1.113	1.269	1	Mascot
2474.1206	2473.991	-0.1296	-52	403	422	AFVHWYVGEGMEEGEF SEAR	44	99.905	(N-term)_iTRAQ[0]	[6] F8 110912	399/391	1.267	1.191	1.075	1	Mascot
2474.1206	2474.1038	-0.0168	-7	403	422	AFVHWYVGEGMEEGEF SEAR	54	99.989	(N-term)_iTRAQ[0]	[4] F7 and F10+11	397/389	1.024	1.067	0.778	1	Mascot

2703.4099	2703.511	0.1011	37	85	105	QLFHPEQLITGKEDAANN YAR	31	97.893	(N-term)_iTRAQ[0], Lysine(K)_iTRAQ[12]	[4] F7 and F10+11	1207/1199	1.179	0.877	1.646	1	Mascot

33	tubulin alpha-4A chain [Mus musculus]	gi|6678467	53372.2	18	940	1.191	1.140	1.071	0.446	0.397	0.586	32	32	32	100

Peptide Information

Calc. Mass	Obsrv. Mass	± da	± ppm	Start Seq.	End Seq.	Sequence	Ion Score	C. I. %	Modification	Plate [#] Name	Gel Idx/Pos [4700 Sample Name]	iTRAQ Ratio 115/114*	iTRAQ Ratio 116/114*	iTRAQ Ratio 117/114*	Rank	Result Type
1063.6146	1063.6521	0.0375	35	106	112	GHYTIGK	40	99.748	(N-term)_iTRAQ[0], Lysine(K)_iTRAQ[7]	[4] F7 and F10+11	1016/1008	0.862	0.680	0.880	1	Mascot
1069.6139	1069.5664	-0.0475	-44	157	163	LSVDYGK	50	99.977	(N-term)_iTRAQ[0], Lysine(K)_iTRAQ[7]	[3] F6 and F9	186/178	1.105	1.103	0.965	1	Mascot
1069.6139	1069.588	-0.0259	-24	157	163	LSVDYGK	35	99.254	(N-term)_iTRAQ[0], Lysine(K)_iTRAQ[7]	[4] F7 and F10+11	184/176	2.054	1.109	1.285	1	Mascot
1175.6381	1175.603	-0.0351	-30	395	401	FDLMYAK	41	99.785	(N-term)_iTRAQ[0], Lysine(K)_iTRAQ[7]	[7] F5 120912	255/247	1.048	1.116	0.986	1	Mascot
1213.6913	1213.6324	-0.0589	-49	113	121	EIIDPVLDR	33	98.782	(N-term)_iTRAQ[0]	[1] F3 030912	262/254	0.816	1.099	0.795	1	Mascot
1273.7726	1273.6857	-0.0869	-68	327	336	DVNAAIAAIK	46	99.94	(N-term)_iTRAQ[0], Lysine(K)_iTRAQ[10]	[5] F4	185/177	1.200	1.164	1.026	1	Mascot
1341.8113	1341.8744	0.0631	47	157	164	LSVDYGKK	47	99.946	(N-term)_iTRAQ[0], Lysine(K)_iTRAQ[7,8]	[4] F7 and F10+11	1058/1050	0.812	0.883	1.166	1	Mascot
1371.5837	1371.552	-0.0317	-23	312	320	YMACCLLYR	32	98.334	(N-term)_iTRAQ[0], MMTS (C)[4,5]	[1] F3 030912	501/493	1.766	1.780	1.455	1	Mascot
1631.9816	1631.9253	-0.0563	-34	230	243	LISQIVSSITASLR	73	100	(N-term)_iTRAQ[0]	[1] F3 030912	580/572	1.272	1.138	1.282	1	Mascot
1631.9816	1631.9677	-0.0139	-9	230	243	LISQIVSSITASLR	52	99.985	(N-term)_iTRAQ[0]	[8] F13-15 and F1+2	211/203	1.352	1.231	1.211	1	Mascot
1698.9789	1698.9282	-0.0507	-30	85	96	QLFHPEQLITGK	73	100	(N-term)_iTRAQ[0], Lysine(K)_iTRAQ[12]	[6] F8 110912	370/362	1.300	1.240	0.957	1	Mascot
1698.9789	1698.9305	-0.0484	-28	85	96	QLFHPEQLITGK	38	99.641	(N-term)_iTRAQ[0], Lysine(K)_iTRAQ[12]	[7] F5 120912	275/267	1.165	1.747	1.318	1	Mascot
1698.9789	1698.9484	-0.0305	-18	85	96	QLFHPEQLITGK	48	99.962	(N-term)_iTRAQ[0], Lysine(K)_iTRAQ[12]	[6] F8 110912	587/579	2.345	2.048	3.682	1	Mascot
1698.9789	1698.9783	-0.0006	0	85	96	QLFHPEQLITGK	81	100	(N-term)_iTRAQ[0], Lysine(K)_iTRAQ[12]	[4] F7 and F10+11	311/303	0.826	0.760	0.890	1	Mascot
1698.9789	1699.0499	0.071	42	85	96	QLFHPEQLITGK	32	98.505	(N-term)_iTRAQ[0], Lysine(K)_iTRAQ[12]	[3] F6 and F9	1265/1257	1.091	1.050	1.033	1	Mascot
1813.0054	1812.9636	-0.0418	-23	391	401	LDHKFDLMYAK	38	99.609	(N-term)_iTRAQ[0], Lysine(K)_iTRAQ[4,11]	[2] F12 040912	317/309	0.885	0.665	1.291	1	Mascot
1829.0002	1828.9752	-0.025	-14	391	401	LDHKFDLMYAK	38	99.613	(N-term)_iTRAQ[0], Lysine(K)_iTRAQ[4,11], Oxidation (M)[8]	[2] F12 040912	276/268	0.839	0.682	1.068	1	Mascot
1860.0238	1860.04	0.0162	9	65	79	AVFVDLEPTVIDEIR	62	99.998	(N-term)_iTRAQ[0]	[8] F13-15 and F1+2	364/356	0.970	0.959	0.878	1	Mascot
1861.9208	1861.8561	-0.0647	-35	340	352	SIQFVDWCPTGFK	31	97.854	(N-term)_iTRAQ[0], Lysine(K)_iTRAQ[13], MMTS (C)[8]	[6] F8 110912	488/480	0.875	1.523	0.343	1	Mascot
1861.9208	1861.8618	-0.059	-32	340	352	SIQFVDWCPTGFK	32	98.315	(N-term)_iTRAQ[0], Lysine(K)_iTRAQ[13], MMTS (C)[8]	[1] F3 030912	478/470	2.663	0.986	2.990	1	Mascot
1861.9208	1861.8782	-0.0426	-23	340	352	SIQFVDWCPTGFK	58	99.996	(N-term)_iTRAQ[0], Lysine(K)_iTRAQ[13], MMTS (C)[8]	[5] F4	373/365	1.178	1.009	1.062	1	Mascot
1862.9845	1862.9556	-0.0289	-16	216	229	NLDIERPTYTNLNR	30	97.732	(N-term)_iTRAQ[0]	[4] F7 and F10+11	238/230	1.553	1.246	1.030	1	Mascot
1997.9712	1997.916	-0.0552	-28	374	390	AVCMLSNTTAIAEAWAR	29	96.623	(N-term)_iTRAQ[0], MMTS (C)[3]	[1] F3 030912	492/484	2.182	2.439	1.956	1	Mascot
1997.9712	1997.9668	-0.0044	-2	374	390	AVCMLSNTTAIAEAWAR	38	99.619	(N-term)_iTRAQ[0], MMTS (C)[3]	[8] F13-15 and F1+2	310/302	1.165	1.072	0.786	1	Mascot
1997.9712	1997.9895	0.0183	9	374	390	AVCMLSNTTAIAEAWAR	39	99.666	(N-term)_iTRAQ[0], MMTS (C)[3]	[4] F7 and F10+11	495/487	1.117	0.977	0.445	1	Mascot
1997.9712	1998.0536	0.0824	41	374	390	AVCMLSNTTAIAEAWAR	53	99.986	(N-term)_iTRAQ[0], MMTS (C)[3]	[4] F7 and F10+11	1343/1335	1.505	1.793	0.689	1	Mascot
2045.1681	2045.1163	-0.0518	-25	265	280	IHFPLATYAPVISAEK	33	98.745	(N-term)_iTRAQ[0], Lysine(K)_iTRAQ[16]	[3] F6 and F9	399/391	0.894	1.154	1.043	1	Mascot
2045.1681	2045.1881	0.02	10	265	280	IHFPLATYAPVISAEK	66	99.999	(N-term)_iTRAQ[0], Lysine(K)_iTRAQ[16]	[4] F7 and F10+11	414/406	0.821	1.282	1.061	1	Mascot
2113.1902	2113.1169	-0.0733	-35	353	370	VGINYQPPTVVPGGDLAK	107	100	(N-term)_iTRAQ[0], Lysine(K)_iTRAQ[18]	[1] F3 030912	309/301	1.136	1.140	0.923	1	Mascot
2474.1206	2473.991	-0.1296	-52	403	422	AFVHWYVGEGMEEGEF SEAR	44	99.905	(N-term)_iTRAQ[0]	[6] F8 110912	399/391	1.267	1.191	1.075	1	Mascot
2474.1206	2474.1038	-0.0168	-7	403	422	AFVHWYVGEGMEEGEF SEAR	54	99.989	(N-term)_iTRAQ[0]	[4] F7 and F10+11	397/389	1.024	1.067	0.778	1	Mascot
2703.4099	2703.511	0.1011	37	85	105	QLFHPEQLITGKEDAANN YAR	31	97.893	(N-term)_iTRAQ[0], Lysine(K)_iTRAQ[12]	[4] F7 and F10+11	1207/1199	1.179	0.877	1.646	1	Mascot

34	malate dehydrogenase, mitochondrial precursor [Rattus norvegicus]	gi|42476181	39775.4	14	918	1.007	1.029	1.063	0.333	0.353	0.395	23	23	23	100

Peptide Information

Calc. Mass	Obsrv. Mass	± da	± ppm	Start Seq.	End Seq.	Sequence	Ion Score	C. I. %	Modification	Plate [#] Name	Gel Idx/Pos [4700 Sample Name]	iTRAQ Ratio 115/114*	iTRAQ Ratio 116/114*	iTRAQ Ratio 117/114*	Rank	Result Type

1151.6558	1151.6045	-0.0513	-45	308	314	ITPFEEK	40	99.746	(N-term)_iTRAQ[0], Lysine(K)_iTRAQ[7]	[3] F6 and F9	212/204	1.200	1.034	1.184	1	Mascot
1280.746	1280.6855	-0.0605	-47	177	185	ANTFVAELK	58	99.996	(N-term)_iTRAQ[0], Lysine(K)_iTRAQ[9]	[7] F5 120912	230/222	0.874	1.009	1.139	1	Mascot
1361.7886	1361.7446	-0.044	-32	230	239	IQEAGTEVVK	69	100	(N-term)_iTRAQ[0], Lysine(K)_iTRAQ[10]	[7] F5 120912	142/134	0.871	1.089	1.099	1	Mascot
1377.8226	1377.7876	-0.035	-25	166	176	IFGVTTLDIVR	60	99.998	(N-term)_iTRAQ[0]	[1] F3 030912	402/394	1.019	1.235	1.235	1	Mascot
1402.8225	1402.7483	-0.0742	-53	315	324	MIAEAIPELK	51	99.983	(N-term)_iTRAQ[0], Lysine(K)_iTRAQ[10]	[5] F4	244/236	0.719	1.040	0.794	1	Mascot
1435.863	1435.8096	-0.0534	-37	192	203	VNVPVIGGHAGK	92	100	(N-term)_iTRAQ[0], Lysine(K)_iTRAQ[12]	[6] F8 110912	216/208	0.923	0.864	0.846	1	Mascot
1435.863	1435.9105	0.0475	33	192	203	VNVPVIGGHAGK	98	100	(N-term)_iTRAQ[0], Lysine(K)_iTRAQ[12]	[3] F6 and F9	1176/1168	1.243	1.442	1.324	1	Mascot
1471.7866	1471.7954	0.0088	6	92	104	GCDVVVIPAGVPR	43	99.883	(N-term)_iTRAQ[0], MMTS (C)[2]	[8] F13-15 and F1+2	483/475	1.994	1.576	1.289	1	Mascot
1471.7866	1471.7983	0.0117	8	92	104	GCDVVVIPAGVPR	59	99.997	(N-term)_iTRAQ[0], MMTS (C)[2]	[8] F13-15 and F1+2	434/426	0.955	0.646	0.824	1	Mascot
1471.7866	1471.803	0.0164	11	92	104	GCDVVVIPAGVPR	59	99.997	(N-term)_iTRAQ[0], MMTS (C)[2]	[8] F13-15 and F1+2	430/422	0.784	0.877	1.038	1	Mascot
1471.7866	1471.819	0.0324	22	92	104	GCDVVVIPAGVPR	43	99.864	(N-term)_iTRAQ[0], MMTS (C)[2]	[8] F13-15 and F1+2	535/527	0.843	0.677	0.529	1	Mascot
1598.808	1598.7439	-0.0641	-40	242	257	AGAGSATLSMAYAGAR	92	100	(N-term)_iTRAQ[0]	[1] F3 030912	224/216	1.089	1.171	0.951	1	Mascot
1615.8763	1615.8264	-0.0499	-31	258	269	FVFSLVDAMNGK	50	99.977	(N-term)_iTRAQ[0], Lysine(K)_iTRAQ[12]	[5] F4	363/355	1.168	1.505	1.639	1	Mascot
1647.9404	1647.8881	-0.0523	-32	204	215	TIIPLISQCTPK	40	99.778	(N-term)_iTRAQ[0], Lysine(K)_iTRAQ[12], MMTS (C)[9]	[1] F3 030912	412/404	1.306	1.268	1.258	1	Mascot
1704.9041	1704.8497	-0.0544	-32	216	229	VDFPQDQLATLTGR	58	99.996	(N-term)_iTRAQ[0]	[1] F3 030912	342/334	1.533	1.736	1.824	1	Mascot
1704.9041	1704.9167	0.0126	7	216	229	VDFPQDQLATLTGR	52	99.983	(N-term)_iTRAQ[0]	[8] F13-15 and F1+2	426/418	1.084	0.859	1.124	1	Mascot
1704.9041	1704.922	0.0179	10	216	229	VDFPQDQLATLTGR	57	99.995	(N-term)_iTRAQ[0]	[8] F13-15 and F1+2	430/422	0.702	0.837	0.985	1	Mascot
1704.9041	1704.9285	0.0244	14	216	229	VDFPQDQLATLTGR	76	100	(N-term)_iTRAQ[0]	[8] F13-15 and F1+2	530/522	0.747	0.670	0.624	1	Mascot
1766.9048	1766.8121	-0.0927	-52	79	91	GYLGPEQLPDCLK	78	100	(N-term)_iTRAQ[0], Lysine(K)_iTRAQ[13], MMTS (C)[11]	[5] F4	290/282	1.101	1.042	1.138	1	Mascot
1766.9048	1766.8628	-0.042	-24	79	91	GYLGPEQLPDCLK	56	99.993	(N-term)_iTRAQ[0], Lysine(K)_iTRAQ[13], MMTS (C)[11]	[1] F3 030912	375/367	0.629	0.615	0.762	1	Mascot
2081.2944	2081.2373	-0.0571	-27	27	45	VAVLGASGGIGQPLSLLLK	97	100	(N-term)_iTRAQ[0], Lysine(K)_iTRAQ[19]	[1] F3 030912	501/493	1.709	1.616	2.172	1	Mascot
2081.2944	2081.2808	-0.0136	-7	27	45	VAVLGASGGIGQPLSLLLK	40	99.743	(N-term)_iTRAQ[0], Lysine(K)_iTRAQ[19]	[8] F13-15 and F1+2	303/295	0.887	0.993	1.073	1	Mascot
2537.3484	2537.3508	0.0024	1	53	74	LTLYDIAHTPGVAADLSHIETR	51	99.98	(N-term)_iTRAQ[0]	[4] F7 and F10+11	432/424	0.798	0.919	0.854	1	Mascot

35 Tpi1 protein [Rattus norvegicus] gi|38512111 30284.8 12 918 1.068 1.043 1.191 0.271 0.326 0.303 14 14 14 100

Peptide Information

Calc. Mass	Obsrv. Mass	± da	± ppm	Start Seq.	End Seq.	Sequence	Ion Score	C. I. %	Modification	Plate [#] Name	Gel Idx/Pos [4700 Sample Name]	iTRAQ Ratio 115/114*	iTRAQ Ratio 116/114*	iTRAQ Ratio 117/114*	Rank	Result Type
1242.6881	1242.6777	-0.0104	-8	6	13	FFVGGNWK	51	99.98	(N-term)_iTRAQ[0], Lysine(K)_iTRAQ[8]	[3] F6 and F9	327/319	1.380	1.380	1.394	1	Mascot
1414.7413	1414.7245	-0.0168	-12	59	68	IAVAAQNCYK	75	100	(N-term)_iTRAQ[0], Lysine(K)_iTRAQ[10], MMTS (C)[8]	[7] F5 120912	201/193	0.910	1.158	0.990	1	Mascot
1502.6306	1502.5817	-0.0489	-33	194	205	CNVSEGVAQCTR	57	99.995	(N-term)_iTRAQ[0], MMTS (C)[1,10]	[1] F3 030912	265/257	0.924	1.041	0.994	1	Mascot
1514.8855	1514.9347	0.0492	32	5	13	KFFVGGNWK	61	99.998	(N-term)_iTRAQ[0], Lysine(K)_iTRAQ[1,9]	[4] F7 and F10+11	1174/1166	1.192	1.304	1.257	1	Mascot
1603.8414	1603.7448	-0.0966	-60	206	218	IIYGGSVTGATCK	86	100	(N-term)_iTRAQ[0], Lysine(K)_iTRAQ[13], MMTS (C)[12]	[5] F4	188/180	0.967	0.977	1.270	1	Mascot
1683.8938	1683.8719	-0.0219	-13	85	98	DLGATWVVLGHSER	91	100	(N-term)_iTRAQ[0]	[4] F7 and F10+11	334/326	0.866	0.938	1.028	1	Mascot
1683.8938	1683.9238	0.03	18	85	98	DLGATWVVLGHSER	48	99.962	(N-term)_iTRAQ[0]	[3] F6 and F9	330/322	1.699	1.161	1.893	1	Mascot
1740.913	1740.9803	0.0673	39	149	159	AIADNVKDWCK	63	99.999	(N-term)_iTRAQ[0], Lysine(K)_iTRAQ[7,11], MMTS (C)[10]	[4] F7 and F10+11	1213/1205	0.841	0.570	1.419	1	Mascot
1754.9283	1754.7814	-0.1469	-84	175	187	TATPQQAQEVHEK	59	99.997	(N-term)_iTRAQ[0], Lysine(K)_iTRAQ[13]	[6] F8 110912	89/81	1.510	1.958	0.960	1	Mascot
1760.9429	1760.8596	-0.0833	-47	100	112	HIFGESDELIGQK	93	100	(N-term)_iTRAQ[0], Lysine(K)_iTRAQ[13]	[6] F8 110912	269/261	0.962	0.947	1.363	1	Mascot
1760.9429	1761.036	0.0931	53	100	112	HIFGESDELIGQK	51	99.981	(N-term)_iTRAQ[0], Lysine(K)_iTRAQ[13]	[3] F6 and F9	1225/1217	1.312	1.010	1.623	1	Mascot
1891.0939	1891.0287	-0.0652	-34	160	174	VVLAYEPVWAIGTGK	80	100	(N-term)_iTRAQ[0], Lysine(K)_iTRAQ[15]	[1] F3 030912	415/407	1.078	1.031	1.184	1	Mascot
1910.0303	1909.9316	-0.0987	-52	69	84	VTNGAFTGEISPGMIK	71	100	(N-term)_iTRAQ[0], Lysine(K)_iTRAQ[16]	[1] F3 030912	316/308	0.871	0.901	0.883	1	Mascot
2144.1433	2144.1838	0.0405	19	113	130	VNHALSEGLGVIACIGEK	132	100	(N-term)_iTRAQ[0], Lysine(K)_iTRAQ[18], MMTS (C)[14]	[4] F7 and F10+11	467/459	0.853	0.792	0.864	1	Mascot

36	NF-M protein [Rattus norvegicus]	gi|205688	110250.9	16	881	1.895	0.939	0.827	0.531	0.255	0.239	19	19	19	100

Protein Group

RecName: Full=Neurofilament medium polypeptide; Short=NF-M; AltName: Full=160 kDa neurofilament pro	gi|128150	110379.9
neurofilament medium polypeptide [Rattus norvegicus]	gi|8393823	110237.8

Peptide Information

Calc. Mass	Obsrv. Mass	± da	± ppm	Start Seq.	End Seq.	Sequence	Ion Score	C. I. %	Modification	Plate [#] Name	Gel Idx/Pos [4700 Sample Name]	iTRAQ Ratio 115/114*	iTRAQ Ratio 116/114*	iTRAQ Ratio 117/114*	Rank	Result Type
1115.6346	1115.5829	-0.0517	-46	111	117	FAGYIEK	28	95.669	(N-term)_iTRAQ[0], Lysine(K)_iTRAQ[7]	[3] F6 and F9	230/222	2.147	1.532	1.464	1	Mascot
1115.6346	1115.5948	-0.0398	-36	111	117	FAGYIEK	45	99.917	(N-term)_iTRAQ[0], Lysine(K)_iTRAQ[7]	[7] F5 120912	191/183	1.825	0.790	0.606	1	Mascot
1303.709	1303.7314	0.0224	17	194	203	LRDDTEAAIR	42	99.858	(N-term)_iTRAQ[0]	[3] F6 and F9	1125/1117	2.232	1.166	0.889	1	Mascot
1324.7345	1324.7064	-0.0281	-21	127	136	EIEAEIHALR	50	99.978	(N-term)_iTRAQ[0]	[4] F7 and F10+11	274/266	1.507	0.715	0.444	1	Mascot
1409.7886	1409.7469	-0.0417	-30	382	390	EYQDLLNVK	60	99.997	(N-term)_iTRAQ[0], Lysine(K)_iTRAQ[9]	[7] F5 120912	243/235	2.284	1.143	1.029	1	Mascot
1422.8124	1422.7605	-0.0519	-36	672	681	AVEEMITITK	33	98.824	(N-term)_iTRAQ[0], Lysine(K)_iTRAQ[10]	[5] F4	211/203	1.787	0.845	0.963	1	Mascot
1459.7825	1459.8003	0.0178	12	158	167	ATLEMVNHEK	62	99.998	(N-term)_iTRAQ[0], Lysine(K)_iTRAQ[10]	[3] F6 and F9	1136/1128	1.999	1.035	0.845	1	Mascot
1548.8506	1548.806	-0.0446	-29	223	234	VQSLQDEVAFLR	58	99.996	(N-term)_iTRAQ[0]	[1] F3 030912	357/349	1.809	0.679	0.852	1	Mascot
1693.8167	1693.8936	0.0769	45	812	822	VEEHEETFEEK	72	100	(N-term)_iTRAQ[0], Lysine(K)_iTRAQ[11]	[3] F6 and F9	1116/1108	1.795	0.848	0.653	1	Mascot
1750.9996	1751.0458	0.0462	26	584	594	VEEMAIKEEIK	27	95.163	(N-term)_iTRAQ[0], Lysine(K)_iTRAQ[7,11]	[4] F7 and F10+11	1151/1143	2.366	1.143	1.155	1	Mascot
1805.0881	1805.0105	-0.0776	-43	591	600	EEIKVEKPEK	33	98.677	(N-term)_iTRAQ[0], Lysine(K)_iTRAQ[4,7,10]	[2] F12 040912	158/150	1.973	1.017	0.745	1	Mascot
1818.9556	1819.0363	0.0807	44	98	110	SNEKEQLQGLNDR	55	99.992	(N-term)_iTRAQ[0], Lysine(K)_iTRAQ[4]	[4] F7 and F10+11	1051/1043	2.490	1.375	0.853	1	Mascot
1818.9556	1819.0453	0.0897	49	98	110	SNEKEQLQGLNDR	46	99.938	(N-term)_iTRAQ[0], Lysine(K)_iTRAQ[4]	[3] F6 and F9	1120/1112	1.668	0.656	0.773	1	Mascot
1945.9489	1945.8771	-0.0718	-37	139	154	QASHAQLGDAYDQEIR	30	97.536	(N-term)_iTRAQ[0]	[3] F6 and F9	195/187	1.251	0.988	0.884	1	Mascot
1945.9489	1945.9336	-0.0153	-8	139	154	QASHAQLGDAYDQEIR	103	100	(N-term)_iTRAQ[0]	[4] F7 and F10+11	191/183	2.066	0.911	0.909	1	Mascot
2014.2046	2014.2894	0.0848	42	608	620	SPVEEVKPKPEAK	55	99.992	(N-term)_iTRAQ[0], Lysine(K)_iTRAQ[7,9,13]	[4] F7 and F10+11	1042/1034	2.274	0.874	0.886	1	Mascot
2553.2175	2553.064	-0.1535	-60	706	725	AEEEGGSEEEVGDKSPQESK	76	100	(N-term)_iTRAQ[0], Lysine(K)_iTRAQ[14,20]	[6] F8 110912	112/104	2.719	0.908	0.575	1	Mascot
2584.2512	2584.3367	0.0855	33	351	370	HNHDLSSYQDTIQQLENELR	59	99.997	(N-term)_iTRAQ[0]	[4] F7 and F10+11	1302/1294	0.913	1.122	0.883	1	Mascot
2681.1772	2681.2708	0.0936	35	274	292	SQLECHSDQNMHQAEEWFK	56	99.994	(N-term)_iTRAQ[0], Lysine(K)_iTRAQ[19], MMTS (C)[5]	[4] F7 and F10+11	1205/1197	1.904	0.622	0.817	1	Mascot

37	14-3-3 protein epsilon [Rattus norvegicus]	gi|13928824	31691	14	880	1.144	1.024	0.912	0.302	0.322	0.277	16	16	16	100

Protein Group

14-3-3 protein epsilon [Homo sapiens]	gi|5803225	32031.3

Peptide Information

Calc. Mass	Obsrv. Mass	± da	± ppm	Start Seq.	End Seq.	Sequence	Ion Score	C. I. %	Modification	Plate [#] Name	Gel Idx/Pos [4700 Sample Name]	iTRAQ Ratio 115/114*	iTRAQ Ratio 116/114*	iTRAQ Ratio 117/114*	Rank	Result Type
1195.7296	1195.6782	-0.0514	-43	43	50	NLLSVAYK	53	99.987	(N-term)_iTRAQ[0], Lysine(K)_iTRAQ[8]	[7] F5 120912	233/225	1.100	1.296	1.119	1	Mascot
1205.7351	1205.6794	-0.0557	-46	62	69	IISSIEQK	44	99.911	(N-term)_iTRAQ[0], Lysine(K)_iTRAQ[8]	[7] F5 120912	188/180	0.966	0.904	0.857	1	Mascot
1265.7021	1265.6588	-0.0433	-34	87	94	QMVETELK	39	99.711	(N-term)_iTRAQ[0], Lysine(K)_iTRAQ[8]	[7] F5 120912	152/144	0.997	0.933	0.888	1	Mascot
1333.7634	1333.7305	-0.0329	-25	216	225	DSTLIMQLLR	42	99.831	(N-term)_iTRAQ[0]	[1] F3 030912	466/458	1.966	2.108	0.925	1	Mascot
1333.7634	1333.7313	-0.0321	-24	216	225	DSTLIMQLLR	48	99.963	(N-term)_iTRAQ[0]	[2] F12 040912	472/464	1.159	0.774	0.827	1	Mascot
1400.693	1400.6388	-0.0542	-39	131	141	YLAEFATGNDR	50	99.976	(N-term)_iTRAQ[0]	[1] F3 030912	284/276	1.161	0.948	0.744	1	Mascot
1419.6746	1419.6129	-0.0617	-43	20	28	YDEMVESMK	58	99.996	(N-term)_iTRAQ[0], Lysine(K)_iTRAQ[9]	[7] F5 120912	219/211	1.396	1.279	1.223	1	Mascot
1482.8049	1482.693	-0.1119	-75	143	153	EAAENSLVAYK	78	100	(N-term)_iTRAQ[0], Lysine(K)_iTRAQ[11]	[5] F4	133/125	0.624	0.654	0.531	1	Mascot
1482.8049	1482.7451	-0.0598	-40	143	153	EAAENSLVAYK	58	99.997	(N-term)_iTRAQ[0], Lysine(K)_iTRAQ[11]	[7] F5 120912	170/162	1.219	0.976	1.109	1	Mascot
1525.8584	1525.9324	0.074	48	107	118	HLIPAANTGESK	81	100	(N-term)_iTRAQ[0], Lysine(K)_iTRAQ[12]	[3] F6 and F9	1118/1110	1.173	1.302	1.247	1	Mascot
1672.8904	1672.9536	0.0632	38	131	142	YLAEFATGNDRK	54	99.991	(N-term)_iTRAQ[0], Lysine(K)_iTRAQ[12]	[3] F6 and F9	1227/1219	0.988	0.834	1.056	1	Mascot

Calc. Mass	Obsrv. Mass	± da	± ppm	Start Seq.	End Seq.	Sequence	Ion Score	C. I. %	Modification	Plate [#] Name	Gel Idx/Pos [4700 Sample Name]	iTRAQ Ratio 115/114*	iTRAQ Ratio 116/114*	iTRAQ Ratio 117/114*	Rank	Result Type
1742.8772	1742.8219	-0.0553	-32	95	106	LICCDILDVLDK	51	99.981	(N-term)_iTRAQ[0], Lysine(K)_iTRAQ[12], MMTS (C)[3,4]	[5] F4	447/439	1.190	0.813	0.913	1	Mascot
1964.0396	1963.9717	-0.0679	-35	154	170	AASDIAMTELPPTHPIR	35	99.179	(N-term)_iTRAQ[0]	[7] F5 120912	236/228	1.487	1.199	0.501	1	Mascot
2217.0776	2217.0642	-0.0134	-6	13	28	LAEQAERYDEMVESMK	62	99.999	(N-term)_iTRAQ[0], Lysine(K)_iTRAQ[16]	[4] F7 and F10+11	373/365	1.044	0.796	1.305	1	Mascot
2376.1704	2376.1084	-0.062	-26	197	215	AAFDDAIAELDTLSEESY K	134	100	(N-term)_iTRAQ[0], Lysine(K)_iTRAQ[19]	[1] F3 030912	506/498	1.040	1.109	0.970	1	Mascot
2469.145	2469.0513	-0.0937	-38	226	244	DNLTLWTSDMQGDGEE QNK	91	100	(N-term)_iTRAQ[0], Lysine(K)_iTRAQ[19]	[1] F3 030912	302/294	1.301	1.129	0.854	1	Mascot

38	hexokinase-1 [Rattus norvegicus]	gi|6981022	112674.5	16	875	0.894	1.116	1.070	0.236	0.325	0.400	17	17	17	100

Peptide Information

Calc. Mass	Obsrv. Mass	± da	± ppm	Start Seq.	End Seq.	Sequence	Ion Score	C. I. %	Modification	Plate [#] Name	Gel Idx/Pos [4700 Sample Name]	iTRAQ Ratio 115/114*	iTRAQ Ratio 116/114*	iTRAQ Ratio 117/114*	Rank	Result Type
993.5675	993.5259	-0.0416	-42	511	517	MLPSFVR	29	97.191	(N-term)_iTRAQ[0]	[5] F4	215/207	0.802	1.301	1.156	1	Mascot
1064.5861	1064.5165	-0.0696	-65	316	323	EGLLFEGR	30	97.479	(N-term)_iTRAQ[0]	[5] F4	175/167	0.845	1.063	0.553	1	Mascot
1253.6796	1253.6191	-0.0605	-48	452	462	GAAMVTAVAYR	62	99.998	(N-term)_iTRAQ[0]	[1] F3 030912	221/213	1.115	1.258	1.318	1	Mascot
1427.7992	1427.6931	-0.1061	-74	408	418	TTVGVDGSLYK	68	100	(N-term)_iTRAQ[0], Lysine(K)_iTRAQ[11]	[5] F4	130/122	0.611	0.635	0.775	1	Mascot
1427.7992	1427.7266	-0.0726	-51	408	418	TTVGVDGSLYK	41	99.822	(N-term)_iTRAQ[0], Lysine(K)_iTRAQ[11]	[7] F5 120912	165/157	0.961	1.175	0.789	1	Mascot
1498.7999	1498.7786	-0.0213	-14	728	738	VVDEYSLNSGK	76	100	(N-term)_iTRAQ[0], Lysine(K)_iTRAQ[11]	[7] F5 120912	163/155	0.697	1.165	1.023	1	Mascot
1512.8038	1512.7576	-0.0462	-31	744	755	MISGMYLGEIVR	53	99.989	(N-term)_iTRAQ[0]	[1] F3 030912	394/386	0.700	1.084	0.972	1	Mascot
1516.7446	1516.6997	-0.0449	-30	296	307	MVSGMYMGELVR	33	98.81	(N-term)_iTRAQ[0]	[1] F3 030912	337/329	1.226	2.155	2.730	1	Mascot
1544.902	1544.8665	-0.0355	-23	31	42	LSDEILIDILTR	57	99.995	(N-term)_iTRAQ[0]	[1] F3 030912	541/533	1.066	0.803	1.041	1	Mascot
1560.7808	1560.7482	-0.0326	-21	148	159	LPVGFTFSFPCR	33	98.869	(N-term)_iTRAQ[0], MMTS (C)[11]	[1] F3 030912	512/504	0.859	0.889	1.009	1	Mascot
1598.8298	1598.7808	-0.049	-31	78	91	GDFIALDLGGSSFR	63	99.999	(N-term)_iTRAQ[0]	[1] F3 030912	395/387	1.044	1.033	1.152	1	Mascot
1627.9615	1627.9412	-0.0203	-12	382	396	SANLVAATLGAILNR	43	99.88	(N-term)_iTRAQ[0]	[8] F13-15 and F1+2	255/247	1.404	1.354	1.466	1	Mascot
1676.7837	1676.7498	-0.0339	-20	625	638	ATDCEGHDVASLLR	47	99.951	(N-term)_iTRAQ[0], MMTS (C)[4]	[4] F7 and F10+11	292/284	0.808	0.983	1.075	1	Mascot
1752.9037	1752.8633	-0.0404	-23	830	844	AAQLCGAGMAAVVEK	87	100	(N-term)_iTRAQ[0], Lysine(K)_iTRAQ[15], MMTS (C)[5]	[5] F4	266/258	0.903	1.086	1.161	1	Mascot
1828.0188	1828.1134	0.0946	52	332	344	GKFNTSDVSAIEK	47	99.952	(N-term)_iTRAQ[0], Lysine(K)_iTRAQ[2,1 3]	[4] F7 and F10+11	1104/1096	1.061	1.503	1.091	1	Mascot
1832.9781	1832.9187	-0.0594	-32	147	159	KLPVGFTFSFPCR	46	99.94	(N-term)_iTRAQ[0], Lysine(K)_iTRAQ[1], MMTS (C)[12]	[6] F8 110912	469/461	0.947	1.040	1.019	1	Mascot
2365.2698	2365.2148	-0.055	-23	802	820	AILQQLGLNSTCDDSILVK	103	100	(N-term)_iTRAQ[0], Lysine(K)_iTRAQ[19], MMTS (C)[12]	[1] F3 030912	468/460	0.574	1.090	0.912	1	Mascot

39	L-lactate dehydrogenase A chain [Rattus norvegicus]	gi|8393706	40836.2	14	875	0.803	0.951	0.872	0.298	0.172	0.173	14	14	14	100

Peptide Information

Calc. Mass	Obsrv. Mass	± da	± ppm	Start Seq.	End Seq.	Sequence	Ion Score	C. I. %	Modification	Plate [#] Name	Gel Idx/Pos [4700 Sample Name]	iTRAQ Ratio 115/114*	iTRAQ Ratio 116/114*	iTRAQ Ratio 117/114*	Rank	Result Type
1057.6854	1057.6223	-0.0631	-60	91	99	LVIITAGAR	29	97.178	(N-term)_iTRAQ[0]	[1] F3 030912	247/239	0.650	1.294	1.053	1	Mascot
1217.7867	1217.7258	-0.0609	-50	119	126	FIIPNVVK	33	98.821	(N-term)_iTRAQ[0], Lysine(K)_iTRAQ[8]	[5] F4	247/239	0.349	0.898	0.850	1	Mascot
1274.6713	1274.6035	-0.0678	-53	306	315	VTLTPDEEAR	40	99.771	(N-term)_iTRAQ[0]	[1] F3 030912	173/165	1.165	0.921	0.681	1	Mascot
1313.7861	1313.8059	0.0198	15	270	278	VHPISTMIK	64	99.999	(N-term)_iTRAQ[0], Lysine(K)_iTRAQ[9]	[3] F6 and F9	1181/1173	0.996	1.153	1.144	1	Mascot
1343.8508	1343.7736	-0.0772	-57	6	14	DQLIVNLLK	54	99.991	(N-term)_iTRAQ[0], Lysine(K)_iTRAQ[9]	[5] F4	295/287	1.301	0.871	0.833	1	Mascot
1406.7889	1406.7416	-0.0473	-34	319	328	SADTLWGIQK	69	100	(N-term)_iTRAQ[0], Lysine(K)_iTRAQ[10]	[7] F5 120912	236/228	0.809	0.929	0.882	1	Mascot
1538.8676	1538.772	-0.0956	-62	233	243	QVVDSAYEVIK	70	100	(N-term)_iTRAQ[0], Lysine(K)_iTRAQ[11]	[5] F4	179/171	0.923	1.084	1.152	1	Mascot
1946.058	1945.9949	-0.0631	-32	43	57	DLADELALVDVIEDK	81	100	(N-term)_iTRAQ[0], Lysine(K)_iTRAQ[15]	[1] F3 030912	491/483	0.907	0.872	0.946	1	Mascot
2232.3254	2232.2744	-0.051	-23	133	149	LLIVSNPVDILTYVAWK	83	100	(N-term)_iTRAQ[0], Lysine(K)_iTRAQ[17]	[1] F3 030912	570/562	0.606	0.916	0.821	1	Mascot
2262.2102	2262.082	-0.1282	-57	213	228	SLNPQLGTDADKEQWK	105	100	(N-term)_iTRAQ[0], Lysine(K)_iTRAQ[12, 16]	[6] F8 110912	244/236	0.736	0.872	0.773	1	Mascot
2279.2627	2279.4395	0.1768	78	58	73	LKGEMMDLQHGSLFLK	59	99.997	(N-term)_iTRAQ[0], Lysine(K)_iTRAQ[2,1 6]	[4] F7 and F10+11	1260/1252	0.834	0.908	1.019	1	Mascot
2364.2791	2364.3296	0.0505	21	60	76	GEMMDLQHGSLFLKTPK	42	99.861	(N-term)_iTRAQ[0], Lysine(K)_iTRAQ[14,	[4] F7 and F10+11	1210/1202	0.885	1.028	0.858	1	Mascot

17]

2400.2729	2400.207	-0.0659	-27	246	265	GYTSWAIGLSVADLAESIMK	103	100	(N-term)_iTRAQ[0], Lysine(K)_iTRAQ[20]	[1] F3 030912	583/575	0.981	1.118	0.805	1	Mascot
2440.4146	2440.3994	-0.0152	-6	6	22	DQLIVNLLKEEQVPQNK	42	99.843	(N-term)_iTRAQ[0], Lysine(K)_iTRAQ[9,17]	[4] F7 and F10+11	399/391	0.622	0.625	0.589	1	Mascot

40 14-3-3 zeta isoform [Rattus norvegicus] gi|1051270 30947.8 12 868 1.017 1.080 1.011 0.543 0.453 0.381 23 23 23 100

Protein Group

14-3-3 protein zeta/delta [Mus musculus] gi|6756041 30917.8

Peptide Information

Calc. Mass	Obsrv. Mass	± da	± ppm	Start Seq.	End Seq.	Sequence	Ion Score	C. I. %	Modification	Plate [#] Name	Gel Idx/Pos [4700 Sample Name]	iTRAQ Ratio 115/114*	iTRAQ Ratio 116/114*	iTRAQ Ratio 117/114*	Rank	Result Type
957.6019	957.6076	0.0057	6	116	120	VFYLK	34	98.922	(N-term)_iTRAQ[0], Lysine(K)_iTRAQ[5]	[3] F6 and F9	262/254	1.285	1.183	1.199	1	Mascot
1177.7039	1177.6533	-0.0506	-43	61	68	VVSSIEQK	62	99.998	(N-term)_iTRAQ[0], Lysine(K)_iTRAQ[8]	[7] F5 120912	140/132	1.111	1.025	1.055	1	Mascot
1195.7296	1195.6782	-0.0514	-43	42	49	NLLSVAYK	53	99.987	(N-term)_iTRAQ[0], Lysine(K)_iTRAQ[8]	[7] F5 120912	233/225	1.100	1.296	1.119	1	Mascot
1333.7634	1333.7305	-0.0329	-25	213	222	DSTLIMQLLR	42	99.831	(N-term)_iTRAQ[0]	[1] F3 030912	466/458	1.966	2.108	0.925	1	Mascot
1333.7634	1333.7313	-0.0321	-24	213	222	DSTLIMQLLR	48	99.963	(N-term)_iTRAQ[0]	[2] F12 040912	472/464	1.159	0.774	0.827	1	Mascot
1381.5851	1381.5438	-0.0413	-30	19	27	YDDMAACMK	56	99.993	(N-term)_iTRAQ[0], Lysine(K)_iTRAQ[9], MMTS (C)[7]	[7] F5 120912	246/238	1.102	1.146	1.237	1	Mascot
1439.7628	1439.6989	-0.0639	-44	128	138	YLAEVAAGDDK	72	100	(N-term)_iTRAQ[0], Lysine(K)_iTRAQ[11]	[5] F4	164/156	0.676	0.739	0.852	1	Mascot
1618.905	1618.806	-0.099	-61	104	115	FLIPNASQPESK	53	99.987	(N-term)_iTRAQ[0], Lysine(K)_iTRAQ[12]	[5] F4	171/163	0.843	0.931	0.987	1	Mascot
1692.816	1692.7153	-0.1007	-59	28	41	SVTEQGAELSNEER	104	100	(N-term)_iTRAQ[0]	[1] F3 030912	151/143	0.883	1.009	0.901	1	Mascot
1695.8888	1695.853	-0.0358	-21	92	103	DICNDVLSLLEK	61	99.998	(N-term)_iTRAQ[0], Lysine(K)_iTRAQ[12], MMTS (C)[3]	[5] F4	424/416	0.871	0.942	1.152	1	Mascot
1695.8888	1695.8668	-0.022	-13	92	103	DICNDVLSLLEK	29	96.834	(N-term)_iTRAQ[0], Lysine(K)_iTRAQ[12], MMTS (C)[3]	[1] F3 030912	559/551	0.400	0.500	0.454	1	Mascot
1695.8888	1695.9105	0.0217	13	92	103	DICNDVLSLLEK	44	99.901	(N-term)_iTRAQ[0], Lysine(K)_iTRAQ[12], MMTS (C)[3]	[7] F5 120912	528/520	2.497	1.953	1.366	1	Mascot
1711.9602	1711.9535	-0.0067	-4	128	139	YLAEVAAGDDKK	49	99.971	(N-term)_iTRAQ[0], Lysine(K)_iTRAQ[11, 12]	[2] F12 040912	150/142	0.689	0.853	0.809	1	Mascot
1711.9602	1712.0057	0.0455	27	128	139	YLAEVAAGDDKK	79	100	(N-term)_iTRAQ[0], Lysine(K)_iTRAQ[11, 12]	[3] F6 and F9	1190/1182	0.686	0.784	0.835	1	Mascot
1711.9602	1712.0275	0.0673	39	128	139	YLAEVAAGDDKK	92	100	(N-term)_iTRAQ[0], Lysine(K)_iTRAQ[11, 12]	[4] F7 and F10+11	1119/1111	0.856	0.920	0.959	1	Mascot
1711.9602	1712.0406	0.0804	47	128	139	YLAEVAAGDDKK	40	99.764	(N-term)_iTRAQ[0], Lysine(K)_iTRAQ[11, 12]	[4] F7 and F10+11	1381/1373	0.774	0.872	0.944	1	Mascot
2329.1921	2329.0911	-0.101	-43	140	157	GIVDQSQQAYQEAFEISK	71	100	(N-term)_iTRAQ[0], Lysine(K)_iTRAQ[18]	[1] F3 030912	319/311	1.928	1.650	1.541	1	Mascot
2329.1921	2329.1199	-0.0722	-31	140	157	GIVDQSQQAYQEAFEISK	104	100	(N-term)_iTRAQ[0], Lysine(K)_iTRAQ[18]	[1] F3 030912	337/329	0.959	0.965	0.761	1	Mascot
2329.1921	2329.1443	-0.0478	-21	140	157	GIVDQSQQAYQEAFEISK	64	99.999	(N-term)_iTRAQ[0], Lysine(K)_iTRAQ[18]	[2] F12 040912	369/361	2.148	1.995	2.536	1	Mascot
2420.1965	2420.1021	-0.0944	-39	194	212	TAFDEAIAELDTLSEESYK	35	99.15	(N-term)_iTRAQ[0], Lysine(K)_iTRAQ[19]	[5] F4	410/402	1.076	1.109	1.139	1	Mascot
2420.1965	2420.1379	-0.0586	-24	194	212	TAFDEAIAELDTLSEESYK	130	100	(N-term)_iTRAQ[0], Lysine(K)_iTRAQ[19]	[1] F3 030912	539/531	1.143	1.080	1.324	1	Mascot
2420.1965	2420.165	-0.0315	-13	194	212	TAFDEAIAELDTLSEESYK	76	100	(N-term)_iTRAQ[0], Lysine(K)_iTRAQ[19]	[1] F3 030912	581/573	0.557	0.872	0.935	1	Mascot
2420.1965	2420.1733	-0.0232	-10	194	212	TAFDEAIAELDTLSEESYK	29	97.217	(N-term)_iTRAQ[0], Lysine(K)_iTRAQ[19]	[7] F5 120912	508/500	0.993	1.760	0.699	1	Mascot

41 dynamin-1 [Rattus norvegicus] gi|18093102 104068.8 18 868 0.910 1.096 1.029 0.284 0.314 0.385 20 20 20 100

Peptide Information

Calc. Mass	Obsrv. Mass	± da	± ppm	Start Seq.	End Seq.	Sequence	Ion Score	C. I. %	Modification	Plate [#] Name	Gel Idx/Pos [4700 Sample Name]	iTRAQ Ratio 115/114*	iTRAQ Ratio 116/114*	iTRAQ Ratio 117/114*	Rank	Result Type
1167.7024	1167.6805	-0.0219	-19	370	376	FPFELVK	33	98.701	(N-term)_iTRAQ[0], Lysine(K)_iTRAQ[7]	[7] F5 120912	356/348	0.702	0.980	0.793	1	Mascot
1174.6552	1174.5952	-0.06	-51	247	256	DITAALAAER	51	99.978	(N-term)_iTRAQ[0]	[1] F3 030912	227/219	1.490	2.381	2.192	1	Mascot
1186.5864	1186.5079	-0.0785	-66	91	98	FTDFEEVR	34	99.03	(N-term)_iTRAQ[0]	[5] F4	180/172	0.675	0.904	0.649	1	Mascot
1209.7439	1209.7145	-0.0294	-24	114	123	GISPVPINLR	37	99.47	(N-term)_iTRAQ[0]	[1] F3 030912	278/270	0.975	1.423	1.950	1	Mascot
1225.686	1225.6469	-0.0391	-32	272	279	MGTPYLQK	28	96.414	(N-term)_iTRAQ[0], Lysine(K)_iTRAQ[8]	[3] F6 and F9	205/197	1.036	1.101	0.899	1	Mascot

Calc. Mass	Obsrv. Mass	± da	± ppm	Start Seq.	End Seq.	Sequence	Ion Score	C. I. %	Modification	Plate [#] Name	Gel Idx/Pos [4700 Sample Name]	iTRAQ Ratio 115/114*	iTRAQ Ratio 116/114*	iTRAQ Ratio 117/114*	Rank	Result Type
1251.6818	1251.6278	-0.054	-43	45	54	SSVLENFVGR	47	99.954	(N-term)_iTRAQ[0]	[5] F4	218/210	1.032	1.015	1.163	1	Mascot
1370.76	1370.6903	-0.0697	-51	563	571	YMLSVDNLK	58	99.996	(N-term)_iTRAQ[0], Lysine(K)_iTRAQ[9]	[5] F4	219/211	1.391	1.431	1.469	1	Mascot
1379.6597	1379.5951	-0.0646	-47	207	217	LDLMDEGTDAR	41	99.787	(N-term)_iTRAQ[0]	[1] F3 030912	232/224	0.735	1.142	0.629	1	Mascot
1400.7528	1400.6843	-0.0685	-49	158	166	DMLMQFVTK	56	99.994	(N-term)_iTRAQ[0], Lysine(K)_iTRAQ[9]	[5] F4	280/272	1.176	1.050	1.051	1	Mascot
1400.7692	1400.741	-0.0282	-20	5	15	GMEDLIPLVNR	30	97.305	(N-term)_iTRAQ[0]	[1] F3 030912	363/355	0.883	0.810	0.929	1	Mascot
1458.7838	1458.8536	0.0698	48	90	98	KFTDFEEVR	40	99.747	(N-term)_iTRAQ[0], Lysine(K)_iTRAQ[1]	[4] F7 and F10+11	1131/1123	1.053	1.548	1.302	1	Mascot
1642.9574	1643.0228	0.0654	40	562	571	KYMLSVDNLK	67	100	(N-term)_iTRAQ[0], Lysine(K)_iTRAQ[1,10]	[4] F7 and F10+11	1169/1161	1.143	0.949	1.234	1	Mascot
1742.8593	1742.8373	-0.022	-13	427	440	CVDMVVSELTSTIR	30	97.502	(N-term)_iTRAQ[0], MMTS (C)[1]	[8] F13-15 and F1+2	251/243	0.531	1.019	0.742	1	Mascot
1853.9882	1853.9949	0.0067	4	143	157	VPVGDQPPDIEFQIR	28	96.512	(N-term)_iTRAQ[0]	[8] F13-15 and F1+2	438/430	0.926	0.901	0.899	1	Mascot
1898.0521	1897.9976	-0.0545	-29	400	414	TGLFTPDLAFEATVK	72	100	(N-term)_iTRAQ[0], Lysine(K)_iTRAQ[15]	[1] F3 030912	404/396	0.913	0.988	0.951	1	Mascot
1920.1476	1920.2443	0.0967	50	523	535	KGWLTINNIGIMK	67	100	(N-term)_iTRAQ[0], Lysine(K)_iTRAQ[1,13]	[4] F7 and F10+11	1263/1255	0.694	1.036	0.863	1	Mascot
2055.1194	2055.0427	-0.0767	-37	328	342	ALLQMVQQFAVDFEK	64	99.999	(N-term)_iTRAQ[0], Lysine(K)_iTRAQ[15]	[5] F4	435/427	0.835	1.228	1.215	1	Mascot
2055.1194	2055.0669	-0.0525	-26	328	342	ALLQMVQQFAVDFEK	108	100	(N-term)_iTRAQ[0], Lysine(K)_iTRAQ[15]	[1] F3 030912	574/566	0.555	0.737	0.869	1	Mascot
2071.1143	2071.0413	-0.073	-35	328	342	ALLQMVQQFAVDFEK	35	99.298	(N-term)_iTRAQ[0], Lysine(K)_iTRAQ[15], Oxidation (M)[5]	[5] F4	435/427	0.967	1.075	0.842	1	Mascot
2168.0703	2168.0359	-0.0344	-16	343	361	RIEGSGDQIDTYELSGGAR	45	99.916	(N-term)_iTRAQ[0]	[4] F7 and F10+11	228/220	1.151	1.003	1.087	1	Mascot

42	myosin-10 [Rattus norvegicus]	gi|13928704	258282.2	18	860	1.060	0.866	0.852	0.280	0.206	0.240	18	18	18	100

Peptide Information

Calc. Mass	Obsrv. Mass	± da	± ppm	Start Seq.	End Seq.	Sequence	Ion Score	C. I. %	Modification	Plate [#] Name	Gel Idx/Pos [4700 Sample Name]	iTRAQ Ratio 115/114*	iTRAQ Ratio 116/114*	iTRAQ Ratio 117/114*	Rank	Result Type
1180.6282	1180.604	-0.0242	-20	588	594	ADEWLMK	28	96.193	(N-term)_iTRAQ[0], Lysine(K)_iTRAQ[7]	[3] F6 and F9	269/261	1.158	1.092	0.788	1	Mascot
1462.8502	1462.8566	0.0064	4	690	700	LDPHLVLDQLR	39	99.693	(N-term)_iTRAQ[0]	[4] F7 and F10+11	364/356	0.987	1.004	0.817	1	Mascot
1531.8689	1531.9469	0.078	51	1088	1098	KEEELQGALAR	33	98.649	(N-term)_iTRAQ[0], Lysine(K)_iTRAQ[1]	[4] F7 and F10+11	1080/1072	0.681	0.588	0.790	1	Mascot
1571.7941	1571.8258	0.0317	20	573	583	ADFCIIHYAGK	54	99.99	(N-term)_iTRAQ[0], Lysine(K)_iTRAQ[11], MMTS (C)[4]	[3] F6 and F9	1292/1284	0.869	0.772	0.591	1	Mascot
1652.9595	1653.0321	0.0726	44	1452	1461	KFDQLLAEEK	53	99.987	(N-term)_iTRAQ[0], Lysine(K)_iTRAQ[1,10]	[4] F7 and F10+11	1163/1155	1.200	1.089	0.894	1	Mascot
1663.7896	1663.6987	-0.0909	-55	1906	1919	ELDDATEANEGLSR	58	99.996	(N-term)_iTRAQ[0]	[1] F3 030912	180/172	1.535	0.563	0.464	1	Mascot
1812.0715	1812.0048	-0.0667	-37	1256	1267	KLDAQVQELHAK	47	99.947	(N-term)_iTRAQ[0], Lysine(K)_iTRAQ[1,12]	[2] F12 040912	204/196	1.186	0.832	1.183	1	Mascot
1813.0469	1812.8848	-0.1621	-89	349	362	VVSSVLQFGNISFK	27	95.174	(N-term)_iTRAQ[0], Lysine(K)_iTRAQ[14]	[5] F4	340/332	1.388	1.330	1.117	1	Mascot
1921.9535	1921.8571	-0.0964	-50	366	380	NTDQASMPENTVAQK	71	100	(N-term)_iTRAQ[0], Lysine(K)_iTRAQ[15]	[5] F4	88/80	0.896	0.970	1.210	1	Mascot
1937.0378	1936.9468	-0.091	-47	19	33	AVIYNPATQADWTAK	61	99.998	(N-term)_iTRAQ[0], Lysine(K)_iTRAQ[15]	[5] F4	205/197	1.431	0.880	0.906	1	Mascot
1952.0222	1952.0109	-0.0113	-6	1491	1504	ALEEALEAKEEFER	72	100	(N-term)_iTRAQ[0], Lysine(K)_iTRAQ[9]	[4] F7 and F10+11	385/377	1.174	0.878	1.001	1	Mascot
2027.1019	2027.244	0.1421	70	917	930	KQELEEILHDLESR	57	99.995	(N-term)_iTRAQ[0], Lysine(K)_iTRAQ[1]	[4] F7 and F10+11	1324/1316	1.165	0.880	0.992	1	Mascot
2134.1614	2134.0339	-0.1275	-60	850	863	QEEELQAKDEELLK	39	99.702	(N-term)_iTRAQ[0], Lysine(K)_iTRAQ[8,14]	[6] F8 110912	250/242	0.701	0.695	0.927	1	Mascot
2135.0461	2134.884	-0.1621	-76	1346	1360	NSLQEQQEEEEEARK	52	99.985	(N-term)_iTRAQ[0], Lysine(K)_iTRAQ[15]	[6] F8 110912	130/122	1.026	1.026	0.806	1	Mascot
2155.1091	2154.9712	-0.1379	-64	1227	1241	QGLETDNKELACEVK	30	97.408	(N-term)_iTRAQ[0], Lysine(K)_iTRAQ[8,15], MMTS (C)[12]	[6] F8 110912	258/250	0.825	0.790	0.936	1	Mascot
2189.1421	2189.0984	-0.0437	-20	1684	1701	SLEAEILQLQEELASSER	28	96.472	(N-term)_iTRAQ[0]	[8] F13-15 and F1+2	256/248	1.397	1.019	0.578	1	Mascot
2233.1809	2233.115	-0.0659	-30	1285	1301	LQNELDNVSTLLEEAEK	61	99.998	(N-term)_iTRAQ[0], Lysine(K)_iTRAQ[17]	[1] F3 030912	486/478	0.848	0.769	0.736	1	Mascot
2505.3784	2505.3804	0.002	1	1285	1302	LQNELDNVSTLLEEAEKK	58	99.996	(N-term)_iTRAQ[0], Lysine(K)_iTRAQ[17,18]	[4] F7 and F10+11	477/469	1.133	0.756	1.052	1	Mascot

43	unnamed protein product [Rattus norvegicus]	gi|1334284	65953.3	13	852	0.913	0.937	1.004	0.238	0.286	0.240	16	16	16	100

Protein Group

60 kDa heat shock protein, mitochondrial [Mus musculus]	gi|183396771	68981

chaperonin 60 [Rattus norvegicus]	gi|1778213	68921.9
heat shock protein (hsp60) precursor [Rattus norvegicus]	gi|56383	68991

Peptide Information

Calc. Mass	Obsrv. Mass	± da	± ppm	Start Seq.	End Seq.	Sequence	Ion Score	C. I. %	Modification	Plate [#] Name	Gel Idx/Pos [4700 Sample Name]	iTRAQ Ratio 115/114*	iTRAQ Ratio 116/114*	iTRAQ Ratio 117/114*	Rank	Result Type
1132.7188	1132.6885	-0.0303	-27	319	326	VGEVIVTK	34	99.059	(N-term)_iTRAQ[0], Lysine(K)_iTRAQ[8]	[7] F5 120912	159/151	0.794	1.297	0.883	1	Mascot
1200.7925	1200.7212	-0.0713	-59	267	275	VGLQVVAVK	34	99.126	(N-term)_iTRAQ[0], Lysine(K)_iTRAQ[9]	[5] F4	167/159	1.035	1.038	1.060	1	Mascot
1503.8628	1503.7688	-0.094	-63	456	467	NAGVEGSLIVEK	69	100	(N-term)_iTRAQ[0], Lysine(K)_iTRAQ[12]	[5] F4	152/144	1.001	0.625	1.220	1	Mascot
1521.8007	1521.7874	-0.0133	-9	380	391	VGGTSDVEVNEK	61	99.998	(N-term)_iTRAQ[0], Lysine(K)_iTRAQ[12]	[7] F5 120912	111/103	0.989	1.628	1.311	1	Mascot
1632.9207	1632.8116	-0.1091	-67	35	46	TVIIEQSWGSPK	64	99.999	(N-term)_iTRAQ[0], Lysine(K)_iTRAQ[12]	[5] F4	185/177	0.936	0.857	1.087	1	Mascot
1717.0179	1716.839	-0.1789	-104	117	130	GVMLAVDAVIAELK	30	97.69	(N-term)_iTRAQ[0], Lysine(K)_iTRAQ[14]	[1] F3 030912	575/567	1.578	1.079	0.904	1	Mascot
1717.0179	1716.9525	-0.0654	-38	117	130	GVMLAVDAVIAELK	55	99.991	(N-term)_iTRAQ[0], Lysine(K)_iTRAQ[14]	[5] F4	433/425	0.573	0.728	0.614	1	Mascot
1717.0179	1716.9891	-0.0288	-17	117	130	GVMLAVDAVIAELK	98	100	(N-term)_iTRAQ[0], Lysine(K)_iTRAQ[14]	[1] F3 030912	571/563	0.904	1.043	0.978	1	Mascot
1792.9612	1792.8893	-0.0719	-40	180	192	TLNDELEIIEGMK	58	99.996	(N-term)_iTRAQ[0], Lysine(K)_iTRAQ[13]	[5] F4	332/324	0.707	0.713	0.873	1	Mascot
1878.9208	1878.8417	-0.0791	-42	211	223	CEFQDAYVLLSEK	43	99.888	(N-term)_iTRAQ[0], Lysine(K)_iTRAQ[13], MMTS (C)[1]	[5] F4	348/340	1.112	0.981	1.308	1	Mascot
2049.1577	2049.1597	0.002	1	225	242	ISSVQSIVPALEIANAHR	39	99.7	(N-term)_iTRAQ[0]	[7] F5 120912	369/361	0.708	0.826	0.676	1	Mascot
2220.1721	2220.1658	-0.0063	-3	421	436	CIPALDSLKPANEDQK	50	99.974	(N-term)_iTRAQ[0], Lysine(K)_iTRAQ[9,16], MMTS (C)[1]	[4] F7 and F10+11	325/317	0.816	1.226	1.087	1	Mascot
2321.355	2321.4348	0.0798	34	224	242	KISSVQSIVPALEIANAHR	100	100	(N-term)_iTRAQ[0], Lysine(K)_iTRAQ[1]	[4] F7 and F10+11	1246/1238	0.878	1.018	1.183	1	Mascot
2401.3445	2401.28	-0.0645	-27	12	32	ALMLQGVDLLADAVAVTMGPK	32	98.446	(N-term)_iTRAQ[0], Lysine(K)_iTRAQ[21]	[8] F13-15 and F1+2	202/194	1.250	1.019	1.285	1	Mascot
2401.3445	2401.2939	-0.0506	-21	12	32	ALMLQGVDLLADAVAVTMGPK	143	100	(N-term)_iTRAQ[0], Lysine(K)_iTRAQ[21]	[1] F3 030912	591/583	0.831	0.899	0.998	1	Mascot
2820.4045	2820.3284	-0.0761	-27	468	490	ILQSSSEVGYDAMLGDFVNMVEK	59	99.997	(N-term)_iTRAQ[0], Lysine(K)_iTRAQ[23]	[1] F3 030912	539/531	0.910	0.558	0.955	1	Mascot

44	alpha-actinin-4 [Rattus norvegicus]	gi|77539778	113431.1	17	817	0.819	0.887	0.833	0.203	0.258	0.203	18	18	18	100

Peptide Information

Calc. Mass	Obsrv. Mass	± da	± ppm	Start Seq.	End Seq.	Sequence	Ion Score	C. I. %	Modification	Plate [#] Name	Gel Idx/Pos [4700 Sample Name]	iTRAQ Ratio 115/114*	iTRAQ Ratio 116/114*	iTRAQ Ratio 117/114*	Rank	Result Type
1307.6705	1307.6019	-0.0686	-52	167	175	EGLLLWCQR	29	97.236	(N-term)_iTRAQ[0], MMTS (C)[7]	[5] F4	340/332	0.867	1.008	0.539	1	Mascot
1359.7756	1359.7264	-0.0492	-36	301	310	LASDLLEWIR	58	99.996	(N-term)_iTRAQ[0]	[5] F4	402/394	0.825	0.958	0.928	1	Mascot
1496.7288	1496.6337	-0.0951	-64	761	771	GISQEQMQEFR	51	99.98	(N-term)_iTRAQ[0]	[5] F4	146/138	0.939	1.071	0.966	1	Mascot
1502.7849	1502.8262	0.0413	27	422	432	ASIHEAWTDGK	28	96.059	(N-term)_iTRAQ[0], Lysine(K)_iTRAQ[11]	[4] F7 and F10+11	1077/1069	0.845	1.008	0.809	1	Mascot
1516.8893	1516.8516	-0.0377	-25	84	95	LMLLLEVISGER	39	99.72	(N-term)_iTRAQ[0]	[1] F3 030912	575/567	0.938	0.971	1.122	1	Mascot
1525.7145	1525.7584	0.0439	29	55	65	TFTAWCNSHLR	59	99.997	(N-term)_iTRAQ[0], MMTS (C)[6]	[3] F6 and F9	1321/1313	0.531	0.626	0.709	1	Mascot
1530.8765	1530.8425	-0.034	-22	734	745	VGWEQLLTTIAR	63	99.999	(N-term)_iTRAQ[0]	[1] F3 030912	508/500	0.920	1.048	0.749	1	Mascot
1543.8102	1543.7512	-0.059	-38	440	450	DYETATLSDIK	36	99.394	(N-term)_iTRAQ[0], Lysine(K)_iTRAQ[11]	[5] F4	164/156	1.114	0.965	0.917	1	Mascot
1565.8083	1565.7439	-0.0644	-41	396	406	GYEEWLLNEIR	50	99.973	(N-term)_iTRAQ[0]	[5] F4	348/340	1.039	0.929	0.835	1	Mascot
1573.8669	1573.8112	-0.0557	-35	746	757	TINEVENQILTR	45	99.925	(N-term)_iTRAQ[0]	[1] F3 030912	305/297	0.714	0.414	0.613	1	Mascot
1587.874	1587.9636	0.0896	56	205	214	HRPELIEYDK	31	97.829	(N-term)_iTRAQ[0], Lysine(K)_iTRAQ[10]	[8] F13-15 and F1+2	1554/1546	1.219	1.108	1.211	1	Mascot
1694.7983	1694.8353	0.037	22	870	882	ELPPDQAEYCIAR	54	99.991	(N-term)_iTRAQ[0], MMTS (C)[10]	[8] F13-15 and F1+2	535/527	0.761	0.697	0.658	1	Mascot
1694.7983	1694.8413	0.043	25	870	882	ELPPDQAEYCIAR	54	99.991	(N-term)_iTRAQ[0], MMTS (C)[10]	[8] F13-15 and F1+2	486/478	0.676	0.789	0.813	1	Mascot
1773.9646	1774.0101	0.0455	26	182	193	NVNVQNFHISWK	35	99.276	(N-term)_iTRAQ[0], Lysine(K)_iTRAQ[12]	[3] F6 and F9	1279/1271	0.646	0.766	1.158	1	Mascot
1825.9794	1825.9082	-0.0712	-39	153	166	FAIQDISVEETSAK	85	100	(N-term)_iTRAQ[0], Lysine(K)_iTRAQ[14]	[1] F3 030912	292/284	0.703	1.212	0.911	1	Mascot
1934.8954	1934.8876	-0.0078	-4	498	512	ICDQWDNLGSLTHSR	48	99.958	(N-term)_iTRAQ[0], MMTS (C)[2]	[4] F7 and F10+11	397/389	0.557	0.736	0.627	1	Mascot
2053.031	2052.9399	-0.0911	-44	883	899	MAPYQGPDAAPGALDYK	64	99.999	(N-term)_iTRAQ[0], Lysine(K)_iTRAQ[17]	[1] F3 030912	259/251	0.810	0.976	0.862	1	Mascot
2209.1345	2209.1313	-0.0032	-1	67	83	AGTQIENIDEDFRDGLK	42	99.86	(N-term)_iTRAQ[0], Lysine(K)_iTRAQ[17]	[4] F7 and F10+11	338/330	1.005	1.161	0.926	1	Mascot

45	6-phosphofructokinase type C [Rattus norvegicus]	gi|57977273	93508.2	14	817	0.917	0.960	0.977	0.184	0.173	0.237	14	14	14	100

Peptide Information

Calc. Mass	Obsrv. Mass	± da	± ppm	Start Seq.	End Seq.	Sequence	Ion Score	C. I. %	Modification	Plate [#] Name	Gel Idx/Pos [4700 Sample Name]	iTRAQ Ratio 115/114*	iTRAQ Ratio 116/114*	iTRAQ Ratio 117/114*	Rank	Result Type
1166.713	1166.7421	0.0291	25	302	310	VTILGHVQR	40	99.771	(N-term)_iTRAQ[0]	[3] F6 and F9	1172/1164	1.209	1.149	0.938	1	Mascot
1176.6776	1176.6406	-0.037	-31	748	753	QQWWLK	31	97.898	(N-term)_iTRAQ[0], Lysine(K)_iTRAQ[6]	[4] F7 and F10+11	292/284	0.759	0.734	0.862	1	Mascot
1195.663	1195.6348	-0.0282	-24	211	219	TFVLEVMGR	32	98.334	(N-term)_iTRAQ[0]	[1] F3 030912	363/355	0.867	1.180	1.266	1	Mascot
1223.6704	1223.6475	-0.0229	-19	693	700	AMEWISAK	46	99.942	(N-term)_iTRAQ[0], Lysine(K)_iTRAQ[8]	[3] F6 and F9	276/268	0.989	1.069	1.066	1	Mascot
1460.7631	1460.7013	-0.0618	-42	386	395	SFEGNLNTYK	61	99.998	(N-term)_iTRAQ[0], Lysine(K)_iTRAQ[10]	[3] F6 and F9	216/208	0.866	1.019	0.977	1	Mascot
1507.824	1507.7795	-0.0445	-30	290	301	ELVVTNLGFDTR	34	98.915	(N-term)_iTRAQ[0]	[1] F3 030912	324/316	0.655	0.677	0.550	1	Mascot
1588.9673	1588.9799	0.0126	8	726	736	DLLFKPVAELR	44	99.912	(N-term)_iTRAQ[0], Lysine(K)_iTRAQ[5]	[4] F7 and F10+11	365/357	0.867	1.040	1.113	1	Mascot
1652.7723	1652.6735	-0.0988	-60	764	775	YEASYDMSDVGK	65	99.999	(N-term)_iTRAQ[0], Lysine(K)_iTRAQ[12]	[5] F4	137/129	0.830	0.939	0.883	1	Mascot
1835.0649	1835.1399	0.075	41	139	150	KEWSGLLEELAK	90	100	(N-term)_iTRAQ[0], Lysine(K)_iTRAQ[1,12]	[4] F7 and F10+11	1291/1283	1.010	0.909	1.348	1	Mascot
1932.0093	1932.0331	0.0238	12	26	44	AIGVLTSGGDAQGMNAAVR	29	96.969	(N-term)_iTRAQ[0]	[8] F13-15 and F1+2	499/491	1.375	1.232	1.062	1	Mascot
1954.9678	1955.0306	0.0628	32	667	683	NVLGHMQQGGAPSPFDR	63	99.999	(N-term)_iTRAQ[0]	[3] F6 and F9	262/254	0.849	0.845	1.058	1	Mascot
2056.0981	2056.0654	-0.0327	-16	194	210	IIEVVDAIMTTAQSHQR	67	100	(N-term)_iTRAQ[0]	[7] F5 120912	502/494	0.816	0.924	1.192	1	Mascot
2097.0444	2096.9856	-0.0588	-28	355	369	LPLMECVQMTQDVQK	76	100	(N-term)_iTRAQ[0], Lysine(K)_iTRAQ[15], MMTS (C)[6]	[1] F3 030912	492/484	1.065	0.877	0.808	1	Mascot
2363.2241	2363.1523	-0.0718	-30	460	480	EIGWGDVGGWTGQGGSILGTK	140	100	(N-term)_iTRAQ[0], Lysine(K)_iTRAQ[21]	[1] F3 030912	401/393	0.906	1.021	0.851	1	Mascot

46	calcium/calmodulin-dependent protein kinase type II subunit alpha [Rattus norvegicus]	gi|6978593	59295.8	15	814	0.872	1.005	1.013	0.340	0.300	0.359	16	16	16	100

Peptide Information

Calc. Mass	Obsrv. Mass	± da	± ppm	Start Seq.	End Seq.	Sequence	Ion Score	C. I. %	Modification	Plate [#] Name	Gel Idx/Pos [4700 Sample Name]	iTRAQ Ratio 115/114*	iTRAQ Ratio 116/114*	iTRAQ Ratio 117/114*	Rank	Result Type
1080.6664	1080.6154	-0.051	-47	221	226	LYQQIK	33	98.745	(N-term)_iTRAQ[0], Lysine(K)_iTRAQ[6]	[3] F6 and F9	190/182	1.043	1.183	1.117	1	Mascot
1104.6875	1104.6415	-0.046	-42	260	267	ITAAEALK	36	99.397	(N-term)_iTRAQ[0], Lysine(K)_iTRAQ[8]	[7] F5 120912	170/162	0.540	0.854	0.725	1	Mascot
1266.698	1266.7488	0.0508	40	462	469	WQIVHFHR	40	99.779	(N-term)_iTRAQ[0]	[8] F13-15 and F1+2	1151/1143	0.545	0.636	0.672	1	Mascot
1269.641	1269.5786	-0.0624	-49	284	291	QETVDCLK	49	99.972	(N-term)_iTRAQ[0], Lysine(K)_iTRAQ[8], MMTS (C)[6]	[7] F5 120912	174/166	1.121	1.135	1.229	1	Mascot
1291.7528	1291.7052	-0.0476	-37	301	311	GAILTTMLATR	39	99.692	(N-term)_iTRAQ[0]	[1] F3 030912	340/332	1.080	1.221	0.835	1	Mascot
1337.7675	1337.7218	-0.0457	-34	33	42	VLAGQEYAAK	63	99.999	(N-term)_iTRAQ[0], Lysine(K)_iTRAQ[10]	[7] F5 120912	149/141	0.893	1.141	1.183	1	Mascot
1405.7025	1405.6403	-0.0622	-44	397	405	FYFENLWSR	53	99.988	(N-term)_iTRAQ[0]	[5] F4	343/335	0.969	0.979	1.083	1	Mascot
1447.8029	1447.7478	-0.0551	-38	434	445	ITQYLDAGGIPR	53	99.988	(N-term)_iTRAQ[0]	[1] F3 030912	315/307	0.778	0.892	1.037	1	Mascot
1447.8029	1447.7965	-0.0064	-4	434	445	ITQYLDAGGIPR	43	99.874	(N-term)_iTRAQ[0]	[4] F7 and F10+11	223/215	0.588	0.765	0.648	1	Mascot
1541.8384	1541.9143	0.0759	49	284	292	QETVDCLKK	33	98.745	(N-term)_iTRAQ[0], Lysine(K)_iTRAQ[8,9], MMTS (C)[6]	[4] F7 and F10+11	1090/1082	1.407	1.208	1.100	1	Mascot
1773.0857	1772.9882	-0.0975	-55	135	146	DLKPENLLLASK	49	99.967	(N-term)_iTRAQ[0], Lysine(K)_iTRAQ[3,12]	[6] F8 110912	295/287	1.190	1.424	1.239	1	Mascot
1920.984	1920.8787	-0.1053	-55	9	21	FTEEYQLFEELGK	68	100	(N-term)_iTRAQ[0], Lysine(K)_iTRAQ[13]	[5] F4	340/332	0.906	1.194	1.150	1	Mascot
2329.1252	2329.0745	-0.0507	-22	329	346	ESSESTNTTIEDEDTKVR	67	100	(N-term)_iTRAQ[0], Lysine(K)_iTRAQ[16]	[4] F7 and F10+11	151/143	1.136	0.856	1.976	1	Mascot
2369.1548	2369.0718	-0.083	-35	227	245	AGAYDFPSPEWDTVTPEAK	68	100	(N-term)_iTRAQ[0], Lysine(K)_iTRAQ[19]	[1] F3 030912	337/329	1.184	1.355	0.954	1	Mascot
2432.2441	2432.1926	-0.0515	-21	353	371	VTEQLIEAISNGDFESYTK	54	99.99	(N-term)_iTRAQ[0], Lysine(K)_iTRAQ[19]	[1] F3 030912	483/475	0.438	0.559	1.416	1	Mascot
2939.3301	2939.2559	-0.0742	-25	372	396	MCDPGMTAFEPEALGNLVEGLDFHR	107	100	(N-term)_iTRAQ[0], MMTS (C)[2]	[1] F3 030912	543/535	0.853	1.194	0.611	1	Mascot

47	alpha-internexin [Rattus norvegicus]	gi|9506811	59966.3	16	808	1.870	0.990	0.895	0.538	0.510	0.412	18	18	18	100

Peptide Information

Calc. Mass	Obsrv. Mass	± da	± ppm	Start Seq.	End Seq.	Sequence	Ion Score	C. I. %	Modification	Plate [#] Name	Gel Idx/Pos [4700 Sample Name]	iTRAQ Ratio 115/114*	iTRAQ Ratio 116/114*	iTRAQ Ratio 117/114*	Rank	Result Type

Calc. Mass	Obsrv. Mass	± da	± ppm	Start Seq.	End Seq.	Sequence	Ion Score	C. I. %	Modification	Plate [#] Name	Gel Idx/Pos [4700 Sample Name]	iTRAQ Ratio 115/114*	iTRAQ Ratio 116/114*	iTRAQ Ratio 117/114*	Rank	Result Type
1074.6279	1074.5662	-0.0617	-57	323	330	TIEIEGLR	30	97.799	(N-term)_iTRAQ[0]	[1] F3 030912	240/232	1.822	1.497	2.793	1	Mascot
1141.6866	1141.6459	-0.0407	-36	105	111	FAVFIEK	30	97.779	(N-term)_iTRAQ[0], Lysine(K)_iTRAQ[7]	[7] F5 120912	289/281	1.601	0.847	1.043	1	Mascot
1173.6599	1173.6062	-0.0537	-46	339	346	QILELEER	34	99.124	(N-term)_iTRAQ[0]	[5] F4	160/152	2.049	1.255	0.705	1	Mascot
1200.7072	1200.6694	-0.0378	-31	121	130	ALEAELAALR	48	99.96	(N-term)_iTRAQ[0]	[1] F3 030912	351/343	2.928	1.147	0.588	1	Mascot
1268.7096	1268.6669	-0.0427	-34	270	278	AQYESLAAK	49	99.971	(N-term)_iTRAQ[0], Lysine(K)_iTRAQ[9]	[7] F5 120912	142/134	2.780	1.699	1.523	1	Mascot
1277.6722	1277.5803	-0.0919	-72	291	300	FANLNEQAAR	38	99.631	(N-term)_iTRAQ[0]	[5] F4	115/107	2.254	1.613	1.124	1	Mascot
1409.7886	1409.7469	-0.0417	-30	378	386	EYQDLLNVK	60	99.997	(N-term)_iTRAQ[0], Lysine(K)_iTRAQ[9]	[7] F5 120912	243/235	2.284	1.143	1.029	1	Mascot
1534.8601	1534.8257	-0.0344	-22	217	228	VESLLDELAFVR	39	99.667	(N-term)_iTRAQ[0]	[1] F3 030912	512/504	1.070	0.620	0.800	1	Mascot
1555.8003	1555.7406	-0.0597	-38	279	288	NLQSAEEWYK	48	99.962	(N-term)_iTRAQ[0], Lysine(K)_iTRAQ[10]	[7] F5 120912	225/217	1.686	1.027	0.990	1	Mascot
1698.9008	1698.7812	-0.1196	-70	492	504	STIEEITTSSSQK	87	100	(N-term)_iTRAQ[0], Lysine(K)_iTRAQ[13]	[5] F4	124/116	2.363	1.208	0.991	1	Mascot
1784.9739	1784.9417	-0.0322	-18	68	83	RLPASDGLDLSQAAAR	44	99.896	(N-term)_iTRAQ[0]	[4] F7 and F10+11	243/235	1.852	1.042	0.841	1	Mascot
1807.0575	1807.0171	-0.0404	-22	216	228	KVESLLDELAFVR	52	99.985	(N-term)_iTRAQ[0], Lysine(K)_iTRAQ[1]	[6] F8 110912	484/476	1.927	1.036	0.821	1	Mascot
1807.0575	1807.0759	0.0184	10	216	228	KVESLLDELAFVR	76	100	(N-term)_iTRAQ[0], Lysine(K)_iTRAQ[1]	[4] F7 and F10+11	481/473	1.205	0.659	0.637	1	Mascot
1832.9712	1833.0073	0.0361	20	92	104	TNEKEQLQGLNDR	73	100	(N-term)_iTRAQ[0], Lysine(K)_iTRAQ[4]	[3] F6 and F9	1125/1117	1.412	0.789	0.627	1	Mascot
1832.9712	1833.0499	0.0787	43	92	104	TNEKEQLQGLNDR	35	99.232	(N-term)_iTRAQ[0], Lysine(K)_iTRAQ[4]	[4] F7 and F10+11	1056/1048	1.775	1.068	0.893	1	Mascot
2172.1169	2172.0225	-0.0944	-43	471	487	VGESFEETLEETVVSTK	84	100	(N-term)_iTRAQ[0], Lysine(K)_iTRAQ[17]	[1] F3 030912	423/415	1.871	0.928	0.649	1	Mascot
2213.0315	2213.1404	0.1089	49	448	462	KEEEEEEEEEEGASK	40	99.767	(N-term)_iTRAQ[0], Lysine(K)_iTRAQ[1,15]	[4] F7 and F10+11	1000/992	1.683	0.266	0.528	1	Mascot
2319.1338	2319.0679	-0.0659	-28	347	366	HSAEVAGYQDSIGQLESDLR	30	97.626	(N-term)_iTRAQ[0]	[3] F6 and F9	364/356	2.155	1.275	0.962	1	Mascot

48	glucose-6-phosphate isomerase [Rattus norvegicus]	gi|46485440	68977.4	13	806	1.078	1.098	1.033	0.227	0.297	0.346	16	16	16	100

Peptide Information

Calc. Mass	Obsrv. Mass	± da	± ppm	Start Seq.	End Seq.	Sequence	Ion Score	C. I. %	Modification	Plate [#] Name	Gel Idx/Pos [4700 Sample Name]	iTRAQ Ratio 115/114*	iTRAQ Ratio 116/114*	iTRAQ Ratio 117/114*	Rank	Result Type
1022.6594	1022.5971	-0.0623	-61	97	104	AVLHVALR	39	99.674	(N-term)_iTRAQ[0]	[6] F8 110912	221/213	0.913	0.740	0.892	1	Mascot
1022.6594	1022.728	0.0686	67	97	104	AVLHVALR	32	98.417	(N-term)_iTRAQ[0]	[3] F6 and F9	1183/1175	1.922	1.519	1.664	1	Mascot
1199.634	1199.599	-0.035	-29	82	89	DNMFSGLK	53	99.988	(N-term)_iTRAQ[0], Lysine(K)_iTRAQ[8]	[3] F6 and F9	256/248	1.216	1.192	0.779	1	Mascot
1249.6184	1249.5433	-0.0751	-60	28	36	ELFEADPER	34	99.063	(N-term)_iTRAQ[0]	[5] F4	146/138	1.098	1.831	0.820	1	Mascot
1249.6184	1249.5477	-0.0707	-57	28	36	ELFEADPER	36	99.443	(N-term)_iTRAQ[0]	[1] F3 030912	216/208	1.014	0.843	0.655	1	Mascot
1280.7249	1280.6808	-0.0441	-34	227	234	EWFLQAAK	48	99.961	(N-term)_iTRAQ[0], Lysine(K)_iTRAQ[8]	[7] F5 120912	283/275	0.915	1.086	0.698	1	Mascot
1464.8798	1464.9573	0.0775	53	253	261	VKEFGIDPK	30	97.604	(N-term)_iTRAQ[0], Lysine(K)_iTRAQ[2,9]	[4] F7 and F10+11	1105/1097	1.093	1.315	1.416	1	Mascot
1555.9127	1555.9303	0.0176	11	63	73	EVLHMLVDLAK	67	100	(N-term)_iTRAQ[0], Lysine(K)_iTRAQ[11]	[4] F7 and F10+11	436/428	1.047	1.309	0.992	1	Mascot
1687.7811	1687.725	-0.0561	-33	262	273	NMFEFWDWVGGR	55	99.991	(N-term)_iTRAQ[0]	[5] F4	409/401	1.062	1.062	0.743	1	Mascot
1875.0375	1875.0432	0.0057	3	181	194	VWFVSNIDGTHIAK	110	100	(N-term)_iTRAQ[0], Lysine(K)_iTRAQ[14]	[4] F7 and F10+11	356/348	0.823	1.004	1.050	1	Mascot
1892.0977	1892.0361	-0.0616	-33	242	254	HFVALSTNTDKVK	66	99.999	(N-term)_iTRAQ[0], Lysine(K)_iTRAQ[11, 13]	[2] F12 040912	216/208	1.045	0.777	0.979	1	Mascot
1944.0172	1943.9193	-0.0979	-50	212	226	TFTTQETITNAETAK	100	100	(N-term)_iTRAQ[0], Lysine(K)_iTRAQ[15]	[1] F3 030912	214/206	0.923	0.914	0.973	1	Mascot
1964.15	1964.0802	-0.0698	-36	424	438	ILLANFLAQTEALMK	53	99.986	(N-term)_iTRAQ[0], Lysine(K)_iTRAQ[15]	[5] F4	431/423	1.175	1.135	1.565	1	Mascot
1964.15	1964.1115	-0.0385	-20	424	438	ILLANFLAQTEALMK	99	100	(N-term)_iTRAQ[0], Lysine(K)_iTRAQ[15]	[1] F3 030912	567/559	0.917	1.024	1.396	1	Mascot
1980.9371	1980.843	-0.0941	-48	348	362	FAAYFQQGDMESNGK	51	99.981	(N-term)_iTRAQ[0], Lysine(K)_iTRAQ[15]	[5] F4	209/201	1.327	1.407	1.310	1	Mascot
1997.1066	1997.0878	-0.0188	-9	467	481	VFEGNRPTNSIVFTK	55	99.992	(N-term)_iTRAQ[0], Lysine(K)_iTRAQ[15]	[4] F7 and F10+11	275/267	1.103	0.939	1.303	1	Mascot

49	actin, cytoplasmic 2 [Homo sapiens]	gi|4501887	44923.8	12	794	1.014	1.003	1.000	0.220	0.248	0.310	24	24	24	100

Protein Group

actin, cytoplasmic 1 [Homo sapiens]	gi|4501885	44867.7

Peptide Information

Calc. Mass	Obsrv. Mass	± da	± ppm	Start Seq.	End Seq.	Sequence	Ion Score	C. I. %	Modification	Plate [#] Name	Gel Idx/Pos [4700 Sample Name]	iTRAQ Ratio 115/114*	iTRAQ Ratio 116/114*	iTRAQ Ratio 117/114*	Rank	Result Type

Calc. Mass	Obsrv. Mass	± da	± ppm	Start Seq.	End Seq.	Sequence	Ion Score	C. I. %	Modification	Plate [#] Name	Gel Idx/Pos [4700 Sample Name]	iTRAQ Ratio 115/114*	iTRAQ Ratio 116/114*	iTRAQ Ratio 117/114*	Rank	Result Type
932.639	932.6142	-0.0248	-27	63	68	GILTLK	34	99.093	(N-term)_iTRAQ[0], Lysine(K)_iTRAQ[6]	[7] F5 120912	201/193	0.986	1.144	0.899	1	Mascot
1276.6294	1276.5454	-0.084	-66	197	206	GYSFTTTAER	44	99.906	(N-term)_iTRAQ[0]	[5] F4	116/108	0.969	0.792	0.678	1	Mascot
1286.6913	1286.6261	-0.0652	-51	184	191	DLTDYLMK	47	99.954	(N-term)_iTRAQ[0], Lysine(K)_iTRAQ[8]	[5] F4	222/214	0.798	0.581	0.735	1	Mascot
1286.6913	1286.6511	-0.0402	-31	184	191	DLTDYLMK	59	99.997	(N-term)_iTRAQ[0], Lysine(K)_iTRAQ[8]	[7] F5 120912	278/270	1.068	0.998	1.197	1	Mascot
1342.8079	1342.7903	-0.0176	-13	29	39	AVFPSIVGRPR	40	99.763	(N-term)_iTRAQ[0]	[4] F7 and F10+11	264/256	0.759	0.689	0.833	1	Mascot
1449.8232	1449.7581	-0.0651	-45	316	326	EITALAPSTMK	86	100	(N-term)_iTRAQ[0], Lysine(K)_iTRAQ[11]	[5] F4	162/154	1.013	1.211	1.294	1	Mascot
1449.8232	1449.7938	-0.0294	-20	316	326	EITALAPSTMK	63	99.999	(N-term)_iTRAQ[0], Lysine(K)_iTRAQ[11]	[5] F4	595/587	0.681	0.930	0.597	1	Mascot
1459.7759	1459.848	0.0721	49	40	50	HQGVMVGMGQK	55	99.991	(N-term)_iTRAQ[0], Lysine(K)_iTRAQ[11]	[4] F7 and F10+11	1056/1048	0.957	1.125	1.181	1	Mascot
1486.7272	1486.6821	-0.0451	-30	51	61	DSYVGDEAQSK	95	100	(N-term)_iTRAQ[0], Lysine(K)_iTRAQ[11]	[7] F5 120912	83/75	1.198	1.220	1.451	1	Mascot
1659.8516	1659.9171	0.0655	39	85	95	IWHHTFYNELR	55	99.993	(N-term)_iTRAQ[0]	[8] F13-15 and F1+2	1151/1143	1.505	1.664	1.398	1	Mascot
1660.8051	1660.7354	-0.0697	-42	360	372	QEYDESGPSIVHR	45	99.928	(N-term)_iTRAQ[0]	[7] F5 120912	137/129	1.644	0.994	2.251	1	Mascot
1660.8051	1660.7783	-0.0268	-16	360	372	QEYDESGPSIVHR	75	100	(N-term)_iTRAQ[0]	[4] F7 and F10+11	159/151	0.996	0.940	1.110	1	Mascot
1660.8051	1660.8872	0.0821	49	360	372	QEYDESGPSIVHR	35	99.299	(N-term)_iTRAQ[0]	[4] F7 and F10+11	1054/1046	0.983	1.010	0.868	1	Mascot
1934.9944	1934.9426	-0.0518	-27	239	254	SYELPDGQVITIGNER	68	100	(N-term)_iTRAQ[0]	[1] F3 030912	354/346	1.415	1.525	0.953	1	Mascot
1934.9944	1934.9534	-0.041	-21	239	254	SYELPDGQVITIGNER	28	96.041	(N-term)_iTRAQ[0]	[8] F13-15 and F1+2	216/208	1.100	0.861	0.883	1	Mascot
1934.9944	1934.9812	-0.0132	-7	239	254	SYELPDGQVITIGNER	48	99.963	(N-term)_iTRAQ[0]	[8] F13-15 and F1+2	476/468	0.981	0.803	0.847	1	Mascot
1934.9944	1934.9906	-0.0038	-2	239	254	SYELPDGQVITIGNER	29	97.125	(N-term)_iTRAQ[0]	[8] F13-15 and F1+2	424/416	1.041	1.046	1.003	1	Mascot
1934.9944	1934.995	0.0006	0	239	254	SYELPDGQVITIGNER	34	99.114	(N-term)_iTRAQ[0]	[8] F13-15 and F1+2	420/412	0.922	0.920	0.943	1	Mascot
1934.9944	1934.9954	0.001	1	239	254	SYELPDGQVITIGNER	35	99.278	(N-term)_iTRAQ[0]	[8] F13-15 and F1+2	466/458	1.097	1.034	0.822	1	Mascot
1934.9944	1935.0089	0.0145	7	239	254	SYELPDGQVITIGNER	38	99.61	(N-term)_iTRAQ[0]	[8] F13-15 and F1+2	576/568	0.850	1.022	1.037	1	Mascot
1934.9944	1935.011	0.0166	9	239	254	SYELPDGQVITIGNER	35	99.285	(N-term)_iTRAQ[0]	[8] F13-15 and F1+2	417/409	0.981	0.923	0.915	1	Mascot
1934.9944	1935.017	0.0226	12	239	254	SYELPDGQVITIGNER	28	96.488	(N-term)_iTRAQ[0]	[8] F13-15 and F1+2	523/515	0.874	1.234	0.895	1	Mascot
2242.2693	2242.2427	-0.0266	-12	96	113	VAPEEHPVLLTEAPLNPK	51	99.979	(N-term)_iTRAQ[0], Lysine(K)_iTRAQ[18]	[7] F5 120912	263/255	1.067	1.023	1.071	1	Mascot
2827.343	2827.2866	-0.0564	-20	216	238	LCYVALDFEQEMATAAS SSSLEK	131	100	(N-term)_iTRAQ[0], Lysine(K)_iTRAQ[23], MMTS (C)[2]	[1] F3 030912	567/559	0.937	0.976	1.105	1	Mascot

50	type II cAMP-dependent protein kinase regulatory subunit [Rattus norvegicus]	gi|206671	49455.1	12	765	0.700	0.945	0.944	0.141	0.237	0.202	16	16	16	100

Protein Group

cAMP-dependent protein kinase type II-beta regulatory subunit [Rattus norvegicus] gi|158081759 49586.1

Peptide Information

Calc. Mass	Obsrv. Mass	± da	± ppm	Start Seq.	End Seq.	Sequence	Ion Score	C. I. %	Modification	Plate [#] Name	Gel Idx/Pos [4700 Sample Name]	iTRAQ Ratio 115/114*	iTRAQ Ratio 116/114*	iTRAQ Ratio 117/114*	Rank	Result Type
1230.698	1230.6449	-0.0531	-43	194	201	GTFDIYVK	43	99.881	(N-term)_iTRAQ[0], Lysine(K)_iTRAQ[8]	[7] F5 120912	228/220	0.843	1.006	0.836	1	Mascot
1468.7627	1468.7351	-0.0276	-19	381	390	LLGPCMEIMK	46	99.937	(N-term)_iTRAQ[0], Lysine(K)_iTRAQ[10], MMTS (C)[5]	[5] F4	365/357	0.878	1.349	1.291	1	Mascot
1472.6801	1472.6351	-0.045	-31	370	380	CLAMDVQAFER	41	99.782	(N-term)_iTRAQ[0], MMTS (C)[1]	[1] F3 030912	385/377	0.679	1.113	0.705	1	Mascot
1691.8599	1691.7723	-0.0876	-52	56	71	TWGDAGAAAGGGTPSK	118	100	(N-term)_iTRAQ[0], Lysine(K)_iTRAQ[16]	[5] F4	106/98	0.639	0.938	1.007	1	Mascot
1691.8599	1691.7827	-0.0772	-46	56	71	TWGDAGAAAGGGTPSK	107	100	(N-term)_iTRAQ[0], Lysine(K)_iTRAQ[16]	[7] F5 120912	140/132	0.826	1.267	1.295	1	Mascot
1699.8597	1699.8033	-0.0564	-33	216	229	GSFGELALMYNTPR	69	100	(N-term)_iTRAQ[0]	[1] F3 030912	370/362	0.749	0.891	0.987	1	Mascot
1699.8597	1699.879	0.0193	11	216	229	GSFGELALMYNTPR	29	96.7	(N-term)_iTRAQ[0]	[8] F13-15 and F1+2	401/393	0.825	0.866	1.022	1	Mascot
1844.999	1844.9969	-0.0021	-1	230	246	AATITATSPGALWGLDR	52	99.984	(N-term)_iTRAQ[0]	[8] F13-15 and F1+2	420/412	0.401	0.524	0.647	1	Mascot
1844.999	1845.0146	0.0156	8	230	246	AATITATSPGALWGLDR	47	99.956	(N-term)_iTRAQ[0]	[8] F13-15 and F1+2	417/409	0.798	0.983	1.031	1	Mascot
1892.0126	1891.9534	-0.0592	-31	263	275	MYESFIESLPFLK	46	99.935	(N-term)_iTRAQ[0], Lysine(K)_iTRAQ[13]	[5] F4	407/399	0.679	1.181	0.891	1	Mascot
1892.0126	1891.9652	-0.0474	-25	263	275	MYESFIESLPFLK	51	99.981	(N-term)_iTRAQ[0], Lysine(K)_iTRAQ[13]	[1] F3 030912	534/526	0.703	0.752	0.927	1	Mascot
1981.1116	1981.1353	0.0237	12	344	358	GQYFGELALVTNKPR	65	99.999	(N-term)_iTRAQ[0], Lysine(K)_iTRAQ[13]	[4] F7 and F10+11	338/330	0.742	0.906	1.027	1	Mascot
2067.0896	2067.1436	0.054	26	22	37	HQPADLLEFALQHFTR	59	99.997	(N-term)_iTRAQ[0]	[4] F7 and F10+11	1357/1349	0.717	0.729	0.951	1	Mascot
2164.21	2164.1306	-0.0794	-37	262	275	KMYESFIESLPFLK	50	99.978	(N-term)_iTRAQ[0], Lysine(K)_iTRAQ[1,14]	[6] F8 110912	506/498	0.616	1.111	1.022	1	Mascot
2283.1248	2283.0652	-0.0596	-26	154	170	NLDPEQMSQVLDAMFEK	93	100	(N-term)_iTRAQ[0], Lysine(K)_iTRAQ[17]	[1] F3 030912	514/506	0.699	0.941	1.070	1	Mascot

Calc. Mass	Obsrv. Mass	± da	± ppm	Start Seq.	End Seq.	Sequence	Ion Score	C. I. %	Modification	Plate [#] Name	Gel Idx/Pos [4700 Sample Name]	iTRAQ Ratio 115/114*	iTRAQ Ratio 116/114*	iTRAQ Ratio 117/114*	Rank	Result Type
2437.1028	2437.0037	-0.0991	-41	174	193	EGEHVIDQGDDGDNFYVIDR	78	100	(N-term)_iTRAQ[0]	[7] F5 120912	242/234	0.583	0.928	0.679	1	Mascot

51	syntaxin-1B [Rattus norvegicus]	gi|6981600	37298.5	12	756	1.161	1.018	1.004	0.303	0.279	0.311	14	14	14	100	

Peptide Information

Calc. Mass	Obsrv. Mass	± da	± ppm	Start Seq.	End Seq.	Sequence	Ion Score	C. I. %	Modification	Plate [#] Name	Gel Idx/Pos [4700 Sample Name]	iTRAQ Ratio 115/114*	iTRAQ Ratio 116/114*	iTRAQ Ratio 117/114*	Rank	Result Type
1323.777	1323.7388	-0.0382	-29	173	181	LAIFTDDIK	69	100	(N-term)_iTRAQ[0], Lysine(K)_iTRAQ[9]	[7] F5 120912	325/317	1.233	1.053	1.079	1	Mascot
1334.7413	1334.7059	-0.0354	-27	46	54	LSEDVEQVK	46	99.941	(N-term)_iTRAQ[0], Lysine(K)_iTRAQ[9]	[7] F5 120912	157/149	1.354	1.058	1.134	1	Mascot
1606.9387	1606.9962	0.0575	36	46	55	LSEDVEQVKK	63	99.999	(N-term)_iTRAQ[0], Lysine(K)_iTRAQ[9,10]	[4] F7 and F10+11	1072/1064	1.505	1.032	1.492	1	Mascot
1759.8947	1759.8065	-0.0882	-50	94	107	AIEQSIEQEEGLNR	76	100	(N-term)_iTRAQ[0]	[1] F3 030912	244/236	0.918	0.847	0.880	1	Mascot
1778.9647	1778.8517	-0.113	-64	56	69	QHSAILAAPNPDEK	87	100	(N-term)_iTRAQ[0], Lysine(K)_iTRAQ[14]	[6] F8 110912	182/174	1.014	1.195	1.411	1	Mascot
1778.9647	1778.9205	-0.0442	-25	56	69	QHSAILAAPNPDEK	47	99.952	(N-term)_iTRAQ[0], Lysine(K)_iTRAQ[14]	[4] F7 and F10+11	182/174	0.763	0.561	1.036	1	Mascot
1895.9259	1895.8894	-0.0365	-19	232	245	IEYNVEHSVDYVER	69	100	(N-term)_iTRAQ[0]	[4] F7 and F10+11	280/272	1.427	1.026	0.965	1	Mascot
1895.9259	1895.8931	-0.0328	-17	232	245	IEYNVEHSVDYVER	47	99.954	(N-term)_iTRAQ[0]	[3] F6 and F9	286/278	0.995	0.965	0.555	1	Mascot
1934.9779	1934.8999	-0.078	-40	126	139	FVEVMTEYNATQSK	74	100	(N-term)_iTRAQ[0], Lysine(K)_iTRAQ[14]	[1] F3 030912	299/291	1.691	0.956	1.250	1	Mascot
1936.0974	1936.1661	0.0687	35	70	82	TKQELEDLTADIK	44	99.891	(N-term)_iTRAQ[0], Lysine(K)_iTRAQ[2,13]	[3] F6 and F9	1273/1265	1.143	1.020	1.161	1	Mascot
1984.9631	1984.8519	-0.1112	-56	158	172	TTTNEELEDMLESGK	40	99.757	(N-term)_iTRAQ[0], Lysine(K)_iTRAQ[15]	[5] F4	236/228	1.648	1.805	1.278	1	Mascot
2051.1621	2051.0918	-0.0703	-34	55	69	KQHSAILAAPNPDEK	72	100	(N-term)_iTRAQ[0], Lysine(K)_iTRAQ[1,15]	[2] F12 040912	180/172	1.065	1.028	0.777	1	Mascot
2114.9614	2115.0208	0.0594	28	26	40	DHFMDEFFEQVEEIR	30	97.804	(N-term)_iTRAQ[0]	[4] F7 and F10+11	503/495	0.928	1.259	0.820	1	Mascot
2207.1753	2207.0608	-0.1145	-52	125	139	KFVEVMTEYNATQSK	85	100	(N-term)_iTRAQ[0], Lysine(K)_iTRAQ[1,15]	[6] F8 110912	290/282	1.010	0.870	0.708	1	Mascot

52	synapsin-1 isoform b [Rattus norvegicus]	gi|160707907	74327.5	12	754	0.890	1.072	1.291	0.324	0.276	0.427	18	18	18	100	

Protein Group

synapsin-1 isoform a [Rattus norvegicus]	gi|9507159	78691.8

Peptide Information

Calc. Mass	Obsrv. Mass	± da	± ppm	Start Seq.	End Seq.	Sequence	Ion Score	C. I. %	Modification	Plate [#] Name	Gel Idx/Pos [4700 Sample Name]	iTRAQ Ratio 115/114*	iTRAQ Ratio 116/114*	iTRAQ Ratio 117/114*	Rank	Result Type
1212.7198	1212.7803	0.0605	50	135	142	IHGEIDIK	38	99.625	(N-term)_iTRAQ[0], Lysine(K)_iTRAQ[8]	[4] F7 and F10+11	1084/1076	0.810	0.875	0.843	1	Mascot
1340.7783	1340.7566	-0.0217	-16	564	574	QASISGPAPPK	44	99.902	(N-term)_iTRAQ[0], Lysine(K)_iTRAQ[11]	[7] F5 120912	107/99	0.422	1.186	2.087	1	Mascot
1614.8625	1614.7589	-0.1036	-64	300	311	TYATAEPFIDAK	89	100	(N-term)_iTRAQ[0], Lysine(K)_iTRAQ[12]	[5] F4	180/172	1.022	1.155	1.277	1	Mascot
1614.8625	1614.8385	-0.024	-15	300	311	TYATAEPFIDAK	32	98.319	(N-term)_iTRAQ[0], Lysine(K)_iTRAQ[12]	[4] F7 and F10+11	228/220	1.532	1.951	2.093	1	Mascot
1705.9105	1705.8505	-0.06	-35	429	444	GSHSQTPSPGALPLGR	32	98.438	(N-term)_iTRAQ[0]	[3] F6 and F9	198/190	0.927	1.300	1.307	1	Mascot
1705.9105	1705.9017	-0.0088	-5	429	444	GSHSQTPSPGALPLGR	70	100	(N-term)_iTRAQ[0]	[4] F7 and F10+11	194/186	1.090	1.221	1.438	1	Mascot
1741.9656	1741.9034	-0.0622	-36	257	269	EMLSSTTYPVVVK	69	100	(N-term)_iTRAQ[0], Lysine(K)_iTRAQ[13]	[1] F3 030912	273/265	0.761	0.868	1.048	1	Mascot
1785.0381	1784.9508	-0.0873	-49	586	600	QGPPQKPPGPAGPIR	34	98.947	(N-term)_iTRAQ[0], Lysine(K)_iTRAQ[6]	[6] F8 110912	158/150	0.999	1.072	1.127	1	Mascot
1785.0381	1785.0195	-0.0186	-10	586	600	QGPPQKPPGPAGPIR	52	99.985	(N-term)_iTRAQ[0], Lysine(K)_iTRAQ[6]	[4] F7 and F10+11	155/147	0.894	1.068	1.039	1	Mascot
1868.8966	1868.9045	0.0079	4	337	352	TNTGSAMLEQIAMSDR	75	100	(N-term)_iTRAQ[0]	[8] F13-15 and F1+2	446/438	0.673	0.893	0.878	1	Mascot
1868.8966	1868.9363	0.0397	21	337	352	TNTGSAMLEQIAMSDR	58	99.996	(N-term)_iTRAQ[0]	[8] F13-15 and F1+2	547/539	1.268	1.410	1.613	1	Mascot
1924.079	1924.0212	-0.0578	-30	115	128	VLLVIDEPHTDWAK	74	100	(N-term)_iTRAQ[0], Lysine(K)_iTRAQ[14]	[3] F6 and F9	364/356	0.822	1.023	1.168	1	Mascot
1924.079	1924.0828	0.0038	2	115	128	VLLVIDEPHTDWAK	102	100	(N-term)_iTRAQ[0], Lysine(K)_iTRAQ[14]	[4] F7 and F10+11	368/360	1.311	1.341	1.310	1	Mascot
2196.0764	2196.1084	0.032	15	86	108	QTTAAAAATFSEQVGGGSGGAGR	30	97.479	(N-term)_iTRAQ[0]	[8] F13-15 and F1+2	542/534	0.862	0.856	1.152	1	Mascot
2196.0764	2196.1362	0.0598	27	86	108	QTTAAAAATFSEQVGGGSGGAGR	40	99.773	(N-term)_iTRAQ[0]	[8] F13-15 and F1+2	545/537	0.589	0.753	1.066	1	Mascot
2659.479	2659.696	0.217	82	280	299	VKVDNQHDFQDIASVVALTK	91	100	(N-term)_iTRAQ[0], Lysine(K)_iTRAQ[2,20]	[4] F7 and F10+11	1260/1252	1.000	0.929	1.113	1	Mascot
2709.4624	2709.5852	0.1228	45	238	256	KLGTEEFPLIDQTFYPNHK	34	99.021	(N-term)_iTRAQ[0], Lysine(K)_iTRAQ[1,19]	[4] F7 and F10+11	1259/1251	0.608	0.818	1.241	1	Mascot

Calc. Mass	Obsrv. Mass	± da	± ppm	Start Seq.	End Seq.	Sequence	Ion Score	C. I. %	Modification	Plate [#] Name	Gel Idx/Pos [4700 Sample Name]	iTRAQ Ratio 115/114*	iTRAQ Ratio 116/114*	iTRAQ Ratio 117/114*	Rank	Result Type
3182.6328	3182.4221	-0.2107	-66	445	474	QTSQQPAGPPAQQRPPP QGGPPQPGPGPQR	50	99.977	(N-term)_iTRAQ[0]	[1] F3 030912	148/140	1.188	1.132	2.475	1	Mascot

53	GDP-dissociation inhibitor [Rattus norvegicus]	gi|516540	55327.1	12	749	1.120	1.103	1.142	0.226	0.202	0.294	16	16	16	100

Protein Group

rab GDI alpha [Rattus norvegicus]	gi|396431	55271.1
rab GDP dissociation inhibitor alpha [Rattus norvegicus]	gi|71534276	55287.1

Peptide Information

Calc. Mass	Obsrv. Mass	± da	± ppm	Start Seq.	End Seq.	Sequence	Ion Score	C. I. %	Modification	Plate [#] Name	Gel Idx/Pos [4700 Sample Name]	iTRAQ Ratio 115/114*	iTRAQ Ratio 116/114*	iTRAQ Ratio 117/114*	Rank	Result Type
1315.7507	1315.726	-0.0247	-19	104	112	VVEGSFVYK	57	99.995	(N-term)_iTRAQ[0], Lysine(K)_iTRAQ[9]	[7] F5 120912	201/193	1.054	1.129	1.241	1	Mascot
1470.8767	1470.8695	-0.0072	-5	300	309	IICILSHPIK	57	99.995	(N-term)_iTRAQ[0], Lysine(K)_iTRAQ[10], MMTS (C)[3]	[4] F7 and F10+11	397/389	1.199	0.992	1.137	1	Mascot
1600.9056	1600.9763	0.0707	44	69	79	GRDWNVDLIPK	74	100	(N-term)_iTRAQ[0], Lysine(K)_iTRAQ[11]	[4] F7 and F10+11	1187/1179	1.222	1.141	1.253	1	Mascot
1604.8226	1604.8287	0.0061	4	56	68	FQLLEGPPESMGR	57	99.996	(N-term)_iTRAQ[0]	[8] F13-15 and F1+2	446/438	0.854	1.064	1.247	1	Mascot
1604.8226	1604.84	0.0174	11	56	68	FQLLEGPPESMGR	54	99.989	(N-term)_iTRAQ[0]	[8] F13-15 and F1+2	442/434	1.076	0.929	0.977	1	Mascot
1604.8226	1604.8485	0.0259	16	56	68	FQLLEGPPESMGR	44	99.91	(N-term)_iTRAQ[0]	[8] F13-15 and F1+2	542/534	1.232	1.306	1.353	1	Mascot
1604.8226	1604.8528	0.0302	19	56	68	FQLLEGPPESMGR	75	100	(N-term)_iTRAQ[0]	[8] F13-15 and F1+2	547/539	1.306	0.993	1.160	1	Mascot
1604.8226	1604.8588	0.0362	23	56	68	FQLLEGPPESMGR	47	99.949	(N-term)_iTRAQ[0]	[8] F13-15 and F1+2	594/586	1.175	1.378	1.540	1	Mascot
1609.782	1609.7303	-0.0517	-32	279	290	QLICDPSYIPDR	43	99.879	(N-term)_iTRAQ[0], MMTS (C)[4]	[1] F3 030912	330/322	1.280	1.405	1.073	1	Mascot
1612.7761	1612.6998	-0.0763	-47	157	169	TFEGVDPQTTSMR	50	99.976	(N-term)_iTRAQ[0]	[1] F3 030912	213/205	0.785	0.934	0.990	1	Mascot
1635.7756	1635.7195	-0.0561	-34	424	435	MAGSAFDFENMK	56	99.994	(N-term)_iTRAQ[0], Lysine(K)_iTRAQ[12]	[7] F5 120912	293/285	1.637	1.458	1.489	1	Mascot
1791.8767	1791.9171	0.0404	23	424	436	MAGSAFDFENMKR	45	99.915	(N-term)_iTRAQ[0], Lysine(K)_iTRAQ[12]	[3] F6 and F9	1246/1238	0.937	1.056	1.560	1	Mascot
2343.1821	2343.0969	-0.0852	-36	119	137	VPSTETEALASNLMGMF EK	99	100	(N-term)_iTRAQ[0], Lysine(K)_iTRAQ[19]	[1] F3 030912	525/517	1.145	1.184	0.861	1	Mascot
2435.2227	2435.1941	-0.0286	-12	36	54	NPYYGGESSSITPLEELY K	78	100	(N-term)_iTRAQ[0], Lysine(K)_iTRAQ[19]	[1] F3 030912	373/365	0.919	1.106	1.068	1	Mascot
2567.3867	2567.4387	0.052	20	174	193	KFDLGQDVIDFTGHALAL YR	90	100	(N-term)_iTRAQ[0], Lysine(K)_iTRAQ[1]	[4] F7 and F10+11	1360/1352	1.385	1.063	1.161	1	Mascot
3859.085	3858.9412	-0.1438	-37	349	379	YIAIASTTVETAEPEKEVE PALELLEPIDQK	28	96.096	(N-term)_iTRAQ[0], Lysine(K)_iTRAQ[16, 31]	[1] F3 030912	495/487	1.012	0.752	0.608	1	Mascot

54	L-lactate dehydrogenase B chain [Rattus norvegicus]	gi|6981146	40565.7	11	744	1.028	0.949	0.904	0.228	0.208	0.230	13	13	13	100

Peptide Information

Calc. Mass	Obsrv. Mass	± da	± ppm	Start Seq.	End Seq.	Sequence	Ion Score	C. I. %	Modification	Plate [#] Name	Gel Idx/Pos [4700 Sample Name]	iTRAQ Ratio 115/114*	iTRAQ Ratio 116/114*	iTRAQ Ratio 117/114*	Rank	Result Type
1057.6853	1057.6285	-0.0568	-54	92	100	IVVVTAGVR	39	99.678	(N-term)_iTRAQ[0]	[1] F3 030912	221/213	1.036	1.223	0.952	1	Mascot
1245.8181	1245.7594	-0.0587	-47	120	127	FIIPQIVK	50	99.977	(N-term)_iTRAQ[0], Lysine(K)_iTRAQ[8]	[5] F4	280/272	1.074	0.886	0.837	1	Mascot
1247.757	1247.7047	-0.0523	-42	300	308	GLTSVINQK	47	99.954	(N-term)_iTRAQ[0], Lysine(K)_iTRAQ[9]	[7] F5 120912	177/169	1.130	0.955	1.171	1	Mascot
1299.7705	1299.8062	0.0357	27	271	279	IHPVSTMVK	70	100	(N-term)_iTRAQ[0], Lysine(K)_iTRAQ[9]	[3] F6 and F9	1153/1145	1.019	0.953	1.011	1	Mascot
1464.7944	1464.7437	-0.0507	-35	320	329	SADTLWDIQK	56	99.993	(N-term)_iTRAQ[0], Lysine(K)_iTRAQ[10]	[7] F5 120912	246/238	1.598	1.138	1.283	1	Mascot
1474.8475	1474.9019	0.0544	37	309	318	LKDDEVAQLR	46	99.938	(N-term)_iTRAQ[0], Lysine(K)_iTRAQ[2]	[3] F6 and F9	1162/1154	0.848	0.766	0.556	1	Mascot
1474.8475	1474.9092	0.0617	42	309	318	LKDDEVAQLR	78	100	(N-term)_iTRAQ[0], Lysine(K)_iTRAQ[2]	[4] F7 and F10+11	1092/1084	0.993	0.938	0.729	1	Mascot
1541.8495	1541.7788	-0.0707	-46	234	244	MVVDSAYEVIK	58	99.996	(N-term)_iTRAQ[0], Lysine(K)_iTRAQ[11]	[5] F4	219/211	1.033	1.183	0.837	1	Mascot
1736.9918	1737.0806	0.0888	51	319	329	KSADTLWDIQK	62	99.998	(N-term)_iTRAQ[0], Lysine(K)_iTRAQ[1,11]	[4] F7 and F10+11	1155/1147	1.001	0.840	1.020	1	Mascot
1918.063	1918.0018	-0.0612	-32	44	58	SLADELALVDVLEDK	92	100	(N-term)_iTRAQ[0], Lysine(K)_iTRAQ[15]	[1] F3 030912	499/491	0.818	0.954	0.789	1	Mascot
1943.082	1942.9374	-0.1446	-74	78	91	IVADKDYSVTANSK	83	100	(N-term)_iTRAQ[0], Lysine(K)_iTRAQ[5,14]	[6] F8 110912	188/180	0.708	0.574	0.735	1	Mascot
1943.082	1943.0897	0.0077	4	78	91	IVADKDYSVTANSK	92	100	(N-term)_iTRAQ[0], Lysine(K)_iTRAQ[5,14]	[3] F6 and F9	1146/1138	1.382	1.173	1.228	1	Mascot
2303.3445	2303.386	0.0415	18	44	60	SLADELALVDVLEDKLK	102	100	(N-term)_iTRAQ[0], Lysine(K)_iTRAQ[15, 17]	[4] F7 and F10+11	532/524	0.997	0.984	0.902	1	Mascot

55	gamma-enolase [Rattus norvegicus]	gi|26023949	51467.7	12	733	1.245	1.326	1.169	0.659	0.747	0.590	16	16	16	100

Peptide Information

Calc. Mass	Obsrv. Mass	± da	± ppm	Start Seq.	End Seq.	Sequence	Ion Score	C. I. %	Modification	Plate [#] Name	Gel Idx/Pos [4700 Sample Name]	iTRAQ Ratio 115/114*	iTRAQ Ratio 116/114*	iTRAQ Ratio 117/114*	Rank	Result Type
1088.5874	1088.5542	-0.0332	-30	257	262	YDLDFK	29	97.132	(N-term)_iTRAQ[0], Lysine(K)_iTRAQ[6]	[3] F6 and F9	256/248	1.076	0.966	1.088	1	Mascot
1318.661	1318.5662	-0.0948	-72	413	422	IEEELGEEAR	46	99.938	(N-term)_iTRAQ[0]	[5] F4	133/125	1.103	1.058	0.923	1	Mascot
1418.8253	1418.8826	0.0573	40	184	193	LGAEVYHTLK	70	100	(N-term)_iTRAQ[0], Lysine(K)_iTRAQ[10]	[3] F6 and F9	1220/1212	1.216	1.566	1.198	1	Mascot
1532.8333	1532.9028	0.0695	45	254	262	DGKYDLDFK	54	99.99	(N-term)_iTRAQ[0], Lysine(K)_iTRAQ[3,9]	[4] F7 and F10+11	1116/1108	0.936	1.274	0.790	1	Mascot
1668.9166	1668.8774	-0.0392	-23	16	28	GNPTVEVDLHTAK	34	98.937	(N-term)_iTRAQ[0], Lysine(K)_iTRAQ[13]	[6] F8 110912	197/189	0.795	0.779	0.637	1	Mascot
1668.9166	1668.8903	-0.0263	-16	16	28	GNPTVEVDLHTAK	119	100	(N-term)_iTRAQ[0], Lysine(K)_iTRAQ[13]	[4] F7 and F10+11	193/185	1.082	0.964	0.999	1	Mascot
1732.8522	1732.8812	0.029	17	240	253	MVIGMDVAASEFYR	28	95.629	(N-term)_iTRAQ[0]	[8] F13-15 and F1+2	386/378	1.194	1.096	0.807	1	Mascot
1796.9993	1796.9619	-0.0374	-21	106	120	FGANAILGVSLAVCK	56	99.994	(N-term)_iTRAQ[0], Lysine(K)_iTRAQ[15], MMTS (C)[14]	[2] F12 040912	523/515	3.175	3.392	3.289	1	Mascot
1796.9993	1796.9692	-0.0301	-17	106	120	FGANAILGVSLAVCK	90	100	(N-term)_iTRAQ[0], Lysine(K)_iTRAQ[15], MMTS (C)[14]	[1] F3 030912	517/509	0.897	1.051	0.925	1	Mascot
1796.9993	1796.983	-0.0163	-9	106	120	FGANAILGVSLAVCK	50	99.973	(N-term)_iTRAQ[0], Lysine(K)_iTRAQ[15], MMTS (C)[14]	[1] F3 030912	589/581	2.528	2.618	1.762	1	Mascot
1894.9957	1894.8981	-0.0976	-52	344	358	VNQIGSVTEAIQACK	84	100	(N-term)_iTRAQ[0], Lysine(K)_iTRAQ[15], MMTS (C)[14]	[5] F4	298/290	0.810	0.745	0.998	1	Mascot
1949.0464	1949.0646	0.0182	9	33	50	AAVPSGASTGIYEALELR	43	99.878	(N-term)_iTRAQ[0]	[8] F13-15 and F1+2	397/389	1.044	1.100	0.973	1	Mascot
1949.0464	1949.1187	0.0723	37	33	50	AAVPSGASTGIYEALELR	47	99.954	(N-term)_iTRAQ[0]	[8] F13-15 and F1+2	1089/1081	3.036	3.154	2.483	1	Mascot
1988.9675	1988.955	-0.0125	-6	270	285	CITGDQLGALYQDFVR	35	99.19	(N-term)_iTRAQ[0], MMTS (C)[1]	[8] F13-15 and F1+2	291/283	1.107	1.138	1.258	1	Mascot
2083.084	2083.0671	-0.0169	-8	163	179	LAMQEFMILPVGAESFR	34	98.999	(N-term)_iTRAQ[0]	[8] F13-15 and F1+2	274/266	1.090	1.209	1.235	1	Mascot
2770.3247	2770.2292	-0.0955	-34	286	306	NYPVVSIEDPFDQDDWAAWSK	97	100	(N-term)_iTRAQ[0], Lysine(K)_iTRAQ[21]	[1] F3 030912	485/477	1.071	1.644	1.235	1	Mascot

56	neural cell adhesion molecule 1 precursor [Rattus norvegicus]	gi|13928706	103889.1	13	733	1.062	1.155	0.957	0.332	0.304	0.437	16	16	16	100	

Peptide Information

Calc. Mass	Obsrv. Mass	± da	± ppm	Start Seq.	End Seq.	Sequence	Ion Score	C. I. %	Modification	Plate [#] Name	Gel Idx/Pos [4700 Sample Name]	iTRAQ Ratio 115/114*	iTRAQ Ratio 116/114*	iTRAQ Ratio 117/114*	Rank	Result Type
1319.7206	1319.67	-0.0506	-38	122	130	NAPTPQEFK	37	99.459	(N-term)_iTRAQ[0], Lysine(K)_iTRAQ[9]	[7] F5 120912	144/136	1.411	1.193	1.103	1	Mascot
1428.7389	1428.6472	-0.0917	-64	363	373	QETLDGHMVVR	56	99.994	(N-term)_iTRAQ[0]	[6] F8 110912	183/175	0.701	0.746	0.665	1	Mascot
1428.7389	1428.7128	-0.0261	-18	363	373	QETLDGHMVVR	36	99.418	(N-term)_iTRAQ[0]	[4] F7 and F10+11	173/165	0.923	1.422	0.948	1	Mascot
1431.7478	1431.8168	0.069	48	550	559	SLGEEAWHSK	35	99.169	(N-term)_iTRAQ[0], Lysine(K)_iTRAQ[10]	[4] F7 and F10+11	1083/1075	0.962	1.101	1.416	1	Mascot
1456.8005	1456.8248	0.0243	17	294	304	AGEQDASIHLK	65	99.999	(N-term)_iTRAQ[0], Lysine(K)_iTRAQ[11]	[3] F6 and F9	1125/1117	0.896	1.055	0.671	1	Mascot
1497.8635	1497.7737	-0.0898	-60	606	616	TQPVREPSAPK	45	99.923	(N-term)_iTRAQ[0], Lysine(K)_iTRAQ[11]	[6] F8 110912	105/97	1.194	1.049	0.843	1	Mascot
1510.8363	1510.7854	-0.0509	-34	594	605	GLGEISAATEFK	91	100	(N-term)_iTRAQ[0], Lysine(K)_iTRAQ[12]	[5] F4	211/203	1.118	1.010	0.982	1	Mascot
1532.7832	1532.7203	-0.0629	-41	38	48	FFLCQVAGDAK	63	99.999	(N-term)_iTRAQ[0], Lysine(K)_iTRAQ[11], MMTS (C)[4]	[5] F4	323/315	0.972	1.241	1.207	1	Mascot
1587.9104	1587.9996	0.0892	56	651	661	ALASEWKPEIR	58	99.996	(N-term)_iTRAQ[0], Lysine(K)_iTRAQ[7]	[3] F6 and F9	1225/1217	1.112	0.945	0.964	1	Mascot
1588.8792	1588.7762	-0.103	-65	782	792	DESKEPIVEVR	35	99.233	(N-term)_iTRAQ[0], Lysine(K)_iTRAQ[4]	[6] F8 110912	175/167	1.319	1.314	0.965	1	Mascot
1665.8363	1665.7753	-0.061	-37	617	629	LEGQMGEDGNSIK	84	100	(N-term)_iTRAQ[0], Lysine(K)_iTRAQ[13]	[7] F5 120912	152/144	1.208	1.204	1.130	1	Mascot
1793.0405	1793.0302	-0.0103	-6	203	217	DIQVIVNVPPTVQAR	37	99.522	(N-term)_iTRAQ[0]	[8] F13-15 and F1+2	474/466	0.678	1.599	0.357	1	Mascot
1793.0405	1793.0404	-0.0001	0	203	217	DIQVIVNVPPTVQAR	41	99.819	(N-term)_iTRAQ[0]	[8] F13-15 and F1+2	470/462	0.871	0.855	0.759	1	Mascot
2159.1567	2158.9878	-0.1689	-78	827	842	SEPQESEAKPAPTEVK	37	99.483	(N-term)_iTRAQ[0], Lysine(K)_iTRAQ[9,16]	[6] F8 110912	129/121	2.132	2.050	2.113	1	Mascot
2159.1567	2159.0759	-0.0808	-37	827	842	SEPQESEAKPAPTEVK	73	100	(N-term)_iTRAQ[0], Lysine(K)_iTRAQ[9,16]	[4] F7 and F10+11	125/117	0.908	1.189	1.271	1	Mascot
2432.179	2432.0146	-0.1644	-68	277	293	NVDKNDEAEYVCIAENK	49	99.971	(N-term)_iTRAQ[0], Lysine(K)_iTRAQ[4,17], MMTS (C)[12]	[6] F8 110912	282/274	1.272	1.050	0.986	1	Mascot

57	ADP/ATP translocase 2 [Rattus norvegicus]	gi|32189350	36666.6	14	729	0.861	0.805	0.885	0.110	0.354	0.255	16	16	16	100	

Peptide Information

Calc. Mass	Obsrv. Mass	± da	± ppm	Start Seq.	End Seq.	Sequence	Ion Score	C. I. %	Modification	Plate [#] Name	Gel Idx/Pos [4700 Sample Name]	iTRAQ Ratio 115/114*	iTRAQ Ratio 116/114*	iTRAQ Ratio 117/114*	Rank	Result Type
961.5928	961.5514	-0.0414	-43	141	147	LAADVGK	32	98.307	(N-term)_iTRAQ[0], Lysine(K)_iTRAQ[7]	[3] F6 and F9	148/140	0.803	0.727	0.511	1	Mascot
1046.5867	1046.5386	-0.0481	-46	273	280	GAWSNVLR	38	99.605	(N-term)_iTRAQ[0]	[7] F5 120912	225/217	0.873	0.909	0.751	1	Mascot
1138.6191	1138.5891	-0.03	-26	156	163	GLGDCLVK	42	99.838	(N-term)_iTRAQ[0], Lysine(K)_iTRAQ[8], MMTS (C)[5]	[7] F5 120912	252/244	0.983	0.991	0.964	1	Mascot
1264.7511	1264.6907	-0.0604	-48	97	105	QIFLGGVDK	54	99.989	(N-term)_iTRAQ[0], Lysine(K)_iTRAQ[9]	[7] F5 120912	217/209	0.918	1.092	0.799	1	Mascot
1265.6763	1265.6188	-0.0575	-45	64	72	EQGVLSFWR	40	99.761	(N-term)_iTRAQ[0]	[5] F4	280/272	0.927	0.519	0.724	1	Mascot
1265.6763	1265.6593	-0.017	-13	64	72	EQGVLSFWR	56	99.994	(N-term)_iTRAQ[0]	[7] F5 120912	349/341	0.937	1.083	1.310	1	Mascot
1420.8522	1420.912	0.0598	42	97	106	QIFLGGVDKR	49	99.97	(N-term)_iTRAQ[0], Lysine(K)_iTRAQ[9]	[4] F7 and F10+11	1120/1112	0.807	0.922	1.109	1	Mascot
1424.8835	1424.818	-0.0655	-46	34	43	LLLQVQHASK	54	99.991	(N-term)_iTRAQ[0], Lysine(K)_iTRAQ[10]	[6] F8 110912	244/236	0.653	0.436	0.690	1	Mascot
1507.8042	1507.765	-0.0392	-26	189	199	AAYFGIYDTAK	60	99.998	(N-term)_iTRAQ[0], Lysine(K)_iTRAQ[11]	[7] F5 120912	252/244	0.797	0.944	1.247	1	Mascot
1683.958	1684.0256	0.0676	40	269	280	AFFKGAWSNVLR	32	98.505	(N-term)_iTRAQ[0], Lysine(K)_iTRAQ[4]	[4] F7 and F10+11	1271/1263	0.712	0.868	1.080	1	Mascot
1734.9465	1734.8595	-0.087	-50	81	92	YFPTQALNFAFK	56	99.994	(N-term)_iTRAQ[0], Lysine(K)_iTRAQ[12]	[6] F8 110912	391/383	0.886	1.042	0.759	1	Mascot
1734.9465	1734.8595	-0.087	-50	81	92	YFPTQALNFAFK	61	99.998	(N-term)_iTRAQ[0], Lysine(K)_iTRAQ[12]	[5] F4	343/335	1.011	1.048	1.017	1	Mascot
1821.8075	1821.761	-0.0465	-26	246	259	GTDIMYTGTLDCWR	47	99.953	(N-term)_iTRAQ[0], MMTS (C)[12]	[1] F3 030912	408/400	0.927	1.053	0.863	1	Mascot
1900.0499	1900.0328	-0.0171	-9	281	295	GMGGAFVLVLYDEIK	79	100	(N-term)_iTRAQ[0], Lysine(K)_iTRAQ[15]	[5] F4	412/404	0.904	0.539	0.698	1	Mascot
2094.0049	2093.9075	-0.0974	-47	245	259	KGTDIMYTGTLDCWR	70	100	(N-term)_iTRAQ[0], Lysine(K)_iTRAQ[1], MMTS (C)[13]	[6] F8 110912	391/383	0.983	1.127	0.850	1	Mascot
2172.2473	2172.3198	0.0725	33	281	296	GMGGAFVLVLYDEIKK	58	99.996	(N-term)_iTRAQ[0], Lysine(K)_iTRAQ[15, 16]	[4] F7 and F10+11	1355/1347	0.756	0.333	1.238	1	Mascot

58	ATP synthase subunit alpha, mitochondrial precursor [Rattus norvegicus]	gi|40538742	64419.9	11	726	0.858	0.937	1.029	0.226	0.215	0.258	14	14	14	100

Protein Group

ATP synthase alpha subunit precursor (EC 3.6.1.3) [Rattus norvegicus]	gi|203055	63493.3
Chain A, Rat Liver F1-Atpase	gi|6729934	59806.2

Peptide Information

Calc. Mass	Obsrv. Mass	± da	± ppm	Start Seq.	End Seq.	Sequence	Ion Score	C. I. %	Modification	Plate [#] Name	Gel Idx/Pos [4700 Sample Name]	iTRAQ Ratio 115/114*	iTRAQ Ratio 116/114*	iTRAQ Ratio 117/114*	Rank	Result Type
1170.6967	1170.6381	-0.0586	-50	195	204	AVDSLVPIGR	60	99.997	(N-term)_iTRAQ[0]	[1] F3 030912	252/244	1.175	1.036	1.367	1	Mascot
1191.6653	1191.6218	-0.0435	-37	187	194	EPMQTGIK	53	99.988	(N-term)_iTRAQ[0], Lysine(K)_iTRAQ[8]	[7] F5 120912	117/109	0.672	0.817	1.208	1	Mascot
1459.8365	1459.7806	-0.0559	-38	150	161	VVDALGNAIDGK	46	99.938	(N-term)_iTRAQ[0], Lysine(K)_iTRAQ[12]	[5] F4	212/204	0.595	0.605	0.798	1	Mascot
1567.7758	1567.722	-0.0538	-34	46	58	TGTAEMSSILEER	70	100	(N-term)_iTRAQ[0]	[1] F3 030912	273/265	0.598	0.774	0.758	1	Mascot
1575.8993	1575.8179	-0.0814	-52	306	316	HALIIYDDLSK	61	99.998	(N-term)_iTRAQ[0], Lysine(K)_iTRAQ[11]	[6] F8 110912	295/287	0.892	0.991	1.046	1	Mascot
1575.8993	1575.9287	0.0294	19	306	316	HALIIYDDLSK	65	99.999	(N-term)_iTRAQ[0], Lysine(K)_iTRAQ[11]	[3] F6 and F9	1252/1244	1.347	1.409	1.604	1	Mascot
1581.9224	1582.0013	0.0789	50	494	503	GYLDKLEPSK	42	99.833	(N-term)_iTRAQ[0], Lysine(K)_iTRAQ[5,10]	[4] F7 and F10+11	1129/1121	1.046	1.207	1.119	1	Mascot
1697.8407	1697.8021	-0.0386	-23	335	347	EAYPGDVFYLHSR	84	100	(N-term)_iTRAQ[0]	[4] F7 and F10+11	290/282	0.731	0.856	0.862	1	Mascot
1697.8407	1697.9202	0.0795	47	335	347	EAYPGDVFYLHSR	51	99.981	(N-term)_iTRAQ[0]	[4] F7 and F10+11	1186/1178	0.774	0.981	0.715	1	Mascot
1719.8885	1719.9108	0.0223	13	59	73	ILGADTSVDLEETGR	74	100	(N-term)_iTRAQ[0]	[8] F13-15 and F1+2	498/490	0.830	1.008	0.992	1	Mascot
1955.9994	1955.9102	-0.0892	-46	89	103	NVQAEEMVEFSSGLK	90	100	(N-term)_iTRAQ[0], Lysine(K)_iTRAQ[15]	[5] F4	252/244	1.055	1.170	1.290	1	Mascot
1955.9994	1955.9253	-0.0741	-38	89	103	NVQAEEMVEFSSGLK	97	100	(N-term)_iTRAQ[0], Lysine(K)_iTRAQ[15]	[1] F3 030912	331/323	0.800	0.893	0.941	1	Mascot
2392.2427	2392.1687	-0.074	-31	104	123	GMSLNLEPDNVGVVVFGNDK	105	100	(N-term)_iTRAQ[0], Lysine(K)_iTRAQ[20]	[1] F3 030912	412/404	0.904	0.774	1.091	1	Mascot
2482.2698	2482.2136	-0.0562	-23	442	463	EVAAFAQFGSDLDAATQQLLSR	33	98.665	(N-term)_iTRAQ[0]	[8] F13-15 and F1+2	243/235	0.932	0.884	0.978	1	Mascot

59	creatine kinase B-type [Rattus norvegicus]	gi|31542401	45941.3	11	722	1.020	0.927	0.851	0.148	0.297	0.194	20	20	20	100

Protein Group

Ckb protein [Rattus norvegicus]	gi|56388799	48521.7

creatine kinase [Rattus norvegicus] gi|203474 45955.3

Peptide Information

Calc. Mass	Obsrv. Mass	± da	± ppm	Start Seq.	End Seq.	Sequence	Ion Score	C. I. %	Modification	Plate [#] Name	Gel Idx/Pos [4700 Sample Name]	iTRAQ Ratio 115/114*	iTRAQ Ratio 116/114*	iTRAQ Ratio 117/114*	Rank	Result Type
1166.7144	1166.7467	0.0323	28	308	314	FSEVLKR	28	96.406	(N-term)_iTRAQ[0], Lysine(K)_iTRAQ[6]	[4] F7 and F10+11	1100/1092	1.156	1.208	0.912	1	Mascot
1175.6578	1175.6068	-0.051	-43	359	366	LLIEMEQR	45	99.928	(N-term)_iTRAQ[0]	[5] F4	212/204	1.196	1.167	1.144	1	Mascot
1175.6578	1175.6089	-0.0489	-42	359	366	LLIEMEQR	48	99.962	(N-term)_iTRAQ[0]	[1] F3 030912	284/276	1.137	0.889	0.852	1	Mascot
1376.7181	1376.6733	-0.0448	-33	87	96	DLFDPIIEDR	54	99.99	(N-term)_iTRAQ[0]	[1] F3 030912	342/334	1.184	0.785	0.801	1	Mascot
1447.8281	1447.7845	-0.0436	-30	33	43	VLTPELYAELR	36	99.359	(N-term)_iTRAQ[0]	[2] F12 040912	394/386	1.036	0.478	0.635	1	Mascot
1447.8281	1447.7926	-0.0355	-25	33	43	VLTPELYAELR	44	99.892	(N-term)_iTRAQ[0]	[1] F3 030912	357/349	1.044	0.979	0.924	1	Mascot
1746.9358	1746.9626	0.0268	15	157	172	LAVEALSSLDGDLSGR	99	100	(N-term)_iTRAQ[0]	[8] F13-15 and F1+2	386/378	0.865	0.754	0.750	1	Mascot
1815.9514	1815.8787	-0.0727	-40	224	236	TFLVWINEEDHLR	55	99.992	(N-term)_iTRAQ[0]	[6] F8 110912	456/448	0.835	0.672	0.787	1	Mascot
1815.9514	1815.928	-0.0234	-13	224	236	TFLVWINEEDHLR	56	99.994	(N-term)_iTRAQ[0]	[3] F6 and F9	446/438	1.010	1.020	0.974	1	Mascot
1815.9514	1815.9694	0.018	10	224	236	TFLVWINEEDHLR	60	99.997	(N-term)_iTRAQ[0]	[4] F7 and F10+11	454/446	0.968	0.969	0.775	1	Mascot
1815.9514	1816.0253	0.0739	41	224	236	TFLVWINEEDHLR	30	97.701	(N-term)_iTRAQ[0]	[4] F7 and F10+11	1320/1312	0.860	1.350	1.465	1	Mascot
1834.9674	1834.9216	-0.0458	-25	253	265	FCTGLTQIETLFK	72	100	(N-term)_iTRAQ[0], Lysine(K)_iTRAQ[13], MMTS (C)[2]	[5] F4	414/406	1.041	0.966	0.934	1	Mascot
1834.9674	1834.9331	-0.0343	-19	253	265	FCTGLTQIETLFK	73	100	(N-term)_iTRAQ[0], Lysine(K)_iTRAQ[13], MMTS (C)[2]	[1] F3 030912	543/535	0.870	0.868	0.796	1	Mascot
1971.0468	1970.9591	-0.0877	-44	367	381	LEQGQPIDDLMPAQK	81	100	(N-term)_iTRAQ[0], Lysine(K)_iTRAQ[15]	[1] F3 030912	286/278	1.032	0.897	0.867	1	Mascot
1971.0468	1970.9734	-0.0734	-37	367	381	LEQGQPIDDLMPAQK	37	99.52	(N-term)_iTRAQ[0], Lysine(K)_iTRAQ[15]	[5] F4	211/203	0.839	0.717	0.821	1	Mascot
2109.0332	2109.0518	0.0186	9	321	341	GTGGVDTAAVGGVFDVSNADR	30	97.267	(N-term)_iTRAQ[0]	[8] F13-15 and F1+2	479/471	1.341	1.112	0.931	1	Mascot
2109.0332	2109.0535	0.0203	10	321	341	GTGGVDTAAVGGVFDVSNADR	36	99.34	(N-term)_iTRAQ[0]	[8] F13-15 and F1+2	579/571	0.882	0.631	0.508	1	Mascot
2137.1824	2137.1338	-0.0486	-23	342	358	LGFSEVELVQMVVDGVK	36	99.326	(N-term)_iTRAQ[0], Lysine(K)_iTRAQ[17]	[5] F4	419/411	1.097	1.509	0.773	1	Mascot
2137.1824	2137.136	-0.0464	-22	342	358	LGFSEVELVQMVVDGVK	83	100	(N-term)_iTRAQ[0], Lysine(K)_iTRAQ[17]	[1] F3 030912	550/542	1.200	1.382	0.802	1	Mascot
2474.1655	2474.2368	0.0713	29	14	32	FPAEDEFPDLSSHNNHMAK	118	100	(N-term)_iTRAQ[0], Lysine(K)_iTRAQ[19]	[4] F7 and F10+11	1183/1175	0.987	0.890	0.927	1	Mascot

60	guanine nucleotide-binding protein G(o) subunit alpha [Rattus norvegicus]	gi|8394152	44393.5	11	708	0.986	1.031	1.021	0.368	0.284	0.380	16	16	16	100

Peptide Information

Calc. Mass	Obsrv. Mass	± da	± ppm	Start Seq.	End Seq.	Sequence	Ion Score	C. I. %	Modification	Plate [#] Name	Gel Idx/Pos [4700 Sample Name]	iTRAQ Ratio 115/114*	iTRAQ Ratio 116/114*	iTRAQ Ratio 117/114*	Rank	Result Type
1188.6022	1188.5197	-0.0825	-69	155	162	YYLDSLDR	49	99.967	(N-term)_iTRAQ[0]	[5] F4	186/178	1.176	1.206	1.051	1	Mascot
1345.8301	1345.7644	-0.0657	-49	36	46	LLLLGAGESGK	84	100	(N-term)_iTRAQ[0], Lysine(K)_iTRAQ[11]	[5] F4	219/211	1.116	1.118	1.283	1	Mascot
1355.7053	1355.6609	-0.0444	-33	146	154	EYQLNDSAK	39	99.674	(N-term)_iTRAQ[0], Lysine(K)_iTRAQ[9]	[7] F5 120912	117/109	0.499	0.605	0.773	1	Mascot
1577.9235	1577.9937	0.0702	44	22	32	NLKEDGISAAK	72	100	(N-term)_iTRAQ[0], Lysine(K)_iTRAQ[3,11]	[4] F7 and F10+11	1056/1048	0.896	1.079	1.067	1	Mascot
1668.9207	1668.8728	-0.0479	-29	182	193	TTGIVETHFTFK	82	100	(N-term)_iTRAQ[0], Lysine(K)_iTRAQ[12]	[4] F7 and F10+11	286/278	1.242	1.237	1.046	1	Mascot
1714.7782	1714.7203	-0.0579	-34	131	143	LWGDSGIQECFNR	38	99.585	(N-term)_iTRAQ[0], MMTS (C)[10]	[5] F4	326/318	0.956	0.952	0.767	1	Mascot
1733.8956	1733.8046	-0.091	-52	55	67	IIHEDGFSGEDVK	105	100	(N-term)_iTRAQ[0], Lysine(K)_iTRAQ[13]	[6] F8 110912	224/216	1.044	0.907	1.019	1	Mascot
1733.8956	1733.9307	0.0351	20	55	67	IIHEDGFSGEDVK	112	100	(N-term)_iTRAQ[0], Lysine(K)_iTRAQ[13]	[3] F6 and F9	1181/1173	1.899	1.720	1.642	1	Mascot
1833.9467	1833.9734	0.0267	15	163	177	IGAADYQPTEQDILR	33	98.671	(N-term)_iTRAQ[0]	[8] F13-15 and F1+2	489/481	1.027	1.062	0.747	1	Mascot
1833.9467	1833.9792	0.0325	18	163	177	IGAADYQPTEQDILR	41	99.816	(N-term)_iTRAQ[0]	[8] F13-15 and F1+2	539/531	1.091	1.189	0.838	1	Mascot
1833.9467	1833.9922	0.0455	25	163	177	IGAADYQPTEQDILR	42	99.848	(N-term)_iTRAQ[0]	[8] F13-15 and F1+2	588/580	1.578	1.462	2.539	1	Mascot
1871.9419	1871.8441	-0.0978	-52	87	100	AMDTLGVEYGDKER	64	99.999	(N-term)_iTRAQ[0], Lysine(K)_iTRAQ[12]	[6] F8 110912	224/216	0.885	0.952	0.967	1	Mascot
1887.9368	1887.8199	-0.1169	-62	87	100	AMDTLGVEYGDKER	39	99.661	(N-term)_iTRAQ[0], Lysine(K)_iTRAQ[12], Oxidation (M)[2]	[6] F8 110912	180/172	0.869	0.755	0.720	1	Mascot
2101.9729	2101.9607	-0.0122	-6	114	130	MEDTEPFSAELLSAMMR	39	99.683	(N-term)_iTRAQ[0]	[8] F13-15 and F1+2	281/273	0.995	0.927	1.058	1	Mascot
2115.9927	2115.9229	-0.0698	-33	244	258	MHESLMLFDSICNNK	89	100	(N-term)_iTRAQ[0], Lysine(K)_iTRAQ[15],	[6] F8 110912	469/461	0.652	0.999	0.785	1	Mascot

Calc. Mass	Obsrv. Mass	± da	± ppm	Start Seq.	End Seq.	Sequence	Ion Score	C. I. %	Modification	Plate [#] Name	Gel Idx/Pos [4700 Sample Name]	iTRAQ Ratio 115/114*	iTRAQ Ratio 116/114*	iTRAQ Ratio 117/114*	Rank	Result Type
2115.9927	2115.9407	-0.052	-25	244	258	MHESLMLFDSICNNK	28	95.709	MMTS (C)[12] (N-term)_iTRAQ[0], Lysine(K)_iTRAQ[15], MMTS (C)[12]	[2] F12 040912	421/413	0.652	0.820	1.016	1	Mascot

61 14-3-3 protein theta [Mus musculus] gi|6756039 30872.8 12 704 1.160 1.214 1.147 0.311 0.359 0.223 13 13 13 100

Peptide Information

Calc. Mass	Obsrv. Mass	± da	± ppm	Start Seq.	End Seq.	Sequence	Ion Score	C. I. %	Modification	Plate [#] Name	Gel Idx/Pos [4700 Sample Name]	iTRAQ Ratio 115/114*	iTRAQ Ratio 116/114*	iTRAQ Ratio 117/114*	Rank	Result Type
957.6019	957.6076	0.0057	6	116	120	VFYLK	34	98.922	(N-term)_iTRAQ[0], Lysine(K)_iTRAQ[5]	[3] F6 and F9	262/254	1.285	1.183	1.199	1	Mascot
1191.7195	1191.6932	-0.0263	-22	61	68	VISSIEQK	55	99.992	(N-term)_iTRAQ[0], Lysine(K)_iTRAQ[8]	[7] F5 120912	159/151	1.339	1.437	1.443	1	Mascot
1195.7296	1195.6782	-0.0514	-43	42	49	NLLSVAYK	53	99.987	(N-term)_iTRAQ[0], Lysine(K)_iTRAQ[8]	[7] F5 120912	233/225	1.100	1.296	1.119	1	Mascot
1333.7634	1333.7305	-0.0329	-25	213	222	DSTLIMQLLR	42	99.831	(N-term)_iTRAQ[0]	[1] F3 030912	466/458	1.966	2.108	0.925	1	Mascot
1333.7634	1333.7313	-0.0321	-24	213	222	DSTLIMQLLR	48	99.963	(N-term)_iTRAQ[0]	[2] F12 040912	472/464	1.159	0.774	0.827	1	Mascot
1401.6244	1401.5737	-0.0507	-36	128	138	YLAEVACGDDR	43	99.877	(N-term)_iTRAQ[0], MMTS (C)[7]	[1] F3 030912	308/300	1.220	1.343	1.015	1	Mascot
1411.5957	1411.5417	-0.054	-38	19	27	YDDMATCMK	51	99.981	(N-term)_iTRAQ[0], Lysine(K)_iTRAQ[9], MMTS (C)[7]	[7] F5 120912	228/220	1.104	1.164	1.123	1	Mascot
1608.8843	1608.7767	-0.1076	-67	104	115	YLIANATNPESK	52	99.985	(N-term)_iTRAQ[0], Lysine(K)_iTRAQ[12]	[5] F4	147/139	0.681	0.884	1.335	1	Mascot
1668.9143	1668.8535	-0.0608	-36	92	103	SICTTVLELLDK	38	99.566	(N-term)_iTRAQ[0], Lysine(K)_iTRAQ[12], MMTS (C)[3]	[5] F4	438/430	0.848	1.057	1.501	1	Mascot
1676.8212	1676.7205	-0.1007	-60	28	41	AVTEQGAELSNEER	90	100	(N-term)_iTRAQ[0]	[1] F3 030912	151/143	1.321	1.585	1.247	1	Mascot
2317.1558	2317.0596	-0.0962	-42	140	157	QTIENSQGAYQEAFDISK	102	100	(N-term)_iTRAQ[0], Lysine(K)_iTRAQ[18]	[1] F3 030912	277/269	1.040	1.433	1.409	1	Mascot
2433.1919	2433.1223	-0.0696	-29	194	212	TAFDEAIAELDTLNEDSYK	103	100	(N-term)_iTRAQ[0], Lysine(K)_iTRAQ[19]	[1] F3 030912	506/498	1.257	1.180	0.947	1	Mascot
2589.3533	2589.2271	-0.1262	-49	139	157	KQTIENSQGAYQEAFDISK	37	99.468	(N-term)_iTRAQ[0], Lysine(K)_iTRAQ[1,19]	[6] F8 110912	271/263	1.191	0.880	1.057	1	Mascot

62 RecName: Full=Aspartate aminotransferase, cytoplasmic; AltName: Full=Glutamate oxaloacetate transam gi|122065118 49753.7 14 700 *1.130* *1.067* *1.069* 0.448 0.181 0.415 18 18 18 100

Protein Group

cytosolic aspartate aminotransferase [Rattus norvegicus] gi|220684 49752.7

Peptide Information

Calc. Mass	Obsrv. Mass	± da	± ppm	Start Seq.	End Seq.	Sequence	Ion Score	C. I. %	Modification	Plate [#] Name	Gel Idx/Pos [4700 Sample Name]	iTRAQ Ratio 115/114*	iTRAQ Ratio 116/114*	iTRAQ Ratio 117/114*	Rank	Result Type
1092.6285	1092.5505	-0.078	-71	34	42	VNLGVGAYR	43	99.876	(N-term)_iTRAQ[0]	[5] F4	146/138	0.977	0.765	0.949	1	Mascot
1099.567	1099.5521	-0.0149	-14	161	166	YWDAEK	31	98.148	(N-term)_iTRAQ[0], Lysine(K)_iTRAQ[6]	[4] F7 and F10+11	194/186	1.293	1.163	1.194	1	Mascot
1122.6439	1122.5862	-0.0577	-51	284	290	VLSQMEK	34	99.095	(N-term)_iTRAQ[0], Lysine(K)_iTRAQ[7]	[3] F6 and F9	193/185	0.526	1.294	1.059	1	Mascot
1156.5872	1156.5284	-0.0588	-51	260	267	NFGLYNER	28	95.968	(N-term)_iTRAQ[0]	[7] F5 120912	172/164	0.972	0.870	0.755	1	Mascot
1217.6584	1217.6837	0.0253	21	379	387	HIYLMPSGR	33	98.804	(N-term)_iTRAQ[0]	[3] F6 and F9	1181/1173	1.128	0.917	1.099	1	Mascot
1364.8259	1364.8851	0.0592	43	33	42	KVNLGVGAYR	29	96.584	(N-term)_iTRAQ[0], Lysine(K)_iTRAQ[1]	[4] F7 and F10+11	1092/1084	1.113	1.074	1.274	1	Mascot
1409.7886	1409.7173	-0.0713	-51	370	378	QVEYLVNEK	64	99.999	(N-term)_iTRAQ[0], Lysine(K)_iTRAQ[9]	[7] F5 120912	180/172	1.045	1.008	1.023	1	Mascot
1415.8091	1415.7471	-0.062	-44	101	114	VGGVQSLGGTGALR	49	99.972	(N-term)_iTRAQ[0]	[1] F3 030912	219/211	1.043	1.233	0.933	1	Mascot
1441.7672	1441.6644	-0.1028	-71	294	305	ITWSNPPAQGAR	62	99.998	(N-term)_iTRAQ[0]	[5] F4	135/127	1.467	1.538	1.547	1	Mascot
1573.8094	1573.7983	-0.0111	-7	21	32	LIADFRDDPDPR	36	99.447	(N-term)_iTRAQ[0]	[4] F7 and F10+11	262/254	0.892	0.877	0.879	1	Mascot
1655.8877	1655.901	0.0133	8	43	55	TDDSQPWVLPVVR	43	99.889	(N-term)_iTRAQ[0]	[8] F13-15 and F1+2	426/418	1.033	1.190	0.981	1	Mascot
1655.8877	1655.9043	0.0166	10	43	55	TDDSQPWVLPVVR	36	99.315	(N-term)_iTRAQ[0]	[8] F13-15 and F1+2	430/422	1.354	1.040	0.881	1	Mascot
1655.8877	1655.9104	0.0227	14	43	55	TDDSQPWVLPVVR	51	99.982	(N-term)_iTRAQ[0]	[8] F13-15 and F1+2	530/522	1.470	1.052	1.047	1	Mascot
1655.8877	1655.9282	0.0405	24	43	55	TDDSQPWVLPVVR	33	98.892	(N-term)_iTRAQ[0]	[8] F13-15 and F1+2	584/576	*3.053*	*1.136*	*3.330*	1	Mascot
1820.91	1820.9156	0.0056	3	82	97	SCASQLVLGDNSPALR	72	100	(N-term)_iTRAQ[0], MMTS (C)[2]	[8] F13-15 and F1+2	458/450	1.254	1.038	0.764	1	Mascot
1820.91	1820.9479	0.0379	21	82	97	SCASQLVLGDNSPALR	51	99.979	(N-term)_iTRAQ[0], MMTS (C)[2]	[8] F13-15 and F1+2	559/551	1.109	1.036	0.796	1	Mascot
1926.043	1925.942	-0.101	-52	397	411	NLDYVATSINEAVTK	49	99.969	(N-term)_iTRAQ[0], Lysine(K)_iTRAQ[15]	[1] F3 030912	365/357	0.803	1.144	0.932	1	Mascot
2574.2803	2574.2205	-0.0598	-23	217	236	FLFPFFDSAYQGFASGDLEK	118	100	(N-term)_iTRAQ[0], Lysine(K)_iTRAQ[20]	[1] F3 030912	554/546	1.107	1.068	1.177	1	Mascot

63	plasma membrane calcium-transporting ATPase 2 [Rattus norvegicus]	gi|6978557	145313.4	11	698	0.998	0.885	0.833	0.574	0.719	0.285	11	11	11	100

Protein Group

RecName: Full=Plasma membrane calcium-transporting ATPase 2; Short=PMCA2; AltName: Full=Plasma memb	gi|14286100	150082.9

Peptide Information

Calc. Mass	Obsrv. Mass	± da	± ppm	Start Seq.	End Seq.	Sequence	Ion Score	C. I. %	Modification	Plate [#] Name	Gel Idx/Pos [4700 Sample Name]	iTRAQ Ratio 115/114*	iTRAQ Ratio 116/114*	iTRAQ Ratio 117/114*	Rank	Result Type
1063.6173	1063.5909	-0.0264	-25	1081	1087	GQILWFR	30	97.593	(N-term)_iTRAQ[0]	[7] F5 120912	330/322	0.913	0.784	0.531	1	Mascot
1679.8208	1679.7271	-0.0937	-56	236	249	IDESSLTGESDQVR	81	100	(N-term)_iTRAQ[0]	[1] F3 030912	202/194	0.971	0.807	0.856	1	Mascot
1730.8557	1730.8104	-0.0453	-26	1117	1129	TSIHNFMAHPEFR	67	99.999	(N-term)_iTRAQ[0]	[2] F12 040912	296/288	0.685	0.744	0.905	1	Mascot
1805.9136	1805.8044	-0.1092	-60	305	318	QQDGAAAMEMQPLK	53	99.987	(N-term)_iTRAQ[0], Lysine(K)_iTRAQ[14]	[5] F4	148/140	1.342	0.945	1.511	1	Mascot
1848.9062	1848.8259	-0.0803	-43	46	58	IKETYGDTESICR	38	99.634	(N-term)_iTRAQ[0], Lysine(K)_iTRAQ[2], MMTS (C)[12]	[6] F8 110912	260/252	3.116	5.020	1.154	1	Mascot
1898.0092	1898.0089	-0.0003	0	464	477	MTVVQAYVGDVHYK	49	99.967	(N-term)_iTRAQ[0], Lysine(K)_iTRAQ[14]	[4] F7 and F10+11	326/318	1.576	0.975	0.810	1	Mascot
1899.0321	1898.9402	-0.0919	-48	62	77	TSPVEGLPGTAPDLEK	52	99.985	(N-term)_iTRAQ[0], Lysine(K)_iTRAQ[16]	[1] F3 030912	255/247	0.974	0.638	1.010	1	Mascot
1930.0491	1929.9447	-0.1044	-54	768	784	QVVAVTGDGTNDGPALK	139	100	(N-term)_iTRAQ[0], Lysine(K)_iTRAQ[17]	[1] F3 030912	207/199	0.811	0.816	0.750	1	Mascot
2155.1379	2155.0815	-0.0564	-26	802	818	EASDIILTDDNFSSIVK	49	99.972	(N-term)_iTRAQ[0], Lysine(K)_iTRAQ[17]	[1] F3 030912	375/367	0.620	0.615	0.556	1	Mascot
2202.2466	2202.2207	-0.0259	-12	768	785	QVVAVTGDGTNDGPALK K	45	99.929	(N-term)_iTRAQ[0], Lysine(K)_iTRAQ[17, 18]	[4] F7 and F10+11	194/186	0.780	0.419	0.852	1	Mascot
2259.9749	2259.9724	-0.0025	-1	438	455	HLDACETMGNATAICSDK	96	100	(N-term)_iTRAQ[0], Lysine(K)_iTRAQ[18], MMTS (C)[5,15]	[4] F7 and F10+11	378/370	0.641	0.897	0.639	1	Mascot

64	glutamate dehydrogenase 1, mitochondrial precursor [Rattus norvegicus]	gi|6980956	66696.8	15	690	1.147	0.996	1.075	0.382	0.335	0.402	18	18	18	100

Protein Group

unnamed protein product [Rattus norvegicus]	gi|56200	66708.8

Peptide Information

Calc. Mass	Obsrv. Mass	± da	± ppm	Start Seq.	End Seq.	Sequence	Ion Score	C. I. %	Modification	Plate [#] Name	Gel Idx/Pos [4700 Sample Name]	iTRAQ Ratio 115/114*	iTRAQ Ratio 116/114*	iTRAQ Ratio 117/114*	Rank	Result Type
1107.6283	1107.5808	-0.0475	-43	528	535	YNLGLDLR	37	99.555	(N-term)_iTRAQ[0]	[5] F4	213/205	1.259	1.411	1.383	1	Mascot
1413.7107	1413.6598	-0.0509	-36	192	200	NYTDNELEK	40	99.756	(N-term)_iTRAQ[0], Lysine(K)_iTRAQ[9]	[7] F5 120912	128/120	0.789	0.714	1.398	1	Mascot
1484.8835	1484.9067	0.0232	16	353	363	LQHGSILGFPK	57	99.995	(N-term)_iTRAQ[0], Lysine(K)_iTRAQ[11]	[3] F6 and F9	1252/1244	0.994	0.926	1.142	1	Mascot
1496.7832	1496.7098	-0.0734	-49	172	183	CAVVDVPFGGAK	51	99.979	(N-term)_iTRAQ[0], Lysine(K)_iTRAQ[12], MMTS (C)[1]	[5] F4	244/236	0.934	0.938	1.172	1	Mascot
1529.7944	1529.6816	-0.1128	-74	152	162	YSTDVSVDEVK	53	99.988	(N-term)_iTRAQ[0], Lysine(K)_iTRAQ[11]	[5] F4	130/122	1.376	0.994	1.192	1	Mascot
1569.7306	1569.665	-0.0656	-42	125	136	DDGSWEVIEGYR	32	98.519	(N-term)_iTRAQ[0]	[5] F4	218/210	0.852	1.145	0.924	1	Mascot
1569.7306	1569.6689	-0.0617	-39	125	136	DDGSWEVIEGYR	41	99.807	(N-term)_iTRAQ[0]	[1] F3 030912	292/284	1.046	0.856	0.734	1	Mascot
1635.8285	1635.7825	-0.046	-28	504	516	DIVHSGLAYTMER	46	99.937	(N-term)_iTRAQ[0]	[3] F6 and F9	266/258	1.045	1.145	0.889	1	Mascot
1635.8285	1635.8041	-0.0244	-15	504	516	DIVHSGLAYTMER	44	99.901	(N-term)_iTRAQ[0]	[4] F7 and F10+11	265/257	2.304	1.485	1.664	1	Mascot
1725.8317	1725.895	0.0633	37	124	136	RDDGSWEVIEGYR	35	99.217	(N-term)_iTRAQ[0]	[4] F7 and F10+11	1173/1165	1.199	1.302	1.325	1	Mascot
1867.9786	1867.9742	-0.0044	-2	481	496	HGGTIPVVPTAEFQDR	72	100	(N-term)_iTRAQ[0]	[3] F6 and F9	273/265	1.159	0.926	0.860	1	Mascot
1892.9924	1892.9335	-0.0589	-31	303	318	TFVVQGFGNVGLHSMR	69	100	(N-term)_iTRAQ[0]	[3] F6 and F9	357/349	1.591	1.374	1.216	1	Mascot
1948.1338	1948.0433	-0.0905	-46	77	90	GASIVEDKLVEDLK	34	99.008	(N-term)_iTRAQ[0], Lysine(K)_iTRAQ[8,1 4]	[6] F8 110912	376/368	1.329	0.560	1.434	1	Mascot
2060.0242	2060.0308	0.0066	3	213	231	GFIGPGIDVPAPDMSTGE R	36	99.331	(N-term)_iTRAQ[0]	[8] F13-15 and F1+2	438/430	1.562	1.196	1.174	1	Mascot
2060.0242	2060.0408	0.0166	8	213	231	GFIGPGIDVPAPDMSTGE R	50	99.975	(N-term)_iTRAQ[0]	[8] F13-15 and F1+2	434/426	0.996	0.934	0.603	1	Mascot
2065.0144	2064.9871	-0.0273	-13	461	476	DSNYHLLMSVQESLER	30	97.396	(N-term)_iTRAQ[0]	[3] F6 and F9	384/376	1.227	1.152	1.666	1	Mascot
2171.2424	2171.3521	0.1097	51	108	123	IIKPCNHVLSLSFPIR	32	98.42	(N-term)_iTRAQ[0], Lysine(K)_iTRAQ[3], MMTS (C)[5]	[4] F7 and F10+11	1310/1302	0.613	1.077	0.973	1	Mascot
2452.2751	2452.3865	0.1114	45	497	516	ISGASEKDIVHSGLAYTM ER	43	99.877	(N-term)_iTRAQ[0], Lysine(K)_iTRAQ[7]	[4] F7 and F10+11	1206/1198	1.252	0.502	0.517	1	Mascot

65	synaptotagmin-1 [Rattus norvegicus]	gi|148356226	55858.5	11	687	0.764	0.944	0.958	0.165	0.341	0.222	13	13	13	100

Peptide Information

Calc. Mass	Obsrv. Mass	± da	± ppm	Start Seq.	End Seq.	Sequence	Ion Score	C. I. %	Modification	Plate [#] Name	Gel Idx/Pos [4700 Sample Name]	iTRAQ Ratio 115/114*	iTRAQ Ratio 116/114*	iTRAQ Ratio 117/114*	Rank	Result Type
1237.7037	1237.6615	-0.0422	-34	214	222	VPYSELGGK	36	99.355	(N-term)_iTRAQ[0], Lysine(K)_iTRAQ[9]	[7] F5 120912	170/162	0.775	1.056	0.889	1	Mascot
1237.7037	1237.6622	-0.0415	-34	214	222	VPYSELGGK	48	99.962	(N-term)_iTRAQ[0], Lysine(K)_iTRAQ[9]	[3] F6 and F9	205/197	0.919	0.825	0.858	1	Mascot
1246.7041	1246.7651	0.061	49	237	244	HDIIGEFK	49	99.968	(N-term)_iTRAQ[0], Lysine(K)_iTRAQ[8]	[4] F7 and F10+11	1135/1127	0.582	0.797	0.842	1	Mascot
1370.6759	1370.748	0.0721	53	389	398	HWSDMLANPR	44	99.898	(N-term)_iTRAQ[0]	[4] F7 and F10+11	1140/1132	0.766	0.650	0.827	1	Mascot
1391.8998	1391.973	0.0732	53	183	190	VFLLPDKK	45	99.923	(N-term)_iTRAQ[0], Lysine(K)_iTRAQ[7,8]	[4] F7 and F10+11	1160/1152	0.666	0.865	0.958	1	Mascot
1473.7532	1473.7119	-0.0413	-28	223	233	TLVMAVYDFDR	69	100	(N-term)_iTRAQ[0]	[1] F3 030912	404/396	1.054	2.286	1.737	1	Mascot
1556.8192	1556.75	-0.0692	-44	376	388	VFVGYNSTGAELR	64	99.999	(N-term)_iTRAQ[0]	[1] F3 030912	262/254	0.861	1.008	1.069	1	Mascot
1568.824	1568.7273	-0.0967	-62	302	313	MDVGGLSDPYVK	67	100	(N-term)_iTRAQ[0], Lysine(K)_iTRAQ[12]	[5] F4	190/182	0.932	1.250	0.828	1	Mascot
1841.0214	1841.1151	0.0937	51	301	313	KMDVGGLSDPYVK	85	100	(N-term)_iTRAQ[0], Lysine(K)_iTRAQ[1,13]	[4] F7 and F10+11	1140/1132	0.762	1.041	1.059	1	Mascot
1865.9828	1866.0343	0.0515	28	261	272	DLQSAEKEEQEK	47	99.946	(N-term)_iTRAQ[0], Lysine(K)_iTRAQ[7,12]	[3] F6 and F9	1101/1093	0.491	0.717	0.716	1	Mascot
1865.9828	1866.0714	0.0886	47	261	272	DLQSAEKEEQEK	81	100	(N-term)_iTRAQ[0], Lysine(K)_iTRAQ[7,12]	[4] F7 and F10+11	1035/1027	0.714	0.729	0.903	1	Mascot
1873.0105	1872.91	-0.1005	-54	201	213	TLNPVFNEQFTFK	100	100	(N-term)_iTRAQ[0], Lysine(K)_iTRAQ[13]	[5] F4	288/280	0.743	0.874	0.964	1	Mascot
2060.9983	2060.9272	-0.0711	-34	245	260	VPMNTVDFGHVTEEWR	35	99.221	(N-term)_iTRAQ[0]	[3] F6 and F9	358/350	0.854	0.882	1.111	1	Mascot

66	microtubule-associated protein 6 [Rattus norvegicus]	gi|8850229	111847.3	15	669	1.080	1.090	0.961	0.417	0.496	0.299	16	16	16	100

Peptide Information

Calc. Mass	Obsrv. Mass	± da	± ppm	Start Seq.	End Seq.	Sequence	Ion Score	C. I. %	Modification	Plate [#] Name	Gel Idx/Pos [4700 Sample Name]	iTRAQ Ratio 115/114*	iTRAQ Ratio 116/114*	iTRAQ Ratio 117/114*	Rank	Result Type
1132.6057	1132.5868	-0.0189	-17	239	247	AGPAWMVTR	38	99.585	(N-term)_iTRAQ[0]	[7] F5 120912	206/198	1.168	1.120	0.779	1	Mascot
1238.678	1238.6539	-0.0241	-19	15	21	FWNQLDK	35	99.235	(N-term)_iTRAQ[0], Lysine(K)_iTRAQ[7]	[3] F6 and F9	289/281	1.096	1.765	1.431	1	Mascot
1341.7988	1341.6992	-0.0996	-74	701	711	DLGPVAPASVK	44	99.892	(N-term)_iTRAQ[0], Lysine(K)_iTRAQ[11]	[5] F4	127/119	0.876	0.508	0.771	1	Mascot
1398.8202	1398.7194	-0.1008	-72	668	678	DQAPVVPGSLK	40	99.763	(N-term)_iTRAQ[0], Lysine(K)_iTRAQ[11]	[5] F4	127/119	1.800	1.600	1.541	1	Mascot
1416.813	1416.72	-0.093	-66	690	700	DQGAVLLGPMK	41	99.814	(N-term)_iTRAQ[0], Lysine(K)_iTRAQ[11]	[5] F4	172/164	0.809	0.579	0.769	1	Mascot
1488.7302	1488.6556	-0.0746	-50	466	477	EEVTSTVSSSYR	47	99.954	(N-term)_iTRAQ[0]	[1] F3 030912	157/149	0.435	0.724	0.601	1	Mascot
1569.7306	1569.6262	-0.1044	-67	139	150	SEYQPSDAPFER	32	98.526	(N-term)_iTRAQ[0]	[5] F4	128/120	1.157	1.557	1.416	1	Mascot
1569.7306	1569.6483	-0.0823	-52	139	150	SEYQPSDAPFER	36	99.335	(N-term)_iTRAQ[0]	[1] F3 030912	201/193	1.535	1.277	0.772	1	Mascot
1574.8094	1574.7231	-0.0863	-55	712	722	DQDHMASELLK	62	99.998	(N-term)_iTRAQ[0], Lysine(K)_iTRAQ[11]	[6] F8 110912	220/212	1.350	1.081	1.017	1	Mascot
1715.8673	1715.9392	0.0719	42	505	516	MVHETSYSAQFK	76	100	(N-term)_iTRAQ[0], Lysine(K)_iTRAQ[12]	[3] F6 and F9	1164/1156	1.214	0.938	1.013	1	Mascot
1844.0177	1844.083	0.0653	35	537	548	SLYSEPFKESPK	50	99.973	(N-term)_iTRAQ[0], Lysine(K)_iTRAQ[8,12]	[4] F7 and F10+11	1117/1109	0.904	0.810	0.737	1	Mascot
1957.011	1957.0863	0.0753	38	60	77	AVAIETQPAQGESDAVAR	43	99.889	(N-term)_iTRAQ[0]	[8] F13-15 and F1+2	596/588	1.054	1.083	0.755	1	Mascot
1972.2456	1972.2273	-0.0183	-9	482	493	AWTDIKPVKPIK	33	98.63	(N-term)_iTRAQ[0], Lysine(K)_iTRAQ[6,9,12]	[2] F12 040912	279/271	1.486	2.081	1.197	1	Mascot
2002.1458	2002.1948	0.049	24	631	645	EPHKDQGPVAPGLPK	53	99.988	(N-term)_iTRAQ[0], Lysine(K)_iTRAQ[4,15]	[4] F7 and F10+11	1066/1058	1.115	1.300	0.934	1	Mascot
2230.2051	2230.1606	-0.0445	-20	813	830	NQGLGGPEPAKDTGTDLK	29	96.848	(N-term)_iTRAQ[0], Lysine(K)_iTRAQ[11,18]	[4] F7 and F10+11	174/166	0.784	1.168	1.080	1	Mascot
2805.3901	2805.2676	-0.1225	-44	340	364	TEGHEETPLPPAQSQTQEGPAAGK	46	99.945	(N-term)_iTRAQ[0], Lysine(K)_iTRAQ[25]	[7] F5 120912	134/126	1.349	1.049	1.149	1	Mascot

67	mitochondrial import receptor subunit TOM70 [Rattus norvegicus]	gi|47058988	76645.8	11	660	0.838	0.933	1.050	0.134	0.222	0.135	11	11	11	100

Peptide Information

Calc. Mass	Obsrv. Mass	± da	± ppm	Start Seq.	End Seq.	Sequence	Ion Score	C. I. %	Modification	Plate [#] Name	Gel Idx/Pos [4700 Sample Name]	iTRAQ Ratio 115/114*	iTRAQ Ratio 116/114*	iTRAQ Ratio 117/114*	Rank	Result Type

Calc. Mass	Obsrv. Mass	± da	± ppm	Start Seq.	End Seq.	Sequence	Ion Score	C. I. %	Modification	Plate [#] Name	Gel Idx/Pos [4700 Sample Name]	iTRAQ Ratio 115/114*	iTRAQ Ratio 116/114*	iTRAQ Ratio 117/114*	Rank	Result Type
1164.6986	1164.655	-0.0436	-37	436	443	FALAQAQK	34	99.033	(N-term)_iTRAQ[0], Lysine(K)_iTRAQ[8]	[7] F5 120912	158/150	1.067	1.024	0.949	1	Mascot
1223.6942	1223.6608	-0.0334	-27	329	337	YMAEALLLR	28	95.729	(N-term)_iTRAQ[0]	[1] F3 030912	383/375	0.862	1.526	1.105	1	Mascot
1273.7878	1273.7649	-0.0229	-18	519	526	GLLQLQWK	43	99.888	(N-term)_iTRAQ[0], Lysine(K)_iTRAQ[8]	[7] F5 120912	336/328	0.768	0.917	1.224	1	Mascot
1293.7412	1293.6777	-0.0635	-49	162	170	AAAFEQLQK	48	99.958	(N-term)_iTRAQ[0], Lysine(K)_iTRAQ[9]	[7] F5 120912	172/164	0.696	0.728	0.933	1	Mascot
1472.7255	1472.6483	-0.0772	-52	151	161	NADLSTFYQNR	67	100	(N-term)_iTRAQ[0]	[5] F4	153/145	0.781	1.036	1.040	1	Mascot
1636.7411	1636.6769	-0.0642	-39	305	314	QYMEEENYDK	60	99.998	(N-term)_iTRAQ[0], Lysine(K)_iTRAQ[10]	[7] F5 120912	140/132	0.828	0.956	0.992	1	Mascot
1685.8707	1685.9541	0.0834	49	171	180	WKEVAQDCTK	41	99.811	(N-term)_iTRAQ[0], Lysine(K)_iTRAQ[2,10], MMTS (C)[8]	[4] F7 and F10+11	1159/1151	0.683	0.918	0.889	1	Mascot
1845.0303	1844.9811	-0.0492	-27	248	260	NREPLMPSPQFIK	34	99.066	(N-term)_iTRAQ[0], Lysine(K)_iTRAQ[13]	[4] F7 and F10+11	292/284	1.114	1.065	1.078	1	Mascot
2000.0117	1999.9014	-0.1103	-55	450	465	QAYTANNSSQVQAAMK	91	100	(N-term)_iTRAQ[0], Lysine(K)_iTRAQ[16]	[5] F4	108/100	0.883	0.790	1.379	1	Mascot
2267.1516	2267.0828	-0.0688	-30	261	277	SYFSSFTDDIISQPMLK	95	100	(N-term)_iTRAQ[0], Lysine(K)_iTRAQ[17]	[1] F3 030912	469/461	0.869	0.894	1.070	1	Mascot
2539.2188	2539.1072	-0.1116	-44	477	496	CAEGYALYAQALTDQQQFGK	119	100	(N-term)_iTRAQ[0], Lysine(K)_iTRAQ[20], MMTS (C)[1]	[1] F3 030912	423/415	0.767	0.653	0.975	1	Mascot

68	aspartate aminotransferase, mitochondrial [Rattus norvegicus]	gi|6980972	52073.2	14	659	0.910	0.947	1.077	0.169	0.205	0.379	17	17	17	100

Peptide Information

Calc. Mass	Obsrv. Mass	± da	± ppm	Start Seq.	End Seq.	Sequence	Ion Score	C. I. %	Modification	Plate [#] Name	Gel Idx/Pos [4700 Sample Name]	iTRAQ Ratio 115/114*	iTRAQ Ratio 116/114*	iTRAQ Ratio 117/114*	Rank	Result Type
1124.6006	1124.5383	-0.0623	-55	60	68	MNLGVGAYR	36	99.378	(N-term)_iTRAQ[0]	[5] F4	153/145	0.721	0.982	0.806	1	Mascot
1218.7092	1218.6653	-0.0439	-36	339	345	QWLQEVK	41	99.802	(N-term)_iTRAQ[0], Lysine(K)_iTRAQ[7]	[3] F6 and F9	252/244	0.705	0.907	1.025	1	Mascot
1257.6926	1257.6716	-0.021	-17	288	296	VGAFTVVCK	32	98.522	(N-term)_iTRAQ[0], Lysine(K)_iTRAQ[9], MMTS (C)[8]	[7] F5 120912	307/299	1.116	0.840	1.343	1	Mascot
1292.6807	1292.6228	-0.0579	-45	397	404	EFSVYMTK	51	99.978	(N-term)_iTRAQ[0], Lysine(K)_iTRAQ[8]	[7] F5 120912	219/211	1.012	1.183	1.384	1	Mascot
1347.8331	1347.884	0.0509	38	82	90	KAEAQIAGK	45	99.917	(N-term)_iTRAQ[0], Lysine(K)_iTRAQ[1,9]	[4] F7 and F10+11	993/985	0.696	1.068	1.014	1	Mascot
1414.839	1414.7878	-0.0512	-36	326	337	IAATILTSPDLR	31	98.079	(N-term)_iTRAQ[0]	[1] F3 030912	332/324	1.140	0.718	1.319	1	Mascot
1462.9329	1462.9888	0.0559	38	356	364	TQLVSNLKK	32	98.268	(N-term)_iTRAQ[0], Lysine(K)_iTRAQ[8,9]	[4] F7 and F10+11	1072/1064	1.290	1.228	1.522	1	Mascot
1490.9066	1490.965	0.0584	39	338	345	KQWLQEVK	48	99.965	(N-term)_iTRAQ[0], Lysine(K)_iTRAQ[1,8]	[4] F7 and F10+11	1123/1115	0.942	1.465	1.333	1	Mascot
1593.9084	1593.9174	0.009	6	126	139	FVTVQTISGTGALR	45	99.93	(N-term)_iTRAQ[0]	[8] F13-15 and F1+2	462/454	0.786	1.002	1.076	1	Mascot
1593.9084	1593.9319	0.0235	15	126	139	FVTVQTISGTGALR	37	99.459	(N-term)_iTRAQ[0]	[8] F13-15 and F1+2	614/606	0.953	0.844	1.347	1	Mascot
1593.9084	1593.9375	0.0291	18	126	139	FVTVQTISGTGALR	34	98.91	(N-term)_iTRAQ[0]	[8] F13-15 and F1+2	621/613	0.833	1.022	1.003	1	Mascot
1759.8989	1759.8247	-0.0742	-42	95	107	EYLPIGGLADFCK	52	99.986	(N-term)_iTRAQ[0], Lysine(K)_iTRAQ[13], MMTS (C)[12]	[5] F4	391/383	0.911	0.711	0.845	1	Mascot
1759.8989	1759.8491	-0.0498	-28	95	107	EYLPIGGLADFCK	61	99.998	(N-term)_iTRAQ[0], Lysine(K)_iTRAQ[13], MMTS (C)[12]	[1] F3 030912	503/495	0.767	0.742	0.432	1	Mascot
1819.0059	1818.895	-0.1109	-61	108	122	ASAELALGENSEVLK	98	100	(N-term)_iTRAQ[0], Lysine(K)_iTRAQ[15]	[5] F4	193/185	0.906	0.783	1.096	1	Mascot
1923.9059	1923.8855	-0.0204	-11	186	200	TCGFDFSGALEDISK	41	99.781	(N-term)_iTRAQ[0], Lysine(K)_iTRAQ[15], MMTS (C)[2]	[5] F4	351/343	1.003	0.837	0.801	1	Mascot
2374.2502	2374.2625	0.0123	5	91	107	NLDKEYLPIGGLADFCK	65	99.999	(N-term)_iTRAQ[0], Lysine(K)_iTRAQ[4,17], MMTS (C)[16]	[4] F7 and F10+11	481/473	0.958	1.067	1.358	1	Mascot
2610.4612	2610.3479	-0.1133	-43	408	430	ISVAGVTSGNVGYLAHAIHQVTK	34	98.944	(N-term)_iTRAQ[0], Lysine(K)_iTRAQ[23]	[6] F8 110912	421/413	0.949	1.015	1.345	1	Mascot

69	2-oxoglutarate dehydrogenase, mitochondrial precursor [Rattus norvegicus]	gi|62945278	124536.3	12	657	0.948	1.006	1.080	0.211	0.280	0.272	12	12	12	100

Peptide Information

Calc. Mass	Obsrv. Mass	± da	± ppm	Start Seq.	End Seq.	Sequence	Ion Score	C. I. %	Modification	Plate [#] Name	Gel Idx/Pos [4700 Sample Name]	iTRAQ Ratio 115/114*	iTRAQ Ratio 116/114*	iTRAQ Ratio 117/114*	Rank	Result Type
1114.5806	1114.5636	-0.017	-15	75	81	SWDIFFR	41	99.795	(N-term)_iTRAQ[0]	[7] F5 120912	401/393	0.791	1.030	1.064	1	Mascot
1690.8938	1690.8452	-0.0486	-29	1009	1020	FLDTAFDLDAFK	56	99.994	(N-term)_iTRAQ[0], Lysine(K)_iTRAQ[12]	[5] F4	368/360	0.815	1.120	0.892	1	Mascot
1738.9221	1738.8257	-0.0964	-55	884	897	VIPEDGPAAQNPDK	72	100	(N-term)_iTRAQ[0], Lysine(K)_iTRAQ[14]	[5] F4	111/103	0.981	0.874	1.163	1	Mascot
1809.8715	1809.8705	-0.001	-1	870	883	TSFDEMLPGTHFQR	77	100	(N-term)_iTRAQ[0]	[4] F7 and F10+11	309/301	1.159	0.663	0.916	1	Mascot
1820.8789	1820.8253	-0.0536	-29	325	336	ELEQIFCQFDSK	52	99.985	(N-term)_iTRAQ[0],	[5] F4	368/360	0.833	1.300	1.202	1	Mascot

Calc. Mass	Obsrv. Mass	± da	± ppm	Start Seq.	End Seq.	Sequence	Ion Score	C. I. %	Modification	Plate [#] Name	Gel Idx/Pos [4700 Sample Name]	iTRAQ Ratio 115/114*	iTRAQ Ratio 116/114*	iTRAQ Ratio 117/114*	Rank	Result Type
									Lysine(K)_iTRAQ[12], MMTS (C)[7]							
1823.0038	1822.9709	-0.0329	-18	278	291	FGLEGCEVLIPALK	38	99.633	(N-term)_iTRAQ[0], Lysine(K)_iTRAQ[14], MMTS (C)[6]	[1] F3 030912	532/524	1.109	1.082	1.046	1	Mascot
1948.1405	1948.1073	-0.0332	-17	930	943	IEQLSPFPFDLLLK	64	99.999	(N-term)_iTRAQ[0], Lysine(K)_iTRAQ[14]	[1] F3 030912	554/546	1.127	1.451	1.749	1	Mascot
1953.9402	1954.0464	0.1062	54	390	402	AEQFYCGDTEGKK	61	99.998	(N-term)_iTRAQ[0], Lysine(K)_iTRAQ[12, 13], MMTS (C)[6]	[4] F7 and F10+11	1106/1098	1.080	0.910	1.298	1	Mascot
1963.0912	1963.0131	-0.0781	-40	1009	1021	FLDTAFDLDAFKK	85	100	(N-term)_iTRAQ[0], Lysine(K)_iTRAQ[12, 13]	[6] F8 110912	432/424	1.030	1.040	1.292	1	Mascot
2051.9546	2051.9487	-0.0059	-3	948	961	YPNAELAWCQEEHK	38	99.602	(N-term)_iTRAQ[0], Lysine(K)_iTRAQ[14], MMTS (C)[9]	[4] F7 and F10+11	329/321	0.734	0.638	0.684	1	Mascot
2093.0764	2093.1392	0.0628	30	324	336	KELEQIFCQFDSK	48	99.958	(N-term)_iTRAQ[0], Lysine(K)_iTRAQ[1,1 3], MMTS (C)[8]	[4] F7 and F10+11	1306/1298	0.657	0.951	1.035	1	Mascot
2110.188	2110.0161	-0.1719	-81	884	899	VIPEDGPAAQNPDKVK	27	95.273	(N-term)_iTRAQ[0], Lysine(K)_iTRAQ[14, 16]	[6] F8 110912	188/180	1.287	1.368	0.956	1	Mascot

70	serine/threonine-protein phosphatase 2B catalytic subunit alpha isoform [Rattus norvegicus]	gi|8394030	63625.3	10	644	0.957	0.915	0.874	0.154	0.186	0.258	11	11	11	100

Peptide Information

Calc. Mass	Obsrv. Mass	± da	± ppm	Start Seq.	End Seq.	Sequence	Ion Score	C. I. %	Modification	Plate [#] Name	Gel Idx/Pos [4700 Sample Name]	iTRAQ Ratio 115/114*	iTRAQ Ratio 116/114*	iTRAQ Ratio 117/114*	Rank	Result Type
1060.6123	1060.5277	-0.0846	-80	56	63	LEESVALR	28	96.28	(N-term)_iTRAQ[0]	[5] F4	133/125	0.893	0.751	1.097	1	Mascot
1216.7385	1216.6914	-0.0471	-39	64	73	IITEGASILR	46	99.934	(N-term)_iTRAQ[0]	[1] F3 030912	298/290	0.973	0.794	0.513	1	Mascot
1296.649	1296.5654	-0.0836	-64	324	332	YENNVMNIR	38	99.585	(N-term)_iTRAQ[0]	[5] F4	146/138	1.168	1.117	1.033	1	Mascot
1391.7402	1391.6711	-0.0691	-50	101	112	LFEVGGSPANTR	79	100	(N-term)_iTRAQ[0]	[1] F3 030912	232/224	0.758	0.709	0.614	1	Mascot
1404.7284	1404.6892	-0.0392	-28	113	122	YLFLGDYVDR	43	99.879	(N-term)_iTRAQ[0]	[1] F3 030912	391/383	1.058	1.012	0.832	1	Mascot
1422.7937	1422.6992	-0.0945	-66	415	424	EESESVLTLK	47	99.955	(N-term)_iTRAQ[0], Lysine(K)_iTRAQ[10]	[5] F4	152/144	1.028	1.122	0.854	1	Mascot
1422.7937	1422.7308	-0.0629	-44	415	424	EESESVLTLK	39	99.722	(N-term)_iTRAQ[0], Lysine(K)_iTRAQ[10]	[7] F5 120912	188/180	1.131	1.291	1.077	1	Mascot
1473.7988	1473.8448	0.046	31	155	163	HLTEYFTFK	59	99.997	(N-term)_iTRAQ[0], Lysine(K)_iTRAQ[9]	[4] F7 and F10+11	1216/1208	0.961	0.986	0.980	1	Mascot
1777.9	1777.7714	-0.1286	-72	488	501	DAMPSDANLNSINK	88	100	(N-term)_iTRAQ[0], Lysine(K)_iTRAQ[14]	[5] F4	142/134	0.823	0.808	1.325	1	Mascot
1860.051	1859.9926	-0.0584	-31	425	441	GLTPTGMLPSGVLSGGK	78	100	(N-term)_iTRAQ[0], Lysine(K)_iTRAQ[17]	[1] F3 030912	335/327	1.100	0.827	0.832	1	Mascot
2205.186	2205.1125	-0.0735	-33	442	459	QTLQSATVEAIEADEAIK	139	100	(N-term)_iTRAQ[0], Lysine(K)_iTRAQ[18]	[1] F3 030912	365/357	0.751	0.827	0.768	1	Mascot

71	heat shock protein 105 kDa [Rattus norvegicus]	gi|58865372	108523.2	10	638	0.946	0.908	0.921	0.296	0.358	0.348	11	11	11	100

Peptide Information

Calc. Mass	Obsrv. Mass	± da	± ppm	Start Seq.	End Seq.	Sequence	Ion Score	C. I. %	Modification	Plate [#] Name	Gel Idx/Pos [4700 Sample Name]	iTRAQ Ratio 115/114*	iTRAQ Ratio 116/114*	iTRAQ Ratio 117/114*	Rank	Result Type
1407.8206	1407.7161	-0.1045	-74	462	471	FVVQNVSAQK	69	100	(N-term)_iTRAQ[0], Lysine(K)_iTRAQ[10]	[5] F4	130/122	0.794	1.596	0.565	1	Mascot
1556.7832	1556.8167	0.0335	22	240	249	LVEHFCAEFK	70	100	(N-term)_iTRAQ[0], Lysine(K)_iTRAQ[10], MMTS (C)[6]	[3] F6 and F9	1370/1362	0.870	0.872	0.913	1	Mascot
1570.8435	1570.8672	0.0237	15	186	196	QDLPNADEKPR	48	99.965	(N-term)_iTRAQ[0], Lysine(K)_iTRAQ[9]	[3] F6 and F9	1086/1078	0.731	0.674	0.837	1	Mascot
1623.8098	1623.7545	-0.0553	-34	20	33	AGGIETIANEFSDR	59	99.997	(N-term)_iTRAQ[0]	[1] F3 030912	331/323	1.190	0.953	0.911	1	Mascot
1761.9456	1761.9307	-0.0149	-8	155	169	SVLDAAQIVGLNCLR	56	99.994	(N-term)_iTRAQ[0], MMTS (C)[13]	[8] F13-15 and F1+2	294/286	0.742	0.855	0.680	1	Mascot
1871.9474	1871.8962	-0.0512	-27	304	316	SQFEELCAELLQK	79	100	(N-term)_iTRAQ[0], Lysine(K)_iTRAQ[13], MMTS (C)[7]	[5] F4	414/406	1.166	0.843	1.012	1	Mascot
1871.9474	1871.9393	-0.0081	-4	304	316	SQFEELCAELLQK	56	99.993	(N-term)_iTRAQ[0], Lysine(K)_iTRAQ[13], MMTS (C)[7]	[7] F5 120912	512/504	0.792	1.166	1.223	1	Mascot
1913.0789	1913.1458	0.0669	35	687	698	QAYIDKLEELMK	43	99.877	(N-term)_iTRAQ[0], Lysine(K)_iTRAQ[6,1 2]	[4] F7 and F10+11	1303/1295	1.482	1.642	1.945	1	Mascot
1977.0399	1976.9119	-0.128	-65	54	68	NQQITHANNTVSSFK	87	100	(N-term)_iTRAQ[0], Lysine(K)_iTRAQ[15]	[6] F8 110912	183/175	1.520	0.612	1.118	1	Mascot
2089.209	2089.3196	0.1106	53	317	331	IEVPLHLLMEQTHLK	100	100	(N-term)_iTRAQ[0], Lysine(K)_iTRAQ[15]	[4] F7 and F10+11	1313/1305	0.810	0.793	0.835	1	Mascot
2179.2358	2179.2271	-0.0087	-4	592	609	VVNVELPVEANLVWQLG R	28	95.986	(N-term)_iTRAQ[0]	[8] F13-15 and F1+2	284/276	0.720	0.576	0.675	1	Mascot

72	optic atrophy 1-like protein [Rattus norvegicus]	gi|37812499	123742	13	633	0.871	1.082	1.124	0.213	0.307	0.345	13	13	13	100

Peptide Information

Calc. Mass	Obsrv. Mass	± da	± ppm	Start Seq.	End Seq.	Sequence	Ion Score	C. I. %	Modification	Plate [#] Name	Gel Idx/Pos [4700 Sample Name]	iTRAQ Ratio 115/114*	iTRAQ Ratio 116/114*	iTRAQ Ratio 117/114*	Rank	Result Type
1083.6238	1083.6249	0.0011	1	775	779	WIYWK	29	96.955	(N-term)_iTRAQ[0], Lysine(K)_iTRAQ[5]	[4] F7 and F10+11	356/348	0.728	0.930	1.271	1	Mascot
1344.7985	1344.7229	-0.0756	-56	825	834	GVEVDPSLIK	31	97.859	(N-term)_iTRAQ[0], Lysine(K)_iTRAQ[10]	[5] F4	168/160	0.947	1.011	1.169	1	Mascot
1555.785	1555.7579	-0.0271	-17	558	568	ESVEQQADSFK	68	100	(N-term)_iTRAQ[0], Lysine(K)_iTRAQ[11]	[7] F5 120912	159/151	1.021	1.932	1.467	1	Mascot
1646.7935	1646.8013	0.0078	5	181	194	DFFTAGSPGETAFR	39	99.696	(N-term)_iTRAQ[0]	[8] F13-15 and F1+2	462/454	0.474	1.247	0.853	1	Mascot
1668.8214	1668.7266	-0.0948	-57	912	923	EVLEDFAEDGEK	44	99.899	(N-term)_iTRAQ[0], Lysine(K)_iTRAQ[12]	[5] F4	192/184	0.749	0.622	0.671	1	Mascot
1701.9421	1701.873	-0.0691	-41	949	960	LDAFIEALHQEK	67	99.999	(N-term)_iTRAQ[0], Lysine(K)_iTRAQ[12]	[6] F8 110912	410/402	0.991	1.045	1.025	1	Mascot
1737.8218	1737.7434	-0.0784	-45	517	527	EYEEEFFQNSK	46	99.945	(N-term)_iTRAQ[0], Lysine(K)_iTRAQ[11]	[7] F5 120912	230/222	0.924	0.957	1.006	1	Mascot
1897.9681	1897.8872	-0.0809	-43	615	627	HWEEILQQSLWER	41	99.792	(N-term)_iTRAQ[0]	[6] F8 110912	447/439	0.947	1.066	1.169	1	Mascot
1923.9573	1923.9088	-0.0485	-25	669	683	AVEVAWETLQDEFSR	73	100	(N-term)_iTRAQ[0]	[1] F3 030912	508/500	0.828	0.890	1.239	1	Mascot
1938.043	1938.0187	-0.0243	-13	347	360	EFDLTKEEDLAALR	59	99.997	(N-term)_iTRAQ[0], Lysine(K)_iTRAQ[6]	[4] F7 and F10+11	341/333	1.142	1.113	1.197	1	Mascot
1996.1324	1996.1334	0.001	1	215	228	IDQLQEELLHTQLK	77	100	(N-term)_iTRAQ[0], Lysine(K)_iTRAQ[14]	[4] F7 and F10+11	383/375	0.803	1.115	1.126	1	Mascot
2174.085	2173.9922	-0.0928	-43	801	818	VNDEHPAYLASDEITTVR	27	95.579	(N-term)_iTRAQ[0]	[7] F5 120912	222/214	0.921	1.291	2.109	1	Mascot
2200.0618	2200.0254	-0.0364	-17	739	755	QQWDAAIYFMEEALQGR	37	99.452	(N-term)_iTRAQ[0]	[1] F3 030912	564/556	1.090	1.294	0.859	1	Mascot

73	Solute carrier family 25 (mitochondrial carrier; adenine nucleotide translocator), member 4 [Rattus	gi|38014819	36669.7	12	627	0.816	0.756	0.827	0.107	0.308	0.252	13	13	13	100

Protein Group

ADP/ATP translocase 1 [Rattus norvegicus] gi|32189355 36754.7

Peptide Information

Calc. Mass	Obsrv. Mass	± da	± ppm	Start Seq.	End Seq.	Sequence	Ion Score	C. I. %	Modification	Plate [#] Name	Gel Idx/Pos [4700 Sample Name]	iTRAQ Ratio 115/114*	iTRAQ Ratio 116/114*	iTRAQ Ratio 117/114*	Rank	Result Type
961.5928	961.5514	-0.0414	-43	141	147	LAADVGK	32	98.307	(N-term)_iTRAQ[0], Lysine(K)_iTRAQ[7]	[3] F6 and F9	148/140	0.803	0.727	0.511	1	Mascot
1046.5867	1046.5386	-0.0481	-46	273	280	GAWSNVLR	38	99.605	(N-term)_iTRAQ[0]	[7] F5 120912	225/217	0.873	0.909	0.751	1	Mascot
1148.6548	1148.5874	-0.0674	-59	97	105	QIFLGGVDR	47	99.954	(N-term)_iTRAQ[0]	[5] F4	170/162	0.662	0.825	0.809	1	Mascot
1313.6763	1313.6611	-0.0152	-12	64	72	EQGFLSFWR	45	99.915	(N-term)_iTRAQ[0]	[7] F5 120912	401/393	0.885	0.911	0.916	1	Mascot
1424.8835	1424.818	-0.0655	-46	34	43	LLLQVQHASK	54	99.991	(N-term)_iTRAQ[0], Lysine(K)_iTRAQ[10]	[6] F8 110912	244/236	0.653	0.436	0.690	1	Mascot
1493.7886	1493.7202	-0.0684	-46	189	199	AAYFGVYDTAK	54	99.991	(N-term)_iTRAQ[0], Lysine(K)_iTRAQ[11]	[7] F5 120912	219/211	0.832	0.914	1.112	1	Mascot
1507.8729	1507.8242	-0.0487	-32	11	23	DFLAGGIAAAVSK	80	100	(N-term)_iTRAQ[0], Lysine(K)_iTRAQ[13]	[5] F4	265/257	0.845	1.009	1.006	1	Mascot
1683.958	1684.0256	0.0676	40	269	280	AFFKGAWSNVLR	32	98.505	(N-term)_iTRAQ[0], Lysine(K)_iTRAQ[4]	[4] F7 and F10+11	1271/1263	0.712	0.868	1.080	1	Mascot
1734.9465	1734.8595	-0.087	-50	81	92	YFPTQALNFAFK	56	99.994	(N-term)_iTRAQ[0], Lysine(K)_iTRAQ[12]	[6] F8 110912	391/383	0.886	1.042	0.759	1	Mascot
1734.9465	1734.8595	-0.087	-50	81	92	YFPTQALNFAFK	61	99.998	(N-term)_iTRAQ[0], Lysine(K)_iTRAQ[12]	[5] F4	343/335	1.011	1.048	1.017	1	Mascot
1777.7812	1777.735	-0.0462	-26	246	259	GADIMYTGTVDCWR	47	99.949	(N-term)_iTRAQ[0], MMTS (C)[12]	[1] F3 030912	383/375	0.868	0.771	0.532	1	Mascot
1900.0499	1900.0328	-0.0171	-9	281	295	GMGGAFVLVLYDEIK	79	100	(N-term)_iTRAQ[0], Lysine(K)_iTRAQ[15]	[5] F4	412/404	0.904	0.539	0.698	1	Mascot
2172.2473	2172.3198	0.0725	33	281	296	GMGGAFVLVLYDEIKK	58	99.996	(N-term)_iTRAQ[0], Lysine(K)_iTRAQ[15, 16]	[4] F7 and F10+11	1355/1347	0.756	0.333	1.238	1	Mascot

74	GluT-R glutamate transporter [Rattus norvegicus]	gi|705397	53461.5	9	621	0.819	0.977	1.015	0.217	0.281	0.332	13	13	13	100

Protein Group

excitatory amino acid transporter 2 isoform a [Rattus norvegicus] gi|78126167 67249

Peptide Information

Calc. Mass	Obsrv. Mass	± da	± ppm	Start Seq.	End Seq.	Sequence	Ion Score	C. I. %	Modification	Plate [#] Name	Gel Idx/Pos [4700 Sample Name]	iTRAQ Ratio 115/114*	iTRAQ Ratio 116/114*	iTRAQ Ratio 117/114*	Rank	Result Type
1358.705	1358.6888	-0.0162	-12	398	406	TQSIYDDTK	34	98.907	(N-term)_iTRAQ[0], Lysine(K)_iTRAQ[9]	[7] F5 120912	111/103	0.890	0.622	1.569	1	Mascot
1421.7014	1421.7634	0.062	44	389	397	MHEDIEMTK	65	99.999	(N-term)_iTRAQ[0], Lysine(K)_iTRAQ[9]	[4] F7 and F10+11	1065/1057	0.646	0.957	1.178	1	Mascot

1444.7152	1444.6207	-0.0945	-65	378	388	SELDTIDSQHR	78	100	(N-term)_iTRAQ[0]	[6] F8 110912	152/144	1.202	1.143	1.281	1	Mascot
1609.8269	1609.748	-0.0789	-49	253	263	CLEDNLGIDKR	50	99.975	(N-term)_iTRAQ[0], Lysine(K)_iTRAQ[10], MMTS (C)[1]	[6] F8 110912	259/251	0.625	0.579	0.597	1	Mascot
1609.8269	1609.8842	0.0573	36	253	263	CLEDNLGIDKR	39	99.723	(N-term)_iTRAQ[0], Lysine(K)_iTRAQ[10], MMTS (C)[1]	[3] F6 and F9	1217/1209	0.923	1.127	1.092	1	Mascot
1713.769	1713.7117	-0.0573	-33	439	450	SADCSVEEEPWK	67	100	(N-term)_iTRAQ[0], Lysine(K)_iTRAQ[12], MMTS (C)[4]	[7] F5 120912	227/219	0.722	1.011	0.831	1	Mascot
1850.962	1850.9292	-0.0328	-18	39	53	NDEVSSLDAFLDLIR	85	100	(N-term)_iTRAQ[0]	[1] F3 030912	543/535	0.681	1.108	0.875	1	Mascot
1850.962	1850.936	-0.026	-14	39	53	NDEVSSLDAFLDLIR	76	100	(N-term)_iTRAQ[0]	[8] F13-15 and F1+2	251/243	0.873	0.957	0.907	1	Mascot
1869.8701	1869.8956	0.0255	14	439	451	SADCSVEEEPWKR	69	100	(N-term)_iTRAQ[0], Lysine(K)_iTRAQ[12], MMTS (C)[4]	[3] F6 and F9	1201/1193	0.624	0.895	0.998	1	Mascot
1869.8701	1869.9639	0.0938	50	439	451	SADCSVEEEPWKR	60	99.998	(N-term)_iTRAQ[0], Lysine(K)_iTRAQ[12], MMTS (C)[4]	[4] F7 and F10+11	1135/1127	1.163	1.154	1.312	1	Mascot
2123.1594	2123.0957	-0.0637	-30	38	53	KNDEVSSLDAFLDLIR	71	100	(N-term)_iTRAQ[0], Lysine(K)_iTRAQ[1]	[6] F8 110912	513/505	0.707	1.018	0.840	1	Mascot
2123.1594	2123.2085	0.0491	23	38	53	KNDEVSSLDAFLDLIR	98	100	(N-term)_iTRAQ[0], Lysine(K)_iTRAQ[1]	[4] F7 and F10+11	520/512	0.722	0.922	0.703	1	Mascot
2655.3865	2655.3411	-0.0454	-17	54	73	NLFPENLVQACFQQIQTV TK	75	100	(N-term)_iTRAQ[0], Lysine(K)_iTRAQ[20], MMTS (C)[11]	[1] F3 030912	595/587	1.178	1.606	1.545	1	Mascot

75	14-3-3 protein beta/alpha [Rattus norvegicus]	gi|9507243	31155	9	615	1.113	1.057	1.004	0.469	0.443	0.315	11	11	11	100

Protein Group

RNH-1/14-3-3 protein	gi|1585294	31146.9

Peptide Information

Calc. Mass	Obsrv. Mass	± da	± ppm	Start Seq.	End Seq.	Sequence	Ion Score	C. I. %	Modification	Plate [#] Name	Gel Idx/Pos [4700 Sample Name]	iTRAQ Ratio 115/114*	iTRAQ Ratio 116/114*	iTRAQ Ratio 117/114*	Rank	Result Type
957.6019	957.6076	0.0057	6	118	122	VFYLK	34	98.922	(N-term)_iTRAQ[0], Lysine(K)_iTRAQ[5]	[3] F6 and F9	262/254	1.285	1.183	1.199	1	Mascot
1191.7195	1191.6932	-0.0263	-22	63	70	VISSIEQK	55	99.992	(N-term)_iTRAQ[0], Lysine(K)_iTRAQ[8]	[7] F5 120912	159/151	1.339	1.437	1.443	1	Mascot
1195.7296	1195.6782	-0.0514	-43	44	51	NLLSVAYK	53	99.987	(N-term)_iTRAQ[0], Lysine(K)_iTRAQ[8]	[7] F5 120912	233/225	1.100	1.296	1.119	1	Mascot
1333.7634	1333.7305	-0.0329	-25	215	224	DSTLIMQLLR	42	99.831	(N-term)_iTRAQ[0]	[1] F3 030912	466/458	1.966	2.108	0.925	1	Mascot
1333.7634	1333.7313	-0.0321	-24	215	224	DSTLIMQLLR	48	99.963	(N-term)_iTRAQ[0]	[2] F12 040912	472/464	1.159	0.774	0.827	1	Mascot
1470.7686	1470.6964	-0.0722	-49	130	140	YLSEVASGDNK	58	99.996	(N-term)_iTRAQ[0], Lysine(K)_iTRAQ[11]	[7] F5 120912	174/166	1.189	1.002	1.042	1	Mascot
1647.9315	1647.9095	-0.022	-13	106	117	YLILNATHAESK	52	99.986	(N-term)_iTRAQ[0], Lysine(K)_iTRAQ[12]	[4] F7 and F10+11	262/254	0.531	0.549	0.514	1	Mascot
1742.8429	1742.7101	-0.1328	-76	30	43	AVTEQGHELSNEER	31	98.025	(N-term)_iTRAQ[0]	[6] F8 110912	120/112	1.420	0.728	0.874	1	Mascot
1742.8429	1742.7874	-0.0555	-32	30	43	AVTEQGHELSNEER	63	99.999	(N-term)_iTRAQ[0]	[4] F7 and F10+11	113/105	0.631	1.229	1.402	1	Mascot
2447.2075	2447.1375	-0.07	-29	196	214	TAFDEAIAELDTLNEESY K	141	100	(N-term)_iTRAQ[0], Lysine(K)_iTRAQ[19]	[1] F3 030912	509/501	1.335	1.155	1.143	1	Mascot
2447.23	2447.1243	-0.1057	-43	141	159	QTTVSNSQQAYQEAFEIS K	114	100	(N-term)_iTRAQ[0], Lysine(K)_iTRAQ[19]	[1] F3 030912	276/268	0.994	0.900	0.944	1	Mascot

76	RecName: Full=4-aminobutyrate aminotransferase, mitochondrial; AltName: Full=(S)-3-amino-2-methylpr	gi|122065191	61484.4	12	607	0.898	1.002	1.066	0.612	0.508	0.697	14	14	14	100

Peptide Information

Calc. Mass	Obsrv. Mass	± da	± ppm	Start Seq.	End Seq.	Sequence	Ion Score	C. I. %	Modification	Plate [#] Name	Gel Idx/Pos [4700 Sample Name]	iTRAQ Ratio 115/114*	iTRAQ Ratio 116/114*	iTRAQ Ratio 117/114*	Rank	Result Type
1343.6849	1343.762	0.0771	57	359	367	MMTGGFFHK	55	99.993	(N-term)_iTRAQ[0], Lysine(K)_iTRAQ[9]	[4] F7 and F10+11	1165/1157	0.861	0.864	1.011	1	Mascot
1365.6882	1365.5725	-0.1157	-85	82	92	GNYLVDVDGNR	39	99.72	(N-term)_iTRAQ[0]	[5] F4	138/130	0.901	1.232	0.994	1	Mascot
1395.7141	1395.7339	0.0198	14	368	377	EEFRPSAPYR	33	98.856	(N-term)_iTRAQ[0]	[3] F6 and F9	1142/1134	1.178	0.910	0.897	1	Mascot
1565.8574	1565.8184	-0.039	-25	378	388	IFNTWLGDPSK	41	99.822	(N-term)_iTRAQ[0], Lysine(K)_iTRAQ[11]	[7] F5 120912	340/332	3.755	3.080	4.426	1	Mascot
1623.8564	1623.8096	-0.0468	-29	269	279	CLEEVEDLIVK	47	99.948	(N-term)_iTRAQ[0], Lysine(K)_iTRAQ[11], MMTS (C)[1]	[5] F4	384/376	0.898	1.067	1.068	1	Mascot
1641.056	1641.0121	-0.0439	-27	389	400	NLLLAEVINIIK	40	99.729	(N-term)_iTRAQ[0], Lysine(K)_iTRAQ[12]	[5] F4	428/420	0.572	0.602	0.464	1	Mascot
1641.056	1641.0173	-0.0387	-24	389	400	NLLLAEVINIIK	63	99.999	(N-term)_iTRAQ[0], Lysine(K)_iTRAQ[12]	[1] F3 030912	562/554	0.587	0.602	0.862	1	Mascot
1748.7725	1748.7274	-0.0451	-26	437	450	GTFCSFDTPDEAIR	37	99.538	(N-term)_iTRAQ[0], MMTS (C)[4]	[1] F3 030912	354/346	0.734	1.060	0.993	1	Mascot
1748.7725	1748.7804	0.0079	5	437	450	GTFCSFDTPDEAIR	41	99.79	(N-term)_iTRAQ[0], MMTS (C)[4]	[8] F13-15 and F1+2	424/416	1.146	1.490	1.498	1	Mascot

Calc. Mass	Obsrv. Mass	± da	± ppm	Start Seq.	End Seq.	Sequence	Ion Score	C. I. %	Modification	Plate [#] Name	Gel Idx/Pos [4700 Sample Name]	iTRAQ Ratio 115/114*	iTRAQ Ratio 116/114*	iTRAQ Ratio 117/114*	Rank	Result Type
1863.9084	1863.8134	-0.095	-51	35	47	VDFEFDYDGPLMK	66	99.999	(N-term)_iTRAQ[0], Lysine(K)_iTRAQ[13]	[5] F4	306/298	0.814	0.725	1.179	1	Mascot
1915.0238	1914.9795	-0.0443	-23	236	250	IDIPSFDWPIAPFPR	50	99.973	(N-term)_iTRAQ[0]	[1] F3 030912	530/522	1.202	0.900	1.086	1	Mascot
2531.2212	2531.292	0.0708	28	318	337	KHGCAFLVDEVQTGGGC TGK	35	99.281	(N-term)_iTRAQ[0], Lysine(K)_iTRAQ[1,2 0], MMTS (C)[4,17]	[4] F7 and F10+11	1245/1237	0.320	0.847	0.792	1	Mascot
2663.2598	2663.345	0.0852	32	338	357	FWAHEHWGLDDPADVM SFSK	41	99.8	(N-term)_iTRAQ[0], Lysine(K)_iTRAQ[20]	[4] F7 and F10+11	1299/1291	0.811	1.374	1.615	1	Mascot
2818.3767	2818.2815	-0.0952	-34	286	310	TVAGIIVEPIQSEGGDNH ASDDFFR	95	100	(N-term)_iTRAQ[0]	[1] F3 030912	385/377	0.998	0.740	0.589	1	Mascot

77	protein kinase C and casein kinase substrate in neurons protein 1 [Rattus norvegicus]	gi|8393896	57034	10	595	1.041	0.921	0.924	0.398	0.365	0.213	10	10	10	100

Peptide Information

Calc. Mass	Obsrv. Mass	± da	± ppm	Start Seq.	End Seq.	Sequence	Ion Score	C. I. %	Modification	Plate [#] Name	Gel Idx/Pos [4700 Sample Name]	iTRAQ Ratio 115/114*	iTRAQ Ratio 116/114*	iTRAQ Ratio 117/114*	Rank	Result Type
1117.7079	1117.6683	-0.0396	-35	244	250	EVLLDIK	41	99.81	(N-term)_iTRAQ[0], Lysine(K)_iTRAQ[7]	[7] F5 120912	245/237	1.069	0.570	0.718	1	Mascot
1498.728	1498.7112	-0.0168	-11	80	90	AWGAMMTEADK	86	100	(N-term)_iTRAQ[0], Lysine(K)_iTRAQ[11]	[7] F5 120912	239/231	1.057	0.950	1.025	1	Mascot
1511.636	1511.5593	-0.0767	-51	412	422	LGEEDEQGWCR	59	99.997	(N-term)_iTRAQ[0], MMTS (C)[10]	[5] F4	197/189	1.746	1.599	1.103	1	Mascot
1582.8475	1582.8372	-0.0103	-7	52	62	AYAQQLTDWAK	55	99.991	(N-term)_iTRAQ[0], Lysine(K)_iTRAQ[11]	[7] F5 120912	259/251	1.357	0.888	0.877	1	Mascot
1646.6915	1646.6437	-0.0478	-29	35	46	LCNDLMSCVQER	32	98.512	(N-term)_iTRAQ[0], MMTS (C)[2,8]	[1] F3 030912	485/477	0.623	0.645	0.667	1	Mascot
1669.8759	1669.9882	0.1123	67	156	165	AYHLACKEEK	33	98.892	(N-term)_iTRAQ[0], Lysine(K)_iTRAQ[7,1 0], MMTS (C)[6]	[8] F13-15 and F1+2	1211/1203	1.500	1.223	1.238	1	Mascot
1960.0161	1960.1202	0.1041	53	252	266	HLNLAENSSYIHVYR	63	99.999	(N-term)_iTRAQ[0]	[4] F7 and F10+11	1165/1157	0.758	0.656	0.905	1	Mascot
2209.0547	2208.9653	-0.0894	-40	389	404	ALYDYDGQEQDELSFK	51	99.98	(N-term)_iTRAQ[0], Lysine(K)_iTRAQ[16]	[1] F3 030912	313/305	0.687	0.775	0.714	1	Mascot
2479.2634	2479.2566	-0.0068	-3	319	341	KAEGAALSNATGAVEST SQAGDR	75	100	(N-term)_iTRAQ[0], Lysine(K)_iTRAQ[1]	[4] F7 and F10+11	210/202	0.933	0.968	1.021	1	Mascot
3044.3376	3044.2566	-0.081	-27	216	237	TTPQYMEGMEQVFEQC QQFEEK	99	100	(N-term)_iTRAQ[0], Lysine(K)_iTRAQ[22], MMTS (C)[16]	[1] F3 030912	533/525	1.231	1.472	1.165	1	Mascot

78	neural adhesion molecule F3	gi|1095168	122312.7	12	591	1.096	0.927	0.923	0.247	0.235	0.287	12	12	12	100

Protein Group

contactin-1 precursor [Rattus norvegicus]	gi|16923964	122326.7

Peptide Information

Calc. Mass	Obsrv. Mass	± da	± ppm	Start Seq.	End Seq.	Sequence	Ion Score	C. I. %	Modification	Plate [#] Name	Gel Idx/Pos [4700 Sample Name]	iTRAQ Ratio 115/114*	iTRAQ Ratio 116/114*	iTRAQ Ratio 117/114*	Rank	Result Type
1196.6925	1196.6445	-0.048	-40	69	76	ASPFPVYK	33	98.9	(N-term)_iTRAQ[0], Lysine(K)_iTRAQ[8]	[7] F5 120912	194/186	0.746	0.527	0.466	1	Mascot
1265.6987	1265.6843	-0.0144	-11	645	652	TILSDDWK	38	99.593	(N-term)_iTRAQ[0], Lysine(K)_iTRAQ[8]	[3] F6 and F9	282/274	1.788	1.413	1.159	1	Mascot
1317.7035	1317.7914	0.0879	67	552	560	EITNIHYQR	49	99.968	(N-term)_iTRAQ[0]	[3] F6 and F9	1128/1120	0.945	0.929	0.994	1	Mascot
1338.8269	1338.8458	0.0189	14	226	235	FIPLIPIPER	32	98.353	(N-term)_iTRAQ[0]	[8] F13-15 and F1+2	386/378	1.218	1.027	0.910	1	Mascot
1430.8127	1430.7643	-0.0484	-34	724	734	ELTITWAPLSR	44	99.898	(N-term)_iTRAQ[0]	[1] F3 030912	370/362	0.887	0.970	0.753	1	Mascot
1686.9174	1686.9116	-0.0058	-3	944	955	ILYRPDGQHDGK	39	99.688	(N-term)_iTRAQ[0], Lysine(K)_iTRAQ[12]	[2] F12 040912	165/157	1.168	1.023	0.968	1	Mascot
1763.7582	1763.6985	-0.0597	-34	477	490	NDGGIYTCFAENNR	52	99.984	(N-term)_iTRAQ[0], MMTS (C)[8]	[1] F3 030912	310/302	1.147	0.770	0.990	1	Mascot
1789.9252	1789.8071	-0.1181	-66	656	669	TDPPIIEGNMESAK	74	100	(N-term)_iTRAQ[0], Lysine(K)_iTRAQ[14]	[5] F4	145/137	1.103	0.993	1.000	1	Mascot
1812.9115	1812.8728	-0.0387	-21	670	682	AVDLIPWMEYEFR	39	99.686	(N-term)_iTRAQ[0]	[1] F3 030912	529/521	1.128	0.940	0.724	1	Mascot
2039.1913	2039.1509	-0.0404	-20	236	249	TTKPYPADIVVQFK	70	100	(N-term)_iTRAQ[0], Lysine(K)_iTRAQ[3,1 4]	[4] F7 and F10+11	334/326	1.200	1.110	1.390	1	Mascot
2399.2524	2399.1948	-0.0576	-24	701	723	IKTDGAAPNVAPSDVGG GGGTNR	76	100	(N-term)_iTRAQ[0], Lysine(K)_iTRAQ[2]	[4] F7 and F10+11	177/169	1.127	0.891	1.213	1	Mascot
2414.2761	2414.3894	0.1133	47	769	785	YVHKDETMTPSTAFQVK	48	99.963	(N-term)_iTRAQ[0], Lysine(K)_iTRAQ[4,1 7]	[4] F7 and F10+11	1140/1132	0.982	0.801	0.888	1	Mascot

79	phosphoglycerate kinase 1 [Rattus norvegicus]	gi|40254752	50884.2	12	586	1.033	1.043	0.988	0.528	0.510	0.330	14	14	14	100

Protein Group

phosphoglycerate kinase [Rattus norvegicus]	gi|206113	50756

Peptide Information

Calc. Mass	Obsrv. Mass	± da	± ppm	Start Seq.	End Seq.	Sequence	Ion Score	C. I. %	Modification	Plate [#] Name	Gel Idx/Pos [4700 Sample Name]	iTRAQ Ratio 115/114*	iTRAQ Ratio 116/114*	iTRAQ Ratio 117/114*	Rank	Result Type
1011.5906	1011.5665	-0.0241	-24	185	191	AGGFLMK	34	98.959	(N-term)_iTRAQ[0], Lysine(K)_iTRAQ[7]	[3] F6 and F9	229/221	1.414	1.155	1.102	1	Mascot
1172.6561	1172.6498	-0.0063	-5	193	199	ELNYFAK	43	99.881	(N-term)_iTRAQ[0], Lysine(K)_iTRAQ[7]	[3] F6 and F9	240/232	0.929	0.932	1.026	1	Mascot
1208.6807	1208.657	-0.0237	-20	354	361	SLMDEVVK	31	97.937	(N-term)_iTRAQ[0], Lysine(K)_iTRAQ[8]	[7] F5 120912	212/204	0.801	1.072	0.990	1	Mascot
1237.686	1237.6414	-0.0446	-36	23	30	VDFNVPMK	48	99.965	(N-term)_iTRAQ[0], Lysine(K)_iTRAQ[8]	[7] F5 120912	236/228	0.615	0.975	1.049	1	Mascot
1444.8535	1444.925	0.0715	49	192	199	KELNYFAK	31	98.043	(N-term)_iTRAQ[0], Lysine(K)_iTRAQ[1,8]	[4] F7 and F10+11	1105/1097	0.855	0.897	0.894	1	Mascot
1507.8617	1507.7834	-0.0783	-52	76	86	YSLEPVAAELK	53	99.987	(N-term)_iTRAQ[0], Lysine(K)_iTRAQ[11]	[5] F4	227/219	1.457	1.141	1.530	1	Mascot
1507.8617	1507.8063	-0.0554	-37	76	86	YSLEPVAAELK	52	99.985	(N-term)_iTRAQ[0], Lysine(K)_iTRAQ[11]	[1] F3 030912	305/297	0.678	0.478	0.565	1	Mascot
1591.8815	1591.9926	0.1111	70	124	133	FHVEEEGKGK	55	99.991	(N-term)_iTRAQ[0], Lysine(K)_iTRAQ[8,10]	[8] F13-15 and F1+2	1261/1253	0.606	0.983	1.028	1	Mascot
1778.8945	1778.8485	-0.046	-26	157	171	LGDVYVNDAFGTAHR	30	97.548	(N-term)_iTRAQ[0]	[3] F6 and F9	301/293	2.382	2.890	1.150	1	Mascot
1778.8945	1778.8489	-0.0456	-26	157	171	LGDVYVNDAFGTAHR	31	98.148	(N-term)_iTRAQ[0]	[4] F7 and F10+11	295/287	1.317	1.026	0.717	1	Mascot
2047.1957	2047.1024	-0.0933	-46	217	230	VADKIQLINNMLDK	65	99.999	(N-term)_iTRAQ[0], Lysine(K)_iTRAQ[4,14]	[6] F8 110912	413/405	0.812	0.800	0.764	1	Mascot
2057.2004	2057.1741	-0.0263	-13	200	216	ALESPERPFLAILGGAK	34	98.968	(N-term)_iTRAQ[0], Lysine(K)_iTRAQ[17]	[3] F6 and F9	427/419	1.031	0.911	0.958	1	Mascot
2271.1423	2271.0583	-0.084	-37	247	264	VLNNMEIGTSLYDEEGAK	112	100	(N-term)_iTRAQ[0], Lysine(K)_iTRAQ[18]	[1] F3 030912	327/319	0.870	0.815	0.814	1	Mascot
2455.3455	2455.3569	0.0114	5	280	297	ITLPVDFVTADKFDENAK	52	99.983	(N-term)_iTRAQ[0], Lysine(K)_iTRAQ[12,18]	[4] F7 and F10+11	411/403	2.072	1.902	1.856	1	Mascot

80 RecName: Full=Glycogen phosphorylase, brain form gi|1730559 104492.4 13 586 1.064 1.189 0.907 0.412 0.425 0.454 14 14 14 100

Peptide Information

Calc. Mass	Obsrv. Mass	± da	± ppm	Start Seq.	End Seq.	Sequence	Ion Score	C. I. %	Modification	Plate [#] Name	Gel Idx/Pos [4700 Sample Name]	iTRAQ Ratio 115/114*	iTRAQ Ratio 116/114*	iTRAQ Ratio 117/114*	Rank	Result Type
1110.6405	1110.5835	-0.057	-51	546	552	FSAQLEK	32	98.35	(N-term)_iTRAQ[0], Lysine(K)_iTRAQ[7]	[3] F6 and F9	196/188	1.239	1.213	1.157	1	Mascot
1197.6752	1197.6108	-0.0644	-54	643	650	VIFLENYR	28	96.365	(N-term)_iTRAQ[0]	[5] F4	247/239	0.580	0.911	0.452	1	Mascot
1282.7882	1282.8562	0.068	53	35	42	HLHFTLVK	45	99.915	(N-term)_iTRAQ[0], Lysine(K)_iTRAQ[8]	[8] F13-15 and F1+2	1500/1492	1.007	1.861	1.229	1	Mascot
1282.7882	1282.8735	0.0853	66	35	42	HLHFTLVK	49	99.97	(N-term)_iTRAQ[0], Lysine(K)_iTRAQ[8]	[8] F13-15 and F1+2	1158/1150	1.059	1.413	1.473	1	Mascot
1433.7675	1433.7289	-0.0386	-27	162	170	YEFGIFNQK	40	99.727	(N-term)_iTRAQ[0], Lysine(K)_iTRAQ[9]	[7] F5 120912	298/290	1.004	1.344	0.904	1	Mascot
1457.741	1457.641	-0.1	-69	471	479	DFYELEPEK	51	99.981	(N-term)_iTRAQ[0], Lysine(K)_iTRAQ[9]	[5] F4	177/169	1.387	1.462	0.990	1	Mascot
1483.7817	1483.7682	-0.0135	-9	51	61	DYFFALAHTVR	55	99.992	(N-term)_iTRAQ[0]	[4] F7 and F10+11	392/384	2.226	2.193	1.247	1	Mascot
1730.8999	1730.8179	-0.082	-47	279	290	VLYPNDNFFEGK	28	96.263	(N-term)_iTRAQ[0], Lysine(K)_iTRAQ[12]	[5] F4	247/239	0.898	0.890	1.046	1	Mascot
1769.8829	1769.8638	-0.0191	-11	257	270	DFNVGDYIEAVLDR	50	99.977	(N-term)_iTRAQ[0]	[8] F13-15 and F1+2	264/256	1.498	1.555	1.515	1	Mascot
1798.9585	1798.8844	-0.0741	-41	741	754	QAVDQISSGFFSPK	64	99.999	(N-term)_iTRAQ[0], Lysine(K)_iTRAQ[14]	[5] F4	220/212	1.024	0.764	0.458	1	Mascot
1805.9419	1806.0172	0.0753	42	411	425	HLDHVAALFPGDVDR	50	99.978	(N-term)_iTRAQ[0]	[4] F7 and F10+11	1186/1178	1.312	1.068	1.178	1	Mascot
1807.0575	1807.0911	0.0336	19	521	533	KLLSLVDDEAFIR	34	98.985	(N-term)_iTRAQ[0], Lysine(K)_iTRAQ[1]	[4] F7 and F10+11	427/419	0.698	0.851	0.460	1	Mascot
1879.9761	1880.0508	0.0747	40	193	206	ARPEYMLPVHFYGR	28	95.912	(N-term)_iTRAQ[0]	[4] F7 and F10+11	1218/1210	0.753	0.890	0.740	1	Mascot
1981.1705	1981.0642	-0.1063	-54	508	521	IGEGFLTDLSQLKK	80	100	(N-term)_iTRAQ[0], Lysine(K)_iTRAQ[13,14]	[6] F8 110912	424/416	1.062	1.058	0.829	1	Mascot

81 78 kDa glucose-regulated protein precursor [Rattus norvegicus] gi|25742763 81374.7 10 582 1.187 1.103 1.203 0.319 0.349 0.525 12 12 12 100

Peptide Information

Calc. Mass	Obsrv. Mass	± da	± ppm	Start Seq.	End Seq.	Sequence	Ion Score	C. I. %	Modification	Plate [#] Name	Gel Idx/Pos [4700 Sample Name]	iTRAQ Ratio 115/114*	iTRAQ Ratio 116/114*	iTRAQ Ratio 117/114*	Rank	Result Type
1361.7332	1361.6792	-0.054	-40	186	197	DAGTIAGLNVMR	34	98.947	(N-term)_iTRAQ[0]	[1] F3 030912	265/257	1.201	0.760	0.712	1	Mascot
1479.8417	1479.9409	0.0992	67	465	474	VYEGERPLTK	40	99.772	(N-term)_iTRAQ[0], Lysine(K)_iTRAQ[10]	[3] F6 and F9	1128/1120	0.971	0.886	1.086	1	Mascot
1710.8823	1710.9039	0.0216	13	61	74	ITPSYVAFTPEGER	34	98.91	(N-term)_iTRAQ[0]	[8] F13-15 and F1+2	479/471	1.120	1.096	1.231	1	Mascot
1710.8823	1710.9175	0.0352	21	61	74	ITPSYVAFTPEGER	42	99.84	(N-term)_iTRAQ[0]	[8] F13-15 and F1+2	482/474	0.849	0.990	0.947	1	Mascot

Calc. Mass	Obsrv. Mass	± da	± ppm	Start Seq.	End Seq.	Sequence	Ion Score	C. I. %	Modification	Plate [#] Name	Gel Idx/Pos [4700 Sample Name]	iTRAQ Ratio 115/114*	iTRAQ Ratio 116/114*	iTRAQ Ratio 117/114*	Rank	Result Type
1718.896	1718.8075	-0.0885	-51	102	113	TWNDPSVQQDIK	72	100	(N-term)_iTRAQ[0], Lysine(K)_iTRAQ[12]	[7] F5 120912	182/174	0.828	1.006	0.946	1	Mascot
1800.9564	1800.8696	-0.0868	-48	325	336	AKFEELNMDLFR	58	99.996	(N-term)_iTRAQ[0], Lysine(K)_iTRAQ[2]	[6] F8 110912	399/391	0.908	0.715	0.952	1	Mascot
1800.9564	1801.0305	0.0741	41	325	336	AKFEELNMDLFR	47	99.947	(N-term)_iTRAQ[0], Lysine(K)_iTRAQ[2]	[3] F6 and F9	1335/1327	1.453	1.697	1.695	1	Mascot
1877.059	1876.9684	-0.0906	-48	353	367	KSDIDEIVLVGGSTR	35	99.244	(N-term)_iTRAQ[0], Lysine(K)_iTRAQ[1]	[6] F8 110912	290/282	1.380	1.787	3.029	1	Mascot
1966.0127	1965.902	-0.1107	-56	82	96	NQLTSNPENTVFDAK	62	99.999	(N-term)_iTRAQ[0], Lysine(K)_iTRAQ[15]	[5] F4	167/159	1.510	1.384	1.665	1	Mascot
2037.1716	2037.056	-0.1156	-57	124	138	TKPYIQVDIGGGQTK	92	100	(N-term)_iTRAQ[0], Lysine(K)_iTRAQ[2,15]	[6] F8 110912	238/230	1.313	1.000	1.211	1	Mascot
2125.1387	2125.0442	-0.0945	-44	448	464	SQIFSTASDNQPTVTIK	53	99.989	(N-term)_iTRAQ[0], Lysine(K)_iTRAQ[17]	[1] F3 030912	259/251	1.823	1.325	1.029	1	Mascot
2263.1128	2263.095	-0.0178	-8	602	617	IEWLESHQDADIEDFK	94	100	(N-term)_iTRAQ[0], Lysine(K)_iTRAQ[16]	[4] F7 and F10+11	381/373	1.294	1.112	1.064	1	Mascot

82	glyceraldehyde 3-phosphate-dehydrogenase [Rattus norvegicus]	gi|56188	40078.1	9	579	1.049	0.961	1.046	0.742	0.692	0.570	17	17	17	100

Protein Group

glyceraldehyde-3-phosphate dehydrogenase [Rattus norvegicus]	gi|8393418	39925.9

Peptide Information

Calc. Mass	Obsrv. Mass	± da	± ppm	Start Seq.	End Seq.	Sequence	Ion Score	C. I. %	Modification	Plate [#] Name	Gel Idx/Pos [4700 Sample Name]	iTRAQ Ratio 115/114*	iTRAQ Ratio 116/114*	iTRAQ Ratio 117/114*	Rank	Result Type
1075.5143	1075.5516	0.0373	35	19	25	AAFSCDK	43	99.886	(N-term)_iTRAQ[0], Lysine(K)_iTRAQ[7], MMTS (C)[5]	[4] F7 and F10+11	209/201	1.279	1.295	1.193	1	Mascot
1515.816	1515.7639	-0.0521	-34	322	332	VVDLMAYMASK	81	100	(N-term)_iTRAQ[0], Lysine(K)_iTRAQ[11]	[5] F4	320/312	1.002	1.016	0.979	1	Mascot
1644.8586	1644.801	-0.0576	-35	322	333	VVDLMAYMASKE	42	99.846	(N-term)_iTRAQ[0], Lysine(K)_iTRAQ[11]	[5] F4	332/324	0.827	0.313	1.116	1	Mascot
1657.9482	1657.8253	-0.1229	-74	199	213	GAAQNIIPASTGAAK	89	100	(N-term)_iTRAQ[0], Lysine(K)_iTRAQ[15]	[5] F4	120/112	0.944	0.992	0.967	1	Mascot
1657.9482	1657.9067	-0.0415	-25	199	213	GAAQNIIPASTGAAK	51	99.982	(N-term)_iTRAQ[0], Lysine(K)_iTRAQ[15]	[5] F4	595/587	1.139	0.822	1.199	1	Mascot
1689.877	1689.892	0.015	9	233	246	VPTPNVSVVDLTCR	59	99.997	(N-term)_iTRAQ[0], MMTS (C)[13]	[8] F13-15 and F1+2	404/396	0.853	1.141	1.182	1	Mascot
1689.877	1689.8938	0.0168	10	233	246	VPTPNVSVVDLTCR	59	99.997	(N-term)_iTRAQ[0], MMTS (C)[13]	[8] F13-15 and F1+2	507/499	0.902	0.798	0.840	1	Mascot
1689.877	1689.8951	0.0181	11	233	246	VPTPNVSVVDLTCR	49	99.965	(N-term)_iTRAQ[0], MMTS (C)[13]	[8] F13-15 and F1+2	397/389	0.881	0.841	0.852	1	Mascot
1689.877	1689.9021	0.0251	15	233	246	VPTPNVSVVDLTCR	41	99.823	(N-term)_iTRAQ[0], MMTS (C)[13]	[8] F13-15 and F1+2	560/552	1.218	1.148	1.126	1	Mascot
1689.877	1689.968	0.091	54	233	246	VPTPNVSVVDLTCR	48	99.96	(N-term)_iTRAQ[0], MMTS (C)[13]	[8] F13-15 and F1+2	1283/1275	0.644	0.805	0.801	1	Mascot
1896.1304	1896.0627	-0.0677	-36	247	257	LEKPAKYDDIK	30	97.647	(N-term)_iTRAQ[0], Lysine(K)_iTRAQ[3,6,11]	[2] F12 040912	207/199	0.964	0.880	1.435	1	Mascot
1923.8997	1923.8331	-0.0666	-35	308	321	LISWYDNEYGYSNR	75	100	(N-term)_iTRAQ[0]	[5] F4	269/261	0.900	1.003	0.938	1	Mascot
1923.8997	1923.8473	-0.0524	-27	308	321	LISWYDNEYGYSNR	89	100	(N-term)_iTRAQ[0]	[1] F3 030912	351/343	0.828	0.742	0.749	1	Mascot
1923.8997	1923.8932	-0.0065	-3	308	321	LISWYDNEYGYSNR	39	99.661	(N-term)_iTRAQ[0]	[4] F7 and F10+11	275/267	0.748	0.627	0.576	1	Mascot
2501.3142	2501.2673	-0.0469	-19	117	137	VIISAPSADAPMFVMGVNHEK	37	99.541	(N-term)_iTRAQ[0], Lysine(K)_iTRAQ[21]	[7] F5 120912	356/348	0.926	0.947	1.017	1	Mascot
2565.2429	2565.1758	-0.0671	-26	85	105	WGDAGAEYVVESTGVFTTMEK	108	100	(N-term)_iTRAQ[0], Lysine(K)_iTRAQ[21]	[1] F3 030912	466/458	0.935	0.946	0.684	1	Mascot
2565.2429	2565.187	-0.0559	-22	85	105	WGDAGAEYVVESTGVFTTMEK	48	99.962	(N-term)_iTRAQ[0], Lysine(K)_iTRAQ[21]	[2] F12 040912	471/463	8.066	5.789	4.643	1	Mascot

83	brain acid soluble protein 1 [Rattus norvegicus]	gi|11560135	25956.4	6	574	1.124	1.326	1.141	0.759	0.712	0.732	10	10	10	100

Peptide Information

Calc. Mass	Obsrv. Mass	± da	± ppm	Start Seq.	End Seq.	Sequence	Ion Score	C. I. %	Modification	Plate [#] Name	Gel Idx/Pos [4700 Sample Name]	iTRAQ Ratio 115/114*	iTRAQ Ratio 116/114*	iTRAQ Ratio 117/114*	Rank	Result Type
1601.9235	1601.991	0.0675	42	146	157	KTEAPAAGPEAK	44	99.899	(N-term)_iTRAQ[0], Lysine(K)_iTRAQ[1,12]	[4] F7 and F10+11	973/965	0.624	0.834	1.108	1	Mascot
1604.8378	1604.719	-0.1188	-74	179	192	ETPAASEAPSSAAK	73	100	(N-term)_iTRAQ[0], Lysine(K)_iTRAQ[14]	[5] F4	37/29	1.941	2.263	3.245	1	Mascot
1733.8804	1733.7686	-0.1118	-64	39	52	ESEPQAAADATEVK	87	100	(N-term)_iTRAQ[0], Lysine(K)_iTRAQ[14]	[5] F4	97/89	0.848	0.969	1.055	1	Mascot
1733.8804	1733.8142	-0.0662	-38	39	52	ESEPQAAADATEVK	46	99.936	(N-term)_iTRAQ[0], Lysine(K)_iTRAQ[14]	[7] F5 120912	130/122	2.434	2.245	2.010	1	Mascot
2251.1914	2251.0637	-0.1277	-57	53	68	ESAEEKPKDAADGEAK	79	100	(N-term)_iTRAQ[0], Lysine(K)_iTRAQ[6,8,16]	[2] F12 040912	92/84	1.287	1.085	1.636	1	Mascot

Calc. Mass	Obsrv. Mass	± da	± ppm	Start Seq.	End Seq.	Sequence	Ion Score	C. I. %	Modification	Plate [#] Name	Gel Idx/Pos [4700 Sample Name]	iTRAQ Ratio 115/114*	iTRAQ Ratio 116/114*	iTRAQ Ratio 117/114*	Rank	Result Type
2421.1262	2420.9922	-0.134	-55	123	145	AGEASAESTGAADGAPQEEGEAK	55	99.992	(N-term)_iTRAQ[0], Lysine(K)_iTRAQ[23]	[5] F4	9/1	2.202	2.259	0.652	1	Mascot
2421.1262	2421	-0.1262	-52	123	145	AGEASAESTGAADGAPQEEGEAK	101	100	(N-term)_iTRAQ[0], Lysine(K)_iTRAQ[23]	[5] F4	60/52	1.411	2.125	1.143	1	Mascot
2421.1262	2421.011	-0.1152	-48	123	145	AGEASAESTGAADGAPQEEGEAK	50	99.975	(N-term)_iTRAQ[0], Lysine(K)_iTRAQ[23]	[1] F3 030912	165/157	0.551	0.911	0.689	1	Mascot
2421.1262	2421.0181	-0.1081	-45	123	145	AGEASAESTGAADGAPQEEGEAK	208	100	(N-term)_iTRAQ[0], Lysine(K)_iTRAQ[23]	[1] F3 030912	114/106	0.823	1.046	0.844	1	Mascot
2693.3237	2693.2219	-0.1018	-38	123	146	AGEASAESTGAADGAPQEEGEAKK	83	100	(N-term)_iTRAQ[0], Lysine(K)_iTRAQ[23, 24]	[4] F7 and F10+11	114/106	0.707	0.825	0.695	1	Mascot

84	actin, alpha cardiac muscle 1 proprotein [Homo sapiens]	gi|4885049	45149.8	10	574	0.974	1.005	0.958	0.208	0.272	0.226	20	20	20	100

Protein Group

actin, alpha skeletal muscle [Homo sapiens]	gi|4501881	45181.8

Peptide Information

Calc. Mass	Obsrv. Mass	± da	± ppm	Start Seq.	End Seq.	Sequence	Ion Score	C. I. %	Modification	Plate [#] Name	Gel Idx/Pos [4700 Sample Name]	iTRAQ Ratio 115/114*	iTRAQ Ratio 116/114*	iTRAQ Ratio 117/114*	Rank	Result Type
932.639	932.6142	-0.0248	-27	65	70	GILTLK	34	99.093	(N-term)_iTRAQ[0], Lysine(K)_iTRAQ[6]	[7] F5 120912	201/193	0.986	1.144	0.899	1	Mascot
1286.6913	1286.6261	-0.0652	-51	186	193	DLTDYLMK	47	99.954	(N-term)_iTRAQ[0], Lysine(K)_iTRAQ[8]	[5] F4	222/214	0.798	0.581	0.735	1	Mascot
1286.6913	1286.6511	-0.0402	-31	186	193	DLTDYLMK	59	99.997	(N-term)_iTRAQ[0], Lysine(K)_iTRAQ[8]	[7] F5 120912	278/270	1.068	0.998	1.197	1	Mascot
1342.8079	1342.7903	-0.0176	-13	31	41	AVFPSIVGRPR	40	99.763	(N-term)_iTRAQ[0]	[4] F7 and F10+11	264/256	0.759	0.689	0.833	1	Mascot
1449.8232	1449.7581	-0.0651	-45	318	328	EITALAPSTMK	86	100	(N-term)_iTRAQ[0], Lysine(K)_iTRAQ[11]	[5] F4	162/154	1.013	1.211	1.294	1	Mascot
1449.8232	1449.7938	-0.0294	-20	318	328	EITALAPSTMK	63	99.999	(N-term)_iTRAQ[0], Lysine(K)_iTRAQ[11]	[5] F4	595/587	0.681	0.930	0.597	1	Mascot
1459.7759	1459.848	0.0721	49	42	52	HQGVMVGMGQK	55	99.991	(N-term)_iTRAQ[0], Lysine(K)_iTRAQ[11]	[4] F7 and F10+11	1056/1048	0.957	1.125	1.181	1	Mascot
1486.7272	1486.6821	-0.0451	-30	53	63	DSYVGDEAQSK	95	100	(N-term)_iTRAQ[0], Lysine(K)_iTRAQ[11]	[7] F5 120912	83/75	1.198	1.220	1.451	1	Mascot
1659.8516	1659.9171	0.0655	39	87	97	IWHHTFYNELR	55	99.993	(N-term)_iTRAQ[0]	[8] F13-15 and F1+2	1151/1143	1.505	1.664	1.398	1	Mascot
1934.9944	1934.9426	-0.0518	-27	241	256	SYELPDGQVITIGNER	68	100	(N-term)_iTRAQ[0]	[1] F3 030912	354/346	1.415	1.525	0.953	1	Mascot
1934.9944	1934.9534	-0.041	-21	241	256	SYELPDGQVITIGNER	28	96.041	(N-term)_iTRAQ[0]	[8] F13-15 and F1+2	216/208	1.100	0.861	0.883	1	Mascot
1934.9944	1934.9812	-0.0132	-7	241	256	SYELPDGQVITIGNER	48	99.963	(N-term)_iTRAQ[0]	[8] F13-15 and F1+2	476/468	0.981	0.803	0.847	1	Mascot
1934.9944	1934.9906	-0.0038	-2	241	256	SYELPDGQVITIGNER	29	97.125	(N-term)_iTRAQ[0]	[8] F13-15 and F1+2	424/416	1.041	1.046	1.003	1	Mascot
1934.9944	1934.995	0.0006	0	241	256	SYELPDGQVITIGNER	34	99.114	(N-term)_iTRAQ[0]	[8] F13-15 and F1+2	420/412	0.922	0.920	0.943	1	Mascot
1934.9944	1934.9954	0.001	1	241	256	SYELPDGQVITIGNER	35	99.278	(N-term)_iTRAQ[0]	[8] F13-15 and F1+2	466/458	1.097	1.034	0.822	1	Mascot
1934.9944	1935.0089	0.0145	7	241	256	SYELPDGQVITIGNER	38	99.61	(N-term)_iTRAQ[0]	[8] F13-15 and F1+2	576/568	0.850	1.022	1.037	1	Mascot
1934.9944	1935.011	0.0166	9	241	256	SYELPDGQVITIGNER	35	99.285	(N-term)_iTRAQ[0]	[8] F13-15 and F1+2	417/409	0.981	0.923	0.915	1	Mascot
1934.9944	1935.017	0.0226	12	241	256	SYELPDGQVITIGNER	28	96.488	(N-term)_iTRAQ[0]	[8] F13-15 and F1+2	523/515	0.874	1.234	0.895	1	Mascot
2244.2485	2243.9731	-0.2754	-123	98	115	VAPEEHPTLLTEAPLNPK	32	98.288	(N-term)_iTRAQ[0], Lysine(K)_iTRAQ[18]	[7] F5 120912	262/254	0.855	0.902	0.873	1	Mascot
2249.1157	2249.1895	0.0738	33	71	86	YPIEHGIITNWDDMEK	49	99.971	(N-term)_iTRAQ[0], Lysine(K)_iTRAQ[16]	[4] F7 and F10+11	1233/1225	0.778	0.862	0.850	1	Mascot

85	band 4.1-like protein 3 [Rattus norvegicus]	gi|16758808	133782.2	8	571	1.312	1.047	0.971	0.510	0.371	0.156	10	10	10	100

Peptide Information

Calc. Mass	Obsrv. Mass	± da	± ppm	Start Seq.	End Seq.	Sequence	Ion Score	C. I. %	Modification	Plate [#] Name	Gel Idx/Pos [4700 Sample Name]	iTRAQ Ratio 115/114*	iTRAQ Ratio 116/114*	iTRAQ Ratio 117/114*	Rank	Result Type
1531.8203	1531.8672	0.0469	31	142	151	VCEHLNLLEK	53	99.989	(N-term)_iTRAQ[0], Lysine(K)_iTRAQ[10], MMTS (C)[2]	[3] F6 and F9	1286/1278	1.207	1.029	0.948	1	Mascot
1658.8934	1658.8459	-0.0475	-29	296	307	LSMYGVDLHHAK	65	99.999	(N-term)_iTRAQ[0], Lysine(K)_iTRAQ[12]	[2] F12 040912	268/260	1.184	1.017	1.001	1	Mascot
1674.8883	1674.8555	-0.0328	-20	296	307	LSMYGVDLHHAK	30	97.542	(N-term)_iTRAQ[0], Lysine(K)_iTRAQ[12], Oxidation (M)[3]	[2] F12 040912	225/217	1.488	0.915	0.985	1	Mascot
1777.949	1777.9266	-0.0224	-13	808	822	LMDGSEILSLLESAR	79	100	(N-term)_iTRAQ[0]	[8] F13-15 and F1+2	255/247	0.986	0.970	0.807	1	Mascot
2004.9794	2004.8462	-0.1332	-66	462	478	DSMSAAEVGTGQYATTK	93	100	(N-term)_iTRAQ[0], Lysine(K)_iTRAQ[17]	[5] F4	121/113	1.920	1.379	0.832	1	Mascot
2004.9794	2004.8495	-0.1299	-65	462	478	DSMSAAEVGTGQYATTK	125	100	(N-term)_iTRAQ[0], Lysine(K)_iTRAQ[17]	[1] F3 030912	190/182	1.337	0.702	0.930	1	Mascot
2064.0139	2064.0173	0.0034	2	279	294	GMTPAEAEMHFLENAK	69	100	(N-term)_iTRAQ[0], Lysine(K)_iTRAQ[16]	[4] F7 and F10+11	376/368	1.217	0.786	0.912	1	Mascot
2104.0281	2104.0208	-0.0073	-3	117	132	VTLLDGSEYGCDVDKR	59	99.997	(N-term)_iTRAQ[0], Lysine(K)_iTRAQ[15],	[4] F7 and F10+11	325/317	1.518	0.821	1.272	1	Mascot

									MMTS (C)[11]							
2114.0938	2114.0151	-0.0787	-37	786	801	QEDAPMIEPLVPEETK	39	99.656	(N-term)_iTRAQ[0], Lysine(K)_iTRAQ[16]	[1] F3 030912	290/282	0.660	2.082	0.869	1	Mascot
2288.1543	2288.0886	-0.0657	-29	757	773	TFLETSTETALTNEWEK	82	100	(N-term)_iTRAQ[0], Lysine(K)_iTRAQ[17]	[1] F3 030912	382/374	2.308	1.302	1.271	1	Mascot

86	tubulin alpha-8 chain [Rattus norvegicus]	gi|66730465	53439.4	10	566	1.154	1.112	1.021	0.465	0.487	0.671	21	21	21	100

Peptide Information

Calc. Mass	Obsrv. Mass	± da	± ppm	Start Seq.	End Seq.	Sequence	Ion Score	C. I. %	Modification	Plate [#] Name	Gel Idx/Pos [4700 Sample Name]	iTRAQ Ratio 115/114*	iTRAQ Ratio 116/114*	iTRAQ Ratio 117/114*	Rank	Result Type
1175.6381	1175.603	-0.0351	-30	395	401	FDLMYAK	41	99.785	(N-term)_iTRAQ[0], Lysine(K)_iTRAQ[7]	[7] F5 120912	255/247	1.048	1.116	0.986	1	Mascot
1631.9816	1631.9253	-0.0563	-34	230	243	LISQIVSSITASLR	73	100	(N-term)_iTRAQ[0]	[1] F3 030912	580/572	1.272	1.138	1.282	1	Mascot
1631.9816	1631.9677	-0.0139	-9	230	243	LISQIVSSITASLR	52	99.985	(N-term)_iTRAQ[0]	[8] F13-15 and F1+2	211/203	1.352	1.231	1.211	1	Mascot
1698.9789	1698.9282	-0.0507	-30	85	96	QLFHPEQLITGK	73	100	(N-term)_iTRAQ[0], Lysine(K)_iTRAQ[12]	[6] F8 110912	370/362	1.300	1.240	0.957	1	Mascot
1698.9789	1698.9305	-0.0484	-28	85	96	QLFHPEQLITGK	38	99.641	(N-term)_iTRAQ[0], Lysine(K)_iTRAQ[12]	[7] F5 120912	275/267	1.165	1.747	1.318	1	Mascot
1698.9789	1698.9484	-0.0305	-18	85	96	QLFHPEQLITGK	48	99.962	(N-term)_iTRAQ[0], Lysine(K)_iTRAQ[12]	[6] F8 110912	587/579	2.345	2.048	3.682	1	Mascot
1698.9789	1698.9783	-0.0006	0	85	96	QLFHPEQLITGK	81	100	(N-term)_iTRAQ[0], Lysine(K)_iTRAQ[12]	[4] F7 and F10+11	311/303	0.826	0.760	0.890	1	Mascot
1698.9789	1699.0499	0.071	42	85	96	QLFHPEQLITGK	32	98.505	(N-term)_iTRAQ[0], Lysine(K)_iTRAQ[12]	[3] F6 and F9	1265/1257	1.091	1.050	1.033	1	Mascot
1813.0054	1812.9636	-0.0418	-23	391	401	LDHKFDLMYAK	38	99.609	(N-term)_iTRAQ[0], Lysine(K)_iTRAQ[4,1 1]	[2] F12 040912	317/309	0.885	0.665	1.291	1	Mascot
1829.0002	1828.9752	-0.025	-14	391	401	LDHKFDLMYAK	38	99.613	(N-term)_iTRAQ[0], Lysine(K)_iTRAQ[4,1 1], Oxidation (M)[8]	[2] F12 040912	276/268	0.839	0.682	1.068	1	Mascot
1862.9845	1862.9556	-0.0289	-16	216	229	NLDIERPTYTNLNR	30	97.732	(N-term)_iTRAQ[0]	[4] F7 and F10+11	238/230	1.553	1.246	1.030	1	Mascot
1875.9364	1875.8833	-0.0531	-28	340	352	TIQFVDWCPTGFK	56	99.993	(N-term)_iTRAQ[0], Lysine(K)_iTRAQ[13], MMTS (C)[8]	[1] F3 030912	486/478	0.440	0.524	0.264	1	Mascot
1875.9364	1875.886	-0.0504	-27	340	352	TIQFVDWCPTGFK	59	99.997	(N-term)_iTRAQ[0], Lysine(K)_iTRAQ[13], MMTS (C)[8]	[5] F4	378/370	0.939	0.969	1.057	1	Mascot
1997.9712	1997.916	-0.0552	-28	374	390	AVCMLSNTTAIAEAWAR	29	96.623	(N-term)_iTRAQ[0], MMTS (C)[3]	[1] F3 030912	492/484	2.182	2.439	1.956	1	Mascot
1997.9712	1997.9668	-0.0044	-2	374	390	AVCMLSNTTAIAEAWAR	38	99.619	(N-term)_iTRAQ[0], MMTS (C)[3]	[8] F13-15 and F1+2	310/302	1.165	1.072	0.786	1	Mascot
1997.9712	1997.9895	0.0183	9	374	390	AVCMLSNTTAIAEAWAR	39	99.666	(N-term)_iTRAQ[0], MMTS (C)[3]	[4] F7 and F10+11	495/487	1.117	0.977	0.445	1	Mascot
1997.9712	1998.0536	0.0824	41	374	390	AVCMLSNTTAIAEAWAR	53	99.986	(N-term)_iTRAQ[0], MMTS (C)[3]	[4] F7 and F10+11	1343/1335	1.505	1.793	0.689	1	Mascot
2113.1902	2113.1169	-0.0733	-35	353	370	VGINYQPPTVVPGGDLAK	107	100	(N-term)_iTRAQ[0], Lysine(K)_iTRAQ[18]	[1] F3 030912	309/301	1.136	1.140	0.923	1	Mascot
2474.1206	2473.991	-0.1296	-52	403	422	AFVHWYVGEGMEEGEF SEAR	44	99.905	(N-term)_iTRAQ[0]	[6] F8 110912	399/391	1.267	1.191	1.075	1	Mascot
2474.1206	2474.1038	-0.0168	-7	403	422	AFVHWYVGEGMEEGEF SEAR	54	99.989	(N-term)_iTRAQ[0]	[4] F7 and F10+11	397/389	1.024	1.067	0.778	1	Mascot
2703.4099	2703.511	0.1011	37	85	105	QLFHPEQLITGKEDAANN YAR	31	97.893	(N-term)_iTRAQ[0], Lysine(K)_iTRAQ[12]	[4] F7 and F10+11	1207/1199	1.179	0.877	1.646	1	Mascot

87	fructose-bisphosphate aldolase A [Rattus norvegicus]	gi|6978487	43585.9	9	566	1.179	1.230	1.050	0.443	0.464	0.177	12	12	12	100

Peptide Information

Calc. Mass	Obsrv. Mass	± da	± ppm	Start Seq.	End Seq.	Sequence	Ion Score	C. I. %	Modification	Plate [#] Name	Gel Idx/Pos [4700 Sample Name]	iTRAQ Ratio 115/114*	iTRAQ Ratio 116/114*	iTRAQ Ratio 117/114*	Rank	Result Type
1068.5922	1068.5229	-0.0693	-65	15	22	ELADIAHR	46	99.942	(N-term)_iTRAQ[0]	[6] F8 110912	171/163	0.884	0.866	0.782	1	Mascot
1068.5922	1068.6146	0.0224	21	15	22	ELADIAHR	37	99.462	(N-term)_iTRAQ[0]	[3] F6 and F9	1133/1125	1.845	1.877	1.155	1	Mascot
1188.6709	1188.618	-0.0529	-45	61	69	QLLLTADDR	47	99.953	(N-term)_iTRAQ[0]	[1] F3 030912	218/210	0.933	1.073	1.137	1	Mascot
1204.5933	1204.5459	-0.0474	-39	202	208	CQYVTEK	45	99.922	(N-term)_iTRAQ[0], Lysine(K)_iTRAQ[7], MMTS (C)[1]	[3] F6 and F9	195/187	1.096	1.252	1.442	1	Mascot
1204.5933	1204.5688	-0.0245	-20	202	208	CQYVTEK	32	98.481	(N-term)_iTRAQ[0], Lysine(K)_iTRAQ[7], MMTS (C)[1]	[7] F5 120912	155/147	0.829	1.079	1.024	1	Mascot
1239.6831	1239.6327	-0.0504	-41	323	330	AAQEEYIK	34	98.922	(N-term)_iTRAQ[0], Lysine(K)_iTRAQ[8]	[3] F6 and F9	136/128	1.119	1.109	1.089	1	Mascot
1620.9054	1620.8074	-0.098	-60	29	42	GILAADESTGSIAK	112	100	(N-term)_iTRAQ[0], Lysine(K)_iTRAQ[14]	[5] F4	150/142	1.094	1.174	1.188	1	Mascot
1630.9163	1630.8309	-0.0854	-52	88	99	ADDGRPFPQVIK	81	100	(N-term)_iTRAQ[0], Lysine(K)_iTRAQ[12]	[6] F8 110912	233/225	0.888	0.825	0.894	1	Mascot
1630.9163	1630.9463	0.03	18	88	99	ADDGRPFPQVIK	41	99.796	(N-term)_iTRAQ[0], Lysine(K)_iTRAQ[12]	[3] F6 and F9	1193/1185	1.865	1.857	1.004	1	Mascot
1665.921	1665.9893	0.0683	41	2	13	PHPYPALTPEQK	84	100	(N-term)_iTRAQ[0], Lysine(K)_iTRAQ[12]	[4] F7 and F10+11	1077/1069	0.928	0.858	0.945	1	Mascot

Calc. Mass	Obsrv. Mass	± da	± ppm	Start Seq.	End Seq.	Sequence	Ion Score	C. I. %	Modification	Plate [#] Name	Gel Idx/Pos [4700 Sample Name]	iTRAQ Ratio 115/114*	iTRAQ Ratio 116/114*	iTRAQ Ratio 117/114*	Rank	Result Type
1790.9117	1790.8063	-0.1054	-59	44	57	LQSIGTENTEENRR	71	100	(N-term)_iTRAQ[0]	[6] F8 110912	148/140	1.262	1.260	1.181	1	Mascot
2267.1938	2267.1411	-0.0527	-23	154	173	IGEHTPSSLAIMENANVL AR	47	99.95	(N-term)_iTRAQ[0]	[7] F5 120912	340/332	2.205	2.354	0.918	1	Mascot

88	collapsin response mediator protein [Rattus norvegicus]	gi|1518520	67502.1	10	560	0.929	1.136	0.942	0.812	0.340	0.766	10	10	10	100

Protein Group

dihydropyrimidinase-related protein 1 [Rattus norvegicus]	gi|25742751	67476.1

Peptide Information

Calc. Mass	Obsrv. Mass	± da	± ppm	Start Seq.	End Seq.	Sequence	Ion Score	C. I. %	Modification	Plate [#] Name	Gel Idx/Pos [4700 Sample Name]	iTRAQ Ratio 115/114*	iTRAQ Ratio 116/114*	iTRAQ Ratio 117/114*	Rank	Result Type
1145.6552	1145.6909	0.0357	31	488	496	VFGLHSVSR	37	99.503	(N-term)_iTRAQ[0]	[3] F6 and F9	1176/1168	0.978	1.021	0.711	1	Mascot
1602.9003	1602.9938	0.0935	58	472	481	KPFPEHLYQR	36	99.429	(N-term)_iTRAQ[0], Lysine(K)_iTRAQ[1]	[8] F13-15 and F1+2	1542/1534	1.135	1.232	1.000	1	Mascot
1611.9679	1611.8875	-0.0804	-50	44	56	QIGENLIVPGGVK	71	100	(N-term)_iTRAQ[0], Lysine(K)_iTRAQ[13]	[5] F4	202/194	0.895	1.286	0.968	1	Mascot
1911.9772	1911.891	-0.0862	-45	497	511	GMYDGPVYEVPATPK	66	99.999	(N-term)_iTRAQ[0], Lysine(K)_iTRAQ[15]	[1] F3 030912	262/254	0.893	1.153	1.059	1	Mascot
2014.0161	2013.9055	-0.1106	-55	375	390	MDENQFVAVTSTNAAK	82	100	(N-term)_iTRAQ[0], Lysine(K)_iTRAQ[16]	[5] F4	165/157	1.268	1.136	1.117	1	Mascot
2371.1511	2371.2476	0.0965	41	533	552	NLHQSNFSLSGAQIDDN NPR	34	99.101	(N-term)_iTRAQ[0]	[4] F7 and F10+11	1113/1105	0.155	0.559	0.183	1	Mascot
2391.2966	2391.2773	-0.0193	-8	401	418	IAVGSDADVVIWDPDKMK	33	98.745	(N-term)_iTRAQ[0], Lysine(K)_iTRAQ[16, 18]	[4] F7 and F10+11	373/365	1.569	1.609	1.500	1	Mascot
2487.2363	2487.1743	-0.062	-25	172	189	DLYQMSDSQLYEAFTFLK	99	100	(N-term)_iTRAQ[0], Lysine(K)_iTRAQ[18]	[1] F3 030912	529/521	0.938	1.222	1.258	1	Mascot
3129.675	3129.5532	-0.1218	-39	95	122	AALAGGTTMIIDHVVPEP GSSLLTSFEK	51	99.983	(N-term)_iTRAQ[0], Lysine(K)_iTRAQ[28]	[1] F3 030912	495/487	1.211	1.321	1.787	1	Mascot
3661.824	3661.6938	-0.1302	-36	64	94	MVIPGGIDVNTYLQKPSQ GMTSADDFFQGTR	50	99.977	(N-term)_iTRAQ[0], Lysine(K)_iTRAQ[15]	[1] F3 030912	466/458	1.534	1.161	1.090	1	Mascot

89	synapsin-2 isoform 1 [Rattus norvegicus]	gi|77404242	68113.9	10	553	0.882	1.180	1.331	0.206	0.222	0.297	12	12	12	100

Peptide Information

Calc. Mass	Obsrv. Mass	± da	± ppm	Start Seq.	End Seq.	Sequence	Ion Score	C. I. %	Modification	Plate [#] Name	Gel Idx/Pos [4700 Sample Name]	iTRAQ Ratio 115/114*	iTRAQ Ratio 116/114*	iTRAQ Ratio 117/114*	Rank	Result Type
1180.6572	1180.6278	-0.0294	-25	330	337	TSISGNWK	58	99.996	(N-term)_iTRAQ[0], Lysine(K)_iTRAQ[8]	[4] F7 and F10+11	184/176	0.847	1.331	1.544	1	Mascot
1224.7086	1224.6964	-0.0122	-10	136	143	ILGDYDIK	37	99.524	(N-term)_iTRAQ[0], Lysine(K)_iTRAQ[8]	[7] F5 120912	239/231	0.866	1.555	1.436	1	Mascot
1393.8076	1393.7667	-0.0409	-29	178	187	SFRPDFVLIR	30	97.553	(N-term)_iTRAQ[0]	[6] F8 110912	363/355	0.855	1.099	1.203	1	Mascot
1496.906	1496.973	0.067	45	135	143	KILGDYDIK	47	99.956	(N-term)_iTRAQ[0], Lysine(K)_iTRAQ[1,9]	[4] F7 and F10+11	1131/1123	0.917	1.198	1.448	1	Mascot
1695.767	1695.6853	-0.0817	-48	188	200	QHAFGMAENEDFR	94	100	(N-term)_iTRAQ[0]	[6] F8 110912	230/222	0.528	0.933	1.091	1	Mascot
1695.767	1695.8116	0.0446	26	188	200	QHAFGMAENEDFR	54	99.991	(N-term)_iTRAQ[0]	[3] F6 and F9	1187/1179	1.063	1.190	2.001	1	Mascot
1821.9408	1821.9102	-0.0306	-17	245	257	FPLIEQTYYPNHR	52	99.986	(N-term)_iTRAQ[0]	[3] F6 and F9	323/315	0.919	0.942	1.245	1	Mascot
1868.8966	1868.9045	0.0079	4	338	353	TNTGSAMLEQIAMSDR	75	100	(N-term)_iTRAQ[0]	[8] F13-15 and F1+2	446/438	0.673	0.893	0.878	1	Mascot
1868.8966	1868.9363	0.0397	21	338	353	TNTGSAMLEQIAMSDR	58	99.996	(N-term)_iTRAQ[0]	[8] F13-15 and F1+2	547/539	1.268	1.410	1.613	1	Mascot
1884.99	1885.0671	0.0771	41	94	112	QTAASAGLVDAPAPSAA SR	54	99.99	(N-term)_iTRAQ[0]	[8] F13-15 and F1+2	596/588	0.980	1.114	1.154	1	Mascot
1910.0634	1910.0428	-0.0206	-11	116	129	VLLVVDEPHTDWAK	78	100	(N-term)_iTRAQ[0], Lysine(K)_iTRAQ[14]	[4] F7 and F10+11	341/333	0.928	1.275	1.303	1	Mascot
2596.3665	2596.2625	-0.104	-40	449	471	TPPQRPAPQGGPGQPQ GMQPPGK	27	95.517	(N-term)_iTRAQ[0], Lysine(K)_iTRAQ[23]	[7] F5 120912	138/130	0.959	1.429	1.384	1	Mascot

90	aldolase A [Rattus norvegicus]	gi|202837	43493.9	9	553	0.924	0.822	1.053	0.883	1.817	0.175	12	12	12	100

Peptide Information

Calc. Mass	Obsrv. Mass	± da	± ppm	Start Seq.	End Seq.	Sequence	Ion Score	C. I. %	Modification	Plate [#] Name	Gel Idx/Pos [4700 Sample Name]	iTRAQ Ratio 115/114*	iTRAQ Ratio 116/114*	iTRAQ Ratio 117/114*	Rank	Result Type
1068.5922	1068.5229	-0.0693	-65	15	22	ELADIAHR	46	99.942	(N-term)_iTRAQ[0]	[6] F8 110912	171/163	0.884	0.866	0.782	1	Mascot
1068.5922	1068.6146	0.0224	21	15	22	ELADIAHR	37	99.462	(N-term)_iTRAQ[0]	[3] F6 and F9	1133/1125	1.845	1.877	1.155	1	Mascot
1188.6709	1188.618	-0.0529	-45	61	69	QLLLTADDR	47	99.953	(N-term)_iTRAQ[0]	[1] F3 030912	218/210	0.933	1.073	1.137	1	Mascot
1204.5933	1204.5459	-0.0474	-39	202	208	CQYVTEK	45	99.922	(N-term)_iTRAQ[0], Lysine(K)_iTRAQ[7], MMTS (C)[1]	[3] F6 and F9	195/187	1.096	1.252	1.442	1	Mascot
1204.5933	1204.5688	-0.0245	-20	202	208	CQYVTEK	32	98.481	(N-term)_iTRAQ[0], Lysine(K)_iTRAQ[7], MMTS (C)[1]	[7] F5 120912	155/147	0.829	1.079	1.024	1	Mascot

1239.6831	1239.6327	-0.0504	-41	323	330	AAQEEYIK	34	98.922	(N-term)_iTRAQ[0], Lysine(K)_iTRAQ[8]	[3] F6 and F9	136/128	1.119	1.109	1.089	1	Mascot
1620.9054	1620.8074	-0.098	-60	29	42	GILAADESTGSIAK	112	100	(N-term)_iTRAQ[0], Lysine(K)_iTRAQ[14]	[5] F4	150/142	1.094	1.174	1.188	1	Mascot
1630.9163	1630.8309	-0.0854	-52	88	99	ADDGRPFPQVIK	81	100	(N-term)_iTRAQ[0], Lysine(K)_iTRAQ[12]	[6] F8 110912	233/225	0.888	0.825	0.894	1	Mascot
1630.9163	1630.9463	0.03	18	88	99	ADDGRPFPQVIK	41	99.796	(N-term)_iTRAQ[0], Lysine(K)_iTRAQ[12]	[3] F6 and F9	1193/1185	1.865	1.857	1.004	1	Mascot
1665.921	1665.9893	0.0683	41	2	13	PHPYPALTPEQK	84	100	(N-term)_iTRAQ[0], Lysine(K)_iTRAQ[12]	[4] F7 and F10+11	1077/1069	0.928	0.858	0.945	1	Mascot
1790.9117	1790.8063	-0.1054	-59	44	57	LQSIGTENTEENRR	71	100	(N-term)_iTRAQ[0]	[6] F8 110912	148/140	1.262	1.260	1.181	1	Mascot
2235.2217	2235.1582	-0.0635	-28	154	173	IGEHTPSSLAIVENANVLA R	34	98.968	(N-term)_iTRAQ[0]	[7] F5 120912	333/325	0.118	0.019	0.946	1	Mascot

91	unnamed protein product [Rattus norvegicus]	gi|56905	64160.7	11	546	0.947	0.917	0.946	0.300	0.204	0.333	12	12	12	100

Protein Group

RecName: Full=Protein disulfide-isomerase A3; AltName: Full=58 kDa glucose-regulated protein; AltNa | gi|1352384 | 64304.8

Peptide Information

Calc. Mass	Obsrv. Mass	± da	± ppm	Start Seq.	End Seq.	Sequence	Ion Score	C. I. %	Modification	Plate [#] Name	Gel Idx/Pos [4700 Sample Name]	iTRAQ Ratio 115/114*	iTRAQ Ratio 116/114*	iTRAQ Ratio 117/114*	Rank	Result Type
1328.7783	1328.8136	0.0353	27	120	129	TADGIVSHLK	61	99.998	(N-term)_iTRAQ[0], Lysine(K)_iTRAQ[10]	[3] F6 and F9	1179/1171	0.870	0.838	0.934	1	Mascot
1335.7029	1335.6305	-0.0724	-54	63	73	LAPEYEAAATR	42	99.835	(N-term)_iTRAQ[0]	[1] F3 030912	194/186	1.068	0.861	1.043	1	Mascot
1541.8083	1541.7587	-0.0496	-32	471	481	ELNDFISYLQR	39	99.704	(N-term)_iTRAQ[0]	[1] F3 030912	427/419	0.676	0.861	0.669	1	Mascot
1661.8784	1661.793	-0.0854	-51	351	361	FLQEYFDGNLK	27	95.295	(N-term)_iTRAQ[0], Lysine(K)_iTRAQ[11]	[5] F4	301/293	1.160	1.000	1.764	1	Mascot
1684.9003	1684.8037	-0.0966	-57	366	378	SEPIPETNEGPVK	78	100	(N-term)_iTRAQ[0], Lysine(K)_iTRAQ[13]	[5] F4	111/103	1.028	0.963	1.036	1	Mascot
1796.8688	1796.7689	-0.0999	-56	105	119	IFRDGEEAGAYDGPR	31	98.007	(N-term)_iTRAQ[0]	[6] F8 110912	224/216	1.368	1.250	1.050	1	Mascot
1817.9796	1817.9089	-0.0707	-39	351	362	FLQEYFDGNLKR	43	99.889	(N-term)_iTRAQ[0], Lysine(K)_iTRAQ[11]	[6] F8 110912	372/364	0.878	0.927	1.178	1	Mascot
1876.0214	1876.0065	-0.0149	-8	148	161	FISDKDASVVGFFR	72	100	(N-term)_iTRAQ[0], Lysine(K)_iTRAQ[5]	[4] F7 and F10+11	399/391	0.872	0.795	1.038	1	Mascot
1924.9572	1924.8719	-0.0853	-44	433	447	MDATANDVPSPYEVK	57	99.995	(N-term)_iTRAQ[0], Lysine(K)_iTRAQ[15]	[5] F4	162/154	0.972	1.013	0.740	1	Mascot
2026.1556	2026.0249	-0.1307	-65	482	495	EATNPPIIQEEKPK	57	99.995	(N-term)_iTRAQ[0], Lysine(K)_iTRAQ[12, 14]	[6] F8 110912	180/172	1.503	1.350	1.212	1	Mascot
2026.1556	2026.1079	-0.0477	-24	482	495	EATNPPIIQEEKPK	56	99.994	(N-term)_iTRAQ[0], Lysine(K)_iTRAQ[12, 14]	[4] F7 and F10+11	180/172	0.520	0.635	0.624	1	Mascot
2590.3511	2590.4639	0.1128	44	195	214	EYDDNGEGITIFRPLHLA NK	42	99.828	(N-term)_iTRAQ[0], Lysine(K)_iTRAQ[20]	[4] F7 and F10+11	1241/1233	0.870	0.741	0.603	1	Mascot

92	neurofilament light polypeptide [Rattus norvegicus]	gi|13929098	66964	9	538	1.921	0.896	0.782	0.640	0.261	0.324	11	11	11	100

Peptide Information

Calc. Mass	Obsrv. Mass	± da	± ppm	Start Seq.	End Seq.	Sequence	Ion Score	C. I. %	Modification	Plate [#] Name	Gel Idx/Pos [4700 Sample Name]	iTRAQ Ratio 115/114*	iTRAQ Ratio 116/114*	iTRAQ Ratio 117/114*	Rank	Result Type
1168.597	1168.5261	-0.0709	-61	178	185	YEEEVLSR	30	97.647	(N-term)_iTRAQ[0]	[1] F3 030912	202/194	1.087	0.661	0.331	1	Mascot
1168.597	1168.5322	-0.0648	-55	178	185	YEEEVLSR	35	99.179	(N-term)_iTRAQ[0]	[5] F4	131/123	2.221	1.163	0.883	1	Mascot
1290.7151	1290.6854	-0.0297	-23	333	340	QLQELEDK	41	99.795	(N-term)_iTRAQ[0], Lysine(K)_iTRAQ[8]	[7] F5 120912	158/150	1.577	0.944	0.812	1	Mascot
1354.7828	1354.7046	-0.0782	-58	285	294	FTVLTESAAK	67	100	(N-term)_iTRAQ[0], Lysine(K)_iTRAQ[10]	[5] F4	167/159	2.153	0.967	1.309	1	Mascot
1409.7886	1409.7469	-0.0417	-30	372	380	EYQDLLNVK	60	99.997	(N-term)_iTRAQ[0], Lysine(K)_iTRAQ[9]	[7] F5 120912	243/235	2.284	1.143	1.029	1	Mascot
1682.9285	1682.8524	-0.0761	-45	213	224	IDSLMDEIAFLK	60	99.997	(N-term)_iTRAQ[0], Lysine(K)_iTRAQ[12]	[5] F4	391/383	2.079	1.037	0.743	1	Mascot
1839.0295	1838.9562	-0.0733	-40	212	224	RIDSLMDEIAFLK	42	99.85	(N-term)_iTRAQ[0], Lysine(K)_iTRAQ[13]	[6] F8 110912	476/468	2.437	0.993	0.800	1	Mascot
1851.9031	1851.8429	-0.0602	-33	423	438	SAYSGLQSSSYLMSAR	71	100	(N-term)_iTRAQ[0]	[1] F3 030912	278/270	1.172	0.730	1.044	1	Mascot
1891.9521	1891.9615	0.0094	5	38	54	SAYSSYSAPVSSSLSVR	52	99.985	(N-term)_iTRAQ[0]	[8] F13-15 and F1+2	524/516	2.690	1.039	0.852	1	Mascot
1891.9521	1891.9781	0.026	14	38	54	SAYSSYSAPVSSSLSVR	46	99.941	(N-term)_iTRAQ[0]	[8] F13-15 and F1+2	573/565	1.801	0.481	0.554	1	Mascot
2454.1377	2453.946	-0.1917	-78	483	500	EKEEGEEEEGAEEEEAA K	113	100	(N-term)_iTRAQ[0], Lysine(K)_iTRAQ[2,1 8]	[6] F8 110912	129/121	2.442	0.988	0.709	1	Mascot

93	RecName: Full=Protein bassoon	gi|51315687	442061.1	10	534	0.925	1.008	1.068	0.289	0.258	0.222	10	10	10	100

Peptide Information

Calc. Mass	Obsrv. Mass	± da	± ppm	Start Seq.	End Seq.	Sequence	Ion Score	C. I. %	Modification	Plate [#] Name	Gel Idx/Pos [4700 Sample Name]	iTRAQ Ratio	iTRAQ Ratio	iTRAQ Ratio	Rank	Result Type

Calc. Mass	Obsrv. Mass	± da	± ppm	Start Seq.	End Seq.	Sequence	Ion Score	C. I. %	Modification	Plate [#] Name	Gel Idx/Pos [4700 Sample Name]	iTRAQ Ratio 115/114*	iTRAQ Ratio 116/114*	iTRAQ Ratio 117/114*	Rank	Result Type
1388.5946	1388.5182	-0.0764	-55	831	840	ADMTDEEFMR	43	99.877	(N-term)_iTRAQ[0]	[5] F4	150/142	1.038	0.990	0.978	1	Mascot
1533.8146	1533.7299	-0.0847	-55	1769	1780	QVEQAVQTAPYR	35	99.199	(N-term)_iTRAQ[0]	[1] F3 030912	177/169	0.982	0.822	1.033	1	Mascot
1536.7931	1536.7174	-0.0757	-49	1740	1751	QPVVYGDPFQSR	30	97.336	(N-term)_iTRAQ[0]	[1] F3 030912	257/249	0.623	0.943	1.090	1	Mascot
1774.0122	1774.0754	0.0632	36	3398	3408	KFQDEITYGLK	56	99.994	(N-term)_iTRAQ[0], Lysine(K)_iTRAQ[1,11]	[4] F7 and F10+11	1166/1158	0.788	0.924	1.019	1	Mascot
1840.8586	1840.7474	-0.1112	-60	3690	3705	GQPGYPSSADYSQPSR	77	100	(N-term)_iTRAQ[0]	[1] F3 030912	151/143	1.152	1.241	1.287	1	Mascot
1879.9998	1880.0735	0.0737	39	281	298	ATSVPGPTQATAPPEVGR	47	99.95	(N-term)_iTRAQ[0]	[8] F13-15 and F1+2	589/581	0.794	1.320	1.561	1	Mascot
2056.918	2056.8049	-0.1131	-55	792	808	EQQDTAESSDDFGSQLR	103	100	(N-term)_iTRAQ[0]	[1] F3 030912	194/186	0.994	0.850	1.116	1	Mascot
2079.0828	2078.9858	-0.097	-47	2956	2969	QAELDEEEKEIDAK	65	99.999	(N-term)_iTRAQ[0], Lysine(K)_iTRAQ[9,14]	[6] F8 110912	210/202	0.737	0.836	0.747	1	Mascot
2095.103	2095.0974	-0.0056	-3	1444	1461	EKPLSGGDGEVGPPQPSR	53	99.988	(N-term)_iTRAQ[0], Lysine(K)_iTRAQ[2]	[4] F7 and F10+11	165/157	0.795	0.803	0.894	1	Mascot
2113.0559	2112.8928	-0.1631	-77	3689	3705	KGQPGYPSSADYSQPSR	28	95.709	(N-term)_iTRAQ[0], Lysine(K)_iTRAQ[1]	[6] F8 110912	140/132	1.725	1.632	1.153	1	Mascot

94	synuclein SYN2 [Rattus sp.]	gi|241081	18431.1	7	533	0.909	1.006	0.995	0.393	0.168	0.348	7	7	7	100

Protein Group

alpha-synuclein [Rattus norvegicus] gi|9507125 16811.8

Peptide Information

Calc. Mass	Obsrv. Mass	± da	± ppm	Start Seq.	End Seq.	Sequence	Ion Score	C. I. %	Modification	Plate [#] Name	Gel Idx/Pos [4700 Sample Name]	iTRAQ Ratio 115/114*	iTRAQ Ratio 116/114*	iTRAQ Ratio 117/114*	Rank	Result Type
1161.6725	1161.6531	-0.0194	-17	13	21	EGVVAAAEK	59	99.997	(N-term)_iTRAQ[0], Lysine(K)_iTRAQ[9]	[7] F5 120912	103/95	0.391	0.989	0.596	1	Mascot
1239.7195	1239.6522	-0.0673	-54	35	43	EGVLYVGSK	45	99.917	(N-term)_iTRAQ[0], Lysine(K)_iTRAQ[9]	[7] F5 120912	165/157	0.952	1.163	0.790	1	Mascot
1612.9645	1613.0343	0.0698	43	33	43	TKEGVLYVGSK	55	99.993	(N-term)_iTRAQ[0], Lysine(K)_iTRAQ[2,11]	[4] F7 and F10+11	1080/1072	1.299	1.041	1.668	1	Mascot
1613.9108	1613.8838	-0.027	-17	46	58	EGVVHGVTTVAEK	101	100	(N-term)_iTRAQ[0], Lysine(K)_iTRAQ[13]	[4] F7 and F10+11	184/176	1.047	1.250	1.034	1	Mascot
1794.0006	1793.9282	-0.0724	-40	81	96	TVEGAGNIAAATGFVK	87	100	(N-term)_iTRAQ[0], Lysine(K)_iTRAQ[16]	[1] F3 030912	284/276	0.925	1.051	1.220	1	Mascot
1987.1559	1987.2457	0.0898	45	44	58	TKEGVVHGVTTVAEK	74	100	(N-term)_iTRAQ[0], Lysine(K)_iTRAQ[2,15]	[4] F7 and F10+11	1084/1076	1.064	0.772	0.942	1	Mascot
2216.2495	2216.1755	-0.074	-33	61	80	EQVTNVGGAVVTGVTAVAQK	114	100	(N-term)_iTRAQ[0], Lysine(K)_iTRAQ[20]	[1] F3 030912	323/315	1.028	0.859	1.033	1	Mascot

95	myelin basic protein [Rattus norvegicus]	gi|4454317	23822.6	7	525	1.860	0.969	0.681	0.591	0.250	0.237	14	14	14	100

Protein Group

myelin basic protein [Rattus norvegicus] gi|4454315 20475.9

myelin basic protein isoform 1 [Rattus norvegicus] gi|70166245 23840.6

Peptide Information

Calc. Mass	Obsrv. Mass	± da	± ppm	Start Seq.	End Seq.	Sequence	Ion Score	C. I. %	Modification	Plate [#] Name	Gel Idx/Pos [4700 Sample Name]	iTRAQ Ratio 115/114*	iTRAQ Ratio 116/114*	iTRAQ Ratio 117/114*	Rank	Result Type
1190.6501	1190.5895	-0.0606	-51	35	44	DTGILDSIGR	71	100	(N-term)_iTRAQ[0]	[1] F3 030912	252/244	1.833	1.067	0.555	1	Mascot
1190.6501	1190.6577	0.0076	6	35	44	DTGILDSIGR	46	99.942	(N-term)_iTRAQ[0]	[1] F3 030912	712/704	1.432	0.565	0.340	1	Mascot
1398.7474	1398.6948	-0.0526	-38	167	177	GAYDAQGTLSK	81	100	(N-term)_iTRAQ[0], Lysine(K)_iTRAQ[11]	[7] F5 120912	121/113	3.183	1.402	0.843	1	Mascot
1419.7842	1419.8342	0.05	35	91	100	TTHYGSLPQK	71	100	(N-term)_iTRAQ[0], Lysine(K)_iTRAQ[10]	[3] F6 and F9	1110/1102	1.674	0.918	0.756	1	Mascot
1419.7842	1419.8473	0.0631	44	91	100	TTHYGSLPQK	78	100	(N-term)_iTRAQ[0], Lysine(K)_iTRAQ[10]	[4] F7 and F10+11	1044/1036	2.860	1.086	0.907	1	Mascot
1480.7339	1480.6578	-0.0761	-51	15	26	YLATASTMDHAR	41	99.811	(N-term)_iTRAQ[0]	[2] F12 040912	136/128	1.376	0.727	0.404	1	Mascot
1480.7339	1480.7552	0.0213	14	15	26	YLATASTMDHAR	76	100	(N-term)_iTRAQ[0]	[4] F7 and F10+11	209/201	2.352	1.245	0.863	1	Mascot
1480.7339	1480.7869	0.053	36	15	26	YLATASTMDHAR	31	97.903	(N-term)_iTRAQ[0]	[4] F7 and F10+11	1100/1092	1.077	0.966	0.567	1	Mascot
1483.8102	1483.892	0.0818	55	33	44	HRDTGILDSIGR	56	99.994	(N-term)_iTRAQ[0]	[8] F13-15 and F1+2	1185/1177	1.821	0.736	0.811	1	Mascot
1748.9218	1748.8163	-0.1055	-60	104	115	TQDENPVVHFFK	52	99.985	(N-term)_iTRAQ[0], Lysine(K)_iTRAQ[12]	[6] F8 110912	205/197	1.660	1.011	0.808	1	Mascot
1748.9218	1748.8369	-0.0849	-49	104	115	TQDENPVVHFFK	81	100	(N-term)_iTRAQ[0], Lysine(K)_iTRAQ[12]	[6] F8 110912	301/293	2.146	1.200	0.830	1	Mascot

Calc. Mass	Obsrv. Mass	± da	± ppm	Start Seq.	End Seq.	Sequence	Ion Score	C. I. %	Modification	Plate [#] Name	Gel Idx/Pos [4700 Sample Name]	iTRAQ Ratio 115/114*	iTRAQ Ratio 116/114*	iTRAQ Ratio 117/114*	Rank	Result Type
1748.9218	1748.8569	-0.0649	-37	104	115	TQDENPVVHFFK	76	100	(N-term)_iTRAQ[0], Lysine(K)_iTRAQ[12]	[6] F8 110912	372/364	1.682	1.008	0.573	1	Mascot
1748.9218	1748.9899	0.0681	39	104	115	TQDENPVVHFFK	91	100	(N-term)_iTRAQ[0], Lysine(K)_iTRAQ[12]	[3] F6 and F9	1266/1258	2.149	1.064	0.770	1	Mascot
2089.05	2088.9338	-0.1162	-56	138	154	FSWGAEGQKPGFGYGG R	72	100	(N-term)_iTRAQ[0], Lysine(K)_iTRAQ[9]	[6] F8 110912	293/285	1.816	0.907	0.896	1	Mascot

96	Peroxiredoxin 5 [Rattus norvegicus]	gi|51261175	24774.2	10	524	0.888	0.923	0.965	0.345	0.267	0.248	10	10	10	100

Protein Group

peroxiredoxin-5, mitochondrial precursor [Rattus norvegicus]	gi|16758404	24746.1

Peptide Information

Calc. Mass	Obsrv. Mass	± da	± ppm	Start Seq.	End Seq.	Sequence	Ion Score	C. I. %	Modification	Plate [#] Name	Gel Idx/Pos [4700 Sample Name]	iTRAQ Ratio 115/114*	iTRAQ Ratio 116/114*	iTRAQ Ratio 117/114*	Rank	Result Type
1127.6381	1127.5847	-0.0534	-47	180	186	FSMVIDK	28	96.52	(N-term)_iTRAQ[0], Lysine(K)_iTRAQ[7]	[7] F5 120912	228/220	0.904	0.627	0.883	1	Mascot
1221.7452	1221.7222	-0.023	-19	75	82	VNLAELFK	37	99.537	(N-term)_iTRAQ[0], Lysine(K)_iTRAQ[8]	[7] F5 120912	376/368	1.101	1.536	1.403	1	Mascot
1493.9426	1493.9956	0.053	35	74	82	KVNLAELFK	58	99.996	(N-term)_iTRAQ[0], Lysine(K)_iTRAQ[1,9]	[4] F7 and F10+11	1244/1236	0.819	0.912	1.005	1	Mascot
1604.9258	1604.8381	-0.0877	-55	146	158	VQLLADPTGAFGK	68	100	(N-term)_iTRAQ[0], Lysine(K)_iTRAQ[13]	[5] F4	242/234	1.059	1.214	1.258	1	Mascot
1756.0004	1756.0035	0.0031	2	102	115	THLPGFVEQAGALK	106	100	(N-term)_iTRAQ[0], Lysine(K)_iTRAQ[14]	[4] F7 and F10+11	300/292	0.395	0.842	0.952	1	Mascot
1870.9785	1870.9294	-0.0491	-26	86	101	GVLFGVPGAFTPGCSK	56	99.994	(N-term)_iTRAQ[0], Lysine(K)_iTRAQ[16], MMTS (C)[14]	[1] F3 030912	412/404	1.150	1.064	1.234	1	Mascot
2048.0796	2048.0125	-0.0671	-33	57	73	VGDTIPSVEVFEGEPGK	44	99.912	(N-term)_iTRAQ[0], Lysine(K)_iTRAQ[17]	[1] F3 030912	323/315	1.427	0.889	0.929	1	Mascot
2051.0781	2051.0583	-0.0198	-10	159	175	ETDLLLDDSLVSLFGNR	36	99.433	(N-term)_iTRAQ[0]	[8] F13-15 and F1+2	260/252	0.786	0.647	0.819	1	Mascot
2143.176	2143.0676	-0.1084	-51	85	101	KGVLFGVPGAFTPGCSK	35	99.217	(N-term)_iTRAQ[0], Lysine(K)_iTRAQ[1,17], MMTS (C)[15]	[6] F8 110912	388/380	0.915	0.859	0.637	1	Mascot
2320.2771	2320.2603	-0.0168	-7	57	74	VGDTIPSVEVFEGEPGKK	55	99.991	(N-term)_iTRAQ[0], Lysine(K)_iTRAQ[17, 18]	[4] F7 and F10+11	307/299	0.756	0.948	0.785	1	Mascot

97	Na+, K+ -ATPase beta subunit protein precursor [Rattus norvegicus]	gi|203039	40288.2	10	517	1.066	0.853	0.957	0.191	0.441	0.282	12	12	12	100

Protein Group

sodium/potassium-transporting ATPase subunit beta-1 [Rattus norvegicus]	gi|148747253	40256.2

Peptide Information

Calc. Mass	Obsrv. Mass	± da	± ppm	Start Seq.	End Seq.	Sequence	Ion Score	C. I. %	Modification	Plate [#] Name	Gel Idx/Pos [4700 Sample Name]	iTRAQ Ratio 115/114*	iTRAQ Ratio 116/114*	iTRAQ Ratio 117/114*	Rank	Result Type
1211.667	1211.6595	-0.0075	-6	15	21	FIWNSEK	44	99.907	(N-term)_iTRAQ[0], Lysine(K)_iTRAQ[7]	[3] F6 and F9	273/265	1.039	0.808	0.927	1	Mascot
1478.8792	1478.9172	0.038	26	171	179	EGKPCIIIK	35	99.192	(N-term)_iTRAQ[0], Lysine(K)_iTRAQ[3,9], MMTS (C)[5]	[4] F7 and F10+11	1152/1144	1.004	1.247	0.632	1	Mascot
1483.8645	1483.9221	0.0576	39	14	21	KFIWNSEK	42	99.84	(N-term)_iTRAQ[0], Lysine(K)_iTRAQ[1,8]	[4] F7 and F10+11	1125/1117	1.038	0.867	0.841	1	Mascot
1484.8232	1484.8033	-0.0199	-13	97	107	SYEAYVLNIIR	27	95.444	(N-term)_iTRAQ[0]	[2] F12 040912	466/458	1.018	0.270	0.956	1	Mascot
1518.7686	1518.7102	-0.0584	-38	279	289	AYGENIGYSEK	76	100	(N-term)_iTRAQ[0], Lysine(K)_iTRAQ[11]	[3] F6 and F9	181/173	1.775	1.714	1.672	1	Mascot
1518.7686	1518.7212	-0.0474	-31	279	289	AYGENIGYSEK	74	100	(N-term)_iTRAQ[0], Lysine(K)_iTRAQ[11]	[7] F5 120912	146/138	1.046	0.849	0.982	1	Mascot
1591.869	1591.9208	0.0518	33	86	96	TEISFRPNDPK	36	99.359	(N-term)_iTRAQ[0], Lysine(K)_iTRAQ[11]	[3] F6 and F9	1158/1150	1.108	0.848	0.972	1	Mascot
1766.916	1766.8284	-0.0876	-50	205	217	YNPNVLPVQCTGK	52	99.985	(N-term)_iTRAQ[0], Lysine(K)_iTRAQ[13], MMTS (C)[10]	[5] F4	223/215	1.084	1.159	0.940	1	Mascot
1778.0786	1778.0085	-0.0701	-39	72	85	VAPPGLTQIPQIQK	58	99.996	(N-term)_iTRAQ[0], Lysine(K)_iTRAQ[14]	[1] F3 030912	299/291	1.062	0.926	0.996	1	Mascot
1789.8966	1789.9386	0.042	23	279	291	AYGENIGYSEKDR	41	99.805	(N-term)_iTRAQ[0], Lysine(K)_iTRAQ[11]	[3] F6 and F9	1125/1117	0.876	0.753	1.194	1	Mascot
1789.8966	1789.9761	0.0795	44	279	291	AYGENIGYSEKDR	79	100	(N-term)_iTRAQ[0], Lysine(K)_iTRAQ[11]	[4] F7 and F10+11	1056/1048	1.000	0.808	1.160	1	Mascot
2134.9031	2134.8413	-0.0618	-29	119	134	DDMIFEDCGSMPSEPK	68	100	(N-term)_iTRAQ[0], Lysine(K)_iTRAQ[16], MMTS (C)[8]	[1] F3 030912	365/357	0.942	0.775	0.603	1	Mascot

98	myelin basic protein isoform 4 [Rattus norvegicus]	gi|70166270	19247.2	7	506	1.917	0.970	0.668	0.612	0.244	0.214	15	15	15	100

Peptide Information

Calc. Mass	Obsrv. Mass	± da	± ppm	Start	End	Sequence	Ion	C. I. %	Modification	Plate [#] Name	Gel Idx/Pos [4700	iTRAQ	iTRAQ	iTRAQ	Rank	Result Type

				Seq.	Seq.		Score				Sample Name]	Ratio 115/114*	Ratio 116/114*	Ratio 117/114*		
1190.6501	1190.5895	-0.0606	-51	35	44	DTGILDSIGR	71	100	(N-term)_iTRAQ[0]	[1] F3 030912	252/244	1.833	1.067	0.555	1	Mascot
1190.6501	1190.6577	0.0076	6	35	44	DTGILDSIGR	46	99.942	(N-term)_iTRAQ[0]	[1] F3 030912	712/704	1.432	0.565	0.340	1	Mascot
1398.7474	1398.6948	-0.0526	-38	130	140	GAYDAQGTLSK	81	100	(N-term)_iTRAQ[0], Lysine(K)_iTRAQ[11]	[7] F5 120912	121/113	3.183	1.402	0.843	1	Mascot
1419.7842	1419.8342	0.05	35	65	74	TTHYGSLPQK	71	100	(N-term)_iTRAQ[0], Lysine(K)_iTRAQ[10]	[3] F6 and F9	1110/1102	1.674	0.918	0.756	1	Mascot
1419.7842	1419.8473	0.0631	44	65	74	TTHYGSLPQK	78	100	(N-term)_iTRAQ[0], Lysine(K)_iTRAQ[10]	[4] F7 and F10+11	1044/1036	2.860	1.086	0.907	1	Mascot
1480.7339	1480.6578	-0.0761	-51	15	26	YLATASTMDHAR	41	99.811	(N-term)_iTRAQ[0]	[2] F12 040912	136/128	1.376	0.727	0.404	1	Mascot
1480.7339	1480.7552	0.0213	14	15	26	YLATASTMDHAR	76	100	(N-term)_iTRAQ[0]	[4] F7 and F10+11	209/201	2.352	1.245	0.863	1	Mascot
1480.7339	1480.7869	0.053	36	15	26	YLATASTMDHAR	31	97.903	(N-term)_iTRAQ[0]	[4] F7 and F10+11	1100/1092	1.077	0.966	0.567	1	Mascot
1483.8102	1483.892	0.0818	55	33	44	HRDTGILDSIGR	56	99.994	(N-term)_iTRAQ[0]	[8] F13-15 and F1+2	1185/1177	1.821	0.736	0.811	1	Mascot
1668.8704	1668.9259	0.0555	33	104	117	GAEGQKPGFGYGGR	53	99.987	(N-term)_iTRAQ[0], Lysine(K)_iTRAQ[6]	[4] F7 and F10+11	1048/1040	2.462	1.055	0.674	1	Mascot
1668.8704	1668.962	0.0916	55	104	117	GAEGQKPGFGYGGR	39	99.656	(N-term)_iTRAQ[0], Lysine(K)_iTRAQ[6]	[3] F6 and F9	1116/1108	2.152	0.848	0.672	1	Mascot
1748.9218	1748.8163	-0.1055	-60	78	89	TQDENPVVHFFK	52	99.985	(N-term)_iTRAQ[0], Lysine(K)_iTRAQ[12]	[6] F8 110912	205/197	1.660	1.011	0.808	1	Mascot
1748.9218	1748.8369	-0.0849	-49	78	89	TQDENPVVHFFK	81	100	(N-term)_iTRAQ[0], Lysine(K)_iTRAQ[12]	[6] F8 110912	301/293	2.146	1.200	0.830	1	Mascot
1748.9218	1748.8569	-0.0649	-37	78	89	TQDENPVVHFFK	76	100	(N-term)_iTRAQ[0], Lysine(K)_iTRAQ[12]	[6] F8 110912	372/364	1.682	1.008	0.573	1	Mascot
1748.9218	1748.9899	0.0681	39	78	89	TQDENPVVHFFK	91	100	(N-term)_iTRAQ[0], Lysine(K)_iTRAQ[12]	[3] F6 and F9	1266/1258	2.149	1.064	0.770	1	Mascot

99	myosin-Va [Rattus norvegicus]	gi|11559935	236596.2	9	504	0.957	0.929	0.871	0.187	0.214	0.169	9	9	9	100

Peptide Information

Calc. Mass	Obsrv. Mass	± da	± ppm	Start Seq.	End Seq.	Sequence	Ion Score	C. I. %	Modification	Plate [#] Name	Gel Idx/Pos [4700 Sample Name]	iTRAQ Ratio 115/114*	iTRAQ Ratio 116/114*	iTRAQ Ratio 117/114*	Rank	Result Type
1357.6919	1357.6592	-0.0327	-24	472	479	LEQEEYMK	45	99.928	(N-term)_iTRAQ[0], Lysine(K)_iTRAQ[8]	[7] F5 120912	154/146	0.920	0.907	0.778	1	Mascot
1634.8959	1634.7833	-0.1126	-69	156	169	NQSIIVSGESGAGK	47	99.952	(N-term)_iTRAQ[0], Lysine(K)_iTRAQ[14]	[5] F4	106/98	0.852	1.033	1.119	1	Mascot
1694.8936	1694.8591	-0.0345	-20	503	514	LGILDLLDEECK	67	99.999	(N-term)_iTRAQ[0], Lysine(K)_iTRAQ[12], MMTS (C)[11]	[5] F4	412/404	1.559	1.214	0.715	1	Mascot
1700.9252	1700.8126	-0.1126	-66	1039	1050	ETLNHLMVEQAK	52	99.985	(N-term)_iTRAQ[0], Lysine(K)_iTRAQ[12]	[6] F8 110912	235/227	0.899	0.730	1.068	1	Mascot
1707.925	1707.9875	0.0625	37	461	471	LQQQFNMHVFK	48	99.96	(N-term)_iTRAQ[0], Lysine(K)_iTRAQ[11]	[3] F6 and F9	1260/1252	0.940	0.774	0.689	1	Mascot
1723.0563	1723.1274	0.0711	41	1147	1157	KVPLDMSLFLK	54	99.99	(N-term)_iTRAQ[0], Lysine(K)_iTRAQ[1,11]	[3] F6 and F9	1345/1337	0.853	0.724	0.947	1	Mascot
1782.9973	1783.0942	0.0969	54	568	578	NKDTVFEEQIK	43	99.867	(N-term)_iTRAQ[0], Lysine(K)_iTRAQ[2,11]	[4] F7 and F10+11	1105/1097	0.843	0.788	0.719	1	Mascot
1838.0758	1838.1294	0.0536	29	1304	1316	DKGEIAQAYIGLK	60	99.998	(N-term)_iTRAQ[0], Lysine(K)_iTRAQ[2,13]	[3] F6 and F9	1232/1224	0.957	1.223	1.021	1	Mascot
2082.2419	2082.1711	-0.0708	-34	1715	1730	ETLEPLIQAAQLLQVK	89	100	(N-term)_iTRAQ[0], Lysine(K)_iTRAQ[16]	[1] F3 030912	522/514	0.951	1.155	0.908	1	Mascot

100	ubiquitin-like modifier-activating enzyme 1 [Rattus norvegicus]	gi|62078893	126460.2	10	499	0.914	1.030	0.991	0.339	0.340	0.277	12	12	12	100

Peptide Information

Calc. Mass	Obsrv. Mass	± da	± ppm	Start Seq.	End Seq.	Sequence	Ion Score	C. I. %	Modification	Plate [#] Name	Gel Idx/Pos [4700 Sample Name]	iTRAQ Ratio 115/114*	iTRAQ Ratio 116/114*	iTRAQ Ratio 117/114*	Rank	Result Type
1101.7241	1101.681	-0.0431	-39	90	97	NIILGGVK	49	99.972	(N-term)_iTRAQ[0], Lysine(K)_iTRAQ[8]	[7] F5 120912	195/187	0.807	0.884	0.602	1	Mascot
1248.6875	1248.6432	-0.0443	-35	974	980	QFLDYFK	39	99.71	(N-term)_iTRAQ[0], Lysine(K)_iTRAQ[7]	[3] F6 and F9	362/354	1.247	1.828	1.477	1	Mascot
1689.917	1690.0149	0.0979	58	912	923	VVQGHQQLDSYK	61	99.998	(N-term)_iTRAQ[0], Lysine(K)_iTRAQ[12]	[3] F6 and F9	1128/1120	0.813	1.073	0.873	1	Mascot
1724.9734	1725.0203	0.0469	27	516	526	QFLFRPWDVTK	52	99.983	(N-term)_iTRAQ[0], Lysine(K)_iTRAQ[11]	[3] F6 and F9	1324/1316	0.854	0.951	0.959	1	Mascot
1724.9734	1725.053	0.0796	46	516	526	QFLFRPWDVTK	27	95.434	(N-term)_iTRAQ[0], Lysine(K)_iTRAQ[11]	[4] F7 and F10+11	1266/1258	0.624	0.903	0.824	1	Mascot
1840.9579	1840.9407	-0.0172	-9	636	649	NFPNAIEHTLQWAR	53	99.988	(N-term)_iTRAQ[0]	[4] F7 and F10+11	395/387	1.107	1.191	0.865	1	Mascot
1894.9756	1894.8809	-0.0947	-50	658	671	QPAENVNQYLTDSK	65	99.999	(N-term)_iTRAQ[0], Lysine(K)_iTRAQ[14]	[5] F4	154/146	1.101	0.990	1.183	1	Mascot
1939.9845	1939.9896	0.0051	3	369	384	SPPAVQQDNVDEDLIR	56	99.994	(N-term)_iTRAQ[0]	[8] F13-15 and F1+2	527/519	1.033	0.996	0.972	1	Mascot
1943.996	1943.9188	-0.0772	-40	451	465	YDGQVAVFGSDLQEK	61	99.998	(N-term)_iTRAQ[0], Lysine(K)_iTRAQ[15]	[5] F4	214/206	1.162	1.157	1.351	1	Mascot

Calc. Mass	Obsrv. Mass	± da	± ppm	Start Seq.	End Seq.	Sequence	Ion Score	C. I. %	Modification	Plate [#] Name	Gel Idx/Pos [4700 Sample Name]	iTRAQ Ratio 115/114*	iTRAQ Ratio 116/114*	iTRAQ Ratio 117/114*	Rank	Result Type
1943.996	1943.9326	-0.0634	-33	451	465	YDGQVAVFGSDLQEK	40	99.777	(N-term)_iTRAQ[0], Lysine(K)_iTRAQ[15]	[1] F3 030912	289/281	0.413	0.501	0.864	1	Mascot
1982.927	1982.9476	0.0206	10	945	957	HQYYNQEWTLWDR	34	99.085	(N-term)_iTRAQ[0]	[3] F6 and F9	1295/1287	0.960	1.192	0.929	1	Mascot
2212.1819	2212.1243	-0.0576	-26	369	385	SPPAVQQDNVDEDLIRK	31	97.859	(N-term)_iTRAQ[0], Lysine(K)_iTRAQ[17]	[3] F6 and F9	229/221	1.339	1.186	1.362	1	Mascot

101	serine/threonine-protein phosphatase 2A 65 kDa regulatory subunit A alpha isoform [Mus musculus]	gi|8394027	70680.1	8	497	0.868	0.845	0.985	0.222	0.252	0.270	10	10	10	100

Peptide Information

Calc. Mass	Obsrv. Mass	± da	± ppm	Start Seq.	End Seq.	Sequence	Ion Score	C. I. %	Modification	Plate [#] Name	Gel Idx/Pos [4700 Sample Name]	iTRAQ Ratio 115/114*	iTRAQ Ratio 116/114*	iTRAQ Ratio 117/114*	Rank	Result Type
1448.7843	1448.7273	-0.057	-39	567	576	LTQDQDVDVK	45	99.922	(N-term)_iTRAQ[0], Lysine(K)_iTRAQ[10]	[7] F5 120912	137/129	0.785	1.103	0.845	1	Mascot
1453.8413	1453.8795	0.0382	26	476	485	EWAHATIIPK	39	99.692	(N-term)_iTRAQ[0], Lysine(K)_iTRAQ[10]	[3] F6 and F9	1212/1204	0.970	0.756	1.541	1	Mascot
1527.8376	1527.7539	-0.0837	-55	332	342	ELVSDANQHVK	58	99.996	(N-term)_iTRAQ[0], Lysine(K)_iTRAQ[11]	[6] F8 110912	146/138	0.774	0.751	1.047	1	Mascot
1527.8376	1527.8845	0.0469	31	332	342	ELVSDANQHVK	53	99.987	(N-term)_iTRAQ[0], Lysine(K)_iTRAQ[11]	[3] F6 and F9	1108/1100	1.620	1.347	0.877	1	Mascot
1616.8339	1616.8162	-0.0177	-11	486	498	VLAMSGDPNYLHR	43	99.865	(N-term)_iTRAQ[0]	[4] F7 and F10+11	254/246	0.777	0.926	1.452	1	Mascot
1836.0023	1835.9445	-0.0578	-31	319	331	ENVIMTQILPCIK	54	99.99	(N-term)_iTRAQ[0], Lysine(K)_iTRAQ[13], MMTS (C)[11]	[1] F3 030912	482/474	0.881	0.779	0.795	1	Mascot
1845.1129	1845.0754	-0.0375	-20	343	358	SALASVIMGLSPILGK	108	100	(N-term)_iTRAQ[0], Lysine(K)_iTRAQ[16]	[1] F3 030912	546/538	0.809	0.475	0.703	1	Mascot
2213.2639	2213.2158	-0.0481	-22	399	416	QLSQSLLPAIVELAEDAK	91	100	(N-term)_iTRAQ[0], Lysine(K)_iTRAQ[18]	[1] F3 030912	546/538	0.663	0.799	1.099	1	Mascot
2501.3398	2501.2981	-0.0417	-17	114	133	AISHEHSPSDLEAHFVPL VK	60	99.998	(N-term)_iTRAQ[0], Lysine(K)_iTRAQ[20]	[2] F12 040912	334/326	0.849	0.838	0.950	1	Mascot
2501.3398	2501.3896	0.0498	20	114	133	AISHEHSPSDLEAHFVPL VK	40	99.748	(N-term)_iTRAQ[0], Lysine(K)_iTRAQ[20]	[4] F7 and F10+11	1203/1195	0.814	0.959	0.851	1	Mascot

102	grp75 [Rattus sp.]	gi|1000439	81710.2	10	486	1.007	1.015	0.958	0.245	0.223	0.293	14	14	14	100

Peptide Information

Calc. Mass	Obsrv. Mass	± da	± ppm	Start Seq.	End Seq.	Sequence	Ion Score	C. I. %	Modification	Plate [#] Name	Gel Idx/Pos [4700 Sample Name]	iTRAQ Ratio 115/114*	iTRAQ Ratio 116/114*	iTRAQ Ratio 117/114*	Rank	Result Type
1386.7825	1386.7136	-0.0689	-50	207	218	DAGQISGLNVLR	27	95.538	(N-term)_iTRAQ[0]	[1] F3 030912	266/258	1.407	0.842	0.792	1	Mascot
1434.7825	1434.7356	-0.0469	-33	395	405	VQQTVQDLFGR	35	99.249	(N-term)_iTRAQ[0]	[5] F4	257/249	1.355	1.267	1.837	1	Mascot
1519.869	1519.8248	-0.0442	-29	635	646	QAASSLQQASLK	69	100	(N-term)_iTRAQ[0], Lysine(K)_iTRAQ[12]	[7] F5 120912	142/134	0.983	0.816	1.185	1	Mascot
1590.8645	1590.8645	0	0	378	391	SDIGEVILVGGMTR	65	99.999	(N-term)_iTRAQ[0]	[8] F13-15 and F1+2	420/412	1.060	1.335	1.093	1	Mascot
1590.8645	1590.8674	0.0029	2	378	391	SDIGEVILVGGMTR	55	99.993	(N-term)_iTRAQ[0]	[8] F13-15 and F1+2	424/416	1.365	1.149	0.800	1	Mascot
1590.8645	1590.8676	0.0031	2	378	391	SDIGEVILVGGMTR	42	99.861	(N-term)_iTRAQ[0]	[8] F13-15 and F1+2	412/404	0.771	0.934	0.973	1	Mascot
1590.8645	1590.8798	0.0153	10	378	391	SDIGEVILVGGMTR	62	99.998	(N-term)_iTRAQ[0]	[8] F13-15 and F1+2	417/409	1.202	1.342	1.354	1	Mascot
1620.8353	1620.8416	0.0063	4	86	99	TTPSVVAFTPDGER	55	99.992	(N-term)_iTRAQ[0]	[8] F13-15 and F1+2	527/519	0.798	0.883	0.908	1	Mascot
1620.8353	1620.8436	0.0083	5	86	99	TTPSVVAFTPDGER	39	99.68	(N-term)_iTRAQ[0]	[8] F13-15 and F1+2	524/516	0.889	0.829	0.794	1	Mascot
1621.8431	1621.7397	-0.1034	-64	176	187	ETAENYLGHTAK	30	97.722	(N-term)_iTRAQ[0], Lysine(K)_iTRAQ[12]	[6] F8 110912	175/167	0.790	1.122	0.933	1	Mascot
1838.952	1838.959	0.007	4	188	202	NAVITVPAYFNDSQR	38	99.631	(N-term)_iTRAQ[0]	[8] F13-15 and F1+2	458/450	1.181	1.300	0.695	1	Mascot
1856.9752	1856.8433	-0.1319	-71	108	121	QAVTNPNNTFYATK	64	99.999	(N-term)_iTRAQ[0], Lysine(K)_iTRAQ[14]	[5] F4	123/115	1.002	0.742	1.119	1	Mascot
2275.0762	2274.9453	-0.1309	-58	596	610	MEEFKDQLPADECNK	58	99.996	(N-term)_iTRAQ[0], Lysine(K)_iTRAQ[5,1 5], MMTS (C)[13]	[6] F8 110912	287/279	0.731	0.902	0.773	1	Mascot
2430.2544	2430.3174	0.063	26	577	595	ERVEAVNMAEGIIHDTET K	44	99.905	(N-term)_iTRAQ[0], Lysine(K)_iTRAQ[19]	[4] F7 and F10+11	1253/1245	0.908	1.026	0.687	1	Mascot

103	unnamed protein product [Rattus norvegicus]	gi|1334163	42672.2	8	475	0.936	0.889	0.846	0.168	0.158	0.174	11	11	11	100

Protein Group

fructose-bisphosphate aldolase C [Rattus norvegicus]	gi|6978489	42751.4

Peptide Information

Calc. Mass	Obsrv. Mass	± da	± ppm	Start Seq.	End Seq.	Sequence	Ion Score	C. I. %	Modification	Plate [#] Name	Gel Idx/Pos [4700 Sample Name]	iTRAQ Ratio 115/114*	iTRAQ Ratio 116/114*	iTRAQ Ratio 117/114*	Rank	Result Type
1060.6123	1060.5637	-0.0486	-46	14	21	ELSDIALR	36	99.406	(N-term)_iTRAQ[0]	[5] F4	161/153	1.053	1.055	0.902	1	Mascot
1317.74	1317.6791	-0.0609	-46	304	314	ALQASALSAWR	56	99.994	(N-term)_iTRAQ[0]	[5] F4	219/211	0.686	0.607	0.669	1	Mascot
1553.8057	1553.7	-0.1057	-68	318	329	DNAGAATEEFIK	92	100	(N-term)_iTRAQ[0], Lysine(K)_iTRAQ[12]	[5] F4	140/132	1.112	1.063	0.845	1	Mascot

Calc. Mass	Obsrv. Mass	± da	± ppm	Start Seq.	End Seq.	Sequence	Ion Score	C. I. %	Modification	Plate [#] Name	Gel Idx/Pos [4700 Sample Name]	iTRAQ Ratio 115/114*	iTRAQ Ratio 116/114*	iTRAQ Ratio 117/114*	Rank	Result Type
1553.8057	1553.7422	-0.0635	-41	318	329	DNAGAATEEFIK	37	99.543	(N-term)_iTRAQ[0], Lysine(K)_iTRAQ[12]	[7] F5 120912	179/171	1.181	1.087	1.172	1	Mascot
1632.8313	1632.7443	-0.087	-53	43	55	LSQIGVENTEENR	48	99.961	(N-term)_iTRAQ[0]	[1] F3 030912	194/186	1.049	0.908	0.646	1	Mascot
1709.9067	1709.8317	-0.075	-44	318	330	DNAGAATEEFIKR	78	100	(N-term)_iTRAQ[0], Lysine(K)_iTRAQ[12]	[6] F8 110912	210/202	0.991	0.812	0.866	1	Mascot
1788.9324	1788.817	-0.1154	-65	43	56	LSQIGVENTEENRR	58	99.996	(N-term)_iTRAQ[0]	[6] F8 110912	183/175	0.798	0.741	0.662	1	Mascot
1788.9324	1788.885	-0.0474	-26	43	56	LSQIGVENTEENRR	37	99.561	(N-term)_iTRAQ[0]	[4] F7 and F10+11	183/175	0.795	0.873	1.121	1	Mascot
1795.9384	1795.933	-0.0054	-3	243	257	YSPEEIAMATVTALR	45	99.929	(N-term)_iTRAQ[0]	[8] F13-15 and F1+2	295/287	0.791	0.912	0.847	1	Mascot
1797.0354	1797.0426	0.0072	4	157	172	TPSALAILENANVLAR	47	99.946	(N-term)_iTRAQ[0]	[8] F13-15 and F1+2	341/333	1.031	0.906	0.922	1	Mascot
1797.0354	1797.0558	0.0204	11	157	172	TPSALAILENANVLAR	62	99.999	(N-term)_iTRAQ[0]	[8] F13-15 and F1+2	350/342	0.948	0.945	0.823	1	Mascot

104	V-type proton ATPase subunit B, brain isoform [Rattus norvegicus]	gi|17105370	60105.1	9	473	1.034	1.006	1.273	0.135	0.128	0.390	9	9	9	100

Peptide Information

Calc. Mass	Obsrv. Mass	± da	± ppm	Start Seq.	End Seq.	Sequence	Ion Score	C. I. %	Modification	Plate [#] Name	Gel Idx/Pos [4700 Sample Name]	iTRAQ Ratio 115/114*	iTRAQ Ratio 116/114*	iTRAQ Ratio 117/114*	Rank	Result Type
1248.6378	1248.5698	-0.068	-54	121	130	TPVSEDMLGR	27	95.423	(N-term)_iTRAQ[0]	[1] F3 030912	207/199	0.892	1.018	1.113	1	Mascot
1482.7461	1482.6669	-0.0792	-53	461	471	NFITQGPYENR	56	99.994	(N-term)_iTRAQ[0]	[1] F3 030912	216/208	1.139	1.063	1.121	1	Mascot
1581.8397	1581.787	-0.0527	-33	495	506	IPQSTLSEFYPR	53	99.988	(N-term)_iTRAQ[0]	[1] F3 030912	335/327	1.164	1.063	1.429	1	Mascot
1703.8688	1703.9479	0.0791	46	109	120	KTSCEFTGDILR	35	99.254	(N-term)_iTRAQ[0], Lysine(K)_iTRAQ[1], MMTS (C)[4]	[3] F6 and F9	1263/1255	0.903	0.923	0.878	1	Mascot
1809.0004	1808.9209	-0.0795	-44	94	108	AVVQVFEGTSGIDAK	96	100	(N-term)_iTRAQ[0], Lysine(K)_iTRAQ[15]	[5] F4	218/210	1.208	1.270	1.112	1	Mascot
2001.1378	2001.2145	0.0767	38	68	82	YAEIVHLTLPDGTKR	43	99.869	(N-term)_iTRAQ[0], Lysine(K)_iTRAQ[14]	[4] F7 and F10+11	1213/1205	0.972	0.954	2.270	1	Mascot
2035.0471	2034.999	-0.0481	-24	277	291	LALTTAEFLAYQCEK	81	100	(N-term)_iTRAQ[0], Lysine(K)_iTRAQ[15], MMTS (C)[13]	[1] F3 030912	506/498	0.963	1.025	1.345	1	Mascot
2040.9861	2041.0027	0.0166	8	322	337	GFPGYMYTDLATIYER	38	99.629	(N-term)_iTRAQ[0]	[8] F13-15 and F1+2	350/342	0.924	1.016	1.025	1	Mascot
2062.1128	2062.0759	-0.0369	-18	292	308	HVLVILTDMSSYAEALR	44	99.911	(N-term)_iTRAQ[0]	[3] F6 and F9	522/514	1.214	0.790	1.610	1	Mascot

105	synaptojanin	gi|1586823	184798.5	9	471	0.802	0.982	1.114	0.328	0.132	0.357	9	9	9	100

Peptide Information

Calc. Mass	Obsrv. Mass	± da	± ppm	Start Seq.	End Seq.	Sequence	Ion Score	C. I. %	Modification	Plate [#] Name	Gel Idx/Pos [4700 Sample Name]	iTRAQ Ratio 115/114*	iTRAQ Ratio 116/114*	iTRAQ Ratio 117/114*	Rank	Result Type
1338.6562	1338.5775	-0.0787	-59	274	284	GFEANAPAFDR	33	98.777	(N-term)_iTRAQ[0]	[5] F4	148/140	0.740	0.836	0.946	1	Mascot
1382.6134	1382.5934	-0.02	-14	519	526	NMCENFYK	27	95.37	(N-term)_iTRAQ[0], Lysine(K)_iTRAQ[8], MMTS (C)[3]	[3] F6 and F9	304/296	0.331	1.194	2.198	1	Mascot
1521.7782	1521.8302	0.052	34	699	709	ERNEDFVEIAR	60	99.997	(N-term)_iTRAQ[0]	[3] F6 and F9	1176/1168	1.133	0.928	0.787	1	Mascot
1552.8428	1552.7866	-0.0562	-36	504	515	VSEQTLQSASSK	83	100	(N-term)_iTRAQ[0], Lysine(K)_iTRAQ[12]	[7] F5 120912	138/130	1.146	1.088	0.931	1	Mascot
1592.9006	1592.9795	0.0789	50	568	578	LAGIQEFQDKR	41	99.811	(N-term)_iTRAQ[0], Lysine(K)_iTRAQ[10]	[4] F7 and F10+11	1133/1125	0.831	0.903	0.950	1	Mascot
1709.0809	1708.9962	-0.0847	-50	347	357	LHSVLKPQVQK	27	95.338	(N-term)_iTRAQ[0], Lysine(K)_iTRAQ[6,11]	[2] F12 040912	203/195	0.799	1.186	1.316	1	Mascot
1899.8746	1899.8063	-0.0683	-36	786	798	YDLFSEDYDTSEK	56	99.994	(N-term)_iTRAQ[0], Lysine(K)_iTRAQ[13]	[5] F4	211/203	0.873	0.848	1.118	1	Mascot
1977.124	1977.0081	-0.1159	-59	1200	1213	LTPESQSKPLETSK	69	100	(N-term)_iTRAQ[0], Lysine(K)_iTRAQ[8,14]	[6] F8 110912	169/161	0.864	0.951	1.149	1	Mascot
2238.1975	2238.0725	-0.125	-56	1075	1094	TPGPLSSQGAPVDTQPAAQK	76	100	(N-term)_iTRAQ[0], Lysine(K)_iTRAQ[20]	[1] F3 030912	192/184	0.864	0.976	1.075	1	Mascot

106	NORBIN [Rattus norvegicus]	gi|2564013	83642.8	10	471	0.884	0.943	1.010	0.165	0.255	0.189	10	10	10	100

Peptide Information

Calc. Mass	Obsrv. Mass	± da	± ppm	Start Seq.	End Seq.	Sequence	Ion Score	C. I. %	Modification	Plate [#] Name	Gel Idx/Pos [4700 Sample Name]	iTRAQ Ratio 115/114*	iTRAQ Ratio 116/114*	iTRAQ Ratio 117/114*	Rank	Result Type
1437.8063	1437.7776	-0.0287	-20	130	140	IPILCTFLTAR	29	96.7	(N-term)_iTRAQ[0], MMTS (C)[5]	[1] F3 030912	562/554	0.782	1.052	1.265	1	Mascot
1498.6884	1498.6764	-0.012	-8	93	104	EAPDGCPDHVLR	36	99.361	(N-term)_iTRAQ[0], MMTS (C)[6]	[4] F7 and F10+11	215/207	0.701	1.056	1.123	1	Mascot
1505.7468	1505.6652	-0.0816	-54	707	718	LQAGEETASHYR	61	99.998	(N-term)_iTRAQ[0]	[6] F8 110912	157/149	1.191	1.200	0.995	1	Mascot
1506.8278	1506.8119	-0.0159	-11	413	423	EVCQLLPFLVR	52	99.985	(N-term)_iTRAQ[0], MMTS (C)[3]	[8] F13-15 and F1+2	259/251	1.021	0.690	0.729	1	Mascot
1527.808	1527.7561	-0.0519	-34	76	87	IFDAVGFTFPNR	70	100	(N-term)_iTRAQ[0]	[1] F3 030912	415/407	0.747	0.916	0.876	1	Mascot

Calc. Mass	Obsrv. Mass	± da	± ppm	Start Seq.	End Seq.	Sequence	Ion Score	C. I. %	Modification	Plate [#] Name	Gel Idx/Pos [4700 Sample Name]	iTRAQ Ratio 115/114*	iTRAQ Ratio 116/114*	iTRAQ Ratio 117/114*	Rank	Result Type
1595.9155	1595.97	0.0545	34	387	397	QKEPFVFASVR	42	99.858	(N-term)_iTRAQ[0], Lysine(K)_iTRAQ[2]	[3] F6 and F9	1239/1231	1.095	1.063	0.990	1	Mascot
1683.9091	1683.8447	-0.0644	-38	75	87	RIFDAVGFTFPNR	45	99.917	(N-term)_iTRAQ[0]	[6] F8 110912	388/380	0.809	0.530	1.283	1	Mascot
1689.9296	1689.8867	-0.0429	-25	398	411	ILGAWLAEETSSLR	60	99.998	(N-term)_iTRAQ[0]	[1] F3 030912	506/498	0.761	1.034	1.063	1	Mascot
2125.2266	2125.2429	0.0163	8	370	386	EAIGAVIHYLLQVGPEK	41	99.804	(N-term)_iTRAQ[0], Lysine(K)_iTRAQ[17]	[3] F6 and F9	533/525	0.904	1.121	1.088	1	Mascot
2131.9927	2131.9692	-0.0235	-11	150	167	SMIDDTYQCLTAVAGTPR	35	99.219	(N-term)_iTRAQ[0], MMTS (C)[9]	[8] F13-15 and F1+2	294/286	0.967	1.011	0.830	1	Mascot

107	14-3-3 protein gamma [Rattus norvegicus]	gi|9507245	31016.7	8	470	1.131	1.102	1.003	0.337	0.433	0.196	9	9	9	100

Peptide Information

Calc. Mass	Obsrv. Mass	± da	± ppm	Start Seq.	End Seq.	Sequence	Ion Score	C. I. %	Modification	Plate [#] Name	Gel Idx/Pos [4700 Sample Name]	iTRAQ Ratio 115/114*	iTRAQ Ratio 116/114*	iTRAQ Ratio 117/114*	Rank	Result Type
957.6019	957.6076	0.0057	6	121	125	VFYLK	34	98.922	(N-term)_iTRAQ[0], Lysine(K)_iTRAQ[5]	[3] F6 and F9	262/254	1.285	1.183	1.199	1	Mascot
1191.7195	1191.6932	-0.0263	-22	62	69	VISSIEQK	55	99.992	(N-term)_iTRAQ[0], Lysine(K)_iTRAQ[8]	[7] F5 120912	159/151	1.339	1.437	1.443	1	Mascot
1195.7296	1195.6782	-0.0514	-43	43	50	NLLSVAYK	53	99.987	(N-term)_iTRAQ[0], Lysine(K)_iTRAQ[8]	[7] F5 120912	233/225	1.100	1.296	1.119	1	Mascot
1333.7634	1333.7305	-0.0329	-25	218	227	DSTLIMQLLR	42	99.831	(N-term)_iTRAQ[0]	[1] F3 030912	466/458	1.966	2.108	0.925	1	Mascot
1333.7634	1333.7313	-0.0321	-24	218	227	DSTLIMQLLR	48	99.963	(N-term)_iTRAQ[0]	[2] F12 040912	472/464	1.159	0.774	0.827	1	Mascot
1368.7621	1368.6984	-0.0637	-47	133	142	YLAEVATGEK	52	99.985	(N-term)_iTRAQ[0], Lysine(K)_iTRAQ[10]	[5] F4	164/156	0.914	0.823	0.955	1	Mascot
1522.6744	1522.6584	-0.016	-11	111	120	NCSETQYESK	54	99.991	(N-term)_iTRAQ[0], Lysine(K)_iTRAQ[10], MMTS (C)[2]	[7] F5 120912	107/99	0.726	0.670	0.782	1	Mascot
1787.8895	1787.9148	0.0253	14	29	42	NVTELNEPLSNEER	48	99.965	(N-term)_iTRAQ[0]	[8] F13-15 and F1+2	563/555	1.006	1.038	0.955	1	Mascot
2419.1763	2419.104	-0.0723	-30	199	217	TAFDDAIAELDTLNEDSYK	125	100	(N-term)_iTRAQ[0], Lysine(K)_iTRAQ[19]	[1] F3 030912	501/493	1.054	1.164	0.973	1	Mascot

108	NAD-dependent deacetylase sirtuin-2 [Rattus norvegicus]	gi|56605812	43546.3	8	466	1.778	0.964	0.892	0.505	0.206	0.240	9	9	9	100

Peptide Information

Calc. Mass	Obsrv. Mass	± da	± ppm	Start Seq.	End Seq.	Sequence	Ion Score	C. I. %	Modification	Plate [#] Name	Gel Idx/Pos [4700 Sample Name]	iTRAQ Ratio 115/114*	iTRAQ Ratio 116/114*	iTRAQ Ratio 117/114*	Rank	Result Type
1180.6824	1180.6154	-0.067	-57	176	183	IFSEATPK	35	99.15	(N-term)_iTRAQ[0], Lysine(K)_iTRAQ[8]	[7] F5 120912	165/157	1.452	0.745	0.601	1	Mascot
1363.6636	1363.6332	-0.0304	-22	166	173	EYTMSWMK	30	97.467	(N-term)_iTRAQ[0], Lysine(K)_iTRAQ[8]	[7] F5 120912	252/244	2.660	1.421	0.975	1	Mascot
1363.6636	1363.6372	-0.0264	-19	166	173	EYTMSWMK	49	99.968	(N-term)_iTRAQ[0], Lysine(K)_iTRAQ[8]	[3] F6 and F9	299/291	2.265	1.087	0.917	1	Mascot
1444.8198	1444.8933	0.0735	51	90	99	HPEPFFALAK	52	99.985	(N-term)_iTRAQ[0], Lysine(K)_iTRAQ[10]	[3] F6 and F9	1269/1261	2.345	0.821	1.054	1	Mascot
1545.7142	1545.6454	-0.0688	-45	127	137	CYTQNIDTLER	42	99.852	(N-term)_iTRAQ[0], MMTS (C)[1]	[1] F3 030912	252/244	1.411	1.102	1.367	1	Mascot
1694.9688	1694.911	-0.0578	-34	6	18	NLFTQTLGLGSQK	87	100	(N-term)_iTRAQ[0], Lysine(K)_iTRAQ[13]	[5] F4	257/249	1.900	1.006	0.792	1	Mascot
1717.0172	1716.9833	-0.0339	-20	89	99	KHPEPFFALAK	51	99.982	(N-term)_iTRAQ[0], Lysine(K)_iTRAQ[1,11]	[2] F12 040912	311/303	1.385	0.757	0.644	1	Mascot
2468.1885	2468.0972	-0.0913	-37	310	332	EHANIDAQSGSQASNPSATVSPR	103	100	(N-term)_iTRAQ[0]	[7] F5 120912	117/109	1.287	0.915	0.895	1	Mascot
2681.2329	2681.1262	-0.1067	-40	253	275	TGQTDPFLGMMMGLGGGMDFDSK	47	99.949	(N-term)_iTRAQ[0], Lysine(K)_iTRAQ[23]	[1] F3 030912	528/520	1.810	0.988	1.009	1	Mascot

109	NADH dehydrogenase [ubiquinone] flavoprotein 1, mitochondrial precursor [Rattus norvegicus]	gi|55741424	55141.2	9	466	1.015	1.111	0.971	0.219	0.315	0.316	9	9	9	100

Peptide Information

Calc. Mass	Obsrv. Mass	± da	± ppm	Start Seq.	End Seq.	Sequence	Ion Score	C. I. %	Modification	Plate [#] Name	Gel Idx/Pos [4700 Sample Name]	iTRAQ Ratio 115/114*	iTRAQ Ratio 116/114*	iTRAQ Ratio 117/114*	Rank	Result Type
1199.6082	1199.5948	-0.0134	-11	258	267	GGTWFAGFGR	36	99.426	(N-term)_iTRAQ[0]	[7] F5 120912	310/302	1.015	0.791	0.974	1	Mascot
1266.6398	1266.6002	-0.0396	-31	387	394	EGVDWMNK	40	99.738	(N-term)_iTRAQ[0], Lysine(K)_iTRAQ[8]	[3] F6 and F9	218/210	1.143	1.772	1.240	1	Mascot
1338.7515	1338.6891	-0.0624	-47	175	184	EAYEAGLIGK	59	99.997	(N-term)_iTRAQ[0], Lysine(K)_iTRAQ[10]	[7] F5 120912	181/173	1.117	1.325	1.305	1	Mascot
1433.7709	1433.741	-0.0299	-21	72	81	GPDWILGEMK	55	99.992	(N-term)_iTRAQ[0], Lysine(K)_iTRAQ[10]	[7] F5 120912	345/337	0.636	0.992	0.517	1	Mascot
1854.947	1854.9543	0.0073	4	160	174	GEFYNEASNLQVAIR	39	99.691	(N-term)_iTRAQ[0]	[8] F13-15 and F1+2	446/438	1.093	0.913	0.735	1	Mascot
1928.9324	1928.8676	-0.0648	-34	112	126	YLVVNADEGEPGTCK	65	99.999	(N-term)_iTRAQ[0], Lysine(K)_iTRAQ[15], MMTS (C)[14]	[1] F3 030912	270/262	1.319	1.489	1.199	1	Mascot
1982.0653	1982.0261	-0.0392	-20	99	111	WSFMNKPSDGRPK	51	99.979	(N-term)_iTRAQ[0], Lysine(K)_iTRAQ[6,13]	[2] F12 040912	259/251	0.897	0.890	1.046	1	Mascot

Calc. Mass	Obsrv. Mass	± da	± ppm	Start Seq.	End Seq.	Sequence	Ion Score	C. I. %	Modification	Plate [#] Name	Gel Idx/Pos [4700 Sample Name]	iTRAQ Ratio 115/114*	iTRAQ Ratio 116/114*	iTRAQ Ratio 117/114*	Rank	Result Type
2075.1018	2075.092	-0.0098	-5	402	417	GDARPAEIDSLWEISK	45	99.928	(N-term)_iTRAQ[0], Lysine(K)_iTRAQ[16]	[4] F7 and F10+11	350/342	1.114	1.014	1.165	1	Mascot
2345.1594	2345.1052	-0.0542	-23	200	219	GAGAYICGEETALIESIEG K	76	100	(N-term)_iTRAQ[0], Lysine(K)_iTRAQ[20], MMTS (C)[7]	[1] F3 030912	534/526	0.961	1.139	0.879	1	Mascot

110	Glul protein [Rattus norvegicus]	gi|38181948	45721.1	9	464	0.864	0.908	0.803	0.223	0.445	0.186	10	10	10	100

Protein Group

glutamine synthetase [Rattus norvegicus]	gi|204349	45739.2
glutamine synthetase [Rattus norvegicus]	gi|142349612	45720.2

Peptide Information

Calc. Mass	Obsrv. Mass	± da	± ppm	Start Seq.	End Seq.	Sequence	Ion Score	C. I. %	Modification	Plate [#] Name	Gel Idx/Pos [4700 Sample Name]	iTRAQ Ratio 115/114*	iTRAQ Ratio 116/114*	iTRAQ Ratio 117/114*	Rank	Result Type
1228.6381	1228.6995	0.0614	50	214	222	MGDHLWVAR	34	99.028	(N-term)_iTRAQ[0]	[4] F7 and F10+11	1140/1132	0.954	1.084	0.550	1	Mascot
1254.6301	1254.6106	-0.0195	-16	269	276	CIEEAIDK	36	99.413	(N-term)_iTRAQ[0], Lysine(K)_iTRAQ[8], MMTS (C)[1]	[7] F5 120912	211/203	0.883	1.192	0.572	1	Mascot
1318.713	1318.7393	0.0263	20	96	103	LVFCEVFK	58	99.996	(N-term)_iTRAQ[0], Lysine(K)_iTRAQ[8], MMTS (C)[4]	[7] F5 120912	470/462	0.946	0.876	0.825	1	Mascot
1626.823	1626.7825	-0.0405	-25	15	25	QMYMNLPQGEK	67	99.999	(N-term)_iTRAQ[0], Lysine(K)_iTRAQ[11]	[7] F5 120912	191/183	0.887	0.821	0.891	1	Mascot
1995.0493	1994.9928	-0.0565	-28	26	41	IQLMYIWVDGTGEGLR	53	99.987	(N-term)_iTRAQ[0]	[1] F3 030912	508/500	0.740	1.176	0.883	1	Mascot
2091.9958	2091.9346	-0.0612	-29	358	372	TCLLNETGDEPFQYK	75	100	(N-term)_iTRAQ[0], Lysine(K)_iTRAQ[15], MMTS (C)[2]	[1] F3 030912	342/334	0.713	0.570	0.753	1	Mascot
2091.9958	2091.9541	-0.0417	-20	358	372	TCLLNETGDEPFQYK	32	98.303	(N-term)_iTRAQ[0], Lysine(K)_iTRAQ[15], MMTS (C)[2]	[5] F4	264/256	1.472	2.220	1.111	1	Mascot
2206.0386	2205.9758	-0.0628	-28	358	373	TCLLNETGDEPFQYKN	60	99.998	(N-term)_iTRAQ[0], Lysine(K)_iTRAQ[15], MMTS (C)[2]	[1] F3 030912	330/322	0.579	0.754	0.753	1	Mascot
2294.1284	2294.0808	-0.0476	-21	300	319	LTGFHETSNINDFSAGVA NR	50	99.973	(N-term)_iTRAQ[0]	[7] F5 120912	263/255	0.876	0.678	0.913	1	Mascot
2450.2297	2450.3359	0.1062	43	299	319	RLTGFHETSNINDFSAGV ANR	32	98.327	(N-term)_iTRAQ[0]	[4] F7 and F10+11	1163/1155	0.831	0.539	0.948	1	Mascot

111	calcium/calmodulin-dependent protein kinase II, beta 3 isoform [Rattus norvegicus]	gi|603581	70858.6	9	461	1.071	1.073	0.969	0.185	0.259	0.278	10	10	10	100

Protein Group

RecName: Full=Calcium/calmodulin-dependent protein kinase type II subunit beta; Short=CaM kinase II	gi|125287	66293.2

Peptide Information

Calc. Mass	Obsrv. Mass	± da	± ppm	Start Seq.	End Seq.	Sequence	Ion Score	C. I. %	Modification	Plate [#] Name	Gel Idx/Pos [4700 Sample Name]	iTRAQ Ratio 115/114*	iTRAQ Ratio 116/114*	iTRAQ Ratio 117/114*	Rank	Result Type
1080.6664	1080.6154	-0.051	-47	222	227	LYQQIK	33	98.745	(N-term)_iTRAQ[0], Lysine(K)_iTRAQ[6]	[3] F6 and F9	190/182	1.043	1.183	1.117	1	Mascot
1291.7528	1291.7052	-0.0476	-37	302	312	GAILTTMLATR	39	99.692	(N-term)_iTRAQ[0]	[1] F3 030912	340/332	1.080	1.221	0.835	1	Mascot
1432.8086	1432.7491	-0.0595	-42	508	516	FYFENLLAK	45	99.917	(N-term)_iTRAQ[0], Lysine(K)_iTRAQ[9]	[5] F4	326/318	1.279	1.003	1.168	1	Mascot
1432.8086	1432.808	-0.0006	0	508	516	FYFENLLAK	36	99.446	(N-term)_iTRAQ[0], Lysine(K)_iTRAQ[9]	[7] F5 120912	409/401	1.064	1.205	0.593	1	Mascot
1547.8414	1547.7534	-0.088	-57	545	556	LTQYIDGQGRPR	30	97.727	(N-term)_iTRAQ[0]	[6] F8 110912	229/221	1.203	1.127	1.403	1	Mascot
1773.0857	1772.9882	-0.0975	-55	136	147	DLKPENLLLASK	49	99.967	(N-term)_iTRAQ[0], Lysine(K)_iTRAQ[3,1 2]	[6] F8 110912	295/287	1.190	1.424	1.239	1	Mascot
1908.9476	1908.896	-0.0516	-27	10	22	FTDEYQLYEDIGK	52	99.986	(N-term)_iTRAQ[0], Lysine(K)_iTRAQ[13]	[5] F4	266/258	1.024	0.936	1.121	1	Mascot
2369.1548	2369.0718	-0.083	-35	228	246	AGAYDFPSPEWDTVTPE AK	68	100	(N-term)_iTRAQ[0], Lysine(K)_iTRAQ[19]	[1] F3 030912	337/329	1.184	1.355	0.954	1	Mascot
2401.2134	2401.1284	-0.085	-35	464	482	TTEQLIEAVNNGDFEAYA K	81	100	(N-term)_iTRAQ[0], Lysine(K)_iTRAQ[19]	[1] F3 030912	392/384	1.069	0.683	0.747	1	Mascot
2937.3687	2937.2876	-0.0811	-28	483	507	ICDPGLTSFEPEALGNLV EGMDFHR	63	99.999	(N-term)_iTRAQ[0], MMTS (C)[2]	[1] F3 030912	537/529	0.697	0.836	0.814	1	Mascot

112	dihydrolipoyl dehydrogenase, mitochondrial precursor [Rattus norvegicus]	gi|40786469	60083.9	6	458	1.103	1.295	0.993	0.617	0.771	0.372	7	7	7	100

Peptide Information

Calc. Mass	Obsrv. Mass	± da	± ppm	Start Seq.	End Seq.	Sequence	Ion Score	C. I. %	Modification	Plate [#] Name	Gel Idx/Pos [4700 Sample Name]	iTRAQ Ratio	iTRAQ Ratio	iTRAQ Ratio	Rank	Result Type

Calc. Mass	Obsrv. Mass	± da	± ppm	Start Seq.	End Seq.	Sequence	Ion Score	C. I. %	Modification	Plate [#] Name	Gel Idx/Pos [4700 Sample Name]	115/114*	116/114*	117/114*	Rank	Result Type
1415.8621	1415.8809	0.0188	13	133	143	ALTGGIAHLFK	83	100	(N-term)_iTRAQ[0], Lysine(K)_iTRAQ[11]	[3] F6 and F9	1296/1288	1.067	1.560	1.283	1	Mascot
1657.8044	1657.7787	-0.0257	-16	483	495	VCHAHPTLSEAFR	54	99.99	(N-term)_iTRAQ[0], MMTS (C)[2]	[2] F12 040912	281/273	1.074	1.185	1.147	1	Mascot
1984.061	1984.097	0.036	18	405	417	SEEQLKEEGVEFK	49	99.967	(N-term)_iTRAQ[0], Lysine(K)_iTRAQ[6,13]	[3] F6 and F9	1186/1178	0.622	0.719	1.081	1	Mascot
1984.061	1984.1447	0.0837	42	405	417	SEEQLKEEGVEFK	88	100	(N-term)_iTRAQ[0], Lysine(K)_iTRAQ[6,13]	[4] F7 and F10+11	1116/1108	0.946	0.970	1.059	1	Mascot
2080.1133	2080.001	-0.1123	-54	160	177	NQVTATTADGSTQVIGTK	123	100	(N-term)_iTRAQ[0], Lysine(K)_iTRAQ[18]	[1] F3 030912	176/168	1.092	1.238	1.232	1	Mascot
2251.2883	2251.2471	-0.0412	-18	347	365	IPNIFAIGDVVAGPMLAHK	58	99.996	(N-term)_iTRAQ[0], Lysine(K)_iTRAQ[19]	[7] F5 120912	502/494	0.905	1.075	0.980	1	Mascot
3487.7124	3487.6089	-0.1035	-30	450	482	ILGAHILGPGAGEMVNEA ALALEYGASCEDVAR	53	99.987	(N-term)_iTRAQ[0], MMTS (C)[28]	[1] F3 030912	555/547	2.980	3.555	0.469	1	Mascot

113 EH domain-containing protein 3 [Rattus norvegicus] gi|34536836 67571.5 8 458 0.938 0.985 0.747 0.345 0.286 0.380 8 8 8 100

Peptide Information

Calc. Mass	Obsrv. Mass	± da	± ppm	Start Seq.	End Seq.	Sequence	Ion Score	C. I. %	Modification	Plate [#] Name	Gel Idx/Pos [4700 Sample Name]	iTRAQ Ratio 115/114*	iTRAQ Ratio 116/114*	iTRAQ Ratio 117/114*	Rank	Result Type
1357.8453	1357.7881	-0.0572	-42	185	193	IILLFDAHK	58	99.996	(N-term)_iTRAQ[0], Lysine(K)_iTRAQ[9]	[6] F8 110912	374/366	0.942	1.217	0.701	1	Mascot
1656.873	1656.7805	-0.0925	-56	270	280	LFEAEEQDLFK	56	99.994	(N-term)_iTRAQ[0], Lysine(K)_iTRAQ[11]	[5] F4	288/280	0.866	0.742	0.579	1	Mascot
1682.9098	1682.8237	-0.0861	-51	194	205	LDISDEFSEVIK	47	99.953	(N-term)_iTRAQ[0], Lysine(K)_iTRAQ[12]	[5] F4	288/280	0.830	0.798	0.361	1	Mascot
1760.9575	1760.8593	-0.0982	-56	399	410	QEETQRPVQMVK	76	100	(N-term)_iTRAQ[0], Lysine(K)_iTRAQ[12]	[6] F8 110912	148/140	1.246	1.259	1.160	1	Mascot
1769.9432	1769.8501	-0.0931	-53	345	357	EHQISPGDFPNLK	59	99.997	(N-term)_iTRAQ[0], Lysine(K)_iTRAQ[13]	[6] F8 110912	264/256	1.089	0.838	1.120	1	Mascot
1929.0704	1929.1245	0.0541	28	269	280	KLFEAEEQDLFK	81	100	(N-term)_iTRAQ[0], Lysine(K)_iTRAQ[1,12]	[4] F7 and F10+11	1234/1226	1.599	1.511	1.302	1	Mascot
2550.3174	2550.2932	-0.0242	-9	444	461	DKPMYDEIFYTLSPVDGK	39	99.713	(N-term)_iTRAQ[0], Lysine(K)_iTRAQ[2,18]	[4] F7 and F10+11	447/439	0.777	1.036	0.695	1	Mascot
3450.6111	3450.4844	-0.1267	-37	411	443	GGAFEGTLQGPFGHGYG EGAGEGIDDAEWVVAR	43	99.862	(N-term)_iTRAQ[0]	[1] F3 030912	425/417	0.523	0.743	0.562	1	Mascot

114 guanine deaminase [Rattus norvegicus] gi|148747414 55363.4 10 457 1.304 1.594 1.403 0.797 1.043 0.832 14 14 14 100

Protein Group

RecName: Full=Guanine deaminase; Short=Guanase; Short=Guanine aminase; AltName: Full=Guanine aminoh gi|9910706 55478.5

Peptide Information

Calc. Mass	Obsrv. Mass	± da	± ppm	Start Seq.	End Seq.	Sequence	Ion Score	C. I. %	Modification	Plate [#] Name	Gel Idx/Pos [4700 Sample Name]	iTRAQ Ratio 115/114*	iTRAQ Ratio 116/114*	iTRAQ Ratio 117/114*	Rank	Result Type
1142.5966	1142.5487	-0.0479	-42	428	435	FLYLGDDR	38	99.633	(N-term)_iTRAQ[0]	[5] F4	212/204	0.941	1.328	1.541	1	Mascot
1173.6401	1173.5914	-0.0487	-41	106	112	YTFPTEK	38	99.574	(N-term)_iTRAQ[0], Lysine(K)_iTRAQ[7]	[7] F5 120912	170/162	1.063	0.898	1.161	1	Mascot
1395.7729	1395.6733	-0.0996	-71	436	445	NIEEVYVGGK	44	99.908	(N-term)_iTRAQ[0], Lysine(K)_iTRAQ[10]	[5] F4	138/130	0.828	0.685	0.693	1	Mascot
1688.8252	1688.7323	-0.0929	-55	114	126	FQSTDVAEEVYTR	61	99.998	(N-term)_iTRAQ[0]	[1] F3 030912	247/239	1.073	1.721	1.893	1	Mascot
1688.8252	1688.839	0.0138	8	114	126	FQSTDVAEEVYTR	58	99.996	(N-term)_iTRAQ[0]	[8] F13-15 and F1+2	516/508	0.785	1.313	0.942	1	Mascot
1688.8252	1688.8575	0.0323	19	114	126	FQSTDVAEEVYTR	45	99.915	(N-term)_iTRAQ[0]	[8] F13-15 and F1+2	565/557	1.110	1.385	0.988	1	Mascot
1690.7786	1690.7968	0.0182	11	57	66	EWCFKPCEIR	28	95.807	(N-term)_iTRAQ[0], Lysine(K)_iTRAQ[5], MMTS (C)[3,7]	[3] F6 and F9	1354/1346	1.240	1.649	1.473	1	Mascot
1690.7786	1690.853	0.0744	44	57	66	EWCFKPCEIR	38	99.644	(N-term)_iTRAQ[0], Lysine(K)_iTRAQ[5], MMTS (C)[3,7]	[4] F7 and F10+11	1294/1286	0.932	1.536	1.368	1	Mascot
1724.9316	1724.8551	-0.0765	-44	42	53	IVFLEESSQQEK	62	99.998	(N-term)_iTRAQ[0], Lysine(K)_iTRAQ[12]	[5] F4	204/196	1.740	2.203	2.018	1	Mascot
1844.9263	1844.8253	-0.101	-55	113	126	RFQSTDVAEEVYTR	31	98.248	(N-term)_iTRAQ[0]	[6] F8 110912	238/230	1.215	1.641	2.048	1	Mascot
2053.9763	2053.9597	-0.0166	-8	15	30	GTFVHSTWTCPMEVLR	31	98.157	(N-term)_iTRAQ[0], MMTS (C)[10]	[3] F6 and F9	439/431	3.295	4.317	1.824	1	Mascot
2053.9763	2054.0295	0.0532	26	15	30	GTFVHSTWTCPMEVLR	28	96.39	(N-term)_iTRAQ[0], MMTS (C)[10]	[7] F5 120912	416/408	1.305	1.083	0.618	1	Mascot
2278.136	2278.0984	-0.0376	-17	214	231	FSLSCTETLMSELGNIAK	47	99.951	(N-term)_iTRAQ[0], Lysine(K)_iTRAQ[18], MMTS (C)[5]	[1] F3 030912	554/546	1.165	1.352	1.391	1	Mascot
2860.3975	2860.3391	-0.0584	-20	404	427	ASDSPIDLFCGDFVGDIS EAVIQK	67	99.999	(N-term)_iTRAQ[0], Lysine(K)_iTRAQ[24], MMTS (C)[10]	[1] F3 030912	585/577	4.323	4.622	4.007	1	Mascot

115	visinin-like protein 1 [Mus musculus]	gi|6755983	25292.1	8	456	0.788	0.931	0.965	0.571	0.388	0.506	10	10	10	100

Peptide Information

Calc. Mass	Obsrv. Mass	± da	± ppm	Start Seq.	End Seq.	Sequence	Ion Score	C. I. %	Modification	Plate [#] Name	Gel Idx/Pos [4700 Sample Name]	iTRAQ Ratio 115/114*	iTRAQ Ratio 116/114*	iTRAQ Ratio 117/114*	Rank	Result Type
1319.6881	1319.6313	-0.0568	-43	55	63	FFPYGDASK	59	99.997	(N-term)_iTRAQ[0], Lysine(K)_iTRAQ[9]	[7] F5 120912	218/210	0.983	0.991	1.220	1	Mascot
1521.7795	1521.8425	0.063	41	19	28	STEFNEHELK	49	99.97	(N-term)_iTRAQ[0], Lysine(K)_iTRAQ[10]	[4] F7 and F10+11	1064/1056	1.011	0.983	0.919	1	Mascot
1531.8651	1531.7897	-0.0754	-49	8	18	LAPEVMEDLVK	65	99.999	(N-term)_iTRAQ[0], Lysine(K)_iTRAQ[11]	[5] F4	282/274	1.355	1.170	1.088	1	Mascot
1738.9911	1738.9431	-0.048	-28	119	130	VEMLEIIEAIYK	71	100	(N-term)_iTRAQ[0], Lysine(K)_iTRAQ[12]	[5] F4	417/409	0.474	1.234	1.230	1	Mascot
1738.9911	1738.9949	0.0038	2	119	130	VEMLEIIEAIYK	43	99.865	(N-term)_iTRAQ[0], Lysine(K)_iTRAQ[12]	[3] F6 and F9	547/539	0.357	0.366	0.311	1	Mascot
1773.9017	1773.943	0.0413	23	71	83	TFDKNGDGTIDFR	53	99.988	(N-term)_iTRAQ[0], Lysine(K)_iTRAQ[4]	[3] F6 and F9	1187/1179	1.195	0.882	1.719	1	Mascot
1812.0153	1811.9591	-0.0562	-31	43	54	LNLEEFQQLYVK	52	99.983	(N-term)_iTRAQ[0], Lysine(K)_iTRAQ[12]	[5] F4	318/310	1.002	1.041	0.831	1	Mascot
1898.0243	1898.1178	0.0935	49	161	172	NKDDQITLDEFK	60	99.997	(N-term)_iTRAQ[0], Lysine(K)_iTRAQ[2,12]	[4] F7 and F10+11	1155/1147	1.286	1.151	0.968	1	Mascot
2046.9841	2046.8805	-0.1036	-51	101	115	LNWAFNMYDLDGDGK	47	99.954	(N-term)_iTRAQ[0], Lysine(K)_iTRAQ[15]	[5] F4	339/331	0.269	0.727	1.002	1	Mascot
2046.9841	2047.0037	0.0196	10	101	115	LNWAFNMYDLDGDGK	32	98.3	(N-term)_iTRAQ[0], Lysine(K)_iTRAQ[15]	[7] F5 120912	421/413	0.980	1.233	1.077	1	Mascot

116	Malate dehydrogenase 1, NAD (soluble) [Rattus norvegicus]	gi|37590235	41066.2	6	453	1.028	1.072	0.920	0.212	0.244	0.166	12	12	12	100

Protein Group

malate dehydrogenase, cytoplasmic [Rattus norvegicus] gi|15100179 41065.2

Peptide Information

Calc. Mass	Obsrv. Mass	± da	± ppm	Start Seq.	End Seq.	Sequence	Ion Score	C. I. %	Modification	Plate [#] Name	Gel Idx/Pos [4700 Sample Name]	iTRAQ Ratio 115/114*	iTRAQ Ratio 116/114*	iTRAQ Ratio 117/114*	Rank	Result Type
1322.7189	1322.6383	-0.0806	-61	221	230	GEFITTVQQR	57	99.995	(N-term)_iTRAQ[0]	[5] F4	131/123	1.401	1.323	0.921	1	Mascot
1322.7189	1322.6489	-0.07	-53	221	230	GEFITTVQQR	70	100	(N-term)_iTRAQ[0]	[1] F3 030912	204/196	0.938	1.043	0.859	1	Mascot
1537.8135	1537.8157	0.0022	1	299	310	FVEGLPINDFSR	55	99.992	(N-term)_iTRAQ[0]	[8] F13-15 and F1+2	408/400	1.027	0.984	0.850	1	Mascot
1537.8135	1537.8164	0.0029	2	299	310	FVEGLPINDFSR	35	99.203	(N-term)_iTRAQ[0]	[8] F13-15 and F1+2	568/560	1.116	1.068	1.014	1	Mascot
1537.8135	1537.8169	0.0034	2	299	310	FVEGLPINDFSR	37	99.517	(N-term)_iTRAQ[0]	[8] F13-15 and F1+2	466/458	0.881	0.995	0.760	1	Mascot
1537.8135	1537.8241	0.0106	7	299	310	FVEGLPINDFSR	60	99.997	(N-term)_iTRAQ[0]	[8] F13-15 and F1+2	412/404	1.054	1.042	1.147	1	Mascot
1537.8135	1537.8245	0.011	7	299	310	FVEGLPINDFSR	37	99.456	(N-term)_iTRAQ[0]	[8] F13-15 and F1+2	512/504	0.861	1.044	0.724	1	Mascot
1537.8135	1537.8525	0.039	25	299	310	FVEGLPINDFSR	29	96.737	(N-term)_iTRAQ[0]	[8] F13-15 and F1+2	417/409	0.942	0.844	0.818	1	Mascot
2035.0907	2035.0219	-0.0688	-34	126	142	VIVVGNPANTNCLTASK	112	100	(N-term)_iTRAQ[0], Lysine(K)_iTRAQ[17], MMTS (C)[12]	[1] F3 030912	303/295	1.576	1.869	1.208	1	Mascot
2119.0693	2119.1658	0.0965	46	319	334	ELTEEKETAFEFLSSA	51	99.983	(N-term)_iTRAQ[0], Lysine(K)_iTRAQ[6]	[4] F7 and F10+11	1325/1317	0.814	1.075	1.120	1	Mascot
2184.1924	2184.0764	-0.116	-53	206	220	EVGVYEALKDDSWLK	86	100	(N-term)_iTRAQ[0], Lysine(K)_iTRAQ[9,15]	[6] F8 110912	404/396	1.024	1.065	1.013	1	Mascot
2568.3206	2568.4358	0.1152	45	180	199	NVIIWGNHSSTQYPDVNHAK	73	100	(N-term)_iTRAQ[0], Lysine(K)_iTRAQ[20]	[4] F7 and F10+11	1155/1147	0.935	0.812	0.767	1	Mascot

117	cytochrome c, somatic [Mus musculus]	gi|6681095	14427.9	6	452	0.858	1.019	1.006	0.327	0.262	0.421	7	7	7	100

Peptide Information

Calc. Mass	Obsrv. Mass	± da	± ppm	Start Seq.	End Seq.	Sequence	Ion Score	C. I. %	Modification	Plate [#] Name	Gel Idx/Pos [4700 Sample Name]	iTRAQ Ratio 115/114*	iTRAQ Ratio 116/114*	iTRAQ Ratio 117/114*	Rank	Result Type
1067.6533	1067.6333	-0.02	-19	81	87	MIFAGIK	40	99.758	(N-term)_iTRAQ[0], Lysine(K)_iTRAQ[7]	[7] F5 120912	263/255	0.873	1.229	1.324	1	Mascot
1194.7344	1194.7029	-0.0315	-26	93	100	ADLIAYLK	63	99.999	(N-term)_iTRAQ[0], Lysine(K)_iTRAQ[8]	[7] F5 120912	330/322	0.810	0.884	1.187	1	Mascot
1312.7246	1312.7697	0.0451	34	29	39	TGPNLHGLFGR	57	99.995	(N-term)_iTRAQ[0]	[3] F6 and F9	1232/1224	0.863	0.862	0.844	1	Mascot
1718.8595	1718.7749	-0.0846	-49	41	54	TGQAAGFSYTDANK	91	100	(N-term)_iTRAQ[0], Lysine(K)_iTRAQ[14]	[5] F4	112/104	0.423	0.684	0.463	1	Mascot
1718.8595	1718.7983	-0.0612	-36	41	54	TGQAAGFSYTDANK	111	100	(N-term)_iTRAQ[0], Lysine(K)_iTRAQ[14]	[7] F5 120912	146/138	0.995	1.402	1.178	1	Mascot
1991.0569	1991.1414	0.0845	42	40	54	KTGQAAGFSYTDANK	106	100	(N-term)_iTRAQ[0], Lysine(K)_iTRAQ[1,15]	[4] F7 and F10+11	1056/1048	1.020	1.078	1.060	1	Mascot

2284.1416	2284.0837	-0.0579	-25	57	73	GITWGEDTLMEYLENPK	76	100	(N-term)_iTRAQ[0], Lysine(K)_iTRAQ[17]	[1] F3 030912	506/498	1.303	1.176	1.360	1	Mascot

118	band 4.1-like protein 1 isoform L [Rattus norvegicus]	gi|11067407	185567.6	8	452	0.985	1.091	1.160	0.230	0.232	0.382	9	9	9	100

Protein Group

band 4.1-like protein 1 isoform S [Rattus norvegicus] gi|25742695 108100.5

Peptide Information

Calc. Mass	Obsrv. Mass	± da	± ppm	Start Seq.	End Seq.	Sequence	Ion Score	C. I. %	Modification	Plate [#] Name	Gel Idx/Pos [4700 Sample Name]	iTRAQ Ratio 115/114*	iTRAQ Ratio 116/114*	iTRAQ Ratio 117/114*	Rank	Result Type
1571.8468	1571.8203	-0.0265	-17	164	174	SSPWNFAFTVK	33	98.832	(N-term)_iTRAQ[0], Lysine(K)_iTRAQ[11]	[7] F5 120912	373/365	1.013	1.321	1.899	1	Mascot
1589.8931	1589.928	0.0349	22	1381	1391	KIEPEAMLQSR	35	99.136	(N-term)_iTRAQ[0], Lysine(K)_iTRAQ[1]	[3] F6 and F9	1193/1185	0.824	1.058	1.006	1	Mascot
1615.8901	1615.7732	-0.1169	-72	481	491	ELKPEQETTPR	47	99.948	(N-term)_iTRAQ[0], Lysine(K)_iTRAQ[3]	[6] F8 110912	120/112	0.971	1.075	1.091	1	Mascot
1658.8934	1658.8459	-0.0475	-29	280	291	LSMYGVDLHHAK	65	99.999	(N-term)_iTRAQ[0], Lysine(K)_iTRAQ[12]	[2] F12 040912	268/260	1.184	1.017	1.001	1	Mascot
1674.8883	1674.8555	-0.0328	-20	280	291	LSMYGVDLHHAK	30	97.542	(N-term)_iTRAQ[0], Lysine(K)_iTRAQ[12], Oxidation (M)[3]	[2] F12 040912	225/217	1.488	0.915	0.985	1	Mascot
1862.9706	1862.8313	-0.1393	-75	1392	1408	VSTADSTQVDGGAPAAK	115	100	(N-term)_iTRAQ[0], Lysine(K)_iTRAQ[17]	[5] F4	77/69	0.848	0.947	1.018	1	Mascot
2001.1716	2001.2418	0.0702	35	479	491	IKELKPEQETTPR	35	99.233	(N-term)_iTRAQ[0], Lysine(K)_iTRAQ[2,5]	[4] F7 and F10+11	1062/1054	1.174	1.723	2.023	1	Mascot
2032.0419	2032.026	-0.0159	-8	263	278	GMTPGEAEIHFLENAK	69	100	(N-term)_iTRAQ[0], Lysine(K)_iTRAQ[16]	[4] F7 and F10+11	373/365	0.799	0.917	0.997	1	Mascot
2189.1487	2189.1523	0.0036	2	338	353	IRPGEYEQFESTIGFK	53	99.987	(N-term)_iTRAQ[0], Lysine(K)_iTRAQ[16]	[4] F7 and F10+11	364/356	0.766	1.043	0.903	1	Mascot

119	plasma membrane calcium-transporting ATPase 3 [Rattus norvegicus]	gi|158138481	138511	6	451	0.798	0.758	0.688	0.117	0.287	0.143	6	6	6	100

Protein Group

RecName: Full=Plasma membrane calcium-transporting ATPase 3; Short=PMCA3; AltName: Full=Plasma memb gi|14285347 150533

Peptide Information

Calc. Mass	Obsrv. Mass	± da	± ppm	Start Seq.	End Seq.	Sequence	Ion Score	C. I. %	Modification	Plate [#] Name	Gel Idx/Pos [4700 Sample Name]	iTRAQ Ratio 115/114*	iTRAQ Ratio 116/114*	iTRAQ Ratio 117/114*	Rank	Result Type
1063.6173	1063.5909	-0.0264	-25	1086	1092	GQILWFR	30	97.593	(N-term)_iTRAQ[0]	[7] F5 120912	330/322	0.913	0.784	0.531	1	Mascot
1550.8312	1550.7489	-0.0823	-53	549	558	EQIPEDQLYK	49	99.966	(N-term)_iTRAQ[0], Lysine(K)_iTRAQ[10]	[7] F5 120912	172/164	0.963	1.201	0.567	1	Mascot
1930.0491	1929.9447	-0.1044	-54	773	789	QVVAVTGDGTNDGPALK	139	100	(N-term)_iTRAQ[0], Lysine(K)_iTRAQ[17]	[1] F3 030912	207/199	0.811	0.816	0.750	1	Mascot
2169.1536	2169.0759	-0.0777	-36	807	823	EASDIILTDDNFTSIVK	94	100	(N-term)_iTRAQ[0], Lysine(K)_iTRAQ[17]	[1] F3 030912	385/377	0.721	0.657	0.864	1	Mascot
2202.2466	2202.2207	-0.0259	-12	773	790	QVVAVTGDGTNDGPALK K	45	99.929	(N-term)_iTRAQ[0], Lysine(K)_iTRAQ[17, 18]	[4] F7 and F10+11	194/186	0.780	0.419	0.852	1	Mascot
2259.9749	2259.9724	-0.0025	-1	443	460	HLDACETMGNATAICSDK	96	100	(N-term)_iTRAQ[0], Lysine(K)_iTRAQ[18], MMTS (C)[5,15]	[4] F7 and F10+11	378/370	0.641	0.897	0.639	1	Mascot

120	isocitrate dehydrogenase [NAD] subunit beta, mitochondrial precursor [Rattus norvegicus]	gi|55926203	46447.5	8	451	0.768	1.047	1.057	0.635	0.248	0.294	9	9	9	100

Peptide Information

Calc. Mass	Obsrv. Mass	± da	± ppm	Start Seq.	End Seq.	Sequence	Ion Score	C. I. %	Modification	Plate [#] Name	Gel Idx/Pos [4700 Sample Name]	iTRAQ Ratio 115/114*	iTRAQ Ratio 116/114*	iTRAQ Ratio 117/114*	Rank	Result Type
1250.6667	1250.6158	-0.0509	-41	200	207	FAFDYATK	48	99.959	(N-term)_iTRAQ[0], Lysine(K)_iTRAQ[8]	[7] F5 120912	232/224	0.873	1.135	1.065	1	Mascot
1332.7119	1332.6873	-0.0246	-18	115	122	IYTPMEYK	47	99.949	(N-term)_iTRAQ[0], Lysine(K)_iTRAQ[8]	[7] F5 120912	214/206	0.868	1.105	0.925	1	Mascot
1426.712	1426.6456	-0.0664	-47	123	133	GELASYDMQLR	56	99.993	(N-term)_iTRAQ[0]	[1] F3 030912	252/244	0.990	1.105	0.913	1	Mascot
1542.9254	1542.851	-0.0744	-48	136	146	LDLFANVVHVK	68	100	(N-term)_iTRAQ[0], Lysine(K)_iTRAQ[11]	[6] F8 110912	393/385	0.920	0.864	0.935	1	Mascot
1723.8458	1723.738	-0.1078	-63	362	374	DMGGYSTTTDFIK	62	99.998	(N-term)_iTRAQ[0], Lysine(K)_iTRAQ[13]	[5] F4	182/174	1.075	0.984	1.106	1	Mascot
1815.1228	1815.0756	-0.0472	-26	135	146	KLDLFANVVHVK	68	100	(N-term)_iTRAQ[0], Lysine(K)_iTRAQ[1,1 2]	[2] F12 040912	372/364	1.431	1.733	1.536	1	Mascot
1815.1228	1815.1836	0.0608	33	135	146	KLDLFANVVHVK	73	100	(N-term)_iTRAQ[0], Lysine(K)_iTRAQ[1,1 2]	[4] F7 and F10+11	1237/1229	0.762	0.939	0.779	1	Mascot

Calc. Mass	Obsrv. Mass	± da	± ppm	Start Seq.	End Seq.	Sequence	Ion Score	C. I. %	Modification	Plate [#] Name	Gel Idx/Pos [4700 Sample Name]	iTRAQ Ratio 115/114*	iTRAQ Ratio 116/114*	iTRAQ Ratio 117/114*	Rank	Result Type
1995.9016	1995.8207	-0.0809	-41	165	180	EQTEGEYSSLEHESAR	68	100	(N-term)_iTRAQ[0]	[3] F6 and F9	171/163	0.756	0.781	0.879	1	Mascot
2056.0015	2055.9106	-0.0909	-44	82	96	EHHLSEVQNMASEEK	29	96.615	(N-term)_iTRAQ[0], Lysine(K)_iTRAQ[15]	[2] F12 040912	189/181	0.151	1.009	1.688	1	Mascot

121 synaptosomal-associated protein 25 [Mus musculus] gi|6755588 25357.5 8 446 1.242 0.994 0.841 0.438 0.464 0.118 12 12 12 100

Protein Group

SNAP-25b [Rattus norvegicus] gi|1314856 22485.1

Peptide Information

Calc. Mass	Obsrv. Mass	± da	± ppm	Start Seq.	End Seq.	Sequence	Ion Score	C. I. %	Modification	Plate [#] Name	Gel Idx/Pos [4700 Sample Name]	iTRAQ Ratio 115/114*	iTRAQ Ratio 116/114*	iTRAQ Ratio 117/114*	Rank	Result Type
1352.7117	1352.6289	-0.0828	-61	125	135	EQMAISGGFIR	39	99.681	(N-term)_iTRAQ[0]	[5] F4	193/185	1.834	1.644	1.019	1	Mascot
1352.7117	1352.6727	-0.039	-29	125	135	EQMAISGGFIR	52	99.983	(N-term)_iTRAQ[0]	[1] F3 030912	269/261	0.957	0.768	0.717	1	Mascot
1364.7705	1364.731	-0.0395	-29	32	40	MLQLVEESK	49	99.97	(N-term)_iTRAQ[0], Lysine(K)_iTRAQ[9]	[7] F5 120912	278/270	1.021	0.922	0.940	1	Mascot
1464.7614	1464.7233	-0.0381	-26	60	69	IEEGMDQINK	59	99.997	(N-term)_iTRAQ[0], Lysine(K)_iTRAQ[10]	[7] F5 120912	162/154	1.419	0.776	0.849	1	Mascot
1578.7732	1578.687	-0.0862	-55	18	30	ADQLADESLESTR	53	99.988	(N-term)_iTRAQ[0]	[1] F3 030912	199/191	1.486	1.254	0.934	1	Mascot
1804.9235	1804.9119	-0.0116	-6	46	59	TLVMLDEQGEQLER	49	99.967	(N-term)_iTRAQ[0]	[8] F13-15 and F1+2	474/466	1.556	0.729	0.713	1	Mascot
1804.9235	1804.9249	0.0014	1	46	59	TLVMLDEQGEQLER	54	99.99	(N-term)_iTRAQ[0]	[8] F13-15 and F1+2	470/462	1.257	0.997	0.868	1	Mascot
1804.9235	1804.9496	0.0261	14	46	59	TLVMLDEQGEQLER	38	99.599	(N-term)_iTRAQ[0]	[8] F13-15 and F1+2	628/620	2.154	2.596	0.841	1	Mascot
1813.9065	1813.782	-0.1245	-69	104	119	AWGNNQDGVVASQPAR	59	99.997	(N-term)_iTRAQ[0]	[5] F4	113/105	0.683	0.624	0.697	1	Mascot
1813.9065	1813.8075	-0.099	-55	104	119	AWGNNQDGVVASQPAR	60	99.997	(N-term)_iTRAQ[0]	[1] F3 030912	180/172	1.153	1.009	1.029	1	Mascot
1888.8766	1888.8724	-0.0042	-2	162	176	HMALDMGNEIDTQNR	93	100	(N-term)_iTRAQ[0]	[4] F7 and F10+11	233/225	0.991	0.746	0.839	1	Mascot
2086.104	2086.1401	0.0361	17	103	119	KAWGNNQDGVVASQPA R	28	95.649	(N-term)_iTRAQ[0], Lysine(K)_iTRAQ[1]	[3] F6 and F9	1125/1117	1.083	0.926	0.738	1	Mascot

122 guanine nucleotide-binding protein G(i) subunit alpha-2 [Rattus norvegicus] gi|13591955 44967.8 8 447 1.043 1.025 1.070 0.132 0.270 0.273 8 8 8 100

Peptide Information

Calc. Mass	Obsrv. Mass	± da	± ppm	Start Seq.	End Seq.	Sequence	Ion Score	C. I. %	Modification	Plate [#] Name	Gel Idx/Pos [4700 Sample Name]	iTRAQ Ratio 115/114*	iTRAQ Ratio 116/114*	iTRAQ Ratio 117/114*	Rank	Result Type
1345.8301	1345.7644	-0.0657	-49	36	46	LLLLGAGESGK	84	100	(N-term)_iTRAQ[0], Lysine(K)_iTRAQ[11]	[5] F4	219/211	1.116	1.118	1.283	1	Mascot
1387.6941	1387.6503	-0.0438	-32	250	258	LFDSICNNK	36	99.432	(N-term)_iTRAQ[0], Lysine(K)_iTRAQ[9], MMTS (C)[6]	[3] F6 and F9	362/354	0.880	0.840	1.463	1	Mascot
1649.7782	1649.7114	-0.0668	-40	131	143	LWADHGVQACFGR	29	97.031	(N-term)_iTRAQ[0], MMTS (C)[10]	[6] F8 110912	378/370	1.059	0.808	1.057	1	Mascot
1668.9207	1668.8728	-0.0479	-29	182	193	TTGIVETHFTFK	82	100	(N-term)_iTRAQ[0], Lysine(K)_iTRAQ[12]	[4] F7 and F10+11	286/278	1.242	1.237	1.046	1	Mascot
1719.8608	1719.7998	-0.061	-35	87	100	AMGNLQIDFADPQR	47	99.945	(N-term)_iTRAQ[0]	[1] F3 030912	315/307	1.182	1.500	1.259	1	Mascot
1769.7576	1769.6696	-0.088	-50	55	67	IIHEDGYSEEECR	47	99.948	(N-term)_iTRAQ[0], MMTS (C)[12]	[6] F8 110912	220/212	0.958	1.201	0.810	1	Mascot
1863.8848	1863.9038	0.019	10	319	331	EIYTHFTCATDTK	53	99.988	(N-term)_iTRAQ[0], Lysine(K)_iTRAQ[13], MMTS (C)[8]	[4] F7 and F10+11	280/272	1.077	1.009	0.706	1	Mascot
2317.1799	2317.1021	-0.0778	-34	279	296	ITQSPLTICFPEYTGANK	70	100	(N-term)_iTRAQ[0], Lysine(K)_iTRAQ[18], MMTS (C)[9]	[1] F3 030912	473/465	0.890	0.717	1.149	1	Mascot

123 AP-1 complex subunit beta-1 [Rattus norvegicus] gi|8392872 114434.4 11 447 0.871 1.026 0.960 0.251 0.228 0.241 13 13 13 100

Peptide Information

Calc. Mass	Obsrv. Mass	± da	± ppm	Start Seq.	End Seq.	Sequence	Ion Score	C. I. %	Modification	Plate [#] Name	Gel Idx/Pos [4700 Sample Name]	iTRAQ Ratio 115/114*	iTRAQ Ratio 116/114*	iTRAQ Ratio 117/114*	Rank	Result Type
927.5913	927.5652	-0.0261	-28	323	327	VFFVK	30	97.642	(N-term)_iTRAQ[0], Lysine(K)_iTRAQ[5]	[3] F6 and F9	276/268	0.689	1.146	1.283	1	Mascot
1001.5328	1001.515	-0.0178	-18	523	528	GYIYWR	32	98.338	(N-term)_iTRAQ[0]	[3] F6 and F9	278/270	0.599	0.615	0.615	1	Mascot
1229.7827	1229.7479	-0.0348	-28	305	312	NINLIVQK	43	99.886	(N-term)_iTRAQ[0], Lysine(K)_iTRAQ[8]	[7] F5 120912	198/190	0.659	0.823	1.064	1	Mascot
1299.7195	1299.6555	-0.064	-49	328	335	YNDPIYVK	33	98.627	(N-term)_iTRAQ[0], Lysine(K)_iTRAQ[8]	[7] F5 120912	179/171	0.982	1.190	1.066	1	Mascot
1361.6295	1361.5603	-0.0692	-51	94	103	DCEDPNPLIR	28	96.439	(N-term)_iTRAQ[0], MMTS (C)[2]	[1] F3 030912	235/227	0.620	1.163	1.095	1	Mascot
1395.8583	1395.9244	0.0661	47	12	19	KGEIFELK	43	99.887	(N-term)_iTRAQ[0], Lysine(K)_iTRAQ[1,8]	[4] F7 and F10+11	1144/1136	0.907	1.168	0.745	1	Mascot
1571.7788	1571.8212	0.0424	27	129	138	CLKDEDPYVR	30	97.576	(N-term)_iTRAQ[0], Lysine(K)_iTRAQ[3],	[3] F6 and F9	1187/1179	0.684	0.880	0.781	1	Mascot

Calc. Mass	Obsrv. Mass	± da	± ppm	Start Seq.	End Seq.	Sequence	Ion Score	C. I. %	Modification	Plate [#] Name	Gel Idx/Pos [4700 Sample Name]	iTRAQ Ratio 115/114*	iTRAQ Ratio 116/114*	iTRAQ Ratio 117/114*	Rank	Result Type
									MMTS (C)[1]							
1586.7822	1586.7245	-0.0577	-36	360	371	EYATEVDVDFVR	50	99.973	(N-term)_iTRAQ[0]	[1] F3 030912	296/288	1.273	1.076	1.201	1	Mascot
1586.7822	1586.7748	-0.0074	-5	360	371	EYATEVDVDFVR	76	100	(N-term)_iTRAQ[0]	[8] F13-15 and F1+2	474/466	1.126	1.045	0.787	1	Mascot
1586.7822	1586.797	0.0148	9	360	371	EYATEVDVDFVR	49	99.972	(N-term)_iTRAQ[0]	[8] F13-15 and F1+2	477/469	0.870	1.026	0.845	1	Mascot
1643.9401	1643.871	-0.0691	-42	809	820	MEPLNNLQVAVK	42	99.851	(N-term)_iTRAQ[0], Lysine(K)_iTRAQ[12]	[5] F4	216/208	1.030	1.115	0.959	1	Mascot
1717.8629	1717.7738	-0.0891	-52	855	867	DIPNENEAQFQIR	41	99.798	(N-term)_iTRAQ[0]	[1] F3 030912	238/230	1.149	0.930	1.002	1	Mascot
1913.0103	1913.0256	0.0153	8	115	127	VDKITEYLCEPLR	48	99.965	(N-term)_iTRAQ[0], Lysine(K)_iTRAQ[3], MMTS (C)[9]	[4] F7 and F10+11	450/442	1.102	1.414	1.346	1	Mascot

124	plectin 2 [Rattus norvegicus]	gi|40849888	555413.9	11	446	0.921	0.959	0.888	0.505	0.300	0.451	11	11	11	100

Protein Group

RecName: Full=Plectin; Short=PCN; Short=PLTN; AltName: Full=Plectin-1	gi|1709655	572820.1
plectin 1 [Rattus norvegicus]	gi|40849886	558584.5
plectin 10 [Rattus norvegicus]	gi|40849904	557125.6
plectin 11 [Rattus norvegicus]	gi|40849906	556396.6
plectin 3 [Rattus norvegicus]	gi|40849890	553690.1
plectin 4 [Rattus norvegicus]	gi|40849892	544254.9
plectin 6 [Rattus norvegicus]	gi|40849896	573058.4
plectin 7 [Rattus norvegicus]	gi|40849898	552565.6
plectin 8 [Rattus norvegicus]	gi|40849900	556116.4

Peptide Information

Calc. Mass	Obsrv. Mass	± da	± ppm	Start Seq.	End Seq.	Sequence	Ion Score	C. I. %	Modification	Plate [#] Name	Gel Idx/Pos [4700 Sample Name]	iTRAQ Ratio 115/114*	iTRAQ Ratio 116/114*	iTRAQ Ratio 117/114*	Rank	Result Type
1377.6705	1377.7186	0.0481	35	549	558	GMHQSIEEFR	35	99.18	(N-term)_iTRAQ[0]	[3] F6 and F9	1162/1154	0.916	0.614	0.691	1	Mascot
1386.7825	1386.7059	-0.0766	-55	1551	1561	QVQVALETAQR	36	99.392	(N-term)_iTRAQ[0]	[1] F3 030912	198/190	0.708	0.625	0.620	1	Mascot
1431.8053	1431.7548	-0.0505	-35	1635	1644	LQAEEVAQQK	34	99.072	(N-term)_iTRAQ[0], Lysine(K)_iTRAQ[10]	[7] F5 120912	136/128	0.945	1.199	1.260	1	Mascot
1444.7516	1444.674	-0.0776	-54	2284	2294	QLAEEDLAQQR	40	99.767	(N-term)_iTRAQ[0]	[1] F3 030912	177/169	1.620	1.310	0.868	1	Mascot
1485.8271	1485.858	0.0309	21	1728	1738	LQHEATAATQK	39	99.695	(N-term)_iTRAQ[0], Lysine(K)_iTRAQ[11]	[3] F6 and F9	1077/1069	0.751	0.739	0.783	1	Mascot
1492.788	1492.6979	-0.0901	-60	3177	3187	EPVTYSQLQQR	33	98.649	(N-term)_iTRAQ[0]	[1] F3 030912	185/177	1.664	1.292	1.363	1	Mascot
1716.9153	1716.8315	-0.0838	-49	1126	1139	AQAEAQQPVFNTLR	36	99.348	(N-term)_iTRAQ[0]	[1] F3 030912	227/219	1.388	1.128	0.833	1	Mascot
1926.8954	1926.786	-0.1094	-57	4462	4479	GYYSPYSVSGSGSTAGSR	55	99.993	(N-term)_iTRAQ[0]	[1] F3 030912	196/188	0.657	0.913	0.986	1	Mascot
2171.2056	2171.2285	0.0229	11	2794	2812	LLEAQIATGGIIDPVHSHR	29	97.018	(N-term)_iTRAQ[0]	[3] F6 and F9	1293/1285	0.623	0.865	0.351	1	Mascot
2246.1257	2246.0093	-0.1164	-52	2108	2126	QSAEEQAQAQAQAQAAAEK	85	100	(N-term)_iTRAQ[0], Lysine(K)_iTRAQ[19]	[5] F4	112/104	1.431	1.295	1.693	1	Mascot
2457.1589	2457.0713	-0.0876	-36	4059	4077	GLFDEEMNEILTDPSDDTK	30	97.378	(N-term)_iTRAQ[0], Lysine(K)_iTRAQ[19]	[1] F3 030912	428/420	0.401	0.954	1.115	1	Mascot

125	AP-2 complex subunit alpha-2 [Rattus norvegicus]	gi|162138932	112625.4	11	446	0.930	1.089	0.813	0.519	0.359	0.581	11	11	11	100

Protein Group

RecName: Full=AP-2 complex subunit alpha-2; AltName: Full=100 kDa coated vesicle protein C; AltName	gi|113337	112496.4

Peptide Information

Calc. Mass	Obsrv. Mass	± da	± ppm	Start Seq.	End Seq.	Sequence	Ion Score	C. I. %	Modification	Plate [#] Name	Gel Idx/Pos [4700 Sample Name]	iTRAQ Ratio 115/114*	iTRAQ Ratio 116/114*	iTRAQ Ratio 117/114*	Rank	Result Type
1234.7279	1234.6732	-0.0547	-44	12	21	GLAVFISDIR	50	99.976	(N-term)_iTRAQ[0]	[1] F3 030912	423/415	0.710	1.020	0.489	1	Mascot

Calc. Mass	Obsrv. Mass	± da	± ppm	Start Seq.	End Seq.	Sequence	Ion Score	C. I. %	Modification	Plate [#] Name	Gel Idx/Pos [4700 Sample Name]	iTRAQ Ratio 115/114*	iTRAQ Ratio 116/114*	iTRAQ Ratio 117/114*	Rank	Result Type
1307.7068	1307.6842	-0.0226	-17	738	745	MFIFYGNK	31	97.922	(N-term)_iTRAQ[0], Lysine(K)_iTRAQ[8]	[3] F6 and F9	339/331	1.114	1.047	0.260	1	Mascot
1459.6848	1459.6503	-0.0345	-24	389	399	AVDLLYAMCDR	36	99.341	(N-term)_iTRAQ[0], MMTS (C)[9]	[1] F3 030912	501/493	1.873	1.964	2.203	1	Mascot
1487.8104	1487.7362	-0.0742	-50	808	818	YGGTFQNVSVK	64	99.999	(N-term)_iTRAQ[0], Lysine(K)_iTRAQ[11]	[7] F5 120912	181/173	0.923	1.154	0.927	1	Mascot
1506.7859	1506.7183	-0.0676	-45	907	917	LEPNLQAQMYR	34	99.061	(N-term)_iTRAQ[0]	[1] F3 030912	252/244	0.665	1.140	0.941	1	Mascot
1536.8553	1536.754	-0.1013	-66	154	165	ILVAGDTMDSVK	34	99.114	(N-term)_iTRAQ[0], Lysine(K)_iTRAQ[12]	[5] F4	182/174	0.909	1.090	0.903	1	Mascot
1580.824	1580.772	-0.052	-33	142	153	EMAEAFAGEIPK	43	99.871	(N-term)_iTRAQ[0], Lysine(K)_iTRAQ[12]	[5] F4	220/212	1.368	0.973	0.697	1	Mascot
1610.8724	1610.8131	-0.0593	-37	280	290	LTECLETILNK	54	99.99	(N-term)_iTRAQ[0], Lysine(K)_iTRAQ[11], MMTS (C)[4]	[5] F4	402/394	0.341	0.604	0.635	1	Mascot
1730.851	1730.7954	-0.0556	-32	452	464	IAGDYVSEEVWYR	30	97.774	(N-term)_iTRAQ[0]	[1] F3 030912	335/327	1.388	1.565	1.329	1	Mascot
1961.0702	1960.9626	-0.1076	-55	843	856	QLSNPQQEVQNIFK	33	98.737	(N-term)_iTRAQ[0], Lysine(K)_iTRAQ[14]	[5] F4	242/234	1.192	1.034	1.269	1	Mascot
1992.0674	1992.0785	0.0111	6	499	516	VGGYILGEFGNLIAGDPR	36	99.41	(N-term)_iTRAQ[0]	[8] F13-15 and F1+2	272/264	0.706	0.893	0.626	1	Mascot

126	heat shock 70 kDa protein 4 [Rattus norvegicus]	gi|24025637	106313.2	11	444	1.013	0.971	0.863	0.249	0.278	0.298	11	11	11	100

Peptide Information

Calc. Mass	Obsrv. Mass	± da	± ppm	Start Seq.	End Seq.	Sequence	Ion Score	C. I. %	Modification	Plate [#] Name	Gel Idx/Pos [4700 Sample Name]	iTRAQ Ratio 115/114*	iTRAQ Ratio 116/114*	iTRAQ Ratio 117/114*	Rank	Result Type
1226.6813	1226.6744	-0.0069	-6	705	711	QIQQYMK	37	99.512	(N-term)_iTRAQ[0], Lysine(K)_iTRAQ[7]	[3] F6 and F9	188/180	0.885	1.136	1.140	1	Mascot
1583.9003	1583.8173	-0.083	-52	186	196	QDLPALEEKPR	27	95.273	(N-term)_iTRAQ[0], Lysine(K)_iTRAQ[9]	[6] F8 110912	197/189	1.121	0.935	0.654	1	Mascot
1639.8047	1639.7368	-0.0679	-41	20	33	AGGIETIANEYSDR	51	99.979	(N-term)_iTRAQ[0]	[1] F3 030912	259/251	1.176	1.231	1.084	1	Mascot
1671.851	1671.7413	-0.1097	-66	592	603	EMLGLYTENEGK	40	99.764	(N-term)_iTRAQ[0], Lysine(K)_iTRAQ[12]	[5] F4	177/169	0.788	0.887	0.607	1	Mascot
1715.7979	1715.7311	-0.0668	-39	737	748	STNEAMEWMNSK	38	99.62	(N-term)_iTRAQ[0], Lysine(K)_iTRAQ[12]	[7] F5 120912	233/225	1.494	1.097	1.525	1	Mascot
1872.9701	1872.8772	-0.0929	-50	639	651	FVSEDDRNNFTLK	39	99.712	(N-term)_iTRAQ[0], Lysine(K)_iTRAQ[13]	[6] F8 110912	259/251	0.833	0.854	0.638	1	Mascot
1880.0289	1880.041	0.0121	6	391	405	EFSITDVVPYPISLR	57	99.995	(N-term)_iTRAQ[0]	[8] F13-15 and F1+2	333/325	0.727	0.541	0.779	1	Mascot
2340.1514	2340.2542	0.1028	44	718	733	NKEDQYEHLDAADMTK	42	99.833	(N-term)_iTRAQ[0], Lysine(K)_iTRAQ[2,16]	[4] F7 and F10+11	1094/1086	0.977	0.839	0.866	1	Mascot
2419.2141	2419.2927	0.0786	32	235	250	KFDEVLVNHFCEEFGK	51	99.979	(N-term)_iTRAQ[0], Lysine(K)_iTRAQ[1,16], MMTS (C)[11]	[4] F7 and F10+11	1340/1332	1.098	1.317	0.683	1	Mascot
2671.2263	2671.116	-0.1103	-41	503	521	SEESEEPMETDQNAKEEEK	30	97.531	(N-term)_iTRAQ[0], Lysine(K)_iTRAQ[15,19]	[4] F7 and F10+11	129/121	0.932	0.837	0.729	1	Mascot
2881.3521	2881.2224	-0.1297	-45	522	543	MQVDQEEPHTEEQQPQTPAENK	35	99.261	(N-term)_iTRAQ[0], Lysine(K)_iTRAQ[22]	[7] F5 120912	134/126	1.394	1.324	1.238	1	Mascot

127	RecName: Full=Septin-7; AltName: Full=CDC10 protein homolog	gi|9789715	57524.6	7	443	1.031	0.953	1.022	0.100	0.202	0.181	8	8	8	100

Peptide Information

Calc. Mass	Obsrv. Mass	± da	± ppm	Start Seq.	End Seq.	Sequence	Ion Score	C. I. %	Modification	Plate [#] Name	Gel Idx/Pos [4700 Sample Name]	iTRAQ Ratio 115/114*	iTRAQ Ratio 116/114*	iTRAQ Ratio 117/114*	Rank	Result Type
1387.6614	1387.5737	-0.0877	-63	137	146	FEDYLNAESR	56	99.994	(N-term)_iTRAQ[0]	[5] F4	182/174	0.887	1.369	1.295	1	Mascot
1476.8268	1476.8982	0.0714	48	371	380	LKDSEAELQR	53	99.988	(N-term)_iTRAQ[0], Lysine(K)_iTRAQ[2]	[4] F7 and F10+11	1050/1042	1.004	0.894	1.048	1	Mascot
1489.7554	1489.6887	-0.0667	-45	333	343	SPLAQMEEERR	35	99.225	(N-term)_iTRAQ[0]	[6] F8 110912	211/203	1.059	0.761	1.025	1	Mascot
1667.7898	1667.7155	-0.0743	-45	298	309	DVTNNVHYENYR	66	99.999	(N-term)_iTRAQ[0]	[6] F8 110912	159/151	1.100	0.974	0.939	1	Mascot
1667.7898	1667.8164	0.0266	16	298	309	DVTNNVHYENYR	28	96.398	(N-term)_iTRAQ[0]	[4] F7 and F10+11	155/147	1.052	0.755	0.867	1	Mascot
1843.8796	1843.8107	-0.0689	-37	195	207	ADTLTPEECQQFK	86	100	(N-term)_iTRAQ[0], Lysine(K)_iTRAQ[13], MMTS (C)[9]	[5] F4	216/208	0.968	0.884	0.817	1	Mascot
2045.9437	2045.8337	-0.11	-54	221	234	IYEFPETDDEEENK	85	100	(N-term)_iTRAQ[0], Lysine(K)_iTRAQ[14]	[5] F4	166/158	0.977	0.953	0.974	1	Mascot
2122.0427	2122.1047	0.062	29	351	363	KMEMEMEQVFEMK	61	99.998	(N-term)_iTRAQ[0], Lysine(K)_iTRAQ[1,13]	[4] F7 and F10+11	1284/1276	1.236	1.186	1.327	1	Mascot

128	protein phosphatase 1 [Mus musculus]	gi|471976	42247.5	8	441	0.946	0.786	0.935	0.287	0.198	0.413	8	8	8	100

Protein Group

protein phosphatase 1, catalytic subunit [Rattus norvegicus]	gi|220873	42281.5
serine/threonine-protein phosphatase PP1-gamma catalytic subunit [Rattus norvegicus]	gi|11968062	41016

Peptide Information

Calc. Mass	Obsrv. Mass	± da	± ppm	Start Seq.	End Seq.	Sequence	Ion Score	C. I. %	Modification	Plate [#] Name	Gel Idx/Pos [4700 Sample Name]	iTRAQ Ratio 115/114*	iTRAQ Ratio 116/114*	iTRAQ Ratio 117/114*	Rank	Result Type
1342.7279	1342.6913	-0.0366	-27	114	122	YPENFFLLR	48	99.964	(N-term)_iTRAQ[0]	[1] F3 030912	408/400	0.861	1.060	0.844	1	Mascot
1386.7461	1386.6702	-0.0759	-55	27	36	NVQLQENEIR	40	99.778	(N-term)_iTRAQ[0]	[1] F3 030912	179/171	0.894	0.579	0.436	1	Mascot
1728.0094	1727.9452	-0.0642	-37	112	122	IKYPENFFLLR	65	99.999	(N-term)_iTRAQ[0], Lysine(K)_iTRAQ[2]	[6] F8 110912	421/413	0.948	0.980	1.128	1	Mascot
1829.0143	1828.9751	-0.0392	-21	99	111	QSLETICLLLAYK	39	99.702	(N-term)_iTRAQ[0], Lysine(K)_iTRAQ[13], MMTS (C)[7]	[5] F4	419/411	1.086	1.055	0.889	1	Mascot
1855.8936	1855.8951	0.0015	1	61	74	ICGDIHGQYYDLLR	48	99.961	(N-term)_iTRAQ[0], MMTS (C)[2]	[4] F7 and F10+11	404/396	0.749	0.707	1.089	1	Mascot
1927.98	1927.9174	-0.0626	-32	247	260	AHQVVEDGYEFFAK	89	100	(N-term)_iTRAQ[0], Lysine(K)_iTRAQ[14]	[6] F8 110912	362/354	0.722	0.750	0.900	1	Mascot
2241.3354	2241.2866	-0.0488	-22	44	60	EIFLSQPILLELEAPLK	73	100	(N-term)_iTRAQ[0], Lysine(K)_iTRAQ[17]	[1] F3 030912	543/535	0.846	0.615	0.909	1	Mascot
2380.127	2380.0686	-0.0584	-25	151	168	TFTDCFNCLPIAAIVDEK	38	99.636	(N-term)_iTRAQ[0], Lysine(K)_iTRAQ[18], MMTS (C)[5,8]	[1] F3 030912	574/566	1.768	0.702	1.780	1	Mascot

129	DeltaSAC-synaptojanin1 [Rattus norvegicus]	gi|3367736	107230.2	8	438	0.820	0.865	1.067	0.359	0.352	0.380	8	8	8	100	

Peptide Information

Calc. Mass	Obsrv. Mass	± da	± ppm	Start Seq.	End Seq.	Sequence	Ion Score	C. I. %	Modification	Plate [#] Name	Gel Idx/Pos [4700 Sample Name]	iTRAQ Ratio 115/114*	iTRAQ Ratio 116/114*	iTRAQ Ratio 117/114*	Rank	Result Type
1382.6134	1382.5934	-0.02	-14	119	126	NMCENFYK	27	95.37	(N-term)_iTRAQ[0], Lysine(K)_iTRAQ[8], MMTS (C)[3]	[3] F6 and F9	304/296	0.331	1.194	2.198	1	Mascot
1521.7782	1521.8302	0.052	34	299	309	ERNEDFVEIAR	60	99.997	(N-term)_iTRAQ[0]	[3] F6 and F9	1176/1168	1.133	0.928	0.787	1	Mascot
1552.8428	1552.7866	-0.0562	-36	104	115	VSEQTLQSASSK	83	100	(N-term)_iTRAQ[0], Lysine(K)_iTRAQ[12]	[7] F5 120912	138/130	1.146	1.088	0.931	1	Mascot
1571.8302	1571.8673	0.0371	24	738	752	EFGGVGAPPSPGVTR	27	95.118	(N-term)_iTRAQ[0]	[8] F13-15 and F1+2	553/545	0.882	0.365	0.797	1	Mascot
1592.9006	1592.9795	0.0789	50	168	178	LAGIQEFQDKR	41	99.811	(N-term)_iTRAQ[0], Lysine(K)_iTRAQ[10]	[4] F7 and F10+11	1133/1125	0.831	0.903	0.950	1	Mascot
1899.8746	1899.8063	-0.0683	-36	386	398	YDLFSEDYDTSEK	56	99.994	(N-term)_iTRAQ[0], Lysine(K)_iTRAQ[13]	[5] F4	211/203	0.873	0.848	1.118	1	Mascot
1977.124	1977.0081	-0.1159	-59	816	829	LTPESQSKPLETSK	69	100	(N-term)_iTRAQ[0], Lysine(K)_iTRAQ[8,14]	[6] F8 110912	169/161	0.864	0.951	1.149	1	Mascot
2238.1975	2238.0725	-0.125	-56	675	694	TPGPLSSQGAPVDTQPAAQK	76	100	(N-term)_iTRAQ[0], Lysine(K)_iTRAQ[20]	[1] F3 030912	192/184	0.864	0.976	1.075	1	Mascot

130	isocitrate dehydrogenase [NAD] subunit alpha, mitochondrial precursor [Rattus norvegicus]	gi|16758446	43702.6	9	437	0.921	0.909	0.970	0.248	0.334	0.144	12	12	12	100	

Peptide Information

Calc. Mass	Obsrv. Mass	± da	± ppm	Start Seq.	End Seq.	Sequence	Ion Score	C. I. %	Modification	Plate [#] Name	Gel Idx/Pos [4700 Sample Name]	iTRAQ Ratio 115/114*	iTRAQ Ratio 116/114*	iTRAQ Ratio 117/114*	Rank	Result Type
1172.6184	1172.5599	-0.0585	-50	59	66	APIQWEER	29	97.018	(N-term)_iTRAQ[0]	[7] F5 120912	174/166	0.803	0.837	0.900	1	Mascot
1326.7338	1326.6909	-0.0429	-32	206	214	MSDGLFLQK	50	99.974	(N-term)_iTRAQ[0], Lysine(K)_iTRAQ[9]	[7] F5 120912	246/238	1.323	1.477	1.149	1	Mascot
1360.7021	1360.6454	-0.0567	-42	179	188	IAEFAFEYAR	52	99.983	(N-term)_iTRAQ[0]	[5] F4	280/272	0.963	0.789	0.940	1	Mascot
1360.7021	1360.6688	-0.0333	-24	179	188	IAEFAFEYAR	31	98.24	(N-term)_iTRAQ[0]	[1] F3 030912	365/357	0.533	0.539	0.794	1	Mascot
1400.7509	1400.7239	-0.027	-19	327	336	IEAACFATIK	34	98.91	(N-term)_iTRAQ[0], Lysine(K)_iTRAQ[10], MMTS (C)[5]	[7] F5 120912	351/343	0.856	1.034	1.195	1	Mascot
1414.7511	1414.8453	0.0942	67	317	326	HMGLFDHAAK	75	100	(N-term)_iTRAQ[0], Lysine(K)_iTRAQ[10]	[8] F13-15 and F1+2	1540/1532	1.328	1.012	0.979	1	Mascot
1438.5557	1438.526	-0.0297	-21	351	360	CSDFTEEICR	34	98.959	(N-term)_iTRAQ[0], MMTS (C)[1,9]	[1] F3 030912	380/372	0.879	0.824	0.879	1	Mascot
1535.8553	1535.8491	-0.0062	-4	135	146	TPYTDVNIVTIR	51	99.98	(N-term)_iTRAQ[0]	[8] F13-15 and F1+2	474/466	0.977	0.927	1.023	1	Mascot
1535.8553	1535.8721	0.0168	11	135	146	TPYTDVNIVTIR	52	99.983	(N-term)_iTRAQ[0]	[8] F13-15 and F1+2	477/469	0.755	0.988	0.954	1	Mascot
1535.8553	1535.8738	0.0185	12	135	146	TPYTDVNIVTIR	54	99.989	(N-term)_iTRAQ[0]	[8] F13-15 and F1+2	578/570	1.137	1.677	1.100	1	Mascot
1734.981	1734.9409	-0.0401	-23	101	115	TPIAAGHPSMNLLLR	49	99.968	(N-term)_iTRAQ[0]	[7] F5 120912	298/290	0.937	0.783	0.746	1	Mascot
1991.0612	1991.0273	-0.0339	-17	300	316	DMANPTALLLSAVMMLR	62	99.999	(N-term)_iTRAQ[0]	[1] F3 030912	607/599	0.868	0.582	1.090	1	Mascot

131	ras-related protein Rab-3A [Mus musculus]	gi|6679593	27155.4	6	437	0.806	1.044	1.041	0.222	0.169	0.125	7	7	7	100	

Peptide Information

Calc. Mass	Obsrv. Mass	± da	± ppm	Start Seq.	End Seq.	Sequence	Ion Score	C. I. %	Modification	Plate [#] Name	Gel Idx/Pos [4700 Sample Name]	iTRAQ Ratio	iTRAQ Ratio	iTRAQ Ratio	Rank	Result Type

Calc. Mass	Obsrv. Mass	± da	± ppm	Start Seq.	End Seq.	Sequence	Ion Score	C. I. %	Modification	Plate [#] Name	Gel Idx/Pos [4700 Sample Name]	iTRAQ Ratio 115/114*	iTRAQ Ratio 116/114*	iTRAQ Ratio 117/114*	Rank	Result Type
1252.6871	1252.6587	-0.0284	-23	179	186	LVDVICEK	49	99.971	(N-term)_iTRAQ[0], Lysine(K)_iTRAQ[8], MMTS (C)[6]	[7] F5 120912	330/322	0.862	0.965	1.169	1	Mascot
1460.7618	1460.6924	-0.0694	-48	73	83	LQIWDTAGQER	70	100	(N-term)_iTRAQ[0]	[5] F4	197/189	0.762	1.084	0.943	1	Mascot
1798.8203	1798.813	-0.0073	-4	13	24	ESSDQNFDYMFK	76	100	(N-term)_iTRAQ[0], Lysine(K)_iTRAQ[12]	[7] F5 120912	259/251	0.745	1.071	1.130	1	Mascot
1880.9521	1880.8672	-0.0849	-45	187	202	MSESLDTADPAVTGAK	94	100	(N-term)_iTRAQ[0], Lysine(K)_iTRAQ[16]	[1] F3 030912	213/205	0.723	0.821	0.944	1	Mascot
1996.075	1995.9971	-0.0779	-39	122	136	TYSWDNAQVLLVGNK	43	99.865	(N-term)_iTRAQ[0], Lysine(K)_iTRAQ[15]	[5] F4	270/262	0.549	1.082	0.883	1	Mascot
2098.0854	2098.0007	-0.0847	-40	152	167	QLADHLGFEFFEASAK	60	99.997	(N-term)_iTRAQ[0], Lysine(K)_iTRAQ[16]	[6] F8 110912	421/413	1.307	1.398	1.210	1	Mascot
2098.0854	2098.1074	0.022	10	152	167	QLADHLGFEFFEASAK	106	100	(N-term)_iTRAQ[0], Lysine(K)_iTRAQ[16]	[4] F7 and F10+11	419/411	0.872	0.973	1.052	1	Mascot

132	myelin proteolipid protein [Rattus norvegicus]	gi|13591880	32574.2	7	437	1.855	0.958	0.740	0.615	0.263	0.239	17	17	17	100

Peptide Information

Calc. Mass	Obsrv. Mass	± da	± ppm	Start Seq.	End Seq.	Sequence	Ion Score	C. I. %	Modification	Plate [#] Name	Gel Idx/Pos [4700 Sample Name]	iTRAQ Ratio 115/114*	iTRAQ Ratio 116/114*	iTRAQ Ratio 117/114*	Rank	Result Type
1139.5405	1139.5735	0.033	29	138	144	VCHCLGK	47	99.947	(N-term)_iTRAQ[0], Lysine(K)_iTRAQ[7], MMTS (C)[2,4]	[4] F7 and F10+11	1151/1143	3.563	1.687	0.743	1	Mascot
1158.6405	1158.5991	-0.0414	-36	99	105	QIFGDYK	45	99.925	(N-term)_iTRAQ[0], Lysine(K)_iTRAQ[7]	[3] F6 and F9	218/210	1.962	0.946	0.900	1	Mascot
1158.6405	1158.6606	0.0201	17	99	105	QIFGDYK	40	99.777	(N-term)_iTRAQ[0], Lysine(K)_iTRAQ[7]	[3] F6 and F9	553/545	3.041	0.966	0.622	1	Mascot
1158.6405	1158.7115	0.071	61	99	105	QIFGDYK	42	99.859	(N-term)_iTRAQ[0], Lysine(K)_iTRAQ[7]	[3] F6 and F9	278/270	1.760	1.007	0.604	1	Mascot
1288.7399	1288.6951	-0.0448	-35	46	53	LIETYFSK	55	99.993	(N-term)_iTRAQ[0], Lysine(K)_iTRAQ[8]	[7] F5 120912	291/283	2.003	1.003	0.773	1	Mascot
1288.7399	1288.7135	-0.0264	-20	46	53	LIETYFSK	30	97.39	(N-term)_iTRAQ[0], Lysine(K)_iTRAQ[8]	[7] F5 120912	493/485	1.445	0.540	0.920	1	Mascot
1288.7399	1288.7915	0.0516	40	46	53	LIETYFSK	34	99.093	(N-term)_iTRAQ[0], Lysine(K)_iTRAQ[8]	[7] F5 120912	723/715	1.339	1.104	1.270	1	Mascot
1288.7399	1288.8353	0.0954	74	46	53	LIETYFSK	48	99.964	(N-term)_iTRAQ[0], Lysine(K)_iTRAQ[8]	[8] F13-15 and F1+2	935/927	1.875	0.816	0.916	1	Mascot
1306.7576	1306.696	-0.0616	-47	112	122	GLSATVTGGQK	83	100	(N-term)_iTRAQ[0], Lysine(K)_iTRAQ[11]	[7] F5 120912	125/117	2.283	1.099	0.736	1	Mascot
1441.688	1441.6317	-0.0563	-39	193	205	TSASIGSLCADAR	73	100	(N-term)_iTRAQ[0], MMTS (C)[9]	[1] F3 030912	296/288	2.128	1.138	0.788	1	Mascot
1516.7567	1516.724	-0.0327	-22	219	229	VCGSNLLSICK	68	100	(N-term)_iTRAQ[0], Lysine(K)_iTRAQ[11], MMTS (C)[2,10]	[7] F5 120912	438/430	1.711	0.983	0.868	1	Mascot
1767.9502	1767.8738	-0.0764	-43	206	218	MYGVLPWNAFPGK	67	99.999	(N-term)_iTRAQ[0], Lysine(K)_iTRAQ[13]	[5] F4	327/319	1.840	1.055	0.848	1	Mascot
1767.9502	1767.8798	-0.0704	-40	206	218	MYGVLPWNAFPGK	53	99.988	(N-term)_iTRAQ[0], Lysine(K)_iTRAQ[13]	[6] F8 110912	443/435	1.907	0.769	0.913	1	Mascot
1767.9502	1767.9003	-0.0499	-28	206	218	MYGVLPWNAFPGK	49	99.966	(N-term)_iTRAQ[0], Lysine(K)_iTRAQ[13]	[6] F8 110912	384/376	1.658	0.830	0.649	1	Mascot
1767.9502	1767.9396	-0.0106	-6	206	218	MYGVLPWNAFPGK	35	99.208	(N-term)_iTRAQ[0], Lysine(K)_iTRAQ[13]	[4] F7 and F10+11	385/377	1.024	0.668	0.542	1	Mascot
1783.9451	1783.8599	-0.0852	-48	206	218	MYGVLPWNAFPGK	33	98.777	(N-term)_iTRAQ[0], Lysine(K)_iTRAQ[13], Oxidation (M)[1]	[5] F4	329/321	2.005	1.171	0.342	1	Mascot
1783.9451	1783.8898	-0.0553	-31	206	218	MYGVLPWNAFPGK	37	99.488	(N-term)_iTRAQ[0], Lysine(K)_iTRAQ[13], Oxidation (M)[1]	[5] F4	293/285	1.346	0.985	0.617	1	Mascot

133	14-3-3 protein eta [Mus musculus]	gi|6756037	31358.1	8	436	0.979	1.131	1.010	0.470	0.386	0.241	10	10	10	100

Peptide Information

Calc. Mass	Obsrv. Mass	± da	± ppm	Start Seq.	End Seq.	Sequence	Ion Score	C. I. %	Modification	Plate [#] Name	Gel Idx/Pos [4700 Sample Name]	iTRAQ Ratio 115/114*	iTRAQ Ratio 116/114*	iTRAQ Ratio 117/114*	Rank	Result Type
957.6019	957.6076	0.0057	6	121	125	VFYLK	34	98.922	(N-term)_iTRAQ[0], Lysine(K)_iTRAQ[5]	[3] F6 and F9	262/254	1.285	1.183	1.199	1	Mascot
1191.7195	1191.6932	-0.0263	-22	62	69	VISSIEQK	55	99.992	(N-term)_iTRAQ[0], Lysine(K)_iTRAQ[8]	[7] F5 120912	159/151	1.339	1.437	1.443	1	Mascot
1195.7296	1195.6782	-0.0514	-43	43	50	NLLSVAYK	53	99.987	(N-term)_iTRAQ[0], Lysine(K)_iTRAQ[8]	[7] F5 120912	233/225	1.100	1.296	1.119	1	Mascot
1333.7634	1333.7305	-0.0329	-25	218	227	DSTLIMQLLR	42	99.831	(N-term)_iTRAQ[0]	[1] F3 030912	466/458	1.966	2.108	0.925	1	Mascot
1333.7634	1333.7313	-0.0321	-24	218	227	DSTLIMQLLR	48	99.963	(N-term)_iTRAQ[0]	[2] F12 040912	472/464	1.159	0.774	0.827	1	Mascot
1354.7463	1354.729	-0.0173	-13	133	142	YLAEVASGEK	55	99.992	(N-term)_iTRAQ[0], Lysine(K)_iTRAQ[10]	[7] F5 120912	206/198	1.095	1.003	1.316	1	Mascot
1555.8213	1555.7111	-0.1102	-71	144	155	NSVVEASEAAYK	73	100	(N-term)_iTRAQ[0], Lysine(K)_iTRAQ[12]	[5] F4	123/115	0.784	1.040	0.948	1	Mascot
1581.6904	1581.6193	-0.0711	-45	111	120	NCNDFQYESK	53	99.987	(N-term)_iTRAQ[0], Lysine(K)_iTRAQ[10], MMTS (C)[2]	[7] F5 120912	167/159	0.494	0.793	0.764	1	Mascot

1581.6904	1581.6396	-0.0508	-32	111	120	NCNDFQYESK	62	99.998	(N-term)_iTRAQ[0], Lysine(K)_iTRAQ[10], MMTS (C)[2]	[3] F6 and F9	205/197	0.712	1.341	1.050	1	Mascot
1730.868	1730.7759	-0.0921	-53	29	42	AVTELNEPLSNEDR	57	99.995	(N-term)_iTRAQ[0]	[1] F3 030912	212/204	0.622	0.861	0.747	1	Mascot

134	heterogeneous nuclear ribonucleoprotein A3 isoform c [Mus musculus]	gi|157277969	40273.3	9	434	0.862	1.094	0.949	0.239	0.289	0.206	10	10	10	100

Protein Group

heterogeneous nuclear ribonucleoprotein A3 isoform a [Mus musculus]	gi|31559916	42981.8

Peptide Information

Calc. Mass	Obsrv. Mass	± da	± ppm	Start Seq.	End Seq.	Sequence	Ion Score	C. I. %	Modification	Plate [#] Name	Gel Idx/Pos [4700 Sample Name]	iTRAQ Ratio 115/114*	iTRAQ Ratio 116/114*	iTRAQ Ratio 117/114*	Rank	Result Type
1021.6655	1021.6119	-0.0536	-52	106	112	IFVGGIK	33	98.734	(N-term)_iTRAQ[0], Lysine(K)_iTRAQ[7]	[7] F5 120912	218/210	1.244	1.380	0.951	1	Mascot
1021.6655	1021.6606	-0.0049	-5	106	112	IFVGGIK	36	99.385	(N-term)_iTRAQ[0], Lysine(K)_iTRAQ[7]	[3] F6 and F9	262/254	1.231	1.890	1.123	1	Mascot
1293.8629	1293.9136	0.0507	39	105	112	KIFVGGIK	39	99.697	(N-term)_iTRAQ[0], Lysine(K)_iTRAQ[1,8]	[4] F7 and F10+11	1131/1123	0.547	0.843	0.855	1	Mascot
1378.7008	1378.6565	-0.0443	-32	130	139	IETIEVMEDR	34	99.087	(N-term)_iTRAQ[0]	[1] F3 030912	269/261	1.038	1.086	1.380	1	Mascot
1470.7008	1470.642	-0.0588	-40	36	46	WGTLTDCVVMR	40	99.727	(N-term)_iTRAQ[0], MMTS (C)[7]	[1] F3 030912	423/415	0.768	1.064	0.716	1	Mascot
1813.0555	1813.136	0.0805	44	92	104	EDSVKPGAHLTVK	59	99.997	(N-term)_iTRAQ[0], Lysine(K)_iTRAQ[5,13]	[4] F7 and F10+11	1058/1050	0.976	0.944	1.094	1	Mascot
1914.9933	1915.0194	0.0261	14	15	30	LFIGGLSFETTDDSLR	28	96.149	(N-term)_iTRAQ[0]	[8] F13-15 and F1+2	381/373	0.718	1.270	0.895	1	Mascot
2001.9803	2001.9365	-0.0438	-22	146	160	GFAFVTFDDHDTVDK	71	100	(N-term)_iTRAQ[0], Lysine(K)_iTRAQ[15]	[4] F7 and F10+11	347/339	0.842	0.891	0.718	1	Mascot
2054.8923	2054.8132	-0.0791	-38	334	355	SSGSPYGGGYGSGGGSGGYGSR	45	99.925	(N-term)_iTRAQ[0]	[4] F7 and F10+11	130/122	0.749	0.991	0.868	1	Mascot
2171.1594	2171.1177	-0.0417	-19	106	121	IFVGGIKEDTEEYNLR	82	100	(N-term)_iTRAQ[0], Lysine(K)_iTRAQ[7]	[4] F7 and F10+11	334/326	0.768	0.911	1.079	1	Mascot

135	voltage dependent anion channel [Rattus norvegicus]	gi|4105605	36396.1	7	434	0.921	0.903	1.273	0.265	0.270	0.422	10	10	10	100

Protein Group

Vdac1 protein [Rattus norvegicus]	gi|38051979	35784.8
voltage-dependent anion-selective channel protein 1 [Mus musculus]	gi|6755963	34575.2

Peptide Information

Calc. Mass	Obsrv. Mass	± da	± ppm	Start Seq.	End Seq.	Sequence	Ion Score	C. I. %	Modification	Plate [#] Name	Gel Idx/Pos [4700 Sample Name]	iTRAQ Ratio 115/114*	iTRAQ Ratio 116/114*	iTRAQ Ratio 117/114*	Rank	Result Type
1142.6819	1142.6461	-0.0358	-31	21	28	GYGFGLIK	27	95.423	(N-term)_iTRAQ[0], Lysine(K)_iTRAQ[8]	[3] F6 and F9	311/303	1.755	1.680	2.472	1	Mascot
1142.6819	1142.6614	-0.0205	-18	21	28	GYGFGLIK	55	99.992	(N-term)_iTRAQ[0], Lysine(K)_iTRAQ[8]	[7] F5 120912	263/255	0.818	0.932	0.932	1	Mascot
1501.826	1501.7594	-0.0666	-44	182	192	VTQSNFAVGYK	64	99.999	(N-term)_iTRAQ[0], Lysine(K)_iTRAQ[11]	[7] F5 120912	179/171	0.958	0.777	1.300	1	Mascot
1662.8625	1662.8094	-0.0531	-32	64	74	WTEYGLTFTEK	81	100	(N-term)_iTRAQ[0], Lysine(K)_iTRAQ[11]	[5] F4	263/255	0.736	0.651	1.008	1	Mascot
1662.8625	1662.827	-0.0355	-21	64	74	WTEYGLTFTEK	59	99.997	(N-term)_iTRAQ[0], Lysine(K)_iTRAQ[11]	[7] F5 120912	330/322	0.895	1.090	1.302	1	Mascot
1677.7843	1677.6975	-0.0868	-52	243	254	YQVDPDACFSAK	73	100	(N-term)_iTRAQ[0], Lysine(K)_iTRAQ[12], MMTS (C)[8]	[5] F4	230/222	0.881	1.058	1.042	1	Mascot
1688.8741	1688.7772	-0.0969	-57	97	109	LTFDSSFSPNTGK	43	99.878	(N-term)_iTRAQ[0], Lysine(K)_iTRAQ[13]	[5] F4	194/186	0.667	0.688	0.910	1	Mascot
1688.8741	1688.822	-0.0521	-31	97	109	LTFDSSFSPNTGK	52	99.984	(N-term)_iTRAQ[0], Lysine(K)_iTRAQ[13]	[7] F5 120912	243/235	1.125	0.903	1.603	1	Mascot
1961.0715	1961.1205	0.049	25	97	110	LTFDSSFSPNTGKK	58	99.996	(N-term)_iTRAQ[0], Lysine(K)_iTRAQ[13, 14]	[4] F7 and F10+11	1151/1143	0.853	0.827	1.471	1	Mascot
2234.2139	2234.1694	-0.0445	-20	219	236	KLETAVNLAWTAGNSNTR	52	99.983	(N-term)_iTRAQ[0], Lysine(K)_iTRAQ[1]	[4] F7 and F10+11	334/326	0.861	0.768	1.274	1	Mascot

136	G protein beta 1 subunit [Rattus norvegicus]	gi|984553	39597.9	6	433	1.266	0.951	1.222	0.357	0.353	0.164	7	7	7	100

Protein Group

guanine nucleotide-binding protein G(I)/G(S)/G(T) subunit beta-1 [Mus musculus]	gi|6680045	39581.9

Peptide Information

Calc. Mass	Obsrv. Mass	± da	± ppm	Start	End	Sequence	Ion	C. I. %	Modification	Plate [#] Name	Gel Idx/Pos [4700	iTRAQ	iTRAQ	iTRAQ	Rank	Result Type

Calc. Mass	Obsrv. Mass	± da	± ppm	Start Seq.	End Seq.	Sequence	Ion Score	C. I. %	Modification	Plate [#] Name	Gel Idx/Pos [4700 Sample Name]	iTRAQ Ratio 115/114*	iTRAQ Ratio 116/114*	iTRAQ Ratio 117/114*	Rank	Result Type
1305.7625	1305.7292	-0.0333	-26	69	78	LLVSASQDGK	79	100	(N-term)_iTRAQ[0], Lysine(K)_iTRAQ[10]	[7] F5 120912	155/147	0.908	0.682	1.078	1	Mascot
1480.7128	1480.8044	0.0916	62	58	68	IYAMHWGTDSR	55	99.991	(N-term)_iTRAQ[0]	[3] F6 and F9	1225/1217	1.100	0.899	1.232	1	Mascot
1641.9098	1641.8633	-0.0465	-28	79	89	LIIWDSYTTNK	47	99.948	(N-term)_iTRAQ[0], Lysine(K)_iTRAQ[11]	[5] F4	266/258	1.600	1.097	1.259	1	Mascot
1641.9098	1641.8676	-0.0422	-26	79	89	LIIWDSYTTNK	58	99.996	(N-term)_iTRAQ[0], Lysine(K)_iTRAQ[11]	[7] F5 120912	333/325	1.089	1.018	1.411	1	Mascot
1645.7041	1645.6837	-0.0204	-12	138	150	ELAGHTGYLSCCR	61	99.998	(N-term)_iTRAQ[0], MMTS (C)[11,12]	[4] F7 and F10+11	334/326	2.006	1.662	1.487	1	Mascot
2404.1907	2404.1719	-0.0188	-8	284	301	LLLAGYDDFNCNVWDALK	45	99.917	(N-term)_iTRAQ[0], Lysine(K)_iTRAQ[18], MMTS (C)[11]	[1] F3 030912	548/540	1.138	0.585	1.121	1	Mascot
2693.2852	2693.2241	-0.0611	-23	315	337	VSCLGVTDDGMAVATGSWDSFLK	137	100	(N-term)_iTRAQ[0], Lysine(K)_iTRAQ[23], MMTS (C)[3]	[1] F3 030912	529/521	1.309	1.058	1.033	1	Mascot

137	brain-specific angiogenesis inhibitor 1-associated protein 2 [Rattus norvegicus]	gi|17105348	63886.3	6	427	0.877	0.846	0.842	0.258	0.197	0.288	6	6	6	100

Protein Group

RecName: Full=Brain-specific angiogenesis inhibitor 1-associated protein 2; Short=BAI-associated pr	gi|73917638	65670.3

Peptide Information

Calc. Mass	Obsrv. Mass	± da	± ppm	Start Seq.	End Seq.	Sequence	Ion Score	C. I. %	Modification	Plate [#] Name	Gel Idx/Pos [4700 Sample Name]	iTRAQ Ratio 115/114*	iTRAQ Ratio 116/114*	iTRAQ Ratio 117/114*	Rank	Result Type
1442.7889	1442.7186	-0.0703	-49	478	488	AFPSQTAGTFK	85	100	(N-term)_iTRAQ[0], Lysine(K)_iTRAQ[11]	[7] F5 120912	174/166	0.861	0.703	0.905	1	Mascot
1479.775	1479.7233	-0.0517	-35	19	29	TIMEQFNPSLR	41	99.802	(N-term)_iTRAQ[0]	[1] F3 030912	303/295	0.665	0.904	0.819	1	Mascot
1569.8972	1569.9136	0.0164	10	399	411	EGDLITLLVPEAR	56	99.994	(N-term)_iTRAQ[0]	[8] F13-15 and F1+2	368/360	0.758	0.589	0.549	1	Mascot
1787.9235	1787.9658	0.0423	24	71	84	ELGDVLFQMAEVHR	94	100	(N-term)_iTRAQ[0]	[3] F6 and F9	441/433	0.699	0.961	0.647	1	Mascot
1859.0272	1859.09	0.0628	34	96	108	AFHNELLTQLEQK	65	99.999	(N-term)_iTRAQ[0], Lysine(K)_iTRAQ[13]	[3] F6 and F9	1289/1281	1.087	1.104	1.368	1	Mascot
2067.1121	2067.1157	0.0036	2	382	398	AIFSHAAGDNSTLLSFK	87	100	(N-term)_iTRAQ[0], Lysine(K)_iTRAQ[17]	[4] F7 and F10+11	358/350	1.376	0.922	0.988	1	Mascot

138	phosphoglycerate mutase 1 [Mus musculus]	gi|114326546	31643.8	8	425	1.018	0.846	1.033	0.317	0.392	0.357	9	9	9	100

Peptide Information

Calc. Mass	Obsrv. Mass	± da	± ppm	Start Seq.	End Seq.	Sequence	Ion Score	C. I. %	Modification	Plate [#] Name	Gel Idx/Pos [4700 Sample Name]	iTRAQ Ratio 115/114*	iTRAQ Ratio 116/114*	iTRAQ Ratio 117/114*	Rank	Result Type
1263.6976	1263.6578	-0.0398	-31	242	251	AMEAVAAQGK	33	98.643	(N-term)_iTRAQ[0], Lysine(K)_iTRAQ[10]	[7] F5 120912	117/109	1.327	0.369	1.056	1	Mascot
1294.7715	1294.6973	-0.0742	-57	181	191	VLIAAHGNSLR	34	99.124	(N-term)_iTRAQ[0]	[6] F8 110912	202/194	0.888	0.740	0.746	1	Mascot
1347.7631	1347.8318	0.0687	51	91	100	HYGGLTGLNK	55	99.993	(N-term)_iTRAQ[0], Lysine(K)_iTRAQ[10]	[4] F7 and F10+11	1083/1075	1.210	1.098	1.126	1	Mascot
1972.1154	1972.0232	-0.0922	-47	163	176	ALPFWNEEIVPQIK	53	99.989	(N-term)_iTRAQ[0], Lysine(K)_iTRAQ[14]	[5] F4	354/346	1.109	0.916	1.180	1	Mascot
1972.1154	1972.0673	-0.0481	-24	163	176	ALPFWNEEIVPQIK	77	100	(N-term)_iTRAQ[0], Lysine(K)_iTRAQ[14]	[1] F3 030912	466/458	0.736	0.694	0.717	1	Mascot
2268.0818	2268.071	-0.0108	-5	22	39	FSGWYDADLSPAGHEEAK	28	95.729	(N-term)_iTRAQ[0], Lysine(K)_iTRAQ[18]	[4] F7 and F10+11	311/303	1.049	0.911	1.051	1	Mascot
2424.1829	2424.2698	0.0869	36	22	40	FSGWYDADLSPAGHEEAKR	52	99.985	(N-term)_iTRAQ[0], Lysine(K)_iTRAQ[18]	[4] F7 and F10+11	1186/1178	0.634	0.754	0.838	1	Mascot
2547.4341	2547.4939	0.0598	23	223	240	NLKPIKPMQFLGDEETVR	90	100	(N-term)_iTRAQ[0], Lysine(K)_iTRAQ[3,6]	[4] F7 and F10+11	1224/1216	0.951	1.061	0.976	1	Mascot
2819.6313	2819.5872	-0.0441	-16	223	241	NLKPIKPMQFLGDEETVRK	57	99.995	(N-term)_iTRAQ[0], Lysine(K)_iTRAQ[3,6,19]	[2] F12 040912	338/330	1.593	1.610	2.070	1	Mascot

139	calcium/calmodulin-dependent 3',5'-cyclic nucleotide phosphodiesterase 1B [Rattus norvegicus]	gi|12083681	66101.8	9	424	0.813	0.807	0.747	0.175	0.364	0.264	9	9	9	100

Peptide Information

Calc. Mass	Obsrv. Mass	± da	± ppm	Start Seq.	End Seq.	Sequence	Ion Score	C. I. %	Modification	Plate [#] Name	Gel Idx/Pos [4700 Sample Name]	iTRAQ Ratio 115/114*	iTRAQ Ratio 116/114*	iTRAQ Ratio 117/114*	Rank	Result Type
1179.6031	1179.5601	-0.043	-36	455	462	SQPSFQWR	38	99.645	(N-term)_iTRAQ[0]	[7] F5 120912	186/178	0.719	0.588	0.805	1	Mascot
1186.6051	1186.5731	-0.032	-27	386	393	ALMEEFFR	35	99.285	(N-term)_iTRAQ[0]	[5] F4	311/303	0.579	0.684	0.651	1	Mascot
1235.7484	1235.7186	-0.0298	-24	181	189	TIVFELLTR	35	99.225	(N-term)_iTRAQ[0]	[1] F3 030912	468/460	0.644	0.336	0.734	1	Mascot
1373.6294	1373.5819	-0.0475	-35	279	288	SECAILYNDR	29	96.782	(N-term)_iTRAQ[0], MMTS (C)[3]	[5] F4	212/204	1.036	1.074	0.723	1	Mascot

Calc. Mass	Obsrv. Mass	± da	± ppm	Start Seq.	End Seq.	Sequence	Ion Score	C. I. %	Modification	Plate [#] Name	Gel Idx/Pos [4700 Sample Name]	iTRAQ Ratio 115/114*	iTRAQ Ratio 116/114*	iTRAQ Ratio 117/114*	Rank	Result Type
1375.719	1375.6598	-0.0592	-43	76	85	QILDTEDELR	56	99.994	(N-term)_iTRAQ[0]	[1] F3 030912	224/216	1.098	0.940	1.149	1	Mascot
1597.8094	1597.7589	-0.0505	-32	98	109	DWLASTFTQQTR	58	99.996	(N-term)_iTRAQ[0]	[1] F3 030912	325/317	0.856	0.911	0.738	1	Mascot
1668.8942	1668.8828	-0.0114	-7	289	301	SVLENHHISSVFR	51	99.981	(N-term)_iTRAQ[0]	[2] F12 040912	279/271	0.834	0.943	0.371	1	Mascot
1975.1586	1975.2349	0.0763	39	360	375	ALSLLLHAADISHPTK	94	100	(N-term)_iTRAQ[0], Lysine(K)_iTRAQ[16]	[4] F7 and F10+11	1291/1283	0.857	1.132	1.051	1	Mascot
2309.1497	2309.1892	0.0395	17	394	411	QGDKEAELGLPFSPLCD R	27	95.273	(N-term)_iTRAQ[0], Lysine(K)_iTRAQ[4], MMTS (C)[16]	[4] F7 and F10+11	415/407	0.834	1.092	0.792	1	Mascot

140	neuron-specific calcium-binding protein hippocalcin [Mus musculus]	gi|6754240	24666.5	8	419	0.887	0.744	0.760	0.270	0.184	0.312	8	8	8	100

Peptide Information

Calc. Mass	Obsrv. Mass	± da	± ppm	Start Seq.	End Seq.	Sequence	Ion Score	C. I. %	Modification	Plate [#] Name	Gel Idx/Pos [4700 Sample Name]	iTRAQ Ratio 115/114*	iTRAQ Ratio 116/114*	iTRAQ Ratio 117/114*	Rank	Result Type
1049.5652	1049.6108	0.0456	43	64	70	FAEHVFR	28	95.816	(N-term)_iTRAQ[0]	[4] F7 and F10+11	1111/1103	1.163	0.819	1.015	1	Mascot
1150.6592	1150.6042	-0.055	-48	164	171	LSLEEFIR	41	99.803	(N-term)_iTRAQ[0]	[5] F4	309/301	1.115	0.919	0.755	1	Mascot
1606.7992	1606.8385	0.0393	24	138	148	MPEDESTPEKR	31	98.24	(N-term)_iTRAQ[0], Lysine(K)_iTRAQ[10]	[3] F6 and F9	1075/1067	0.953	0.733	0.376	1	Mascot
1632.7626	1632.6801	-0.0825	-51	71	83	TFDTNSDGTIDFR	89	100	(N-term)_iTRAQ[0]	[1] F3 030912	244/236	1.018	0.779	0.986	1	Mascot
1753.9656	1753.9149	-0.0507	-29	119	130	EEMLEIVQAIYK	57	99.995	(N-term)_iTRAQ[0], Lysine(K)_iTRAQ[12]	[5] F4	370/362	1.164	1.098	0.917	1	Mascot
1780.9155	1780.8464	-0.0691	-39	51	63	IYANFFPYGDASK	85	100	(N-term)_iTRAQ[0], Lysine(K)_iTRAQ[13]	[5] F4	273/265	0.683	0.625	1.060	1	Mascot
2057.0764	2057.0791	0.0027	1	37	50	DCPTGILNVDEFKK	54	99.99	(N-term)_iTRAQ[0], Lysine(K)_iTRAQ[13, 14], MMTS (C)[2]	[4] F7 and F10+11	360/352	0.570	0.566	0.517	1	Mascot
2233.0911	2232.9714	-0.1197	-54	18	32	ENTEFSELELQEWYK	34	98.917	(N-term)_iTRAQ[0], Lysine(K)_iTRAQ[15]	[5] F4	301/293	0.674	0.564	0.785	1	Mascot

141	cAMP and cAMP-inhibited cGMP 3',5'-cyclic phosphodiesterase 10A [Rattus norvegicus]	gi|13489075	97835.6	8	418	1.191	0.707	0.745	0.175	0.302	0.242	9	9	9	100

Peptide Information

Calc. Mass	Obsrv. Mass	± da	± ppm	Start Seq.	End Seq.	Sequence	Ion Score	C. I. %	Modification	Plate [#] Name	Gel Idx/Pos [4700 Sample Name]	iTRAQ Ratio 115/114*	iTRAQ Ratio 116/114*	iTRAQ Ratio 117/114*	Rank	Result Type
1331.7206	1331.6669	-0.0537	-40	569	577	GFSNSYLQK	66	99.999	(N-term)_iTRAQ[0], Lysine(K)_iTRAQ[9]	[3] F6 and F9	212/204	1.224	0.792	0.916	1	Mascot
1334.6951	1334.6415	-0.0536	-40	757	764	DNLNQWEK	46	99.934	(N-term)_iTRAQ[0], Lysine(K)_iTRAQ[8]	[3] F6 and F9	198/190	1.245	0.756	0.909	1	Mascot
1518.8923	1518.8224	-0.0699	-46	386	397	GSVIGVVQMVNK	52	99.984	(N-term)_iTRAQ[0], Lysine(K)_iTRAQ[12]	[5] F4	219/211	1.145	1.012	0.974	1	Mascot
1774.9095	1774.9254	0.0159	9	347	361	TGEVLNIPDAYADPR	37	99.52	(N-term)_iTRAQ[0]	[8] F13-15 and F1+2	479/471	1.267	1.218	0.506	1	Mascot
1774.9095	1774.944	0.0345	19	347	361	TGEVLNIPDAYADPR	34	99.024	(N-term)_iTRAQ[0]	[8] F13-15 and F1+2	482/474	1.115	0.769	0.701	1	Mascot
1845.9368	1845.8741	-0.0627	-34	308	320	ELYSDLFDIGEEK	69	100	(N-term)_iTRAQ[0], Lysine(K)_iTRAQ[13]	[5] F4	315/307	1.188	0.734	0.842	1	Mascot
1852.936	1852.8146	-0.1214	-66	768	782	GEETAMWISGPATSK	74	100	(N-term)_iTRAQ[0], Lysine(K)_iTRAQ[15]	[5] F4	185/177	1.061	0.525	0.815	1	Mascot
1916.9576	1916.9023	-0.0553	-29	15	28	LTECFLSPSLTDEK	41	99.784	(N-term)_iTRAQ[0], Lysine(K)_iTRAQ[14], MMTS (C)[4]	[1] F3 030912	403/395	0.957	0.331	0.410	1	Mascot
2232.177	2232.0796	-0.0974	-44	306	320	NKELYSDLFDIGEEK	36	99.428	(N-term)_iTRAQ[0], Lysine(K)_iTRAQ[2,1 5]	[6] F8 110912	378/370	1.622	0.610	0.868	1	Mascot

142	calcium-dependent secretion activator 1 [Rattus norvegicus]	gi|6978599	159485.8	9	418	0.944	0.943	1.045	0.229	0.282	0.155	9	9	9	100

Peptide Information

Calc. Mass	Obsrv. Mass	± da	± ppm	Start Seq.	End Seq.	Sequence	Ion Score	C. I. %	Modification	Plate [#] Name	Gel Idx/Pos [4700 Sample Name]	iTRAQ Ratio 115/114*	iTRAQ Ratio 116/114*	iTRAQ Ratio 117/114*	Rank	Result Type
1084.64	1084.598	-0.042	-39	589	595	AFFNAVK	38	99.625	(N-term)_iTRAQ[0], Lysine(K)_iTRAQ[7]	[3] F6 and F9	248/240	0.885	0.700	0.847	1	Mascot
1513.8611	1513.8632	0.0021	1	786	796	VLLENQITHFR	36	99.383	(N-term)_iTRAQ[0]	[4] F7 and F10+11	313/305	0.859	0.812	1.099	1	Mascot
1551.9131	1551.9342	0.0211	14	957	968	HLQDLFAPLVVR	45	99.915	(N-term)_iTRAQ[0]	[4] F7 and F10+11	425/417	0.838	0.962	1.110	1	Mascot
1641.9309	1641.8424	-0.0885	-54	822	833	DIVTPVPQEEVK	54	99.989	(N-term)_iTRAQ[0], Lysine(K)_iTRAQ[12]	[1] F3 030912	216/208	0.710	0.595	0.946	1	Mascot
1674.8373	1674.882	0.0447	27	1016	1026	DLHWPEEEFGK	46	99.938	(N-term)_iTRAQ[0], Lysine(K)_iTRAQ[11]	[3] F6 and F9	1255/1247	1.068	0.881	1.167	1	Mascot
1713.8727	1713.9396	0.0669	39	1182	1192	DKVNEEMYIER	39	99.688	(N-term)_iTRAQ[0], Lysine(K)_iTRAQ[2]	[3] F6 and F9	1162/1154	1.346	1.283	1.103	1	Mascot
1716.8434	1716.7609	-0.0825	-48	332	342	EMENMYIEELK	51	99.982	(N-term)_iTRAQ[0], Lysine(K)_iTRAQ[11]	[5] F4	238/230	1.094	0.982	1.122	1	Mascot
2014.0929	2014.0498	-0.0431	-21	269	282	EQLYEMFQNILGIK	54	99.99	(N-term)_iTRAQ[0], Lysine(K)_iTRAQ[14]	[5] F4	422/414	0.697	1.191	0.829	1	Mascot

2135.231	2135.3115	0.0805	38	620	635	ATGQSHKPVPPTQVQK	55	99.993	(N-term)_iTRAQ[0], Lysine(K)_iTRAQ[7,16]	[4] F7 and F10+11	1026/1018	1.206	1.369	1.270	1	Mascot	

143	syntaxin A [Rattus norvegicus]	gi|207127	36124.9	8	414	0.890	0.858	1.053	0.247	0.356	0.135	10	10	10	100		

Protein Group

HPC-1 antigen [Rattus norvegicus]	gi|220777	37564.6
syntaxin 1A [Rattus norvegicus]	gi|6665797	36774.1
syntaxin-1A [Rattus norvegicus]	gi|33667087	36643.1

Peptide Information

Calc. Mass	Obsrv. Mass	± da	± ppm	Start Seq.	End Seq.	Sequence	Ion Score	C. I. %	Modification	Plate [#] Name	Gel Idx/Pos [4700 Sample Name]	iTRAQ Ratio 115/114*	iTRAQ Ratio 116/114*	iTRAQ Ratio 117/114*	Rank	Result Type
1318.7463	1318.7161	-0.0302	-23	44	52	IAENVEEVK	52	99.983	(N-term)_iTRAQ[0], Lysine(K)_iTRAQ[9]	[7] F5 120912	155/147	0.773	0.741	0.892	1	Mascot
1474.8475	1474.9032	0.0557	38	44	53	IAENVEEVKR	33	98.662	(N-term)_iTRAQ[0], Lysine(K)_iTRAQ[9]	[4] F7 and F10+11	1068/1060	0.800	0.740	1.028	1	Mascot
1666.901	1666.8057	-0.0953	-57	55	67	HSAILASPNPDEK	77	100	(N-term)_iTRAQ[0], Lysine(K)_iTRAQ[13]	[6] F8 110912	164/156	0.735	0.912	1.087	1	Mascot
1775.8895	1775.8175	-0.072	-41	92	105	SIEQSIEQEEGLNR	70	100	(N-term)_iTRAQ[0]	[1] F3 030912	271/263	0.750	0.904	1.037	1	Mascot
1862.8755	1862.8386	-0.0369	-20	26	38	FMDEFFEQVEEIR	64	99.999	(N-term)_iTRAQ[0]	[1] F3 030912	529/521	0.962	1.025	1.142	1	Mascot
1862.8755	1862.8632	-0.0123	-7	26	38	FMDEFFEQVEEIR	37	99.526	(N-term)_iTRAQ[0]	[8] F13-15 and F1+2	266/258	0.825	0.884	1.326	1	Mascot
1879.931	1879.9606	0.0296	16	230	243	IEYNVEHAVDYVER	32	98.495	(N-term)_iTRAQ[0]	[3] F6 and F9	330/322	0.956	0.364	1.011	1	Mascot
1997.0848	1997.0837	-0.0011	-1	68	80	TKEELEELMSDIK	39	99.722	(N-term)_iTRAQ[0], Lysine(K)_iTRAQ[2,13]	[3] F6 and F9	1351/1343	0.959	1.238	1.227	1	Mascot
1997.0848	1997.1819	0.0971	49	68	80	TKEELEELMSDIK	37	99.52	(N-term)_iTRAQ[0], Lysine(K)_iTRAQ[2,13]	[4] F7 and F10+11	1291/1283	1.728	1.420	0.916	1	Mascot
2355.1536	2355.1409	-0.0127	-5	123	139	KFVEVMSEYNATQSDYR	47	99.955	(N-term)_iTRAQ[0], Lysine(K)_iTRAQ[1]	[4] F7 and F10+11	325/317	0.725	0.828	0.943	1	Mascot

144	pyruvate dehydrogenase E1 component subunit beta, mitochondrial precursor [Rattus norvegicus]	gi|56090293	42115	7	409	0.945	0.988	0.993	0.141	0.089	0.125	7	7	7	100

Peptide Information

Calc. Mass	Obsrv. Mass	± da	± ppm	Start Seq.	End Seq.	Sequence	Ion Score	C. I. %	Modification	Plate [#] Name	Gel Idx/Pos [4700 Sample Name]	iTRAQ Ratio 115/114*	iTRAQ Ratio 116/114*	iTRAQ Ratio 117/114*	Rank	Result Type
1190.7395	1190.6749	-0.0646	-54	220	227	DFLIPIGK	38	99.632	(N-term)_iTRAQ[0], Lysine(K)_iTRAQ[8]	[5] F4	250/242	0.735	0.965	1.128	1	Mascot
1519.8003	1519.7384	-0.0619	-41	174	184	VVSPWNSEDAK	71	100	(N-term)_iTRAQ[0], Lysine(K)_iTRAQ[11]	[7] F5 120912	177/169	1.015	0.996	1.043	1	Mascot
1536.8341	1536.7657	-0.0684	-45	325	336	VTGADVPMPYAK	75	100	(N-term)_iTRAQ[0], Lysine(K)_iTRAQ[12]	[5] F4	163/155	1.006	0.905	1.190	1	Mascot
1543.8942	1543.799	-0.0952	-62	337	347	ILEDNSIPQVK	55	99.993	(N-term)_iTRAQ[0], Lysine(K)_iTRAQ[11]	[5] F4	180/172	1.051	0.994	0.880	1	Mascot
1891.986	1892.0032	0.0172	9	309	324	IMEGPAFNFLDAPAVR	30	97.764	(N-term)_iTRAQ[0]	[8] F13-15 and F1+2	371/363	1.141	1.199	0.927	1	Mascot
2090.1055	2090.0249	-0.0806	-39	53	68	VFLLGEEVAQYDGAYK	79	100	(N-term)_iTRAQ[0], Lysine(K)_iTRAQ[16]	[1] F3 030912	427/419	0.854	0.968	0.840	1	Mascot
2194.0542	2194.0579	0.0037	2	37	52	EAINQGMDEELERDEK	60	99.997	(N-term)_iTRAQ[0], Lysine(K)_iTRAQ[16]	[4] F7 and F10+11	220/212	0.877	0.916	0.993	1	Mascot

145	Chain A, New Crystal Forms Of A Mu Class Glutathione S-Transferase From Rat Liver	gi|442967	28786.1	9	409	0.940	1.184	1.253	0.281	0.273	0.222	9	9	9	100

Protein Group

Chain A, First-Sphere And Second-Sphere Electrostatic Effects In The Active Site Of A Class Mu Glut	gi|1943435	28784.1
Chain A, First-Sphere And Second-Sphere Electrostatic Effects In The Active Site Of A Class Mu Glut	gi|1943433	28772.1
Chain A, First-Sphere And Second-Sphere Electrostatic Effects In The Active Site Of A Class Mu Glut	gi|1943431	28756.1
Chain A, Glutathione Transferase Mutant Y115f	gi|29726512	28770.1
glutathione S-transferase Mu 1 [Rattus norvegicus]	gi|8393502	28917.1

glutathione S-transferase Yb-1 subunit (EC 2.5.1.18) [Rattus norvegicus] | gi|204503 | 28820.9

Peptide Information

Calc. Mass	Obsrv. Mass	± da	± ppm	Start Seq.	End Seq.	Sequence	Ion Score	C. I. %	Modification	Plate [#] Name	Gel Idx/Pos [4700 Sample Name]	iTRAQ Ratio 115/114*	iTRAQ Ratio 116/114*	iTRAQ Ratio 117/114*	Rank	Result Type
1134.6517	1134.6339	-0.0178	-16	211	217	LAQWSNK	35	99.251	(N-term)_iTRAQ[0], Lysine(K)_iTRAQ[7]	[4] F7 and F10+11	191/183	0.861	1.160	1.403	1	Mascot
1192.6572	1192.6332	-0.024	-20	43	49	SQWLNEK	39	99.7	(N-term)_iTRAQ[0], Lysine(K)_iTRAQ[7]	[4] F7 and F10+11	191/183	0.761	1.000	1.259	1	Mascot
1244.7136	1244.6593	-0.0543	-44	136	143	LYSEFLGK	30	97.581	(N-term)_iTRAQ[0], Lysine(K)_iTRAQ[8]	[7] F5 120912	287/279	0.779	0.885	1.093	1	Mascot
1264.7048	1264.6775	-0.0273	-22	144	151	RPWFAGDK	36	99.392	(N-term)_iTRAQ[0], Lysine(K)_iTRAQ[8]	[2] F12 040912	229/221	1.212	1.612	1.847	1	Mascot
1392.7582	1392.73	-0.0282	-20	1	10	PMILGYWNVR	35	99.167	(N-term)_iTRAQ[0]	[3] F6 and F9	398/390	0.628	1.003	1.007	1	Mascot
1547.7607	1547.6836	-0.0771	-50	96	107	ADIVENQVMDNR	56	99.994	(N-term)_iTRAQ[0]	[1] F3 030912	211/203	1.007	1.252	1.146	1	Mascot
1877.963	1877.8645	-0.0985	-52	18	30	LLLEYTDSSYEEK	72	100	(N-term)_iTRAQ[0], Lysine(K)_iTRAQ[13]	[5] F4	238/230	1.154	1.487	1.246	1	Mascot
1934.0508	1934.0342	-0.0166	-9	52	67	LGLDFPNLPYLIDGSR	38	99.6	(N-term)_iTRAQ[0]	[8] F13-15 and F1+2	282/274	1.532	1.515	1.227	1	Mascot
2034.0641	2034.0514	-0.0127	-6	18	31	LLLEYTDSSYEEKR	69	100	(N-term)_iTRAQ[0], Lysine(K)_iTRAQ[13]	[4] F7 and F10+11	304/296	0.826	0.979	1.212	1	Mascot

146	NADH-ubiquinone oxidoreductase 75 kDa subunit, mitochondrial precursor [Rattus norvegicus]	gi|53850628	86195.6	9	409	0.856	0.952	0.999	0.225	0.188	0.255	10	10	10	100

Peptide Information

Calc. Mass	Obsrv. Mass	± da	± ppm	Start Seq.	End Seq.	Sequence	Ion Score	C. I. %	Modification	Plate [#] Name	Gel Idx/Pos [4700 Sample Name]	iTRAQ Ratio 115/114*	iTRAQ Ratio 116/114*	iTRAQ Ratio 117/114*	Rank	Result Type
1124.6912	1124.6254	-0.0658	-59	608	617	VAVTPPGLAR	40	99.756	(N-term)_iTRAQ[0]	[1] F3 030912	209/201	0.628	0.782	0.739	1	Mascot
1208.6548	1208.5956	-0.0592	-49	409	417	FEAPLFNAR	31	97.854	(N-term)_iTRAQ[0]	[5] F4	225/217	0.722	0.757	0.842	1	Mascot
1277.71	1277.6566	-0.0534	-42	502	511	VASGAAAEWK	74	100	(N-term)_iTRAQ[0], Lysine(K)_iTRAQ[10]	[3] F6 and F9	195/187	0.906	1.033	1.120	1	Mascot
1449.7948	1449.7657	-0.0291	-20	99	108	GWNILTNSEK	72	100	(N-term)_iTRAQ[0], Lysine(K)_iTRAQ[10]	[3] F6 and F9	289/281	0.912	1.232	1.220	1	Mascot
1547.8917	1547.8828	-0.0089	-6	429	441	VALIGSPVDLTYR	28	95.949	(N-term)_iTRAQ[0]	[8] F13-15 and F1+2	476/468	1.277	0.856	0.679	1	Mascot
1642.8356	1642.7454	-0.0902	-55	201	212	GNDMQVGTYIEK	42	99.837	(N-term)_iTRAQ[0], Lysine(K)_iTRAQ[12]	[5] F4	150/142	1.145	0.848	0.951	1	Mascot
1673.9982	1673.9069	-0.0913	-55	471	483	KPMVVLGSSALQR	35	99.268	(N-term)_iTRAQ[0], Lysine(K)_iTRAQ[1]	[6] F8 110912	259/251	0.931	0.985	1.073	1	Mascot
1933.9211	1933.9926	0.0715	37	277	289	MHEDINEEWISDK	39	99.713	(N-term)_iTRAQ[0], Lysine(K)_iTRAQ[13]	[3] F6 and F9	1220/1212	0.577	0.882	0.992	1	Mascot
1949.916	1949.9415	0.0255	13	277	289	MHEDINEEWISDK	35	99.298	(N-term)_iTRAQ[0], Lysine(K)_iTRAQ[13], Oxidation (M)[1]	[3] F6 and F9	1198/1190	0.879	1.360	1.523	1	Mascot
2219.2043	2219.2229	0.0186	8	625	643	ALSEIAGITLPYDTLDQVR	50	99.974	(N-term)_iTRAQ[0]	[8] F13-15 and F1+2	337/329	0.814	0.943	1.106	1	Mascot

147	peptidyl-prolyl cis-trans isomerase A [Rattus norvegicus]	gi|8394009	20208.3	9	409	1.041	0.853	1.034	0.462	0.318	0.382	10	10	10	100

Peptide Information

Calc. Mass	Obsrv. Mass	± da	± ppm	Start Seq.	End Seq.	Sequence	Ion Score	C. I. %	Modification	Plate [#] Name	Gel Idx/Pos [4700 Sample Name]	iTRAQ Ratio 115/114*	iTRAQ Ratio 116/114*	iTRAQ Ratio 117/114*	Rank	Result Type
1136.6198	1136.5684	-0.0514	-45	119	125	TEWLDGK	47	99.951	(N-term)_iTRAQ[0], Lysine(K)_iTRAQ[7]	[3] F6 and F9	216/208	1.072	1.019	1.105	1	Mascot
1395.6732	1395.6257	-0.0475	-34	134	144	EGMSIVEAMER	39	99.698	(N-term)_iTRAQ[0]	[1] F3 030912	318/310	0.718	1.066	0.786	1	Mascot
1405.7086	1405.7041	-0.0045	-3	20	28	VCFELFADK	46	99.936	(N-term)_iTRAQ[0], Lysine(K)_iTRAQ[9], MMTS (C)[2]	[7] F5 120912	426/418	0.760	0.678	1.005	1	Mascot
1411.7516	1411.7322	-0.0194	-14	155	164	KITISDCGQL	50	99.976	(N-term)_iTRAQ[0], Lysine(K)_iTRAQ[1], MMTS (C)[7]	[7] F5 120912	307/299	0.871	0.958	0.881	1	Mascot
1442.7777	1442.7284	-0.0493	-34	83	91	FEDENFILK	40	99.748	(N-term)_iTRAQ[0], Lysine(K)_iTRAQ[9]	[7] F5 120912	291/283	1.165	1.071	1.129	1	Mascot
1731.8121	1731.7415	-0.0706	-41	56	69	IIPGFMCQGGDFTR	38	99.61	(N-term)_iTRAQ[0], MMTS (C)[7]	[1] F3 030912	442/434	2.679	1.444	2.281	1	Mascot
1874.0272	1873.9437	-0.0835	-45	20	31	VCFELFADKVPK	35	99.239	(N-term)_iTRAQ[0], Lysine(K)_iTRAQ[9,12], MMTS (C)[2]	[6] F8 110912	447/439	1.209	0.515	1.229	1	Mascot
2092.0835	2092.0923	0.0088	4	2	19	VNPTVFFDITADGEPLGR	38	99.638	(N-term)_iTRAQ[0]	[8] F13-15 and F1+2	356/348	1.159	0.932	0.852	1	Mascot
2264.2185	2264.1487	-0.0698	-31	77	91	SIYGEKFEDENFILK	76	100	(N-term)_iTRAQ[0], Lysine(K)_iTRAQ[6,15]	[6] F8 110912	366/358	0.914	0.757	0.997	1	Mascot
2264.2185	2264.218	-0.0005	0	77	91	SIYGEKFEDENFILK	51	99.98	(N-term)_iTRAQ[0],	[4] F7 and F10+11	364/356	0.735	0.517	0.676	1	Mascot

Lysine(K)_iTRAQ[6,1 5]

148	calmodulin dependent protein kinase II	gi|225775	53911	9	408	0.954	1.101	0.983	0.363	0.293	0.241	9	9	9	100

Peptide Information

Calc. Mass	Obsrv. Mass	± da	± ppm	Start Seq.	End Seq.	Sequence	Ion Score	C. I. %	Modification	Plate [#] Name	Gel Idx/Pos [4700 Sample Name]	iTRAQ Ratio 115/114*	iTRAQ Ratio 116/114*	iTRAQ Ratio 117/114*	Rank	Result Type
1080.6664	1080.6154	-0.051	-47	210	215	LYQQIK	33	98.745	(N-term)_iTRAQ[0], Lysine(K)_iTRAQ[6]	[3] F6 and F9	190/182	1.043	1.183	1.117	1	Mascot
1104.6875	1104.6415	-0.046	-42	249	256	ITAAEALK	36	99.397	(N-term)_iTRAQ[0], Lysine(K)_iTRAQ[8]	[7] F5 120912	170/162	0.540	0.854	0.725	1	Mascot
1266.698	1266.7488	0.0508	40	422	429	WQXVHFHR	40	99.779	(N-term)_iTRAQ[0]	[8] F13-15 and F1+2	1151/1143	0.545	0.636	0.672	1	Mascot
1269.641	1269.5786	-0.0624	-49	273	280	QETVDCLK	49	99.972	(N-term)_iTRAQ[0], Lysine(K)_iTRAQ[8], MMTS (C)[6]	[7] F5 120912	174/166	1.121	1.135	1.229	1	Mascot
1291.7528	1291.7052	-0.0476	-37	290	300	GAILTTMLATR	39	99.692	(N-term)_iTRAQ[0]	[1] F3 030912	340/332	1.080	1.221	0.835	1	Mascot
1337.7675	1337.7218	-0.0457	-34	29	38	VXAGQEYAAK	63	99.999	(N-term)_iTRAQ[0], Lysine(K)_iTRAQ[10]	[7] F5 120912	149/141	0.893	1.141	1.183	1	Mascot
1541.8384	1541.9143	0.0759	49	273	281	QETVDCLKK	33	98.745	(N-term)_iTRAQ[0], Lysine(K)_iTRAQ[8,9] , MMTS (C)[6]	[4] F7 and F10+11	1090/1082	1.407	1.208	1.100	1	Mascot
1773.0857	1772.9882	-0.0975	-55	124	135	DLKPENLLLASK	49	99.967	(N-term)_iTRAQ[0], Lysine(K)_iTRAQ[3,1 2]	[6] F8 110912	295/287	1.190	1.424	1.239	1	Mascot
2369.1548	2369.0718	-0.083	-35	216	234	AGAYDFPSPEWDTVTPE AK	68	100	(N-term)_iTRAQ[0], Lysine(K)_iTRAQ[19]	[1] F3 030912	337/329	1.184	1.355	0.954	1	Mascot

149	serine/threonine-protein phosphatase PP1-beta catalytic subunit isoform 1 [Homo sapiens]	gi|4506005	40688.5	7	408	0.967	0.836	0.987	0.303	0.183	0.302	7	7	7	100

Peptide Information

Calc. Mass	Obsrv. Mass	± da	± ppm	Start Seq.	End Seq.	Sequence	Ion Score	C. I. %	Modification	Plate [#] Name	Gel Idx/Pos [4700 Sample Name]	iTRAQ Ratio 115/114*	iTRAQ Ratio 116/114*	iTRAQ Ratio 117/114*	Rank	Result Type
1342.7279	1342.6913	-0.0366	-27	113	121	YPENFFLLR	48	99.964	(N-term)_iTRAQ[0]	[1] F3 030912	408/400	0.861	1.060	0.844	1	Mascot
1728.0094	1727.9452	-0.0642	-37	111	121	IKYPENFFLLR	65	99.999	(N-term)_iTRAQ[0], Lysine(K)_iTRAQ[2]	[6] F8 110912	421/413	0.948	0.980	1.128	1	Mascot
1793.8779	1793.8839	0.006	3	60	73	ICGDIHGQYTDLLR	55	99.992	(N-term)_iTRAQ[0], MMTS (C)[2]	[4] F7 and F10+11	383/375	0.828	0.802	0.741	1	Mascot
1829.0143	1828.9751	-0.0392	-21	98	110	QSLETICLLLAYK	39	99.702	(N-term)_iTRAQ[0], Lysine(K)_iTRAQ[13], MMTS (C)[7]	[5] F4	419/411	1.086	1.055	0.889	1	Mascot
1927.98	1927.9174	-0.0626	-32	246	259	AHQVVEDGYEFFAK	89	100	(N-term)_iTRAQ[0], Lysine(K)_iTRAQ[14]	[6] F8 110912	362/354	0.722	0.750	0.900	1	Mascot
2241.3354	2241.2866	-0.0488	-22	43	59	EIFLSQPILLELEAPLK	73	100	(N-term)_iTRAQ[0], Lysine(K)_iTRAQ[17]	[1] F3 030912	543/535	0.846	0.615	0.909	1	Mascot
2380.127	2380.0686	-0.0584	-25	150	167	TFTDCFNCLPIAAIVDEK	38	99.636	(N-term)_iTRAQ[0], Lysine(K)_iTRAQ[18], MMTS (C)[5,8]	[1] F3 030912	574/566	1.768	0.702	1.780	1	Mascot

150	isocitrate dehydrogenase [NAD] subunit gamma 1, mitochondrial precursor [Rattus norvegicus]	gi|54020666	46315.4	7	407	0.792	1.110	0.992	0.322	0.520	0.294	7	7	7	100

Peptide Information

Calc. Mass	Obsrv. Mass	± da	± ppm	Start Seq.	End Seq.	Sequence	Ion Score	C. I. %	Modification	Plate [#] Name	Gel Idx/Pos [4700 Sample Name]	iTRAQ Ratio 115/114*	iTRAQ Ratio 116/114*	iTRAQ Ratio 117/114*	Rank	Result Type
1129.6503	1129.6068	-0.0435	-39	200	206	IAEYAFK	31	97.974	(N-term)_iTRAQ[0], Lysine(K)_iTRAQ[7]	[3] F6 and F9	252/244	0.730	1.494	0.746	1	Mascot
1460.6604	1460.6139	-0.0465	-32	227	237	LGDGLFLQCCR	38	99.627	(N-term)_iTRAQ[0], MMTS (C)[9,10]	[5] F4	395/387	1.324	1.409	0.960	1	Mascot
1509.9363	1509.8971	-0.0392	-26	158	167	HKDIDILIVR	39	99.711	(N-term)_iTRAQ[0], Lysine(K)_iTRAQ[2]	[2] F12 040912	296/288	0.656	0.991	0.861	1	Mascot
1810.9423	1810.9358	-0.0065	-4	137	149	TSLDLYANVIHCK	63	99.999	(N-term)_iTRAQ[0], Lysine(K)_iTRAQ[13], MMTS (C)[12]	[4] F7 and F10+11	385/377	1.236	1.951	1.688	1	Mascot
1845.9817	1846.0542	0.0725	39	116	129	GNIETNHNLPPSHK	72	100	(N-term)_iTRAQ[0], Lysine(K)_iTRAQ[14]	[4] F7 and F10+11	1016/1008	0.633	0.858	1.029	1	Mascot
2433.3245	2433.2219	-0.1026	-42	55	74	HTVTMIPGDGIGPELMLH VK	32	98.546	(N-term)_iTRAQ[0], Lysine(K)_iTRAQ[20]	[6] F8 110912	410/402	0.476	0.551	0.771	1	Mascot
3427.6707	3427.5413	-0.1294	-38	351	381	AVLASMDNENMHTPDIG GQGTTSQAIQDIIR	132	100	(N-term)_iTRAQ[0]	[1] F3 030912	394/386	0.827	1.080	1.146	1	Mascot

151	Chain A, Mu2 Adaptin Subunit (Ap50) Of Ap2 Adaptor (Second Domain), Complexed With Egfr Internaliza	gi|6729920	41607.5	8	407	0.880	1.030	1.048	0.712	0.345	0.820	8	8	8	100

Protein Group

AP-2 complex subunit mu [Mus musculus]	gi|6753074	55518.9

	Chain A, Mu2 Adaptin Subunit (Ap50) Of Ap2 Clathrin Adaptor, Complexed With Egfr Internalization Pe	gi|13399864	40654.1													

Peptide Information

Calc. Mass	Obsrv. Mass	± da	± ppm	Start Seq.	End Seq.	Sequence	Ion Score	C. I. %	Modification	Plate [#] Name	Gel Idx/Pos [4700 Sample Name]	iTRAQ Ratio 115/114*	iTRAQ Ratio 116/114*	iTRAQ Ratio 117/114*	Rank	Result Type
1305.7412	1305.7052	-0.036	-28	232	240	ASENAIVWK	52	99.985	(N-term)_iTRAQ[0], Lysine(K)_iTRAQ[9]	[3] F6 and F9	229/221	0.932	1.484	1.268	1	Mascot
1323.7882	1323.7239	-0.0643	-49	17	25	TFITQQGIK	52	99.985	(N-term)_iTRAQ[0], Lysine(K)_iTRAQ[9]	[7] F5 120912	179/171	0.603	0.998	0.769	1	Mascot
1491.8053	1491.8717	0.0664	45	297	306	LNYSDHDVIK	28	95.912	(N-term)_iTRAQ[0], Lysine(K)_iTRAQ[10]	[4] F7 and F10+11	1092/1084	0.282	0.774	0.477	1	Mascot
1665.0071	1665.0851	0.078	47	195	205	SNFKPSLLAQK	57	99.995	(N-term)_iTRAQ[0], Lysine(K)_iTRAQ[4,11]	[4] F7 and F10+11	1128/1120	1.310	0.956	1.050	1	Mascot
1881.9541	1881.9834	0.0293	16	148	162	SISFIPPDGEFELMR	36	99.364	(N-term)_iTRAQ[0]	[8] F13-15 and F1+2	381/373	0.748	0.802	0.836	1	Mascot
2035.098	2035.0145	-0.0835	-41	210	225	IPTPLNTSGVQVICMK	49	99.967	(N-term)_iTRAQ[0], Lysine(K)_iTRAQ[16], MMTS (C)[14]	[1] F3 030912	423/415	0.790	0.696	0.655	1	Mascot
2075.1118	2075.0469	-0.0649	-31	249	264	ESQISAEIELLPTNDK	35	99.266	(N-term)_iTRAQ[0], Lysine(K)_iTRAQ[16]	[1] F3 030912	326/318	2.495	1.523	3.595	1	Mascot
2091.0591	2090.9688	-0.0903	-43	32	48	EEQSQITSQVTGQIGWR	99	100	(N-term)_iTRAQ[0]	[1] F3 030912	294/286	1.174	1.357	1.508	1	Mascot

152	Chain A, Tetra-(5-Fluorotryptophanyl)-Glutathione Transferase	gi|4388948	28485.8	9	407	0.940	1.184	1.253	0.281	0.273	0.222	9	9	9	100	

Peptide Information

Calc. Mass	Obsrv. Mass	± da	± ppm	Start Seq.	End Seq.	Sequence	Ion Score	C. I. %	Modification	Plate [#] Name	Gel Idx/Pos [4700 Sample Name]	iTRAQ Ratio 115/114*	iTRAQ Ratio 116/114*	iTRAQ Ratio 117/114*	Rank	Result Type
1134.6517	1134.6339	-0.0178	-16	211	217	LAQXSNK	35	99.251	(N-term)_iTRAQ[0], Lysine(K)_iTRAQ[7]	[4] F7 and F10+11	191/183	0.861	1.160	1.403	1	Mascot
1192.6572	1192.6332	-0.024	-20	43	49	SQXLNEK	39	99.7	(N-term)_iTRAQ[0], Lysine(K)_iTRAQ[7]	[4] F7 and F10+11	191/183	0.761	1.000	1.259	1	Mascot
1244.7136	1244.6593	-0.0543	-44	136	143	LYSEFLGK	30	97.581	(N-term)_iTRAQ[0], Lysine(K)_iTRAQ[8]	[7] F5 120912	287/279	0.779	0.885	1.093	1	Mascot
1264.7048	1264.6775	-0.0273	-22	144	151	RPXFAGDK	36	99.392	(N-term)_iTRAQ[0], Lysine(K)_iTRAQ[8]	[2] F12 040912	229/221	1.212	1.612	1.847	1	Mascot
1392.7582	1392.73	-0.0282	-20	1	10	PMILGXWNVR	35	99.167	(N-term)_iTRAQ[0]	[3] F6 and F9	398/390	0.628	1.003	1.007	1	Mascot
1547.7607	1547.6836	-0.0771	-50	96	107	ADIVENQVMDNR	56	99.994	(N-term)_iTRAQ[0]	[1] F3 030912	211/203	1.007	1.252	1.146	1	Mascot
1877.963	1877.8645	-0.0985	-52	18	30	LLLEYTDSSYEEK	72	100	(N-term)_iTRAQ[0], Lysine(K)_iTRAQ[13]	[5] F4	238/230	1.154	1.487	1.246	1	Mascot
1934.0508	1934.0342	-0.0166	-9	52	67	LGLDFPNLPYLIDGSR	38	99.6	(N-term)_iTRAQ[0]	[8] F13-15 and F1+2	282/274	1.532	1.515	1.227	1	Mascot
2034.0641	2034.0514	-0.0127	-6	18	31	LLLEYTDSSYEEKR	69	100	(N-term)_iTRAQ[0], Lysine(K)_iTRAQ[13]	[4] F7 and F10+11	304/296	0.826	0.979	1.212	1	Mascot

153	RecName: Full=Dynamin-1-like protein; AltName: Full=Dynamin-like protein	gi|68566301	91186.9	8	405	0.832	0.976	0.847	0.169	0.247	0.516	8	8	8	100	

Peptide Information

Calc. Mass	Obsrv. Mass	± da	± ppm	Start Seq.	End Seq.	Sequence	Ion Score	C. I. %	Modification	Plate [#] Name	Gel Idx/Pos [4700 Sample Name]	iTRAQ Ratio 115/114*	iTRAQ Ratio 116/114*	iTRAQ Ratio 117/114*	Rank	Result Type
1292.6732	1292.6112	-0.062	-48	97	105	GVEAEEWGK	30	97.496	(N-term)_iTRAQ[0], Lysine(K)_iTRAQ[9]	[3] F6 and F9	192/184	0.876	0.835	0.798	1	Mascot
1582.9137	1582.8794	-0.0343	-22	688	698	AVMHFLVNHVK	84	100	(N-term)_iTRAQ[0], Lysine(K)_iTRAQ[11]	[2] F12 040912	342/334	0.768	1.160	1.051	1	Mascot
1631.7898	1631.6775	-0.1123	-69	596	609	IGDGGQEPTTGNWR	52	99.984	(N-term)_iTRAQ[0]	[5] F4	124/116	0.869	0.837	0.759	1	Mascot
1700.9932	1700.9708	-0.0224	-13	639	653	GHAVNLLDVPVPVAR	46	99.945	(N-term)_iTRAQ[0]	[3] F6 and F9	338/330	0.747	0.923	0.387	1	Mascot
1781.9895	1781.9263	-0.0632	-35	699	711	DTLQSELVGQLYK	41	99.793	(N-term)_iTRAQ[0], Lysine(K)_iTRAQ[13]	[5] F4	265/257	1.164	1.607	2.247	1	Mascot
1967.936	1967.9146	-0.0214	-11	230	246	LDLMDAGTDAMDVLMGR	41	99.816	(N-term)_iTRAQ[0]	[8] F13-15 and F1+2	255/247	0.836	0.884	0.785	1	Mascot
2060.0911	2060.0586	-0.0325	-16	270	284	SVTDSIRDEYAFLQK	67	99.999	(N-term)_iTRAQ[0], Lysine(K)_iTRAQ[15]	[4] F7 and F10+11	348/340	0.582	0.756	0.587	1	Mascot
2067.0037	2066.9807	-0.023	-11	712	728	SSLLDDLLTESEDMAQR	45	99.914	(N-term)_iTRAQ[0]	[8] F13-15 and F1+2	266/258	0.927	1.030	1.041	1	Mascot

154	cytochrome c oxidase subunit 4 isoform 1, mitochondrial precursor [Rattus norvegicus]	gi|8393180	22141.9	7	405	0.892	0.994	1.055	0.332	0.196	0.325	8	8	8	100	

Peptide Information

Calc. Mass	Obsrv. Mass	± da	± ppm	Start Seq.	End Seq.	Sequence	Ion Score	C. I. %	Modification	Plate [#] Name	Gel Idx/Pos [4700 Sample Name]	iTRAQ Ratio 115/114*	iTRAQ Ratio 116/114*	iTRAQ Ratio 117/114*	Rank	Result Type
1266.6763	1266.6213	-0.055	-43	136	143	DWVAMQTK	51	99.979	(N-term)_iTRAQ[0], Lysine(K)_iTRAQ[8]	[7] F5 120912	179/171	0.919	0.898	1.100	1	Mascot

Calc. Mass	Obsrv. Mass	± da	± ppm	Start Seq.	End Seq.	Sequence	Ion Score	C. I. %	Modification	Plate [#] Name	Gel Idx/Pos [4700 Sample Name]	iTRAQ Ratio 115/114*	iTRAQ Ratio 116/114*	iTRAQ Ratio 117/114*	Rank	Result Type
1348.7834	1348.764	-0.0194	-14	150	159	VNPIQGFSAK	65	99.999	(N-term)_iTRAQ[0], Lysine(K)_iTRAQ[10]	[7] F5 120912	201/193	1.062	1.119	1.327	1	Mascot
1541.8574	1541.8462	-0.0112	-7	43	53	DYPLPDVAHVK	66	99.999	(N-term)_iTRAQ[0], Lysine(K)_iTRAQ[11]	[4] F7 and F10+11	253/245	1.231	1.119	1.104	1	Mascot
1541.8574	1541.8931	0.0357	23	43	53	DYPLPDVAHVK	42	99.848	(N-term)_iTRAQ[0], Lysine(K)_iTRAQ[11]	[4] F7 and F10+11	260/252	0.454	0.650	0.582	1	Mascot
1558.751	1558.6663	-0.0847	-54	30	41	SEDYALPSYVDR	65	99.999	(N-term)_iTRAQ[0]	[1] F3 030912	242/234	1.255	1.068	1.103	1	Mascot
1697.9585	1697.9004	-0.0581	-34	42	53	RDYPLPDVAHVK	38	99.61	(N-term)_iTRAQ[0], Lysine(K)_iTRAQ[12]	[2] F12 040912	247/239	0.890	1.091	1.145	1	Mascot
1714.8925	1714.9718	0.0793	46	160	168	WDYNKNEWK	50	99.977	(N-term)_iTRAQ[0], Lysine(K)_iTRAQ[5,9]	[4] F7 and F10+11	1131/1123	0.988	1.168	1.493	1	Mascot
1745.8778	1745.8196	-0.0582	-33	84	95	IQFNESFAEMNK	70	100	(N-term)_iTRAQ[0], Lysine(K)_iTRAQ[12]	[7] F5 120912	283/275	0.667	0.959	0.865	1	Mascot

155	RecName: Full=V-type proton ATPase 116 kDa subunit a isoform 1; Short=V-ATPase 116 kDa isoform a1;	gi|139352	102384.8	8	404	0.748	0.935	0.884	0.219	0.173	0.168	8	8	8	100

Peptide Information

Calc. Mass	Obsrv. Mass	± da	± ppm	Start Seq.	End Seq.	Sequence	Ion Score	C. I. %	Modification	Plate [#] Name	Gel Idx/Pos [4700 Sample Name]	iTRAQ Ratio 115/114*	iTRAQ Ratio 116/114*	iTRAQ Ratio 117/114*	Rank	Result Type
1328.7559	1328.7777	0.0218	16	668	678	HLGTLNFGGIR	34	99.024	(N-term)_iTRAQ[0]	[3] F6 and F9	1248/1240	0.701	1.007	0.926	1	Mascot
1436.781	1436.7084	-0.0726	-51	823	832	FLPFSFEHIR	48	99.96	(N-term)_iTRAQ[0]	[6] F8 110912	424/416	0.859	0.938	1.073	1	Mascot
1460.7618	1460.7095	-0.0523	-36	39	49	DLNPDVNVFQR	56	99.994	(N-term)_iTRAQ[0]	[1] F3 030912	265/257	1.231	1.232	1.025	1	Mascot
1680.7827	1680.7067	-0.076	-45	242	254	ASLYPCPETPQER	35	99.216	(N-term)_iTRAQ[0], MMTS (C)[6]	[1] F3 030912	255/247	0.690	0.881	0.900	1	Mascot
1710.8732	1710.8105	-0.0627	-37	355	366	MQTNQTPPTYNK	63	99.999	(N-term)_iTRAQ[0], Lysine(K)_iTRAQ[12]	[7] F5 120912	115/107	0.455	0.919	0.745	1	Mascot
1830.0411	1829.9711	-0.07	-38	218	230	SVFIIFFQGDQLK	57	99.995	(N-term)_iTRAQ[0], Lysine(K)_iTRAQ[13]	[5] F4	395/387	0.728	1.036	1.099	1	Mascot
1970.9727	1970.9059	-0.0668	-34	265	279	IDDLQMVLNQTEDHR	62	99.998	(N-term)_iTRAQ[0]	[3] F6 and F9	360/352	0.757	0.649	0.705	1	Mascot
2180.1436	2180.1619	0.0183	8	302	317	AIYHTLNLCNIDVTQK	49	99.968	(N-term)_iTRAQ[0], Lysine(K)_iTRAQ[16], MMTS (C)[9]	[4] F7 and F10+11	411/403	0.761	0.920	0.705	1	Mascot

156	adenylyl cyclase-associated protein 1 [Rattus norvegicus]	gi|59709467	57740.6	6	404	1.197	1.032	1.015	0.247	0.305	0.446	7	7	7	100

Protein Group

cyclase-associated protein [Rattus norvegicus]	gi|310174	57698.5

Peptide Information

Calc. Mass	Obsrv. Mass	± da	± ppm	Start Seq.	End Seq.	Sequence	Ion Score	C. I. %	Modification	Plate [#] Name	Gel Idx/Pos [4700 Sample Name]	iTRAQ Ratio 115/114*	iTRAQ Ratio 116/114*	iTRAQ Ratio 117/114*	Rank	Result Type
1410.7773	1410.833	0.0557	39	71	80	HAEMVHTGLK	31	98.219	(N-term)_iTRAQ[0], Lysine(K)_iTRAQ[10]	[8] F13-15 and F1+2	1256/1248	1.165	0.868	0.909	1	Mascot
1564.8369	1564.8936	0.0567	36	198	208	EFHTTGLAWSK	72	100	(N-term)_iTRAQ[0], Lysine(K)_iTRAQ[11]	[4] F7 and F10+11	1150/1142	1.200	0.894	0.750	1	Mascot
1606.7114	1606.6085	-0.1029	-64	155	166	EMNDAAMFYTNR	42	99.847	(N-term)_iTRAQ[0]	[5] F4	180/172	1.135	1.015	0.694	1	Mascot
1606.7114	1606.6356	-0.0758	-47	155	166	EMNDAAMFYTNR	58	99.996	(N-term)_iTRAQ[0]	[1] F3 030912	252/244	1.078	0.936	0.961	1	Mascot
1659.0063	1659.0736	0.0673	41	316	326	KEPALLELEGK	57	99.995	(N-term)_iTRAQ[0], Lysine(K)_iTRAQ[1,11]	[4] F7 and F10+11	1169/1161	0.945	0.838	0.967	1	Mascot
1963.0332	1962.9075	-0.1257	-64	84	99	ALLVTASQCQQPAGNK	63	99.999	(N-term)_iTRAQ[0], Lysine(K)_iTRAQ[16], MMTS (C)[9]	[5] F4	180/172	1.820	1.888	2.299	1	Mascot
2653.4487	2653.3953	-0.0534	-20	38	59	GAVPYVQAFDSLLANPVAEYLK	123	100	(N-term)_iTRAQ[0], Lysine(K)_iTRAQ[22]	[1] F3 030912	560/552	1.201	1.067	1.101	1	Mascot

157	rab GDP dissociation inhibitor beta [Rattus norvegicus]	gi|40254781	56394.4	7	402	1.162	0.952	0.926	0.517	0.250	0.228	7	7	7	100

Protein Group

rab GDI beta [Rattus norvegicus]	gi|396433	56398.3

Peptide Information

Calc. Mass	Obsrv. Mass	± da	± ppm	Start Seq.	End Seq.	Sequence	Ion Score	C. I. %	Modification	Plate [#] Name	Gel Idx/Pos [4700 Sample Name]	iTRAQ Ratio 115/114*	iTRAQ Ratio 116/114*	iTRAQ Ratio 117/114*	Rank	Result Type
1495.7877	1495.7437	-0.044	-29	391	402	DLGTDSQIFISR	35	99.179	(N-term)_iTRAQ[0]	[1] F3 030912	269/261	1.577	1.019	0.627	1	Mascot
1539.9032	1539.837	-0.0662	-43	380	390	FVSISDLFVPK	54	99.99	(N-term)_iTRAQ[0], Lysine(K)_iTRAQ[11]	[5] F4	354/346	1.600	0.984	0.791	1	Mascot
1600.9056	1600.9763	0.0707	44	69	79	GRDWNVDLIPK	74	100	(N-term)_iTRAQ[0], Lysine(K)_iTRAQ[11]	[4] F7 and F10+11	1187/1179	1.222	1.141	1.253	1	Mascot

Calc. Mass	Obsrv. Mass	± da	± ppm	Start Seq.	End Seq.	Sequence	Ion Score	C. I. %	Modification	Plate [#] Name	Gel Idx/Pos [4700 Sample Name]	iTRAQ Ratio 115/114*	iTRAQ Ratio 116/114*	iTRAQ Ratio 117/114*	Rank	Result Type
1673.9407	1673.9261	-0.0146	-9	56	68	FKLPGQPPASMGR	57	99.995	(N-term)_iTRAQ[0], Lysine(K)_iTRAQ[2]	[4] F7 and F10+11	253/245	1.019	0.747	0.903	1	Mascot
1894.8925	1894.798	-0.0945	-50	424	436	MTGSEFDFEEMKR	47	99.946	(N-term)_iTRAQ[0], Lysine(K)_iTRAQ[12]	[6] F8 110912	290/282	1.322	0.988	1.139	1	Mascot
2268.2043	2268.1555	-0.0488	-22	119	137	VPSTEAEALASSLMGLFE K	106	100	(N-term)_iTRAQ[0], Lysine(K)_iTRAQ[19]	[1] F3 030912	569/561	1.364	1.329	1.073	1	Mascot
2583.3816	2583.4573	0.0757	29	174	193	KFDLGQDVIDFTGHSLAL YR	31	98.074	(N-term)_iTRAQ[0], Lysine(K)_iTRAQ[1]	[4] F7 and F10+11	1338/1330	0.506	0.630	0.853	1	Mascot

158	cofilin-1 [Rattus norvegicus]	gi|8393101	22451.3	7	400	1.101	0.958	0.965	0.200	0.251	0.195	7	7	7	100

Peptide Information

Calc. Mass	Obsrv. Mass	± da	± ppm	Start Seq.	End Seq.	Sequence	Ion Score	C. I. %	Modification	Plate [#] Name	Gel Idx/Pos [4700 Sample Name]	iTRAQ Ratio 115/114*	iTRAQ Ratio 116/114*	iTRAQ Ratio 117/114*	Rank	Result Type
1041.5648	1041.5504	-0.0144	-14	14	19	VFNDMK	30	97.342	(N-term)_iTRAQ[0], Lysine(K)_iTRAQ[6]	[4] F7 and F10+11	191/183	1.493	1.286	1.331	1	Mascot
1203.7195	1203.6732	-0.0463	-38	46	53	NIILEEGK	51	99.98	(N-term)_iTRAQ[0], Lysine(K)_iTRAQ[8]	[7] F5 120912	170/162	1.066	1.130	0.983	1	Mascot
1311.645	1311.7112	0.0662	50	74	81	MLPDKDCR	36	99.368	(N-term)_iTRAQ[0], Lysine(K)_iTRAQ[5], MMTS (C)[7]	[4] F7 and F10+11	1088/1080	0.855	0.672	0.696	1	Mascot
1625.8308	1625.7245	-0.1063	-65	82	92	YALYDATYETK	66	99.999	(N-term)_iTRAQ[0], Lysine(K)_iTRAQ[11]	[5] F4	173/165	1.003	0.885	0.976	1	Mascot
1730.9537	1730.9944	0.0407	24	35	45	AVLFCLSEDKK	82	100	(N-term)_iTRAQ[0], Lysine(K)_iTRAQ[10, 11], MMTS (C)[5]	[3] F6 and F9	1303/1295	1.251	0.785	0.839	1	Mascot
1796.8538	1796.8938	0.04	22	133	144	HELQANCYEEVK	79	100	(N-term)_iTRAQ[0], Lysine(K)_iTRAQ[12], MMTS (C)[7]	[3] F6 and F9	1193/1185	1.151	1.275	1.037	1	Mascot
2067.9819	2067.9243	-0.0576	-28	133	146	HELQANCYEEVKDR	58	99.996	(N-term)_iTRAQ[0], Lysine(K)_iTRAQ[12], MMTS (C)[7]	[2] F12 040912	262/254	1.000	0.855	1.011	1	Mascot

159	beta-enolase [Rattus norvegicus]	gi|126723393	52880.2	6	397	1.289	1.405	1.152	0.982	0.955	0.932	10	10	10	100

Peptide Information

Calc. Mass	Obsrv. Mass	± da	± ppm	Start Seq.	End Seq.	Sequence	Ion Score	C. I. %	Modification	Plate [#] Name	Gel Idx/Pos [4700 Sample Name]	iTRAQ Ratio 115/114*	iTRAQ Ratio 116/114*	iTRAQ Ratio 117/114*	Rank	Result Type
1088.5874	1088.5542	-0.0332	-30	257	262	YDLDFK	29	97.132	(N-term)_iTRAQ[0], Lysine(K)_iTRAQ[6]	[3] F6 and F9	256/248	1.076	0.966	1.088	1	Mascot
1098.6517	1098.6979	0.0462	42	65	71	AVEHINK	40	99.78	(N-term)_iTRAQ[0], Lysine(K)_iTRAQ[7]	[4] F7 and F10+11	989/981	0.558	1.078	0.387	1	Mascot
1668.9166	1668.8774	-0.0392	-23	16	28	GNPTVEVDLHTAK	34	98.937	(N-term)_iTRAQ[0], Lysine(K)_iTRAQ[13]	[6] F8 110912	197/189	0.795	0.779	0.637	1	Mascot
1668.9166	1668.8903	-0.0263	-16	16	28	GNPTVEVDLHTAK	119	100	(N-term)_iTRAQ[0], Lysine(K)_iTRAQ[13]	[4] F7 and F10+11	193/185	1.082	0.964	0.999	1	Mascot
1796.9993	1796.9619	-0.0374	-21	106	120	FGANAILGVSLAVCK	56	99.994	(N-term)_iTRAQ[0], Lysine(K)_iTRAQ[15], MMTS (C)[14]	[2] F12 040912	523/515	3.175	3.392	3.289	1	Mascot
1796.9993	1796.9692	-0.0301	-17	106	120	FGANAILGVSLAVCK	90	100	(N-term)_iTRAQ[0], Lysine(K)_iTRAQ[15], MMTS (C)[14]	[1] F3 030912	517/509	0.897	1.051	0.925	1	Mascot
1796.9993	1796.983	-0.0163	-9	106	120	FGANAILGVSLAVCK	50	99.973	(N-term)_iTRAQ[0], Lysine(K)_iTRAQ[15], MMTS (C)[14]	[1] F3 030912	589/581	2.528	2.618	1.762	1	Mascot
1910.9906	1910.9333	-0.0573	-30	344	358	VNQIGSVTESIQACK	72	100	(N-term)_iTRAQ[0], Lysine(K)_iTRAQ[15], MMTS (C)[14]	[5] F4	258/250	1.070	1.182	1.188	1	Mascot
1949.0464	1949.0646	0.0182	9	33	50	AAVPSGASTGIYEALELR	43	99.878	(N-term)_iTRAQ[0]	[8] F13-15 and F1+2	397/389	1.044	1.100	0.973	1	Mascot
1949.0464	1949.1187	0.0723	37	33	50	AAVPSGASTGIYEALELR	47	99.954	(N-term)_iTRAQ[0]	[8] F13-15 and F1+2	1089/1081	3.036	3.154	2.483	1	Mascot

160	citrate synthase, mitochondrial precursor [Rattus norvegicus]	gi|18543177	56144.2	9	397	0.914	1.016	1.015	0.682	0.977	0.580	13	13	13	100

Peptide Information

Calc. Mass	Obsrv. Mass	± da	± ppm	Start Seq.	End Seq.	Sequence	Ion Score	C. I. %	Modification	Plate [#] Name	Gel Idx/Pos [4700 Sample Name]	iTRAQ Ratio 115/114*	iTRAQ Ratio 116/114*	iTRAQ Ratio 117/114*	Rank	Result Type
1122.7133	1122.6899	-0.0234	-21	376	382	LVAQLYK	41	99.794	(N-term)_iTRAQ[0], Lysine(K)_iTRAQ[7]	[7] F5 120912	212/204	1.064	1.048	1.098	1	Mascot
1311.7657	1311.6892	-0.0765	-58	341	351	VVPGYGHAVLR	44	99.899	(N-term)_iTRAQ[0]	[6] F8 110912	221/213	1.331	1.253	1.107	1	Mascot
1311.7657	1311.7389	-0.0268	-20	341	351	VVPGYGHAVLR	30	97.658	(N-term)_iTRAQ[0]	[4] F7 and F10+11	218/210	0.233	0.262	0.382	1	Mascot
1415.8621	1415.9155	0.0534	38	441	450	ALGFPLERPK	39	99.709	(N-term)_iTRAQ[0], Lysine(K)_iTRAQ[10]	[3] F6 and F9	1227/1219	0.913	1.167	0.934	1	Mascot
1470.8916	1470.8638	-0.0278	-19	429	440	ALGVLAQLIWSR	53	99.988	(N-term)_iTRAQ[0]	[1] F3 030912	548/540	1.639	1.700	2.103	1	Mascot
1482.7461	1482.682	-0.0641	-43	330	340	DYIWNTLNSGR	51	99.98	(N-term)_iTRAQ[0]	[5] F4	252/244	1.112	1.294	1.264	1	Mascot
1482.7461	1482.7007	-0.0454	-31	330	340	DYIWNTLNSGR	27	95.434	(N-term)_iTRAQ[0]	[1] F3 030912	328/320	0.822	0.324	0.607	1	Mascot

Calc. Mass	Obsrv. Mass	± da	± ppm	Start Seq.	End Seq.	Sequence	Ion Score	C. I. %	Modification	Plate [#] Name	Gel Idx/Pos [4700 Sample Name]	iTRAQ Ratio 115/114*	iTRAQ Ratio 116/114*	iTRAQ Ratio 117/114*	Rank	Result Type
1511.9407	1511.8917	-0.049	-32	383	393	IVPNILLEQGK	52	99.984	(N-term)_iTRAQ[0], Lysine(K)_iTRAQ[11]	[5] F4	257/249	0.800	0.920	0.923	1	Mascot
1537.8445	1537.9113	0.0668	43	318	327	EVGKDVSDEK	37	99.515	(N-term)_iTRAQ[0], Lysine(K)_iTRAQ[4,10]	[4] F7 and F10+11	993/985	2.522	4.389	2.359	1	Mascot
1544.8994	1544.9691	0.0697	45	367	375	HLPKDPMFK	33	98.695	(N-term)_iTRAQ[0], Lysine(K)_iTRAQ[4,9]	[8] F13-15 and F1+2	1191/1183	0.702	0.923	1.026	1	Mascot
1544.8994	1544.9988	0.0994	64	367	375	HLPKDPMFK	33	98.821	(N-term)_iTRAQ[0], Lysine(K)_iTRAQ[4,9]	[8] F13-15 and F1+2	1522/1514	1.040	0.759	1.145	1	Mascot
1906.9882	1907.0054	0.0172	9	77	92	GLVYETSVLDPDEGIR	47	99.949	(N-term)_iTRAQ[0]	[8] F13-15 and F1+2	430/422	1.016	1.475	0.760	1	Mascot
1906.9882	1907.0146	0.0264	14	77	92	GLVYETSVLDPDEGIR	33	98.692	(N-term)_iTRAQ[0]	[8] F13-15 and F1+2	530/522	0.459	1.021	0.891	1	Mascot

161	dual specificity mitogen-activated protein kinase kinase 1 [Rattus norvegicus]	gi|13928886	47892.2	8	394	1.062	1.082	1.125	0.256	0.263	0.285	9	9	9	100

Peptide Information

Calc. Mass	Obsrv. Mass	± da	± ppm	Start Seq.	End Seq.	Sequence	Ion Score	C. I. %	Modification	Plate [#] Name	Gel Idx/Pos [4700 Sample Name]	iTRAQ Ratio 115/114*	iTRAQ Ratio 116/114*	iTRAQ Ratio 117/114*	Rank	Result Type
1185.7452	1185.6991	-0.0461	-39	161	168	IPEQILGK	40	99.737	(N-term)_iTRAQ[0], Lysine(K)_iTRAQ[8]	[7] F5 120912	227/219	0.995	1.030	0.929	1	Mascot
1374.8177	1374.8523	0.0346	25	354	362	QLMVHAFIK	56	99.994	(N-term)_iTRAQ[0], Lysine(K)_iTRAQ[9]	[3] F6 and F9	1246/1238	1.478	0.864	1.602	1	Mascot
1393.8413	1393.8921	0.0508	36	49	57	RLEAFLTQK	39	99.674	(N-term)_iTRAQ[0], Lysine(K)_iTRAQ[9]	[4] F7 and F10+11	1176/1168	1.052	1.057	1.009	1	Mascot
1569.9144	1569.8627	-0.0517	-33	85	96	VSHKPSGLVMAR	45	99.927	(N-term)_iTRAQ[0], Lysine(K)_iTRAQ[4]	[2] F12 040912	180/172	0.813	1.114	0.911	1	Mascot
1629.9534	1629.8768	-0.0766	-47	190	201	DVKPSNILVNSR	37	99.544	(N-term)_iTRAQ[0], Lysine(K)_iTRAQ[3]	[6] F8 110912	206/198	1.111	1.833	1.304	1	Mascot
1726.9235	1727.0132	0.0897	52	60	70	VGELKDDDFEK	43	99.878	(N-term)_iTRAQ[0], Lysine(K)_iTRAQ[5,11]	[4] F7 and F10+11	1104/1096	0.978	1.117	1.148	1	Mascot
1817.9855	1817.8859	-0.0996	-55	36	47	KLEELELDEQQR	43	99.865	(N-term)_iTRAQ[0], Lysine(K)_iTRAQ[1]	[6] F8 110912	247/239	0.791	0.866	0.781	1	Mascot
1817.9855	1818.0144	0.0289	16	36	47	KLEELELDEQQR	62	99.998	(N-term)_iTRAQ[0], Lysine(K)_iTRAQ[1]	[3] F6 and F9	1201/1193	1.542	1.207	1.495	1	Mascot
2115.1372	2115.0659	-0.0713	-34	325	340	LPSGVFSLEFQDFVNK	74	100	(N-term)_iTRAQ[0], Lysine(K)_iTRAQ[16]	[1] F3 030912	496/488	1.029	0.908	1.212	1	Mascot

162	6-phosphofructokinase, muscle type [Rattus norvegicus]	gi|13929002	92536	8	393	1.270	1.174	1.176	0.475	0.318	0.578	8	8	8	100

Peptide Information

Calc. Mass	Obsrv. Mass	± da	± ppm	Start Seq.	End Seq.	Sequence	Ion Score	C. I. %	Modification	Plate [#] Name	Gel Idx/Pos [4700 Sample Name]	iTRAQ Ratio 115/114*	iTRAQ Ratio 116/114*	iTRAQ Ratio 117/114*	Rank	Result Type
1195.663	1195.6348	-0.0282	-24	202	210	TFVLEVMGR	32	98.334	(N-term)_iTRAQ[0]	[1] F3 030912	363/355	0.867	1.180	1.266	1	Mascot
1532.9298	1532.8793	-0.0505	-33	717	727	ALVFQPVTELK	57	99.995	(N-term)_iTRAQ[0], Lysine(K)_iTRAQ[11]	[5] F4	257/249	1.014	1.189	0.945	1	Mascot
1594.8597	1594.8016	-0.0581	-36	346	356	LPLMECVQVTK	48	99.957	(N-term)_iTRAQ[0], Lysine(K)_iTRAQ[11], MMTS (C)[6]	[5] F4	332/324	2.377	1.926	2.517	1	Mascot
1671.9679	1671.9431	-0.0248	-15	433	445	VLVVHDGFEGLAK	65	99.999	(N-term)_iTRAQ[0], Lysine(K)_iTRAQ[13]	[4] F7 and F10+11	305/297	1.016	0.830	1.025	1	Mascot
1709.9796	1709.9335	-0.0461	-27	604	615	DLQVNVEHLVQK	39	99.663	(N-term)_iTRAQ[0], Lysine(K)_iTRAQ[12]	[4] F7 and F10+11	290/282	1.416	0.915	0.930	1	Mascot
1735.9113	1735.9109	-0.0004	0	130	141	SEWSDLLNDLQK	68	100	(N-term)_iTRAQ[0], Lysine(K)_iTRAQ[12]	[7] F5 120912	409/401	0.967	1.113	0.600	1	Mascot
2295.1125	2295.0935	-0.019	-8	755	772	YEIDLDTSDHAHLEHISR	54	99.99	(N-term)_iTRAQ[0]	[2] F12 040912	274/266	1.658	1.138	1.295	1	Mascot
2308.2139	2308.1223	-0.0916	-40	398	420	GGLHTVAVMNVGAPAAGMNAAVR	32	98.449	(N-term)_iTRAQ[0]	[5] F4	216/208	1.408	1.393	1.638	1	Mascot

163	dynamin-3 [Rattus norvegicus]	gi|19924077	106388.7	8	389	0.792	0.876	0.792	0.317	0.395	0.304	11	11	11	100

Protein Group

testicular dynamin [Rattus norvegicus]	gi|391872	104215.4

Peptide Information

Calc. Mass	Obsrv. Mass	± da	± ppm	Start Seq.	End Seq.	Sequence	Ion Score	C. I. %	Modification	Plate [#] Name	Gel Idx/Pos [4700 Sample Name]	iTRAQ Ratio 115/114*	iTRAQ Ratio 116/114*	iTRAQ Ratio 117/114*	Rank	Result Type
1167.7024	1167.6805	-0.0219	-19	370	376	FPFEIVK	33	98.701	(N-term)_iTRAQ[0], Lysine(K)_iTRAQ[7]	[7] F5 120912	356/348	0.702	0.980	0.793	1	Mascot
1251.6818	1251.6278	-0.054	-43	45	54	SSVLENFVGR	47	99.954	(N-term)_iTRAQ[0]	[5] F4	218/210	1.032	1.015	1.163	1	Mascot
1379.6597	1379.5951	-0.0646	-47	207	217	LDLMDEGTDAR	41	99.787	(N-term)_iTRAQ[0]	[1] F3 030912	232/224	0.735	1.142	0.629	1	Mascot
1394.7964	1394.7635	-0.0329	-24	569	577	YMLPLDNLK	29	97.145	(N-term)_iTRAQ[0], Lysine(K)_iTRAQ[9]	[5] F4	268/260	0.445	0.628	0.429	1	Mascot
1444.7682	1444.8235	0.0553	38	90	98	KFTDFDEVR	31	98.236	(N-term)_iTRAQ[0], Lysine(K)_iTRAQ[1]	[4] F7 and F10+11	1123/1115	1.543	1.543	0.744	1	Mascot

Calc. Mass	Obsrv. Mass	± da	± ppm	Start Seq.	End Seq.	Sequence	Ion Score	C. I. %	Modification	Plate [#] Name	Gel Idx/Pos [4700 Sample Name]	iTRAQ Ratio 115/114*	iTRAQ Ratio 116/114*	iTRAQ Ratio 117/114*	Rank	Result Type
1544.7468	1544.7826	0.0358	23	78	87	AEYAEFLHCK	44	99.913	(N-term)_iTRAQ[0], Lysine(K)_iTRAQ[10], MMTS (C)[9]	[3] F6 and F9	1283/1275	0.569	0.395	0.471	1	Mascot
1544.7468	1544.8055	0.0587	38	78	87	AEYAEFLHCK	56	99.993	(N-term)_iTRAQ[0], Lysine(K)_iTRAQ[10], MMTS (C)[9]	[4] F7 and F10+11	1220/1212	1.084	0.945	0.939	1	Mascot
1928.0449	1927.9723	-0.0726	-38	400	414	TGLFTPDMAFEAIVK	45	99.93	(N-term)_iTRAQ[0], Lysine(K)_iTRAQ[15]	[1] F3 030912	471/463	0.762	0.583	1.059	1	Mascot
2055.1194	2055.0427	-0.0767	-37	328	342	ALLQMVQQFAVDFEK	64	99.999	(N-term)_iTRAQ[0], Lysine(K)_iTRAQ[15]	[5] F4	435/427	0.835	1.228	1.215	1	Mascot
2055.1194	2055.0669	-0.0525	-26	328	342	ALLQMVQQFAVDFEK	108	100	(N-term)_iTRAQ[0], Lysine(K)_iTRAQ[15]	[1] F3 030912	574/566	0.555	0.737	0.869	1	Mascot
2071.1143	2071.0413	-0.073	-35	328	342	ALLQMVQQFAVDFEK	35	99.298	(N-term)_iTRAQ[0], Lysine(K)_iTRAQ[15], Oxidation (M)[5]	[5] F4	435/427	0.967	1.075	0.842	1	Mascot

164	neurofascin isoform 3 precursor [Rattus norvegicus]	gi|19924211	140570.6	8	386	1.107	0.990	1.040	0.237	0.411	0.625	8	8	8	100

Protein Group

ankyrin binding cell adhesion molecule neurofascin [Rattus norvegicus]	gi|1842427	139724.1
ankyrin binding cell adhesion molecule neurofascin [Rattus norvegicus]	gi|1842429	144138.4
neurofascin isoform 1 precursor [Rattus norvegicus]	gi|237858623	146631.8

Peptide Information

Calc. Mass	Obsrv. Mass	± da	± ppm	Start Seq.	End Seq.	Sequence	Ion Score	C. I. %	Modification	Plate [#] Name	Gel Idx/Pos [4700 Sample Name]	iTRAQ Ratio 115/114*	iTRAQ Ratio 116/114*	iTRAQ Ratio 117/114*	Rank	Result Type
1298.7189	1298.6626	-0.0563	-43	356	366	NLILAPGEDGR	31	97.824	(N-term)_iTRAQ[0]	[1] F3 030912	219/211	0.856	0.920	0.636	1	Mascot
1413.7147	1413.7523	0.0376	27	61	71	GNPAPSFHWTR	40	99.778	(N-term)_iTRAQ[0]	[3] F6 and F9	1190/1182	1.178	0.764	0.847	1	Mascot
1477.7937	1477.7745	-0.0192	-13	346	355	AAPYWLDEPK	62	99.998	(N-term)_iTRAQ[0], Lysine(K)_iTRAQ[10]	[7] F5 120912	239/231	1.169	1.214	0.888	1	Mascot
1574.8689	1574.9769	0.108	69	211	220	FHFTHTIQQK	47	99.951	(N-term)_iTRAQ[0], Lysine(K)_iTRAQ[10]	[8] F13-15 and F1+2	1534/1526	1.470	1.931	2.699	1	Mascot
1752.8918	1752.8783	-0.0135	-8	461	474	LDCPFFGSPIPTLR	45	99.922	(N-term)_iTRAQ[0], MMTS (C)[3]	[8] F13-15 and F1+2	274/266	0.810	1.078	0.534	1	Mascot
2174.0935	2174.0369	-0.0566	-26	718	736	TSGAPPESNPSDVKGEG TR	52	99.984	(N-term)_iTRAQ[0], Lysine(K)_iTRAQ[14]	[4] F7 and F10+11	105/97	1.142	0.528	1.282	1	Mascot
2307.2595	2307.2639	0.0044	2	564	580	LTVSWLKDDEPLYIGNR	36	99.371	(N-term)_iTRAQ[0], Lysine(K)_iTRAQ[7]	[4] F7 and F10+11	422/414	1.028	1.025	1.242	1	Mascot
2331.2405	2331.1453	-0.0952	-41	504	520	KEDQGIYTCVATNILGK	74	100	(N-term)_iTRAQ[0], Lysine(K)_iTRAQ[1,1 7], MMTS (C)[9]	[6] F8 110912	410/402	1.368	0.963	1.246	1	Mascot

165	assembly protein 180 (AP180) [Rattus norvegicus]	gi|55725	97177.3	7	386	0.839	1.038	0.967	0.141	0.203	0.415	8	8	8	100

Protein Group

clathrin coat assembly protein AP180 [Rattus norvegicus]	gi|13994177	99310.2

Peptide Information

Calc. Mass	Obsrv. Mass	± da	± ppm	Start Seq.	End Seq.	Sequence	Ion Score	C. I. %	Modification	Plate [#] Name	Gel Idx/Pos [4700 Sample Name]	iTRAQ Ratio 115/114*	iTRAQ Ratio 116/114*	iTRAQ Ratio 117/114*	Rank	Result Type
1129.5585	1129.4935	-0.065	-58	140	147	QMAFDFAR	41	99.787	(N-term)_iTRAQ[0]	[5] F4	192/184	0.725	1.345	0.772	1	Mascot
1141.649	1141.5953	-0.0537	-47	93	100	FIQYLASR	34	99.07	(N-term)_iTRAQ[0]	[5] F4	250/242	0.698	0.729	1.246	1	Mascot
1601.7502	1601.6802	-0.07	-44	113	125	SGSHGYDMSTFIR	54	99.991	(N-term)_iTRAQ[0]	[6] F8 110912	260/252	0.837	1.207	0.959	1	Mascot
1661.936	1661.8787	-0.0573	-34	885	896	DPLADLNIKDFL	78	100	(N-term)_iTRAQ[0], Lysine(K)_iTRAQ[9]	[5] F4	405/397	1.100	1.047	1.351	1	Mascot
1661.936	1661.9028	-0.0332	-20	885	896	DPLADLNIKDFL	27	95.262	(N-term)_iTRAQ[0], Lysine(K)_iTRAQ[9]	[1] F3 030912	530/522	0.943	0.931	0.495	1	Mascot
1677.9296	1677.9954	0.0658	39	770	780	KGDLQWNAGEK	65	99.999	(N-term)_iTRAQ[0], Lysine(K)_iTRAQ[1,1 1]	[4] F7 and F10+11	1071/1063	0.688	1.096	0.698	1	Mascot
1713.9421	1713.8822	-0.0599	-35	101	112	NTLFNLSNFLDK	67	99.999	(N-term)_iTRAQ[0], Lysine(K)_iTRAQ[12]	[5] F4	366/358	0.876	1.155	1.516	1	Mascot
1972.0835	1972.1641	0.0806	41	289	304	KPGNNEGSGAPSPLSK	49	99.967	(N-term)_iTRAQ[0], Lysine(K)_iTRAQ[1,1 6]	[4] F7 and F10+11	1037/1029	0.926	0.924	1.173	1	Mascot

166	glial fibrillary acidic protein [Rattus norvegicus]	gi|158186732	52998.7	8	386	3.437	1.939	1.527	2.581	0.865	1.001	8	8	8	100

Protein Group

glial fibrillary acidic protein [Rattus norvegicus]	gi|430721	52984.7

glial fibrillary acidic protein delta [Rattus norvegicus] gi|5030428 51536.2

Peptide Information

Calc. Mass	Obsrv. Mass	± da	± ppm	Start Seq.	End Seq.	Sequence	Ion Score	C. I. %	Modification	Plate [#] Name	Gel Idx/Pos [4700 Sample Name]	iTRAQ Ratio 115/114*	iTRAQ Ratio 116/114*	iTRAQ Ratio 117/114*	Rank	Result Type
1242.729	1242.665	-0.064	-51	94	103	ALAAELNQLR	39	99.704	(N-term)_iTRAQ[0]	[5] F4	227/219	1.873	1.839	1.628	1	Mascot
1252.5786	1252.4935	-0.0851	-68	69	77	AEMMELNDR	48	99.963	(N-term)_iTRAQ[0]	[5] F4	128/120	2.178	1.675	0.729	1	Mascot
1435.7665	1435.6912	-0.0753	-52	161	171	LEAENNLAVYR	32	98.519	(N-term)_iTRAQ[0]	[5] F4	158/150	2.450	1.306	1.090	1	Mascot
1679.8562	1679.8771	0.0209	12	286	298	QLQALTCDLESLR	62	99.998	(N-term)_iTRAQ[0], MMTS (C)[7]	[8] F13-15 and F1+2	374/366	3.046	1.184	0.875	1	Mascot
1697.9471	1697.8851	-0.062	-37	48	61	VDFSLAGALNAGFK	39	99.698	(N-term)_iTRAQ[0], Lysine(K)_iTRAQ[14]	[5] F4	454/446	3.214	2.780	2.224	1	Mascot
1787.9901	1788.0612	0.0711	40	343	354	HLQEYQDLLNVK	87	100	(N-term)_iTRAQ[0], Lysine(K)_iTRAQ[12]	[3] F6 and F9	1263/1255	10.678	3.686	3.547	1	Mascot
1908.0687	1907.9751	-0.0936	-49	187	199	KVESLEEEIQFLR	48	99.957	(N-term)_iTRAQ[0], Lysine(K)_iTRAQ[1]	[6] F8 110912	404/396	6.795	2.584	2.385	1	Mascot
1936.9895	1936.9554	-0.0341	-18	329	342	LEEEGQSLKEEMAR	32	98.272	(N-term)_iTRAQ[0], Lysine(K)_iTRAQ[9]	[4] F7 and F10+11	274/266	2.747	1.585	1.389	1	Mascot

167	sarcoplasmic/endoplasmic reticulum calcium ATPase 2 isoform a [Rattus norvegicus]	gi|161016776	124673.4	8	384	1.113	0.915	0.968	0.333	0.289	0.489	9	9	9	100

Protein Group

sarcoplasmic/endoplasmic reticulum calcium ATPase 2 isoform b [Rattus norvegicus] gi|158635975 119163.9

Peptide Information

Calc. Mass	Obsrv. Mass	± da	± ppm	Start Seq.	End Seq.	Sequence	Ion Score	C. I. %	Modification	Plate [#] Name	Gel Idx/Pos [4700 Sample Name]	iTRAQ Ratio 115/114*	iTRAQ Ratio 116/114*	iTRAQ Ratio 117/114*	Rank	Result Type
1384.7393	1384.7166	-0.0227	-16	452	460	MNVFDTELK	29	97.132	(N-term)_iTRAQ[0], Lysine(K)_iTRAQ[9]	[7] F5 120912	262/254	1.749	1.254	2.008	1	Mascot
1422.6985	1422.6243	-0.0742	-52	656	666	EFDELSPSAQR	66	99.999	(N-term)_iTRAQ[0]	[1] F3 030912	207/199	0.876	0.673	0.845	1	Mascot
1604.8165	1604.7437	-0.0728	-45	36	47	WGSNELPAEEGK	67	99.999	(N-term)_iTRAQ[0], Lysine(K)_iTRAQ[12]	[7] F5 120912	179/171	1.579	1.188	0.886	1	Mascot
1620.8136	1620.7177	-0.0959	-59	235	246	IRDEMVATEQER	41	99.816	(N-term)_iTRAQ[0]	[6] F8 110912	185/177	1.006	0.721	0.752	1	Mascot
1850.9204	1850.8259	-0.0945	-51	573	585	EEMHLEDSANFIK	31	97.918	(N-term)_iTRAQ[0], Lysine(K)_iTRAQ[13]	[6] F8 110912	271/263	1.310	0.709	1.027	1	Mascot
1863.0685	1862.9674	-0.1011	-54	175	189	VDQSILTGESVSVIK	29	96.692	(N-term)_iTRAQ[0], Lysine(K)_iTRAQ[15]	[5] F4	229/221	0.820	1.417	1.675	1	Mascot
1863.0685	1863.0052	-0.0633	-34	175	189	VDQSILTGESVSVIK	65	99.999	(N-term)_iTRAQ[0], Lysine(K)_iTRAQ[15]	[1] F3 030912	305/297	1.215	1.087	1.160	1	Mascot
1933.9895	1933.916	-0.0735	-38	864	876	VSFYQLSHFLQCK	58	99.996	(N-term)_iTRAQ[0], Lysine(K)_iTRAQ[13], MMTS (C)[12]	[6] F8 110912	488/480	0.983	0.748	0.590	1	Mascot
2468.2715	2468.2437	-0.0278	-11	111	128	NAENAIEALKEYEPEMGK	28	95.679	(N-term)_iTRAQ[0], Lysine(K)_iTRAQ[10, 18]	[4] F7 and F10+11	388/380	0.838	0.764	0.561	1	Mascot

168	A-kinase anchor protein 5 [Rattus norvegicus]	gi|19424156	84198.9	5	384	0.710	1.040	1.196	0.219	0.232	0.297	5	5	5	100

Peptide Information

Calc. Mass	Obsrv. Mass	± da	± ppm	Start Seq.	End Seq.	Sequence	Ion Score	C. I. %	Modification	Plate [#] Name	Gel Idx/Pos [4700 Sample Name]	iTRAQ Ratio 115/114*	iTRAQ Ratio 116/114*	iTRAQ Ratio 117/114*	Rank	Result Type
1504.8104	1504.7324	-0.078	-52	147	157	VQGEADDLEIK	65	99.999	(N-term)_iTRAQ[0], Lysine(K)_iTRAQ[11]	[7] F5 120912	172/164	1.049	1.399	1.772	1	Mascot
1701.9633	1701.885	-0.0783	-46	374	386	LSQIEEPAISQAK	87	100	(N-term)_iTRAQ[0], Lysine(K)_iTRAQ[13]	[5] F4	160/152	0.776	1.072	1.051	1	Mascot
1960.0055	1959.9912	-0.0143	-7	412	427	ATMGQAEEATVGHIEK	74	100	(N-term)_iTRAQ[0], Lysine(K)_iTRAQ[16]	[4] F7 and F10+11	230/222	0.543	0.760	0.913	1	Mascot
2287.1975	2287.1904	-0.0071	-3	96	112	QKPSEAEMQPEDGALPK	66	99.999	(N-term)_iTRAQ[0], Lysine(K)_iTRAQ[2,17]	[4] F7 and F10+11	191/183	0.506	0.949	1.234	1	Mascot
2300.2483	2300.1809	-0.0674	-29	671	688	TSEQYETLLIETASSLVK	91	100	(N-term)_iTRAQ[0], Lysine(K)_iTRAQ[18]	[1] F3 030912	523/515	0.805	1.125	1.167	1	Mascot

169	ATP synthase subunit b, mitochondrial precursor [Rattus norvegicus]	gi|19705465	32256.8	7	383	0.953	1.085	0.925	0.178	0.245	0.521	10	10	10	100

Peptide Information

Calc. Mass	Obsrv. Mass	± da	± ppm	Start Seq.	End Seq.	Sequence	Ion Score	C. I. %	Modification	Plate [#] Name	Gel Idx/Pos [4700 Sample Name]	iTRAQ Ratio 115/114*	iTRAQ Ratio 116/114*	iTRAQ Ratio 117/114*	Rank	Result Type

Calc. Mass	Obsrv. Mass	± da	± ppm	Start Seq.	End Seq.	Sequence	Ion Score	C. I. %	Modification	Plate [#] Name	Gel Idx/Pos [4700 Sample Name]	iTRAQ Ratio 115/114*	iTRAQ Ratio 116/114*	iTRAQ Ratio 117/114*	Rank	Result Type
982.5292	982.5006	-0.0286	-29	239	244	CIGDLK	27	95.24	(N-term)_iTRAQ[0], Lysine(K)_iTRAQ[6], MMTS (C)[1]	[3] F6 and F9	229/221	1.205	1.125	1.321	1	Mascot
1221.65	1221.6987	0.0487	40	164	171	HYLFDVQR	31	98.011	(N-term)_iTRAQ[0]	[3] F6 and F9	1210/1202	0.699	0.896	1.207	1	Mascot
1221.65	1221.7079	0.0579	47	164	171	HYLFDVQR	49	99.972	(N-term)_iTRAQ[0]	[4] F7 and F10+11	1144/1136	1.117	1.359	1.147	1	Mascot
1231.7507	1231.6946	-0.0561	-46	132	139	IAQLEEIK	53	99.986	(N-term)_iTRAQ[0], Lysine(K)_iTRAQ[8]	[7] F5 120912	219/211	0.869	0.903	0.885	1	Mascot
1655.9326	1655.9668	0.0342	21	222	233	HVIQSISAQQEK	94	100	(N-term)_iTRAQ[0], Lysine(K)_iTRAQ[12]	[3] F6 and F9	1121/1113	0.826	1.030	0.950	1	Mascot
1659.8411	1659.9261	0.085	51	211	221	EGEHMINWVEK	53	99.989	(N-term)_iTRAQ[0], Lysine(K)_iTRAQ[11]	[3] F6 and F9	1225/1217	0.947	0.866	0.687	1	Mascot
1659.8411	1659.9321	0.091	55	211	221	EGEHMINWVEK	66	99.999	(N-term)_iTRAQ[0], Lysine(K)_iTRAQ[11]	[4] F7 and F10+11	1160/1152	0.842	1.209	1.056	1	Mascot
2129.1931	2129.1206	-0.0725	-34	56	70	LGLIPEEFFQFLYPK	32	98.284	(N-term)_iTRAQ[0], Lysine(K)_iTRAQ[15]	[1] F3 030912	580/572	1.162	0.945	0.275	1	Mascot
2245.2087	2245.1138	-0.0949	-42	116	131	YGASIGEFIDKLNEEK	56	99.993	(N-term)_iTRAQ[0], Lysine(K)_iTRAQ[11, 16]	[6] F8 110912	444/436	1.118	1.707	1.338	1	Mascot
2245.2087	2245.2141	0.0054	2	116	131	YGASIGEFIDKLNEEK	62	99.998	(N-term)_iTRAQ[0], Lysine(K)_iTRAQ[11, 16]	[4] F7 and F10+11	446/438	0.885	1.049	1.115	1	Mascot

170	RecName: Full=Beta-adducin; AltName: Full=Adducin-63; AltName: Full=Erythrocyte adducin subunit bet	gi|10720378	89314	6	381	0.716	0.827	0.771	0.118	0.184	0.149	6	6	6	100

Peptide Information

Calc. Mass	Obsrv. Mass	± da	± ppm	Start Seq.	End Seq.	Sequence	Ion Score	C. I. %	Modification	Plate [#] Name	Gel Idx/Pos [4700 Sample Name]	iTRAQ Ratio 115/114*	iTRAQ Ratio 116/114*	iTRAQ Ratio 117/114*	Rank	Result Type
1440.7402	1440.7079	-0.0323	-22	43	51	QDFNLMEQK	44	99.909	(N-term)_iTRAQ[0], Lysine(K)_iTRAQ[9]	[7] F5 120912	191/183	0.814	1.087	0.928	1	Mascot
1501.826	1501.8651	0.0391	26	164	173	EQDHFLISPK	66	99.999	(N-term)_iTRAQ[0], Lysine(K)_iTRAQ[10]	[3] F6 and F9	1190/1182	0.764	0.984	0.692	1	Mascot
1714.8945	1714.8113	-0.0832	-49	174	187	GVSCSEVTASSLIK	37	99.536	(N-term)_iTRAQ[0], Lysine(K)_iTRAQ[14], MMTS (C)[4]	[5] F4	236/228	0.668	0.583	0.616	1	Mascot
2045.9332	2045.7894	-0.1438	-70	664	680	GPGQMTTNADTDGDSYK	100	100	(N-term)_iTRAQ[0], Lysine(K)_iTRAQ[17]	[5] F4	95/87	0.863	0.784	0.805	1	Mascot
2169.0896	2169.054	-0.0356	-16	289	305	NHGMVALGDTVEEAFYK	88	100	(N-term)_iTRAQ[0], Lysine(K)_iTRAQ[17]	[4] F7 and F10+11	393/385	0.541	0.750	0.997	1	Mascot
2433.1575	2433.0046	-0.1529	-63	664	682	GPGQMTTNADTDGDSYKDK	46	99.941	(N-term)_iTRAQ[0], Lysine(K)_iTRAQ[17, 19]	[6] F8 110912	148/140	0.695	0.873	0.661	1	Mascot

171	cytochrome b-c1 complex subunit 2, mitochondrial precursor [Rattus norvegicus]	gi|55741544	52447	6	379	0.898	0.994	1.120	0.261	0.278	0.148	6	6	6	100

Peptide Information

Calc. Mass	Obsrv. Mass	± da	± ppm	Start Seq.	End Seq.	Sequence	Ion Score	C. I. %	Modification	Plate [#] Name	Gel Idx/Pos [4700 Sample Name]	iTRAQ Ratio 115/114*	iTRAQ Ratio 116/114*	iTRAQ Ratio 117/114*	Rank	Result Type
988.5699	988.5148	-0.0551	-56	148	154	WEVAALR	29	96.715	(N-term)_iTRAQ[0]	[5] F4	192/184	0.584	0.604	0.893	1	Mascot
1362.7501	1362.7007	-0.0494	-36	231	240	EVAEQFLNIR	61	99.998	(N-term)_iTRAQ[0]	[1] F3 030912	320/312	0.835	1.001	1.058	1	Mascot
1817.9967	1817.9021	-0.0946	-52	359	374	AVAQGNLSSADVQAAK	95	100	(N-term)_iTRAQ[0], Lysine(K)_iTRAQ[16]	[5] F4	112/104	0.953	0.955	1.096	1	Mascot
1886.952	1886.9396	-0.0124	-7	70	83	YENYNYLGTSHLLR	54	99.989	(N-term)_iTRAQ[0]	[4] F7 and F10+11	318/310	1.196	1.250	1.157	1	Mascot
2187.1907	2187.0962	-0.0945	-43	24	41	TSAPGGVPLQPQELEFTK	81	100	(N-term)_iTRAQ[0], Lysine(K)_iTRAQ[18]	[1] F3 030912	316/308	1.219	1.267	1.279	1	Mascot
2323.1592	2323.2637	0.1045	45	199	216	ITSEELHYFVQNHFTSAR	60	99.997	(N-term)_iTRAQ[0]	[4] F7 and F10+11	1265/1257	0.773	1.057	1.286	1	Mascot

172	B-36 VDAC=36 kda voltage dependent anion channel [rats, hippocampus, Peptide, 295 aa]	gi|299036	35663.9	5	379	0.959	1.039	1.213	0.280	0.245	0.439	5	5	5	100

Protein Group

voltage-dependent anion-selective channel protein 2 [Rattus norvegicus]	gi|13786202	35689.9

Peptide Information

Calc. Mass	Obsrv. Mass	± da	± ppm	Start Seq.	End Seq.	Sequence	Ion Score	C. I. %	Modification	Plate [#] Name	Gel Idx/Pos [4700 Sample Name]	iTRAQ Ratio 115/114*	iTRAQ Ratio 116/114*	iTRAQ Ratio 117/114*	Rank	Result Type
1112.6714	1112.6371	-0.0343	-31	33	40	GFGFGLVK	46	99.942	(N-term)_iTRAQ[0], Lysine(K)_iTRAQ[8]	[7] F5 120912	289/281	0.610	0.847	0.742	1	Mascot
1581.8734	1581.799	-0.0744	-47	237	248	YQLDPTASISAK	94	100	(N-term)_iTRAQ[0], Lysine(K)_iTRAQ[12]	[5] F4	158/150	1.290	1.222	1.484	1	Mascot
1716.9054	1716.8417	-0.0637	-37	109	121	LTFDTTFSPNTGK	64	99.999	(N-term)_iTRAQ[0], Lysine(K)_iTRAQ[13]	[5] F4	212/204	0.939	0.762	0.957	1	Mascot
2184.9617	2184.8027	-0.159	-73	47	65	SCSGVEFSTSGSSNTDT	90	100	(N-term)_iTRAQ[0],	[5] F4	133/125	1.153	1.233	1.565	1	Mascot

Calc. Mass	Obsrv. Mass	± da	± ppm	Start Seq.	End Seq.	Sequence	Ion Score	C. I. %	Modification	Plate [#] Name	Gel Idx/Pos [4700 Sample Name]	iTRAQ Ratio 115/114*	iTRAQ Ratio 116/114*	iTRAQ Ratio 117/114*	Rank	Result Type
						GK			Lysine(K)_iTRAQ[19], MMTS (C)[2]							
2796.3774	2796.3059	-0.0715	-26	87	108	WNTDNTLGTEIAIEDQIC QGLK	85	100	(N-term)_iTRAQ[0], Lysine(K)_iTRAQ[22], MMTS (C)[18]	[1] F3 030912	529/521	0.952	1.242	1.589	1	Mascot

173	high molecular-weight neurofilament [Rattus norvegicus]	gi|2642598	136692.3	8	377	1.977	0.839	0.726	0.747	0.217	0.272	8	8	8	100

Peptide Information

Calc. Mass	Obsrv. Mass	± da	± ppm	Start Seq.	End Seq.	Sequence	Ion Score	C. I. %	Modification	Plate [#] Name	Gel Idx/Pos [4700 Sample Name]	iTRAQ Ratio 115/114*	iTRAQ Ratio 116/114*	iTRAQ Ratio 117/114*	Rank	Result Type
1101.619	1101.5853	-0.0337	-31	105	111	FAGYIDK	28	96.237	(N-term)_iTRAQ[0], Lysine(K)_iTRAQ[7]	[3] F6 and F9	229/221	1.825	0.681	0.610	1	Mascot
1183.5901	1183.5057	-0.0844	-71	136	144	AAMGELYER	32	98.417	(N-term)_iTRAQ[0]	[5] F4	128/120	0.918	0.547	0.348	1	Mascot
1409.7886	1409.7469	-0.0417	-30	380	388	EYQDLLNVK	60	99.997	(N-term)_iTRAQ[0], Lysine(K)_iTRAQ[9]	[7] F5 120912	243/235	2.284	1.143	1.029	1	Mascot
1512.9121	1512.9808	0.0687	45	1032	1041	GLPQEPSKPK	31	98.195	(N-term)_iTRAQ[0], Lysine(K)_iTRAQ[8,10]	[4] F7 and F10+11	1035/1027	1.931	0.891	0.918	1	Mascot
1551.7424	1551.7681	0.0257	17	39	52	SAAGSSSGFHSWAR	58	99.996	(N-term)_iTRAQ[0]	[3] F6 and F9	1144/1136	2.098	1.120	0.828	1	Mascot
1718.9282	1718.9795	0.0513	30	93	104	SEKEQLQALNDR	35	99.299	(N-term)_iTRAQ[0], Lysine(K)_iTRAQ[3]	[4] F7 and F10+11	1066/1058	2.986	0.768	0.733	1	Mascot
1845.9943	1846.0576	0.0633	34	165	178	LEQEHLLEDIAHVR	93	100	(N-term)_iTRAQ[0]	[4] F7 and F10+11	1246/1238	2.237	0.904	0.708	1	Mascot
1975.1572	1975.0532	-0.104	-53	924	935	AEEKEPLTEKPK	43	99.882	(N-term)_iTRAQ[0], Lysine(K)_iTRAQ[4,10,12]	[2] F12 040912	130/122	2.249	0.832	0.896	1	Mascot

174	V-type proton ATPase subunit C 1 [Rattus norvegicus]	gi|58865560	49630.9	8	375	0.896	1.039	0.815	0.302	0.201	0.570	9	9	9	100

Peptide Information

Calc. Mass	Obsrv. Mass	± da	± ppm	Start Seq.	End Seq.	Sequence	Ion Score	C. I. %	Modification	Plate [#] Name	Gel Idx/Pos [4700 Sample Name]	iTRAQ Ratio 115/114*	iTRAQ Ratio 116/114*	iTRAQ Ratio 117/114*	Rank	Result Type
1213.6285	1213.6519	0.0234	19	105	111	FQWDMAK	30	97.42	(N-term)_iTRAQ[0], Lysine(K)_iTRAQ[7]	[3] F6 and F9	287/279	0.631	0.889	0.972	1	Mascot
1327.7368	1327.8136	0.0768	58	192	199	LNHNDWIK	37	99.489	(N-term)_iTRAQ[0], Lysine(K)_iTRAQ[8]	[4] F7 and F10+11	1106/1098	0.995	0.965	0.782	1	Mascot
1357.6471	1357.59	-0.0571	-42	14	21	TCQQTWEK	34	98.947	(N-term)_iTRAQ[0], Lysine(K)_iTRAQ[8], MMTS (C)[2]	[3] F6 and F9	196/188	0.915	1.166	1.147	1	Mascot
1390.7788	1390.7037	-0.0751	-54	128	137	GVTQIDNDLK	58	99.996	(N-term)_iTRAQ[0], Lysine(K)_iTRAQ[10]	[7] F5 120912	165/157	1.060	0.917	1.220	1	Mascot
1428.7457	1428.7412	-0.0045	-3	374	382	IDCNLLEFK	52	99.986	(N-term)_iTRAQ[0], Lysine(K)_iTRAQ[9], MMTS (C)[3]	[7] F5 120912	412/404	0.650	0.949	0.964	1	Mascot
1504.6498	1504.5508	-0.099	-66	250	259	DFQYNEEEMR	62	99.998	(N-term)_iTRAQ[0]	[5] F4	128/120	1.388	1.327	1.220	1	Mascot
1504.6498	1504.573	-0.0768	-51	250	259	DFQYNEEEMR	59	99.997	(N-term)_iTRAQ[0]	[1] F3 030912	201/193	0.788	1.089	1.005	1	Mascot
2135.1831	2135.1416	-0.0415	-19	87	104	VQENLLASGVDLVTYITR	37	99.473	(N-term)_iTRAQ[0]	[8] F13-15 and F1+2	243/235	1.351	1.391	0.205	1	Mascot
2144.1282	2144.0305	-0.0977	-46	72	86	VAQYMADVLEDSKDK	67	99.999	(N-term)_iTRAQ[0], Lysine(K)_iTRAQ[13,15]	[6] F8 110912	399/391	0.637	0.803	0.618	1	Mascot

175	septin-5 [Rattus norvegicus]	gi|90577179	48727.4	7	375	0.718	0.917	1.004	0.089	0.222	0.285	7	7	7	100

Protein Group

CDCrel-1AI [Rattus norvegicus]	gi|19909845	46577.3

Peptide Information

Calc. Mass	Obsrv. Mass	± da	± ppm	Start Seq.	End Seq.	Sequence	Ion Score	C. I. %	Modification	Plate [#] Name	Gel Idx/Pos [4700 Sample Name]	iTRAQ Ratio 115/114*	iTRAQ Ratio 116/114*	iTRAQ Ratio 117/114*	Rank	Result Type
1198.7279	1198.6877	-0.0402	-34	11	19	LVEQLLSPR	28	96.023	(N-term)_iTRAQ[0]	[1] F3 030912	335/327	0.726	1.170	1.326	1	Mascot
1480.7301	1480.7996	0.0695	47	318	327	AHCIQQMTSK	65	99.999	(N-term)_iTRAQ[0], Lysine(K)_iTRAQ[10], MMTS (C)[3]	[4] F7 and F10+11	1085/1077	0.651	1.109	0.900	1	Mascot
1703.7258	1703.6991	-0.0267	-16	306	317	DVTCDVHYENYR	39	99.714	(N-term)_iTRAQ[0], MMTS (C)[4]	[4] F7 and F10+11	243/235	0.766	0.888	0.659	1	Mascot
1773.952	1773.9346	-0.0174	-10	32	45	QYVGFATLPNQVHR	62	99.998	(N-term)_iTRAQ[0]	[3] F6 and F9	275/267	0.731	0.771	1.024	1	Mascot
1925.0994	1925.1102	0.0108	6	67	80	STLVHSLFLTDLYK	82	100	(N-term)_iTRAQ[0], Lysine(K)_iTRAQ[14]	[4] F7 and F10+11	432/424	0.613	0.907	0.869	1	Mascot
2007.1008	2007.0211	-0.0797	-40	249	265	ESAPFAVIGSNTVVEAK	62	99.998	(N-term)_iTRAQ[0], Lysine(K)_iTRAQ[17]	[1] F3 030912	308/300	0.672	0.610	0.993	1	Mascot
2196.2275	2196.2908	0.0633	29	67	82	STLVHSLFLTDLYKDR	37	99.515	(N-term)_iTRAQ[0], Lysine(K)_iTRAQ[14]	[4] F7 and F10+11	1283/1275	0.903	1.110	1.477	1	Mascot

176	protein kinase C type II [Rattus norvegicus]	gi|206189	85925.6	8	373	1.064	0.942	0.781	0.204	0.227	0.383	8	8	8	100

Protein Group

RecName: Full=Protein kinase C beta type; Short=PKC-B; Short=PKC-beta	gi|55977078	86266.9
protein kinase C (EC 2.7.1.-) beta-II - rat	gi|66724	86343.8
protein kinase C beta-1 [Rattus norvegicus]	gi|206175	86364.9
unnamed protein product [Rattus norvegicus]	gi|56959	86298.9

Peptide Information

Calc. Mass	Obsrv. Mass	± da	± ppm	Start Seq.	End Seq.	Sequence	Ion Score	C. I. %	Modification	Plate [#] Name	Gel Idx/Pos [4700 Sample Name]	iTRAQ Ratio 115/114*	iTRAQ Ratio 116/114*	iTRAQ Ratio 117/114*	Rank	Result Type
1167.6409	1167.6414	0.0005	0	269	276	AGVDGWFK	46	99.939	(N-term)_iTRAQ[0], Lysine(K)_iTRAQ[8]	[4] F7 and F10+11	279/271	1.060	1.029	0.349	1	Mascot
1450.7788	1450.7539	-0.0249	-17	490	499	ENIWDGVTTK	64	99.999	(N-term)_iTRAQ[0], Lysine(K)_iTRAQ[10]	[7] F5 120912	212/204	0.924	0.837	0.847	1	Mascot
1555.9067	1555.9735	0.0668	43	362	371	KGTDELYAVK	28	95.921	(N-term)_iTRAQ[0], Lysine(K)_iTRAQ[1,10]	[4] F7 and F10+11	1077/1069	0.918	1.003	0.772	1	Mascot
1685.7517	1685.7072	-0.0445	-26	217	228	CSLNPEWNETFR	40	99.76	(N-term)_iTRAQ[0], MMTS (C)[1]	[1] F3 030912	354/346	0.990	0.802	0.868	1	Mascot
1685.9546	1685.8931	-0.0615	-36	339	350	LTDFNFLMVLGK	48	99.959	(N-term)_iTRAQ[0], Lysine(K)_iTRAQ[12]	[5] F4	406/398	1.390	1.143	1.406	1	Mascot
1758.9305	1758.9281	-0.0024	-1	469	481	LDNVMLDSEGHIK	34	99.01	(N-term)_iTRAQ[0], Lysine(K)_iTRAQ[13]	[4] F7 and F10+11	275/267	1.466	1.395	0.523	1	Mascot
1763.9088	1763.8513	-0.0575	-33	240	252	LSVEIWDWDLTSR	46	99.94	(N-term)_iTRAQ[0]	[1] F3 030912	482/474	0.949	0.669	1.004	1	Mascot
2259.239	2259.2986	0.0596	26	466	481	DLKLDNVMLDSEGHIK	70	100	(N-term)_iTRAQ[0], Lysine(K)_iTRAQ[3,16]	[4] F7 and F10+11	1200/1192	0.952	0.842	0.947	1	Mascot

177	V-type proton ATPase subunit E 1 [Rattus norvegicus]	gi|38454230	29760.4	7	371	0.905	1.161	0.969	0.224	0.199	0.157	9	9	9	100

Peptide Information

Calc. Mass	Obsrv. Mass	± da	± ppm	Start Seq.	End Seq.	Sequence	Ion Score	C. I. %	Modification	Plate [#] Name	Gel Idx/Pos [4700 Sample Name]	iTRAQ Ratio 115/114*	iTRAQ Ratio 116/114*	iTRAQ Ratio 117/114*	Rank	Result Type
1263.6541	1263.6073	-0.0468	-37	53	59	IMEYYEK	29	97.178	(N-term)_iTRAQ[0], Lysine(K)_iTRAQ[7]	[7] F5 120912	193/185	1.176	1.332	0.827	1	Mascot
1263.6541	1263.6243	-0.0298	-24	53	59	IMEYYEK	38	99.621	(N-term)_iTRAQ[0], Lysine(K)_iTRAQ[7]	[3] F6 and F9	232/224	0.977	1.311	0.941	1	Mascot
1396.7206	1396.6531	-0.0675	-48	34	42	AEEEFNIEK	48	99.959	(N-term)_iTRAQ[0], Lysine(K)_iTRAQ[9]	[7] F5 120912	172/164	0.878	1.147	1.048	1	Mascot
1396.7206	1396.6687	-0.0519	-37	34	42	AEEEFNIEK	58	99.996	(N-term)_iTRAQ[0], Lysine(K)_iTRAQ[9]	[3] F6 and F9	212/204	1.429	1.536	1.347	1	Mascot
1406.8743	1406.9495	0.0752	53	138	145	KQDFPLVK	32	98.391	(N-term)_iTRAQ[0], Lysine(K)_iTRAQ[1,8]	[4] F7 and F10+11	1104/1096	0.714	0.932	0.898	1	Mascot
1449.7427	1449.6643	-0.0784	-54	70	80	IQMSNLMNQAR	35	99.23	(N-term)_iTRAQ[0]	[5] F4	192/184	0.744	0.917	0.786	1	Mascot
1865.9136	1865.9812	0.0676	36	14	26	HMMAFIEQEANEK	67	99.999	(N-term)_iTRAQ[0], Lysine(K)_iTRAQ[13]	[3] F6 and F9	1277/1269	0.770	1.199	0.917	1	Mascot
1875.0433	1874.9589	-0.0844	-45	86	99	ARDDLITDLLNEAK	98	100	(N-term)_iTRAQ[0], Lysine(K)_iTRAQ[14]	[6] F8 110912	425/417	0.918	1.061	1.070	1	Mascot
2766.3813	2766.4709	0.0896	32	14	33	HMMAFIEQEANEKAEEIDAK	44	99.904	(N-term)_iTRAQ[0], Lysine(K)_iTRAQ[13,20]	[4] F7 and F10+11	1288/1280	0.754	1.146	0.991	1	Mascot

178	tenascin-R precursor [Rattus norvegicus]	gi|6981668	156926	7	369	1.248	1.183	0.938	0.640	0.413	0.241	8	8	8	100

Peptide Information

Calc. Mass	Obsrv. Mass	± da	± ppm	Start Seq.	End Seq.	Sequence	Ion Score	C. I. %	Modification	Plate [#] Name	Gel Idx/Pos [4700 Sample Name]	iTRAQ Ratio 115/114*	iTRAQ Ratio 116/114*	iTRAQ Ratio 117/114*	Rank	Result Type
1098.5983	1098.5168	-0.0815	-74	1294	1299	GAWWYK	29	97.031	(N-term)_iTRAQ[0], Lysine(K)_iTRAQ[6]	[6] F8 110912	282/274	1.893	1.657	1.423	1	Mascot
1572.8883	1572.8174	-0.0709	-45	1064	1074	AAIENYVLTYK	41	99.819	(N-term)_iTRAQ[0], Lysine(K)_iTRAQ[11]	[5] F4	250/242	1.314	1.162	1.027	1	Mascot
1603.8452	1603.8016	-0.0436	-27	1082	1093	ELIVDAEDTWIR	46	99.943	(N-term)_iTRAQ[0]	[1] F3 030912	399/391	0.671	0.930	0.677	1	Mascot
1693.8319	1693.7277	-0.1042	-62	1230	1241	DGQEAVFAYYDK	65	99.999	(N-term)_iTRAQ[0], Lysine(K)_iTRAQ[12]	[5] F4	186/178	2.044	1.164	0.969	1	Mascot
1693.8319	1693.7599	-0.072	-43	1230	1241	DGQEAVFAYYDK	70	100	(N-term)_iTRAQ[0], Lysine(K)_iTRAQ[12]	[7] F5 120912	233/225	1.461	1.398	0.989	1	Mascot
1708.8978	1708.8241	-0.0737	-43	1326	1337	GHEFSIPFVEMK	75	100	(N-term)_iTRAQ[0], Lysine(K)_iTRAQ[12]	[6] F8 110912	382/374	0.895	0.945	0.965	1	Mascot

Calc. Mass	Obsrv. Mass	± da	± ppm	Start Seq.	End Seq.	Sequence	Ion Score	C. I. %	Modification	Plate [#] Name	Gel Idx/Pos [4700 Sample Name]	iTRAQ Ratio 115/114*	iTRAQ Ratio 116/114*	iTRAQ Ratio 117/114*	Rank	Result Type
1743.8965	1743.9536	0.0571	33	1315	1325	HSQGINWYHWK	27	95.496	(N-term)_iTRAQ[0], Lysine(K)_iTRAQ[11]	[8] F13-15 and F1+2	1151/1143	0.727	0.727	0.995	1	Mascot
2588.2249	2588.1477	-0.0772	-30	605	624	TATSLDLEWDNSEAEAQ EYK	79	100	(N-term)_iTRAQ[0], Lysine(K)_iTRAQ[20]	[1] F3 030912	322/314	1.812	1.910	0.661	1	Mascot

179	V-type proton ATPase subunit H [Rattus norvegicus]	gi|62078587	55275.4	6	367	1.190	1.089	1.009	0.698	0.238	0.701	7	7	7	100

Peptide Information

Calc. Mass	Obsrv. Mass	± da	± ppm	Start Seq.	End Seq.	Sequence	Ion Score	C. I. %	Modification	Plate [#] Name	Gel Idx/Pos [4700 Sample Name]	iTRAQ Ratio 115/114*	iTRAQ Ratio 116/114*	iTRAQ Ratio 117/114*	Rank	Result Type
1131.6984	1131.6696	-0.0288	-25	385	392	VIEQLGGK	48	99.963	(N-term)_iTRAQ[0], Lysine(K)_iTRAQ[8]	[7] F5 120912	162/154	1.195	0.979	0.899	1	Mascot
1677.9032	1677.9376	0.0344	21	417	427	LMVHNWEYLGK	83	100	(N-term)_iTRAQ[0], Lysine(K)_iTRAQ[11]	[3] F6 and F9	1281/1273	0.692	0.823	0.844	1	Mascot
1677.9032	1677.9861	0.0829	49	417	427	LMVHNWEYLGK	51	99.979	(N-term)_iTRAQ[0], Lysine(K)_iTRAQ[11]	[4] F7 and F10+11	1218/1210	1.769	1.168	1.371	1	Mascot
1739.9064	1739.8563	-0.0501	-29	120	132	STAWPYFLPMLNR	33	98.722	(N-term)_iTRAQ[0]	[1] F3 030912	495/487	0.809	0.941	0.320	1	Mascot
1786.8364	1786.7162	-0.1202	-67	63	75	QEMLQTEGSQCAK	62	99.999	(N-term)_iTRAQ[0], Lysine(K)_iTRAQ[13], MMTS (C)[11]	[5] F4	127/119	1.546	1.063	1.441	1	Mascot
2119.2261	2119.1821	-0.044	-21	263	278	YNIIPVLSDILQESVK	85	100	(N-term)_iTRAQ[0], Lysine(K)_iTRAQ[16]	[1] F3 030912	563/555	0.724	1.204	1.250	1	Mascot
2639.3801	2639.293	-0.0871	-33	355	377	LLEVSDDPQVLAVAAHDV GEYVR	56	99.993	(N-term)_iTRAQ[0]	[1] F3 030912	498/490	2.544	1.597	1.776	1	Mascot

180	Chain A, G Protein Heterotrimer Mutant Gi_alpha_1(G203a) Beta_1 Gamma_2 With Gdp Bound	gi|1942391	45129.2	6	361	0.991	1.212	1.112	0.323	0.342	0.282	7	7	7	100

Protein Group

Chain A, G Protein Heterotrimer Gi_alpha_1 Beta_1 Gamma_2 With Gdp Bound	gi|1942397	45115.2
guanine nucleotide-binding protein G(i) subunit alpha-1 [Rattus norvegicus]	gi|6980962	45246.3

Peptide Information

Calc. Mass	Obsrv. Mass	± da	± ppm	Start Seq.	End Seq.	Sequence	Ion Score	C. I. %	Modification	Plate [#] Name	Gel Idx/Pos [4700 Sample Name]	iTRAQ Ratio 115/114*	iTRAQ Ratio 116/114*	iTRAQ Ratio 117/114*	Rank	Result Type
1345.8301	1345.7644	-0.0657	-49	35	45	LLLLGAGESGK	84	100	(N-term)_iTRAQ[0], Lysine(K)_iTRAQ[11]	[5] F4	219/211	1.116	1.118	1.283	1	Mascot
1387.6941	1387.6503	-0.0438	-32	248	256	LFDSICNNK	36	99.432	(N-term)_iTRAQ[0], Lysine(K)_iTRAQ[9], MMTS (C)[6]	[3] F6 and F9	362/354	0.880	0.840	1.463	1	Mascot
1668.9207	1668.8728	-0.0479	-29	180	191	TTGIVETHFTFK	82	100	(N-term)_iTRAQ[0], Lysine(K)_iTRAQ[12]	[4] F7 and F10+11	286/278	1.242	1.237	1.046	1	Mascot
1841.864	1841.7537	-0.1103	-60	54	66	IIHEAGYSEEECK	72	100	(N-term)_iTRAQ[0], Lysine(K)_iTRAQ[13], MMTS (C)[12]	[6] F8 110912	229/221	1.525	1.889	1.376	1	Mascot
1863.8848	1863.9038	0.019	10	317	329	EIYTHFTCATDTK	53	99.988	(N-term)_iTRAQ[0], Lysine(K)_iTRAQ[13], MMTS (C)[8]	[4] F7 and F10+11	280/272	1.077	1.009	0.706	1	Mascot
1900.0413	1900.0767	0.0354	19	161	175	IAQPNYIPTQQDVLR	29	96.848	(N-term)_iTRAQ[0]	[8] F13-15 and F1+2	535/527	0.731	1.534	1.048	1	Mascot
1900.0413	1900.0839	0.0426	22	161	175	IAQPNYIPTQQDVLR	35	99.134	(N-term)_iTRAQ[0]	[8] F13-15 and F1+2	486/478	0.640	1.129	1.050	1	Mascot

181	synaptic ras GTPase-activating protein p135 SynGAP [Rattus norvegicus]	gi|2935448	153316.7	7	359	0.615	0.833	1.059	0.096	0.337	0.301	7	7	7	100

Protein Group

GTPase activating protein SynGAP-c [Rattus norvegicus]	gi|10140855	137646.4
SynGAP-a [Rattus norvegicus]	gi|10122138	152273.2
SynGAP-b [Rattus norvegicus]	gi|3722229	147512.6

Peptide Information

Calc. Mass	Obsrv. Mass	± da	± ppm	Start Seq.	End Seq.	Sequence	Ion Score	C. I. %	Modification	Plate [#] Name	Gel Idx/Pos [4700 Sample Name]	iTRAQ Ratio 115/114*	iTRAQ Ratio 116/114*	iTRAQ Ratio 117/114*	Rank	Result Type
1501.6721	1501.592	-0.0801	-53	735	746	GPSAEMQGYMMR	27	95.306	(N-term)_iTRAQ[0]	[1] F3 030912	240/232	0.681	0.924	1.202	1	Mascot
1552.8733	1552.9342	0.0609	39	549	558	ELKEVFASWR	35	99.304	(N-term)_iTRAQ[0], Lysine(K)_iTRAQ[3]	[4] F7 and F10+11	1255/1247	0.576	1.255	1.799	1	Mascot
1719.8986	1719.882	-0.0166	-10	1261	1273	DHPAMAEPLPEPK	44	99.909	(N-term)_iTRAQ[0], Lysine(K)_iTRAQ[13]	[4] F7 and F10+11	198/190	0.792	0.664	1.017	1	Mascot

Calc. Mass	Obsrv. Mass	± da	± ppm	Start Seq.	End Seq.	Sequence	Ion Score	C. I. %	Modification	Plate [#] Name	Gel Idx/Pos [4700 Sample Name]	iTRAQ Ratio 115/114*	iTRAQ Ratio 116/114*	iTRAQ Ratio 117/114*	Rank	Result Type
1936.0763	1936.0193	-0.057	-29	1180	1191	VKEYEEEIHSLK	59	99.997	(N-term)_iTRAQ[0], Lysine(K)_iTRAQ[2,12]	[2] F12 040912	299/291	0.569	0.858	0.985	1	Mascot
2002.9824	2002.999	0.0166	8	446	460	AKDFLSDMAMSEVDR	27	95.434	(N-term)_iTRAQ[0], Lysine(K)_iTRAQ[2]	[4] F7 and F10+11	365/357	0.515	0.439	0.935	1	Mascot
2072.9746	2072.9072	-0.0674	-33	501	515	ALYESEENCEVDPIK	44	99.912	(N-term)_iTRAQ[0], Lysine(K)_iTRAQ[15], MMTS (C)[9]	[1] F3 030912	302/294	0.684	1.245	0.757	1	Mascot
2299.3357	2299.4246	0.0889	39	428	445	GKEEVASALVHILQSTGK	121	100	(N-term)_iTRAQ[0], Lysine(K)_iTRAQ[2,18]	[4] F7 and F10+11	1318/1310	0.534	0.772	0.974	1	Mascot

182	myosin-9 [Rattus norvegicus]	gi|6981236	256986.2	8	357	1.177	0.975	0.856	0.330	0.219	0.276	8	8	8	100

Peptide Information

Calc. Mass	Obsrv. Mass	± da	± ppm	Start Seq.	End Seq.	Sequence	Ion Score	C. I. %	Modification	Plate [#] Name	Gel Idx/Pos [4700 Sample Name]	iTRAQ Ratio 115/114*	iTRAQ Ratio 116/114*	iTRAQ Ratio 117/114*	Rank	Result Type
1180.6282	1180.604	-0.0242	-20	581	587	ADEWLMK	28	96.193	(N-term)_iTRAQ[0], Lysine(K)_iTRAQ[7]	[3] F6 and F9	269/261	1.158	1.092	0.788	1	Mascot
1394.6229	1394.5562	-0.0667	-48	1594	1603	EMEAELEDER	38	99.572	(N-term)_iTRAQ[0]	[1] F3 030912	196/188	1.286	0.951	0.870	1	Mascot
1462.8502	1462.8566	0.0064	4	683	693	LDPHLVLDQLR	39	99.693	(N-term)_iTRAQ[0]	[4] F7 and F10+11	364/356	0.987	1.004	0.817	1	Mascot
1475.7098	1475.6353	-0.0745	-50	1879	1889	QLEEAEEEAQR	39	99.685	(N-term)_iTRAQ[0]	[1] F3 030912	172/164	0.888	0.661	0.674	1	Mascot
1571.7941	1571.8258	0.0317	20	566	576	ADFCIIHYAGK	54	99.99	(N-term)_iTRAQ[0], Lysine(K)_iTRAQ[11], MMTS (C)[4]	[3] F6 and F9	1292/1284	0.869	0.772	0.591	1	Mascot
1652.9595	1653.0321	0.0726	44	1446	1455	KFDQLLAEEK	53	99.987	(N-term)_iTRAQ[0], Lysine(K)_iTRAQ[1,10]	[4] F7 and F10+11	1163/1155	1.200	1.089	0.894	1	Mascot
2047.0179	2047.0944	0.0765	37	910	923	KQELEEICHDLEAR	50	99.978	(N-term)_iTRAQ[0], Lysine(K)_iTRAQ[1], MMTS (C)[8]	[4] F7 and F10+11	1303/1295	1.935	1.309	1.631	1	Mascot
2094.0952	2094.114	0.0188	9	1419	1434	LQQELDDLLVDLDHQR	60	99.998	(N-term)_iTRAQ[0]	[3] F6 and F9	533/525	1.400	1.074	0.889	1	Mascot

183	calcium/calmodulin-dependent protein kinase type II subunit delta [Rattus norvegicus]	gi|6978595	66312.5	8	357	1.164	1.235	0.986	0.115	0.180	0.376	8	8	8	100

Peptide Information

Calc. Mass	Obsrv. Mass	± da	± ppm	Start Seq.	End Seq.	Sequence	Ion Score	C. I. %	Modification	Plate [#] Name	Gel Idx/Pos [4700 Sample Name]	iTRAQ Ratio 115/114*	iTRAQ Ratio 116/114*	iTRAQ Ratio 117/114*	Rank	Result Type
1080.6664	1080.6154	-0.051	-47	222	227	LYQQIK	33	98.745	(N-term)_iTRAQ[0], Lysine(K)_iTRAQ[6]	[3] F6 and F9	190/182	1.043	1.183	1.117	1	Mascot
1269.641	1269.5786	-0.0624	-49	285	292	QETVDCLK	49	99.972	(N-term)_iTRAQ[0], Lysine(K)_iTRAQ[8], MMTS (C)[6]	[7] F5 120912	174/166	1.121	1.135	1.229	1	Mascot
1291.7528	1291.7052	-0.0476	-37	302	312	GAILTTMLATR	39	99.692	(N-term)_iTRAQ[0]	[1] F3 030912	340/332	1.080	1.221	0.835	1	Mascot
1365.7625	1365.7091	-0.0534	-39	34	43	IPTGQEYAAK	57	99.995	(N-term)_iTRAQ[0], Lysine(K)_iTRAQ[10]	[7] F5 120912	144/136	1.269	1.501	1.314	1	Mascot
1541.8384	1541.9143	0.0759	49	285	293	QETVDCLKK	33	98.745	(N-term)_iTRAQ[0], Lysine(K)_iTRAQ[8,9], MMTS (C)[6]	[4] F7 and F10+11	1090/1082	1.407	1.208	1.100	1	Mascot
1773.0857	1772.9882	-0.0975	-55	136	147	DLKPENLLLASK	49	99.967	(N-term)_iTRAQ[0], Lysine(K)_iTRAQ[3,12]	[6] F8 110912	295/287	1.190	1.424	1.239	1	Mascot
2369.1548	2369.0718	-0.083	-35	228	246	AGAYDFPSPEWDTVTPEAK	68	100	(N-term)_iTRAQ[0], Lysine(K)_iTRAQ[19]	[1] F3 030912	337/329	1.184	1.355	0.954	1	Mascot
2921.3735	2921.3066	-0.0669	-23	406	430	ICDPGLTAFEPEALGNLVEGMDFHR	30	97.39	(N-term)_iTRAQ[0], MMTS (C)[2]	[1] F3 030912	544/536	1.063	0.945	0.455	1	Mascot

184	guanine nucleotide-binding protein G(I)/G(S)/G(T) subunit beta-2 [Mus musculus]	gi|13937391	39201.8	5	357	1.228	1.016	1.178	0.268	0.275	0.133	6	6	6	100

Peptide Information

Calc. Mass	Obsrv. Mass	± da	± ppm	Start Seq.	End Seq.	Sequence	Ion Score	C. I. %	Modification	Plate [#] Name	Gel Idx/Pos [4700 Sample Name]	iTRAQ Ratio 115/114*	iTRAQ Ratio 116/114*	iTRAQ Ratio 117/114*	Rank	Result Type
1305.7625	1305.7292	-0.0333	-26	69	78	LLVSASQDGK	79	100	(N-term)_iTRAQ[0], Lysine(K)_iTRAQ[10]	[7] F5 120912	155/147	0.908	0.682	1.078	1	Mascot
1480.7128	1480.8044	0.0916	62	58	68	IYAMHWGTDSR	55	99.991	(N-term)_iTRAQ[0]	[3] F6 and F9	1225/1217	1.100	0.899	1.232	1	Mascot
1641.9098	1641.8633	-0.0465	-28	79	89	LIIWDSYTTNK	47	99.948	(N-term)_iTRAQ[0], Lysine(K)_iTRAQ[11]	[5] F4	266/258	1.600	1.097	1.259	1	Mascot
1641.9098	1641.8676	-0.0422	-26	79	89	LIIWDSYTTNK	58	99.996	(N-term)_iTRAQ[0], Lysine(K)_iTRAQ[11]	[7] F5 120912	333/325	1.089	1.018	1.411	1	Mascot
1671.7197	1671.7158	-0.0039	-2	138	150	ELPGHTGYLSCCR	28	96.254	(N-term)_iTRAQ[0], MMTS (C)[11,12]	[4] F7 and F10+11	338/330	1.506	1.522	1.097	1	Mascot
2693.2852	2693.2241	-0.0611	-23	315	337	VSCLGVTDDGMAVATGSWDSFLK	137	100	(N-term)_iTRAQ[0], Lysine(K)_iTRAQ[23], MMTS (C)[3]	[1] F3 030912	529/521	1.309	1.058	1.033	1	Mascot

185	glutathione S-transferase Yb-3 [Rattus norvegicus]	gi|13592152	28107.6	7	356	0.896	1.163	1.314	0.187	0.327	0.280	7	7	7	100

Peptide Information

Calc. Mass	Obsrv. Mass	± da	± ppm	Start Seq.	End Seq.	Sequence	Ion Score	C. I. %	Modification	Plate [#] Name	Gel Idx/Pos [4700 Sample Name]	iTRAQ Ratio 115/114*	iTRAQ Ratio 116/114*	iTRAQ Ratio 117/114*	Rank	Result Type
1192.6572	1192.6332	-0.024	-20	44	50	SQWLNEK	39	99.7	(N-term)_iTRAQ[0], Lysine(K)_iTRAQ[7]	[4] F7 and F10+11	191/183	0.761	1.000	1.259	1	Mascot
1244.7136	1244.6593	-0.0543	-44	137	144	LYSEFLGK	30	97.581	(N-term)_iTRAQ[0], Lysine(K)_iTRAQ[8]	[7] F5 120912	287/279	0.779	0.885	1.093	1	Mascot
1264.7048	1264.6775	-0.0273	-22	145	152	RPWFAGDK	36	99.392	(N-term)_iTRAQ[0], Lysine(K)_iTRAQ[8]	[2] F12 040912	229/221	1.212	1.612	1.847	1	Mascot
1603.8234	1603.7704	-0.053	-33	97	108	VDILENQLMDNR	54	99.99	(N-term)_iTRAQ[0]	[1] F3 030912	322/314	0.928	0.906	1.062	1	Mascot
1844.9918	1844.9594	-0.0324	-18	153	166	ITFVDFIAYDVLER	58	99.996	(N-term)_iTRAQ[0]	[1] F3 030912	566/558	0.730	1.527	1.659	1	Mascot
1877.963	1877.8645	-0.0985	-52	19	31	LLLEYTDSSYEEK	72	100	(N-term)_iTRAQ[0], Lysine(K)_iTRAQ[13]	[5] F4	238/230	1.154	1.487	1.246	1	Mascot
2034.0641	2034.0514	-0.0127	-6	19	32	LLLEYTDSSYEEKR	69	100	(N-term)_iTRAQ[0], Lysine(K)_iTRAQ[13]	[4] F7 and F10+11	304/296	0.826	0.979	1.212	1	Mascot

186 succinate dehydrogenase [ubiquinone] flavoprotein subunit, mitochondrial precursor [Rattus norvegic — gi|18426858 — 76576.4 — 7 — 353 — *0.842* — *1.237* — *1.258* — 0.741 — 0.783 — 1.189 — 7 — 7 — 7 — 100

Peptide Information

Calc. Mass	Obsrv. Mass	± da	± ppm	Start Seq.	End Seq.	Sequence	Ion Score	C. I. %	Modification	Plate [#] Name	Gel Idx/Pos [4700 Sample Name]	iTRAQ Ratio 115/114*	iTRAQ Ratio 116/114*	iTRAQ Ratio 117/114*	Rank	Result Type
1383.7307	1383.7817	0.051	37	113	120	WHFYDTVK	43	99.871	(N-term)_iTRAQ[0], Lysine(K)_iTRAQ[8]	[4] F7 and F10+11	1176/1168	0.608	0.840	0.788	1	Mascot
1473.7781	1473.6908	-0.0873	-59	305	317	GEGGILINSQGER	64	99.999	(N-term)_iTRAQ[0]	[1] F3 030912	189/181	1.289	1.023	1.276	1	Mascot
1617.9448	1617.9127	-0.0321	-20	444	457	LGANSLLDLVVFGR	57	99.995	(N-term)_iTRAQ[0]	[1] F3 030912	546/538	1.605	2.105	1.314	1	Mascot
2035.0219	2034.9639	-0.058	-29	68	84	AAFGLSEAGFNTACLTK	54	99.99	(N-term)_iTRAQ[0], Lysine(K)_iTRAQ[17], MMTS (C)[14]	[1] F3 030912	401/393	0.633	0.858	1.020	1	Mascot
2245.1724	2245.0581	-0.1143	-51	593	607	IDEYDYSKPIEGQQK	67	100	(N-term)_iTRAQ[0], Lysine(K)_iTRAQ[8,15]	[6] F8 110912	214/206	0.894	0.937	0.833	1	Mascot
2311.2432	2311.178	-0.0652	-28	354	371	DHVYLQLHHLPPEQLATR	38	99.569	(N-term)_iTRAQ[0]	[2] F12 040912	302/294	0.247	0.939	0.750	1	Mascot
3073.5713	3073.45	-0.1213	-39	39	67	VSDAISTQYPVVDHEFDAVVVGAGGAGLR	32	98.611	(N-term)_iTRAQ[0]	[1] F3 030912	430/422	*1.704*	*3.241*	*5.915*	1	Mascot

187 actin-related protein 2 [Rattus norvegicus] — gi|57164143 — 48681.8 — 6 — 351 — 1.055 — 1.288 — 0.965 — 0.470 — 0.683 — 0.338 — 6 — 6 — 6 — 100

Peptide Information

Calc. Mass	Obsrv. Mass	± da	± ppm	Start Seq.	End Seq.	Sequence	Ion Score	C. I. %	Modification	Plate [#] Name	Gel Idx/Pos [4700 Sample Name]	iTRAQ Ratio 115/114*	iTRAQ Ratio 116/114*	iTRAQ Ratio 117/114*	Rank	Result Type
1478.728	1478.7535	0.0255	17	54	65	DLMVGDEASELR	72	100	(N-term)_iTRAQ[0]	[8] F13-15 and F1+2	565/557	0.802	1.129	0.737	1	Mascot
1500.7516	1500.8239	0.0723	48	367	375	DKDNFWMTR	28	95.797	(N-term)_iTRAQ[0], Lysine(K)_iTRAQ[2]	[4] F7 and F10+11	1135/1127	0.812	1.318	1.278	1	Mascot
1757.8367	1757.8221	-0.0146	-8	201	214	GYAFNHSADFETVR	61	99.998	(N-term)_iTRAQ[0]	[4] F7 and F10+11	248/240	1.067	1.054	0.814	1	Mascot
1890.0591	1890.0776	0.0185	10	352	366	HMVFLGGAVLADIMK	117	100	(N-term)_iTRAQ[0], Lysine(K)_iTRAQ[15]	[4] F7 and F10+11	505/497	0.948	0.950	0.700	1	Mascot
1916.0184	1916.0135	-0.0049	-3	300	316	HIVLSGGSTMYPGLPSR	36	99.356	(N-term)_iTRAQ[0]	[3] F6 and F9	299/291	2.336	3.226	1.603	1	Mascot
1920.9426	1920.8451	-0.0975	-51	220	232	LCYVGYNIEQEQK	38	99.587	(N-term)_iTRAQ[0], Lysine(K)_iTRAQ[13], MMTS (C)[2]	[5] F4	244/236	0.896	0.948	0.940	1	Mascot

188 RecName: Full=Glutaminase kidney isoform, mitochondrial; Short=GLS; AltName: Full=K-glutaminase; Al — gi|121447 — 80862.3 — 6 — 346 — 0.907 — 0.907 — 0.914 — 0.234 — 0.283 — 0.130 — 6 — 6 — 6 — 100

Peptide Information

Calc. Mass	Obsrv. Mass	± da	± ppm	Start Seq.	End Seq.	Sequence	Ion Score	C. I. %	Modification	Plate [#] Name	Gel Idx/Pos [4700 Sample Name]	iTRAQ Ratio 115/114*	iTRAQ Ratio 116/114*	iTRAQ Ratio 117/114*	Rank	Result Type
1376.7823	1376.749	-0.0333	-24	393	401	NFAIGYYLK	45	99.922	(N-term)_iTRAQ[0], Lysine(K)_iTRAQ[9]	[7] F5 120912	326/318	0.718	1.035	0.879	1	Mascot
1472.7324	1472.824	0.0916	62	180	188	LKECMDMLR	42	99.85	(N-term)_iTRAQ[0], Lysine(K)_iTRAQ[2], MMTS (C)[4]	[4] F7 and F10+11	1273/1265	0.986	1.021	1.124	1	Mascot
1592.8392	1592.7744	-0.0648	-41	360	369	FDYVMQFLNK	48	99.957	(N-term)_iTRAQ[0], Lysine(K)_iTRAQ[10]	[5] F4	359/351	0.847	0.635	0.920	1	Mascot
1781.9507	1781.9569	0.0062	3	208	221	CVQSNIVLLTQAFR	37	99.451	(N-term)_iTRAQ[0], MMTS (C)[1]	[8] F13-15 and F1+2	323/315	0.869	0.653	1.026	1	Mascot
2151.989	2151.9028	-0.0862	-40	370	387	MAGNEYVGFSNATFQSER	92	100	(N-term)_iTRAQ[0]	[1] F3 030912	289/281	0.744	0.921	0.839	1	Mascot
2896.4912	2896.2805	-0.2107	-73	86	111	GGTPPQQQQQQQQQPGASPPAAPGPK	83	100	(N-term)_iTRAQ[0], Lysine(K)_iTRAQ[26]	[1] F3 030912	126/118	1.434	1.381	0.748	1	Mascot

189	amphiphysin [Rattus norvegicus]	gi|11560002	81120.4	5	345	0.847	1.086	1.149	0.450	0.279	0.392	6	6	6	100

Peptide Information

Calc. Mass	Obsrv. Mass	± da	± ppm	Start Seq.	End Seq.	Sequence	Ion Score	C. I. %	Modification	Plate [#] Name	Gel Idx/Pos [4700 Sample Name]	iTRAQ Ratio 115/114*	iTRAQ Ratio 116/114*	iTRAQ Ratio 117/114*	Rank	Result Type
1646.8622	1646.78	-0.0822	-50	242	256	AFSIQGAPSDSGPLR	66	99.999	(N-term)_iTRAQ[0]	[1] F3 030912	257/249	0.743	0.961	0.880	1	Mascot
1671.8799	1671.7563	-0.1236	-74	487	501	AALPAGEGESPEGAK	114	100	(N-term)_iTRAQ[0], Lysine(K)_iTRAQ[15]	[5] F4	93/85	1.302	1.511	1.254	1	Mascot
1753.8269	1753.7443	-0.0826	-47	102	112	CDVLWEDFHQK	66	99.999	(N-term)_iTRAQ[0], Lysine(K)_iTRAQ[11], MMTS (C)[1]	[6] F8 110912	393/385	0.881	0.800	0.918	1	Mascot
1753.8269	1753.8617	0.0348	20	102	112	CDVLWEDFHQK	61	99.998	(N-term)_iTRAQ[0], Lysine(K)_iTRAQ[11], MMTS (C)[1]	[3] F6 and F9	1330/1322	0.988	1.160	1.067	1	Mascot
1786.9069	1786.7812	-0.1257	-70	557	571	EATEDVAPQGPAGEK	38	99.62	(N-term)_iTRAQ[0], Lysine(K)_iTRAQ[15]	[5] F4	77/69	0.360	0.892	1.012	1	Mascot
2332.1541	2332.0952	-0.0589	-25	615	633	VETLHDFEAANSDELTLQR	61	99.998	(N-term)_iTRAQ[0]	[7] F5 120912	298/290	1.216	1.364	2.103	1	Mascot

190	Slc25a3 protein [Rattus norvegicus]	gi|47718004	43277.8	8	343	0.844	0.985	1.033	0.336	0.228	0.360	9	9	9	100

Peptide Information

Calc. Mass	Obsrv. Mass	± da	± ppm	Start Seq.	End Seq.	Sequence	Ion Score	C. I. %	Modification	Plate [#] Name	Gel Idx/Pos [4700 Sample Name]	iTRAQ Ratio 115/114*	iTRAQ Ratio 116/114*	iTRAQ Ratio 117/114*	Rank	Result Type
1073.605	1073.5975	-0.0075	-7	214	221	GVAPLWMR	36	99.359	(N-term)_iTRAQ[0]	[7] F5 120912	270/262	0.798	0.845	0.875	1	Mascot
1324.7188	1324.6949	-0.0239	-18	133	140	FGFYEVFK	29	96.584	(N-term)_iTRAQ[0], Lysine(K)_iTRAQ[8]	[7] F5 120912	382/374	0.795	0.749	0.850	1	Mascot
1358.7202	1358.6727	-0.0475	-35	205	213	EEGLNAFYK	36	99.43	(N-term)_iTRAQ[0], Lysine(K)_iTRAQ[9]	[3] F6 and F9	249/241	0.776	1.020	0.667	1	Mascot
1505.8197	1505.7096	-0.1101	-73	185	196	IQTQPGYANTLR	33	98.713	(N-term)_iTRAQ[0]	[5] F4	125/117	1.847	1.129	1.665	1	Mascot
1505.8197	1505.7369	-0.0828	-55	185	196	IQTQPGYANTLR	46	99.937	(N-term)_iTRAQ[0]	[1] F3 030912	196/188	0.871	0.936	1.057	1	Mascot
1591.9014	1591.9542	0.0528	33	289	300	EKGSTASQVLQR	42	99.839	(N-term)_iTRAQ[0], Lysine(K)_iTRAQ[2]	[3] F6 and F9	1101/1093	0.600	0.801	0.742	1	Mascot
1925.0214	1925.0792	0.0578	30	202	213	MYKEEGLNAFYK	76	100	(N-term)_iTRAQ[0], Lysine(K)_iTRAQ[3,12]	[4] F7 and F10+11	1176/1168	1.020	1.133	1.162	1	Mascot
2059.0405	2058.9636	-0.0769	-37	117	132	GWAPTLIGYSMQGLCK	44	99.901	(N-term)_iTRAQ[0], Lysine(K)_iTRAQ[16], MMTS (C)[15]	[5] F4	395/387	0.524	1.543	1.594	1	Mascot
2086.073	2086.0771	0.0041	2	141	156	ALYSNILGEENTYLWR	34	98.992	(N-term)_iTRAQ[0]	[8] F13-15 and F1+2	373/365	0.857	0.910	1.119	1	Mascot

191	NADH dehydrogenase [ubiquinone] iron-sulfur protein 2, mitochondrial precursor [Rattus norvegicus]	gi|58865384	56163.8	7	340	1.139	1.169	1.041	0.456	0.484	0.437	7	7	7	100

Peptide Information

Calc. Mass	Obsrv. Mass	± da	± ppm	Start Seq.	End Seq.	Sequence	Ion Score	C. I. %	Modification	Plate [#] Name	Gel Idx/Pos [4700 Sample Name]	iTRAQ Ratio 115/114*	iTRAQ Ratio 116/114*	iTRAQ Ratio 117/114*	Rank	Result Type
1294.709	1294.6796	-0.0294	-23	343	350	IIEQCLNK	40	99.771	(N-term)_iTRAQ[0], Lysine(K)_iTRAQ[8], MMTS (C)[5]	[7] F5 120912	302/294	1.006	0.932	0.850	1	Mascot
1389.7896	1389.7651	-0.0245	-18	97	107	LVLELSGEMVR	35	99.205	(N-term)_iTRAQ[0]	[1] F3 030912	389/381	1.060	1.512	1.298	1	Mascot
1484.8472	1484.8953	0.0481	32	426	437	APGFAHLAGLDK	36	99.32	(N-term)_iTRAQ[0], Lysine(K)_iTRAQ[12]	[3] F6 and F9	1237/1229	1.167	1.283	1.191	1	Mascot
1530.8076	1530.7675	-0.0401	-26	128	138	TYLQALPYFDR	41	99.791	(N-term)_iTRAQ[0]	[1] F3 030912	383/375	0.693	0.738	0.493	1	Mascot
1606.7866	1606.7372	-0.0494	-31	255	266	IDEVEEMLTNNR	30	97.514	(N-term)_iTRAQ[0]	[1] F3 030912	342/334	2.298	2.268	1.459	1	Mascot
1617.8668	1617.8256	-0.0412	-25	373	383	TSMESLIHHFK	69	100	(N-term)_iTRAQ[0], Lysine(K)_iTRAQ[11]	[2] F12 040912	349/341	0.991	1.065	1.379	1	Mascot
2657.2957	2657.2251	-0.0706	-27	37	56	WQPDIEWAEQFSGAVMYPSK	90	100	(N-term)_iTRAQ[0], Lysine(K)_iTRAQ[20]	[1] F3 030912	506/498	1.270	0.925	1.013	1	Mascot

192	elongation factor 2 [Rattus norvegicus]	gi|8393296	105371.3	8	340	1.120	1.036	0.936	0.206	0.172	0.249	8	8	8	100

Peptide Information

Calc. Mass	Obsrv. Mass	± da	± ppm	Start Seq.	End Seq.	Sequence	Ion Score	C. I. %	Modification	Plate [#] Name	Gel Idx/Pos [4700 Sample Name]	iTRAQ Ratio 115/114*	iTRAQ Ratio 116/114*	iTRAQ Ratio 117/114*	Rank	Result Type
1235.6868	1235.6055	-0.0813	-66	2	10	VNFTVDQIR	29	97.165	(N-term)_iTRAQ[0]	[5] F4	180/172	0.972	0.981	0.955	1	Mascot
1282.6188	1282.5443	-0.0745	-58	639	647	YEWDVAEAR	29	97.171	(N-term)_iTRAQ[0]	[5] F4	170/162	1.094	1.177	0.856	1	Mascot
1374.7338	1374.7146	-0.0192	-14	227	235	QFAEMYVAK	52	99.985	(N-term)_iTRAQ[0], Lysine(K)_iTRAQ[9]	[7] F5 120912	212/204	1.203	1.064	0.922	1	Mascot
1562.9152	1562.8267	-0.0885	-57	440	449	EDLYLKPIQR	31	97.824	(N-term)_iTRAQ[0], Lysine(K)_iTRAQ[6]	[6] F8 110912	244/236	1.071	1.013	1.179	1	Mascot

1732.9731	1732.9093	-0.0638	-37	846	858	EGIPALDNFLDKL	71	100	(N-term)_iTRAQ[0], Lysine(K)_iTRAQ[12]	[5] F4	402/394	1.628	1.308	1.200	1	Mascot
1835.1127	1835.0508	-0.0619	-34	439	449	KEDLYLKPIQR	31	98.088	(N-term)_iTRAQ[0], Lysine(K)_iTRAQ[1,7]	[2] F12 040912	237/229	0.997	1.150	1.088	1	Mascot
2382.2065	2382.1587	-0.0478	-20	649	667	IWCFGPDGTGPNILTDITK	61	99.998	(N-term)_iTRAQ[0], Lysine(K)_iTRAQ[19], MMTS (C)[3]	[1] F3 030912	529/521	1.214	0.767	0.541	1	Mascot
2431.2827	2431.3613	0.0786	32	606	625	ARPFPDGLAEDIDKGEVSAR	35	99.201	(N-term)_iTRAQ[0], Lysine(K)_iTRAQ[14]	[4] F7 and F10+11	1196/1188	0.919	0.924	0.936	1	Mascot

193	tropomyosin [Rattus norvegicus]	gi|438880	32495.3	6	338	0.960	0.960	1.124	0.156	0.145	0.274	6	6	6	100

Protein Group

tropomyosin alpha-3 chain isoform 1 [Rattus norvegicus]	gi|52353308	32242.2

Peptide Information

Calc. Mass	Obsrv. Mass	± da	± ppm	Start Seq.	End Seq.	Sequence	Ion Score	C. I. %	Modification	Plate [#] Name	Gel Idx/Pos [4700 Sample Name]	iTRAQ Ratio 115/114*	iTRAQ Ratio 116/114*	iTRAQ Ratio 117/114*	Rank	Result Type
1419.8127	1419.7928	-0.0199	-14	105	113	MELQEIQLK	39	99.703	(N-term)_iTRAQ[0], Lysine(K)_iTRAQ[9]	[7] F5 120912	263/255	1.018	1.260	1.489	1	Mascot
1460.7465	1460.6509	-0.0956	-65	43	54	EQAEAEVASLNR	34	99.015	(N-term)_iTRAQ[0]	[1] F3 030912	187/179	0.698	0.786	0.908	1	Mascot
1614.8597	1614.9342	0.0745	46	182	190	EDKYEEEIK	38	99.64	(N-term)_iTRAQ[0], Lysine(K)_iTRAQ[3,9]	[4] F7 and F10+11	1054/1046	0.993	0.930	0.822	1	Mascot
1786.9055	1786.8246	-0.0809	-45	14	27	IQVLQQQADDAEER	80	100	(N-term)_iTRAQ[0]	[1] F3 030912	221/213	1.004	0.955	1.091	1	Mascot
2059.103	2059.0034	-0.0996	-48	13	27	KIQVLQQQADDAEER	94	100	(N-term)_iTRAQ[0], Lysine(K)_iTRAQ[1]	[6] F8 110912	217/209	1.131	0.981	1.155	1	Mascot
2341.2095	2341.1135	-0.096	-41	154	169	CSELEEELKNVTNNLK	52	99.986	(N-term)_iTRAQ[0], Lysine(K)_iTRAQ[9,16], MMTS (C)[1]	[6] F8 110912	476/468	0.975	0.908	1.439	1	Mascot

194	subunit d of mitochondrial H-ATP synthase [Rattus norvegicus]	gi|220904	21553.5	6	335	1.020	1.022	0.956	0.288	0.270	0.467	8	8	8	100

Protein Group

ATP synthase subunit d, mitochondrial [Rattus norvegicus]	gi|9506411	21535.5

Peptide Information

Calc. Mass	Obsrv. Mass	± da	± ppm	Start Seq.	End Seq.	Sequence	Ion Score	C. I. %	Modification	Plate [#] Name	Gel Idx/Pos [4700 Sample Name]	iTRAQ Ratio 115/114*	iTRAQ Ratio 116/114*	iTRAQ Ratio 117/114*	Rank	Result Type
1321.641	1321.7068	0.0658	50	33	41	SWNETFHTR	32	98.288	(N-term)_iTRAQ[0]	[4] F7 and F10+11	1088/1080	1.607	1.324	1.982	1	Mascot
1471.6887	1471.6172	-0.0715	-49	100	111	NCAQFVTGSQAR	51	99.982	(N-term)_iTRAQ[0], MMTS (C)[2]	[1] F3 030912	230/222	0.773	0.683	0.486	1	Mascot
1850.0396	1850.0952	0.0556	30	59	71	ANVDKPGLVDDFK	63	99.999	(N-term)_iTRAQ[0], Lysine(K)_iTRAQ[5,13]	[3] F6 and F9	1208/1200	0.791	0.929	0.699	1	Mascot
1850.0396	1850.1238	0.0842	46	59	71	ANVDKPGLVDDFK	61	99.998	(N-term)_iTRAQ[0], Lysine(K)_iTRAQ[5,13]	[4] F7 and F10+11	1144/1136	1.049	1.204	1.264	1	Mascot
1973.0531	1972.9829	-0.0702	-36	149	161	KYPYWPHQPIENL	44	99.896	(N-term)_iTRAQ[0], Lysine(K)_iTRAQ[1]	[6] F8 110912	360/352	1.320	0.893	0.808	1	Mascot
2042.1029	2041.9952	-0.1077	-53	86	99	YTALVDAEEKEDVK	80	100	(N-term)_iTRAQ[0], Lysine(K)_iTRAQ[10,14]	[6] F8 110912	266/258	1.136	1.399	0.965	1	Mascot
2042.1029	2042.0746	-0.0283	-14	86	99	YTALVDAEEKEDVK	47	99.951	(N-term)_iTRAQ[0], Lysine(K)_iTRAQ[10,14]	[4] F7 and F10+11	265/257	0.791	0.828	0.862	1	Mascot
2223.1729	2223.0952	-0.0777	-35	10	25	TIDWVSFVEIMPQNQK	65	99.999	(N-term)_iTRAQ[0], Lysine(K)_iTRAQ[16]	[1] F3 030912	488/480	0.960	1.139	1.223	1	Mascot

195	calnexin precursor [Rattus norvegicus]	gi|25282419	75892.5	6	334	1.081	1.023	0.896	0.434	0.141	0.314	6	6	6	100

Peptide Information

Calc. Mass	Obsrv. Mass	± da	± ppm	Start Seq.	End Seq.	Sequence	Ion Score	C. I. %	Modification	Plate [#] Name	Gel Idx/Pos [4700 Sample Name]	iTRAQ Ratio 115/114*	iTRAQ Ratio 116/114*	iTRAQ Ratio 117/114*	Rank	Result Type
1335.7882	1335.7493	-0.0389	-29	79	88	GSLSGWILSK	64	99.999	(N-term)_iTRAQ[0], Lysine(K)_iTRAQ[10]	[7] F5 120912	326/318	1.629	1.129	1.221	1	Mascot
1734.8909	1734.8295	-0.0614	-35	172	183	TSELNLDQFHDK	47	99.95	(N-term)_iTRAQ[0], Lysine(K)_iTRAQ[12]	[6] F8 110912	244/236	1.239	1.110	0.737	1	Mascot
1762.901	1762.8561	-0.0449	-25	447	459	VVDDWANDGWGLK	75	100	(N-term)_iTRAQ[0], Lysine(K)_iTRAQ[13]	[7] F5 120912	346/338	0.979	0.886	0.758	1	Mascot
1831.9271	1832.0176	0.0905	49	101	111	YDGKWEVDEMK	34	98.987	(N-term)_iTRAQ[0], Lysine(K)_iTRAQ[4,11]	[4] F7 and F10+11	1142/1134	0.818	1.037	1.271	1	Mascot
2035.0984	2035.1844	0.086	42	447	460	VVDDWANDGWGLKK	54	99.99	(N-term)_iTRAQ[0], Lysine(K)_iTRAQ[13,14]	[4] F7 and F10+11	1218/1210	0.638	0.838	0.558	1	Mascot

Calc. Mass	Obsrv. Mass	± da	± ppm	Start Seq.	End Seq.	Sequence	Ion Score	C. I. %	Modification	Plate [#] Name	Gel Idx/Pos [4700 Sample Name]	iTRAQ Ratio 115/114*	iTRAQ Ratio 116/114*	iTRAQ Ratio 117/114*	Rank	Result Type
2152.1323	2152.1626	0.0303	14	402	416	KIPNPDFFEDLEPFR	60	99.998	(N-term)_iTRAQ[0], Lysine(K)_iTRAQ[1]	[4] F7 and F10+11	466/458	1.551	1.189	1.071	1	Mascot

196	calbindin-d28k [Rattus norvegicus]	gi|203237	33927.7	6	331	0.890	1.080	1.203	0.185	0.176	0.176	6	6	6	100	

Protein Group

calbindin [Rattus norvegicus]	gi|14010887	33905.7

Peptide Information

Calc. Mass	Obsrv. Mass	± da	± ppm	Start Seq.	End Seq.	Sequence	Ion Score	C. I. %	Modification	Plate [#] Name	Gel Idx/Pos [4700 Sample Name]	iTRAQ Ratio 115/114*	iTRAQ Ratio 116/114*	iTRAQ Ratio 117/114*	Rank	Result Type
1207.5388	1207.5054	-0.0334	-28	99	105	SCEEFMK	35	99.263	(N-term)_iTRAQ[0], Lysine(K)_iTRAQ[7], MMTS (C)[2]	[3] F6 and F9	256/248	1.237	1.301	1.132	1	Mascot
1484.828	1484.7599	-0.0681	-46	143	152	LAEYTDLMLK	50	99.977	(N-term)_iTRAQ[0], Lysine(K)_iTRAQ[10]	[5] F4	278/270	0.673	0.794	0.994	1	Mascot
1601.9877	1601.9294	-0.0583	-36	170	180	LLPVQENFLLK	51	99.982	(N-term)_iTRAQ[0], Lysine(K)_iTRAQ[11]	[5] F4	315/307	0.829	1.092	1.481	1	Mascot
1711.9827	1711.9572	-0.0255	-15	35	47	ELQNLIQELLQAR	61	99.998	(N-term)_iTRAQ[0]	[1] F3 030912	564/556	0.840	1.167	1.174	1	Mascot
2241.1431	2241.0867	-0.0564	-25	153	169	LFDSNNDGKLELTEMAR	38	99.563	(N-term)_iTRAQ[0], Lysine(K)_iTRAQ[9]	[4] F7 and F10+11	347/339	0.848	1.116	1.112	1	Mascot
2344.2043	2344.2988	0.0945	40	109	124	KYDTDHSGFIETEELK	95	100	(N-term)_iTRAQ[0], Lysine(K)_iTRAQ[1,16]	[4] F7 and F10+11	1131/1123	1.011	1.078	1.393	1	Mascot

197	beta-synuclein [Rattus norvegicus]	gi|77404215	16230.4	5	330	0.818	1.165	0.899	0.406	0.201	0.372	5	5	5	100	

Protein Group

RecName: Full=Beta-synuclein; AltName: Full=Phosphoneuroprotein 14; Short=PNP 14	gi|2501106	16656.7

Peptide Information

Calc. Mass	Obsrv. Mass	± da	± ppm	Start Seq.	End Seq.	Sequence	Ion Score	C. I. %	Modification	Plate [#] Name	Gel Idx/Pos [4700 Sample Name]	iTRAQ Ratio 115/114*	iTRAQ Ratio 116/114*	iTRAQ Ratio 117/114*	Rank	Result Type
1161.6725	1161.6531	-0.0194	-17	13	21	EGVVAAAEK	59	99.997	(N-term)_iTRAQ[0], Lysine(K)_iTRAQ[9]	[7] F5 120912	103/95	0.391	0.989	0.596	1	Mascot
1239.7195	1239.6522	-0.0673	-54	35	43	EGVLYVGSK	45	99.917	(N-term)_iTRAQ[0], Lysine(K)_iTRAQ[9]	[7] F5 120912	165/157	0.952	1.163	0.790	1	Mascot
1560.8843	1560.7977	-0.0866	-55	46	58	EGVVQGVASVAEK	96	100	(N-term)_iTRAQ[0], Lysine(K)_iTRAQ[13]	[5] F4	156/148	0.966	1.564	0.769	1	Mascot
1612.9645	1613.0343	0.0698	43	33	43	TKEGVLYVGSK	55	99.993	(N-term)_iTRAQ[0], Lysine(K)_iTRAQ[2,11]	[4] F7 and F10+11	1080/1072	1.299	1.041	1.668	1	Mascot
1934.1293	1934.0133	-0.116	-60	44	58	TKEGVVQGVASVAEK	77	100	(N-term)_iTRAQ[0], Lysine(K)_iTRAQ[2,15]	[6] F8 110912	239/231	0.783	1.148	0.973	1	Mascot

198	synaptic vesicle glycoprotein 2B [Rattus norvegicus]	gi|17105360	82994.5	6	329	1.232	1.436	1.562	0.398	0.262	0.442	6	6	6	100	

Peptide Information

Calc. Mass	Obsrv. Mass	± da	± ppm	Start Seq.	End Seq.	Sequence	Ion Score	C. I. %	Modification	Plate [#] Name	Gel Idx/Pos [4700 Sample Name]	iTRAQ Ratio 115/114*	iTRAQ Ratio 116/114*	iTRAQ Ratio 117/114*	Rank	Result Type
1218.7456	1218.7994	0.0538	44	334	341	VFTVSHIK	41	99.811	(N-term)_iTRAQ[0], Lysine(K)_iTRAQ[8]	[4] F7 and F10+11	1115/1107	0.710	1.159	1.460	1	Mascot
1409.6835	1409.6313	-0.0522	-37	417	424	YFQDEEYK	47	99.947	(N-term)_iTRAQ[0], Lysine(K)_iTRAQ[8]	[3] F6 and F9	212/204	1.450	1.778	1.909	1	Mascot
1430.7712	1430.8364	0.0652	46	310	318	HDEAWMILK	58	99.996	(N-term)_iTRAQ[0], Lysine(K)_iTRAQ[9]	[4] F7 and F10+11	1208/1200	1.076	1.226	1.171	1	Mascot
1840.7898	1840.7008	-0.089	-48	8	21	DNYEGYAPNDGYYR	63	99.999	(N-term)_iTRAQ[0]	[1] F3 030912	211/203	1.273	1.601	1.126	1	Mascot
2052.916	2052.8579	-0.0581	-28	477	490	CYFEDVTSTDTYFK	42	99.833	(N-term)_iTRAQ[0], Lysine(K)_iTRAQ[14], MMTS (C)[1]	[1] F3 030912	393/385	1.554	1.283	1.802	1	Mascot
2159.9543	2159.8232	-0.1311	-61	6	21	YRDNYEGYAPNDGYYR	78	100	(N-term)_iTRAQ[0]	[6] F8 110912	228/220	1.596	1.691	2.198	1	Mascot

199	mitogen-activated protein kinase 1 [Mus musculus]	gi|6754632	45029.5	7	326	0.958	1.138	1.126	0.246	0.268	0.227	7	7	7	100	

Peptide Information

Calc. Mass	Obsrv. Mass	± da	± ppm	Start Seq.	End Seq.	Sequence	Ion Score	C. I. %	Modification	Plate [#] Name	Gel Idx/Pos [4700 Sample Name]	iTRAQ Ratio 115/114*	iTRAQ Ratio 116/114*	iTRAQ Ratio 117/114*	Rank	Result Type
1118.6078	1118.5389	-0.0689	-62	14	22	GQVFDVGPR	43	99.866	(N-term)_iTRAQ[0]	[5] F4	131/123	0.697	0.963	0.982	1	Mascot
1251.6705	1251.6093	-0.0612	-49	343	351	ELIFEETAR	40	99.777	(N-term)_iTRAQ[0]	[1] F3 030912	257/249	0.940	1.372	1.239	1	Mascot
1698.7833	1698.7009	-0.0824	-49	54	65	ISPFEHQTYCQR	31	98.083	(N-term)_iTRAQ[0], MMTS (C)[10]	[6] F8 110912	264/256	1.373	1.376	1.189	1	Mascot

Calc. Mass	Obsrv. Mass	± da	± ppm	Start Seq.	End Seq.	Sequence	Ion Score	C. I. %	Modification	Plate [#] Name	Gel Idx/Pos [4700 Sample Name]	iTRAQ Ratio 115/114*	iTRAQ Ratio 116/114*	iTRAQ Ratio 117/114*	Rank	Result Type
1854.047	1854.001	-0.046	-25	76	89	FRHENIIGINDIIR	43	99.879	(N-term)_iTRAQ[0]	[2] F12 040912	372/364	0.826	0.885	0.963	1	Mascot
1970.9807	1970.9188	-0.0619	-31	53	65	KISPFEHQTYCQR	30	97.531	(N-term)_iTRAQ[0], Lysine(K)_iTRAQ[1], MMTS (C)[11]	[2] F12 040912	257/249	0.918	1.068	1.516	1	Mascot
2133.1035	2133.041	-0.0625	-29	98	112	DVYIVQDLMETDLYK	81	100	(N-term)_iTRAQ[0], Lysine(K)_iTRAQ[15]	[1] F3 030912	477/469	0.834	0.919	0.851	1	Mascot
2266.2502	2266.2617	0.0115	5	147	162	DLKPSNLLLNTTCDLK	58	99.996	(N-term)_iTRAQ[0], Lysine(K)_iTRAQ[3,16], MMTS (C)[13]	[4] F7 and F10+11	407/399	1.296	1.563	1.273	1	Mascot

200	tropomyosin 5 [Rattus sp.]	gi|9653293	32572	6	325	1.040	1.018	1.158	0.283	0.194	0.342	6	6	6	100

Protein Group

RecName: Full=Tropomyosin alpha-3 chain; AltName: Full=Gamma-tropomyosin; AltName: Full=Tropomyosin	gi|148840439	32631.1
tropomyosin alpha-3 chain isoform 2 [Rattus norvegicus]	gi|29336093	32659.1

Peptide Information

Calc. Mass	Obsrv. Mass	± da	± ppm	Start Seq.	End Seq.	Sequence	Ion Score	C. I. %	Modification	Plate [#] Name	Gel Idx/Pos [4700 Sample Name]	iTRAQ Ratio 115/114*	iTRAQ Ratio 116/114*	iTRAQ Ratio 117/114*	Rank	Result Type
1419.8127	1419.7928	-0.0199	-14	105	113	MELQEIQLK	39	99.703	(N-term)_iTRAQ[0], Lysine(K)_iTRAQ[9]	[7] F5 120912	263/255	1.018	1.260	1.489	1	Mascot
1460.7465	1460.6509	-0.0956	-65	43	54	EQAEAEVASLNR	34	99.015	(N-term)_iTRAQ[0]	[1] F3 030912	187/179	0.698	0.786	0.908	1	Mascot
1614.8597	1614.9342	0.0745	46	182	190	EDKYEEEIK	38	99.64	(N-term)_iTRAQ[0], Lysine(K)_iTRAQ[3,9]	[4] F7 and F10+11	1054/1046	0.993	0.930	0.822	1	Mascot
1621.9384	1621.9894	0.051	31	216	225	TIDDLEDKLK	40	99.741	(N-term)_iTRAQ[0], Lysine(K)_iTRAQ[8,10]	[4] F7 and F10+11	1177/1169	1.584	1.291	1.724	1	Mascot
1786.9055	1786.8246	-0.0809	-45	14	27	IQVLQQQADDAEER	80	100	(N-term)_iTRAQ[0]	[1] F3 030912	221/213	1.004	0.955	1.091	1	Mascot
2059.103	2059.0034	-0.0996	-48	13	27	KIQVLQQQADDAEER	94	100	(N-term)_iTRAQ[0], Lysine(K)_iTRAQ[1]	[6] F8 110912	217/209	1.131	0.981	1.155	1	Mascot

201	PMES-2C [Rattus norvegicus]	gi|55700833	34935	4	318	1.866	0.894	0.892	0.621	0.143	0.090	4	4	4	100

Protein Group

PMES-2B [Rattus norvegicus]	gi|55700831	44463.8
PMES-2D [Rattus norvegicus]	gi|55700835	33344.3
band83 [Rattus norvegicus]	gi|21668480	37419.1

Peptide Information

Calc. Mass	Obsrv. Mass	± da	± ppm	Start Seq.	End Seq.	Sequence	Ion Score	C. I. %	Modification	Plate [#] Name	Gel Idx/Pos [4700 Sample Name]	iTRAQ Ratio 115/114*	iTRAQ Ratio 116/114*	iTRAQ Ratio 117/114*	Rank	Result Type
1794.9484	1794.8364	-0.112	-62	160	174	TPSPPEPEPAGTAQK	110	100	(N-term)_iTRAQ[0], Lysine(K)_iTRAQ[15]	[1] F3 030912	143/135	2.799	1.115	0.997	1	Mascot
2055.1206	2055.0305	-0.0901	-44	66	80	HKDAENSPTTPANLK	37	99.488	(N-term)_iTRAQ[0], Lysine(K)_iTRAQ[2,15]	[2] F12 040912	127/119	1.258	0.748	0.810	1	Mascot
2122.1362	2121.969	-0.1672	-79	81	96	SDKADLTPQETQGTAK	102	100	(N-term)_iTRAQ[0], Lysine(K)_iTRAQ[3,16]	[6] F8 110912	130/122	1.994	0.826	0.813	1	Mascot
2143.1677	2143.1101	-0.0576	-27	250	267	MLDAQVQTDPVSIGPVGK	69	100	(N-term)_iTRAQ[0], Lysine(K)_iTRAQ[18]	[1] F3 030912	304/296	1.726	0.925	0.966	1	Mascot

202	solute carrier family 12 member 5 [Rattus norvegicus]	gi|19705463	131163.6	6	316	0.929	0.976	0.870	0.149	0.100	0.356	6	6	6	100

Peptide Information

Calc. Mass	Obsrv. Mass	± da	± ppm	Start Seq.	End Seq.	Sequence	Ion Score	C. I. %	Modification	Plate [#] Name	Gel Idx/Pos [4700 Sample Name]	iTRAQ Ratio 115/114*	iTRAQ Ratio 116/114*	iTRAQ Ratio 117/114*	Rank	Result Type
1391.6862	1391.613	-0.0732	-53	810	820	NVSMFPGNPER	41	99.808	(N-term)_iTRAQ[0]	[1] F3 030912	216/208	0.909	0.989	0.911	1	Mascot
1422.7866	1422.8087	0.0221	16	874	883	DLTTFLYHLR	63	99.999	(N-term)_iTRAQ[0]	[4] F7 and F10+11	419/411	0.758	0.979	0.617	1	Mascot
1694.984	1694.9519	-0.0321	-19	873	883	KDLTTFLYHLR	60	99.997	(N-term)_iTRAQ[0], Lysine(K)_iTRAQ[1]	[2] F12 040912	390/382	0.940	1.091	1.168	1	Mascot
1742.8695	1742.75	-0.1195	-69	24	36	ESSPFINSTDTEK	56	99.994	(N-term)_iTRAQ[0], Lysine(K)_iTRAQ[13]	[5] F4	123/115	1.236	0.798	1.285	1	Mascot
1802.021	1802.0552	0.0342	19	516	529	DGIVPFLQVFGHGK	65	99.999	(N-term)_iTRAQ[0], Lysine(K)_iTRAQ[14]	[4] F7 and F10+11	459/451	0.843	0.991	0.496	1	Mascot
2529.2441	2529.2424	-0.0017	-1	1031	1048	DFFSMKPEWENLNQSNVR	32	98.272	(N-term)_iTRAQ[0], Lysine(K)_iTRAQ[6]	[4] F7 and F10+11	387/379	0.952	1.036	1.035	1	Mascot

203	creatine kinase U-type, mitochondrial precursor [Rattus norvegicus]	gi|60678254	50280.3	7	313	0.774	0.883	0.827	0.189	0.243	0.188	12	12	12	100

Peptide Information

Calc. Mass	Obsrv. Mass	± da	± ppm	Start Seq.	End Seq.	Sequence	Ion Score	C. I. %	Modification	Plate [#] Name	Gel Idx/Pos [4700 Sample Name]	iTRAQ Ratio 115/114*	iTRAQ Ratio 116/114*	iTRAQ Ratio 117/114*	Rank	Result Type
1204.6283	1204.6053	-0.023	-19	173	182	GLSLPPACTR	36	99.402	(N-term)_iTRAQ[0], MMTS (C)[8]	[1] F3 030912	304/296	0.610	0.572	0.666	1	Mascot
1245.7928	1245.7469	-0.0459	-37	409	417	IPPPLVHGK	41	99.813	(N-term)_iTRAQ[0], Lysine(K)_iTRAQ[9]	[6] F8 110912	206/198	0.512	0.630	0.640	1	Mascot
1398.6385	1398.6619	0.0234	17	302	310	GWEFMWNER	35	99.194	(N-term)_iTRAQ[0]	[7] F5 120912	366/358	0.821	0.814	1.103	1	Mascot
1564.8131	1564.7521	-0.061	-39	47	58	LYPPSAEYPDLR	28	96.219	(N-term)_iTRAQ[0]	[1] F3 030912	284/276	0.617	1.343	0.955	1	Mascot
1564.8131	1564.8314	0.0183	12	47	58	LYPPSAEYPDLR	37	99.522	(N-term)_iTRAQ[0]	[8] F13-15 and F1+2	479/471	0.717	0.823	0.618	1	Mascot
1564.8131	1564.8455	0.0324	21	47	58	LYPPSAEYPDLR	34	98.989	(N-term)_iTRAQ[0]	[8] F13-15 and F1+2	482/474	0.962	0.894	1.028	1	Mascot
1564.8131	1564.85	0.0369	24	47	58	LYPPSAEYPDLR	49	99.97	(N-term)_iTRAQ[0]	[8] F13-15 and F1+2	486/478	0.815	0.975	0.785	1	Mascot
1789.8993	1789.8091	-0.0902	-50	258	270	SFLIWVNEEDHTR	45	99.923	(N-term)_iTRAQ[0]	[6] F8 110912	397/389	0.625	0.726	0.672	1	Mascot
1789.8993	1789.8953	-0.004	-2	258	270	SFLIWVNEEDHTR	71	100	(N-term)_iTRAQ[0]	[4] F7 and F10+11	395/387	0.890	0.800	0.947	1	Mascot
1867.9875	1868.0056	0.0181	10	311	326	LGYILTCPSNLGTGLR	45	99.915	(N-term)_iTRAQ[0], MMTS (C)[7]	[8] F13-15 and F1+2	356/348	0.947	1.166	0.788	1	Mascot
2197.0857	2197.1018	0.0161	7	355	375	GTGGVDTAATGSVFDISN LDR	37	99.47	(N-term)_iTRAQ[0]	[8] F13-15 and F1+2	430/422	1.026	1.071	1.117	1	Mascot
2197.0857	2197.1108	0.0251	11	355	375	GTGGVDTAATGSVFDISN LDR	28	96.068	(N-term)_iTRAQ[0]	[8] F13-15 and F1+2	530/522	0.964	1.093	0.810	1	Mascot

204	nucleoside diphosphate kinase B [Rattus norvegicus]	gi|55926145	19237.2	5	312	1.115	0.975	1.037	0.334	0.273	0.333	7	7	7	100

Peptide Information

Calc. Mass	Obsrv. Mass	± da	± ppm	Start Seq.	End Seq.	Sequence	Ion Score	C. I. %	Modification	Plate [#] Name	Gel Idx/Pos [4700 Sample Name]	iTRAQ Ratio 115/114*	iTRAQ Ratio 116/114*	iTRAQ Ratio 117/114*	Rank	Result Type
1184.5657	1184.5066	-0.0591	-50	106	114	GDFCIQVGR	32	98.456	(N-term)_iTRAQ[0], MMTS (C)[4]	[5] F4	202/194	0.729	0.901	0.624	1	Mascot
1632.9683	1632.8651	-0.1032	-63	7	18	TFIAIKPDGVQR	29	96.722	(N-term)_iTRAQ[0], Lysine(K)_iTRAQ[6]	[6] F8 110912	250/242	1.843	1.399	1.235	1	Mascot
1632.9683	1632.9409	-0.0274	-17	7	18	TFIAIKPDGVQR	60	99.997	(N-term)_iTRAQ[0], Lysine(K)_iTRAQ[6]	[4] F7 and F10+11	243/235	1.187	1.016	1.214	1	Mascot
2074.1213	2074.0786	-0.0427	-21	89	105	VMLGETNPADSKPGTIR	54	99.991	(N-term)_iTRAQ[0], Lysine(K)_iTRAQ[12]	[4] F7 and F10+11	226/218	1.134	1.322	1.327	1	Mascot
2074.1213	2074.0818	-0.0395	-19	89	105	VMLGETNPADSKPGTIR	38	99.638	(N-term)_iTRAQ[0], Lysine(K)_iTRAQ[12]	[3] F6 and F9	227/219	0.979	0.721	0.963	1	Mascot
2312.2913	2312.3005	0.0092	4	129	143	EIGLWFKPEELIDYK	88	100	(N-term)_iTRAQ[0], Lysine(K)_iTRAQ[7,15]	[4] F7 and F10+11	484/476	1.230	0.971	1.384	1	Mascot
2381.2607	2381.2058	-0.0549	-23	67	85	YMNSGPVVAMVWEGLN VVK	78	100	(N-term)_iTRAQ[0], Lysine(K)_iTRAQ[19]	[1] F3 030912	544/536	0.985	0.706	0.780	1	Mascot

205	adenylate kinase isoenzyme 1 [Rattus norvegicus]	gi|61889092	24544.2	5	310	0.890	1.013	0.894	0.122	0.088	0.120	6	6	6	100

Protein Group

adenylate kinase isozyme 1 [Rattus norvegicus]	gi|8918488	24516.2

Peptide Information

Calc. Mass	Obsrv. Mass	± da	± ppm	Start Seq.	End Seq.	Sequence	Ion Score	C. I. %	Modification	Plate [#] Name	Gel Idx/Pos [4700 Sample Name]	iTRAQ Ratio 115/114*	iTRAQ Ratio 116/114*	iTRAQ Ratio 117/114*	Rank	Result Type
1418.8617	1418.7902	-0.0715	-50	10	21	IIFVVGGPGSGK	70	100	(N-term)_iTRAQ[0], Lysine(K)_iTRAQ[12]	[5] F4	222/214	0.796	0.943	0.801	1	Mascot
1557.8411	1557.76	-0.0811	-52	156	166	ATEPVISFYDK	57	99.995	(N-term)_iTRAQ[0], Lysine(K)_iTRAQ[11]	[5] F4	200/192	0.765	1.151	0.808	1	Mascot
1639.8564	1639.8191	-0.0373	-23	32	44	YGYTHLSTGDLLR	44	99.896	(N-term)_iTRAQ[0]	[4] F7 and F10+11	298/290	1.065	0.977	0.817	1	Mascot
1713.9421	1713.8613	-0.0808	-47	156	167	ATEPVISFYDKR	65	99.999	(N-term)_iTRAQ[0], Lysine(K)_iTRAQ[11]	[6] F8 110912	266/258	0.973	0.942	1.150	1	Mascot
1728.969	1728.9246	-0.0444	-26	64	77	GELVPLETVLDMLR	53	99.987	(N-term)_iTRAQ[0]	[1] F3 030912	577/569	0.795	0.960	0.923	1	Mascot
1728.969	1728.9392	-0.0298	-17	64	77	GELVPLETVLDMLR	73	100	(N-term)_iTRAQ[0]	[8] F13-15 and F1+2	218/210	0.989	1.125	0.913	1	Mascot

206	neuroplastin [Rattus norvegicus]	gi|9507073	47350.4	7	307	0.912	0.916	0.858	0.145	0.237	0.251	7	7	7	100

Protein Group

Nptn protein [Rattus norvegicus]	gi|47718024	33450.3

Peptide Information

Calc. Mass	Obsrv. Mass	± da	± ppm	Start	End	Sequence	Ion	C. I. %	Modification	Plate [#] Name	Gel Idx/Pos [4700	iTRAQ	iTRAQ	iTRAQ	Rank	Result Type

Calc. Mass	Obsrv. Mass	± da	± ppm	Start Seq.	End Seq.	Sequence	Ion Score	C. I. %	Modification	Plate [#] Name	Gel Idx/Pos [4700 Sample Name]	iTRAQ Ratio 115/114*	iTRAQ Ratio 116/114*	iTRAQ Ratio 117/114*	Rank	Result Type
1069.6655	1069.6301	-0.0354	-33	288	293	FFIINK	28	95.864	(N-term)_iTRAQ[0], Lysine(K)_iTRAQ[6]	[7] F5 120912	283/275	1.099	1.400	1.495	1	Mascot
1302.7753	1302.7097	-0.0656	-50	150	159	IVTSEEVIIR	40	99.726	(N-term)_iTRAQ[0]	[1] F3 030912	262/254	1.038	0.929	0.900	1	Mascot
1599.8411	1599.8734	0.0323	20	364	375	RPDEVPDAGPMK	29	96.877	(N-term)_iTRAQ[0], Lysine(K)_iTRAQ[12]	[3] F6 and F9	1125/1117	0.707	0.685	0.641	1	Mascot
1688.8127	1688.8389	0.0262	16	260	271	SVGYPHPEWMWR	53	99.989	(N-term)_iTRAQ[0]	[3] F6 and F9	1296/1288	0.923	0.748	0.805	1	Mascot
1872.0385	1871.9714	-0.0671	-36	363	375	KRPDEVPDAGPMK	41	99.816	(N-term)_iTRAQ[0], Lysine(K)_iTRAQ[1,13]	[2] F12 040912	156/148	1.014	1.143	0.926	1	Mascot
2226.0015	2226.0969	0.0954	43	245	259	SENKNEGQDAMMYCK	60	99.998	(N-term)_iTRAQ[0], Lysine(K)_iTRAQ[4,15], MMTS (C)[14]	[4] F7 and F10+11	1131/1123	0.889	0.872	0.755	1	Mascot
2373.0869	2373.1665	0.0796	34	209	226	AEDSGEYHCVYHFVSAPK	57	99.995	(N-term)_iTRAQ[0], Lysine(K)_iTRAQ[18], MMTS (C)[9]	[4] F7 and F10+11	1192/1184	0.780	0.812	0.708	1	Mascot

207	cullin-associated NEDD8-dissociated protein 1 [Rattus norvegicus]	gi|16758920	149038.3	6	307	0.923	0.970	1.237	0.298	0.394	0.284	6	6	6	100

Peptide Information

Calc. Mass	Obsrv. Mass	± da	± ppm	Start Seq.	End Seq.	Sequence	Ion Score	C. I. %	Modification	Plate [#] Name	Gel Idx/Pos [4700 Sample Name]	iTRAQ Ratio 115/114*	iTRAQ Ratio 116/114*	iTRAQ Ratio 117/114*	Rank	Result Type
1224.6544	1224.6073	-0.0471	-38	1065	1072	EVEMGPFK	36	99.44	(N-term)_iTRAQ[0], Lysine(K)_iTRAQ[8]	[7] F5 120912	193/185	1.266	1.074	1.352	1	Mascot
1247.6881	1247.6373	-0.0508	-41	794	801	QSYYSIAK	29	96.591	(N-term)_iTRAQ[0], Lysine(K)_iTRAQ[8]	[3] F6 and F9	190/182	0.805	0.467	1.135	1	Mascot
1627.9867	1627.964	-0.0227	-14	730	743	ISGSILNELIGLVR	54	99.991	(N-term)_iTRAQ[0]	[1] F3 030912	564/556	0.815	1.205	1.039	1	Mascot
1664.9622	1664.9116	-0.0506	-30	392	403	ADVFHAYLSLLK	94	100	(N-term)_iTRAQ[0], Lysine(K)_iTRAQ[12]	[6] F8 110912	459/451	1.212	1.056	1.072	1	Mascot
2022.094	2022.0297	-0.0643	-32	778	793	MLTGPVYSQSTALTHK	66	99.999	(N-term)_iTRAQ[0], Lysine(K)_iTRAQ[16]	[3] F6 and F9	269/261	1.084	1.329	1.886	1	Mascot
2407.1968	2407.1228	-0.074	-31	145	164	QEDVSVQLEALDIMADMLSR	27	95.507	(N-term)_iTRAQ[0]	[1] F3 030912	580/572	0.568	0.982	1.111	1	Mascot

208	cytochrome b-c1 complex subunit 1, mitochondrial precursor [Rattus norvegicus]	gi|51948476	55384.7	5	306	0.932	1.073	1.147	0.462	0.508	0.576	5	5	5	100

Peptide Information

Calc. Mass	Obsrv. Mass	± da	± ppm	Start Seq.	End Seq.	Sequence	Ion Score	C. I. %	Modification	Plate [#] Name	Gel Idx/Pos [4700 Sample Name]	iTRAQ Ratio 115/114*	iTRAQ Ratio 116/114*	iTRAQ Ratio 117/114*	Rank	Result Type
1333.6906	1333.6328	-0.0578	-43	433	442	IEEVDAQMVR	49	99.97	(N-term)_iTRAQ[0]	[1] F3 030912	219/211	0.614	0.844	0.861	1	Mascot
1400.777	1400.8312	0.0542	39	423	432	RIPLAEWESR	36	99.405	(N-term)_iTRAQ[0]	[4] F7 and F10+11	1169/1161	0.688	0.950	0.961	1	Mascot
1790.9156	1790.8566	-0.059	-33	112	126	EVESIGAHLNAYSTR	76	100	(N-term)_iTRAQ[0]	[3] F6 and F9	252/244	1.283	1.381	1.263	1	Mascot
2201.0796	2201.092	0.0124	6	397	415	NALISHLDGTTPVCEDIGR	41	99.785	(N-term)_iTRAQ[0], MMTS (C)[14]	[7] F5 120912	366/358	1.742	1.969	2.424	1	Mascot
2643.4702	2643.4187	-0.0515	-19	143	163	VVELLADIVQNISLEDSQIEK	104	100	(N-term)_iTRAQ[0], Lysine(K)_iTRAQ[21]	[1] F3 030912	602/594	0.742	0.653	0.785	1	Mascot

209	ATP-citrate lyase [Rattus norvegicus]	gi|17028103	100655.2	7	306	0.900	0.918	0.969	0.379	0.398	0.292	7	7	7	100

Protein Group

RecName: Full=ATP-citrate synthase; AltName: Full=ATP-citrate (pro-S-)-lyase; AltName: Full=Citrate	gi|113116	132102.1

Peptide Information

Calc. Mass	Obsrv. Mass	± da	± ppm	Start Seq.	End Seq.	Sequence	Ion Score	C. I. %	Modification	Plate [#] Name	Gel Idx/Pos [4700 Sample Name]	iTRAQ Ratio 115/114*	iTRAQ Ratio 116/114*	iTRAQ Ratio 117/114*	Rank	Result Type
1653.8204	1653.7384	-0.082	-50	427	441	TTDGVYEGVAIGGDR	29	96.819	(N-term)_iTRAQ[0]	[1] F3 030912	214/206	0.812	0.973	0.787	1	Mascot
1683.8774	1683.9263	0.0489	29	816	827	SMGFIGHYLDQK	65	99.999	(N-term)_iTRAQ[0], Lysine(K)_iTRAQ[12]	[3] F6 and F9	1287/1279	0.916	0.918	0.931	1	Mascot
1712.0078	1712.0258	0.018	11	343	357	TIAIIAEGIPEALTR	42	99.848	(N-term)_iTRAQ[0]	[8] F13-15 and F1+2	363/355	0.581	0.725	0.698	1	Mascot
1768.9625	1768.9618	-0.0007	0	220	233	AKPAMPQDSVPSPR	38	99.579	(N-term)_iTRAQ[0], Lysine(K)_iTRAQ[2]	[4] F7 and F10+11	171/163	0.642	0.454	0.949	1	Mascot
1818.8779	1818.8196	-0.0583	-32	586	598	KPASFMTSICDER	37	99.552	(N-term)_iTRAQ[0], Lysine(K)_iTRAQ[1], MMTS (C)[10]	[6] F8 110912	367/359	0.813	0.967	0.880	1	Mascot
1855.9873	1855.9231	-0.0642	-35	699	712	AFDSGIIPMEFVNK	50	99.976	(N-term)_iTRAQ[0], Lysine(K)_iTRAQ[14]	[5] F4	315/307	1.763	1.472	1.679	1	Mascot
2026.1694	2026.2498	0.0804	40	312	326	KHPEVDVLINFASLR	46	99.941	(N-term)_iTRAQ[0], Lysine(K)_iTRAQ[1]	[4] F7 and F10+11	1291/1283	1.204	1.309	1.123	1	Mascot

210	cAMP-dependent protein kinase type II-alpha regulatory	gi|29789096	49344.2	5	305	1.017	1.060	1.180	0.355	0.211	0.291	6	6	6	100

subunit [Rattus norvegicus]

Peptide Information

Calc. Mass	Obsrv. Mass	± da	± ppm	Start Seq.	End Seq.	Sequence	Ion Score	C. I. %	Modification	Plate [#] Name	Gel Idx/Pos [4700 Sample Name]	iTRAQ Ratio 115/114*	iTRAQ Ratio 116/114*	iTRAQ Ratio 117/114*	Rank	Result Type
1339.7467	1339.7014	-0.0453	-34	345	355	AASAYAVGDVK	45	99.92	(N-term)_iTRAQ[0], Lysine(K)_iTRAQ[11]	[7] F5 120912	147/139	1.441	1.287	1.470	1	Mascot
1699.8597	1699.8033	-0.0564	-33	201	214	GSFGELALMYNTPR	69	100	(N-term)_iTRAQ[0]	[1] F3 030912	370/362	0.749	0.891	0.987	1	Mascot
1699.8597	1699.879	0.0193	11	201	214	GSFGELALMYNTPR	29	96.7	(N-term)_iTRAQ[0]	[8] F13-15 and F1+2	401/393	0.825	0.866	1.022	1	Mascot
1981.1116	1981.1353	0.0237	12	330	344	GQYFGELALVTNKPR	65	99.999	(N-term)_iTRAQ[0], Lysine(K)_iTRAQ[13]	[4] F7 and F10+11	338/330	0.742	0.906	1.027	1	Mascot
2296.1741	2296.1138	-0.0603	-26	139	155	NLDQEQLSQVLDAMFEK	99	100	(N-term)_iTRAQ[0], Lysine(K)_iTRAQ[17]	[1] F3 030912	515/507	1.085	1.332	1.737	1	Mascot
2481.1289	2481.0281	-0.1008	-41	159	178	TDEHVIDQGDDGDNFYVIER	28	96.455	(N-term)_iTRAQ[0]	[7] F5 120912	244/236	1.546	1.183	1.019	1	Mascot

211	neurabin-1 [Rattus norvegicus]	gi|16758224	135669.7	6	305	0.776	0.929	0.905	0.136	0.160	0.118	6	6	6	100

Peptide Information

Calc. Mass	Obsrv. Mass	± da	± ppm	Start Seq.	End Seq.	Sequence	Ion Score	C. I. %	Modification	Plate [#] Name	Gel Idx/Pos [4700 Sample Name]	iTRAQ Ratio 115/114*	iTRAQ Ratio 116/114*	iTRAQ Ratio 117/114*	Rank	Result Type
1391.7767	1391.8273	0.0506	36	811	820	IRDLEAEVFR	35	99.24	(N-term)_iTRAQ[0]	[3] F6 and F9	1289/1281	0.640	0.952	0.944	1	Mascot
1492.8719	1492.8616	-0.0103	-7	799	810	AHLVEVQGLQVR	47	99.954	(N-term)_iTRAQ[0]	[4] F7 and F10+11	254/246	0.741	1.089	0.969	1	Mascot
1656.7938	1656.6937	-0.1001	-60	127	138	FDTMHDGPSYAK	59	99.997	(N-term)_iTRAQ[0], Lysine(K)_iTRAQ[12]	[6] F8 110912	193/185	0.947	0.835	0.925	1	Mascot
1748.8337	1748.7714	-0.0623	-36	166	179	AGEAEPQDEWGGSK	76	100	(N-term)_iTRAQ[0], Lysine(K)_iTRAQ[14]	[7] F5 120912	138/130	0.980	1.183	0.710	1	Mascot
1931.0192	1931.0494	0.0302	16	717	729	NQLQQNIEENKER	32	98.307	(N-term)_iTRAQ[0], Lysine(K)_iTRAQ[11]	[3] F6 and F9	1123/1115	0.746	0.752	0.867	1	Mascot
2053.1426	2053.0845	-0.0581	-28	32	44	STFDKPKPDGEQK	57	99.995	(N-term)_iTRAQ[0], Lysine(K)_iTRAQ[5,7,13]	[2] F12 040912	119/111	0.668	0.837	1.053	1	Mascot

212	RecName: Full=Hyaluronan and proteoglycan link protein 1; AltName: Full=Cartilage-linking protein 1	gi|1346731	43479.9	6	305	0.781	1.195	1.274	0.335	0.299	0.316	6	6	6	100

Peptide Information

Calc. Mass	Obsrv. Mass	± da	± ppm	Start Seq.	End Seq.	Sequence	Ion Score	C. I. %	Modification	Plate [#] Name	Gel Idx/Pos [4700 Sample Name]	iTRAQ Ratio 115/114*	iTRAQ Ratio 116/114*	iTRAQ Ratio 117/114*	Rank	Result Type
1307.6616	1307.7052	0.0436	33	170	178	YNLNFHEAR	41	99.803	(N-term)_iTRAQ[0]	[3] F6 and F9	1176/1168	0.915	0.963	1.180	1	Mascot
1369.8215	1369.8793	0.0578	42	334	341	FVGFPDKK	39	99.654	(N-term)_iTRAQ[0], Lysine(K)_iTRAQ[7,8]	[4] F7 and F10+11	1119/1111	0.667	1.347	1.196	1	Mascot
1417.7686	1417.6622	-0.1064	-75	66	76	DPTAFGSGIHK	33	98.624	(N-term)_iTRAQ[0], Lysine(K)_iTRAQ[11]	[6] F8 110912	188/180	1.282	1.469	1.353	1	Mascot
1604.8445	1604.9181	0.0736	46	237	245	NYGFWDKDK	45	99.917	(N-term)_iTRAQ[0], Lysine(K)_iTRAQ[7,9]	[4] F7 and F10+11	1124/1116	0.523	0.907	0.930	1	Mascot
2357.1707	2357.1006	-0.0701	-30	270	288	LTYDEAVQACLNDGAQIAK	84	100	(N-term)_iTRAQ[0], Lysine(K)_iTRAQ[19], MMTS (C)[10]	[1] F3 030912	469/461	0.505	1.029	1.235	1	Mascot
2618.2122	2618.1606	-0.0516	-20	179	199	QACLDQDAVIASFDQLYDAWR	65	99.999	(N-term)_iTRAQ[0], MMTS (C)[3]	[1] F3 030912	560/552	1.099	1.635	1.951	1	Mascot

213	transketolase [Rattus norvegicus]	gi|12018252	78557.9	5	304	1.272	0.973	1.055	0.318	0.502	0.238	6	6	6	100

Protein Group

RecName: Full=Transketolase; Short=TK	gi|1729977	74827.1

Peptide Information

Calc. Mass	Obsrv. Mass	± da	± ppm	Start Seq.	End Seq.	Sequence	Ion Score	C. I. %	Modification	Plate [#] Name	Gel Idx/Pos [4700 Sample Name]	iTRAQ Ratio 115/114*	iTRAQ Ratio 116/114*	iTRAQ Ratio 117/114*	Rank	Result Type
1235.6704	1235.6445	-0.0259	-21	335	342	MPTPPNYK	33	98.807	(N-term)_iTRAQ[0], Lysine(K)_iTRAQ[8]	[7] F5 120912	155/147	1.501	1.365	1.356	1	Mascot
1546.8713	1546.8326	-0.0387	-25	207	218	LDNLVAIFDINR	32	98.387	(N-term)_iTRAQ[0]	[1] F3 030912	501/493	1.104	0.432	1.242	1	Mascot
1967.1371	1967.0742	-0.0629	-32	636	649	MFGIDKDAIVQAVK	63	99.999	(N-term)_iTRAQ[0], Lysine(K)_iTRAQ[6,14]	[6] F8 110912	367/359	1.144	0.956	1.160	1	Mascot
1967.1371	1967.1104	-0.0267	-14	636	649	MFGIDKDAIVQAVK	37	99.535	(N-term)_iTRAQ[0], Lysine(K)_iTRAQ[6,14]	[4] F7 and F10+11	368/360	1.012	0.934	0.825	1	Mascot
2203.1201	2203.0352	-0.0849	-39	471	488	SVPMSTVFYPSDGVATEK	94	100	(N-term)_iTRAQ[0], Lysine(K)_iTRAQ[18]	[1] F3 030912	308/300	1.935	1.624	1.091	1	Mascot
2297.2056	2297.1531	-0.0525	-23	297	313	NMAEQIIQEIYSQVQSK	83	100	(N-term)_iTRAQ[0], Lysine(K)_iTRAQ[17]	[1] F3 030912	549/541	1.139	0.992	0.784	1	Mascot

214	stress-induced-phosphoprotein 1 [Rattus norvegicus]	gi|20302113	72114.7	8	304	1.090	0.856	0.905	0.266	0.261	0.146	8	8	8	100

Peptide Information

Calc. Mass	Obsrv. Mass	± da	± ppm	Start Seq.	End Seq.	Sequence	Ion Score	C. I. %	Modification	Plate [#] Name	Gel Idx/Pos [4700 Sample Name]	iTRAQ Ratio 115/114*	iTRAQ Ratio 116/114*	iTRAQ Ratio 117/114*	Rank	Result Type
1244.7521	1244.7186	-0.0335	-27	534	543	LMDVGLIAIR	34	98.968	(N-term)_iTRAQ[0]	[1] F3 030912	402/394	1.468	0.530	0.991	1	Mascot
1290.7701	1290.7155	-0.0546	-42	506	513	LILEQMQK	41	99.816	(N-term)_iTRAQ[0], Lysine(K)_iTRAQ[8]	[7] F5 120912	237/229	0.815	0.709	1.033	1	Mascot
1425.7948	1425.7065	-0.0883	-62	514	523	DPQALSEHLK	36	99.434	(N-term)_iTRAQ[0], Lysine(K)_iTRAQ[10]	[6] F8 110912	193/185	0.886	0.807	0.791	1	Mascot
1599.8363	1599.8086	-0.0277	-17	33	44	LDPQNHVLYSNR	34	99.063	(N-term)_iTRAQ[0]	[6] F8 110912	197/189	0.916	0.842	0.840	1	Mascot
1730.9601	1731.0381	0.078	45	316	325	YKDAIHFYNK	32	98.477	(N-term)_iTRAQ[0], Lysine(K)_iTRAQ[2,10]	[8] F13-15 and F1+2	1514/1506	0.992	1.167	0.854	1	Mascot
2188.1179	2187.9741	-0.1438	-66	208	222	EAKPEPMEEDLPENK	54	99.989	(N-term)_iTRAQ[0], Lysine(K)_iTRAQ[3,15]	[6] F8 110912	197/189	1.060	0.800	0.761	1	Mascot
2245.2776	2245.2378	-0.0398	-18	154	169	ELIEQLQNKPSDLGTK	37	99.495	(N-term)_iTRAQ[0], Lysine(K)_iTRAQ[9,16]	[4] F7 and F10+11	280/272	1.456	1.329	1.222	1	Mascot
2460.3152	2460.4021	0.0869	35	208	223	EAKPEPMEEDLPENKK	37	99.465	(N-term)_iTRAQ[0], Lysine(K)_iTRAQ[3,15,16]	[4] F7 and F10+11	1071/1063	1.338	0.909	0.836	1	Mascot

215	hsc70-interacting protein [Rattus norvegicus]	gi|13592093	45858.5	6	303	0.916	0.943	0.847	0.362	0.240	0.239	6	6	6	100

Peptide Information

Calc. Mass	Obsrv. Mass	± da	± ppm	Start Seq.	End Seq.	Sequence	Ion Score	C. I. %	Modification	Plate [#] Name	Gel Idx/Pos [4700 Sample Name]	iTRAQ Ratio 115/114*	iTRAQ Ratio 116/114*	iTRAQ Ratio 117/114*	Rank	Result Type
1310.666	1310.6292	-0.0368	-28	33	41	EWVESMGGK	55	99.992	(N-term)_iTRAQ[0], Lysine(K)_iTRAQ[9]	[3] F6 and F9	219/211	1.574	1.418	0.902	1	Mascot
1325.7086	1325.7494	0.0408	31	193	202	LLGHWEEAAR	51	99.979	(N-term)_iTRAQ[0]	[3] F6 and F9	1235/1227	1.113	0.864	1.059	1	Mascot
1394.8141	1394.7914	-0.0227	-16	132	141	AIDLFTDAIK	34	98.989	(N-term)_iTRAQ[0], Lysine(K)_iTRAQ[10]	[7] F5 120912	354/346	0.900	0.787	0.677	1	Mascot
1585.7765	1585.7506	-0.0259	-16	18	29	QDPSVLHTEEMR	69	100	(N-term)_iTRAQ[0]	[4] F7 and F10+11	174/166	0.748	1.143	1.024	1	Mascot
1733.932	1733.8251	-0.1069	-62	173	185	AIEINPDSAQPYK	52	99.985	(N-term)_iTRAQ[0], Lysine(K)_iTRAQ[13]	[5] F4	152/144	0.533	0.749	0.545	1	Mascot
1975.1195	1975.1836	0.0641	32	117	131	KGAAIDALNDGELQK	41	99.826	(N-term)_iTRAQ[0], Lysine(K)_iTRAQ[1,15]	[4] F7 and F10+11	1125/1117	0.942	0.850	1.023	1	Mascot

216	hippocalcin-like protein 1 [Mus musculus]	gi|7949055	25009.8	5	302	0.944	0.822	0.854	0.182	0.223	0.437	5	5	5	100

Peptide Information

Calc. Mass	Obsrv. Mass	± da	± ppm	Start Seq.	End Seq.	Sequence	Ion Score	C. I. %	Modification	Plate [#] Name	Gel Idx/Pos [4700 Sample Name]	iTRAQ Ratio 115/114*	iTRAQ Ratio 116/114*	iTRAQ Ratio 117/114*	Rank	Result Type
1049.5652	1049.6108	0.0456	43	64	70	FAEHVFR	28	95.816	(N-term)_iTRAQ[0]	[4] F7 and F10+11	1111/1103	1.163	0.819	1.015	1	Mascot
1606.7992	1606.8385	0.0393	24	138	148	MPEDESTPEKR	31	98.24	(N-term)_iTRAQ[0], Lysine(K)_iTRAQ[10]	[3] F6 and F9	1075/1067	0.953	0.733	0.376	1	Mascot
1632.7626	1632.6801	-0.0825	-51	71	83	TFDTNSDGTIDFR	89	100	(N-term)_iTRAQ[0]	[1] F3 030912	244/236	1.018	0.779	0.986	1	Mascot
1780.9155	1780.8464	-0.0691	-39	51	63	IYANFFPYGDASK	85	100	(N-term)_iTRAQ[0], Lysine(K)_iTRAQ[13]	[5] F4	273/265	0.683	0.625	1.060	1	Mascot
2280.0818	2280.1677	0.0859	38	18	32	EHTEFTDHELQEWYK	69	100	(N-term)_iTRAQ[0], Lysine(K)_iTRAQ[15]	[4] F7 and F10+11	1186/1178	0.974	1.282	1.140	1	Mascot

217	rho GDP-dissociation inhibitor 1 [Mus musculus]	gi|31982030	26320.8	5	300	1.116	1.351	1.005	0.388	0.502	0.153	5	5	5	100

Peptide Information

Calc. Mass	Obsrv. Mass	± da	± ppm	Start Seq.	End Seq.	Sequence	Ion Score	C. I. %	Modification	Plate [#] Name	Gel Idx/Pos [4700 Sample Name]	iTRAQ Ratio 115/114*	iTRAQ Ratio 116/114*	iTRAQ Ratio 117/114*	Rank	Result Type
1389.6593	1389.5762	-0.0831	-60	142	152	TDYMVGSYGPR	30	97.348	(N-term)_iTRAQ[0]	[5] F4	137/129	0.772	2.093	1.056	1	Mascot
1795.0198	1795.041	0.0212	12	59	74	VAVSADPNVPNVIVTR	44	99.894	(N-term)_iTRAQ[0]	[8] F13-15 and F1+2	495/487	0.951	0.923	0.824	1	Mascot
1889.9677	1889.8638	-0.1039	-55	139	152	IDKTDYMVGSYGPR	77	100	(N-term)_iTRAQ[0], Lysine(K)_iTRAQ[3]	[6] F8 110912	250/242	1.099	1.100	1.089	1	Mascot
2072.0144	2071.958	-0.0564	-27	153	167	AEEYEFLTPMEEAPK	80	100	(N-term)_iTRAQ[0], Lysine(K)_iTRAQ[15]	[1] F3 030912	332/324	1.128	1.153	0.890	1	Mascot
2206.1448	2206.1194	-0.0254	-12	34	49	SIQEIQELDKDDESLR	70	100	(N-term)_iTRAQ[0], Lysine(K)_iTRAQ[10]	[4] F7 and F10+11	284/276	1.902	1.839	1.218	1	Mascot

218	neural cell adhesion molecule long domain form, sprouting neuron - rat (fragment)	gi|281037	46504	5	299	1.260	1.248	1.313	0.476	0.454	0.478	6	6	6	100

Peptide Information

Calc. Mass	Obsrv. Mass	± da	± ppm	Start Seq.	End Seq.	Sequence	Ion Score	C. I. %	Modification	Plate [#] Name	Gel Idx/Pos [4700 Sample Name]	iTRAQ Ratio 115/114*	iTRAQ Ratio 116/114*	iTRAQ Ratio 117/114*	Rank	Result Type
1588.8792	1588.7762	-0.103	-65	74	84	DESKEPIVEVR	35	99.233	(N-term)_iTRAQ[0], Lysine(K)_iTRAQ[4]	[6] F8 110912	175/167	1.319	1.314	0.965	1	Mascot
1626.8949	1626.7991	-0.0958	-59	285	297	GLDPEPTQPGTVK	62	99.998	(N-term)_iTRAQ[0], Lysine(K)_iTRAQ[13]	[5] F4	111/103	0.803	0.726	0.840	1	Mascot
1659.9164	1659.8293	-0.0871	-52	182	196	GVTASSSSPPASVPK	52	99.986	(N-term)_iTRAQ[0], Lysine(K)_iTRAQ[15]	[5] F4	88/80	1.470	1.123	1.628	1	Mascot
1918.9968	1918.892	-0.1048	-55	313	328	APSVSTTNPSQGEDLK	77	100	(N-term)_iTRAQ[0], Lysine(K)_iTRAQ[16]	[5] F4	109/101	1.328	1.449	1.449	1	Mascot
2159.1567	2158.9878	-0.1689	-78	390	405	SEPQESEAKPAPTEVK	37	99.483	(N-term)_iTRAQ[0], Lysine(K)_iTRAQ[9,16]	[6] F8 110912	129/121	2.132	2.050	2.113	1	Mascot
2159.1567	2159.0759	-0.0808	-37	390	405	SEPQESEAKPAPTEVK	73	100	(N-term)_iTRAQ[0], Lysine(K)_iTRAQ[9,16]	[4] F7 and F10+11	125/117	0.908	1.189	1.271	1	Mascot

219	trifunctional enzyme subunit alpha, mitochondrial precursor [Rattus norvegicus]	gi|148747393	92963.3	6	298	0.963	1.100	0.815	0.232	0.251	0.246	6	6	6	100

Protein Group

mitochondrial long-chain enoyl-CoA hydratase/3-hydroxycyl-CoA dehydrogenase alpha-subunit [Rattus n	gi|510108	92857.4

Peptide Information

Calc. Mass	Obsrv. Mass	± da	± ppm	Start Seq.	End Seq.	Sequence	Ion Score	C. I. %	Modification	Plate [#] Name	Gel Idx/Pos [4700 Sample Name]	iTRAQ Ratio 115/114*	iTRAQ Ratio 116/114*	iTRAQ Ratio 117/114*	Rank	Result Type
1286.7566	1286.7158	-0.0408	-32	561	569	ILQEGVDPK	41	99.794	(N-term)_iTRAQ[0], Lysine(K)_iTRAQ[9]	[7] F5 120912	164/156	0.622	0.716	0.602	1	Mascot
1437.7623	1437.7059	-0.0564	-39	635	644	GFYIYQSGSK	77	100	(N-term)_iTRAQ[0], Lysine(K)_iTRAQ[10]	[7] F5 120912	195/187	1.206	1.216	1.118	1	Mascot
1739.8767	1739.8705	-0.0062	-4	191	205	MVGVPAAFDMMLTGR	35	99.14	(N-term)_iTRAQ[0]	[8] F13-15 and F1+2	298/290	0.943	1.179	0.957	1	Mascot
1901.0266	1900.9017	-0.1249	-66	423	436	DSIFSNLIGQLDYK	38	99.604	(N-term)_iTRAQ[0], Lysine(K)_iTRAQ[14]	[5] F4	379/371	0.952	1.049	0.904	1	Mascot
2070.0237	2069.8821	-0.1416	-68	310	326	TGLEQGNDAGYLAESEK	80	100	(N-term)_iTRAQ[0], Lysine(K)_iTRAQ[17]	[5] F4	144/136	1.029	1.344	0.538	1	Mascot
2339.1755	2339.1746	-0.0009	0	735	751	KYESAYGTQFTPCQLLR	28	96.087	(N-term)_iTRAQ[0], Lysine(K)_iTRAQ[1], MMTS (C)[13]	[4] F7 and F10+11	422/414	1.152	1.225	0.935	1	Mascot

220	mitochondrial aldehyde dehydrogenase [Rattus norvegicus]	gi|25990263	58321	6	297	0.802	1.063	1.004	0.440	0.791	0.805	8	8	8	100

Protein Group

aldehyde dehydrogenase [Rattus norvegicus]	gi|16073616	52740.3
aldehyde dehydrogenase, mitochondrial precursor [Rattus norvegicus]	gi|14192933	61477.8
mitochondrial aldehyde dehydrogenase precursor [Rattus norvegicus]	gi|45737864	60563.3

Peptide Information

Calc. Mass	Obsrv. Mass	± da	± ppm	Start Seq.	End Seq.	Sequence	Ion Score	C. I. %	Modification	Plate [#] Name	Gel Idx/Pos [4700 Sample Name]	iTRAQ Ratio 115/114*	iTRAQ Ratio 116/114*	iTRAQ Ratio 117/114*	Rank	Result Type
1261.6462	1261.6189	-0.0273	-22	119	126	YYAGWADK	29	96.877	(N-term)_iTRAQ[0], Lysine(K)_iTRAQ[8]	[3] F6 and F9	234/226	0.913	1.035	1.272	1	Mascot
1546.8251	1546.7543	-0.0708	-46	53	65	AAQAAFQLGSPWR	63	99.999	(N-term)_iTRAQ[0]	[5] F4	247/239	0.418	0.296	0.470	1	Mascot
1691.9652	1691.9044	-0.0608	-36	386	397	EEIFGPVMQILK	62	99.999	(N-term)_iTRAQ[0], Lysine(K)_iTRAQ[12]	[1] F3 030912	473/465	0.589	1.452	1.226	1	Mascot
1758.9677	1758.8774	-0.0903	-51	366	378	GYFIQPTVFGDVK	51	99.981	(N-term)_iTRAQ[0], Lysine(K)_iTRAQ[13]	[5] F4	283/275	1.234	1.336	1.626	1	Mascot
1758.9677	1758.908	-0.0597	-34	366	378	GYFIQPTVFGDVK	63	99.999	(N-term)_iTRAQ[0], Lysine(K)_iTRAQ[13]	[1] F3 030912	371/363	0.532	0.830	0.320	1	Mascot
1794.912	1794.8044	-0.1076	-60	327	339	TEQGPQVDETQFK	27	95.402	(N-term)_iTRAQ[0], Lysine(K)_iTRAQ[13]	[5] F4	115/107	1.723	1.551	1.771	1	Mascot
1887.995	1887.8922	-0.1028	-54	464	477	ELGEYGLQAYTEVK	46	99.942	(N-term)_iTRAQ[0], Lysine(K)_iTRAQ[14]	[5] F4	238/230	0.970	2.122	1.631	1	Mascot
1887.995	1887.9086	-0.0864	-46	464	477	ELGEYGLQAYTEVK	52	99.985	(N-term)_iTRAQ[0], Lysine(K)_iTRAQ[14]	[1] F3 030912	319/311	0.694	1.008	0.939	1	Mascot

221	RII-B-binding protein [Rattus norvegicus]	gi|557585	51909.2	4	294	0.692	0.942	1.013	0.115	0.164	0.104	4	4	4	100

Peptide Information

Calc. Mass	Obsrv. Mass	± da	± ppm	Start Seq.	End Seq.	Sequence	Ion Score	C. I. %	Modification	Plate [#] Name	Gel Idx/Pos [4700 Sample Name]	iTRAQ Ratio	iTRAQ Ratio	iTRAQ Ratio	Rank	Result Type

Calc. Mass	Obsrv. Mass	± da	± ppm	Start Seq.	End Seq.	Sequence	Ion Score	C. I. %	Modification	Plate [#] Name	Gel Idx/Pos [4700 Sample Name]	iTRAQ Ratio 115/114*	iTRAQ Ratio 116/114*	iTRAQ Ratio 117/114*	Rank	Result Type
1633.9006	1633.7593	-0.1413	-86	100	112	LSQAEEATVAQAK	42	99.843	(N-term)_iTRAQ[0], Lysine(K)_iTRAQ[13]	[5] F4	129/121	0.674	0.858	0.939	1	Mascot
1701.9633	1701.885	-0.0783	-46	124	136	LSQIEEPAISQAK	87	100	(N-term)_iTRAQ[0], Lysine(K)_iTRAQ[13]	[5] F4	160/152	0.776	1.072	1.051	1	Mascot
1960.0055	1959.9912	-0.0143	-7	162	177	ATMGQAEEATVGHIEK	74	100	(N-term)_iTRAQ[0], Lysine(K)_iTRAQ[16]	[4] F7 and F10+11	230/222	0.543	0.760	0.913	1	Mascot
2300.2483	2300.1809	-0.0674	-29	421	438	TSEQYETLLIETASSLVK	91	100	(N-term)_iTRAQ[0], Lysine(K)_iTRAQ[18]	[1] F3 030912	523/515	0.805	1.125	1.167	1	Mascot

222	protein piccolo isoform 2 [Rattus norvegicus]	gi|160707980	584432.8	5	292	0.833	0.815	0.819	0.208	0.019	0.185	5	5	5	100

Protein Group

protein piccolo isoform 1 [Rattus norvegicus] gi|10048483 610047.4

Peptide Information

Calc. Mass	Obsrv. Mass	± da	± ppm	Start Seq.	End Seq.	Sequence	Ion Score	C. I. %	Modification	Plate [#] Name	Gel Idx/Pos [4700 Sample Name]	iTRAQ Ratio 115/114*	iTRAQ Ratio 116/114*	iTRAQ Ratio 117/114*	Rank	Result Type
1663.8578	1663.7896	-0.0682	-41	3641	3652	APFQYSEGFTTK	66	99.999	(N-term)_iTRAQ[0], Lysine(K)_iTRAQ[12]	[7] F5 120912	233/225	0.978	0.781	0.896	1	Mascot
1795.9147	1795.8033	-0.1114	-62	1942	1953	LQEQIYDDPMQK	47	99.955	(N-term)_iTRAQ[0], Lysine(K)_iTRAQ[12]	[5] F4	171/163	0.568	0.822	0.575	1	Mascot
1998.9978	1998.865	-0.1328	-66	4219	4234	SALQDEADKPYSSGSR	53	99.988	(N-term)_iTRAQ[0], Lysine(K)_iTRAQ[9]	[6] F8 110912	152/144	1.015	0.809	1.017	1	Mascot
2079.0828	2078.9858	-0.097	-47	3730	3743	QAELDEEEKEIDAK	65	99.999	(N-term)_iTRAQ[0], Lysine(K)_iTRAQ[9,14]	[6] F8 110912	210/202	0.737	0.836	0.747	1	Mascot
2258.2363	2258.2271	-0.0092	-4	953	970	AAAAENLESKPEQAPTAK	63	99.999	(N-term)_iTRAQ[0], Lysine(K)_iTRAQ[10, 18]	[4] F7 and F10+11	165/157	0.964	0.825	0.941	1	Mascot

223	succinate semialdehyde dehydrogenase [Rattus norvegicus]	gi|556395	57325	6	292	1.055	1.279	1.287	1.797	1.237	1.708	6	6	6	100

Peptide Information

Calc. Mass	Obsrv. Mass	± da	± ppm	Start Seq.	End Seq.	Sequence	Ion Score	C. I. %	Modification	Plate [#] Name	Gel Idx/Pos [4700 Sample Name]	iTRAQ Ratio 115/114*	iTRAQ Ratio 116/114*	iTRAQ Ratio 117/114*	Rank	Result Type
1433.7311	1433.7013	-0.0298	-21	57	66	AAYDAFSSWK	58	99.996	(N-term)_iTRAQ[0], Lysine(K)_iTRAQ[10]	[3] F6 and F9	316/308	0.805	1.008	1.027	1	Mascot
1477.8247	1477.8073	-0.0174	-12	1	13	VGGPADLHADLLR	30	97.609	(N-term)_iTRAQ[0]	[4] F7 and F10+11	270/262	7.314	5.465	8.137	1	Mascot
1490.9053	1490.8414	-0.0639	-43	244	254	ILLHHAANSVK	44	99.901	(N-term)_iTRAQ[0], Lysine(K)_iTRAQ[11]	[2] F12 040912	189/181	0.877	0.695	0.824	1	Mascot
1516.8145	1516.733	-0.0815	-54	472	481	YGIDEYLEVK	41	99.8	(N-term)_iTRAQ[0], Lysine(K)_iTRAQ[10]	[5] F4	242/234	0.914	1.153	0.842	1	Mascot
2148.0957	2148.0405	-0.0552	-26	366	383	HQSGGNFFEPTLLSNVTR	42	99.861	(N-term)_iTRAQ[0]	[2] F12 040912	372/364	0.252	0.822	0.663	1	Mascot
2199.1855	2199.0708	-0.1147	-52	80	93	WYDLMIQNKDELAK	78	100	(N-term)_iTRAQ[0], Lysine(K)_iTRAQ[9,14]	[6] F8 110912	393/385	1.158	1.205	1.183	1	Mascot

224	Chain A, Structural Basis Of Rab Effector Specificity: Crystal Structure Of The Small G Protein Rab	gi|4557959	24963.4	4	291	0.829	1.031	1.044	0.271	0.198	0.134	5	5	5	100

Peptide Information

Calc. Mass	Obsrv. Mass	± da	± ppm	Start Seq.	End Seq.	Sequence	Ion Score	C. I. %	Modification	Plate [#] Name	Gel Idx/Pos [4700 Sample Name]	iTRAQ Ratio 115/114*	iTRAQ Ratio 116/114*	iTRAQ Ratio 117/114*	Rank	Result Type
1252.6871	1252.6587	-0.0284	-23	165	172	LVDVICEK	49	99.971	(N-term)_iTRAQ[0], Lysine(K)_iTRAQ[8], MMTS (C)[6]	[7] F5 120912	330/322	0.862	0.965	1.169	1	Mascot
1880.9521	1880.8672	-0.0849	-45	173	188	XSESLDTADPAVTGAK	94	100	(N-term)_iTRAQ[0], Lysine(K)_iTRAQ[16]	[1] F3 030912	213/205	0.723	0.821	0.944	1	Mascot
1996.075	1995.9971	-0.0779	-39	108	122	TYSWDNAQVLLVGNK	43	99.865	(N-term)_iTRAQ[0], Lysine(K)_iTRAQ[15]	[5] F4	270/262	0.549	1.082	0.883	1	Mascot
2098.0854	2098.0007	-0.0847	-40	138	153	QLADHLGFEFFEASAK	60	99.997	(N-term)_iTRAQ[0], Lysine(K)_iTRAQ[16]	[6] F8 110912	421/413	1.307	1.398	1.210	1	Mascot
2098.0854	2098.1074	0.022	10	138	153	QLADHLGFEFFEASAK	106	100	(N-term)_iTRAQ[0], Lysine(K)_iTRAQ[16]	[4] F7 and F10+11	419/411	0.872	0.973	1.052	1	Mascot

225	calreticulin precursor [Rattus norvegicus]	gi|11693172	54300.2	4	291	1.102	1.097	1.141	0.163	0.160	0.159	5	5	5	100

Peptide Information

Calc. Mass	Obsrv. Mass	± da	± ppm	Start Seq.	End Seq.	Sequence	Ion Score	C. I. %	Modification	Plate [#] Name	Gel Idx/Pos [4700 Sample Name]	iTRAQ Ratio 115/114*	iTRAQ Ratio 116/114*	iTRAQ Ratio 117/114*	Rank	Result Type
1262.7355	1262.6843	-0.0512	-41	112	120	LFPGGLDQK	55	99.992	(N-term)_iTRAQ[0], Lysine(K)_iTRAQ[9]	[7] F5 120912	225/217	0.892	1.087	1.286	1	Mascot

1595.7574	1595.6885	-0.0689	-43	25	36	EQFLDGDAWTNR	79	100	(N-term)_iTRAQ[0]	[5] F4	209/201	1.062	0.858	1.020	1	Mascot
1753.8229	1753.9128	0.0899	51	99	111	HEQNIDCGGGYVK	86	100	(N-term)_iTRAQ[0], Lysine(K)_iTRAQ[13], MMTS (C)[7]	[4] F7 and F10+11	1094/1086	1.362	1.283	0.973	1	Mascot
2217.1523	2217.1296	-0.0227	-10	208	222	IKDPDAAKPEDWDER	68	100	(N-term)_iTRAQ[0], Lysine(K)_iTRAQ[2,8]	[2] F12 040912	218/210	1.171	1.196	1.109	1	Mascot
2217.1523	2217.2529	0.1006	45	208	222	IKDPDAAKPEDWDER	71	100	(N-term)_iTRAQ[0], Lysine(K)_iTRAQ[2,8]	[4] F7 and F10+11	1094/1086	1.076	1.112	1.364	1	Mascot

226	transgelin-3 [Rattus norvegicus]	gi|78214333	24641.6	5	291	1.092	1.197	0.958	0.248	0.299	0.337	5	5	5	100

Peptide Information

Calc. Mass	Obsrv. Mass	± da	± ppm	Start Seq.	End Seq.	Sequence	Ion Score	C. I. %	Modification	Plate [#] Name	Gel Idx/Pos [4700 Sample Name]	iTRAQ Ratio 115/114*	iTRAQ Ratio 116/114*	iTRAQ Ratio 117/114*	Rank	Result Type
1499.7651	1499.7085	-0.0566	-38	55	64	WLMDGTVLCK	40	99.753	(N-term)_iTRAQ[0], Lysine(K)_iTRAQ[10], MMTS (C)[9]	[5] F4	326/318	0.836	1.015	0.661	1	Mascot
1527.7168	1527.6337	-0.0831	-54	183	196	GASQAGMTGYGMPR	72	100	(N-term)_iTRAQ[0]	[1] F3 030912	192/184	0.882	0.838	0.866	1	Mascot
1539.8451	1539.8337	-0.0114	-7	89	98	QMEQISQFLK	68	100	(N-term)_iTRAQ[0], Lysine(K)_iTRAQ[10]	[7] F5 120912	310/302	1.370	1.460	1.247	1	Mascot
1761.9528	1761.8629	-0.0899	-51	169	182	QGQNVIGLQMGSNK	72	100	(N-term)_iTRAQ[0], Lysine(K)_iTRAQ[14]	[7] F5 120912	180/172	1.162	1.454	0.767	1	Mascot
1941.0215	1940.9149	-0.1066	-55	107	120	TTDIFQTVDLWEGK	39	99.707	(N-term)_iTRAQ[0], Lysine(K)_iTRAQ[14]	[5] F4	339/331	1.323	1.361	1.477	1	Mascot

227	dihydrolipoamide acetyltransferase [Rattus norvegicus]	gi|220838	63188	5	290	1.064	1.031	1.175	0.252	0.293	0.270	5	5	5	100

Peptide Information

Calc. Mass	Obsrv. Mass	± da	± ppm	Start Seq.	End Seq.	Sequence	Ion Score	C. I. %	Modification	Plate [#] Name	Gel Idx/Pos [4700 Sample Name]	iTRAQ Ratio 115/114*	iTRAQ Ratio 116/114*	iTRAQ Ratio 117/114*	Rank	Result Type
1148.7289	1148.6688	-0.0601	-52	250	257	VFVSPLAK	34	99.048	(N-term)_iTRAQ[0], Lysine(K)_iTRAQ[8]	[7] F5 120912	227/219	1.281	1.527	1.171	1	Mascot
1778.0157	1777.9584	-0.0573	-32	427	441	GLETIASDVVSLASK	90	100	(N-term)_iTRAQ[0], Lysine(K)_iTRAQ[15]	[1] F3 030912	489/481	1.291	1.244	1.403	1	Mascot
1831.0299	1830.9755	-0.0544	-30	182	195	DVPLGTPLCIIVEK	54	99.991	(N-term)_iTRAQ[0], Lysine(K)_iTRAQ[14], MMTS (C)[9]	[1] F3 030912	485/477	1.087	0.871	1.052	1	Mascot
1878.0371	1877.9425	-0.0946	-50	516	530	VVDGAVGAQWLAEFK	78	100	(N-term)_iTRAQ[0], Lysine(K)_iTRAQ[15]	[5] F4	338/330	1.055	0.902	1.521	1	Mascot
2024.1664	2024.1819	0.0155	8	306	323	VAPTPAGVFIDIPISNIR	34	99.015	(N-term)_iTRAQ[0]	[8] F13-15 and F1+2	361/353	0.719	0.782	0.851	1	Mascot

228	Ca2+/calmodulin-dependent protein kinase II isoform gamma-b [Rattus sp.]	gi|560651	64669.6	6	290	1.165	1.054	1.165	0.254	0.330	0.288	6	6	6	100

Protein Group

Ca2+/calmodulin-dependent protein kinase II gamma-c [Rattus sp.]	gi|560653	61723
calcium/calmodulin-dependent protein kinase type II subunit gamma [Rattus norvegicus]	gi|19424316	65028.8

Peptide Information

Calc. Mass	Obsrv. Mass	± da	± ppm	Start Seq.	End Seq.	Sequence	Ion Score	C. I. %	Modification	Plate [#] Name	Gel Idx/Pos [4700 Sample Name]	iTRAQ Ratio 115/114*	iTRAQ Ratio 116/114*	iTRAQ Ratio 117/114*	Rank	Result Type
1080.6664	1080.6154	-0.051	-47	222	227	LYQQIK	33	98.745	(N-term)_iTRAQ[0], Lysine(K)_iTRAQ[6]	[3] F6 and F9	190/182	1.043	1.183	1.117	1	Mascot
1547.8414	1547.7534	-0.088	-57	474	485	LTQYIDGQGRPR	30	97.727	(N-term)_iTRAQ[0]	[6] F8 110912	229/221	1.203	1.127	1.403	1	Mascot
1773.0857	1772.9882	-0.0975	-55	136	147	DLKPENLLLASK	49	99.967	(N-term)_iTRAQ[0], Lysine(K)_iTRAQ[3,12]	[6] F8 110912	295/287	1.190	1.424	1.239	1	Mascot
1892.9528	1892.889	-0.0638	-34	10	22	FTDDYQLFEELGK	33	98.785	(N-term)_iTRAQ[0], Lysine(K)_iTRAQ[13]	[5] F4	317/309	1.655	0.697	1.611	1	Mascot
2063.8977	2063.7966	-0.1011	-49	369	384	GSTESCNTTTEDEDLK	77	100	(N-term)_iTRAQ[0], Lysine(K)_iTRAQ[16], MMTS (C)[6]	[5] F4	112/104	0.854	0.767	0.840	1	Mascot
2369.1548	2369.0718	-0.083	-35	228	246	AGAYDFPSPEWDTVTPEAK	68	100	(N-term)_iTRAQ[0], Lysine(K)_iTRAQ[19]	[1] F3 030912	337/329	1.184	1.355	0.954	1	Mascot

229	protein phosphatase 1 regulatory subunit 7 [Rattus norvegicus]	gi|57634526	45110.1	4	285	0.835	0.964	0.889	0.573	0.265	0.418	4	4	4	100

Peptide Information

Calc. Mass	Obsrv. Mass	± da	± ppm	Start Seq.	End Seq.	Sequence	Ion Score	C. I. %	Modification	Plate [#] Name	Gel Idx/Pos [4700 Sample Name]	iTRAQ Ratio 115/114*	iTRAQ Ratio 116/114*	iTRAQ Ratio 117/114*	Rank	Result Type
1412.8345	1412.797	-0.0375	-27	245	255	IEGLQNLVNLR	42	99.843	(N-term)_iTRAQ[0]	[1] F3 030912	382/374	1.396	1.350	1.267	1	Mascot

1538.8312	1538.7701	-0.0611	-40	124	133	ELDLYDNQIK	42	99.841	(N-term)_iTRAQ[0], Lysine(K)_iTRAQ[10]	[7] F5 120912	220/212	1.008	0.835	0.895	1	Mascot
1837.8292	1837.7385	-0.0907	-49	6	20	GAGQQQSQEMMEVDR	107	100	(N-term)_iTRAQ[0]	[1] F3 030912	171/163	0.992	1.074	1.163	1	Mascot
2279.2744	2279.2241	-0.0503	-22	200	217	AIENIDTLTNLESLFLGK	95	100	(N-term)_iTRAQ[0], Lysine(K)_iTRAQ[18]	[1] F3 030912	548/540	0.349	0.713	0.474	1	Mascot

230	annexin A6 [Rattus norvegicus]	gi|130502086	84100.1	7	283	0.950	0.807	0.609	0.367	0.152	0.374	7	7	7	100

Protein Group

RecName: Full=Annexin A6; AltName: Full=Annexin VI; AltName: Full=Annexin-6; AltName: Full=Calcium-	gi|1351943	84098.1

Peptide Information

Calc. Mass	Obsrv. Mass	± da	± ppm	Start Seq.	End Seq.	Sequence	Ion Score	C. I. %	Modification	Plate [#] Name	Gel Idx/Pos [4700 Sample Name]	iTRAQ Ratio 115/114*	iTRAQ Ratio 116/114*	iTRAQ Ratio 117/114*	Rank	Result Type
1198.7081	1198.7064	-0.0017	-1	562	568	VFQEFIK	29	96.544	(N-term)_iTRAQ[0], Lysine(K)_iTRAQ[7]	[7] F5 120912	287/279	0.740	0.800	0.676	1	Mascot
1314.7555	1314.7274	-0.0281	-21	213	220	LVFDEYLK	30	97.267	(N-term)_iTRAQ[0], Lysine(K)_iTRAQ[8]	[7] F5 120912	336/328	0.939	1.104	0.412	1	Mascot
1354.8093	1354.8569	0.0476	35	561	568	RVFQEFIK	29	97.21	(N-term)_iTRAQ[0], Lysine(K)_iTRAQ[8]	[4] F7 and F10+11	1176/1168	1.289	0.776	1.314	1	Mascot
1464.8672	1464.786	-0.0812	-55	588	598	DAFVAIVQSVK	53	99.988	(N-term)_iTRAQ[0], Lysine(K)_iTRAQ[11]	[5] F4	292/284	0.951	0.893	0.299	1	Mascot
1913.014	1912.9684	-0.0456	-24	472	483	AINEAYKEDYHK	52	99.983	(N-term)_iTRAQ[0], Lysine(K)_iTRAQ[7,12]	[2] F12 040912	170/162	1.146	0.852	0.848	1	Mascot
1958.0853	1958.0608	-0.0245	-13	428	442	LILGLMMPPAHYDAK	40	99.757	(N-term)_iTRAQ[0], Lysine(K)_iTRAQ[15]	[3] F6 and F9	430/422	0.505	0.616	0.413	1	Mascot
2040.1223	2040.1527	0.0304	15	35	50	GFGSDKESILELITSR	52	99.983	(N-term)_iTRAQ[0], Lysine(K)_iTRAQ[6]	[4] F7 and F10+11	466/458	1.412	0.693	0.808	1	Mascot

231	chaperonin 10, cpn10 [Rattus norvegicus=rats, liver, Peptide Mitochondrial, 101 aa]	gi|400542	12493	5	284	0.844	0.861	0.982	0.193	0.174	0.177	7	7	7	100

Protein Group

RecName: Full=10 kDa heat shock protein, mitochondrial; Short=Hsp10; AltName: Full=10 kDa chaperoni	gi|461731	12624.1

Peptide Information

Calc. Mass	Obsrv. Mass	± da	± ppm	Start Seq.	End Seq.	Sequence	Ion Score	C. I. %	Modification	Plate [#] Name	Gel Idx/Pos [4700 Sample Name]	iTRAQ Ratio 115/114*	iTRAQ Ratio 116/114*	iTRAQ Ratio 117/114*	Rank	Result Type
1051.6061	1051.549	-0.0571	-54	8	14	FLPLFDR	31	98.14	(N-term)_iTRAQ[0]	[5] F4	305/297	0.756	0.656	0.905	1	Mascot
1323.8035	1323.8528	0.0493	37	7	14	KFLPLFDR	29	96.7	(N-term)_iTRAQ[0], Lysine(K)_iTRAQ[1]	[4] F7 and F10+11	1231/1223	0.805	1.011	1.159	1	Mascot
1364.8035	1364.729	-0.0745	-55	70	79	VLLPEYGGTK	33	98.894	(N-term)_iTRAQ[0], Lysine(K)_iTRAQ[10]	[5] F4	170/162	0.646	0.673	0.882	1	Mascot
1364.8035	1364.7817	-0.0218	-16	70	79	VLLPEYGGTK	56	99.993	(N-term)_iTRAQ[0], Lysine(K)_iTRAQ[10]	[7] F5 120912	212/204	0.743	0.979	0.715	1	Mascot
1573.9523	1573.8579	-0.0944	-60	40	53	VLQATVVAVGSGGK	107	100	(N-term)_iTRAQ[0], Lysine(K)_iTRAQ[14]	[5] F4	193/185	1.114	0.823	1.069	1	Mascot
1818.0048	1817.9064	-0.0984	-54	80	91	VVLDDKDYFLFR	61	99.998	(N-term)_iTRAQ[0], Lysine(K)_iTRAQ[6]	[6] F8 110912	401/393	1.184	1.095	1.182	1	Mascot
1818.0048	1817.9974	-0.0074	-4	80	91	VVLDDKDYFLFR	54	99.99	(N-term)_iTRAQ[0], Lysine(K)_iTRAQ[6]	[4] F7 and F10+11	397/389	0.792	0.894	1.051	1	Mascot

232	reticulon-4 [Rattus norvegicus]	gi|13929188	137629.8	5	281	1.361	1.180	1.063	0.258	0.235	0.094	6	6	6	100

Peptide Information

Calc. Mass	Obsrv. Mass	± da	± ppm	Start Seq.	End Seq.	Sequence	Ion Score	C. I. %	Modification	Plate [#] Name	Gel Idx/Pos [4700 Sample Name]	iTRAQ Ratio 115/114*	iTRAQ Ratio 116/114*	iTRAQ Ratio 117/114*	Rank	Result Type
1387.7817	1387.8184	0.0367	26	92	104	GPLPAAPPAAPER	27	95.219	(N-term)_iTRAQ[0]	[8] F13-15 and F1+2	586/578	1.273	1.225	0.947	1	Mascot
1387.7817	1387.8419	0.0602	43	92	104	GPLPAAPPAAPER	30	97.604	(N-term)_iTRAQ[0]	[8] F13-15 and F1+2	589/581	1.184	0.960	0.988	1	Mascot
1706.7742	1706.7362	-0.038	-22	901	913	DEVHVSDEFSENR	45	99.927	(N-term)_iTRAQ[0]	[4] F7 and F10+11	186/178	1.056	0.961	1.057	1	Mascot
1924.0651	1924.0132	-0.0519	-27	1129	1142	HQVQIDHYLGLANK	97	100	(N-term)_iTRAQ[0], Lysine(K)_iTRAQ[14]	[2] F12 040912	294/286	1.579	1.120	1.055	1	Mascot
2357.1104	2357.0193	-0.0911	-39	276	293	DLAEFSELEYSEMGSSFK	57	99.995	(N-term)_iTRAQ[0], Lysine(K)_iTRAQ[18]	[1] F3 030912	430/422	1.763	1.326	1.232	1	Mascot
2448.2458	2448.1077	-0.1381	-56	364	379	EEYADFKPFEQAWEVK	52	99.984	(N-term)_iTRAQ[0], Lysine(K)_iTRAQ[7,16]	[6] F8 110912	407/399	1.439	1.607	1.119	1	Mascot

233	RecName: Full=Guanine nucleotide-binding protein G(olf) subunit alpha; AltName: Full=Adenylate cycl	gi|2851469	48813.2	5	281	0.789	1.015	1.083	0.263	0.241	0.199	5	5	5	100

Peptide Information

Calc. Mass	Obsrv. Mass	± da	± ppm	Start	End	Sequence	Ion	C. I. %	Modification	Plate [#] Name	Gel Idx/Pos [4700	iTRAQ	iTRAQ	iTRAQ	Rank	Result Type

Calc. Mass	Obsrv. Mass	± da	± ppm	Start Seq.	End Seq.	Sequence	Ion Score	C. I. %	Modification	Plate [#] Name	Gel Idx/Pos [4700 Sample Name]	iTRAQ Ratio 115/114*	iTRAQ Ratio 116/114*	iTRAQ Ratio 117/114*	Rank	Result Type
1249.6675	1249.6285	-0.039	-31	140	147	LWDDEGVK	40	99.768	(N-term)_iTRAQ[0], Lysine(K)_iTRAQ[8]	[3] F6 and F9	234/226	0.488	0.923	0.982	1	Mascot
1345.8301	1345.7644	-0.0657	-49	45	55	LLLLGAGESGK	84	100	(N-term)_iTRAQ[0], Lysine(K)_iTRAQ[11]	[5] F4	219/211	1.116	1.118	1.283	1	Mascot
1521.8649	1521.9462	0.0813	53	139	147	KLWDDEGVK	31	97.884	(N-term)_iTRAQ[0], Lysine(K)_iTRAQ[1,9]	[4] F7 and F10+11	1104/1096	0.713	0.760	0.850	1	Mascot
1550.7657	1550.8171	0.0514	33	204	215	VNFHMFDVGGQR	59	99.997	(N-term)_iTRAQ[0]	[3] F6 and F9	1279/1271	0.788	0.955	1.043	1	Mascot
1766.8833	1766.8252	-0.0581	-33	255	267	ESLDLFESIWNNR	67	100	(N-term)_iTRAQ[0]	[1] F3 030912	488/480	0.997	1.436	1.335	1	Mascot

234	calmodulin [Homo sapiens]	gi|4502549	18123.8	4	281	1.093	0.941	1.156	0.346	0.260	0.721	6	6	6	100

Protein Group

calmodulin - salmon	gi|71664	17992.7

Peptide Information

Calc. Mass	Obsrv. Mass	± da	± ppm	Start Seq.	End Seq.	Sequence	Ion Score	C. I. %	Modification	Plate [#] Name	Gel Idx/Pos [4700 Sample Name]	iTRAQ Ratio 115/114*	iTRAQ Ratio 116/114*	iTRAQ Ratio 117/114*	Rank	Result Type
1640.8047	1640.8135	0.0088	5	77	87	MKDTDSEEEIR	69	100	(N-term)_iTRAQ[0], Lysine(K)_iTRAQ[2]	[3] F6 and F9	1095/1087	0.799	0.985	0.757	1	Mascot
1640.8047	1640.9094	0.1047	64	77	87	MKDTDSEEEIR	42	99.86	(N-term)_iTRAQ[0], Lysine(K)_iTRAQ[2]	[4] F7 and F10+11	1029/1021	1.156	0.962	0.556	1	Mascot
1740.816	1740.7812	-0.0348	-20	79	91	DTDSEEEIREAFR	61	99.998	(N-term)_iTRAQ[0]	[4] F7 and F10+11	246/238	0.881	0.780	1.491	1	Mascot
2043.0757	2042.9711	-0.1046	-51	92	107	VFDKDGNGYISAAELR	74	100	(N-term)_iTRAQ[0], Lysine(K)_iTRAQ[4]	[6] F8 110912	290/282	1.331	1.540	2.023	1	Mascot
2277.1985	2277.1162	-0.0823	-36	15	31	EAFSLFDKDGDGTITTK	49	99.971	(N-term)_iTRAQ[0], Lysine(K)_iTRAQ[8,17]	[6] F8 110912	370/362	1.760	0.827	1.965	1	Mascot
2277.1985	2277.1946	-0.0039	-2	15	31	EAFSLFDKDGDGTITTK	78	100	(N-term)_iTRAQ[0], Lysine(K)_iTRAQ[8,17]	[4] F7 and F10+11	321/313	0.894	0.735	0.957	1	Mascot

235	Chain A, Crystal Structure Of Endothelial Nitric Oxide Synthase Peptide Bound To Calmodulin	gi|28948873	17812.3	4	281	1.093	0.941	1.156	0.346	0.260	0.721	6	6	6	100

Peptide Information

Calc. Mass	Obsrv. Mass	± da	± ppm	Start Seq.	End Seq.	Sequence	Ion Score	C. I. %	Modification	Plate [#] Name	Gel Idx/Pos [4700 Sample Name]	iTRAQ Ratio 115/114*	iTRAQ Ratio 116/114*	iTRAQ Ratio 117/114*	Rank	Result Type
1640.8047	1640.8135	0.0088	5	76	86	XKDTDSEEEIR	69	100	(N-term)_iTRAQ[0], Lysine(K)_iTRAQ[2]	[3] F6 and F9	1095/1087	0.799	0.985	0.757	1	Mascot
1640.8047	1640.9094	0.1047	64	76	86	XKDTDSEEEIR	42	99.86	(N-term)_iTRAQ[0], Lysine(K)_iTRAQ[2]	[4] F7 and F10+11	1029/1021	1.156	0.962	0.556	1	Mascot
1740.816	1740.7812	-0.0348	-20	78	90	DTDSEEEIREAFR	61	99.998	(N-term)_iTRAQ[0]	[4] F7 and F10+11	246/238	0.881	0.780	1.491	1	Mascot
2043.0757	2042.9711	-0.1046	-51	91	106	VFDKDGNGYISAAELR	74	100	(N-term)_iTRAQ[0], Lysine(K)_iTRAQ[4]	[6] F8 110912	290/282	1.331	1.540	2.023	1	Mascot
2277.1985	2277.1162	-0.0823	-36	14	30	EAFSLFDKDGDGTITTK	49	99.971	(N-term)_iTRAQ[0], Lysine(K)_iTRAQ[8,17]	[6] F8 110912	370/362	1.760	0.827	1.965	1	Mascot
2277.1985	2277.1946	-0.0039	-2	14	30	EAFSLFDKDGDGTITTK	78	100	(N-term)_iTRAQ[0], Lysine(K)_iTRAQ[8,17]	[4] F7 and F10+11	321/313	0.894	0.735	0.957	1	Mascot

236	TUC-4b [Rattus norvegicus]	gi|21666559	79984.9	5	280	1.120	0.999	1.143	0.295	0.236	0.182	5	5	5	100

Protein Group

dihydropyrimidinase-related protein 3 [Rattus norvegicus]	gi|25742568	67293.5

Peptide Information

Calc. Mass	Obsrv. Mass	± da	± ppm	Start Seq.	End Seq.	Sequence	Ion Score	C. I. %	Modification	Plate [#] Name	Gel Idx/Pos [4700 Sample Name]	iTRAQ Ratio 115/114*	iTRAQ Ratio 116/114*	iTRAQ Ratio 117/114*	Rank	Result Type
1166.6489	1166.6199	-0.029	-25	475	481	MSVIWDK	38	99.583	(N-term)_iTRAQ[0], Lysine(K)_iTRAQ[7]	[3] F6 and F9	289/281	1.472	0.900	1.205	1	Mascot
1854.9615	1854.9171	-0.0444	-24	565	580	IMLEDGNLHVTQGAGR	46	99.94	(N-term)_iTRAQ[0]	[3] F6 and F9	266/258	0.897	1.103	1.123	1	Mascot
2014.0161	2013.9055	-0.1106	-55	488	503	MDENQFVAVTSTNAAK	82	100	(N-term)_iTRAQ[0], Lysine(K)_iTRAQ[16]	[5] F4	165/157	1.268	1.136	1.117	1	Mascot
2188.1748	2188.1025	-0.0723	-33	514	531	IAVGSDSDLVIWDPDAVK	64	99.999	(N-term)_iTRAQ[0], Lysine(K)_iTRAQ[18]	[1] F3 030912	399/391	1.307	1.270	1.428	1	Mascot
3217.6548	3217.5391	-0.1157	-36	208	235	AALAGGTTMIIDHVVPEPESSLTEAYEK	50	99.974	(N-term)_iTRAQ[0], Lysine(K)_iTRAQ[28]	[1] F3 030912	412/404	0.804	0.696	0.902	1	Mascot

237	isocitrate dehydrogenase [NADP], mitochondrial precursor [Rattus norvegicus]	gi|62079055	56778.7	5	277	1.207	1.484	1.350	0.577	1.002	1.059	5	5	5	100

Peptide Information

Calc. Mass	Obsrv. Mass	± da	± ppm	Start Seq.	End Seq.	Sequence	Ion Score	C. I. %	Modification	Plate [#] Name	Gel Idx/Pos [4700 Sample Name]	iTRAQ Ratio 115/114*	iTRAQ Ratio 116/114*	iTRAQ Ratio 117/114*	Rank	Result Type
1235.7762	1235.7621	-0.0141	-11	61	67	IIWQFIK	38	99.573	(N-term)_iTRAQ[0], Lysine(K)_iTRAQ[7]	[3] F6 and F9	436/428	2.589	4.043	4.244	1	Mascot
1726.0625	1726.0366	-0.0259	-15	160	172	LVPGWTKPITIGR	28	96.406	(N-term)_iTRAQ[0], Lysine(K)_iTRAQ[7]	[4] F7 and F10+11	348/340	0.895	0.984	0.949	1	Mascot
1862.0436	1862.1136	0.07	38	262	272	FKDIFQEIFDK	73	100	(N-term)_iTRAQ[0], Lysine(K)_iTRAQ[2,11]	[4] F7 and F10+11	1342/1334	0.924	1.035	0.898	1	Mascot
2209.175	2209.2275	0.0525	24	427	442	LNEHFLNTTDFLDTIK	87	100	(N-term)_iTRAQ[0], Lysine(K)_iTRAQ[16]	[4] F7 and F10+11	439/431	1.094	1.318	1.111	1	Mascot
2408.29	2408.334	0.044	18	44	60	IKVEKPVVEMDGDEMTR	50	99.978	(N-term)_iTRAQ[0], Lysine(K)_iTRAQ[2,5]	[4] F7 and F10+11	1152/1144	1.093	1.328	1.117	1	Mascot

238	succinyl-CoA synthetase alpha subunit (EC 6.2.1.4) [Rattus norvegicus]	gi|204356	39366.2	5	276	0.691	0.980	0.911	0.263	0.255	0.196	5	5	5	100

Peptide Information

Calc. Mass	Obsrv. Mass	± da	± ppm	Start Seq.	End Seq.	Sequence	Ion Score	C. I. %	Modification	Plate [#] Name	Gel Idx/Pos [4700 Sample Name]	iTRAQ Ratio 115/114*	iTRAQ Ratio 116/114*	iTRAQ Ratio 117/114*	Rank	Result Type
1390.8239	1390.785	-0.0389	-28	170	179	IGIMPGHIHK	46	99.941	(N-term)_iTRAQ[0], Lysine(K)_iTRAQ[10]	[2] F12 040912	237/229	0.511	0.744	0.788	1	Mascot
1785.1122	1785.0653	-0.0469	-26	81	92	KHLGLPVFNTVK	61	99.998	(N-term)_iTRAQ[0], Lysine(K)_iTRAQ[1,12]	[2] F12 040912	320/312	0.507	0.890	0.973	1	Mascot
1969.1844	1969.156	-0.0284	-14	266	282	AKPVVSFIAGITAPPGR	34	98.949	(N-term)_iTRAQ[0], Lysine(K)_iTRAQ[2]	[3] F6 and F9	390/382	0.610	1.198	1.024	1	Mascot
1983.0182	1982.8933	-0.1249	-63	54	68	QGTFHSQQALEYGTK	104	100	(N-term)_iTRAQ[0], Lysine(K)_iTRAQ[15]	[6] F8 110912	190/182	1.162	1.374	1.175	1	Mascot
2004.995	2004.9259	-0.0691	-34	154	169	LIGPNCPGIINPGECK	31	98.02	(N-term)_iTRAQ[0], Lysine(K)_iTRAQ[16], MMTS (C)[6,15]	[1] F3 030912	415/407	0.857	0.827	0.679	1	Mascot

239	Hnrpl protein [Rattus norvegicus]	gi|55562839	29814.6	4	276	1.116	1.239	1.184	0.180	0.279	0.185	4	4	4	100

Peptide Information

Calc. Mass	Obsrv. Mass	± da	± ppm	Start Seq.	End Seq.	Sequence	Ion Score	C. I. %	Modification	Plate [#] Name	Gel Idx/Pos [4700 Sample Name]	iTRAQ Ratio 115/114*	iTRAQ Ratio 116/114*	iTRAQ Ratio 117/114*	Rank	Result Type
1923.0156	1922.9218	-0.0938	-49	91	104	AITHLNNNFMFGQK	101	100	(N-term)_iTRAQ[0], Lysine(K)_iTRAQ[14]	[6] F8 110912	301/293	1.338	0.917	1.285	1	Mascot
2155.1104	2155.135	0.0246	11	209	224	SDALETLGFLNHYQMK	60	99.997	(N-term)_iTRAQ[0], Lysine(K)_iTRAQ[16]	[4] F7 and F10+11	414/406	0.884	1.180	1.046	1	Mascot
2156.0725	2156.052	-0.0205	-10	73	90	SKPGAAMVEMADGYAVDR	37	99.539	(N-term)_iTRAQ[0], Lysine(K)_iTRAQ[2]	[4] F7 and F10+11	307/299	1.146	1.371	1.440	1	Mascot
2465.1377	2465.0378	-0.0999	-41	112	131	QPAIMPGQSYGLEDGSCSYK	78	100	(N-term)_iTRAQ[0], Lysine(K)_iTRAQ[20], MMTS (C)[17]	[1] F3 030912	313/305	1.146	1.588	1.015	1	Mascot

240	adenylate cyclase type 5 [Rattus norvegicus]	gi|12018268	148388.4	5	274	0.926	0.860	0.832	0.165	0.101	0.179	6	6	6	100

Peptide Information

Calc. Mass	Obsrv. Mass	± da	± ppm	Start Seq.	End Seq.	Sequence	Ion Score	C. I. %	Modification	Plate [#] Name	Gel Idx/Pos [4700 Sample Name]	iTRAQ Ratio 115/114*	iTRAQ Ratio 116/114*	iTRAQ Ratio 117/114*	Rank	Result Type
1501.8499	1501.8333	-0.0166	-11	635	645	EHSIETFLILR	44	99.908	(N-term)_iTRAQ[0]	[4] F7 and F10+11	392/384	0.728	0.958	0.941	1	Mascot
1650.0186	1650.0217	0.0031	2	1178	1193	IGLNIGPVVAGVIGAR	27	95.23	(N-term)_iTRAQ[0]	[8] F13-15 and F1+2	347/339	1.251	0.940	0.797	1	Mascot
1650.0186	1650.0273	0.0087	5	1178	1193	IGLNIGPVVAGVIGAR	35	99.246	(N-term)_iTRAQ[0]	[8] F13-15 and F1+2	341/333	0.835	0.788	0.620	1	Mascot
1871.9735	1872.0393	0.0658	35	6	23	SVSPPGYAAQTAASPAPR	36	99.321	(N-term)_iTRAQ[0]	[8] F13-15 and F1+2	603/595	0.945	0.728	0.801	1	Mascot
2136.1448	2136.0227	-0.1221	-57	1194	1209	KPQYDIWGNTVNVASR	57	99.995	(N-term)_iTRAQ[0], Lysine(K)_iTRAQ[1]	[6] F8 110912	295/287	0.955	0.972	1.167	1	Mascot
2281.1768	2281.0918	-0.085	-37	131	155	APPAGGSGSSAAAAAAAGGTEVRPR	102	100	(N-term)_iTRAQ[0]	[7] F5 120912	144/136	0.921	0.804	0.764	1	Mascot

241	alpha-adducin [Rattus norvegicus]	gi|78365244	87976.4	5	273	0.943	0.893	0.922	0.253	0.356	0.338	8	8	8	100

Protein Group

alpha-adducin, hypertensive phenotype [Rattus norvegicus]	gi|1200129	87960.4
alpha-adducin, normotensive phenotype [Rattus norvegicus]	gi|785039	87986.4

Peptide Information

Calc. Mass	Obsrv. Mass	± da	± ppm	Start	End	Sequence	Ion	C. I. %	Modification	Plate [#] Name	Gel Idx/Pos [4700	iTRAQ	iTRAQ	iTRAQ	Rank	Result Type

				Seq.	Seq.		Score				Sample Name]	Ratio 115/114*	Ratio 116/114*	Ratio 117/114*		
1272.7032	1272.6283	-0.0749	-59	200	209	VNLQGDIVDR	28	96.398	(N-term)_iTRAQ[0]	[5] F4	148/140	1.161	1.184	0.961	1	Mascot
1392.688	1392.6154	-0.0726	-52	28	37	VDENNPEYLR	33	98.719	(N-term)_iTRAQ[0]	[5] F4	112/104	0.760	0.963	0.878	1	Mascot
1458.6967	1458.6327	-0.064	-44	47	55	QDFNMMEQK	54	99.991	(N-term)_iTRAQ[0], Lysine(K)_iTRAQ[9]	[7] F5 120912	168/160	0.598	0.398	0.507	1	Mascot
1458.6967	1458.644	-0.0527	-36	47	55	QDFNMMEQK	52	99.986	(N-term)_iTRAQ[0], Lysine(K)_iTRAQ[9]	[3] F6 and F9	205/197	1.346	1.214	1.505	1	Mascot
1762.0109	1761.9274	-0.0835	-47	7	21	AAVVTSPPPTTAPHK	88	100	(N-term)_iTRAQ[0], Lysine(K)_iTRAQ[15]	[3] F6 and F9	155/147	0.987	1.001	1.017	1	Mascot
1780.8812	1780.826	-0.0552	-31	370	382	WQIGEQEFEALMR	67	99.999	(N-term)_iTRAQ[0]	[1] F3 030912	469/461	1.038	1.073	1.232	1	Mascot
1780.8812	1780.8906	0.0094	5	370	382	WQIGEQEFEALMR	69	100	(N-term)_iTRAQ[0]	[8] F13-15 and F1+2	347/339	0.848	0.873	0.936	1	Mascot
1780.8812	1780.895	0.0138	8	370	382	WQIGEQEFEALMR	58	99.996	(N-term)_iTRAQ[0]	[8] F13-15 and F1+2	350/342	1.017	0.781	0.691	1	Mascot

242	eukaryotic initiation factor 4A-II [Rattus norvegicus]	gi|56605748	49871.1	4	272	1.231	1.215	1.150	0.082	0.070	0.243	4	4	4	100

Peptide Information

Calc. Mass	Obsrv. Mass	± da	± ppm	Start Seq.	End Seq.	Sequence	Ion Score	C. I. %	Modification	Plate [#] Name	Gel Idx/Pos [4700 Sample Name]	iTRAQ Ratio 115/114*	iTRAQ Ratio 116/114*	iTRAQ Ratio 117/114*	Rank	Result Type
1682.8959	1682.7755	-0.1204	-72	70	83	GYDVIAQAQSGTGK	99	100	(N-term)_iTRAQ[0], Lysine(K)_iTRAQ[14]	[5] F4	133/125	1.329	1.313	1.087	1	Mascot
1699.8156	1699.829	0.0134	8	179	191	MFVLDEADEMLSR	56	99.994	(N-term)_iTRAQ[0]	[8] F13-15 and F1+2	356/348	1.111	1.188	1.164	1	Mascot
1948.0916	1948.155	0.0634	33	192	203	GFKDQIYEIFQK	80	100	(N-term)_iTRAQ[0], Lysine(K)_iTRAQ[3,12]	[4] F7 and F10+11	1280/1272	1.254	1.128	1.535	1	Mascot
2116.1436	2116.0852	-0.0584	-28	47	62	GIYAYGFEKPSAIQQR	37	99.482	(N-term)_iTRAQ[0], Lysine(K)_iTRAQ[9]	[4] F7 and F10+11	286/278	1.238	1.240	0.900	1	Mascot

243	nucleoside diphosphate kinase A [Rattus norvegicus]	gi|19924089	19003	5	272	1.051	1.009	1.029	0.315	0.241	0.315	8	8	8	100

Peptide Information

Calc. Mass	Obsrv. Mass	± da	± ppm	Start Seq.	End Seq.	Sequence	Ion Score	C. I. %	Modification	Plate [#] Name	Gel Idx/Pos [4700 Sample Name]	iTRAQ Ratio 115/114*	iTRAQ Ratio 116/114*	iTRAQ Ratio 117/114*	Rank	Result Type
1184.5657	1184.5066	-0.0591	-50	106	114	GDFCIQVGR	32	98.456	(N-term)_iTRAQ[0], MMTS (C)[4]	[5] F4	202/194	0.729	0.901	0.624	1	Mascot
1453.8413	1453.8757	0.0344	24	57	66	DRPFFSGLVK	41	99.811	(N-term)_iTRAQ[0], Lysine(K)_iTRAQ[10]	[4] F7 and F10+11	1203/1195	1.015	1.078	1.312	1	Mascot
1453.8413	1453.9021	0.0608	42	57	66	DRPFFSGLVK	33	98.64	(N-term)_iTRAQ[0], Lysine(K)_iTRAQ[10]	[3] F6 and F9	1264/1256	0.801	0.794	0.721	1	Mascot
1632.9683	1632.8651	-0.1032	-63	7	18	TFIAIKPDGVQR	29	96.722	(N-term)_iTRAQ[0], Lysine(K)_iTRAQ[6]	[6] F8 110912	250/242	1.843	1.399	1.235	1	Mascot
1632.9683	1632.9409	-0.0274	-17	7	18	TFIAIKPDGVQR	60	99.997	(N-term)_iTRAQ[0], Lysine(K)_iTRAQ[6]	[4] F7 and F10+11	243/235	1.187	1.016	1.214	1	Mascot
2074.1213	2074.0786	-0.0427	-21	89	105	VMLGETNPADSKPGTIR	54	99.991	(N-term)_iTRAQ[0], Lysine(K)_iTRAQ[12]	[4] F7 and F10+11	226/218	1.134	1.322	1.327	1	Mascot
2074.1213	2074.0818	-0.0395	-19	89	105	VMLGETNPADSKPGTIR	38	99.638	(N-term)_iTRAQ[0], Lysine(K)_iTRAQ[12]	[3] F6 and F9	227/219	0.979	0.721	0.963	1	Mascot
2184.1475	2184.0791	-0.0684	-31	129	143	EISLWFQPEELVDYK	85	100	(N-term)_iTRAQ[0], Lysine(K)_iTRAQ[15]	[1] F3 030912	503/495	1.032	1.030	1.110	1	Mascot

244	ubiquitin-40S ribosomal protein S27a precursor [Mus musculus]	gi|13195690	21962.1	5	272	1.065	0.936	1.132	0.198	0.129	0.191	5	5	5	100

Protein Group

polyubiquitin [Rattus norvegicus]	gi|1050930	12675
polyubiquitin-B [Mus musculus]	gi|6755919	38526.5
polyubiquitin-C precursor [Rattus norvegicus]	gi|8394502	102218.9
ubiquitin-60S ribosomal protein L40 precursor [Homo sapiens]	gi|4507761	17542.7

Peptide Information

Calc. Mass	Obsrv. Mass	± da	± ppm	Start Seq.	End Seq.	Sequence	Ion Score	C. I. %	Modification	Plate [#] Name	Gel Idx/Pos [4700 Sample Name]	iTRAQ Ratio 115/114*	iTRAQ Ratio 116/114*	iTRAQ Ratio 117/114*	Rank	Result Type
1053.6377	1053.646	0.0083	8	1	6	MQIFVK	35	99.254	(N-term)_iTRAQ[0], Lysine(K)_iTRAQ[6]	[3] F6 and F9	262/254	1.104	0.892	1.232	1	Mascot
1211.7233	1211.6556	-0.0677	-56	64	72	ESTLHLVLR	31	98.057	(N-term)_iTRAQ[0]	[6] F8 110912	264/256	0.770	0.763	0.839	1	Mascot
1369.7573	1369.7258	-0.0315	-23	55	63	TLSDYNIQK	57	99.995	(N-term)_iTRAQ[0], Lysine(K)_iTRAQ[9]	[7] F5 120912	164/156	1.090	1.101	1.184	1	Mascot

1811.9862	1811.8832	-0.103	-57	30	42	IQDKEGIPPDQQR	60	99.998	(N-term)_iTRAQ[0], Lysine(K)_iTRAQ[4]	[6] F8 110912	146/138	1.171	0.917	1.154	1	Mascot
2076.1321	2076.0603	-0.0718	-35	12	27	TITLEVEPSDTIENVK	89	100	(N-term)_iTRAQ[0], Lysine(K)_iTRAQ[16]	[1] F3 030912	308/300	1.262	1.045	1.313	1	Mascot

245	protein-L-isoaspartate(D-aspartate) O-methyltransferase [Rattus norvegicus]	gi|56961640	27265.5	5	271	0.821	0.891	0.825	0.213	0.062	0.112	5	5	5	100

Peptide Information

Calc. Mass	Obsrv. Mass	± da	± ppm	Start Seq.	End Seq.	Sequence	Ion Score	C. I. %	Modification	Plate [#] Name	Gel Idx/Pos [4700 Sample Name]	iTRAQ Ratio 115/114*	iTRAQ Ratio 116/114*	iTRAQ Ratio 117/114*	Rank	Result Type
1324.7054	1324.6584	-0.047	-35	28	37	VFEVMLATDR	49	99.97	(N-term)_iTRAQ[0]	[1] F3 030912	345/337	1.105	0.875	0.871	1	Mascot
1812.9775	1812.984	0.0065	4	25	37	TDKVFEVMLATDR	43	99.869	(N-term)_iTRAQ[0], Lysine(K)_iTRAQ[3]	[4] F7 and F10+11	368/360	1.009	0.952	0.973	1	Mascot
1827.9198	1827.9302	0.0104	6	82	98	ALDVGSGSGILTACFAR	54	99.99	(N-term)_iTRAQ[0], MMTS (C)[14]	[8] F13-15 and F1+2	333/325	0.774	0.972	0.672	1	Mascot
2331.2627	2331.1855	-0.0772	-33	179	197	LILPVGPAGGNQMLEQY DK	96	100	(N-term)_iTRAQ[0], Lysine(K)_iTRAQ[19]	[1] F3 030912	391/383	0.579	0.856	0.876	1	Mascot
2538.49	2538.5933	0.1033	41	205	221	MKPLMGVIYVPLTDKEK	29	97.21	(N-term)_iTRAQ[0], Lysine(K)_iTRAQ[2,15,17]	[4] F7 and F10+11	1269/1261	0.747	0.810	0.767	1	Mascot

246	pyruvate carboxylase, mitochondrial precursor [Rattus norvegicus]	gi|31543464	138794.5	5	270	1.237	0.862	0.848	0.200	0.275	0.109	5	5	5	100

Protein Group

pyruvate carboxylase [Rattus norvegicus]	gi|929988	138707.4

Peptide Information

Calc. Mass	Obsrv. Mass	± da	± ppm	Start Seq.	End Seq.	Sequence	Ion Score	C. I. %	Modification	Plate [#] Name	Gel Idx/Pos [4700 Sample Name]	iTRAQ Ratio 115/114*	iTRAQ Ratio 116/114*	iTRAQ Ratio 117/114*	Rank	Result Type
1507.7764	1507.7229	-0.0535	-35	1044	1054	IAEEFEVELER	34	98.934	(N-term)_iTRAQ[0]	[1] F3 030912	369/361	1.046	0.906	0.870	1	Mascot
1691.8361	1691.7545	-0.0816	-48	929	942	AEAEAQAEELSFPR	76	100	(N-term)_iTRAQ[0]	[1] F3 030912	252/244	1.121	0.978	0.730	1	Mascot
1728.0002	1727.9862	-0.014	-8	632	644	ELIPNIPFQMLLR	39	99.685	(N-term)_iTRAQ[0]	[8] F13-15 and F1+2	260/252	1.494	1.320	1.050	1	Mascot
1803.0249	1802.9861	-0.0388	-22	274	288	VVEIAPATHLDPQLR	39	99.696	(N-term)_iTRAQ[0]	[7] F5 120912	256/248	1.126	0.623	0.809	1	Mascot
1865.8936	1865.845	-0.0486	-26	63	77	TVAVYSEQDTGQMHR	82	100	(N-term)_iTRAQ[0]	[3] F6 and F9	174/166	1.469	0.653	0.814	1	Mascot

247	dynamin [Rattus norvegicus]	gi|404073	105997.4	7	270	0.849	1.129	0.845	0.364	0.387	0.470	7	7	7	100

Protein Group

dynamin-2 [Rattus norvegicus]	gi|6978771	105938.3

Peptide Information

Calc. Mass	Obsrv. Mass	± da	± ppm	Start Seq.	End Seq.	Sequence	Ion Score	C. I. %	Modification	Plate [#] Name	Gel Idx/Pos [4700 Sample Name]	iTRAQ Ratio 115/114*	iTRAQ Ratio 116/114*	iTRAQ Ratio 117/114*	Rank	Result Type
1167.7024	1167.6805	-0.0219	-19	370	376	FPFELVK	33	98.701	(N-term)_iTRAQ[0], Lysine(K)_iTRAQ[7]	[7] F5 120912	356/348	0.702	0.980	0.793	1	Mascot
1209.7439	1209.7145	-0.0294	-24	114	123	GISPVPINLR	37	99.47	(N-term)_iTRAQ[0]	[1] F3 030912	278/270	0.975	1.423	1.950	1	Mascot
1251.6818	1251.6278	-0.054	-43	45	54	SSVLENFVGR	47	99.954	(N-term)_iTRAQ[0]	[5] F4	218/210	1.032	1.015	1.163	1	Mascot
1379.6597	1379.5951	-0.0646	-47	207	217	LDLMDEGTDAR	41	99.787	(N-term)_iTRAQ[0]	[1] F3 030912	232/224	0.735	1.142	0.629	1	Mascot
1394.7964	1394.7635	-0.0329	-24	563	571	YMLPLDNLK	29	97.145	(N-term)_iTRAQ[0], Lysine(K)_iTRAQ[9]	[5] F4	268/260	0.445	0.628	0.429	1	Mascot
1444.7682	1444.8235	0.0553	38	90	98	KFTDFDEVR	31	98.236	(N-term)_iTRAQ[0], Lysine(K)_iTRAQ[1]	[4] F7 and F10+11	1123/1115	1.543	1.543	0.744	1	Mascot
1986.0793	1986.0034	-0.0759	-38	143	157	VPVGDQPPDIEYQIK	52	99.983	(N-term)_iTRAQ[0], Lysine(K)_iTRAQ[15]	[1] F3 030912	280/272	0.889	1.496	0.853	1	Mascot

248	carbonic anhydrase 2 [Rattus norvegicus]	gi|9506445	32403.8	4	269	1.789	0.975	0.917	0.545	0.362	0.239	6	6	6	100

Peptide Information

Calc. Mass	Obsrv. Mass	± da	± ppm	Start Seq.	End Seq.	Sequence	Ion Score	C. I. %	Modification	Plate [#] Name	Gel Idx/Pos [4700 Sample Name]	iTRAQ Ratio 115/114*	iTRAQ Ratio 116/114*	iTRAQ Ratio 117/114*	Rank	Result Type
1286.7678	1286.7246	-0.0432	-34	149	158	IGPASQGLQK	41	99.801	(N-term)_iTRAQ[0], Lysine(K)_iTRAQ[10]	[7] F5 120912	142/134	2.048	1.092	1.127	1	Mascot
1791.882	1791.8373	-0.0447	-25	213	226	EPITVSSEQMSHFR	54	99.99	(N-term)_iTRAQ[0]	[3] F6 and F9	249/241	1.680	1.236	0.925	1	Mascot
1791.882	1791.8728	-0.0092	-5	213	226	EPITVSSEQMSHFR	87	100	(N-term)_iTRAQ[0]	[4] F7 and F10+11	250/242	1.372	0.801	0.634	1	Mascot
1870.0221	1869.9741	-0.048	-26	114	126	YAAELHLVHWNTK	44	99.901	(N-term)_iTRAQ[0], Lysine(K)_iTRAQ[13]	[2] F12 040912	349/341	2.423	0.925	1.038	1	Mascot

1870.0221	1870.1035	0.0814	44	114	126	YAAELHLVHWNTK	66	99.999	(N-term)_iTRAQ[0], Lysine(K)_iTRAQ[13]	[4] F7 and F10+11	1213/1205	1.203	0.567	0.724	1	Mascot
1966.1735	1966.1669	-0.0066	-3	133	148	AVQHPDGLAVLGIFLK	75	100	(N-term)_iTRAQ[0], Lysine(K)_iTRAQ[16]	[3] F6 and F9	489/481	2.382	1.516	1.196	1	Mascot

249	homer protein homolog 1 [Rattus norvegicus]	gi|13928988	45550.8	5	268	0.732	0.848	1.061	0.260	0.130	0.129	6	6	6	100

Peptide Information

Calc. Mass	Obsrv. Mass	± da	± ppm	Start Seq.	End Seq.	Sequence	Ion Score	C. I. %	Modification	Plate [#] Name	Gel Idx/Pos [4700 Sample Name]	iTRAQ Ratio 115/114*	iTRAQ Ratio 116/114*	iTRAQ Ratio 117/114*	Rank	Result Type
1485.8312	1485.9071	0.0759	51	194	203	HWEAELATLK	56	99.994	(N-term)_iTRAQ[0], Lysine(K)_iTRAQ[10]	[4] F7 and F10+11	1206/1198	0.636	1.022	1.165	1	Mascot
1557.8635	1557.917	0.0535	34	11	21	AHVFQIDPNTK	60	99.998	(N-term)_iTRAQ[0], Lysine(K)_iTRAQ[11]	[3] F6 and F9	1176/1168	0.623	0.725	1.010	1	Mascot
1579.7836	1579.6963	-0.0873	-55	225	236	QQLAAYQEEAER	55	99.992	(N-term)_iTRAQ[0]	[1] F3 030912	182/174	0.539	1.038	0.976	1	Mascot
1911.9434	1911.8695	-0.0739	-39	164	180	AEPAQNALPFSHSAGDR	47	99.949	(N-term)_iTRAQ[0]	[3] F6 and F9	208/200	1.214	0.804	0.887	1	Mascot
1911.9434	1911.9482	0.0048	3	164	180	AEPAQNALPFSHSAGDR	31	98.19	(N-term)_iTRAQ[0]	[4] F7 and F10+11	210/202	0.585	0.744	1.243	1	Mascot
2036.101	2036.0533	-0.0477	-23	259	273	TELSQTVQELEETLK	49	99.971	(N-term)_iTRAQ[0], Lysine(K)_iTRAQ[15]	[1] F3 030912	483/475	1.016	0.809	1.130	1	Mascot

250	Chain A, Crystal Structure Of Tetradeca-(3-Fluorotyrosyl)- Glutathione S-Transferase	gi|5107744	28109.2	7	266	0.929	1.178	1.260	0.303	0.271	0.255	7	7	7	100

Peptide Information

Calc. Mass	Obsrv. Mass	± da	± ppm	Start Seq.	End Seq.	Sequence	Ion Score	C. I. %	Modification	Plate [#] Name	Gel Idx/Pos [4700 Sample Name]	iTRAQ Ratio 115/114*	iTRAQ Ratio 116/114*	iTRAQ Ratio 117/114*	Rank	Result Type
1134.6517	1134.6339	-0.0178	-16	211	217	LAQWSNK	35	99.251	(N-term)_iTRAQ[0], Lysine(K)_iTRAQ[7]	[4] F7 and F10+11	191/183	0.861	1.160	1.403	1	Mascot
1192.6572	1192.6332	-0.024	-20	43	49	SQWLNEK	39	99.7	(N-term)_iTRAQ[0], Lysine(K)_iTRAQ[7]	[4] F7 and F10+11	191/183	0.761	1.000	1.259	1	Mascot
1244.7136	1244.6593	-0.0543	-44	136	143	LXSEFLGK	30	97.581	(N-term)_iTRAQ[0], Lysine(K)_iTRAQ[8]	[7] F5 120912	287/279	0.779	0.885	1.093	1	Mascot
1264.7048	1264.6775	-0.0273	-22	144	151	RPWFAGDK	36	99.392	(N-term)_iTRAQ[0], Lysine(K)_iTRAQ[8]	[2] F12 040912	229/221	1.212	1.612	1.847	1	Mascot
1392.7582	1392.73	-0.0282	-20	1	10	PMILGXWNVR	35	99.167	(N-term)_iTRAQ[0]	[3] F6 and F9	398/390	0.628	1.003	1.007	1	Mascot
1547.7607	1547.6836	-0.0771	-50	96	107	ADIVENQVMDNR	56	99.994	(N-term)_iTRAQ[0]	[1] F3 030912	211/203	1.007	1.252	1.146	1	Mascot
1934.0508	1934.0342	-0.0166	-9	52	67	LGLDFPNLPXLIDGSR	38	99.6	(N-term)_iTRAQ[0]	[8] F13-15 and F1+2	282/274	1.532	1.515	1.227	1	Mascot

251	histone H4 [Homo sapiens]	gi|4504301	13089.6	5	266	1.064	1.015	1.026	0.183	0.242	0.320	6	6	6	100

Peptide Information

Calc. Mass	Obsrv. Mass	± da	± ppm	Start Seq.	End Seq.	Sequence	Ion Score	C. I. %	Modification	Plate [#] Name	Gel Idx/Pos [4700 Sample Name]	iTRAQ Ratio 115/114*	iTRAQ Ratio 116/114*	iTRAQ Ratio 117/114*	Rank	Result Type
1133.6803	1133.6256	-0.0547	-48	61	68	VFLENVIR	35	99.15	(N-term)_iTRAQ[0]	[5] F4	252/244	1.029	0.937	1.390	1	Mascot
1133.6803	1133.6371	-0.0432	-38	61	68	VFLENVIR	35	99.165	(N-term)_iTRAQ[0]	[1] F3 030912	331/323	0.815	0.758	0.746	1	Mascot
1324.7233	1324.6725	-0.0508	-38	47	56	ISGLIYEETR	42	99.85	(N-term)_iTRAQ[0]	[5] F4	212/204	1.239	1.245	0.736	1	Mascot
1422.7474	1422.7561	0.0087	6	69	78	DAVTYTEHAK	50	99.976	(N-term)_iTRAQ[0], Lysine(K)_iTRAQ[10]	[3] F6 and F9	1090/1082	1.315	1.407	1.486	1	Mascot
1613.9585	1613.8887	-0.0698	-43	25	36	DNIQGITKPAIR	39	99.692	(N-term)_iTRAQ[0], Lysine(K)_iTRAQ[8]	[6] F8 110912	211/203	0.967	0.846	1.003	1	Mascot
1871.1047	1871.0298	-0.0749	-40	80	92	KTVTAMDVVYALK	100	100	(N-term)_iTRAQ[0], Lysine(K)_iTRAQ[1,1 3]	[6] F8 110912	388/380	1.099	1.041	1.027	1	Mascot

252	RecName: Full=Ras-related protein Rab-1B	gi|131803	24736.9	5	266	0.942	1.123	1.122	0.251	0.188	0.333	5	5	5	100

Protein Group

rab1B protein	gi|226486	24763.9

Peptide Information

Calc. Mass	Obsrv. Mass	± da	± ppm	Start Seq.	End Seq.	Sequence	Ion Score	C. I. %	Modification	Plate [#] Name	Gel Idx/Pos [4700 Sample Name]	iTRAQ Ratio 115/114*	iTRAQ Ratio 116/114*	iTRAQ Ratio 117/114*	Rank	Result Type
1231.6555	1231.5989	-0.0566	-46	101	108	QWLQEIDR	38	99.621	(N-term)_iTRAQ[0]	[5] F4	209/201	1.180	1.107	1.271	1	Mascot
1359.8457	1359.778	-0.0677	-50	11	21	LLLIGDSGVGK	58	99.996	(N-term)_iTRAQ[0], Lysine(K)_iTRAQ[11]	[5] F4	229/221	0.740	1.446	1.508	1	Mascot
1460.7618	1460.6924	-0.0694	-48	59	69	LQIWDTAGQER	70	100	(N-term)_iTRAQ[0]	[5] F4	197/189	0.762	1.084	0.943	1	Mascot
1729.9265	1729.8306	-0.0959	-55	173	187	MGPGAASGGERPNLK	37	99.497	(N-term)_iTRAQ[0], Lysine(K)_iTRAQ[15]	[6] F8 110912	161/153	0.846	0.891	0.739	1	Mascot
2157.093	2157.0515	-0.0415	-19	154	170	NATNVEQAFMTMAAEIK	63	99.999	(N-term)_iTRAQ[0], Lysine(K)_iTRAQ[17]	[1] F3 030912	512/504	1.319	1.157	1.335	1	Mascot

253	unnamed protein product [Rattus norvegicus]	gi|56691	27301.3	5	263	0.854	1.049	1.068	0.303	0.204	0.166	5	5	5	100

Protein Group

superoxide dismutase [Mn], mitochondrial precursor [Rattus norvegicus]	gi|8394331	27292.3

Peptide Information

Calc. Mass	Obsrv. Mass	± da	± ppm	Start Seq.	End Seq.	Sequence	Ion Score	C. I. %	Modification	Plate [#] Name	Gel Idx/Pos [4700 Sample Name]	iTRAQ Ratio 115/114*	iTRAQ Ratio 116/114*	iTRAQ Ratio 117/114*	Rank	Result Type
1117.5775	1117.5283	-0.0492	-44	124	130	DFGSFEK	42	99.845	(N-term)_iTRAQ[0], Lysine(K)_iTRAQ[7]	[3] F6 and F9	207/199	1.089	1.288	1.266	1	Mascot
1160.7136	1160.6799	-0.0337	-29	115	122	GELLEAIK	36	99.377	(N-term)_iTRAQ[0], Lysine(K)_iTRAQ[8]	[7] F5 120912	239/231	1.002	1.146	0.827	1	Mascot
1729.0106	1728.9246	-0.086	-50	76	89	GDVTTQVALQPALK	62	99.999	(N-term)_iTRAQ[0], Lysine(K)_iTRAQ[14]	[1] F3 030912	232/224	0.721	0.756	1.032	1	Mascot
1872.984	1872.8887	-0.0953	-51	203	216	AIWNVINWENVSQR	73	100	(N-term)_iTRAQ[0]	[5] F4	346/338	1.134	1.072	1.097	1	Mascot
2057.0662	2056.9863	-0.0799	-39	54	68	HHATYVNNLNVTEEK	50	99.975	(N-term)_iTRAQ[0], Lysine(K)_iTRAQ[15]	[2] F12 040912	201/193	0.509	1.061	1.171	1	Mascot

254	cGMP-dependent 3',5'-cyclic phosphodiesterase isoform 2 [Rattus norvegicus]	gi|13592021	112945.3	6	262	1.449	1.122	1.101	0.645	0.343	0.766	6	6	6	100

Peptide Information

Calc. Mass	Obsrv. Mass	± da	± ppm	Start Seq.	End Seq.	Sequence	Ion Score	C. I. %	Modification	Plate [#] Name	Gel Idx/Pos [4700 Sample Name]	iTRAQ Ratio 115/114*	iTRAQ Ratio 116/114*	iTRAQ Ratio 117/114*	Rank	Result Type
1487.7628	1487.7052	-0.0576	-39	817	826	EFFSQGDLEK	63	99.999	(N-term)_iTRAQ[0], Lysine(K)_iTRAQ[10]	[7] F5 120912	220/212	1.378	0.830	0.735	1	Mascot
1544.6989	1544.6495	-0.0494	-32	341	352	LGGDFFTDEDER	32	98.569	(N-term)_iTRAQ[0]	[5] F4	211/203	1.251	1.296	0.790	1	Mascot
1631.9353	1632.0015	0.0662	41	751	763	DIILATDLAHHLR	66	99.999	(N-term)_iTRAQ[0]	[4] F7 and F10+11	1239/1231	2.826	1.757	2.990	1	Mascot
1843.8834	1843.8708	-0.0126	-7	432	446	VFDGGVVDDESYEIR	30	97.636	(N-term)_iTRAQ[0]	[8] F13-15 and F1+2	474/466	0.968	1.234	0.727	1	Mascot
1910.8995	1910.8867	-0.0128	-7	552	564	SHLANEMMMYHMK	42	99.858	(N-term)_iTRAQ[0], Lysine(K)_iTRAQ[13]	[2] F12 040912	301/293	1.039	0.816	0.855	1	Mascot
2025.0012	2024.9672	-0.034	-17	83	97	LVCEDPPHELPQEGK	27	95.391	(N-term)_iTRAQ[0], Lysine(K)_iTRAQ[15], MMTS (C)[3]	[3] F6 and F9	274/266	1.887	1.051	1.651	1	Mascot

255	TGF-beta resistance-associated protein TRAG [Rattus norvegicus]	gi|15624077	172011	6	261	0.860	1.174	0.978	0.848	0.595	0.590	6	6	6	100

Protein Group

WD repeat-containing protein 7 [Rattus norvegicus]	gi|13027430	175052.4

Peptide Information

Calc. Mass	Obsrv. Mass	± da	± ppm	Start Seq.	End Seq.	Sequence	Ion Score	C. I. %	Modification	Plate [#] Name	Gel Idx/Pos [4700 Sample Name]	iTRAQ Ratio 115/114*	iTRAQ Ratio 116/114*	iTRAQ Ratio 117/114*	Rank	Result Type
1382.877	1382.901	0.024	17	559	567	HLFPIQVIK	58	99.996	(N-term)_iTRAQ[0], Lysine(K)_iTRAQ[9]	[3] F6 and F9	1292/1284	1.185	1.195	1.285	1	Mascot
1431.7716	1431.7075	-0.0641	-45	1362	1374	GPITAVSFAPDGR	45	99.926	(N-term)_iTRAQ[0]	[1] F3 030912	262/254	0.409	0.692	0.913	1	Mascot
1472.6604	1472.6261	-0.0343	-23	1312	1322	GLQECFPAICR	31	98.096	(N-term)_iTRAQ[0], MMTS (C)[5,10]	[1] F3 030912	503/495	2.091	2.308	2.140	1	Mascot
1829.0015	1828.8739	-0.1276	-70	934	948	DSPPASSNIVQGQIK	66	99.999	(N-term)_iTRAQ[0], Lysine(K)_iTRAQ[15]	[5] F4	123/115	1.796	1.474	1.017	1	Mascot
2045.9093	2045.9596	0.0503	25	756	770	EHLLDEEEDEEEVMR	30	97.743	(N-term)_iTRAQ[0]	[3] F6 and F9	260/252	0.597	0.739	0.460	1	Mascot
2976.6311	2976.5515	-0.0796	-27	426	451	EDGSIIIVPATQTAIVQLLQGEHMLR	31	97.998	(N-term)_iTRAQ[0]	[1] F3 030912	539/531	0.371	1.261	0.745	1	Mascot

256	eukaryotic initiation factor 4A-I isoform 1 [Homo sapiens]	gi|4503529	49334.6	4	261	1.341	1.191	1.106	0.251	0.107	0.162	4	4	4	100

Peptide Information

Calc. Mass	Obsrv. Mass	± da	± ppm	Start Seq.	End Seq.	Sequence	Ion Score	C. I. %	Modification	Plate [#] Name	Gel Idx/Pos [4700 Sample Name]	iTRAQ Ratio 115/114*	iTRAQ Ratio 116/114*	iTRAQ Ratio 117/114*	Rank	Result Type
1682.8959	1682.7755	-0.1204	-72	69	82	GYDVIAQAQSGTGK	99	100	(N-term)_iTRAQ[0], Lysine(K)_iTRAQ[14]	[5] F4	133/125	1.329	1.313	1.087	1	Mascot
1699.8156	1699.829	0.0134	8	178	190	MFVLDEADEMLSR	56	99.994	(N-term)_iTRAQ[0]	[8] F13-15 and F1+2	356/348	1.111	1.188	1.164	1	Mascot
1934.0759	1934.1582	0.0823	43	191	202	GFKDQIYDIFQK	69	100	(N-term)_iTRAQ[0], Lysine(K)_iTRAQ[3,12]	[4] F7 and F10+11	1269/1261	1.767	1.039	1.314	1	Mascot
2116.1436	2116.0852	-0.0584	-28	46	61	GIYAYGFEKPSAIQQR	37	99.482	(N-term)_iTRAQ[0], Lysine(K)_iTRAQ[9]	[4] F7 and F10+11	286/278	1.238	1.240	0.900	1	Mascot

257	RecName: Full=Dihydropyrimidinase-related protein 4; Short=DRP-4; AltName: Full=Collapsin response	gi|3122037	65830	6	260	1.384	1.156	1.033	0.350	0.309	0.433	7	7	7	100

Peptide Information

Calc. Mass	Obsrv. Mass	± da	± ppm	Start Seq.	End Seq.	Sequence	Ion Score	C. I. %	Modification	Plate [#] Name	Gel Idx/Pos [4700 Sample Name]	iTRAQ Ratio 115/114*	iTRAQ Ratio 116/114*	iTRAQ Ratio 117/114*	Rank	Result Type
1135.6708	1135.7211	0.0503	44	480	488	LAEIHGVPR	40	99.775	(N-term)_iTRAQ[0]	[3] F6 and F9	1164/1156	1.116	0.862	1.107	1	Mascot
1288.7168	1288.6454	-0.0714	-55	433	443	GMPTVVISQGR	41	99.798	(N-term)_iTRAQ[0]	[1] F3 030912	216/208	1.277	1.256	0.682	1	Mascot
1576.911	1576.9716	0.0606	38	464	472	KTFPDFVYK	42	99.853	(N-term)_iTRAQ[0], Lysine(K)_iTRAQ[1,9]	[4] F7 and F10+11	1179/1171	1.192	1.237	0.773	1	Mascot
1755.9514	1755.9109	-0.0405	-23	393	407	VAVGSDADLVIWNPR	38	99.637	(N-term)_iTRAQ[0]	[1] F3 030912	359/351	1.804	1.259	1.220	1	Mascot
2015.0001	2014.868	-0.1321	-66	367	382	MDENEFVAVTSTNAAK	67	100	(N-term)_iTRAQ[0], Lysine(K)_iTRAQ[16]	[5] F4	183/175	1.416	0.811	0.790	1	Mascot
2015.0001	2014.913	-0.0871	-43	367	382	MDENEFVAVTSTNAAK	71	100	(N-term)_iTRAQ[0], Lysine(K)_iTRAQ[16]	[5] F4	163/155	2.050	1.730	2.086	1	Mascot
2015.0807	2015.0343	-0.0464	-23	136	149	WHESTKEELEALVR	30	97.432	(N-term)_iTRAQ[0], Lysine(K)_iTRAQ[6]	[2] F12 040912	364/356	1.093	1.167	1.069	1	Mascot

258	Phosphofructokinase, liver [Rattus norvegicus]	gi|38197562	91361	6	260	1.090	1.130	1.077	0.557	0.493	0.562	7	7	7	100

Protein Group

6-phosphofructokinase, liver type [Rattus norvegicus] gi|6981352 91404.1

Peptide Information

Calc. Mass	Obsrv. Mass	± da	± ppm	Start Seq.	End Seq.	Sequence	Ion Score	C. I. %	Modification	Plate [#] Name	Gel Idx/Pos [4700 Sample Name]	iTRAQ Ratio 115/114*	iTRAQ Ratio 116/114*	iTRAQ Ratio 117/114*	Rank	Result Type
1195.663	1195.6348	-0.0282	-24	202	210	TFVLEVMGR	32	98.334	(N-term)_iTRAQ[0]	[1] F3 030912	363/355	0.867	1.180	1.266	1	Mascot
1594.8597	1594.8016	-0.0581	-36	346	356	LPLMECVQVTK	48	99.957	(N-term)_iTRAQ[0], Lysine(K)_iTRAQ[11], MMTS (C)[6]	[5] F4	332/324	2.377	1.926	2.517	1	Mascot
1704.9418	1704.8687	-0.0731	-43	130	141	NEWGSLLEELVK	57	99.995	(N-term)_iTRAQ[0], Lysine(K)_iTRAQ[12]	[5] F4	398/390	0.603	0.874	0.666	1	Mascot
1704.9418	1704.9088	-0.033	-19	130	141	NEWGSLLEELVK	58	99.996	(N-term)_iTRAQ[0], Lysine(K)_iTRAQ[12]	[7] F5 120912	492/484	0.875	1.059	1.266	1	Mascot
1932.0093	1932.0331	0.0238	12	17	35	AIGVLTSGGDAQGMNAAVR	29	96.969	(N-term)_iTRAQ[0]	[8] F13-15 and F1+2	499/491	1.375	1.232	1.062	1	Mascot
2038.0378	2038.0428	0.005	2	445	461	GQVQEVGWHDVAGWLGR	33	98.71	(N-term)_iTRAQ[0]	[4] F7 and F10+11	407/399	1.349	1.566	0.767	1	Mascot
2052.0747	2052.0266	-0.0481	-23	655	672	TNVLGHLQQGGAPTPFDR	63	99.999	(N-term)_iTRAQ[0]	[7] F5 120912	255/247	0.907	0.579	0.769	1	Mascot

259	atlastin-1 [Rattus norvegicus]	gi|57770372	69663.5	6	260	0.854	0.842	0.870	0.258	0.132	0.284	6	6	6	100

Peptide Information

Calc. Mass	Obsrv. Mass	± da	± ppm	Start Seq.	End Seq.	Sequence	Ion Score	C. I. %	Modification	Plate [#] Name	Gel Idx/Pos [4700 Sample Name]	iTRAQ Ratio 115/114*	iTRAQ Ratio 116/114*	iTRAQ Ratio 117/114*	Rank	Result Type
1303.6663	1303.6385	-0.0278	-21	81	89	SFLMDFMLR	31	98.039	(N-term)_iTRAQ[0]	[1] F3 030912	510/502	0.585	0.702	0.557	1	Mascot
1587.8893	1587.9153	0.026	16	536	546	HLYQQAFPAPK	45	99.916	(N-term)_iTRAQ[0], Lysine(K)_iTRAQ[11]	[3] F6 and F9	1184/1176	1.207	0.932	1.110	1	Mascot
1653.8429	1653.7325	-0.1104	-67	242	254	VSGNQHEELQNVR	75	100	(N-term)_iTRAQ[0]	[6] F8 110912	134/126	0.636	0.684	0.620	1	Mascot
1681.9747	1682.0563	0.0816	49	286	295	LKEIDDEFIK	51	99.982	(N-term)_iTRAQ[0], Lysine(K)_iTRAQ[2,10]	[4] F7 and F10+11	1181/1173	1.055	1.018	1.102	1	Mascot
1952.9447	1953.078	0.1333	68	18	31	SSDWSSEEEEPVRK	29	96.56	(N-term)_iTRAQ[0], Lysine(K)_iTRAQ[14]	[3] F6 and F9	1128/1120	0.989	0.901	0.978	1	Mascot
2025.2246	2025.1638	-0.0608	-30	299	313	ILIPWLLSPESLDIK	29	97.197	(N-term)_iTRAQ[0], Lysine(K)_iTRAQ[15]	[1] F3 030912	559/551	0.830	0.866	1.052	1	Mascot

260	RecName: Full=Phosphoglucomutase-1; Short=PGM 1; AltName: Full=Glucose phosphomutase 1	gi|730311	66926.3	5	259	1.078	1.070	0.958	0.264	0.184	0.147	5	5	5	100

Peptide Information

Calc. Mass	Obsrv. Mass	± da	± ppm	Start Seq.	End Seq.	Sequence	Ion Score	C. I. %	Modification	Plate [#] Name	Gel Idx/Pos [4700 Sample Name]	iTRAQ Ratio 115/114*	iTRAQ Ratio 116/114*	iTRAQ Ratio 117/114*	Rank	Result Type
1023.6448	1023.6011	-0.0437	-43	147	152	IFQISK	28	96.104	(N-term)_iTRAQ[0], Lysine(K)_iTRAQ[6]	[3] F6 and F9	248/240	1.040	1.192	0.958	1	Mascot
1673.9294	1673.8953	-0.0341	-20	222	234	IDAMHGVVGPYVK	52	99.984	(N-term)_iTRAQ[0], Lysine(K)_iTRAQ[13]	[4] F7 and F10+11	274/266	0.884	0.882	0.823	1	Mascot
1796.7595	1796.6802	-0.0793	-44	278	293	SGEHDFGAAFDGDGDR	81	100	(N-term)_iTRAQ[0]	[6] F8 110912	206/198	1.621	1.367	0.821	1	Mascot
1804.8486	1804.7135	-0.1351	-75	428	440	YDYEEVEAEGANK	53	99.988	(N-term)_iTRAQ[0], Lysine(K)_iTRAQ[13]	[5] F4	135/127	1.086	0.942	1.046	1	Mascot

Calc. Mass	Obsrv. Mass	± da	± ppm	Start Seq.	End Seq.	Sequence	Ion Score	C. I. %	Modification	Plate [#] Name	Gel Idx/Pos [4700 Sample Name]	iTRAQ Ratio 115/114*	iTRAQ Ratio 116/114*	iTRAQ Ratio 117/114*	Rank	Result Type
1907.0232	1906.9784	-0.0448	-23	9	23	TQAYPDQKPGTSGLR	47	99.948	(N-term)_iTRAQ[0], Lysine(K)_iTRAQ[8]	[4] F7 and F10+11	150/142	0.898	1.036	1.193	1	Mascot

261	adenylyl cyclase-associated protein 2 [Rattus norvegicus]	gi|16758742	58008.5	6	258	0.877	1.023	1.024	0.220	0.127	0.287	7	7	7	100

Peptide Information

Calc. Mass	Obsrv. Mass	± da	± ppm	Start Seq.	End Seq.	Sequence	Ion Score	C. I. %	Modification	Plate [#] Name	Gel Idx/Pos [4700 Sample Name]	iTRAQ Ratio 115/114*	iTRAQ Ratio 116/114*	iTRAQ Ratio 117/114*	Rank	Result Type
1178.6654	1178.5963	-0.0691	-59	115	122	IQEIQTFR	38	99.581	(N-term)_iTRAQ[0]	[5] F4	157/149	0.940	1.184	1.004	1	Mascot
1380.8097	1380.8964	0.0867	63	321	330	HAPVLELEGK	35	99.152	(N-term)_iTRAQ[0], Lysine(K)_iTRAQ[10]	[3] F6 and F9	1183/1175	0.953	1.220	0.658	1	Mascot
1422.7878	1422.7397	-0.0481	-34	452	460	EFPIPEQFK	48	99.963	(N-term)_iTRAQ[0], Lysine(K)_iTRAQ[9]	[7] F5 120912	283/275	0.986	0.995	0.903	1	Mascot
1552.9019	1552.8383	-0.0636	-41	53	63	LINSMVAEFLK	40	99.725	(N-term)_iTRAQ[0], Lysine(K)_iTRAQ[11]	[5] F4	398/390	0.568	0.891	1.368	1	Mascot
1576.7186	1576.6238	-0.0948	-60	159	170	EMNDAATFYTNR	57	99.995	(N-term)_iTRAQ[0]	[5] F4	146/138	1.218	1.019	0.879	1	Mascot
1576.7186	1576.6392	-0.0794	-50	159	170	EMNDAATFYTNR	43	99.865	(N-term)_iTRAQ[0]	[1] F3 030912	216/208	0.878	1.026	1.205	1	Mascot
1853.8823	1853.7896	-0.0927	-50	437	451	SSEMNVLVPQGDDYR	42	99.843	(N-term)_iTRAQ[0]	[1] F3 030912	257/249	0.742	0.876	1.361	1	Mascot

262	ras-related protein Rab-3C [Mus musculus]	gi|13470090	28345.2	4	256	0.736	1.043	1.107	0.147	0.105	0.105	5	5	5	100

Peptide Information

Calc. Mass	Obsrv. Mass	± da	± ppm	Start Seq.	End Seq.	Sequence	Ion Score	C. I. %	Modification	Plate [#] Name	Gel Idx/Pos [4700 Sample Name]	iTRAQ Ratio 115/114*	iTRAQ Ratio 116/114*	iTRAQ Ratio 117/114*	Rank	Result Type
1460.7618	1460.6924	-0.0694	-48	81	91	LQIWDTAGQER	70	100	(N-term)_iTRAQ[0]	[5] F4	197/189	0.762	1.084	0.943	1	Mascot
1686.7812	1686.7174	-0.0638	-38	3	16	HEAPMQMASAQDAR	60	99.997	(N-term)_iTRAQ[0]	[6] F8 110912	150/142	0.861	1.132	1.166	1	Mascot
1686.7812	1686.7601	-0.0211	-13	3	16	HEAPMQMASAQDAR	78	100	(N-term)_iTRAQ[0]	[4] F7 and F10+11	148/140	0.652	0.880	1.067	1	Mascot
1784.8047	1784.8021	-0.0026	-1	21	32	DSSDQNFDYMFK	44	99.898	(N-term)_iTRAQ[0], Lysine(K)_iTRAQ[12]	[7] F5 120912	259/251	0.556	1.006	1.200	1	Mascot
1968.0437	1967.9445	-0.0992	-50	130	144	TYSWDNAQVILAGNK	65	99.999	(N-term)_iTRAQ[0], Lysine(K)_iTRAQ[15]	[5] F4	247/239	0.912	1.137	1.183	1	Mascot

263	Na channel II protein	gi|224960	248760.9	6	256	0.795	0.865	0.879	0.264	0.213	0.319	6	6	6	100

Protein Group

sodium channel protein type 2 subunit alpha [Rattus norvegicus]	gi|6981506	248783

Peptide Information

Calc. Mass	Obsrv. Mass	± da	± ppm	Start Seq.	End Seq.	Sequence	Ion Score	C. I. %	Modification	Plate [#] Name	Gel Idx/Pos [4700 Sample Name]	iTRAQ Ratio 115/114*	iTRAQ Ratio 116/114*	iTRAQ Ratio 117/114*	Rank	Result Type
1658.7791	1658.7382	-0.0409	-25	1423	1435	GWMDIMYAAVDSR	42	99.838	(N-term)_iTRAQ[0]	[1] F3 030912	486/478	0.707	0.695	0.513	1	Mascot
1687.9952	1687.953	-0.0422	-25	1903	1914	KQEEVSAIVIQR	61	99.998	(N-term)_iTRAQ[0], Lysine(K)_iTRAQ[1]	[6] F8 110912	216/208	1.235	1.085	1.239	1	Mascot
1832.8859	1832.7756	-0.1103	-60	456	472	QQEEAQAAAAAASAESR	37	99.55	(N-term)_iTRAQ[0]	[1] F3 030912	189/181	0.479	0.734	0.691	1	Mascot
1894.8947	1894.8416	-0.0531	-28	1805	1817	FDPDATQFIEFCK	41	99.79	(N-term)_iTRAQ[0], Lysine(K)_iTRAQ[13], MMTS (C)[12]	[5] F4	370/362	0.938	0.928	1.175	1	Mascot
2054.1328	2054.0391	-0.0937	-46	443	455	EAEFQQMLEQLKK	30	97.348	(N-term)_iTRAQ[0], Lysine(K)_iTRAQ[12, 13]	[6] F8 110912	410/402	0.779	0.685	0.867	1	Mascot
2091.9944	2091.884	-0.1104	-53	310	323	TVNMFNWDEYIEDK	45	99.93	(N-term)_iTRAQ[0], Lysine(K)_iTRAQ[14]	[5] F4	335/327	0.828	1.196	1.031	1	Mascot

264	synaptic vesicle protein [Rattus norvegicus]	gi|207092	87140.6	5	255	0.908	0.863	0.794	0.331	0.188	0.213	5	5	5	100

Peptide Information

Calc. Mass	Obsrv. Mass	± da	± ppm	Start Seq.	End Seq.	Sequence	Ion Score	C. I. %	Modification	Plate [#] Name	Gel Idx/Pos [4700 Sample Name]	iTRAQ Ratio 115/114*	iTRAQ Ratio 116/114*	iTRAQ Ratio 117/114*	Rank	Result Type
1201.6483	1201.5807	-0.0676	-56	113	123	MADGAPLAGVR	39	99.666	(N-term)_iTRAQ[0]	[1] F3 030912	207/199	0.700	0.701	0.869	1	Mascot
1287.693	1287.7686	0.0756	59	474	483	HLQAVDYAAR	56	99.993	(N-term)_iTRAQ[0]	[3] F6 and F9	1128/1120	1.048	1.086	1.019	1	Mascot
1907.9948	1907.9829	-0.0119	-6	144	157	DREELAQQYETILR	72	100	(N-term)_iTRAQ[0]	[4] F7 and F10+11	307/299	0.711	0.989	0.932	1	Mascot
2173.3206	2173.2766	-0.044	-20	713	732	AAPILFASAALALGSSLALK	59	99.997	(N-term)_iTRAQ[0], Lysine(K)_iTRAQ[20]	[1] F3 030912	589/581	1.570	0.958	0.519	1	Mascot
2706.3132	2706.2468	-0.0664	-25	399	419	TIHQEDELIEIQSDTGTWYQR	30	97.647	(N-term)_iTRAQ[0]	[7] F5 120912	330/322	0.756	0.664	0.736	1	Mascot

265	high molecular weight tau, HMW [rats, dorsal root ganglia, Peptide, 686 aa]	gi|433289	79383.2	4	254	0.865	0.993	0.916	0.343	0.192	0.426	4	4	4	100

Protein Group

RecName: Full=Microtubule-associated protein tau; AltName: Full=Neurofibrillary tangle protein; Alt	gi|13432197	87248.3
big tau [Rattus norvegicus]	gi|207158	79460.3

Peptide Information

Calc. Mass	Obsrv. Mass	± da	± ppm	Start Seq.	End Seq.	Sequence	Ion Score	C. I. %	Modification	Plate [#] Name	Gel Idx/Pos [4700 Sample Name]	iTRAQ Ratio 115/114*	iTRAQ Ratio 116/114*	iTRAQ Ratio 117/114*	Rank	Result Type
1210.6917	1210.6266	-0.0651	-54	457	466	TPSLPTPPTR	28	95.855	(N-term)_iTRAQ[0]	[1] F3 030912	201/193	0.512	0.770	0.474	1	Mascot
1597.9233	1597.8442	-0.0791	-50	488	499	LQTAPVPMPDLK	38	99.575	(N-term)_iTRAQ[0], Lysine(K)_iTRAQ[12]	[5] F4	217/209	1.098	1.232	1.089	1	Mascot
1764.0027	1763.9224	-0.0803	-46	629	640	AKTDHGAEIVYK	53	99.989	(N-term)_iTRAQ[0], Lysine(K)_iTRAQ[2,12]	[2] F12 040912	152/144	0.823	0.934	1.193	1	Mascot
2615.1843	2615.0134	-0.1709	-65	34	56	ESPPQPPADDGSEEPGSETSDAK	136	100	(N-term)_iTRAQ[0], Lysine(K)_iTRAQ[23]	[1] F3 030912	143/135	1.211	1.096	1.144	1	Mascot

266	neuronal cell adhesion molecule long isoform Nc3 [Rattus norvegicus]	gi|46369973	143576.4	3	252	0.869	0.902	1.045	0.172	0.090	0.137	3	3	3	100

Protein Group

RecName: Full=Neuronal cell adhesion molecule; Short=Nr-CAM; AltName: Full=Ankyrin-binding cell adh	gi|38372401	145613.6
ankyrin binding cell adhesion molecule NrCAM [Rattus norvegicus]	gi|1842431	145668.6
neuronal cell adhesion molecule [Rattus norvegicus]	gi|47058952	155130.3
neuronal cell adhesion molecule long isoform Nc1 [Rattus norvegicus]	gi|46369969	144940.2
neuronal cell adhesion molecule long isoform Nc14 [Rattus norvegicus]	gi|46369971	143720.5
neuronal cell adhesion molecule long isoform Nc6 [Rattus norvegicus]	gi|46369975	144338.8
neuronal cell adhesion molecule long isoform Nc7 [Rattus norvegicus]	gi|46369977	143119.1

Peptide Information

Calc. Mass	Obsrv. Mass	± da	± ppm	Start Seq.	End Seq.	Sequence	Ion Score	C. I. %	Modification	Plate [#] Name	Gel Idx/Pos [4700 Sample Name]	iTRAQ Ratio 115/114*	iTRAQ Ratio 116/114*	iTRAQ Ratio 117/114*	Rank	Result Type
1726.9109	1726.7877	-0.1232	-71	725	737	SVPSEASEQYLTK	61	99.998	(N-term)_iTRAQ[0], Lysine(K)_iTRAQ[13]	[5] F4	135/127	0.675	0.814	1.182	1	Mascot
1887.0182	1886.8677	-0.1505	-80	691	705	HQAEVSGTQTTAQLK	131	100	(N-term)_iTRAQ[0], Lysine(K)_iTRAQ[15]	[6] F8 110912	133/125	1.015	1.024	1.094	1	Mascot
1899.0222	1898.9899	-0.0323	-17	939	954	GFHTPEGVPSAPSSLK	60	99.998	(N-term)_iTRAQ[0], Lysine(K)_iTRAQ[16]	[4] F7 and F10+11	250/242	0.957	0.880	0.883	1	Mascot

267	neuromodulin [Rattus norvegicus]	gi|8393415	28148.4	3	252	0.784	0.952	1.054	0.467	0.576	0.668	5	5	5	100

Peptide Information

Calc. Mass	Obsrv. Mass	± da	± ppm	Start Seq.	End Seq.	Sequence	Ion Score	C. I. %	Modification	Plate [#] Name	Gel Idx/Pos [4700 Sample Name]	iTRAQ Ratio 115/114*	iTRAQ Ratio 116/114*	iTRAQ Ratio 117/114*	Rank	Result Type
1804.9664	1804.8452	-0.1212	-67	68	80	EKDDAPVADGVEK	74	100	(N-term)_iTRAQ[0], Lysine(K)_iTRAQ[2,13]	[6] F8 110912	124/116	0.410	0.491	0.532	1	Mascot
1804.9664	1804.9738	0.0074	4	68	80	EKDDAPVADGVEK	80	100	(N-term)_iTRAQ[0], Lysine(K)_iTRAQ[2,13]	[3] F6 and F9	1090/1082	0.545	0.723	0.693	1	Mascot
1985.8934	1985.7396	-0.1538	-77	212	226	QDEGKEDPEADQEHA	66	99.999	(N-term)_iTRAQ[0], Lysine(K)_iTRAQ[5]	[6] F8 110912	70/62	0.803	1.049	1.180	1	Mascot
2191.0613	2190.8911	-0.1702	-78	181	198	AAQPPTETAESSQAEEEK	106	100	(N-term)_iTRAQ[0], Lysine(K)_iTRAQ[18]	[5] F4	68/60	1.507	2.055	2.056	1	Mascot
2191.0613	2190.9192	-0.1421	-65	181	198	AAQPPTETAESSQAEEEK	100	100	(N-term)_iTRAQ[0], Lysine(K)_iTRAQ[18]	[1] F3 030912	123/115	1.095	1.021	1.454	1	Mascot

268	ES1 protein homolog, mitochondrial precursor [Rattus norvegicus]	gi|51948422	31601	4	252	0.906	0.904	0.837	0.235	0.081	0.093	4	4	4	100

Peptide Information

Calc. Mass	Obsrv. Mass	± da	± ppm	Start Seq.	End Seq.	Sequence	Ion Score	C. I. %	Modification	Plate [#] Name	Gel Idx/Pos [4700 Sample Name]	iTRAQ Ratio 115/114*	iTRAQ Ratio 116/114*	iTRAQ Ratio 117/114*	Rank	Result Type
1339.7467	1339.6797	-0.067	-50	140	149	NLSTFAVDGK	42	99.855	(N-term)_iTRAQ[0], Lysine(K)_iTRAQ[10]	[7] F5 120912	189/181	1.342	0.984	0.861	1	Mascot
1480.8046	1480.709	-0.0956	-65	202	212	WPYAGTAEAVK	48	99.965	(N-term)_iTRAQ[0], Lysine(K)_iTRAQ[11]	[5] F4	175/167	0.776	0.784	0.811	1	Mascot
1842.9443	1842.9086	-0.0357	-19	187	201	GVEVTVGHEQEEGGK	129	100	(N-term)_iTRAQ[0], Lysine(K)_iTRAQ[15]	[4] F7 and F10+11	145/137	0.761	0.922	0.723	1	Mascot
2021.1481	2021.1744	0.0263	13	169	183	KPIGLCCIAPVLAAK	32	98.3	(N-term)_iTRAQ[0], Lysine(K)_iTRAQ[1,15], MMTS (C)[6,7]	[4] F7 and F10+11	486/478	0.852	0.937	0.969	1	Mascot

269	peroxiredoxin-6 [Rattus norvegicus]	gi|16758348	27586.9	5	252	1.102	1.085	0.892	0.546	0.541	0.430	6	6	6	100

Peptide Information

Calc. Mass	Obsrv. Mass	± da	± ppm	Start Seq.	End Seq.	Sequence	Ion Score	C. I. %	Modification	Plate [#] Name	Gel Idx/Pos [4700 Sample Name]	iTRAQ Ratio 115/114*	iTRAQ Ratio 116/114*	iTRAQ Ratio 117/114*	Rank	Result Type
1528.7241	1528.7305	0.0064	4	42	53	DFTPVCTTELGR	52	99.985	(N-term)_iTRAQ[0], MMTS (C)[6]	[8] F13-15 and F1+2	454/446	0.847	0.639	0.498	1	Mascot
1528.7241	1528.7545	0.0304	20	42	53	DFTPVCTTELGR	49	99.97	(N-term)_iTRAQ[0], MMTS (C)[6]	[8] F13-15 and F1+2	555/547	2.232	1.917	1.805	1	Mascot
1581.974	1582.031	0.057	36	133	142	VVFIFGPDKK	45	99.915	(N-term)_iTRAQ[0], Lysine(K)_iTRAQ[9,10]	[4] F7 and F10+11	1216/1208	1.117	0.847	0.940	1	Mascot
1651.8538	1651.7523	-0.1015	-61	85	97	DINAYNGAAPTEK	82	100	(N-term)_iTRAQ[0], Lysine(K)_iTRAQ[13]	[5] F4	103/95	1.122	1.128	0.761	1	Mascot
2175.0896	2175.1787	0.0891	41	25	41	FHDFLGDSWGILFSHPR	43	99.878	(N-term)_iTRAQ[0]	[4] F7 and F10+11	1346/1338	0.593	0.802	0.743	1	Mascot
2289.2747	2289.2686	-0.0061	-3	183	199	KGESVMVLPTLPEEEAK	29	97.059	(N-term)_iTRAQ[0], Lysine(K)_iTRAQ[1,17]	[4] F7 and F10+11	313/305	1.272	1.739	1.056	1	Mascot

270	ras-related C3 botulinum toxin substrate 1 isoform Rac1 [Homo sapiens]	gi|9845511	24352	5	252	0.804	0.949	0.900	0.125	0.232	0.288	5	5	5	100

Peptide Information

Calc. Mass	Obsrv. Mass	± da	± ppm	Start Seq.	End Seq.	Sequence	Ion Score	C. I. %	Modification	Plate [#] Name	Gel Idx/Pos [4700 Sample Name]	iTRAQ Ratio 115/114*	iTRAQ Ratio 116/114*	iTRAQ Ratio 117/114*	Rank	Result Type
1094.5966	1094.5447	-0.0519	-47	167	174	TVFDEAIR	45	99.925	(N-term)_iTRAQ[0]	[5] F4	162/154	0.878	1.058	0.589	1	Mascot
1373.6659	1373.6151	-0.0508	-37	154	163	YLECSALTQR	41	99.782	(N-term)_iTRAQ[0], MMTS (C)[4]	[1] F3 030912	320/312	0.863	0.905	0.843	1	Mascot
1792.0288	1791.9604	-0.0684	-38	134	147	LTPITYPQGLAMAK	54	99.99	(N-term)_iTRAQ[0], Lysine(K)_iTRAQ[14]	[1] F3 030912	331/323	0.901	1.011	1.121	1	Mascot
1864.0164	1863.9987	-0.0177	-9	103	116	HHCPNTPIILVGTK	50	99.973	(N-term)_iTRAQ[0], Lysine(K)_iTRAQ[14], MMTS (C)[3]	[2] F12 040912	301/293	0.607	1.236	1.310	1	Mascot
2064.2263	2064.2341	0.0078	4	133	147	KLTPITYPQGLAMAK	63	99.999	(N-term)_iTRAQ[0], Lysine(K)_iTRAQ[1,15]	[4] F7 and F10+11	309/301	0.811	0.644	0.809	1	Mascot

271	elongation factor-1 alpha [Rattus norvegicus]	gi|1220484	57310.9	5	249	1.150	1.138	0.957	0.189	0.341	0.213	5	5	5	100

Protein Group

elongation factor 1-alpha 1 [Rattus norvegicus]	gi|28460696	57274.9

Peptide Information

Calc. Mass	Obsrv. Mass	± da	± ppm	Start Seq.	End Seq.	Sequence	Ion Score	C. I. %	Modification	Plate [#] Name	Gel Idx/Pos [4700 Sample Name]	iTRAQ Ratio 115/114*	iTRAQ Ratio 116/114*	iTRAQ Ratio 117/114*	Rank	Result Type
1169.7126	1169.6604	-0.0522	-45	256	266	IGGIGTVPVGR	51	99.98	(N-term)_iTRAQ[0]	[1] F3 030912	224/216	1.456	1.769	0.841	1	Mascot
1202.7719	1202.746	-0.0259	-22	431	439	QTVAVGVIK	63	99.999	(N-term)_iTRAQ[0], Lysine(K)_iTRAQ[9]	[7] F5 120912	159/151	1.136	1.157	1.166	1	Mascot
1263.7559	1263.7404	-0.0155	-12	248	255	LPLQDVYK	33	98.815	(N-term)_iTRAQ[0], Lysine(K)_iTRAQ[8]	[7] F5 120912	239/231	1.134	0.952	0.852	1	Mascot
1408.8046	1408.8396	0.035	25	21	30	STTTGHLIYK	44	99.911	(N-term)_iTRAQ[0], Lysine(K)_iTRAQ[10]	[3] F6 and F9	1133/1125	0.902	0.813	0.757	1	Mascot
1548.8295	1548.8048	-0.0247	-16	85	96	YYVTIIDAPGHR	58	99.996	(N-term)_iTRAQ[0]	[3] F6 and F9	289/281	1.185	1.206	1.267	1	Mascot

272	T-complex protein 1 subunit epsilon [Rattus norvegicus]	gi|51890219	65774.9	5	249	1.044	0.965	1.064	0.314	0.775	0.377	5	5	5	100

Peptide Information

Calc. Mass	Obsrv. Mass	± da	± ppm	Start Seq.	End Seq.	Sequence	Ion Score	C. I. %	Modification	Plate [#] Name	Gel Idx/Pos [4700 Sample Name]	iTRAQ Ratio 115/114*	iTRAQ Ratio 116/114*	iTRAQ Ratio 117/114*	Rank	Result Type
1297.6909	1297.6511	-0.0398	-31	371	378	MLVIEQCK	39	99.68	(N-term)_iTRAQ[0], Lysine(K)_iTRAQ[8],	[7] F5 120912	289/281	1.009	0.897	0.603	1	Mascot

Calc. Mass	Obsrv. Mass	± da	± ppm	Start Seq.	End Seq.	Sequence	Ion Score	C. I. %	Modification	Plate [#] Name	Gel Idx/Pos [4700 Sample Name]	iTRAQ Ratio 115/114*	iTRAQ Ratio 116/114*	iTRAQ Ratio 117/114*	Rank	Result Type
									MMTS (C)[7]							
1690.9884	1691.0194	0.031	18	514	525	KQQISLATQMVR	34	98.98	(N-term)_iTRAQ[0], Lysine(K)_iTRAQ[1]	[3] F6 and F9	1196/1188	1.283	1.385	1.333	1	Mascot
1798.0156	1798.0673	0.0517	29	283	293	EKFEEMIAQIK	66	99.999	(N-term)_iTRAQ[0], Lysine(K)_iTRAQ[2,11]	[4] F7 and F10+11	1253/1245	1.041	1.144	1.256	1	Mascot
1883.051	1883.0287	-0.0223	-12	324	340	WVGGPEIELIAIATGGR	44	99.895	(N-term)_iTRAQ[0]	[8] F13-15 and F1+2	259/251	0.657	0.326	1.006	1	Mascot
2221.1533	2221.1619	0.0086	4	497	513	GSNDMQYQHVIETLIGK	67	99.999	(N-term)_iTRAQ[0], Lysine(K)_iTRAQ[17]	[4] F7 and F10+11	432/424	1.403	1.808	1.345	1	Mascot

273	cAMP-dependent protein kinase catalytic subunit beta isoform 1 [Mus musculus]	gi|6755076	45719.5	5	248	0.903	1.054	1.280	0.230	0.195	0.613	6	6	6	100

Peptide Information

Calc. Mass	Obsrv. Mass	± da	± ppm	Start Seq.	End Seq.	Sequence	Ion Score	C. I. %	Modification	Plate [#] Name	Gel Idx/Pos [4700 Sample Name]	iTRAQ Ratio 115/114*	iTRAQ Ratio 116/114*	iTRAQ Ratio 117/114*	Rank	Result Type
1392.7079	1392.6631	-0.0448	-32	65	73	ATEQYYAMK	49	99.969	(N-term)_iTRAQ[0], Lysine(K)_iTRAQ[9]	[7] F5 120912	152/144	0.752	0.875	1.225	1	Mascot
1452.7733	1452.8361	0.0628	43	258	267	FPSHFSSDLK	39	99.711	(N-term)_iTRAQ[0], Lysine(K)_iTRAQ[10]	[3] F6 and F9	1208/1200	0.810	0.823	0.926	1	Mascot
1452.7733	1452.8463	0.073	50	258	267	FPSHFSSDLK	41	99.799	(N-term)_iTRAQ[0], Lysine(K)_iTRAQ[10]	[4] F7 and F10+11	1140/1132	1.102	1.260	1.795	1	Mascot
1460.9213	1460.9834	0.0621	43	310	318	KVEAPFIPK	42	99.854	(N-term)_iTRAQ[0], Lysine(K)_iTRAQ[1,9]	[4] F7 and F10+11	1128/1120	1.028	1.222	1.402	1	Mascot
1575.9382	1575.9302	-0.008	-5	95	106	ILQAVEFPFLVR	50	99.975	(N-term)_iTRAQ[0]	[8] F13-15 and F1+2	275/267	1.221	1.221	2.230	1	Mascot
1814.9351	1814.8776	-0.0575	-32	297	309	WFATTDWIAIYQR	66	99.999	(N-term)_iTRAQ[0]	[1] F3 030912	496/488	0.644	1.016	0.690	1	Mascot

274	elongation factor 1-gamma [Rattus norvegicus]	gi|51948418	55204.6	5	247	1.217	1.101	0.949	0.168	0.254	0.200	5	5	5	100

Peptide Information

Calc. Mass	Obsrv. Mass	± da	± ppm	Start Seq.	End Seq.	Sequence	Ion Score	C. I. %	Modification	Plate [#] Name	Gel Idx/Pos [4700 Sample Name]	iTRAQ Ratio 115/114*	iTRAQ Ratio 116/114*	iTRAQ Ratio 117/114*	Rank	Result Type
1410.8931	1410.8186	-0.0745	-53	138	147	ILGLLDTHLK	52	99.985	(N-term)_iTRAQ[0], Lysine(K)_iTRAQ[10]	[6] F8 110912	404/396	1.404	1.472	1.310	1	Mascot
1505.856	1505.782	-0.074	-49	18	30	ALIAAQYSGAQIR	29	96.654	(N-term)_iTRAQ[0]	[1] F3 030912	244/236	1.419	1.295	1.052	1	Mascot
1555.9332	1556.0438	0.1106	71	276	285	AKDPFAHLPK	42	99.849	(N-term)_iTRAQ[0], Lysine(K)_iTRAQ[2,10]	[8] F13-15 and F1+2	1179/1171	1.118	1.108	0.870	1	Mascot
1861.0277	1860.9279	-0.0998	-54	401	414	KLDPGSEETQTLVR	49	99.968	(N-term)_iTRAQ[0], Lysine(K)_iTRAQ[1]	[6] F8 110912	202/194	1.177	0.876	0.837	1	Mascot
1972.9802	1972.8999	-0.0803	-41	415	428	EYFSWEGAFQHVGK	76	100	(N-term)_iTRAQ[0], Lysine(K)_iTRAQ[14]	[6] F8 110912	388/380	1.019	0.876	0.768	1	Mascot

275	T-complex protein 1 subunit beta [Rattus norvegicus]	gi|54400730	63318	5	247	1.152	0.940	1.206	0.553	0.273	0.581	5	5	5	100

Peptide Information

Calc. Mass	Obsrv. Mass	± da	± ppm	Start Seq.	End Seq.	Sequence	Ion Score	C. I. %	Modification	Plate [#] Name	Gel Idx/Pos [4700 Sample Name]	iTRAQ Ratio 115/114*	iTRAQ Ratio 116/114*	iTRAQ Ratio 117/114*	Rank	Result Type
1525.8948	1525.8002	-0.0946	-62	192	203	GSGNLEAIHVIK	33	98.754	(N-term)_iTRAQ[0], Lysine(K)_iTRAQ[12]	[6] F8 110912	239/231	1.168	0.831	1.070	1	Mascot
1714.9711	1714.9391	-0.032	-19	264	274	VAEIEHAEKEK	57	99.995	(N-term)_iTRAQ[0], Lysine(K)_iTRAQ[9,11]	[2] F12 040912	171/163	0.998	0.729	0.982	1	Mascot
1792.083	1792.045	-0.038	-21	26	40	LSSFIGAIAIGDLVK	39	99.677	(N-term)_iTRAQ[0], Lysine(K)_iTRAQ[15]	[1] F3 030912	530/522	2.418	1.518	2.631	1	Mascot
1832.0001	1832.011	0.0109	6	389	402	SLHDALCVLAQTVK	80	100	(N-term)_iTRAQ[0], Lysine(K)_iTRAQ[14], MMTS (C)[7]	[4] F7 and F10+11	470/462	0.910	0.832	1.001	1	Mascot
2100.1045	2100.0461	-0.0584	-28	155	170	FWQDLMNIAGTTLSSK	38	99.6	(N-term)_iTRAQ[0], Lysine(K)_iTRAQ[16]	[5] F4	416/408	0.791	0.957	0.922	1	Mascot

276	caM kinase-like vesicle-associated protein [Rattus norvegicus]	gi|13027458	59483.5	3	247	0.806	0.977	1.253	0.164	0.568	0.237	3	3	3	100

Peptide Information

Calc. Mass	Obsrv. Mass	± da	± ppm	Start Seq.	End Seq.	Sequence	Ion Score	C. I. %	Modification	Plate [#] Name	Gel Idx/Pos [4700 Sample Name]	iTRAQ Ratio 115/114*	iTRAQ Ratio 116/114*	iTRAQ Ratio 117/114*	Rank	Result Type
1759.0364	1759.053	0.0166	9	128	140	QVLEAVAYLHSLK	63	99.999	(N-term)_iTRAQ[0], Lysine(K)_iTRAQ[13]	[4] F7 and F10+11	430/422	1.015	1.583	1.562	1	Mascot
2063.9834	2063.9233	-0.0601	-29	106	120	EVFDWILDQGYYSER	47	99.952	(N-term)_iTRAQ[0]	[1] F3 030912	495/487	0.645	0.528	1.229	1	Mascot
2344.2241	2344.1025	-0.1216	-52	437	458	ATPATEESTVPAAQSSAAPAAK	136	100	(N-term)_iTRAQ[0], Lysine(K)_iTRAQ[22]	[1] F3 030912	157/149	0.801	1.116	1.024	1	Mascot

277	electron transfer flavoprotein subunit alpha, mitochondrial precursor [Rattus norvegicus]	gi|57527204	39096.1	3	246	1.429	1.539	1.256	0.391	0.653	1.068	3	3	3	100

Peptide Information

Calc. Mass	Obsrv. Mass	± da	± ppm	Start Seq.	End Seq.	Sequence	Ion Score	C. I. %	Modification	Plate [#] Name	Gel Idx/Pos [4700 Sample Name]	iTRAQ Ratio 115/114*	iTRAQ Ratio 116/114*	iTRAQ Ratio 117/114*	Rank	Result Type
1902.9291	1902.8159	-0.1132	-59	170	187	GTSFEAAAASGGSASSE K	142	100	(N-term)_iTRAQ[0], Lysine(K)_iTRAQ[18]	[5] F4	102/94	1.117	1.196	1.073	1	Mascot
1951.0018	1950.9023	-0.0995	-51	188	203	APSSSSAGISEWLDQK	59	99.997	(N-term)_iTRAQ[0], Lysine(K)_iTRAQ[16]	[5] F4	234/226	1.315	1.200	0.647	1	Mascot
1957.0627	1957.0609	-0.0018	-1	233	249	LLYDLADQLHAAVGASR	45	99.92	(N-term)_iTRAQ[0]	[7] F5 120912	473/465	1.985	2.537	2.850	1	Mascot

278 GNAS complex locus GNASL [Rattus norvegicus] gi|9506737 49749.5 5 243 1.328 1.238 1.154 0.659 0.273 0.268 5 5 5 100

Protein Group

GTP-binding regulatory protein Gs alpha-XL chain - rat gi|1086315 97253.8

XLalphas protein [Rattus norvegicus] gi|14161099 83150.6

Peptide Information

Calc. Mass	Obsrv. Mass	± da	± ppm	Start Seq.	End Seq.	Sequence	Ion Score	C. I. %	Modification	Plate [#] Name	Gel Idx/Pos [4700 Sample Name]	iTRAQ Ratio 115/114*	iTRAQ Ratio 116/114*	iTRAQ Ratio 117/114*	Rank	Result Type
1345.8301	1345.7644	-0.0657	-49	43	53	LLLLGAGESGK	84	100	(N-term)_iTRAQ[0], Lysine(K)_iTRAQ[11]	[5] F4	219/211	1.116	1.118	1.283	1	Mascot
1550.7657	1550.8171	0.0514	33	217	228	VNFHMFDVGGQR	59	99.997	(N-term)_iTRAQ[0]	[3] F6 and F9	1279/1271	0.788	0.955	1.043	1	Mascot
1789.937	1789.8633	-0.0737	-41	306	317	SKIEDYFPEFAR	36	99.367	(N-term)_iTRAQ[0], Lysine(K)_iTRAQ[2]	[6] F8 110912	378/370	1.139	1.207	0.866	1	Mascot
1918.879	1918.9526	0.0736	38	318	333	YTTPEDATPEPGEDPR	35	99.285	(N-term)_iTRAQ[0]	[8] F13-15 and F1+2	609/601	1.590	1.745	1.093	1	Mascot
2284.0857	2284.0154	-0.0703	-31	166	181	SNEYQLIDCAQYFLDK	28	96.339	(N-term)_iTRAQ[0], Lysine(K)_iTRAQ[16], MMTS (C)[9]	[5] F4	409/401	2.600	1.292	1.613	1	Mascot

279 myc box-dependent-interacting protein 1 [Rattus norvegicus] gi|16758846 71161.8 5 242 1.703 1.387 1.412 2.469 0.752 1.046 5 5 5 100

Peptide Information

Calc. Mass	Obsrv. Mass	± da	± ppm	Start Seq.	End Seq.	Sequence	Ion Score	C. I. %	Modification	Plate [#] Name	Gel Idx/Pos [4700 Sample Name]	iTRAQ Ratio 115/114*	iTRAQ Ratio 116/114*	iTRAQ Ratio 117/114*	Rank	Result Type
1322.6576	1322.6245	-0.0331	-25	181	189	AAPQWCQGK	59	99.997	(N-term)_iTRAQ[0], Lysine(K)_iTRAQ[9], MMTS (C)[6]	[3] F6 and F9	232/224	1.311	1.555	1.586	1	Mascot
1339.6766	1339.5911	-0.0855	-64	577	586	GVFPENFTER	27	95.207	(N-term)_iTRAQ[0]	[5] F4	190/182	1.412	1.230	1.287	1	Mascot
1819.931	1819.9845	0.0535	29	296	312	SPSPPPDGSPAATPEIR	44	99.898	(N-term)_iTRAQ[0]	[8] F13-15 and F1+2	600/592	9.711	3.020	3.609	1	Mascot
2178.0898	2178.0508	-0.039	-18	103	117	IAENNDLLWMDYHQK	34	99.024	(N-term)_iTRAQ[0], Lysine(K)_iTRAQ[15]	[4] F7 and F10+11	337/329	0.769	0.886	0.652	1	Mascot
2569.3833	2569.3276	-0.0557	-22	118	137	LVDQALLTMDTYLGQFP DIK	79	100	(N-term)_iTRAQ[0], Lysine(K)_iTRAQ[20]	[1] F3 030912	567/559	1.036	1.005	1.171	1	Mascot

280 importin subunit beta-1 [Rattus norvegicus] gi|8393610 104316.1 5 242 1.263 1.029 0.586 0.510 0.328 0.375 5 5 5 100

Peptide Information

Calc. Mass	Obsrv. Mass	± da	± ppm	Start Seq.	End Seq.	Sequence	Ion Score	C. I. %	Modification	Plate [#] Name	Gel Idx/Pos [4700 Sample Name]	iTRAQ Ratio 115/114*	iTRAQ Ratio 116/114*	iTRAQ Ratio 117/114*	Rank	Result Type
1173.65	1173.5891	-0.0609	-52	79	87	WLAIDANAR	46	99.94	(N-term)_iTRAQ[0]	[5] F4	205/197	0.966	1.090	0.633	1	Mascot
1589.8805	1589.8358	-0.0447	-28	169	181	SNEILTAIIQGMR	44	99.908	(N-term)_iTRAQ[0]	[1] F3 030912	495/487	1.737	1.195	0.788	1	Mascot
1803.0137	1803.0066	-0.0071	-4	28	42	AAVENLPTFLVELSR	27	95.579	(N-term)_iTRAQ[0]	[8] F13-15 and F1+2	286/278	1.915	1.490	0.264	1	Mascot
1977.9937	1977.9215	-0.0722	-37	777	792	GDQENVHPDVMLVQPR	68	100	(N-term)_iTRAQ[0]	[7] F5 120912	193/185	0.783	0.655	0.460	1	Mascot
2047.2413	2046.981	-0.2603	-127	316	331	GALQYLVPILTQTLTK	56	99.994	(N-term)_iTRAQ[0], Lysine(K)_iTRAQ[16]	[1] F3 030912	554/546	1.275	0.906	1.136	1	Mascot

281 hypoxanthine-guanine phosphoribosyltransferase [Rattus norvegicus] gi|51092266 27239.3 4 242 0.940 1.106 1.146 0.286 0.537 0.187 4 4 4 100

Peptide Information

Calc. Mass	Obsrv. Mass	± da	± ppm	Start Seq.	End Seq.	Sequence	Ion Score	C. I. %	Modification	Plate [#] Name	Gel Idx/Pos [4700 Sample Name]	iTRAQ Ratio 115/114*	iTRAQ Ratio 116/114*	iTRAQ Ratio 117/114*	Rank	Result Type
1441.811	1441.8129	0.0019	1	35	45	VFIPHGLIMDR	56	99.994	(N-term)_iTRAQ[0]	[4] F7 and F10+11	356/348	0.959	0.956	1.144	1	Mascot
1532.8611	1532.8396	-0.0215	-14	74	83	FFADLLDYIK	57	99.995	(N-term)_iTRAQ[0], Lysine(K)_iTRAQ[10]	[5] F4	412/404	0.636	0.745	1.082	1	Mascot

1664.7487	1664.7109	-0.0378	-23	104	115	SYCNDQSTGDIK	63	99.999	(N-term)_iTRAQ[0], Lysine(K)_iTRAQ[12], MMTS (C)[3]	[7] F5 120912	154/146	0.952	0.982	0.958	1	Mascot
2114.1167	2114.1084	-0.0083	-4	171	186	SVGYRPDFVGFEIPDK	65	99.999	(N-term)_iTRAQ[0], Lysine(K)_iTRAQ[16]	[4] F7 and F10+11	373/365	1.345	2.139	1.453	1	Mascot

282	RecName: Full=Myristoylated alanine-rich C-kinase substrate; Short=MARCKS; AltName: Full=Protein ki	gi|266495	33281.4	3	239	1.236	1.128	1.059	0.481	0.620	0.385	5	5	5	100

Peptide Information

Calc. Mass	Obsrv. Mass	± da	± ppm	Start Seq.	End Seq.	Sequence	Ion Score	C. I. %	Modification	Plate [#] Name	Gel Idx/Pos [4700 Sample Name]	iTRAQ Ratio 115/114*	iTRAQ Ratio 116/114*	iTRAQ Ratio 117/114*	Rank	Result Type
2004.0621	2003.9344	-0.1277	-64	131	146	AEDGAAPSPSSETPKK	90	100	(N-term)_iTRAQ[0], Lysine(K)_iTRAQ[15, 16]	[6] F8 110912	99/91	0.808	0.520	1.242	1	Mascot
2004.0621	2004.1083	0.0462	23	131	146	AEDGAAPSPSSETPKK	65	99.999	(N-term)_iTRAQ[0], Lysine(K)_iTRAQ[15, 16]	[3] F6 and F9	1067/1059	1.947	1.631	1.452	1	Mascot
2073.0823	2072.9778	-0.1045	-50	12	30	GEAAAERPGEAAVASSP SK	76	100	(N-term)_iTRAQ[0], Lysine(K)_iTRAQ[19]	[3] F6 and F9	130/122	1.107	0.925	0.925	1	Mascot
2073.0823	2072.9988	-0.0835	-40	12	30	GEAAAERPGEAAVASSP SK	104	100	(N-term)_iTRAQ[0], Lysine(K)_iTRAQ[19]	[4] F7 and F10+11	130/122	1.673	1.557	1.299	1	Mascot
3846.804	3846.5459	-0.2581	-67	220	252	EAEAAEPEQPEQPEQPA AEEPRAEEPSEAVGEK	45	99.922	(N-term)_iTRAQ[0], Lysine(K)_iTRAQ[33]	[1] F3 030912	194/186	0.991	1.497	0.616	1	Mascot

283	myelin-oligodendrocyte glycoprotein precursor [Rattus norvegicus]	gi|158262022	29239.4	4	239	1.850	1.000	0.732	0.196	0.380	0.085	5	5	5	100

Protein Group

RecName: Full=Myelin-oligodendrocyte glycoprotein; Flags: Precursor	gi|2497314	29246.5
myelin/oligodendrocyte glycoprotein [Rattus norvegicus]	gi|8381631	26284.9

Peptide Information

Calc. Mass	Obsrv. Mass	± da	± ppm	Start Seq.	End Seq.	Sequence	Ion Score	C. I. %	Modification	Plate [#] Name	Gel Idx/Pos [4700 Sample Name]	iTRAQ Ratio 115/114*	iTRAQ Ratio 116/114*	iTRAQ Ratio 117/114*	Rank	Result Type
1319.7444	1319.7113	-0.0331	-25	233	242	LAGQFLEELR	32	98.361	(N-term)_iTRAQ[0]	[1] F3 030912	399/391	1.866	1.565	0.722	1	Mascot
1618.6771	1618.6234	-0.0537	-33	117	128	FSDEGGYTCFFR	50	99.977	(N-term)_iTRAQ[0], MMTS (C)[9]	[5] F4	307/299	1.675	0.983	0.597	1	Mascot
1618.6771	1618.6261	-0.051	-32	117	128	FSDEGGYTCFFR	47	99.948	(N-term)_iTRAQ[0], MMTS (C)[9]	[1] F3 030912	397/389	2.228	1.042	0.789	1	Mascot
1677.9084	1677.8652	-0.0432	-26	233	245	LAGQFLEELRNPF	47	99.953	(N-term)_iTRAQ[0]	[1] F3 030912	514/506	1.816	0.574	0.815	1	Mascot
1806.912	1806.8303	-0.0817	-45	129	141	DHSYQEEAAVELK	110	100	(N-term)_iTRAQ[0], Lysine(K)_iTRAQ[13]	[6] F8 110912	205/197	1.715	1.088	0.758	1	Mascot

284	NADH dehydrogenase [ubiquinone] 1 subunit C2 [Rattus norvegicus]	gi|57164133	15548.1	5	238	0.780	0.939	0.928	0.291	0.232	0.461	5	5	5	100

Peptide Information

Calc. Mass	Obsrv. Mass	± da	± ppm	Start Seq.	End Seq.	Sequence	Ion Score	C. I. %	Modification	Plate [#] Name	Gel Idx/Pos [4700 Sample Name]	iTRAQ Ratio 115/114*	iTRAQ Ratio 116/114*	iTRAQ Ratio 117/114*	Rank	Result Type
1399.7467	1399.8186	0.0719	51	96	104	LHPEDFPEK	47	99.949	(N-term)_iTRAQ[0], Lysine(K)_iTRAQ[9]	[4] F7 and F10+11	1093/1085	0.446	0.620	0.512	1	Mascot
1413.7083	1413.7781	0.0698	49	87	95	DHDMFGYIK	54	99.989	(N-term)_iTRAQ[0], Lysine(K)_iTRAQ[9]	[4] F7 and F10+11	1158/1150	0.812	1.116	1.440	1	Mascot
1715.9241	1715.8783	-0.0458	-27	108	120	TYAEILEPFHPVR	48	99.957	(N-term)_iTRAQ[0]	[3] F6 and F9	364/356	0.723	0.909	0.651	1	Mascot
1800.9867	1800.9095	-0.0772	-43	96	106	LHPEDFPEKEK	45	99.919	(N-term)_iTRAQ[0], Lysine(K)_iTRAQ[9,1 1]	[2] F12 040912	199/191	0.983	1.121	1.122	1	Mascot
1988.1215	1988.1749	0.0534	27	107	120	KTYAEILEPFHPVR	45	99.929	(N-term)_iTRAQ[0], Lysine(K)_iTRAQ[1]	[4] F7 and F10+11	1225/1217	1.119	1.035	1.281	1	Mascot

285	ubiquitin carboxyl-terminal hydrolase PGP9.5 [Rattus norvegicus]	gi|220924	27347.9	4	238	1.028	1.106	1.186	0.259	0.310	0.434	5	5	5	100

Protein Group

ubiquitin carboxyl-terminal hydrolase isozyme L1 [Rattus norvegicus]	gi|61098212	27548.1

Peptide Information

Calc. Mass	Obsrv. Mass	± da	± ppm	Start Seq.	End Seq.	Sequence	Ion Score	C. I. %	Modification	Plate [#] Name	Gel Idx/Pos [4700 Sample Name]	iTRAQ Ratio 115/114*	iTRAQ Ratio 116/114*	iTRAQ Ratio 117/114*	Rank	Result Type

Calc. Mass	Obsrv. Mass	± da	± ppm	Start Seq.	End Seq.	Sequence	Ion Score	C. I. %	Modification	Plate [#] Name	Gel Idx/Pos [4700 Sample Name]	iTRAQ Ratio 115/114*	iTRAQ Ratio 116/114*	iTRAQ Ratio 117/114*	Rank	Result Type
1917.1028	1916.9924	-0.1104	-58	66	78	QIEELKGQEVSPK	101	100	(N-term)_iTRAQ[0], Lysine(K)_iTRAQ[6,13]	[6] F8 110912	195/187	0.980	0.864	1.042	1	Mascot
1917.1028	1917.1539	0.0511	27	66	78	QIEELKGQEVSPK	86	100	(N-term)_iTRAQ[0], Lysine(K)_iTRAQ[6,13]	[3] F6 and F9	1153/1145	1.505	1.634	2.102	1	Mascot
2116.9604	2116.8691	-0.0913	-43	136	153	NEAIQAAHDSVAQEGQCR	43	99.875	(N-term)_iTRAQ[0], MMTS (C)[17]	[3] F6 and F9	209/201	1.078	1.192	1.229	1	Mascot
2248.2239	2248.2178	-0.0061	-3	1	15	MQLKPMEINPEMLNK	59	99.997	(N-term)_iTRAQ[0], Lysine(K)_iTRAQ[4,15]	[4] F7 and F10+11	353/345	0.961	1.184	1.048	1	Mascot
2502.2544	2502.2	-0.0544	-22	179	199	MPFPVNHGASSEDSLLQDAAK	36	99.388	(N-term)_iTRAQ[0], Lysine(K)_iTRAQ[21]	[7] F5 120912	303/295	0.752	0.831	0.833	1	Mascot

286	microtubule-associated protein RP/EB family member 3 [Mus musculus]	gi|39930509	35490.2	5	236	1.400	1.387	1.032	0.772	0.647	0.151	6	6	6	100

Peptide Information

Calc. Mass	Obsrv. Mass	± da	± ppm	Start Seq.	End Seq.	Sequence	Ion Score	C. I. %	Modification	Plate [#] Name	Gel Idx/Pos [4700 Sample Name]	iTRAQ Ratio 115/114*	iTRAQ Ratio 116/114*	iTRAQ Ratio 117/114*	Rank	Result Type
1094.5768	1094.5365	-0.0403	-37	224	229	DFYFSK	33	98.84	(N-term)_iTRAQ[0], Lysine(K)_iTRAQ[6]	[3] F6 and F9	242/234	1.362	1.423	1.078	1	Mascot
1617.8635	1617.9457	0.0822	51	67	76	LEHEYIHNFK	47	99.947	(N-term)_iTRAQ[0], Lysine(K)_iTRAQ[10]	[8] F13-15 and F1+2	1185/1177	0.856	0.709	0.955	1	Mascot
1617.8635	1617.9532	0.0897	55	67	76	LEHEYIHNFK	41	99.805	(N-term)_iTRAQ[0], Lysine(K)_iTRAQ[10]	[8] F13-15 and F1+2	1517/1509	3.181	2.283	1.230	1	Mascot
1636.8706	1636.9392	0.0686	42	113	122	KFFDANYDGK	69	100	(N-term)_iTRAQ[0], Lysine(K)_iTRAQ[1,10]	[4] F7 and F10+11	1111/1103	1.279	1.255	1.208	1	Mascot
1936.9844	1936.9209	-0.0635	-33	101	112	FQDNFEFIQWFK	30	97.414	(N-term)_iTRAQ[0], Lysine(K)_iTRAQ[12]	[7] F5 120912	489/481	1.743	1.202	0.849	1	Mascot
2210.145	2210.0295	-0.1155	-52	131	148	QGQDVAPPPNPGDQIFNK	58	99.996	(N-term)_iTRAQ[0], Lysine(K)_iTRAQ[18]	[1] F3 030912	241/233	0.911	2.047	0.933	1	Mascot

287	RecName: Full=ATP synthase subunit gamma, mitochondrial; AltName: Full=F-ATPase gamma subunit	gi|728931	34252.6	4	236	0.819	0.813	1.137	0.280	0.468	0.237	4	4	4	100

Protein Group

ATP synthase subunit gamma, mitochondrial [Rattus norvegicus]	gi|39930503	37102
Chain G, Rat Liver F1-Atpase	gi|6729936	33851.3
LRRGT00199 [Rattus norvegicus]	gi|45478238	74321.2

Peptide Information

Calc. Mass	Obsrv. Mass	± da	± ppm	Start Seq.	End Seq.	Sequence	Ion Score	C. I. %	Modification	Plate [#] Name	Gel Idx/Pos [4700 Sample Name]	iTRAQ Ratio 115/114*	iTRAQ Ratio 116/114*	iTRAQ Ratio 117/114*	Rank	Result Type
1362.7913	1362.7122	-0.0791	-58	102	111	EVMIVGIGEK	64	99.999	(N-term)_iTRAQ[0], Lysine(K)_iTRAQ[10]	[5] F4	192/184	1.361	1.492	1.122	1	Mascot
1588.8832	1588.8009	-0.0823	-52	43	54	VYGTGSLALYEK	61	99.998	(N-term)_iTRAQ[0], Lysine(K)_iTRAQ[12]	[5] F4	197/189	0.701	0.633	0.963	1	Mascot
1596.8632	1596.8882	0.025	16	119	129	THSDQFLVSFK	79	100	(N-term)_iTRAQ[0], Lysine(K)_iTRAQ[11]	[3] F6 and F9	1248/1240	0.708	1.023	0.995	1	Mascot
2027.0841	2027.1075	0.0234	12	238	252	NASDMIDKLTLTFNR	33	98.677	(N-term)_iTRAQ[0], Lysine(K)_iTRAQ[8]	[4] F7 and F10+11	419/411	0.665	0.453	1.553	1	Mascot

288	prohibitin [Mus musculus]	gi|6679299	31721.2	6	235	0.674	0.965	0.816	0.255	0.209	0.411	6	6	6	100

Peptide Information

Calc. Mass	Obsrv. Mass	± da	± ppm	Start Seq.	End Seq.	Sequence	Ion Score	C. I. %	Modification	Plate [#] Name	Gel Idx/Pos [4700 Sample Name]	iTRAQ Ratio 115/114*	iTRAQ Ratio 116/114*	iTRAQ Ratio 117/114*	Rank	Result Type
1293.6923	1293.6521	-0.0402	-31	134	143	FDAGELITQR	31	98.057	(N-term)_iTRAQ[0]	[1] F3 030912	269/261	0.338	0.958	0.362	1	Mascot
1311.7042	1311.6744	-0.0298	-23	178	186	EFTEAVEAK	48	99.963	(N-term)_iTRAQ[0], Lysine(K)_iTRAQ[9]	[7] F5 120912	158/150	0.886	0.908	1.020	1	Mascot
1329.7611	1329.6885	-0.0726	-55	84	93	DLQNVNITLR	35	99.233	(N-term)_iTRAQ[0]	[1] F3 030912	246/238	0.754	1.346	1.015	1	Mascot
1604.7565	1604.6727	-0.0838	-52	106	117	IYTSIGEDYDER	44	99.897	(N-term)_iTRAQ[0]	[1] F3 030912	238/230	0.668	0.722	0.864	1	Mascot
1895.0483	1894.9716	-0.0767	-40	240	253	KLEAAEDIAYQLSR	32	98.361	(N-term)_iTRAQ[0], Lysine(K)_iTRAQ[1]	[6] F8 110912	378/370	0.817	0.865	0.705	1	Mascot
2142.189	2142.1646	-0.0244	-11	220	239	AAELIANSLATAGDGLIELR	45	99.927	(N-term)_iTRAQ[0]	[8] F13-15 and F1+2	260/252	0.764	1.107	1.298	1	Mascot

289	dihydropyrimidinase-related protein 5 [Rattus norvegicus]	gi|12711692	66572.4	5	235	0.827	0.891	0.866	0.181	0.243	0.375	6	6	6	100

Protein Group

dihydropyrimidinase-related protein [Rattus norvegicus]	gi|6714522	66237.2

Peptide Information

Calc. Mass	Obsrv. Mass	± da	± ppm	Start Seq.	End Seq.	Sequence	Ion Score	C. I. %	Modification	Plate [#] Name	Gel Idx/Pos [4700 Sample Name]	iTRAQ Ratio 115/114*	iTRAQ Ratio 116/114*	iTRAQ Ratio 117/114*	Rank	Result Type
1382.7889	1382.7042	-0.0847	-61	373	383	FVAVTSSNAAK	27	95.05	(N-term)_iTRAQ[0], Lysine(K)_iTRAQ[11]	[5] F4	115/107	0.808	1.448	1.419	1	Mascot
1657.7568	1657.6913	-0.0655	-40	171	182	DSELYQVFHACR	36	99.385	(N-term)_iTRAQ[0], MMTS (C)[11]	[6] F8 110912	416/408	0.986	0.952	1.058	1	Mascot
1657.7568	1657.7792	0.0224	14	171	182	DSELYQVFHACR	37	99.526	(N-term)_iTRAQ[0], MMTS (C)[11]	[4] F7 and F10+11	414/406	0.543	0.746	0.509	1	Mascot
1817.0419	1816.9812	-0.0607	-33	490	504	TPYLGDVAVVVNPGK	57	99.995	(N-term)_iTRAQ[0], Lysine(K)_iTRAQ[15]	[1] F3 030912	323/315	0.936	0.688	0.959	1	Mascot
2089.2393	2089.2258	-0.0135	-6	490	505	TPYLGDVAVVVNPGKK	50	99.973	(N-term)_iTRAQ[0], Lysine(K)_iTRAQ[15, 16]	[4] F7 and F10+11	309/301	0.883	0.802	1.011	1	Mascot
2184.1797	2184.1313	-0.0484	-22	394	411	IIPGADADVVVWDPEATK	65	99.999	(N-term)_iTRAQ[0], Lysine(K)_iTRAQ[18]	[1] F3 030912	389/381	0.896	0.881	0.568	1	Mascot

290	statin-related protein [Rattus norvegicus]	gi|206440	57356	5	234	1.103	1.155	0.910	0.199	0.353	0.147	5	5	5	100

Protein Group

elongation factor 1-alpha 2 [Mus musculus]	gi|6681273	57471

Peptide Information

Calc. Mass	Obsrv. Mass	± da	± ppm	Start Seq.	End Seq.	Sequence	Ion Score	C. I. %	Modification	Plate [#] Name	Gel Idx/Pos [4700 Sample Name]	iTRAQ Ratio 115/114*	iTRAQ Ratio 116/114*	iTRAQ Ratio 117/114*	Rank	Result Type
1169.7126	1169.6604	-0.0522	-45	256	266	IGGIGTVPVGR	51	99.98	(N-term)_iTRAQ[0]	[1] F3 030912	224/216	1.456	1.769	0.841	1	Mascot
1202.7719	1202.746	-0.0259	-22	431	439	QTVAVGVIK	63	99.999	(N-term)_iTRAQ[0], Lysine(K)_iTRAQ[9]	[7] F5 120912	159/151	1.136	1.157	1.166	1	Mascot
1263.7559	1263.7404	-0.0155	-12	248	255	LPLQDVYK	33	98.815	(N-term)_iTRAQ[0], Lysine(K)_iTRAQ[8]	[7] F5 120912	239/231	1.134	0.952	0.852	1	Mascot
1408.8046	1408.8396	0.035	25	21	30	STTTGHLIYK	44	99.911	(N-term)_iTRAQ[0], Lysine(K)_iTRAQ[10]	[3] F6 and F9	1133/1125	0.902	0.813	0.757	1	Mascot
1562.8451	1562.8141	-0.031	-20	85	96	YYITIIDAPGHR	43	99.888	(N-term)_iTRAQ[0]	[3] F6 and F9	315/307	0.966	1.296	0.985	1	Mascot

291	ras-related protein Rab-3C [Rattus norvegicus]	gi|19424194	28368.2	4	234	0.666	1.033	1.044	0.128	0.096	0.132	5	5	5	100

Peptide Information

Calc. Mass	Obsrv. Mass	± da	± ppm	Start Seq.	End Seq.	Sequence	Ion Score	C. I. %	Modification	Plate [#] Name	Gel Idx/Pos [4700 Sample Name]	iTRAQ Ratio 115/114*	iTRAQ Ratio 116/114*	iTRAQ Ratio 117/114*	Rank	Result Type
1460.7618	1460.6924	-0.0694	-48	81	91	LQIWDTAGQER	70	100	(N-term)_iTRAQ[0]	[5] F4	197/189	0.762	1.084	0.943	1	Mascot
1686.7812	1686.7174	-0.0638	-38	3	16	HEAPMQMASAQDAR	60	99.997	(N-term)_iTRAQ[0]	[6] F8 110912	150/142	0.861	1.132	1.166	1	Mascot
1686.7812	1686.7601	-0.0211	-13	3	16	HEAPMQMASAQDAR	78	100	(N-term)_iTRAQ[0]	[4] F7 and F10+11	148/140	0.652	0.880	1.067	1	Mascot
1784.8047	1784.8021	-0.0026	-1	21	32	DSSDQNFDYMFK	44	99.898	(N-term)_iTRAQ[0], Lysine(K)_iTRAQ[12]	[7] F5 120912	259/251	0.556	1.006	1.200	1	Mascot
1996.075	1995.9971	-0.0779	-39	130	144	TYSWDNAQVILVGNK	43	99.865	(N-term)_iTRAQ[0], Lysine(K)_iTRAQ[15]	[5] F4	270/262	0.549	1.082	0.883	1	Mascot

292	voltage-dependent calcium channel subunit alpha-2/delta-1 isoform 2 [Rattus norvegicus]	gi|161086906	132920.6	5	234	0.964	0.895	1.095	0.059	0.184	0.420	5	5	5	100

Protein Group

RecName: Full=Voltage-dependent calcium channel subunit alpha-2/delta-1; AltName: Full=Voltage-gate	gi|1705853	134607.7
voltage-dependent calcium channel subunit alpha-2/delta-1 isoform 1 [Rattus norvegicus]	gi|31542335	134252.4

Peptide Information

Calc. Mass	Obsrv. Mass	± da	± ppm	Start Seq.	End Seq.	Sequence	Ion Score	C. I. %	Modification	Plate [#] Name	Gel Idx/Pos [4700 Sample Name]	iTRAQ Ratio 115/114*	iTRAQ Ratio 116/114*	iTRAQ Ratio 117/114*	Rank	Result Type
1308.7773	1308.751	-0.0263	-20	778	786	AVELYIQGK	45	99.924	(N-term)_iTRAQ[0], Lysine(K)_iTRAQ[9]	[7] F5 120912	211/203	0.954	0.983	1.388	1	Mascot
1524.7852	1524.74	-0.0452	-30	357	368	IIMLFTDGGEER	30	97.508	(N-term)_iTRAQ[0]	[1] F3 030912	392/384	0.982	0.925	1.397	1	Mascot
1726.8521	1726.7705	-0.0816	-47	64	76	YQDLYTVEPNNAR	48	99.963	(N-term)_iTRAQ[0]	[1] F3 030912	227/219	0.863	0.954	1.217	1	Mascot
2464.2705	2464.2	-0.0705	-29	534	552	SQEPVTLDFLDAELENDIK	64	99.999	(N-term)_iTRAQ[0], Lysine(K)_iTRAQ[19]	[1] F3 030912	477/469	1.008	1.062	1.143	1	Mascot

Calc. Mass	Obsrv. Mass	± da	± ppm	Start Seq.	End Seq.	Sequence	Ion Score	C. I. %	Modification	Plate [#] Name	Gel Idx/Pos [4700 Sample Name]	iTRAQ Ratio 115/114*	iTRAQ Ratio 116/114*	iTRAQ Ratio 117/114*	Rank	Result Type
2624.1675	2624.0647	-0.1028	-39	728	746	EAGENWQENPETYEDSF YK	46	99.941	(N-term)_iTRAQ[0], Lysine(K)_iTRAQ[19]	[1] F3 030912	299/291	1.020	0.622	0.583	1	Mascot

293	serine/threonine-protein phosphatase 2A 55 kDa regulatory subunit B alpha isoform [Rattus norvegicu	gi|16758910	56526.5	4	233	0.964	0.907	0.976	0.265	0.124	0.234	4	4	4	100

Peptide Information

Calc. Mass	Obsrv. Mass	± da	± ppm	Start Seq.	End Seq.	Sequence	Ion Score	C. I. %	Modification	Plate [#] Name	Gel Idx/Pos [4700 Sample Name]	iTRAQ Ratio 115/114*	iTRAQ Ratio 116/114*	iTRAQ Ratio 117/114*	Rank	Result Type
1390.8206	1390.9143	0.0937	67	419	427	ILHTAWHPK	30	97.732	(N-term)_iTRAQ[0], Lysine(K)_iTRAQ[9]	[8] F13-15 and F1+2	1519/1511	1.325	0.800	1.344	1	Mascot
1553.8561	1553.7853	-0.0708	-46	200	210	INLWHLEITDR	65	99.999	(N-term)_iTRAQ[0]	[6] F8 110912	393/385	0.716	0.841	0.858	1	Mascot
1649.9108	1649.8556	-0.0552	-33	52	62	VVIFQQEQENK	58	99.996	(N-term)_iTRAQ[0], Lysine(K)_iTRAQ[11]	[7] F5 120912	186/178	1.110	1.119	0.760	1	Mascot
1847.0048	1846.9648	-0.04	-22	279	292	SFFSEIISSISDVK	79	100	(N-term)_iTRAQ[0], Lysine(K)_iTRAQ[14]	[5] F4	422/414	0.818	0.899	1.034	1	Mascot

294	alpha-soluble NSF attachment protein [Rattus norvegicus]	gi|18034791	37429.9	3	232	0.914	0.932	0.752	0.182	0.147	0.276	4	4	4	100

Peptide Information

Calc. Mass	Obsrv. Mass	± da	± ppm	Start Seq.	End Seq.	Sequence	Ion Score	C. I. %	Modification	Plate [#] Name	Gel Idx/Pos [4700 Sample Name]	iTRAQ Ratio 115/114*	iTRAQ Ratio 116/114*	iTRAQ Ratio 117/114*	Rank	Result Type
1533.8584	1533.8179	-0.0405	-26	272	282	LDQWLTTMLLR	44	99.908	(N-term)_iTRAQ[0]	[5] F4	416/408	0.872	0.814	1.109	1	Mascot
1533.8584	1533.8236	-0.0348	-23	272	282	LDQWLTTMLLR	59	99.997	(N-term)_iTRAQ[0]	[1] F3 030912	544/536	0.693	0.865	0.691	1	Mascot
1846.9221	1846.8303	-0.0918	-50	141	153	AIAHYEQSADYYK	87	100	(N-term)_iTRAQ[0], Lysine(K)_iTRAQ[13]	[6] F8 110912	217/209	1.058	0.899	0.877	1	Mascot
2339.2415	2339.1401	-0.1014	-43	181	199	AIDIYEQVGTSAMDSPLL K	86	100	(N-term)_iTRAQ[0], Lysine(K)_iTRAQ[19]	[1] F3 030912	423/415	1.091	1.192	0.476	1	Mascot

295	ADP-ribosylation factor 2 [Mus musculus]	gi|6671571	22796.2	3	229	0.891	1.061	1.255	0.139	0.229	0.196	3	3	3	100

Protein Group

ADP-ribosylation factor 1 [Homo sapiens]	gi|4502201	22458.9
ADP-ribosylation factor 3 [Homo sapiens]	gi|4502203	22651.2

Peptide Information

Calc. Mass	Obsrv. Mass	± da	± ppm	Start Seq.	End Seq.	Sequence	Ion Score	C. I. %	Modification	Plate [#] Name	Gel Idx/Pos [4700 Sample Name]	iTRAQ Ratio 115/114*	iTRAQ Ratio 116/114*	iTRAQ Ratio 117/114*	Rank	Result Type
1375.8229	1375.7595	-0.0634	-46	20	30	ILMVGLDAAGK	67	100	(N-term)_iTRAQ[0], Lysine(K)_iTRAQ[11]	[5] F4	250/242	1.041	1.326	1.428	1	Mascot
1853.9644	1853.9092	-0.0552	-30	60	73	NISFTVWDVGGQDK	83	100	(N-term)_iTRAQ[0], Lysine(K)_iTRAQ[14]	[5] F4	268/260	0.925	1.093	1.025	1	Mascot
1918.979	1918.8563	-0.1227	-64	128	142	QDLPNAMNAAEITDK	78	100	(N-term)_iTRAQ[0], Lysine(K)_iTRAQ[15]	[5] F4	177/169	0.734	0.824	1.351	1	Mascot

296	CD166 antigen precursor [Rattus norvegicus]	gi|13929058	72017.3	4	228	1.159	1.159	1.195	0.289	0.324	0.565	4	4	4	100

Peptide Information

Calc. Mass	Obsrv. Mass	± da	± ppm	Start Seq.	End Seq.	Sequence	Ion Score	C. I. %	Modification	Plate [#] Name	Gel Idx/Pos [4700 Sample Name]	iTRAQ Ratio 115/114*	iTRAQ Ratio 116/114*	iTRAQ Ratio 117/114*	Rank	Result Type
1549.8658	1549.8235	-0.0423	-27	45	55	LDVPQNLMFGK	69	100	(N-term)_iTRAQ[0], Lysine(K)_iTRAQ[11]	[5] F4	268/260	1.058	1.177	1.341	1	Mascot
1832.0779	1832.0059	-0.072	-39	176	189	VLQPVDGEVSILFK	56	99.994	(N-term)_iTRAQ[0], Lysine(K)_iTRAQ[14]	[1] F3 030912	427/419	1.677	1.649	2.128	1	Mascot
1901.9491	1901.8312	-0.1179	-62	77	89	SVQYDDVPEYKDR	62	99.998	(N-term)_iTRAQ[0], Lysine(K)_iTRAQ[11]	[6] F8 110912	193/185	0.926	0.823	0.781	1	Mascot
1914.0371	1913.9943	-0.0428	-22	58	71	YEKPDGSPVFIAFR	41	99.826	(N-term)_iTRAQ[0], Lysine(K)_iTRAQ[3]	[4] F7 and F10+11	344/336	1.099	1.129	0.913	1	Mascot

297	RecName: Full=Acetyl-CoA acetyltransferase, mitochondrial; AltName: Full=Acetoacetyl-CoA thiolase;	gi|135757	49841.8	3	227	0.867	1.005	0.941	0.058	0.210	0.232	4	4	4	100

Protein Group

acetyl-CoA acetyltransferase, mitochondrial precursor [Rattus norvegicus]	gi|8392836	49855.8

Peptide Information

Calc. Mass	Obsrv. Mass	± da	± ppm	Start Seq.	End Seq.	Sequence	Ion Score	C. I. %	Modification	Plate [#] Name	Gel Idx/Pos [4700 Sample Name]	iTRAQ Ratio 115/114*	iTRAQ Ratio 116/114*	iTRAQ Ratio 117/114*	Rank	Result Type

Calc. Mass	Obsrv. Mass	± da	± ppm	Start Seq.	End Seq.	Sequence	Ion Score	C. I. %	Modification	Plate [#] Name	Gel Idx/Pos [4700 Sample Name]	iTRAQ Ratio 115/114*	iTRAQ Ratio 116/114*	iTRAQ Ratio 117/114*	Rank	Result Type
1388.8988	1388.8638	-0.035	-25	391	400	IVVHLAHALK	75	100	(N-term)_iTRAQ[0], Lysine(K)_iTRAQ[10]	[2] F12 040912	304/296	0.958	1.197	1.233	1	Mascot
1847.9385	1847.8262	-0.1123	-61	206	218	EEQDKYAIGSYTR	65	99.999	(N-term)_iTRAQ[0], Lysine(K)_iTRAQ[5]	[6] F8 110912	193/185	0.838	0.824	0.766	1	Mascot
1847.9385	1848.0151	0.0766	41	206	218	EEQDKYAIGSYTR	91	100	(N-term)_iTRAQ[0], Lysine(K)_iTRAQ[5]	[3] F6 and F9	1153/1145	0.806	0.839	1.108	1	Mascot
2211.2368	2211.1333	-0.1035	-47	241	254	GKPDVVVKEDEEYK	61	99.998	(N-term)_iTRAQ[0], Lysine(K)_iTRAQ[2,8, 14]	[2] F12 040912	193/185	0.875	1.233	0.748	1	Mascot

298	transcriptional activator protein Pur-beta [Rattus norvegicus]	gi|62945366	36083.4	3	224	0.900	1.004	0.916	0.192	0.209	0.246	4	4	4	100

Peptide Information

Calc. Mass	Obsrv. Mass	± da	± ppm	Start Seq.	End Seq.	Sequence	Ion Score	C. I. %	Modification	Plate [#] Name	Gel Idx/Pos [4700 Sample Name]	iTRAQ Ratio 115/114*	iTRAQ Ratio 116/114*	iTRAQ Ratio 117/114*	Rank	Result Type
1072.6289	1072.5894	-0.0395	-37	49	54	FYLDVK	31	97.839	(N-term)_iTRAQ[0], Lysine(K)_iTRAQ[6]	[7] F5 120912	243/235	0.949	0.789	0.819	1	Mascot
1889.9449	1889.8087	-0.1362	-72	24	40	GGGGPGGEQETQELAS K	121	100	(N-term)_iTRAQ[0], Lysine(K)_iTRAQ[17]	[5] F4	94/86	0.809	0.922	0.949	1	Mascot
1889.9449	1889.8628	-0.0821	-43	24	40	GGGGPGGEQETQELAS K	45	99.923	(N-term)_iTRAQ[0], Lysine(K)_iTRAQ[17]	[7] F5 120912	128/120	1.197	1.056	0.687	1	Mascot
3399.6213	3399.4949	-0.1264	-37	88	119	DSLGDFIEHYAQLGPSSP EQLAAGAEEGGGPR	73	100	(N-term)_iTRAQ[0]	[1] F3 030912	469/461	0.713	1.322	1.316	1	Mascot

299	2-oxoglutarate carrier protein	gi|1580888	36858.8	5	224	0.886	0.961	0.895	0.132	0.205	0.152	5	5	5	100

Peptide Information

Calc. Mass	Obsrv. Mass	± da	± ppm	Start Seq.	End Seq.	Sequence	Ion Score	C. I. %	Modification	Plate [#] Name	Gel Idx/Pos [4700 Sample Name]	iTRAQ Ratio 115/114*	iTRAQ Ratio 116/114*	iTRAQ Ratio 117/114*	Rank	Result Type
1090.6493	1090.5864	-0.0629	-58	163	170	NVFNALIR	27	95.423	(N-term)_iTRAQ[0]	[5] F4	242/234	0.902	1.241	1.012	1	Mascot
1230.6603	1230.5975	-0.0628	-51	174	182	EEGVPTLWR	29	96.669	(N-term)_iTRAQ[0]	[5] F4	199/191	1.049	0.909	0.907	1	Mascot
1364.8021	1364.7601	-0.042	-31	79	90	GIYTGLSAGLLR	41	99.801	(N-term)_iTRAQ[0]	[1] F3 030912	344/336	0.688	0.705	0.716	1	Mascot
1512.8468	1512.8959	0.0491	32	252	260	MIDGKPEYK	30	97.479	(N-term)_iTRAQ[0], Lysine(K)_iTRAQ[5,9]	[4] F7 and F10+11	1060/1052	0.947	0.930	1.102	1	Mascot
1924.075	1923.9724	-0.1026	-53	191	206	AVVVNAAQLASYSQSK	97	100	(N-term)_iTRAQ[0], Lysine(K)_iTRAQ[16]	[5] F4	201/193	0.885	1.110	0.793	1	Mascot

300	annexin A5 [Rattus norvegicus]	gi|6978505	38938.5	5	222	1.020	0.972	1.003	0.267	0.305	0.137	5	5	5	100

Protein Group

Annexin A5 [Rattus norvegicus]	gi|51858950	38966.6
Chain A, Annexin V Complex With Heparin Oligosaccharides	gi|14488466	38807.5
Chain A, Crystal Structure Of Annexin V R149e Mutant	gi|28373862	38911.5
Chain A, Crystal Structure Of Annexin V R23e Mutant	gi|28373863	38911.5
lipocortin V [Rattus norvegicus]	gi|2981437	36826.5

Peptide Information

Calc. Mass	Obsrv. Mass	± da	± ppm	Start Seq.	End Seq.	Sequence	Ion Score	C. I. %	Modification	Plate [#] Name	Gel Idx/Pos [4700 Sample Name]	iTRAQ Ratio 115/114*	iTRAQ Ratio 116/114*	iTRAQ Ratio 117/114*	Rank	Result Type
1280.7096	1280.692	-0.0176	-14	49	56	QQIAEEFK	42	99.826	(N-term)_iTRAQ[0], Lysine(K)_iTRAQ[8]	[7] F5 120912	163/155	0.908	0.827	0.920	1	Mascot
1302.7191	1302.6675	-0.0516	-40	88	95	LYDAYELK	28	96.365	(N-term)_iTRAQ[0], Lysine(K)_iTRAQ[8]	[7] F5 120912	236/228	1.150	0.861	0.939	1	Mascot
1831.9885	1831.9844	-0.0041	-2	28	43	GLGTDEDSILNLLTAR	50	99.975	(N-term)_iTRAQ[0]	[8] F13-15 and F1+2	312/304	1.262	1.639	1.151	1	Mascot
1835.0472	1835.097	0.0498	27	288	299	KNFATSLYSMIK	62	99.998	(N-term)_iTRAQ[0], Lysine(K)_iTRAQ[1,1 2]	[4] F7 and F10+11	1275/1267	0.682	0.977	1.184	1	Mascot
2022.0868	2022.0254	-0.0614	-30	244	258	SIPAYLAETLYYAMK	40	99.732	(N-term)_iTRAQ[0], Lysine(K)_iTRAQ[15]	[5] F4	428/420	1.227	0.763	0.860	1	Mascot

301	NADH dehydrogenase [ubiquinone] iron-sulfur protein 7, mitochondrial [Rattus norvegicus]	gi|56606108	25600.4	4	221	1.026	1.043	0.966	0.175	0.210	0.250	4	4	4	100

Peptide Information

Calc. Mass	Obsrv. Mass	± da	± ppm	Start Seq.	End Seq.	Sequence	Ion Score	C. I. %	Modification	Plate [#] Name	Gel Idx/Pos [4700 Sample Name]	iTRAQ Ratio	iTRAQ Ratio	iTRAQ Ratio	Rank	Result Type

Calc. Mass	Obsrv. Mass	± da	± ppm	Start Seq.	End Seq.	Sequence	Ion Score	C. I. %	Modification	Plate [#] Name	Gel Idx/Pos [4700 Sample Name]	iTRAQ Ratio 115/114*	iTRAQ Ratio 116/114*	iTRAQ Ratio 117/114*	Rank	Result Type
1259.6869	1259.6219	-0.065	-52	72	80	LDDLINWAR	34	99.072	(N-term)_iTRAQ[0]	[5] F4	306/298	1.026	1.111	1.164	1	Mascot
1278.6273	1278.5516	-0.0757	-59	142	150	VYDQMPEPR	30	97.565	(N-term)_iTRAQ[0]	[5] F4	121/113	1.227	1.249	0.658	1	Mascot
1550.8247	1550.8873	0.0626	40	141	150	KVYDQMPEPR	41	99.803	(N-term)_iTRAQ[0], Lysine(K)_iTRAQ[1]	[4] F7 and F10+11	1068/1060	1.100	1.112	1.143	1	Mascot
2189.1409	2189.0825	-0.0584	-27	28	47	AHQSVAATGSPSSTQSA VSK	116	100	(N-term)_iTRAQ[0], Lysine(K)_iTRAQ[20]	[4] F7 and F10+11	104/96	0.799	0.767	0.994	1	Mascot

302	hypoxia up-regulated protein 1 [Rattus norvegicus]	gi|77404375	122575	4	221	0.906	0.840	1.046	0.312	0.122	0.349	4	4	4	100

Protein Group

RecName: Full=Hypoxia up-regulated protein 1; AltName: Full=150 kDa oxygen-regulated protein; Short	gi|10720174	122644.1

Peptide Information

Calc. Mass	Obsrv. Mass	± da	± ppm	Start Seq.	End Seq.	Sequence	Ion Score	C. I. %	Modification	Plate [#] Name	Gel Idx/Pos [4700 Sample Name]	iTRAQ Ratio 115/114*	iTRAQ Ratio 116/114*	iTRAQ Ratio 117/114*	Rank	Result Type
1775.8085	1775.7791	-0.0294	-17	346	358	VEFEELCADLFDR	42	99.855	(N-term)_iTRAQ[0], MMTS (C)[7]	[1] F3 030912	570/562	1.039	0.967	1.481	1	Mascot
2152.1621	2152.1257	-0.0364	-17	670	686	GQAGPEGVPPAPEEEKK	61	99.998	(N-term)_iTRAQ[0], Lysine(K)_iTRAQ[16, 17]	[4] F7 and F10+11	145/137	0.556	0.765	0.669	1	Mascot
2239.2207	2239.2043	-0.0164	-7	199	217	VLQLINDNTATALSYGVF R	30	97.576	(N-term)_iTRAQ[0]	[8] F13-15 and F1+2	288/280	0.948	0.950	1.161	1	Mascot
2541.3079	2541.2432	-0.0647	-25	841	859	LIPEMDQIFTDVEMTTLEK	87	100	(N-term)_iTRAQ[0], Lysine(K)_iTRAQ[19]	[1] F3 030912	572/564	1.228	0.708	1.042	1	Mascot

303	inositol-trisphosphate 3-kinase A [Rattus norvegicus]	gi|13591973	53197.3	4	220	0.867	0.947	0.840	0.194	0.423	0.207	4	4	4	100

Protein Group

RecName: Full=Inositol-trisphosphate 3-kinase A; AltName: Full=Inositol 1,4,5-trisphosphate 3-kinas	gi|124808	54088.8

Peptide Information

Calc. Mass	Obsrv. Mass	± da	± ppm	Start Seq.	End Seq.	Sequence	Ion Score	C. I. %	Modification	Plate [#] Name	Gel Idx/Pos [4700 Sample Name]	iTRAQ Ratio 115/114*	iTRAQ Ratio 116/114*	iTRAQ Ratio 117/114*	Rank	Result Type
1386.7025	1386.6519	-0.0506	-36	371	380	DTLEISDFFR	31	97.974	(N-term)_iTRAQ[0]	[1] F3 030912	414/406	0.934	1.340	0.822	1	Mascot
1791.9333	1791.8387	-0.0946	-53	140	153	GNVQLETSEDVGQK	99	100	(N-term)_iTRAQ[0], Lysine(K)_iTRAQ[14]	[5] F4	107/99	0.615	0.870	0.999	1	Mascot
2066.9937	2066.8916	-0.1021	-49	281	297	MLAVDPEAPTEEEHAQR	58	99.996	(N-term)_iTRAQ[0]	[7] F5 120912	182/174	1.032	0.536	0.591	1	Mascot
2086.0889	2086.0994	0.0105	5	347	361	VFEEFMQGDAEVLKR	32	98.592	(N-term)_iTRAQ[0], Lysine(K)_iTRAQ[14]	[4] F7 and F10+11	410/402	0.953	1.288	1.026	1	Mascot

304	acyl-CoA synthetase isoform 6 variant2 [Rattus norvegicus]	gi|48256734	85587.6	5	220	0.866	0.855	0.798	0.403	0.387	0.724	6	6	6	100

Protein Group

RecName: Full=Long-chain-fatty-acid--CoA ligase 6; AltName: Full=Long-chain acyl-CoA synthetase 6;	gi|417242	85442.2
long-chain-fatty-acid--CoA ligase 6 [Rattus norvegicus]	gi|18543341	85420.2

Peptide Information

Calc. Mass	Obsrv. Mass	± da	± ppm	Start Seq.	End Seq.	Sequence	Ion Score	C. I. %	Modification	Plate [#] Name	Gel Idx/Pos [4700 Sample Name]	iTRAQ Ratio 115/114*	iTRAQ Ratio 116/114*	iTRAQ Ratio 117/114*	Rank	Result Type
1336.8073	1336.7802	-0.0271	-20	206	215	AILLLEHVER	47	99.952	(N-term)_iTRAQ[0]	[4] F7 and F10+11	347/339	0.414	0.580	0.253	1	Mascot
1565.8447	1565.8052	-0.0395	-25	12	23	LPELSDLGQFFR	53	99.987	(N-term)_iTRAQ[0]	[1] F3 030912	506/498	1.229	0.920	1.033	1	Mascot
1675.9213	1675.9092	-0.0121	-7	223	235	LVILMEPFDDALR	43	99.883	(N-term)_iTRAQ[0]	[8] F13-15 and F1+2	282/274	0.797	0.707	0.662	1	Mascot
1745.7997	1745.7006	-0.0991	-57	247	260	SMQAIEDSGQENHR	28	96.149	(N-term)_iTRAQ[0]	[6] F8 110912	146/138	0.785	0.840	0.842	1	Mascot
1745.7997	1745.7839	-0.0158	-9	247	260	SMQAIEDSGQENHR	42	99.832	(N-term)_iTRAQ[0]	[4] F7 and F10+11	145/137	1.302	1.837	2.255	1	Mascot
1855.9688	1855.8651	-0.1037	-56	491	503	LVDAEELNYWTSK	35	99.159	(N-term)_iTRAQ[0], Lysine(K)_iTRAQ[13]	[5] F4	290/282	1.018	0.669	0.787	1	Mascot

305	transforming protein RhoA precursor [Rattus norvegicus]	gi|16923986	24637.8	5	219	1.043	1.020	0.926	0.125	0.148	0.205	5	5	5	100

Protein Group

RhoA [Rattus norvegicus]	gi|2225894	21777

Peptide Information

Calc. Mass	Obsrv. Mass	± da	± ppm	Start Seq.	End Seq.	Sequence	Ion Score	C. I. %	Modification	Plate [#] Name	Gel Idx/Pos [4700 Sample Name]	iTRAQ Ratio 115/114*	iTRAQ Ratio 116/114*	iTRAQ Ratio 117/114*	Rank	Result Type
1047.6085	1047.5614	-0.0471	-45	99	104	WTPEVK	28	96.014	(N-term)_iTRAQ[0], Lysine(K)_iTRAQ[6]	[3] F6 and F9	201/193	1.007	0.966	0.841	1	Mascot
1126.5687	1126.5083	-0.0604	-54	169	176	EVFEMATR	31	97.998	(N-term)_iTRAQ[0]	[5] F4	155/147	1.015	1.077	0.799	1	Mascot
1860.0385	1860.0217	-0.0168	-9	134	145	MKQEPVKPEEGR	38	99.566	(N-term)_iTRAQ[0], Lysine(K)_iTRAQ[2,7]	[2] F12 040912	113/105	0.883	0.853	1.117	1	Mascot
1885.0419	1885.0616	0.0197	10	105	118	HFCPNVPIILVGNK	83	100	(N-term)_iTRAQ[0], Lysine(K)_iTRAQ[14], MMTS (C)[3]	[4] F7 and F10+11	411/403	1.248	1.280	1.233	1	Mascot
2153.0271	2153.0405	0.0134	6	52	68	QVELALWDTAGQEDYDR	40	99.753	(N-term)_iTRAQ[0]	[8] F13-15 and F1+2	417/409	1.098	0.971	0.737	1	Mascot

306 reticulon-1 [Rattus norvegicus] gi|16758732 88892.4 4 218 0.936 0.864 0.932 0.277 0.416 0.467 5 5 5 100

Peptide Information

Calc. Mass	Obsrv. Mass	± da	± ppm	Start Seq.	End Seq.	Sequence	Ion Score	C. I. %	Modification	Plate [#] Name	Gel Idx/Pos [4700 Sample Name]	iTRAQ Ratio 115/114*	iTRAQ Ratio 116/114*	iTRAQ Ratio 117/114*	Rank	Result Type
1193.6803	1193.6367	-0.0436	-37	590	597	AIDLLYWR	39	99.661	(N-term)_iTRAQ[0]	[5] F4	315/307	0.632	0.452	0.611	1	Mascot
1621.9159	1621.8337	-0.0822	-51	194	204	YIDITRPQEAK	29	97.018	(N-term)_iTRAQ[0], Lysine(K)_iTRAQ[11]	[6] F8 110912	217/209	0.925	0.769	0.688	1	Mascot
1659.8839	1659.7925	-0.0914	-55	116	127	EDSAYFTGILQK	62	99.998	(N-term)_iTRAQ[0], Lysine(K)_iTRAQ[12]	[5] F4	237/229	1.360	1.374	1.893	1	Mascot
1670.9098	1670.8424	-0.0674	-40	743	755	HQAQVDQYLGLVR	60	99.998	(N-term)_iTRAQ[0]	[6] F8 110912	301/293	1.098	1.225	1.104	1	Mascot
1670.9098	1670.9161	0.0063	4	743	755	HQAQVDQYLGLVR	88	100	(N-term)_iTRAQ[0]	[4] F7 and F10+11	300/292	0.822	0.822	0.801	1	Mascot

307 S-phase kinase-associated protein 1 [Rattus norvegicus] gi|56090475 21392.1 4 218 1.330 0.974 1.059 0.658 0.248 0.180 4 4 4 100

Peptide Information

Calc. Mass	Obsrv. Mass	± da	± ppm	Start Seq.	End Seq.	Sequence	Ion Score	C. I. %	Modification	Plate [#] Name	Gel Idx/Pos [4700 Sample Name]	iTRAQ Ratio 115/114*	iTRAQ Ratio 116/114*	iTRAQ Ratio 117/114*	Rank	Result Type
1399.6213	1399.6145	-0.0068	-5	156	163	ENQWCEEK	30	97.479	(N-term)_iTRAQ[0], Lysine(K)_iTRAQ[8], MMTS (C)[5]	[4] F7 and F10+11	215/207	2.656	1.379	1.362	1	Mascot
1610.7418	1610.6448	-0.097	-60	143	154	NDFTEEEEAQVR	84	100	(N-term)_iTRAQ[0]	[1] F3 030912	185/177	1.115	1.009	1.073	1	Mascot
1671.8187	1671.9019	0.0832	50	155	163	KENQWCEEK	46	99.938	(N-term)_iTRAQ[0], Lysine(K)_iTRAQ[1,9] , MMTS (C)[6]	[4] F7 and F10+11	1088/1080	1.077	0.746	0.930	1	Mascot
2050.0854	2050.0022	-0.0832	-41	81	94	RTDDIPVWDQEFLK	58	99.996	(N-term)_iTRAQ[0], Lysine(K)_iTRAQ[14]	[6] F8 110912	374/366	0.983	0.868	0.925	1	Mascot

308 cortactin isoform B [Rattus norvegicus] gi|2996046 63605.9 3 217 1.133 1.076 1.116 0.056 0.215 0.097 3 3 3 100

Protein Group

cortactin isoform B [Rattus norvegicus] gi|76563930 63818.1

cortactin isoform C [Rattus norvegicus] gi|2996044 58594.3

Peptide Information

Calc. Mass	Obsrv. Mass	± da	± ppm	Start Seq.	End Seq.	Sequence	Ion Score	C. I. %	Modification	Plate [#] Name	Gel Idx/Pos [4700 Sample Name]	iTRAQ Ratio 115/114*	iTRAQ Ratio 116/114*	iTRAQ Ratio 117/114*	Rank	Result Type
1373.7311	1373.7009	-0.0302	-22	209	218	SAVGFEYQGK	55	99.993	(N-term)_iTRAQ[0], Lysine(K)_iTRAQ[10]	[7] F5 120912	157/149	1.168	1.191	1.216	1	Mascot
1660.8905	1660.9661	0.0756	46	60	70	ENVFQEHQTLK	79	100	(N-term)_iTRAQ[0], Lysine(K)_iTRAQ[11]	[3] F6 and F9	1164/1156	1.176	0.832	1.148	1	Mascot
1715.885	1715.8053	-0.0797	-46	132	144	VDQSAVGFEYQGK	83	100	(N-term)_iTRAQ[0], Lysine(K)_iTRAQ[13]	[7] F5 120912	179/171	1.059	1.255	0.997	1	Mascot

309 connexin 43 gi|227943 47482.7 3 217 1.223 1.029 1.051 0.383 0.339 0.443 4 4 4 100

Protein Group

gap junction alpha-1 protein [Rattus norvegicus] gi|6978896 47452.6

Peptide Information

Calc. Mass	Obsrv. Mass	± da	± ppm	Start Seq.	End Seq.	Sequence	Ion Score	C. I. %	Modification	Plate [#] Name	Gel Idx/Pos [4700 Sample Name]	iTRAQ Ratio 115/114*	iTRAQ Ratio 116/114*	iTRAQ Ratio 117/114*	Rank	Result Type

Calc. Mass	Obsrv. Mass	± da	± ppm	Start Seq.	End Seq.	Sequence	Ion Score	C. I. %	Modification	Plate [#] Name	Gel Idx/Pos [4700 Sample Name]	iTRAQ Ratio 115/114*	iTRAQ Ratio 116/114*	iTRAQ Ratio 117/114*	Rank	Result Type
1828.9836	1828.9504	-0.0332	-18	115	128	VAQTDGVNVEMHLK	83	100	(N-term)_iTRAQ[0], Lysine(K)_iTRAQ[14]	[4] F7 and F10+11	243/235	1.536	1.060	1.012	1	Mascot
1845.9592	1845.9155	-0.0437	-24	244	258	SDPYHATTGPLSPSK	43	99.879	(N-term)_iTRAQ[0], Lysine(K)_iTRAQ[15]	[4] F7 and F10+11	177/169	0.952	0.878	1.011	1	Mascot
2039.9291	2039.7971	-0.132	-65	304	319	QASEQNWANYSAEQNR	91	100	(N-term)_iTRAQ[0]	[5] F4	121/113	1.671	1.607	1.791	1	Mascot
2039.9291	2039.8093	-0.1198	-59	304	319	QASEQNWANYSAEQNR	50	99.975	(N-term)_iTRAQ[0]	[1] F3 030912	191/183	0.915	0.751	0.667	1	Mascot

310	ras-related protein Rab-10 [Mus musculus]	gi|7710086	25880.8	4	217	0.979	1.108	0.871	0.413	0.304	0.600	4	4	4	100

Peptide Information

Calc. Mass	Obsrv. Mass	± da	± ppm	Start Seq.	End Seq.	Sequence	Ion Score	C. I. %	Modification	Plate [#] Name	Gel Idx/Pos [4700 Sample Name]	iTRAQ Ratio 115/114*	iTRAQ Ratio 116/114*	iTRAQ Ratio 117/114*	Rank	Result Type
1359.8457	1359.778	-0.0677	-50	12	22	LLLIGDSGVGK	58	99.996	(N-term)_iTRAQ[0], Lysine(K)_iTRAQ[11]	[5] F4	229/221	0.740	1.446	1.508	1	Mascot
1405.8175	1405.791	-0.0265	-19	162	172	AFLTLAEDILR	59	99.997	(N-term)_iTRAQ[0]	[1] F3 030912	546/538	1.770	1.270	1.096	1	Mascot
1460.7618	1460.6924	-0.0694	-48	60	70	LQIWDTAGQER	70	100	(N-term)_iTRAQ[0]	[5] F4	197/189	0.762	1.084	0.943	1	Mascot
2221.0981	2220.9583	-0.1398	-63	178	196	EPNSENVDISSGGGVTGWK	30	97.794	(N-term)_iTRAQ[0], Lysine(K)_iTRAQ[19]	[5] F4	172/164	0.920	0.757	0.369	1	Mascot

311	calcium-binding protein P23K beta [Rattus norvegicus]	gi|2160456	18524.3	4	217	1.030	1.040	1.224	0.150	0.135	0.284	4	4	4	100

Protein Group

hippocalcin-like protein 4 [Rattus norvegicus] gi|8393861 24962.8

Peptide Information

Calc. Mass	Obsrv. Mass	± da	± ppm	Start Seq.	End Seq.	Sequence	Ion Score	C. I. %	Modification	Plate [#] Name	Gel Idx/Pos [4700 Sample Name]	iTRAQ Ratio 115/114*	iTRAQ Ratio 116/114*	iTRAQ Ratio 117/114*	Rank	Result Type
1319.6881	1319.6313	-0.0568	-43	55	63	FFPYGDASK	59	99.997	(N-term)_iTRAQ[0], Lysine(K)_iTRAQ[9]	[7] F5 120912	218/210	0.983	0.991	1.220	1	Mascot
1773.9017	1773.943	0.0413	23	71	83	TFDKNGDGTIDFR	53	99.988	(N-term)_iTRAQ[0], Lysine(K)_iTRAQ[4]	[3] F6 and F9	1187/1179	1.195	0.882	1.719	1	Mascot
2444.2432	2444.1582	-0.085	-35	37	54	DCPSGILNLEEFQQLYIK	48	99.958	(N-term)_iTRAQ[0], Lysine(K)_iTRAQ[18], MMTS (C)[2]	[1] F3 030912	515/507	0.843	1.232	1.004	1	Mascot
2749.4028	2749.3167	-0.0861	-31	8	28	LAPEELEDLVQNTEFSEQELK	57	99.995	(N-term)_iTRAQ[0], Lysine(K)_iTRAQ[21]	[1] F3 030912	485/477	1.136	1.084	1.067	1	Mascot

312	peroxiredoxin-1 [Rattus norvegicus]	gi|16923958	25161.3	4	217	1.196	0.972	0.886	0.193	0.083	0.240	5	5	5	100

Protein Group

Chain A, Crystal Structure Of A Mammalian 2-Cys Peroxiredoxin, Hbp23. gi|6435547 25099.3

Peptide Information

Calc. Mass	Obsrv. Mass	± da	± ppm	Start Seq.	End Seq.	Sequence	Ion Score	C. I. %	Modification	Plate [#] Name	Gel Idx/Pos [4700 Sample Name]	iTRAQ Ratio 115/114*	iTRAQ Ratio 116/114*	iTRAQ Ratio 117/114*	Rank	Result Type
1241.7252	1241.765	0.0398	32	8	16	IGHPAPSFK	38	99.632	(N-term)_iTRAQ[0], Lysine(K)_iTRAQ[9]	[4] F7 and F10+11	1066/1058	1.002	1.133	0.562	1	Mascot
1395.8093	1395.7238	-0.0855	-61	111	120	TIAQDYGVLK	57	99.995	(N-term)_iTRAQ[0], Lysine(K)_iTRAQ[10]	[5] F4	174/166	1.017	0.967	1.146	1	Mascot
1395.8093	1395.7372	-0.0721	-52	111	120	TIAQDYGVLK	33	98.871	(N-term)_iTRAQ[0], Lysine(K)_iTRAQ[10]	[7] F5 120912	217/209	1.307	0.932	0.965	1	Mascot
1452.7766	1452.7031	-0.0735	-51	17	27	ATAVMPDGQFK	52	99.983	(N-term)_iTRAQ[0], Lysine(K)_iTRAQ[11]	[7] F5 120912	174/166	1.478	0.959	0.937	1	Mascot
1484.8359	1484.8063	-0.0296	-20	159	168	LVQAFQFTDK	70	100	(N-term)_iTRAQ[0], Lysine(K)_iTRAQ[10]	[7] F5 120912	303/295	1.243	0.888	0.935	1	Mascot

313	ras-related protein Rab-2A [Rattus norvegicus]	gi|13929006	25532	3	215	0.873	1.098	0.878	0.102	0.082	0.114	3	3	3	100

Peptide Information

Calc. Mass	Obsrv. Mass	± da	± ppm	Start Seq.	End Seq.	Sequence	Ion Score	C. I. %	Modification	Plate [#] Name	Gel Idx/Pos [4700 Sample Name]	iTRAQ Ratio 115/114*	iTRAQ Ratio 116/114*	iTRAQ Ratio 117/114*	Rank	Result Type
1694.8622	1694.8073	-0.0549	-32	57	69	LQIWDTAGQESFR	55	99.993	(N-term)_iTRAQ[0]	[1] F3 030912	335/327	0.906	1.191	0.863	1	Mascot
1782.9484	1782.882	-0.0664	-37	152	165	TASNVEEAFINTAK	62	99.998	(N-term)_iTRAQ[0], Lysine(K)_iTRAQ[14]	[5] F4	212/204	0.751	0.999	0.764	1	Mascot
1862.9158	1862.9288	0.013	7	92	105	DTFNHLTTWLEDAR	98	100	(N-term)_iTRAQ[0]	[4] F7 and F10+11	422/414	0.977	1.112	1.028	1	Mascot

314	1-phosphatidylinositol-4,5-bisphosphate phosphodiesterase beta-1 [Rattus norvegicus]	gi|117647200	156815.7	4	213	0.803	0.961	0.946	0.535	0.164	0.419	4	4	4	100

Protein Group

1-phosphatidylinositol-4,5-bisphosphate gi|1083573 150927.6

phosphodiesterase (EC 3.1.4.11) beta-1b - rat

Peptide Information

Calc. Mass	Obsrv. Mass	± da	± ppm	Start Seq.	End Seq.	Sequence	Ion Score	C. I. %	Modification	Plate [#] Name	Gel Idx/Pos [4700 Sample Name]	iTRAQ Ratio 115/114*	iTRAQ Ratio 116/114*	iTRAQ Ratio 117/114*	Rank	Result Type
1499.7992	1499.73	-0.0692	-46	582	591	SPVEFVEYNK	32	98.543	(N-term)_iTRAQ[0], Lysine(K)_iTRAQ[10]	[7] F5 120912	230/222	1.707	1.134	1.690	1	Mascot
1657.941	1657.8578	-0.0832	-50	1101	1111	SYIQEVVQYIK	57	99.995	(N-term)_iTRAQ[0], Lysine(K)_iTRAQ[11]	[5] F4	338/330	0.460	1.097	0.944	1	Mascot
1736.9415	1736.9238	-0.0177	-10	88	101	ELLDVGNIGHLEQR	64	99.999	(N-term)_iTRAQ[0]	[3] F6 and F9	324/316	0.557	0.772	0.621	1	Mascot
2468.2363	2468.1655	-0.0708	-29	129	148	EWTNEVFSLATNLLAQN MSR	60	99.998	(N-term)_iTRAQ[0]	[1] F3 030912	576/568	0.951	0.888	0.808	1	Mascot

315	FK506-binding protein 12 [Rattus norvegicus]	gi|1843430	13262	3	212	0.975	1.076	0.929	0.386	0.116	0.125	3	3	3	100

Protein Group

peptidyl-prolyl cis-trans isomerase FKBP1A [Rattus norvegicus]	gi|47271544	13258

Peptide Information

Calc. Mass	Obsrv. Mass	± da	± ppm	Start Seq.	End Seq.	Sequence	Ion Score	C. I. %	Modification	Plate [#] Name	Gel Idx/Pos [4700 Sample Name]	iTRAQ Ratio 115/114*	iTRAQ Ratio 116/114*	iTRAQ Ratio 117/114*	Rank	Result Type
1448.7465	1448.6671	-0.0794	-55	2	14	GVQVETISSGDGR	67	100	(N-term)_iTRAQ[0]	[1] F3 030912	164/156	0.622	0.942	0.801	1	Mascot
1677.8138	1677.7661	-0.0477	-28	59	72	GWEEGVAQMSVGQR	96	100	(N-term)_iTRAQ[0]	[1] F3 030912	269/261	1.081	1.092	1.091	1	Mascot
2344.1816	2344.2515	0.0699	30	20	36	GQTCVVHYTGMLEDGKK	49	99.972	(N-term)_iTRAQ[0], Lysine(K)_iTRAQ[16, 17], MMTS (C)[4]	[4] F7 and F10+11	1176/1168	1.379	1.210	0.919	1	Mascot

316	RNA binding protein p37 AUF1 [Rattus norvegicus]	gi|9588102	35344.4	4	212	0.714	1.012	1.286	0.302	0.266	0.478	6	6	6	100

Protein Group

RNA binding protein p40 AUF1 [Rattus norvegicus]	gi|9588100	37519.4
RNA binding protein p42 AUF1 [Rattus norvegicus]	gi|9588098	40886.6
RecName: Full=Heterogeneous nuclear ribonucleoprotein D0; Short=hnRNP D0; AltName: Full=AU-rich ele	gi|13124487	43061.5

Peptide Information

Calc. Mass	Obsrv. Mass	± da	± ppm	Start Seq.	End Seq.	Sequence	Ion Score	C. I. %	Modification	Plate [#] Name	Gel Idx/Pos [4700 Sample Name]	iTRAQ Ratio 115/114*	iTRAQ Ratio 116/114*	iTRAQ Ratio 117/114*	Rank	Result Type
1202.7183	1202.718	-0.0003	0	118	125	GFGFVLFK	33	98.856	(N-term)_iTRAQ[0], Lysine(K)_iTRAQ[8]	[7] F5 120912	405/397	0.532	0.825	1.179	1	Mascot
1643.8713	1643.7803	-0.091	-55	78	89	MFIGGLSWDTTK	34	98.934	(N-term)_iTRAQ[0], Lysine(K)_iTRAQ[12]	[5] F4	292/284	0.381	0.699	0.740	1	Mascot
1643.8713	1643.8678	-0.0035	-2	78	89	MFIGGLSWDTTK	66	99.999	(N-term)_iTRAQ[0], Lysine(K)_iTRAQ[12]	[7] F5 120912	363/355	0.779	1.031	2.018	1	Mascot
1776.9629	1776.8513	-0.1116	-63	163	176	IFVGGLSPDTPEEK	32	98.522	(N-term)_iTRAQ[0], Lysine(K)_iTRAQ[14]	[1] F3 030912	281/273	0.843	1.332	1.141	1	Mascot
1776.9629	1776.8881	-0.0748	-42	163	176	IFVGGLSPDTPEEK	74	100	(N-term)_iTRAQ[0], Lysine(K)_iTRAQ[14]	[5] F4	207/199	0.962	1.325	1.670	1	Mascot
1916.0687	1916.1554	0.0867	45	78	90	MFIGGLSWDTTKK	39	99.697	(N-term)_iTRAQ[0], Lysine(K)_iTRAQ[12, 13]	[4] F7 and F10+11	1239/1231	1.039	1.023	1.348	1	Mascot

317	RecName: Full=Keratin, type II cytoskeletal 1; AltName: Full=Cytokeratin-1; Short=CK-1; AltName: Fu	gi|81891716	69580.2	4	212	0.782	0.760	2.015	0.160	0.064	0.195	4	4	4	100

Peptide Information

Calc. Mass	Obsrv. Mass	± da	± ppm	Start Seq.	End Seq.	Sequence	Ion Score	C. I. %	Modification	Plate [#] Name	Gel Idx/Pos [4700 Sample Name]	iTRAQ Ratio 115/114*	iTRAQ Ratio 116/114*	iTRAQ Ratio 117/114*	Rank	Result Type
1429.7244	1429.6526	-0.0718	-50	463	471	DYQELMNTK	57	99.995	(N-term)_iTRAQ[0], Lysine(K)_iTRAQ[9]	[7] F5 120912	180/172	0.673	0.676	2.331	1	Mascot
1553.842	1553.783	-0.059	-38	277	287	TNAENEFVTIK	65	99.999	(N-term)_iTRAQ[0], Lysine(K)_iTRAQ[11]	[7] F5 120912	196/188	0.680	0.800	1.877	1	Mascot
1619.8513	1619.8011	-0.0502	-31	211	222	WELLQQVDTSTR	33	98.829	(N-term)_iTRAQ[0]	[1] F3 030912	337/329	1.067	0.738	2.042	1	Mascot
1763.9901	1763.8978	-0.0923	-52	199	210	FLEQQNQVLQTK	57	99.995	(N-term)_iTRAQ[0], Lysine(K)_iTRAQ[12]	[5] F4	170/162	0.767	0.837	1.847	1	Mascot

318	C2 domain-containing protein 2-like [Rattus norvegicus]	gi|58865568	80705	3	212	1.045	1.002	0.975	0.272	0.415	0.295	4	4	4	100

Peptide Information

Calc. Mass	Obsrv. Mass	± da	± ppm	Start Seq.	End Seq.	Sequence	Ion Score	C. I. %	Modification	Plate [#] Name	Gel Idx/Pos [4700 Sample Name]	iTRAQ Ratio 115/114*	iTRAQ Ratio 116/114*	iTRAQ Ratio 117/114*	Rank	Result Type
1354.8344	1354.7899	-0.0445	-33	87	96	GLLASLFAFK	75	100	(N-term)_iTRAQ[0], Lysine(K)_iTRAQ[10]	[5] F4	407/399	0.844	1.009	0.765	1	Mascot
1354.8344	1354.8105	-0.0239	-18	87	96	GLLASLFAFK	54	99.991	(N-term)_iTRAQ[0], Lysine(K)_iTRAQ[10]	[7] F5 120912	502/494	1.505	1.680	0.950	1	Mascot
2330.1345	2330.0422	-0.0923	-40	656	676	EAGLSQSHDDLSNTTATPSVR	94	100	(N-term)_iTRAQ[0]	[7] F5 120912	152/144	1.080	0.635	1.509	1	Mascot
2332.2129	2332.1973	-0.0156	-7	227	243	ERDEEQPELSTVEELIK	43	99.871	(N-term)_iTRAQ[0], Lysine(K)_iTRAQ[17]	[4] F7 and F10+11	380/372	0.869	0.936	0.823	1	Mascot

319	Ser/Thr protein kinase	gi|739718	67803.5	3	211	1.046	0.989	1.125	0.501	0.331	0.369	3	3	3	100

Protein Group

serine/threonine-protein kinase PAK 1 [Rattus norvegicus]	gi|8393901	67686.6

Peptide Information

Calc. Mass	Obsrv. Mass	± da	± ppm	Start Seq.	End Seq.	Sequence	Ion Score	C. I. %	Modification	Plate [#] Name	Gel Idx/Pos [4700 Sample Name]	iTRAQ Ratio 115/114*	iTRAQ Ratio 116/114*	iTRAQ Ratio 117/114*	Rank	Result Type
1443.8569	1443.9071	0.0502	35	513	521	ELLQHQFLK	56	99.994	(N-term)_iTRAQ[0], Lysine(K)_iTRAQ[9]	[3] F6 and F9	1220/1212	0.633	0.810	0.871	1	Mascot
1829.9127	1829.8418	-0.0709	-39	149	162	SAEDYNSSNTLNVK	85	100	(N-term)_iTRAQ[0], Lysine(K)_iTRAQ[14]	[7] F5 120912	144/136	1.643	1.488	1.671	1	Mascot
2297.2876	2297.1917	-0.0959	-42	119	134	KNPQAVLDVLEFYNSK	71	100	(N-term)_iTRAQ[0], Lysine(K)_iTRAQ[1,16]	[6] F8 110912	439/431	1.101	0.802	0.977	1	Mascot

320	peptidyl-prolyl cis-trans isomerase D [Rattus norvegicus]	gi|51948528	46969.9	5	211	0.994	1.088	0.678	0.328	0.461	0.317	5	5	5	100

Peptide Information

Calc. Mass	Obsrv. Mass	± da	± ppm	Start Seq.	End Seq.	Sequence	Ion Score	C. I. %	Modification	Plate [#] Name	Gel Idx/Pos [4700 Sample Name]	iTRAQ Ratio 115/114*	iTRAQ Ratio 116/114*	iTRAQ Ratio 117/114*	Rank	Result Type
1175.6782	1175.6661	-0.0121	-10	314	321	AQGWQGLK	39	99.715	(N-term)_iTRAQ[0], Lysine(K)_iTRAQ[8]	[4] F7 and F10+11	201/193	0.972	1.254	1.078	1	Mascot
1394.7347	1394.6888	-0.0459	-33	236	244	SQNWEMAIK	54	99.989	(N-term)_iTRAQ[0], Lysine(K)_iTRAQ[9]	[3] F6 and F9	252/244	1.086	0.922	1.075	1	Mascot
1453.7784	1453.7108	-0.0676	-46	322	331	EYDQALADLK	40	99.74	(N-term)_iTRAQ[0], Lysine(K)_iTRAQ[10]	[7] F5 120912	243/235	1.033	1.327	0.549	1	Mascot
1465.7573	1465.7157	-0.0416	-28	186	195	EGDEWGIFPK	37	99.491	(N-term)_iTRAQ[0], Lysine(K)_iTRAQ[10]	[7] F5 120912	287/279	1.467	1.661	0.503	1	Mascot
1516.7318	1516.811	0.0792	52	103	111	FEDENFHYK	42	99.836	(N-term)_iTRAQ[0], Lysine(K)_iTRAQ[9]	[4] F7 and F10+11	1108/1100	0.606	0.599	0.448	1	Mascot

321	paralemmin-1 [Rattus norvegicus]	gi|18677765	45785.6	3	211	0.801	0.898	0.929	0.379	0.109	0.092	3	3	3	100

Peptide Information

Calc. Mass	Obsrv. Mass	± da	± ppm	Start Seq.	End Seq.	Sequence	Ion Score	C. I. %	Modification	Plate [#] Name	Gel Idx/Pos [4700 Sample Name]	iTRAQ Ratio 115/114*	iTRAQ Ratio 116/114*	iTRAQ Ratio 117/114*	Rank	Result Type
1961.0477	1960.9824	-0.0653	-33	94	109	EIDVLEFGESAPAAPK	84	100	(N-term)_iTRAQ[0], Lysine(K)_iTRAQ[16]	[1] F3 030912	334/326	0.985	0.967	1.000	1	Mascot
2031.9955	2032.0303	0.0348	17	238	256	ADEVTLSEAGSTTGPAEPR	39	99.704	(N-term)_iTRAQ[0]	[8] F13-15 and F1+2	575/567	1.122	0.763	0.987	1	Mascot
2496.1658	2496.0166	-0.1492	-60	354	373	EENQTGPTTTPSDTQDLDMK	89	100	(N-term)_iTRAQ[0], Lysine(K)_iTRAQ[20]	[1] F3 030912	189/181	0.465	0.981	0.813	1	Mascot

322	cytochrome c oxidase subunit 5A, mitochondrial precursor [Rattus norvegicus]	gi|24233541	17456.1	5	211	0.861	0.833	0.995	0.183	0.139	0.150	6	6	6	100

Peptide Information

Calc. Mass	Obsrv. Mass	± da	± ppm	Start Seq.	End Seq.	Sequence	Ion Score	C. I. %	Modification	Plate [#] Name	Gel Idx/Pos [4700 Sample Name]	iTRAQ Ratio 115/114*	iTRAQ Ratio 116/114*	iTRAQ Ratio 117/114*	Rank	Result Type
915.5747	915.5259	-0.0488	-53	84	90	IIDAALR	29	96.976	(N-term)_iTRAQ[0]	[5] F4	160/152	0.784	1.037	0.932	1	Mascot
988.6652	988.6498	-0.0154	-16	104	109	ILEVVK	29	96.877	(N-term)_iTRAQ[0], Lysine(K)_iTRAQ[6]	[7] F5 120912	206/198	0.847	0.783	0.831	1	Mascot
1292.7195	1292.7903	0.0708	55	94	103	RLNDFASAVR	30	97.536	(N-term)_iTRAQ[0]	[4] F7 and F10+11	1109/1101	0.671	0.793	0.855	1	Mascot
1375.8895	1375.9547	0.0652	47	104	111	ILEVVKDK	50	99.976	(N-term)_iTRAQ[0], Lysine(K)_iTRAQ[6,8]	[4] F7 and F10+11	1144/1136	0.745	0.779	1.101	1	Mascot
2341.2227	2341.2288	0.0061	3	52	67	WVTYFNKPDIDAWELR	73	100	(N-term)_iTRAQ[0], Lysine(K)_iTRAQ[7]	[4] F7 and F10+11	456/448	1.061	1.001	1.098	1	Mascot
2341.2227	2341.4243	0.2016	86	52	67	WVTYFNKPDIDAWELR	43	99.89	(N-term)_iTRAQ[0], Lysine(K)_iTRAQ[7]	[4] F7 and F10+11	458/450	1.157	0.666	1.211	1	Mascot

323 SAP90A [Rattus norvegicus] gi|297480 86270.8 5 209 0.810 0.817 0.880 0.258 0.161 0.291 5 5 5 100

Protein Group

disks large homolog 4 [Rattus norvegicus] gi|9665227 86311.7

Peptide Information

Calc. Mass	Obsrv. Mass	± da	± ppm	Start Seq.	End Seq.	Sequence	Ion Score	C. I. %	Modification	Plate [#] Name	Gel Idx/Pos [4700 Sample Name]	iTRAQ Ratio 115/114*	iTRAQ Ratio 116/114*	iTRAQ Ratio 117/114*	Rank	Result Type
1533.7295	1533.6887	-0.0408	-27	444	455	DCGFLSQALSFR	54	99.99	(N-term)_iTRAQ[0], MMTS (C)[2]	[1] F3 030912	478/470	0.863	0.785	0.621	1	Mascot
1602.9425	1603.0151	0.0726	45	655	665	SLENVLEINKR	61	99.998	(N-term)_iTRAQ[0], Lysine(K)_iTRAQ[10]	[3] F6 and F9	1237/1229	0.643	0.730	0.828	1	Mascot
1682.8833	1682.8419	-0.0414	-25	300	312	DLLGEEDIPREPR	36	99.326	(N-term)_iTRAQ[0]	[3] F6 and F9	256/248	0.766	0.809	0.791	1	Mascot
1794.9139	1794.9359	0.022	12	409	424	EQLMNSSLGSGTASLR	33	98.701	(N-term)_iTRAQ[0]	[8] F13-15 and F1+2	573/565	1.330	1.148	1.480	1	Mascot
1859.0187	1859.0461	0.0274	15	708	722	VIEDLSGPYIWVPAR	28	95.958	(N-term)_iTRAQ[0]	[8] F13-15 and F1+2	386/378	0.616	0.685	0.879	1	Mascot

324 bifunctional purine biosynthesis protein PURH [Rattus norvegicus] gi|48675845 69625.6 4 209 1.130 0.926 0.994 0.393 0.252 0.260 4 4 4 100

Protein Group

5-aminoimidazole-4-carboxamide ribonucleotide formyltransferase/IMP cyclohydrolase [Rattus norvegic gi|2541906 69649.6

Peptide Information

Calc. Mass	Obsrv. Mass	± da	± ppm	Start Seq.	End Seq.	Sequence	Ion Score	C. I. %	Modification	Plate [#] Name	Gel Idx/Pos [4700 Sample Name]	iTRAQ Ratio 115/114*	iTRAQ Ratio 116/114*	iTRAQ Ratio 117/114*	Rank	Result Type
1597.7581	1597.6807	-0.0774	-48	427	437	YTQSNSVCYAK	48	99.961	(N-term)_iTRAQ[0], Lysine(K)_iTRAQ[11], MMTS (C)[8]	[7] F5 120912	167/159	1.172	0.762	1.341	1	Mascot
2006.0944	2006.0305	-0.0639	-32	510	524	ALFEEVPELLTEAEK	39	99.714	(N-term)_iTRAQ[0], Lysine(K)_iTRAQ[15]	[1] F3 030912	501/493	0.908	0.873	1.126	1	Mascot
2094.0852	2093.9961	-0.0891	-43	336	352	EVSDGIVAPGYEEEALK	74	100	(N-term)_iTRAQ[0], Lysine(K)_iTRAQ[17]	[1] F3 030912	275/267	0.846	0.794	0.725	1	Mascot
2278.2917	2278.3157	0.024	11	510	525	ALFEEVPELLTEAEKK	50	99.972	(N-term)_iTRAQ[0], Lysine(K)_iTRAQ[15, 16]	[4] F7 and F10+11	486/478	1.813	1.392	0.893	1	Mascot

325 PREDICTED: histone cluster 1, H2bp [Rattus norvegicus] gi|27686409 16927.7 3 208 1.147 1.210 1.009 0.029 0.125 0.071 3 3 3 100

Protein Group

histone H2B type 1 [Rattus norvegicus] gi|12025526 16863.7

Peptide Information

Calc. Mass	Obsrv. Mass	± da	± ppm	Start Seq.	End Seq.	Sequence	Ion Score	C. I. %	Modification	Plate [#] Name	Gel Idx/Pos [4700 Sample Name]	iTRAQ Ratio 115/114*	iTRAQ Ratio 116/114*	iTRAQ Ratio 117/114*	Rank	Result Type
1425.7511	1425.6967	-0.0544	-38	36	44	ESYSVYVYK	54	99.989	(N-term)_iTRAQ[0], Lysine(K)_iTRAQ[9]	[7] F5 120912	196/188	1.114	1.097	1.003	1	Mascot
1697.9485	1698.022	0.0735	43	35	44	KESYSVYVYK	66	99.999	(N-term)_iTRAQ[0], Lysine(K)_iTRAQ[1,10]	[4] F7 and F10+11	1111/1103	1.185	1.384	1.100	1	Mascot
1887.9218	1887.8857	-0.0361	-19	59	73	AMGIMNSFVNDIFER	88	100	(N-term)_iTRAQ[0]	[1] F3 030912	536/528	1.142	1.168	0.932	1	Mascot

326 histone H2B gi|223096 16792.2 3 208 1.147 1.210 1.009 0.029 0.125 0.071 3 3 3 100

Peptide Information

Calc. Mass	Obsrv. Mass	± da	± ppm	Start Seq.	End Seq.	Sequence	Ion Score	C. I. %	Modification	Plate [#] Name	Gel Idx/Pos [4700 Sample Name]	iTRAQ Ratio 115/114*	iTRAQ Ratio 116/114*	iTRAQ Ratio 117/114*	Rank	Result Type
1425.7511	1425.6967	-0.0544	-38	35	43	ESYSVYVYK	54	99.989	(N-term)_iTRAQ[0], Lysine(K)_iTRAQ[9]	[7] F5 120912	196/188	1.114	1.097	1.003	1	Mascot
1697.9485	1698.022	0.0735	43	34	43	KESYSVYVYK	66	99.999	(N-term)_iTRAQ[0], Lysine(K)_iTRAQ[1,10]	[4] F7 and F10+11	1111/1103	1.185	1.384	1.100	1	Mascot
1887.9218	1887.8857	-0.0361	-19	58	72	AMGIMNSFVNDIFZR	88	100	(N-term)_iTRAQ[0]	[1] F3 030912	536/528	1.142	1.168	0.932	1	Mascot

327 glutathione S-transferase Mu 5 [Rattus norvegicus] gi|25282395 29861 5 207 1.261 1.334 1.192 0.501 0.623 0.290 5 5 5 100

Peptide Information

Calc. Mass	Obsrv. Mass	± da	± ppm	Start Seq.	End Seq.	Sequence	Ion Score	C. I. %	Modification	Plate [#] Name	Gel Idx/Pos [4700 Sample Name]	iTRAQ Ratio 115/114*	iTRAQ Ratio 116/114*	iTRAQ Ratio 117/114*	Rank	Result Type

Calc. Mass	Obsrv. Mass	± da	± ppm	Start Seq.	End Seq.	Sequence	Ion Score	C. I. %	Modification	Plate [#] Name	Gel Idx/Pos [4700 Sample Name]	iTRAQ Ratio 115/114*	iTRAQ Ratio 116/114*	iTRAQ Ratio 117/114*	Rank	Result Type
1383.7214	1383.6536	-0.0678	-49	6	15	SMVLGYWDIR	35	99.194	(N-term)_iTRAQ[0]	[5] F4	305/297	1.249	0.968	1.079	1	Mascot
1607.6571	1607.5782	-0.0789	-49	36	47	QYTCGEAPDYDR	54	99.99	(N-term)_iTRAQ[0], MMTS (C)[4]	[1] F3 030912	205/197	0.936	1.003	1.013	1	Mascot
1654.8053	1654.7612	-0.0441	-27	101	112	VDIMENQIMDFR	33	98.889	(N-term)_iTRAQ[0]	[1] F3 030912	383/375	2.333	2.471	1.836	1	Mascot
1893.9402	1893.8645	-0.0757	-40	23	35	MLLEFTDTSYEEK	52	99.986	(N-term)_iTRAQ[0], Lysine(K)_iTRAQ[13]	[5] F4	276/268	1.256	0.990	1.087	1	Mascot
2103.0884	2103.0479	-0.0405	-19	157	172	LTFVDFLTYDVLDQNR	33	98.745	(N-term)_iTRAQ[0]	[1] F3 030912	558/550	0.932	1.776	1.101	1	Mascot

328	reticulon-3 isoform A [Rattus norvegicus]	gi|57977297	111712.9	4	206	0.722	0.948	0.929	0.176	0.291	0.225	5	5	5	100

Peptide Information

Calc. Mass	Obsrv. Mass	± da	± ppm	Start Seq.	End Seq.	Sequence	Ion Score	C. I. %	Modification	Plate [#] Name	Gel Idx/Pos [4700 Sample Name]	iTRAQ Ratio 115/114*	iTRAQ Ratio 116/114*	iTRAQ Ratio 117/114*	Rank	Result Type
1416.772	1416.7031	-0.0689	-49	907	917	TQIDHYVGIAR	45	99.918	(N-term)_iTRAQ[0]	[6] F8 110912	217/209	0.673	1.041	0.927	1	Mascot
1416.772	1416.762	-0.01	-7	907	917	TQIDHYVGIAR	65	99.999	(N-term)_iTRAQ[0]	[4] F7 and F10+11	215/207	0.693	0.611	0.703	1	Mascot
1661.8831	1661.7903	-0.0928	-56	651	663	VSSDLEQEQLTIR	29	96.819	(N-term)_iTRAQ[0]	[1] F3 030912	247/239	0.831	0.877	0.847	1	Mascot
2215.1619	2215.0576	-0.1043	-47	366	381	STGDWTETFTEGKPVK	31	98.252	(N-term)_iTRAQ[0], Lysine(K)_iTRAQ[13, 16]	[6] F8 110912	271/263	0.984	1.396	1.366	1	Mascot
2508.1182	2508.0801	-0.0381	-15	488	507	SEMYENSEQQQAHAETP TQR	80	100	(N-term)_iTRAQ[0]	[7] F5 120912	111/103	0.514	0.986	0.918	1	Mascot

329	alpha-endosulfine isoform b [Mus musculus]	gi|71061466	15651.5	3	205	1.309	1.024	1.013	0.373	0.197	0.135	3	3	3	100

Protein Group

alpha-endosulfine isoform a [Mus musculus] gi|9624979 16064.7

Peptide Information

Calc. Mass	Obsrv. Mass	± da	± ppm	Start Seq.	End Seq.	Sequence	Ion Score	C. I. %	Modification	Plate [#] Name	Gel Idx/Pos [4700 Sample Name]	iTRAQ Ratio 115/114*	iTRAQ Ratio 116/114*	iTRAQ Ratio 117/114*	Rank	Result Type
1598.7406	1598.7157	-0.0249	-16	64	74	YFDSGDYNMAK	81	100	(N-term)_iTRAQ[0], Lysine(K)_iTRAQ[11]	[7] F5 120912	211/203	1.002	0.798	0.921	1	Mascot
1746.0021	1746.078	0.0759	43	25	36	EGILPEKAEEAK	57	99.996	(N-term)_iTRAQ[0], Lysine(K)_iTRAQ[7,1 2]	[3] F6 and F9	1164/1156	1.222	1.154	0.934	1	Mascot
2045.09	2045.015	-0.075	-37	90	106	NLVTGDHIPTPQDLPQR	67	100	(N-term)_iTRAQ[0]	[7] F5 120912	236/228	1.831	1.165	1.209	1	Mascot

330	Peroxiredoxin 2 [Rattus norvegicus]	gi|34849738	23939.5	4	205	0.861	1.020	1.104	0.387	0.082	0.209	4	4	4	100

Protein Group

peroxiredoxin-2 [Rattus norvegicus] gi|8394432 23925.4

Peptide Information

Calc. Mass	Obsrv. Mass	± da	± ppm	Start Seq.	End Seq.	Sequence	Ion Score	C. I. %	Modification	Plate [#] Name	Gel Idx/Pos [4700 Sample Name]	iTRAQ Ratio 115/114*	iTRAQ Ratio 116/114*	iTRAQ Ratio 117/114*	Rank	Result Type
1164.7239	1164.6797	-0.0442	-38	128	135	GLFIIDAK	48	99.961	(N-term)_iTRAQ[0], Lysine(K)_iTRAQ[8]	[7] F5 120912	291/283	1.126	1.026	0.912	1	Mascot
1355.7767	1355.7054	-0.0713	-53	140	150	QITVNDLPVGR	33	98.879	(N-term)_iTRAQ[0]	[1] F3 030912	242/234	0.458	0.944	0.946	1	Mascot
1396.8046	1396.7303	-0.0743	-53	110	119	SLSQNYGVLK	55	99.993	(N-term)_iTRAQ[0], Lysine(K)_iTRAQ[10]	[7] F5 120912	180/172	0.939	0.967	1.307	1	Mascot
2267.3708	2267.4045	0.0337	15	92	109	KEGGLGPLNIPLLADVTK	68	100	(N-term)_iTRAQ[0], Lysine(K)_iTRAQ[1,1 8]	[4] F7 and F10+11	463/455	1.131	1.154	1.319	1	Mascot

331	dihydropteridine reductase [Rattus norvegicus]	gi|11693160	28025.5	2	204	1.748	1.038	0.925	0.211	0.230	0.034	2	2	2	100

Peptide Information

Calc. Mass	Obsrv. Mass	± da	± ppm	Start Seq.	End Seq.	Sequence	Ion Score	C. I. %	Modification	Plate [#] Name	Gel Idx/Pos [4700 Sample Name]	iTRAQ Ratio 115/114*	iTRAQ Ratio 116/114*	iTRAQ Ratio 117/114*	Rank	Result Type
1840.9547	1840.8778	-0.0769	-42	136	151	AALDGTPGMIGYGMAK	94	100	(N-term)_iTRAQ[0], Lysine(K)_iTRAQ[16]	[5] F4	213/205	1.560	1.269	0.892	1	Mascot
2245.0903	2244.9978	-0.0925	-41	53	70	MTDSFTEQADQVTAEVG K	110	100	(N-term)_iTRAQ[0], Lysine(K)_iTRAQ[18]	[1] F3 030912	313/305	1.959	0.850	0.959	1	Mascot

332	NADH dehydrogenase (ubiquinone) 1 alpha subcomplex 10-like [Rattus norvegicus]	gi|32996721	44207	4	204	1.007	1.306	1.266	0.383	0.401	0.155	4	4	4	100

Peptide Information

Calc. Mass	Obsrv. Mass	± da	± ppm	Start	End	Sequence	Ion	C. I. %	Modification	Plate [#] Name	Gel Idx/Pos [4700	iTRAQ	iTRAQ	iTRAQ	Rank	Result Type

				Seq.	Seq.		Score				Sample Name]	Ratio 115/114*	Ratio 116/114*	Ratio 117/114*		
1437.8563	1437.8309	-0.0254	-18	38	48	YGLLASILGDK	51	99.981	(N-term)_iTRAQ[0], Lysine(K)_iTRAQ[11]	[5] F4	365/357	1.241	2.060	1.467	1	Mascot
1557.7795	1557.7245	-0.055	-35	339	350	YAPGYNADVGDK	59	99.997	(N-term)_iTRAQ[0], Lysine(K)_iTRAQ[12]	[7] F5 120912	144/136	0.843	1.186	1.110	1	Mascot
1786.9473	1786.8459	-0.1014	-57	269	280	VVEDIEYLNYNK	41	99.824	(N-term)_iTRAQ[0], Lysine(K)_iTRAQ[12]	[5] F4	242/234	1.498	1.039	1.153	1	Mascot
1829.9769	1830.061	0.0841	46	338	350	KYAPGYNADVGDK	53	99.988	(N-term)_iTRAQ[0], Lysine(K)_iTRAQ[1,13]	[4] F7 and F10+11	1050/1042	0.657	1.146	1.368	1	Mascot

333	V-type proton ATPase subunit d 1 [Mus musculus]	gi|31981304	43381	5	204	0.915	1.095	1.250	0.321	0.526	0.428	6	6	6	100

Peptide Information

Calc. Mass	Obsrv. Mass	± da	± ppm	Start Seq.	End Seq.	Sequence	Ion Score	C. I. %	Modification	Plate [#] Name	Gel Idx/Pos [4700 Sample Name]	iTRAQ Ratio 115/114*	iTRAQ Ratio 116/114*	iTRAQ Ratio 117/114*	Rank	Result Type
1223.667	1223.7729	0.1059	87	294	300	FFEHEVK	32	98.488	(N-term)_iTRAQ[0], Lysine(K)_iTRAQ[7]	[4] F7 and F10+11	1102/1094	1.413	2.020	1.707	1	Mascot
1308.7086	1308.7001	-0.0085	-6	188	195	AYLESFYK	43	99.873	(N-term)_iTRAQ[0], Lysine(K)_iTRAQ[8]	[7] F5 120912	259/251	0.537	0.610	0.872	1	Mascot
1374.7865	1374.7405	-0.046	-33	247	257	LYPEGLAQLAR	36	99.421	(N-term)_iTRAQ[0]	[1] F3 030912	335/327	0.989	0.960	1.350	1	Mascot
1549.7784	1549.6871	-0.0913	-59	266	275	NVADYYPEYK	46	99.943	(N-term)_iTRAQ[0], Lysine(K)_iTRAQ[10]	[5] F4	152/144	0.761	0.798	0.845	1	Mascot
1549.7784	1549.7399	-0.0385	-25	266	275	NVADYYPEYK	58	99.996	(N-term)_iTRAQ[0], Lysine(K)_iTRAQ[10]	[7] F5 120912	191/183	1.085	1.303	1.244	1	Mascot
1605.8346	1605.8009	-0.0337	-21	328	339	NIVWIAECIAQR	35	99.201	(N-term)_iTRAQ[0], MMTS (C)[8]	[1] F3 030912	541/533	0.943	1.400	1.803	1	Mascot

334	glutathione S-transferase P [Rattus norvegicus]	gi|25453420	25625.4	4	204	1.479	1.032	1.040	0.180	0.073	0.116	4	4	4	100

Peptide Information

Calc. Mass	Obsrv. Mass	± da	± ppm	Start Seq.	End Seq.	Sequence	Ion Score	C. I. %	Modification	Plate [#] Name	Gel Idx/Pos [4700 Sample Name]	iTRAQ Ratio 115/114*	iTRAQ Ratio 116/114*	iTRAQ Ratio 117/114*	Rank	Result Type
1025.6241	1025.5682	-0.0559	-55	76	82	SLGLYGK	34	99.053	(N-term)_iTRAQ[0], Lysine(K)_iTRAQ[7]	[3] F6 and F9	199/191	1.602	1.105	1.044	1	Mascot
1495.8434	1495.7679	-0.0755	-50	2	12	PPYTIVYFPVR	32	98.546	(N-term)_iTRAQ[0]	[5] F4	283/275	1.504	0.997	0.974	1	Mascot
1564.8403	1564.7924	-0.0479	-31	20	30	MLLADQGQSWK	66	99.999	(N-term)_iTRAQ[0], Lysine(K)_iTRAQ[11]	[7] F5 120912	248/240	1.221	0.939	0.934	1	Mascot
2567.468	2567.5576	0.0896	35	122	141	ALPGHLKPFETLLSQNQGGK	72	100	(N-term)_iTRAQ[0], Lysine(K)_iTRAQ[7,20]	[4] F7 and F10+11	1241/1233	1.628	1.099	1.233	1	Mascot

335	leucine-rich glioma-inactivated protein 1 precursor [Rattus norvegicus]	gi|21955128	70049.3	5	203	0.777	1.068	0.804	0.986	0.239	0.403	5	5	5	100

Peptide Information

Calc. Mass	Obsrv. Mass	± da	± ppm	Start Seq.	End Seq.	Sequence	Ion Score	C. I. %	Modification	Plate [#] Name	Gel Idx/Pos [4700 Sample Name]	iTRAQ Ratio 115/114*	iTRAQ Ratio 116/114*	iTRAQ Ratio 117/114*	Rank	Result Type
1345.7349	1345.6696	-0.0653	-49	514	523	FQELNVQAPR	30	97.467	(N-term)_iTRAQ[0]	[1] F3 030912	230/222	0.765	0.751	0.678	1	Mascot
1348.7511	1348.7322	-0.0189	-14	534	542	NFLFASSFK	31	97.908	(N-term)_iTRAQ[0], Lysine(K)_iTRAQ[9]	[7] F5 120912	341/333	0.160	1.098	0.389	1	Mascot
1424.7042	1424.6329	-0.0713	-50	460	470	WGGSSFQDIQR	56	99.993	(N-term)_iTRAQ[0]	[5] F4	170/162	1.240	1.359	1.101	1	Mascot
1635.799	1635.7444	-0.0546	-33	218	228	DFDCIITEFAK	42	99.849	(N-term)_iTRAQ[0], Lysine(K)_iTRAQ[11], MMTS (C)[4]	[5] F4	366/358	1.356	1.223	1.071	1	Mascot
1752.868	1752.8192	-0.0488	-28	260	270	CIFLEWDHVEK	46	99.936	(N-term)_iTRAQ[0], Lysine(K)_iTRAQ[11], MMTS (C)[1]	[6] F8 110912	459/451	1.379	1.014	1.082	1	Mascot

336	complement component 1 Q subcomponent-binding protein, mitochondrial precursor [Rattus norvegicus]	gi|48675371	33847.3	3	203	1.027	1.201	1.140	0.469	0.437	0.340	3	3	3	100

Protein Group

glycoprotein gC1qBP [Rattus norvegicus]	gi|2330657	33544.2

Peptide Information

Calc. Mass	Obsrv. Mass	± da	± ppm	Start Seq.	End Seq.	Sequence	Ion Score	C. I. %	Modification	Plate [#] Name	Gel Idx/Pos [4700 Sample Name]	iTRAQ Ratio 115/114*	iTRAQ Ratio 116/114*	iTRAQ Ratio 117/114*	Rank	Result Type
1599.8879	1599.8226	-0.0653	-41	79	89	AFVEFLTDEIK	42	99.842	(N-term)_iTRAQ[0], Lysine(K)_iTRAQ[11]	[5] F4	358/350	1.662	1.820	1.614	1	Mascot
1685.7891	1685.6982	-0.0909	-54	205	217	EVSFQTTGDSEWR	74	100	(N-term)_iTRAQ[0]	[1] F3 030912	242/234	0.664	0.864	0.861	1	Mascot
2130.1707	2130.0928	-0.0779	-37	79	92	AFVEFLTDEIKEEK	87	100	(N-term)_iTRAQ[0],	[6] F8 110912	469/461	0.982	1.103	1.067	1	Mascot

Lysine(K)_iTRAQ[11, 14]

337	secernin-1 [Rattus norvegicus]	gi|68163565	49898.6	4	201	1.449	1.257	1.486	1.032	0.476	0.764	4	4	4	100

Peptide Information

Calc. Mass	Obsrv. Mass	± da	± ppm	Start Seq.	End Seq.	Sequence	Ion Score	C. I. %	Modification	Plate [#] Name	Gel Idx/Pos [4700 Sample Name]	iTRAQ Ratio 115/114*	iTRAQ Ratio 116/114*	iTRAQ Ratio 117/114*	Rank	Result Type
1299.6123	1299.5416	-0.0707	-54	197	205	MDEEHPELR	44	99.895	(N-term)_iTRAQ[0]	[6] F8 110912	146/138	0.915	0.866	1.013	1	Mascot
1684.7538	1684.6753	-0.0785	-47	320	332	AQSPCFGDDDPAK	43	99.879	(N-term)_iTRAQ[0], Lysine(K)_iTRAQ[13], MMTS (C)[5]	[7] F5 120912	180/172	1.176	1.424	1.269	1	Mascot
1987.1639	1987.0964	-0.0675	-34	303	315	SIFKPFIFVDDVK	79	100	(N-term)_iTRAQ[0], Lysine(K)_iTRAQ[4,1 3]	[6] F8 110912	459/451	1.132	1.017	1.266	1	Mascot
2479.2239	2479.1221	-0.1018	-41	33	51	DEVQEVVYFPAVDHEAE SK	36	99.358	(N-term)_iTRAQ[0], Lysine(K)_iTRAQ[19]	[7] F5 120912	282/274	3.625	1.992	2.996	1	Mascot

338	rhodanese [Rattus norvegicus]	gi|57069	35501.1	4	201	2.159	1.960	1.545	3.113	2.666	1.657	4	4	4	100

Protein Group

thiosulfate sulfurtransferase [Rattus norvegicus]	gi|57528682	35731.2

Peptide Information

Calc. Mass	Obsrv. Mass	± da	± ppm	Start Seq.	End Seq.	Sequence	Ion Score	C. I. %	Modification	Plate [#] Name	Gel Idx/Pos [4700 Sample Name]	iTRAQ Ratio 115/114*	iTRAQ Ratio 116/114*	iTRAQ Ratio 117/114*	Rank	Result Type
1495.7665	1495.7073	-0.0592	-40	29	40	VLDASWYSPGTR	30	97.473	(N-term)_iTRAQ[0]	[5] F4	213/205	1.108	1.001	0.830	1	Mascot
2169.106	2169.0203	-0.0857	-40	186	204	YLGTQPEPDAVGLDSGHI R	62	99.998	(N-term)_iTRAQ[0]	[7] F5 120912	237/229	1.677	1.345	0.936	1	Mascot
2252.1921	2252.2949	0.1028	46	135	152	EGHPVTSEPSRPEPAVF K	33	98.782	(N-term)_iTRAQ[0], Lysine(K)_iTRAQ[18]	[4] F7 and F10+11	1088/1080	9.891	8.565	5.213	1	Mascot
2319.1577	2319.0989	-0.0588	-25	205	222	GSVNVPFMNFLTEDGFE K	77	100	(N-term)_iTRAQ[0], Lysine(K)_iTRAQ[18]	[1] F3 030912	506/498	1.183	1.279	1.405	1	Mascot

339	sulfated glycoprotein 1 isoform D preproprotein [Rattus norvegicus]	gi|298231229	67802.8	4	201	0.855	0.978	0.959	0.280	0.241	0.303	7	7	7	100

Protein Group

RecName: Full=Sulfated glycoprotein 1; Short=SGP-1; AltName: Full=Prosaposin; Flags: Precursor	gi|134219	67886.9

Peptide Information

Calc. Mass	Obsrv. Mass	± da	± ppm	Start Seq.	End Seq.	Sequence	Ion Score	C. I. %	Modification	Plate [#] Name	Gel Idx/Pos [4700 Sample Name]	iTRAQ Ratio 115/114*	iTRAQ Ratio 116/114*	iTRAQ Ratio 117/114*	Rank	Result Type
1588.7731	1588.7279	-0.0452	-28	468	478	GCSFLPDPYQK	43	99.874	(N-term)_iTRAQ[0], Lysine(K)_iTRAQ[11], MMTS (C)[2]	[5] F4	258/250	0.828	0.734	0.695	1	Mascot
1588.7731	1588.7394	-0.0337	-21	468	478	GCSFLPDPYQK	44	99.902	(N-term)_iTRAQ[0], Lysine(K)_iTRAQ[11], MMTS (C)[2]	[7] F5 120912	326/318	1.219	1.079	1.530	1	Mascot
1779.0024	1778.8713	-0.1311	-74	410	421	EPAPPKQPEEPK	31	97.874	(N-term)_iTRAQ[0], Lysine(K)_iTRAQ[6,1 2]	[6] F8 110912	124/116	0.977	0.906	1.089	1	Mascot
1931.1702	1931.1251	-0.0451	-23	443	454	KLVIYLEHNLEK	36	99.318	(N-term)_iTRAQ[0], Lysine(K)_iTRAQ[1,1 2]	[2] F12 040912	390/382	0.750	1.450	1.022	1	Mascot
1931.1702	1931.1984	0.0282	15	443	454	KLVIYLEHNLEK	27	95.207	(N-term)_iTRAQ[0], Lysine(K)_iTRAQ[1,1 2]	[4] F7 and F10+11	1255/1247	0.484	0.768	0.661	1	Mascot
2022.1443	2022.0963	-0.048	-24	108	122	EVVDSYLPVILDMIK	31	98.092	(N-term)_iTRAQ[0], Lysine(K)_iTRAQ[15]	[8] F13-15 and F1+2	237/229	0.835	0.929	0.839	1	Mascot
2022.1443	2022.1058	-0.0385	-19	108	122	EVVDSYLPVILDMIK	93	100	(N-term)_iTRAQ[0], Lysine(K)_iTRAQ[15]	[1] F3 030912	558/550	1.122	1.152	1.138	1	Mascot

340	GLAST-1a [Rattus norvegicus]	gi|8118689	58370.5	4	200	0.863	0.906	0.947	0.176	0.232	0.260	5	5	5	100

Protein Group

RecName: Full=Excitatory amino acid transporter 1; AltName: Full=Glial glutamate transporter; AltNa	gi|232176	63975.6
excitatory amino acid transporter 1 [Rattus norvegicus]	gi|9507115	63961.6

Peptide Information

Calc. Mass	Obsrv. Mass	± da	± ppm	Start Seq.	End Seq.	Sequence	Ion Score	C. I. %	Modification	Plate [#] Name	Gel Idx/Pos [4700 Sample Name]	iTRAQ Ratio	iTRAQ Ratio	iTRAQ Ratio	Rank	Result Type

Calc. Mass	Obsrv. Mass	± da	± ppm	Start Seq.	End Seq.	Sequence	Ion Score	C. I. %	Modification	Plate [#] Name	Gel Idx/Pos [4700 Sample Name]	iTRAQ Ratio 115/114*	iTRAQ Ratio 116/114*	iTRAQ Ratio 117/114*	Rank	Result Type
1789.0344	1789.0107	-0.0237	-13	115	129	IVQVTAADAFLDLIR	74	100	(N-term)_iTRAQ[0]	[8] F13-15 and F1+2	251/243	0.876	0.891	0.827	1	Mascot
1843.9136	1843.8354	-0.0782	-42	130	142	NMFPPNLVEACFK	56	99.994	(N-term)_iTRAQ[0], Lysine(K)_iTRAQ[13], MMTS (C)[11]	[5] F4	398/390	0.768	1.186	1.035	1	Mascot
1843.9136	1843.874	-0.0396	-21	130	142	NMFPPNLVEACFK	60	99.997	(N-term)_iTRAQ[0], Lysine(K)_iTRAQ[13], MMTS (C)[11]	[1] F3 030912	517/509	1.155	1.144	1.423	1	Mascot
2011.9562	2011.8481	-0.1081	-54	460	474	DVEMGNSVIEENEMK	40	99.73	(N-term)_iTRAQ[0], Lysine(K)_iTRAQ[15]	[1] F3 030912	235/227	0.927	0.773	0.905	1	Mascot
2184.1743	2184.1355	-0.0388	-18	434	453	TTTNVLGDSLGAGIVEHL SR	27	95.005	(N-term)_iTRAQ[0]	[7] F5 120912	376/368	0.664	0.653	0.690	1	Mascot

341	diacylglycerol kinase beta [Rattus norvegicus]	gi|9506535	101413.9	4	200	0.570	0.986	0.948	0.199	0.197	0.243	4	4	4	100

Peptide Information

Calc. Mass	Obsrv. Mass	± da	± ppm	Start Seq.	End Seq.	Sequence	Ion Score	C. I. %	Modification	Plate [#] Name	Gel Idx/Pos [4700 Sample Name]	iTRAQ Ratio 115/114*	iTRAQ Ratio 116/114*	iTRAQ Ratio 117/114*	Rank	Result Type
1628.7988	1628.7377	-0.0611	-38	532	543	WGGGYEGENLMK	67	99.999	(N-term)_iTRAQ[0], Lysine(K)_iTRAQ[12]	[7] F5 120912	236/228	0.525	1.026	0.901	1	Mascot
1753.9343	1754.0184	0.0841	48	561	571	FEVTPNDKDEK	33	98.677	(N-term)_iTRAQ[0], Lysine(K)_iTRAQ[8,11]	[4] F7 and F10+11	1058/1050	0.532	0.727	0.679	1	Mascot
1956.0323	1955.9255	-0.1068	-55	51	64	QDILNQTIDFEGFK	52	99.986	(N-term)_iTRAQ[0], Lysine(K)_iTRAQ[14]	[5] F4	302/294	0.409	1.178	1.037	1	Mascot
2365.0754	2365.1443	0.0689	29	302	318	NTDVMHHYWVEGNCPT K	49	99.966	(N-term)_iTRAQ[0], Lysine(K)_iTRAQ[17], MMTS (C)[14]	[4] F7 and F10+11	1173/1165	0.928	1.074	1.271	1	Mascot

342	microtubule-associated protein-2 kinase [Rattus norvegicus]	gi|515499	45559.9	4	200	1.081	1.205	1.182	0.259	0.279	0.275	4	4	4	100

Protein Group

extracellular signal-regulated kinase 1 [Rattus norvegicus]	gi|204052	45460.8
extracellular signal-regulated kinase 1b [Rattus norvegicus]	gi|8050445	49179.6
extracellular-signal-regulated kinase 1 [Rattus norvegicus]	gi|204054	45914
mitogen-activated protein kinase 3 [Rattus norvegicus]	gi|8393331	46400.2

Peptide Information

Calc. Mass	Obsrv. Mass	± da	± ppm	Start Seq.	End Seq.	Sequence	Ion Score	C. I. %	Modification	Plate [#] Name	Gel Idx/Pos [4700 Sample Name]	iTRAQ Ratio 115/114*	iTRAQ Ratio 116/114*	iTRAQ Ratio 117/114*	Rank	Result Type
1698.7833	1698.7009	-0.0824	-49	61	72	ISPFEHQTYCQR	31	98.083	(N-term)_iTRAQ[0], MMTS (C)[10]	[6] F8 110912	264/256	1.373	1.376	1.189	1	Mascot
1970.9807	1970.9188	-0.0619	-31	60	72	KISPFEHQTYCQR	30	97.531	(N-term)_iTRAQ[0], Lysine(K)_iTRAQ[1], MMTS (C)[11]	[2] F12 040912	257/249	0.918	1.068	1.516	1	Mascot
2133.1035	2133.041	-0.0625	-29	105	119	DVYIVQDLMETDLYK	81	100	(N-term)_iTRAQ[0], Lysine(K)_iTRAQ[15]	[1] F3 030912	477/469	0.834	0.919	0.851	1	Mascot
2266.2502	2266.2617	0.0115	5	154	169	DLKPSNLLINTTCDLK	58	99.996	(N-term)_iTRAQ[0], Lysine(K)_iTRAQ[3,16], MMTS (C)[13]	[4] F7 and F10+11	407/399	1.296	1.563	1.273	1	Mascot

343	RecName: Full=Versican core protein; AltName: Full=Chondroitin sulfate proteoglycan core protein 2;	gi|21431624	317904	4	200	1.608	1.006	1.043	0.659	0.678	0.583	4	4	4	100

Protein Group

versican core protein isoform 4 [Rattus norvegicus]	gi|281604092	80601.1

Peptide Information

Calc. Mass	Obsrv. Mass	± da	± ppm	Start Seq.	End Seq.	Sequence	Ion Score	C. I. %	Modification	Plate [#] Name	Gel Idx/Pos [4700 Sample Name]	iTRAQ Ratio 115/114*	iTRAQ Ratio 116/114*	iTRAQ Ratio 117/114*	Rank	Result Type
1675.9476	1675.8193	-0.1283	-77	80	92	ETTVLVAQDGNIK	69	100	(N-term)_iTRAQ[0], Lysine(K)_iTRAQ[13]	[5] F4	130/122	2.158	1.303	1.242	1	Mascot
1706.7255	1706.6814	-0.0441	-26	261	273	FTFEEAEAECANR	37	99.478	(N-term)_iTRAQ[0], MMTS (C)[10]	[1] F3 030912	354/346	1.261	0.583	0.778	1	Mascot
1832.9541	1833.016	0.0619	34	2561	2573	VGHDYQWIGLNDK	54	99.991	(N-term)_iTRAQ[0], Lysine(K)_iTRAQ[13]	[4] F7 and F10+11	1179/1171	2.345	2.058	1.971	1	Mascot
2256.1792	2256.0923	-0.0869	-39	101	119	VSVPTHPDDVGDASLTM VK	40	99.732	(N-term)_iTRAQ[0], Lysine(K)_iTRAQ[19]	[7] F5 120912	246/238	1.048	0.655	0.622	1	Mascot

344	SH3p4 [Rattus norvegicus]	gi|2293468	30796.7	3	197	0.867	0.937	1.187	0.169	0.092	0.157	3	3	3	100

Peptide Information

Calc. Mass	Obsrv. Mass	± da	± ppm	Start Seq.	End Seq.	Sequence	Ion Score	C. I. %	Modification	Plate [#] Name	Gel Idx/Pos [4700 Sample Name]	iTRAQ Ratio 115/114*	iTRAQ Ratio 116/114*	iTRAQ Ratio 117/114*	Rank	Result Type
1526.9138	1526.8612	-0.0526	-34	124	135	QAVQILQQVTVR	44	99.892	(N-term)_iTRAQ[0]	[1] F3 030912	305/297	1.090	0.970	1.066	1	Mascot
2146.0591	2145.9907	-0.0684	-32	193	208	ALYDFEPENEGELGFK	92	100	(N-term)_iTRAQ[0], Lysine(K)_iTRAQ[16]	[1] F3 030912	369/361	0.707	0.825	1.110	1	Mascot
2254.219	2254.292	0.073	32	33	48	QNFIDPLQNLHDKDLR	62	99.998	(N-term)_iTRAQ[0], Lysine(K)_iTRAQ[13]	[4] F7 and F10+11	1217/1209	0.844	1.028	1.412	1	Mascot

345	superoxide dismutase [Cu-Zn] [Rattus norvegicus]	gi|8394328	17480.8	3	196	0.733	0.997	0.698	0.346	0.132	0.375	4	4	4	100

Protein Group

Cu-Zn superoxide dismutase (EC 1.15.1.1) [Rattus norvegicus]	gi|203658	17278.7
Cu/Zn superoxide dismutase [Rattus norvegicus]	gi|1213217	17440.7
Unknown (protein for IMAGE:6890907) [Rattus norvegicus]	gi|34784756	19626.5
dismutase [Rattus norvegicus]	gi|818029	17147.7

Peptide Information

Calc. Mass	Obsrv. Mass	± da	± ppm	Start Seq.	End Seq.	Sequence	Ion Score	C. I. %	Modification	Plate [#] Name	Gel Idx/Pos [4700 Sample Name]	iTRAQ Ratio 115/114*	iTRAQ Ratio 116/114*	iTRAQ Ratio 117/114*	Rank	Result Type
1318.6722	1318.6018	-0.0704	-53	93	103	DGVANVSIEDR	44	99.898	(N-term)_iTRAQ[0]	[1] F3 030912	177/169	0.420	0.864	0.331	1	Mascot
1425.8059	1425.8921	0.0862	60	81	92	HVGDLGNVAAGK	44	99.898	(N-term)_iTRAQ[0], Lysine(K)_iTRAQ[12]	[4] F7 and F10+11	1063/1055	0.629	0.928	0.866	1	Mascot
1798.9697	1798.8586	-0.1111	-62	11	24	GDGPVQGVIHFEQK	109	100	(N-term)_iTRAQ[0], Lysine(K)_iTRAQ[14]	[6] F8 110912	261/253	1.121	1.202	0.886	1	Mascot
1798.9697	1798.9426	-0.0271	-15	11	24	GDGPVQGVIHFEQK	76	100	(N-term)_iTRAQ[0], Lysine(K)_iTRAQ[14]	[4] F7 and F10+11	262/254	0.977	1.025	0.932	1	Mascot

346	protein phosphatase 1 regulatory subunit 1B [Rattus norvegicus]	gi|61889054	24241.8	4	196	0.924	0.606	0.671	0.306	0.367	0.147	6	6	6	100

Peptide Information

Calc. Mass	Obsrv. Mass	± da	± ppm	Start Seq.	End Seq.	Sequence	Ion Score	C. I. %	Modification	Plate [#] Name	Gel Idx/Pos [4700 Sample Name]	iTRAQ Ratio 115/114*	iTRAQ Ratio 116/114*	iTRAQ Ratio 117/114*	Rank	Result Type
1795.9827	1795.9944	0.0117	7	9	23	IQFSVPAPPSQLDPR	29	97.178	(N-term)_iTRAQ[0]	[8] F13-15 and F1+2	434/426	1.457	1.238	0.846	1	Mascot
1795.9827	1795.9969	0.0142	8	9	23	IQFSVPAPPSQLDPR	35	99.159	(N-term)_iTRAQ[0]	[8] F13-15 and F1+2	483/475	1.213	0.871	0.672	1	Mascot
1795.9827	1795.9993	0.0166	9	9	23	IQFSVPAPPSQLDPR	32	98.559	(N-term)_iTRAQ[0]	[8] F13-15 and F1+2	430/422	0.945	0.605	0.521	1	Mascot
2068.1802	2068.1743	-0.0059	-3	8	23	KIQFSVPAPPSQLDPR	29	96.752	(N-term)_iTRAQ[0], Lysine(K)_iTRAQ[1]	[4] F7 and F10+11	325/317	0.744	0.558	0.889	1	Mascot
2248.0603	2247.9851	-0.0752	-33	186	205	ATQSEPGEEPRHPTPPE SGT	32	98.35	(N-term)_iTRAQ[0]	[7] F5 120912	93/85	0.638	0.268	0.564	1	Mascot
3285.5842	3285.4675	-0.1167	-36	85	112	IAESHLQTISNLSENQASE EEDELGELR	101	100	(N-term)_iTRAQ[0]	[1] F3 030912	361/353	0.786	0.507	0.615	1	Mascot

347	puromycin-sensitive aminopeptidase [Rattus norvegicus]	gi|17221643	21627	4	196	1.283	0.904	1.058	0.512	0.314	0.607	6	6	6	100

Peptide Information

Calc. Mass	Obsrv. Mass	± da	± ppm	Start Seq.	End Seq.	Sequence	Ion Score	C. I. %	Modification	Plate [#] Name	Gel Idx/Pos [4700 Sample Name]	iTRAQ Ratio 115/114*	iTRAQ Ratio 116/114*	iTRAQ Ratio 117/114*	Rank	Result Type
1437.7834	1437.7273	-0.0561	-39	89	98	IDFVGELNDK	54	99.991	(N-term)_iTRAQ[0], Lysine(K)_iTRAQ[10]	[7] F5 120912	273/265	1.243	0.829	0.719	1	Mascot
1586.7935	1586.8016	0.0081	5	116	128	YAAVTQFEATDAR	46	99.945	(N-term)_iTRAQ[0]	[8] F13-15 and F1+2	524/516	0.865	0.651	0.861	1	Mascot
1586.7935	1586.8057	0.0122	8	116	128	YAAVTQFEATDAR	45	99.923	(N-term)_iTRAQ[0]	[8] F13-15 and F1+2	527/519	1.719	1.183	1.836	1	Mascot
1586.7935	1586.8164	0.0229	14	116	128	YAAVTQFEATDAR	38	99.617	(N-term)_iTRAQ[0]	[8] F13-15 and F1+2	573/565	1.078	0.727	0.698	1	Mascot
1610.7938	1610.7913	-0.0025	-2	130	140	AFPCWDEPAIK	42	99.841	(N-term)_iTRAQ[0], Lysine(K)_iTRAQ[11], MMTS (C)[4]	[7] F5 120912	363/355	2.281	1.525	2.147	1	Mascot
1978.0868	1978.1277	0.0409	21	165	177	KPYPDDENLVEVK	53	99.989	(N-term)_iTRAQ[0], Lysine(K)_iTRAQ[1,1 3]	[3] F6 and F9	1187/1179	0.981	0.772	0.822	1	Mascot

348	RecName: Full=cAMP-dependent protein kinase	gi|125207	45873.6	4	196	0.802	0.967	1.007	0.239	0.270	0.493	5	5	5	100

catalytic subunit alpha; Short=PKA C-alpha

Peptide Information

Calc. Mass	Obsrv. Mass	± da	± ppm	Start Seq.	End Seq.	Sequence	Ion Score	C. I. %	Modification	Plate [#] Name	Gel Idx/Pos [4700 Sample Name]	iTRAQ Ratio 115/114*	iTRAQ Ratio 116/114*	iTRAQ Ratio 117/114*	Rank	Result Type
1452.7733	1452.8361	0.0628	43	258	267	FPSHFSSDLK	39	99.711	(N-term)_iTRAQ[0], Lysine(K)_iTRAQ[10]	[3] F6 and F9	1208/1200	0.810	0.823	0.926	1	Mascot
1452.7733	1452.8463	0.073	50	258	267	FPSHFSSDLK	41	99.799	(N-term)_iTRAQ[0], Lysine(K)_iTRAQ[10]	[4] F7 and F10+11	1140/1132	1.102	1.260	1.795	1	Mascot
1460.9213	1460.9834	0.0621	43	310	318	KVEAPFIPK	42	99.854	(N-term)_iTRAQ[0], Lysine(K)_iTRAQ[1,9]	[4] F7 and F10+11	1128/1120	1.028	1.222	1.402	1	Mascot
1677.0349	1676.97	-0.0649	-39	95	106	ILQAVNFPFLVK	46	99.943	(N-term)_iTRAQ[0], Lysine(K)_iTRAQ[12]	[5] F4	384/376	0.560	0.657	0.642	1	Mascot
1814.9351	1814.8776	-0.0575	-32	297	309	WFATTDWIAIYQR	66	99.999	(N-term)_iTRAQ[0]	[1] F3 030912	496/488	0.644	1.016	0.690	1	Mascot

349	metalloendopeptidase [Rattus norvegicus]	gi|205374	79144.6	4	196	1.642	1.106	1.367	0.734	0.427	1.530	4	4	4	100

Protein Group

thimet oligopeptidase [Rattus norvegicus]	gi|126723582	85221.6
thimet oligopeptidase=soluble angiotensin II-binding protein homolog {EC 3.4.24.15} [rats, testis,	gi|425685	85116.6

Peptide Information

Calc. Mass	Obsrv. Mass	± da	± ppm	Start Seq.	End Seq.	Sequence	Ion Score	C. I. %	Modification	Plate [#] Name	Gel Idx/Pos [4700 Sample Name]	iTRAQ Ratio 115/114*	iTRAQ Ratio 116/114*	iTRAQ Ratio 117/114*	Rank	Result Type
1711.8776	1711.8361	-0.0415	-24	181	193	NLNEDTTFLPFTR	35	99.219	(N-term)_iTRAQ[0]	[1] F3 030912	356/348	1.316	0.705	0.539	1	Mascot
2078.0903	2078.0342	-0.0561	-27	194	209	EELGGLPEDFLNSLEK	55	99.992	(N-term)_iTRAQ[0], Lysine(K)_iTRAQ[16]	[1] F3 030912	478/470	1.818	1.491	1.258	1	Mascot
2330.1384	2330.04	-0.0984	-42	560	578	VDQVLHTQTDVDPAEEYAR	34	99.046	(N-term)_iTRAQ[0]	[7] F5 120912	225/217	2.844	1.528	4.390	1	Mascot
2396.1843	2396.1121	-0.0722	-30	499	515	DFVEAPSQMLENWVWEK	72	100	(N-term)_iTRAQ[0], Lysine(K)_iTRAQ[17]	[1] F3 030912	515/507	1.068	0.930	1.174	1	Mascot

350	Tra1 protein [Rattus norvegicus]	gi|51858886	83712.2	4	196	1.053	1.040	1.179	0.432	0.329	0.401	4	4	4	100

Peptide Information

Calc. Mass	Obsrv. Mass	± da	± ppm	Start Seq.	End Seq.	Sequence	Ion Score	C. I. %	Modification	Plate [#] Name	Gel Idx/Pos [4700 Sample Name]	iTRAQ Ratio 115/114*	iTRAQ Ratio 116/114*	iTRAQ Ratio 117/114*	Rank	Result Type
1303.678	1303.6322	-0.0458	-35	396	404	GLFDEYGSK	53	99.988	(N-term)_iTRAQ[0], Lysine(K)_iTRAQ[9]	[3] F6 and F9	242/234	1.770	1.470	1.847	1	Mascot
1563.8475	1563.792	-0.0555	-35	103	114	ELISNASDALDK	49	99.97	(N-term)_iTRAQ[0], Lysine(K)_iTRAQ[12]	[5] F4	164/156	0.906	0.999	0.864	1	Mascot
1883.9281	1883.928	-0.0001	0	416	428	VFITDDFHDMMPK	60	99.997	(N-term)_iTRAQ[0], Lysine(K)_iTRAQ[13]	[4] F7 and F10+11	383/375	1.109	1.156	1.257	1	Mascot
2297.2222	2297.2341	0.0119	5	609	623	EATEKEFEPLLNWMK	34	98.934	(N-term)_iTRAQ[0], Lysine(K)_iTRAQ[5,15]	[4] F7 and F10+11	446/438	0.692	0.689	0.964	1	Mascot

351	RecName: Full=Nck-associated protein 1; Short=NAP 1; AltName: Full=Membrane-associated protein HEM-	gi|1708980	139494	5	195	0.686	1.016	1.161	0.507	0.436	0.416	5	5	5	100

Peptide Information

Calc. Mass	Obsrv. Mass	± da	± ppm	Start Seq.	End Seq.	Sequence	Ion Score	C. I. %	Modification	Plate [#] Name	Gel Idx/Pos [4700 Sample Name]	iTRAQ Ratio 115/114*	iTRAQ Ratio 116/114*	iTRAQ Ratio 117/114*	Rank	Result Type
1484.7141	1484.6581	-0.056	-38	474	484	QVEDGEVFDFR	31	98.252	(N-term)_iTRAQ[0]	[1] F3 030912	281/273	0.489	0.614	0.682	1	Mascot
1522.7644	1522.6897	-0.0747	-49	192	201	MMEEFVPHSK	42	99.859	(N-term)_iTRAQ[0], Lysine(K)_iTRAQ[10]	[6] F8 110912	266/258	0.387	0.974	1.279	1	Mascot
1756.8286	1756.7732	-0.0554	-32	552	564	MFQQCLELPSQSR	41	99.819	(N-term)_iTRAQ[0], MMTS (C)[5]	[1] F3 030912	392/384	1.738	1.879	1.648	1	Mascot
1799.0789	1799.0964	0.0175	10	1035	1048	AINQIAAALFTIHK	39	99.707	(N-term)_iTRAQ[0], Lysine(K)_iTRAQ[14]	[4] F7 and F10+11	472/464	0.484	0.956	1.417	1	Mascot
1935.9279	1935.8823	-0.0456	-24	595	608	SLSLCNMFLDEMAK	40	99.761	(N-term)_iTRAQ[0], Lysine(K)_iTRAQ[14], MMTS (C)[5]	[5] F4	421/413	0.950	1.006	1.037	1	Mascot

352	guanine nucleotide-binding protein G(z) subunit alpha [Rattus norvegicus]	gi|6980966	44582.1	3	194	1.011	1.273	1.143	0.209	0.105	0.035	3	3	3	100

Peptide Information

Calc. Mass	Obsrv. Mass	± da	± ppm	Start Seq.	End Seq.	Sequence	Ion Score	C. I. %	Modification	Plate [#] Name	Gel Idx/Pos [4700 Sample Name]	iTRAQ Ratio 115/114*	iTRAQ Ratio 116/114*	iTRAQ Ratio 117/114*	Rank	Result Type

Calc. Mass	Obsrv. Mass	± da	± ppm	Start Seq.	End Seq.	Sequence	Ion Score	C. I. %	Modification	Plate [#] Name	Gel Idx/Pos [4700 Sample Name]	iTRAQ Ratio 115/114*	iTRAQ Ratio 116/114*	iTRAQ Ratio 117/114*	Rank	Result Type
1785.8892	1785.804	-0.0852	-48	293	306	GQNTYEEAAVYIQR	66	99.999	(N-term)_iTRAQ[0]	[1] F3 030912	252/244	0.803	1.163	1.124	1	Mascot
1830.0133	1830.0264	0.0131	7	163	177	IAAPDYIPTVEDILR	54	99.989	(N-term)_iTRAQ[0]	[8] F13-15 and F1+2	337/329	1.012	1.259	1.193	1	Mascot
2069.1165	2069.0522	-0.0643	-31	101	117	AYDAVQLFALTGPAESK	76	100	(N-term)_iTRAQ[0], Lysine(K)_iTRAQ[17]	[1] F3 030912	395/387	1.273	1.409	1.113	1	Mascot

353	serine/threonine-protein phosphatase 2A catalytic subunit alpha isoform [Rattus norvegicus]	gi|8394018	37918.6	4	194	1.183	1.126	1.198	0.684	0.253	0.497	4	4	4	100

Peptide Information

Calc. Mass	Obsrv. Mass	± da	± ppm	Start Seq.	End Seq.	Sequence	Ion Score	C. I. %	Modification	Plate [#] Name	Gel Idx/Pos [4700 Sample Name]	iTRAQ Ratio 115/114*	iTRAQ Ratio 116/114*	iTRAQ Ratio 117/114*	Rank	Result Type
1484.7659	1484.7206	-0.0453	-31	284	294	YSFLQFDPAPR	42	99.835	(N-term)_iTRAQ[0]	[1] F3 030912	369/361	1.232	1.079	1.116	1	Mascot
1511.8706	1511.8051	-0.0655	-43	136	144	KYGNANVWK	36	99.358	(N-term)_iTRAQ[0], Lysine(K)_iTRAQ[1,9]	[2] F12 040912	193/185	2.308	1.584	2.109	1	Mascot
1981.959	1981.8925	-0.0665	-34	9	21	ELDQWIEQLNECK	47	99.955	(N-term)_iTRAQ[0], Lysine(K)_iTRAQ[13], MMTS (C)[12]	[5] F4	378/370	1.075	0.989	1.067	1	Mascot
2048.8994	2048.8923	-0.0071	-3	240	254	AHQLVMEGYNWCHDR	69	100	(N-term)_iTRAQ[0], MMTS (C)[12]	[2] F12 040912	383/375	0.641	0.950	0.821	1	Mascot

354	dynein light chain 2, cytoplasmic [Mus musculus]	gi|18087731	12020.2	3	193	0.818	1.027	0.966	0.171	0.373	0.227	3	3	3	100

Peptide Information

Calc. Mass	Obsrv. Mass	± da	± ppm	Start Seq.	End Seq.	Sequence	Ion Score	C. I. %	Modification	Plate [#] Name	Gel Idx/Pos [4700 Sample Name]	iTRAQ Ratio 115/114*	iTRAQ Ratio 116/114*	iTRAQ Ratio 117/114*	Rank	Result Type
1081.6503	1081.5962	-0.0541	-50	37	43	DIAAYIK	48	99.96	(N-term)_iTRAQ[0], Lysine(K)_iTRAQ[7]	[7] F5 120912	219/211	0.764	0.717	0.837	1	Mascot
1570.8112	1570.8656	0.0544	35	61	71	NFGSYVTHETK	48	99.957	(N-term)_iTRAQ[0], Lysine(K)_iTRAQ[11]	[3] F6 and F9	1161/1153	0.675	0.991	0.827	1	Mascot
2765.1423	2765.0532	-0.0891	-32	10	31	NADMSEDMQQDAVDCATQAMEK	98	100	(N-term)_iTRAQ[0], Lysine(K)_iTRAQ[22], MMTS (C)[15]	[1] F3 030912	401/393	1.059	1.526	1.302	1	Mascot

355	Chain A, Crystal Structure Of Recombinant Rat-Liver D244e Mutant S- Adenosylhomocysteine Hydrolase	gi|13096481	52271.2	5	193	1.003	1.238	0.972	0.443	0.345	0.238	5	5	5	100

Protein Group

Chain A, Rat Liver S-Adenosylhomocystein Hydrolase	gi|4139571	52257.2
adenosylhomocysteinase [Mus musculus]	gi|262263372	52538.3
adenosylhomocysteinase [Rattus norvegicus]	gi|8392878	52388.3

Peptide Information

Calc. Mass	Obsrv. Mass	± da	± ppm	Start Seq.	End Seq.	Sequence	Ion Score	C. I. %	Modification	Plate [#] Name	Gel Idx/Pos [4700 Sample Name]	iTRAQ Ratio 115/114*	iTRAQ Ratio 116/114*	iTRAQ Ratio 117/114*	Rank	Result Type
1272.7185	1272.6497	-0.0688	-54	8	18	VADIGLAAWGR	37	99.508	(N-term)_iTRAQ[0]	[5] F4	252/244	1.044	0.994	1.061	1	Mascot
1536.7529	1536.829	0.0761	50	186	195	SKFDNLYGCR	30	97.542	(N-term)_iTRAQ[0], Lysine(K)_iTRAQ[2], MMTS (C)[9]	[4] F7 and F10+11	1163/1155	1.196	1.673	1.323	1	Mascot
1813.0554	1812.995	-0.0604	-33	388	400	KLDEAVAEAHLGK	63	99.999	(N-term)_iTRAQ[0], Lysine(K)_iTRAQ[1,13]	[2] F12 040912	262/254	0.896	1.232	1.038	1	Mascot
1976.0554	1976.0555	0.0001	0	19	33	KALDIAENEMPGLMR	35	99.167	(N-term)_iTRAQ[0], Lysine(K)_iTRAQ[1]	[4] F7 and F10+11	356/348	0.547	0.898	0.687	1	Mascot
2540.3276	2540.2502	-0.0774	-30	121	141	DGPLNMILDDGGDLTNLIHTK	29	96.707	(N-term)_iTRAQ[0], Lysine(K)_iTRAQ[21]	[7] F5 120912	486/478	1.662	1.581	0.867	1	Mascot

356	protein NDRG2 [Rattus norvegicus]	gi|19424278	41538.7	4	191	1.099	0.907	0.818	0.082	0.131	0.171	4	4	4	100

Protein Group

RecName: Full=Protein NDRG2; AltName: Full=Antidepressant-related protein ADRG123; AltName: Full=ND	gi|81867103	43046.5
antidepressant-related protein ADRG123 [Rattus norvegicus]	gi|18478482	43031.5
antidepressant-related protein ADRG123 splice variant [Rattus norvegicus]	gi|18478484	41523.7

Peptide Information

Calc. Mass	Obsrv. Mass	± da	± ppm	Start	End	Sequence	Ion	C. I. %	Modification	Plate [#] Name	Gel Idx/Pos [4700	iTRAQ	iTRAQ	iTRAQ	Rank	Result Type

Calc. Mass	Obsrv. Mass	± da	± ppm	Start Seq.	End Seq.	Sequence	Ion Score	C. I. %	Modification	Plate [#] Name	Gel Idx/Pos [4700 Sample Name]	iTRAQ Ratio 115/114*	iTRAQ Ratio 116/114*	iTRAQ Ratio 117/114*	Rank	Result Type
1389.6984	1389.7478	0.0494	36	163	171	GWMDWAAHK	47	99.956	(N-term)_iTRAQ[0], Lysine(K)_iTRAQ[9]	[4] F7 and F10+11	1197/1189	1.088	0.744	0.869	1	Mascot
1437.8199	1437.7205	-0.0994	-69	264	273	LDPTQTSFLK	32	98.579	(N-term)_iTRAQ[0], Lysine(K)_iTRAQ[10]	[5] F4	183/175	0.990	0.896	0.794	1	Mascot
1802.9316	1802.8219	-0.1097	-61	274	288	MADSGGQPQLTQPGK	79	100	(N-term)_iTRAQ[0], Lysine(K)_iTRAQ[15]	[5] F4	102/94	1.212	1.087	1.048	1	Mascot
2082.1382	2082.2004	0.0622	30	48	62	RPAIFTYHDVGLNYK	32	98.353	(N-term)_iTRAQ[0], Lysine(K)_iTRAQ[15]	[4] F7 and F10+11	1199/1191	1.119	0.935	0.620	1	Mascot

357	astrocytic phosphoprotein PEA-15 [Homo sapiens]	gi|4505705	17094.2	4	191	1.088	1.337	1.255	0.315	0.283	0.306	4	4	4	100

Peptide Information

Calc. Mass	Obsrv. Mass	± da	± ppm	Start Seq.	End Seq.	Sequence	Ion Score	C. I. %	Modification	Plate [#] Name	Gel Idx/Pos [4700 Sample Name]	iTRAQ Ratio 115/114*	iTRAQ Ratio 116/114*	iTRAQ Ratio 117/114*	Rank	Result Type
1466.7472	1466.6957	-0.0515	-35	89	98	ISEEDELDTK	44	99.902	(N-term)_iTRAQ[0], Lysine(K)_iTRAQ[10]	[7] F5 120912	149/141	1.001	1.112	1.386	1	Mascot
1684.8602	1684.9319	0.0717	43	25	35	SACKEDIPSEK	47	99.95	(N-term)_iTRAQ[0], Lysine(K)_iTRAQ[4,11], MMTS (C)[3]	[4] F7 and F10+11	1065/1057	1.616	1.527	1.334	1	Mascot
2380.2759	2380.3425	0.0666	28	55	71	LDKDNLSYIEHIFEISR	44	99.897	(N-term)_iTRAQ[0], Lysine(K)_iTRAQ[3]	[4] F7 and F10+11	1334/1326	0.800	1.704	1.541	1	Mascot
2458.2136	2458.2908	0.0772	31	36	54	SEEITTGSAWFSFLESHNK	56	99.994	(N-term)_iTRAQ[0], Lysine(K)_iTRAQ[19]	[4] F7 and F10+11	444/436	1.084	1.104	0.870	1	Mascot

358	G-septin beta [Rattus norvegicus]	gi|4455011	44242.1	4	190	0.873	0.876	1.023	0.312	0.247	0.273	5	5	5	100

Peptide Information

Calc. Mass	Obsrv. Mass	± da	± ppm	Start Seq.	End Seq.	Sequence	Ion Score	C. I. %	Modification	Plate [#] Name	Gel Idx/Pos [4700 Sample Name]	iTRAQ Ratio 115/114*	iTRAQ Ratio 116/114*	iTRAQ Ratio 117/114*	Rank	Result Type
1374.7151	1374.6661	-0.049	-36	146	153	YINEQYEK	44	99.895	(N-term)_iTRAQ[0], Lysine(K)_iTRAQ[8]	[3] F6 and F9	182/174	1.326	1.375	1.525	1	Mascot
1374.7151	1374.6681	-0.047	-34	146	153	YINEQYEK	30	97.33	(N-term)_iTRAQ[0], Lysine(K)_iTRAQ[8]	[7] F5 120912	149/141	0.857	0.902	1.038	1	Mascot
1496.8682	1496.8013	-0.0669	-45	108	119	AIGHVIEEGGVK	78	100	(N-term)_iTRAQ[0], Lysine(K)_iTRAQ[12]	[6] F8 110912	210/202	0.590	0.654	0.727	1	Mascot
1506.8889	1506.8123	-0.0766	-51	154	163	FLKEEVNIAR	29	96.684	(N-term)_iTRAQ[0], Lysine(K)_iTRAQ[3]	[6] F8 110912	266/258	0.668	0.774	0.963	1	Mascot
1705.8418	1705.7975	-0.0443	-26	315	326	EVTHNIHYETYR	39	99.691	(N-term)_iTRAQ[0]	[2] F12 040912	173/165	1.131	0.824	1.010	1	Mascot

359	sodium/calcium exchanger 2 precursor [Rattus norvegicus]	gi|17530967	106566.6	4	189	0.894	0.957	0.986	0.216	0.271	0.098	4	4	4	100

Peptide Information

Calc. Mass	Obsrv. Mass	± da	± ppm	Start Seq.	End Seq.	Sequence	Ion Score	C. I. %	Modification	Plate [#] Name	Gel Idx/Pos [4700 Sample Name]	iTRAQ Ratio 115/114*	iTRAQ Ratio 116/114*	iTRAQ Ratio 117/114*	Rank	Result Type
1427.7152	1427.6635	-0.0517	-36	595	603	IVDDEEYEK	55	99.993	(N-term)_iTRAQ[0], Lysine(K)_iTRAQ[9]	[7] F5 120912	146/138	0.910	0.994	0.982	1	Mascot
1611.9091	1611.9067	-0.0024	-1	680	692	TNLALVIGTHSWR	43	99.865	(N-term)_iTRAQ[0]	[4] F7 and F10+11	360/352	1.263	1.416	1.077	1	Mascot
1699.9126	1699.9919	0.0793	47	595	604	IVDDEEYEKK	45	99.925	(N-term)_iTRAQ[0], Lysine(K)_iTRAQ[9,10]	[4] F7 and F10+11	1058/1050	0.763	0.790	1.056	1	Mascot
2023.0898	2023.0122	-0.0776	-38	605	618	DNFFIELGQPQWLK	46	99.942	(N-term)_iTRAQ[0], Lysine(K)_iTRAQ[14]	[5] F4	377/369	0.727	0.754	0.846	1	Mascot

360	Similar to 14-3-3 protein sigma [Rattus norvegicus]	gi|51260816	31414.2	4	189	1.340	1.292	1.082	0.304	0.492	0.234	5	5	5	100

Protein Group

hypothetical protein LOC298795 [Rattus norvegicus]	gi|62177108	30682.8

Peptide Information

Calc. Mass	Obsrv. Mass	± da	± ppm	Start Seq.	End Seq.	Sequence	Ion Score	C. I. %	Modification	Plate [#] Name	Gel Idx/Pos [4700 Sample Name]	iTRAQ Ratio 115/114*	iTRAQ Ratio 116/114*	iTRAQ Ratio 117/114*	Rank	Result Type
957.6019	957.6076	0.0057	6	125	129	VFYLK	34	98.922	(N-term)_iTRAQ[0], Lysine(K)_iTRAQ[5]	[3] F6 and F9	262/254	1.285	1.183	1.199	1	Mascot
1191.7195	1191.6932	-0.0263	-22	68	75	VLSSIEQK	55	99.992	(N-term)_iTRAQ[0], Lysine(K)_iTRAQ[8]	[7] F5 120912	159/151	1.339	1.437	1.443	1	Mascot
1195.7296	1195.6782	-0.0514	-43	49	56	NLLSVAYK	53	99.987	(N-term)_iTRAQ[0], Lysine(K)_iTRAQ[8]	[7] F5 120912	233/225	1.100	1.296	1.119	1	Mascot
1333.7634	1333.7305	-0.0329	-25	222	231	DSTLIMQLLR	42	99.831	(N-term)_iTRAQ[0]	[1] F3 030912	466/458	1.966	2.108	0.925	1	Mascot
1333.7634	1333.7313	-0.0321	-24	222	231	DSTLIMQLLR	48	99.963	(N-term)_iTRAQ[0]	[2] F12 040912	472/464	1.159	0.774	0.827	1	Mascot

361	propionyl-CoA carboxylase beta chain, mitochondrial	gi|148747119	62605.4	2	187	0.911	1.014	1.150	0.104	0.171	0.078	2	2	2	100

precursor [Rattus norvegicus]

Peptide Information

Calc. Mass	Obsrv. Mass	± da	± ppm	Start Seq.	End Seq.	Sequence	Ion Score	C. I. %	Modification	Plate [#] Name	Gel Idx/Pos [4700 Sample Name]	iTRAQ Ratio 115/114*	iTRAQ Ratio 116/114*	iTRAQ Ratio 117/114*	Rank	Result Type
1961.9814	1961.8634	-0.118	-60	477	491	GHQDVEAAQAEYVEK	95	100	(N-term)_iTRAQ[0], Lysine(K)_iTRAQ[15]	[6] F8 110912	171/163	1.015	0.867	1.227	1	Mascot
1964.0182	1963.9117	-0.1065	-54	235	250	SVTNEDVTQEQLGGAK	92	100	(N-term)_iTRAQ[0], Lysine(K)_iTRAQ[16]	[5] F4	112/104	0.817	1.185	1.077	1	Mascot

362	RecName: Full=Myosin regulatory light chain 12B; AltName: Full=Myosin RLC-B; AltName: Full=Myosin r	gi|1170970	22177.2	4	187	1.628	1.359	1.174	0.907	0.709	0.976	4	4	4	100

Protein Group

myosin regulatory light chain - rat	gi|2119367	22206.2
myosin regulatory light chain 12B [Homo sapiens]	gi|15809016	22118.1
myosin:SUBUNIT=regulatory light chain	gi|228542	21987.1

Peptide Information

Calc. Mass	Obsrv. Mass	± da	± ppm	Start Seq.	End Seq.	Sequence	Ion Score	C. I. %	Modification	Plate [#] Name	Gel Idx/Pos [4700 Sample Name]	iTRAQ Ratio 115/114*	iTRAQ Ratio 116/114*	iTRAQ Ratio 117/114*	Rank	Result Type
1381.6654	1381.5959	-0.0695	-50	36	45	EAFNMIDQNR	45	99.93	(N-term)_iTRAQ[0]	[5] F4	153/145	0.990	0.902	0.742	1	Mascot
1404.7032	1404.6366	-0.0666	-47	152	161	GNFNYIEFTR	62	99.998	(N-term)_iTRAQ[0]	[5] F4	222/214	1.743	0.955	1.050	1	Mascot
1559.735	1559.6698	-0.0652	-42	134	144	FTDEEVDELYR	30	97.753	(N-term)_iTRAQ[0]	[1] F3 030912	286/278	3.226	2.547	3.256	1	Mascot
2436.2815	2436.3572	0.0757	31	46	63	DGFIDKEDLHDMLASLGK	49	99.972	(N-term)_iTRAQ[0], Lysine(K)_iTRAQ[6,18]	[4] F7 and F10+11	1305/1297	1.262	1.555	0.750	1	Mascot

363	synaptophysin [Rattus norvegicus]	gi|6981622	35536.5	4	186	0.587	0.911	1.019	0.181	0.330	0.174	5	5	5	100

Peptide Information

Calc. Mass	Obsrv. Mass	± da	± ppm	Start Seq.	End Seq.	Sequence	Ion Score	C. I. %	Modification	Plate [#] Name	Gel Idx/Pos [4700 Sample Name]	iTRAQ Ratio 115/114*	iTRAQ Ratio 116/114*	iTRAQ Ratio 117/114*	Rank	Result Type
1309.6483	1309.5748	-0.0735	-56	220	229	ETGWAAPFMR	47	99.955	(N-term)_iTRAQ[0]	[5] F4	242/234	0.596	1.009	0.899	1	Mascot
1419.7764	1419.7234	-0.053	-37	164	173	MATDPENIIK	27	95.005	(N-term)_iTRAQ[0], Lysine(K)_iTRAQ[10]	[7] F5 120912	195/187	0.650	1.000	1.119	1	Mascot
1677.8866	1677.849	-0.0376	-22	1	14	MDVVNQLVAGGQFR	41	99.792	(N-term)_iTRAQ[0]	[1] F3 030912	363/355	0.849	1.033	1.156	1	Mascot
1840.9316	1840.8728	-0.0588	-32	71	83	LHQVYFDAPSCVK	59	99.997	(N-term)_iTRAQ[0], Lysine(K)_iTRAQ[13], MMTS (C)[11]	[6] F8 110912	367/359	0.573	1.207	1.187	1	Mascot
1840.9316	1840.9318	0.0002	0	71	83	LHQVYFDAPSCVK	71	100	(N-term)_iTRAQ[0], Lysine(K)_iTRAQ[13], MMTS (C)[11]	[4] F7 and F10+11	326/318	0.370	0.499	0.797	1	Mascot

364	cytosol aminopeptidase [Rattus norvegicus]	gi|58865398	61192	5	186	1.534	1.016	1.008	0.399	0.609	0.573	5	5	5	100

Peptide Information

Calc. Mass	Obsrv. Mass	± da	± ppm	Start Seq.	End Seq.	Sequence	Ion Score	C. I. %	Modification	Plate [#] Name	Gel Idx/Pos [4700 Sample Name]	iTRAQ Ratio 115/114*	iTRAQ Ratio 116/114*	iTRAQ Ratio 117/114*	Rank	Result Type
1123.6609	1123.6116	-0.0493	-44	215	221	FAEVIEK	28	96.237	(N-term)_iTRAQ[0], Lysine(K)_iTRAQ[7]	[7] F5 120912	193/185	1.171	0.903	0.880	1	Mascot
1376.777	1376.7115	-0.0655	-48	189	200	GVLFASGQNLAR	28	96.447	(N-term)_iTRAQ[0]	[1] F3 030912	257/249	2.008	2.265	2.287	1	Mascot
2129.0835	2128.9675	-0.116	-54	238	253	SWIEEQEMGSFLSVAK	27	95.338	(N-term)_iTRAQ[0], Lysine(K)_iTRAQ[16]	[5] F4	335/327	1.207	1.044	0.853	1	Mascot
2372.1477	2372.231	0.0833	35	105	122	SAGVDDQENWHEGKENIR	39	99.673	(N-term)_iTRAQ[0], Lysine(K)_iTRAQ[14]	[4] F7 and F10+11	1064/1056	1.516	0.522	0.586	1	Mascot
2459.2063	2459.0593	-0.147	-60	44	61	DKDDDVPQFTSAGENFNK	64	99.999	(N-term)_iTRAQ[0], Lysine(K)_iTRAQ[2,18]	[6] F8 110912	247/239	1.975	0.973	1.035	1	Mascot

365	heterogeneous nuclear ribonucleoprotein K isoform a [Homo sapiens]	gi|14165437	54396.6	4	186	0.993	1.315	1.110	0.505	0.466	0.683	4	4	4	100

Protein Group

heterogeneous nuclear ribonucleoprotein K [Mus musculus]	gi|13384620	54488.7

Peptide Information

Calc. Mass	Obsrv. Mass	± da	± ppm	Start Seq.	End Seq.	Sequence	Ion Score	C. I. %	Modification	Plate [#] Name	Gel Idx/Pos [4700 Sample Name]	iTRAQ Ratio 115/114*	iTRAQ Ratio 116/114*	iTRAQ Ratio 117/114*	Rank	Result Type
1629.0084	1628.9587	-0.0497	-31	208	219	IILDLISESPIK	50	99.974	(N-term)_iTRAQ[0], Lysine(K)_iTRAQ[12]	[1] F3 030912	497/489	0.843	1.230	0.655	1	Mascot
1671.6833	1671.6931	0.0098	6	180	191	LFQECCPHSTDR	36	99.399	(N-term)_iTRAQ[0], MMTS (C)[5,6]	[4] F7 and F10+11	368/360	2.011	2.003	1.818	1	Mascot
1867.9105	1867.9912	0.0807	43	22	34	RPAEDMEEEQAFK	54	99.989	(N-term)_iTRAQ[0], Lysine(K)_iTRAQ[13]	[3] F6 and F9	1153/1145	0.722	0.859	0.722	1	Mascot
1924.9009	1924.7953	-0.1056	-55	70	86	TDYNASVSVPDSSGPER	46	99.941	(N-term)_iTRAQ[0]	[1] F3 030912	184/176	0.794	1.413	1.764	1	Mascot

366	reticulon-1 isoform RTN1-C [Mus musculus]	gi|56090141	25652.1	3	186	5.203	5.394	5.763	108.448	115.766	111.351	5	5	5	100

Protein Group

tropomyosin-related protein, neuronal - rat gi|281046 32654.7

Peptide Information

Calc. Mass	Obsrv. Mass	± da	± ppm	Start Seq.	End Seq.	Sequence	Ion Score	C. I. %	Modification	Plate [#] Name	Gel Idx/Pos [4700 Sample Name]	iTRAQ Ratio 115/114*	iTRAQ Ratio 116/114*	iTRAQ Ratio 117/114*	Rank	Result Type
1408.7709	1408.7255	-0.0454	-32	19	28	SQAIDLLYWR	44	99.899	(N-term)_iTRAQ[0]	[5] F4	307/299	2,408.702	2,676.709	2,305.796	1	Mascot
1502.6821	1502.6479	-0.0342	-23	10	18	MDCVWSNWK	53	99.987	(N-term)_iTRAQ[0], Lysine(K)_iTRAQ[9], MMTS (C)[3]	[3] F6 and F9	396/388	0.891	1.030	1.576	1	Mascot
1502.6821	1502.6831	0.001	1	10	18	MDCVWSNWK	53	99.989	(N-term)_iTRAQ[0], Lysine(K)_iTRAQ[9], MMTS (C)[3]	[4] F7 and F10+11	404/396	1.967	1.645	1.980	1	Mascot
1670.9098	1670.8424	-0.0674	-40	174	186	HQAQVDQYLGLVR	60	99.998	(N-term)_iTRAQ[0]	[6] F8 110912	301/293	1.098	1.225	1.104	1	Mascot
1670.9098	1670.9161	0.0063	4	174	186	HQAQVDQYLGLVR	88	100	(N-term)_iTRAQ[0]	[4] F7 and F10+11	300/292	0.822	0.822	0.801	1	Mascot

367	metabotropic glutamate receptor 5 [Rattus norvegicus]	gi|8393490	138699.1	4	184	0.944	1.002	1.474	0.700	0.437	2.377	4	4	4	100

Protein Group

RecName: Full=Metabotropic glutamate receptor 5; Short=mGluR5; Flags: Precursor gi|1170947 142770.1

Peptide Information

Calc. Mass	Obsrv. Mass	± da	± ppm	Start Seq.	End Seq.	Sequence	Ion Score	C. I. %	Modification	Plate [#] Name	Gel Idx/Pos [4700 Sample Name]	iTRAQ Ratio 115/114*	iTRAQ Ratio 116/114*	iTRAQ Ratio 117/114*	Rank	Result Type
1350.7263	1350.6859	-0.0404	-30	881	889	SVTWAQNEK	51	99.979	(N-term)_iTRAQ[0], Lysine(K)_iTRAQ[9]	[3] F6 and F9	176/168	0.612	1.138	1.151	1	Mascot
1437.73	1437.713	-0.017	-12	335	342	WFDDYYLK	33	98.624	(N-term)_iTRAQ[0], Lysine(K)_iTRAQ[8]	[3] F6 and F9	382/374	2.358	1.712	7.341	1	Mascot
1646.8271	1646.7968	-0.0303	-18	247	258	IYSNAGEQSFDK	55	99.992	(N-term)_iTRAQ[0], Lysine(K)_iTRAQ[12]	[7] F5 120912	162/154	0.601	0.703	0.580	1	Mascot
2015.1661	2015.0449	-0.1212	-60	908	921	ENPNQTAVIKPFPK	46	99.938	(N-term)_iTRAQ[0], Lysine(K)_iTRAQ[10, 14]	[6] F8 110912	221/213	0.918	0.736	0.964	1	Mascot

368	lanC-like protein 2 [Rattus norvegicus]	gi|62079109	55568.3	4	183	1.226	1.732	1.545	0.413	0.300	0.433	4	4	4	100

Peptide Information

Calc. Mass	Obsrv. Mass	± da	± ppm	Start Seq.	End Seq.	Sequence	Ion Score	C. I. %	Modification	Plate [#] Name	Gel Idx/Pos [4700 Sample Name]	iTRAQ Ratio 115/114*	iTRAQ Ratio 116/114*	iTRAQ Ratio 117/114*	Rank	Result Type
1573.8961	1573.9644	0.0683	43	334	342	AYQVFKEEK	30	97.444	(N-term)_iTRAQ[0], Lysine(K)_iTRAQ[6,9]	[4] F7 and F10+11	1096/1088	1.419	1.888	1.674	1	Mascot
1656.7285	1656.6821	-0.0464	-28	346	357	EAMECSDVIWQR	49	99.966	(N-term)_iTRAQ[0], MMTS (C)[5]	[1] F3 030912	344/336	1.838	2.144	2.187	1	Mascot
1685.9148	1685.8589	-0.0559	-33	439	450	FPAFELGFVQKD	35	99.27	(N-term)_iTRAQ[0], Lysine(K)_iTRAQ[11]	[5] F4	317/309	0.917	1.564	1.113	1	Mascot
1977.1426	1977.2903	0.1477	75	81	93	IKDLLQQMEEGLK	69	100	(N-term)_iTRAQ[0], Lysine(K)_iTRAQ[2,13]	[4] F7 and F10+11	1324/1316	0.945	1.423	1.396	1	Mascot

369	GTP-binding protein [Rattus norvegicus]	gi|5852192	14328.4	3	183	0.818	1.351	1.204	0.352	0.496	0.580	3	3	3	100

Protein Group

ras-related protein Rab-3B [Rattus norvegicus] gi|13592037 26826.4

Peptide Information

Calc. Mass	Obsrv. Mass	± da	± ppm	Start Seq.	End Seq.	Sequence	Ion Score	C. I. %	Modification	Plate [#] Name	Gel Idx/Pos [4700 Sample Name]	iTRAQ Ratio	iTRAQ Ratio	iTRAQ Ratio	Rank	Result Type

Calc. Mass	Obsrv. Mass	± da	± ppm	Start Seq.	End Seq.	Sequence	Ion Score	C. I. %	Modification	Plate [#] Name	Gel Idx/Pos [4700 Sample Name]	iTRAQ Ratio 115/114*	iTRAQ Ratio 116/114*	iTRAQ Ratio 117/114*	Rank	Result Type
1460.7618	1460.6924	-0.0694	-48	12	22	LQIWDTAGQER	70	100	(N-term)_iTRAQ[0]	[5] F4	197/189	0.762	1.084	0.943	1	Mascot
1996.075	1995.9971	-0.0779	-39	61	75	TYSWDNAQVILVGNK	43	99.865	(N-term)_iTRAQ[0], Lysine(K)_iTRAQ[15]	[5] F4	270/262	0.549	1.082	0.883	1	Mascot
2074.1106	2074.0574	-0.0532	-26	91	106	LLAEQLGFDFFEASAK	70	100	(N-term)_iTRAQ[0], Lysine(K)_iTRAQ[16]	[1] F3 030912	533/525	1.308	2.102	2.096	1	Mascot

370 calcineurin subunit B type 1 [Homo sapiens] gi|4506025 21685.2 3 183 1.035 1.012 0.997 0.115 0.137 0.164 3 3 3 100

Protein Group

calcineurin B [Rattus sp.] gi|286206 28225.7

Peptide Information

Calc. Mass	Obsrv. Mass	± da	± ppm	Start Seq.	End Seq.	Sequence	Ion Score	C. I. %	Modification	Plate [#] Name	Gel Idx/Pos [4700 Sample Name]	iTRAQ Ratio 115/114*	iTRAQ Ratio 116/114*	iTRAQ Ratio 117/114*	Rank	Result Type
1194.6584	1194.6083	-0.0501	-42	118	125	MMVGNNLK	53	99.988	(N-term)_iTRAQ[0], Lysine(K)_iTRAQ[8]	[3] F6 and F9	198/190	0.954	0.992	1.233	1	Mascot
1475.8315	1475.7544	-0.0771	-52	126	135	DTQLQQIVDK	66	99.999	(N-term)_iTRAQ[0], Lysine(K)_iTRAQ[10]	[5] F4	155/147	0.968	0.875	0.922	1	Mascot
1657.9047	1657.8152	-0.0895	-54	74	85	EFIEGVSQFSVK	63	99.999	(N-term)_iTRAQ[0], Lysine(K)_iTRAQ[12]	[5] F4	244/236	1.202	1.193	0.872	1	Mascot

371 phosphacan (3F8 proteoglycan) [Rattus norvegicus] gi|461372 185648.6 3 182 1.266 1.219 1.552 0.347 0.358 0.778 3 3 3 100

Protein Group

RecName: Full=Receptor-type tyrosine-protein phosphatase zeta; Short=R-PTP-zeta; AltName: Full=3F8 gi|3183128 271325.6

Peptide Information

Calc. Mass	Obsrv. Mass	± da	± ppm	Start Seq.	End Seq.	Sequence	Ion Score	C. I. %	Modification	Plate [#] Name	Gel Idx/Pos [4700 Sample Name]	iTRAQ Ratio 115/114*	iTRAQ Ratio 116/114*	iTRAQ Ratio 117/114*	Rank	Result Type
2011.0746	2011.0066	-0.068	-34	346	360	FAVLYQPLEGNDQTK	64	99.999	(N-term)_iTRAQ[0], Lysine(K)_iTRAQ[15]	[1] F3 030912	327/319	1.736	1.754	2.725	1	Mascot
2328.2656	2328.1824	-0.0832	-36	61	78	QSPINIDEDLTQVNVNLK	67	100	(N-term)_iTRAQ[0], Lysine(K)_iTRAQ[18]	[1] F3 030912	371/363	0.964	1.023	1.296	1	Mascot
2368.3247	2368.2041	-0.1206	-51	31	47	KLVEEIGWSYTGALNQK	51	99.979	(N-term)_iTRAQ[0], Lysine(K)_iTRAQ[1,17]	[6] F8 110912	373/365	1.214	1.008	1.059	1	Mascot

372 leucine-rich PPR motif-containing protein, mitochondrial precursor [Rattus norvegicus] gi|56605990 172407.7 3 181 0.743 1.061 0.931 0.460 0.233 0.380 3 3 3 100

Peptide Information

Calc. Mass	Obsrv. Mass	± da	± ppm	Start Seq.	End Seq.	Sequence	Ion Score	C. I. %	Modification	Plate [#] Name	Gel Idx/Pos [4700 Sample Name]	iTRAQ Ratio 115/114*	iTRAQ Ratio 116/114*	iTRAQ Ratio 117/114*	Rank	Result Type
1747.9285	1747.9175	-0.011	-6	424	437	AVMEALRDEGFPIR	38	99.621	(N-term)_iTRAQ[0]	[4] F7 and F10+11	392/384	1.164	0.883	0.674	1	Mascot
1927.9706	1927.8282	-0.1424	-74	1020	1036	SSLSSSSPSAGDTVTEK	100	100	(N-term)_iTRAQ[0], Lysine(K)_iTRAQ[17]	[5] F4	84/76	0.381	0.966	0.803	1	Mascot
1976.111	1975.994	-0.117	-59	1222	1234	KVIEEQMEPALEK	43	99.866	(N-term)_iTRAQ[0], Lysine(K)_iTRAQ[1,13]	[6] F8 110912	240/232	0.925	1.398	1.494	1	Mascot

373 translationally-controlled tumor protein [Mus musculus] gi|6678437 21703.1 3 180 1.200 1.093 1.226 0.211 0.128 0.159 3 3 3 100

Peptide Information

Calc. Mass	Obsrv. Mass	± da	± ppm	Start Seq.	End Seq.	Sequence	Ion Score	C. I. %	Modification	Plate [#] Name	Gel Idx/Pos [4700 Sample Name]	iTRAQ Ratio 115/114*	iTRAQ Ratio 116/114*	iTRAQ Ratio 117/114*	Rank	Result Type
1645.9608	1646.0446	0.0838	51	101	110	GKLEEQKPER	42	99.854	(N-term)_iTRAQ[0], Lysine(K)_iTRAQ[2,7]	[8] F13-15 and F1+2	1296/1288	0.981	1.005	1.119	1	Mascot
1852.0737	1852.1324	0.0587	32	111	123	VKPFMTGAAEQIK	56	99.994	(N-term)_iTRAQ[0], Lysine(K)_iTRAQ[2,13]	[3] F6 and F9	1223/1215	1.207	1.015	1.130	1	Mascot
1983.0321	1983.0317	-0.0004	0	6	19	DLISHDELFSDIYK	82	100	(N-term)_iTRAQ[0], Lysine(K)_iTRAQ[14]	[4] F7 and F10+11	376/368	1.458	1.278	1.456	1	Mascot

374 heterogeneous nuclear ribonucleoprotein H2 [Rattus norvegicus] gi|62078769 51942 3 180 0.955 1.075 0.947 0.072 0.033 0.347 3 3 3 100

Peptide Information

Calc. Mass	Obsrv. Mass	± da	± ppm	Start Seq.	End Seq.	Sequence	Ion Score	C. I. %	Modification	Plate [#] Name	Gel Idx/Pos [4700 Sample Name]	iTRAQ Ratio 115/114*	iTRAQ Ratio 116/114*	iTRAQ Ratio 117/114*	Rank	Result Type
1236.6821	1236.7222	0.0401	32	317	326	VHIEIGPDGR	29	96.79	(N-term)_iTRAQ[0]	[3] F6 and F9	1170/1162	1.005	1.030	1.229	1	Mascot

Calc. Mass	Obsrv. Mass	± da	± ppm	Start Seq.	End Seq.	Sequence	Ion Score	C. I. %	Modification	Plate [#] Name	Gel Idx/Pos [4700 Sample Name]	iTRAQ Ratio 115/114*	iTRAQ Ratio 116/114*	iTRAQ Ratio 117/114*	Rank	Result Type
1828.8699	1828.7589	-0.111	-61	99	114	HTGPNSPDTANDGFVR	63	99.999	(N-term)_iTRAQ[0]	[6] F8 110912	154/146	0.862	1.105	1.132	1	Mascot
2130.0964	2130.0371	-0.0593	-28	151	167	STGEAFVQFASQEIAEK	88	100	(N-term)_iTRAQ[0], Lysine(K)_iTRAQ[17]	[1] F3 030912	401/393	1.005	1.091	0.610	1	Mascot

375	SH3P7r3 [Rattus norvegicus]	gi|7248379	51526.4	4	179	0.651	0.899	0.983	0.390	0.143	0.272	4	4	4	100

Protein Group

RecName: Full=Drebrin-like protein; AltName: Full=Actin-binding protein 1; Short=Abp1; AltName: Ful	gi|51315733	51884.6
drebrin-like protein [Rattus norvegicus]	gi|13786198	51613.5

Peptide Information

Calc. Mass	Obsrv. Mass	± da	± ppm	Start Seq.	End Seq.	Sequence	Ion Score	C. I. %	Modification	Plate [#] Name	Gel Idx/Pos [4700 Sample Name]	iTRAQ Ratio 115/114*	iTRAQ Ratio 116/114*	iTRAQ Ratio 117/114*	Rank	Result Type
1068.5724	1068.5516	-0.0208	-19	177	182	DNFWAK	29	96.812	(N-term)_iTRAQ[0], Lysine(K)_iTRAQ[6]	[4] F7 and F10+11	221/213	1.134	1.066	1.466	1	Mascot
1764.8538	1764.7689	-0.0849	-48	249	262	QEWESAGQQAPHPR	73	100	(N-term)_iTRAQ[0]	[6] F8 110912	158/150	0.673	0.896	0.787	1	Mascot
1855.8354	1855.7762	-0.0592	-32	119	131	AEEDVEPECIMEK	46	99.935	(N-term)_iTRAQ[0], Lysine(K)_iTRAQ[13], MMTS (C)[9]	[5] F4	204/196	0.757	0.958	0.829	1	Mascot
2007.9869	2007.9126	-0.0743	-37	247	262	SRQEWESAGQQAPHPR	31	98.165	(N-term)_iTRAQ[0]	[2] F12 040912	154/146	0.310	0.713	0.975	1	Mascot

376	mu-crystallin homolog [Rattus norvegicus]	gi|16758840	36120.8	4	179	1.026	1.464	1.528	0.612	0.329	0.490	4	4	4	100

Peptide Information

Calc. Mass	Obsrv. Mass	± da	± ppm	Start Seq.	End Seq.	Sequence	Ion Score	C. I. %	Modification	Plate [#] Name	Gel Idx/Pos [4700 Sample Name]	iTRAQ Ratio 115/114*	iTRAQ Ratio 116/114*	iTRAQ Ratio 117/114*	Rank	Result Type
1591.8975	1591.8364	-0.0611	-38	291	303	SLGMAVEDLVAAK	43	99.869	(N-term)_iTRAQ[0], Lysine(K)_iTRAQ[13]	[5] F4	313/305	0.523	1.288	1.207	1	Mascot
1812.9364	1812.8969	-0.0395	-22	4	18	APAFLSADEVQDHLR	29	97.092	(N-term)_iTRAQ[0]	[3] F6 and F9	319/311	1.763	1.735	1.624	1	Mascot
2146.2368	2146.1875	-0.0493	-23	19	36	SSSLLIPPLEAALANFSK	78	100	(N-term)_iTRAQ[0], Lysine(K)_iTRAQ[18]	[1] F3 030912	540/532	1.398	1.827	2.350	1	Mascot
2230.1675	2230.1882	0.0207	9	57	75	GFLGVMPAYSAAEDALTTK	29	96.819	(N-term)_iTRAQ[0], Lysine(K)_iTRAQ[19]	[8] F13-15 and F1+2	378/370	0.859	1.125	1.184	1	Mascot

377	seryl-tRNA synthetase, cytoplasmic [Rattus norvegicus]	gi|56090265	65593.4	3	178	1.150	1.093	1.028	0.372	0.385	0.252	3	3	3	100

Peptide Information

Calc. Mass	Obsrv. Mass	± da	± ppm	Start Seq.	End Seq.	Sequence	Ion Score	C. I. %	Modification	Plate [#] Name	Gel Idx/Pos [4700 Sample Name]	iTRAQ Ratio 115/114*	iTRAQ Ratio 116/114*	iTRAQ Ratio 117/114*	Rank	Result Type
1851.9487	1851.8418	-0.1069	-58	324	336	IEQFVYSSPHDNK	56	99.994	(N-term)_iTRAQ[0], Lysine(K)_iTRAQ[13]	[6] F8 110912	240/232	0.857	0.902	0.810	1	Mascot
1982.0746	1982.0089	-0.0657	-33	376	390	KLDLEAWFPGSGAFR	48	99.958	(N-term)_iTRAQ[0], Lysine(K)_iTRAQ[1]	[6] F8 110912	469/461	1.059	0.865	0.975	1	Mascot
2474.2371	2474.1558	-0.0813	-33	232	249	EVMQEVAQLSQFDEELYK	75	100	(N-term)_iTRAQ[0], Lysine(K)_iTRAQ[18]	[1] F3 030912	480/472	1.676	1.674	1.375	1	Mascot

378	dipeptidyl aminopeptidase-like protein 6 [Rattus norvegicus]	gi|12408298	105286	4	177	1.013	0.849	0.796	0.202	0.090	0.373	4	4	4	100

Protein Group

BSPL=neural membrane CD26 peptidase-like protein [rats, brain, Peptide, 803 aa]	gi|913778	99055
dipeptidyl aminopeptidase-related protein [Rattus norvegicus]	gi|408716	99263.2

Peptide Information

Calc. Mass	Obsrv. Mass	± da	± ppm	Start Seq.	End Seq.	Sequence	Ion Score	C. I. %	Modification	Plate [#] Name	Gel Idx/Pos [4700 Sample Name]	iTRAQ Ratio 115/114*	iTRAQ Ratio 116/114*	iTRAQ Ratio 117/114*	Rank	Result Type
1307.7358	1307.7467	0.0109	8	236	244	LQYAGWGPK	50	99.974	(N-term)_iTRAQ[0], Lysine(K)_iTRAQ[9]	[3] F6 and F9	262/254	1.266	0.939	1.072	1	Mascot
1693.8319	1693.9314	0.0995	59	294	306	SHIAHWWSPDGTR	42	99.844	(N-term)_iTRAQ[0]	[8] F13-15 and F1+2	1162/1154	0.769	0.772	0.728	1	Mascot
1953.0361	1953.0225	-0.0136	-7	674	687	LGFLEEKDQMEAVR	43	99.868	(N-term)_iTRAQ[0], Lysine(K)_iTRAQ[7]	[4] F7 and F10+11	330/322	1.093	0.763	0.442	1	Mascot
2080.2166	2080.2676	0.051	25	789	803	IHFQHTAELITQLIK	44	99.903	(N-term)_iTRAQ[0], Lysine(K)_iTRAQ[15]	[4] F7 and F10+11	1308/1300	0.988	0.940	1.168	1	Mascot

379	complexin-2 [Homo sapiens]	gi|5729783	18502.8	3	177	0.769	0.617	0.595	0.157	0.135	0.161	4	4	4	100

Peptide Information

Calc. Mass	Obsrv. Mass	± da	± ppm	Start Seq.	End Seq.	Sequence	Ion Score	C. I. %	Modification	Plate [#] Name	Gel Idx/Pos [4700 Sample Name]	iTRAQ Ratio 115/114*	iTRAQ Ratio 116/114*	iTRAQ Ratio 117/114*	Rank	Result Type
1596.8706	1596.7845	-0.0861	-54	123	133	YLPGPLQDMFK	58	99.996	(N-term)_iTRAQ[0], Lysine(K)_iTRAQ[11]	[5] F4	340/332	0.857	0.600	0.741	1	Mascot
1934.0066	1933.9896	-0.017	-9	84	98	AALEQPCEGSLTRPK	59	99.997	(N-term)_iTRAQ[0], Lysine(K)_iTRAQ[15], MMTS (C)[7]	[4] F7 and F10+11	248/240	0.777	0.729	0.659	1	Mascot
1979.0127	1978.8989	-0.1138	-58	19	32	MLGGEEEKDPDAQK	32	98.536	(N-term)_iTRAQ[0], Lysine(K)_iTRAQ[8,14]	[6] F8 110912	155/147	0.567	0.451	0.396	1	Mascot
1979.0127	1979.1145	0.1018	51	19	32	MLGGEEEKDPDAQK	60	99.997	(N-term)_iTRAQ[0], Lysine(K)_iTRAQ[8,14]	[3] F6 and F9	1116/1108	0.925	0.733	0.646	1	Mascot

380	Rap1b [Rattus norvegicus]	gi|595280	23163	3	176	1.253	0.910	0.948	0.078	0.205	0.118	4	4	4	100

Protein Group

ras-related protein Rap-1b precursor [Rattus norvegicus] gi|52138628 23130.1

Peptide Information

Calc. Mass	Obsrv. Mass	± da	± ppm	Start Seq.	End Seq.	Sequence	Ion Score	C. I. %	Modification	Plate [#] Name	Gel Idx/Pos [4700 Sample Name]	iTRAQ Ratio 115/114*	iTRAQ Ratio 116/114*	iTRAQ Ratio 117/114*	Rank	Result Type
1402.6611	1402.5878	-0.0733	-52	32	41	YDPTIEDSYR	55	99.993	(N-term)_iTRAQ[0]	[1] F3 030912	205/197	1.343	1.248	1.026	1	Mascot
1638.8976	1638.8448	-0.0528	-32	152	163	INVNEIFYDLVR	39	99.715	(N-term)_iTRAQ[0]	[5] F4	409/401	1.270	0.773	1.002	1	Mascot
1638.8976	1638.8578	-0.0398	-24	152	163	INVNEIFYDLVR	62	99.998	(N-term)_iTRAQ[0]	[1] F3 030912	534/526	1.271	0.947	1.013	1	Mascot
1804.8589	1804.8196	-0.0393	-22	137	149	QWSNCAFLESSAK	59	99.997	(N-term)_iTRAQ[0], Lysine(K)_iTRAQ[13], MMTS (C)[5]	[7] F5 120912	340/332	1.137	0.751	0.774	1	Mascot

381	Ndufa9 protein [Rattus norvegicus]	gi|60688426	44759.9	4	175	0.752	0.856	0.848	0.257	0.412	0.270	4	4	4	100

Peptide Information

Calc. Mass	Obsrv. Mass	± da	± ppm	Start Seq.	End Seq.	Sequence	Ion Score	C. I. %	Modification	Plate [#] Name	Gel Idx/Pos [4700 Sample Name]	iTRAQ Ratio 115/114*	iTRAQ Ratio 116/114*	iTRAQ Ratio 117/114*	Rank	Result Type
1101.6289	1101.6431	0.0142	13	61	68	YVVNHLGR	37	99.548	(N-term)_iTRAQ[0]	[3] F6 and F9	1136/1128	0.592	0.562	0.630	1	Mascot
1755.8826	1755.8925	0.0099	6	131	143	NFDFEDVFVNIPR	75	100	(N-term)_iTRAQ[0]	[8] F13-15 and F1+2	333/325	0.579	0.800	0.864	1	Mascot
1765.0662	1765.0361	-0.0301	-17	215	227	WFLAVPLVSLGFK	35	99.263	(N-term)_iTRAQ[0], Lysine(K)_iTRAQ[13]	[1] F3 030912	564/556	1.202	1.627	1.313	1	Mascot
1810.9989	1810.9398	-0.0591	-33	297	309	LFGLSPFEPWTTK	28	95.816	(N-term)_iTRAQ[0], Lysine(K)_iTRAQ[13]	[5] F4	377/369	0.774	0.735	0.725	1	Mascot

382	NADH dehydrogenase [ubiquinone] iron-sulfur protein 4, mitochondrial precursor [Rattus norvegicus]	gi|68341995	22079.8	3	174	0.743	0.731	0.828	0.275	0.137	0.172	3	3	3	100

Peptide Information

Calc. Mass	Obsrv. Mass	± da	± ppm	Start Seq.	End Seq.	Sequence	Ion Score	C. I. %	Modification	Plate [#] Name	Gel Idx/Pos [4700 Sample Name]	iTRAQ Ratio 115/114*	iTRAQ Ratio 116/114*	iTRAQ Ratio 117/114*	Rank	Result Type
1267.678	1267.6534	-0.0246	-19	132	140	EDAVAFAEK	47	99.953	(N-term)_iTRAQ[0], Lysine(K)_iTRAQ[9]	[7] F5 120912	159/151	0.509	0.591	0.666	1	Mascot
1461.7372	1461.6865	-0.0507	-35	159	168	SYGANFSWNK	71	100	(N-term)_iTRAQ[0], Lysine(K)_iTRAQ[10]	[3] F6 and F9	255/247	0.735	0.900	1.055	1	Mascot
1936.1001	1936.0448	-0.0553	-29	59	73	LDVTPLTGVPEEHIK	56	99.994	(N-term)_iTRAQ[0], Lysine(K)_iTRAQ[15]	[3] F6 and F9	314/306	1.099	0.733	0.809	1	Mascot

383	fumarate hydratase, mitochondrial precursor [Rattus norvegicus]	gi|158186722	59415.3	3	174	0.671	1.064	0.842	0.355	0.237	0.085	3	3	3	100

Protein Group

RecName: Full=Fumarate hydratase, mitochondrial; Short=Fumarase; Flags: Precursor gi|120605 59558.5

Peptide Information

Calc. Mass	Obsrv. Mass	± da	± ppm	Start Seq.	End Seq.	Sequence	Ion Score	C. I. %	Modification	Plate [#] Name	Gel Idx/Pos [4700 Sample Name]	iTRAQ Ratio 115/114*	iTRAQ Ratio 116/114*	iTRAQ Ratio 117/114*	Rank	Result Type
1502.7988	1502.7561	-0.0427	-28	49	58	IEYDTFGELK	36	99.437	(N-term)_iTRAQ[0], Lysine(K)_iTRAQ[10]	[7] F5 120912	278/270	1.037	1.413	0.830	1	Mascot
1792.9326	1792.823	-0.1096	-61	99	112	AAAEVNQEYGLDPK	73	100	(N-term)_iTRAQ[0], Lysine(K)_iTRAQ[14]	[5] F4	131/123	0.771	0.904	0.755	1	Mascot
3340.6392	3340.5352	-0.104	-31	231	258	THTQDAVPLTLGQEFSGYVQQVQYAMER	65	99.999	(N-term)_iTRAQ[0]	[1] F3 030912	534/526	0.377	0.943	0.953	1	Mascot

384	neuron-specific protein PEP-19 [Rattus norvegicus]	gi|23477207	12589.5	3	174	1.012	0.906	0.761	0.493	0.889	0.576	5	5	5	100

Protein Group

Purkinje cell protein 4 [Mus musculus]	gi|6679227	8100.2

Peptide Information

Calc. Mass	Obsrv. Mass	± da	± ppm	Start Seq.	End Seq.	Sequence	Ion Score	C. I. %	Modification	Plate [#] Name	Gel Idx/Pos [4700 Sample Name]	iTRAQ Ratio 115/114*	iTRAQ Ratio 116/114*	iTRAQ Ratio 117/114*	Rank	Result Type
1234.7028	1234.6211	-0.0817	-66	74	83	AAVAIQSQFR	70	100	(N-term)_iTRAQ[0]	[5] F4	145/137	0.904	0.823	0.741	1	Mascot
2067.9302	2067.9189	-0.0113	-5	58	73	VQEEFDIDMDAPETER	31	98.19	(N-term)_iTRAQ[0]	[8] F13-15 and F1+2	474/466	1.176	0.849	0.924	1	Mascot
2067.9302	2067.9656	0.0354	17	58	73	VQEEFDIDMDAPETER	34	98.949	(N-term)_iTRAQ[0]	[8] F13-15 and F1+2	621/613	0.560	0.376	0.293	1	Mascot
2067.9302	2067.9666	0.0364	18	58	73	VQEEFDIDMDAPETER	40	99.754	(N-term)_iTRAQ[0]	[8] F13-15 and F1+2	628/620	1.900	3.100	1.690	1	Mascot
2340.1274	2340.1138	-0.0136	-6	57	73	KVQEEFDIDMDAPETER	64	99.999	(N-term)_iTRAQ[0], Lysine(K)_iTRAQ[1]	[4] F7 and F10+11	284/276	0.937	0.752	0.752	1	Mascot

385	aldose reductase [Rattus norvegicus]	gi|6978491	39888.8	4	173	1.051	0.807	0.892	0.293	0.223	0.249	5	5	5	100		

Peptide Information

Calc. Mass	Obsrv. Mass	± da	± ppm	Start Seq.	End Seq.	Sequence	Ion Score	C. I. %	Modification	Plate [#] Name	Gel Idx/Pos [4700 Sample Name]	iTRAQ Ratio 115/114*	iTRAQ Ratio 116/114*	iTRAQ Ratio 117/114*	Rank	Result Type
1391.7966	1391.78	-0.0166	-12	13	22	MPTLGLGTWK	29	96.884	(N-term)_iTRAQ[0], Lysine(K)_iTRAQ[10]	[7] F5 120912	341/333	0.676	0.843	1.140	1	Mascot
1530.795	1530.8546	0.0596	39	307	316	HKDYPFHAEV	42	99.829	(N-term)_iTRAQ[0], Lysine(K)_iTRAQ[2]	[8] F13-15 and F1+2	1201/1193	1.105	1.250	1.101	1	Mascot
1530.795	1530.9	0.105	69	307	316	HKDYPFHAEV	29	96.782	(N-term)_iTRAQ[0], Lysine(K)_iTRAQ[2]	[8] F13-15 and F1+2	1534/1526	1.251	0.675	0.687	1	Mascot
1701.9408	1701.9017	-0.0391	-23	156	169	AIGVSNFNPLQIER	51	99.982	(N-term)_iTRAQ[0]	[1] F3 030912	359/351	1.374	0.619	1.023	1	Mascot
1781.8541	1781.9338	0.0797	45	42	53	HIDCAQVYQNEK	51	99.98	(N-term)_iTRAQ[0], Lysine(K)_iTRAQ[12], MMTS (C)[4]	[4] F7 and F10+11	1124/1116	0.997	0.780	0.639	1	Mascot

386	T-complex protein 1 subunit gamma [Rattus norvegicus]	gi|40018616	66255.9	3	172	0.884	0.915	0.911	0.105	0.039	0.154	3	3	3	100

Peptide Information

Calc. Mass	Obsrv. Mass	± da	± ppm	Start Seq.	End Seq.	Sequence	Ion Score	C. I. %	Modification	Plate [#] Name	Gel Idx/Pos [4700 Sample Name]	iTRAQ Ratio 115/114*	iTRAQ Ratio 116/114*	iTRAQ Ratio 117/114*	Rank	Result Type
1408.8158	1408.7056	-0.1102	-78	69	78	EIQVQHPAAK	75	100	(N-term)_iTRAQ[0], Lysine(K)_iTRAQ[10]	[6] F8 110912	125/117	1.007	0.965	0.775	1	Mascot
1481.7332	1481.6462	-0.087	-59	428	438	AMTGVEQWPYR	31	98.228	(N-term)_iTRAQ[0]	[5] F4	193/185	0.897	0.909	1.126	1	Mascot
1542.9141	1542.8298	-0.0843	-55	492	502	ELGIWEPLAVK	66	99.999	(N-term)_iTRAQ[0], Lysine(K)_iTRAQ[11]	[5] F4	302/294	0.766	0.872	0.867	1	Mascot

387	ras-related protein Rab-35 isoform 1 [Homo sapiens]	gi|5803135	25978.6	3	172	0.848	1.020	0.950	0.159	0.375	0.432	3	3	3	100

Peptide Information

Calc. Mass	Obsrv. Mass	± da	± ppm	Start Seq.	End Seq.	Sequence	Ion Score	C. I. %	Modification	Plate [#] Name	Gel Idx/Pos [4700 Sample Name]	iTRAQ Ratio 115/114*	iTRAQ Ratio 116/114*	iTRAQ Ratio 117/114*	Rank	Result Type
1359.8457	1359.778	-0.0677	-50	11	21	LLIIGDSGVGK	58	99.996	(N-term)_iTRAQ[0], Lysine(K)_iTRAQ[11]	[5] F4	229/221	0.740	1.446	1.508	1	Mascot
1460.7618	1460.6924	-0.0694	-48	59	69	LQIWDTAGQER	70	100	(N-term)_iTRAQ[0]	[5] F4	197/189	0.762	1.084	0.943	1	Mascot
1980.827	1980.8373	0.0103	5	102	115	WLHEINQNCDDVCR	45	99.915	(N-term)_iTRAQ[0], MMTS (C)[9,13]	[4] F7 and F10+11	442/434	1.080	0.676	0.602	1	Mascot

388	cytochrome c oxidase subunit Vb [Rattus norvegicus]	gi|473729	14855.3	4	171	1.112	1.106	1.325	0.346	0.373	1.059	4	4	4	100

Protein Group

cytochrome c oxidase subunit 5B, mitochondrial precursor [Rattus norvegicus]	gi|16758362	14856.3
cytochrome c oxidase subunit VIa (AA 1 - 118) [Rattus norvegicus]	gi|55992	13631.6

Peptide Information

Calc. Mass	Obsrv. Mass	± da	± ppm	Start Seq.	End Seq.	Sequence	Ion Score	C. I. %	Modification	Plate [#] Name	Gel Idx/Pos [4700 Sample Name]	iTRAQ Ratio 115/114*	iTRAQ Ratio 116/114*	iTRAQ Ratio 117/114*	Rank	Result Type
1146.6101	1146.5508	-0.0593	-52	122	129	LVPYQMVH	28	96.245	(N-term)_iTRAQ[0], Oxidation (M)[6]	[1] F3 030912	205/197	1.778	1.716	3.505	1	Mascot
1532.8392	1532.7677	-0.0715	-47	58	68	GLDPYNMLPPK	38	99.575	(N-term)_iTRAQ[0], Lysine(K)_iTRAQ[11]	[5] F4	232/224	0.970	0.767	0.945	1	Mascot
1586.8635	1586.7567	-0.1068	-67	75	86	EDPNLVPSVSNK	45	99.918	(N-term)_iTRAQ[0],	[5] F4	128/120	0.944	1.003	0.757	1	Mascot

Calc. Mass	Obsrv. Mass	± da	± ppm	Start Seq.	End Seq.	Sequence	Ion Score	C. I. %	Modification	Plate [#] Name	Gel Idx/Pos [4700 Sample Name]	iTRAQ Ratio 115/114*	iTRAQ Ratio 116/114*	iTRAQ Ratio 117/114*	Rank	Result Type
									Lysine(K)_iTRAQ[12]							
1742.9646	1742.8527	-0.1119	-64	75	87	EDPNLVPSVSNKR	61	99.998	(N-term)_iTRAQ[0], Lysine(K)_iTRAQ[12]	[6] F8 110912	186/178	0.939	1.134	1.228	1	Mascot

389	guanine nucleotide-binding protein G(I)/G(S)/G(O) subunit gamma-7 [Rattus norvegicus]	gi|13162316	8750.7	3	171	0.937	0.822	0.893	0.045	0.144	0.128	3	3	3	100	

Protein Group

RecName: Full=Guanine nucleotide-binding protein G(I)/G(S)/G(O) subunit gamma-7; Flags: Precursor	gi|52788203	8619.6

Peptide Information

Calc. Mass	Obsrv. Mass	± da	± ppm	Start Seq.	End Seq.	Sequence	Ion Score	C. I. %	Modification	Plate [#] Name	Gel Idx/Pos [4700 Sample Name]	iTRAQ Ratio 115/114*	iTRAQ Ratio 116/114*	iTRAQ Ratio 117/114*	Rank	Result Type
1801.7772	1801.7736	-0.0036	-2	32	45	ASSELMSYCEQHAR	62	99.999	(N-term)_iTRAQ[0], MMTS (C)[9]	[4] F7 and F10+11	353/345	0.968	1.016	0.909	1	Mascot
1985.0953	1985.022	-0.0733	-37	46	61	NDPLLVGVPASENPFK	70	100	(N-term)_iTRAQ[0], Lysine(K)_iTRAQ[16]	[1] F3 030912	370/362	0.876	0.687	0.752	1	Mascot
2372.3196	2372.324	0.0044	2	46	63	NDPLLVGVPASENPFKD K	39	99.704	(N-term)_iTRAQ[0], Lysine(K)_iTRAQ[16, 18]	[4] F7 and F10+11	356/348	0.969	0.795	1.043	1	Mascot

390	clathryn light chain (LCA2) [Rattus norvegicus]	gi|203276	27483.7	4	170	1.235	0.847	1.289	0.170	0.358	0.200	4	4	4	100	

Protein Group

clathrin light chain A [Rattus norvegicus]	gi|14010873	28929.3

Peptide Information

Calc. Mass	Obsrv. Mass	± da	± ppm	Start Seq.	End Seq.	Sequence	Ion Score	C. I. %	Modification	Plate [#] Name	Gel Idx/Pos [4700 Sample Name]	iTRAQ Ratio 115/114*	iTRAQ Ratio 116/114*	iTRAQ Ratio 117/114*	Rank	Result Type
1170.5878	1170.5598	-0.028	-24	205	211	LCDFNPK	35	99.192	(N-term)_iTRAQ[0], Lysine(K)_iTRAQ[7], MMTS (C)[2]	[3] F6 and F9	304/296	1.324	0.662	1.393	1	Mascot
1230.6616	1230.6093	-0.0523	-42	163	170	VADEAFYK	55	99.992	(N-term)_iTRAQ[0], Lysine(K)_iTRAQ[8]	[3] F6 and F9	207/199	1.024	1.116	1.064	1	Mascot
1239.613	1239.5315	-0.0815	-66	142	149	ELEEWYAR	36	99.405	(N-term)_iTRAQ[0]	[5] F4	186/178	1.447	1.276	1.199	1	Mascot
1474.8475	1474.8973	0.0498	34	103	112	LQSEPESIRK	44	99.913	(N-term)_iTRAQ[0], Lysine(K)_iTRAQ[10]	[4] F7 and F10+11	1048/1040	1.186	0.547	1.553	1	Mascot

391	3-hydroxyisobutyrate dehydrogenase [Rattus norvegicus]	gi|556389	39834.2	2	170	0.983	1.109	1.070	0.253	0.254	0.277	3	3	3	100	

Protein Group

3-hydroxyisobutyrate dehydrogenase, mitochondrial precursor [Rattus norvegicus]	gi|83977457	38771.8

Peptide Information

Calc. Mass	Obsrv. Mass	± da	± ppm	Start Seq.	End Seq.	Sequence	Ion Score	C. I. %	Modification	Plate [#] Name	Gel Idx/Pos [4700 Sample Name]	iTRAQ Ratio 115/114*	iTRAQ Ratio 116/114*	iTRAQ Ratio 117/114*	Rank	Result Type
1677.921	1678.0173	0.0963	57	331	341	KDFSSVFQYLR	62	99.998	(N-term)_iTRAQ[0], Lysine(K)_iTRAQ[1]	[4] F7 and F10+11	1273/1265	1.264	1.236	1.256	1	Mascot
1875.9546	1875.8491	-0.1055	-56	90	105	EAGEQVASSPADVAEK	108	100	(N-term)_iTRAQ[0], Lysine(K)_iTRAQ[16]	[5] F4	101/93	1.035	1.329	1.262	1	Mascot
1875.9546	1875.8557	-0.0989	-53	90	105	EAGEQVASSPADVAEK	52	99.984	(N-term)_iTRAQ[0], Lysine(K)_iTRAQ[16]	[1] F3 030912	164/156	0.727	0.831	0.773	1	Mascot

392	Chain A, Solution Structure Of Oxidized Rat Microsomal Cytochrome B5 In The Presence Of 2 M Guanidi	gi|6980893	12369.5	3	169	0.987	0.744	0.782	0.229	0.059	0.224	3	3	3	100	

Protein Group

Chain A, Solution Structure Of The Water-Soluble Fragment Of Rat Hepatic Apocytochrome B5	gi|14277784	12788.7
cytochrome b5	gi|224985	11889.2
cytochrome b5 [Rattus norvegicus]	gi|11560046	16930.7
cytochrome b5 [Rattus norvegicus]	gi|2257955	12984.8

Peptide Information

Calc. Mass	Obsrv. Mass	± da	± ppm	Start Seq.	End Seq.	Sequence	Ion Score	C. I. %	Modification	Plate [#] Name	Gel Idx/Pos [4700 Sample Name]	iTRAQ Ratio	iTRAQ Ratio	iTRAQ Ratio	Rank	Result Type

												iTRAQ Ratio 115/114*	iTRAQ Ratio 116/114*	iTRAQ Ratio 117/114*		
1572.8142	1572.7936	-0.0206	-13	73	84	TYIIGELHPDDR	28	96.528	(N-term)_iTRAQ[0]	[3] F6 and F9	263/255	0.742	0.667	0.707	1	Mascot
1655.8513	1655.804	-0.0473	-29	35	47	FLEEHPGGEEVLR	48	99.958	(N-term)_iTRAQ[0]	[3] F6 and F9	266/258	1.214	0.785	0.613	1	Mascot
2350.0303	2349.9524	-0.0779	-33	48	68	EQAGGDATENFEDVGHS TDAR	93	100	(N-term)_iTRAQ[0]	[7] F5 120912	150/142	1.069	0.785	1.106	1	Mascot

393	glycerol-3-phosphate dehydrogenase [NAD+], cytoplasmic [Rattus norvegicus]	gi|57527919	41582.6	3	165	1.993	1.283	0.997	0.316	0.353	0.150	3	3	3	100

Protein Group

glycerol 3-phosphate dehydrogenase [Rattus norvegicus]	gi|2317252	41481.7

Peptide Information

Calc. Mass	Obsrv. Mass	± da	± ppm	Start Seq.	End Seq.	Sequence	Ion Score	C. I. %	Modification	Plate [#] Name	Gel Idx/Pos [4700 Sample Name]	iTRAQ Ratio 115/114*	iTRAQ Ratio 116/114*	iTRAQ Ratio 117/114*	Rank	Result Type
1392.8209	1392.9139	0.093	67	305	313	ELHSILQHK	45	99.928	(N-term)_iTRAQ[0], Lysine(K)_iTRAQ[9]	[8] F13-15 and F1+2	1534/1526	1.742	1.326	0.853	1	Mascot
2030.2062	2030.2147	0.0085	4	314	327	GLVDKFPLFTAVYK	80	100	(N-term)_iTRAQ[0], Lysine(K)_iTRAQ[5,14]	[4] F7 and F10+11	454/446	1.857	0.938	0.969	1	Mascot
2099.2195	2099.3081	0.0886	42	49	62	KLTEIINTQHENVK	40	99.751	(N-term)_iTRAQ[0], Lysine(K)_iTRAQ[1,14]	[4] F7 and F10+11	1130/1122	2.446	1.697	1.198	1	Mascot

394	CDC42 protein [Rattus norvegicus]	gi|24637541	24140.8	3	165	0.889	0.750	1.039	0.365	0.444	0.220	4	4	4	100

Protein Group

cell division control protein 42 homolog isoform 1 [Homo sapiens]	gi|4757952	24114.8

Peptide Information

Calc. Mass	Obsrv. Mass	± da	± ppm	Start Seq.	End Seq.	Sequence	Ion Score	C. I. %	Modification	Plate [#] Name	Gel Idx/Pos [4700 Sample Name]	iTRAQ Ratio 115/114*	iTRAQ Ratio 116/114*	iTRAQ Ratio 117/114*	Rank	Result Type
1673.981	1673.9935	0.0125	7	134	144	QKPITPETAEK	45	99.924	(N-term)_iTRAQ[0], Lysine(K)_iTRAQ[2,11]	[3] F6 and F9	1092/1084	1.043	0.717	0.824	1	Mascot
1680.8582	1680.8195	-0.0387	-23	97	107	WVPEITHHCPK	38	99.642	(N-term)_iTRAQ[0], Lysine(K)_iTRAQ[11], MMTS (C)[9]	[2] F12 040912	320/312	1.083	1.056	1.239	1	Mascot
1680.8582	1680.8621	0.0039	2	97	107	WVPEITHHCPK	35	99.161	(N-term)_iTRAQ[0], Lysine(K)_iTRAQ[11], MMTS (C)[9]	[2] F12 040912	338/330	0.491	0.358	0.898	1	Mascot
2141.1741	2141.1194	-0.0547	-26	167	183	NVFDEAILAALEPPEPK	81	100	(N-term)_iTRAQ[0], Lysine(K)_iTRAQ[17]	[1] F3 030912	521/513	1.126	1.170	1.273	1	Mascot

395	rabaptin [Rattus norvegicus]	gi|1575770	109878.5	3	165	1.006	1.309	1.049	0.305	1.173	0.226	3	3	3	100

Protein Group

rab GTPase-binding effector protein 1 [Rattus norvegicus]	gi|9506845	109919.6

Peptide Information

Calc. Mass	Obsrv. Mass	± da	± ppm	Start Seq.	End Seq.	Sequence	Ion Score	C. I. %	Modification	Plate [#] Name	Gel Idx/Pos [4700 Sample Name]	iTRAQ Ratio 115/114*	iTRAQ Ratio 116/114*	iTRAQ Ratio 117/114*	Rank	Result Type
1778.9282	1778.8444	-0.0838	-47	31	42	AQQQLEQEFNQK	81	100	(N-term)_iTRAQ[0], Lysine(K)_iTRAQ[12]	[7] F5 120912	188/180	1.105	0.665	0.797	1	Mascot
1824.8838	1824.9386	0.0548	30	258	270	ELHEVCHLLEQER	28	95.883	(N-term)_iTRAQ[0], MMTS (C)[6]	[4] F7 and F10+11	1275/1267	0.702	1.093	1.221	1	Mascot
2394.2761	2394.207	-0.0691	-29	603	621	AQASEVLLEELQQSFSQ AK	56	99.994	(N-term)_iTRAQ[0], Lysine(K)_iTRAQ[19]	[1] F3 030912	497/489	1.315	3.087	1.187	1	Mascot

396	thy-1 membrane glycoprotein preproprotein [Rattus norvegicus]	gi|6981654	19641.5	4	164	0.945	0.811	0.867	0.152	0.189	0.123	6	6	6	100

Protein Group

antigen thy1	gi|223595	14994.9
thy-1 glycoprotein [Rattus norvegicus]	gi|207308	17588.4

Peptide Information

Calc. Mass	Obsrv. Mass	± da	± ppm	Start Seq.	End Seq.	Sequence	Ion Score	C. I. %	Modification	Plate [#] Name	Gel Idx/Pos [4700 Sample Name]	iTRAQ Ratio	iTRAQ Ratio	iTRAQ Ratio	Rank	Result Type

Calc. Mass	Obsrv. Mass	± da	± ppm	Start Seq.	End Seq.	Sequence	Ion Score	C. I. %	Modification	Plate [#] Name	Gel Idx/Pos [4700 Sample Name]	115/114*	116/114*	117/114*	Rank	Result Type
994.5441	994.5058	-0.0383	-39	78	84	VNLFSDR	29	97.106	(N-term)_iTRAQ[0]	[7] F5 120912	195/187	1.152	1.018	0.915	1	Mascot
1420.5648	1420.5096	-0.0552	-39	98	107	DEGDYMCELR	31	98.079	(N-term)_iTRAQ[0], MMTS (C)[7]	[1] F3 030912	281/273	1.056	0.973	0.850	1	Mascot
1733.9507	1733.9468	-0.0039	-2	22	35	VISLTACLVNQNLR	30	97.496	(N-term)_iTRAQ[0], MMTS (C)[7]	[8] F13-15 and F1+2	341/333	0.812	0.818	0.677	1	Mascot
1733.9507	1733.958	0.0073	4	22	35	VISLTACLVNQNLR	55	99.992	(N-term)_iTRAQ[0], MMTS (C)[7]	[8] F13-15 and F1+2	347/339	0.855	0.762	0.988	1	Mascot
1733.9507	1733.9674	0.0167	10	22	35	VISLTACLVNQNLR	46	99.933	(N-term)_iTRAQ[0], MMTS (C)[7]	[8] F13-15 and F1+2	350/342	1.071	0.861	0.817	1	Mascot
1809.9731	1810.0455	0.0724	40	61	75	HVLSGTLGVPEHTYR	49	99.97	(N-term)_iTRAQ[0]	[4] F7 and F10+11	1123/1115	0.787	0.536	1.000	1	Mascot

397	clathryn light chain (LCB3) [Rattus norvegicus]	gi|203361	25647.9	4	164	0.954	1.099	0.916	0.069	0.561	0.223	4	4	4	100

Protein Group

clathrin light chain B [Rattus norvegicus] gi|16758690 27787.9

Peptide Information

Calc. Mass	Obsrv. Mass	± da	± ppm	Start Seq.	End Seq.	Sequence	Ion Score	C. I. %	Modification	Plate [#] Name	Gel Idx/Pos [4700 Sample Name]	iTRAQ Ratio 115/114*	iTRAQ Ratio 116/114*	iTRAQ Ratio 117/114*	Rank	Result Type
1233.5984	1233.5698	-0.0286	-23	135	142	DLEEWNQR	31	98.079	(N-term)_iTRAQ[0]	[7] F5 120912	164/156	0.950	0.816	0.641	1	Mascot
1468.752	1468.7411	-0.0109	-7	177	186	VAQLCDFNPK	29	96.775	(N-term)_iTRAQ[0], Lysine(K)_iTRAQ[10], MMTS (C)[5]	[7] F5 120912	322/314	0.892	2.181	0.947	1	Mascot
1493.7369	1493.6881	-0.0488	-33	167	176	EETPGTEWEK	37	99.456	(N-term)_iTRAQ[0], Lysine(K)_iTRAQ[10]	[7] F5 120912	149/141	1.069	1.056	1.150	1	Mascot
1982.009	1982.0817	0.0727	37	164	176	ESKEETPGTEWEK	68	100	(N-term)_iTRAQ[0], Lysine(K)_iTRAQ[3,13]	[4] F7 and F10+11	1060/1052	0.916	0.777	1.010	1	Mascot

398	amino acid starvation-induced protein [Rattus norvegicus]	gi|202990	16551.2	3	164	0.833	0.995	0.890	0.133	0.254	0.189	3	3	3	100

Protein Group

60S ribosomal protein L17 [Rattus norvegicus] gi|42627879 25458.1

Peptide Information

Calc. Mass	Obsrv. Mass	± da	± ppm	Start Seq.	End Seq.	Sequence	Ion Score	C. I. %	Modification	Plate [#] Name	Gel Idx/Pos [4700 Sample Name]	iTRAQ Ratio 115/114*	iTRAQ Ratio 116/114*	iTRAQ Ratio 117/114*	Rank	Result Type
1476.8494	1476.7849	-0.0645	-44	23	32	SAEFLLHMLK	68	100	(N-term)_iTRAQ[0], Lysine(K)_iTRAQ[10]	[6] F8 110912	434/426	1.001	0.850	0.764	1	Mascot
1749.0469	1749.0101	-0.0368	-21	22	32	KSAEFLLHMLK	36	99.416	(N-term)_iTRAQ[0], Lysine(K)_iTRAQ[1,11]	[2] F12 040912	402/394	0.697	0.845	0.790	1	Mascot
2056.166	2056.1477	-0.0183	-9	90	103	EQIVPKPEEEVAQK	59	99.997	(N-term)_iTRAQ[0], Lysine(K)_iTRAQ[6,14]	[4] F7 and F10+11	198/190	0.827	1.373	1.168	1	Mascot

399	high mobility group protein B1 [Mus musculus]	gi|6754208	31356.6	3	163	1.014	0.954	0.871	0.054	0.039	0.258	3	3	3	100

Peptide Information

Calc. Mass	Obsrv. Mass	± da	± ppm	Start Seq.	End Seq.	Sequence	Ion Score	C. I. %	Modification	Plate [#] Name	Gel Idx/Pos [4700 Sample Name]	iTRAQ Ratio 115/114*	iTRAQ Ratio 116/114*	iTRAQ Ratio 117/114*	Rank	Result Type
1752.8802	1752.9478	0.0676	39	31	43	HPDASVNFSEFSK	67	99.999	(N-term)_iTRAQ[0], Lysine(K)_iTRAQ[13]	[3] F6 and F9	1211/1203	1.091	0.911	1.065	1	Mascot
1953.1504	1953.2367	0.0863	44	113	127	IKGEHPGLSIGDVAK	36	99.355	(N-term)_iTRAQ[0], Lysine(K)_iTRAQ[2,15]	[4] F7 and F10+11	1124/1116	0.980	0.951	0.604	1	Mascot
2025.0776	2025.009	-0.0686	-34	30	43	KHPDASVNFSEFSK	64	99.999	(N-term)_iTRAQ[0], Lysine(K)_iTRAQ[1,14]	[2] F12 040912	257/249	0.975	1.003	1.028	1	Mascot

400	neuronal membrane glycoprotein M6-a [Rattus norvegicus]	gi|30017437	33645.5	3	163	1.069	1.038	1.204	0.535	0.323	0.297	4	4	4	100

Peptide Information

Calc. Mass	Obsrv. Mass	± da	± ppm	Start Seq.	End Seq.	Sequence	Ion Score	C. I. %	Modification	Plate [#] Name	Gel Idx/Pos [4700 Sample Name]	iTRAQ Ratio 115/114*	iTRAQ Ratio 116/114*	iTRAQ Ratio 117/114*	Rank	Result Type
1508.8569	1508.7887	-0.0682	-45	179	189	QFGIVTIGEEK	72	100	(N-term)_iTRAQ[0], Lysine(K)_iTRAQ[11]	[5] F4	207/199	0.935	1.020	1.197	1	Mascot
1643.884	1643.9189	0.0349	21	190	200	KICTVSENFLR	49	99.972	(N-term)_iTRAQ[0], Lysine(K)_iTRAQ[1], MMTS (C)[3]	[3] F6 and F9	1292/1284	1.026	1.011	1.082	1	Mascot

Calc. Mass	Obsrv. Mass	± da	± ppm	Start Seq.	End Seq.	Sequence	Ion Score	C. I. %	Modification	Plate [#] Name	Gel Idx/Pos [4700 Sample Name]	iTRAQ Ratio 115/114*	iTRAQ Ratio 116/114*	iTRAQ Ratio 117/114*	Rank	Result Type
1643.884	1643.9454	0.0614	37	190	200	KICTVSENFLR	48	99.964	(N-term)_iTRAQ[0], Lysine(K)_iTRAQ[1], MMTS (C)[3]	[4] F7 and F10+11	1231/1223	2.041	1.555	1.715	1	Mascot
1781.0544	1781.114	0.0596	33	179	190	QFGIVTIGEEKK	42	99.86	(N-term)_iTRAQ[0], Lysine(K)_iTRAQ[11, 12]	[3] F6 and F9	1227/1219	0.667	0.725	0.947	1	Mascot

401	keratin, type I cytoskeletal 10 [Rattus norvegicus]	gi|57012436	60833.4	3	163	0.752	0.703	1.358	0.113	0.194	0.364	4	4	4	100

Peptide Information

Calc. Mass	Obsrv. Mass	± da	± ppm	Start Seq.	End Seq.	Sequence	Ion Score	C. I. %	Modification	Plate [#] Name	Gel Idx/Pos [4700 Sample Name]	iTRAQ Ratio 115/114*	iTRAQ Ratio 116/114*	iTRAQ Ratio 117/114*	Rank	Result Type
1397.6947	1397.6748	-0.0199	-14	334	342	DAEAWFNEK	45	99.924	(N-term)_iTRAQ[0], Lysine(K)_iTRAQ[9]	[3] F6 and F9	274/266	0.603	0.643	1.479	1	Mascot
1522.8839	1522.9526	0.0687	45	235	244	LKYENEVALR	29	97.018	(N-term)_iTRAQ[0], Lysine(K)_iTRAQ[2]	[4] F7 and F10+11	1116/1108	0.886	0.854	0.953	1	Mascot
2384.2517	2384.2097	-0.042	-18	266	283	ADLEMQIESLTEELAYLK	86	100	(N-term)_iTRAQ[0], Lysine(K)_iTRAQ[18]	[1] F3 030912	564/556	0.761	0.492	1.838	1	Mascot
2400.2466	2400.1865	-0.0601	-25	266	283	ADLEMQIESLTEELAYLK	89	100	(N-term)_iTRAQ[0], Lysine(K)_iTRAQ[18], Oxidation (M)[5]	[1] F3 030912	562/554	0.788	0.905	1.314	1	Mascot

402	cytoplasmic dynein 1 light intermediate chain 1 [Rattus norvegicus]	gi|21955134	62705.3	3	161	1.253	0.957	1.173	0.120	0.438	0.147	3	3	3	100

Peptide Information

Calc. Mass	Obsrv. Mass	± da	± ppm	Start Seq.	End Seq.	Sequence	Ion Score	C. I. %	Modification	Plate [#] Name	Gel Idx/Pos [4700 Sample Name]	iTRAQ Ratio 115/114*	iTRAQ Ratio 116/114*	iTRAQ Ratio 117/114*	Rank	Result Type
1856.9384	1856.8806	-0.0578	-31	366	378	EIMAEDDQVFLMK	38	99.594	(N-term)_iTRAQ[0], Lysine(K)_iTRAQ[13]	[1] F3 030912	344/336	1.101	0.601	1.383	1	Mascot
1909.9531	1909.8884	-0.0647	-34	117	129	CNVWILDGDLYHK	52	99.985	(N-term)_iTRAQ[0], Lysine(K)_iTRAQ[13], MMTS (C)[1]	[6] F8 110912	437/429	1.330	1.515	1.107	1	Mascot
2074.1431	2074.1321	-0.011	-5	345	359	IEDNFEDIITKPPVR	71	100	(N-term)_iTRAQ[0], Lysine(K)_iTRAQ[11]	[4] F7 and F10+11	362/354	1.344	0.961	1.054	1	Mascot

403	endoplasmic reticulum resident protein 29 precursor [Rattus norvegicus]	gi|16758848	32493.7	3	161	1.141	1.276	1.204	0.811	0.598	0.767	3	3	3	100

Protein Group

Chain A, Nmr Structure Of N-Domain Of Erp29 Protein gi|11513363 15864.4

Peptide Information

Calc. Mass	Obsrv. Mass	± da	± ppm	Start Seq.	End Seq.	Sequence	Ion Score	C. I. %	Modification	Plate [#] Name	Gel Idx/Pos [4700 Sample Name]	iTRAQ Ratio 115/114*	iTRAQ Ratio 116/114*	iTRAQ Ratio 117/114*	Rank	Result Type
1464.7648	1464.7312	-0.0336	-23	113	122	ESYPVFYLFR	37	99.473	(N-term)_iTRAQ[0]	[1] F3 030912	468/460	0.537	0.741	0.764	1	Mascot
1535.7628	1535.6897	-0.0731	-48	60	69	FDTQYPYGEK	48	99.959	(N-term)_iTRAQ[0], Lysine(K)_iTRAQ[10]	[7] F5 120912	174/166	1.805	1.641	2.389	1	Mascot
1882.9432	1882.8455	-0.0977	-52	123	137	DGDFENPVPYSGAVK	77	100	(N-term)_iTRAQ[0], Lysine(K)_iTRAQ[15]	[5] F4	170/162	1.532	1.708	0.957	1	Mascot

404	RecName: Full=Methylmalonate-semialdehyde dehydrogenase [acylating], mitochondrial; Short=MMSDH; Sh	gi|400269	62893.8	4	161	0.992	0.916	0.829	0.273	0.410	0.113	4	4	4	100

Peptide Information

Calc. Mass	Obsrv. Mass	± da	± ppm	Start Seq.	End Seq.	Sequence	Ion Score	C. I. %	Modification	Plate [#] Name	Gel Idx/Pos [4700 Sample Name]	iTRAQ Ratio 115/114*	iTRAQ Ratio 116/114*	iTRAQ Ratio 117/114*	Rank	Result Type
1185.6752	1185.6394	-0.0358	-30	332	339	WLPELVER	48	99.963	(N-term)_iTRAQ[0]	[1] F3 030912	357/349	0.816	1.038	0.873	1	Mascot
1513.8624	1513.8302	-0.0322	-21	501	510	QGIQFYTQLK	50	99.977	(N-term)_iTRAQ[0], Lysine(K)_iTRAQ[10]	[7] F5 120912	257/249	0.930	0.840	0.665	1	Mascot
1864.979	1864.9264	-0.0526	-28	56	70	WIDIHNPATNEVVGR	35	99.304	(N-term)_iTRAQ[0]	[3] F6 and F9	311/303	1.499	0.539	0.892	1	Mascot
2032.026	2031.9714	-0.0546	-27	256	272	AISFVGSNQAGEYIFER	27	95.016	(N-term)_iTRAQ[0]	[1] F3 030912	376/368	0.850	1.498	0.912	1	Mascot

405	4F2 cell-surface antigen heavy chain [Rattus norvegicus]	gi|9506891	62162.9	4	160	1.331	1.631	1.416	0.681	0.737	0.551	4	4	4	100

Peptide Information

Calc. Mass	Obsrv. Mass	± da	± ppm	Start Seq.	End Seq.	Sequence	Ion Score	C. I. %	Modification	Plate [#] Name	Gel Idx/Pos [4700 Sample Name]	iTRAQ Ratio 115/114*	iTRAQ Ratio 116/114*	iTRAQ Ratio 117/114*	Rank	Result Type
1705.8629	1705.7827	-0.0802	-47	405	418	GQNEDPGSLLTQFR	36	99.375	(N-term)_iTRAQ[0]	[1] F3 030912	316/308	0.683	0.928	1.083	1	Mascot

Calc. Mass	Obsrv. Mass	± da	± ppm	Start Seq.	End Seq.	Sequence	Ion Score	C. I. %	Modification	Plate [#] Name	Gel Idx/Pos [4700 Sample Name]	iTRAQ Ratio 115/114*	iTRAQ Ratio 116/114*	iTRAQ Ratio 117/114*	Rank	Result Type
2051.0317	2051.0439	0.0122	6	226	242	EALSSWLQDGVDGFQVR	40	99.727	(N-term)_iTRAQ[0]	[8] F13-15 and F1+2	361/353	1.406	1.580	1.166	1	Mascot
2144.175	2144.1082	-0.0668	-31	207	223	GQNAWFLPPQADIVATK	55	99.992	(N-term)_iTRAQ[0], Lysine(K)_iTRAQ[17]	[1] F3 030912	391/383	1.548	2.612	2.478	1	Mascot
2402.2112	2402.1562	-0.055	-23	429	449	SLLHGDFDALSSSSGLFS YVR	29	97.112	(N-term)_iTRAQ[0]	[7] F5 120912	441/433	2.111	1.847	1.283	1	Mascot

406	N(G),N(G)-dimethylarginine dimethylaminohydrolase 2 [Rattus norvegicus]	gi|47087079	31098.1	3	160	1.421	1.667	1.483	1.036	0.436	0.590	3	3	3	100

Peptide Information

Calc. Mass	Obsrv. Mass	± da	± ppm	Start Seq.	End Seq.	Sequence	Ion Score	C. I. %	Modification	Plate [#] Name	Gel Idx/Pos [4700 Sample Name]	iTRAQ Ratio 115/114*	iTRAQ Ratio 116/114*	iTRAQ Ratio 117/114*	Rank	Result Type
1214.7031	1214.6616	-0.0415	-34	135	142	EFFVGLSK	49	99.967	(N-term)_iTRAQ[0], Lysine(K)_iTRAQ[8]	[7] F5 120912	275/267	1.234	1.296	1.310	1	Mascot
1701.9017	1701.8356	-0.0661	-39	238	251	GGGDLPNSQEALQK	69	100	(N-term)_iTRAQ[0], Lysine(K)_iTRAQ[14]	[7] F5 120912	144/136	2.949	1.574	2.344	1	Mascot
1786.9572	1786.8842	-0.073	-41	158	173	DFAVSTVPVSGASHLR	42	99.859	(N-term)_iTRAQ[0]	[7] F5 120912	220/212	0.789	2.270	1.062	1	Mascot

407	phosphatidylethanolamine-binding protein 1 [Rattus norvegicus]	gi|8393910	22897.8	2	159	0.999	0.955	0.989	0.010	0.111	0.110	2	2	2	100

Peptide Information

Calc. Mass	Obsrv. Mass	± da	± ppm	Start Seq.	End Seq.	Sequence	Ion Score	C. I. %	Modification	Plate [#] Name	Gel Idx/Pos [4700 Sample Name]	iTRAQ Ratio 115/114*	iTRAQ Ratio 116/114*	iTRAQ Ratio 117/114*	Rank	Result Type
1200.6986	1200.7537	0.0551	46	149	155	FKVESFR	34	98.994	(N-term)_iTRAQ[0], Lysine(K)_iTRAQ[2]	[4] F7 and F10+11	1113/1105	1.009	1.065	0.890	1	Mascot
2252.1655	2252.0708	-0.0947	-42	94	113	GNDISSGTVLSEYVGSGP PK	125	100	(N-term)_iTRAQ[0], Lysine(K)_iTRAQ[20]	[1] F3 030912	301/293	0.989	0.855	1.099	1	Mascot

408	dynein light chain 1, cytoplasmic [Homo sapiens]	gi|4505813	12082.2	2	159	0.842	1.064	0.897	0.208	0.079	0.076	2	2	2	100

Peptide Information

Calc. Mass	Obsrv. Mass	± da	± ppm	Start Seq.	End Seq.	Sequence	Ion Score	C. I. %	Modification	Plate [#] Name	Gel Idx/Pos [4700 Sample Name]	iTRAQ Ratio 115/114*	iTRAQ Ratio 116/114*	iTRAQ Ratio 117/114*	Rank	Result Type
1570.8112	1570.8656	0.0544	35	61	71	NFGSYVTHETK	48	99.957	(N-term)_iTRAQ[0], Lysine(K)_iTRAQ[11]	[3] F6 and F9	1161/1153	0.675	0.991	0.827	1	Mascot
2791.2122	2791.1125	-0.0997	-36	10	31	NADMSEEMQQDSVECA TQALEK	111	100	(N-term)_iTRAQ[0], Lysine(K)_iTRAQ[22], MMTS (C)[15]	[1] F3 030912	386/378	1.050	1.143	0.973	1	Mascot

409	brevican core protein [Rattus norvegicus]	gi|1143285	99691.5	3	158	0.971	0.997	0.921	0.078	0.145	0.281	3	3	3	100

Peptide Information

Calc. Mass	Obsrv. Mass	± da	± ppm	Start Seq.	End Seq.	Sequence	Ion Score	C. I. %	Modification	Plate [#] Name	Gel Idx/Pos [4700 Sample Name]	iTRAQ Ratio 115/114*	iTRAQ Ratio 116/114*	iTRAQ Ratio 117/114*	Rank	Result Type
1218.6967	1218.6287	-0.068	-56	312	320	YPIITPSQR	35	99.281	(N-term)_iTRAQ[0]	[1] F3 030912	216/208	0.933	0.839	1.030	1	Mascot
1437.7246	1437.6691	-0.0555	-39	717	726	EYQWIGLNDR	60	99.998	(N-term)_iTRAQ[0]	[5] F4	215/207	1.082	1.014	1.189	1	Mascot
1728.8888	1728.9141	0.0253	15	539	554	EVAGETGSPELSGVPR	64	99.999	(N-term)_iTRAQ[0]	[8] F13-15 and F1+2	566/558	0.908	1.168	0.638	1	Mascot

410	clathrin-associated protein 17 [Rattus norvegicus]	gi|1809320	18540.7	3	156	1.144	1.297	1.177	0.400	0.141	0.272	3	3	3	100

Protein Group

AP-2 complex subunit sigma [Rattus norvegicus]	gi|56961624	18585.7

Peptide Information

Calc. Mass	Obsrv. Mass	± da	± ppm	Start Seq.	End Seq.	Sequence	Ion Score	C. I. %	Modification	Plate [#] Name	Gel Idx/Pos [4700 Sample Name]	iTRAQ Ratio 115/114*	iTRAQ Ratio 116/114*	iTRAQ Ratio 117/114*	Rank	Result Type
1047.6436	1047.5839	-0.0597	-57	4	10	FILIQNR	29	96.99	(N-term)_iTRAQ[0]	[5] F4	197/189	1.744	1.396	1.579	1	Mascot
1664.7512	1664.697	-0.0542	-33	19	28	WYMQFDDDEK	64	99.999	(N-term)_iTRAQ[0], Lysine(K)_iTRAQ[10]	[7] F5 120912	291/283	0.892	1.394	1.011	1	Mascot
2065.0073	2065.094	0.0867	42	19	30	WYMQFDDDEKQK	63	99.999	(N-term)_iTRAQ[0], Lysine(K)_iTRAQ[10, 12]	[4] F7 and F10+11	1181/1173	0.961	1.121	1.022	1	Mascot

411	thioredoxin [Rattus norvegicus]	gi|16758644	13814.9	3	156	0.859	1.008	0.918	0.235	0.243	0.261	4	4	4	100

Peptide Information

Calc. Mass	Obsrv. Mass	± da	± ppm	Start Seq.	End Seq.	Sequence	Ion Score	C. I. %	Modification	Plate [#] Name	Gel Idx/Pos [4700 Sample Name]	iTRAQ Ratio 115/114*	iTRAQ Ratio 116/114*	iTRAQ Ratio 117/114*	Rank	Result Type

1498.7124	1498.7091	-0.0033	-2	73	81	CMPTFQFYK	38	99.609	(N-term)_iTRAQ[0], Lysine(K)_iTRAQ[9], MMTS (C)[1]	[7] F5 120912	363/355	1.183	1.270	1.185	1	Mascot
1608.8479	1608.7834	-0.0645	-40	9	21	EAFQEALAAAGDK	70	100	(N-term)_iTRAQ[0], Lysine(K)_iTRAQ[13]	[5] F4	215/207	0.963	1.054	0.707	1	Mascot
1944.0262	1943.9792	-0.047	-24	37	48	MIKPFFHSLCDK	43	99.878	(N-term)_iTRAQ[0], Lysine(K)_iTRAQ[3,12], MMTS (C)[10]	[2] F12 040912	417/409	0.774	1.091	1.173	1	Mascot
1944.0262	1944.097	0.0708	36	37	48	MIKPFFHSLCDK	48	99.964	(N-term)_iTRAQ[0], Lysine(K)_iTRAQ[3,12], MMTS (C)[10]	[4] F7 and F10+11	1276/1268	0.619	0.707	0.723	1	Mascot

412	ATP synthase subunit delta, mitochondrial precursor [Rattus norvegicus]	gi|20806153	18160.6	2	156	0.866	1.140	0.765	0.275	0.348	0.245	3	3	3	100

Peptide Information

Calc. Mass	Obsrv. Mass	± da	± ppm	Start Seq.	End Seq.	Sequence	Ion Score	C. I. %	Modification	Plate [#] Name	Gel Idx/Pos [4700 Sample Name]	iTRAQ Ratio 115/114*	iTRAQ Ratio 116/114*	iTRAQ Ratio 117/114*	Rank	Result Type
1274.7566	1274.6863	-0.0703	-55	157	165	IEANEALVK	37	99.551	(N-term)_iTRAQ[0], Lysine(K)_iTRAQ[9]	[7] F5 120912	165/157	0.642	0.958	0.894	1	Mascot
1519.7472	1519.6541	-0.0931	-61	137	150	AQSELSGAADEAAR	47	99.953	(N-term)_iTRAQ[0]	[5] F4	101/93	1.250	1.662	0.518	1	Mascot
1519.7472	1519.6664	-0.0808	-53	137	150	AQSELSGAADEAAR	118	100	(N-term)_iTRAQ[0]	[1] F3 030912	161/153	0.810	0.931	0.967	1	Mascot

413	phosphatidylinositol-5-phosphate 4-kinase type-2 beta [Rattus norvegicus]	gi|16758316	53031.6	2	155	0.864	0.841	0.783	0.027	0.023	0.014	2	2	2	100

Peptide Information

Calc. Mass	Obsrv. Mass	± da	± ppm	Start Seq.	End Seq.	Sequence	Ion Score	C. I. %	Modification	Plate [#] Name	Gel Idx/Pos [4700 Sample Name]	iTRAQ Ratio 115/114*	iTRAQ Ratio 116/114*	iTRAQ Ratio 117/114*	Rank	Result Type
1801.8477	1801.7637	-0.084	-47	110	123	FGIDDQDYQNSVTR	70	100	(N-term)_iTRAQ[0]	[1] F3 030912	230/222	0.838	0.818	0.796	1	Mascot
2076.0608	2075.9695	-0.0913	-44	389	405	HGAGAEISTVNPEQYSK	85	100	(N-term)_iTRAQ[0], Lysine(K)_iTRAQ[17]	[6] F8 110912	206/198	0.891	0.864	0.770	1	Mascot

414	activated RNA polymerase II transcriptional coactivator p15 [Rattus norvegicus]	gi|57526804	17314.5	3	154	0.870	1.006	0.983	0.030	0.239	0.043	3	3	3	100

Peptide Information

Calc. Mass	Obsrv. Mass	± da	± ppm	Start Seq.	End Seq.	Sequence	Ion Score	C. I. %	Modification	Plate [#] Name	Gel Idx/Pos [4700 Sample Name]	iTRAQ Ratio 115/114*	iTRAQ Ratio 116/114*	iTRAQ Ratio 117/114*	Rank	Result Type
1404.7091	1404.6313	-0.0778	-55	115	125	EQISDIDDAVR	31	98.016	(N-term)_iTRAQ[0]	[1] F3 030912	209/201	0.830	0.946	0.927	1	Mascot
1821.9779	1821.8792	-0.0987	-54	102	114	GISLNMEQWSQLK	78	100	(N-term)_iTRAQ[0], Lysine(K)_iTRAQ[13]	[5] F4	290/282	0.884	0.803	0.999	1	Mascot
2002.9248	2002.8075	-0.1173	-59	87	100	EYWMDSEGEMKPGR	45	99.92	(N-term)_iTRAQ[0], Lysine(K)_iTRAQ[11]	[6] F8 110912	252/244	0.897	1.340	1.027	1	Mascot

415	tetraspanin-2 [Rattus norvegicus]	gi|12018246	26120.1	2	153	2.850	1.189	1.234	0.044	0.029	0.017	2	2	2	100

Peptide Information

Calc. Mass	Obsrv. Mass	± da	± ppm	Start Seq.	End Seq.	Sequence	Ion Score	C. I. %	Modification	Plate [#] Name	Gel Idx/Pos [4700 Sample Name]	iTRAQ Ratio 115/114*	iTRAQ Ratio 116/114*	iTRAQ Ratio 117/114*	Rank	Result Type
1666.8008	1666.7115	-0.0893	-54	155	166	ESSEQVQPTCPK	64	99.999	(N-term)_iTRAQ[0], Lysine(K)_iTRAQ[12], MMTS (C)[10]	[5] F4	111/103	2.806	1.160	1.218	1	Mascot
2020.9194	2020.907	-0.0124	-6	119	133	HVQSMYEEAYSDYVR	89	100	(N-term)_iTRAQ[0]	[4] F7 and F10+11	311/303	2.894	1.218	1.251	1	Mascot

416	ATP synthase subunit g, mitochondrial [Rattus norvegicus]	gi|47058994	12605.9	2	153	0.957	1.084	1.193	0.076	0.161	0.034	2	2	2	100

Peptide Information

Calc. Mass	Obsrv. Mass	± da	± ppm	Start Seq.	End Seq.	Sequence	Ion Score	C. I. %	Modification	Plate [#] Name	Gel Idx/Pos [4700 Sample Name]	iTRAQ Ratio 115/114*	iTRAQ Ratio 116/114*	iTRAQ Ratio 117/114*	Rank	Result Type
1308.6974	1308.74	0.0426	33	27	35	LATFWHYAR	56	99.994	(N-term)_iTRAQ[0]	[4] F7 and F10+11	1233/1225	0.887	0.944	1.160	1	Mascot
1617.8959	1617.9465	0.0506	31	55	66	NIIHSAQTGNFK	97	100	(N-term)_iTRAQ[0], Lysine(K)_iTRAQ[12]	[3] F6 and F9	1153/1145	1.033	1.245	1.227	1	Mascot

417	neuronal growth regulator 1 precursor [Rattus norvegicus]	gi|11067409	40547.3	2	152	1.090	1.426	0.475	0.002	0.206	0.575	2	2	2	100

Peptide Information

Calc. Mass	Obsrv. Mass	± da	± ppm	Start Seq.	End Seq.	Sequence	Ion Score	C. I. %	Modification	Plate [#] Name	Gel Idx/Pos [4700 Sample Name]	iTRAQ Ratio 115/114*	iTRAQ Ratio 116/114*	iTRAQ Ratio 117/114*	Rank	Result Type

Calc. Mass	Obsrv. Mass	± da	± ppm	Start Seq.	End Seq.	Sequence	Ion Score	C. I. %	Modification	Plate [#] Name	Gel Idx/Pos [4700 Sample Name]	iTRAQ Ratio 115/114*	iTRAQ Ratio 116/114*	iTRAQ Ratio 117/114*	Rank	Result Type
1746.0411	1745.9916	-0.0495	-28	213	225	VVVNFAPTIQEIK	67	100	(N-term)_iTRAQ[0], Lysine(K)_iTRAQ[13]	[1] F3 030912	361/353	1.092	1.246	1.051	1	Mascot
3370.542	3370.4329	-0.1091	-32	94	121	DYSLQIQNVDVTDDGPYT CSVQTQHTPR	85	100	(N-term)_iTRAQ[0], MMTS (C)[19]	[1] F3 030912	345/337	1.088	1.632	0.215	1	Mascot

418 BIT [Rattus norvegicus] gi|2190166 60787.2 3 151 1.220 1.182 1.073 0.138 0.423 0.099 3 3 3 100

Protein Group

RecName: Full=Tyrosine-protein phosphatase non-receptor type substrate 1; Short=SHP substrate 1; Sh gi|29427383 60773.1

tyrosine-protein phosphatase non-receptor type substrate 1 [Rattus norvegicus] gi|31543529 60738.2

Peptide Information

Calc. Mass	Obsrv. Mass	± da	± ppm	Start Seq.	End Seq.	Sequence	Ion Score	C. I. %	Modification	Plate [#] Name	Gel Idx/Pos [4700 Sample Name]	iTRAQ Ratio 115/114*	iTRAQ Ratio 116/114*	iTRAQ Ratio 117/114*	Rank	Result Type
1647.8337	1647.9413	0.1076	65	297	307	TDKPEHFTDNR	33	98.785	(N-term)_iTRAQ[0], Lysine(K)_iTRAQ[3]	[8] F13-15 and F1+2	1286/1278	1.154	0.967	1.063	1	Mascot
1750.9037	1750.9021	-0.0016	-1	77	90	SPIYSFIGGEHFPR	68	100	(N-term)_iTRAQ[0]	[4] F7 and F10+11	397/389	1.111	0.938	1.201	1	Mascot
2174.0974	2174.0759	-0.0215	-10	451	467	VPEPNNHTEYASIETGK	50	99.977	(N-term)_iTRAQ[0], Lysine(K)_iTRAQ[17]	[4] F7 and F10+11	201/193	1.417	1.821	0.967	1	Mascot

419 ubiquitin-conjugating enzyme E2 variant 2 [Rattus norvegicus] gi|34101284 17829.2 4 150 0.799 0.829 0.892 0.257 0.211 0.398 4 4 4 100

Protein Group

putative ubiquitin-conjugating enzyme variant MMS2 [Rattus norvegicus] gi|24817674 14314.4

Peptide Information

Calc. Mass	Obsrv. Mass	± da	± ppm	Start Seq.	End Seq.	Sequence	Ion Score	C. I. %	Modification	Plate [#] Name	Gel Idx/Pos [4700 Sample Name]	iTRAQ Ratio 115/114*	iTRAQ Ratio 116/114*	iTRAQ Ratio 117/114*	Rank	Result Type
1159.6232	1159.5565	-0.0667	-58	73	81	YPEAPPSVR	28	95.883	(N-term)_iTRAQ[0]	[1] F3 030912	180/172	0.582	0.751	0.629	1	Mascot
1271.7054	1271.6777	-0.0277	-22	46	55	WTGMIIGPPR	38	99.628	(N-term)_iTRAQ[0]	[1] F3 030912	332/324	0.739	0.640	0.769	1	Mascot
1313.71	1313.6732	-0.0368	-28	109	116	WQNSYSIK	36	99.365	(N-term)_iTRAQ[0], Lysine(K)_iTRAQ[8]	[3] F6 and F9	223/215	1.253	1.188	1.658	1	Mascot
1475.8203	1475.7777	-0.0426	-29	15	24	LLEELEEGQK	49	99.966	(N-term)_iTRAQ[0], Lysine(K)_iTRAQ[10]	[7] F5 120912	298/290	0.756	0.828	0.790	1	Mascot

420 dynactin subunit 1 [Rattus norvegicus] gi|13162302 155841.3 3 150 1.321 1.292 0.925 0.814 0.200 0.095 3 3 3 100

Peptide Information

Calc. Mass	Obsrv. Mass	± da	± ppm	Start Seq.	End Seq.	Sequence	Ion Score	C. I. %	Modification	Plate [#] Name	Gel Idx/Pos [4700 Sample Name]	iTRAQ Ratio 115/114*	iTRAQ Ratio 116/114*	iTRAQ Ratio 117/114*	Rank	Result Type
1846.0432	1845.9421	-0.1011	-55	1070	1087	GGTPGQAPGALPGPGPV K	66	99.999	(N-term)_iTRAQ[0], Lysine(K)_iTRAQ[18]	[1] F3 030912	197/189	0.786	1.384	0.813	1	Mascot
2074.0498	2074.1506	0.1008	49	1236	1249	AKEEQQDDTVYMGK	50	99.977	(N-term)_iTRAQ[0], Lysine(K)_iTRAQ[2,1 4]	[4] F7 and F10+11	1050/1042	1.172	1.057	0.945	1	Mascot
2613.2488	2613.2334	-0.0154	-6	1010	1029	EFEETMDALQADIDQLEA EK	34	99.015	(N-term)_iTRAQ[0], Lysine(K)_iTRAQ[20]	[1] F3 030912	541/533	2.502	1.473	1.030	1	Mascot

421 transitional endoplasmic reticulum ATPase [Rattus norvegicus] gi|17865351 96761.5 3 150 0.964 1.065 1.025 0.166 0.371 0.257 3 3 3 100

Peptide Information

Calc. Mass	Obsrv. Mass	± da	± ppm	Start Seq.	End Seq.	Sequence	Ion Score	C. I. %	Modification	Plate [#] Name	Gel Idx/Pos [4700 Sample Name]	iTRAQ Ratio 115/114*	iTRAQ Ratio 116/114*	iTRAQ Ratio 117/114*	Rank	Result Type
1337.7562	1337.6844	-0.0718	-54	669	677	DVDLEFLAK	41	99.799	(N-term)_iTRAQ[0], Lysine(K)_iTRAQ[9]	[5] F4	244/236	0.903	1.237	1.322	1	Mascot
2096.0242	2096.0229	-0.0013	-1	544	560	GPELLTMWFGESEANVR	28	96.356	(N-term)_iTRAQ[0], Oxidation (M)[7]	[8] F13-15 and F1+2	310/302	0.827	0.702	0.768	1	Mascot
2099.1997	2099.1533	-0.0464	-22	296	312	NAPAIIFIDELDAIAPK	80	100	(N-term)_iTRAQ[0], Lysine(K)_iTRAQ[17]	[1] F3 030912	546/538	1.200	1.391	1.062	1	Mascot

422 tropomodulin-2 [Rattus norvegicus] gi|13928838 44511.2 4 149 1.107 0.963 0.952 0.263 0.256 0.128 4 4 4 100

Peptide Information

Calc. Mass	Obsrv. Mass	± da	± ppm	Start Seq.	End Seq.	Sequence	Ion Score	C. I. %	Modification	Plate [#] Name	Gel Idx/Pos [4700 Sample Name]	iTRAQ Ratio 115/114*	iTRAQ Ratio 116/114*	iTRAQ Ratio 117/114*	Rank	Result Type
1178.6456	1178.6412	-0.0044	-4	317	323	FGYQFTK	27	95.579	(N-term)_iTRAQ[0], Lysine(K)_iTRAQ[7]	[3] F6 and F9	246/238	1.285	1.153	0.968	1	Mascot

1727.885	1727.9519	0.0669	39	82	93	DREDFVPFTGEK	51	99.981	(N-term)_iTRAQ[0], Lysine(K)_iTRAQ[12]	[3] F6 and F9	1212/1204	0.940	0.917	0.841	1	Mascot
1793.9717	1793.9141	-0.0576	-32	238	251	SNDPVALAFAEMLK	41	99.805	(N-term)_iTRAQ[0], Lysine(K)_iTRAQ[14]	[5] F4	389/381	0.862	0.669	0.870	1	Mascot
1957.0601	1956.946	-0.1141	-58	194	208	ANDPSLQEVNLNNIK	30	97.42	(N-term)_iTRAQ[0], Lysine(K)_iTRAQ[15]	[5] F4	194/186	1.441	1.218	1.162	1	Mascot

423	growth factor receptor-bound protein 2 isoform 1 [Homo sapiens]	gi|4504111	27732.2	3	149	1.394	1.030	1.338	0.536	0.411	0.380	3	3	3	100	

Protein Group

Ash-m [Rattus norvegicus]	gi|914957	26082.4

Peptide Information

Calc. Mass	Obsrv. Mass	± da	± ppm	Start Seq.	End Seq.	Sequence	Ion Score	C. I. %	Modification	Plate [#] Name	Gel Idx/Pos [4700 Sample Name]	iTRAQ Ratio 115/114*	iTRAQ Ratio 116/114*	iTRAQ Ratio 117/114*	Rank	Result Type
1242.7496	1242.7886	0.039	31	118	124	YFLWVVK	32	98.284	(N-term)_iTRAQ[0], Lysine(K)_iTRAQ[7]	[7] F5 120912	416/408	1.955	1.367	1.731	1	Mascot
1451.6447	1451.6934	0.0487	34	196	207	GACHGQTGMFPR	61	99.998	(N-term)_iTRAQ[0], MMTS (C)[3]	[3] F6 and F9	1170/1162	0.899	0.643	0.954	1	Mascot
1874.8644	1874.8147	-0.0497	-27	27	38	VLNEECDQNWYK	57	99.995	(N-term)_iTRAQ[0], Lysine(K)_iTRAQ[12], MMTS (C)[6]	[7] F5 120912	294/286	1.543	1.244	1.452	1	Mascot

424	vimentin [Rattus norvegicus]	gi|14389299	57060.4	3	149	1.432	1.687	1.690	0.596	0.613	0.718	3	3	3	100	

Peptide Information

Calc. Mass	Obsrv. Mass	± da	± ppm	Start Seq.	End Seq.	Sequence	Ion Score	C. I. %	Modification	Plate [#] Name	Gel Idx/Pos [4700 Sample Name]	iTRAQ Ratio 115/114*	iTRAQ Ratio 116/114*	iTRAQ Ratio 117/114*	Rank	Result Type
1409.7886	1409.7469	-0.0417	-30	382	390	EYQDLLNVK	60	99.997	(N-term)_iTRAQ[0], Lysine(K)_iTRAQ[9]	[7] F5 120912	243/235	2.284	1.143	1.029	1	Mascot
1457.9188	1457.8785	-0.0403	-28	130	139	ILLAELEQLK	39	99.701	(N-term)_iTRAQ[0], Lysine(K)_iTRAQ[10]	[5] F4	370/362	0.991	2.440	2.040	1	Mascot
1597.8108	1597.7561	-0.0547	-34	283	292	NLQEAEEWYK	50	99.976	(N-term)_iTRAQ[0], Lysine(K)_iTRAQ[10]	[7] F5 120912	236/228	1.297	1.720	2.300	1	Mascot

425	LRRGT00113 [Rattus norvegicus]	gi|37361912	61844.1	4	149	0.888	0.895	1.023	0.241	0.277	0.177	4	4	4	100	

Peptide Information

Calc. Mass	Obsrv. Mass	± da	± ppm	Start Seq.	End Seq.	Sequence	Ion Score	C. I. %	Modification	Plate [#] Name	Gel Idx/Pos [4700 Sample Name]	iTRAQ Ratio 115/114*	iTRAQ Ratio 116/114*	iTRAQ Ratio 117/114*	Rank	Result Type
1541.7543	1541.7056	-0.0487	-32	339	349	MNYSDAIEWLR	40	99.772	(N-term)_iTRAQ[0]	[5] F4	315/307	0.684	1.005	0.784	1	Mascot
1743.8687	1743.8333	-0.0354	-20	427	438	SWDSEEILEGYK	46	99.941	(N-term)_iTRAQ[0], Lysine(K)_iTRAQ[12]	[7] F5 120912	301/293	1.311	1.252	1.202	1	Mascot
1821.9377	1821.9448	0.0071	4	374	387	LMTDTINEPILLCR	28	95.709	(N-term)_iTRAQ[0], MMTS (C)[13]	[8] F13-15 and F1+2	341/333	0.805	0.598	1.070	1	Mascot
1899.9698	1900.0028	0.033	17	427	439	SWDSEEILEGYKR	35	99.132	(N-term)_iTRAQ[0], Lysine(K)_iTRAQ[12]	[3] F6 and F9	1255/1247	0.860	0.851	1.086	1	Mascot

426	glutamate receptor 2 isoform 1 precursor [Rattus norvegicus]	gi|8393475	108582.8	3	147	0.749	1.085	1.049	0.173	0.138	0.041	3	3	3	100	

Protein Group

RecName: Full=Glutamate receptor 2; Short=GluR-2; AltName: Full=AMPA-selective glutamate receptor 2	gi|3287964	108353.5
glutamate receptor 2 isoform 2 precursor [Rattus norvegicus]	gi|139394534	108381.6

Peptide Information

Calc. Mass	Obsrv. Mass	± da	± ppm	Start Seq.	End Seq.	Sequence	Ion Score	C. I. %	Modification	Plate [#] Name	Gel Idx/Pos [4700 Sample Name]	iTRAQ Ratio 115/114*	iTRAQ Ratio 116/114*	iTRAQ Ratio 117/114*	Rank	Result Type
1085.5667	1085.4974	-0.0693	-64	787	791	WWYDK	27	95.085	(N-term)_iTRAQ[0], Lysine(K)_iTRAQ[5]	[6] F8 110912	287/279	0.992	1.175	1.023	1	Mascot
1475.8104	1475.7202	-0.0902	-61	284	294	EYPGAHTATIK	66	99.999	(N-term)_iTRAQ[0], Lysine(K)_iTRAQ[11]	[6] F8 110912	146/138	0.701	1.187	1.107	1	Mascot
1652.9958	1653.0605	0.0647	39	194	203	SLFQDLELKK	55	99.992	(N-term)_iTRAQ[0], Lysine(K)_iTRAQ[9,10]	[4] F7 and F10+11	1239/1231	0.605	0.916	1.019	1	Mascot

427	dynactin subunit 2 [Rattus norvegicus]	gi|51948450	49112.2	2	147	1.142	0.816	0.992	0.013	0.095	0.127	2	2	2	100	

Peptide Information

Calc. Mass	Obsrv. Mass	± da	± ppm	Start Seq.	End Seq.	Sequence	Ion Score	C. I. %	Modification	Plate [#] Name	Gel Idx/Pos [4700 Sample Name]	iTRAQ Ratio	iTRAQ Ratio	iTRAQ Ratio	Rank	Result Type

												115/114*	116/114*	117/114*		
1440.842	1440.8511	0.0091	6	147	156	QQLVASHLEK	62	99.998	(N-term)_iTRAQ[0], Lysine(K)_iTRAQ[10]	[3] F6 and F9	1142/1134	1.156	0.910	0.879	1	Mascot
1956.09	1956.1061	0.0161	8	106	119	LLHEVQELTTEVEK	86	100	(N-term)_iTRAQ[0], Lysine(K)_iTRAQ[14]	[4] F7 and F10+11	436/428	1.129	0.731	1.119	1	Mascot

428	myosin light polypeptide 6 [Rattus norvegicus]	gi|155369650	18543.1	3	146	1.210	1.044	0.925	0.605	0.339	0.139	3	3	3	100

Peptide Information

Calc. Mass	Obsrv. Mass	± da	± ppm	Start Seq.	End Seq.	Sequence	Ion Score	C. I. %	Modification	Plate [#] Name	Gel Idx/Pos [4700 Sample Name]	iTRAQ Ratio 115/114*	iTRAQ Ratio 116/114*	iTRAQ Ratio 117/114*	Rank	Result Type
1169.6075	1169.5468	-0.0607	-52	14	21	EAFQLFDR	34	99.028	(N-term)_iTRAQ[0]	[5] F4	232/224	1.345	1.068	0.794	1	Mascot
1642.9374	1642.8345	-0.1029	-63	38	50	ALGQNPTNAEVLK	78	100	(N-term)_iTRAQ[0], Lysine(K)_iTRAQ[13]	[5] F4	131/123	0.704	0.732	0.894	1	Mascot
1688.7888	1688.7233	-0.0655	-39	82	94	DQGTYEDYVEGLR	34	98.982	(N-term)_iTRAQ[0]	[1] F3 030912	271/263	1.870	1.454	1.113	1	Mascot

429	epsin-1 [Rattus norvegicus]	gi|16923990	63382.9	3	146	0.976	0.875	1.010	0.092	0.075	0.138	3	3	3	100

Peptide Information

Calc. Mass	Obsrv. Mass	± da	± ppm	Start Seq.	End Seq.	Sequence	Ion Score	C. I. %	Modification	Plate [#] Name	Gel Idx/Pos [4700 Sample Name]	iTRAQ Ratio 115/114*	iTRAQ Ratio 116/114*	iTRAQ Ratio 117/114*	Rank	Result Type
1484.8029	1484.7587	-0.0442	-30	98	107	ENMYAVQTLK	50	99.976	(N-term)_iTRAQ[0], Lysine(K)_iTRAQ[10]	[7] F5 120912	191/183	1.080	0.876	0.843	1	Mascot
1492.8403	1492.8818	0.0415	28	238	247	LQMAIEESKR	28	95.689	(N-term)_iTRAQ[0], Lysine(K)_iTRAQ[9]	[3] F6 and F9	1179/1171	0.869	0.790	1.096	1	Mascot
1704.9166	1704.821	-0.0956	-56	12	23	NIVHNYSEAEIK	68	100	(N-term)_iTRAQ[0], Lysine(K)_iTRAQ[12]	[6] F8 110912	200/192	0.992	0.968	1.115	1	Mascot

430	hemoglobin alpha 2 chain [Rattus norvegicus]	gi|60678292	17286.1	2	146	0.323	0.336	0.348	0.009	0.039	0.042	2	2	2	100

Protein Group

hemoglobin subunit alpha-1/2 [Rattus norvegicus]	gi|6981010	17330.1

Peptide Information

Calc. Mass	Obsrv. Mass	± da	± ppm	Start Seq.	End Seq.	Sequence	Ion Score	C. I. %	Modification	Plate [#] Name	Gel Idx/Pos [4700 Sample Name]	iTRAQ Ratio 115/114*	iTRAQ Ratio 116/114*	iTRAQ Ratio 117/114*	Rank	Result Type
1716.8425	1716.7297	-0.1128	-66	18	32	IGGHGGEYGEEALQR	80	100	(N-term)_iTRAQ[0]	[6] F8 110912	188/180	0.315	0.375	0.311	1	Mascot
2585.3569	2585.4358	0.0789	31	70	91	AADHVEDLPGALSTLSDL HAHK	65	99.999	(N-term)_iTRAQ[0], Lysine(K)_iTRAQ[22]	[4] F7 and F10+11	1232/1224	0.333	0.301	0.391	1	Mascot

431	dnaJ homolog subfamily C member 5 [Mus musculus]	gi|7949027	24891.7	2	144	1.059	0.987	1.025	0.102	0.204	0.067	2	2	2	100

Peptide Information

Calc. Mass	Obsrv. Mass	± da	± ppm	Start Seq.	End Seq.	Sequence	Ion Score	C. I. %	Modification	Plate [#] Name	Gel Idx/Pos [4700 Sample Name]	iTRAQ Ratio 115/114*	iTRAQ Ratio 116/114*	iTRAQ Ratio 117/114*	Rank	Result Type
1798.9908	1798.9523	-0.0385	-21	59	72	EINNAHAILTDATK	81	100	(N-term)_iTRAQ[0], Lysine(K)_iTRAQ[14]	[4] F7 and F10+11	243/235	0.967	0.818	0.962	1	Mascot
2143.0791	2143.0232	-0.0559	-26	42	56	YHPDKNPDNPEAADK	63	99.999	(N-term)_iTRAQ[0], Lysine(K)_iTRAQ[5,15]	[2] F12 040912	117/109	1.161	1.191	1.092	1	Mascot

432	vesicular inhibitory amino acid transporter [Rattus norvegicus]	gi|13929106	61465.6	3	144	1.958	1.319	1.276	1.318	0.586	0.476	3	3	3	100

Peptide Information

Calc. Mass	Obsrv. Mass	± da	± ppm	Start Seq.	End Seq.	Sequence	Ion Score	C. I. %	Modification	Plate [#] Name	Gel Idx/Pos [4700 Sample Name]	iTRAQ Ratio 115/114*	iTRAQ Ratio 116/114*	iTRAQ Ratio 117/114*	Rank	Result Type
1072.6289	1072.5894	-0.0395	-37	299	304	FYIDVK	31	97.839	(N-term)_iTRAQ[0], Lysine(K)_iTRAQ[6]	[7] F5 120912	243/235	0.949	0.789	0.819	1	Mascot
1450.6349	1450.5891	-0.0458	-32	416	427	AFFPACYGGDGR	41	99.79	(N-term)_iTRAQ[0], MMTS (C)[6]	[5] F4	260/252	2.985	1.835	1.689	1	Mascot
2102.1001	2102.1855	0.0854	41	99	115	DQAVGAGGEFGGHDKP K	73	100	(N-term)_iTRAQ[0], Lysine(K)_iTRAQ[15, 17]	[4] F7 and F10+11	1041/1033	2.651	1.582	1.502	1	Mascot

433	ORF2 [Rattus norvegicus]	gi|202959	40509.2	3	144	1.008	1.325	1.024	0.033	0.201	0.456	4	4	4	100

Protein Group

Apolipoprotein E [Rattus norvegicus]	gi|37805241	37660.7
apolipoprotein E [Rattus norvegicus]	gi|295916	37934.8

	apolipoprotein E [Rattus sp.]	gi|913986	37766.7
	apolipoprotein E precursor [Rattus norvegicus]	gi|162287337	37650.6

Peptide Information

Calc. Mass	Obsrv. Mass	± da	± ppm	Start Seq.	End Seq.	Sequence	Ion Score	C. I. %	Modification	Plate [#] Name	Gel Idx/Pos [4700 Sample Name]	iTRAQ Ratio 115/114*	iTRAQ Ratio 116/114*	iTRAQ Ratio 117/114*	Rank	Result Type
1112.6548	1112.58	-0.0748	-67	215	223	LGPLVEQGR	29	97.004	(N-term)_iTRAQ[0]	[5] F4	137/129	1.012	1.125	0.591	1	Mascot
1307.7028	1307.6967	-0.0061	-5	309	316	QWANLMEK	44	99.891	(N-term)_iTRAQ[0], Lysine(K)_iTRAQ[8]	[3] F6 and F9	283/275	1.030	1.205	0.955	1	Mascot
1743.8884	1743.897	0.0086	5	111	124	ELEEQLGPVAEETR	69	100	(N-term)_iTRAQ[0]	[8] F13-15 and F1+2	517/509	0.955	1.622	1.617	1	Mascot
1743.8884	1743.9119	0.0235	13	111	124	ELEEQLGPVAEETR	71	100	(N-term)_iTRAQ[0]	[8] F13-15 and F1+2	520/512	1.038	1.400	1.204	1	Mascot

434	RNA-binding protein FUS [Rattus norvegicus]	gi|58865844	54987	3	144	0.765	1.268	0.919	0.120	0.213	0.058	3	3	3	100

Peptide Information

Calc. Mass	Obsrv. Mass	± da	± ppm	Start Seq.	End Seq.	Sequence	Ion Score	C. I. %	Modification	Plate [#] Name	Gel Idx/Pos [4700 Sample Name]	iTRAQ Ratio 115/114*	iTRAQ Ratio 116/114*	iTRAQ Ratio 117/114*	Rank	Result Type
1310.6991	1310.683	-0.0161	-12	342	350	AAIDWFDGK	60	99.997	(N-term)_iTRAQ[0], Lysine(K)_iTRAQ[9]	[3] F6 and F9	327/319	0.679	1.558	0.849	1	Mascot
2094.1455	2094.022	-0.1235	-59	326	341	LKGEATVSFDDPPSAK	55	99.993	(N-term)_iTRAQ[0], Lysine(K)_iTRAQ[2,16]	[6] F8 110912	234/226	0.703	1.071	0.931	1	Mascot
2541.1787	2541.3015	0.1228	48	442	465	APKPDGPGGGPGGSHMGGNYGDDR	28	96.504	(N-term)_iTRAQ[0], Lysine(K)_iTRAQ[3]	[4] F7 and F10+11	1038/1030	0.940	1.222	0.983	1	Mascot

435	cystatin C	gi|227013	14633.4	3	143	1.141	0.805	0.713	0.193	0.057	0.141	3	3	3	100

Protein Group

cystatin-C precursor [Rattus norvegicus]	gi|54234046	16763.6
unnamed protein product [Rattus norvegicus]	gi|736290	15366.8

Peptide Information

Calc. Mass	Obsrv. Mass	± da	± ppm	Start Seq.	End Seq.	Sequence	Ion Score	C. I. %	Modification	Plate [#] Name	Gel Idx/Pos [4700 Sample Name]	iTRAQ Ratio 115/114*	iTRAQ Ratio 116/114*	iTRAQ Ratio 117/114*	Rank	Result Type
1544.8206	1544.7791	-0.0415	-27	26	36	ALDFAVSEYNK	69	100	(N-term)_iTRAQ[0], Lysine(K)_iTRAQ[11]	[7] F5 120912	252/244	0.969	0.745	0.661	1	Mascot
1913.9689	1914.0045	0.0356	19	9	25	LLGAPQEADASEEGVQR	38	99.564	(N-term)_iTRAQ[0]	[8] F13-15 and F1+2	553/545	1.087	0.880	0.600	1	Mascot
2329.0742	2329.1562	0.082	35	76	93	SQTNLTNCPFHDQPHLMR	36	99.361	(N-term)_iTRAQ[0], MMTS (C)[8]	[4] F7 and F10+11	1195/1187	1.409	0.798	0.915	1	Mascot

436	protein NipSnap homolog 2 [Rattus norvegicus]	gi|62945328	35508.6	3	143	0.668	0.899	0.755	0.058	0.107	0.274	3	3	3	100

Peptide Information

Calc. Mass	Obsrv. Mass	± da	± ppm	Start Seq.	End Seq.	Sequence	Ion Score	C. I. %	Modification	Plate [#] Name	Gel Idx/Pos [4700 Sample Name]	iTRAQ Ratio 115/114*	iTRAQ Ratio 116/114*	iTRAQ Ratio 117/114*	Rank	Result Type
1299.6454	1299.577	-0.0684	-53	141	149	ENQEFVSFR	42	99.831	(N-term)_iTRAQ[0]	[5] F4	157/149	0.701	0.789	0.487	1	Mascot
1568.8304	1568.8915	0.0611	39	139	149	LRENQEFVSFR	44	99.901	(N-term)_iTRAQ[0]	[3] F6 and F9	1219/1211	0.715	1.038	0.934	1	Mascot
2397.2412	2397.3362	0.095	40	71	86	LQFHNVKPECLDAYNK	57	99.995	(N-term)_iTRAQ[0], Lysine(K)_iTRAQ[7,16], MMTS (C)[10]	[4] F7 and F10+11	1207/1199	0.594	0.889	0.945	1	Mascot

437	LEDGFB [Rattus norvegicus]	gi|33150808	45408.7	3	142	1.067	1.060	1.152	0.098	0.158	0.157	3	3	3	100

Protein Group

PC4 and SFRS1-interacting protein [Rattus norvegicus]	gi|28461167	71799.1

Peptide Information

Calc. Mass	Obsrv. Mass	± da	± ppm	Start Seq.	End Seq.	Sequence	Ion Score	C. I. %	Modification	Plate [#] Name	Gel Idx/Pos [4700 Sample Name]	iTRAQ Ratio 115/114*	iTRAQ Ratio 116/114*	iTRAQ Ratio 117/114*	Rank	Result Type
1682.9852	1683.0375	0.0523	31	4	14	DFKPGDLIFAK	32	98.276	(N-term)_iTRAQ[0], Lysine(K)_iTRAQ[3,1	[3] F6 and F9	1286/1278	0.966	0.886	1.143	1	Mascot

Calc. Mass	Obsrv. Mass	± da	± ppm	Start Seq.	End Seq.	Sequence	Ion Score	C. I. %	Modification	Plate [#] Name	Gel Idx/Pos [4700 Sample Name]	iTRAQ Ratio 115/114*	iTRAQ Ratio 116/114*	iTRAQ Ratio 117/114*	Rank	Result Type
									1]							
1998.1243	1998.0002	-0.1241	-62	25	39	VDEVPDGAVKPPTNK	58	99.996	(N-term)_iTRAQ[0], Lysine(K)_iTRAQ[10, 15]	[6] F8 110912	185/177	1.051	1.245	1.352	1	Mascot
2193.1675	2193.2437	0.0762	35	75	89	KGFNEGLWEIDNNPK	53	99.989	(N-term)_iTRAQ[0], Lysine(K)_iTRAQ[1,15]	[4] F7 and F10+11	1192/1184	1.195	1.080	0.989	1	Mascot

438	phosphatidylinositol transfer protein alpha isoform [Rattus norvegicus]	gi|8393962	36394.1	4	142	0.870	0.787	0.907	0.324	0.453	0.209	4	4	4	100

Peptide Information

Calc. Mass	Obsrv. Mass	± da	± ppm	Start Seq.	End Seq.	Sequence	Ion Score	C. I. %	Modification	Plate [#] Name	Gel Idx/Pos [4700 Sample Name]	iTRAQ Ratio 115/114*	iTRAQ Ratio 116/114*	iTRAQ Ratio 117/114*	Rank	Result Type
1219.6833	1219.6714	-0.0119	-10	203	209	WWGLQNK	38	99.644	(N-term)_iTRAQ[0], Lysine(K)_iTRAQ[7]	[4] F7 and F10+11	298/290	1.273	0.778	1.216	1	Mascot
1386.7141	1386.7302	0.0161	12	228	235	QLFCWLDK	44	99.903	(N-term)_iTRAQ[0], Lysine(K)_iTRAQ[8], MMTS (C)[4]	[7] F5 120912	421/413	1.017	1.565	0.999	1	Mascot
1386.7549	1386.6892	-0.0657	-47	97	105	TVITNEYMK	29	96.92	(N-term)_iTRAQ[0], Lysine(K)_iTRAQ[9]	[7] F5 120912	172/164	0.819	0.723	0.740	1	Mascot
1563.8074	1563.8092	0.0018	1	236	246	WVDLTMDDIRR	33	98.782	(N-term)_iTRAQ[0]	[4] F7 and F10+11	353/345	0.539	0.437	0.752	1	Mascot

439	glutamate decarboxylase 1 [Rattus norvegicus]	gi|8393406	73103.6	2	142	1.183	1.070	0.883	0.056	0.044	0.156	2	2	2	100

Protein Group

Glu decarboxylase	gi|227913	74389.2
glutamate decarboxylase [Rattus norvegicus]	gi|56186	73056.6

Peptide Information

Calc. Mass	Obsrv. Mass	± da	± ppm	Start Seq.	End Seq.	Sequence	Ion Score	C. I. %	Modification	Plate [#] Name	Gel Idx/Pos [4700 Sample Name]	iTRAQ Ratio 115/114*	iTRAQ Ratio 116/114*	iTRAQ Ratio 117/114*	Rank	Result Type
1494.8162	1494.7802	-0.036	-24	471	481	GTVGFENQINK	68	100	(N-term)_iTRAQ[0], Lysine(K)_iTRAQ[11]	[7] F5 120912	159/151	1.130	1.115	1.039	1	Mascot
1578.7534	1578.688	-0.0654	-41	440	450	QYDVSYDTGDK	74	100	(N-term)_iTRAQ[0], Lysine(K)_iTRAQ[11]	[7] F5 120912	137/129	1.238	1.028	0.750	1	Mascot

440	spectrin beta chain, erythrocyte [Rattus norvegicus]	gi|47058982	267282	4	142	1.347	1.226	1.278	0.573	0.704	1.116	6	6	6	100

Peptide Information

Calc. Mass	Obsrv. Mass	± da	± ppm	Start Seq.	End Seq.	Sequence	Ion Score	C. I. %	Modification	Plate [#] Name	Gel Idx/Pos [4700 Sample Name]	iTRAQ Ratio 115/114*	iTRAQ Ratio 116/114*	iTRAQ Ratio 117/114*	Rank	Result Type
1404.7206	1404.7928	0.0722	51	110	118	IHCLENVDK	31	98.123	(N-term)_iTRAQ[0], Lysine(K)_iTRAQ[9], MMTS (C)[3]	[4] F7 and F10+11	1137/1129	0.916	0.913	1.142	1	Mascot
1543.8479	1543.9353	0.0874	57	215	224	HRPDLIDFDK	28	95.649	(N-term)_iTRAQ[0], Lysine(K)_iTRAQ[10]	[8] F13-15 and F1+2	1505/1497	1.391	1.034	1.085	1	Mascot
1543.8479	1543.9387	0.0908	59	215	224	HRPDLIDFDK	36	99.331	(N-term)_iTRAQ[0], Lysine(K)_iTRAQ[10]	[8] F13-15 and F1+2	1171/1163	1.318	1.175	1.061	1	Mascot
1554.8074	1554.7385	-0.0689	-44	177	186	DALLLWCQMK	31	98.178	(N-term)_iTRAQ[0], Lysine(K)_iTRAQ[10], MMTS (C)[7]	[5] F4	394/386	1.364	0.935	1.012	1	Mascot
1554.8074	1554.7823	-0.0251	-16	177	186	DALLLWCQMK	39	99.696	(N-term)_iTRAQ[0], Lysine(K)_iTRAQ[10], MMTS (C)[7]	[7] F5 120912	482/474	2.711	3.327	4.912	1	Mascot
2108.0005	2107.9695	-0.031	-15	1878	1895	EQEVSAAWQALLDACAGR	37	99.5	(N-term)_iTRAQ[0], MMTS (C)[15]	[1] F3 030912	564/556	0.962	0.987	0.668	1	Mascot

441	gelsolin precursor [Rattus norvegicus]	gi|51854227	92388.3	3	140	1.548	0.989	0.799	0.528	0.351	0.303	3	3	3	100

Peptide Information

Calc. Mass	Obsrv. Mass	± da	± ppm	Start Seq.	End Seq.	Sequence	Ion Score	C. I. %	Modification	Plate [#] Name	Gel Idx/Pos [4700 Sample Name]	iTRAQ Ratio 115/114*	iTRAQ Ratio 116/114*	iTRAQ Ratio 117/114*	Rank	Result Type
1419.8192	1419.7949	-0.0243	-17	176	186	HVVPNEVVVQR	44	99.912	(N-term)_iTRAQ[0]	[4] F7 and F10+11	178/170	2.271	1.519	1.129	1	Mascot
2042.0116	2041.9426	-0.069	-34	146	160	EVQGFESSTFQGYFK	40	99.735	(N-term)_iTRAQ[0], Lysine(K)_iTRAQ[15]	[5] F4	264/256	1.467	0.792	0.520	1	Mascot
2098.0886	2097.9597	-0.1289	-61	712	726	DSQEEEKTEALTSAK	56	99.994	(N-term)_iTRAQ[0], Lysine(K)_iTRAQ[7,15]	[6] F8 110912	176/168	1.113	0.804	0.869	1	Mascot

442	casein kinase II subunit alpha [Rattus norvegicus]	gi|16758674	48595.2	3	140	0.964	1.041	0.860	0.455	0.091	0.442	3	3	3	100

Peptide Information

Calc. Mass	Obsrv. Mass	± da	± ppm	Start	End Sequence	Ion	C. I. %	Modification	Plate [#] Name	Gel Idx/Pos [4700	iTRAQ	iTRAQ	iTRAQ	Rank	Result Type

				Seq.	Seq.		Score				Sample Name]	Ratio 115/114*	Ratio 116/114*	Ratio 117/114*		
1901.9717	1901.951	-0.0207	-11	317	329	EAMEHPYFYTVVK	65	99.999	(N-term)_iTRAQ[0], Lysine(K)_iTRAQ[13]	[4] F7 and F10+11	298/290	1.635	1.172	1.207	1	Mascot
2017.964	2018.0303	0.0663	33	230	244	EPFFHGHDNYDQLVR	30	97.609	(N-term)_iTRAQ[0]	[4] F7 and F10+11	1173/1165	0.656	0.988	0.479	1	Mascot
2020.075	2020.0348	-0.0402	-20	108	122	TPALVFEHVNNTDFK	45	99.918	(N-term)_iTRAQ[0], Lysine(K)_iTRAQ[15]	[4] F7 and F10+11	334/326	0.834	0.973	1.098	1	Mascot

443 aminopeptidase-B [Rattus norvegicus] gi|1754515 77899.4 3 138 0.988 1.314 0.943 0.231 0.306 0.359 3 3 3 100

Protein Group

Arginyl aminopeptidase (aminopeptidase B) [Rattus norvegicus]	gi|38512104	77956.5
RecName: Full=Aminopeptidase B; Short=AP-B; AltName: Full=Arginine aminopeptidase; AltName: Full=Ar	gi|21903366	77972.5
aminopeptidase B [Rattus norvegicus]	gi|13592047	77725.4

Peptide Information

Calc. Mass	Obsrv. Mass	± da	± ppm	Start Seq.	End Seq.	Sequence	Ion Score	C. I. %	Modification	Plate [#] Name	Gel Idx/Pos [4700 Sample Name]	iTRAQ Ratio 115/114*	iTRAQ Ratio 116/114*	iTRAQ Ratio 117/114*	Rank	Result Type
1620.9346	1620.9927	0.0581	36	33	44	AFEILHLHLDLR	44	99.893	(N-term)_iTRAQ[0]	[4] F7 and F10+11	1304/1296	0.734	0.999	0.648	1	Mascot
1885.9324	1885.8103	-0.1221	-65	380	393	QHMDVSGEENPLNK	65	99.999	(N-term)_iTRAQ[0], Lysine(K)_iTRAQ[14]	[6] F8 110912	186/178	1.123	1.660	1.425	1	Mascot
2181.2878	2181.218	-0.0698	-32	161	175	KKPFVYTQGQAVLNR	29	97.197	(N-term)_iTRAQ[0], Lysine(K)_iTRAQ[1,2]	[2] F12 040912	257/249	1.168	1.369	0.909	1	Mascot

444 sorting and assembly machinery component 50 homolog [Rattus norvegicus] gi|51948454 55465.6 3 138 1.224 1.592 1.945 0.873 0.212 0.576 3 3 3 100

Peptide Information

Calc. Mass	Obsrv. Mass	± da	± ppm	Start Seq.	End Seq.	Sequence	Ion Score	C. I. %	Modification	Plate [#] Name	Gel Idx/Pos [4700 Sample Name]	iTRAQ Ratio 115/114*	iTRAQ Ratio 116/114*	iTRAQ Ratio 117/114*	Rank	Result Type
1634.8148	1634.7446	-0.0702	-43	210	221	GVSAEYSFPLCK	45	99.919	(N-term)_iTRAQ[0], Lysine(K)_iTRAQ[12], MMTS (C)[11]	[5] F4	283/275	1.343	1.733	2.624	1	Mascot
1793.9419	1794.0292	0.0873	49	44	57	DVVVQHVHFDGLER	52	99.983	(N-term)_iTRAQ[0]	[4] F7 and F10+11	1155/1147	2.249	1.746	2.012	1	Mascot
2086.1067	2086.0005	-0.1062	-51	281	297	VNQELAGYTGGDVSFIK	42	99.837	(N-term)_iTRAQ[0], Lysine(K)_iTRAQ[17]	[5] F4	248/240	0.607	1.334	1.393	1	Mascot

445 plasminogen activator inhibitor 1 RNA-binding protein [Rattus norvegicus] gi|57977275 47903.9 3 138 0.979 0.984 1.082 0.237 0.571 1.201 3 3 3 100

Protein Group

RecName: Full=Plasminogen activator inhibitor 1 RNA-binding protein; AltName: Full=PAI1 RNA-binding	gi|52783155	50007
hypothetical RNA binding protein RDA288 [Rattus norvegicus]	gi|18041977	47932

Peptide Information

Calc. Mass	Obsrv. Mass	± da	± ppm	Start Seq.	End Seq.	Sequence	Ion Score	C. I. %	Modification	Plate [#] Name	Gel Idx/Pos [4700 Sample Name]	iTRAQ Ratio 115/114*	iTRAQ Ratio 116/114*	iTRAQ Ratio 117/114*	Rank	Result Type
1301.7058	1301.6204	-0.0854	-66	40	52	EAGGGGVGGPGAK	31	97.824	(N-term)_iTRAQ[0], Lysine(K)_iTRAQ[13]	[5] F4	116/108	0.742	0.723	0.405	1	Mascot
1339.6814	1339.6687	-0.0127	-9	271	278	EMTLDEWK	28	96.263	(N-term)_iTRAQ[0], Lysine(K)_iTRAQ[8]	[3] F6 and F9	283/275	1.259	1.879	2.470	1	Mascot
2232.0957	2232.0291	-0.0666	-30	17	32	FDQLFDDESDPFEVLK	79	100	(N-term)_iTRAQ[0], Lysine(K)_iTRAQ[16]	[1] F3 030912	491/483	1.005	0.702	1.268	1	Mascot

446 voltage-dependent calcium channel subunit alpha-2/delta-3 [Rattus norvegicus] gi|28212256 133946 2 138 1.102 0.888 1.055 0.169 0.047 0.072 2 2 2 100

Peptide Information

Calc. Mass	Obsrv. Mass	± da	± ppm	Start Seq.	End Seq.	Sequence	Ion Score	C. I. %	Modification	Plate [#] Name	Gel Idx/Pos [4700 Sample Name]	iTRAQ Ratio 115/114*	iTRAQ Ratio 116/114*	iTRAQ Ratio 117/114*	Rank	Result Type
1466.8253	1466.7985	-0.0268	-18	44	54	LWASAFGGEIK	59	99.997	(N-term)_iTRAQ[0], Lysine(K)_iTRAQ[11]	[7] F5 120912	351/343	0.956	0.844	0.988	1	Mascot
2238.238	2238.1611	-0.0769	-34	731	747	INLFVGAEQLTNQDFLK	79	100	(N-term)_iTRAQ[0], Lysine(K)_iTRAQ[17]	[1] F3 030912	488/480	1.271	0.935	1.127	1	Mascot

447 RecName: Full=Fatty aldehyde dehydrogenase; gi|399362 59026.5 2 138 1.100 1.024 1.073 0.154 0.033 0.028 2 2 2 100

AltName: Full=Aldehyde dehydrogenase 4; AltName: Full=A

Peptide Information

Calc. Mass	Obsrv. Mass	± da	± ppm	Start Seq.	End Seq.	Sequence	Ion Score	C. I. %	Modification	Plate [#] Name	Gel Idx/Pos [4700 Sample Name]	iTRAQ Ratio 115/114*	iTRAQ Ratio 116/114*	iTRAQ Ratio 117/114*	Rank	Result Type
1259.6517	1259.6101	-0.0416	-33	267	274	DFYGENVK	57	99.995	(N-term)_iTRAQ[0], Lysine(K)_iTRAQ[8]	[3] F6 and F9	189/181	0.964	1.057	1.101	1	Mascot
1890.1283	1890.0614	-0.0669	-35	38	51	EKDILAAIAADLSK	81	100	(N-term)_iTRAQ[0], Lysine(K)_iTRAQ[2,14]	[6] F8 110912	491/483	1.254	0.992	1.046	1	Mascot

448	profilin [Rattus norvegicus]	gi|1628436	16556.5	3	138	1.020	0.847	0.908	0.094	0.175	0.046	3	3	3	100

Protein Group

profilin-1 [Mus musculus] gi|6755040 16526.5

Peptide Information

Calc. Mass	Obsrv. Mass	± da	± ppm	Start Seq.	End Seq.	Sequence	Ion Score	C. I. %	Modification	Plate [#] Name	Gel Idx/Pos [4700 Sample Name]	iTRAQ Ratio 115/114*	iTRAQ Ratio 116/114*	iTRAQ Ratio 117/114*	Rank	Result Type
1299.575	1299.621	0.046	35	128	136	CYEMASHLR	38	99.565	(N-term)_iTRAQ[0], MMTS (C)[1]	[3] F6 and F9	1213/1205	0.901	0.998	0.881	1	Mascot
1501.826	1501.7321	-0.0939	-63	27	38	DSPSVWAAVPGK	60	99.997	(N-term)_iTRAQ[0], Lysine(K)_iTRAQ[12]	[5] F4	179/171	1.098	0.935	0.873	1	Mascot
1905.1306	1905.1505	0.0199	10	39	54	TFVSITPAEVGVLVGK	40	99.775	(N-term)_iTRAQ[0], Lysine(K)_iTRAQ[16]	[8] F13-15 and F1+2	378/370	1.074	0.651	0.973	1	Mascot

449	ALG-2 interacting protein 1 [Rattus norvegicus]	gi|6319138	48062.1	2	137	1.239	1.179	0.948	0.286	0.502	0.013	2	2	2	100

Peptide Information

Calc. Mass	Obsrv. Mass	± da	± ppm	Start Seq.	End Seq.	Sequence	Ion Score	C. I. %	Modification	Plate [#] Name	Gel Idx/Pos [4700 Sample Name]	iTRAQ Ratio 115/114*	iTRAQ Ratio 116/114*	iTRAQ Ratio 117/114*	Rank	Result Type
1604.8643	1604.9114	0.0471	29	161	171	NIQVSHQEFSK	79	100	(N-term)_iTRAQ[0], Lysine(K)_iTRAQ[11]	[3] F6 and F9	1133/1125	1.007	0.827	0.961	1	Mascot
2183.1958	2183.147	-0.0488	-22	188	204	NLATAYDNFVELVANLK	58	99.996	(N-term)_iTRAQ[0], Lysine(K)_iTRAQ[17]	[1] F3 030912	572/564	1.524	1.681	0.935	1	Mascot

450	F-actin-capping protein subunit beta isoform 1 [Homo sapiens]	gi|4826659	34343.8	3	137	0.926	0.895	0.828	0.243	0.212	0.276	4	4	4	100

Peptide Information

Calc. Mass	Obsrv. Mass	± da	± ppm	Start Seq.	End Seq.	Sequence	Ion Score	C. I. %	Modification	Plate [#] Name	Gel Idx/Pos [4700 Sample Name]	iTRAQ Ratio 115/114*	iTRAQ Ratio 116/114*	iTRAQ Ratio 117/114*	Rank	Result Type
1364.608	1364.5695	-0.0385	-28	58	66	DYLLCDYNR	32	98.488	(N-term)_iTRAQ[0], MMTS (C)[5]	[1] F3 030912	324/316	0.623	0.870	0.573	1	Mascot
1459.8042	1459.7694	-0.0348	-24	226	235	STLNEIYFGK	55	99.992	(N-term)_iTRAQ[0], Lysine(K)_iTRAQ[10]	[7] F5 120912	252/244	1.015	0.661	0.721	1	Mascot
1985.0338	1984.9003	-0.1335	-67	95	108	KLEVEANNAFDQYR	35	99.208	(N-term)_iTRAQ[0], Lysine(K)_iTRAQ[1]	[6] F8 110912	282/274	1.020	0.929	0.912	1	Mascot
1985.0338	1985.1022	0.0684	34	95	108	KLEVEANNAFDQYR	51	99.981	(N-term)_iTRAQ[0], Lysine(K)_iTRAQ[1]	[3] F6 and F9	1237/1229	1.139	1.202	1.247	1	Mascot

451	ADP-ribosylation factor 5 [Homo sapiens]	gi|4502209	22337.8	3	136	1.042	0.949	1.358	0.040	0.405	0.160	3	3	3	100

Peptide Information

Calc. Mass	Obsrv. Mass	± da	± ppm	Start Seq.	End Seq.	Sequence	Ion Score	C. I. %	Modification	Plate [#] Name	Gel Idx/Pos [4700 Sample Name]	iTRAQ Ratio 115/114*	iTRAQ Ratio 116/114*	iTRAQ Ratio 117/114*	Rank	Result Type
1375.8229	1375.7595	-0.0634	-46	20	30	ILMVGLDAAGK	67	100	(N-term)_iTRAQ[0], Lysine(K)_iTRAQ[11]	[5] F4	250/242	1.041	1.326	1.428	1	Mascot
1915.9272	1915.8524	-0.0748	-39	60	73	NICFTVWDVGGQDK	32	98.615	(N-term)_iTRAQ[0], Lysine(K)_iTRAQ[14], MMTS (C)[3]	[5] F4	348/340	0.994	1.110	1.164	1	Mascot
1963.9714	1963.8757	-0.0957	-49	128	142	QDMPNAMPVSELTDK	36	99.436	(N-term)_iTRAQ[0], Lysine(K)_iTRAQ[15]	[1] F3 030912	268/260	1.091	0.580	1.508	1	Mascot

452	Arfgap1 protein [Rattus norvegicus]	gi|47477812	37148.7	1	136	1.346	1.330	1.928	0.000	0.000	0.000	1	1	1	100

Protein Group

ADP-ribosylation factor GTPase-activating protein 1 [Rattus norvegicus] gi|21489979 49967.6

Peptide Information

Calc. Mass	Obsrv. Mass	± da	± ppm	Start Seq.	End Seq.	Sequence	Ion Score	C. I. %	Modification	Plate [#] Name	Gel Idx/Pos [4700 Sample Name]	iTRAQ Ratio	iTRAQ Ratio	iTRAQ Ratio	Rank	Result Type

												iTRAQ Ratio 115/114*	iTRAQ Ratio 116/114*	iTRAQ Ratio 117/114*		
2976.3818	2976.2964	-0.0854	-29	196	220	EDDFLNSAMSSLYSGWS SFTTGASK	136	100	(N-term)_iTRAQ[0], Lysine(K)_iTRAQ[25]	[1] F3 030912	546/538	1.346	1.330	1.928	1	Mascot

453 26S proteasome non-ATPase regulatory subunit 3 [Rattus norvegicus] | gi|56605666 | 65785.9 | 3 | 136 | 1.060 | 0.981 | 1.126 | 0.220 | 0.291 | 0.506 | 3 | 3 | 3 | 100

Peptide Information

Calc. Mass	Obsrv. Mass	± da	± ppm	Start Seq.	End Seq.	Sequence	Ion Score	C. I. %	Modification	Plate [#] Name	Gel Idx/Pos [4700 Sample Name]	iTRAQ Ratio 115/114*	iTRAQ Ratio 116/114*	iTRAQ Ratio 117/114*	Rank	Result Type
1395.7366	1395.7146	-0.022	-16	511	519	EQQDLEFAK	35	99.232	(N-term)_iTRAQ[0], Lysine(K)_iTRAQ[9]	[7] F5 120912	159/151	1.313	1.413	1.641	1	Mascot
1805.869	1805.769	-0.1	-55	270	283	SVFPEQANNNEWAR	40	99.765	(N-term)_iTRAQ[0]	[1] F3 030912	247/239	0.829	0.787	0.680	1	Mascot
2085.1438	2085.2371	0.0933	45	62	76	ELDTVTLEDIKEHVR	60	99.998	(N-term)_iTRAQ[0], Lysine(K)_iTRAQ[11]	[4] F7 and F10+11	1241/1233	1.095	0.850	1.281	1	Mascot

454 voltage dependent anion channel [Rattus norvegicus] | gi|4558732 | 33897.8 | 3 | 136 | 0.832 | 0.909 | 0.902 | 0.164 | 0.189 | 0.101 | 3 | 3 | 3 | 100

Protein Group

Voltage-dependent anion channel 3 [Rattus norvegicus]	gi|38512108	34832.9
voltage dependent anion channel [Rattus norvegicus]	gi|4558734	34028.8
voltage-dependent anion channel 3 [Rattus norvegicus]	gi|8810249	34859.9
voltage-dependent anion-selective channel protein 3 [Rattus norvegicus]	gi|13786204	34991.1

Peptide Information

Calc. Mass	Obsrv. Mass	± da	± ppm	Start Seq.	End Seq.	Sequence	Ion Score	C. I. %	Modification	Plate [#] Name	Gel Idx/Pos [4700 Sample Name]	iTRAQ Ratio 115/114*	iTRAQ Ratio 116/114*	iTRAQ Ratio 117/114*	Rank	Result Type
1146.6227	1146.6111	-0.0116	-10	12	19	GYGFGMVK	59	99.997	(N-term)_iTRAQ[0], Lysine(K)_iTRAQ[8]	[3] F6 and F9	246/238	0.860	1.169	0.937	1	Mascot
1604.8156	1604.8041	-0.0115	-7	155	165	LCQNNFALGYK	48	99.957	(N-term)_iTRAQ[0], Lysine(K)_iTRAQ[11], MMTS (C)[2]	[7] F5 120912	312/304	0.657	0.742	1.004	1	Mascot
2196.0818	2195.9783	-0.1035	-47	66	81	WNTDNTLGTEISWENK	29	96.568	(N-term)_iTRAQ[0], Lysine(K)_iTRAQ[16]	[5] F4	250/242	1.017	0.865	0.781	1	Mascot

455 cytosolic branched chain aminotransferase [Rattus norvegicus] | gi|13374912 | 49236.1 | 3 | 135 | 1.499 | 0.914 | 1.029 | 0.542 | 0.199 | 0.375 | 3 | 3 | 3 | 100

Protein Group

branched-chain-amino-acid aminotransferase, cytosolic [Rattus norvegicus]	gi|8392971	50366.7

Peptide Information

Calc. Mass	Obsrv. Mass	± da	± ppm	Start Seq.	End Seq.	Sequence	Ion Score	C. I. %	Modification	Plate [#] Name	Gel Idx/Pos [4700 Sample Name]	iTRAQ Ratio 115/114*	iTRAQ Ratio 116/114*	iTRAQ Ratio 117/114*	Rank	Result Type
1109.6075	1109.5249	-0.0826	-74	382	389	LTDIQYGR	28	96.314	(N-term)_iTRAQ[0]	[5] F4	130/122	2.298	0.851	1.407	1	Mascot
1888.9194	1888.9189	-0.0005	0	323	337	HITMDDLSTALEENR	31	98.096	(N-term)_iTRAQ[0]	[4] F7 and F10+11	330/322	1.120	0.751	0.673	1	Mascot
1953.0215	1952.9385	-0.083	-42	305	318	QSILELGEEWGEFK	76	100	(N-term)_iTRAQ[0], Lysine(K)_iTRAQ[14]	[5] F4	358/350	1.309	1.196	1.150	1	Mascot

456 brain alpha-tropomyosin (TMBr-3) [Rattus norvegicus] | gi|207359 | 26492.1 | 3 | 134 | 1.023 | 1.085 | 1.102 | 0.028 | 0.143 | 0.302 | 3 | 3 | 3 | 100

Protein Group

brain alpha-tropomyosin (TMBr-1) [Rattus norvegicus]	gi|207355	38011.5

Peptide Information

Calc. Mass	Obsrv. Mass	± da	± ppm	Start Seq.	End Seq.	Sequence	Ion Score	C. I. %	Modification	Plate [#] Name	Gel Idx/Pos [4700 Sample Name]	iTRAQ Ratio 115/114*	iTRAQ Ratio 116/114*	iTRAQ Ratio 117/114*	Rank	Result Type
1419.8127	1419.7928	-0.0199	-14	60	68	MEIQEIQLK	39	99.703	(N-term)_iTRAQ[0], Lysine(K)_iTRAQ[9]	[7] F5 120912	263/255	1.018	1.260	1.489	1	Mascot
1614.8597	1614.9342	0.0745	46	137	145	EDKYEEEIK	38	99.64	(N-term)_iTRAQ[0], Lysine(K)_iTRAQ[3,9]	[4] F7 and F10+11	1054/1046	0.993	0.930	0.822	1	Mascot
2416.1978	2416.2817	0.0839	35	171	188	SIDDLEDQLYHQLEQNRR	57	99.995	(N-term)_iTRAQ[0]	[4] F7 and F10+11	1286/1278	1.059	1.089	1.094	1	Mascot

457	drebrin E [Rattus norvegicus]	gi|3192363	76602.3	3	134	0.891	1.014	1.112	0.166	0.174	0.045	4	4	4	100

Protein Group

drebrin [Rattus norvegicus]	gi|13591936	81670.6

Peptide Information

Calc. Mass	Obsrv. Mass	± da	± ppm	Start Seq.	End Seq.	Sequence	Ion Score	C. I. %	Modification	Plate [#] Name	Gel Idx/Pos [4700 Sample Name]	iTRAQ Ratio 115/114*	iTRAQ Ratio 116/114*	iTRAQ Ratio 117/114*	Rank	Result Type
1353.7048	1353.6696	-0.0352	-26	178	185	EQFWEQAK	42	99.851	(N-term)_iTRAQ[0], Lysine(K)_iTRAQ[8]	[3] F6 and F9	229/221	0.695	0.931	1.134	1	Mascot
1941.9415	1941.9458	0.0043	2	395	411	VAAAPQVWAGCAEEPPR	30	97.402	(N-term)_iTRAQ[0], MMTS (C)[11]	[8] F13-15 and F1+2	446/438	0.978	0.833	1.058	1	Mascot
2138.0974	2137.9573	-0.1401	-66	150	165	LREDENAEPVGTTYQK	46	99.944	(N-term)_iTRAQ[0], Lysine(K)_iTRAQ[16]	[6] F8 110912	175/167	1.098	1.270	1.173	1	Mascot
2138.0974	2138.0486	-0.0488	-23	150	165	LREDENAEPVGTTYQK	62	99.999	(N-term)_iTRAQ[0], Lysine(K)_iTRAQ[16]	[4] F7 and F10+11	173/165	0.843	1.074	1.087	1	Mascot

458	mitochondrial glutamate carrier 1 [Rattus norvegicus]	gi|62078785	27009.1	3	134	0.906	1.014	1.001	0.083	0.190	0.356	3	3	3	100

Peptide Information

Calc. Mass	Obsrv. Mass	± da	± ppm	Start Seq.	End Seq.	Sequence	Ion Score	C. I. %	Modification	Plate [#] Name	Gel Idx/Pos [4700 Sample Name]	iTRAQ Ratio 115/114*	iTRAQ Ratio 116/114*	iTRAQ Ratio 117/114*	Rank	Result Type
1253.5745	1253.551	-0.0235	-19	59	67	SEGYFGMYR	31	97.844	(N-term)_iTRAQ[0]	[7] F5 120912	215/207	1.017	1.233	1.209	1	Mascot
1595.7533	1595.6803	-0.073	-46	45	55	MYASMSDCLIK	39	99.674	(N-term)_iTRAQ[0], Lysine(K)_iTRAQ[11], MMTS (C)[8]	[5] F4	282/274	0.822	1.041	1.273	1	Mascot
1836.7997	1836.7488	-0.0509	-28	165	179	GVNEDTYSGFLDCAR	65	99.999	(N-term)_iTRAQ[0], MMTS (C)[13]	[1] F3 030912	354/346	0.889	0.812	0.652	1	Mascot

459	copine-9 [Rattus norvegicus]	gi|68163393	66607.1	4	133	0.870	1.088	0.921	0.393	0.563	1.804	4	4	4	100

Peptide Information

Calc. Mass	Obsrv. Mass	± da	± ppm	Start Seq.	End Seq.	Sequence	Ion Score	C. I. %	Modification	Plate [#] Name	Gel Idx/Pos [4700 Sample Name]	iTRAQ Ratio 115/114*	iTRAQ Ratio 116/114*	iTRAQ Ratio 117/114*	Rank	Result Type
1264.7174	1264.689	-0.0284	-22	489	497	DIVQFVPFR	33	98.821	(N-term)_iTRAQ[0]	[1] F3 030912	375/367	0.488	0.832	1.052	1	Mascot
1287.6858	1287.6431	-0.0427	-33	168	176	SDPFLVFYR	28	95.94	(N-term)_iTRAQ[0]	[5] F4	307/299	1.304	1.873	3.160	1	Mascot
1875.8633	1875.8489	-0.0144	-8	229	243	DGSHDFIGEFTTSYR	28	95.816	(N-term)_iTRAQ[0]	[4] F7 and F10+11	321/313	0.820	0.648	0.162	1	Mascot
1897.0482	1897.0997	0.0515	27	70	80	KFVLDYFFEEK	44	99.906	(N-term)_iTRAQ[0], Lysine(K)_iTRAQ[1,11]	[4] F7 and F10+11	1308/1300	1.099	1.390	1.340	1	Mascot

460	phosphatidate cytidylyltransferase 2 [Rattus norvegicus]	gi|16758454	54342.5	2	133	1.479	1.051	1.485	0.855	0.509	0.327	2	2	2	100

Peptide Information

Calc. Mass	Obsrv. Mass	± da	± ppm	Start Seq.	End Seq.	Sequence	Ion Score	C. I. %	Modification	Plate [#] Name	Gel Idx/Pos [4700 Sample Name]	iTRAQ Ratio 115/114*	iTRAQ Ratio 116/114*	iTRAQ Ratio 117/114*	Rank	Result Type
2093.0259	2092.8486	-0.1773	-85	11	25	EDAPPEDKESESEAK	59	99.997	(N-term)_iTRAQ[0], Lysine(K)_iTRAQ[8,15]	[6] F8 110912	88/80	0.938	0.708	1.217	1	Mascot
2232.2637	2232.2075	-0.0562	-25	311	327	LQEYNIPGVIQSLVGWK	74	100	(N-term)_iTRAQ[0], Lysine(K)_iTRAQ[17]	[1] F3 030912	546/538	2.334	1.560	1.812	1	Mascot

461	RecName: Full=Neuronal migration protein doublecortin	gi|59797481	45519.6	2	132	1.083	1.303	1.495	0.305	0.015	0.461	2	2	2	100

Peptide Information

Calc. Mass	Obsrv. Mass	± da	± ppm	Start Seq.	End Seq.	Sequence	Ion Score	C. I. %	Modification	Plate [#] Name	Gel Idx/Pos [4700 Sample Name]	iTRAQ Ratio 115/114*	iTRAQ Ratio 116/114*	iTRAQ Ratio 117/114*	Rank	Result Type
1558.8588	1558.8287	-0.0301	-19	141	151	NVNPNWSVNVK	52	99.983	(N-term)_iTRAQ[0], Lysine(K)_iTRAQ[11]	[7] F5 120912	191/183	0.845	1.318	1.142	1	Mascot
2191.1855	2191.2388	0.0533	24	203	219	TAHSFEQVLTDITEAIK	80	100	(N-term)_iTRAQ[0], Lysine(K)_iTRAQ[17]	[4] F7 and F10+11	535/527	1.388	1.288	1.956	1	Mascot

462	adapter molecule crk [Rattus norvegicus]	gi|9506515	35599.2	2	131	0.934	0.957	1.222	0.059	0.040	0.224	2	2	2	100

Peptide Information

Calc. Mass	Obsrv. Mass	± da	± ppm	Start Seq.	End Seq.	Sequence	Ion Score	C. I. %	Modification	Plate [#] Name	Gel Idx/Pos [4700 Sample Name]	iTRAQ Ratio 115/114*	iTRAQ Ratio 116/114*	iTRAQ Ratio 117/114*	Rank	Result Type
2273.1951	2273.1484	-0.0467	-21	89	105	IGDQEFDSLPALLEFYK	66	99.999	(N-term)_iTRAQ[0], Lysine(K)_iTRAQ[17]	[1] F3 030912	529/521	0.878	0.998	1.446	1	Mascot
2536.2439	2536.1538	-0.0901	-36	163	179	DKPEEQWWNAEDSEGK	65	99.999	(N-term)_iTRAQ[0],	[2] F12 040912	253/245	0.993	0.919	1.032	1	Mascot

					R			Lysine(K)_iTRAQ[2,16]									

463	Sideroflexin 3 [Rattus norvegicus]	gi|38304020	37340.8	4	130	0.910	1.243	1.165	0.055	0.570	0.220	4	4	4	100

Protein Group

sideroflexin-3 [Rattus norvegicus]	gi|12621120	37513.9

Peptide Information

Calc. Mass	Obsrv. Mass	± da	± ppm	Start Seq.	End Seq.	Sequence	Ion Score	C. I. %	Modification	Plate [#] Name	Gel Idx/Pos [4700 Sample Name]	iTRAQ Ratio 115/114*	iTRAQ Ratio 116/114*	iTRAQ Ratio 117/114*	Rank	Result Type
1253.6399	1253.5841	-0.0558	-45	15	23	WDQSTFLGR	43	99.875	(N-term)_iTRAQ[0]	[5] F4	204/196	0.979	2.179	1.552	1	Mascot
1263.6606	1263.6863	0.0257	20	26	34	HFFTVTDPR	28	96.041	(N-term)_iTRAQ[0]	[3] F6 and F9	1193/1185	0.890	1.181	0.994	1	Mascot
1755.9362	1755.9437	0.0075	4	199	213	ELQVGIPVTDEAGQR	29	96.797	(N-term)_iTRAQ[0]	[8] F13-15 and F1+2	509/501	0.940	1.233	1.141	1	Mascot
1916.9276	1916.8901	-0.0375	-20	72	85	YVYDSAFHPDTGEK	31	97.859	(N-term)_iTRAQ[0], Lysine(K)_iTRAQ[14]	[4] F7 and F10+11	236/228	0.837	0.752	1.047	1	Mascot

464	glycogen synthase kinase-3 alpha [Rattus norvegicus]	gi|8393496	54631.3	1	129	2.238	1.691	0.968	0.000	0.000	0.000	1	1	1	100

Peptide Information

Calc. Mass	Obsrv. Mass	± da	± ppm	Start Seq.	End Seq.	Sequence	Ion Score	C. I. %	Modification	Plate [#] Name	Gel Idx/Pos [4700 Sample Name]	iTRAQ Ratio 115/114*	iTRAQ Ratio 116/114*	iTRAQ Ratio 117/114*	Rank	Result Type
2721.271	2721.1392	-0.1318	-48	19	50	TSSFAEPGGGGGGGGG GPGGSASGPGGTGGGK	129	100	(N-term)_iTRAQ[0], Lysine(K)_iTRAQ[32]	[1] F3 030912	157/149	2.238	1.691	0.968	1	Mascot

465	RecName: Full=Glycyl-tRNA synthetase; AltName: Full=Diadenosine tetraphosphate synthetase; Short=AP	gi|81889021	79164.9	3	129	1.544	1.177	1.019	0.241	0.091	0.270	3	3	3	100

Peptide Information

Calc. Mass	Obsrv. Mass	± da	± ppm	Start Seq.	End Seq.	Sequence	Ion Score	C. I. %	Modification	Plate [#] Name	Gel Idx/Pos [4700 Sample Name]	iTRAQ Ratio 115/114*	iTRAQ Ratio 116/114*	iTRAQ Ratio 117/114*	Rank	Result Type
1745.9877	1745.9235	-0.0642	-37	501	513	TFFSFPAVVAPFK	71	100	(N-term)_iTRAQ[0], Lysine(K)_iTRAQ[13]	[1] F3 030912	503/495	1.284	1.276	1.403	1	Mascot
1829.0181	1829.093	0.0749	41	621	632	YPLFEGQETGKK	28	96.381	(N-term)_iTRAQ[0], Lysine(K)_iTRAQ[11, 12]	[4] F7 and F10+11	1123/1115	1.829	1.198	0.802	1	Mascot
2832.425	2832.5002	0.0752	27	243	262	EFTMAEIEHFVDPTEKDH PK	31	98.052	(N-term)_iTRAQ[0], Lysine(K)_iTRAQ[16, 20]	[4] F7 and F10+11	1280/1272	1.568	1.065	0.941	1	Mascot

466	pyruvate dehydrogenase E1 alpha form 1 subunit [Rattus rattus]	gi|57657	47178.9	3	128	0.901	0.860	1.030	0.154	0.086	0.258	3	3	3	100

Peptide Information

Calc. Mass	Obsrv. Mass	± da	± ppm	Start Seq.	End Seq.	Sequence	Ion Score	C. I. %	Modification	Plate [#] Name	Gel Idx/Pos [4700 Sample Name]	iTRAQ Ratio 115/114*	iTRAQ Ratio 116/114*	iTRAQ Ratio 117/114*	Rank	Result Type
1104.6411	1104.6127	-0.0284	-26	379	385	GANQWIK	40	99.748	(N-term)_iTRAQ[0], Lysine(K)_iTRAQ[7]	[4] F7 and F10+11	189/181	0.930	0.780	1.194	1	Mascot
1464.8005	1464.7484	-0.0521	-36	278	288	GPILMELQTYR	51	99.982	(N-term)_iTRAQ[0]	[1] F3 030912	370/362	0.732	0.978	0.751	1	Mascot
1555.8816	1555.9187	0.0371	24	46	58	LEEGPPVTTVLTR	37	99.504	(N-term)_iTRAQ[0]	[8] F13-15 and F1+2	486/478	1.074	0.833	1.218	1	Mascot

467	isocitrate dehydrogenase [NADP] cytoplasmic [Rattus norvegicus]	gi|13928690	52456.3	3	127	0.996	0.995	1.221	0.231	0.067	0.119	3	3	3	100

Peptide Information

Calc. Mass	Obsrv. Mass	± da	± ppm	Start Seq.	End Seq.	Sequence	Ion Score	C. I. %	Modification	Plate [#] Name	Gel Idx/Pos [4700 Sample Name]	iTRAQ Ratio 115/114*	iTRAQ Ratio 116/114*	iTRAQ Ratio 117/114*	Rank	Result Type
1202.7759	1202.786	0.0101	8	21	27	IIWELIK	31	97.819	(N-term)_iTRAQ[0], Lysine(K)_iTRAQ[7]	[7] F5 120912	423/415	1.248	1.088	1.347	1	Mascot
1740.1145	1740.1315	0.017	10	120	132	LVTGWVKPIIIGR	31	98.101	(N-term)_iTRAQ[0], Lysine(K)_iTRAQ[7]	[4] F7 and F10+11	411/403	0.755	0.969	1.255	1	Mascot
1878.0385	1878.1086	0.0701	37	223	233	FKDIFQEIYDK	66	99.999	(N-term)_iTRAQ[0], Lysine(K)_iTRAQ[2,11]	[4] F7 and F10+11	1288/1280	1.047	0.934	1.077	1	Mascot

468	ras-related protein Rab-15 [Rattus norvegicus]	gi|38454238	26849.6	2	127	0.751	1.252	1.193	0.011	0.194	0.316	2	2	2	100

Peptide Information

Calc. Mass	Obsrv. Mass	± da	± ppm	Start Seq.	End Seq.	Sequence	Ion Score	C. I. %	Modification	Plate [#] Name	Gel Idx/Pos [4700 Sample Name]	iTRAQ Ratio 115/114*	iTRAQ Ratio 116/114*	iTRAQ Ratio 117/114*	Rank	Result Type

Calc. Mass	Obsrv. Mass	± da	± ppm	Start Seq.	End Seq.	Sequence	Ion Score	C. I. %	Modification	Plate [#] Name	Gel Idx/Pos [4700 Sample Name]	iTRAQ Ratio 115/114*	iTRAQ Ratio 116/114*	iTRAQ Ratio 117/114*	Rank	Result Type
1359.8457	1359.778	-0.0677	-50	11	21	LLLIGDSGVGK	58	99.996	(N-term)_iTRAQ[0], Lysine(K)_iTRAQ[11]	[5] F4	229/221	0.740	1.446	1.508	1	Mascot
1460.7618	1460.6924	-0.0694	-48	59	69	IQIWDTAGQER	70	100	(N-term)_iTRAQ[0]	[5] F4	197/189	0.762	1.084	0.943	1	Mascot

469	40S ribosomal protein S19 [Mus musculus]	gi|12963511	18381.2	3	127	1.073	1.147	1.349	0.678	0.453	0.821	3	3	3	100

Peptide Information

Calc. Mass	Obsrv. Mass	± da	± ppm	Start Seq.	End Seq.	Sequence	Ion Score	C. I. %	Modification	Plate [#] Name	Gel Idx/Pos [4700 Sample Name]	iTRAQ Ratio 115/114*	iTRAQ Ratio 116/114*	iTRAQ Ratio 117/114*	Rank	Result Type
1746.0537	1746.1111	0.0574	33	28	38	LKVPEWVDTVK	58	99.996	(N-term)_iTRAQ[0], Lysine(K)_iTRAQ[2,11]	[4] F7 and F10+11	1230/1222	1.063	1.017	0.925	1	Mascot
1847.8724	1847.8073	-0.0651	-35	44	56	ELAPYDENWFYTR	38	99.616	(N-term)_iTRAQ[0]	[1] F3 030912	371/363	0.591	0.821	1.007	1	Mascot
2257.1287	2257.075	-0.0537	-24	42	56	HKELAPYDENWFYTR	31	97.984	(N-term)_iTRAQ[0], Lysine(K)_iTRAQ[2]	[2] F12 040912	346/338	1.963	1.806	2.637	1	Mascot

470	guanine nucleotide-binding protein G(q) subunit alpha [Rattus norvegicus]	gi|13591957	45661.6	3	127	0.777	0.952	0.953	0.321	0.279	0.229	3	3	3	100

Peptide Information

Calc. Mass	Obsrv. Mass	± da	± ppm	Start Seq.	End Seq.	Sequence	Ion Score	C. I. %	Modification	Plate [#] Name	Gel Idx/Pos [4700 Sample Name]	iTRAQ Ratio 115/114*	iTRAQ Ratio 116/114*	iTRAQ Ratio 117/114*	Rank	Result Type
1069.6655	1069.6462	-0.0193	-18	339	345	FVFAAVK	28	96.167	(N-term)_iTRAQ[0], Lysine(K)_iTRAQ[7]	[7] F5 120912	262/254	0.501	0.744	0.925	1	Mascot
1740.9418	1740.8655	-0.0763	-44	121	133	VSAFENPYVDAIK	49	99.971	(N-term)_iTRAQ[0], Lysine(K)_iTRAQ[13]	[5] F4	252/244	1.166	1.356	1.258	1	Mascot
1885.8314	1885.7748	-0.0566	-30	134	147	SLWNDPGIQECYDR	49	99.971	(N-term)_iTRAQ[0], MMTS (C)[11]	[1] F3 030912	367/359	0.803	0.856	0.744	1	Mascot

471	protein kinase C gamma type [Mus musculus]	gi|6755080	84938.8	3	126	0.825	1.028	0.616	0.058	0.380	1.024	3	3	3	100

Peptide Information

Calc. Mass	Obsrv. Mass	± da	± ppm	Start Seq.	End Seq.	Sequence	Ion Score	C. I. %	Modification	Plate [#] Name	Gel Idx/Pos [4700 Sample Name]	iTRAQ Ratio 115/114*	iTRAQ Ratio 116/114*	iTRAQ Ratio 117/114*	Rank	Result Type
1739.9048	1739.8768	-0.028	-16	167	181	APTSDEIHITVGEAR	66	99.999	(N-term)_iTRAQ[0]	[3] F6 and F9	227/219	0.764	1.158	1.407	1	Mascot
2034.0286	2033.9635	-0.0651	-32	253	268	NDFMGAMSFGVSELLK	29	96.631	(N-term)_iTRAQ[0], Lysine(K)_iTRAQ[16]	[1] F3 030912	519/511	0.901	1.403	1.067	1	Mascot
3179.5923	3179.4775	-0.1148	-36	660	686	LVLASIDQADFQGFTYVNPDFVHPDAR	30	97.794	(N-term)_iTRAQ[0]	[1] F3 030912	492/484	0.815	0.668	0.155	1	Mascot

472	rabphilin-3A [Rattus norvegicus]	gi|19424162	83679.6	2	125	0.747	0.698	0.756	0.006	0.103	0.085	2	2	2	100

Peptide Information

Calc. Mass	Obsrv. Mass	± da	± ppm	Start Seq.	End Seq.	Sequence	Ion Score	C. I. %	Modification	Plate [#] Name	Gel Idx/Pos [4700 Sample Name]	iTRAQ Ratio 115/114*	iTRAQ Ratio 116/114*	iTRAQ Ratio 117/114*	Rank	Result Type
1597.7651	1597.6785	-0.0866	-54	523	534	GMALYEEEQVER	50	99.977	(N-term)_iTRAQ[0]	[1] F3 030912	228/220	0.741	0.608	0.679	1	Mascot
2530.1934	2530.2668	0.0734	29	470	488	ISVCDEDKFGHNEFIGETR	75	100	(N-term)_iTRAQ[0], Lysine(K)_iTRAQ[8], MMTS (C)[4]	[4] F7 and F10+11	1217/1209	0.753	0.801	0.840	1	Mascot

473	transaldolase [Rattus norvegicus]	gi|42476292	42329.8	2	125	1.077	0.778	0.927	0.169	0.087	0.306	2	2	2	100

Protein Group

transaldolase [Rattus norvegicus]	gi|12002054	42345.8

Peptide Information

Calc. Mass	Obsrv. Mass	± da	± ppm	Start Seq.	End Seq.	Sequence	Ion Score	C. I. %	Modification	Plate [#] Name	Gel Idx/Pos [4700 Sample Name]	iTRAQ Ratio 115/114*	iTRAQ Ratio 116/114*	iTRAQ Ratio 117/114*	Rank	Result Type
1787.8997	1787.9702	0.0705	39	296	307	WLHNEDQMAVEK	64	99.999	(N-term)_iTRAQ[0], Lysine(K)_iTRAQ[12]	[3] F6 and F9	1208/1200	0.931	0.700	0.697	1	Mascot
1872.0715	1872.0354	-0.0361	-19	193	204	ILDWHVANTDKK	62	99.998	(N-term)_iTRAQ[0], Lysine(K)_iTRAQ[11, 12]	[2] F12 040912	281/273	1.246	0.865	1.233	1	Mascot

474	tropomyosin alpha-1 chain isoform g [Rattus norvegicus]	gi|78000199	32188	3	125	1.212	1.497	1.354	0.367	0.909	0.614	3	3	3	100

Protein Group

tropomyosin alpha isoform [Rattus norvegicus]	gi|14134101	32288.1
tropomyosin alpha-1 chain isoform f [Rattus norvegicus]	gi|14192925	32473.1

	tropomyosin alpha-1 chain isoform h [Rattus norvegicus]	gi|78000201	31992.9

Peptide Information

Calc. Mass	Obsrv. Mass	± da	± ppm	Start Seq.	End Seq.	Sequence	Ion Score	C. I. %	Modification	Plate [#] Name	Gel Idx/Pos [4700 Sample Name]	iTRAQ Ratio 115/114*	iTRAQ Ratio 116/114*	iTRAQ Ratio 117/114*	Rank	Result Type
1419.72	1419.6377	-0.0823	-58	43	54	ETAEADVASLNR	50	99.976	(N-term)_iTRAQ[0]	[1] F3 030912	183/175	1.762	2.861	2.026	1	Mascot
1419.8127	1419.7928	-0.0199	-14	105	113	MEIQEIQLK	39	99.703	(N-term)_iTRAQ[0], Lysine(K)_iTRAQ[9]	[7] F5 120912	263/255	1.018	1.260	1.489	1	Mascot
1614.8597	1614.9342	0.0745	46	182	190	EDKYEEEIK	38	99.64	(N-term)_iTRAQ[0], Lysine(K)_iTRAQ[3,9]	[4] F7 and F10+11	1054/1046	0.993	0.930	0.822	1	Mascot

475	neuronal membrane glycoprotein M6-b [Rattus norvegicus]	gi|20301986	31274	2	125	1.074	0.908	0.839	0.236	0.063	0.104	2	2	2	100

Peptide Information

Calc. Mass	Obsrv. Mass	± da	± ppm	Start Seq.	End Seq.	Sequence	Ion Score	C. I. %	Modification	Plate [#] Name	Gel Idx/Pos [4700 Sample Name]	iTRAQ Ratio 115/114*	iTRAQ Ratio 116/114*	iTRAQ Ratio 117/114*	Rank	Result Type
1778.9839	1778.9343	-0.0496	-28	191	203	QYGIIPWNAFPGK	73	100	(N-term)_iTRAQ[0], Lysine(K)_iTRAQ[13]	[5] F4	311/303	0.881	0.849	0.943	1	Mascot
2468.1643	2468.0979	-0.0664	-27	1	19	MKPAMETAAEENTEQSQER	52	99.983	(N-term)_iTRAQ[0], Lysine(K)_iTRAQ[2]	[4] F7 and F10+11	182/174	1.309	0.971	0.746	1	Mascot

476	calretinin [Rattus norvegicus]	gi|16758892	35367.2	3	125	0.684	1.142	1.149	0.236	0.232	0.324	3	3	3	100

Peptide Information

Calc. Mass	Obsrv. Mass	± da	± ppm	Start Seq.	End Seq.	Sequence	Ion Score	C. I. %	Modification	Plate [#] Name	Gel Idx/Pos [4700 Sample Name]	iTRAQ Ratio 115/114*	iTRAQ Ratio 116/114*	iTRAQ Ratio 117/114*	Rank	Result Type
1411.7678	1411.7048	-0.063	-45	124	133	SGYIEANELK	41	99.788	(N-term)_iTRAQ[0], Lysine(K)_iTRAQ[10]	[7] F5 120912	168/160	0.857	1.460	1.248	1	Mascot
1540.7914	1540.7341	-0.0573	-37	234	244	EMNIQQLTTYR	33	98.627	(N-term)_iTRAQ[0]	[1] F3 030912	284/276	0.450	0.935	0.820	1	Mascot
1601.9877	1601.9294	-0.0583	-36	179	189	LLPVQENFLLK	51	99.982	(N-term)_iTRAQ[0], Lysine(K)_iTRAQ[11]	[5] F4	315/307	0.829	1.092	1.481	1	Mascot

477	ras-related protein Rap-2b precursor [Mus musculus]	gi|13386338	22404.4	3	124	1.420	1.359	1.319	0.453	0.484	0.227	3	3	3	100

Peptide Information

Calc. Mass	Obsrv. Mass	± da	± ppm	Start Seq.	End Seq.	Sequence	Ion Score	C. I. %	Modification	Plate [#] Name	Gel Idx/Pos [4700 Sample Name]	iTRAQ Ratio 115/114*	iTRAQ Ratio 116/114*	iTRAQ Ratio 117/114*	Rank	Result Type
1462.6975	1462.6495	-0.048	-33	32	41	YDPTIEDFYR	37	99.504	(N-term)_iTRAQ[0]	[1] F3 030912	309/301	0.978	0.991	1.054	1	Mascot
1492.8131	1492.7517	-0.0614	-41	151	162	ASVDELFAEIVR	48	99.962	(N-term)_iTRAQ[0]	[1] F3 030912	476/468	1.542	2.052	1.465	1	Mascot
1954.9967	1954.9115	-0.0852	-44	118	132	VDLEGEREVSYGEGK	39	99.716	(N-term)_iTRAQ[0], Lysine(K)_iTRAQ[15]	[6] F8 110912	206/198	1.898	1.235	1.485	1	Mascot

478	polyubiquitin [Rattus norvegicus]	gi|1675359	5470	2	124	1.181	0.965	1.272	0.081	0.080	0.041	2	2	2	100

Peptide Information

Calc. Mass	Obsrv. Mass	± da	± ppm	Start Seq.	End Seq.	Sequence	Ion Score	C. I. %	Modification	Plate [#] Name	Gel Idx/Pos [4700 Sample Name]	iTRAQ Ratio 115/114*	iTRAQ Ratio 116/114*	iTRAQ Ratio 117/114*	Rank	Result Type
1053.6377	1053.646	0.0083	8	1	6	MQIFVK	35	99.254	(N-term)_iTRAQ[0], Lysine(K)_iTRAQ[6]	[3] F6 and F9	262/254	1.104	0.892	1.232	1	Mascot
2076.1321	2076.0603	-0.0718	-35	12	27	TITLEVEPXDTIENVK	89	100	(N-term)_iTRAQ[0], Lysine(K)_iTRAQ[16]	[1] F3 030912	308/300	1.262	1.045	1.313	1	Mascot

479	heat shock protein 75 kDa, mitochondrial precursor [Rattus norvegicus]	gi|84781723	87367.5	3	124	1.113	1.309	1.114	0.562	0.859	0.314	3	3	3	100

Peptide Information

Calc. Mass	Obsrv. Mass	± da	± ppm	Start Seq.	End Seq.	Sequence	Ion Score	C. I. %	Modification	Plate [#] Name	Gel Idx/Pos [4700 Sample Name]	iTRAQ Ratio 115/114*	iTRAQ Ratio 116/114*	iTRAQ Ratio 117/114*	Rank	Result Type
1657.8881	1657.9056	0.0175	11	391	404	GVVDSEDIPLNLSR	39	99.693	(N-term)_iTRAQ[0]	[8] F13-15 and F1+2	578/570	1.457	2.128	1.058	1	Mascot
1743.8098	1743.8048	-0.005	-3	307	318	DISEFQHEEFYR	32	98.413	(N-term)_iTRAQ[0]	[4] F7 and F10+11	270/262	1.514	1.613	1.544	1	Mascot
2041.0344	2040.973	-0.0614	-30	576	589	LSEKETEELMAWMR	53	99.987	(N-term)_iTRAQ[0], Lysine(K)_iTRAQ[4]	[6] F8 110912	459/451	0.624	0.653	0.847	1	Mascot

480	heterogeneous nuclear ribonucleoprotein A1 isoform a [Homo sapiens]	gi|4504445	37005.2	3	123	1.008	1.270	1.007	0.454	0.413	0.254	5	5	5	100

Protein Group

Hnrpa1 protein [Rattus norvegicus]	gi|38328245	36428.9
heterogeneous nuclear ribonucleoprotein A1 [Rattus norvegicus]	gi|8393547	37067.1

Peptide Information

Calc. Mass	Obsrv. Mass	± da	± ppm	Start Seq.	End Seq.	Sequence	Ion Score	C. I. %	Modification	Plate [#] Name	Gel Idx/Pos [4700 Sample Name]	iTRAQ Ratio 115/114*	iTRAQ Ratio 116/114*	iTRAQ Ratio 117/114*	Rank	Result Type
1021.6655	1021.6119	-0.0536	-52	107	113	IFVGGIK	33	98.734	(N-term)_iTRAQ[0], Lysine(K)_iTRAQ[7]	[7] F5 120912	218/210	1.244	1.380	0.951	1	Mascot
1021.6655	1021.6606	-0.0049	-5	107	113	IFVGGIK	36	99.385	(N-term)_iTRAQ[0], Lysine(K)_iTRAQ[7]	[3] F6 and F9	262/254	1.231	1.890	1.123	1	Mascot
1293.8629	1293.9136	0.0507	39	106	113	KIFVGGIK	39	99.697	(N-term)_iTRAQ[0], Lysine(K)_iTRAQ[1,8]	[4] F7 and F10+11	1131/1123	0.547	0.843	0.855	1	Mascot
1337.6624	1337.619	-0.0434	-32	123	130	DYFEQYGK	42	99.836	(N-term)_iTRAQ[0], Lysine(K)_iTRAQ[8]	[7] F5 120912	186/178	0.805	1.032	0.773	1	Mascot
1337.6624	1337.6331	-0.0293	-22	123	130	DYFEQYGK	48	99.963	(N-term)_iTRAQ[0], Lysine(K)_iTRAQ[8]	[3] F6 and F9	227/219	1.546	1.459	1.466	1	Mascot

481	valyl-tRNA synthetase [Rattus norvegicus]	gi|158186770	150370.2	2	123	0.866	0.946	1.132	0.358	0.044	0.279	2	2	2	100

Peptide Information

Calc. Mass	Obsrv. Mass	± da	± ppm	Start Seq.	End Seq.	Sequence	Ion Score	C. I. %	Modification	Plate [#] Name	Gel Idx/Pos [4700 Sample Name]	iTRAQ Ratio 115/114*	iTRAQ Ratio 116/114*	iTRAQ Ratio 117/114*	Rank	Result Type
1753.8167	1753.7189	-0.0978	-56	24	39	YGEAGDGPGWGGPHPR	40	99.751	(N-term)_iTRAQ[0]	[6] F8 110912	195/187	0.613	0.904	0.908	1	Mascot
1859.0121	1858.9156	-0.0965	-52	215	229	SVTQQPGSEITAPQK	83	100	(N-term)_iTRAQ[0], Lysine(K)_iTRAQ[15]	[5] F4	106/98	1.224	0.990	1.411	1	Mascot

482	platelet-activating factor acetylhydrolase IB subunit alpha [Mus musculus]	gi|7305363	52143.6	3	123	1.774	1.393	1.365	0.404	0.093	0.415	3	3	3	100

Peptide Information

Calc. Mass	Obsrv. Mass	± da	± ppm	Start Seq.	End Seq.	Sequence	Ion Score	C. I. %	Modification	Plate [#] Name	Gel Idx/Pos [4700 Sample Name]	iTRAQ Ratio 115/114*	iTRAQ Ratio 116/114*	iTRAQ Ratio 117/114*	Rank	Result Type
1560.7091	1560.6453	-0.0638	-41	134	144	VWDYETGDFER	40	99.749	(N-term)_iTRAQ[0]	[5] F4	209/201	1.338	1.316	0.941	1	Mascot
1603.7502	1603.6826	-0.0676	-42	176	186	LWDFQGFECIR	40	99.737	(N-term)_iTRAQ[0], MMTS (C)[9]	[5] F4	391/383	1.920	1.347	1.712	1	Mascot
1996.028	1996.1696	0.1416	71	34	46	EAELDMNEELDKK	45	99.914	(N-term)_iTRAQ[0], Lysine(K)_iTRAQ[12, 13]	[3] F6 and F9	1183/1175	2.172	1.525	1.581	1	Mascot

483	NADH dehydrogenase [ubiquinone] iron-sulfur protein 6, mitochondrial precursor [Rattus norvegicus]	gi|9506913	14065.4	2	123	0.883	0.924	0.901	0.195	0.129	0.030	2	2	2	100

Peptide Information

Calc. Mass	Obsrv. Mass	± da	± ppm	Start Seq.	End Seq.	Sequence	Ion Score	C. I. %	Modification	Plate [#] Name	Gel Idx/Pos [4700 Sample Name]	iTRAQ Ratio 115/114*	iTRAQ Ratio 116/114*	iTRAQ Ratio 117/114*	Rank	Result Type
1654.9751	1655.0461	0.071	43	91	100	VYINLDKETK	45	99.927	(N-term)_iTRAQ[0], Lysine(K)_iTRAQ[7,1 0]	[4] F7 and F10+11	1122/1114	1.078	1.053	0.932	1	Mascot
1699.885	1699.8687	-0.0163	-10	76	90	IIACDGGGGALGHPK	78	100	(N-term)_iTRAQ[0], Lysine(K)_iTRAQ[15], MMTS (C)[4]	[4] F7 and F10+11	262/254	0.723	0.810	0.872	1	Mascot

484	T-complex protein 1 subunit alpha [Rattus norvegicus]	gi|6981642	66643.8	3	123	1.000	0.937	0.996	0.331	0.249	0.328	3	3	3	100

Peptide Information

Calc. Mass	Obsrv. Mass	± da	± ppm	Start Seq.	End Seq.	Sequence	Ion Score	C. I. %	Modification	Plate [#] Name	Gel Idx/Pos [4700 Sample Name]	iTRAQ Ratio 115/114*	iTRAQ Ratio 116/114*	iTRAQ Ratio 117/114*	Rank	Result Type
1291.713	1291.6649	-0.0481	-37	434	443	EQLAIAEFAR	28	95.968	(N-term)_iTRAQ[0]	[1] F3 030912	298/290	0.971	0.866	1.062	1	Mascot
1555.7737	1555.8257	0.052	33	469	480	AFHNEAQVNPER	51	99.979	(N-term)_iTRAQ[0]	[3] F6 and F9	1101/1093	0.715	0.736	0.684	1	Mascot
1679.0002	1678.9573	-0.0429	-26	485	496	WIGLDLVHGKPR	44	99.91	(N-term)_iTRAQ[0], Lysine(K)_iTRAQ[10]	[2] F12 040912	351/343	1.438	1.290	1.361	1	Mascot

485	cell adhesion-like molecule [Rattus norvegicus]	gi|203246	39945.4	2	123	1.200	0.821	0.828	0.133	0.309	0.235	2	2	2	100

Protein Group

opioid-binding protein/cell adhesion molecule precursor [Rattus norvegicus]	gi|16758710	40907

Peptide Information

Calc. Mass	Obsrv. Mass	± da	± ppm	Start Seq.	End Seq.	Sequence	Ion Score	C. I. %	Modification	Plate [#] Name	Gel Idx/Pos [4700 Sample Name]	iTRAQ Ratio 115/114*	iTRAQ Ratio 116/114*	iTRAQ Ratio 117/114*	Rank	Result Type
2346.1599	2346.0898	-0.0701	-30	169	186	EGQGFVSEDEYLEISDIK	52	99.986	(N-term)_iTRAQ[0], Lysine(K)_iTRAQ[18]	[1] F3 030912	365/357	1.081	0.596	0.645	1	Mascot
2448.1992	2448.1401	-0.0591	-24	233	251	GILSCEASAVPMAEFQWFK	70	100	(N-term)_iTRAQ[0], Lysine(K)_iTRAQ[19], MMTS (C)[5]	[1] F3 030912	538/530	1.333	1.130	1.063	1	Mascot

486	Chain A, Crystal Structure Of The Catalytic Core Of Inositol 1,4,5- Trisphosphate 3-Kinase	gi|55670081	33692	3	121	0.972	0.975	0.793	0.043	0.513	0.202	3	3	3	100

Peptide Information

Calc. Mass	Obsrv. Mass	± da	± ppm	Start Seq.	End Seq.	Sequence	Ion Score	C. I. %	Modification	Plate [#] Name	Gel Idx/Pos [4700 Sample Name]	iTRAQ Ratio 115/114*	iTRAQ Ratio 116/114*	iTRAQ Ratio 117/114*	Rank	Result Type
1386.7025	1386.6519	-0.0506	-36	196	205	DTLEISDFFR	31	97.974	(N-term)_iTRAQ[0]	[1] F3 030912	414/406	0.934	1.340	0.822	1	Mascot
2066.9937	2066.8916	-0.1021	-49	106	122	XLAVDPEAPTEEEHAQR	58	99.996	(N-term)_iTRAQ[0]	[7] F5 120912	182/174	1.032	0.536	0.591	1	Mascot
2086.0889	2086.0994	0.0105	5	172	186	VFEEFXQGDAEVLKR	32	98.592	(N-term)_iTRAQ[0], Lysine(K)_iTRAQ[14]	[4] F7 and F10+11	410/402	0.953	1.288	1.026	1	Mascot

487	protein phosphatase 1F [Rattus norvegicus]	gi|28461153	51572.2	2	121	0.772	0.678	0.663	0.364	0.094	0.180	2	2	2	100

Peptide Information

Calc. Mass	Obsrv. Mass	± da	± ppm	Start Seq.	End Seq.	Sequence	Ion Score	C. I. %	Modification	Plate [#] Name	Gel Idx/Pos [4700 Sample Name]	iTRAQ Ratio 115/114*	iTRAQ Ratio 116/114*	iTRAQ Ratio 117/114*	Rank	Result Type
1947.8427	1947.7426	-0.1001	-51	97	110	LPEQEEEEEEEEER	82	100	(N-term)_iTRAQ[0]	[1] F3 030912	171/163	1.136	0.595	0.843	1	Mascot
2103.9438	2103.8167	-0.1271	-60	96	110	RLPEQEEEEEEEEER	39	99.677	(N-term)_iTRAQ[0]	[6] F8 110912	148/140	0.525	0.772	0.521	1	Mascot

488	RSP29 [Rattus norvegicus]	gi|2459803	32422.1	2	121	1.083	0.836	1.203	0.194	0.579	0.092	3	3	3	100

Peptide Information

Calc. Mass	Obsrv. Mass	± da	± ppm	Start Seq.	End Seq.	Sequence	Ion Score	C. I. %	Modification	Plate [#] Name	Gel Idx/Pos [4700 Sample Name]	iTRAQ Ratio 115/114*	iTRAQ Ratio 116/114*	iTRAQ Ratio 117/114*	Rank	Result Type
1641.7716	1641.6835	-0.0881	-54	144	154	FYEGTADEMYK	43	99.885	(N-term)_iTRAQ[0], Lysine(K)_iTRAQ[11]	[5] F4	165/157	0.942	1.105	1.125	1	Mascot
1914.9464	1914.8634	-0.083	-43	231	246	TVQQHAGETDPVTTMR	78	100	(N-term)_iTRAQ[0]	[3] F6 and F9	166/158	1.365	1.321	1.334	1	Mascot
1914.9464	1914.8685	-0.0779	-41	231	246	TVQQHAGETDPVTTMR	27	95.609	(N-term)_iTRAQ[0]	[7] F5 120912	137/129	0.987	0.400	1.161	1	Mascot

489	alcohol dehydrogenase [NADP+] [Rattus norvegicus]	gi|13591894	39837.1	3	121	0.670	1.126	0.640	0.297	0.800	0.394	3	3	3	100

Peptide Information

Calc. Mass	Obsrv. Mass	± da	± ppm	Start Seq.	End Seq.	Sequence	Ion Score	C. I. %	Modification	Plate [#] Name	Gel Idx/Pos [4700 Sample Name]	iTRAQ Ratio 115/114*	iTRAQ Ratio 116/114*	iTRAQ Ratio 117/114*	Rank	Result Type
1403.833	1403.8065	-0.0265	-19	14	23	MPLIGLGTWK	29	96.722	(N-term)_iTRAQ[0], Lysine(K)_iTRAQ[10]	[7] F5 120912	393/385	0.399	2.364	0.427	1	Mascot
1649.772	1649.7098	-0.0622	-38	313	325	DAGHPLYPFNDPY	31	97.869	(N-term)_iTRAQ[0]	[1] F3 030912	313/305	0.845	0.893	0.490	1	Mascot
2474.2886	2474.1875	-0.1011	-41	270	287	ILQNIQVFDFTFSPEEMK	61	99.998	(N-term)_iTRAQ[0], Lysine(K)_iTRAQ[18]	[1] F3 030912	528/520	0.892	0.676	1.256	1	Mascot

490	Na+/Cl(-)-dependent neurotransmitter transporter [Rattus sp.]	gi|545078	86417.5	2	120	0.966	1.253	1.078	0.360	0.053	0.492	2	2	2	100

Protein Group

sodium-dependent neutral amino acid transporter SLC6A17 [Rattus norvegicus]	gi|74271849	86401.5

Peptide Information

Calc. Mass	Obsrv. Mass	± da	± ppm	Start Seq.	End Seq.	Sequence	Ion Score	C. I. %	Modification	Plate [#] Name	Gel Idx/Pos [4700 Sample Name]	iTRAQ Ratio 115/114*	iTRAQ Ratio 116/114*	iTRAQ Ratio 117/114*	Rank	Result Type
1549.7917	1549.7451	-0.0466	-30	680	692	VPSEAPSPMPTHR	64	99.999	(N-term)_iTRAQ[0]	[3] F6 and F9	170/162	1.327	1.202	1.570	1	Mascot
2247.1138	2247.0625	-0.0513	-23	50	66	VAEEELDAEDRPAWNSK	56	99.993	(N-term)_iTRAQ[0], Lysine(K)_iTRAQ[17]	[4] F7 and F10+11	246/238	0.704	1.307	0.740	1	Mascot

491	protein IMPACT [Rattus norvegicus]	gi|58866042	40715.3	3	119	0.729	1.487	1.123	0.964	0.118	0.254	3	3	3	100

Peptide Information

Calc. Mass	Obsrv. Mass	± da	± ppm	Start Seq.	End Seq.	Sequence	Ion Score	C. I. %	Modification	Plate [#] Name	Gel Idx/Pos [4700 Sample Name]	iTRAQ Ratio	iTRAQ Ratio	iTRAQ Ratio	Rank	Result Type

												115/114*	116/114*	117/114*		
1867.0211	1866.9432	-0.0779	-42	96	108	NIGESILYQWVEK	28	95.883	(N-term)_iTRAQ[0], Lysine(K)_iTRAQ[13]	[5] F4	358/350	1.357	1.345	1.378	1	Mascot
2040.923	2040.8364	-0.0866	-42	228	245	QTFLQDSEDDGETAAGG R	45	99.913	(N-term)_iTRAQ[0]	[1] F3 030912	213/205	1.290	1.618	1.210	1	Mascot
2544.3066	2544.2134	-0.0932	-37	158	180	ISESAPEAEELPPIAHGAP ITDR	47	99.952	(N-term)_iTRAQ[0]	[1] F3 030912	289/281	0.222	1.509	0.850	1	Mascot

492	profilin-2 [Rattus norvegicus]	gi|13540707	17964.9	2	119	1.245	1.078	1.101	0.118	0.139	0.185	2	2	2	100

Protein Group

RecName: Full=Profilin-2; AltName: Full=Profilin II	gi|20178018	16709.2

Peptide Information

Calc. Mass	Obsrv. Mass	± da	± ppm	Start Seq.	End Seq.	Sequence	Ion Score	C. I. %	Modification	Plate [#] Name	Gel Idx/Pos [4700 Sample Name]	iTRAQ Ratio 115/114*	iTRAQ Ratio 116/114*	iTRAQ Ratio 117/114*	Rank	Result Type
1180.7373	1180.7063	-0.031	-26	109	116	VLVFVMGK	46	99.942	(N-term)_iTRAQ[0], Lysine(K)_iTRAQ[8]	[7] F5 120912	345/337	1.363	0.955	0.943	1	Mascot
1578.7996	1578.7158	-0.0838	-53	92	105	SQGGEPTYNVAVGR	73	100	(N-term)_iTRAQ[0]	[1] F3 030912	170/162	1.137	1.217	1.286	1	Mascot

493	Chain A, Crystal Structure Of The Enth Domain Of Rat Epsin 1	gi|8569615	19183.9	2	119	1.035	0.920	0.970	0.045	0.047	0.146	2	2	2	100

Peptide Information

Calc. Mass	Obsrv. Mass	± da	± ppm	Start Seq.	End Seq.	Sequence	Ion Score	C. I. %	Modification	Plate [#] Name	Gel Idx/Pos [4700 Sample Name]	iTRAQ Ratio 115/114*	iTRAQ Ratio 116/114*	iTRAQ Ratio 117/114*	Rank	Result Type
1484.8029	1484.7587	-0.0442	-30	87	96	ENXYAVQTLK	50	99.976	(N-term)_iTRAQ[0], Lysine(K)_iTRAQ[10]	[7] F5 120912	191/183	1.080	0.876	0.843	1	Mascot
1704.9166	1704.821	-0.0956	-56	1	12	NIVHNYSEAEIK	68	100	(N-term)_iTRAQ[0], Lysine(K)_iTRAQ[12]	[6] F8 110912	200/192	0.992	0.968	1.115	1	Mascot

494	Rieske Fe-S protein precursor [Rattus norvegicus]	gi|206681	30351	2	118	1.090	1.119	1.138	0.353	0.189	0.499	4	4	4	100

Protein Group

cytochrome b-c1 complex subunit Rieske, mitochondrial [Rattus norvegicus]	gi|57114330	32106.9

Peptide Information

Calc. Mass	Obsrv. Mass	± da	± ppm	Start Seq.	End Seq.	Sequence	Ion Score	C. I. %	Modification	Plate [#] Name	Gel Idx/Pos [4700 Sample Name]	iTRAQ Ratio 115/114*	iTRAQ Ratio 116/114*	iTRAQ Ratio 117/114*	Rank	Result Type
1730.9044	1730.9371	0.0327	19	165	178	EIDQEAAVEVSQLR	51	99.979	(N-term)_iTRAQ[0]	[8] F13-15 and F1+2	535/527	1.347	1.120	0.884	1	Mascot
1730.9044	1730.9425	0.0381	22	165	178	EIDQEAAVEVSQLR	85	100	(N-term)_iTRAQ[0]	[8] F13-15 and F1+2	486/478	0.775	0.873	0.735	1	Mascot
1730.9044	1730.9501	0.0457	26	165	178	EIDQEAAVEVSQLR	31	98.182	(N-term)_iTRAQ[0]	[8] F13-15 and F1+2	588/580	0.890	1.336	1.406	1	Mascot
2637.4807	2637.4082	-0.0725	-27	35	59	ESLSGQAATRPLVATVGL NVPASVR	33	98.771	(N-term)_iTRAQ[0]	[1] F3 030912	332/324	1.522	1.200	1.836	1	Mascot

495	T-complex protein 1 subunit delta [Rattus norvegicus]	gi|33414505	64240.7	2	118	0.836	1.031	0.993	0.120	0.052	0.002	2	2	2	100

Peptide Information

Calc. Mass	Obsrv. Mass	± da	± ppm	Start Seq.	End Seq.	Sequence	Ion Score	C. I. %	Modification	Plate [#] Name	Gel Idx/Pos [4700 Sample Name]	iTRAQ Ratio 115/114*	iTRAQ Ratio 116/114*	iTRAQ Ratio 117/114*	Rank	Result Type
1294.7174	1294.6517	-0.0657	-51	80	89	QMQVLHPAAR	41	99.807	(N-term)_iTRAQ[0]	[6] F8 110912	158/150	0.731	0.982	0.995	1	Mascot
1744.9844	1745.0116	0.0272	16	307	319	DALSDLALHFLNK	78	100	(N-term)_iTRAQ[0], Lysine(K)_iTRAQ[13]	[4] F7 and F10+11	475/467	0.956	1.083	0.990	1	Mascot

496	neurocan core protein precursor [Rattus norvegicus]	gi|13928904	141778.9	2	118	0.770	1.299	1.513	0.499	0.186	0.494	3	3	3	100

Peptide Information

Calc. Mass	Obsrv. Mass	± da	± ppm	Start Seq.	End Seq.	Sequence	Ion Score	C. I. %	Modification	Plate [#] Name	Gel Idx/Pos [4700 Sample Name]	iTRAQ Ratio 115/114*	iTRAQ Ratio 116/114*	iTRAQ Ratio 117/114*	Rank	Result Type
1537.8135	1537.7529	-0.0606	-39	256	268	ELGGEVFYVGPAR	66	99.999	(N-term)_iTRAQ[0]	[1] F3 030912	301/293	1.150	1.525	1.536	1	Mascot
1537.8135	1537.8452	0.0317	21	256	268	ELGGEVFYVGPAR	32	98.533	(N-term)_iTRAQ[0]	[8] F13-15 and F1+2	621/613	0.381	1.100	1.062	1	Mascot
2217.0122	2216.9785	-0.0337	-15	1096	1111	DFQWTDNTGLQYENWR	52	99.985	(N-term)_iTRAQ[0]	[1] F3 030912	373/365	1.043	1.307	2.122	1	Mascot

497	Chain A, Rat Procathepsin B	gi|1942645	38523.2	3	118	1.245	0.978	1.126	0.157	0.235	0.430	3	3	3	100

Protein Group

Chain A, Crystal Structures Of Recombinant Rat	gi|1311050	30625

Cathepsin B And A Cathepsin B-Inhibitor Complex: Imp		
Chain A, Crystal Structures Of Recombinant Rat Cathepsin B And A Cathepsin B-Inhibitor Complex: Imp	gi|1127275	29919.7
RecName: Full=Cathepsin B; AltName: Full=Cathepsin B1; AltName: Full=RSG-2; Contains: RecName: Full	gi|1705630	40342.9
cathepsin (EC 3.4.22.1) [Rattus norvegicus]	gi|203648	32044.7
cathepsin B preproprotein [Rattus norvegicus]	gi|82830420	40561.1

Peptide Information

Calc. Mass	Obsrv. Mass	± da	± ppm	Start Seq.	End Seq.	Sequence	Ion Score	C. I. %	Modification	Plate [#] Name	Gel Idx/Pos [4700 Sample Name]	iTRAQ Ratio 115/114*	iTRAQ Ratio 116/114*	iTRAQ Ratio 117/114*	Rank	Result Type
1735.8361	1735.7869	-0.0492	-28	71	83	EQWSNCPTIAQIR	27	95.185	(N-term)_iTRAQ[0], MMTS (C)[6]	[1] F3 030912	337/329	1.310	0.842	1.559	1	Mascot
1910.9017	1910.9515	0.0498	26	207	220	HYGYTSYSVSDSEK	58	99.996	(N-term)_iTRAQ[0], Lysine(K)_iTRAQ[14]	[3] F6 and F9	1143/1135	1.394	1.326	1.264	1	Mascot
2280.1797	2280.1865	0.0068	3	229	246	NGPVEGAFTVFSDFLTYK	32	98.579	(N-term)_iTRAQ[0], Lysine(K)_iTRAQ[18]	[1] F3 030912	563/555	1.057	0.838	0.725	1	Mascot

498	60S ribosomal protein L6 [Rattus norvegicus]	gi|162287391	40936.1	3	118	0.882	0.997	0.931	0.263	0.130	0.127	3	3	3	100

Protein Group

Rpl6 protein [Rattus norvegicus]	gi|38511552	40848.1
malignancy-related C140 product [Rattus sp.]	gi|9488975	30927.8
ribosomal protein L6 [Rattus norvegicus]	gi|1490384	40990.1

Peptide Information

Calc. Mass	Obsrv. Mass	± da	± ppm	Start Seq.	End Seq.	Sequence	Ion Score	C. I. %	Modification	Plate [#] Name	Gel Idx/Pos [4700 Sample Name]	iTRAQ Ratio 115/114*	iTRAQ Ratio 116/114*	iTRAQ Ratio 117/114*	Rank	Result Type
1283.6393	1283.5708	-0.0685	-53	125	133	YYPTEDVPR	33	98.698	(N-term)_iTRAQ[0]	[1] F3 030912	207/199	0.651	1.121	0.869	1	Mascot
1620.8115	1620.8732	0.0617	38	237	247	HQEGEIFDTEK	52	99.986	(N-term)_iTRAQ[0], Lysine(K)_iTRAQ[11]	[4] F7 and F10+11	1072/1064	1.232	1.051	1.114	1	Mascot
2022.0515	2021.9817	-0.0698	-35	237	249	HQEGEIFDTEKEK	33	98.662	(N-term)_iTRAQ[0], Lysine(K)_iTRAQ[11, 13]	[2] F12 040912	180/172	0.856	0.841	0.834	1	Mascot

499	RecName: Full=Amine oxidase [flavin-containing] A; AltName: Full=Monoamine oxidase type A; Short=MA	gi|113979	65883.6	3	117	0.781	0.996	1.007	0.141	0.102	0.197	3	3	3	100

Protein Group

Chain A, The Structure Basis Of Specific Recognitions For Substrates And Inhibitors Of Rat Monoamin	gi|48425080	66907.1
Maoa protein [Rattus norvegicus]	gi|183986052	65035.3
monoamine oxidase A [Rattus sp.]	gi|8170739	65058.4
monoamine oxidase [Rattus sp.]	gi|220810	65865.7

Peptide Information

Calc. Mass	Obsrv. Mass	± da	± ppm	Start Seq.	End Seq.	Sequence	Ion Score	C. I. %	Modification	Plate [#] Name	Gel Idx/Pos [4700 Sample Name]	iTRAQ Ratio 115/114*	iTRAQ Ratio 116/114*	iTRAQ Ratio 117/114*	Rank	Result Type
1425.7611	1425.7098	-0.0513	-36	137	147	EIPVDAPWQAR	46	99.938	(N-term)_iTRAQ[0]	[1] F3 030912	273/265	0.758	1.000	1.271	1	Mascot
1762.8997	1762.9299	0.0302	17	62	76	WVDVGGAYVGPTQNR	44	99.901	(N-term)_iTRAQ[0]	[8] F13-15 and F1+2	492/484	0.969	1.120	0.975	1	Mascot
1791.9865	1792.0806	0.0941	53	469	479	KDIWVEEPESK	27	95.141	(N-term)_iTRAQ[0], Lysine(K)_iTRAQ[1,1 1]	[4] F7 and F10+11	1107/1099	0.647	0.882	0.824	1	Mascot

500	prohibitin-2 [Rattus norvegicus]	gi|61556754	36174	2	117	0.919	1.052	0.858	0.355	0.196	0.567	2	2	2	100

Peptide Information

Calc. Mass	Obsrv. Mass	± da	± ppm	Start Seq.	End Seq.	Sequence	Ion Score	C. I. %	Modification	Plate [#] Name	Gel Idx/Pos [4700 Sample Name]	iTRAQ Ratio 115/114*	iTRAQ Ratio 116/114*	iTRAQ Ratio 117/114*	Rank	Result Type
1503.8264	1503.7474	-0.079	-53	225	236	IVQAEGEAEAAK	82	100	(N-term)_iTRAQ[0], Lysine(K)_iTRAQ[12]	[5] F4	112/104	1.273	1.248	1.425	1	Mascot
1868.0231	1867.9889	-0.0342	-18	72	84	IPWFQYPIIYDIR	35	99.278	(N-term)_iTRAQ[0]	[1] F3 030912	536/528	0.663	0.887	0.517	1	Mascot

501	V-type proton ATPase subunit G 2 [Rattus norvegicus]	gi|47059104	14857.8	3	117	0.942	0.824	1.094	0.254	0.113	0.132	3	3	3	100

Peptide Information

Calc. Mass	Obsrv. Mass	± da	± ppm	Start Seq.	End Seq.	Sequence	Ion Score	C. I. %	Modification	Plate [#] Name	Gel Idx/Pos [4700 Sample Name]	iTRAQ Ratio 115/114*	iTRAQ Ratio 116/114*	iTRAQ Ratio 117/114*	Rank	Result Type
1555.7183	1555.6277	-0.0906	-58	38	48	EEAQMEVEQYR	57	99.995	(N-term)_iTRAQ[0]	[1] F3 030912	191/183	0.887	0.945	0.932	1	Mascot
1711.8193	1711.72	-0.0993	-58	38	49	EEAQMEVEQYRR	32	98.413	(N-term)_iTRAQ[0]	[6] F8 110912	176/168	1.295	0.694	1.162	1	Mascot
2220.0798	2220.1018	0.022	10	59	78	QQAAMGSQGNLSAEVE QATR	29	96.819	(N-term)_iTRAQ[0]	[8] F13-15 and F1+2	520/512	0.728	0.853	1.208	1	Mascot

502	doublecortin kinase-2 [Rattus norvegicus]	gi|56269660	92891.1	2	116	1.190	0.680	1.245	0.198	0.608	0.711	2	2	2	100

Protein Group

doublecortin kinase-2 [Rattus norvegicus]	gi|56269585	86665
doublecortin kinase-2 [Rattus norvegicus]	gi|56269604	92854

Peptide Information

Calc. Mass	Obsrv. Mass	± da	± ppm	Start Seq.	End Seq.	Sequence	Ion Score	C. I. %	Modification	Plate [#] Name	Gel Idx/Pos [4700 Sample Name]	iTRAQ Ratio 115/114*	iTRAQ Ratio 116/114*	iTRAQ Ratio 117/114*	Rank	Result Type
1421.8124	1421.7764	-0.036	-25	98	108	SFDALLIELTR	36	99.391	(N-term)_iTRAQ[0]	[1] F3 030912	521/513	1.020	0.359	0.793	1	Mascot
2191.1855	2191.2388	0.0533	24	219	235	TAHSFEQVLTDITEAIK	80	100	(N-term)_iTRAQ[0], Lysine(K)_iTRAQ[17]	[4] F7 and F10+11	535/527	1.388	1.288	1.956	1	Mascot

503	endophilin-B2 [Rattus norvegicus]	gi|57527421	39129.1	3	116	1.173	1.383	1.141	0.286	0.325	0.691	3	3	3	100

Peptide Information

Calc. Mass	Obsrv. Mass	± da	± ppm	Start Seq.	End Seq.	Sequence	Ion Score	C. I. %	Modification	Plate [#] Name	Gel Idx/Pos [4700 Sample Name]	iTRAQ Ratio 115/114*	iTRAQ Ratio 116/114*	iTRAQ Ratio 117/114*	Rank	Result Type
1296.6833	1296.6532	-0.0301	-23	72	79	NFLEGDWK	37	99.561	(N-term)_iTRAQ[0], Lysine(K)_iTRAQ[8]	[3] F6 and F9	315/307	1.596	1.771	2.184	1	Mascot
1573.688	1573.6113	-0.0767	-49	189	199	SQTTYYAQCYR	27	95.24	(N-term)_iTRAQ[0], MMTS (C)[9]	[5] F4	153/145	0.991	1.058	0.948	1	Mascot
1862.0297	1862.0475	0.0178	10	57	71	DFIHTASLSFLTPLR	52	99.984	(N-term)_iTRAQ[0]	[7] F5 120912	422/414	1.020	1.412	0.717	1	Mascot

504	far upstream element-binding protein 2 [Rattus norvegicus]	gi|19424312	79021.7	2	116	0.533	0.746	0.657	0.095	0.240	0.018	2	2	2	100

Peptide Information

Calc. Mass	Obsrv. Mass	± da	± ppm	Start Seq.	End Seq.	Sequence	Ion Score	C. I. %	Modification	Plate [#] Name	Gel Idx/Pos [4700 Sample Name]	iTRAQ Ratio 115/114*	iTRAQ Ratio 116/114*	iTRAQ Ratio 117/114*	Rank	Result Type
1276.646	1276.6344	-0.0116	-9	622	628	AWEEYYK	38	99.582	(N-term)_iTRAQ[0], Lysine(K)_iTRAQ[7]	[4] F7 and F10+11	248/240	0.452	0.564	0.675	1	Mascot
2269.1824	2269.0552	-0.1272	-56	630	647	IGGQPQQPGAPPQQDYT K	80	100	(N-term)_iTRAQ[0], Lysine(K)_iTRAQ[18]	[1] F3 030912	172/164	0.628	0.986	0.639	1	Mascot

505	claudin-11 [Mus musculus]	gi|6679186	23561.7	1	116	1.646	0.720	0.481	0.000	0.000	0.000	1	1	1	100

Protein Group

RecName: Full=Claudin-11	gi|20532024	23493.7

Peptide Information

Calc. Mass	Obsrv. Mass	± da	± ppm	Start Seq.	End Seq.	Sequence	Ion Score	C. I. %	Modification	Plate [#] Name	Gel Idx/Pos [4700 Sample Name]	iTRAQ Ratio 115/114*	iTRAQ Ratio 116/114*	iTRAQ Ratio 117/114*	Rank	Result Type
1806.8909	1806.7831	-0.1078	-60	190	203	FYYSSGSSSPTHAK	116	100	(N-term)_iTRAQ[0], Lysine(K)_iTRAQ[14]	[6] F8 110912	176/168	1.646	0.720	0.481	1	Mascot

506	proteasome subunit alpha type-7 [Rattus norvegicus]	gi|56550075	31290.9	2	114	1.211	1.037	1.327	0.286	0.084	0.409	2	2	2	100

Protein Group

RecName: Full=Proteasome subunit alpha type-7;	gi|1346784	31761.2

AltName: Full=Proteasome subunit RC6-1

Peptide Information

Calc. Mass	Obsrv. Mass	± da	± ppm	Start Seq.	End Seq.	Sequence	Ion Score	C. I. %	Modification	Plate [#] Name	Gel Idx/Pos [4700 Sample Name]	iTRAQ Ratio 115/114*	iTRAQ Ratio 116/114*	iTRAQ Ratio 117/114*	Rank	Result Type
1954.991	1954.8768	-0.1142	-58	144	157	LYQTDPSGTYHAWK	43	99.878	(N-term)_iTRAQ[0], Lysine(K)_iTRAQ[14]	[6] F8 110912	252/244	1.497	1.121	1.736	1	Mascot
2015.0067	2014.9215	-0.0852	-42	175	189	NYTDDAIETDDLTIK	71	100	(N-term)_iTRAQ[0], Lysine(K)_iTRAQ[15]	[1] F3 030912	276/268	0.980	0.960	1.014	1	Mascot

507	phosphoribosyl pyrophosphate synthase-associated protein 2 isoform b [Mus musculus]	gi|256773295	34096.2	2	114	0.547	0.804	0.905	0.459	0.138	0.243	2	2	2	100

Protein Group

phosphoribosyl pyrophosphate synthase-associated protein 2 [Rattus norvegicus] gi|16923984 44764.8

Peptide Information

Calc. Mass	Obsrv. Mass	± da	± ppm	Start Seq.	End Seq.	Sequence	Ion Score	C. I. %	Modification	Plate [#] Name	Gel Idx/Pos [4700 Sample Name]	iTRAQ Ratio 115/114*	iTRAQ Ratio 116/114*	iTRAQ Ratio 117/114*	Rank	Result Type
1711.9952	1711.9802	-0.015	-9	164	177	EKPPITVVGDVGGR	74	100	(N-term)_iTRAQ[0], Lysine(K)_iTRAQ[2]	[4] F7 and F10+11	225/217	1.006	0.686	1.147	1	Mascot
1805.967	1805.9974	0.0304	17	65	78	EIQGFFNIPVDNLR	40	99.772	(N-term)_iTRAQ[0]	[8] F13-15 and F1+2	384/376	0.298	0.942	0.713	1	Mascot

508	oligodendrocyte-myelin glycoprotein [Rattus norvegicus]	gi|33414042	52772.1	3	114	1.013	0.842	0.702	0.424	0.187	0.426	3	3	3	100

Protein Group

oligodendrocyte myelin glycoprotein [Rattus norvegicus] gi|32454276 52772.1

Peptide Information

Calc. Mass	Obsrv. Mass	± da	± ppm	Start Seq.	End Seq.	Sequence	Ion Score	C. I. %	Modification	Plate [#] Name	Gel Idx/Pos [4700 Sample Name]	iTRAQ Ratio 115/114*	iTRAQ Ratio 116/114*	iTRAQ Ratio 117/114*	Rank	Result Type
1113.5682	1113.5391	-0.0291	-26	241	246	WMMETK	28	96.455	(N-term)_iTRAQ[0], Lysine(K)_iTRAQ[6]	[3] F6 and F9	232/224	1.104	0.864	0.920	1	Mascot
1513.7897	1513.8041	0.0144	10	117	126	SDTAYQWNLK	47	99.953	(N-term)_iTRAQ[0], Lysine(K)_iTRAQ[10]	[3] F6 and F9	246/238	1.478	1.062	1.042	1	Mascot
3061.5867	3061.5	-0.0867	-28	203	226	FTFIPDQSFDQLLQLQEIT LHNNR	39	99.665	(N-term)_iTRAQ[0]	[1] F3 030912	532/524	0.636	0.651	0.360	1	Mascot

509	neuronal calcium sensor 1 [Mus musculus]	gi|9790115	24504.7	2	114	1.275	1.652	1.763	0.150	0.679	0.233	2	2	2	100

Peptide Information

Calc. Mass	Obsrv. Mass	± da	± ppm	Start Seq.	End Seq.	Sequence	Ion Score	C. I. %	Modification	Plate [#] Name	Gel Idx/Pos [4700 Sample Name]	iTRAQ Ratio 115/114*	iTRAQ Ratio 116/114*	iTRAQ Ratio 117/114*	Rank	Result Type
1268.6885	1268.6415	-0.047	-37	26	32	EVQQWYK	38	99.62	(N-term)_iTRAQ[0], Lysine(K)_iTRAQ[7]	[3] F6 and F9	205/197	1.425	1.171	1.557	1	Mascot
1770.8381	1770.7325	-0.1056	-60	37	50	DCPSGQLDAAGFQK	75	100	(N-term)_iTRAQ[0], Lysine(K)_iTRAQ[14], MMTS (C)[2]	[5] F4	172/164	1.140	2.331	1.996	1	Mascot

510	ras-related protein Ral-A precursor [Rattus norvegicus]	gi|13592039	26846.2	2	112	0.929	0.829	0.923	0.416	0.316	0.391	2	2	2	100

Peptide Information

Calc. Mass	Obsrv. Mass	± da	± ppm	Start Seq.	End Seq.	Sequence	Ion Score	C. I. %	Modification	Plate [#] Name	Gel Idx/Pos [4700 Sample Name]	iTRAQ Ratio 115/114*	iTRAQ Ratio 116/114*	iTRAQ Ratio 117/114*	Rank	Result Type
1291.7654	1291.705	-0.0604	-47	17	27	VIMVGSGGVGK	28	96.52	(N-term)_iTRAQ[0], Lysine(K)_iTRAQ[11]	[7] F5 120912	175/167	0.642	0.600	0.648	1	Mascot
1912.9651	1912.8947	-0.0704	-37	146	159	ADQWNVNYVETSAK	83	100	(N-term)_iTRAQ[0], Lysine(K)_iTRAQ[14]	[7] F5 120912	220/212	1.345	1.144	1.314	1	Mascot

511	hypothetical protein LOC100272906 [Zea mays]	gi|226530797	28168.2	1	112	1.994	1.260	1.050	0.000	0.000	0.000	1	1	1	100

Protein Group

CD81 antigen [Rattus norvegicus] gi|6978639 28152.2

Peptide Information

Calc. Mass	Obsrv. Mass	± da	± ppm	Start Seq.	End Seq.	Sequence	Ion Score	C. I. %	Modification	Plate [#] Name	Gel Idx/Pos [4700 Sample Name]	iTRAQ Ratio 115/114*	iTRAQ Ratio 116/114*	iTRAQ Ratio 117/114*	Rank	Result Type
2573.2188	2573.1304	-0.0884	-34	125	144	QFYDQALQQAVMDDDA	112	100	(N-term)_iTRAQ[0],	[1] F3 030912	326/318	1.994	1.260	1.050	1	Mascot

						NNAK			Lysine(K)_iTRAQ[20]															

512	Ribophorin I [Rattus norvegicus]	gi|38512106	74358.8	3	112	0.951	1.223	0.955	0.630	0.166	0.433	3	3	3	100

Peptide Information

Calc. Mass	Obsrv. Mass	± da	± ppm	Start Seq.	End Seq.	Sequence	Ion Score	C. I. %	Modification	Plate [#] Name	Gel Idx/Pos [4700 Sample Name]	iTRAQ Ratio 115/114*	iTRAQ Ratio 116/114*	iTRAQ Ratio 117/114*	Rank	Result Type
1239.7136	1239.6963	-0.0173	-14	319	326	FPLFGGWK	31	98.074	(N-term)_iTRAQ[0], Lysine(K)_iTRAQ[8]	[3] F6 and F9	427/419	1.537	1.433	1.601	1	Mascot
1572.8156	1572.7336	-0.082	-52	204	214	DIPAYSQDTFK	45	99.92	(N-term)_iTRAQ[0], Lysine(K)_iTRAQ[11]	[5] F4	155/147	1.189	1.051	0.815	1	Mascot
1765.968	1765.8977	-0.0703	-40	48	64	VTAEVVLAHPGGGSTAR	36	99.37	(N-term)_iTRAQ[0]	[3] F6 and F9	216/208	0.471	1.214	0.668	1	Mascot

513	sorting nexin-3 isoform a [Homo sapiens]	gi|4507143	20381.9	2	112	1.250	1.518	1.589	0.297	0.212	0.258	2	2	2	100

Peptide Information

Calc. Mass	Obsrv. Mass	± da	± ppm	Start Seq.	End Seq.	Sequence	Ion Score	C. I. %	Modification	Plate [#] Name	Gel Idx/Pos [4700 Sample Name]	iTRAQ Ratio 115/114*	iTRAQ Ratio 116/114*	iTRAQ Ratio 117/114*	Rank	Result Type
1785.8052	1785.7593	-0.0459	-26	105	118	GDDGIFDDNFIEER	81	100	(N-term)_iTRAQ[0]	[1] F3 030912	332/324	1.010	1.332	1.368	1	Mascot
1938.9718	1938.9868	0.015	8	140	152	CLHMFLQDEIIDK	31	97.941	(N-term)_iTRAQ[0], Lysine(K)_iTRAQ[13], MMTS (C)[1]	[4] F7 and F10+11	450/442	1.548	1.729	1.847	1	Mascot

514	40S ribosomal protein S3 [Mus musculus]	gi|6755372	29821.5	3	111	0.771	0.962	1.081	0.124	0.471	0.073	3	3	3	100

Peptide Information

Calc. Mass	Obsrv. Mass	± da	± ppm	Start Seq.	End Seq.	Sequence	Ion Score	C. I. %	Modification	Plate [#] Name	Gel Idx/Pos [4700 Sample Name]	iTRAQ Ratio 115/114*	iTRAQ Ratio 116/114*	iTRAQ Ratio 117/114*	Rank	Result Type
1421.7346	1421.6948	-0.0398	-28	95	106	GLCAIAQAESLR	33	98.879	(N-term)_iTRAQ[0], MMTS (C)[3]	[1] F3 030912	369/361	0.891	0.569	1.043	1	Mascot
1567.7723	1567.6986	-0.0737	-47	28	40	ELAEDGYSGVEVR	50	99.977	(N-term)_iTRAQ[0]	[1] F3 030912	214/206	0.820	1.047	1.022	1	Mascot
1860.9629	1860.9147	-0.0482	-26	77	90	FGFPEGSVELYAEK	27	95.527	(N-term)_iTRAQ[0], Lysine(K)_iTRAQ[14]	[1] F3 030912	361/353	0.627	1.495	1.184	1	Mascot

515	ribose-phosphate pyrophosphokinase I -like [Rattus norvegicus]	gi|57527447	38368.1	3	111	0.938	0.958	0.714	0.010	0.204	0.357	3	3	3	100

Protein Group

ribose-phosphate pyrophosphokinase 1 isoform 1 [Homo sapiens]	gi|4506127	38251.9

Peptide Information

Calc. Mass	Obsrv. Mass	± da	± ppm	Start Seq.	End Seq.	Sequence	Ion Score	C. I. %	Modification	Plate [#] Name	Gel Idx/Pos [4700 Sample Name]	iTRAQ Ratio 115/114*	iTRAQ Ratio 116/114*	iTRAQ Ratio 117/114*	Rank	Result Type
1148.6959	1148.6737	-0.0222	-19	205	212	MVLVGDVK	46	99.941	(N-term)_iTRAQ[0], Lysine(K)_iTRAQ[8]	[7] F5 120912	206/198	0.925	1.176	1.074	1	Mascot
1526.7965	1526.8243	0.0278	18	85	96	VTAVIPCFPYAR	28	96.365	(N-term)_iTRAQ[0], MMTS (C)[7]	[8] F13-15 and F1+2	384/376	0.949	0.740	0.411	1	Mascot
1721.9069	1721.9143	0.0074	4	6	18	IFSGSSHQDLSQK	36	99.416	(N-term)_iTRAQ[0], Lysine(K)_iTRAQ[13]	[3] F6 and F9	1146/1138	0.939	1.012	0.826	1	Mascot

516	prelamin-A/C isoform C2 [Rattus norvegicus]	gi|50355947	77791.4	2	110	1.333	0.984	1.250	0.186	0.107	0.078	2	2	2	100

Protein Group

lamin A	gi|383110	80195.7
lamin C2 [Rattus norvegicus]	gi|1556433	57130

Peptide Information

Calc. Mass	Obsrv. Mass	± da	± ppm	Start Seq.	End Seq.	Sequence	Ion Score	C. I. %	Modification	Plate [#] Name	Gel Idx/Pos [4700 Sample Name]	iTRAQ Ratio 115/114*	iTRAQ Ratio 116/114*	iTRAQ Ratio 117/114*	Rank	Result Type
1676.033	1676.0892	0.0562	34	133	143	LKDLEALLNSK	56	99.994	(N-term)_iTRAQ[0], Lysine(K)_iTRAQ[2,11]	[4] F7 and F10+11	1276/1268	1.170	0.888	1.327	1	Mascot
1708.7833	1708.6967	-0.0866	-51	457	469	SNEDQSMGNWQIR	54	99.99	(N-term)_iTRAQ[0]	[5] F4	155/147	1.519	1.091	1.177	1	Mascot

517	RecName: Full=Nucleosome assembly protein 1-like 1; AltName: Full=NAP-1-related protein; Flags: Pre	gi|34098357	51135.8	3	110	1.082	1.232	0.980	0.186	0.263	0.345	3	3	3	100

Protein Group

nucleosome assembly protein 1-like 1 isoform 2 [Mus musculus]	gi|7657357	51022.7

Peptide Information

Calc. Mass	Obsrv. Mass	± da	± ppm	Start Seq.	End Seq.	Sequence	Ion Score	C. I. %	Modification	Plate [#] Name	Gel Idx/Pos [4700 Sample Name]	iTRAQ Ratio 115/114*	iTRAQ Ratio 116/114*	iTRAQ Ratio 117/114*	Rank	Result Type
1624.9348	1624.9218	-0.013	-8	166	176	GIPEFWLTVFK	48	99.965	(N-term)_iTRAQ[0], Lysine(K)_iTRAQ[11]	[7] F5 120912	508/500	1.246	1.225	0.643	1	Mascot
1801.0258	1801.0243	-0.0015	-1	106	117	YAVLYQPLFDKR	27	95.039	(N-term)_iTRAQ[0], Lysine(K)_iTRAQ[11]	[4] F7 and F10+11	366/358	0.867	0.975	1.273	1	Mascot
1917.1221	1917.1576	0.0355	19	105	116	KYAVLYQPLFDK	35	99.14	(N-term)_iTRAQ[0], Lysine(K)_iTRAQ[1,12]	[3] F6 and F9	1303/1295	1.174	1.565	1.151	1	Mascot

518	pyridoxal kinase [Rattus norvegicus]	gi|13929082	37663.4	2	110	0.929	1.049	0.965	0.142	0.044	0.224	2	2	2	100

Peptide Information

Calc. Mass	Obsrv. Mass	± da	± ppm	Start Seq.	End Seq.	Sequence	Ion Score	C. I. %	Modification	Plate [#] Name	Gel Idx/Pos [4700 Sample Name]	iTRAQ Ratio 115/114*	iTRAQ Ratio 116/114*	iTRAQ Ratio 117/114*	Rank	Result Type
2026.03	2026.0862	0.0562	28	162	177	IHSQEEAFAVMDVLHR	44	99.902	(N-term)_iTRAQ[0]	[4] F7 and F10+11	1250/1242	0.806	1.007	0.783	1	Mascot
2069.1235	2069.1155	-0.008	-4	276	292	AEAGEGQKPSPAQLELR	66	99.999	(N-term)_iTRAQ[0], Lysine(K)_iTRAQ[8]	[4] F7 and F10+11	209/201	1.071	1.093	1.188	1	Mascot

519	vesicular glutamate transporter 1 [Rattus norvegicus]	gi|16758726	64482.7	2	109	0.777	1.051	1.085	0.156	0.347	0.454	2	2	2	100

Peptide Information

Calc. Mass	Obsrv. Mass	± da	± ppm	Start Seq.	End Seq.	Sequence	Ion Score	C. I. %	Modification	Plate [#] Name	Gel Idx/Pos [4700 Sample Name]	iTRAQ Ratio 115/114*	iTRAQ Ratio 116/114*	iTRAQ Ratio 117/114*	Rank	Result Type
1755.9865	1756.0454	0.0589	34	272	283	KYIEDAIGESAK	47	99.953	(N-term)_iTRAQ[0], Lysine(K)_iTRAQ[1,12]	[4] F7 and F10+11	1122/1114	0.646	0.790	0.765	1	Mascot
1877.8837	1877.8005	-0.0832	-44	495	507	QPWAEPEEMSEEK	62	99.999	(N-term)_iTRAQ[0], Lysine(K)_iTRAQ[13]	[5] F4	162/154	0.933	1.398	1.539	1	Mascot

520	cytochrome c oxidase subunit VIc [Rattus norvegicus]	gi|203515	8320.5	2	109	0.690	0.823	0.924	0.282	0.101	0.207	3	3	3	100

Protein Group

cytochrome c oxidase subunit 6C-2 [Rattus norvegicus]	gi|160333459	9602.3
cytochrome oxidase subunit VIC [Rattus norvegicus]	gi|203714	4725.4

Peptide Information

Calc. Mass	Obsrv. Mass	± da	± ppm	Start Seq.	End Seq.	Sequence	Ion Score	C. I. %	Modification	Plate [#] Name	Gel Idx/Pos [4700 Sample Name]	iTRAQ Ratio 115/114*	iTRAQ Ratio 116/114*	iTRAQ Ratio 117/114*	Rank	Result Type
1223.6993	1223.6501	-0.0492	-40	57	65	QAGVFQSAK	36	99.38	(N-term)_iTRAQ[0], Lysine(K)_iTRAQ[9]	[3] F6 and F9	148/140	0.442	0.865	0.960	1	Mascot
1852.8456	1852.7557	-0.0899	-49	45	56	NYDSMKDFEEMR	67	100	(N-term)_iTRAQ[0], Lysine(K)_iTRAQ[6]	[6] F8 110912	269/261	0.732	0.701	0.709	1	Mascot
1852.8456	1852.9521	0.1065	57	45	56	NYDSMKDFEEMR	73	100	(N-term)_iTRAQ[0], Lysine(K)_iTRAQ[6]	[3] F6 and F9	1225/1217	1.015	0.919	1.158	1	Mascot

521	M4 protein [Rattus norvegicus]	gi|16124253	80819	3	109	0.853	0.846	1.003	0.263	0.068	0.475	3	3	3	100

Peptide Information

Calc. Mass	Obsrv. Mass	± da	± ppm	Start Seq.	End Seq.	Sequence	Ion Score	C. I. %	Modification	Plate [#] Name	Gel Idx/Pos [4700 Sample Name]	iTRAQ Ratio 115/114*	iTRAQ Ratio 116/114*	iTRAQ Ratio 117/114*	Rank	Result Type
1354.7253	1354.6987	-0.0266	-20	67	75	FEPYANPTK	28	96.068	(N-term)_iTRAQ[0], Lysine(K)_iTRAQ[9]	[7] F5 120912	159/151	0.650	0.758	0.697	1	Mascot
1552.8984	1552.8171	-0.0813	-52	79	89	AFITNIPFDVK	28	95.995	(N-term)_iTRAQ[0], Lysine(K)_iTRAQ[11]	[5] F4	298/290	1.229	0.895	1.716	1	Mascot
2230.1353	2230.2231	0.0878	39	638	652	DKFNECGHVLYADIK	54	99.99	(N-term)_iTRAQ[0], Lysine(K)_iTRAQ[2,15], MMTS (C)[6]	[4] F7 and F10+11	1239/1231	0.778	0.893	0.843	1	Mascot

522	endophilin-A2 [Rattus norvegicus]	gi|13676847	46405.5	2	108	1.369	1.276	1.757	1.458	0.422	1.761	3	3	3	100

Peptide Information

Calc. Mass	Obsrv. Mass	± da	± ppm	Start Seq.	End Seq.	Sequence	Ion Score	C. I. %	Modification	Plate [#] Name	Gel Idx/Pos [4700 Sample Name]	iTRAQ Ratio 115/114*	iTRAQ Ratio 116/114*	iTRAQ Ratio 117/114*	Rank	Result Type
1532.8193	1532.8523	0.033	22	54	65	TIEYLQPNPASR	31	97.884	(N-term)_iTRAQ[0]	[8] F13-15 and F1+2	550/542	1.230	1.159	1.433	1	Mascot

Calc. Mass	Obsrv. Mass	± da	± ppm	Start Seq.	End Seq.	Sequence	Ion Score	C. I. %	Modification	Plate [#] Name	Gel Idx/Pos [4700 Sample Name]	iTRAQ Ratio 115/114*	iTRAQ Ratio 116/114*	iTRAQ Ratio 117/114*	Rank	Result Type
1532.8193	1532.8674	0.0481	31	54	65	TIEYLQPNPASR	44	99.896	(N-term)_iTRAQ[0]	[8] F13-15 and F1+2	545/537	0.597	0.953	0.847	1	Mascot
2328.1274	2328.0562	-0.0712	-31	103	122	ELGGESNFGDALLDAGE SMK	65	99.999	(N-term)_iTRAQ[0], Lysine(K)_iTRAQ[20]	[1] F3 030912	401/393	3.492	1.881	4.469	1	Mascot

523	prothymosin alpha [Rattus norvegicus]	gi|11120684	13816.2	1	108	0.839	0.912	0.942	0.578	0.229	0.050	2	2	2	100

Peptide Information

Calc. Mass	Obsrv. Mass	± da	± ppm	Start Seq.	End Seq.	Sequence	Ion Score	C. I. %	Modification	Plate [#] Name	Gel Idx/Pos [4700 Sample Name]	iTRAQ Ratio 115/114*	iTRAQ Ratio 116/114*	iTRAQ Ratio 117/114*	Rank	Result Type
2039.9993	2039.8568	-0.1425	-70	91	104	VAEDDEDDDVETKK	108	100	(N-term)_iTRAQ[0], Lysine(K)_iTRAQ[13, 14]	[6] F8 110912	124/116	0.496	0.729	0.895	1	Mascot
2039.9993	2040.0244	0.0251	12	91	104	VAEDDEDDDVETKK	35	99.237	(N-term)_iTRAQ[0], Lysine(K)_iTRAQ[13, 14]	[3] F6 and F9	1088/1080	1.417	1.141	0.993	1	Mascot

524	complexin-1 [Rattus norvegicus]	gi|12408324	17896.4	2	108	0.863	0.575	0.616	0.007	0.025	0.125	2	2	2	100

Peptide Information

Calc. Mass	Obsrv. Mass	± da	± ppm	Start Seq.	End Seq.	Sequence	Ion Score	C. I. %	Modification	Plate [#] Name	Gel Idx/Pos [4700 Sample Name]	iTRAQ Ratio 115/114*	iTRAQ Ratio 116/114*	iTRAQ Ratio 117/114*	Rank	Result Type
1596.8706	1596.7845	-0.0861	-54	123	133	YLPGPLQDMFK	58	99.996	(N-term)_iTRAQ[0], Lysine(K)_iTRAQ[11]	[5] F4	340/332	0.857	0.600	0.741	1	Mascot
1907.9756	1907.8719	-0.1037	-54	19	32	MLGGDEEKDPDAAK	50	99.976	(N-term)_iTRAQ[0], Lysine(K)_iTRAQ[8,1 4]	[6] F8 110912	157/149	0.870	0.551	0.512	1	Mascot

525	proliferation-associated protein 2G4 [Rattus norvegicus]	gi|51948384	50101.5	3	108	0.737	1.148	0.999	0.458	0.775	0.289	3	3	3	100

Peptide Information

Calc. Mass	Obsrv. Mass	± da	± ppm	Start Seq.	End Seq.	Sequence	Ion Score	C. I. %	Modification	Plate [#] Name	Gel Idx/Pos [4700 Sample Name]	iTRAQ Ratio 115/114*	iTRAQ Ratio 116/114*	iTRAQ Ratio 117/114*	Rank	Result Type
1573.8795	1573.7908	-0.0887	-56	200	210	TIIQNPTDQQK	30	97.658	(N-term)_iTRAQ[0], Lysine(K)_iTRAQ[11]	[5] F4	102/94	1.149	2.292	1.391	1	Mascot
2359.3105	2359.1602	-0.1503	-64	156	172	LVKPGNQNTQVTEAWNK	43	99.868	(N-term)_iTRAQ[0], Lysine(K)_iTRAQ[3,1 7]	[6] F8 110912	237/229	0.927	0.664	0.750	1	Mascot
2446.2273	2446.3188	0.0915	37	173	191	VAHSFNCTPIEGMLSHQL K	35	99.246	(N-term)_iTRAQ[0], Lysine(K)_iTRAQ[19], MMTS (C)[7]	[4] F7 and F10+11	1302/1294	0.376	0.994	0.955	1	Mascot

526	neural cell adhesion molecule 2 [Rattus norvegicus]	gi|42821116	102125.2	3	108	0.976	0.920	0.830	0.120	0.157	0.108	3	3	3	100

Protein Group

fasciclin II GPI-linked protein isoform [Rattus norvegicus]	gi|42528323	88361.5

Peptide Information

Calc. Mass	Obsrv. Mass	± da	± ppm	Start Seq.	End Seq.	Sequence	Ion Score	C. I. %	Modification	Plate [#] Name	Gel Idx/Pos [4700 Sample Name]	iTRAQ Ratio 115/114*	iTRAQ Ratio 116/114*	iTRAQ Ratio 117/114*	Rank	Result Type
1792.944	1792.8832	-0.0608	-34	593	607	EPSPPSIHGQPSSGK	38	99.63	(N-term)_iTRAQ[0], Lysine(K)_iTRAQ[15]	[4] F7 and F10+11	140/132	0.934	1.146	0.711	1	Mascot
2008.0511	2008.0526	0.0015	1	485	500	FQEYILELADVPSSPR	30	97.658	(N-term)_iTRAQ[0]	[8] F13-15 and F1+2	330/322	1.145	0.851	0.840	1	Mascot
2039.0291	2038.9878	-0.0413	-20	337	353	AIDGVTFSEGDKSPDGR	40	99.726	(N-term)_iTRAQ[0], Lysine(K)_iTRAQ[12]	[4] F7 and F10+11	218/210	0.870	0.798	0.958	1	Mascot

527	brain Y-box binding protein 1 [Rattus norvegicus]	gi|38142418	38230.3	1	107	1.371	1.255	0.652	0.000	0.000	0.000	1	1	1	100

Protein Group

TSH receptor suppressor element-binding protein-1 [Rattus sp.]	gi|9653686	37959.2
enhancer factor-1-alpha [Rattus norvegicus]	gi|203999	38148.3
nuclease-sensitive element-binding protein 1 [Rattus norvegicus]	gi|92373398	38158.3
putative [Rattus norvegicus]	gi|203398	38184.3

Peptide Information

Calc. Mass	Obsrv. Mass	± da	± ppm	Start Seq.	End Seq.	Sequence	Ion Score	C. I. %	Modification	Plate [#] Name	Gel Idx/Pos [4700 Sample Name]	iTRAQ Ratio 115/114*	iTRAQ Ratio 116/114*	iTRAQ Ratio 117/114*	Rank	Result Type

1984.0709	1983.9642	-0.1067	-54	117	135	GAEAANVTGPGGVPVQGSK	107	100	(N-term)_iTRAQ[0], Lysine(K)_iTRAQ[19]	[1] F3 030912	172/164	1.371	1.255	0.652	1	Mascot

528	GTP-binding nuclear protein Ran [Homo sapiens]	gi|5453555	27283.5	2	107	1.078	1.192	1.179	0.049	0.166	0.149	2	2	2	100

Peptide Information

Calc. Mass	Obsrv. Mass	± da	± ppm	Start Seq.	End Seq.	Sequence	Ion Score	C. I. %	Modification	Plate [#] Name	Gel Idx/Pos [4700 Sample Name]	iTRAQ Ratio 115/114*	iTRAQ Ratio 116/114*	iTRAQ Ratio 117/114*	Rank	Result Type
1582.8112	1582.7565	-0.0547	-35	61	71	FNVWDTAGQEK	68	100	(N-term)_iTRAQ[0], Lysine(K)_iTRAQ[11]	[7] F5 120912	236/228	1.127	1.358	1.047	1	Mascot
2196.2048	2196.1282	-0.0766	-35	39	56	YVATLGVEVHPLVFHTNR	39	99.706	(N-term)_iTRAQ[0]	[6] F8 110912	372/364	1.031	1.047	1.328	1	Mascot

529	ATP synthase subunit O, mitochondrial precursor [Rattus norvegicus]	gi|20302061	26454.9	2	106	0.763	0.923	1.073	0.281	0.409	0.689	2	2	2	100

Peptide Information

Calc. Mass	Obsrv. Mass	± da	± ppm	Start Seq.	End Seq.	Sequence	Ion Score	C. I. %	Modification	Plate [#] Name	Gel Idx/Pos [4700 Sample Name]	iTRAQ Ratio 115/114*	iTRAQ Ratio 116/114*	iTRAQ Ratio 117/114*	Rank	Result Type
1301.8038	1301.74	-0.0638	-49	168	176	GQILNLEVK	36	99.354	(N-term)_iTRAQ[0], Lysine(K)_iTRAQ[9]	[7] F5 120912	228/220	0.558	0.640	0.653	1	Mascot
1504.9348	1504.8528	-0.082	-54	74	84	VSLAVLNPYIK	71	100	(N-term)_iTRAQ[0], Lysine(K)_iTRAQ[11]	[5] F4	290/282	1.044	1.332	1.761	1	Mascot

530	V-type proton ATPase subunit D [Rattus norvegicus]	gi|40786463	32326.2	3	106	1.076	1.349	0.985	0.022	0.272	0.122	3	3	3	100

Peptide Information

Calc. Mass	Obsrv. Mass	± da	± ppm	Start Seq.	End Seq.	Sequence	Ion Score	C. I. %	Modification	Plate [#] Name	Gel Idx/Pos [4700 Sample Name]	iTRAQ Ratio 115/114*	iTRAQ Ratio 116/114*	iTRAQ Ratio 117/114*	Rank	Result Type
1560.9458	1560.9038	-0.042	-27	168	179	RVNAIEHVIIPR	28	96.032	(N-term)_iTRAQ[0]	[2] F12 040912	292/284	1.100	1.318	1.063	1	Mascot
1580.8656	1580.8523	-0.0133	-8	183	194	TLAYIITELDER	30	97.372	(N-term)_iTRAQ[0]	[8] F13-15 and F1+2	294/286	1.047	1.708	0.835	1	Mascot
2030.0804	2030.0082	-0.0722	-36	74	89	FTAGDFSTTVIQNVNK	48	99.964	(N-term)_iTRAQ[0], Lysine(K)_iTRAQ[16]	[1] F3 030912	327/319	1.083	1.091	1.076	1	Mascot

531	Coq9 protein [Rattus norvegicus]	gi|51259441	36068.1	2	106	1.027	1.045	1.671	0.701	0.234	0.339	2	2	2	100

Peptide Information

Calc. Mass	Obsrv. Mass	± da	± ppm	Start Seq.	End Seq.	Sequence	Ion Score	C. I. %	Modification	Plate [#] Name	Gel Idx/Pos [4700 Sample Name]	iTRAQ Ratio 115/114*	iTRAQ Ratio 116/114*	iTRAQ Ratio 117/114*	Rank	Result Type
1598.8638	1598.8802	0.0164	10	184	194	MLIPYIEHWPR	39	99.695	(N-term)_iTRAQ[0]	[4] F7 and F10+11	436/428	0.610	0.854	1.389	1	Mascot
2745.1519	2745.0386	-0.1133	-41	74	94	YTDQSGEEEEDYESEEQIQHR	67	99.999	(N-term)_iTRAQ[0]	[7] F5 120912	152/144	1.728	1.279	2.010	1	Mascot

532	probable regulatory protein 322 - rat	gi|2143936	159946.9	2	106	1.062	0.574	0.655	0.242	0.138	0.200	2	2	2	100

Protein Group

A kinase anchoring protein 12 gamma [Rattus norvegicus]	gi|56199809	188370.7
A-kinase anchor protein 12 isoform alpha [Rattus norvegicus]	gi|75905809	199716.1
A-kinase anchor protein 12 isoform beta [Rattus norvegicus]	gi|75905811	191386.3
PKC binding protein and substrate [Rattus norvegicus]	gi|4096820	190348.8
SSeCKS [Rattus norvegicus]	gi|1022683	190480.8
protein kinase C-binding protein	gi|1589163	199812.1

Peptide Information

Calc. Mass	Obsrv. Mass	± da	± ppm	Start Seq.	End Seq.	Sequence	Ion Score	C. I. %	Modification	Plate [#] Name	Gel Idx/Pos [4700 Sample Name]	iTRAQ Ratio 115/114*	iTRAQ Ratio 116/114*	iTRAQ Ratio 117/114*	Rank	Result Type
1988.1022	1988.0127	-0.0895	-45	642	656	VKEESQVPATQTVQR	77	100	(N-term)_iTRAQ[0], Lysine(K)_iTRAQ[2]	[4] F7 and F10+11	136/128	1.304	0.712	0.855	1	Mascot
2079.0127	2078.9062	-0.1065	-51	842	857	TEVGQEGEVDWLDGEK	29	97.052	(N-term)_iTRAQ[0], Lysine(K)_iTRAQ[16]	[5] F4	205/197	0.865	0.462	0.502	1	Mascot

533	cell cycle exit and neuronal differentiation protein 1 [Rattus norvegicus]	gi|62079059	17529.6	2	105	0.810	0.721	0.933	0.150	0.252	0.194	3	3	3	100

Peptide Information

Calc. Mass	Obsrv. Mass	± da	± ppm	Start Seq.	End Seq.	Sequence	Ion Score	C. I. %	Modification	Plate [#] Name	Gel Idx/Pos [4700 Sample Name]	iTRAQ Ratio 115/114*	iTRAQ Ratio 116/114*	iTRAQ Ratio 117/114*	Rank	Result Type
1202.6991	1202.6526	-0.0465	-39	17	25	VPQATAEAK	31	97.918	(N-term)_iTRAQ[0], Lysine(K)_iTRAQ[9]	[7] F5 120912	75/67	0.985	1.038	1.046	1	Mascot
2370.2637	2370.1067	-0.157	-66	43	61	DAQAEKQEQPAAPGPATTK	30	97.525	(N-term)_iTRAQ[0], Lysine(K)_iTRAQ[6,19]	[6] F8 110912	113/105	0.651	0.498	0.715	1	Mascot
2370.2637	2370.1987	-0.065	-27	43	61	DAQAEKQEQPAAPGPATTK	74	100	(N-term)_iTRAQ[0], Lysine(K)_iTRAQ[6,19]	[4] F7 and F10+11	109/101	0.829	0.725	1.087	1	Mascot

534	eukaryotic translation initiation factor 4H [Rattus norvegicus]	gi|55741853	29082.6	2	105	1.036	0.893	0.654	0.037	0.034	0.319	2	2	2	100

Peptide Information

Calc. Mass	Obsrv. Mass	± da	± ppm	Start Seq.	End Seq.	Sequence	Ion Score	C. I. %	Modification	Plate [#] Name	Gel Idx/Pos [4700 Sample Name]	iTRAQ Ratio 115/114*	iTRAQ Ratio 116/114*	iTRAQ Ratio 117/114*	Rank	Result Type
1537.7982	1537.7426	-0.0556	-36	97	109	EALTYDGALLGDR	43	99.869	(N-term)_iTRAQ[0]	[1] F3 030912	281/273	1.000	0.927	0.440	1	Mascot
1840.8256	1840.8256	0	0	192	205	GSNMDFREPTEEER	62	99.999	(N-term)_iTRAQ[0]	[4] F7 and F10+11	171/163	1.073	0.860	0.973	1	Mascot

535	heterogeneous nuclear ribonucleoprotein R [Rattus norvegicus]	gi|27903509	77401.5	2	105	1.115	1.065	1.356	0.125	0.215	0.060	2	2	2	100

Peptide Information

Calc. Mass	Obsrv. Mass	± da	± ppm	Start Seq.	End Seq.	Sequence	Ion Score	C. I. %	Modification	Plate [#] Name	Gel Idx/Pos [4700 Sample Name]	iTRAQ Ratio 115/114*	iTRAQ Ratio 116/114*	iTRAQ Ratio 117/114*	Rank	Result Type
1721.7894	1721.8848	0.0954	55	290	300	GFCFLEYEDHK	56	99.994	(N-term)_iTRAQ[0], Lysine(K)_iTRAQ[11], MMTS (C)[3]	[3] F6 and F9	1320/1312	1.002	1.280	1.299	1	Mascot
1770.0021	1770.0588	0.0567	32	258	268	TKENILEEFSK	50	99.974	(N-term)_iTRAQ[0], Lysine(K)_iTRAQ[2,11]	[4] F7 and F10+11	1200/1192	1.240	0.886	1.416	1	Mascot

536	Chain A, High Resolution Structure Of A Truncated Neuronal Snare Complex	gi|27574127	8080.3	1	105	0.892	1.151	1.088	0.000	0.000	0.000	1	1	1	100

Protein Group

Chain A, Neuronal Synaptic Fusion Complex	gi|6729809	11664.2
Chain A, Three-Dimensional Structure Of The ComplexinSNARE COMPLEX	gi|20150809	8652.7
vesicle associated membrane protein 2B [Rattus norvegicus]	gi|4894188	15698.4
vesicle-associated membrane protein 2 [Mus musculus]	gi|6678551	13881.5
vesicle-associated membrane protein 3 [Mus musculus]	gi|6678553	12717.8

Peptide Information

Calc. Mass	Obsrv. Mass	± da	± ppm	Start Seq.	End Seq.	Sequence	Ion Score	C. I. %	Modification	Plate [#] Name	Gel Idx/Pos [4700 Sample Name]	iTRAQ Ratio 115/114*	iTRAQ Ratio 116/114*	iTRAQ Ratio 117/114*	Rank	Result Type
1954.0127	1953.9033	-0.1094	-56	41	57	ADALQAGASQFETSAAK	105	100	(N-term)_iTRAQ[0], Lysine(K)_iTRAQ[17]	[5] F4	167/159	0.892	1.151	1.088	1	Mascot

537	glutathione S-transferase omega-1 [Rattus norvegicus]	gi|56090550	31209.3	2	105	0.980	1.080	1.067	0.203	0.021	0.112	2	2	2	100

Protein Group

RecName: Full=Glutathione S-transferase omega-1; Short=GSTO-1; AltName: Full=Glutathione-dependent	gi|12585231	31051.2

Peptide Information

Calc. Mass	Obsrv. Mass	± da	± ppm	Start Seq.	End Seq.	Sequence	Ion Score	C. I. %	Modification	Plate [#] Name	Gel Idx/Pos [4700 Sample Name]	iTRAQ Ratio 115/114*	iTRAQ Ratio 116/114*	iTRAQ Ratio 117/114*	Rank	Result Type

Calc. Mass	Obsrv. Mass	± da	± ppm	Start Seq.	End Seq.	Sequence	Ion Score	C. I. %	Modification	Plate [#] Name	Gel Idx/Pos [4700 Sample Name]	iTRAQ Ratio 115/114*	iTRAQ Ratio 116/114*	iTRAQ Ratio 117/114*	Rank	Result Type
1505.8196	1505.8778	0.0582	39	12	25	GSAPPGPVPEGQIR	48	99.961	(N-term)_iTRAQ[0]	[8] F13-15 and F1+2	609/601	1.184	1.060	1.179	1	Mascot
1656.9121	1656.9675	0.0554	33	58	66	NKPEWFFEK	57	99.995	(N-term)_iTRAQ[0], Lysine(K)_iTRAQ[2,9]	[4] F7 and F10+11	1196/1188	0.812	1.102	0.965	1	Mascot

538	aldehyde dehydrogenase X, mitochondrial precursor [Rattus norvegicus]	gi|58865518	62613.6	2	104	1.308	1.497	1.765	0.127	0.217	0.176	2	2	2	100

Peptide Information

Calc. Mass	Obsrv. Mass	± da	± ppm	Start Seq.	End Seq.	Sequence	Ion Score	C. I. %	Modification	Plate [#] Name	Gel Idx/Pos [4700 Sample Name]	iTRAQ Ratio 115/114*	iTRAQ Ratio 116/114*	iTRAQ Ratio 117/114*	Rank	Result Type
1743.7758	1743.7782	0.0024	1	162	174	TIPMDGEHFCFTR	58	99.996	(N-term)_iTRAQ[0], MMTS (C)[10]	[4] F7 and F10+11	355/347	1.435	1.307	1.941	1	Mascot
2266.1501	2266.1479	-0.0022	-1	38	53	YNQLFINNEWHDAVSK	46	99.944	(N-term)_iTRAQ[0], Lysine(K)_iTRAQ[16]	[4] F7 and F10+11	360/352	1.192	1.713	1.604	1	Mascot

539	brain and heart protein NDRG4-B1 [Rattus norvegicus]	gi|37788067	44650.6	2	104	1.549	1.139	1.311	0.113	0.227	0.307	2	2	2	100

Protein Group

brain and heart protein NDRG4-C1 [Rattus norvegicus]	gi|37788071	47017
protein NDRG4 [Rattus norvegicus]	gi|14010841	40801.4

Peptide Information

Calc. Mass	Obsrv. Mass	± da	± ppm	Start Seq.	End Seq.	Sequence	Ion Score	C. I. %	Modification	Plate [#] Name	Gel Idx/Pos [4700 Sample Name]	iTRAQ Ratio 115/114*	iTRAQ Ratio 116/114*	iTRAQ Ratio 117/114*	Rank	Result Type
1798.0068	1797.9744	-0.0324	-18	242	255	DLDINRPGTVPNAK	34	99.039	(N-term)_iTRAQ[0], Lysine(K)_iTRAQ[14]	[4] F7 and F10+11	193/185	1.661	1.366	1.617	1	Mascot
1829.0029	1829.0139	0.011	6	313	325	YFLQGMGYIAHLK	70	100	(N-term)_iTRAQ[0], Lysine(K)_iTRAQ[13]	[4] F7 and F10+11	450/442	1.444	0.949	1.062	1	Mascot

540	transforming, acidic coiled-coil containing protein 1 [Rattus norvegicus]	gi|52486747	94235	1	104	1.616	1.220	1.221	0.000	0.000	0.000	1	1	1	100

Peptide Information

Calc. Mass	Obsrv. Mass	± da	± ppm	Start Seq.	End Seq.	Sequence	Ion Score	C. I. %	Modification	Plate [#] Name	Gel Idx/Pos [4700 Sample Name]	iTRAQ Ratio 115/114*	iTRAQ Ratio 116/114*	iTRAQ Ratio 117/114*	Rank	Result Type
2965.2551	2965.0527	-0.2024	-68	24	50	GSGAGEDEAGGPEGDP EEEDSQAETK	104	100	(N-term)_iTRAQ[0], Lysine(K)_iTRAQ[27]	[1] F3 030912	133/125	1.616	1.220	1.221	1	Mascot

541	keratin, type II cytoskeletal 2 epidermal [Rattus norvegicus]	gi|57114290	74018.4	2	104	0.618	0.801	0.848	0.019	0.065	0.116	2	2	2	100

Peptide Information

Calc. Mass	Obsrv. Mass	± da	± ppm	Start Seq.	End Seq.	Sequence	Ion Score	C. I. %	Modification	Plate [#] Name	Gel Idx/Pos [4700 Sample Name]	iTRAQ Ratio 115/114*	iTRAQ Ratio 116/114*	iTRAQ Ratio 117/114*	Rank	Result Type
1329.7625	1329.7147	-0.0478	-36	186	194	VDPEIQNVK	58	99.996	(N-term)_iTRAQ[0], Lysine(K)_iTRAQ[9]	[7] F5 120912	150/142	0.637	0.741	0.746	1	Mascot
1427.745	1427.6891	-0.0559	-39	482	490	DYQELMNVK	45	99.927	(N-term)_iTRAQ[0], Lysine(K)_iTRAQ[9]	[7] F5 120912	222/214	0.599	0.865	0.965	1	Mascot

542	myelin-associated glycoprotein precursor [Rattus norvegicus]	gi|8393742	72448.4	2	103	2.578	0.894	0.804	0.023	0.214	0.039	2	2	2	100

Protein Group

myelin-associated glycoprotein precursor, short splice form - rat	gi|69988	66919.3

Peptide Information

Calc. Mass	Obsrv. Mass	± da	± ppm	Start Seq.	End Seq.	Sequence	Ion Score	C. I. %	Modification	Plate [#] Name	Gel Idx/Pos [4700 Sample Name]	iTRAQ Ratio 115/114*	iTRAQ Ratio 116/114*	iTRAQ Ratio 117/114*	Rank	Result Type
1431.7465	1431.6432	-0.1033	-72	78	88	TQVVHESFQGR	50	99.974	(N-term)_iTRAQ[0]	[6] F8 110912	143/135	2.601	1.108	0.767	1	Mascot
2075.0793	2075.0613	-0.018	-9	492	508	NLYGTQSLELPFQGAHR	54	99.989	(N-term)_iTRAQ[0]	[7] F5 120912	312/304	2.555	0.722	0.844	1	Mascot

543	ras-related protein Rab-14 [Rattus norvegicus]	gi|16758368	25969.2	2	103	0.681	0.911	0.788	0.081	0.174	0.155	2	2	2	100

Peptide Information

Calc. Mass	Obsrv. Mass	± da	± ppm	Start Seq.	End Seq.	Sequence	Ion Score	C. I. %	Modification	Plate [#] Name	Gel Idx/Pos [4700 Sample Name]	iTRAQ Ratio 115/114*	iTRAQ Ratio 116/114*	iTRAQ Ratio 117/114*	Rank	Result Type

Calc. Mass	Obsrv. Mass	± da	± ppm	Start Seq.	End Seq.	Sequence	Ion Score	C. I. %	Modification	Plate [#] Name	Gel Idx/Pos [4700 Sample Name]	iTRAQ Ratio 115/114*	iTRAQ Ratio 116/114*	iTRAQ Ratio 117/114*	Rank	Result Type
1460.7618	1460.6924	-0.0694	-48	62	72	LQIWDTAGQER	70	100	(N-term)_iTRAQ[0]	[5] F4	197/189	0.762	1.084	0.943	1	Mascot
1970.9028	1970.9073	0.0045	2	36	51	FMADCPHTIGVEFGTR	34	98.917	(N-term)_iTRAQ[0], MMTS (C)[5]	[7] F5 120912	372/364	0.608	0.765	0.659	1	Mascot

544	rho-related GTP-binding protein RhoB precursor [Homo sapiens]	gi|4757764	24638.6	2	103	1.168	0.889	0.986	0.075	0.082	0.332	2	2	2	100

Peptide Information

Calc. Mass	Obsrv. Mass	± da	± ppm	Start Seq.	End Seq.	Sequence	Ion Score	C. I. %	Modification	Plate [#] Name	Gel Idx/Pos [4700 Sample Name]	iTRAQ Ratio 115/114*	iTRAQ Ratio 116/114*	iTRAQ Ratio 117/114*	Rank	Result Type
1899.0575	1899.0776	0.0201	11	105	118	HFCPNVPIILVANK	63	99.999	(N-term)_iTRAQ[0], Lysine(K)_iTRAQ[14], MMTS (C)[3]	[4] F7 and F10+11	419/411	1.243	0.814	1.317	1	Mascot
2153.0271	2153.0405	0.0134	6	52	68	QVELALWDTAGQEDYDR	40	99.753	(N-term)_iTRAQ[0]	[8] F13-15 and F1+2	417/409	1.098	0.971	0.737	1	Mascot

545	LRRGT00046 [Rattus norvegicus]	gi|37654308	73588.4	3	103	1.157	0.846	1.069	0.389	0.444	0.263	3	3	3	100

Protein Group

acyl-CoA-binding protein [Rattus norvegicus] gi|13937379 12326.8

Peptide Information

Calc. Mass	Obsrv. Mass	± da	± ppm	Start Seq.	End Seq.	Sequence	Ion Score	C. I. %	Modification	Plate [#] Name	Gel Idx/Pos [4700 Sample Name]	iTRAQ Ratio 115/114*	iTRAQ Ratio 116/114*	iTRAQ Ratio 117/114*	Rank	Result Type
1123.5782	1123.5079	-0.0703	-63	155	160	WDSWNK	31	98.07	(N-term)_iTRAQ[0], Lysine(K)_iTRAQ[6]	[6] F8 110912	238/230	1.604	1.411	1.389	1	Mascot
1466.8127	1466.7661	-0.0466	-32	153	160	AKWDSWNK	44	99.896	(N-term)_iTRAQ[0], Lysine(K)_iTRAQ[2,8]	[2] F12 040912	237/229	1.218	0.856	1.084	1	Mascot
2274.1362	2274.1245	-0.0117	-5	117	132	TQPTDEEMLFIYSHFK	28	96.297	(N-term)_iTRAQ[0], Lysine(K)_iTRAQ[16]	[4] F7 and F10+11	447/439	0.793	0.502	0.811	1	Mascot

546	RecName: Full=Neural cell adhesion molecule L1; Short=N-CAM-L1; Short=NCAM-L1; AltName: Full=Nerve-	gi|20455467	150706.6	3	103	0.820	1.061	1.036	0.389	0.101	0.096	3	3	3	100

Protein Group

neural cell adhesion molecule L1 [Rattus norvegicus] gi|8393820 150221.3

Peptide Information

Calc. Mass	Obsrv. Mass	± da	± ppm	Start Seq.	End Seq.	Sequence	Ion Score	C. I. %	Modification	Plate [#] Name	Gel Idx/Pos [4700 Sample Name]	iTRAQ Ratio 115/114*	iTRAQ Ratio 116/114*	iTRAQ Ratio 117/114*	Rank	Result Type
1092.6815	1092.7274	0.0459	42	1046	1051	FHILFK	28	95.709	(N-term)_iTRAQ[0], Lysine(K)_iTRAQ[6]	[4] F7 and F10+11	1211/1203	0.495	0.961	1.163	1	Mascot
1499.8944	1499.963	0.0686	46	832	841	WRPVDLAQVK	41	99.801	(N-term)_iTRAQ[0], Lysine(K)_iTRAQ[10]	[4] F7 and F10+11	1181/1173	1.271	1.197	0.938	1	Mascot
1751.8448	1751.8218	-0.023	-13	739	750	WMDWNAPQIQYR	35	99.266	(N-term)_iTRAQ[0]	[1] F3 030912	373/365	0.876	1.038	1.020	1	Mascot

547	ras-related protein Rab-5A [Rattus norvegicus]	gi|12083645	25801.2	2	102	0.942	0.985	0.447	0.249	0.101	0.480	2	2	2	100

Peptide Information

Calc. Mass	Obsrv. Mass	± da	± ppm	Start Seq.	End Seq.	Sequence	Ion Score	C. I. %	Modification	Plate [#] Name	Gel Idx/Pos [4700 Sample Name]	iTRAQ Ratio 115/114*	iTRAQ Ratio 116/114*	iTRAQ Ratio 117/114*	Rank	Result Type
1397.7509	1397.6699	-0.081	-58	198	209	GVDLTEPAQPAR	34	98.985	(N-term)_iTRAQ[0]	[1] F3 030912	174/166	1.191	0.894	0.215	1	Mascot
1495.7301	1495.6857	-0.0444	-30	71	81	FEIWDTAGQER	68	100	(N-term)_iTRAQ[0]	[1] F3 030912	300/292	0.745	1.086	0.927	1	Mascot

548	brain protein 44 [Rattus norvegicus]	gi|117647218	15735.6	2	101	0.750	0.696	0.831	0.055	0.045	0.098	2	2	2	100

Peptide Information

Calc. Mass	Obsrv. Mass	± da	± ppm	Start Seq.	End Seq.	Sequence	Ion Score	C. I. %	Modification	Plate [#] Name	Gel Idx/Pos [4700 Sample Name]	iTRAQ Ratio 115/114*	iTRAQ Ratio 116/114*	iTRAQ Ratio 117/114*	Rank	Result Type
1527.8644	1527.8151	-0.0493	-32	40	49	TVFFWAPIMK	46	99.933	(N-term)_iTRAQ[0], Lysine(K)_iTRAQ[10]	[5] F4	378/370	0.698	0.654	0.743	1	Mascot
1731.0825	1731.0099	-0.0726	-42	16	26	LMDKVELLLPK	55	99.993	(N-term)_iTRAQ[0], Lysine(K)_iTRAQ[4,11]	[6] F8 110912	439/431	0.805	0.741	0.929	1	Mascot

549	N(G),N(G)-dimethylarginine dimethylaminohydrolase 1 [Rattus norvegicus]	gi|11560131	34465.8	2	100	1.013	1.011	1.069	0.221	0.284	0.241	2	2	2	100

Peptide Information

Calc. Mass	Obsrv. Mass	± da	± ppm	Start	End	Sequence	Ion	C. I. %	Modification	Plate [#] Name	Gel Idx/Pos [4700	iTRAQ	iTRAQ	iTRAQ	Rank	Result Type

Calc. Mass	Obsrv. Mass	± da	± ppm	Start Seq.	End Seq.	Sequence	Ion Score	C. I. %	Modification	Plate [#] Name	Gel Idx/Pos [4700 Sample Name]	iTRAQ Ratio 115/114*	iTRAQ Ratio 116/114*	iTRAQ Ratio 117/114*	Rank	Result Type
1214.7031	1214.6616	-0.0415	-34	137	144	EFFVGLSK	49	99.967	(N-term)_iTRAQ[0], Lysine(K)_iTRAQ[8]	[7] F5 120912	275/267	1.234	1.296	1.310	1	Mascot
2432.1851	2432.0874	-0.0977	-40	176	196	SFCSMAGPNLIAIGSSES AQK	52	99.984	(N-term)_iTRAQ[0], Lysine(K)_iTRAQ[21], MMTS (C)[3]	[1] F3 030912	428/420	0.831	0.789	0.873	1	Mascot

550	sideroflexin-5 [Rattus norvegicus]	gi|23463279	39436.2	2	100	1.200	1.089	1.237	0.132	0.153	0.029	3	3	3	100

Peptide Information

Calc. Mass	Obsrv. Mass	± da	± ppm	Start Seq.	End Seq.	Sequence	Ion Score	C. I. %	Modification	Plate [#] Name	Gel Idx/Pos [4700 Sample Name]	iTRAQ Ratio 115/114*	iTRAQ Ratio 116/114*	iTRAQ Ratio 117/114*	Rank	Result Type
1277.6399	1277.5612	-0.0787	-62	34	42	FQQTSFYGR	30	97.647	(N-term)_iTRAQ[0]	[5] F4	137/129	1.103	0.905	1.274	1	Mascot
1553.8534	1553.743	-0.1104	-71	94	104	QAILHPDTNEK	35	99.276	(N-term)_iTRAQ[0], Lysine(K)_iTRAQ[11]	[6] F8 110912	143/135	1.127	1.205	1.205	1	Mascot
1553.8534	1553.8971	0.0437	28	94	104	QAILHPDTNEK	69	100	(N-term)_iTRAQ[0], Lysine(K)_iTRAQ[11]	[3] F6 and F9	1107/1099	1.390	1.186	1.234	1	Mascot

551	hepatoma-derived growth factor-related protein 3 [Rattus norvegicus]	gi|21955178	26657.9	2	99	0.794	1.055	0.876	0.195	0.241	0.136	2	2	2	100

Peptide Information

Calc. Mass	Obsrv. Mass	± da	± ppm	Start Seq.	End Seq.	Sequence	Ion Score	C. I. %	Modification	Plate [#] Name	Gel Idx/Pos [4700 Sample Name]	iTRAQ Ratio 115/114*	iTRAQ Ratio 116/114*	iTRAQ Ratio 117/114*	Rank	Result Type
2010.1606	2010.1567	-0.0039	-2	29	43	IDELPEGAVKPPANK	61	99.998	(N-term)_iTRAQ[0], Lysine(K)_iTRAQ[10, 15]	[4] F7 and F10+11	223/215	0.637	0.859	0.758	1	Mascot
2363.2729	2363.3408	0.0679	29	79	95	KGFNEGLWEIENNPGVK	39	99.658	(N-term)_iTRAQ[0], Lysine(K)_iTRAQ[1,1 7]	[4] F7 and F10+11	1216/1208	0.989	1.295	1.012	1	Mascot

552	RecName: Full=Fatty acid synthase; Includes: RecName: Full=[Acyl-carrier-protein] S-acetyltransfera	gi|2506136	289435.8	2	98	1.664	1.006	0.875	0.105	0.138	0.122	2	2	2	100

Protein Group

fatty acid synthase [Rattus norvegicus]	gi|8394158	289437.8
fatty acid synthase [Rattus norvegicus]	gi|204099	289451.8
unnamed protein product [Rattus norvegicus]	gi|56133	280208.9

Peptide Information

Calc. Mass	Obsrv. Mass	± da	± ppm	Start Seq.	End Seq.	Sequence	Ion Score	C. I. %	Modification	Plate [#] Name	Gel Idx/Pos [4700 Sample Name]	iTRAQ Ratio 115/114*	iTRAQ Ratio 116/114*	iTRAQ Ratio 117/114*	Rank	Result Type
1554.7687	1554.6913	-0.0774	-50	958	967	VYQWEDPDSK	41	99.806	(N-term)_iTRAQ[0], Lysine(K)_iTRAQ[10]	[7] F5 120912	182/174	1.566	0.885	0.997	1	Mascot
1947.8739	1947.7609	-0.113	-58	2021	2037	GNAGQSNYGFANSTMER	58	99.996	(N-term)_iTRAQ[0]	[1] F3 030912	196/188	1.769	1.144	0.768	1	Mascot

553	carbonyl reductase [NADPH] 1 [Rattus norvegicus]	gi|9506467	33815	2	99	1.548	1.463	1.494	0.652	0.221	0.013	2	2	2	100

Protein Group

PREDICTED: inducible carbonyl reductase-like [Rattus norvegicus]	gi|293352109	33746.9

Peptide Information

Calc. Mass	Obsrv. Mass	± da	± ppm	Start Seq.	End Seq.	Sequence	Ion Score	C. I. %	Modification	Plate [#] Name	Gel Idx/Pos [4700 Sample Name]	iTRAQ Ratio 115/114*	iTRAQ Ratio 116/114*	iTRAQ Ratio 117/114*	Rank	Result Type
1596.8268	1596.791	-0.0358	-22	187	198	EGWPNSAYGVTK	56	99.994	(N-term)_iTRAQ[0], Lysine(K)_iTRAQ[12]	[7] F5 120912	191/183	2.200	1.684	1.482	1	Mascot
1726.8997	1726.8754	-0.0243	-14	59	71	FHQLDIDNPQSIR	43	99.862	(N-term)_iTRAQ[0]	[4] F7 and F10+11	265/257	1.089	1.271	1.507	1	Mascot

554	Adh5 protein [Rattus norvegicus]	gi|54035294	45186.4	3	99	0.855	0.640	0.651	0.012	0.138	0.263	3	3	3	100

Protein Group

Ac1002 [Rattus norvegicus]	gi|32264595	104665

Peptide Information

Calc. Mass	Obsrv. Mass	± da	± ppm	Start	End	Sequence	Ion	C. I. %	Modification	Plate [#] Name	Gel Idx/Pos [4700	iTRAQ	iTRAQ	iTRAQ	Rank	Result Type

				Seq.	Seq.		Score				Sample Name]	Ratio 115/114*	Ratio 116/114*	Ratio 117/114*		
1111.6146	1111.6176	0.003	3	321	328	GTAFGGWK	35	99.146	(N-term)_iTRAQ[0], Lysine(K)_iTRAQ[8]	[4] F7 and F10+11	230/222	0.862	0.579	0.878	1	Mascot
2019.9382	2019.8773	-0.0609	-30	239	253	EFGATECINPQDFSK	33	98.662	(N-term)_iTRAQ[0], Lysine(K)_iTRAQ[15], MMTS (C)[7]	[5] F4	265/257	0.839	0.841	0.776	1	Mascot
2499.3831	2499.3577	-0.0254	-10	345	362	IKVDEFVTGNLSFDQINK	31	98.186	(N-term)_iTRAQ[0], Lysine(K)_iTRAQ[2,18]	[4] F7 and F10+11	399/391	0.865	0.539	0.406	1	Mascot

555 Scamp1 protein [Rattus norvegicus] gi|60422761 31937.2 1 98 1.261 1.144 1.427 0.000 0.000 0.000 1 1 1 100

Protein Group

secretory carrier-associated membrane protein 1 [Rattus norvegicus] gi|158749626 40601.4

Peptide Information

Calc. Mass	Obsrv. Mass	± da	± ppm	Start Seq.	End Seq.	Sequence	Ion Score	C. I. %	Modification	Plate [#] Name	Gel Idx/Pos [4700 Sample Name]	iTRAQ Ratio 115/114*	iTRAQ Ratio 116/114*	iTRAQ Ratio 117/114*	Rank	Result Type
1698.873	1698.7915	-0.0815	-48	224	236	AQQEFATGVMSNK	98	100	(N-term)_iTRAQ[0], Lysine(K)_iTRAQ[13]	[7] F5 120912	174/166	1.261	1.144	1.427	1	Mascot

556 malic enzyme [Rattus norvegicus] gi|205294 71216.3 2 98 1.102 1.073 0.823 0.175 0.132 0.173 2 2 2 100

Protein Group

RecName: Full=NADP-dependent malic enzyme; Short=NADP-ME; AltName: Full=Malic enzyme 1 gi|266504 69943.8

Peptide Information

Calc. Mass	Obsrv. Mass	± da	± ppm	Start Seq.	End Seq.	Sequence	Ion Score	C. I. %	Modification	Plate [#] Name	Gel Idx/Pos [4700 Sample Name]	iTRAQ Ratio 115/114*	iTRAQ Ratio 116/114*	iTRAQ Ratio 117/114*	Rank	Result Type
2023.1222	2023.1163	-0.0059	-3	131	146	GHIASVLNAWPEDVVK	31	98.248	(N-term)_iTRAQ[0], Lysine(K)_iTRAQ[16]	[4] F7 and F10+11	383/375	0.951	0.955	0.995	1	Mascot
2295.0737	2295.0417	-0.032	-14	218	235	GPEYDAFLDEFMEAASSK	67	99.999	(N-term)_iTRAQ[0], Lysine(K)_iTRAQ[18]	[1] F3 030912	517/509	1.276	1.204	0.680	1	Mascot

557 glycerol-3-phosphate dehydrogenase, mitochondrial precursor [Rattus norvegicus] gi|6980978 87191.2 3 98 0.524 0.942 0.712 0.301 0.107 0.479 3 3 3 100

Peptide Information

Calc. Mass	Obsrv. Mass	± da	± ppm	Start Seq.	End Seq.	Sequence	Ion Score	C. I. %	Modification	Plate [#] Name	Gel Idx/Pos [4700 Sample Name]	iTRAQ Ratio 115/114*	iTRAQ Ratio 116/114*	iTRAQ Ratio 117/114*	Rank	Result Type
1465.7771	1465.7002	-0.0769	-52	125	135	AITNLDVEQYR	28	96.314	(N-term)_iTRAQ[0]	[1] F3 030912	246/238	0.955	1.071	0.959	1	Mascot
1466.8657	1466.8375	-0.0282	-19	340	348	VIFFLPWEK	35	99.244	(N-term)_iTRAQ[0], Lysine(K)_iTRAQ[9]	[7] F5 120912	482/474	0.319	0.949	1.089	1	Mascot
1812.004	1811.9344	-0.0696	-38	526	538	LVSEFPYIEAEVK	35	99.173	(N-term)_iTRAQ[0], Lysine(K)_iTRAQ[13]	[1] F3 030912	397/389	0.472	0.822	0.345	1	Mascot

558 heterogeneous nuclear ribonucleoprotein Q [Rattus norvegicus] gi|114145636 65287.4 2 98 0.982 1.187 0.905 0.020 0.093 0.394 2 2 2 100

Peptide Information

Calc. Mass	Obsrv. Mass	± da	± ppm	Start Seq.	End Seq.	Sequence	Ion Score	C. I. %	Modification	Plate [#] Name	Gel Idx/Pos [4700 Sample Name]	iTRAQ Ratio 115/114*	iTRAQ Ratio 116/114*	iTRAQ Ratio 117/114*	Rank	Result Type
1721.7894	1721.8848	0.0954	55	197	207	GFCFLEYEDHK	56	99.994	(N-term)_iTRAQ[0], Lysine(K)_iTRAQ[11], MMTS (C)[3]	[3] F6 and F9	1320/1312	1.002	1.280	1.299	1	Mascot
1882.0095	1881.9462	-0.0633	-34	82	94	DLFEDELVPLFEK	42	99.836	(N-term)_iTRAQ[0], Lysine(K)_iTRAQ[13]	[1] F3 030912	500/492	0.963	1.101	0.630	1	Mascot

559 disks large-associated protein 4 [Rattus norvegicus] gi|27465621 116918 1 97 0.659 0.750 0.780 0.000 0.000 0.000 1 1 1 100

Peptide Information

Calc. Mass	Obsrv. Mass	± da	± ppm	Start Seq.	End Seq.	Sequence	Ion Score	C. I. %	Modification	Plate [#] Name	Gel Idx/Pos [4700 Sample Name]	iTRAQ Ratio 115/114*	iTRAQ Ratio 116/114*	iTRAQ Ratio 117/114*	Rank	Result Type
2380.1309	2379.9717	-0.1592	-67	371	390	AMGDEDSDESGGGSPKPSPK	97	100	(N-term)_iTRAQ[0], Lysine(K)_iTRAQ[16,20]	[6] F8 110912	105/97	0.659	0.750	0.780	1	Mascot

560 phytanoyl-CoA hydroxylase-interacting protein-like [Rattus norvegicus] gi|58865722 41924.4 2 97 1.011 0.857 0.941 0.087 0.012 0.068 2 2 2 100

Peptide Information

Calc. Mass	Obsrv. Mass	± da	± ppm	Start Seq.	End Seq.	Sequence	Ion Score	C. I. %	Modification	Plate [#] Name	Gel Idx/Pos [4700 Sample Name]	iTRAQ Ratio 115/114*	iTRAQ Ratio 116/114*	iTRAQ Ratio 117/114*	Rank	Result Type
1143.6184	1143.6781	0.0597	52	74	81	GHWFLSPR	41	99.793	(N-term)_iTRAQ[0]	[4] F7 and F10+11	1165/1157	0.932	0.846	0.877	1	Mascot
1824.0754	1824.0271	-0.0483	-26	34	44	ITHYFIDLNKK	57	99.995	(N-term)_iTRAQ[0], Lysine(K)_iTRAQ[10, 11]	[2] F12 040912	320/312	1.098	0.869	1.009	1	Mascot

561	flotillin-2 [Rattus norvegicus]	gi|13929186	52498.1	2	96	1.013	1.135	0.886	0.155	0.490	0.170	2	2	2	100

Protein Group

Flot2 protein [Rattus norvegicus]	gi|53733398	43541
R-Reggie-1.1 [Rattus norvegicus]	gi|4097589	43685.1
flotillin-2 isoform 1 [Mus musculus]	gi|94536791	52374.1
reggie1-4 [Rattus norvegicus]	gi|4079713	46808.6

Peptide Information

Calc. Mass	Obsrv. Mass	± da	± ppm	Start Seq.	End Seq.	Sequence	Ion Score	C. I. %	Modification	Plate [#] Name	Gel Idx/Pos [4700 Sample Name]	iTRAQ Ratio 115/114*	iTRAQ Ratio 116/114*	iTRAQ Ratio 117/114*	Rank	Result Type
1303.7467	1303.7014	-0.0453	-35	297	305	IQQIAEGEK	58	99.996	(N-term)_iTRAQ[0], Lysine(K)_iTRAQ[9]	[7] F5 120912	149/141	0.878	0.793	1.056	1	Mascot
1754.9412	1754.9022	-0.039	-22	82	94	ELLAVACEQFLGK	39	99.667	(N-term)_iTRAQ[0], Lysine(K)_iTRAQ[13], MMTS (C)[7]	[5] F4	419/411	1.168	1.625	0.743	1	Mascot

562	disks large homolog 2 [Rattus norvegicus]	gi|11560113	102672.5	3	96	1.279	1.177	1.378	0.577	0.275	0.343	4	4	4	100

Protein Group

RecName: Full=Disks large homolog 2; AltName: Full=Channel-associated protein of synapse-110; Short	gi|2497503	102736.5
isoform of PSD-95/SAP90 [Rattus norvegicus]	gi|1517940	102549.3
synaptic density protein PSD-93 [Rattus norvegicus]	gi|1256761	100856.4

Peptide Information

Calc. Mass	Obsrv. Mass	± da	± ppm	Start Seq.	End Seq.	Sequence	Ion Score	C. I. %	Modification	Plate [#] Name	Gel Idx/Pos [4700 Sample Name]	iTRAQ Ratio 115/114*	iTRAQ Ratio 116/114*	iTRAQ Ratio 117/114*	Rank	Result Type
1693.8894	1693.8003	-0.0891	-53	675	686	INDDLISEFPDK	29	97.099	(N-term)_iTRAQ[0], Lysine(K)_iTRAQ[12]	[5] F4	244/236	0.729	0.897	1.105	1	Mascot
1716.9014	1716.7856	-0.1158	-67	153	165	VNEVDVSEVSHSK	32	98.533	(N-term)_iTRAQ[0], Lysine(K)_iTRAQ[13]	[6] F8 110912	178/170	2.075	1.400	1.967	1	Mascot
1763.9299	1763.9353	0.0054	3	643	656	GQEDLILSYEPVTR	29	96.877	(N-term)_iTRAQ[0]	[8] F13-15 and F1+2	458/450	1.310	1.030	1.188	1	Mascot
1763.9299	1763.9357	0.0058	3	643	656	GQEDLILSYEPVTR	35	99.182	(N-term)_iTRAQ[0]	[8] F13-15 and F1+2	462/454	1.350	1.486	1.395	1	Mascot

563	proteasome subunit beta type-7 precursor [Rattus norvegicus]	gi|16758298	33066.2	2	96	0.932	0.935	0.802	0.085	0.006	0.067	2	2	2	100

Peptide Information

Calc. Mass	Obsrv. Mass	± da	± ppm	Start Seq.	End Seq.	Sequence	Ion Score	C. I. %	Modification	Plate [#] Name	Gel Idx/Pos [4700 Sample Name]	iTRAQ Ratio 115/114*	iTRAQ Ratio 116/114*	iTRAQ Ratio 117/114*	Rank	Result Type
1668.8148	1668.8353	0.0205	12	185	195	FRPDMEEEEAK	38	99.591	(N-term)_iTRAQ[0], Lysine(K)_iTRAQ[11]	[3] F6 and F9	1142/1134	1.018	0.941	0.869	1	Mascot
1941.0123	1940.9302	-0.0821	-42	185	196	FRPDMEEEEAKK	58	99.996	(N-term)_iTRAQ[0], Lysine(K)_iTRAQ[11, 12]	[2] F12 040912	186/178	0.854	0.930	0.740	1	Mascot

564	ribosomal protein L5 [Rattus norvegicus]	gi|206734	39389.1	3	96	0.816	1.028	0.799	0.356	0.385	0.825	3	3	3	100

Protein Group

60S ribosomal protein L5 [Rattus norvegicus]	gi|13592051	39520.1

Peptide Information

Calc. Mass	Obsrv. Mass	± da	± ppm	Start	End Sequence	Ion	C. I. %	Modification	Plate [#] Name	Gel Idx/Pos [4700	iTRAQ	iTRAQ	iTRAQ	Rank	Result Type

Calc. Mass	Obsrv. Mass	± da	± ppm	Start Seq.	End Seq.	Sequence	Ion Score	C. I. %	Modification	Plate [#] Name	Gel Idx/Pos [4700 Sample Name]	iTRAQ Ratio 115/114*	iTRAQ Ratio 116/114*	iTRAQ Ratio 117/114*	Rank	Result Type
1201.6827	1201.6327	-0.05	-42	222	228	QFSQYIK	32	98.602	(N-term)_iTRAQ[0], Lysine(K)_iTRAQ[7]	[3] F6 and F9	224/216	1.291	1.560	1.629	1	Mascot
1578.7642	1578.6882	-0.076	-48	198	209	HIMGQNVADYMR	31	97.829	(N-term)_iTRAQ[0]	[6] F8 110912	259/251	0.791	0.965	1.033	1	Mascot
1965.9851	1965.8619	-0.1232	-63	210	221	YLMEEDEDAYKK	33	98.757	(N-term)_iTRAQ[0], Lysine(K)_iTRAQ[11, 12]	[6] F8 110912	233/225	0.533	0.721	0.304	1	Mascot

565 FK506 binding protein 4 [Rattus norvegicus] | gi|22324680 | 50967.4 | 1 | 96 | 1.823 | 1.461 | 1.047 | 0.000 | 0.000 | 0.000 | 1 | 1 | 1 | 100

Peptide Information

Calc. Mass	Obsrv. Mass	± da	± ppm	Start Seq.	End Seq.	Sequence	Ion Score	C. I. %	Modification	Plate [#] Name	Gel Idx/Pos [4700 Sample Name]	iTRAQ Ratio 115/114*	iTRAQ Ratio 116/114*	iTRAQ Ratio 117/114*	Rank	Result Type
1859.9055	1859.8258	-0.0797	-43	371	385	TEVAAGDHPTDAEMK	96	100	(N-term)_iTRAQ[0], Lysine(K)_iTRAQ[15]	[4] F7 and F10+11	134/126	1.823	1.461	1.047	1	Mascot

566 26S protease regulatory subunit 6A [Mus musculus] | gi|228008337 | 55079.3 | 2 | 96 | 0.770 | 1.113 | 1.224 | 0.127 | 0.266 | 0.097 | 2 | 2 | 2 | 100

Protein Group

RecName: Full=26S protease regulatory subunit 6A; AltName: Full=26S proteasome AAA-ATPase subunit R	gi|2492523	54691.1

Peptide Information

Calc. Mass	Obsrv. Mass	± da	± ppm	Start Seq.	End Seq.	Sequence	Ion Score	C. I. %	Modification	Plate [#] Name	Gel Idx/Pos [4700 Sample Name]	iTRAQ Ratio 115/114*	iTRAQ Ratio 116/114*	iTRAQ Ratio 117/114*	Rank	Result Type
2392.3711	2392.3967	0.0256	11	280	297	EKAPSIIFIDELDAIGTK	28	96.348	(N-term)_iTRAQ[0], Lysine(K)_iTRAQ[2,18]	[4] F7 and F10+11	486/478	0.661	0.898	1.321	1	Mascot
2477.332	2477.2693	-0.0627	-25	312	330	TMLELLNQLDGFQPNTQVK	68	100	(N-term)_iTRAQ[0], Lysine(K)_iTRAQ[19]	[1] F3 030912	521/513	0.896	1.379	1.134	1	Mascot

567 LIM domain only protein 7 [Rattus norvegicus] | gi|48040390 | 212025.7 | 3 | 96 | 0.462 | 1.201 | 1.081 | 0.984 | 1.077 | 1.243 | 3 | 3 | 3 | 100

Peptide Information

Calc. Mass	Obsrv. Mass	± da	± ppm	Start Seq.	End Seq.	Sequence	Ion Score	C. I. %	Modification	Plate [#] Name	Gel Idx/Pos [4700 Sample Name]	iTRAQ Ratio 115/114*	iTRAQ Ratio 116/114*	iTRAQ Ratio 117/114*	Rank	Result Type
1308.6191	1308.5562	-0.0629	-48	487	496	FSEPDDAVER	28	96.068	(N-term)_iTRAQ[0]	[1] F3 030912	172/164	0.092	0.522	0.448	1	Mascot
1388.689	1388.5947	-0.0943	-68	1413	1424	AEAQAQAEAEAR	41	99.789	(N-term)_iTRAQ[0]	[5] F4	45/37	1.051	2.473	2.896	1	Mascot
1551.7512	1551.7313	-0.0199	-13	372	381	GWTPEDMNWK	27	95.005	(N-term)_iTRAQ[0], Lysine(K)_iTRAQ[10]	[3] F6 and F9	323/315	1.019	1.343	0.973	1	Mascot

568 disks large-associated protein 3 [Rattus norvegicus] | gi|27465607 | 112932.8 | 2 | 95 | 0.706 | 0.579 | 0.739 | 0.018 | 0.111 | 0.303 | 2 | 2 | 2 | 100

Protein Group

RecName: Full=Disks large-associated protein 3; Short=DAP-3; AltName: Full=PSD-95/SAP90-binding pro	gi|71153510	112902.8
synapse-associated protein 90/postsynaptic density-95-associated protein 3(-) [Rattus norvegicus]	gi|46389546	111702.2

Peptide Information

Calc. Mass	Obsrv. Mass	± da	± ppm	Start Seq.	End Seq.	Sequence	Ion Score	C. I. %	Modification	Plate [#] Name	Gel Idx/Pos [4700 Sample Name]	iTRAQ Ratio 115/114*	iTRAQ Ratio 116/114*	iTRAQ Ratio 117/114*	Rank	Result Type
1534.7379	1534.7805	0.0426	28	328	339	SAWHTMMVSQGR	34	98.961	(N-term)_iTRAQ[0]	[3] F6 and F9	1190/1182	0.724	0.690	0.524	1	Mascot
1646.837	1646.8899	0.0529	32	145	162	GPAGPGPGPGSGAAPEAR	61	99.998	(N-term)_iTRAQ[0]	[8] F13-15 and F1+2	667/659	0.688	0.485	1.043	1	Mascot

569 tyrosine-protein phosphatase non-receptor type 5 [Rattus norvegicus] | gi|94400885 | 45006.3 | 2 | 95 | 1.030 | 0.729 | 1.030 | 0.066 | 0.158 | 0.018 | 2 | 2 | 2 | 100

Peptide Information

Calc. Mass	Obsrv. Mass	± da	± ppm	Start Seq.	End Seq.	Sequence	Ion Score	C. I. %	Modification	Plate [#] Name	Gel Idx/Pos [4700 Sample Name]	iTRAQ Ratio 115/114*	iTRAQ Ratio 116/114*	iTRAQ Ratio 117/114*	Rank	Result Type
1782.885	1782.9401	0.0551	31	257	267	HYWFTSWPDQK	55	99.992	(N-term)_iTRAQ[0], Lysine(K)_iTRAQ[11]	[4] F7 and F10+11	1245/1237	0.968	0.888	1.012	1	Mascot
2163.165	2163.1147	-0.0503	-23	196	211	TPIIVMITNIEEMNEK	40	99.756	(N-term)_iTRAQ[0], Lysine(K)_iTRAQ[16]	[1] F3 030912	544/536	1.097	0.599	1.048	1	Mascot

570 metabotropic glutamate receptor 3 precursor [Rattus norvegicus] | gi|157787068 | 107042.1 | 3 | 95 | 1.049 | 0.978 | 0.910 | 0.517 | 0.425 | 0.192 | 3 | 3 | 3 | 100

Peptide Information

Calc. Mass	Obsrv. Mass	± da	± ppm	Start Seq.	End Seq.	Sequence	Ion Score	C. I. %	Modification	Plate [#] Name	Gel Idx/Pos [4700 Sample Name]	iTRAQ Ratio 115/114*	iTRAQ Ratio 116/114*	iTRAQ Ratio 117/114*	Rank	Result Type
1140.5935	1140.5709	-0.0226	-20	353	358	DFWEQK	27	95.13	(N-term)_iTRAQ[0], Lysine(K)_iTRAQ[6]	[4] F7 and F10+11	228/220	0.604	0.626	0.700	1	Mascot
1656.8955	1656.8326	-0.0629	-38	466	477	YNVFNLQQTGGK	32	98.516	(N-term)_iTRAQ[0], Lysine(K)_iTRAQ[12]	[7] F5 120912	241/233	1.235	0.988	1.098	1	Mascot
1908.955	1908.9686	0.0136	7	376	389	HLAIDSSNYEQESK	36	99.355	(N-term)_iTRAQ[0], Lysine(K)_iTRAQ[14]	[3] F6 and F9	1142/1134	1.547	1.514	0.981	1	Mascot

571 basigin isoform 1 [Rattus norvegicus] | gi|158081773 | 45370.9 | 1 | 95 | 1.217 | 1.090 | 0.940 | 0.077 | 0.056 | 0.084 | 2 | 2 | 2 | 100

Protein Group

basigin isoform 2 [Rattus norvegicus] | gi|7709992 | 32102.6

Peptide Information

Calc. Mass	Obsrv. Mass	± da	± ppm	Start Seq.	End Seq.	Sequence	Ion Score	C. I. %	Modification	Plate [#] Name	Gel Idx/Pos [4700 Sample Name]	iTRAQ Ratio 115/114*	iTRAQ Ratio 116/114*	iTRAQ Ratio 117/114*	Rank	Result Type
2102.0593	2101.9617	-0.0976	-46	244	258	SEASHPPVDEWVWFK	95	100	(N-term)_iTRAQ[0], Lysine(K)_iTRAQ[15]	[6] F8 110912	402/394	1.294	1.037	1.023	1	Mascot
2102.0593	2102.0449	-0.0144	-7	244	258	SEASHPPVDEWVWFK	54	99.99	(N-term)_iTRAQ[0], Lysine(K)_iTRAQ[15]	[4] F7 and F10+11	399/391	1.144	1.145	0.863	1	Mascot

572 acyl-CoA hydrolase [Rattus norvegicus] | gi|61651581 | 46636.3 | 2 | 94 | 1.014 | 1.271 | 1.199 | 0.168 | 0.693 | 0.163 | 2 | 2 | 2 | 100

Protein Group

cytosolic acyl coenzyme A thioester hydrolase [Rattus norvegicus] | gi|2130523 | 41078.2

cytosolic peroxisome proliferator-induced acyl-coA thioesterase [Rattus norvegicus] | gi|1915948 | 41173.4

Peptide Information

Calc. Mass	Obsrv. Mass	± da	± ppm	Start Seq.	End Seq.	Sequence	Ion Score	C. I. %	Modification	Plate [#] Name	Gel Idx/Pos [4700 Sample Name]	iTRAQ Ratio 115/114*	iTRAQ Ratio 116/114*	iTRAQ Ratio 117/114*	Rank	Result Type
1441.8903	1441.9045	0.0142	10	174	184	VLEVPPIVYLR	41	99.822	(N-term)_iTRAQ[0]	[8] F13-15 and F1+2	378/370	1.181	1.964	1.362	1	Mascot
1578.9506	1578.8787	-0.0719	-46	159	169	ATLWYVPLSLK	53	99.988	(N-term)_iTRAQ[0], Lysine(K)_iTRAQ[11]	[5] F4	348/340	0.870	0.822	1.055	1	Mascot

573 microtubule associated protein [Rattus norvegicus] | gi|517394 | 44949.1 | 3 | 94 | 0.841 | 1.080 | 0.960 | 0.353 | 0.295 | 0.674 | 3 | 3 | 3 | 100

Peptide Information

Calc. Mass	Obsrv. Mass	± da	± ppm	Start Seq.	End Seq.	Sequence	Ion Score	C. I. %	Modification	Plate [#] Name	Gel Idx/Pos [4700 Sample Name]	iTRAQ Ratio 115/114*	iTRAQ Ratio 116/114*	iTRAQ Ratio 117/114*	Rank	Result Type
1210.6917	1210.6266	-0.0651	-54	145	154	TPSLPTPPTR	28	95.855	(N-term)_iTRAQ[0]	[1] F3 030912	201/193	0.512	0.770	0.474	1	Mascot
1597.9233	1597.8442	-0.0791	-50	176	187	LQTAPVPMPDLK	38	99.575	(N-term)_iTRAQ[0], Lysine(K)_iTRAQ[12]	[5] F4	217/209	1.098	1.232	1.089	1	Mascot
2612.2131	2612.0281	-0.185	-71	34	57	AEEAGIGDTPNMEDQAAGHVTQAR	29	96.669	(N-term)_iTRAQ[0]	[5] F4	134/126	1.056	1.328	1.713	1	Mascot

574 trifunctional enzyme subunit beta, mitochondrial precursor [Rattus norvegicus] | gi|19424338 | 56655.1 | 2 | 94 | 2.203 | 1.205 | 2.484 | 1.716 | 1.599 | 2.846 | 2 | 2 | 2 | 100

Peptide Information

Calc. Mass	Obsrv. Mass	± da	± ppm	Start Seq.	End Seq.	Sequence	Ion Score	C. I. %	Modification	Plate [#] Name	Gel Idx/Pos [4700 Sample Name]	iTRAQ Ratio 115/114*	iTRAQ Ratio 116/114*	iTRAQ Ratio 117/114*	Rank	Result Type
1835.7965	1835.7263	-0.0702	-38	393	406	AMDSDWFAQNYMGR	31	98.17	(N-term)_iTRAQ[0]	[5] F4	270/262	3.919	2.804	5.330	1	Mascot
2977.4485	2977.3589	-0.0896	-30	205	230	LNFLSPELPAVAEFSTNETMGHSADR	63	99.999	(N-term)_iTRAQ[0]	[1] F3 030912	468/460	1.238	0.518	1.158	1	Mascot

575 probable saccharopine dehydrogenase [Rattus norvegicus] | gi|62078699 | 51460.9 | 3 | 94 | 1.323 | 0.979 | 1.274 | 0.610 | 0.204 | 0.378 | 3 | 3 | 3 | 100

Peptide Information

Calc. Mass	Obsrv. Mass	± da	± ppm	Start Seq.	End Seq.	Sequence	Ion Score	C. I. %	Modification	Plate [#] Name	Gel Idx/Pos [4700 Sample Name]	iTRAQ Ratio 115/114*	iTRAQ Ratio 116/114*	iTRAQ Ratio 117/114*	Rank	Result Type
1226.7031	1226.6583	-0.0448	-37	104	111	FYGEPVVK	30	97.615	(N-term)_iTRAQ[0], Lysine(K)_iTRAQ[8]	[7] F5 120912	186/178	0.832	0.750	0.952	1	Mascot
1245.6725	1245.6283	-0.0442	-35	199	207	SAIYGFGDK	30	97.491	(N-term)_iTRAQ[0], Lysine(K)_iTRAQ[9]	[3] F6 and F9	224/216	1.322	1.094	1.214	1	Mascot

Calc. Mass	Obsrv. Mass	± da	± ppm	Start Seq.	End Seq.	Sequence	Ion Score	C. I. %	Modification	Plate [#] Name	Gel Idx/Pos [4700 Sample Name]	iTRAQ Ratio 115/114*	iTRAQ Ratio 116/114*	iTRAQ Ratio 117/114*	Rank	Result Type
1367.7191	1367.6312	-0.0879	-64	395	407	GGGVFTPGAAFSR	34	98.994	(N-term)_iTRAQ[0]	[5] F4	182/174	2.106	1.144	1.790	1	Mascot

576	heterogeneous nuclear ribonucleoprotein A/B [Rattus norvegicus]	gi|140971918	35249.3	2	94	2.096	2.084	2.029	3.124	1.874	1.447	2	2	2	100

Protein Group

AIF-C1 [Rattus norvegicus]	gi|3377279	40598.4
hypothetical protein LOC100382653 [Zea mays]	gi|293334069	40625.5
nucleic acid binding factor pRM10 [Rattus norvegicus]	gi|4378711	39061.3

Peptide Information

Calc. Mass	Obsrv. Mass	± da	± ppm	Start Seq.	End Seq.	Sequence	Ion Score	C. I. %	Modification	Plate [#] Name	Gel Idx/Pos [4700 Sample Name]	iTRAQ Ratio 115/114*	iTRAQ Ratio 116/114*	iTRAQ Ratio 117/114*	Rank	Result Type
1216.734	1216.7163	-0.0177	-15	117	124	GFGFILFK	31	98.19	(N-term)_iTRAQ[0], Lysine(K)_iTRAQ[8]	[7] F5 120912	431/423	5.220	3.958	3.475	1	Mascot
1791.9738	1791.9023	-0.0715	-40	161	174	IFVGGLNPEATEEK	62	99.999	(N-term)_iTRAQ[0], Lysine(K)_iTRAQ[14]	[5] F4	212/204	0.842	1.097	1.184	1	Mascot

577	four and a half LIM domains protein 1 isoform 2 [Rattus norvegicus]	gi|21728390	28731.7	1	94	1.068	1.029	0.850	0.000	0.000	0.000	1	1	1	100

Protein Group

RecName: Full=Four and a half LIM domains protein 1; Short=FHL-1	gi|81907626	38633.1
four and a half LIM domains protein 1 isoform 1 [Rattus norvegicus]	gi|76781465	40451

Peptide Information

Calc. Mass	Obsrv. Mass	± da	± ppm	Start Seq.	End Seq.	Sequence	Ion Score	C. I. %	Modification	Plate [#] Name	Gel Idx/Pos [4700 Sample Name]	iTRAQ Ratio 115/114*	iTRAQ Ratio 116/114*	iTRAQ Ratio 117/114*	Rank	Result Type
1594.8687	1594.8245	-0.0442	-28	123	134	AIVAGDQNVEYK	94	100	(N-term)_iTRAQ[0], Lysine(K)_iTRAQ[12]	[7] F5 120912	154/146	1.068	1.029	0.850	1	Mascot

578	CD9 antigen [Rattus norvegicus]	gi|148229054	27819.3	2	93	1.804	0.922	1.012	0.576	0.168	0.422	3	3	3	100

Peptide Information

Calc. Mass	Obsrv. Mass	± da	± ppm	Start Seq.	End Seq.	Sequence	Ion Score	C. I. %	Modification	Plate [#] Name	Gel Idx/Pos [4700 Sample Name]	iTRAQ Ratio 115/114*	iTRAQ Ratio 116/114*	iTRAQ Ratio 117/114*	Rank	Result Type
1244.6772	1244.6437	-0.0335	-27	118	124	ELQEFYK	32	98.481	(N-term)_iTRAQ[0], Lysine(K)_iTRAQ[7]	[7] F5 120912	198/190	1.337	0.728	0.745	1	Mascot
1244.6772	1244.6801	0.0029	2	118	124	ELQEFYK	40	99.748	(N-term)_iTRAQ[0], Lysine(K)_iTRAQ[7]	[3] F6 and F9	240/232	1.682	1.062	0.844	1	Mascot
2024.0712	2024.1544	0.0832	41	118	129	ELQEFYKDTYQK	53	99.988	(N-term)_iTRAQ[0], Lysine(K)_iTRAQ[7,12]	[4] F7 and F10+11	1165/1157	2.609	1.013	1.649	1	Mascot

579	ADP-ribosylation factor-like protein 8B [Homo sapiens]	gi|8922601	23726.5	2	93	1.796	1.295	1.778	1.079	0.822	0.776	2	2	2	100

Peptide Information

Calc. Mass	Obsrv. Mass	± da	± ppm	Start Seq.	End Seq.	Sequence	Ion Score	C. I. %	Modification	Plate [#] Name	Gel Idx/Pos [4700 Sample Name]	iTRAQ Ratio 115/114*	iTRAQ Ratio 116/114*	iTRAQ Ratio 117/114*	Rank	Result Type
2053.0999	2053.1304	0.0305	15	88	103	GVNAIVYMIDAADREK	52	99.984	(N-term)_iTRAQ[0], Lysine(K)_iTRAQ[16]	[4] F7 and F10+11	466/458	2.875	2.117	2.554	1	Mascot
2513.4099	2513.4778	0.0679	27	166	182	EKDNIDITLQWLIQHSK	41	99.805	(N-term)_iTRAQ[0], Lysine(K)_iTRAQ[2,17]	[4] F7 and F10+11	1333/1325	1.122	0.792	1.238	1	Mascot

580	RecName: Full=Epsin-2; AltName: Full=EPS-15-interacting protein 2	gi|41016936	67203.7	2	92	1.153	0.992	0.826	0.160	0.148	0.153	2	2	2	100

Peptide Information

Calc. Mass	Obsrv. Mass	± da	± ppm	Start Seq.	End Seq.	Sequence	Ion Score	C. I. %	Modification	Plate [#] Name	Gel Idx/Pos [4700 Sample Name]	iTRAQ Ratio 115/114*	iTRAQ Ratio 116/114*	iTRAQ Ratio 117/114*	Rank	Result Type
1450.9131	1450.8621	-0.051	-35	77	86	ALTLLDYLIK	60	99.997	(N-term)_iTRAQ[0], Lysine(K)_iTRAQ[10]	[5] F4	405/397	1.013	1.140	0.980	1	Mascot
1813.9655	1813.8247	-0.1408	-78	414	428	KPAESGASVPPQDSR	32	98.428	(N-term)_iTRAQ[0], Lysine(K)_iTRAQ[1]	[6] F8 110912	83/75	1.314	0.863	0.697	1	Mascot

581	regulator of G-protein signaling 7 [Rattus norvegicus]	gi|9507047	61217.8	2	92	1.084	0.769	0.784	0.346	0.598	0.040	2	2	2	100

Peptide Information

Calc. Mass	Obsrv. Mass	± da	± ppm	Start Seq.	End Seq.	Sequence	Ion Score	C. I. %	Modification	Plate [#] Name	Gel Idx/Pos [4700 Sample Name]	iTRAQ Ratio 115/114*	iTRAQ Ratio 116/114*	iTRAQ Ratio 117/114*	Rank	Result Type
1966.0103	1966.0116	0.0013	1	330	344	WGFGMDEALKDPVGR	56	99.994	(N-term)_iTRAQ[0], Lysine(K)_iTRAQ[10]	[4] F7 and F10+11	363/355	1.430	1.367	0.824	1	Mascot
2077.1943	2077.1538	-0.0405	-19	53	68	IPSVFSGSDIVQWLIK	35	99.253	(N-term)_iTRAQ[0], Lysine(K)_iTRAQ[16]	[1] F3 030912	549/541	0.822	0.433	0.746	1	Mascot

582	fatty acid-binding protein, epidermal [Rattus norvegicus]	gi|22024394	17642.1	2	92	1.081	1.015	0.799	0.127	0.203	0.041	2	2	2	100

Protein Group

DA11 [Rattus sp.]	gi|1836058	17426.9
RecName: Full=Fatty acid-binding protein, epidermal; AltName: Full=Cutaneous fatty acid-binding pro	gi|1706754	17585.1

Peptide Information

Calc. Mass	Obsrv. Mass	± da	± ppm	Start Seq.	End Seq.	Sequence	Ion Score	C. I. %	Modification	Plate [#] Name	Gel Idx/Pos [4700 Sample Name]	iTRAQ Ratio 115/114*	iTRAQ Ratio 116/114*	iTRAQ Ratio 117/114*	Rank	Result Type
1742.8669	1742.7595	-0.1074	-62	13	24	LVESHGFEDYMK	33	98.845	(N-term)_iTRAQ[0], Lysine(K)_iTRAQ[12]	[6] F8 110912	282/274	0.967	0.846	0.840	1	Mascot
2484.2942	2484.3831	0.0889	36	82	99	KTETVCTFTDGALVQHQK	58	99.996	(N-term)_iTRAQ[0], Lysine(K)_iTRAQ[1,18], MMTS (C)[6]	[4] F7 and F10+11	1193/1185	1.208	1.218	0.760	1	Mascot

583	tubulin beta chain - rat (fragments)	gi|2144007	4826.3	1	92	1.013	0.961	1.005	0.202	0.720	0.228	10	10	10	100

Peptide Information

Calc. Mass	Obsrv. Mass	± da	± ppm	Start Seq.	End Seq.	Sequence	Ion Score	C. I. %	Modification	Plate [#] Name	Gel Idx/Pos [4700 Sample Name]	iTRAQ Ratio 115/114*	iTRAQ Ratio 116/114*	iTRAQ Ratio 117/114*	Rank	Result Type
1759.9384	1759.9335	-0.0049	-3	24	38	AILVDLEPGTMDXVR	75	100	(N-term)_iTRAQ[0]	[8] F13-15 and F1+2	408/400	1.012	1.229	0.951	1	Mascot
1759.9384	1759.9364	-0.002	-1	24	38	AILVDLEPGTMDXVR	64	99.999	(N-term)_iTRAQ[0]	[8] F13-15 and F1+2	420/412	1.115	1.414	1.398	1	Mascot
1759.9384	1759.9373	-0.0011	-1	24	38	AILVDLEPGTMDXVR	52	99.984	(N-term)_iTRAQ[0]	[8] F13-15 and F1+2	461/453	1.241	1.771	1.343	1	Mascot
1759.9384	1759.9391	0.0007	0	24	38	AILVDLEPGTMDXVR	73	100	(N-term)_iTRAQ[0]	[8] F13-15 and F1+2	466/458	1.176	0.994	0.979	1	Mascot
1759.9384	1759.9392	0.0008	0	24	38	AILVDLEPGTMDXVR	30	97.57	(N-term)_iTRAQ[0]	[8] F13-15 and F1+2	516/508	1.051	1.080	0.935	1	Mascot
1759.9384	1759.946	0.0076	4	24	38	AILVDLEPGTMDXVR	93	100	(N-term)_iTRAQ[0]	[8] F13-15 and F1+2	412/404	1.164	1.244	1.086	1	Mascot
1759.9384	1759.9512	0.0128	7	24	38	AILVDLEPGTMDXVR	33	98.686	(N-term)_iTRAQ[0]	[8] F13-15 and F1+2	512/504	1.092	1.100	1.180	1	Mascot
1759.9384	1759.9528	0.0144	8	24	38	AILVDLEPGTMDXVR	68	100	(N-term)_iTRAQ[0]	[8] F13-15 and F1+2	404/396	0.658	0.704	0.767	1	Mascot
1759.9384	1759.954	0.0156	9	24	38	AILVDLEPGTMDXVR	66	99.999	(N-term)_iTRAQ[0]	[8] F13-15 and F1+2	417/409	0.952	1.015	0.884	1	Mascot
1759.9384	1759.9597	0.0213	12	24	38	AILVDLEPGTMDXVR	28	95.748	(N-term)_iTRAQ[0]	[8] F13-15 and F1+2	523/515	0.828	0.208	0.738	1	Mascot

584	serine-threonine kinase receptor-associated protein [Rattus norvegicus]	gi|58865512	42552.7	2	90	0.992	1.029	0.970	0.335	0.729	0.164	2	2	2	100

Peptide Information

Calc. Mass	Obsrv. Mass	± da	± ppm	Start Seq.	End Seq.	Sequence	Ion Score	C. I. %	Modification	Plate [#] Name	Gel Idx/Pos [4700 Sample Name]	iTRAQ Ratio 115/114*	iTRAQ Ratio 116/114*	iTRAQ Ratio 117/114*	Rank	Result Type
1849.0442	1849.0989	0.0547	30	126	137	IYDLNKPEAEPK	37	99.506	(N-term)_iTRAQ[0], Lysine(K)_iTRAQ[6,12]	[3] F6 and F9	1166/1158	1.327	1.758	1.134	1	Mascot
1884.8749	1884.7861	-0.0888	-47	250	262	YDYNSGEELESYK	54	99.99	(N-term)_iTRAQ[0], Lysine(K)_iTRAQ[13]	[5] F4	160/152	0.741	0.602	0.829	1	Mascot

585	abl interactor 2 [Rattus norvegicus]	gi|27465617	64110	1	89	0.930	1.011	0.961	0.000	0.000	0.000	1	1	1	100

Peptide Information

Calc. Mass	Obsrv. Mass	± da	± ppm	Start Seq.	End Seq.	Sequence	Ion Score	C. I. %	Modification	Plate [#] Name	Gel Idx/Pos [4700 Sample Name]	iTRAQ Ratio 115/114*	iTRAQ Ratio 116/114*	iTRAQ Ratio 117/114*	Rank	Result Type
2216.2661	2216.3264	0.0603	27	69	84	KPIDYTILDDIGHGVK	89	100	(N-term)_iTRAQ[0], Lysine(K)_iTRAQ[1,16]	[4] F7 and F10+11	1252/1244	0.930	1.011	0.961	1	Mascot

586	Hiob2 protein [Rattus norvegicus]	gi|27527762	24862.2	2	88	1.460	1.679	1.028	0.266	0.612	0.235	2	2	2	100

Protein Group

L-asparaginase [Rattus norvegicus]	gi|21489983	38791.3

Peptide Information

Calc. Mass	Obsrv. Mass	± da	± ppm	Start Seq.	End Seq.	Sequence	Ion Score	C. I. %	Modification	Plate [#] Name	Gel Idx/Pos [4700 Sample Name]	iTRAQ Ratio 115/114*	iTRAQ Ratio 116/114*	iTRAQ Ratio 117/114*	Rank	Result Type
1429.8413	1429.8678	0.0265	19	125	134	LALFHVEQGK	54	99.989	(N-term)_iTRAQ[0], Lysine(K)_iTRAQ[10]	[3] F6 and F9	1244/1236	1.235	1.231	0.837	1	Mascot
1622.8611	1622.798	-0.0631	-39	170	181	WTSASMPWAAVK	35	99.294	(N-term)_iTRAQ[0], Lysine(K)_iTRAQ[12]	[5] F4	269/261	1.726	2.291	1.264	1	Mascot

587	Dync1li2 protein [Rattus norvegicus]	gi|60422784	60074.6	2	88	1.129	1.197	0.880	0.200	0.318	0.227	2	2	2	100

Protein Group

cytoplasmic dynein 1 light intermediate chain 2 [Rattus norvegicus]	gi|13591938	60652.5

Peptide Information

Calc. Mass	Obsrv. Mass	± da	± ppm	Start Seq.	End Seq.	Sequence	Ion Score	C. I. %	Modification	Plate [#] Name	Gel Idx/Pos [4700 Sample Name]	iTRAQ Ratio 115/114*	iTRAQ Ratio 116/114*	iTRAQ Ratio 117/114*	Rank	Result Type
1686.9272	1686.8112	-0.116	-69	398	413	GGPASVPSASPGTSVK	36	99.394	(N-term)_iTRAQ[0], Lysine(K)_iTRAQ[16]	[5] F4	95/87	0.959	0.945	0.700	1	Mascot
1909.9531	1909.8884	-0.0647	-34	104	116	CNVWILDGDLYHK	52	99.985	(N-term)_iTRAQ[0], Lysine(K)_iTRAQ[13], MMTS (C)[1]	[6] F8 110912	437/429	1.330	1.515	1.107	1	Mascot

588	RecName: Full=Annexin A3; AltName: Full=35-alpha calcimedin; AltName: Full=Annexin III; AltName: Fu	gi|122065130	40415.3	2	88	1.039	1.098	1.250	0.113	0.127	0.250	2	2	2	100

Protein Group

annexin A3 [Rattus norvegicus]	gi|6978503	40374.3

Peptide Information

Calc. Mass	Obsrv. Mass	± da	± ppm	Start Seq.	End Seq.	Sequence	Ion Score	C. I. %	Modification	Plate [#] Name	Gel Idx/Pos [4700 Sample Name]	iTRAQ Ratio 115/114*	iTRAQ Ratio 116/114*	iTRAQ Ratio 117/114*	Rank	Result Type
1087.5544	1087.5396	-0.0148	-14	212	218	LTFDEYR	32	98.402	(N-term)_iTRAQ[0]	[7] F5 120912	206/198	0.937	1.225	1.500	1	Mascot
1624.8468	1624.7551	-0.0917	-56	128	138	EISQAYYTAYK	56	99.994	(N-term)_iTRAQ[0], Lysine(K)_iTRAQ[11]	[5] F4	155/147	1.152	0.984	1.041	1	Mascot

589	SH3 and multiple ankyrin repeat domains protein 3 [Rattus norvegicus]	gi|11067399	197972.2	2	88	0.729	1.106	0.769	0.027	0.595	0.284	2	2	2	100

Protein Group

Proline rich synapse associated protein 2 [Rattus norvegicus]	gi|5262748	203727.1
RecName: Full=SH3 and multiple ankyrin repeat domains protein 3; Short=Shank3; AltName: Full=Prolin	gi|22001983	204643.5

Peptide Information

Calc. Mass	Obsrv. Mass	± da	± ppm	Start Seq.	End Seq.	Sequence	Ion Score	C. I. %	Modification	Plate [#] Name	Gel Idx/Pos [4700 Sample Name]	iTRAQ Ratio 115/114*	iTRAQ Ratio 116/114*	iTRAQ Ratio 117/114*	Rank	Result Type
1243.6919	1243.6295	-0.0624	-50	1156	1166	GPLASPAFSPR	27	95.039	(N-term)_iTRAQ[0]	[1] F3 030912	227/219	0.756	0.719	0.562	1	Mascot
2054.0215	2054.0906	0.0691	34	1680	1695	FDVGDWLESIHLGEHR	61	99.998	(N-term)_iTRAQ[0]	[4] F7 and F10+11	1329/1321	0.703	1.701	1.053	1	Mascot

590	ornithine aminotransferase, mitochondrial precursor [Rattus norvegicus]	gi|11968102	52514.7	2	87	1.203	0.821	1.358	0.160	0.259	0.064	2	2	2	100

Peptide Information

Calc. Mass	Obsrv. Mass	± da	± ppm	Start Seq.	End Seq.	Sequence	Ion Score	C. I. %	Modification	Plate [#] Name	Gel Idx/Pos [4700 Sample Name]	iTRAQ Ratio 115/114*	iTRAQ Ratio 116/114*	iTRAQ Ratio 117/114*	Rank	Result Type
1955.0835	1955.0216	-0.0619	-32	256	271	HQVLFIADEIQTGLAR	28	96.288	(N-term)_iTRAQ[0]	[3] F6 and F9	417/409	1.363	1.080	1.297	1	Mascot
2056.0596	2055.9377	-0.1219	-59	32	46	KTEQGPPSSEYIFER	59	99.997	(N-term)_iTRAQ[0], Lysine(K)_iTRAQ[1]	[6] F8 110912	239/231	1.061	0.624	1.421	1	Mascot

591	RecName: Full=Protein disulfide-isomerase; Short=PDI; AltName: Full=Cellular thyroid hormone-bindin	gi|129731	64730.9	2	87	2.116	1.585	1.354	0.655	0.278	0.040	2	2	2	100

Protein Group

iodothyronine 5' monodeiodinase [Rattus norvegicus]	gi|202549	61802.5

protein disulfide-isomerase [Rattus norvegicus] gi|6981324 64643.8

Peptide Information

Calc. Mass	Obsrv. Mass	± da	± ppm	Start Seq.	End Seq.	Sequence	Ion Score	C. I. %	Modification	Plate [#] Name	Gel Idx/Pos [4700 Sample Name]	iTRAQ Ratio 115/114*	iTRAQ Ratio 116/114*	iTRAQ Ratio 117/114*	Rank	Result Type
1490.8101	1490.7786	-0.0315	-21	123	132	EADDIVNWLK	35	99.247	(N-term)_iTRAQ[0], Lysine(K)_iTRAQ[10]	[7] F5 120912	353/345	2.771	1.863	1.315	1	Mascot
1841.9868	1842.0581	0.0713	39	329	340	YKPESDELTAEK	52	99.983	(N-term)_iTRAQ[0], Lysine(K)_iTRAQ[2,12]	[4] F7 and F10+11	1065/1057	1.616	1.348	1.394	1	Mascot

592 Histone H2a [Rattus norvegicus] gi|66730355 16197.3 2 87 0.897 0.949 1.199 0.066 0.243 0.101 2 2 2 100

Protein Group

RecName: Full=Histone H2A type 1-F gi|81884124 16184.4

histone H2A type 4 [Rattus norvegicus] gi|12025520 16004.2

Peptide Information

Calc. Mass	Obsrv. Mass	± da	± ppm	Start Seq.	End Seq.	Sequence	Ion Score	C. I. %	Modification	Plate [#] Name	Gel Idx/Pos [4700 Sample Name]	iTRAQ Ratio 115/114*	iTRAQ Ratio 116/114*	iTRAQ Ratio 117/114*	Rank	Result Type
1088.6337	1088.5828	-0.0509	-47	22	30	AGLQFPVGR	54	99.991	(N-term)_iTRAQ[0]	[5] F4	162/154	0.963	1.192	1.106	1	Mascot
2219.3735	2219.3823	0.0088	4	101	119	VTIAQGGVLPNIQAVLLPK	33	98.627	(N-term)_iTRAQ[0], Lysine(K)_iTRAQ[19]	[8] F13-15 and F1+2	344/336	0.836	0.756	1.300	1	Mascot

593 voltage-gated potassium channel subunit beta-2 [Rattus norvegicus] gi|8393646 44539.3 2 86 1.051 0.968 0.808 0.044 0.199 0.173 2 2 2 100

Protein Group

Chain A, Structure Of A Voltage-Dependent K+ Channel Beta Subunit gi|6435563 39400.7

Chain A, Structure Of The Cytoplasmic Beta Subunit-T1 Assembly Of Voltage-Dependent K Channels gi|9955221 40553.3

Peptide Information

Calc. Mass	Obsrv. Mass	± da	± ppm	Start Seq.	End Seq.	Sequence	Ion Score	C. I. %	Modification	Plate [#] Name	Gel Idx/Pos [4700 Sample Name]	iTRAQ Ratio 115/114*	iTRAQ Ratio 116/114*	iTRAQ Ratio 117/114*	Rank	Result Type
2028.1865	2028.2456	0.0591	29	223	235	EKVEVQLPELFHK	54	99.991	(N-term)_iTRAQ[0], Lysine(K)_iTRAQ[2,13]	[4] F7 and F10+11	1228/1220	1.095	0.803	0.665	1	Mascot
2181.146	2181.0957	-0.0503	-23	236	254	IGVGAMTWSPLACGIVSGK	32	98.556	(N-term)_iTRAQ[0], Lysine(K)_iTRAQ[19], MMTS (C)[13]	[1] F3 030912	505/497	1.009	1.167	0.981	1	Mascot

594 Mat2a protein [Rattus norvegicus] gi|38383124 43145.4 1 86 0.825 0.769 0.587 0.000 0.000 0.000 1 1 1 100

Protein Group

S-adenosylmethionine synthase isoform type-2 [Rattus norvegicus] gi|19705457 47855

Peptide Information

Calc. Mass	Obsrv. Mass	± da	± ppm	Start Seq.	End Seq.	Sequence	Ion Score	C. I. %	Modification	Plate [#] Name	Gel Idx/Pos [4700 Sample Name]	iTRAQ Ratio 115/114*	iTRAQ Ratio 116/114*	iTRAQ Ratio 117/114*	Rank	Result Type
1588.8567	1588.8789	0.0222	14	250	264	FVIGGPQGDAGLTGR	86	100	(N-term)_iTRAQ[0]	[8] F13-15 and F1+2	498/490	0.825	0.769	0.587	1	Mascot

595 Chain A, Three-Dimensional Structure Of Nadph-Cytochrome P450 Reductase: Prototype For Fmn-And Fad- gi|3318958 75204.1 2 86 1.184 0.827 0.726 0.116 0.229 0.050 2 2 2 100

Protein Group

Chain A, Cypor-Triple Mutant gi|15826748 75817.6

Chain A, Cypor-W677g gi|15826744 75783.5

Chain A, Cypor-W677x gi|15826746 75639.4

NADPH--cytochrome P450 reductase [Rattus norvegicus]	gi|13928780	82856.1
NADPH-cytochrome P-450 oxidoreductase [Rattus norvegicus]	gi|205660	82828

Peptide Information

Calc. Mass	Obsrv. Mass	± da	± ppm	Start Seq.	End Seq.	Sequence	Ion Score	C. I. %	Modification	Plate [#] Name	Gel Idx/Pos [4700 Sample Name]	iTRAQ Ratio 115/114*	iTRAQ Ratio 116/114*	iTRAQ Ratio 117/114*	Rank	Result Type
1287.8035	1287.8514	0.0479	37	540	547	VYVQHLLK	45	99.917	(N-term)_iTRAQ[0], Lysine(K)_iTRAQ[8]	[3] F6 and F9	1210/1202	1.300	1.056	0.776	1	Mascot
1485.7406	1485.8187	0.0781	53	114	123	TYEHFNAMGK	41	99.822	(N-term)_iTRAQ[0], Lysine(K)_iTRAQ[10]	[4] F7 and F10+11	1082/1074	1.079	0.647	0.679	1	Mascot

596	4-trimethylaminobutyraldehyde dehydrogenase [Rattus norvegicus]	gi|75905479	59074	2	86	3.117	2.081	1.821	1.915	2.334	0.738	2	2	2	100

Protein Group

RecName: Full=4-trimethylaminobutyraldehyde dehydrogenase; Short=TMABADH; AltName: Full=Aldehyde de	gi|78099320	58676.9

Peptide Information

Calc. Mass	Obsrv. Mass	± da	± ppm	Start Seq.	End Seq.	Sequence	Ion Score	C. I. %	Modification	Plate [#] Name	Gel Idx/Pos [4700 Sample Name]	iTRAQ Ratio 115/114*	iTRAQ Ratio 116/114*	iTRAQ Ratio 117/114*	Rank	Result Type
1531.8618	1531.8304	-0.0314	-20	472	481	VTIEYYSQLK	39	99.688	(N-term)_iTRAQ[0], Lysine(K)_iTRAQ[10]	[7] F5 120912	256/248	5.031	4.415	2.559	1	Mascot
1658.0587	1658.0892	0.0305	18	247	258	GIKPITLELGGK	48	99.964	(N-term)_iTRAQ[0], Lysine(K)_iTRAQ[3,12]	[3] F6 and F9	1249/1241	1.931	0.981	1.296	1	Mascot

597	190 kDa ankyrin isoform [Rattus norvegicus]	gi|3202046	208689.3	2	86	1.116	0.793	0.746	0.048	0.263	0.163	2	2	2	100

Protein Group

270 kDa ankyrin G isoform [Rattus norvegicus]	gi|3885972	312234.8
ankyrin G107 [Rattus norvegicus]	gi|18376635	116901

Peptide Information

Calc. Mass	Obsrv. Mass	± da	± ppm	Start Seq.	End Seq.	Sequence	Ion Score	C. I. %	Modification	Plate [#] Name	Gel Idx/Pos [4700 Sample Name]	iTRAQ Ratio 115/114*	iTRAQ Ratio 116/114*	iTRAQ Ratio 117/114*	Rank	Result Type
1736.8575	1736.7792	-0.0783	-45	1340	1352	TLEQQENFEEVAR	44	99.908	(N-term)_iTRAQ[0]	[1] F3 030912	221/213	1.070	1.056	0.909	1	Mascot
2055.2185	2055.2197	0.0012	1	1171	1185	VGLQAQPVPEETVKK	42	99.836	(N-term)_iTRAQ[0], Lysine(K)_iTRAQ[14,15]	[4] F7 and F10+11	207/199	1.164	0.596	0.612	1	Mascot

598	secretory carrier membrane protein 3 [Rattus norvegicus]	gi|10764631	25861.9	1	86	2.939	2.484	2.322	0.000	0.000	0.000	1	1	1	100

Peptide Information

Calc. Mass	Obsrv. Mass	± da	± ppm	Start Seq.	End Seq.	Sequence	Ion Score	C. I. %	Modification	Plate [#] Name	Gel Idx/Pos [4700 Sample Name]	iTRAQ Ratio 115/114*	iTRAQ Ratio 116/114*	iTRAQ Ratio 117/114*	Rank	Result Type
1835.9524	1835.885	-0.0674	-37	193	208	AQQEFAAGVFSNPAVR	86	100	(N-term)_iTRAQ[0]	[1] F3 030912	298/290	2.939	2.484	2.322	1	Mascot

599	synaptotagmin VIId [Rattus norvegicus]	gi|12667456	75099.4	2	86	0.690	0.683	0.898	0.186	0.060	0.200	2	2	2	100

Protein Group

synaptotagmin VIIL [Rattus norvegicus]	gi|12667448	83151.8
synaptotagmin VIIa [Rattus norvegicus]	gi|12667450	64166.4
synaptotagmin VIIb [Rattus norvegicus]	gi|12667452	65029.8
synaptotagmin VIIc [Rattus norvegicus]	gi|12667454	69615.7
synaptotagmin VIIe [Rattus norvegicus]	gi|12667458	77668.1

synaptotagmin-7 [Rattus norvegicus]	gi|11067375	51493.7

Peptide Information

Calc. Mass	Obsrv. Mass	± da	± ppm	Start Seq.	End Seq.	Sequence	Ion Score	C. I. %	Modification	Plate [#] Name	Gel Idx/Pos [4700 Sample Name]	iTRAQ Ratio 115/114*	iTRAQ Ratio 116/114*	iTRAQ Ratio 117/114*	Rank	Result Type
1522.874	1522.9211	0.0471	31	601	610	QPVAQWHQLK	42	99.854	(N-term)_iTRAQ[0], Lysine(K)_iTRAQ[10]	[3] F6 and F9	1170/1162	0.544	0.628	0.734	1	Mascot
1684.8978	1684.8999	0.0021	1	453	463	VDLTQMQTFWK	44	99.892	(N-term)_iTRAQ[0], Lysine(K)_iTRAQ[11]	[7] F5 120912	372/364	0.876	0.743	1.098	1	Mascot

600	RecName: Full=Caskin-1; AltName: Full=CASK-interacting protein 1	gi|61212441	162433	2	86	0.932	0.749	0.869	0.335	0.085	0.289	2	2	2	100

Peptide Information

Calc. Mass	Obsrv. Mass	± da	± ppm	Start Seq.	End Seq.	Sequence	Ion Score	C. I. %	Modification	Plate [#] Name	Gel Idx/Pos [4700 Sample Name]	iTRAQ Ratio 115/114*	iTRAQ Ratio 116/114*	iTRAQ Ratio 117/114*	Rank	Result Type
2030.1029	2030.0624	-0.0405	-20	786	804	AQPGSPQALGGPHGPATAK	42	99.846	(N-term)_iTRAQ[0], Lysine(K)_iTRAQ[19]	[3] F6 and F9	158/150	0.686	0.833	0.652	1	Mascot
2150.124	2150.0122	-0.1118	-52	446	463	SQPPAAHAGQVYGEQPPK	44	99.895	(N-term)_iTRAQ[0], Lysine(K)_iTRAQ[18]	[3] F6 and F9	154/146	1.267	0.673	1.158	1	Mascot

601	flotillin-1 [Rattus norvegicus]	gi|158636004	52022.6	2	85	0.757	0.831	1.009	0.097	0.144	0.003	2	2	2	100

Protein Group

RAREG-2.2 [Rattus norvegicus]	gi|4079647	43525.7
flotillin-1 [Mus musculus]	gi|6679809	52036.6

Peptide Information

Calc. Mass	Obsrv. Mass	± da	± ppm	Start Seq.	End Seq.	Sequence	Ion Score	C. I. %	Modification	Plate [#] Name	Gel Idx/Pos [4700 Sample Name]	iTRAQ Ratio 115/114*	iTRAQ Ratio 116/114*	iTRAQ Ratio 117/114*	Rank	Result Type
1613.8732	1613.781	-0.0922	-57	262	274	AQQVAVQEQEIAR	48	99.963	(N-term)_iTRAQ[0]	[1] F3 030912	175/167	0.855	0.976	1.007	1	Mascot
1746.8641	1746.8285	-0.0356	-20	79	91	EMLAAACQMFLGK	37	99.497	(N-term)_iTRAQ[0], Lysine(K)_iTRAQ[13], MMTS (C)[7]	[5] F4	421/413	0.671	0.708	1.012	1	Mascot

602	SNIP-a [Rattus norvegicus]	gi|6693834	136239.7	2	85	0.454	0.770	0.596	0.171	0.197	0.192	2	2	2	100

Protein Group

SRC kinase signaling inhibitor 1 [Rattus norvegicus]	gi|9507127	139019

Peptide Information

Calc. Mass	Obsrv. Mass	± da	± ppm	Start Seq.	End Seq.	Sequence	Ion Score	C. I. %	Modification	Plate [#] Name	Gel Idx/Pos [4700 Sample Name]	iTRAQ Ratio 115/114*	iTRAQ Ratio 116/114*	iTRAQ Ratio 117/114*	Rank	Result Type
1614.8163	1614.8096	-0.0067	-4	141	150	EQQPNYWSFK	44	99.905	(N-term)_iTRAQ[0], Lysine(K)_iTRAQ[10]	[3] F6 and F9	283/275	0.624	0.966	0.451	1	Mascot
1890.947	1890.9708	0.0238	13	427	442	GEGLYADPYGLLHEGR	41	99.796	(N-term)_iTRAQ[0]	[3] F6 and F9	330/322	0.330	0.613	0.788	1	Mascot

603	neutral amino acid transporter A [Rattus norvegicus]	gi|38454264	58832.6	2	85	1.064	1.135	0.458	0.218	0.202	0.252	2	2	2	100

Peptide Information

Calc. Mass	Obsrv. Mass	± da	± ppm	Start Seq.	End Seq.	Sequence	Ion Score	C. I. %	Modification	Plate [#] Name	Gel Idx/Pos [4700 Sample Name]	iTRAQ Ratio 115/114*	iTRAQ Ratio 116/114*	iTRAQ Ratio 117/114*	Rank	Result Type
1451.7866	1451.7395	-0.0471	-32	163	173	ETVDSFLDLLR	46	99.931	(N-term)_iTRAQ[0]	[1] F3 030912	489/481	1.282	0.963	0.296	1	Mascot
1619.934	1619.9828	0.0488	30	481	490	KGEQELQEVK	39	99.699	(N-term)_iTRAQ[0], Lysine(K)_iTRAQ[1,10]	[4] F7 and F10+11	1048/1040	0.884	1.337	0.710	1	Mascot

604	guanine nucleotide-binding protein subunit alpha-13 [Rattus norvegicus]	gi|61557003	48006.3	1	84	1.116	1.118	1.283	0.000	0.000	0.000	1	1	1	100

Protein Group

guanine nucleotide-binding protein subunit alpha-12 [Rattus norvegicus]	gi|13591953	48210.2

Peptide Information

Calc. Mass	Obsrv. Mass	± da	± ppm	Start Seq.	End Seq.	Sequence	Ion Score	C. I. %	Modification	Plate [#] Name	Gel Idx/Pos [4700 Sample Name]	iTRAQ Ratio 115/114*	iTRAQ Ratio 116/114*	iTRAQ Ratio 117/114*	Rank	Result Type
1345.8301	1345.7644	-0.0657	-49	51	61	ILLLGAGESGK	84	100	(N-term)_iTRAQ[0], Lysine(K)_iTRAQ[11]	[5] F4	219/211	1.116	1.118	1.283	1	Mascot

605	integrin-associated protein form 4 [Rattus norvegicus]	gi|2394318	38481.8	2	84	0.995	1.034	0.964	0.126	0.025	0.096	2	2	2	100

Peptide Information

Calc. Mass	Obsrv. Mass	± da	± ppm	Start Seq.	End Seq.	Sequence	Ion Score	C. I. %	Modification	Plate [#] Name	Gel Idx/Pos [4700 Sample Name]	iTRAQ Ratio 115/114*	iTRAQ Ratio 116/114*	iTRAQ Ratio 117/114*	Rank	Result Type
1162.7294	1162.7009	-0.0285	-25	83	90	ISVSDLLK	33	98.637	(N-term)_iTRAQ[0], Lysine(K)_iTRAQ[8]	[7] F5 120912	306/298	0.883	1.059	1.061	1	Mascot
1405.7937	1405.7339	-0.0598	-43	300	309	AVEEPLNAFK	51	99.982	(N-term)_iTRAQ[0], Lysine(K)_iTRAQ[10]	[7] F5 120912	225/217	1.121	1.009	0.877	1	Mascot

606	hypothetical protein LOC361118 precursor [Rattus norvegicus]	gi|62079015	29296.9	1	84	1.504	1.045	0.803	0.000	0.000	0.000	1	1	1	100

Peptide Information

Calc. Mass	Obsrv. Mass	± da	± ppm	Start Seq.	End Seq.	Sequence	Ion Score	C. I. %	Modification	Plate [#] Name	Gel Idx/Pos [4700 Sample Name]	iTRAQ Ratio 115/114*	iTRAQ Ratio 116/114*	iTRAQ Ratio 117/114*	Rank	Result Type
1472.9298	1472.8646	-0.0652	-44	207	217	VNLLSVLEAVK	84	100	(N-term)_iTRAQ[0], Lysine(K)_iTRAQ[11]	[5] F4	399/391	1.504	1.045	0.803	1	Mascot

607	LIM and SH3 domain protein 1 [Rattus norvegicus]	gi|14249130	33443.5	2	84	0.675	0.740	0.746	0.444	0.142	0.152	2	2	2	100

Peptide Information

Calc. Mass	Obsrv. Mass	± da	± ppm	Start Seq.	End Seq.	Sequence	Ion Score	C. I. %	Modification	Plate [#] Name	Gel Idx/Pos [4700 Sample Name]	iTRAQ Ratio 115/114*	iTRAQ Ratio 116/114*	iTRAQ Ratio 117/114*	Rank	Result Type
1211.6868	1211.6222	-0.0646	-53	190	199	EPAAPVSIQR	45	99.92	(N-term)_iTRAQ[0]	[1] F3 030912	171/163	0.407	0.621	0.620	1	Mascot
1615.7982	1615.7056	-0.0926	-57	131	144	MGPSGGEGIEPERR	40	99.744	(N-term)_iTRAQ[0]	[6] F8 110912	146/138	1.118	0.882	0.898	1	Mascot

608	Wars protein [Rattus norvegicus]	gi|38197552	47308.2	2	83	1.693	1.674	1.747	2.020	0.835	1.517	2	2	2	100

Peptide Information

Calc. Mass	Obsrv. Mass	± da	± ppm	Start Seq.	End Seq.	Sequence	Ion Score	C. I. %	Modification	Plate [#] Name	Gel Idx/Pos [4700 Sample Name]	iTRAQ Ratio 115/114*	iTRAQ Ratio 116/114*	iTRAQ Ratio 117/114*	Rank	Result Type
1565.772	1565.7228	-0.0492	-31	39	50	ASEDFVDPWTVR	29	96.775	(N-term)_iTRAQ[0]	[1] F3 030912	328/320	3.713	2.509	3.264	1	Mascot
2418.1936	2418.1089	-0.0847	-35	191	208	TFIFSDLEYMGQSPGFYK	55	99.991	(N-term)_iTRAQ[0], Lysine(K)_iTRAQ[18]	[1] F3 030912	494/486	0.772	1.116	0.935	1	Mascot

609	WW domain-binding protein 2 [Rattus norvegicus]	gi|20376818	30510.1	1	83	1.444	1.312	1.441	0.000	0.000	0.000	1	1	1	100

Peptide Information

Calc. Mass	Obsrv. Mass	± da	± ppm	Start Seq.	End Seq.	Sequence	Ion Score	C. I. %	Modification	Plate [#] Name	Gel Idx/Pos [4700 Sample Name]	iTRAQ Ratio 115/114*	iTRAQ Ratio 116/114*	iTRAQ Ratio 117/114*	Rank	Result Type
1657.8068	1657.803	-0.0038	-2	98	111	AEAGGGWEGSASYK	83	100	(N-term)_iTRAQ[0], Lysine(K)_iTRAQ[14]	[3] F6 and F9	188/180	1.444	1.312	1.441	1	Mascot

610	KH domain-containing, RNA-binding, signal transduction-associated protein 1 [Rattus norvegicus]	gi|18426824	52169.9	1	83	1.166	1.337	1.481	0.000	0.000	0.000	1	1	1	100

Peptide Information

Calc. Mass	Obsrv. Mass	± da	± ppm	Start Seq.	End Seq.	Sequence	Ion Score	C. I. %	Modification	Plate [#] Name	Gel Idx/Pos [4700 Sample Name]	iTRAQ Ratio 115/114*	iTRAQ Ratio 116/114*	iTRAQ Ratio 117/114*	Rank	Result Type
2185.1147	2185.0632	-0.0515	-24	139	152	KDDEENYLDLFSHK	83	100	(N-term)_iTRAQ[0], Lysine(K)_iTRAQ[1,14]	[2] F12 040912	342/334	1.166	1.337	1.481	1	Mascot

611	L-lactate dehydrogenase A-like 6B [Rattus norvegicus]	gi|34328538	47053.5	1	83	0.606	0.916	0.821	0.000	0.000	0.000	1	1	1	100

Peptide Information

Calc. Mass	Obsrv. Mass	± da	± ppm	Start Seq.	End Seq.	Sequence	Ion Score	C. I. %	Modification	Plate [#] Name	Gel Idx/Pos [4700 Sample Name]	iTRAQ Ratio 115/114*	iTRAQ Ratio 116/114*	iTRAQ Ratio 117/114*	Rank	Result Type
2232.3254	2232.2744	-0.051	-23	183	199	IIIVSNPVDILTYVAWK	83	100	(N-term)_iTRAQ[0], Lysine(K)_iTRAQ[17]	[1] F3 030912	570/562	0.606	0.916	0.821	1	Mascot

612	Ca2+ channel beta subunit 3b [rats, brain, Peptide Partial, 477 aa]	gi|633789	56908.9	2	82	1.177	0.773	0.956	1.317	0.895	0.471	2	2	2	100

Protein Group

voltage-dependent L-type calcium channel subunit beta-3 [Rattus norvegicus]	gi|6978583	58316.8

Peptide Information

Calc. Mass	Obsrv. Mass	± da	± ppm	Start Seq.	End Seq.	Sequence	Ion Score	C. I. %	Modification	Plate [#] Name	Gel Idx/Pos [4700 Sample Name]	iTRAQ Ratio 115/114*	iTRAQ Ratio 116/114*	iTRAQ Ratio 117/114*	Rank	Result Type
1588.8051	1588.7146	-0.0905	-57	43	54	EVESQAQQQLER	37	99.492	(N-term)_iTRAQ[0]	[1] F3 030912	164/156	2.494	1.668	1.427	1	Mascot
2407.1074	2407.1814	0.074	31	417	434	HLEEDYADAYQDLYQPHR	45	99.929	(N-term)_iTRAQ[0]	[4] F7 and F10+11	1196/1188	0.555	0.358	0.640	1	Mascot

613	casein kinase II subunit beta [Mus musculus]	gi|7106277	26642.9	2	82	0.946	0.832	0.774	0.293	0.106	0.188	2	2	2	100

Peptide Information

Calc. Mass	Obsrv. Mass	± da	± ppm	Start Seq.	End Seq.	Sequence	Ion Score	C. I. %	Modification	Plate [#] Name	Gel Idx/Pos [4700 Sample Name]	iTRAQ Ratio 115/114*	iTRAQ Ratio 116/114*	iTRAQ Ratio 117/114*	Rank	Result Type
1523.6512	1523.5573	-0.0939	-62	101	111	YQQGDFGYCPR	44	99.892	(N-term)_iTRAQ[0], MMTS (C)[9]	[5] F4	189/181	1.240	0.938	0.962	1	Mascot
1831.9575	1831.9436	-0.0139	-8	34	47	FNLTGLNEQVPHYR	38	99.637	(N-term)_iTRAQ[0]	[4] F7 and F10+11	323/315	0.723	0.737	0.623	1	Mascot

614	thioredoxin reductase 2, mitochondrial precursor [Rattus norvegicus]	gi|12018236	61512.9	2	82	0.959	1.014	1.073	0.073	0.139	0.268	2	2	2	100

Peptide Information

Calc. Mass	Obsrv. Mass	± da	± ppm	Start Seq.	End Seq.	Sequence	Ion Score	C. I. %	Modification	Plate [#] Name	Gel Idx/Pos [4700 Sample Name]	iTRAQ Ratio 115/114*	iTRAQ Ratio 116/114*	iTRAQ Ratio 117/114*	Rank	Result Type
1485.8093	1485.8265	0.0172	12	129	139	AMAEAVQNHVK	49	99.971	(N-term)_iTRAQ[0], Lysine(K)_iTRAQ[11]	[3] F6 and F9	1131/1123	1.032	0.892	1.341	1	Mascot
1827.96	1827.8484	-0.1116	-61	161	174	ASFVNEHTVHGVDK	32	98.519	(N-term)_iTRAQ[0], Lysine(K)_iTRAQ[14]	[2] F12 040912	193/185	0.892	1.153	0.858	1	Mascot

615	NBC-like protein [Rattus norvegicus]	gi|9437326	135199.7	2	81	1.309	1.078	0.943	0.091	0.071	0.060	2	2	2	100

Protein Group

electrogenic Na+ bicarbonate cotransporter [Rattus norvegicus]	gi|2897075	126887.9
electrogenic sodium bicarbonate cotransporter 1 [Rattus norvegicus]	gi|16758164	133529.5
sodium bicarbonate cotransporter [Rattus norvegicus]	gi|6644384	133579.6
sodium bicarbonate cotransporter [Rattus norvegicus]	gi|2598120	127074

Peptide Information

Calc. Mass	Obsrv. Mass	± da	± ppm	Start Seq.	End Seq.	Sequence	Ion Score	C. I. %	Modification	Plate [#] Name	Gel Idx/Pos [4700 Sample Name]	iTRAQ Ratio 115/114*	iTRAQ Ratio 116/114*	iTRAQ Ratio 117/114*	Rank	Result Type
1332.7185	1332.7701	0.0516	39	935	943	HQPDFIYLR	47	99.956	(N-term)_iTRAQ[0]	[3] F6 and F9	1237/1229	1.400	1.012	1.003	1	Mascot
1617.8033	1617.7487	-0.0546	-34	607	618	LADYYPINSDFR	34	98.932	(N-term)_iTRAQ[0]	[1] F3 030912	327/319	1.224	1.149	0.886	1	Mascot

616	myotrophin [Mus musculus]	gi|6679961	14863.9	1	81	0.876	1.029	0.826	0.000	0.000	0.000	1	1	1	100

Peptide Information

Calc. Mass	Obsrv. Mass	± da	± ppm	Start Seq.	End Seq.	Sequence	Ion Score	C. I. %	Modification	Plate [#] Name	Gel Idx/Pos [4700 Sample Name]	iTRAQ Ratio 115/114*	iTRAQ Ratio 116/114*	iTRAQ Ratio 117/114*	Rank	Result Type
2002.0702	2001.9924	-0.0778	-39	98	114	GPDGLTALEATDNQAIK	81	100	(N-term)_iTRAQ[0], Lysine(K)_iTRAQ[17]	[1] F3 030912	273/265	0.876	1.029	0.826	1	Mascot

617	metabotropic glutamate receptor 2 precursor [Rattus norvegicus]	gi|157787064	100596.3	2	81	0.594	0.989	1.025	0.068	0.097	0.024	2	2	2	100

Peptide Information

Calc. Mass	Obsrv. Mass	± da	± ppm	Start Seq.	End Seq.	Sequence	Ion Score	C. I. %	Modification	Plate [#] Name	Gel Idx/Pos [4700 Sample Name]	iTRAQ Ratio 115/114*	iTRAQ Ratio 116/114*	iTRAQ Ratio 117/114*	Rank	Result Type
1641.7214	1641.7178	-0.0036	-2	44	57	GGPAEECGPVNEHR	33	98.874	(N-term)_iTRAQ[0], MMTS (C)[7]	[4] F7 and F10+11	169/161	0.533	0.900	1.002	1	Mascot
1658.8622	1658.848	-0.0142	-9	95	107	DTHALEQALDFVR	48	99.958	(N-term)_iTRAQ[0]	[4] F7 and F10+11	386/378	0.662	1.086	1.049	1	Mascot

618	PREDICTED: rCG38845-like [Rattus norvegicus]	gi|27718097	18102.7	2	81	1.011	1.018	0.964	0.104	0.114	0.037	2	2	2	100

Peptide Information

Calc. Mass	Obsrv. Mass	± da	± ppm	Start Seq.	End Seq.	Sequence	Ion Score	C. I. %	Modification	Plate [#] Name	Gel Idx/Pos [4700 Sample Name]	iTRAQ Ratio 115/114*	iTRAQ Ratio 116/114*	iTRAQ Ratio 117/114*	Rank	Result Type
1606.8607	1606.793	-0.0677	-42	89	99	ENLEEEAIIMK	46	99.944	(N-term)_iTRAQ[0], Lysine(K)_iTRAQ[11]	[5] F4	209/201	1.115	0.915	1.001	1	Mascot
1656.9597	1656.9163	-0.0434	-26	116	128	WVPPLIGELYGLR	34	99.097	(N-term)_iTRAQ[0]	[1] F3 030912	540/532	0.917	1.132	0.928	1	Mascot

619	vacuolar protein sorting-associated protein 45 [Rattus norvegicus]	gi|25742604	71082.9	2	81	0.806	1.036	0.903	0.511	0.320	0.493	2	2	2	100

Peptide Information

Calc. Mass	Obsrv. Mass	± da	± ppm	Start Seq.	End Seq.	Sequence	Ion Score	C. I. %	Modification	Plate [#] Name	Gel Idx/Pos [4700 Sample Name]	iTRAQ Ratio 115/114*	iTRAQ Ratio 116/114*	iTRAQ Ratio 117/114*	Rank	Result Type
1530.8202	1530.7585	-0.0617	-40	313	322	AFVENYPQFK	37	99.52	(N-term)_iTRAQ[0], Lysine(K)_iTRAQ[10]	[7] F5 120912	244/236	0.493	0.792	0.584	1	Mascot
1688.9092	1688.9299	0.0207	12	542	555	SFLEEVLASGLHSR	44	99.893	(N-term)_iTRAQ[0]	[4] F7 and F10+11	481/473	1.317	1.355	1.396	1	Mascot

620	hemoglobin alpha-2 chain - rat (tentative sequence) (fragments)	gi|483109	15897.8	1	80	0.315	0.375	0.311	0.000	0.000	0.000	1	1	1	100

Peptide Information

Calc. Mass	Obsrv. Mass	± da	± ppm	Start Seq.	End Seq.	Sequence	Ion Score	C. I. %	Modification	Plate [#] Name	Gel Idx/Pos [4700 Sample Name]	iTRAQ Ratio 115/114*	iTRAQ Ratio 116/114*	iTRAQ Ratio 117/114*	Rank	Result Type
1716.8425	1716.7297	-0.1128	-66	17	31	IGGHGGEYGEEALXR	80	100	(N-term)_iTRAQ[0]	[6] F8 110912	188/180	0.315	0.375	0.311	1	Mascot

621	thymosin beta-4 [Mus musculus]	gi|10946578	6490.5	1	80	0.953	0.810	1.224	0.000	0.000	0.000	1	1	1	100

Protein Group

thymosin beta-4 peptide [Rattus norvegicus]	gi|207316	6518.6
thymosin beta-4 precursor [Rattus norvegicus]	gi|207318	7757.3

Peptide Information

Calc. Mass	Obsrv. Mass	± da	± ppm	Start Seq.	End Seq.	Sequence	Ion Score	C. I. %	Modification	Plate [#] Name	Gel Idx/Pos [4700 Sample Name]	iTRAQ Ratio 115/114*	iTRAQ Ratio 116/114*	iTRAQ Ratio 117/114*	Rank	Result Type
1945.0978	1945.0077	-0.0901	-46	27	39	NPLPSKETIEQEK	80	100	(N-term)_iTRAQ[0], Lysine(K)_iTRAQ[6,13]	[6] F8 110912	210/202	0.953	0.810	1.224	1	Mascot

622	soluble guanylyl cyclase beta 1 subunit [Rattus norvegicus]	gi|27552477	75882.8	2	80	0.820	0.695	0.822	0.219	0.120	0.156	2	2	2	100

Protein Group

guanylate cyclase 70kd subunit (EC 4.6.1.2) [Rattus norvegicus]	gi|204274	75975.9
guanylate cyclase soluble subunit beta-1 [Rattus norvegicus]	gi|52138593	75954.9
soluble guanylyl cyclase beta 1 subunit [Rattus norvegicus]	gi|27374983	75963.9
soluble guanylyl cyclase beta 1 subunit [Rattus norvegicus]	gi|28564567	75896.9

Peptide Information

Calc. Mass	Obsrv. Mass	± da	± ppm	Start Seq.	End Seq.	Sequence	Ion Score	C. I. %	Modification	Plate [#] Name	Gel Idx/Pos [4700 Sample Name]	iTRAQ Ratio 115/114*	iTRAQ Ratio 116/114*	iTRAQ Ratio 117/114*	Rank	Result Type
1406.7705	1406.7178	-0.0527	-37	216	225	AFPFHIIFDR	43	99.88	(N-term)_iTRAQ[0]	[6] F8 110912	439/431	0.647	0.593	0.691	1	Mascot
1910.0396	1910.1062	0.0666	35	16	27	NYGPEVWEDIKK	37	99.506	(N-term)_iTRAQ[0], Lysine(K)_iTRAQ[11,12]	[4] F7 and F10+11	1190/1182	1.039	0.815	0.978	1	Mascot

623	annexin VII [Rattus norvegicus]	gi|16930147	52294.7	1	80	0.916	1.047	1.400	0.000	0.000	0.000	1	1	1	100

Protein Group

annexin A7 [Rattus norvegicus] gi|148539909 52234.7

Peptide Information

Calc. Mass	Obsrv. Mass	± da	± ppm	Start Seq.	End Seq.	Sequence	Ion Score	C. I. %	Modification	Plate [#] Name	Gel Idx/Pos [4700 Sample Name]	iTRAQ Ratio 115/114*	iTRAQ Ratio 116/114*	iTRAQ Ratio 117/114*	Rank	Result Type
2099.9536	2099.8384	-0.1152	-55	307	323	DESPSINHQMAQEDAQR	80	100	(N-term)_iTRAQ[0]	[3] F6 and F9	141/133	0.916	1.047	1.400	1	Mascot

624	macrophage migration inhibitory factor [Rattus norvegicus]	gi|694108	13252.6	2	80	0.948	1.254	1.069	0.276	0.145	0.099	3	3	3	100		

Protein Group

macrophage migration inhibitory factor [Rattus norvegicus] gi|13591985 13183.5

Peptide Information

Calc. Mass	Obsrv. Mass	± da	± ppm	Start Seq.	End Seq.	Sequence	Ion Score	C. I. %	Modification	Plate [#] Name	Gel Idx/Pos [4700 Sample Name]	iTRAQ Ratio 115/114*	iTRAQ Ratio 116/114*	iTRAQ Ratio 117/114*	Rank	Result Type
1179.6331	1179.5746	-0.0585	-50	79	87	LLCGLLSDR	36	99.354	(N-term)_iTRAQ[0], MMTS (C)[3]	[5] F4	352/344	1.144	1.363	1.056	1	Mascot
1431.7903	1431.7443	-0.046	-32	2	12	PMFIVNTNVPR	44	99.903	(N-term)_iTRAQ[0]	[5] F4	211/203	0.660	1.075	0.966	1	Mascot
1431.7903	1431.782	-0.0083	-6	2	12	PMFIVNTNVPR	41	99.814	(N-term)_iTRAQ[0]	[7] F5 120912	259/251	1.127	1.347	1.198	1	Mascot

625	enoyl-CoA delta isomerase 2, mitochondrial [Rattus norvegicus]	gi|55741520	47881.2	2	80	1.010	0.912	1.086	0.159	0.144	0.065	3	3	3	100

Peptide Information

Calc. Mass	Obsrv. Mass	± da	± ppm	Start Seq.	End Seq.	Sequence	Ion Score	C. I. %	Modification	Plate [#] Name	Gel Idx/Pos [4700 Sample Name]	iTRAQ Ratio 115/114*	iTRAQ Ratio 116/114*	iTRAQ Ratio 117/114*	Rank	Result Type
1267.733	1267.7034	-0.0296	-23	293	301	AAEMLLFGK	31	98.252	(N-term)_iTRAQ[0], Lysine(K)_iTRAQ[9]	[7] F5 120912	330/322	1.163	0.947	1.003	1	Mascot
1911.948	1911.829	-0.119	-62	36	49	ATQQDFENAMNQVK	32	98.605	(N-term)_iTRAQ[0], Lysine(K)_iTRAQ[14]	[5] F4	185/177	1.074	0.750	1.109	1	Mascot
1911.948	1911.8689	-0.0791	-41	36	49	ATQQDFENAMNQVK	48	99.963	(N-term)_iTRAQ[0], Lysine(K)_iTRAQ[14]	[7] F5 120912	233/225	0.825	1.067	1.153	1	Mascot

626	glutathione S-transferase A6 [Rattus norvegicus]	gi|66730521	28817.3	2	80	1.142	1.067	1.181	0.019	0.140	0.217	2	2	2	100

Peptide Information

Calc. Mass	Obsrv. Mass	± da	± ppm	Start Seq.	End Seq.	Sequence	Ion Score	C. I. %	Modification	Plate [#] Name	Gel Idx/Pos [4700 Sample Name]	iTRAQ Ratio 115/114*	iTRAQ Ratio 116/114*	iTRAQ Ratio 117/114*	Rank	Result Type
1189.6503	1189.6371	-0.0132	-11	132	138	YFPAFEK	38	99.579	(N-term)_iTRAQ[0], Lysine(K)_iTRAQ[7]	[7] F5 120912	259/251	1.161	0.943	0.998	1	Mascot
1880.9752	1880.8701	-0.1051	-56	139	152	VFESHGQDYLVGNK	42	99.842	(N-term)_iTRAQ[0], Lysine(K)_iTRAQ[14]	[6] F8 110912	245/237	1.124	1.207	1.398	1	Mascot

627	solute carrier family 12 member 2 [Rattus norvegicus]	gi|13929130	141052.9	2	79	1.411	0.506	0.799	0.064	0.403	0.304	2	2	2	100

Peptide Information

Calc. Mass	Obsrv. Mass	± da	± ppm	Start Seq.	End Seq.	Sequence	Ion Score	C. I. %	Modification	Plate [#] Name	Gel Idx/Pos [4700 Sample Name]	iTRAQ Ratio 115/114*	iTRAQ Ratio 116/114*	iTRAQ Ratio 117/114*	Rank	Result Type
1462.7411	1462.6642	-0.0769	-53	77	88	FQVDPVSENAGR	44	99.91	(N-term)_iTRAQ[0]	[1] F3 030912	207/199	1.350	0.909	1.103	1	Mascot
1518.7825	1518.7726	-0.0099	-7	844	855	AFYAPVHADDLR	35	99.239	(N-term)_iTRAQ[0]	[4] F7 and F10+11	248/240	1.475	0.282	0.579	1	Mascot

628	E-septin long form [Rattus norvegicus]	gi|6010652	51068.7	2	79	0.944	0.864	1.053	0.258	0.233	0.496	3	3	3	100

Protein Group

RecName: Full=Septin-9; AltName: Full=Eighth septin; AltName: Full=Eseptin; AltName: Full=Septin-li gi|56749368 69936.1

septin-9 isoform 3 [Rattus norvegicus] gi|164698512 42977.8

septin-like protein SLP-b [Rattus norvegicus] gi|6090911 59818.6

Peptide Information

Calc. Mass	Obsrv. Mass	± da	± ppm	Start Seq.	End Seq.	Sequence	Ion Score	C. I. %	Modification	Plate [#] Name	Gel Idx/Pos [4700 Sample Name]	iTRAQ Ratio 115/114*	iTRAQ Ratio 116/114*	iTRAQ Ratio 117/114*	Rank	Result Type

1344.7046	1344.6533	-0.0513	-38	202	209	FINDQYEK	48	99.957	(N-term)_iTRAQ[0], Lysine(K)_iTRAQ[8]	[3] F6 and F9	201/193	0.998	1.129	1.550	1	Mascot
1344.7046	1344.6995	-0.0051	-4	202	209	FINDQYEK	28	95.968	(N-term)_iTRAQ[0], Lysine(K)_iTRAQ[8]	[7] F5 120912	163/155	1.229	0.903	1.211	1	Mascot
1609.8094	1609.7778	-0.0316	-20	370	381	DITSNIHFEAYR	31	98.232	(N-term)_iTRAQ[0]	[4] F7 and F10+11	267/259	0.686	0.633	0.622	1	Mascot

629	guanylyl cyclase alpha 1 subunit [Rattus norvegicus]	gi|25006379	86830	2	79	1.502	0.764	1.068	0.315	0.073	0.335	2	2	2	100

Protein Group

RecName: Full=Guanylate cyclase soluble subunit alpha-3; Short=GCS-alpha-3; AltName: Full=GCS-alpha	gi|118060	85065.1
guanylate cyclase soluble subunit alpha-3 [Rattus norvegicus]	gi|148747455	85037.1

Peptide Information

Calc. Mass	Obsrv. Mass	± da	± ppm	Start Seq.	End Seq.	Sequence	Ion Score	C. I. %	Modification	Plate [#] Name	Gel Idx/Pos [4700 Sample Name]	iTRAQ Ratio 115/114*	iTRAQ Ratio 116/114*	iTRAQ Ratio 117/114*	Rank	Result Type
1454.7375	1454.6763	-0.0612	-42	284	293	TFPFHFMLDR	46	99.937	(N-term)_iTRAQ[0]	[6] F8 110912	421/413	1.242	0.698	0.814	1	Mascot
1572.8883	1572.8241	-0.0642	-41	160	170	DFLNSFSTLLK	33	98.751	(N-term)_iTRAQ[0], Lysine(K)_iTRAQ[11]	[5] F4	363/355	1.818	0.836	1.403	1	Mascot

630	protein SET isoform 2 [Mus musculus]	gi|325910859	35111.8	1	78	0.830	0.951	1.073	0.000	0.000	0.000	1	1	1	100

Protein Group

PREDICTED: SET translocation-like [Rattus norvegicus]	gi|34855799	35194.3
RecName: Full=Protein SET; AltName: Full=Liver regeneration-related protein LRRGR00002; AltName: Fu	gi|46396568	36844.2
SET translocation [Rattus norvegicus]	gi|35215295	36326.1
protein SET [Rattus norvegicus]	gi|60499029	36866.1
protein kinase N3 [Rattus norvegicus]	gi|114145515	115853.1

Peptide Information

Calc. Mass	Obsrv. Mass	± da	± ppm	Start Seq.	End Seq.	Sequence	Ion Score	C. I. %	Modification	Plate [#] Name	Gel Idx/Pos [4700 Sample Name]	iTRAQ Ratio 115/114*	iTRAQ Ratio 116/114*	iTRAQ Ratio 117/114*	Rank	Result Type
1734.8545	1734.8848	0.0303	17	142	154	EFHLNESGDPSSK	78	100	(N-term)_iTRAQ[0], Lysine(K)_iTRAQ[13]	[3] F6 and F9	1136/1128	0.830	0.951	1.073	1	Mascot

631	RecName: Full=Leukotriene A-4 hydrolase; Short=LTA-4 hydrolase; AltName: Full=Leukotriene A(4) hydr	gi|266480	75113.9	1	78	0.858	1.162	0.983	0.000	0.000	0.000	1	1	1	100

Peptide Information

Calc. Mass	Obsrv. Mass	± da	± ppm	Start Seq.	End Seq.	Sequence	Ion Score	C. I. %	Modification	Plate [#] Name	Gel Idx/Pos [4700 Sample Name]	iTRAQ Ratio 115/114*	iTRAQ Ratio 116/114*	iTRAQ Ratio 117/114*	Rank	Result Type
2096.1287	2096.0811	-0.0476	-23	339	354	HFHALGGWGELQNTIK	78	100	(N-term)_iTRAQ[0], Lysine(K)_iTRAQ[16]	[2] F12 040912	364/356	0.858	1.162	0.983	1	Mascot

632	glutamate decarboxylase 2 [Rattus norvegicus]	gi|6978871	71669.4	2	78	2.481	2.107	1.522	0.660	0.889	0.119	2	2	2	100

Peptide Information

Calc. Mass	Obsrv. Mass	± da	± ppm	Start Seq.	End Seq.	Sequence	Ion Score	C. I. %	Modification	Plate [#] Name	Gel Idx/Pos [4700 Sample Name]	iTRAQ Ratio 115/114*	iTRAQ Ratio 116/114*	iTRAQ Ratio 117/114*	Rank	Result Type
1048.5886	1048.5776	-0.011	-10	455	460	LWLMWR	28	96.211	(N-term)_iTRAQ[0]	[3] F6 and F9	456/448	3.141	2.997	1.412	1	Mascot
1267.6519	1267.6543	0.0024	2	28	35	AWCQVAQK	50	99.974	(N-term)_iTRAQ[0], Lysine(K)_iTRAQ[8], MMTS (C)[3]	[3] F6 and F9	260/252	1.959	1.482	1.642	1	Mascot

633	thioredoxin-like protein 1 [Rattus norvegicus]	gi|18266686	35288.6	1	78	0.828	1.023	0.811	0.000	0.000	0.000	1	1	1	100

Peptide Information

Calc. Mass	Obsrv. Mass	± da	± ppm	Start Seq.	End Seq.	Sequence	Ion Score	C. I. %	Modification	Plate [#] Name	Gel Idx/Pos [4700 Sample Name]	iTRAQ Ratio 115/114*	iTRAQ Ratio 116/114*	iTRAQ Ratio 117/114*	Rank	Result Type

Calc. Mass	Obsrv. Mass	± da	± ppm	Start Seq.	End Seq.	Sequence	Ion Score	C. I. %	Modification	Plate [#] Name	Gel Idx/Pos [4700 Sample Name]	iTRAQ Ratio 115/114*	iTRAQ Ratio 116/114*	iTRAQ Ratio 117/114*	Rank	Result Type
1923.991	1923.896	-0.095	-49	88	102	IDQYQGADAVGLEEK	78	100	(N-term)_iTRAQ[0], Lysine(K)_iTRAQ[15]	[5] F4	157/149	0.828	1.023	0.811	1	Mascot

634	serotransferrin precursor [Rattus norvegicus]	gi|61556986	86018.7	2	78	2.426	1.561	1.949	0.327	0.656	0.711	2	2	2	100

Protein Group

Ab2-417 [Rattus norvegicus]	gi|33086606	119610.1
Ba1-667 [Rattus norvegicus]	gi|33086638	119646
TF [Rattus norvegicus]	gi|510196	85862.6
transferrin [Rattus norvegicus]	gi|1854476	86033.6

Peptide Information

Calc. Mass	Obsrv. Mass	± da	± ppm	Start Seq.	End Seq.	Sequence	Ion Score	C. I. %	Modification	Plate [#] Name	Gel Idx/Pos [4700 Sample Name]	iTRAQ Ratio 115/114*	iTRAQ Ratio 116/114*	iTRAQ Ratio 117/114*	Rank	Result Type
1929.8741	1929.7891	-0.085	-44	497	510	FDEFFSQGCAPGYK	33	98.728	(N-term)_iTRAQ[0], Lysine(K)_iTRAQ[14], MMTS (C)[9]	[5] F4	302/294	2.753	2.217	2.661	1	Mascot
2328.1931	2328.1624	-0.0307	-13	572	587	DLKQEDFQLLCPDGTK	45	99.92	(N-term)_iTRAQ[0], Lysine(K)_iTRAQ[3,16], MMTS (C)[11]	[4] F7 and F10+11	372/364	2.138	1.099	1.428	1	Mascot

635	PEX5-related protein [Rattus norvegicus]	gi|27465633	72536.4	2	78	1.433	1.015	1.187	0.102	0.271	0.346	2	2	2	100

Peptide Information

Calc. Mass	Obsrv. Mass	± da	± ppm	Start Seq.	End Seq.	Sequence	Ion Score	C. I. %	Modification	Plate [#] Name	Gel Idx/Pos [4700 Sample Name]	iTRAQ Ratio 115/114*	iTRAQ Ratio 116/114*	iTRAQ Ratio 117/114*	Rank	Result Type
1685.8268	1685.7535	-0.0733	-43	247	258	AAVESDTEFWDK	46	99.943	(N-term)_iTRAQ[0], Lysine(K)_iTRAQ[12]	[7] F5 120912	230/222	1.339	0.800	0.919	1	Mascot
1692.8955	1692.9482	0.0527	31	301	312	DWPGAFEEGLKR	33	98.63	(N-term)_iTRAQ[0], Lysine(K)_iTRAQ[11]	[3] F6 and F9	1258/1250	1.535	1.286	1.533	1	Mascot

636	NonO/p54nrb homolog [Rattus norvegicus]	gi|2674209	23246.5	2	77	0.803	0.872	1.015	0.385	0.015	0.187	2	2	2	100

Peptide Information

Calc. Mass	Obsrv. Mass	± da	± ppm	Start Seq.	End Seq.	Sequence	Ion Score	C. I. %	Modification	Plate [#] Name	Gel Idx/Pos [4700 Sample Name]	iTRAQ Ratio 115/114*	iTRAQ Ratio 116/114*	iTRAQ Ratio 117/114*	Rank	Result Type
1906.8844	1906.8639	-0.0205	-11	6	19	FAQHGTFEYEYSQR	39	99.708	(N-term)_iTRAQ[0]	[4] F7 and F10+11	233/225	1.188	0.887	1.202	1	Mascot
2716.3247	2716.2644	-0.0603	-22	43	62	DKLESEMEDAYHEHQANLLR	38	99.63	(N-term)_iTRAQ[0], Lysine(K)_iTRAQ[2]	[2] F12 040912	356/348	0.543	0.857	0.857	1	Mascot

637	PREDICTED: ribosomal protein L10a-like [Rattus norvegicus]	gi|27707472	29624.7	1	77	1.471	1.522	1.709	0.000	0.000	0.000	1	1	1	100

Protein Group

60S ribosomal protein L10a [Rattus norvegicus]	gi|13592009	29564.8
PREDICTED: ribosomal protein L10a-like [Rattus norvegicus]	gi|34865680	29743.9

Peptide Information

Calc. Mass	Obsrv. Mass	± da	± ppm	Start Seq.	End Seq.	Sequence	Ion Score	C. I. %	Modification	Plate [#] Name	Gel Idx/Pos [4700 Sample Name]	iTRAQ Ratio 115/114*	iTRAQ Ratio 116/114*	iTRAQ Ratio 117/114*	Rank	Result Type
1917.1068	1917.1718	0.065	34	106	118	KYDAFLASESLIK	77	100	(N-term)_iTRAQ[0], Lysine(K)_iTRAQ[1,13]	[4] F7 and F10+11	1237/1229	1.471	1.522	1.709	1	Mascot

638	Dync1i2 protein [Rattus norvegicus]	gi|50926121	76739.8	1	77	1.324	0.892	1.017	0.000	0.000	0.000	1	1	1	100

Protein Group

cytoplasmic dynein 1 intermediate chain 2 [Rattus norvegicus]	gi|16758748	77364.1
cytoplasmic dynein intermediate chain 2B [Rattus norvegicus]	gi|1151093	76578.8
cytoplasmic dynein intermediate chain 2C [Rattus norvegicus]	gi|1151095	74389.8

Peptide Information

Calc. Mass	Obsrv. Mass	± da	± ppm	Start Seq.	End Seq.	Sequence	Ion Score	C. I. %	Modification	Plate [#] Name	Gel Idx/Pos [4700 Sample Name]	iTRAQ Ratio 115/114*	iTRAQ Ratio 116/114*	iTRAQ Ratio 117/114*	Rank	Result Type
1935.0406	1934.9249	-0.1157	-60	245	258	DLEDKEGEIQAGAK	77	100	(N-term)_iTRAQ[0], Lysine(K)_iTRAQ[5,14]	[6] F8 110912	185/177	1.324	0.892	1.017	1	Mascot

639	ezrin [Rattus norvegicus]	gi|52138521	77509.3	2	77	1.393	1.234	1.279	0.132	0.218	0.149	2	2	2	100

Protein Group

EZRIN [Rattus norvegicus]	gi|17902245	61148.9
ezrin [Rattus norvegicus]	gi|40804381	77524.3
moesin [Rattus norvegicus]	gi|13540689	77201.4
radixin [Rattus norvegicus]	gi|56799432	78005.9
radixin [Rattus norvegicus]	gi|40804379	77889.8

Peptide Information

Calc. Mass	Obsrv. Mass	± da	± ppm	Start Seq.	End Seq.	Sequence	Ion Score	C. I. %	Modification	Plate [#] Name	Gel Idx/Pos [4700 Sample Name]	iTRAQ Ratio 115/114*	iTRAQ Ratio 116/114*	iTRAQ Ratio 117/114*	Rank	Result Type
1598.894	1598.9431	0.0491	31	263	273	KAPDFVFYAPR	46	99.937	(N-term)_iTRAQ[0], Lysine(K)_iTRAQ[1]	[3] F6 and F9	1258/1250	1.525	1.452	1.428	1	Mascot
1664.0972	1664.0306	-0.0666	-40	255	263	FVIKPIDKK	32	98.467	(N-term)_iTRAQ[0], Lysine(K)_iTRAQ[4,8,9]	[2] F12 040912	245/237	1.273	1.049	1.146	1	Mascot

640	RecName: Full=Sodium/potassium-transporting ATPase subunit beta-2; AltName: Full=Sodium/potassium-d	gi|114400	36594.5	2	77	1.272	1.078	0.939	0.193	0.180	0.055	2	2	2	100

Protein Group

Na+,K+ -ATPae beta2 subunit [Rattus norvegicus]	gi|220665	5232.7
sodium/potassium-transporting ATPase subunit beta-2 [Rattus norvegicus]	gi|56799390	36580.5

Peptide Information

Calc. Mass	Obsrv. Mass	± da	± ppm	Start Seq.	End Seq.	Sequence	Ion Score	C. I. %	Modification	Plate [#] Name	Gel Idx/Pos [4700 Sample Name]	iTRAQ Ratio 115/114*	iTRAQ Ratio 116/114*	iTRAQ Ratio 117/114*	Rank	Result Type
1091.5758	1091.5341	-0.0417	-38	19	25	EFVWNPR	29	96.92	(N-term)_iTRAQ[0]	[7] F5 120912	227/219	1.105	0.923	0.994	1	Mascot
1498.7261	1498.6832	-0.0429	-29	9	18	SCGQVVEEWK	48	99.958	(N-term)_iTRAQ[0], Lysine(K)_iTRAQ[10], MMTS (C)[2]	[7] F5 120912	284/276	1.465	1.258	0.887	1	Mascot

641	V-type proton ATPase subunit F [Rattus norvegicus]	gi|16758754	14370.7	2	77	0.926	0.927	0.879	0.151	0.013	0.251	2	2	2	100

Peptide Information

Calc. Mass	Obsrv. Mass	± da	± ppm	Start Seq.	End Seq.	Sequence	Ion Score	C. I. %	Modification	Plate [#] Name	Gel Idx/Pos [4700 Sample Name]	iTRAQ Ratio 115/114*	iTRAQ Ratio 116/114*	iTRAQ Ratio 117/114*	Rank	Result Type
1183.5902	1183.5128	-0.0774	-65	111	119	GMFTAEDLR	45	99.918	(N-term)_iTRAQ[0]	[5] F4	175/167	1.077	0.913	1.130	1	Mascot
2120.1545	2120.1123	-0.0422	-20	59	75	DDIGIILINQYIAEMVR	32	98.38	(N-term)_iTRAQ[0]	[1] F3 030912	585/577	0.797	0.940	0.684	1	Mascot

642	acyl-coenzyme A thioesterase 9, mitochondrial [Rattus norvegicus]	gi|62078649	55730.4	2	77	0.724	0.902	1.008	0.178	0.038	0.167	2	2	2	100

Peptide Information

Calc. Mass	Obsrv. Mass	± da	± ppm	Start Seq.	End Seq.	Sequence	Ion Score	C. I. %	Modification	Plate [#] Name	Gel Idx/Pos [4700 Sample Name]	iTRAQ Ratio 115/114*	iTRAQ Ratio 116/114*	iTRAQ Ratio 117/114*	Rank	Result Type
1467.7941	1467.7039	-0.0902	-61	156	165	YSLSPEQDIK	41	99.803	(N-term)_iTRAQ[0], Lysine(K)_iTRAQ[10]	[5] F4	150/142	0.903	0.940	1.175	1	Mascot
1832.1129	1832.0923	-0.0206	-11	64	75	KLLHNFLATSQK	36	99.317	(N-term)_iTRAQ[0], Lysine(K)_iTRAQ[1,12]	[2] F12 040912	301/293	0.581	0.865	0.865	1	Mascot

643	elongator complex protein 1 [Rattus norvegicus]	gi|18266706	162652.1	2	76	1.064	1.080	1.507	0.035	0.118	0.374	2	2	2	100

Peptide Information

Calc. Mass	Obsrv. Mass	± da	± ppm	Start Seq.	End Seq.	Sequence	Ion Score	C. I. %	Modification	Plate [#] Name	Gel Idx/Pos [4700 Sample Name]	iTRAQ Ratio 115/114*	iTRAQ Ratio 116/114*	iTRAQ Ratio 117/114*	Rank	Result Type
1598.0012	1597.9634	-0.0378	-24	1231	1240	LKDEVHAILK	36	99.446	(N-term)_iTRAQ[0], Lysine(K)_iTRAQ[2,10]	[2] F12 040912	302/294	1.030	1.198	1.208	1	Mascot
1715.9802	1715.9685	-0.0117	-7	749	760	INLNLIHDHNPK	40	99.749	(N-term)_iTRAQ[0], Lysine(K)_iTRAQ[12]	[2] F12 040912	279/271	1.098	0.974	1.881	1	Mascot

644	serum albumin precursor [Rattus norvegicus]	gi|158138568	78249.2	2	76	2.881	0.862	1.007	1.996	0.216	0.187	2	2	2	100

Protein Group

unnamed protein product [Rattus norvegicus]	gi|55628	78209.2

Peptide Information

Calc. Mass	Obsrv. Mass	± da	± ppm	Start Seq.	End Seq.	Sequence	Ion Score	C. I. %	Modification	Plate [#] Name	Gel Idx/Pos [4700 Sample Name]	iTRAQ Ratio 115/114*	iTRAQ Ratio 116/114*	iTRAQ Ratio 117/114*	Rank	Result Type
1437.8199	1437.7444	-0.0755	-53	66	75	LVQEVTDFAK	39	99.672	(N-term)_iTRAQ[0], Lysine(K)_iTRAQ[10]	[5] F4	247/239	1.702	0.690	0.849	1	Mascot
1680.9445	1681.0175	0.073	43	35	44	FKDLGEQHFK	37	99.531	(N-term)_iTRAQ[0], Lysine(K)_iTRAQ[2,10]	[8] F13-15 and F1+2	1191/1183	4.877	1.078	1.194	1	Mascot

645	prenylcysteine oxidase precursor [Rattus norvegicus]	gi|21489987	61190.9	1	75	0.704	1.148	1.012	0.000	0.000	0.000	1	1	1	100

Peptide Information

Calc. Mass	Obsrv. Mass	± da	± ppm	Start Seq.	End Seq.	Sequence	Ion Score	C. I. %	Modification	Plate [#] Name	Gel Idx/Pos [4700 Sample Name]	iTRAQ Ratio 115/114*	iTRAQ Ratio 116/114*	iTRAQ Ratio 117/114*	Rank	Result Type
1704.8699	1704.906	0.0361	21	152	162	MHMWVEDLLDK	75	100	(N-term)_iTRAQ[0], Lysine(K)_iTRAQ[11]	[3] F6 and F9	1370/1362	0.704	1.148	1.012	1	Mascot

646	NSFL1 cofactor p47 [Rattus norvegicus]	gi|14010837	43105.1	2	75	0.841	1.264	0.826	0.022	0.116	0.037	2	2	2	100

Peptide Information

Calc. Mass	Obsrv. Mass	± da	± ppm	Start Seq.	End Seq.	Sequence	Ion Score	C. I. %	Modification	Plate [#] Name	Gel Idx/Pos [4700 Sample Name]	iTRAQ Ratio 115/114*	iTRAQ Ratio 116/114*	iTRAQ Ratio 117/114*	Rank	Result Type
1466.7247	1466.6538	-0.0709	-48	11	22	EFVAVTGAEEDR	44	99.912	(N-term)_iTRAQ[0]	[1] F3 030912	211/203	0.819	1.380	0.790	1	Mascot
1898.9369	1898.8621	-0.0748	-39	200	214	SYQDPSNAQFLESIR	31	97.984	(N-term)_iTRAQ[0]	[1] F3 030912	299/291	0.863	1.157	0.862	1	Mascot

647	D-beta-hydroxybutyrate dehydrogenase, mitochondrial [Rattus norvegicus]	gi|55742813	42088.7	2	75	1.241	1.103	0.886	0.288	0.187	0.040	2	2	2	100

Protein Group

D-beta-hydroxybutyrate dehydrogenase [Rattus norvegicus]	gi|203921	41958.6

Peptide Information

Calc. Mass	Obsrv. Mass	± da	± ppm	Start Seq.	End Seq.	Sequence	Ion Score	C. I. %	Modification	Plate [#] Name	Gel Idx/Pos [4700 Sample Name]	iTRAQ Ratio 115/114*	iTRAQ Ratio 116/114*	iTRAQ Ratio 117/114*	Rank	Result Type
1361.7498	1361.802	0.0522	38	225	233	YEMHPLGVK	43	99.869	(N-term)_iTRAQ[0], Lysine(K)_iTRAQ[9]	[3] F6 and F9	1179/1171	1.007	0.942	0.926	1	Mascot
1773.8333	1773.8767	0.0434	24	316	326	YHPMDYYWWLR	32	98.481	(N-term)_iTRAQ[0]	[3] F6 and F9	1374/1366	1.529	1.290	0.848	1	Mascot

648	D-dopachrome decarboxylase [Rattus norvegicus]	gi|13162287	14081.4	2	75	0.685	0.925	0.634	0.660	0.091	0.126	2	2	2	100

Peptide Information

Calc. Mass	Obsrv. Mass	± da	± ppm	Start Seq.	End Seq.	Sequence	Ion Score	C. I. %	Modification	Plate [#] Name	Gel Idx/Pos [4700 Sample Name]	iTRAQ Ratio 115/114*	iTRAQ Ratio 116/114*	iTRAQ Ratio 117/114*	Rank	Result Type
1609.8193	1609.826	0.0067	4	84	95	FLTEELSLDQDR	34	99.044	(N-term)_iTRAQ[0]	[8] F13-15 and F1+2	483/475	0.349	0.842	0.529	1	Mascot
1649.9302	1649.8536	-0.0766	-46	100	110	FFPLEPWQIGK	41	99.791	(N-term)_iTRAQ[0], Lysine(K)_iTRAQ[11]	[5] F4	348/340	1.345	1.017	0.760	1	Mascot

649	proteasome subunit beta type-4 [Rattus norvegicus]	gi|13928866	26878.5	1	75	1.973	1.982	1.047	0.000	0.000	0.000	1	1	1	100

Protein Group

RecName: Full=Proteasome subunit beta type-4;	gi|3915806	30325.1

Short=Proteasome beta chain; AltName: Full=Macropain

Peptide Information

Calc. Mass	Obsrv. Mass	± da	± ppm	Start Seq.	End Seq.	Sequence	Ion Score	C. I. %	Modification	Plate [#] Name	Gel Idx/Pos [4700 Sample Name]	iTRAQ Ratio 115/114*	iTRAQ Ratio 116/114*	iTRAQ Ratio 117/114*	Rank	Result Type
2483.2009	2483.1301	-0.0708	-29	58	77	VNDSTMLGASGDYADFQ YLK	75	100	(N-term)_iTRAQ[0], Lysine(K)_iTRAQ[20]	[1] F3 030912	375/367	1.973	1.982	1.047	1	Mascot

650	up-regulated during skeletal muscle growth protein 5 [Rattus norvegicus]	gi|19424210	7458.1	1	74	0.787	1.311	0.691	0.120	0.069	0.042	2	2	2	100

Peptide Information

Calc. Mass	Obsrv. Mass	± da	± ppm	Start Seq.	End Seq.	Sequence	Ion Score	C. I. %	Modification	Plate [#] Name	Gel Idx/Pos [4700 Sample Name]	iTRAQ Ratio 115/114*	iTRAQ Ratio 116/114*	iTRAQ Ratio 117/114*	Rank	Result Type
1869.9592	1869.8528	-0.1064	-57	2	16	AGPESDGQFQFTGIK	74	100	(N-term)_iTRAQ[0], Lysine(K)_iTRAQ[15]	[5] F4	194/186	0.683	1.246	0.651	1	Mascot
1869.9592	1869.906	-0.0532	-28	2	16	AGPESDGQFQFTGIK	65	99.999	(N-term)_iTRAQ[0], Lysine(K)_iTRAQ[15]	[7] F5 120912	243/235	0.907	1.379	0.733	1	Mascot

651	scrapie-responsive protein 1 precursor [Rattus norvegicus]	gi|15721935	12721.2	1	74	1.285	1.242	1.203	0.000	0.000	0.000	1	1	1	100

Peptide Information

Calc. Mass	Obsrv. Mass	± da	± ppm	Start Seq.	End Seq.	Sequence	Ion Score	C. I. %	Modification	Plate [#] Name	Gel Idx/Pos [4700 Sample Name]	iTRAQ Ratio 115/114*	iTRAQ Ratio 116/114*	iTRAQ Ratio 117/114*	Rank	Result Type
2040.0548	2040.0099	-0.0449	-22	50	63	LIDENVQHHFWEGK	74	100	(N-term)_iTRAQ[0], Lysine(K)_iTRAQ[14]	[2] F12 040912	317/309	1.285	1.242	1.203	1	Mascot

652	CLIP-associating protein 2 [Rattus norvegicus]	gi|16758540	152526.5	2	74	1.800	1.284	0.922	0.294	0.069	0.106	2	2	2	100

Peptide Information

Calc. Mass	Obsrv. Mass	± da	± ppm	Start Seq.	End Seq.	Sequence	Ion Score	C. I. %	Modification	Plate [#] Name	Gel Idx/Pos [4700 Sample Name]	iTRAQ Ratio 115/114*	iTRAQ Ratio 116/114*	iTRAQ Ratio 117/114*	Rank	Result Type
1540.9508	1540.9946	0.0438	28	1070	1078	KIALYELMK	39	99.709	(N-term)_iTRAQ[0], Lysine(K)_iTRAQ[1,9]	[4] F7 and F10+11	1234/1226	1.547	1.354	1.028	1	Mascot
1944.9662	1944.9529	-0.0133	-7	103	115	EILSDDKHDWDQR	35	99.134	(N-term)_iTRAQ[0], Lysine(K)_iTRAQ[7]	[2] F12 040912	219/211	2.094	1.218	0.827	1	Mascot

653	ankyrin [Rattus norvegicus]	gi|1841966	97886.5	2	74	0.797	1.204	0.541	0.971	1.373	0.642	2	2	2	100

Peptide Information

Calc. Mass	Obsrv. Mass	± da	± ppm	Start Seq.	End Seq.	Sequence	Ion Score	C. I. %	Modification	Plate [#] Name	Gel Idx/Pos [4700 Sample Name]	iTRAQ Ratio 115/114*	iTRAQ Ratio 116/114*	iTRAQ Ratio 117/114*	Rank	Result Type
1550.8774	1550.8776	0.0002	0	39	51	EGHVGLVQELLGR	40	99.74	(N-term)_iTRAQ[0]	[4] F7 and F10+11	385/377	1.768	2.577	1.183	1	Mascot
2454.345	2454.3096	-0.0354	-14	505	526	EGQVDVASVLLEAGAAH SLATK	34	98.973	(N-term)_iTRAQ[0], Lysine(K)_iTRAQ[22]	[7] F5 120912	396/388	0.360	0.563	0.248	1	Mascot

654	Park7 protein [Rattus norvegicus]	gi|60551773	25496.2	1	73	1.304	1.187	0.983	0.000	0.000	0.000	1	1	1	100

Protein Group

protein DJ-1 [Rattus norvegicus]	gi|16924002	22451

Peptide Information

Calc. Mass	Obsrv. Mass	± da	± ppm	Start Seq.	End Seq.	Sequence	Ion Score	C. I. %	Modification	Plate [#] Name	Gel Idx/Pos [4700 Sample Name]	iTRAQ Ratio 115/114*	iTRAQ Ratio 116/114*	iTRAQ Ratio 117/114*	Rank	Result Type
1852.9263	1852.8523	-0.074	-40	49	62	DVVICPDTSLEEAK	73	100	(N-term)_iTRAQ[0], Lysine(K)_iTRAQ[14], MMTS (C)[5]	[1] F3 030912	313/305	1.304	1.187	0.983	1	Mascot

655	reticulocalbin-2 [Rattus norvegicus]	gi|8394171	39603.5	1	73	1.014	1.114	0.844	0.000	0.000	0.000	1	1	1	100

Peptide Information

Calc. Mass	Obsrv. Mass	± da	± ppm	Start Seq.	End Seq.	Sequence	Ion Score	C. I. %	Modification	Plate [#] Name	Gel Idx/Pos [4700 Sample Name]	iTRAQ Ratio 115/114*	iTRAQ Ratio 116/114*	iTRAQ Ratio 117/114*	Rank	Result Type
1995.0532	1994.9756	-0.0776	-39	43	57	EALLGVQEDVDEYVK	73	100	(N-term)_iTRAQ[0], Lysine(K)_iTRAQ[15]	[1] F3 030912	367/359	1.014	1.114	0.844	1	Mascot

656	platelet-activating factor acetylhydrolase IB subunit beta [Rattus norvegicus]	gi|11693154	27864.7	1	72	0.594	0.730	0.712	0.000	0.000	0.000	1	1	1	100

Peptide Information

Calc. Mass	Obsrv. Mass	± da	± ppm	Start Seq.	End Seq.	Sequence	Ion Score	C. I. %	Modification	Plate [#] Name	Gel Idx/Pos [4700 Sample Name]	iTRAQ Ratio 115/114*	iTRAQ Ratio 116/114*	iTRAQ Ratio 117/114*	Rank	Result Type
2189.1475	2189.1113	-0.0362	-17	61	79	ELFSPLHALNFGIGGDTTR	72	100	(N-term)_iTRAQ[0]	[7] F5 120912	425/417	0.594	0.730	0.712	1	Mascot

657	hypothetical protein LOC292267 [Rattus norvegicus]	gi|62945250	55274.7	1	72	1.239	1.447	1.521	0.000	0.000	0.000	1	1	1	100

Peptide Information

Calc. Mass	Obsrv. Mass	± da	± ppm	Start Seq.	End Seq.	Sequence	Ion Score	C. I. %	Modification	Plate [#] Name	Gel Idx/Pos [4700 Sample Name]	iTRAQ Ratio 115/114*	iTRAQ Ratio 116/114*	iTRAQ Ratio 117/114*	Rank	Result Type
1621.8295	1621.8673	0.0378	23	294	303	DFEWIVEHMK	72	100	(N-term)_iTRAQ[0], Lysine(K)_iTRAQ[10]	[3] F6 and F9	1376/1368	1.239	1.447	1.521	1	Mascot

658	Nipsnap1 protein [Rattus norvegicus]	gi|59808764	36450.1	2	72	1.023	1.040	0.953	0.585	0.156	0.065	2	2	2	100

Peptide Information

Calc. Mass	Obsrv. Mass	± da	± ppm	Start Seq.	End Seq.	Sequence	Ion Score	C. I. %	Modification	Plate [#] Name	Gel Idx/Pos [4700 Sample Name]	iTRAQ Ratio 115/114*	iTRAQ Ratio 116/114*	iTRAQ Ratio 117/114*	Rank	Result Type
1854.951	1854.9008	-0.0502	-27	254	267	GWDENVYYTVPLVR	39	99.701	(N-term)_iTRAQ[0]	[1] F3 030912	380/372	1.608	1.196	0.893	1	Mascot
2061.095	2061.0137	-0.0813	-39	191	205	LKPGTMIEWGNNWAR	33	98.695	(N-term)_iTRAQ[0], Lysine(K)_iTRAQ[2]	[6] F8 110912	376/368	0.651	0.904	1.018	1	Mascot

659	nucleolin-related protein NRP [Rattus norvegicus]	gi|5929884	90407.9	1	71	0.940	0.960	1.071	0.000	0.000	0.000	1	1	1	100

Protein Group

RecName: Full=Nucleolin; AltName: Full=Protein C23	gi|128844	89972.1
nucleolin [Rattus norvegicus]	gi|161760644	90101.1

Peptide Information

Calc. Mass	Obsrv. Mass	± da	± ppm	Start Seq.	End Seq.	Sequence	Ion Score	C. I. %	Modification	Plate [#] Name	Gel Idx/Pos [4700 Sample Name]	iTRAQ Ratio 115/114*	iTRAQ Ratio 116/114*	iTRAQ Ratio 117/114*	Rank	Result Type
1849.8854	1849.7855	-0.0999	-54	616	629	GFGFVDFNSEEDAK	71	100	(N-term)_iTRAQ[0], Lysine(K)_iTRAQ[14]	[5] F4	242/234	0.940	0.960	1.071	1	Mascot

660	COP9 signalosome complex subunit 3 [Rattus norvegicus]	gi|51948372	52466.8	1	71	0.895	0.906	1.024	0.000	0.000	0.000	1	1	1	100

Peptide Information

Calc. Mass	Obsrv. Mass	± da	± ppm	Start Seq.	End Seq.	Sequence	Ion Score	C. I. %	Modification	Plate [#] Name	Gel Idx/Pos [4700 Sample Name]	iTRAQ Ratio 115/114*	iTRAQ Ratio 116/114*	iTRAQ Ratio 117/114*	Rank	Result Type
1473.8159	1473.759	-0.0569	-39	326	336	VQLSGPQEAEK	71	100	(N-term)_iTRAQ[0], Lysine(K)_iTRAQ[11]	[7] F5 120912	138/130	0.895	0.906	1.024	1	Mascot

661	dnaJ homolog subfamily A member 1 [Mus musculus]	gi|6680297	50769.1	2	71	0.640	1.166	0.872	0.325	0.342	0.523	2	2	2	100

Peptide Information

Calc. Mass	Obsrv. Mass	± da	± ppm	Start Seq.	End Seq.	Sequence	Ion Score	C. I. %	Modification	Plate [#] Name	Gel Idx/Pos [4700 Sample Name]	iTRAQ Ratio 115/114*	iTRAQ Ratio 116/114*	iTRAQ Ratio 117/114*	Rank	Result Type
1681.0259	1680.9744	-0.0515	-31	284	296	TIVITSHPGQIVK	32	98.431	(N-term)_iTRAQ[0], Lysine(K)_iTRAQ[13]	[3] F6 and F9	230/222	0.425	0.902	0.545	1	Mascot
1859.9623	1860.038	0.0757	41	33	44	YHPDKNPNEGEK	39	99.716	(N-term)_iTRAQ[0], Lysine(K)_iTRAQ[5,12]	[8] F13-15 and F1+2	1302/1294	0.965	1.508	1.395	1	Mascot

662	40S ribosomal protein S10 [Mus musculus]	gi|13399310	20921.2	2	71	1.634	1.382	1.310	0.469	0.254	0.073	2	2	2	100

Peptide Information

Calc. Mass	Obsrv. Mass	± da	± ppm	Start Seq.	End Seq.	Sequence	Ion Score	C. I. %	Modification	Plate [#] Name	Gel Idx/Pos [4700 Sample Name]	iTRAQ Ratio 115/114*	iTRAQ Ratio 116/114*	iTRAQ Ratio 117/114*	Rank	Result Type
1397.8654	1397.8292	-0.0362	-26	9	17	IAIYELLFK	29	96.941	(N-term)_iTRAQ[0], Lysine(K)_iTRAQ[9]	[7] F5 120912	486/478	2.103	1.636	1.383	1	Mascot
1857.9705	1858.0178	0.0473	25	139	153	KAEAGAGSATEFQFR	42	99.849	(N-term)_iTRAQ[0], Lysine(K)_iTRAQ[1]	[3] F6 and F9	1179/1171	1.269	1.167	1.241	1	Mascot

663	RecName: Full=Huntingtin; AltName: Full=Huntington disease protein homolog; Short=HD protein homolo	gi|1708162	363864.9	1	71	1.101	1.050	0.955	0.000	0.000	0.000	1	1	1	100

Peptide Information

Calc. Mass	Obsrv. Mass	± da	± ppm	Start Seq.	End Seq.	Sequence	Ion Score	C. I. %	Modification	Plate [#] Name	Gel Idx/Pos [4700 Sample Name]	iTRAQ Ratio 115/114*	iTRAQ Ratio 116/114*	iTRAQ Ratio 117/114*	Rank	Result Type
2078.0811	2077.925	-0.1561	-75	1158	1172	EKEPGEQTSTPMSPK	72	100	(N-term)_iTRAQ[0], Lysine(K)_iTRAQ[2,15]	[6] F8 110912	128/120	1.101	1.050	0.955	1	Mascot

664	heterogeneous nuclear ribonucleoprotein U [Rattus norvegicus]	gi|148747541	98120.4	1	71	0.979	1.076	1.168	0.000	0.000	0.000	1	1	1	100

Protein Group

SP120 [Rattus norvegicus]	gi|624918	98136.4

Peptide Information

Calc. Mass	Obsrv. Mass	± da	± ppm	Start Seq.	End Seq.	Sequence	Ion Score	C. I. %	Modification	Plate [#] Name	Gel Idx/Pos [4700 Sample Name]	iTRAQ Ratio 115/114*	iTRAQ Ratio 116/114*	iTRAQ Ratio 117/114*	Rank	Result Type
1579.7889	1579.7488	-0.0401	-25	230	239	GYFEYIEENK	71	100	(N-term)_iTRAQ[0], Lysine(K)_iTRAQ[10]	[7] F5 120912	298/290	0.979	1.076	1.168	1	Mascot

665	hearing impairment protein DFNA5 [Rattus norvegicus]	gi|31742522	27056.5	1	71	1.228	1.067	0.953	0.000	0.000	0.000	1	1	1	100

Peptide Information

Calc. Mass	Obsrv. Mass	± da	± ppm	Start Seq.	End Seq.	Sequence	Ion Score	C. I. %	Modification	Plate [#] Name	Gel Idx/Pos [4700 Sample Name]	iTRAQ Ratio 115/114*	iTRAQ Ratio 116/114*	iTRAQ Ratio 117/114*	Rank	Result Type
2037.0247	2036.8801	-0.1446	-71	84	99	VQGEHPGPQDEVSNQK	71	100	(N-term)_iTRAQ[0], Lysine(K)_iTRAQ[16]	[6] F8 110912	114/106	1.228	1.067	0.953	1	Mascot

666	cytochrome c oxidase subunit II [Rattus norvegicus]	gi|110189718	26913.6	1	71	1.276	1.056	1.061	0.000	0.000	0.000	1	1	1	100

Protein Group

cytochrome c oxidase subunit II [Rattus norvegicus]	gi|829018	26994.7
cytochrome c oxidase subunit II [Rattus norvegicus]	gi|110189666	26927.6
cytochrome c oxidase subunit II [Rattus tanezumi]	gi|218436966	26917.6
cytochrome oxidase subunit II [Rattus norvegicus]	gi|829023	26984.7

Peptide Information

Calc. Mass	Obsrv. Mass	± da	± ppm	Start Seq.	End Seq.	Sequence	Ion Score	C. I. %	Modification	Plate [#] Name	Gel Idx/Pos [4700 Sample Name]	iTRAQ Ratio 115/114*	iTRAQ Ratio 116/114*	iTRAQ Ratio 117/114*	Rank	Result Type
2181.1909	2181.1201	-0.0708	-32	83	98	ILYMMDEINNPVLTVK	71	100	(N-term)_iTRAQ[0], Lysine(K)_iTRAQ[16]	[1] F3 030912	485/477	1.276	1.056	1.061	1	Mascot

667	ubiquitin carboxyl-terminal hydrolase CYLD [Rattus norvegicus]	gi|62821831	116623.9	1	71	0.521	0.948	0.741	0.000	0.000	0.000	1	1	1	100

Peptide Information

Calc. Mass	Obsrv. Mass	± da	± ppm	Start Seq.	End Seq.	Sequence	Ion Score	C. I. %	Modification	Plate [#] Name	Gel Idx/Pos [4700 Sample Name]	iTRAQ Ratio 115/114*	iTRAQ Ratio 116/114*	iTRAQ Ratio 117/114*	Rank	Result Type
1544.7954	1544.7456	-0.0498	-32	173	184	GQGFTDGVYQGK	71	100	(N-term)_iTRAQ[0], Lysine(K)_iTRAQ[12]	[7] F5 120912	150/142	0.521	0.948	0.741	1	Mascot

668	centaurin/PIP3-binding protein [Rattus norvegicus]	gi|4691714	47522.5	2	70	1.226	1.342	0.987	0.093	0.132	0.055	2	2	2	100

Protein Group

centaurin alpha [Rattus norvegicus]	gi|1435195	52551.9
centaurin, alpha 1 [Rattus norvegicus]	gi|19424252	47565.5

Peptide Information

Calc. Mass	Obsrv. Mass	± da	± ppm	Start Seq.	End Seq.	Sequence	Ion Score	C. I. %	Modification	Plate [#] Name	Gel Idx/Pos [4700 Sample Name]	iTRAQ Ratio 115/114*	iTRAQ Ratio 116/114*	iTRAQ Ratio 117/114*	Rank	Result Type

Calc. Mass	Obsrv. Mass	± da	± ppm	Start Seq.	End Seq.	Sequence	Ion Score	C. I. %	Modification	Plate [#] Name	Gel Idx/Pos [4700 Sample Name]	iTRAQ Ratio 115/114*	iTRAQ Ratio 116/114*	iTRAQ Ratio 117/114*	Rank	Result Type
1406.7552	1406.7183	-0.0369	-26	217	226	EIVDWFNALR	29	97.204	(N-term)_iTRAQ[0]	[1] F3 030912	477/469	1.319	1.222	1.041	1	Mascot
1751.0214	1751.0269	0.0055	3	189	201	IGHPHGLQVTYLK	41	99.806	(N-term)_iTRAQ[0], Lysine(K)_iTRAQ[13]	[2] F12 040912	272/264	1.140	1.474	0.935	1	Mascot

669	methylcrotonoyl-CoA carboxylase beta chain, mitochondrial [Rattus norvegicus]	gi|58865926	65494.8	2	70	0.962	1.249	1.222	0.211	0.076	0.039	2	2	2	100

Peptide Information

Calc. Mass	Obsrv. Mass	± da	± ppm	Start Seq.	End Seq.	Sequence	Ion Score	C. I. %	Modification	Plate [#] Name	Gel Idx/Pos [4700 Sample Name]	iTRAQ Ratio 115/114*	iTRAQ Ratio 116/114*	iTRAQ Ratio 117/114*	Rank	Result Type
1658.8511	1658.8057	-0.0454	-27	526	538	LWDDGIIDPVDTR	34	98.968	(N-term)_iTRAQ[0]	[1] F3 030912	368/360	0.789	1.178	1.184	1	Mascot
1848.8636	1848.8848	0.0212	11	512	525	RFEEEGNPYYSSAR	37	99.46	(N-term)_iTRAQ[0]	[3] F6 and F9	1150/1142	1.173	1.325	1.261	1	Mascot

670	60S acidic ribosomal protein P1 [Rattus norvegicus]	gi|56090277	12735.5	1	70	1.202	0.988	0.974	0.026	0.014	0.067	2	2	2	100

Peptide Information

Calc. Mass	Obsrv. Mass	± da	± ppm	Start Seq.	End Seq.	Sequence	Ion Score	C. I. %	Modification	Plate [#] Name	Gel Idx/Pos [4700 Sample Name]	iTRAQ Ratio 115/114*	iTRAQ Ratio 116/114*	iTRAQ Ratio 117/114*	Rank	Result Type
1991.1	1991.0338	-0.0662	-33	34	49	AAGVNVEPFWPGLFAK	58	99.996	(N-term)_iTRAQ[0], Lysine(K)_iTRAQ[16]	[5] F4	368/360	1.176	0.974	1.041	1	Mascot
1991.1	1991.0392	-0.0608	-31	34	49	AAGVNVEPFWPGLFAK	70	100	(N-term)_iTRAQ[0], Lysine(K)_iTRAQ[16]	[1] F3 030912	469/461	1.227	1.002	0.912	1	Mascot

671	Scg2 protein [Rattus norvegicus]	gi|38181552	72674.4	2	70	1.855	3.046	2.486	2.977	5.182	3.122	2	2	2	100

Protein Group

secretogranin-2 precursor [Rattus norvegicus]	gi|12083601	77039.7

Peptide Information

Calc. Mass	Obsrv. Mass	± da	± ppm	Start Seq.	End Seq.	Sequence	Ion Score	C. I. %	Modification	Plate [#] Name	Gel Idx/Pos [4700 Sample Name]	iTRAQ Ratio 115/114*	iTRAQ Ratio 116/114*	iTRAQ Ratio 117/114*	Rank	Result Type
1402.6876	1402.6028	-0.0848	-60	412	421	SPYFPNQYSR	31	98.19	(N-term)_iTRAQ[0]	[5] F4	137/129	0.712	1.128	1.102	1	Mascot
1747.853	1747.8928	0.0398	23	511	523	EHLGQGSSQEMEK	39	99.687	(N-term)_iTRAQ[0], Lysine(K)_iTRAQ[13]	[3] F6 and F9	1088/1080	4.833	8.229	5.607	1	Mascot

672	histone H1b subtype [rats, liver, Peptide Partial, 97 aa]	gi|253345	12869.8	1	70	1.000	1.025	1.088	0.000	0.000	0.000	1	1	1	100

Protein Group

histone H1.b, hepatic - rat (fragments)	gi|111782	10871.8

Peptide Information

Calc. Mass	Obsrv. Mass	± da	± ppm	Start Seq.	End Seq.	Sequence	Ion Score	C. I. %	Modification	Plate [#] Name	Gel Idx/Pos [4700 Sample Name]	iTRAQ Ratio 115/114*	iTRAQ Ratio 116/114*	iTRAQ Ratio 117/114*	Rank	Result Type
1381.7573	1381.7245	-0.0328	-24	12	22	ALAAGGYDVEK	70	100	(N-term)_iTRAQ[0], Lysine(K)_iTRAQ[11]	[7] F5 120912	157/149	1.000	1.025	1.088	1	Mascot

673	striatin [Rattus norvegicus]	gi|9507157	93601.7	2	70	0.679	0.560	0.845	0.278	0.217	0.158	2	2	2	100

Peptide Information

Calc. Mass	Obsrv. Mass	± da	± ppm	Start Seq.	End Seq.	Sequence	Ion Score	C. I. %	Modification	Plate [#] Name	Gel Idx/Pos [4700 Sample Name]	iTRAQ Ratio 115/114*	iTRAQ Ratio 116/114*	iTRAQ Ratio 117/114*	Rank	Result Type
1718.8292	1718.772	-0.0572	-33	637	649	GYTSIFNMETQQR	40	99.774	(N-term)_iTRAQ[0]	[1] F3 030912	303/295	0.957	0.777	1.003	1	Mascot
2364.2332	2364.1484	-0.0848	-36	563	583	GPLLGHTDAVWGLAYSA AHQR	30	97.426	(N-term)_iTRAQ[0]	[6] F8 110912	410/402	0.482	0.403	0.712	1	Mascot

674	synaptopodin [Rattus norvegicus]	gi|3986121	78724.4	1	69	1.086	1.502	1.811	0.000	0.000	0.000	1	1	1	100

Protein Group

RecName: Full=Synaptopodin	gi|48428501	105912.3

Peptide Information

Calc. Mass	Obsrv. Mass	± da	± ppm	Start Seq.	End Seq.	Sequence	Ion Score	C. I. %	Modification	Plate [#] Name	Gel Idx/Pos [4700 Sample Name]	iTRAQ Ratio 115/114*	iTRAQ Ratio 116/114*	iTRAQ Ratio 117/114*	Rank	Result Type
2180.0593	2179.9973	-0.062	-28	434	451	SGPAAAEETVPEWASCL K	70	100	(N-term)_iTRAQ[0], Lysine(K)_iTRAQ[18], MMTS (C)[16]	[1] F3 030912	404/396	1.086	1.502	1.811	1	Mascot

675	mitochondrial import receptor subunit TOM22 homolog [Rattus norvegicus]	gi|47058998	16345.4	1	70	0.987	1.087	1.174	0.000	0.000	0.000	1	1	1	100

Peptide Information

Calc. Mass	Obsrv. Mass	± da	± ppm	Start Seq.	End Seq.	Sequence	Ion Score	C. I. %	Modification	Plate [#] Name	Gel Idx/Pos [4700 Sample Name]	iTRAQ Ratio 115/114*	iTRAQ Ratio 116/114*	iTRAQ Ratio 117/114*	Rank	Result Type
1701.8827	1701.7931	-0.0896	-53	106	117	LQMEQQQQLQQR	70	100	(N-term)_iTRAQ[0]	[1] F3 030912	176/168	0.987	1.087	1.174	1	Mascot

676	Splicing factor, arginine/serine-rich 6 [Rattus norvegicus]	gi|56270156	43032.9	1	69	1.161	1.128	1.683	0.000	0.000	0.000	1	1	1	100

Peptide Information

Calc. Mass	Obsrv. Mass	± da	± ppm	Start Seq.	End Seq.	Sequence	Ion Score	C. I. %	Modification	Plate [#] Name	Gel Idx/Pos [4700 Sample Name]	iTRAQ Ratio 115/114*	iTRAQ Ratio 116/114*	iTRAQ Ratio 117/114*	Rank	Result Type
1577.8169	1577.8385	0.0216	14	132	143	QAGEVTYADAHK	69	100	(N-term)_iTRAQ[0], Lysine(K)_iTRAQ[12]	[3] F6 and F9	1094/1086	1.161	1.128	1.683	1	Mascot

677	histone (H1d) [Rattus norvegicus]	gi|204595	30766.3	2	69	0.933	1.054	1.020	0.132	0.013	0.325	2	2	2	100

Protein Group

histone H1.2 [Rattus norvegicus]	gi|18959218	30908.4

Peptide Information

Calc. Mass	Obsrv. Mass	± da	± ppm	Start Seq.	End Seq.	Sequence	Ion Score	C. I. %	Modification	Plate [#] Name	Gel Idx/Pos [4700 Sample Name]	iTRAQ Ratio 115/114*	iTRAQ Ratio 116/114*	iTRAQ Ratio 117/114*	Rank	Result Type
1486.8727	1486.7725	-0.1002	-67	35	46	ASGPPVSELITK	28	96.365	(N-term)_iTRAQ[0], Lysine(K)_iTRAQ[12]	[5] F4	174/166	0.817	1.066	0.774	1	Mascot
1759.0701	1759.0966	0.0265	15	34	46	KASGPPVSELITK	41	99.785	(N-term)_iTRAQ[0], Lysine(K)_iTRAQ[1,13]	[3] F6 and F9	1198/1190	1.065	1.042	1.345	1	Mascot

678	eukaryotic translation initiation factor 2 subunit 1 [Rattus norvegicus]	gi|9506571	39485.6	1	69	1.498	1.036	1.202	0.000	0.000	0.000	1	1	1	100

Peptide Information

Calc. Mass	Obsrv. Mass	± da	± ppm	Start Seq.	End Seq.	Sequence	Ion Score	C. I. %	Modification	Plate [#] Name	Gel Idx/Pos [4700 Sample Name]	iTRAQ Ratio 115/114*	iTRAQ Ratio 116/114*	iTRAQ Ratio 117/114*	Rank	Result Type
2154.9231	2154.8103	-0.1128	-52	296	312	ENAEVDGDDDAEEMEAK	69	100	(N-term)_iTRAQ[0], Lysine(K)_iTRAQ[17]	[5] F4	108/100	1.498	1.036	1.202	1	Mascot

679	proteasome subunit alpha type-1 [Rattus norvegicus]	gi|8394060	31602.1	1	69	0.930	1.079	1.095	0.000	0.000	0.000	1	1	1	100

Peptide Information

Calc. Mass	Obsrv. Mass	± da	± ppm	Start Seq.	End Seq.	Sequence	Ion Score	C. I. %	Modification	Plate [#] Name	Gel Idx/Pos [4700 Sample Name]	iTRAQ Ratio 115/114*	iTRAQ Ratio 116/114*	iTRAQ Ratio 117/114*	Rank	Result Type
1922.9117	1922.8165	-0.0952	-50	4	18	NQYDNDVTVWSPQGR	69	100	(N-term)_iTRAQ[0]	[1] F3 030912	241/233	0.930	1.079	1.095	1	Mascot

680	kinesin light chain 4 [Rattus norvegicus]	gi|57527498	73756.8	1	69	1.025	0.706	0.835	0.000	0.000	0.000	1	1	1	100

Peptide Information

Calc. Mass	Obsrv. Mass	± da	± ppm	Start Seq.	End Seq.	Sequence	Ion Score	C. I. %	Modification	Plate [#] Name	Gel Idx/Pos [4700 Sample Name]	iTRAQ Ratio 115/114*	iTRAQ Ratio 116/114*	iTRAQ Ratio 117/114*	Rank	Result Type
1788.9113	1788.8304	-0.0809	-45	194	210	GQGAAAAQQGGYEIPAR	69	100	(N-term)_iTRAQ[0]	[1] F3 030912	172/164	1.025	0.706	0.835	1	Mascot

681	B-cell receptor-associated protein 31 [Rattus norvegicus]	gi|51948420	32119	1	68	0.738	0.556	0.403	0.000	0.000	0.000	1	1	1	100

Peptide Information

Calc. Mass	Obsrv. Mass	± da	± ppm	Start Seq.	End Seq.	Sequence	Ion Score	C. I. %	Modification	Plate [#] Name	Gel Idx/Pos [4700 Sample Name]	iTRAQ Ratio 115/114*	iTRAQ Ratio 116/114*	iTRAQ Ratio 117/114*	Rank	Result Type
2155.0786	2155.1213	0.0427	20	80	95	VNLQNNPGAMEHFHMK	68	100	(N-term)_iTRAQ[0], Lysine(K)_iTRAQ[16]	[4] F7 and F10+11	1152/1144	0.738	0.556	0.403	1	Mascot

682	ectonucleoside triphosphate diphosphohydrolase 3 [Rattus norvegicus]	gi|30017439	62555.9	1	68	0.814	1.049	1.060	0.000	0.000	0.000	1	1	1	100

Peptide Information

Calc. Mass	Obsrv. Mass	± da	± ppm	Start Seq.	End Seq.	Sequence	Ion Score	C. I. %	Modification	Plate [#] Name	Gel Idx/Pos [4700 Sample Name]	iTRAQ Ratio	iTRAQ Ratio	iTRAQ Ratio	Rank	Result Type

Calc. Mass	Obsrv. Mass	± da	± ppm	Start Seq.	End Seq.	Sequence	Ion Score	C. I. %	Modification	Plate [#] Name	Gel Idx/Pos [4700 Sample Name]	iTRAQ Ratio 115/114*	iTRAQ Ratio 116/114*	iTRAQ Ratio 117/114*	Rank	Result Type
1951.9607	1951.8429	-0.1178	-60	96	111	GSGISSYENNPQDAPK	68	100	(N-term)_iTRAQ[0], Lysine(K)_iTRAQ[16]	[5] F4	102/94	0.814	1.049	1.060	1	Mascot

683	GABAB receptor 1c [Rattus norvegicus]	gi|3929913	105964.3	1	68	0.826	0.778	0.724	0.000	0.000	0.000	1	1	1	100

Protein Group

GABA type B receptor 1f [Rattus norvegicus]	gi|14582574	119517
GABA-BR1b receptor [Rattus norvegicus]	gi|1929421	102590.7
GABAB receptor 1d [Rattus norvegicus]	gi|3929915	98331.5
RecName: Full=Gamma-aminobutyric acid type B receptor subunit 1; Short=GABA-B receptor 1; Short=GAB	gi|12643494	120308.4
gamma-aminobutyric acid type B receptor subunit 1 [Rattus norvegicus]	gi|77404238	116934.8

Peptide Information

Calc. Mass	Obsrv. Mass	± da	± ppm	Start Seq.	End Seq.	Sequence	Ion Score	C. I. %	Modification	Plate [#] Name	Gel Idx/Pos [4700 Sample Name]	iTRAQ Ratio 115/114*	iTRAQ Ratio 116/114*	iTRAQ Ratio 117/114*	Rank	Result Type
1727.8157	1727.7561	-0.0596	-34	778	789	GEWQSETQDTMK	68	100	(N-term)_iTRAQ[0], Lysine(K)_iTRAQ[12]	[7] F5 120912	152/144	0.826	0.778	0.724	1	Mascot

684	RecName: Full=Phosphatase and actin regulator 1	gi|48428187	72018.4	2	68	0.790	0.822	0.672	0.008	0.017	0.500	2	2	2	100

Peptide Information

Calc. Mass	Obsrv. Mass	± da	± ppm	Start Seq.	End Seq.	Sequence	Ion Score	C. I. %	Modification	Plate [#] Name	Gel Idx/Pos [4700 Sample Name]	iTRAQ Ratio 115/114*	iTRAQ Ratio 116/114*	iTRAQ Ratio 117/114*	Rank	Result Type
1578.8247	1578.7701	-0.0546	-35	77	89	ISFNLGAAEEVER	35	99.167	(N-term)_iTRAQ[0]	[1] F3 030912	327/319	0.798	0.806	0.385	1	Mascot
1700.9052	1700.879	-0.0262	-15	466	478	LSQRPTAEELQR	33	98.779	(N-term)_iTRAQ[0]	[4] F7 and F10+11	184/176	0.781	0.838	1.172	1	Mascot

685	calpain small subunit [Rattus norvegicus]	gi|1794205	29738.8	1	68	0.724	0.537	0.916	0.000	0.000	0.000	1	1	1	100

Peptide Information

Calc. Mass	Obsrv. Mass	± da	± ppm	Start Seq.	End Seq.	Sequence	Ion Score	C. I. %	Modification	Plate [#] Name	Gel Idx/Pos [4700 Sample Name]	iTRAQ Ratio 115/114*	iTRAQ Ratio 116/114*	iTRAQ Ratio 117/114*	Rank	Result Type
1937.8597	1937.7135	-0.1462	-75	83	97	SHYSNIEANESEEER	68	100	(N-term)_iTRAQ[0]	[6] F8 110912	127/119	0.724	0.537	0.916	1	Mascot

686	secretory carrier-associated membrane protein 5 [Rattus norvegicus]	gi|13929020	27752	1	67	0.984	0.999	1.016	0.269	0.222	0.386	2	2	2	100

Peptide Information

Calc. Mass	Obsrv. Mass	± da	± ppm	Start Seq.	End Seq.	Sequence	Ion Score	C. I. %	Modification	Plate [#] Name	Gel Idx/Pos [4700 Sample Name]	iTRAQ Ratio 115/114*	iTRAQ Ratio 116/114*	iTRAQ Ratio 117/114*	Rank	Result Type
1594.8112	1594.75	-0.0612	-38	186	196	AQEEWTTGAWK	67	100	(N-term)_iTRAQ[0], Lysine(K)_iTRAQ[11]	[7] F5 120912	222/214	0.773	0.818	0.736	1	Mascot
1594.8112	1594.7667	-0.0445	-28	186	196	AQEEWTTGAWK	59	99.997	(N-term)_iTRAQ[0], Lysine(K)_iTRAQ[11]	[3] F6 and F9	266/258	1.253	1.221	1.402	1	Mascot

687	prion-related protein [Rattus norvegicus]	gi|206392	26055.7	1	67	1.979	1.393	1.447	0.000	0.000	0.000	1	1	1	100

Protein Group

major prion protein precursor [Rattus norvegicus]	gi|6981410	29699.6

Peptide Information

Calc. Mass	Obsrv. Mass	± da	± ppm	Start Seq.	End Seq.	Sequence	Ion Score	C. I. %	Modification	Plate [#] Name	Gel Idx/Pos [4700 Sample Name]	iTRAQ Ratio 115/114*	iTRAQ Ratio 116/114*	iTRAQ Ratio 117/114*	Rank	Result Type
1789.8877	1789.8093	-0.0784	-44	181	192	VVEQMCVTQYQK	67	100	(N-term)_iTRAQ[0], Lysine(K)_iTRAQ[12], MMTS (C)[6]	[5] F4	207/199	1.979	1.393	1.447	1	Mascot

688	S100 alpha [Rattus sp.]	gi|8318583	10868.7	1	67	2.470	2.375	2.563	0.000	0.000	0.000	1	1	1	100

Protein Group

protein S100-A1 [Rattus norvegicus]	gi|56090267	12040.2

Peptide Information

Calc. Mass	Obsrv. Mass	± da	± ppm	Start Seq.	End Seq.	Sequence	Ion Score	C. I. %	Modification	Plate [#] Name	Gel Idx/Pos [4700 Sample Name]	iTRAQ Ratio 115/114*	iTRAQ Ratio 116/114*	iTRAQ Ratio 117/114*	Rank	Result Type
2024.1162	2024.0776	-0.0386	-19	26	40	DLLQTELSSFLDVQK	67	100	(N-term)_iTRAQ[0], Lysine(K)_iTRAQ[15]	[1] F3 030912	517/509	2.470	2.375	2.563	1	Mascot

689	single-stranded DNA-binding protein, mitochondrial precursor [Rattus norvegicus]	gi|34328536	18596.9	1	67	0.809	1.132	0.755	0.000	0.000	0.000	1	1	1	99.999

Peptide Information

Calc. Mass	Obsrv. Mass	± da	± ppm	Start Seq.	End Seq.	Sequence	Ion Score	C. I. %	Modification	Plate [#] Name	Gel Idx/Pos [4700 Sample Name]	iTRAQ Ratio 115/114*	iTRAQ Ratio 116/114*	iTRAQ Ratio 117/114*	Rank	Result Type
1916.8906	1916.8156	-0.075	-39	67	81	SGDNEAYQMGDVSQK	67	99.999	(N-term)_iTRAQ[0], Lysine(K)_iTRAQ[15]	[7] F5 120912	140/132	0.809	1.132	0.755	1	Mascot

690	lanthionine synthetase C-like protein 1 [Rattus norvegicus]	gi|11140166	18590.4	2	66	0.930	0.703	0.865	0.100	0.222	0.183	2	2	2	99.999

Protein Group

lanC-like protein 1 [Rattus norvegicus]	gi|39930499	49266.8

Peptide Information

Calc. Mass	Obsrv. Mass	± da	± ppm	Start Seq.	End Seq.	Sequence	Ion Score	C. I. %	Modification	Plate [#] Name	Gel Idx/Pos [4700 Sample Name]	iTRAQ Ratio 115/114*	iTRAQ Ratio 116/114*	iTRAQ Ratio 117/114*	Rank	Result Type
1190.6323	1190.5665	-0.0658	-55	42	49	ELLQQMER	34	98.98	(N-term)_iTRAQ[0]	[5] F4	155/147	1.029	0.925	0.714	1	Mascot
1503.72	1503.631	-0.089	-59	16	27	SLAENYFDSTGR	33	98.659	(N-term)_iTRAQ[0]	[5] F4	175/167	0.840	0.534	1.048	1	Mascot

691	Ribosomal protein L4 [Rattus norvegicus]	gi|39645153	55859	2	66	0.967	1.001	0.830	0.041	0.036	0.077	2	2	2	99.999

Protein Group

60S ribosomal protein L4 [Rattus norvegicus]	gi|11968086	55860.9

Peptide Information

Calc. Mass	Obsrv. Mass	± da	± ppm	Start Seq.	End Seq.	Sequence	Ion Score	C. I. %	Modification	Plate [#] Name	Gel Idx/Pos [4700 Sample Name]	iTRAQ Ratio 115/114*	iTRAQ Ratio 116/114*	iTRAQ Ratio 117/114*	Rank	Result Type
1034.5881	1034.5717	-0.0164	-16	176	181	AWNDIK	31	97.844	(N-term)_iTRAQ[0], Lysine(K)_iTRAQ[6]	[4] F7 and F10+11	198/190	1.008	1.037	0.907	1	Mascot
1568.8683	1568.9218	0.0535	34	259	268	KLDELYGTWR	37	99.515	(N-term)_iTRAQ[0], Lysine(K)_iTRAQ[1]	[4] F7 and F10+11	1200/1192	0.927	0.966	0.760	1	Mascot

692	beta-globin [Rattus norvegicus]	gi|984679	17920.5	2	66	0.175	0.319	0.208	0.031	0.101	0.015	2	2	2	99.999

Protein Group

beta 1 globin [rats, Sprague-Dawley, Peptide, 146 aa]	gi|546056	17789.5
hemoglobin subunit beta-1 [Rattus norvegicus]	gi|17985949	17934.6
major beta-hemoglobin [Rattus norvegicus]	gi|204570	17948.6

Peptide Information

Calc. Mass	Obsrv. Mass	± da	± ppm	Start Seq.	End Seq.	Sequence	Ion Score	C. I. %	Modification	Plate [#] Name	Gel Idx/Pos [4700 Sample Name]	iTRAQ Ratio 115/114*	iTRAQ Ratio 116/114*	iTRAQ Ratio 117/114*	Rank	Result Type
1418.828	1418.7878	-0.0402	-28	32	41	LLVVYPWTQR	31	98.101	(N-term)_iTRAQ[0]	[1] F3 030912	395/387	0.207	0.420	0.223	1	Mascot
1442.736	1442.6644	-0.0716	-50	19	31	VNPDDVGGEALGR	35	99.261	(N-term)_iTRAQ[0]	[1] F3 030912	201/193	0.149	0.243	0.194	1	Mascot

693	NYGGF2 [Rattus norvegicus]	gi|31323299	19389.5	2	66	0.842	0.720	0.508	0.012	0.333	0.036	2	2	2	99.999

Peptide Information

Calc. Mass	Obsrv. Mass	± da	± ppm	Start Seq.	End Seq.	Sequence	Ion Score	C. I. %	Modification	Plate [#] Name	Gel Idx/Pos [4700 Sample Name]	iTRAQ Ratio 115/114*	iTRAQ Ratio 116/114*	iTRAQ Ratio 117/114*	Rank	Result Type
1407.7141	1407.74	0.0259	18	38	47	LEENVHYFGR	39	99.663	(N-term)_iTRAQ[0]	[3] F6 and F9	1195/1187	0.854	1.053	0.474	1	Mascot

Calc. Mass	Obsrv. Mass	± da	± ppm	Start Seq.	End Seq.	Sequence	Ion Score	C. I. %	Modification	Plate [#] Name	Gel Idx/Pos [4700 Sample Name]	iTRAQ Ratio 115/114*	iTRAQ Ratio 116/114*	iTRAQ Ratio 117/114*	Rank	Result Type
1880.05	1880.0813	0.0313	17	126	137	YAPENLDEQIKK	28	95.738	(N-term)_iTRAQ[0], Lysine(K)_iTRAQ[11, 12]	[3] F6 and F9	1178/1170	0.831	0.492	0.544	1	Mascot

694	CB1 cannabinoid receptor-interacting protein 1 [Rattus norvegicus]	gi|62079191	20657.9	1	66	0.822	1.137	1.368	0.000	0.000	0.000	1	1	1	99.999

Peptide Information

Calc. Mass	Obsrv. Mass	± da	± ppm	Start Seq.	End Seq.	Sequence	Ion Score	C. I. %	Modification	Plate [#] Name	Gel Idx/Pos [4700 Sample Name]	iTRAQ Ratio 115/114*	iTRAQ Ratio 116/114*	iTRAQ Ratio 117/114*	Rank	Result Type
1549.8624	1549.8396	-0.0228	-15	16	26	IQPNDGPVFFK	66	99.999	(N-term)_iTRAQ[0], Lysine(K)_iTRAQ[11]	[7] F5 120912	262/254	0.822	1.137	1.368	1	Mascot

695	RecName: Full=Solute carrier family 2, facilitated glucose transporter member 3; AltName: Full=Gluc	gi|585228	55787.2	1	66	0.838	0.764	0.798	0.051	0.132	0.242	2	2	2	99.999

Protein Group

Slc2a3 protein [Rattus norvegicus]	gi|38648899	37572.9
solute carrier family 2, facilitated glucose transporter member 3 [Rattus norvegicus]	gi|148747140	55769.1

Peptide Information

Calc. Mass	Obsrv. Mass	± da	± ppm	Start Seq.	End Seq.	Sequence	Ion Score	C. I. %	Modification	Plate [#] Name	Gel Idx/Pos [4700 Sample Name]	iTRAQ Ratio 115/114*	iTRAQ Ratio 116/114*	iTRAQ Ratio 117/114*	Rank	Result Type
1747.9436	1748.0023	0.0587	34	217	228	KEEDQATEILQR	66	99.999	(N-term)_iTRAQ[0], Lysine(K)_iTRAQ[1]	[3] F6 and F9	1153/1145	0.889	0.895	1.040	1	Mascot
1747.9436	1748.0272	0.0836	48	217	228	KEEDQATEILQR	28	95.874	(N-term)_iTRAQ[0], Lysine(K)_iTRAQ[1]	[4] F7 and F10+11	1084/1076	0.791	0.652	0.613	1	Mascot

696	PSD-95 binding protein [Rattus norvegicus]	gi|2766159	83063.4	1	66	0.904	1.023	1.085	0.000	0.000	0.000	1	1	1	99.999

Protein Group

disks large-associated protein 1 [Rattus norvegicus]	gi|19923689	119190.3
guanylate kinase associated protein [Rattus norvegicus]	gi|1857139	79828.6

Peptide Information

Calc. Mass	Obsrv. Mass	± da	± ppm	Start Seq.	End Seq.	Sequence	Ion Score	C. I. %	Modification	Plate [#] Name	Gel Idx/Pos [4700 Sample Name]	iTRAQ Ratio 115/114*	iTRAQ Ratio 116/114*	iTRAQ Ratio 117/114*	Rank	Result Type
2353.1201	2352.9817	-0.1384	-59	56	74	AMGDEDSGDSDTSPKPSPK	66	99.999	(N-term)_iTRAQ[0], Lysine(K)_iTRAQ[15, 19]	[6] F8 110912	109/101	0.904	1.023	1.085	1	Mascot

697	ubiquilin-1 [Rattus norvegicus]	gi|48675852	63704.5	2	66	1.174	1.155	0.984	0.476	0.394	0.274	2	2	2	99.999

Protein Group

RecName: Full=Ubiquilin-1; AltName: Full=Protein linking IAP with cytoskeleton 1; Short=PLIC-1	gi|48474728	63761.5

Peptide Information

Calc. Mass	Obsrv. Mass	± da	± ppm	Start Seq.	End Seq.	Sequence	Ion Score	C. I. %	Modification	Plate [#] Name	Gel Idx/Pos [4700 Sample Name]	iTRAQ Ratio 115/114*	iTRAQ Ratio 116/114*	iTRAQ Ratio 117/114*	Rank	Result Type
1729.0258	1728.9698	-0.056	-32	62	74	SHIDQLVLIFAGK	34	98.992	(N-term)_iTRAQ[0], Lysine(K)_iTRAQ[13]	[6] F8 110912	470/462	0.835	0.861	0.770	1	Mascot
2428.2732	2428.1245	-0.1487	-61	37	53	EKEEFAVPENSSVQQFK	32	98.477	(N-term)_iTRAQ[0], Lysine(K)_iTRAQ[2,17]	[6] F8 110912	261/253	1.650	1.549	1.257	1	Mascot

698	ADP-ribosylation factor-like protein 6-interacting protein 1 [Rattus norvegicus]	gi|38454218	25735	1	66	0.630	1.067	1.093	0.000	0.000	0.000	1	1	1	99.999

Peptide Information

Calc. Mass	Obsrv. Mass	± da	± ppm	Start Seq.	End Seq.	Sequence	Ion Score	C. I. %	Modification	Plate [#] Name	Gel Idx/Pos [4700 Sample Name]	iTRAQ Ratio 115/114*	iTRAQ Ratio 116/114*	iTRAQ Ratio 117/114*	Rank	Result Type
1523.7941	1523.8387	0.0446	29	105	114	FHEICSNLVK	66	99.999	(N-term)_iTRAQ[0], Lysine(K)_iTRAQ[10], MMTS (C)[5]	[3] F6 and F9	1287/1279	0.630	1.067	1.093	1	Mascot

699	RecName: Full=[Pyruvate dehydrogenase [acetyl-transferring]]-phosphatase 1, mitochondrial;	gi|12585293	64942.9	1	66	0.703	0.833	0.735	0.000	0.000	0.000	1	1	1	99.999

Short=PD

Peptide Information

Calc. Mass	Obsrv. Mass	± da	± ppm	Start Seq.	End Seq.	Sequence	Ion Score	C. I. %	Modification	Plate [#] Name	Gel Idx/Pos [4700 Sample Name]	iTRAQ Ratio 115/114*	iTRAQ Ratio 116/114*	iTRAQ Ratio 117/114*	Rank	Result Type
1506.9407	1506.8792	-0.0615	-41	185	194	ALLPILQWHK	66	99.999	(N-term)_iTRAQ[0], Lysine(K)_iTRAQ[10]	[6] F8 110912	417/409	0.703	0.833	0.735	1	Mascot

700	protein RUFY3 [Rattus norvegicus]	gi|68341991	59587.7	1	65	0.971	0.813	0.954	0.000	0.000	0.000	1	1	1	99.999	

Peptide Information

Calc. Mass	Obsrv. Mass	± da	± ppm	Start Seq.	End Seq.	Sequence	Ion Score	C. I. %	Modification	Plate [#] Name	Gel Idx/Pos [4700 Sample Name]	iTRAQ Ratio 115/114*	iTRAQ Ratio 116/114*	iTRAQ Ratio 117/114*	Rank	Result Type
1841.9855	1842.0616	0.0761	41	316	328	VKEESSYLLESNR	67	99.999	(N-term)_iTRAQ[0], Lysine(K)_iTRAQ[2]	[3] F6 and F9	1183/1175	0.971	0.813	0.954	1	Mascot

701	glutathione S-transferase Mu 3 [Rattus norvegicus]	gi|10120486	28260.6	2	65	0.972	1.194	1.420	0.241	0.418	0.426	2	2	2	99.999

Peptide Information

Calc. Mass	Obsrv. Mass	± da	± ppm	Start Seq.	End Seq.	Sequence	Ion Score	C. I. %	Modification	Plate [#] Name	Gel Idx/Pos [4700 Sample Name]	iTRAQ Ratio 115/114*	iTRAQ Ratio 116/114*	iTRAQ Ratio 117/114*	Rank	Result Type
1244.7136	1244.6593	-0.0543	-44	137	144	IYSEFLGK	30	97.581	(N-term)_iTRAQ[0], Lysine(K)_iTRAQ[8]	[7] F5 120912	287/279	0.779	0.885	1.093	1	Mascot
1264.7048	1264.6775	-0.0273	-22	145	152	RPWFAGDK	36	99.392	(N-term)_iTRAQ[0], Lysine(K)_iTRAQ[8]	[2] F12 040912	229/221	1.212	1.612	1.847	1	Mascot

702	Chain A, CtbpBARS IN TERNARY COMPLEX WITH NAD(H) AND PIDLSKK PEPTIDE	gi|33357012	41667.4	2	65	1.034	1.430	1.017	0.705	0.945	0.341	2	2	2	99.999

Protein Group

C-terminal binding protein 1 [Rattus norvegicus]	gi|50925463	44890
C-terminal-binding protein 1 [Rattus norvegicus]	gi|15011859	48984.2
Chain A, CtbpBARS: A DUAL-Function Protein Involved In Transcription Corepression And Golgi Membran	gi|33356997	41629.4

Peptide Information

Calc. Mass	Obsrv. Mass	± da	± ppm	Start Seq.	End Seq.	Sequence	Ion Score	C. I. %	Modification	Plate [#] Name	Gel Idx/Pos [4700 Sample Name]	iTRAQ Ratio 115/114*	iTRAQ Ratio 116/114*	iTRAQ Ratio 117/114*	Rank	Result Type
1269.7189	1269.7715	0.0526	41	140	148	TTWLHQALR	33	98.643	(N-term)_iTRAQ[0]	[3] F6 and F9	1218/1210	0.614	0.861	0.762	1	Mascot
1466.8101	1466.8019	-0.0082	-6	95	105	IGSGFDNIDIK	32	98.556	(N-term)_iTRAQ[0], Lysine(K)_iTRAQ[11]	[7] F5 120912	259/251	1.739	2.375	1.359	1	Mascot

703	sodium channel subunit beta-3 precursor [Rattus norvegicus]	gi|20806175	26356.2	2	65	1.031	0.867	0.779	0.091	0.249	0.327	2	2	2	99.999

Peptide Information

Calc. Mass	Obsrv. Mass	± da	± ppm	Start Seq.	End Seq.	Sequence	Ion Score	C. I. %	Modification	Plate [#] Name	Gel Idx/Pos [4700 Sample Name]	iTRAQ Ratio 115/114*	iTRAQ Ratio 116/114*	iTRAQ Ratio 117/114*	Rank	Result Type
1262.6542	1262.6243	-0.0299	-24	71	78	DFLIYEYR	34	98.922	(N-term)_iTRAQ[0]	[1] F3 030912	332/324	0.947	0.674	0.549	1	Mascot
1823.969	1823.9226	-0.0464	-25	125	136	EFEFEAHRPFVK	31	98.101	(N-term)_iTRAQ[0], Lysine(K)_iTRAQ[12]	[2] F12 040912	303/295	1.121	1.116	1.106	1	Mascot

704	xaa-Pro aminopeptidase 1 [Rattus norvegicus]	gi|18777778	75398.6	2	65	0.708	0.742	0.859	0.226	0.145	0.217	2	2	2	99.999

Peptide Information

Calc. Mass	Obsrv. Mass	± da	± ppm	Start Seq.	End Seq.	Sequence	Ion Score	C. I. %	Modification	Plate [#] Name	Gel Idx/Pos [4700 Sample Name]	iTRAQ Ratio 115/114*	iTRAQ Ratio 116/114*	iTRAQ Ratio 117/114*	Rank	Result Type
1744.0004	1743.9943	-0.0061	-3	450	464	GHIAVSAAVFPTGTK	37	99.552	(N-term)_iTRAQ[0], Lysine(K)_iTRAQ[15]	[4] F7 and F10+11	254/246	0.536	0.621	0.686	1	Mascot
1968.0283	1968.0129	-0.0154	-8	355	370	GGVTEISAADKAEEFR	27	95.262	(N-term)_iTRAQ[0], Lysine(K)_iTRAQ[11]	[4] F7 and F10+11	250/242	0.934	0.887	1.076	1	Mascot

705	vesicle-associated membrane protein-associated protein B [Rattus norvegicus]	gi|11177880	30253.2	2	64	1.154	0.940	1.039	0.227	0.033	0.097	2	2	2	99.999

Peptide Information

Calc. Mass	Obsrv. Mass	± da	± ppm	Start Seq.	End Seq.	Sequence	Ion Score	C. I. %	Modification	Plate [#] Name	Gel Idx/Pos [4700 Sample Name]	iTRAQ Ratio	iTRAQ Ratio	iTRAQ Ratio	Rank	Result Type

Calc. Mass	Obsrv. Mass	± da	± ppm	Start Seq.	End Seq.	Sequence	Ion Score	C. I. %	Modification	Plate [#] Name	Gel Idx/Pos [4700 Sample Name]	iTRAQ Ratio 115/114*	iTRAQ Ratio 116/114*	iTRAQ Ratio 117/114*	Rank	Result Type
1694.9006	1694.9674	0.0668	39	108	118	EAKPEDLMDSK	35	99.217	(N-term)_iTRAQ[0], Lysine(K)_iTRAQ[3,11]	[4] F7 and F10+11	1068/1060	1.381	0.908	1.136	1	Mascot
1937.0953	1937.0344	-0.0609	-31	4	17	VEQVLSLEPQHELK	29	97.052	(N-term)_iTRAQ[0], Lysine(K)_iTRAQ[14]	[3] F6 and F9	304/296	0.964	0.972	0.950	1	Mascot

706	COP9 signalosome complex subunit 8 [Rattus norvegicus]	gi|61557351	24131.6	2	64	0.982	1.125	1.024	0.187	0.105	0.047	2	2	2	99.999

Peptide Information

Calc. Mass	Obsrv. Mass	± da	± ppm	Start Seq.	End Seq.	Sequence	Ion Score	C. I. %	Modification	Plate [#] Name	Gel Idx/Pos [4700 Sample Name]	iTRAQ Ratio 115/114*	iTRAQ Ratio 116/114*	iTRAQ Ratio 117/114*	Rank	Result Type
1691.8473	1691.769	-0.0783	-46	147	160	GVLEQGWQADSTTR	34	98.93	(N-term)_iTRAQ[0]	[1] F3 030912	227/219	1.169	1.230	1.070	1	Mascot
1704.8789	1704.8121	-0.0668	-39	66	80	SANSELGGIWSVGQR	30	97.809	(N-term)_iTRAQ[0]	[1] F3 030912	301/293	0.824	1.028	0.979	1	Mascot

707	ribosomal protein S6 kinase alpha-1 [Rattus norvegicus]	gi|13592065	91510.7	1	64	1.170	0.796	1.326	0.000	0.000	0.000	1	1	1	99.999

Peptide Information

Calc. Mass	Obsrv. Mass	± da	± ppm	Start Seq.	End Seq.	Sequence	Ion Score	C. I. %	Modification	Plate [#] Name	Gel Idx/Pos [4700 Sample Name]	iTRAQ Ratio 115/114*	iTRAQ Ratio 116/114*	iTRAQ Ratio 117/114*	Rank	Result Type
2295.2932	2295.4028	0.1096	48	187	202	DLKPENILLDEEGHIK	64	99.999	(N-term)_iTRAQ[0], Lysine(K)_iTRAQ[3,16]	[4] F7 and F10+11	1218/1210	1.170	0.796	1.326	1	Mascot

708	hsp90 co-chaperone Cdc37 [Rattus norvegicus]	gi|16758570	50670	1	63	0.887	0.942	0.947	0.000	0.000	0.000	1	1	1	99.999

Protein Group

RecName: Full=Hsp90 co-chaperone Cdc37; AltName: Full=Hsp90 chaperone protein kinase-targeting subu	gi|32172404	50948.1

Peptide Information

Calc. Mass	Obsrv. Mass	± da	± ppm	Start Seq.	End Seq.	Sequence	Ion Score	C. I. %	Modification	Plate [#] Name	Gel Idx/Pos [4700 Sample Name]	iTRAQ Ratio 115/114*	iTRAQ Ratio 116/114*	iTRAQ Ratio 117/114*	Rank	Result Type
1605.7828	1605.7357	-0.0471	-29	244	254	TADQQYMEGFK	63	99.999	(N-term)_iTRAQ[0], Lysine(K)_iTRAQ[11]	[7] F5 120912	186/178	0.887	0.942	0.947	1	Mascot

709	cyclic AMP-regulated phosphoprotein isoform 1 [Rattus norvegicus]	gi|224451113	10698.5	1	63	0.758	0.696	0.821	0.024	0.242	0.077	2	2	2	99.999

Peptide Information

Calc. Mass	Obsrv. Mass	± da	± ppm	Start Seq.	End Seq.	Sequence	Ion Score	C. I. %	Modification	Plate [#] Name	Gel Idx/Pos [4700 Sample Name]	iTRAQ Ratio 115/114*	iTRAQ Ratio 116/114*	iTRAQ Ratio 117/114*	Rank	Result Type
1993.0336	1992.9263	-0.1073	-54	30	43	SESLDEEEKLELQR	63	99.999	(N-term)_iTRAQ[0], Lysine(K)_iTRAQ[9]	[6] F8 110912	225/217	0.782	0.938	0.751	1	Mascot
1993.0336	1993.0419	0.0083	4	30	43	SESLDEEEKLELQR	61	99.998	(N-term)_iTRAQ[0], Lysine(K)_iTRAQ[9]	[4] F7 and F10+11	220/212	0.734	0.516	0.898	1	Mascot

710	poly(rC)-binding protein 2 [Rattus norvegicus]	gi|61557337	41471.3	1	63	0.642	0.964	1.295	0.000	0.000	0.000	1	1	1	99.999

Protein Group

poly(rC)-binding protein 2 isoform b [Homo sapiens]	gi|14141166	41113.1

Peptide Information

Calc. Mass	Obsrv. Mass	± da	± ppm	Start Seq.	End Seq.	Sequence	Ion Score	C. I. %	Modification	Plate [#] Name	Gel Idx/Pos [4700 Sample Name]	iTRAQ Ratio 115/114*	iTRAQ Ratio 116/114*	iTRAQ Ratio 117/114*	Rank	Result Type
1990.1307	1990.0778	-0.0529	-27	145	160	AITIAGIPQSIIECVK	63	99.999	(N-term)_iTRAQ[0], Lysine(K)_iTRAQ[16], MMTS (C)[14]	[1] F3 030912	521/513	0.642	0.964	1.295	1	Mascot

711	proton myo-inositol cotransporter [Rattus norvegicus]	gi|19424326	69947.4	1	63	1.197	1.161	1.176	0.000	0.000	0.000	1	1	1	99.999

Peptide Information

Calc. Mass	Obsrv. Mass	± da	± ppm	Start Seq.	End Seq.	Sequence	Ion Score	C. I. %	Modification	Plate [#] Name	Gel Idx/Pos [4700 Sample Name]	iTRAQ Ratio 115/114*	iTRAQ Ratio 116/114*	iTRAQ Ratio 117/114*	Rank	Result Type
1803.9851	1804.0504	0.0653	36	571	582	KLEEIESLFDHR	63	99.999	(N-term)_iTRAQ[0], Lysine(K)_iTRAQ[1]	[4] F7 and F10+11	1284/1276	1.197	1.161	1.176	1	Mascot

712	probable ATP-dependent RNA helicase DDX5 [Rattus norvegicus]	gi|56090441	74653.2	2	63	0.946	1.056	1.131	0.055	0.175	0.039	2	2	2	99.999

Peptide Information

Calc. Mass	Obsrv. Mass	± da	± ppm	Start Seq.	End Seq.	Sequence	Ion Score	C. I. %	Modification	Plate [#] Name	Gel Idx/Pos [4700 Sample Name]	iTRAQ Ratio 115/114*	iTRAQ Ratio 116/114*	iTRAQ Ratio 117/114*	Rank	Result Type
1480.7412	1480.6935	-0.0477	-32	253	263	MLDMGFEPQIR	31	97.884	(N-term)_iTRAQ[0]	[1] F3 030912	345/337	1.002	1.231	1.093	1	Mascot
1533.7571	1533.6981	-0.059	-38	57	67	NFYQEHPDLAR	32	98.47	(N-term)_iTRAQ[0]	[6] F8 110912	206/198	0.894	0.905	1.169	1	Mascot

713	limbic system-associated membrane protein 6c-isoform [Rattus norvegicus]	gi|37499114	43276.6	1	63	0.914	1.579	1.181	0.000	0.000	0.000	1	1	1	99.999

Protein Group

limbic system-associated membrane protein precursor [Rattus norvegicus] gi|25742796 40308.9

Peptide Information

Calc. Mass	Obsrv. Mass	± da	± ppm	Start Seq.	End Seq.	Sequence	Ion Score	C. I. %	Modification	Plate [#] Name	Gel Idx/Pos [4700 Sample Name]	iTRAQ Ratio 115/114*	iTRAQ Ratio 116/114*	iTRAQ Ratio 117/114*	Rank	Result Type
2071.0354	2070.9812	-0.0542	-26	174	189	EFEGEEEYLEILGITR	63	99.999	(N-term)_iTRAQ[0]	[1] F3 030912	492/484	0.914	1.579	1.181	1	Mascot

714	S15 ribosomal protein [Rattus norvegicus]	gi|40287237	15080.4	1	62	0.959	1.010	1.177	0.000	0.000	0.000	1	1	1	99.999

Protein Group

40S ribosomal protein S15 [Homo sapiens] gi|4506687 19478.9

Peptide Information

Calc. Mass	Obsrv. Mass	± da	± ppm	Start Seq.	End Seq.	Sequence	Ion Score	C. I. %	Modification	Plate [#] Name	Gel Idx/Pos [4700 Sample Name]	iTRAQ Ratio 115/114*	iTRAQ Ratio 116/114*	iTRAQ Ratio 117/114*	Rank	Result Type
1786.0156	1785.8997	-0.1159	-65	49	60	EAPPMEKPEVVK	62	99.999	(N-term)_iTRAQ[0], Lysine(K)_iTRAQ[7,12]	[6] F8 110912	193/185	0.959	1.010	1.177	1	Mascot

715	OX-2 membrane glycoprotein precursor [Rattus norvegicus]	gi|13928702	33839.7	1	62	0.687	0.991	0.837	0.000	0.000	0.000	1	1	1	99.998

Peptide Information

Calc. Mass	Obsrv. Mass	± da	± ppm	Start Seq.	End Seq.	Sequence	Ion Score	C. I. %	Modification	Plate [#] Name	Gel Idx/Pos [4700 Sample Name]	iTRAQ Ratio 115/114*	iTRAQ Ratio 116/114*	iTRAQ Ratio 117/114*	Rank	Result Type
1500.8784	1500.8844	0.006	4	81	91	AHGVVIQPTYK	62	99.998	(N-term)_iTRAQ[0], Lysine(K)_iTRAQ[11]	[3] F6 and F9	1146/1138	0.687	0.991	0.837	1	Mascot

716	monoglyceride lipase [Rattus norvegicus]	gi|19923092	35627.5	2	62	1.079	1.087	1.152	0.079	0.090	0.221	2	2	2	99.998

Peptide Information

Calc. Mass	Obsrv. Mass	± da	± ppm	Start Seq.	End Seq.	Sequence	Ion Score	C. I. %	Modification	Plate [#] Name	Gel Idx/Pos [4700 Sample Name]	iTRAQ Ratio 115/114*	iTRAQ Ratio 116/114*	iTRAQ Ratio 117/114*	Rank	Result Type
1635.8563	1635.8009	-0.0554	-34	263	273	MYEGAYHVLHK	29	96.87	(N-term)_iTRAQ[0], Lysine(K)_iTRAQ[11]	[2] F12 040912	249/241	1.159	1.004	0.967	1	Mascot
1687.9867	1687.9779	-0.0088	-5	227	240	LTLPFLLLQGSADR	33	98.793	(N-term)_iTRAQ[0]	[8] F13-15 and F1+2	279/271	1.005	1.177	1.373	1	Mascot

717	microtubule-associated protein 4 [Rattus norvegicus]	gi|66730360	122031	1	62	1.312	0.951	1.328	0.655	0.079	0.641	2	2	2	99.998

Peptide Information

Calc. Mass	Obsrv. Mass	± da	± ppm	Start Seq.	End Seq.	Sequence	Ion Score	C. I. %	Modification	Plate [#] Name	Gel Idx/Pos [4700 Sample Name]	iTRAQ Ratio 115/114*	iTRAQ Ratio 116/114*	iTRAQ Ratio 117/114*	Rank	Result Type
1774.8878	1774.9127	0.0249	14	846	862	NTTPTGATPPAGMASTR	62	99.998	(N-term)_iTRAQ[0]	[8] F13-15 and F1+2	630/622	0.875	1.031	0.896	1	Mascot
1774.8878	1774.9327	0.0449	25	846	862	NTTPTGATPPAGMASTR	32	98.315	(N-term)_iTRAQ[0]	[8] F13-15 and F1+2	633/625	1.967	0.878	1.970	1	Mascot

718	type I inositol-3,4-bisphosphate 4-phosphatase [Rattus norvegicus]	gi|215272386	113634	2	62	0.849	0.805	0.789	0.039	0.013	0.138	2	2	2	99.998

Peptide Information

Calc. Mass	Obsrv. Mass	± da	± ppm	Start Seq.	End Seq.	Sequence	Ion Score	C. I. %	Modification	Plate [#] Name	Gel Idx/Pos [4700 Sample Name]	iTRAQ Ratio 115/114*	iTRAQ Ratio 116/114*	iTRAQ Ratio 117/114*	Rank	Result Type
1565.9176	1565.9259	0.0083	5	734	746	IPLPGPLFDSLPR	31	98.203	(N-term)_iTRAQ[0]	[8] F13-15 and F1+2	347/339	0.888	0.792	0.927	1	Mascot
1883.1113	1883.1772	0.0659	35	330	342	KLEFVPTNLHIQR	30	97.664	(N-term)_iTRAQ[0], Lysine(K)_iTRAQ[1]	[4] F7 and F10+11	1196/1188	0.812	0.819	0.671	1	Mascot

719	glucose-6-phosphate dehydrogenase [Rattus norvegicus]	gi|204197	59408.6	1	62	0.994	1.208	1.342	0.000	0.000	0.000	1	1	1	99.998

Protein Group

glucose-6-phosphate 1-dehydrogenase [Rattus norvegicus]	gi|8393381	63884.7

Peptide Information

Calc. Mass	Obsrv. Mass	± da	± ppm	Start Seq.	End Seq.	Sequence	Ion Score	C. I. %	Modification	Plate [#] Name	Gel Idx/Pos [4700 Sample Name]	iTRAQ Ratio 115/114*	iTRAQ Ratio 116/114*	iTRAQ Ratio 117/114*	Rank	Result Type
1417.7236	1417.6803	-0.0433	-31	207	217	GGYFDEFGIIR	62	99.998	(N-term)_iTRAQ[0]	[5] F4	293/285	0.994	1.208	1.342	1	Mascot

720	protein S100-B [Rattus norvegicus]	gi|6981498	12125.9	1	61	1.452	1.439	1.416	0.000	0.000	0.000	1	1	1	99.998

Peptide Information

Calc. Mass	Obsrv. Mass	± da	± ppm	Start Seq.	End Seq.	Sequence	Ion Score	C. I. %	Modification	Plate [#] Name	Gel Idx/Pos [4700 Sample Name]	iTRAQ Ratio 115/114*	iTRAQ Ratio 116/114*	iTRAQ Ratio 117/114*	Rank	Result Type
1850.9707	1850.9756	0.0049	3	7	21	AMVALIDVFHQYSGR	61	99.998	(N-term)_iTRAQ[0]	[3] F6 and F9	492/484	1.452	1.439	1.416	1	Mascot

721	contactin-associated protein 1 precursor [Rattus norvegicus]	gi|14091742	161124.9	2	61	1.186	0.836	1.088	0.399	0.331	0.233	2	2	2	99.998

Peptide Information

Calc. Mass	Obsrv. Mass	± da	± ppm	Start Seq.	End Seq.	Sequence	Ion Score	C. I. %	Modification	Plate [#] Name	Gel Idx/Pos [4700 Sample Name]	iTRAQ Ratio 115/114*	iTRAQ Ratio 116/114*	iTRAQ Ratio 117/114*	Rank	Result Type
1480.752	1480.8724	0.1204	81	574	583	GVTCHEPLYK	32	98.477	(N-term)_iTRAQ[0], Lysine(K)_iTRAQ[10], MMTS (C)[4]	[3] F6 and F9	1183/1175	1.585	1.167	1.321	1	Mascot
1952.9739	1952.929	-0.0449	-23	85	100	AVATQGAFNSWDWVTR	29	96.848	(N-term)_iTRAQ[0]	[1] F3 030912	389/381	0.888	0.599	0.896	1	Mascot

722	hypothetical protein LOC299612 [Rattus norvegicus]	gi|56090409	12855.8	1	61	0.685	0.728	1.026	0.000	0.000	0.000	1	1	1	99.998

Peptide Information

Calc. Mass	Obsrv. Mass	± da	± ppm	Start Seq.	End Seq.	Sequence	Ion Score	C. I. %	Modification	Plate [#] Name	Gel Idx/Pos [4700 Sample Name]	iTRAQ Ratio 115/114*	iTRAQ Ratio 116/114*	iTRAQ Ratio 117/114*	Rank	Result Type
2081.9861	2081.8728	-0.1133	-54	52	67	AEDSEVQQEQIYQQSR	61	99.998	(N-term)_iTRAQ[0]	[1] F3 030912	170/162	0.685	0.728	1.026	1	Mascot

723	guanine nucleotide binding protein gamma 3 subunit [Rattus norvegicus]	gi|2582400	7221.6	1	61	2.970	2.231	6.123	0.000	0.000	0.000	1	1	1	99.998

Peptide Information

Calc. Mass	Obsrv. Mass	± da	± ppm	Start Seq.	End Seq.	Sequence	Ion Score	C. I. %	Modification	Plate [#] Name	Gel Idx/Pos [4700 Sample Name]	iTRAQ Ratio 115/114*	iTRAQ Ratio 116/114*	iTRAQ Ratio 117/114*	Rank	Result Type
3484.5437	3484.4485	-0.0952	-27	29	58	AAADLMTYCDAHACEDP LITPVPTSENPFR	61	99.998	(N-term)_iTRAQ[0], MMTS (C)[9,14]	[1] F3 030912	532/524	2.970	2.231	6.123	1	Mascot

724	24-kDa mitochondrial NADH dehydrogenase precursor (EC 1.6.99.3) [Rattus norvegicus]	gi|205628	28516.6	1	60	0.556	0.887	0.855	0.000	0.000	0.000	1	1	1	99.998

Protein Group

NADH dehydrogenase [ubiquinone] flavoprotein 2, mitochondrial precursor [Rattus norvegicus]	gi|51092268	29366

Peptide Information

Calc. Mass	Obsrv. Mass	± da	± ppm	Start Seq.	End Seq.	Sequence	Ion Score	C. I. %	Modification	Plate [#] Name	Gel Idx/Pos [4700 Sample Name]	iTRAQ Ratio 115/114*	iTRAQ Ratio 116/114*	iTRAQ Ratio 117/114*	Rank	Result Type
1388.7394	1388.7069	-0.0325	-23	192	201	DIEEIIDELR	60	99.998	(N-term)_iTRAQ[0]	[1] F3 030912	466/458	0.556	0.887	0.855	1	Mascot

725	CAK beta (cell adhesion kinase beta) [Rattus norvegicus]	gi|1000680	126796.9	1	60	0.974	1.750	1.204	0.000	0.000	0.000	1	1	1	99.997

Protein Group

focal adhesion kinase-related protein [Rattus norvegicus]	gi|3372804	29380.6
protein-tyrosine kinase 2-beta [Rattus norvegicus]	gi|148747528	126854.9

Peptide Information

Calc. Mass	Obsrv. Mass	± da	± ppm	Start Seq.	End Seq.	Sequence	Ion Score	C. I. %	Modification	Plate [#] Name	Gel Idx/Pos [4700 Sample Name]	iTRAQ Ratio 115/114*	iTRAQ Ratio 116/114*	iTRAQ Ratio 117/114*	Rank	Result Type
1648.8428	1648.7854	-0.0574	-35	791	801	EEAQQLWEAEK	60	99.997	(N-term)_iTRAQ[0], Lysine(K)_iTRAQ[11]	[7] F5 120912	203/195	0.974	1.750	1.204	1	Mascot

726	PDGF associated protein [Rattus norvegicus]	gi|1136586	24558.3	1	60	0.649	0.867	1.193	0.000	0.000	0.000	1	1	1	99.997	

Protein Group

28 kDa heat- and acid-stable phosphoprotein [Rattus norvegicus] gi|12018258 24627.4

Peptide Information

Calc. Mass	Obsrv. Mass	± da	± ppm	Start Seq.	End Seq.	Sequence	Ion Score	C. I. %	Modification	Plate [#] Name	Gel Idx/Pos [4700 Sample Name]	iTRAQ Ratio 115/114*	iTRAQ Ratio 116/114*	iTRAQ Ratio 117/114*	Rank	Result Type
2138.0862	2138	-0.0862	-40	16	31	QYTSPEEIDAQLQAEK	60	99.997	(N-term)_iTRAQ[0], Lysine(K)_iTRAQ[16]	[1] F3 030912	275/267	0.649	0.867	1.193	1	Mascot

727	cathepsin D [Rattus norvegicus]	gi|42476045	49042.6	1	60	0.896	0.910	0.527	0.000	0.000	0.000	1	1	1	99.997

Protein Group

RecName: Full=Cathepsin D; Contains: RecName: Full=Cathepsin D 12 kDa light chain; Contains: RecNam gi|115720 49244.7

Peptide Information

Calc. Mass	Obsrv. Mass	± da	± ppm	Start Seq.	End Seq.	Sequence	Ion Score	C. I. %	Modification	Plate [#] Name	Gel Idx/Pos [4700 Sample Name]	iTRAQ Ratio 115/114*	iTRAQ Ratio 116/114*	iTRAQ Ratio 117/114*	Rank	Result Type
1668.8945	1668.8646	-0.0299	-18	112	122	LLDIACWVHHK	60	99.997	(N-term)_iTRAQ[0], Lysine(K)_iTRAQ[11], MMTS (C)[6]	[2] F12 040912	441/433	0.896	0.910	0.527	1	Mascot

728	guanine nucleotide binding protein beta 5 subunit [Rattus norvegicus]	gi|2582396	23456.6	1	59	0.369	0.387	0.651	0.000	0.000	0.000	1	1	1	99.997

Protein Group

guanine nucleotide-binding protein subunit beta-5 isoform 2 [Mus musculus] gi|41281679 42376.5

Peptide Information

Calc. Mass	Obsrv. Mass	± da	± ppm	Start Seq.	End Seq.	Sequence	Ion Score	C. I. %	Modification	Plate [#] Name	Gel Idx/Pos [4700 Sample Name]	iTRAQ Ratio 115/114*	iTRAQ Ratio 116/114*	iTRAQ Ratio 117/114*	Rank	Result Type
1597.8835	1597.8695	-0.014	-9	77	87	VIVWDSFTTNK	59	99.997	(N-term)_iTRAQ[0], Lysine(K)_iTRAQ[11]	[7] F5 120912	322/314	0.369	0.387	0.651	1	Mascot

729	lon protease homolog, mitochondrial precursor [Rattus norvegicus]	gi|19173766	115264.6	2	59	0.845	0.547	1.410	0.067	0.097	0.694	2	2	2	99.997

Peptide Information

Calc. Mass	Obsrv. Mass	± da	± ppm	Start Seq.	End Seq.	Sequence	Ion Score	C. I. %	Modification	Plate [#] Name	Gel Idx/Pos [4700 Sample Name]	iTRAQ Ratio 115/114*	iTRAQ Ratio 116/114*	iTRAQ Ratio 117/114*	Rank	Result Type
1764.9193	1764.8859	-0.0334	-19	450	462	NYLDWLTSIPWGR	32	98.463	(N-term)_iTRAQ[0]	[1] F3 030912	548/540	0.913	0.464	2.104	1	Mascot
2138.0322	2137.9985	-0.0337	-16	473	488	AQSVLEEDHYGMEDVK	27	95.476	(N-term)_iTRAQ[0], Lysine(K)_iTRAQ[16]	[4] F7 and F10+11	254/246	0.783	0.644	0.945	1	Mascot

730	liprin alpha 3 [Rattus norvegicus]	gi|16555340	124356.5	2	59	0.831	0.956	1.122	0.238	0.266	0.342	2	2	2	99.997

Protein Group

RecName: Full=Liprin-alpha-3; AltName: Full=Protein tyrosine phosphatase receptor type f polypeptid gi|42558929 143029.2

Peptide Information

Calc. Mass	Obsrv. Mass	± da	± ppm	Start Seq.	End Seq.	Sequence	Ion Score	C. I. %	Modification	Plate [#] Name	Gel Idx/Pos [4700 Sample Name]	iTRAQ Ratio 115/114*	iTRAQ Ratio 116/114*	iTRAQ Ratio 117/114*	Rank	Result Type
1791.9698	1791.8342	-0.1356	-76	669	680	LIQEEKETTEQR	32	98.582	(N-term)_iTRAQ[0], Lysine(K)_iTRAQ[6]	[6] F8 110912	175/167	0.646	0.748	0.860	1	Mascot
2017.1063	2017.0481	-0.0582	-29	145	158	EQLLEREEEIAELK	28	96.211	(N-term)_iTRAQ[0], Lysine(K)_iTRAQ[14]	[4] F7 and F10+11	344/336	1.068	1.222	1.464	1	Mascot

731	Chain A, Gdp-Bound Rab7	gi|51247925	23384.2	2	59	0.983	0.936	0.732	0.488	0.035	0.106	2	2	2	99.997

Protein Group

ras-related protein Rab-7a [Rattus norvegicus] gi|13027392 26312.6

Peptide Information

Calc. Mass	Obsrv. Mass	± da	± ppm	Start Seq.	End Seq.	Sequence	Ion Score	C. I. %	Modification	Plate [#] Name	Gel Idx/Pos [4700 Sample Name]	iTRAQ Ratio 115/114*	iTRAQ Ratio 116/114*	iTRAQ Ratio 117/114*	Rank	Result Type
1290.6201	1290.6021	-0.018	-14	139	146	AQAWCYSK	29	96.863	(N-term)_iTRAQ[0], Lysine(K)_iTRAQ[8], MMTS (C)[5]	[4] F7 and F10+11	238/230	1.471	0.971	0.838	1	Mascot
1791.9183	1791.8617	-0.0566	-32	56	69	LVTMQIWDTAGQER	30	97.293	(N-term)_iTRAQ[0]	[1] F3 030912	369/361	0.657	0.902	0.640	1	Mascot

732 non muscle caldesmon gi|227429 70794.8 1 58 0.958 0.902 1.058 0.634 0.455 0.943 2 2 2 99.996

Protein Group

non-muscle caldesmon [Rattus norvegicus] gi|6978589 69718.2

Peptide Information

Calc. Mass	Obsrv. Mass	± da	± ppm	Start Seq.	End Seq.	Sequence	Ion Score	C. I. %	Modification	Plate [#] Name	Gel Idx/Pos [4700 Sample Name]	iTRAQ Ratio 115/114*	iTRAQ Ratio 116/114*	iTRAQ Ratio 117/114*	Rank	Result Type
2076.0945	2075.9414	-0.1531	-74	267	283	ASGDKEAEGAPQVEAGK	50	99.976	(N-term)_iTRAQ[0], Lysine(K)_iTRAQ[5,17]	[6] F8 110912	120/112	0.577	0.600	0.559	1	Mascot
2076.0945	2076.137	0.0425	20	267	283	ASGDKEAEGAPQVEAGK	58	99.996	(N-term)_iTRAQ[0], Lysine(K)_iTRAQ[5,17]	[3] F6 and F9	1084/1076	1.592	1.357	2.001	1	Mascot

733 coronin-1A [Rattus norvegicus] gi|18426834 54899.1 1 58 1.121 1.464 0.904 0.000 0.000 0.000 1 1 1 99.996

Peptide Information

Calc. Mass	Obsrv. Mass	± da	± ppm	Start Seq.	End Seq.	Sequence	Ion Score	C. I. %	Modification	Plate [#] Name	Gel Idx/Pos [4700 Sample Name]	iTRAQ Ratio 115/114*	iTRAQ Ratio 116/114*	iTRAQ Ratio 117/114*	Rank	Result Type
1778.8893	1778.8203	-0.069	-39	417	432	ATPEPSSTLSSDTVSR	58	99.996	(N-term)_iTRAQ[0]	[1] F3 030912	167/159	1.121	1.464	0.904	1	Mascot

734 RecName: Full=Neuroligin-3; AltName: Full=Gliotactin homolog; Flags: Precursor gi|31076783 99522.6 1 58 0.928 0.988 0.738 0.000 0.000 0.000 1 1 1 99.996

Peptide Information

Calc. Mass	Obsrv. Mass	± da	± ppm	Start Seq.	End Seq.	Sequence	Ion Score	C. I. %	Modification	Plate [#] Name	Gel Idx/Pos [4700 Sample Name]	iTRAQ Ratio 115/114*	iTRAQ Ratio 116/114*	iTRAQ Ratio 117/114*	Rank	Result Type
1441.7771	1441.6957	-0.0814	-56	359	369	ELVEQDIQPAR	58	99.996	(N-term)_iTRAQ[0]	[1] F3 030912	196/188	0.928	0.988	0.738	1	Mascot

735 NADH dehydrogenase [ubiquinone] 1 alpha subcomplex subunit 5 [Rattus norvegicus] gi|6981260 15610.8 1 58 0.748 0.972 0.955 0.000 0.000 0.000 1 1 1 99.996

Peptide Information

Calc. Mass	Obsrv. Mass	± da	± ppm	Start Seq.	End Seq.	Sequence	Ion Score	C. I. %	Modification	Plate [#] Name	Gel Idx/Pos [4700 Sample Name]	iTRAQ Ratio 115/114*	iTRAQ Ratio 116/114*	iTRAQ Ratio 117/114*	Rank	Result Type
1386.7362	1386.6892	-0.047	-34	47	55	YTEQITSEK	58	99.996	(N-term)_iTRAQ[0], Lysine(K)_iTRAQ[9]	[7] F5 120912	140/132	0.748	0.972	0.955	1	Mascot

736 guanine nucleotide binding protein gamma 2 subunit [Rattus norvegicus] gi|2582398 4560.4 1 58 1.162 1.121 1.056 0.348 0.007 0.010 2 2 2 99.996

Peptide Information

Calc. Mass	Obsrv. Mass	± da	± ppm	Start Seq.	End Seq.	Sequence	Ion Score	C. I. %	Modification	Plate [#] Name	Gel Idx/Pos [4700 Sample Name]	iTRAQ Ratio 115/114*	iTRAQ Ratio 116/114*	iTRAQ Ratio 117/114*	Rank	Result Type
1798.8517	1798.8723	0.0206	11	7	20	AAADLMAYCEAHAK	56	99.994	(N-term)_iTRAQ[0], Lysine(K)_iTRAQ[14], MMTS (C)[9]	[4] F7 and F10+11	425/417	0.895	1.127	1.045	1	Mascot
1798.8517	1798.929	0.0773	43	7	20	AAADLMAYCEAHAK	58	99.996	(N-term)_iTRAQ[0], Lysine(K)_iTRAQ[14], MMTS (C)[9]	[4] F7 and F10+11	1294/1286	1.510	1.114	1.066	1	Mascot

737 Ab2-076 [Rattus norvegicus] gi|33086552 110654.4 1 56 1.148 0.742 1.106 0.000 0.000 0.000 1 1 1 99.994

Protein Group

acetyl-CoA acetyltransferase, cytosolic [Rattus norvegicus] gi|55741502 44620.2

Peptide Information

Calc. Mass	Obsrv. Mass	± da	± ppm	Start Seq.	End Seq.	Sequence	Ion Score	C. I. %	Modification	Plate [#] Name	Gel Idx/Pos [4700 Sample Name]	iTRAQ Ratio 115/114*	iTRAQ Ratio 116/114*	iTRAQ Ratio 117/114*	Rank	Result Type
2800.3862	2800.2952	-0.091	-32	47	71	VAPEEVSEVIFGHVLTAG CGQNPTR	57	99.996	(N-term)_iTRAQ[0], MMTS (C)[19]	[1] F3 030912	515/507	1.148	0.742	1.106	1	Mascot

738	RecName: Full=Estradiol 17-beta-dehydrogenase 12; AltName: Full=17-beta-hydroxysteroid dehydrogenas	gi|81892292	39135.6	1	57	0.888	0.841	0.781	0.000	0.000	0.000	1	1	1	99.996

Peptide Information

Calc. Mass	Obsrv. Mass	± da	± ppm	Start Seq.	End Seq.	Sequence	Ion Score	C. I. %	Modification	Plate [#] Name	Gel Idx/Pos [4700 Sample Name]	iTRAQ Ratio 115/114*	iTRAQ Ratio 116/114*	iTRAQ Ratio 117/114*	Rank	Result Type
1941.1459	1941.1069	-0.039	-20	224	238	GIFVQSVLPFFVATK	57	99.996	(N-term)_iTRAQ[0], Lysine(K)_iTRAQ[15]	[1] F3 030912	534/526	0.888	0.841	0.781	1	Mascot

739	syntaxin-7 [Rattus norvegicus]	gi|55741787	32085.8	1	57	1.490	1.249	1.772	0.000	0.000	0.000	1	1	1	99.995

Protein Group

syntaxin 7 [Rattus norvegicus] gi|3152727 32027.8

Peptide Information

Calc. Mass	Obsrv. Mass	± da	± ppm	Start Seq.	End Seq.	Sequence	Ion Score	C. I. %	Modification	Plate [#] Name	Gel Idx/Pos [4700 Sample Name]	iTRAQ Ratio 115/114*	iTRAQ Ratio 116/114*	iTRAQ Ratio 117/114*	Rank	Result Type
2136.1294	2135.9866	-0.1428	-67	50	64	QQLQQEQQYTNQLAK	57	99.995	(N-term)_iTRAQ[0], Lysine(K)_iTRAQ[15]	[5] F4	128/120	1.490	1.249	1.772	1	Mascot

740	glutathione S-transferase alpha-4 [Rattus norvegicus]	gi|157820217	28277.4	1	57	0.820	1.158	0.968	0.000	0.000	0.000	1	1	1	99.995

Peptide Information

Calc. Mass	Obsrv. Mass	± da	± ppm	Start Seq.	End Seq.	Sequence	Ion Score	C. I. %	Modification	Plate [#] Name	Gel Idx/Pos [4700 Sample Name]	iTRAQ Ratio 115/114*	iTRAQ Ratio 116/114*	iTRAQ Ratio 117/114*	Rank	Result Type
1945.1342	1945.0117	-0.1225	-63	113	126	APQEKEESLALAVK	57	99.995	(N-term)_iTRAQ[0], Lysine(K)_iTRAQ[5,1 4]	[6] F8 110912	247/239	0.820	1.158	0.968	1	Mascot

741	renin receptor [Rattus norvegicus]	gi|347300186	41074.7	1	57	1.215	1.336	0.790	0.000	0.000	0.000	1	1	1	99.995

Peptide Information

Calc. Mass	Obsrv. Mass	± da	± ppm	Start Seq.	End Seq.	Sequence	Ion Score	C. I. %	Modification	Plate [#] Name	Gel Idx/Pos [4700 Sample Name]	iTRAQ Ratio 115/114*	iTRAQ Ratio 116/114*	iTRAQ Ratio 117/114*	Rank	Result Type
1843.9436	1843.8506	-0.093	-50	286	298	QENTQSPYNLAYK	57	99.995	(N-term)_iTRAQ[0], Lysine(K)_iTRAQ[13]	[7] F5 120912	172/164	1.215	1.336	0.790	1	Mascot

742	apoptosis-inducing factor [Rattus norvegicus]	gi|14279176	72118.5	1	57	1.124	1.309	0.741	0.000	0.000	0.000	1	1	1	99.995

Protein Group

apoptosis-inducing factor 1, mitochondrial precursor [Rattus norvegicus] gi|25742626 72674.8

Peptide Information

Calc. Mass	Obsrv. Mass	± da	± ppm	Start Seq.	End Seq.	Sequence	Ion Score	C. I. %	Modification	Plate [#] Name	Gel Idx/Pos [4700 Sample Name]	iTRAQ Ratio 115/114*	iTRAQ Ratio 116/114*	iTRAQ Ratio 117/114*	Rank	Result Type
1771.8708	1771.7441	-0.1267	-72	589	601	DGEQHEDLNEVAK	57	99.995	(N-term)_iTRAQ[0], Lysine(K)_iTRAQ[13]	[6] F8 110912	134/126	1.124	1.309	0.741	1	Mascot

743	dystrobrevin beta [Rattus norvegicus]	gi|58865954	78888.8	1	57	1.022	0.709	0.755	0.000	0.000	0.000	1	1	1	99.995

Peptide Information

Calc. Mass	Obsrv. Mass	± da	± ppm	Start Seq.	End Seq.	Sequence	Ion Score	C. I. %	Modification	Plate [#] Name	Gel Idx/Pos [4700 Sample Name]	iTRAQ Ratio 115/114*	iTRAQ Ratio 116/114*	iTRAQ Ratio 117/114*	Rank	Result Type
1781.928	1781.8309	-0.0971	-54	459	471	LEHEQASQPTPEK	57	99.995	(N-term)_iTRAQ[0], Lysine(K)_iTRAQ[13]	[6] F8 110912	112/104	1.022	0.709	0.755	1	Mascot

744	fatty acid-binding protein, brain [Rattus norvegicus]	gi|13540630	16957.7	1	56	1.442	1.062	0.973	0.000	0.000	0.000	1	1	1	99.994

Peptide Information

Calc. Mass	Obsrv. Mass	± da	± ppm	Start Seq.	End Seq.	Sequence	Ion Score	C. I. %	Modification	Plate [#] Name	Gel Idx/Pos [4700 Sample Name]	iTRAQ Ratio 115/114*	iTRAQ Ratio 116/114*	iTRAQ Ratio 117/114*	Rank	Result Type
2287.3357	2287.3047	-0.031	-14	32	49	QVGNVTKPTVIISQEGGK	56	99.994	(N-term)_iTRAQ[0], Lysine(K)_iTRAQ[7,1	[4] F7 and F10+11	230/222	1.442	1.062	0.973	1	Mascot

8]

745	palmitoyl-protein thioesterase 1 precursor [Rattus norvegicus]	gi|11968070	38016.5	2	56	1.374	1.176	1.071	0.260	0.063	0.576	2	2	2	99.994

Peptide Information

Calc. Mass	Obsrv. Mass	± da	± ppm	Start Seq.	End Seq.	Sequence	Ion Score	C. I. %	Modification	Plate [#] Name	Gel Idx/Pos [4700 Sample Name]	iTRAQ Ratio 115/114*	iTRAQ Ratio 116/114*	iTRAQ Ratio 117/114*	Rank	Result Type
1952.9937	1953.0271	0.0334	17	254	268	ETIPLQETTLYTEDR	27	95.05	(N-term)_iTRAQ[0]	[8] F13-15 and F1+2	482/474	1.634	1.239	1.648	1	Mascot
2137.1062	2137.0762	-0.03	-14	105	122	LQHGYNAIGFSQGGQFLR	29	97.204	(N-term)_iTRAQ[0]	[4] F7 and F10+11	341/333	1.156	1.116	0.697	1	Mascot

746	RecName: Full=Synaptic vesicle 2-related protein; Short=SV2-related protein	gi|81907791	64100.2	1	56	1.614	2.332	3.025	0.000	0.000	0.000	1	1	1	99.994

Peptide Information

Calc. Mass	Obsrv. Mass	± da	± ppm	Start Seq.	End Seq.	Sequence	Ion Score	C. I. %	Modification	Plate [#] Name	Gel Idx/Pos [4700 Sample Name]	iTRAQ Ratio 115/114*	iTRAQ Ratio 116/114*	iTRAQ Ratio 117/114*	Rank	Result Type
2057.9495	2057.8823	-0.0672	-33	25	42	SEDDAASGEHDVQIEGVR	56	99.994	(N-term)_iTRAQ[0]	[3] F6 and F9	182/174	1.614	2.332	3.025	1	Mascot

747	large neutral amino acids transporter small subunit 1 [Rattus norvegicus]	gi|8394405	59300.6	1	56	1.010	0.809	0.793	0.002	0.470	0.019	2	2	2	99.994

Peptide Information

Calc. Mass	Obsrv. Mass	± da	± ppm	Start Seq.	End Seq.	Sequence	Ion Score	C. I. %	Modification	Plate [#] Name	Gel Idx/Pos [4700 Sample Name]	iTRAQ Ratio 115/114*	iTRAQ Ratio 116/114*	iTRAQ Ratio 117/114*	Rank	Result Type
1670.8707	1670.7739	-0.0968	-58	223	235	DIGQGDASNLHQK	56	99.994	(N-term)_iTRAQ[0], Lysine(K)_iTRAQ[13]	[6] F8 110912	117/109	1.012	0.512	0.813	1	Mascot
1670.8707	1670.9082	0.0375	22	223	235	DIGQGDASNLHQK	42	99.849	(N-term)_iTRAQ[0], Lysine(K)_iTRAQ[13]	[3] F6 and F9	1084/1076	1.008	1.279	0.774	1	Mascot

748	Prdx3 protein [Rattus norvegicus]	gi|38051985	29388.2	1	56	0.892	1.164	1.034	0.000	0.000	0.000	1	1	1	99.994

Protein Group

thioredoxin-dependent peroxide reductase, mitochondrial precursor [Rattus norvegicus]	gi|11968132	30550.8

Peptide Information

Calc. Mass	Obsrv. Mass	± da	± ppm	Start Seq.	End Seq.	Sequence	Ion Score	C. I. %	Modification	Plate [#] Name	Gel Idx/Pos [4700 Sample Name]	iTRAQ Ratio 115/114*	iTRAQ Ratio 116/114*	iTRAQ Ratio 117/114*	Rank	Result Type
1620.9081	1620.9279	0.0198	12	172	185	DYGVLLESAGIALR	56	99.994	(N-term)_iTRAQ[0]	[8] F13-15 and F1+2	382/374	0.892	1.164	1.034	1	Mascot

749	dual specificity mitogen-activated protein kinase kinase 6 [Rattus norvegicus]	gi|16758520	41875.1	2	56	1.378	1.471	0.993	0.288	0.569	0.012	2	2	2	99.993

Peptide Information

Calc. Mass	Obsrv. Mass	± da	± ppm	Start Seq.	End Seq.	Sequence	Ion Score	C. I. %	Modification	Plate [#] Name	Gel Idx/Pos [4700 Sample Name]	iTRAQ Ratio 115/114*	iTRAQ Ratio 116/114*	iTRAQ Ratio 117/114*	Rank	Result Type
1718.847	1718.7518	-0.0952	-55	18	31	EAFEQPQTSSTPPR	27	95.507	(N-term)_iTRAQ[0]	[1] F3 030912	161/153	1.140	1.061	0.981	1	Mascot
2157.2979	2157.2874	-0.0105	-5	179	194	DVKPSNVLINTLGQVK	28	96.381	(N-term)_iTRAQ[0], Lysine(K)_iTRAQ[3,16]	[4] F7 and F10+11	359/351	1.666	2.040	1.004	1	Mascot

750	keratin K6 [Rattus norvegicus]	gi|554464	3537.9	1	56	0.911	0.844	1.729	0.000	0.000	0.000	1	1	1	99.993

Peptide Information

Calc. Mass	Obsrv. Mass	± da	± ppm	Start Seq.	End Seq.	Sequence	Ion Score	C. I. %	Modification	Plate [#] Name	Gel Idx/Pos [4700 Sample Name]	iTRAQ Ratio 115/114*	iTRAQ Ratio 116/114*	iTRAQ Ratio 117/114*	Rank	Result Type
1323.7029	1323.6447	-0.0582	-44	5	14	YEELQITAGR	56	99.993	(N-term)_iTRAQ[0]	[1] F3 030912	224/216	0.911	0.844	1.729	1	Mascot

751	CD82 antigen [Rattus norvegicus]	gi|162287200	32254.3	1	55	2.398	0.854	1.103	0.000	0.000	0.000	1	1	1	99.993

Protein Group

RecName: Full=CD82 antigen; AltName: Full=Metastasis suppressor Kangai-1 homolog; AltName: CD_antig	gi|9296930	32325.3

Peptide Information

Calc. Mass	Obsrv. Mass	± da	± ppm	Start Seq.	End Seq.	Sequence	Ion Score	C. I. %	Modification	Plate [#] Name	Gel Idx/Pos [4700 Sample Name]	iTRAQ Ratio	iTRAQ Ratio	iTRAQ Ratio	Rank	Result Type

												115/114*	116/114*	117/114*		
1753.9006	1753.8325	-0.0681	-39	137	148	EEAWDYVQAQVK	55	99.993	(N-term)_iTRAQ[0], Lysine(K)_iTRAQ[12]	[5] F4	215/207	2.398	0.854	1.103	1	Mascot

752	Acyl-Coenzyme A dehydrogenase, very long chain [Rattus norvegicus]	gi|56541110	77104.9	1	55	1.429	1.691	1.238	0.000	0.000	0.000	1	1	1	99.992

Protein Group

very long-chain specific acyl-CoA dehydrogenase, mitochondrial precursor [Rattus norvegicus]	gi|6978435	77032.9

Peptide Information

Calc. Mass	Obsrv. Mass	± da	± ppm	Start Seq.	End Seq.	Sequence	Ion Score	C. I. %	Modification	Plate [#] Name	Gel Idx/Pos [4700 Sample Name]	iTRAQ Ratio 115/114*	iTRAQ Ratio 116/114*	iTRAQ Ratio 117/114*	Rank	Result Type
1704.869	1704.7959	-0.0731	-43	304	316	ASNTSEVYFDGVK	55	99.992	(N-term)_iTRAQ[0], Lysine(K)_iTRAQ[13]	[7] F5 120912	187/179	1.429	1.691	1.238	1	Mascot

753	p62 dynactin subunit [Rattus norvegicus]	gi|6090975	57829.9	1	55	1.066	1.199	0.781	0.000	0.000	0.000	1	1	1	99.992

Protein Group

dynactin subunit 4 [Rattus norvegicus]	gi|16758134	58644.3

Peptide Information

Calc. Mass	Obsrv. Mass	± da	± ppm	Start Seq.	End Seq.	Sequence	Ion Score	C. I. %	Modification	Plate [#] Name	Gel Idx/Pos [4700 Sample Name]	iTRAQ Ratio 115/114*	iTRAQ Ratio 116/114*	iTRAQ Ratio 117/114*	Rank	Result Type
1993.9601	1993.9407	-0.0194	-10	127	143	SVASGGWQEPENPHAQR	55	99.992	(N-term)_iTRAQ[0]	[4] F7 and F10+11	169/161	1.066	1.199	0.781	1	Mascot

754	mitochondrial chaperone BCS1 [Rattus norvegicus]	gi|56090628	49518.7	1	55	0.552	1.246	0.991	0.000	0.000	0.000	1	1	1	99.991

Peptide Information

Calc. Mass	Obsrv. Mass	± da	± ppm	Start Seq.	End Seq.	Sequence	Ion Score	C. I. %	Modification	Plate [#] Name	Gel Idx/Pos [4700 Sample Name]	iTRAQ Ratio 115/114*	iTRAQ Ratio 116/114*	iTRAQ Ratio 117/114*	Rank	Result Type
1653.9574	1653.9683	0.0109	7	145	155	KVFFNILEEAR	55	99.991	(N-term)_iTRAQ[0], Lysine(K)_iTRAQ[1]	[3] F6 and F9	1341/1333	0.552	1.246	0.991	1	Mascot

755	cytochrome oxidase III [Rattus norvegicus]	gi|829025	30432.5	1	54	1.096	1.411	0.743	0.332	0.387	0.545	2	2	2	99.991

Protein Group

cytochrome c oxidase subunit III [Rattus norvegicus]	gi|110189669	30519.5
cytochrome c oxidase subunit III [Rattus sp.]	gi|5705944	9352
cytochrome oxidase subunit III [Rattus norvegicus]	gi|19577320	30518.6
cytochrome oxidase subunit III [Rattus norvegicus]	gi|20805884	30502.5

Peptide Information

Calc. Mass	Obsrv. Mass	± da	± ppm	Start Seq.	End Seq.	Sequence	Ion Score	C. I. %	Modification	Plate [#] Name	Gel Idx/Pos [4700 Sample Name]	iTRAQ Ratio 115/114*	iTRAQ Ratio 116/114*	iTRAQ Ratio 117/114*	Rank	Result Type
1883.0021	1882.912	-0.0901	-48	64	77	EGTYQGHHTPIVQK	54	99.991	(N-term)_iTRAQ[0], Lysine(K)_iTRAQ[14]	[2] F12 040912	144/136	1.428	1.799	1.288	1	Mascot
1883.0021	1883.0947	0.0926	49	64	77	EGTYQGHHTPIVQK	30	97.559	(N-term)_iTRAQ[0], Lysine(K)_iTRAQ[14]	[4] F7 and F10+11	1038/1030	0.842	1.107	0.429	1	Mascot

756	60S ribosomal protein L8 [Homo sapiens]	gi|4506663	32082	1	54	0.970	0.919	1.138	0.000	0.000	0.000	1	1	1	99.99

Peptide Information

Calc. Mass	Obsrv. Mass	± da	± ppm	Start Seq.	End Seq.	Sequence	Ion Score	C. I. %	Modification	Plate [#] Name	Gel Idx/Pos [4700 Sample Name]	iTRAQ Ratio 115/114*	iTRAQ Ratio 116/114*	iTRAQ Ratio 117/114*	Rank	Result Type
1977.0287	1976.9645	-0.0642	-32	129	144	ASGNYATVISHNPETK	54	99.99	(N-term)_iTRAQ[0], Lysine(K)_iTRAQ[16]	[4] F7 and F10+11	189/181	0.970	0.919	1.138	1	Mascot

757	RecName: Full=Non-specific lipid-transfer protein; Short=NSL-TP; AltName: Full=Propanoyl-CoA C-acyl	gi|128391	66002	1	54	1.330	1.126	0.649	0.000	0.000	0.000	1	1	1	99.99

Protein Group

60 kDa protein [Rattus norvegicus]	gi|202553	65976
non-specific lipid transfer protein [Rattus norvegicus]	gi|56772	18215.9
non-specific lipid transfer protein [Rattus sp.]	gi|205770	62330
non-specific lipid-transfer protein [Rattus norvegicus]	gi|50356003	66016
sterol carrier protein-2 (SCP-2) [Rattus norvegicus]	gi|206872	33076.4
unnamed protein product [Rattus norvegicus]	gi|56773	65930

Peptide Information

Calc. Mass	Obsrv. Mass	± da	± ppm	Start Seq.	End Seq.	Sequence	Ion Score	C. I. %	Modification	Plate [#] Name	Gel Idx/Pos [4700 Sample Name]	iTRAQ Ratio 115/114*	iTRAQ Ratio 116/114*	iTRAQ Ratio 117/114*	Rank	Result Type
1542.7985	1542.751	-0.0475	-31	512	522	MNPQSAFFQGK	54	99.99	(N-term)_iTRAQ[0], Lysine(K)_iTRAQ[11]	[7] F5 120912	227/219	1.330	1.126	0.649	1	Mascot

758	NADH dehydrogenase [ubiquinone] flavoprotein 3, mitochondrial isoform 2 [Rattus norvegicus]	gi|155369704	13231.2	1	53	0.875	1.548	1.193	0.000	0.000	0.000	1	1	1	99.989

Peptide Information

Calc. Mass	Obsrv. Mass	± da	± ppm	Start Seq.	End Seq.	Sequence	Ion Score	C. I. %	Modification	Plate [#] Name	Gel Idx/Pos [4700 Sample Name]	iTRAQ Ratio 115/114*	iTRAQ Ratio 116/114*	iTRAQ Ratio 117/114*	Rank	Result Type
1956.006	1955.9258	-0.0802	-41	58	72	EPTLVPESSDTTTYK	53	99.989	(N-term)_iTRAQ[0], Lysine(K)_iTRAQ[15]	[1] F3 030912	217/209	0.875	1.548	1.193	1	Mascot

759	RecName: Full=Rho GTPase-activating protein 35; AltName: Full=GAP-associated protein p190; AltName:	gi|29611706	189213.1	1	53	1.238	1.520	0.815	0.000	0.000	0.000	1	1	1	99.989

Peptide Information

Calc. Mass	Obsrv. Mass	± da	± ppm	Start Seq.	End Seq.	Sequence	Ion Score	C. I. %	Modification	Plate [#] Name	Gel Idx/Pos [4700 Sample Name]	iTRAQ Ratio 115/114*	iTRAQ Ratio 116/114*	iTRAQ Ratio 117/114*	Rank	Result Type
1465.8453	1465.8735	0.0282	19	931	939	LELFHPFFK	53	99.989	(N-term)_iTRAQ[0], Lysine(K)_iTRAQ[9]	[3] F6 and F9	1365/1357	1.238	1.520	0.815	1	Mascot

760	Chain A, Crystal Structure Analysis Of Rat Enoyl-Coa Hydratase In Complex With Hexadienoyl-Coa	gi|24159081	31756.7	1	53	0.955	0.793	0.747	0.000	0.000	0.000	1	1	1	99.987

Protein Group

Chain A, 2-Enoyl-Coa Hydratase, Data Collected At 100 K, Ph 6.5	gi|2392291	31813.8
Chain A, Structure Of Enoyl-Coa Hydratase Complexed With The Substrate Dac-Coa	gi|20149805	31571.7
enoyl-CoA hydratase, mitochondrial precursor [Rattus norvegicus]	gi|17530977	35132.4

Peptide Information

Calc. Mass	Obsrv. Mass	± da	± ppm	Start Seq.	End Seq.	Sequence	Ion Score	C. I. %	Modification	Plate [#] Name	Gel Idx/Pos [4700 Sample Name]	iTRAQ Ratio 115/114*	iTRAQ Ratio 116/114*	iTRAQ Ratio 117/114*	Rank	Result Type
1151.667	1151.6342	-0.0328	-28	63	71	AFAAGADIK	53	99.987	(N-term)_iTRAQ[0], Lysine(K)_iTRAQ[9]	[7] F5 120912	157/149	0.955	0.793	0.747	1	Mascot

761	matrin 3 - rat	gi|111944	105580.3	1	53	0.798	0.985	0.947	0.000	0.000	0.000	1	1	1	99.986

Protein Group

Matr3 protein [Rattus norvegicus]	gi|38328275	48392.5
RecName: Full=Matrin-3; AltName: Full=Nuclear scaffold protein p130/MAT3	gi|12643398	104457.4
matrin-3 [Rattus norvegicus]	gi|61889073	104512.5

Peptide Information

Calc. Mass	Obsrv. Mass	± da	± ppm	Start Seq.	End Seq.	Sequence	Ion Score	C. I. %	Modification	Plate [#] Name	Gel Idx/Pos [4700 Sample Name]	iTRAQ Ratio 115/114*	iTRAQ Ratio 116/114*	iTRAQ Ratio 117/114*	Rank	Result Type
1908.178	1908.118	-0.06	-31	133	146	ITPENLPQILLQLK	53	99.986	(N-term)_iTRAQ[0], Lysine(K)_iTRAQ[14]	[1] F3 030912	525/517	0.798	0.985	0.947	1	Mascot

762	mercurial-insensitive water channel [Rattus norvegicus]	gi|459951	34395	1	53	3.197	2.642	1.950	0.000	0.000	0.000	1	1	1	99.986

Protein Group

aquaporin 4.M23 [Rattus norvegicus]	gi|23955260	5528
aquaporin-4 isoform 1 [Rattus norvegicus]	gi|6978531	36987.2

Peptide Information

Calc. Mass	Obsrv. Mass	± da	± ppm	Start Seq.	End Seq.	Sequence	Ion Score	C. I. %	Modification	Plate [#] Name	Gel Idx/Pos [4700 Sample Name]	iTRAQ Ratio 115/114*	iTRAQ Ratio 116/114*	iTRAQ Ratio 117/114*	Rank	Result Type
1410.778	1410.7618	-0.0162	-11	6	14	GVWTQAFWK	53	99.986	(N-term)_iTRAQ[0], Lysine(K)_iTRAQ[9]	[3] F6 and F9	382/374	3.197	2.642	1.950	1	Mascot

763	RecName: Full=Sideroflexin-1; AltName: Full=Tricarboxylate carrier protein; Short=TCC	gi|20139869	37914.7	1	52	0.635	0.767	0.639	0.000	0.000	0.000	1	1	1	99.985

Protein Group

sideroflexin-1 [Rattus norvegicus]	gi|58865994	37812.6
tricarboxylate carrier [Rattus sp.]	gi|545998	40927.2

Peptide Information

Calc. Mass	Obsrv. Mass	± da	± ppm	Start Seq.	End Seq.	Sequence	Ion Score	C. I. %	Modification	Plate [#] Name	Gel Idx/Pos [4700 Sample Name]	iTRAQ Ratio 115/114*	iTRAQ Ratio 116/114*	iTRAQ Ratio 117/114*	Rank	Result Type
1253.6399	1253.5599	-0.08	-64	16	24	WDQSTFIGR	52	99.985	(N-term)_iTRAQ[0]	[5] F4	185/177	0.635	0.767	0.639	1	Mascot

764	prolyl endopeptidase [Rattus norvegicus]	gi|13786144	88244.5	1	52	1.086	1.024	1.017	0.000	0.000	0.000	1	1	1	99.985

Peptide Information

Calc. Mass	Obsrv. Mass	± da	± ppm	Start Seq.	End Seq.	Sequence	Ion Score	C. I. %	Modification	Plate [#] Name	Gel Idx/Pos [4700 Sample Name]	iTRAQ Ratio 115/114*	iTRAQ Ratio 116/114*	iTRAQ Ratio 117/114*	Rank	Result Type
1822.868	1822.7853	-0.0827	-45	184	196	GMFYNSYPQQDGK	52	99.985	(N-term)_iTRAQ[0], Lysine(K)_iTRAQ[13]	[7] F5 120912	188/180	1.086	1.024	1.017	1	Mascot

765	ras-related protein Rab-18 [Rattus norvegicus]	gi|60223069	25497.2	1	51	1.539	1.775	1.443	0.000	0.000	0.000	1	1	1	99.982

Peptide Information

Calc. Mass	Obsrv. Mass	± da	± ppm	Start Seq.	End Seq.	Sequence	Ion Score	C. I. %	Modification	Plate [#] Name	Gel Idx/Pos [4700 Sample Name]	iTRAQ Ratio 115/114*	iTRAQ Ratio 116/114*	iTRAQ Ratio 117/114*	Rank	Result Type
1403.7404	1403.6814	-0.059	-42	59	69	LAIWDTAGQER	51	99.982	(N-term)_iTRAQ[0]	[5] F4	209/201	1.539	1.775	1.443	1	Mascot

766	nitrilase homolog 1 isoform a [Rattus norvegicus]	gi|128485833	37964.9	1	51	1.022	1.037	1.139	0.000	0.000	0.000	1	1	1	99.982

Protein Group

RecName: Full=Nitrilase homolog 1	gi|51702765	33872.7

Peptide Information

Calc. Mass	Obsrv. Mass	± da	± ppm	Start Seq.	End Seq.	Sequence	Ion Score	C. I. %	Modification	Plate [#] Name	Gel Idx/Pos [4700 Sample Name]	iTRAQ Ratio 115/114*	iTRAQ Ratio 116/114*	iTRAQ Ratio 117/114*	Rank	Result Type
1444.7855	1444.7292	-0.0563	-39	297	306	IDLHFLQQMR	51	99.982	(N-term)_iTRAQ[0]	[6] F8 110912	388/380	1.022	1.037	1.139	1	Mascot

767	probable G-protein coupled receptor 37 precursor [Rattus norvegicus]	gi|17105356	71375.6	1	51	1.295	0.753	0.646	0.000	0.000	0.000	1	1	1	99.981

Peptide Information

Calc. Mass	Obsrv. Mass	± da	± ppm	Start Seq.	End Seq.	Sequence	Ion Score	C. I. %	Modification	Plate [#] Name	Gel Idx/Pos [4700 Sample Name]	iTRAQ Ratio 115/114*	iTRAQ Ratio 116/114*	iTRAQ Ratio 117/114*	Rank	Result Type

1520.7465	1520.6603	-0.0862	-57	396	408	EDLGFSGQAPAER	51	99.981	(N-term)_iTRAQ[0]	[1] F3 030912	196/188	1.295	0.753	0.646	1	Mascot			

768	cytochrome b-c1 complex subunit 8 [Rattus norvegicus]	gi|68341999	10707.8	1	51	0.967	0.823	0.978	0.000	0.000	0.000	1	1	1	99.98

Peptide Information

Calc. Mass	Obsrv. Mass	± da	± ppm	Start Seq.	End Seq.	Sequence	Ion Score	C. I. %	Modification	Plate [#] Name	Gel Idx/Pos [4700 Sample Name]	iTRAQ Ratio 115/114*	iTRAQ Ratio 116/114*	iTRAQ Ratio 117/114*	Rank	Result Type
1284.6986	1284.7633	0.0647	50	26	33	AFPHYFSK	51	99.98	(N-term)_iTRAQ[0], Lysine(K)_iTRAQ[8]	[4] F7 and F10+11	1135/1127	0.967	0.823	0.978	1	Mascot

769	triose-phosphate isomerase (EC 5.3.1.1) - rat (fragment)	gi|628038	2107	1	51	1.380	1.380	1.394	0.000	0.000	0.000	1	1	1	99.98

Peptide Information

Calc. Mass	Obsrv. Mass	± da	± ppm	Start Seq.	End Seq.	Sequence	Ion Score	C. I. %	Modification	Plate [#] Name	Gel Idx/Pos [4700 Sample Name]	iTRAQ Ratio 115/114*	iTRAQ Ratio 116/114*	iTRAQ Ratio 117/114*	Rank	Result Type
1242.6881	1242.6777	-0.0104	-8	6	13	FFVGGNXK	51	99.98	(N-term)_iTRAQ[0], Lysine(K)_iTRAQ[8]	[3] F6 and F9	327/319	1.380	1.380	1.394	1	Mascot

770	proteasome subunit alpha type-2 [Rattus norvegicus]	gi|8394063	28019.7	1	50	1.849	1.650	1.669	0.000	0.000	0.000	1	1	1	99.978

Peptide Information

Calc. Mass	Obsrv. Mass	± da	± ppm	Start Seq.	End Seq.	Sequence	Ion Score	C. I. %	Modification	Plate [#] Name	Gel Idx/Pos [4700 Sample Name]	iTRAQ Ratio 115/114*	iTRAQ Ratio 116/114*	iTRAQ Ratio 117/114*	Rank	Result Type
1708.8601	1708.8256	-0.0345	-20	71	84	HIGLVYSGMGPDYR	50	99.978	(N-term)_iTRAQ[0]	[4] F7 and F10+11	280/272	1.849	1.650	1.669	1	Mascot

771	ribosomal protein S17 [Rattus norvegicus]	gi|206745	17562.8	1	50	1.851	1.341	1.969	0.000	0.000	0.000	1	1	1	99.978

Protein Group

40S ribosomal protein S17 [Rattus norvegicus]	gi|297515469	17419.6

Peptide Information

Calc. Mass	Obsrv. Mass	± da	± ppm	Start Seq.	End Seq.	Sequence	Ion Score	C. I. %	Modification	Plate [#] Name	Gel Idx/Pos [4700 Sample Name]	iTRAQ Ratio 115/114*	iTRAQ Ratio 116/114*	iTRAQ Ratio 117/114*	Rank	Result Type
1420.8232	1420.8638	0.0406	29	50	59	IAGYVTHLMK	50	99.978	(N-term)_iTRAQ[0], Lysine(K)_iTRAQ[10]	[3] F6 and F9	1287/1279	1.851	1.341	1.969	1	Mascot

772	ATP synthase subunit e, mitochondrial [Rattus norvegicus]	gi|17978459	9402.3	1	50	1.063	1.067	1.095	0.000	0.000	0.000	1	1	1	99.977

Peptide Information

Calc. Mass	Obsrv. Mass	± da	± ppm	Start Seq.	End Seq.	Sequence	Ion Score	C. I. %	Modification	Plate [#] Name	Gel Idx/Pos [4700 Sample Name]	iTRAQ Ratio 115/114*	iTRAQ Ratio 116/114*	iTRAQ Ratio 117/114*	Rank	Result Type
1638.8837	1638.7949	-0.0888	-54	60	71	ELAEAEDVSIFK	50	99.977	(N-term)_iTRAQ[0], Lysine(K)_iTRAQ[12]	[5] F4	238/230	1.063	1.067	1.095	1	Mascot

773	glia maturation factor beta [Rattus norvegicus]	gi|13624295	18880.9	1	50	0.759	0.977	1.082	0.000	0.000	0.000	1	1	1	99.977

Peptide Information

Calc. Mass	Obsrv. Mass	± da	± ppm	Start Seq.	End Seq.	Sequence	Ion Score	C. I. %	Modification	Plate [#] Name	Gel Idx/Pos [4700 Sample Name]	iTRAQ Ratio 115/114*	iTRAQ Ratio 116/114*	iTRAQ Ratio 117/114*	Rank	Result Type
1549.7618	1549.703	-0.0588	-38	125	135	NTEDLTEEWLR	50	99.977	(N-term)_iTRAQ[0]	[1] F3 030912	320/312	0.759	0.977	1.082	1	Mascot

774	myeloid-associated differentiation marker [Rattus norvegicus]	gi|34328542	36155.2	1	50	0.452	0.678	0.535	0.000	0.000	0.000	1	1	1	99.977

Peptide Information

Calc. Mass	Obsrv. Mass	± da	± ppm	Start Seq.	End Seq.	Sequence	Ion Score	C. I. %	Modification	Plate [#] Name	Gel Idx/Pos [4700 Sample Name]	iTRAQ Ratio 115/114*	iTRAQ Ratio 116/114*	iTRAQ Ratio 117/114*	Rank	Result Type
1799.7946	1799.7327	-0.0619	-34	276	288	SYVQPNEVCNWDR	50	99.977	(N-term)_iTRAQ[0], MMTS (C)[9]	[1] F3 030912	327/319	0.452	0.678	0.535	1	Mascot

775	3'(2'),5'-bisphosphate nucleotidase 1 [Rattus norvegicus]	gi|25282455	36455.1	1	50	1.350	0.840	1.037	0.000	0.000	0.000	1	1	1	99.976

Peptide Information

Calc. Mass	Obsrv. Mass	± da	± ppm	Start Seq.	End Seq.	Sequence	Ion Score	C. I. %	Modification	Plate [#] Name	Gel Idx/Pos [4700 Sample Name]	iTRAQ Ratio 115/114*	iTRAQ Ratio 116/114*	iTRAQ Ratio 117/114*	Rank	Result Type

Calc. Mass	Obsrv. Mass	± da	± ppm	Start Seq.	End Seq.	Sequence	Ion Score	C. I. %	Modification	Plate [#] Name	Gel Idx/Pos [4700 Sample Name]	iTRAQ Ratio 115/114*	iTRAQ Ratio 116/114*	iTRAQ Ratio 117/114*	Rank	Result Type
2302.2405	2302.3062	0.0657	29	245	261	KWDTCAPEVILHAVGGK	51	99.981	(N-term)_iTRAQ[0], Lysine(K)_iTRAQ[1,17], MMTS (C)[5]	[4] F7 and F10+11	1306/1298	1.350	0.840	1.037	1	Mascot

776 amyloid beta A4 protein [Rattus norvegicus] gi|27436861 93673.2 1 49 1.634 1.626 2.068 0.000 0.000 0.000 1 1 1 99.967

Protein Group

App protein [Rattus norvegicus]	gi|38303889	89480.9
unnamed protein product [Rattus rattus]	gi|55617	84749.4

Peptide Information

Calc. Mass	Obsrv. Mass	± da	± ppm	Start Seq.	End Seq.	Sequence	Ion Score	C. I. %	Modification	Plate [#] Name	Gel Idx/Pos [4700 Sample Name]	iTRAQ Ratio 115/114*	iTRAQ Ratio 116/114*	iTRAQ Ratio 117/114*	Rank	Result Type
2203.0667	2203.1177	0.051	23	378	393	YLETPGDENEHAHFQK	50	99.974	(N-term)_iTRAQ[0], Lysine(K)_iTRAQ[16]	[4] F7 and F10+11	1100/1092	1.634	1.626	2.068	1	Mascot

777 ion channel [Rattus norvegicus] gi|7415517 92642.4 1 50 1.354 3.773 3.415 0.000 0.000 0.000 1 1 1 99.974

Protein Group

RecName: Full=Transient receptor potential cation channel subfamily V member 2; Short=TrpV2; AltNam	gi|62901124	92580.3
transient receptor potential cation channel subfamily V member 2 [Rattus norvegicus]	gi|78187981	92604.3
vanilloid receptor-like protein 1 [Rattus norvegicus]	gi|4589143	92564.3

Peptide Information

Calc. Mass	Obsrv. Mass	± da	± ppm	Start Seq.	End Seq.	Sequence	Ion Score	C. I. %	Modification	Plate [#] Name	Gel Idx/Pos [4700 Sample Name]	iTRAQ Ratio 115/114*	iTRAQ Ratio 116/114*	iTRAQ Ratio 117/114*	Rank	Result Type
1994.9399	1994.7957	-0.1442	-72	12	27	LETSDGDEEGNAEVNK	50	99.974	(N-term)_iTRAQ[0], Lysine(K)_iTRAQ[16]	[5] F4	84/76	1.354	3.773	3.415	1	Mascot

778 exocyst complex component 7 [Rattus norvegicus] gi|12083643 83108.4 1 50 0.895 1.078 0.787 0.000 0.000 0.000 1 1 1 99.974

Peptide Information

Calc. Mass	Obsrv. Mass	± da	± ppm	Start Seq.	End Seq.	Sequence	Ion Score	C. I. %	Modification	Plate [#] Name	Gel Idx/Pos [4700 Sample Name]	iTRAQ Ratio 115/114*	iTRAQ Ratio 116/114*	iTRAQ Ratio 117/114*	Rank	Result Type
1330.6876	1330.6212	-0.0664	-50	590	599	AWAIPDTEQR	50	99.974	(N-term)_iTRAQ[0]	[5] F4	157/149	0.895	1.078	0.787	1	Mascot

779 JWA protein [Rattus norvegicus] gi|7331276 22970.3 1 50 1.256 1.205 0.752 0.044 0.190 0.095 2 2 2 99.974

Protein Group

PRA1 family protein 3 [Rattus norvegicus]	gi|13027426	23263.6

Peptide Information

Calc. Mass	Obsrv. Mass	± da	± ppm	Start Seq.	End Seq.	Sequence	Ion Score	C. I. %	Modification	Plate [#] Name	Gel Idx/Pos [4700 Sample Name]	iTRAQ Ratio 115/114*	iTRAQ Ratio 116/114*	iTRAQ Ratio 117/114*	Rank	Result Type
1456.6617	1456.6154	-0.0463	-32	10	20	AWDDFFPGSDR	50	99.974	(N-term)_iTRAQ[0]	[5] F4	276/268	1.214	1.041	0.847	1	Mascot
1456.6617	1456.636	-0.0257	-18	10	20	AWDDFFPGSDR	44	99.907	(N-term)_iTRAQ[0]	[7] F5 120912	345/337	1.301	1.395	0.668	1	Mascot

780 60S acidic ribosomal protein P2 [Rattus norvegicus] gi|71795613 13270 1 49 1.646 0.750 1.374 0.000 0.000 0.000 1 1 1 99.971

Peptide Information

Calc. Mass	Obsrv. Mass	± da	± ppm	Start Seq.	End Seq.	Sequence	Ion Score	C. I. %	Modification	Plate [#] Name	Gel Idx/Pos [4700 Sample Name]	iTRAQ Ratio 115/114*	iTRAQ Ratio 116/114*	iTRAQ Ratio 117/114*	Rank	Result Type
2184.1909	2184.1206	-0.0703	-32	3	21	YVASYLLAALGGNSNPSAK	49	99.971	(N-term)_iTRAQ[0], Lysine(K)_iTRAQ[19]	[1] F3 030912	489/481	1.646	0.750	1.374	1	Mascot

781 cytoplasmic dynein 1 intermediate chain 1 [Rattus norvegicus] gi|11276091 78310.1 1 49 1.310 0.561 0.731 0.000 0.000 0.000 1 1 1 99.968

Peptide Information

Calc. Mass	Obsrv. Mass	± da	± ppm	Start Seq.	End Seq.	Sequence	Ion Score	C. I. %	Modification	Plate [#] Name	Gel Idx/Pos [4700 Sample Name]	iTRAQ Ratio 115/114*	iTRAQ Ratio 116/114*	iTRAQ Ratio 117/114*	Rank	Result Type

Calc. Mass	Obsrv. Mass	± da	± ppm	Start Seq.	End Seq.	Sequence	Ion Score	C. I. %	Modification	Plate [#] Name	Gel Idx/Pos [4700 Sample Name]	iTRAQ Ratio 115/114*	iTRAQ Ratio 116/114*	iTRAQ Ratio 117/114*	Rank	Result Type
1673.8593	1673.9163	0.057	34	43	54	KEPVPDDSDLDR	50	99.976	(N-term)_iTRAQ[0], Lysine(K)_iTRAQ[1]	[3] F6 and F9	1110/1102	1.310	0.561	0.731	1	Mascot

782 phosphatidylinositol 4-kinase type 2-alpha [Rattus norvegicus] gi|16758554 58673.4 1 49 1.717 1.172 1.678 0.000 0.000 0.000 1 1 1 99.966

Peptide Information

Calc. Mass	Obsrv. Mass	± da	± ppm	Start Seq.	End Seq.	Sequence	Ion Score	C. I. %	Modification	Plate [#] Name	Gel Idx/Pos [4700 Sample Name]	iTRAQ Ratio 115/114*	iTRAQ Ratio 116/114*	iTRAQ Ratio 117/114*	Rank	Result Type
1623.8628	1623.8334	-0.0294	-18	129	140	IYQGSSGSYFVK	49	99.966	(N-term)_iTRAQ[0], Lysine(K)_iTRAQ[12]	[7] F5 120912	214/206	1.717	1.172	1.678	1	Mascot

783 trans-2-enoyl-CoA reductase, mitochondrial precursor [Rattus norvegicus] gi|8393848 43655.5 1 48 3.564 2.103 3.875 0.000 0.000 0.000 1 1 1 99.965

Peptide Information

Calc. Mass	Obsrv. Mass	± da	± ppm	Start Seq.	End Seq.	Sequence	Ion Score	C. I. %	Modification	Plate [#] Name	Gel Idx/Pos [4700 Sample Name]	iTRAQ Ratio 115/114*	iTRAQ Ratio 116/114*	iTRAQ Ratio 117/114*	Rank	Result Type
1339.7408	1339.7551	0.0143	11	309	316	GFWLSQWK	48	99.965	(N-term)_iTRAQ[0], Lysine(K)_iTRAQ[8]	[4] F7 and F10+11	411/403	3.564	2.103	3.875	1	Mascot

784 tumor-susceptibility gene 101 protein [Rattus norvegicus] gi|31415564 48241.4 1 48 1.477 1.706 1.432 0.000 0.000 0.000 1 1 1 99.965

Protein Group

tumor susceptibility gene 101 protein [Rattus norvegicus] gi|48374087 48222.4

Peptide Information

Calc. Mass	Obsrv. Mass	± da	± ppm	Start Seq.	End Seq.	Sequence	Ion Score	C. I. %	Modification	Plate [#] Name	Gel Idx/Pos [4700 Sample Name]	iTRAQ Ratio 115/114*	iTRAQ Ratio 116/114*	iTRAQ Ratio 117/114*	Rank	Result Type
1635.9146	1635.9229	0.0083	5	109	118	IYLPYLHDWK	48	99.965	(N-term)_iTRAQ[0], Lysine(K)_iTRAQ[10]	[3] F6 and F9	1350/1342	1.477	1.706	1.432	1	Mascot

785 dolichyl-diphosphooligosaccharide--protein glycosyltransferase subunit 2 [Rattus norvegicus] gi|13928974 73577.1 1 48 0.772 0.867 1.057 0.000 0.000 0.000 1 1 1 99.962

Protein Group

RecName: Full=Dolichyl-diphosphooligosaccharide--protein glycosyltransferase subunit 2; AltName: Fu gi|62512124 73594.1

Peptide Information

Calc. Mass	Obsrv. Mass	± da	± ppm	Start Seq.	End Seq.	Sequence	Ion Score	C. I. %	Modification	Plate [#] Name	Gel Idx/Pos [4700 Sample Name]	iTRAQ Ratio 115/114*	iTRAQ Ratio 116/114*	iTRAQ Ratio 117/114*	Rank	Result Type
1543.8452	1543.8115	-0.0337	-22	179	190	NIVEEIEDLVAR	48	99.962	(N-term)_iTRAQ[0]	[8] F13-15 and F1+2	242/234	0.772	0.867	1.057	1	Mascot

786 bifunctional protein NCOAT [Rattus norvegicus] gi|18777747 112515.8 1 48 1.146 1.126 0.852 0.000 0.000 0.000 1 1 1 99.961

Peptide Information

Calc. Mass	Obsrv. Mass	± da	± ppm	Start Seq.	End Seq.	Sequence	Ion Score	C. I. %	Modification	Plate [#] Name	Gel Idx/Pos [4700 Sample Name]	iTRAQ Ratio 115/114*	iTRAQ Ratio 116/114*	iTRAQ Ratio 117/114*	Rank	Result Type
1614.8323	1614.8759	0.0436	27	157	167	KLDQVSQFGCR	48	99.961	(N-term)_iTRAQ[0], Lysine(K)_iTRAQ[1], MMTS (C)[10]	[4] F7 and F10+11	1152/1144	1.146	1.126	0.852	1	Mascot

787 actin-related protein 3 [Rattus norvegicus] gi|11037751 10879.7 1 48 0.780 1.105 0.575 0.283 0.123 0.403 2 2 2 99.961

Peptide Information

Calc. Mass	Obsrv. Mass	± da	± ppm	Start Seq.	End Seq.	Sequence	Ion Score	C. I. %	Modification	Plate [#] Name	Gel Idx/Pos [4700 Sample Name]	iTRAQ Ratio 115/114*	iTRAQ Ratio 116/114*	iTRAQ Ratio 117/114*	Rank	Result Type
1743.8636	1743.9075	0.0439	25	65	76	KDYEEIGPSICR	38	99.605	(N-term)_iTRAQ[0], Lysine(K)_iTRAQ[1], MMTS (C)[11]	[3] F6 and F9	1246/1238	0.573	0.994	0.978	1	Mascot
1743.8636	1743.9247	0.0611	35	65	76	KDYEEIGPSICR	48	99.961	(N-term)_iTRAQ[0], Lysine(K)_iTRAQ[1], MMTS (C)[11]	[4] F7 and F10+11	1183/1175	1.063	1.228	0.338	1	Mascot

788 twinfilin-1 [Rattus norvegicus] gi|56606100 44140.2 1 48 1.275 1.406 0.523 0.000 0.000 0.000 1 1 1 99.958

Peptide Information

Calc. Mass	Obsrv. Mass	± da	± ppm	Start Seq.	End Seq.	Sequence	Ion Score	C. I. %	Modification	Plate [#] Name	Gel Idx/Pos [4700 Sample Name]	iTRAQ Ratio	iTRAQ Ratio	iTRAQ Ratio	Rank	Result Type

Calc. Mass	Obsrv. Mass	± da	± ppm	Start Seq.	End Seq.	Sequence	Ion Score	C. I. %	Modification	Plate [#] Name	Gel Idx/Pos [4700 Sample Name]	iTRAQ Ratio 115/114*	iTRAQ Ratio 116/114*	iTRAQ Ratio 117/114*	Rank	Result Type
2152.1772	2152.2578	0.0806	37	111	126	EFGGGHIKDEVFGTVK	48	99.958	(N-term)_iTRAQ[0], Lysine(K)_iTRAQ[8,16]	[4] F7 and F10+11	1186/1178	1.275	1.406	0.523	1	Mascot

789 F-box/LRR-repeat protein 16 [Rattus norvegicus] gi|57222336 55475.2 1 47 0.640 0.987 0.736 0.000 0.000 0.000 1 1 1 99.956

Peptide Information

Calc. Mass	Obsrv. Mass	± da	± ppm	Start Seq.	End Seq.	Sequence	Ion Score	C. I. %	Modification	Plate [#] Name	Gel Idx/Pos [4700 Sample Name]	iTRAQ Ratio 115/114*	iTRAQ Ratio 116/114*	iTRAQ Ratio 117/114*	Rank	Result Type
1627.957	1627.9443	-0.0127	-8	133	144	FWAGLTPVLHAK	47	99.956	(N-term)_iTRAQ[0], Lysine(K)_iTRAQ[12]	[4] F7 and F10+11	395/387	0.640	0.987	0.736	1	Mascot

790 Proteasome (prosome, macropain) subunit, beta type 1 [Rattus norvegicus] gi|37231712 28591.7 1 47 1.050 1.753 1.616 0.000 0.000 0.000 1 1 1 99.954

Protein Group

proteasome subunit beta type-1 precursor [Rattus norvegicus]	gi|16758370	28663.7

Peptide Information

Calc. Mass	Obsrv. Mass	± da	± ppm	Start Seq.	End Seq.	Sequence	Ion Score	C. I. %	Modification	Plate [#] Name	Gel Idx/Pos [4700 Sample Name]	iTRAQ Ratio 115/114*	iTRAQ Ratio 116/114*	iTRAQ Ratio 117/114*	Rank	Result Type
1689.8357	1689.7719	-0.0638	-38	146	159	GAVYSFDPVGSYQR	47	99.954	(N-term)_iTRAQ[0]	[1] F3 030912	281/273	1.050	1.753	1.616	1	Mascot

791 proSAAS [Rattus norvegicus] gi|9507061 27876.1 1 47 0.934 2.034 1.197 0.000 0.000 0.000 1 1 1 99.954

Peptide Information

Calc. Mass	Obsrv. Mass	± da	± ppm	Start Seq.	End Seq.	Sequence	Ion Score	C. I. %	Modification	Plate [#] Name	Gel Idx/Pos [4700 Sample Name]	iTRAQ Ratio 115/114*	iTRAQ Ratio 116/114*	iTRAQ Ratio 117/114*	Rank	Result Type
1639.8776	1639.8976	0.02	12	202	216	ILTGSSEPEAAPAPR	47	99.954	(N-term)_iTRAQ[0]	[8] F13-15 and F1+2	580/572	0.934	2.034	1.197	1	Mascot

792 ribosomal protein S2 [Rattus norvegicus] gi|2920829 14647.1 1 47 1.376 1.281 1.221 0.000 0.000 0.000 1 1 1 99.948

Protein Group

40S ribosomal protein S2 [Mus musculus]	gi|18087805	35044.1
LOC100125366 protein [Rattus norvegicus]	gi|38014846	10344.8
ribosomal protein S2 [Rattus norvegicus]	gi|2920833	31046
ribosomal protein S2 [Rattus norvegicus]	gi|2920831	31609.2
ribosomal protein S2 [Rattus norvegicus]	gi|2920825	31950.5
ribosomal protein S2 [Rattus norvegicus]	gi|2920827	32407.6

Peptide Information

Calc. Mass	Obsrv. Mass	± da	± ppm	Start Seq.	End Seq.	Sequence	Ion Score	C. I. %	Modification	Plate [#] Name	Gel Idx/Pos [4700 Sample Name]	iTRAQ Ratio 115/114*	iTRAQ Ratio 116/114*	iTRAQ Ratio 117/114*	Rank	Result Type
1840.0718	1840.0166	-0.0552	-30	52	64	SLEEIYLFSLPIK	47	99.948	(N-term)_iTRAQ[0], Lysine(K)_iTRAQ[13]	[1] F3 030912	507/499	1.376	1.281	1.221	1	Mascot

793 heat shock factor-binding protein 1 [Rattus norvegicus] gi|27465567 9443.8 1 46 1.524 0.801 1.565 0.000 0.000 0.000 1 1 1 99.945

Peptide Information

Calc. Mass	Obsrv. Mass	± da	± ppm	Start Seq.	End Seq.	Sequence	Ion Score	C. I. %	Modification	Plate [#] Name	Gel Idx/Pos [4700 Sample Name]	iTRAQ Ratio 115/114*	iTRAQ Ratio 116/114*	iTRAQ Ratio 117/114*	Rank	Result Type
1451.78	1451.7324	-0.0476	-33	27	37	FQIMSDQIIGR	46	99.945	(N-term)_iTRAQ[0]	[1] F3 030912	328/320	1.524	0.801	1.565	1	Mascot

794 Pdhx protein [Rattus norvegicus] gi|60688224 44893.4 1 46 0.819 1.077 0.957 0.000 0.000 0.000 1 1 1 99.943

Protein Group

pyruvate dehydrogenase complex, component X [Rattus norvegicus]	gi|113205496	63739.1

Peptide Information

Calc. Mass	Obsrv. Mass	± da	± ppm	Start Seq.	End Seq.	Sequence	Ion Score	C. I. %	Modification	Plate [#] Name	Gel Idx/Pos [4700 Sample Name]	iTRAQ Ratio 115/114*	iTRAQ Ratio 116/114*	iTRAQ Ratio 117/114*	Rank	Result Type
1902.9629	1902.859	-0.1039	-55	221	235	QMPGVNVTWDGEGPK	46	99.943	(N-term)_iTRAQ[0], Lysine(K)_iTRAQ[15]	[5] F4	193/185	0.819	1.077	0.957	1	Mascot

795	monocarboxylate transporter 1 [Rattus norvegicus]	gi|6981542	57744.1	1	46	1.502	0.931	0.444	0.000	0.000	0.000	1	1	1	99.943

Peptide Information

Calc. Mass	Obsrv. Mass	± da	± ppm	Start Seq.	End Seq.	Sequence	Ion Score	C. I. %	Modification	Plate [#] Name	Gel Idx/Pos [4700 Sample Name]	iTRAQ Ratio 115/114*	iTRAQ Ratio 116/114*	iTRAQ Ratio 117/114*	Rank	Result Type
1323.7882	1323.7239	-0.0643	-49	236	244	LSVFQTVNK	46	99.943	(N-term)_iTRAQ[0], Lysine(K)_iTRAQ[9]	[7] F5 120912	230/222	1.502	0.931	0.444	1	Mascot

796	zinc transporter 3 [Rattus norvegicus]	gi|61557417	43392.1	1	46	0.849	0.946	0.888	0.000	0.000	0.000	1	1	1	99.941

Peptide Information

Calc. Mass	Obsrv. Mass	± da	± ppm	Start Seq.	End Seq.	Sequence	Ion Score	C. I. %	Modification	Plate [#] Name	Gel Idx/Pos [4700 Sample Name]	iTRAQ Ratio 115/114*	iTRAQ Ratio 116/114*	iTRAQ Ratio 117/114*	Rank	Result Type
1916.0262	1915.957	-0.0692	-36	32	46	SLFTEPSEPLPEGPK	46	99.941	(N-term)_iTRAQ[0], Lysine(K)_iTRAQ[15]	[1] F3 030912	290/282	0.849	0.946	0.888	1	Mascot

797	Eif4g2 protein [Rattus norvegicus]	gi|60551878	54087	1	46	1.274	1.033	1.697	0.000	0.000	0.000	1	1	1	99.94

Peptide Information

Calc. Mass	Obsrv. Mass	± da	± ppm	Start Seq.	End Seq.	Sequence	Ion Score	C. I. %	Modification	Plate [#] Name	Gel Idx/Pos [4700 Sample Name]	iTRAQ Ratio 115/114*	iTRAQ Ratio 116/114*	iTRAQ Ratio 117/114*	Rank	Result Type
1422.897	1422.8639	-0.0331	-23	242	251	GLSFLFPLLK	46	99.94	(N-term)_iTRAQ[0], Lysine(K)_iTRAQ[10]	[5] F4	414/406	1.274	1.033	1.697	1	Mascot

798	sodium- and chloride-dependent GABA transporter 2 [Rattus norvegicus]	gi|19424348	72491.5	1	46	1.568	0.690	0.565	0.000	0.000	0.000	1	1	1	99.94

Protein Group

RecName: Full=Sodium- and chloride-dependent betaine transporter; AltName: Full=Na(+)/Cl(-) betaine	gi|1352527	74172.4
dopamine transporter [Rattus sp.]	gi|243203	72324.4
norepinephrine transporter [Rattus norvegicus]	gi|2598034	73484.4
norepinephrine transporter a (rNETa) [Rattus norvegicus]	gi|4630792	67697.5
sodium- and chloride-dependent GABA transporter 3 [Rattus norvegicus]	gi|13242271	74940.7
sodium- and chloride-dependent betaine transporter [Rattus norvegicus]	gi|8394200	75679.1
sodium- and chloride-dependent creatine transporter 1 [Rattus norvegicus]	gi|8393113	74766.6
sodium- and chloride-dependent taurine transporter [Rattus norvegicus]	gi|8394318	74443
sodium-dependent dopamine transporter [Rattus norvegicus]	gi|7106413	72181.2
sodium-dependent noradrenaline transporter [Rattus norvegicus]	gi|13786180	71179.4

Peptide Information

Calc. Mass	Obsrv. Mass	± da	± ppm	Start Seq.	End Seq.	Sequence	Ion Score	C. I. %	Modification	Plate [#] Name	Gel Idx/Pos [4700 Sample Name]	iTRAQ Ratio 115/114*	iTRAQ Ratio 116/114*	iTRAQ Ratio 117/114*	Rank	Result Type
1267.6445	1267.6418	-0.0027	-2	58	64	FPYLCYK	46	99.94	(N-term)_iTRAQ[0], Lysine(K)_iTRAQ[7], MMTS (C)[5]	[7] F5 120912	359/351	1.568	0.690	0.565	1	Mascot

799	proteasome activator complex subunit 2 [Rattus	gi|8394091	30194.2	1	46	1.220	1.369	1.004	0.000	0.000	0.000	1	1	1	99.939

norvegicus]

Peptide Information

Calc. Mass	Obsrv. Mass	± da	± ppm	Start Seq.	End Seq.	Sequence	Ion Score	C. I. %	Modification	Plate [#] Name	Gel Idx/Pos [4700 Sample Name]	iTRAQ Ratio 115/114*	iTRAQ Ratio 116/114*	iTRAQ Ratio 117/114*	Rank	Result Type
2160.1699	2160.2783	0.1084	50	210	225	AFYAELHHIISSNLEK	46	99.939	(N-term)_iTRAQ[0], Lysine(K)_iTRAQ[16]	[4] F7 and F10+11	1312/1304	1.220	1.369	1.004	1	Mascot

800	neuroglycan C	gi|1585922	61773.7	1	46	0.880	0.907	1.482	0.000	0.000	0.000	1	1	1	99.939

Protein Group

chondroitin sulfate proteoglycan 5 isoform 1 [Rattus norvegicus]	gi|9506523	60910.6
chondroitin sulfate proteoglycan 5 isoform 2 [Rattus norvegicus]	gi|41281651	64158

Peptide Information

Calc. Mass	Obsrv. Mass	± da	± ppm	Start Seq.	End Seq.	Sequence	Ion Score	C. I. %	Modification	Plate [#] Name	Gel Idx/Pos [4700 Sample Name]	iTRAQ Ratio 115/114*	iTRAQ Ratio 116/114*	iTRAQ Ratio 117/114*	Rank	Result Type
2313.2131	2313.0833	-0.1298	-56	508	523	LKEEESFNIQNSMSPK	46	99.939	(N-term)_iTRAQ[0], Lysine(K)_iTRAQ[2,16]	[6] F8 110912	264/256	0.880	0.907	1.482	1	Mascot

801	NADH dehydrogenase subunit 4 [Rattus norvegicus]	gi|110189724	53902.9	1	46	0.992	0.916	1.044	0.000	0.000	0.000	1	1	1	99.933

Protein Group

NADH dehydrogenase subunit 4 [Rattus norvegicus]	gi|110189672	53920.9
unknown protein [Rattus norvegicus]	gi|829028	53884.8

Peptide Information

Calc. Mass	Obsrv. Mass	± da	± ppm	Start Seq.	End Seq.	Sequence	Ion Score	C. I. %	Modification	Plate [#] Name	Gel Idx/Pos [4700 Sample Name]	iTRAQ Ratio 115/114*	iTRAQ Ratio 116/114*	iTRAQ Ratio 117/114*	Rank	Result Type
1742.0073	1742.0122	0.0049	3	207	218	MPLYGVHLWLPK	46	99.933	(N-term)_iTRAQ[0], Lysine(K)_iTRAQ[12]	[4] F7 and F10+11	450/442	0.992	0.916	1.044	1	Mascot

802	isovaleryl-CoA dehydrogenase, mitochondrial precursor [Rattus norvegicus]	gi|6981112	50232.1	1	45	0.746	1.534	0.893	0.000	0.000	0.000	1	1	1	99.923

Peptide Information

Calc. Mass	Obsrv. Mass	± da	± ppm	Start Seq.	End Seq.	Sequence	Ion Score	C. I. %	Modification	Plate [#] Name	Gel Idx/Pos [4700 Sample Name]	iTRAQ Ratio 115/114*	iTRAQ Ratio 116/114*	iTRAQ Ratio 117/114*	Rank	Result Type
1582.7959	1582.7384	-0.0575	-36	66	76	AQEIDQSNDFK	45	99.923	(N-term)_iTRAQ[0], Lysine(K)_iTRAQ[11]	[7] F5 120912	140/132	0.746	1.534	0.893	1	Mascot

803	ras-related protein Rab-11B [Rattus norvegicus]	gi|14249144	26582.9	1	45	1.240	1.178	0.971	0.000	0.000	0.000	1	1	1	99.919

Protein Group

GTP binding protein [Rattus norvegicus]	gi|2463536	26504.9
ras-related protein Rab-11A isoform 1 [Homo sapiens]	gi|4758984	26343.7

Peptide Information

Calc. Mass	Obsrv. Mass	± da	± ppm	Start Seq.	End Seq.	Sequence	Ion Score	C. I. %	Modification	Plate [#] Name	Gel Idx/Pos [4700 Sample Name]	iTRAQ Ratio 115/114*	iTRAQ Ratio 116/114*	iTRAQ Ratio 117/114*	Rank	Result Type
1418.7148	1418.6317	-0.0831	-59	62	72	AQIWDTAGQER	45	99.919	(N-term)_iTRAQ[0]	[5] F4	131/123	1.240	1.178	0.971	1	Mascot

804	RecName: Full=Coronin-7; Short=Crn7; AltName: Full=70 kDa WD repeat tumor rejection antigen	gi|3123027	51971.6	1	45	0.494	0.890	1.101	0.000	0.000	0.000	1	1	1	99.915

Peptide Information

Calc. Mass	Obsrv. Mass	± da	± ppm	Start Seq.	End Seq.	Sequence	Ion Score	C. I. %	Modification	Plate [#] Name	Gel Idx/Pos [4700 Sample Name]	iTRAQ Ratio 115/114*	iTRAQ Ratio 116/114*	iTRAQ Ratio 117/114*	Rank	Result Type
1707.8785	1707.7915	-0.087	-51	187	202	SSPLPLQDGPGPEGGR	45	99.915	(N-term)_iTRAQ[0]	[1] F3 030912	209/201	0.494	0.890	1.101	1	Mascot

805	NEDD8 precursor [Mus musculus]	gi|6679034	10407.9	1	44	1.092	0.854	1.204	0.000	0.000	0.000	1	1	1	99.911

Peptide Information

Calc. Mass	Obsrv. Mass	± da	± ppm	Start Seq.	End Seq.	Sequence	Ion Score	C. I. %	Modification	Plate [#] Name	Gel Idx/Pos [4700 Sample Name]	iTRAQ Ratio 115/114*	iTRAQ Ratio 116/114*	iTRAQ Ratio 117/114*	Rank	Result Type
1974.0641	1974.0802	0.0161	8	12	25	EIEIDIEPTDKVER	44	99.911	(N-term)_iTRAQ[0], Lysine(K)_iTRAQ[11]	[3] F6 and F9	273/265	1.092	0.854	1.204	1	Mascot

806	polyadenylate-binding protein 1 [Rattus norvegicus]	gi|19705459	76892.1	1	44	0.721	0.730	0.472	0.000	0.000	0.000	1	1	1	99.909

Peptide Information

Calc. Mass	Obsrv. Mass	± da	± ppm	Start Seq.	End Seq.	Sequence	Ion Score	C. I. %	Modification	Plate [#] Name	Gel Idx/Pos [4700 Sample Name]	iTRAQ Ratio 115/114*	iTRAQ Ratio 116/114*	iTRAQ Ratio 117/114*	Rank	Result Type
1884.9	1884.8679	-0.0321	-17	139	153	GYGFVHFETQEAAER	44	99.909	(N-term)_iTRAQ[0]	[4] F7 and F10+11	280/272	0.721	0.730	0.472	1	Mascot

807	translation initiation factor eIF-2B subunit delta [Rattus norvegicus]	gi|16758830	63178.5	1	44	0.619	1.804	1.272	0.000	0.000	0.000	1	1	1	99.905

Peptide Information

Calc. Mass	Obsrv. Mass	± da	± ppm	Start Seq.	End Seq.	Sequence	Ion Score	C. I. %	Modification	Plate [#] Name	Gel Idx/Pos [4700 Sample Name]	iTRAQ Ratio 115/114*	iTRAQ Ratio 116/114*	iTRAQ Ratio 117/114*	Rank	Result Type
1850.9706	1850.8478	-0.1228	-66	75	90	ELQGTGSQLGGTTGEK	44	99.905	(N-term)_iTRAQ[0], Lysine(K)_iTRAQ[16]	[5] F4	101/93	0.619	1.804	1.272	1	Mascot

808	cysteine-rich protein 2 [Rattus norvegicus]	gi|11968068	26639.2	1	44	0.793	0.221	0.515	0.000	0.000	0.000	1	1	1	99.904

Peptide Information

Calc. Mass	Obsrv. Mass	± da	± ppm	Start Seq.	End Seq.	Sequence	Ion Score	C. I. %	Modification	Plate [#] Name	Gel Idx/Pos [4700 Sample Name]	iTRAQ Ratio 115/114*	iTRAQ Ratio 116/114*	iTRAQ Ratio 117/114*	Rank	Result Type
3582.9417	3582.8093	-0.1324	-37	66	97	GVNIGGAGSYIYEKPPTE APQVTGPIEVPVVR	44	99.904	(N-term)_iTRAQ[0], Lysine(K)_iTRAQ[14]	[1] F3 030912	383/375	0.793	0.221	0.515	1	Mascot

809	ATP-dependent RNA helicase DDX1 [Rattus norvegicus]	gi|61097941	92016.8	1	44	0.855	1.200	0.384	0.000	0.000	0.000	1	1	1	99.901

Peptide Information

Calc. Mass	Obsrv. Mass	± da	± ppm	Start Seq.	End Seq.	Sequence	Ion Score	C. I. %	Modification	Plate [#] Name	Gel Idx/Pos [4700 Sample Name]	iTRAQ Ratio 115/114*	iTRAQ Ratio 116/114*	iTRAQ Ratio 117/114*	Rank	Result Type
1674.9287	1674.9573	0.0286	17	422	432	IMHFPTWVDLK	44	99.901	(N-term)_iTRAQ[0], Lysine(K)_iTRAQ[11]	[3] F6 and F9	1361/1353	0.855	1.200	0.384	1	Mascot

810	SH3 and multiple ankyrin repeat domains protein 2 isoform c [Rattus norvegicus]	gi|19263336	145805.2	1	44	1.666	2.259	1.825	0.000	0.000	0.000	1	1	1	99.899

Protein Group

RecName: Full=SH3 and multiple ankyrin repeat domains protein 2; Short=Shank2; AltName: Full=Cortac	gi|22001984	171392.7
SH3 and multiple ankyrin repeat domains protein 2 isoform a [Rattus norvegicus]	gi|41235797	215545.7
SH3 and multiple ankyrin repeat domains protein 2 isoform b [Rattus norvegicus]	gi|51921383	144561.3
SH3 and multiple ankyrin repeat domains protein 2 isoform d [Rattus norvegicus]	gi|40255311	171022.5
proline rich synapse associated protein 1 [Rattus norvegicus]	gi|4995818	144575.3
proline rich synapse associated protein 1 [Rattus norvegicus]	gi|4995819	144603.5

Peptide Information

Calc. Mass	Obsrv. Mass	± da	± ppm	Start Seq.	End Seq.	Sequence	Ion Score	C. I. %	Modification	Plate [#] Name	Gel Idx/Pos [4700 Sample Name]	iTRAQ Ratio 115/114*	iTRAQ Ratio 116/114*	iTRAQ Ratio 117/114*	Rank	Result Type
1974.0563	1974.0187	-0.0376	-19	1031	1046	ANVISELNSILQQMNR	44	99.899	(N-term)_iTRAQ[0]	[1] F3 030912	555/547	1.666	2.259	1.825	1	Mascot

811	WD repeat-containing protein 1 [Rattus norvegicus]	gi|62078997	73032.2	1	44	0.942	1.024	1.252	0.000	0.000	0.000	1	1	1	99.895

Peptide Information

Calc. Mass	Obsrv. Mass	± da	± ppm	Start Seq.	End Seq.	Sequence	Ion Score	C. I. %	Modification	Plate [#] Name	Gel Idx/Pos [4700 Sample Name]	iTRAQ Ratio 115/114*	iTRAQ Ratio 116/114*	iTRAQ Ratio 117/114*	Rank	Result Type
1390.7252	1390.6755	-0.0497	-36	96	104	YEYQPFAGK	44	99.895	(N-term)_iTRAQ[0], Lysine(K)_iTRAQ[9]	[7] F5 120912	193/185	0.942	1.024	1.252	1	Mascot

812	SUMO-conjugating enzyme UBC9 [Homo sapiens]	gi|4507785	20340.7	1	44	0.873	1.258	1.040	0.000	0.000	0.000	1	1	1	99.894

Peptide Information

Calc. Mass	Obsrv. Mass	± da	± ppm	Start Seq.	End Seq.	Sequence	Ion Score	C. I. %	Modification	Plate [#] Name	Gel Idx/Pos [4700 Sample Name]	iTRAQ Ratio 115/114*	iTRAQ Ratio 116/114*	iTRAQ Ratio 117/114*	Rank	Result Type
1875.0864	1875.0278	-0.0586	-31	18	30	KDHPFGFVAVPTK	44	99.894	(N-term)_iTRAQ[0], Lysine(K)_iTRAQ[1,13]	[2] F12 040912	295/287	0.873	1.258	1.040	1	Mascot

813	Ba1-651 [Rattus norvegicus]	gi|33086632	175229.2	1	44	1.045	0.903	1.039	0.000	0.000	0.000	1	1	1	99.89

Peptide Information

Calc. Mass	Obsrv. Mass	± da	± ppm	Start Seq.	End Seq.	Sequence	Ion Score	C. I. %	Modification	Plate [#] Name	Gel Idx/Pos [4700 Sample Name]	iTRAQ Ratio 115/114*	iTRAQ Ratio 116/114*	iTRAQ Ratio 117/114*	Rank	Result Type
1591.8312	1591.7421	-0.0891	-56	866	880	STGSVVGQQPFGGAR	44	99.89	(N-term)_iTRAQ[0]	[1] F3 030912	192/184	1.045	0.903	1.039	1	Mascot

814	protein CDV3 homolog [Rattus norvegicus]	gi|62078927	26889.5	1	43	0.885	1.509	0.853	1.859	1.966	0.665	2	2	2	99.889

Peptide Information

Calc. Mass	Obsrv. Mass	± da	± ppm	Start Seq.	End Seq.	Sequence	Ion Score	C. I. %	Modification	Plate [#] Name	Gel Idx/Pos [4700 Sample Name]	iTRAQ Ratio 115/114*	iTRAQ Ratio 116/114*	iTRAQ Ratio 117/114*	Rank	Result Type
2078.9148	2078.7883	-0.1265	-61	142	159	EDPGDNWEEGGGGSGAEK	43	99.889	(N-term)_iTRAQ[0], Lysine(K)_iTRAQ[18]	[5] F4	101/93	0.286	0.655	0.479	1	Mascot
2078.9148	2078.825	-0.0898	-43	142	159	EDPGDNWEEGGGGSGAEK	29	97.119	(N-term)_iTRAQ[0], Lysine(K)_iTRAQ[18]	[7] F5 120912	135/127	2.744	3.475	1.518	1	Mascot

815	protein kinase C epsilon [Rattus norvegicus]	gi|49359177	92839.5	1	43	2.009	2.105	3.246	0.000	0.000	0.000	1	1	1	99.888

Protein Group

protein kinase [Rattus norvegicus]	gi|206193	29130.9

Peptide Information

Calc. Mass	Obsrv. Mass	± da	± ppm	Start Seq.	End Seq.	Sequence	Ion Score	C. I. %	Modification	Plate [#] Name	Gel Idx/Pos [4700 Sample Name]	iTRAQ Ratio 115/114*	iTRAQ Ratio 116/114*	iTRAQ Ratio 117/114*	Rank	Result Type
2275.2144	2275.2959	0.0815	36	532	547	DLKLDNILLDAEGHCK	43	99.888	(N-term)_iTRAQ[0], Lysine(K)_iTRAQ[3,16], MMTS (C)[15]	[4] F7 and F10+11	1304/1296	2.009	2.105	3.246	1	Mascot

816	nitric oxide-enhanced ADP-ribosylated protein, NAP, glyceraldehyde-3-phosphate dehydrogenase, GAPDH {N-terminal} [rats, brain, Peptide Partial, 29 aa]	gi|257764	3541	1	43	1.279	1.295	1.193	0.000	0.000	0.000	1	1	1	99.886

Peptide Information

Calc. Mass	Obsrv. Mass	± da	± ppm	Start Seq.	End Seq.	Sequence	Ion Score	C. I. %	Modification	Plate [#] Name	Gel Idx/Pos [4700 Sample Name]	iTRAQ Ratio 115/114*	iTRAQ Ratio 116/114*	iTRAQ Ratio 117/114*	Rank	Result Type
1075.5143	1075.5516	0.0373	35	18	24	AAFSXDK	43	99.886	(N-term)_iTRAQ[0], Lysine(K)_iTRAQ[7]	[4] F7 and F10+11	209/201	1.279	1.295	1.193	1	Mascot

817	Rps16 protein [Rattus norvegicus]	gi|39850096	20558.7	1	43	0.759	1.387	0.585	0.000	0.000	0.000	1	1	1	99.884

Protein Group

40S ribosomal protein S16 [Homo sapiens]	gi|4506691	18976.8
Rps16 protein [Rattus norvegicus]	gi|54261550	20430.6

Peptide Information

Calc. Mass	Obsrv. Mass	± da	± ppm	Start Seq.	End Seq.	Sequence	Ion Score	C. I. %	Modification	Plate [#] Name	Gel Idx/Pos [4700 Sample Name]	iTRAQ Ratio 115/114*	iTRAQ Ratio 116/114*	iTRAQ Ratio 117/114*	Rank	Result Type
1331.7556	1331.699	-0.0566	-43	20	30	GPLQSVQVFGR	43	99.884	(N-term)_iTRAQ[0]	[1] F3 030912	291/283	0.759	1.387	0.585	1	Mascot

818	NEDD8-activating enzyme E1 regulatory subunit [Rattus norvegicus]	gi|14091760	65848	1	43	0.934	1.286	1.344	0.000	0.000	0.000	1	1	1	99.883

Peptide Information

Calc. Mass	Obsrv. Mass	± da	± ppm	Start Seq.	End Seq.	Sequence	Ion Score	C. I. %	Modification	Plate [#] Name	Gel Idx/Pos [4700 Sample Name]	iTRAQ Ratio 115/114*	iTRAQ Ratio 116/114*	iTRAQ Ratio 117/114*	Rank	Result Type
1966.9215	1966.8582	-0.0633	-32	194	206	EHFQSYDLDHMEK	43	99.883	(N-term)_iTRAQ[0], Lysine(K)_iTRAQ[13]	[2] F12 040912	249/241	0.934	1.286	1.344	1	Mascot

819	nucleobindin-1 precursor [Rattus norvegicus]	gi|16758210	57652.9	1	43	1.203	0.722	1.199	0.000	0.000	0.000	1	1	1	99.881

Peptide Information

Calc. Mass	Obsrv. Mass	± da	± ppm	Start Seq.	End Seq.	Sequence	Ion Score	C. I. %	Modification	Plate [#] Name	Gel Idx/Pos [4700 Sample Name]	iTRAQ Ratio 115/114*	iTRAQ Ratio 116/114*	iTRAQ Ratio 117/114*	Rank	Result Type
1650.9187	1650.9176	-0.0011	-1	310	322	LVTLEEFLASTQR	43	99.881	(N-term)_iTRAQ[0]	[8] F13-15 and F1+2	305/297	1.203	0.722	1.199	1	Mascot

820	69-C11 protein [Rattus norvegicus]	gi|24415860	9249.2	1	43	0.976	1.010	1.302	0.000	0.000	0.000	1	1	1	99.881

Protein Group

NADH dehydrogenase subunit 1 [Rattus norvegicus]	gi|19577314	37163.6
NADH dehydrogenase subunit 1 [Rattus norvegicus]	gi|110189715	37175.7

Peptide Information

Calc. Mass	Obsrv. Mass	± da	± ppm	Start Seq.	End Seq.	Sequence	Ion Score	C. I. %	Modification	Plate [#] Name	Gel Idx/Pos [4700 Sample Name]	iTRAQ Ratio 115/114*	iTRAQ Ratio 116/114*	iTRAQ Ratio 117/114*	Rank	Result Type
2276.2358	2276.1692	-0.0666	-29	20	38	GPNIVGPYGILQPFADAMK	43	99.881	(N-term)_iTRAQ[0], Lysine(K)_iTRAQ[19]	[1] F3 030912	504/496	0.976	1.010	1.302	1	Mascot

821	Smarce1 protein [Rattus norvegicus]	gi|60552765	38589.1	1	43	1.142	1.430	0.736	0.000	0.000	0.000	1	1	1	99.879

Peptide Information

Calc. Mass	Obsrv. Mass	± da	± ppm	Start Seq.	End Seq.	Sequence	Ion Score	C. I. %	Modification	Plate [#] Name	Gel Idx/Pos [4700 Sample Name]	iTRAQ Ratio 115/114*	iTRAQ Ratio 116/114*	iTRAQ Ratio 117/114*	Rank	Result Type
1874.9706	1874.8696	-0.101	-54	210	224	SQGSIAPEEEQVANK	43	99.879	(N-term)_iTRAQ[0], Lysine(K)_iTRAQ[15]	[5] F4	106/98	1.142	1.430	0.736	1	Mascot

822	F-box only protein 2 [Rattus norvegicus]	gi|16758270	35251.8	1	43	1.750	1.089	0.661	0.000	0.000	0.000	1	1	1	99.879

Peptide Information

Calc. Mass	Obsrv. Mass	± da	± ppm	Start Seq.	End Seq.	Sequence	Ion Score	C. I. %	Modification	Plate [#] Name	Gel Idx/Pos [4700 Sample Name]	iTRAQ Ratio 115/114*	iTRAQ Ratio 116/114*	iTRAQ Ratio 117/114*	Rank	Result Type
1740.8591	1740.9493	0.0902	52	269	280	FEHGGQDSVYWK	43	99.879	(N-term)_iTRAQ[0], Lysine(K)_iTRAQ[12]	[4] F7 and F10+11	1129/1121	1.750	1.089	0.661	1	Mascot

823	Pitpnb protein [Rattus norvegicus]	gi|38181812	35725.5	1	43	1.017	1.565	0.999	0.000	0.000	0.000	1	1	1	99.873

Protein Group

phosphatidylinositol transfer protein beta isoform [Rattus norvegicus]	gi|16758568	35838.8

Peptide Information

Calc. Mass	Obsrv. Mass	± da	± ppm	Start Seq.	End Seq.	Sequence	Ion Score	C. I. %	Modification	Plate [#] Name	Gel Idx/Pos [4700 Sample Name]	iTRAQ Ratio 115/114*	iTRAQ Ratio 116/114*	iTRAQ Ratio 117/114*	Rank	Result Type
1386.7141	1386.7302	0.0161	12	227	234	QLFCWIDK	44	99.903	(N-term)_iTRAQ[0], Lysine(K)_iTRAQ[8], MMTS (C)[4]	[7] F5 120912	421/413	1.017	1.565	0.999	1	Mascot

824	proteasome subunit alpha type-6 [Rattus norvegicus]	gi|8394076	30631.7	1	43	0.752	0.874	0.972	0.000	0.000	0.000	1	1	1	99.863

Peptide Information

Calc. Mass	Obsrv. Mass	± da	± ppm	Start Seq.	End Seq.	Sequence	Ion Score	C. I. %	Modification	Plate [#] Name	Gel Idx/Pos [4700 Sample Name]	iTRAQ Ratio 115/114*	iTRAQ Ratio 116/114*	iTRAQ Ratio 117/114*	Rank	Result Type
1448.8035	1448.7822	-0.0213	-15	22	30	LYQVEYAFK	43	99.863	(N-term)_iTRAQ[0], Lysine(K)_iTRAQ[9]	[7] F5 120912	305/297	0.752	0.874	0.972	1	Mascot

825	3-phosphoinositide-dependent protein kinase 1 [Rattus norvegicus]	gi|13592023	69649	1	43	1.448	1.185	1.274	0.000	0.000	0.000	1	1	1	99.863

Peptide Information

Calc. Mass	Obsrv. Mass	± da	± ppm	Start	End	Sequence	Ion	C. I. %	Modification	Plate [#] Name	Gel Idx/Pos [4700	iTRAQ	iTRAQ	iTRAQ	Rank	Result Type

				Seq.	Seq.		Score				Sample Name]	Ratio 115/114*	Ratio 116/114*	Ratio 117/114*		
1816.9161	1816.8145	-0.1016	-56	31	47	SQTEPSSSPGIPSGVSR	43	99.863	(N-term)_iTRAQ[0]	[1] F3 030912	164/156	1.448	1.185	1.274	1	Mascot

826	fumarylacetoacetase [Rattus norvegicus]	gi|8393349	48769.8	1	42	1.820	0.873	1.526	0.000	0.000	0.000	1	1	1	99.86

Peptide Information

Calc. Mass	Obsrv. Mass	± da	± ppm	Start Seq.	End Seq.	Sequence	Ion Score	C. I. %	Modification	Plate [#] Name	Gel Idx/Pos [4700 Sample Name]	iTRAQ Ratio 115/114*	iTRAQ Ratio 116/114*	iTRAQ Ratio 117/114*	Rank	Result Type
2178.1846	2178.1162	-0.0684	-31	238	253	DIQQWEYVPLGPFLGK	42	99.86	(N-term)_iTRAQ[0], Lysine(K)_iTRAQ[16]	[1] F3 030912	494/486	1.820	0.873	1.526	1	Mascot

827	Acyl-Coenzyme A binding domain containing 3 [Rattus norvegicus]	gi|54035576	64862	1	42	1.082	1.081	0.914	0.000	0.000	0.000	1	1	1	99.859

Protein Group

Golgi resident protein GCP60 [Rattus norvegicus]	gi|33469059	65093.1

Peptide Information

Calc. Mass	Obsrv. Mass	± da	± ppm	Start Seq.	End Seq.	Sequence	Ion Score	C. I. %	Modification	Plate [#] Name	Gel Idx/Pos [4700 Sample Name]	iTRAQ Ratio 115/114*	iTRAQ Ratio 116/114*	iTRAQ Ratio 117/114*	Rank	Result Type
1601.7527	1601.6901	-0.0626	-39	56	70	EPGEAAAEGAAEEAR	42	99.859	(N-term)_iTRAQ[0]	[1] F3 030912	167/159	1.082	1.081	0.914	1	Mascot

828	ras and Rab interactor 1 [Rattus norvegicus]	gi|20514768	92002.1	1	42	0.988	1.273	1.341	0.000	0.000	0.000	1	1	1	99.858

Protein Group

RecName: Full=Ras and Rab interactor 1; AltName: Full=Ras interaction/interference protein 1	gi|28202254	89018.6

Peptide Information

Calc. Mass	Obsrv. Mass	± da	± ppm	Start Seq.	End Seq.	Sequence	Ion Score	C. I. %	Modification	Plate [#] Name	Gel Idx/Pos [4700 Sample Name]	iTRAQ Ratio 115/114*	iTRAQ Ratio 116/114*	iTRAQ Ratio 117/114*	Rank	Result Type
1833.9552	1833.8386	-0.1166	-64	766	779	SEIAVDQEGKDQAR	42	99.858	(N-term)_iTRAQ[0], Lysine(K)_iTRAQ[10]	[6] F8 110912	121/113	0.988	1.273	1.341	1	Mascot

829	ATP-citrate (pro-S-)-lyase [Rattus norvegicus]	gi|4007923	4418.7	1	42	0.581	0.725	0.698	0.000	0.000	0.000	1	1	1	99.848

Peptide Information

Calc. Mass	Obsrv. Mass	± da	± ppm	Start Seq.	End Seq.	Sequence	Ion Score	C. I. %	Modification	Plate [#] Name	Gel Idx/Pos [4700 Sample Name]	iTRAQ Ratio 115/114*	iTRAQ Ratio 116/114*	iTRAQ Ratio 117/114*	Rank	Result Type
1712.0078	1712.0258	0.018	11	3	17	TLAIIAEGIPEALTR	42	99.848	(N-term)_iTRAQ[0]	[8] F13-15 and F1+2	363/355	0.581	0.725	0.698	1	Mascot

830	synaptoporin [Rattus norvegicus]	gi|13027428	31093.4	1	42	0.660	0.510	0.825	0.000	0.000	0.000	1	1	1	99.846

Peptide Information

Calc. Mass	Obsrv. Mass	± da	± ppm	Start Seq.	End Seq.	Sequence	Ion Score	C. I. %	Modification	Plate [#] Name	Gel Idx/Pos [4700 Sample Name]	iTRAQ Ratio 115/114*	iTRAQ Ratio 116/114*	iTRAQ Ratio 117/114*	Rank	Result Type
1921.9742	1921.9805	0.0063	3	51	64	LHQVTFEVPTCEGK	42	99.846	(N-term)_iTRAQ[0], Lysine(K)_iTRAQ[14], MMTS (C)[11]	[4] F7 and F10+11	338/330	0.660	0.510	0.825	1	Mascot

831	vitamin K epoxide reductase complex subunit 1-like protein 1 [Rattus norvegicus]	gi|42627869	21148.4	1	41	0.819	1.046	1.022	0.000	0.000	0.000	1	1	1	99.817

Peptide Information

Calc. Mass	Obsrv. Mass	± da	± ppm	Start Seq.	End Seq.	Sequence	Ion Score	C. I. %	Modification	Plate [#] Name	Gel Idx/Pos [4700 Sample Name]	iTRAQ Ratio 115/114*	iTRAQ Ratio 116/114*	iTRAQ Ratio 117/114*	Rank	Result Type
1383.8246	1383.845	0.0204	15	69	79	GFGLLGSIFGK	41	99.817	(N-term)_iTRAQ[0], Lysine(K)_iTRAQ[11]	[7] F5 120912	470/462	0.819	1.046	1.022	1	Mascot

832	peroxisomal multifunctional enzyme type 2 [Rattus norvegicus]	gi|162287198	86852.9	1	41	0.828	0.959	1.104	0.000	0.000	0.000	1	1	1	99.797

Protein Group

Chain A, (3r)-Hydroxyacyl-Coa Dehydrogenase Fragment Of Rat Peroxisomal Multifunctional Enzyme Type	gi|28373303	37132.3
Hsd17b4 protein [Rattus norvegicus]	gi|48734844	88512.8

	multifunctional protein 2 [Rattus norvegicus]	gi|1620451	86471.6

Peptide Information

Calc. Mass	Obsrv. Mass	± da	± ppm	Start Seq.	End Seq.	Sequence	Ion Score	C. I. %	Modification	Plate [#] Name	Gel Idx/Pos [4700 Sample Name]	iTRAQ Ratio 115/114*	iTRAQ Ratio 116/114*	iTRAQ Ratio 117/114*	Rank	Result Type
1640.8376	1640.7683	-0.0693	-42	69	81	AVANYDSVEAGEK	41	99.797	(N-term)_iTRAQ[0], Lysine(K)_iTRAQ[13]	[7] F5 120912	142/134	0.828	0.959	1.104	1	Mascot

833	adenine phosphoribosyltransferase [Rattus norvegicus]	gi|61556832	21302.5	1	41	0.883	0.679	0.780	0.000	0.000	0.000	1	1	1	99.796

Peptide Information

Calc. Mass	Obsrv. Mass	± da	± ppm	Start Seq.	End Seq.	Sequence	Ion Score	C. I. %	Modification	Plate [#] Name	Gel Idx/Pos [4700 Sample Name]	iTRAQ Ratio 115/114*	iTRAQ Ratio 116/114*	iTRAQ Ratio 117/114*	Rank	Result Type
1635.9019	1635.9008	-0.0011	-1	15	27	SFPDFPIPGVLFR	41	99.796	(N-term)_iTRAQ[0]	[8] F13-15 and F1+2	277/269	0.883	0.679	0.780	1	Mascot

834	MARCKS-related protein [Rattus norvegicus]	gi|13540687	22763.4	1	41	1.508	0.631	0.598	0.000	0.000	0.000	1	1	1	99.792

Peptide Information

Calc. Mass	Obsrv. Mass	± da	± ppm	Start Seq.	End Seq.	Sequence	Ion Score	C. I. %	Modification	Plate [#] Name	Gel Idx/Pos [4700 Sample Name]	iTRAQ Ratio 115/114*	iTRAQ Ratio 116/114*	iTRAQ Ratio 117/114*	Rank	Result Type
1661.8591	1661.7316	-0.1275	-77	11	25	GDVTAEEAAGASPAK	41	99.792	(N-term)_iTRAQ[0], Lysine(K)_iTRAQ[15]	[5] F4	77/69	1.508	0.631	0.598	1	Mascot

835	glyoxalase domain-containing protein 4 [Rattus norvegicus]	gi|62079189	36790.8	1	41	1.434	1.144	0.931	0.000	0.000	0.000	1	1	1	99.788

Peptide Information

Calc. Mass	Obsrv. Mass	± da	± ppm	Start Seq.	End Seq.	Sequence	Ion Score	C. I. %	Modification	Plate [#] Name	Gel Idx/Pos [4700 Sample Name]	iTRAQ Ratio 115/114*	iTRAQ Ratio 116/114*	iTRAQ Ratio 117/114*	Rank	Result Type
1716.8317	1716.7516	-0.0801	-47	172	183	WALLGYADDQCK	41	99.788	(N-term)_iTRAQ[0], Lysine(K)_iTRAQ[12], MMTS (C)[11]	[5] F4	306/298	1.434	1.144	0.931	1	Mascot

836	serine racemase [Rattus norvegicus]	gi|38454254	38828.7	1	40	0.670	0.648	0.641	0.000	0.000	0.000	1	1	1	99.779

Peptide Information

Calc. Mass	Obsrv. Mass	± da	± ppm	Start Seq.	End Seq.	Sequence	Ion Score	C. I. %	Modification	Plate [#] Name	Gel Idx/Pos [4700 Sample Name]	iTRAQ Ratio 115/114*	iTRAQ Ratio 116/114*	iTRAQ Ratio 117/114*	Rank	Result Type
2026.0477	2025.9772	-0.0705	-35	254	268	DLVDDVFTVTEDEIK	40	99.779	(N-term)_iTRAQ[0], Lysine(K)_iTRAQ[15]	[1] F3 030912	489/481	0.670	0.648	0.641	1	Mascot

837	pirin [Rattus norvegicus]	gi|57164095	35564.6	1	40	2.258	1.645	2.324	0.000	0.000	0.000	1	1	1	99.777

Peptide Information

Calc. Mass	Obsrv. Mass	± da	± ppm	Start Seq.	End Seq.	Sequence	Ion Score	C. I. %	Modification	Plate [#] Name	Gel Idx/Pos [4700 Sample Name]	iTRAQ Ratio 115/114*	iTRAQ Ratio 116/114*	iTRAQ Ratio 117/114*	Rank	Result Type
1802.9648	1802.8999	-0.0649	-36	35	46	MLDPFLLFDEFK	40	99.777	(N-term)_iTRAQ[0], Lysine(K)_iTRAQ[12]	[5] F4	431/423	2.258	1.645	2.324	1	Mascot

838	liver nuclear protein p47 [Rattus norvegicus]	gi|205942	50179.2	1	40	0.829	0.529	1.101	0.000	0.000	0.000	1	1	1	99.777

Protein Group

ATP-dependent RNA helicase DDX39A [Rattus norvegicus]	gi|55926219	53526.7
Bat1 [Rattus norvegicus]	gi|19773876	53634.9
nuclear RNA helicase [Rattus norvegicus]	gi|3132829	53355.6
spliceosome RNA helicase Ddx39b [Mus musculus]	gi|9790069	53694.9

Peptide Information

Calc. Mass	Obsrv. Mass	± da	± ppm	Start Seq.	End Seq.	Sequence	Ion Score	C. I. %	Modification	Plate [#] Name	Gel Idx/Pos [4700 Sample Name]	iTRAQ Ratio 115/114*	iTRAQ Ratio 116/114*	iTRAQ Ratio 117/114*	Rank	Result Type

1531.9458	1531.9417	-0.0041	-3	49	58	DFLLKPELLR	40	99.777	(N-term)_iTRAQ[0], Lysine(K)_iTRAQ[5]	[4] F7 and F10+11	378/370	0.829	0.529	1.101	1	Mascot

839	diacylglycerol kinase iota-2 [Rattus norvegicus]	gi|29466779	101087.5	1	40	0.689	0.729	0.603	0.000	0.000	0.000	1	1	1	99.777

Protein Group

diacylglycerol kinase iota-1 [Rattus norvegicus]	gi|29466777	125666

Peptide Information

Calc. Mass	Obsrv. Mass	± da	± ppm	Start Seq.	End Seq.	Sequence	Ion Score	C. I. %	Modification	Plate [#] Name	Gel Idx/Pos [4700 Sample Name]	iTRAQ Ratio 115/114*	iTRAQ Ratio 116/114*	iTRAQ Ratio 117/114*	Rank	Result Type
1650.7997	1650.6853	-0.1144	-69	783	794	VHYQDQETSFPR	40	99.777	(N-term)_iTRAQ[0]	[6] F8 110912	188/180	0.689	0.729	0.603	1	Mascot

840	CAP-Gly domain-containing linker protein 2 [Rattus norvegicus]	gi|11496271	127685.9	1	40	1.191	0.966	0.848	0.000	0.000	0.000	1	1	1	99.775

Peptide Information

Calc. Mass	Obsrv. Mass	± da	± ppm	Start Seq.	End Seq.	Sequence	Ion Score	C. I. %	Modification	Plate [#] Name	Gel Idx/Pos [4700 Sample Name]	iTRAQ Ratio 115/114*	iTRAQ Ratio 116/114*	iTRAQ Ratio 117/114*	Rank	Result Type
2390.2898	2390.2161	-0.0737	-31	48	68	QASGPSSAGATTTVSEK PGPK	40	99.775	(N-term)_iTRAQ[0], Lysine(K)_iTRAQ[17, 21]	[4] F7 and F10+11	123/115	1.191	0.966	0.848	1	Mascot

841	MAP/microtubule affinity-regulating kinase 3 [Rattus norvegicus]	gi|18543359	97029.5	1	40	1.048	1.364	0.716	0.000	0.000	0.000	1	1	1	99.771

Protein Group

serine/threonine-protein kinase MARK1 [Rattus norvegicus]	gi|16758824	96565.3
serine/threonine-protein kinase MARK2 [Rattus norvegicus]	gi|11067437	89167.1

Peptide Information

Calc. Mass	Obsrv. Mass	± da	± ppm	Start Seq.	End Seq.	Sequence	Ion Score	C. I. %	Modification	Plate [#] Name	Gel Idx/Pos [4700 Sample Name]	iTRAQ Ratio 115/114*	iTRAQ Ratio 116/114*	iTRAQ Ratio 117/114*	Rank	Result Type
1395.7981	1395.7418	-0.0563	-40	118	126	LFEVIETEK	40	99.771	(N-term)_iTRAQ[0], Lysine(K)_iTRAQ[9]	[5] F4	252/244	1.048	1.364	0.716	1	Mascot

842	RecName: Full=60S ribosomal protein L7	gi|51704206	36120.1	1	40	1.049	1.043	1.523	0.000	0.000	0.000	1	1	1	99.765

Peptide Information

Calc. Mass	Obsrv. Mass	± da	± ppm	Start Seq.	End Seq.	Sequence	Ion Score	C. I. %	Modification	Plate [#] Name	Gel Idx/Pos [4700 Sample Name]	iTRAQ Ratio 115/114*	iTRAQ Ratio 116/114*	iTRAQ Ratio 117/114*	Rank	Result Type
2260.0825	2260.1523	0.0698	31	236	254	TTHFVEGGDAGNREDQI NR	40	99.765	(N-term)_iTRAQ[0]	[4] F7 and F10+11	1048/1040	1.049	1.043	1.523	1	Mascot

843	aflatoxin B1 aldehyde reductase [Rattus norvegicus]	gi|27527649	39909.9	1	40	1.141	2.322	1.020	0.000	0.000	0.000	1	1	1	99.752

Protein Group

RecName: Full=Aflatoxin B1 aldehyde reductase member 2; Short=rAFAR2; AltName: Full=Succinic semial	gi|39930812	43034.6
aflatoxin B1 aldehyde reductase member 2 [Rattus norvegicus]	gi|19705537	39912.9
aldehyde reductase AFAR2 subunit [Rattus norvegicus]	gi|22652804	42904.4

Peptide Information

Calc. Mass	Obsrv. Mass	± da	± ppm	Start Seq.	End Seq.	Sequence	Ion Score	C. I. %	Modification	Plate [#] Name	Gel Idx/Pos [4700 Sample Name]	iTRAQ Ratio 115/114*	iTRAQ Ratio 116/114*	iTRAQ Ratio 117/114*	Rank	Result Type
1710.9425	1710.8866	-0.0559	-33	246	257	EHHFEAIALVEK	40	99.752	(N-term)_iTRAQ[0], Lysine(K)_iTRAQ[12]	[2] F12 040912	298/290	1.141	2.322	1.020	1	Mascot

844	sodium/potassium-transporting ATPase subunit beta-3 [Rattus norvegicus]	gi|6978553	35687.6	1	40	1.255	0.937	1.067	0.000	0.000	0.000	1	1	1	99.748

Peptide Information

Calc. Mass	Obsrv. Mass	± da	± ppm	Start Seq.	End Seq.	Sequence	Ion Score	C. I. %	Modification	Plate [#] Name	Gel Idx/Pos [4700 Sample Name]	iTRAQ Ratio 115/114*	iTRAQ Ratio 116/114*	iTRAQ Ratio 117/114*	Rank	Result Type

Calc. Mass	Obsrv. Mass	± da	± ppm	Start Seq.	End Seq.	Sequence	Ion Score	C. I. %	Modification	Plate [#] Name	Gel Idx/Pos [4700 Sample Name]	iTRAQ Ratio 115/114*	iTRAQ Ratio 116/114*	iTRAQ Ratio 117/114*	Rank	Result Type
1757.9346	1757.8761	-0.0585	-33	18	31	LFIYNPTSGEFLGR	40	99.748	(N-term)_iTRAQ[0]	[1] F3 030912	415/407	1.255	0.937	1.067	1	Mascot

845	low molecular weight phosphotyrosine protein phosphatase isoform A [Rattus norvegicus]	gi|10863989	20236.9	1	40	0.716	0.828	0.997	0.000	0.000	0.000	1	1	1	99.74

Protein Group

low M(r) phosphotyrosine protein phosphatase isoenzyme AcP1 [rats, liver, Peptide, 159 aa]	gi|257209	20325.9
low M(r) phosphotyrosine protein phosphatase isoenzyme AcP2 [rats, liver, Peptide, 159 aa]	gi|257210	19967.8
protein-tyrosine-phosphatase (EC 3.1.3.48) isoenzyme AcP2 - rat	gi|2117994	19747.8

Peptide Information

Calc. Mass	Obsrv. Mass	± da	± ppm	Start Seq.	End Seq.	Sequence	Ion Score	C. I. %	Modification	Plate [#] Name	Gel Idx/Pos [4700 Sample Name]	iTRAQ Ratio 115/114*	iTRAQ Ratio 116/114*	iTRAQ Ratio 117/114*	Rank	Result Type
1550.8676	1550.8073	-0.0603	-39	114	124	IELLGSYDPQK	40	99.74	(N-term)_iTRAQ[0], Lysine(K)_iTRAQ[11]	[5] F4	216/208	0.716	0.828	0.997	1	Mascot

846	glutaredoxin [Rattus norvegicus]	gi|12746263	13137.8	1	40	1.112	1.172	1.304	0.000	0.000	0.000	1	1	1	99.734

Protein Group

glutaredoxin-1 [Rattus norvegicus]	gi|11560105	13253.9

Peptide Information

Calc. Mass	Obsrv. Mass	± da	± ppm	Start Seq.	End Seq.	Sequence	Ion Score	C. I. %	Modification	Plate [#] Name	Gel Idx/Pos [4700 Sample Name]	iTRAQ Ratio 115/114*	iTRAQ Ratio 116/114*	iTRAQ Ratio 117/114*	Rank	Result Type
1864.1279	1864.1884	0.0605	32	28	39	KTQEILSQLPFK	40	99.734	(N-term)_iTRAQ[0], Lysine(K)_iTRAQ[1,12]	[4] F7 and F10+11	1237/1229	1.112	1.172	1.304	1	Mascot

847	ras GTPase-activating protein-binding protein 2 [Rattus norvegicus]	gi|62078707	53811.2	1	40	0.389	0.663	0.870	0.000	0.000	0.000	1	1	1	99.729

Peptide Information

Calc. Mass	Obsrv. Mass	± da	± ppm	Start Seq.	End Seq.	Sequence	Ion Score	C. I. %	Modification	Plate [#] Name	Gel Idx/Pos [4700 Sample Name]	iTRAQ Ratio 115/114*	iTRAQ Ratio 116/114*	iTRAQ Ratio 117/114*	Rank	Result Type
1738.9697	1738.9094	-0.0603	-35	245	257	VDAKPEVQSQPPR	40	99.729	(N-term)_iTRAQ[0], Lysine(K)_iTRAQ[4]	[4] F7 and F10+11	129/121	0.389	0.663	0.870	1	Mascot

848	prostaglandin E synthase 3 [Rattus norvegicus]	gi|62079275	19444.1	1	40	0.822	0.920	1.005	0.000	0.000	0.000	1	1	1	99.725

Peptide Information

Calc. Mass	Obsrv. Mass	± da	± ppm	Start Seq.	End Seq.	Sequence	Ion Score	C. I. %	Modification	Plate [#] Name	Gel Idx/Pos [4700 Sample Name]	iTRAQ Ratio 115/114*	iTRAQ Ratio 116/114*	iTRAQ Ratio 117/114*	Rank	Result Type
1823.969	1823.9702	0.0012	1	85	96	LNWLSVDFNNWK	40	99.725	(N-term)_iTRAQ[0], Lysine(K)_iTRAQ[12]	[3] F6 and F9	489/481	0.822	0.920	1.005	1	Mascot

849	exocyst complex component 8 [Rattus norvegicus]	gi|20514778	86875.8	1	39	1.142	0.700	0.830	0.000	0.000	0.000	1	1	1	99.708

Peptide Information

Calc. Mass	Obsrv. Mass	± da	± ppm	Start Seq.	End Seq.	Sequence	Ion Score	C. I. %	Modification	Plate [#] Name	Gel Idx/Pos [4700 Sample Name]	iTRAQ Ratio 115/114*	iTRAQ Ratio 116/114*	iTRAQ Ratio 117/114*	Rank	Result Type
1719.9528	1719.8739	-0.0789	-46	263	273	EWLEVLEETKR	40	99.777	(N-term)_iTRAQ[0], Lysine(K)_iTRAQ[10]	[6] F8 110912	403/395	1.142	0.700	0.830	1	Mascot

850	40S ribosomal protein S13 [Homo sapiens]	gi|4506685	19949.6	1	39	1.438	1.163	0.983	0.000	0.000	0.000	1	1	1	99.705

Peptide Information

Calc. Mass	Obsrv. Mass	± da	± ppm	Start Seq.	End Seq.	Sequence	Ion Score	C. I. %	Modification	Plate [#] Name	Gel Idx/Pos [4700 Sample Name]	iTRAQ Ratio 115/114*	iTRAQ Ratio 116/114*	iTRAQ Ratio 117/114*	Rank	Result Type
1982.1208	1982.0842	-0.0366	-18	79	93	GLAPDLPEDLYHLIK	39	99.705	(N-term)_iTRAQ[0], Lysine(K)_iTRAQ[15]	[3] F6 and F9	474/466	1.438	1.163	0.983	1	Mascot

851	hypothetical protein LOC500956 [Rattus norvegicus]	gi|68163533	21289.9	1	39	0.740	0.812	0.562	0.000	0.000	0.000	1	1	1	99.704

Peptide Information

Calc. Mass	Obsrv. Mass	± da	± ppm	Start	End	Sequence	Ion	C. I. %	Modification	Plate [#] Name	Gel Idx/Pos [4700	iTRAQ	iTRAQ	iTRAQ	Rank	Result Type

				Seq.	Seq.		Score				Sample Name]	Ratio 115/114*	Ratio 116/114*	Ratio 117/114*		
1652.7979	1652.7955	-0.0024	-1	147	157	MWGLAEFHCPK	39	99.704	(N-term)_iTRAQ[0], Lysine(K)_iTRAQ[11], MMTS (C)[9]	[3] F6 and F9	1360/1352	0.740	0.812	0.562	1	Mascot

852	SEC14-like protein 2 [Rattus norvegicus]	gi|60097947	51731.2	1	39	0.987	0.827	0.387	0.000	0.000	0.000	1	1	1	99.702

Protein Group

RecName: Full=SEC14-like protein 2; AltName: Full=Alpha-tocopherol-associated protein; Short=TAP; A	gi|21542226	51692.1

Peptide Information

Calc. Mass	Obsrv. Mass	± da	± ppm	Start Seq.	End Seq.	Sequence	Ion Score	C. I. %	Modification	Plate [#] Name	Gel Idx/Pos [4700 Sample Name]	iTRAQ Ratio 115/114*	iTRAQ Ratio 116/114*	iTRAQ Ratio 117/114*	Rank	Result Type
1488.76	1488.679	-0.081	-54	330	341	AGEMTEVLPNQR	39	99.702	(N-term)_iTRAQ[0]	[1] F3 030912	205/197	0.987	0.827	0.387	1	Mascot

853	proteasome subunit beta type-2 [Rattus norvegicus]	gi|8394079	24764.9	1	39	0.955	1.164	0.932	0.037	0.210	0.129	2	2	2	99.691

Peptide Information

Calc. Mass	Obsrv. Mass	± da	± ppm	Start Seq.	End Seq.	Sequence	Ion Score	C. I. %	Modification	Plate [#] Name	Gel Idx/Pos [4700 Sample Name]	iTRAQ Ratio 115/114*	iTRAQ Ratio 116/114*	iTRAQ Ratio 117/114*	Rank	Result Type
1450.8542	1450.8594	0.0052	4	171	181	FILNLPTFSVR	31	98.174	(N-term)_iTRAQ[0]	[8] F13-15 and F1+2	341/333	0.992	0.986	1.062	1	Mascot
1450.8542	1450.8622	0.008	6	171	181	FILNLPTFSVR	39	99.691	(N-term)_iTRAQ[0]	[8] F13-15 and F1+2	344/336	0.919	1.374	0.819	1	Mascot

854	3-hydroxyisobutyryl-CoA hydrolase, mitochondrial [Rattus norvegicus]	gi|61556993	37713.2	1	39	1.321	1.165	1.377	0.022	0.533	0.033	2	2	2	99.678

Peptide Information

Calc. Mass	Obsrv. Mass	± da	± ppm	Start Seq.	End Seq.	Sequence	Ion Score	C. I. %	Modification	Plate [#] Name	Gel Idx/Pos [4700 Sample Name]	iTRAQ Ratio 115/114*	iTRAQ Ratio 116/114*	iTRAQ Ratio 117/114*	Rank	Result Type
1695.0302	1694.9745	-0.0557	-33	221	232	LHVLEEELLALK	35	99.161	(N-term)_iTRAQ[0], Lysine(K)_iTRAQ[12]	[6] F8 110912	486/478	1.343	1.697	1.410	1	Mascot
1695.0302	1695.051	0.0208	12	221	232	LHVLEEELLALK	39	99.678	(N-term)_iTRAQ[0], Lysine(K)_iTRAQ[12]	[4] F7 and F10+11	486/478	1.299	0.799	1.344	1	Mascot

855	40S ribosomal protein S20 isoform 2 [Homo sapiens]	gi|4506697	15473.7	1	39	1.435	0.874	1.725	0.000	0.000	0.000	1	1	1	99.668

Peptide Information

Calc. Mass	Obsrv. Mass	± da	± ppm	Start Seq.	End Seq.	Sequence	Ion Score	C. I. %	Modification	Plate [#] Name	Gel Idx/Pos [4700 Sample Name]	iTRAQ Ratio 115/114*	iTRAQ Ratio 116/114*	iTRAQ Ratio 117/114*	Rank	Result Type
1391.7767	1391.7281	-0.0486	-35	9	19	TPVEPEVAIHR	39	99.668	(N-term)_iTRAQ[0]	[3] F6 and F9	189/181	1.435	0.874	1.725	1	Mascot

856	glycine cleavage system H protein, mitochondrial precursor [Rattus norvegicus]	gi|126723054	20386.6	1	38	1.116	0.942	1.018	0.000	0.000	0.000	1	1	1	99.649

Peptide Information

Calc. Mass	Obsrv. Mass	± da	± ppm	Start Seq.	End Seq.	Sequence	Ion Score	C. I. %	Modification	Plate [#] Name	Gel Idx/Pos [4700 Sample Name]	iTRAQ Ratio 115/114*	iTRAQ Ratio 116/114*	iTRAQ Ratio 117/114*	Rank	Result Type
1547.7465	1547.7137	-0.0328	-21	134	143	SCYEDGWLIK	38	99.649	(N-term)_iTRAQ[0], Lysine(K)_iTRAQ[10], MMTS (C)[2]	[7] F5 120912	330/322	1.116	0.942	1.018	1	Mascot

857	electron transfer flavoprotein-ubiquinone oxidoreductase, mitochondrial precursor [Rattus norvegicu	gi|52138635	75006.9	1	38	0.568	0.676	0.722	0.000	0.000	0.000	1	1	1	99.631

Protein Group

RecName: Full=Electron transfer flavoprotein-ubiquinone oxidoreductase, mitochondrial; Short=ETF-QO	gi|52000614	75040.9

Peptide Information

Calc. Mass	Obsrv. Mass	± da	± ppm	Start Seq.	End Seq.	Sequence	Ion Score	C. I. %	Modification	Plate [#] Name	Gel Idx/Pos [4700 Sample Name]	iTRAQ Ratio 115/114*	iTRAQ Ratio 116/114*	iTRAQ Ratio 117/114*	Rank	Result Type
1435.7301	1435.6556	-0.0745	-52	548	558	NLSIYDGPEQR	39	99.718	(N-term)_iTRAQ[0]	[1] F3 030912	207/199	0.568	0.676	0.722	1	Mascot

858	ATPase, H+ transporting, lysosomal accessory protein 1 [Rattus norvegicus]	gi|47718012	53036.5	1	38	0.704	0.750	0.781	0.000	0.000	0.000	1	1	1	99.63

Peptide Information

Calc. Mass	Obsrv. Mass	± da	± ppm	Start Seq.	End Seq.	Sequence	Ion Score	C. I. %	Modification	Plate [#] Name	Gel Idx/Pos [4700 Sample Name]	iTRAQ Ratio 115/114*	iTRAQ Ratio 116/114*	iTRAQ Ratio 117/114*	Rank	Result Type
1973.0953	1973.1277	0.0324	16	449	463	FDDHKGPTITLTQIV	38	99.63	(N-term)_iTRAQ[0], Lysine(K)_iTRAQ[5]	[4] F7 and F10+11	371/363	0.704	0.750	0.781	1	Mascot

859	Cc2-27 [Rattus norvegicus]	gi|33086672	132122.2	1	38	1.507	0.790	0.808	0.000	0.000	0.000	1	1	1	99.625

Peptide Information

Calc. Mass	Obsrv. Mass	± da	± ppm	Start Seq.	End Seq.	Sequence	Ion Score	C. I. %	Modification	Plate [#] Name	Gel Idx/Pos [4700 Sample Name]	iTRAQ Ratio 115/114*	iTRAQ Ratio 116/114*	iTRAQ Ratio 117/114*	Rank	Result Type
1994.0845	1994.0389	-0.0456	-23	578	591	NPELQNLLLDDFFK	38	99.625	(N-term)_iTRAQ[0], Lysine(K)_iTRAQ[14]	[5] F4	399/391	1.507	0.790	0.808	1	Mascot

860	synaptogyrin	gi|1587032	26906.9	1	38	0.838	1.009	0.953	0.000	0.000	0.000	1	1	1	99.618

Protein Group

synaptogyrin-1 [Rattus norvegicus]	gi|9507167	26845

Peptide Information

Calc. Mass	Obsrv. Mass	± da	± ppm	Start Seq.	End Seq.	Sequence	Ion Score	C. I. %	Modification	Plate [#] Name	Gel Idx/Pos [4700 Sample Name]	iTRAQ Ratio 115/114*	iTRAQ Ratio 116/114*	iTRAQ Ratio 117/114*	Rank	Result Type
1410.7501	1410.6917	-0.0584	-41	11	22	AGGAFDPYTLVR	38	99.618	(N-term)_iTRAQ[0]	[1] F3 030912	319/311	0.838	1.009	0.953	1	Mascot

861	mitochondrial import inner membrane translocase subunit Tim8 A [Rattus norvegicus]	gi|16758098	12228	1	38	0.905	0.563	0.921	0.000	0.000	0.000	1	1	1	99.604

Peptide Information

Calc. Mass	Obsrv. Mass	± da	± ppm	Start Seq.	End Seq.	Sequence	Ion Score	C. I. %	Modification	Plate [#] Name	Gel Idx/Pos [4700 Sample Name]	iTRAQ Ratio 115/114*	iTRAQ Ratio 116/114*	iTRAQ Ratio 117/114*	Rank	Result Type
1497.8186	1497.7858	-0.0328	-22	70	80	FIDTSQFILNR	38	99.604	(N-term)_iTRAQ[0]	[1] F3 030912	363/355	0.905	0.563	0.921	1	Mascot

862	bifunctional coenzyme A synthase [Rattus norvegicus]	gi|55741469	65837.1	1	38	1.061	1.391	0.772	0.000	0.000	0.000	1	1	1	99.604

Peptide Information

Calc. Mass	Obsrv. Mass	± da	± ppm	Start Seq.	End Seq.	Sequence	Ion Score	C. I. %	Modification	Plate [#] Name	Gel Idx/Pos [4700 Sample Name]	iTRAQ Ratio 115/114*	iTRAQ Ratio 116/114*	iTRAQ Ratio 117/114*	Rank	Result Type
1161.6877	1161.6478	-0.0399	-34	547	553	AWDLLQK	38	99.604	(N-term)_iTRAQ[0], Lysine(K)_iTRAQ[7]	[3] F6 and F9	311/303	1.061	1.391	0.772	1	Mascot

863	lupus La protein homolog [Rattus norvegicus]	gi|52138613	56108.7	1	38	0.870	1.270	0.865	0.000	0.000	0.000	1	1	1	99.586

Protein Group

RecName: Full=Lupus La protein homolog; AltName: Full=La autoantigen homolog; AltName: Full=La ribo	gi|729919	56099.7

Peptide Information

Calc. Mass	Obsrv. Mass	± da	± ppm	Start Seq.	End Seq.	Sequence	Ion Score	C. I. %	Modification	Plate [#] Name	Gel Idx/Pos [4700 Sample Name]	iTRAQ Ratio 115/114*	iTRAQ Ratio 116/114*	iTRAQ Ratio 117/114*	Rank	Result Type
1800.1007	1800.1407	0.04	22	175	185	YKDTNLLILFK	38	99.586	(N-term)_iTRAQ[0], Lysine(K)_iTRAQ[2,11]	[3] F6 and F9	1357/1349	0.870	1.270	0.865	1	Mascot

864	Ribosomal protein S11 [Rattus norvegicus]	gi|34849855	21743.2	1	38	1.254	1.375	1.408	0.000	0.000	0.000	1	1	1	99.584

Protein Group

40S ribosomal protein S11 [Homo sapiens]	gi|4506681	21727.2

Peptide Information

Calc. Mass	Obsrv. Mass	± da	± ppm	Start Seq.	End Seq.	Sequence	Ion Score	C. I. %	Modification	Plate [#] Name	Gel Idx/Pos [4700 Sample Name]	iTRAQ Ratio 115/114*	iTRAQ Ratio 116/114*	iTRAQ Ratio 117/114*	Rank	Result Type
1263.7307	1263.6689	-0.0618	-49	13	20	QPTIFQNK	38	99.584	(N-term)_iTRAQ[0], Lysine(K)_iTRAQ[8]	[3] F6 and F9	196/188	1.254	1.375	1.408	1	Mascot

865	transcription elongation factor B polypeptide 1 isoform a [Homo sapiens]	gi|5032161	13611.7	1	38	1.114	1.259	1.167	0.000	0.000	0.000	1	1	1	99.578

Peptide Information

Calc. Mass	Obsrv. Mass	± da	± ppm	Start Seq.	End Seq.	Sequence	Ion Score	C. I. %	Modification	Plate [#] Name	Gel Idx/Pos [4700 Sample Name]	iTRAQ Ratio 115/114*	iTRAQ Ratio 116/114*	iTRAQ Ratio 117/114*	Rank	Result Type
1824.8197	1824.7079	-0.1118	-61	7	20	TYGGCEGPDAMYVK	38	99.578	(N-term)_iTRAQ[0], Lysine(K)_iTRAQ[14], MMTS (C)[5]	[5] F4	188/180	1.114	1.259	1.167	1	Mascot

866	protein NipSnap homolog 3A [Rattus norvegicus]	gi|57164107	30431.8	1	38	1.252	0.780	0.539	0.000	0.000	0.000	1	1	1	99.577		

Peptide Information

Calc. Mass	Obsrv. Mass	± da	± ppm	Start Seq.	End Seq.	Sequence	Ion Score	C. I. %	Modification	Plate [#] Name	Gel Idx/Pos [4700 Sample Name]	iTRAQ Ratio 115/114*	iTRAQ Ratio 116/114*	iTRAQ Ratio 117/114*	Rank	Result Type
1728.8578	1728.8992	0.0414	24	194	206	VHVLWWNDSADSR	38	99.577	(N-term)_iTRAQ[0]	[3] F6 and F9	1291/1283	1.252	0.780	0.539	1	Mascot

867	Pde4b protein [Rattus norvegicus]	gi|55716045	88263.7	1	38	1.262	0.787	0.997	0.000	0.000	0.000	1	1	1	99.576

Protein Group

cAMP phosphodiesterase [Rattus norvegicus]	gi|203968	69457
cAMP-specific 3',5'-cyclic phosphodiesterase 4B [Rattus norvegicus]	gi|19923680	88560.9
cAMP-specific phosphodiesterase isoform PDE4B1 [Rattus norvegicus]	gi|16930143	89277.7
cAMP-specific phosphodiesterase isoform PDE4B4 [Rattus norvegicus]	gi|16930145	80279.2

Peptide Information

Calc. Mass	Obsrv. Mass	± da	± ppm	Start Seq.	End Seq.	Sequence	Ion Score	C. I. %	Modification	Plate [#] Name	Gel Idx/Pos [4700 Sample Name]	iTRAQ Ratio 115/114*	iTRAQ Ratio 116/114*	iTRAQ Ratio 117/114*	Rank	Result Type
1498.7999	1498.7518	-0.0481	-32	193	202	VSLQEESYQK	38	99.576	(N-term)_iTRAQ[0], Lysine(K)_iTRAQ[10]	[7] F5 120912	150/142	1.262	0.787	0.997	1	Mascot

868	plakoglobin [Rattus norvegicus]	gi|1497985	87078.9	1	38	1.430	0.760	1.159	0.000	0.000	0.000	1	1	1	99.573

Protein Group

junction plakoglobin [Rattus norvegicus]	gi|41529837	87102

Peptide Information

Calc. Mass	Obsrv. Mass	± da	± ppm	Start Seq.	End Seq.	Sequence	Ion Score	C. I. %	Modification	Plate [#] Name	Gel Idx/Pos [4700 Sample Name]	iTRAQ Ratio 115/114*	iTRAQ Ratio 116/114*	iTRAQ Ratio 117/114*	Rank	Result Type
1743.8898	1743.8022	-0.0876	-50	541	555	HVAAGTQQPYTDGVR	38	99.573	(N-term)_iTRAQ[0]	[4] F7 and F10+11	138/130	1.430	0.760	1.159	1	Mascot

869	protein phosphatase inhibitor 2 [Rattus norvegicus]	gi|41281691	25840.9	1	38	0.665	1.431	0.951	0.000	0.000	0.000	1	1	1	99.567

Peptide Information

Calc. Mass	Obsrv. Mass	± da	± ppm	Start Seq.	End Seq.	Sequence	Ion Score	C. I. %	Modification	Plate [#] Name	Gel Idx/Pos [4700 Sample Name]	iTRAQ Ratio 115/114*	iTRAQ Ratio 116/114*	iTRAQ Ratio 117/114*	Rank	Result Type
1761.0394	1761.1686	0.1292	73	145	155	KLHYNEGLNIK	38	99.567	(N-term)_iTRAQ[0], Lysine(K)_iTRAQ[1,11]	[8] F13-15 and F1+2	1531/1523	0.665	1.431	0.951	1	Mascot

870	receptor expression-enhancing protein 6 [Rattus norvegicus]	gi|61557326	24877.2	1	37	1.149	0.961	1.024	0.000	0.000	0.000	1	1	1	99.561

Peptide Information

Calc. Mass	Obsrv. Mass	± da	± ppm	Start Seq.	End Seq.	Sequence	Ion Score	C. I. %	Modification	Plate [#] Name	Gel Idx/Pos [4700 Sample Name]	iTRAQ Ratio 115/114*	iTRAQ Ratio 116/114*	iTRAQ Ratio 117/114*	Rank	Result Type
1273.8606	1273.916	0.0554	43	139	146	VIRPLFLK	37	99.561	(N-term)_iTRAQ[0], Lysine(K)_iTRAQ[8]	[4] F7 and F10+11	1207/1199	1.149	0.961	1.024	1	Mascot

871	long-chain-fatty-acid--CoA ligase ACSBG1 [Rattus norvegicus]	gi|19705503	86587.2	1	37	0.627	0.661	0.741	0.000	0.000	0.000	1	1	1	99.556

Peptide Information

Calc. Mass	Obsrv. Mass	± da	± ppm	Start Seq.	End Seq.	Sequence	Ion Score	C. I. %	Modification	Plate [#] Name	Gel Idx/Pos [4700 Sample Name]	iTRAQ Ratio 115/114*	iTRAQ Ratio 116/114*	iTRAQ Ratio 117/114*	Rank	Result Type

Calc. Mass	Obsrv. Mass	± da	± ppm	Start Seq.	End Seq.	Sequence	Ion Score	C. I. %	Modification	Plate [#] Name	Gel Idx/Pos [4700 Sample Name]	iTRAQ Ratio 115/114*	iTRAQ Ratio 116/114*	iTRAQ Ratio 117/114*	Rank	Result Type
1735.8922	1735.8333	-0.0589	-34	288	301	GVMLSQDNITWTAR	37	99.556	(N-term)_iTRAQ[0]	[1] F3 030912	303/295	0.627	0.661	0.741	1	Mascot

872 RecName: Full=GTPase KRas; AltName: Full=K-Ras 2; AltName: Full=Ki-Ras; AltName: Full=c-K-ras; AltN | gi|417590 | 24321.7 | 1 | 37 | 0.886 | 1.144 | 0.919 | 0.000 | 0.000 | 0.000 | 1 | 1 | 1 | 99.552

Protein Group

GTPase KRas [Rattus norvegicus]	gi|13928698	24546.9
GTPase NRas precursor [Rattus norvegicus]	gi|18158431	23332.8

Peptide Information

Calc. Mass	Obsrv. Mass	± da	± ppm	Start Seq.	End Seq.	Sequence	Ion Score	C. I. %	Modification	Plate [#] Name	Gel Idx/Pos [4700 Sample Name]	iTRAQ Ratio 115/114*	iTRAQ Ratio 116/114*	iTRAQ Ratio 117/114*	Rank	Result Type
1600.8832	1600.7971	-0.0861	-54	136	147	SYGIPFIETSAK	37	99.552	(N-term)_iTRAQ[0], Lysine(K)_iTRAQ[12]	[5] F4	248/240	0.886	1.144	0.919	1	Mascot

873 Txnrd1 protein [Rattus norvegicus] | gi|55250051 | 70327 | 1 | 37 | 5.615 | 3.996 | 2.982 | 0.000 | 0.000 | 0.000 | 1 | 1 | 1 | 99.551

Protein Group

thioredoxin reductase 1 [Rattus norvegicus]	gi|6942219	22246.2

Peptide Information

Calc. Mass	Obsrv. Mass	± da	± ppm	Start Seq.	End Seq.	Sequence	Ion Score	C. I. %	Modification	Plate [#] Name	Gel Idx/Pos [4700 Sample Name]	iTRAQ Ratio 115/114*	iTRAQ Ratio 116/114*	iTRAQ Ratio 117/114*	Rank	Result Type
1790.0006	1790.0205	0.0199	11	119	133	VMVLDFVTPTPLGTR	37	99.551	(N-term)_iTRAQ[0]	[8] F13-15 and F1+2	368/360	5.615	3.996	2.982	1	Mascot

874 protein tyrosine phosphatase [Rattus norvegicus] | gi|458333 | 75185.7 | 1 | 37 | 0.966 | 0.984 | 1.176 | 0.000 | 0.000 | 0.000 | 1 | 1 | 1 | 99.548

Protein Group

protein-tyrosine phosphatase [Rattus norvegicus]	gi|487402	74862.5

Peptide Information

Calc. Mass	Obsrv. Mass	± da	± ppm	Start Seq.	End Seq.	Sequence	Ion Score	C. I. %	Modification	Plate [#] Name	Gel Idx/Pos [4700 Sample Name]	iTRAQ Ratio 115/114*	iTRAQ Ratio 116/114*	iTRAQ Ratio 117/114*	Rank	Result Type
1608.8287	1608.7581	-0.0706	-44	583	594	VYENVGLMQQQR	39	99.655	(N-term)_iTRAQ[0]	[1] F3 030912	224/216	0.966	0.984	1.176	1	Mascot

875 RecName: Full=Peptidyl-prolyl cis-trans isomerase B; Short=PPIase B; AltName: Full=CYP-S1; AltName: | gi|215274190 | 27724.3 | 1 | 37 | 0.875 | 1.138 | 0.916 | 0.000 | 0.000 | 0.000 | 1 | 1 | 1 | 99.54

Protein Group

peptidyl-prolyl cis-trans isomerase B [Rattus norvegicus]	gi|11968126	26724.8

Peptide Information

Calc. Mass	Obsrv. Mass	± da	± ppm	Start Seq.	End Seq.	Sequence	Ion Score	C. I. %	Modification	Plate [#] Name	Gel Idx/Pos [4700 Sample Name]	iTRAQ Ratio 115/114*	iTRAQ Ratio 116/114*	iTRAQ Ratio 117/114*	Rank	Result Type
1430.6858	1430.636	-0.0498	-35	99	109	DFMIQGGDFTR	37	99.54	(N-term)_iTRAQ[0]	[1] F3 030912	284/276	0.875	1.138	0.916	1	Mascot

876 P70=70 kda seizure activity-linked albumin-like glycoprotein {N-terminal} [rats, brain, Peptide Partial, 20 aa] | gi|1311701 | 2956.6 | 1 | 37 | 4.877 | 1.078 | 1.194 | 0.000 | 0.000 | 0.000 | 1 | 1 | 1 | 99.531

Peptide Information

Calc. Mass	Obsrv. Mass	± da	± ppm	Start Seq.	End Seq.	Sequence	Ion Score	C. I. %	Modification	Plate [#] Name	Gel Idx/Pos [4700 Sample Name]	iTRAQ Ratio 115/114*	iTRAQ Ratio 116/114*	iTRAQ Ratio 117/114*	Rank	Result Type
1680.9445	1681.0175	0.073	43	11	20	FKDLGEQXFK	37	99.531	(N-term)_iTRAQ[0], Lysine(K)_iTRAQ[2,10]	[8] F13-15 and F1+2	1191/1183	4.877	1.078	1.194	1	Mascot

877 nicalin precursor [Rattus norvegicus] | gi|62078897 | 66018.5 | 1 | 37 | 1.502 | 1.770 | 0.788 | 0.000 | 0.000 | 0.000 | 1 | 1 | 1 | 99.526

Peptide Information

Calc. Mass	Obsrv. Mass	± da	± ppm	Start Seq.	End Seq.	Sequence	Ion Score	C. I. %	Modification	Plate [#] Name	Gel Idx/Pos [4700 Sample Name]	iTRAQ Ratio 115/114*	iTRAQ Ratio 116/114*	iTRAQ Ratio 117/114*	Rank	Result Type

Calc. Mass	Obsrv. Mass	± da	± ppm	Start Seq.	End Seq.	Sequence	Ion Score	C. I. %	Modification	Plate [#] Name	Gel Idx/Pos [4700 Sample Name]	iTRAQ Ratio 115/114*	iTRAQ Ratio 116/114*	iTRAQ Ratio 117/114*	Rank	Result Type
1824.9364	1824.978	0.0416	23	361	374	INLADDVLAWEHER	37	99.526	(N-term)_iTRAQ[0]	[4] F7 and F10+11	439/431	1.502	1.770	0.788	1	Mascot

878 LETM1 and EF-hand domain-containing protein 1, mitochondrial precursor [Rattus norvegicus] | gi\|54400736 | 93600.9 | 1 | 37 | 0.465 | 1.015 | 1.018 | 0.000 | 0.000 | 0.000 | 1 | 1 | 1 | 99.521

Peptide Information

Calc. Mass	Obsrv. Mass	± da	± ppm	Start Seq.	End Seq.	Sequence	Ion Score	C. I. %	Modification	Plate [#] Name	Gel Idx/Pos [4700 Sample Name]	iTRAQ Ratio 115/114*	iTRAQ Ratio 116/114*	iTRAQ Ratio 117/114*	Rank	Result Type
1951.0457	1950.9855	-0.0602	-31	419	433	AMYLPDTLSPADQLK	37	99.521	(N-term)_iTRAQ[0], Lysine(K)_iTRAQ[15]	[1] F3 030912	334/326	0.465	1.015	1.018	1	Mascot

879 mitochondrial import inner membrane translocase subunit TIM44 precursor [Rattus norvegicus] | gi\|8394449 | 57788.8 | 1 | 37 | 1.228 | 1.495 | 0.551 | 0.000 | 0.000 | 0.000 | 1 | 1 | 1 | 99.52

Peptide Information

Calc. Mass	Obsrv. Mass	± da	± ppm	Start Seq.	End Seq.	Sequence	Ion Score	C. I. %	Modification	Plate [#] Name	Gel Idx/Pos [4700 Sample Name]	iTRAQ Ratio 115/114*	iTRAQ Ratio 116/114*	iTRAQ Ratio 117/114*	Rank	Result Type
1737.0646	1737.12	0.0554	32	58	69	KGFLSGLLDNIK	37	99.52	(N-term)_iTRAQ[0], Lysine(K)_iTRAQ[1,12]	[4] F7 and F10+11	1308/1300	1.228	1.495	0.551	1	Mascot

880 60S ribosomal protein L22 [Mus musculus] | gi\|6677775 | 17677.8 | 1 | 37 | 0.331 | 0.619 | 0.328 | 0.000 | 0.000 | 0.000 | 1 | 1 | 1 | 99.517

Protein Group

60S ribosomal protein L22 [Rattus norvegicus] | gi\|13592059 | 17707.8

Peptide Information

Calc. Mass	Obsrv. Mass	± da	± ppm	Start Seq.	End Seq.	Sequence	Ion Score	C. I. %	Modification	Plate [#] Name	Gel Idx/Pos [4700 Sample Name]	iTRAQ Ratio 115/114*	iTRAQ Ratio 116/114*	iTRAQ Ratio 117/114*	Rank	Result Type
3216.4541	3216.3567	-0.0974	-30	21	46	FTLDCTHPVEDGIMDAANFEQFLQER	37	99.517	(N-term)_iTRAQ[0], MMTS (C)[5]	[1] F3 030912	544/536	0.331	0.619	0.328	1	Mascot

881 NADH dehydrogenase subunit 3 [Rattus norvegicus] | gi\|110189670 | 13684.3 | 1 | 37 | 0.889 | 0.940 | 0.826 | 0.000 | 0.000 | 0.000 | 1 | 1 | 1 | 99.516

Protein Group

NADH dehydrogenase subunit 3 [Rattus norvegicus] | gi\|110189722 | 13700.3

unknown protein [Rattus norvegicus] | gi\|829026 | 13654.2

Peptide Information

Calc. Mass	Obsrv. Mass	± da	± ppm	Start Seq.	End Seq.	Sequence	Ion Score	C. I. %	Modification	Plate [#] Name	Gel Idx/Pos [4700 Sample Name]	iTRAQ Ratio 115/114*	iTRAQ Ratio 116/114*	iTRAQ Ratio 117/114*	Rank	Result Type
1804.7736	1804.7048	-0.0688	-38	34	48	ANPYECGFDPTSSAR	37	99.516	(N-term)_iTRAQ[0], MMTS (C)[6]	[1] F3 030912	271/263	0.889	0.940	0.826	1	Mascot

882 Ngef protein [Rattus norvegicus] | gi\|50927565 | 40359.2 | 1 | 37 | 0.552 | 1.647 | 1.506 | 0.000 | 0.000 | 0.000 | 1 | 1 | 1 | 99.514

Protein Group

Ngef protein [Rattus norvegicus] | gi\|60688157 | 46705.9

brain-enriched SH3-domain protein [Rattus norvegicus] | gi\|7689361 | 17100.8

Peptide Information

Calc. Mass	Obsrv. Mass	± da	± ppm	Start Seq.	End Seq.	Sequence	Ion Score	C. I. %	Modification	Plate [#] Name	Gel Idx/Pos [4700 Sample Name]	iTRAQ Ratio 115/114*	iTRAQ Ratio 116/114*	iTRAQ Ratio 117/114*	Rank	Result Type
2024.0409	2023.9368	-0.1041	-51	264	278	GWFPSSMTEEILNPK	37	99.514	(N-term)_iTRAQ[0], Lysine(K)_iTRAQ[15]	[5] F4	335/327	0.552	1.647	1.506	1	Mascot

883 F-actin-capping protein subunit alpha-2 [Mus musculus] | gi\|6671672 | 36254.8 | 1 | 37 | 0.388 | 0.814 | 0.968 | 0.000 | 0.000 | 0.000 | 1 | 1 | 1 | 99.513

Peptide Information

Calc. Mass	Obsrv. Mass	± da	± ppm	Start Seq.	End Seq.	Sequence	Ion Score	C. I. %	Modification	Plate [#] Name	Gel Idx/Pos [4700 Sample Name]	iTRAQ Ratio 115/114*	iTRAQ Ratio 116/114*	iTRAQ Ratio 117/114*	Rank	Result Type
2722.4373	2722.5088	0.0715	26	147	166	KVDGQQTIIACIESHQFQAK	37	99.513	(N-term)_iTRAQ[0], Lysine(K)_iTRAQ[1,20], MMTS (C)[11]	[4] F7 and F10+11	1282/1274	0.388	0.814	0.968	1	Mascot

884	Clybl protein [Rattus norvegicus]	gi|56972641	41333.3	1	37	2.250	2.751	1.458	0.000	0.000	0.000	1	1	1	99.512

Peptide Information

Calc. Mass	Obsrv. Mass	± da	± ppm	Start Seq.	End Seq.	Sequence	Ion Score	C. I. %	Modification	Plate [#] Name	Gel Idx/Pos [4700 Sample Name]	iTRAQ Ratio 115/114*	iTRAQ Ratio 116/114*	iTRAQ Ratio 117/114*	Rank	Result Type
1465.8301	1465.8057	-0.0244	-17	290	299	WAEELIAAFK	37	99.512	(N-term)_iTRAQ[0], Lysine(K)_iTRAQ[10]	[7] F5 120912	493/485	2.250	2.751	1.458	1	Mascot

885	phospholipase D3 [Rattus norvegicus]	gi|58865906	57182	1	37	1.551	1.934	1.600	0.000	0.000	0.000	1	1	1	99.493

Peptide Information

Calc. Mass	Obsrv. Mass	± da	± ppm	Start Seq.	End Seq.	Sequence	Ion Score	C. I. %	Modification	Plate [#] Name	Gel Idx/Pos [4700 Sample Name]	iTRAQ Ratio 115/114*	iTRAQ Ratio 116/114*	iTRAQ Ratio 117/114*	Rank	Result Type
1489.8036	1489.8519	0.0483	32	338	348	RFWPAIDDGLR	38	99.613	(N-term)_iTRAQ[0]	[4] F7 and F10+11	1230/1222	1.551	1.934	1.600	1	Mascot

886	cytochrome b-c1 complex subunit 6, mitochondrial [Rattus norvegicus]	gi|57164091	11799.7	1	37	0.898	0.954	1.137	0.000	0.000	0.000	1	1	1	99.473

Peptide Information

Calc. Mass	Obsrv. Mass	± da	± ppm	Start Seq.	End Seq.	Sequence	Ion Score	C. I. %	Modification	Plate [#] Name	Gel Idx/Pos [4700 Sample Name]	iTRAQ Ratio 115/114*	iTRAQ Ratio 116/114*	iTRAQ Ratio 117/114*	Rank	Result Type
2360.0276	2359.9817	-0.0459	-19	59	76	SQTEEDCTEELFDFLHAR	37	99.473	(N-term)_iTRAQ[0], MMTS (C)[7]	[7] F5 120912	492/484	0.898	0.954	1.137	1	Mascot

887	RecName: Full=Protein lin-7 homolog A; Short=Lin-7A; AltName: Full=Mammalian lin-seven protein 1; S	gi|59798090	28246.1	1	37	0.388	0.867	0.833	0.000	0.000	0.000	1	1	1	99.465

Protein Group

lin-7-Ba [Rattus norvegicus]	gi|3885830	22069.8
protein lin-7 homolog A [Rattus norvegicus]	gi|16758276	26787.3
protein lin-7 homolog C [Mus musculus]	gi|6755973	23929.9

Peptide Information

Calc. Mass	Obsrv. Mass	± da	± ppm	Start Seq.	End Seq.	Sequence	Ion Score	C. I. %	Modification	Plate [#] Name	Gel Idx/Pos [4700 Sample Name]	iTRAQ Ratio 115/114*	iTRAQ Ratio 116/114*	iTRAQ Ratio 117/114*	Rank	Result Type
1752.8901	1752.9771	0.087	50	92	107	ATVAAFAASEGHSHPR	37	99.465	(N-term)_iTRAQ[0]	[4] F7 and F10+11	1083/1075	0.388	0.867	0.833	1	Mascot

888	partitioning defective 3 homolog [Rattus norvegicus]	gi|13676839	161436.5	1	36	0.967	1.462	1.053	0.000	0.000	0.000	1	1	1	99.447

Peptide Information

Calc. Mass	Obsrv. Mass	± da	± ppm	Start Seq.	End Seq.	Sequence	Ion Score	C. I. %	Modification	Plate [#] Name	Gel Idx/Pos [4700 Sample Name]	iTRAQ Ratio 115/114*	iTRAQ Ratio 116/114*	iTRAQ Ratio 117/114*	Rank	Result Type
1631.8625	1631.8347	-0.0278	-17	408	420	LSQPPEQLDAHPR	36	99.447	(N-term)_iTRAQ[0]	[4] F7 and F10+11	178/170	0.967	1.462	1.053	1	Mascot

889	glutaredoxin-3 [Rattus norvegicus]	gi|78187979	42378.4	1	36	0.789	0.958	0.461	0.000	0.000	0.000	1	1	1	99.428

Peptide Information

Calc. Mass	Obsrv. Mass	± da	± ppm	Start Seq.	End Seq.	Sequence	Ion Score	C. I. %	Modification	Plate [#] Name	Gel Idx/Pos [4700 Sample Name]	iTRAQ Ratio 115/114*	iTRAQ Ratio 116/114*	iTRAQ Ratio 117/114*	Rank	Result Type
1866.0299	1865.9791	-0.0508	-27	82	94	YEISSVPTFLFFK	36	99.428	(N-term)_iTRAQ[0], Lysine(K)_iTRAQ[13]	[1] F3 030912	508/500	0.789	0.958	0.461	1	Mascot

890	actin-binding LIM protein 2 isoform b [Rattus norvegicus]	gi|48040491	70326.2	1	36	0.699	0.809	0.880	0.000	0.000	0.000	1	1	1	99.401

Peptide Information

Calc. Mass	Obsrv. Mass	± da	± ppm	Start Seq.	End Seq.	Sequence	Ion Score	C. I. %	Modification	Plate [#] Name	Gel Idx/Pos [4700 Sample Name]	iTRAQ Ratio 115/114*	iTRAQ Ratio 116/114*	iTRAQ Ratio 117/114*	Rank	Result Type
1576.7927	1576.7656	-0.0271	-17	499	509	MPGDSLYSQYK	36	99.401	(N-term)_iTRAQ[0], Lysine(K)_iTRAQ[11]	[7] F5 120912	214/206	0.699	0.809	0.880	1	Mascot

891	Chain A, Syntaxin 6	gi|22219301	13508.9	1	36	1.260	0.725	0.843	0.000	0.000	0.000	1	1	1	99.397

Protein Group

syntaxin-6 [Rattus norvegicus] gi|13928922 30669.9

Peptide Information

Calc. Mass	Obsrv. Mass	± da	± ppm	Start Seq.	End Seq.	Sequence	Ion Score	C. I. %	Modification	Plate [#] Name	Gel Idx/Pos [4700 Sample Name]	iTRAQ Ratio 115/114*	iTRAQ Ratio 116/114*	iTRAQ Ratio 117/114*	Rank	Result Type
1549.7618	1549.7026	-0.0592	-38	41	51	EEIDWTTNELR	36	99.397	(N-term)_iTRAQ[0]	[1] F3 030912	271/263	1.260	0.725	0.843	1	Mascot

892	Sf1 protein [Rattus norvegicus]	gi|51858916	58199.4	1	36	1.062	1.372	1.679	0.000	0.000	0.000	1	1	1	99.358

Protein Group

splicing factor 1 homolog [Rattus norvegicus] gi|3406779 21811.7

Peptide Information

Calc. Mass	Obsrv. Mass	± da	± ppm	Start Seq.	End Seq.	Sequence	Ion Score	C. I. %	Modification	Plate [#] Name	Gel Idx/Pos [4700 Sample Name]	iTRAQ Ratio 115/114*	iTRAQ Ratio 116/114*	iTRAQ Ratio 117/114*	Rank	Result Type
1931.0079	1930.9019	-0.106	-55	182	195	QGIETPEDQNDLRK	36	99.358	(N-term)_iTRAQ[0], Lysine(K)_iTRAQ[14]	[6] F8 110912	161/153	1.062	1.372	1.679	1	Mascot

893	CaBP1 [Rattus norvegicus]	gi|488838	52412.1	1	36	0.749	0.527	0.537	0.000	0.000	0.000	1	1	1	99.349

Protein Group

protein disulfide-isomerase A6 [Rattus norvegicus] gi|52345385 53951

Peptide Information

Calc. Mass	Obsrv. Mass	± da	± ppm	Start Seq.	End Seq.	Sequence	Ion Score	C. I. %	Modification	Plate [#] Name	Gel Idx/Pos [4700 Sample Name]	iTRAQ Ratio 115/114*	iTRAQ Ratio 116/114*	iTRAQ Ratio 117/114*	Rank	Result Type
1627.8199	1627.7687	-0.0512	-31	365	377	GSFSEQGINEFLR	36	99.349	(N-term)_iTRAQ[0]	[1] F3 030912	342/334	0.749	0.527	0.537	1	Mascot

894	RecName: Full=UDP-N-acetylglucosamine--peptide N-acetylglucosaminyltransferase 110 kDa subunit; Alt	gi|3914191	124181.4	1	36	0.715	0.836	0.499	0.000	0.000	0.000	1	1	1	99.337

Peptide Information

Calc. Mass	Obsrv. Mass	± da	± ppm	Start Seq.	End Seq.	Sequence	Ion Score	C. I. %	Modification	Plate [#] Name	Gel Idx/Pos [4700 Sample Name]	iTRAQ Ratio 115/114*	iTRAQ Ratio 116/114*	iTRAQ Ratio 117/114*	Rank	Result Type
1420.7869	1420.8125	0.0256	18	367	375	LQEALMHYK	36	99.337	(N-term)_iTRAQ[0], Lysine(K)_iTRAQ[9]	[3] F6 and F9	1238/1230	0.715	0.836	0.499	1	Mascot

895	disks large homolog 1 [Rattus norvegicus]	gi|6978763	109188.9	1	36	3.109	2.559	2.936	0.000	0.000	0.000	1	1	1	99.328

Protein Group

Chain A, Hetero-Tetrameric L27 (Lin-2, Lin-7) Domain Complexes As Organization Platforms Of Supra-M gi|48425374 7655.1

SAP97 protein gi|1095318 109160.9

Peptide Information

Calc. Mass	Obsrv. Mass	± da	± ppm	Start Seq.	End Seq.	Sequence	Ion Score	C. I. %	Modification	Plate [#] Name	Gel Idx/Pos [4700 Sample Name]	iTRAQ Ratio 115/114*	iTRAQ Ratio 116/114*	iTRAQ Ratio 117/114*	Rank	Result Type
1287.7181	1287.7672	0.0491	38	11	19	ALHLLEEYR	36	99.328	(N-term)_iTRAQ[0]	[3] F6 and F9	1265/1257	3.109	2.559	2.936	1	Mascot

896	neuroligin-2 precursor [Rattus norvegicus]	gi|16758898	95110.5	1	36	1.020	0.769	1.082	0.000	0.000	0.000	1	1	1	99.309

Peptide Information

Calc. Mass	Obsrv. Mass	± da	± ppm	Start Seq.	End Seq.	Sequence	Ion Score	C. I. %	Modification	Plate [#] Name	Gel Idx/Pos [4700 Sample Name]	iTRAQ Ratio 115/114*	iTRAQ Ratio 116/114*	iTRAQ Ratio 117/114*	Rank	Result Type
1267.6919	1267.6245	-0.0674	-53	41	50	FPVVNTAYGR	36	99.309	(N-term)_iTRAQ[0]	[1] F3 030912	238/230	1.020	0.769	1.082	1	Mascot

897	isoform of PSD-95/SAP90 [Rattus norvegicus]	gi|1517938	99203.2	1	35	0.865	1.188	0.890	0.000	0.000	0.000	1	1	1	99.268

Protein Group

disks large homolog 3 [Rattus norvegicus] gi|13928878 100713.9

Peptide Information

Calc. Mass	Obsrv. Mass	± da	± ppm	Start Seq.	End Seq.	Sequence	Ion Score	C. I. %	Modification	Plate [#] Name	Gel Idx/Pos [4700 Sample Name]	iTRAQ Ratio 115/114*	iTRAQ Ratio 116/114*	iTRAQ Ratio 117/114*	Rank	Result Type
1735.8425	1735.7744	-0.0681	-39	610	622	DFPGLSDDYYGAK	35	99.268	(N-term)_iTRAQ[0], Lysine(K)_iTRAQ[13]	[5] F4	206/198	0.865	1.188	0.890	1	Mascot

898	ubiquitin-conjugating enzyme E2 D3 isoform 1 [Homo sapiens]	gi|4507777	18157.2	1	35	1.938	1.814	1.850	0.000	0.000	0.000	1	1	1	99.254

Protein Group

ubiquitin-conjugating enzyme E2 D2 isoform 1 [Homo sapiens]	gi|4507775	18205.3
ubiquitin-conjugating enzyme E2 D2B [Rattus norvegicus]	gi|13591896	18083.2

Peptide Information

Calc. Mass	Obsrv. Mass	± da	± ppm	Start Seq.	End Seq.	Sequence	Ion Score	C. I. %	Modification	Plate [#] Name	Gel Idx/Pos [4700 Sample Name]	iTRAQ Ratio 115/114*	iTRAQ Ratio 116/114*	iTRAQ Ratio 117/114*	Rank	Result Type
1505.8574	1505.8068	-0.0506	-34	91	101	SQWSPALTISK	35	99.254	(N-term)_iTRAQ[0], Lysine(K)_iTRAQ[11]	[7] F5 120912	240/232	1.938	1.814	1.850	1	Mascot

899	Adpgk protein [Rattus norvegicus]	gi|60422768	46826	1	35	2.082	1.392	0.930	0.000	0.000	0.000	1	1	1	99.242

Peptide Information

Calc. Mass	Obsrv. Mass	± da	± ppm	Start Seq.	End Seq.	Sequence	Ion Score	C. I. %	Modification	Plate [#] Name	Gel Idx/Pos [4700 Sample Name]	iTRAQ Ratio 115/114*	iTRAQ Ratio 116/114*	iTRAQ Ratio 117/114*	Rank	Result Type
1797.9091	1797.9436	0.0345	19	28	40	SDLEEAFVHFMGK	35	99.242	(N-term)_iTRAQ[0], Lysine(K)_iTRAQ[13]	[4] F7 and F10+11	467/459	2.082	1.392	0.930	1	Mascot

900	protein phosphatase-2Ba [Rattus norvegicus]	gi|206307	8799.2	1	35	1.137	0.862	1.160	0.000	0.000	0.000	1	1	1	99.232

Protein Group

protein phosphatase-2Bb [Rattus norvegicus]	gi|206309	8763.2

Peptide Information

Calc. Mass	Obsrv. Mass	± da	± ppm	Start Seq.	End Seq.	Sequence	Ion Score	C. I. %	Modification	Plate [#] Name	Gel Idx/Pos [4700 Sample Name]	iTRAQ Ratio 115/114*	iTRAQ Ratio 116/114*	iTRAQ Ratio 117/114*	Rank	Result Type
1828.0466	1828.0464	-0.0002	0	58	71	KSQTTGFPSLITIF	35	99.232	(N-term)_iTRAQ[0], Lysine(K)_iTRAQ[1]	[7] F5 120912	473/465	1.137	0.862	1.160	1	Mascot

901	putative hydrolase RBBP9 [Rattus norvegicus]	gi|9625029	22704.5	1	35	0.770	0.609	0.446	0.000	0.000	0.000	1	1	1	99.179

Peptide Information

Calc. Mass	Obsrv. Mass	± da	± ppm	Start Seq.	End Seq.	Sequence	Ion Score	C. I. %	Modification	Plate [#] Name	Gel Idx/Pos [4700 Sample Name]	iTRAQ Ratio 115/114*	iTRAQ Ratio 116/114*	iTRAQ Ratio 117/114*	Rank	Result Type
1771.9	1771.8577	-0.0423	-24	164	176	GHFQNTEFHELIR	35	99.179	(N-term)_iTRAQ[0]	[2] F12 040912	288/280	0.770	0.609	0.446	1	Mascot

902	GMP reductase 1 [Rattus norvegicus]	gi|17105340	41290.2	1	35	0.706	0.394	0.289	0.000	0.000	0.000	1	1	1	99.171

Peptide Information

Calc. Mass	Obsrv. Mass	± da	± ppm	Start Seq.	End Seq.	Sequence	Ion Score	C. I. %	Modification	Plate [#] Name	Gel Idx/Pos [4700 Sample Name]	iTRAQ Ratio 115/114*	iTRAQ Ratio 116/114*	iTRAQ Ratio 117/114*	Rank	Result Type
1728.9615	1728.9355	-0.026	-15	299	313	GDVENTILDILGGLR	35	99.171	(N-term)_iTRAQ[0]	[1] F3 030912	564/556	0.706	0.394	0.289	1	Mascot

903	phospholipid hydroperoxide glutathione peroxidase [Rattus norvegicus]	gi|1041645	21915.6	1	35	1.462	1.070	0.923	0.000	0.000	0.000	1	1	1	99.165

Protein Group

nucleolar phospholipid hydroperoxide glutathione peroxidase [Rattus norvegicus]	gi|34850331	32055.2
phospholipid hydroperoxide glutathione peroxidase [Rattus norvegicus]	gi|28411222	32144.3
phospholipid hydroperoxide glutathione peroxidase [Rattus norvegicus]	gi|14717812	21955.6
phospholipid hydroperoxide glutathione peroxidase [Rattus norvegicus]	gi|14971097	32299.4

phospholipid hydroperoxide glutathione peroxidase [Rattus norvegicus]	gi|28411221	21971.6
phospholipid hydroperoxide glutathione peroxidase [Rattus norvegicus]	gi|14717786	24894.2
sperm nucleus phospholipid-hydroperoxide glutathione peroxidase [Rattus norvegicus]	gi|45758901	32101.3

Peptide Information

Calc. Mass	Obsrv. Mass	± da	± ppm	Start Seq.	End Seq.	Sequence	Ion Score	C. I. %	Modification	Plate [#] Name	Gel Idx/Pos [4700 Sample Name]	iTRAQ Ratio 115/114*	iTRAQ Ratio 116/114*	iTRAQ Ratio 117/114*	Rank	Result Type
1707.8873	1707.7903	-0.097	-57	153	164	YGPMEEPQVIEK	35	99.165	(N-term)_iTRAQ[0], Lysine(K)_iTRAQ[12]	[1] F3 030912	240/232	1.462	1.070	0.923	1	Mascot

904	Methylenetetrahydrofolate dehydrogenase (NADP+ dependent) 1, methenyltetrahydrofolate cyclohydrolas	gi|59808745	110544.3	1	35	1.093	0.499	1.038	0.000	0.000	0.000	1	1	1	99.164	

Protein Group

C-1-tetrahydrofolate synthase, cytoplasmic [Rattus norvegicus]	gi|11968082	110562.3

Peptide Information

Calc. Mass	Obsrv. Mass	± da	± ppm	Start Seq.	End Seq.	Sequence	Ion Score	C. I. %	Modification	Plate [#] Name	Gel Idx/Pos [4700 Sample Name]	iTRAQ Ratio 115/114*	iTRAQ Ratio 116/114*	iTRAQ Ratio 117/114*	Rank	Result Type
1240.6936	1240.6554	-0.0382	-31	303	309	WTIQYNK	36	99.362	(N-term)_iTRAQ[0], Lysine(K)_iTRAQ[7]	[3] F6 and F9	234/226	1.093	0.499	1.038	1	Mascot

905	staphylococcal nuclease domain-containing protein 1 [Rattus norvegicus]	gi|77404395	110798.2	1	35	2.422	0.757	2.491	0.000	0.000	0.000	1	1	1	99.163

Peptide Information

Calc. Mass	Obsrv. Mass	± da	± ppm	Start Seq.	End Seq.	Sequence	Ion Score	C. I. %	Modification	Plate [#] Name	Gel Idx/Pos [4700 Sample Name]	iTRAQ Ratio 115/114*	iTRAQ Ratio 116/114*	iTRAQ Ratio 117/114*	Rank	Result Type
1826.9156	1826.916	0.0004	0	715	730	SDISSHPPVEGAYAPR	35	99.163	(N-term)_iTRAQ[0]	[3] F6 and F9	188/180	2.422	0.757	2.491	1	Mascot

906	OCIA domain-containing protein 1 [Rattus norvegicus]	gi|62078483	30563.7	1	35	1.033	1.131	0.917	0.000	0.000	0.000	1	1	1	99.158

Peptide Information

Calc. Mass	Obsrv. Mass	± da	± ppm	Start Seq.	End Seq.	Sequence	Ion Score	C. I. %	Modification	Plate [#] Name	Gel Idx/Pos [4700 Sample Name]	iTRAQ Ratio 115/114*	iTRAQ Ratio 116/114*	iTRAQ Ratio 117/114*	Rank	Result Type
1785.0394	1784.942	-0.0974	-55	122	133	SLPPGHYTQKPK	35	99.158	(N-term)_iTRAQ[0], Lysine(K)_iTRAQ[10, 12]	[2] F12 040912	133/125	1.033	1.131	0.917	1	Mascot

907	ZW10 interactor [Rattus norvegicus]	gi|22129759	35017.1	1	35	1.016	0.980	1.138	0.000	0.000	0.000	1	1	1	99.152

Peptide Information

Calc. Mass	Obsrv. Mass	± da	± ppm	Start Seq.	End Seq.	Sequence	Ion Score	C. I. %	Modification	Plate [#] Name	Gel Idx/Pos [4700 Sample Name]	iTRAQ Ratio 115/114*	iTRAQ Ratio 116/114*	iTRAQ Ratio 117/114*	Rank	Result Type
1217.7252	1217.6676	-0.0576	-47	177	183	QWVLQQK	35	99.152	(N-term)_iTRAQ[0], Lysine(K)_iTRAQ[7]	[3] F6 and F9	214/206	1.016	0.980	1.138	1	Mascot

908	Cyclic Protein-2 [Rattus sp.]	gi|246148	30840.2	1	35	1.650	1.246	2.150	0.000	0.000	0.000	1	1	1	99.144

Protein Group

RecName: Full=Cathepsin L1; AltName: Full=Cyclic protein 2; Short=CP-2; AltName: Full=Major excrete	gi|115743	41698.5
cathepsin L1 preproprotein [Rattus norvegicus]	gi|6978723	41724.5

Peptide Information

Calc. Mass	Obsrv. Mass	± da	± ppm	Start Seq.	End Seq.	Sequence	Ion Score	C. I. %	Modification	Plate [#] Name	Gel Idx/Pos [4700 Sample Name]	iTRAQ Ratio 115/114*	iTRAQ Ratio 116/114*	iTRAQ Ratio 117/114*	Rank	Result Type
1386.6974	1386.6764	-0.021	-15	218	226	EWGMDGYIK	35	99.144	(N-term)_iTRAQ[0], Lysine(K)_iTRAQ[9]	[3] F6 and F9	286/278	1.650	1.246	2.150	1	Mascot

909	methylcrotonoyl-CoA carboxylase subunit alpha, mitochondrial [Rattus norvegicus]	gi|57528264	85705.7	1	34	1.262	1.284	1.799	0.000	0.000	0.000	1	1	1	99.126

Peptide Information

Calc. Mass	Obsrv. Mass	± da	± ppm	Start Seq.	End Seq.	Sequence	Ion Score	C. I. %	Modification	Plate [#] Name	Gel Idx/Pos [4700 Sample Name]	iTRAQ Ratio 115/114*	iTRAQ Ratio 116/114*	iTRAQ Ratio 117/114*	Rank	Result Type
1678.9463	1678.9011	-0.0452	-27	502	514	ESVCQAALGLILK	34	99.126	(N-term)_iTRAQ[0], Lysine(K)_iTRAQ[13], MMTS (C)[4]	[5] F4	422/414	1.262	1.284	1.799	1	Mascot

910	Cct6a protein [Rattus norvegicus]	gi|60688220	29082.5	1	34	1.393	1.131	1.080	0.000	0.000	0.000	1	1	1	99.112

Peptide Information

Calc. Mass	Obsrv. Mass	± da	± ppm	Start Seq.	End Seq.	Sequence	Ion Score	C. I. %	Modification	Plate [#] Name	Gel Idx/Pos [4700 Sample Name]	iTRAQ Ratio 115/114*	iTRAQ Ratio 116/114*	iTRAQ Ratio 117/114*	Rank	Result Type
1534.8727	1534.78	-0.0927	-60	9	20	GIDPFSLDALAK	34	99.112	(N-term)_iTRAQ[0], Lysine(K)_iTRAQ[12]	[5] F4	303/295	1.393	1.131	1.080	1	Mascot

911	regulator of G-protein signalling 9 [Rattus norvegicus]	gi|2707359	54387.2	1	34	0.370	0.440	1.077	0.000	0.000	0.000	1	1	1	99.11

Protein Group

regulator of G-protein signaling 9 [Rattus norvegicus]	gi|9507051	85035.5

Peptide Information

Calc. Mass	Obsrv. Mass	± da	± ppm	Start Seq.	End Seq.	Sequence	Ion Score	C. I. %	Modification	Plate [#] Name	Gel Idx/Pos [4700 Sample Name]	iTRAQ Ratio 115/114*	iTRAQ Ratio 116/114*	iTRAQ Ratio 117/114*	Rank	Result Type
1426.7604	1426.7166	-0.0438	-31	63	72	WAFNFSELIR	34	99.11	(N-term)_iTRAQ[0]	[5] F4	389/381	0.370	0.440	1.077	1	Mascot

912	calpain-2 catalytic subunit precursor [Rattus norvegicus]	gi|8393038	86610.9	1	34	0.864	1.087	1.062	0.000	0.000	0.000	1	1	1	99.072

Protein Group

Chain A, Crystal Structure Analysis Of Rat M-Calpain Mutant Lys10 Thr	gi|60593581	86377.8
Chain A, Crystal Structure Of Calcium-Bound Protease Core Of Calpain Ii Reveals The Basis For Intri	gi|30749455	39378.7
Chain A, Crystal Structure Of M-Calpain	gi|9257037	86548.9

Peptide Information

Calc. Mass	Obsrv. Mass	± da	± ppm	Start Seq.	End Seq.	Sequence	Ion Score	C. I. %	Modification	Plate [#] Name	Gel Idx/Pos [4700 Sample Name]	iTRAQ Ratio 115/114*	iTRAQ Ratio 116/114*	iTRAQ Ratio 117/114*	Rank	Result Type
2121.9224	2121.8643	-0.0581	-27	297	312	WNDNCPSWNTVDPEVR	34	99.072	(N-term)_iTRAQ[0], MMTS (C)[5]	[1] F3 030912	365/357	0.864	1.087	1.062	1	Mascot

913	copper transport protein ATOX1 [Rattus norvegicus]	gi|16758088	8578.5	1	34	1.739	1.454	1.110	0.000	0.000	0.000	1	1	1	99.063

Peptide Information

Calc. Mass	Obsrv. Mass	± da	± ppm	Start Seq.	End Seq.	Sequence	Ion Score	C. I. %	Modification	Plate [#] Name	Gel Idx/Pos [4700 Sample Name]	iTRAQ Ratio 115/114*	iTRAQ Ratio 116/114*	iTRAQ Ratio 117/114*	Rank	Result Type
1976.1552	1976.2228	0.0676	34	26	39	LGGVEFNIDLPNKK	34	99.063	(N-term)_iTRAQ[0], Lysine(K)_iTRAQ[13, 14]	[3] F6 and F9	1286/1278	1.739	1.454	1.110	1	Mascot

914	mitochondrial import inner membrane translocase subunit Tim13 [Mus musculus]	gi|7305581	11643.6	1	34	0.657	0.921	0.694	0.000	0.000	0.000	1	1	1	99.042

Peptide Information

Calc. Mass	Obsrv. Mass	± da	± ppm	Start Seq.	End Seq.	Sequence	Ion Score	C. I. %	Modification	Plate [#] Name	Gel Idx/Pos [4700 Sample Name]	iTRAQ Ratio 115/114*	iTRAQ Ratio 116/114*	iTRAQ Ratio 117/114*	Rank	Result Type
1386.6597	1386.5887	-0.071	-51	73	82	YMDAWNTVSR	34	99.042	(N-term)_iTRAQ[0]	[5] F4	199/191	0.657	0.921	0.694	1	Mascot

915	actin-related protein 10 [Rattus norvegicus]	gi|57527131	50390.7	1	34	2.684	2.400	1.551	0.000	0.000	0.000	1	1	1	99.028

Peptide Information

Calc. Mass	Obsrv. Mass	± da	± ppm	Start Seq.	End Seq.	Sequence	Ion Score	C. I. %	Modification	Plate [#] Name	Gel Idx/Pos [4700 Sample Name]	iTRAQ Ratio 115/114*	iTRAQ Ratio 116/114*	iTRAQ Ratio 117/114*	Rank	Result Type
1381.7753	1381.7206	-0.0547	-40	70	78	EFIHILYFR	34	99.028	(N-term)_iTRAQ[0]	[6] F8 110912	421/413	2.684	2.400	1.551	1	Mascot

916	geranylgeranyl transferase type-2 subunit alpha [Rattus	gi|13928906	68900.8	1	34	1.467	1.594	0.942	0.000	0.000	0.000	1	1	1	99.026

norvegicus]

Protein Group

Chain A, Crystal Structure Of Rab Geranylgeranyltransferase From Rat Brain	gi|7546395	68880.7

Peptide Information

Calc. Mass	Obsrv. Mass	± da	± ppm	Start Seq.	End Seq.	Sequence	Ion Score	C. I. %	Modification	Plate [#] Name	Gel Idx/Pos [4700 Sample Name]	iTRAQ Ratio 115/114*	iTRAQ Ratio 116/114*	iTRAQ Ratio 117/114*	Rank	Result Type
1427.7128	1427.681	-0.0318	-22	91	101	AELGFLESCLR	34	99.026	(N-term)_iTRAQ[0], MMTS (C)[9]	[1] F3 030912	503/495	1.467	1.594	0.942	1	Mascot

917	phosphoserine aminotransferase [Rattus norvegicus]	gi|29692074	44441.2	1	34	1.410	1.339	1.390	0.000	0.000	0.000	1	1	1	99.021

Protein Group

phosphoserine aminotransferase [Rattus norvegicus]	gi|61889115	44623.4

Peptide Information

Calc. Mass	Obsrv. Mass	± da	± ppm	Start Seq.	End Seq.	Sequence	Ion Score	C. I. %	Modification	Plate [#] Name	Gel Idx/Pos [4700 Sample Name]	iTRAQ Ratio 115/114*	iTRAQ Ratio 116/114*	iTRAQ Ratio 117/114*	Rank	Result Type
1672.0282	1671.9829	-0.0453	-27	117	127	KFGTVNIVHPK	34	99.021	(N-term)_iTRAQ[0], Lysine(K)_iTRAQ[1,11]	[2] F12 040912	232/224	1.410	1.339	1.390	1	Mascot

918	RecName: Full=DNA damage-binding protein 1; AltName: Full=Damage-specific DNA-binding protein 1	gi|81868411	135718.6	1	34	0.983	0.555	0.772	0.000	0.000	0.000	1	1	1	99.017

Peptide Information

Calc. Mass	Obsrv. Mass	± da	± ppm	Start Seq.	End Seq.	Sequence	Ion Score	C. I. %	Modification	Plate [#] Name	Gel Idx/Pos [4700 Sample Name]	iTRAQ Ratio 115/114*	iTRAQ Ratio 116/114*	iTRAQ Ratio 117/114*	Rank	Result Type
1491.8967	1491.8231	-0.0736	-49	61	70	IAVMELFRPK	34	99.017	(N-term)_iTRAQ[0], Lysine(K)_iTRAQ[10]	[6] F8 110912	399/391	0.983	0.555	0.772	1	Mascot

919	membrane-associated progesterone receptor component 2 [Rattus norvegicus]	gi|56605824	25354	1	34	1.030	1.094	1.059	0.000	0.000	0.000	1	1	1	99.01

Peptide Information

Calc. Mass	Obsrv. Mass	± da	± ppm	Start Seq.	End Seq.	Sequence	Ion Score	C. I. %	Modification	Plate [#] Name	Gel Idx/Pos [4700 Sample Name]	iTRAQ Ratio 115/114*	iTRAQ Ratio 116/114*	iTRAQ Ratio 117/114*	Rank	Result Type
1285.6497	1285.619	-0.0307	-24	179	185	EWEMQFK	34	99.01	(N-term)_iTRAQ[0], Lysine(K)_iTRAQ[7]	[3] F6 and F9	292/284	1.030	1.094	1.059	1	Mascot

920	catenin beta-1 [Mus musculus]	gi|6671684	89812.8	1	34	0.505	0.820	0.783	0.091	0.077	0.001	2	2	2	98.966

Protein Group

catenin beta-1 [Rattus norvegicus]	gi|46048609	89796.8

Peptide Information

Calc. Mass	Obsrv. Mass	± da	± ppm	Start Seq.	End Seq.	Sequence	Ion Score	C. I. %	Modification	Plate [#] Name	Gel Idx/Pos [4700 Sample Name]	iTRAQ Ratio 115/114*	iTRAQ Ratio 116/114*	iTRAQ Ratio 117/114*	Rank	Result Type
1527.7458	1527.6361	-0.1097	-72	475	486	HQEAEMAQNAVR	32	98.446	(N-term)_iTRAQ[0]	[6] F8 110912	127/119	0.595	0.897	0.784	1	Mascot
1527.7458	1527.7629	0.0171	11	475	486	HQEAEMAQNAVR	34	98.966	(N-term)_iTRAQ[0]	[3] F6 and F9	1092/1084	0.428	0.750	0.783	1	Mascot

921	guanine nucleotide-binding protein subunit beta-2-like 1 [Rattus norvegicus]	gi|18543331	38034.2	1	34	4.087	4.210	3.394	0.000	0.000	0.000	1	1	1	98.964

Protein Group

guanine nucleotide-binding protein subunit beta-2-like 1 [Homo sapiens]	gi|5174447	38016.3

Peptide Information

Calc. Mass	Obsrv. Mass	± da	± ppm	Start Seq.	End Seq.	Sequence	Ion Score	C. I. %	Modification	Plate [#] Name	Gel Idx/Pos [4700 Sample Name]	iTRAQ Ratio 115/114*	iTRAQ Ratio 116/114*	iTRAQ Ratio 117/114*	Rank	Result Type
1148.6561	1148.6348	-0.0213	-19	258	264	IWDLEGK	34	98.964	(N-term)_iTRAQ[0], Lysine(K)_iTRAQ[7]	[3] F6 and F9	284/276	4.087	4.210	3.394	1	Mascot

922	cytochrome b	gi|223536	53232.1	1	34	1.076	0.978	1.126	0.076	0.048	0.035	2	2	2	98.942

Protein Group

RecName: Full=Cytochrome b; AltName: Full=Complex III subunit 3; AltName: Full=Complex III subunit	gi|117884	44692.8
cytochrome B gene [Rattus norvegicus]	gi|829020	44680.8
cytochrome b [Rattus argentiventer]	gi|6139023	44596.8
cytochrome b [Rattus norvegicus]	gi|110189727	44706.9
cytochrome b [Rattus norvegicus]	gi|110189675	44683.8
cytochrome b [Rattus norvegicus]	gi|19577326	44709.9
cytochrome b [Rattus norvegicus]	gi|10121867	44570.7
unnamed protein product [Rattus norvegicus]	gi|13469	44692.8

Peptide Information

Calc. Mass	Obsrv. Mass	± da	± ppm	Start Seq.	End Seq.	Sequence	Ion Score	C. I. %	Modification	Plate [#] Name	Gel Idx/Pos [4700 Sample Name]	iTRAQ Ratio 115/114*	iTRAQ Ratio 116/114*	iTRAQ Ratio 117/114*	Rank	Result Type
1288.8113	1288.8303	0.019	15	85	91	KSHPLFK	33	98.621	(N-term)_iTRAQ[0], Lysine(K)_iTRAQ[1,7]	[8] F13-15 and F1+2	630/622	1.005	0.932	1.092	1	Mascot
1288.8113	1288.8447	0.0334	26	85	91	KSHPLFK	34	98.942	(N-term)_iTRAQ[0], Lysine(K)_iTRAQ[1,7]	[8] F13-15 and F1+2	633/625	1.152	1.027	1.162	1	Mascot

923	probable E3 ubiquitin-protein ligase HERC4 [Rattus norvegicus]	gi|58865718	128836.5	1	34	1.340	0.717	0.476	0.094	0.161	0.145	2	2	2	98.939

Peptide Information

Calc. Mass	Obsrv. Mass	± da	± ppm	Start Seq.	End Seq.	Sequence	Ion Score	C. I. %	Modification	Plate [#] Name	Gel Idx/Pos [4700 Sample Name]	iTRAQ Ratio 115/114*	iTRAQ Ratio 116/114*	iTRAQ Ratio 117/114*	Rank	Result Type
1503.923	1503.7482	-0.1748	-116	827	835	RKPSLDDLK	30	97.462	(N-term)_iTRAQ[0], Lysine(K)_iTRAQ[2,9]	[5] F4	276/268	1.252	0.878	0.365	1	Mascot
1503.923	1503.7634	-0.1596	-106	827	835	RKPSLDDLK	34	98.939	(N-term)_iTRAQ[0], Lysine(K)_iTRAQ[2,9]	[7] F5 120912	347/339	1.434	0.586	0.620	1	Mascot

924	eukaryotic peptide chain release factor GTP-binding subunit ERF3A [Rattus norvegicus]	gi|51571949	76124.7	1	33	0.954	1.134	1.006	0.000	0.000	0.000	1	1	1	98.874

Peptide Information

Calc. Mass	Obsrv. Mass	± da	± ppm	Start Seq.	End Seq.	Sequence	Ion Score	C. I. %	Modification	Plate [#] Name	Gel Idx/Pos [4700 Sample Name]	iTRAQ Ratio 115/114*	iTRAQ Ratio 116/114*	iTRAQ Ratio 117/114*	Rank	Result Type
1603.8702	1603.9268	0.0566	35	324	333	KGEFETGFEK	33	98.874	(N-term)_iTRAQ[0], Lysine(K)_iTRAQ[1,10]	[4] F7 and F10+11	1091/1083	0.954	1.134	1.006	1	Mascot

925	Chain A, Crystal Structure Of Lc3	gi|50513823	15842.5	1	33	0.670	1.022	0.610	0.000	0.000	0.000	1	1	1	98.84

Protein Group

RecName: Full=Microtubule-associated proteins 1A/1B light chain 3B; AltName: Full=Autophagy-related	gi|3024150	17870.6
microtubule-associated proteins 1A/1B light chain 3B [Rattus norvegicus]	gi|41054912	15886.6
zbs559 [Rattus norvegicus]	gi|32264635	48881.6

Peptide Information

Calc. Mass	Obsrv. Mass	± da	± ppm	Start Seq.	End Seq.	Sequence	Ion Score	C. I. %	Modification	Plate [#] Name	Gel Idx/Pos [4700 Sample Name]	iTRAQ Ratio 115/114*	iTRAQ Ratio 116/114*	iTRAQ Ratio 117/114*	Rank	Result Type
1930.0718	1930.0316	-0.0402	-21	57	70	FLVPDHVNMSELIK	33	98.84	(N-term)_iTRAQ[0], Lysine(K)_iTRAQ[14]	[3] F6 and F9	396/388	0.670	1.022	0.610	1	Mascot

926	eukaryotic translation initiation factor 2 subunit 2 [Rattus norvegicus]	gi|40786451	45412.1	1	33	0.931	0.726	1.046	0.000	0.000	0.000	1	1	1	98.813

Peptide Information

Calc. Mass	Obsrv. Mass	± da	± ppm	Start Seq.	End Seq.	Sequence	Ion Score	C. I. %	Modification	Plate [#] Name	Gel Idx/Pos [4700 Sample Name]	iTRAQ Ratio 115/114*	iTRAQ Ratio 116/114*	iTRAQ Ratio 117/114*	Rank	Result Type
2167.187	2167.1941	0.0071	3	88	102	IFDIDEAEEAIKDVK	33	98.813	(N-term)_iTRAQ[0], Lysine(K)_iTRAQ[12, 15]	[4] F7 and F10+11	438/430	0.931	0.726	1.046	1	Mascot

927	phosphatidylinositol 3-kinase catalytic subunit type 3 [Rattus norvegicus]	gi|12621140	112252.9	1	33	1.848	0.839	0.998	0.000	0.000	0.000	1	1	1	98.793

Peptide Information

Calc. Mass	Obsrv. Mass	± da	± ppm	Start Seq.	End Seq.	Sequence	Ion Score	C. I. %	Modification	Plate [#] Name	Gel Idx/Pos [4700 Sample Name]	iTRAQ Ratio 115/114*	iTRAQ Ratio 116/114*	iTRAQ Ratio 117/114*	Rank	Result Type
1463.7682	1463.7703	0.0021	1	85	92	WNWNEWLK	33	98.793	(N-term)_iTRAQ[0], Lysine(K)_iTRAQ[8]	[4] F7 and F10+11	440/432	1.848	0.839	0.998	1	Mascot

928	synaptophysin-like protein 1 [Rattus norvegicus]	gi|62079261	30902.9	1	33	1.443	1.281	0.547	0.000	0.000	0.000	1	1	1	98.791

Peptide Information

Calc. Mass	Obsrv. Mass	± da	± ppm	Start Seq.	End Seq.	Sequence	Ion Score	C. I. %	Modification	Plate [#] Name	Gel Idx/Pos [4700 Sample Name]	iTRAQ Ratio 115/114*	iTRAQ Ratio 116/114*	iTRAQ Ratio 117/114*	Rank	Result Type
1417.76	1417.7225	-0.0375	-26	73	83	TITATFGYPFR	33	98.791	(N-term)_iTRAQ[0]	[1] F3 030912	354/346	1.443	1.281	0.547	1	Mascot

929	unnamed protein product [Rattus norvegicus]	gi|2326263	36570.6	1	33	3.889	3.370	2.580	0.000	0.000	0.000	1	1	1	98.779

Protein Group

Clu protein [Rattus norvegicus]	gi|38181879	56070.2
RecName: Full=Clusterin; AltName: Full=Dimeric acid glycoprotein; Short=DAG; AltName: Full=Sulfated	gi|461756	56027.1
clusterin [Rattus norvegicus]	gi|46048420	56049.1

Peptide Information

Calc. Mass	Obsrv. Mass	± da	± ppm	Start Seq.	End Seq.	Sequence	Ion Score	C. I. %	Modification	Plate [#] Name	Gel Idx/Pos [4700 Sample Name]	iTRAQ Ratio 115/114*	iTRAQ Ratio 116/114*	iTRAQ Ratio 117/114*	Rank	Result Type
1897.9198	1897.8422	-0.0776	-41	76	90	QQSQVLDAMQDSFTR	33	98.779	(N-term)_iTRAQ[0]	[1] F3 030912	308/300	3.889	3.370	2.580	1	Mascot

930	asparagine synthetase [Rattus norvegicus]	gi|460631	70177.3	1	33	0.453	0.352	0.870	0.000	0.000	0.000	1	1	1	98.751

Protein Group

asparagine synthetase [glutamine-hydrolyzing] [Rattus norvegicus]	gi|148747576	70141.2

Peptide Information

Calc. Mass	Obsrv. Mass	± da	± ppm	Start Seq.	End Seq.	Sequence	Ion Score	C. I. %	Modification	Plate [#] Name	Gel Idx/Pos [4700 Sample Name]	iTRAQ Ratio 115/114*	iTRAQ Ratio 116/114*	iTRAQ Ratio 117/114*	Rank	Result Type
1311.7433	1311.6987	-0.0446	-34	396	404	ELYLFDVLR	33	98.751	(N-term)_iTRAQ[0]	[1] F3 030912	471/463	0.453	0.352	0.870	1	Mascot

931	peptidyl-prolyl cis-trans isomerase FKBP1B [Rattus norvegicus]	gi|12083613	13320	1	33	1.592	1.709	1.468	0.000	0.000	0.000	1	1	1	98.713

Peptide Information

Calc. Mass	Obsrv. Mass	± da	± ppm	Start Seq.	End Seq.	Sequence	Ion Score	C. I. %	Modification	Plate [#] Name	Gel Idx/Pos [4700 Sample Name]	iTRAQ Ratio 115/114*	iTRAQ Ratio 116/114*	iTRAQ Ratio 117/114*	Rank	Result Type
1624.7874	1624.7136	-0.0738	-45	59	72	GFEEGAAQMSLGQR	33	98.713	(N-term)_iTRAQ[0]	[1] F3 030912	230/222	1.592	1.709	1.468	1	Mascot

932	phosphatidylinositol 4-kinase alpha [Rattus norvegicus]	gi|25742825	250820.8	1	33	0.876	0.907	0.411	0.000	0.000	0.000	1	1	1	98.713

Peptide Information

Calc. Mass	Obsrv. Mass	± da	± ppm	Start Seq.	End Seq.	Sequence	Ion Score	C. I. %	Modification	Plate [#] Name	Gel Idx/Pos [4700 Sample Name]	iTRAQ Ratio 115/114*	iTRAQ Ratio 116/114*	iTRAQ Ratio 117/114*	Rank	Result Type
1454.7837	1454.7424	-0.0413	-28	263	272	EFYIPLEMLR	33	98.713	(N-term)_iTRAQ[0]	[1] F3 030912	483/475	0.876	0.907	0.411	1	Mascot

933	rho guanine nucleotide exchange factor 2 [Rattus	gi|58865728	119826.4	1	33	0.814	1.110	0.752	0.000	0.000	0.000	1	1	1	98.704

norvegicus]

Peptide Information

Calc. Mass	Obsrv. Mass	± da	± ppm	Start Seq.	End Seq.	Sequence	Ion Score	C. I. %	Modification	Plate [#] Name	Gel Idx/Pos [4700 Sample Name]	iTRAQ Ratio 115/114*	iTRAQ Ratio 116/114*	iTRAQ Ratio 117/114*	Rank	Result Type
1531.8356	1531.8496	0.014	9	474	483	LIHDGCLLWK	33	98.704	(N-term)_iTRAQ[0], Lysine(K)_iTRAQ[10], MMTS (C)[6]	[3] F6 and F9	1349/1341	0.814	1.110	0.752	1	Mascot

934	Chain A, Rat Outer Mitochondrial Membrane Cytochrome B5	gi|15988286	10707.3	1	32	0.758	0.457	0.851	0.000	0.000	0.000	1	1	1	98.611

Protein Group

Chain A, Rat Outer Mitochondrial Membrane Cytochrome B5	gi|3891604	11120.5
Chain A, Structure Of Quintuple Mutant Of The Rat Outer Mitocondrial Cytochrome B5.	gi|27065383	11124.5
cytochrome b5 type B precursor [Rattus norvegicus]	gi|13399338	17453.6
outer membrane cytochrome b(5) [Rattus norvegicus]	gi|1217655	12719.4

Peptide Information

Calc. Mass	Obsrv. Mass	± da	± ppm	Start Seq.	End Seq.	Sequence	Ion Score	C. I. %	Modification	Plate [#] Name	Gel Idx/Pos [4700 Sample Name]	iTRAQ Ratio 115/114*	iTRAQ Ratio 116/114*	iTRAQ Ratio 117/114*	Rank	Result Type
2219.2195	2219.3306	0.1111	50	73	87	QYYIGDVHPNDLKPK	32	98.611	(N-term)_iTRAQ[0], Lysine(K)_iTRAQ[13, 15]	[4] F7 and F10+11	1137/1129	0.758	0.457	0.851	1	Mascot

935	membrane-associated progesterone receptor component 1 [Rattus norvegicus]	gi|11120720	26326.4	1	32	1.035	1.047	1.216	0.000	0.000	0.000	1	1	1	98.566

Protein Group

hypothetical protein LOC100501557 [Zea mays]	gi|308044601	23550

Peptide Information

Calc. Mass	Obsrv. Mass	± da	± ppm	Start Seq.	End Seq.	Sequence	Ion Score	C. I. %	Modification	Plate [#] Name	Gel Idx/Pos [4700 Sample Name]	iTRAQ Ratio 115/114*	iTRAQ Ratio 116/114*	iTRAQ Ratio 117/114*	Rank	Result Type
1933.0217	1932.9569	-0.0648	-34	105	119	KFYGPEGPYGVFAGR	32	98.566	(N-term)_iTRAQ[0], Lysine(K)_iTRAQ[1]	[6] F8 110912	360/352	1.035	1.047	1.216	1	Mascot

936	protein kinase C delta [Rattus norvegicus]	gi|7019312	87690	1	32	1.783	1.597	2.340	0.000	0.000	0.000	1	1	1	98.533

Protein Group

protein kinase C delta III splice variant [Rattus sp.]	gi|6942180	50389.6
protein kinase C delta type [Rattus norvegicus]	gi|18959250	87704

Peptide Information

Calc. Mass	Obsrv. Mass	± da	± ppm	Start Seq.	End Seq.	Sequence	Ion Score	C. I. %	Modification	Plate [#] Name	Gel Idx/Pos [4700 Sample Name]	iTRAQ Ratio 115/114*	iTRAQ Ratio 116/114*	iTRAQ Ratio 117/114*	Rank	Result Type
2255.2295	2255.1206	-0.1089	-48	301	316	KPETPETVGIYQGFEK	32	98.533	(N-term)_iTRAQ[0], Lysine(K)_iTRAQ[1,1 6]	[6] F8 110912	271/263	1.783	1.597	2.340	1	Mascot

937	Ac2-067 [Rattus norvegicus]	gi|32527721	30684.2	1	32	1.192	1.038	1.090	0.000	0.000	0.000	1	1	1	98.529

Peptide Information

Calc. Mass	Obsrv. Mass	± da	± ppm	Start Seq.	End Seq.	Sequence	Ion Score	C. I. %	Modification	Plate [#] Name	Gel Idx/Pos [4700 Sample Name]	iTRAQ Ratio 115/114*	iTRAQ Ratio 116/114*	iTRAQ Ratio 117/114*	Rank	Result Type
1906.0531	1905.9614	-0.0917	-48	93	107	TPAGLQVLNDYLADK	32	98.529	(N-term)_iTRAQ[0], Lysine(K)_iTRAQ[15]	[1] F3 030912	417/409	1.192	1.038	1.090	1	Mascot

938	60S ribosomal protein L10-like [Mus musculus]	gi|242397462	28512.4	1	32	3.372	0.994	0.873	0.000	0.000	0.000	1	1	1	98.428

Protein Group

60S ribosomal protein L10 [Rattus norvegicus]	gi|13592053	28414.2

Peptide Information

Calc. Mass	Obsrv. Mass	± da	± ppm	Start Seq.	End Seq.	Sequence	Ion Score	C. I. %	Modification	Plate [#] Name	Gel Idx/Pos [4700 Sample Name]	iTRAQ Ratio 115/114*	iTRAQ Ratio 116/114*	iTRAQ Ratio 117/114*	Rank	Result Type
3215.4436	3215.3604	-0.0832	-26	43	69	VDEFPLCGHMVSDEYEQ LSSEALEAAR	32	98.428	(N-term)_iTRAQ[0], MMTS (C)[7]	[1] F3 030912	532/524	3.372	0.994	0.873	1	Mascot

939	PREDICTED: ubiquitin carboxyl-terminal hydrolase CYLD [Rattus norvegicus]	gi|293355131	59817.9	1	32	1.439	1.692	1.505	0.000	0.000	0.000	1	1	1	98.398		

Protein Group

40S ribosomal protein S3a [Rattus norvegicus] gi|8394221 35729.7

Peptide Information

Calc. Mass	Obsrv. Mass	± da	± ppm	Start Seq.	End Seq.	Sequence	Ion Score	C. I. %	Modification	Plate [#] Name	Gel Idx/Pos [4700 Sample Name]	iTRAQ Ratio 115/114*	iTRAQ Ratio 116/114*	iTRAQ Ratio 117/114*	Rank	Result Type
1385.78	1385.7668	-0.0132	-10	28	34	KDWYDVK	32	98.398	(N-term)_iTRAQ[0], Lysine(K)_iTRAQ[1,7]	[2] F12 040912	223/215	1.439	1.692	1.505	1	Mascot

940	inositol-1,4,5-triphosphate receptor [Rattus norvegicus]	gi|204674	337950.6	1	32	1.013	1.221	1.609	0.000	0.000	0.000	1	1	1	98.391

Protein Group

RecName: Full=Inositol 1,4,5-trisphosphate receptor type 1; AltName: Full=IP3 receptor isoform 1; S gi|17380349 339889.4

inositol 1,4,5-trisphosphate receptor type 1 [Rattus norvegicus] gi|55925610 339761.3

Peptide Information

Calc. Mass	Obsrv. Mass	± da	± ppm	Start Seq.	End Seq.	Sequence	Ion Score	C. I. %	Modification	Plate [#] Name	Gel Idx/Pos [4700 Sample Name]	iTRAQ Ratio 115/114*	iTRAQ Ratio 116/114*	iTRAQ Ratio 117/114*	Rank	Result Type
2142.9607	2142.8474	-0.1133	-53	1121	1138	GQGPDEPMDGASGENE HK	32	98.391	(N-term)_iTRAQ[0], Lysine(K)_iTRAQ[18]	[6] F8 110912	103/95	1.013	1.221	1.609	1	Mascot

941	xin actin-binding repeat-containing protein 2 [Rattus norvegicus]	gi|42538984	417530.2	1	32	0.647	0.803	1.038	0.000	0.000	0.000	1	1	1	98.363

Peptide Information

Calc. Mass	Obsrv. Mass	± da	± ppm	Start Seq.	End Seq.	Sequence	Ion Score	C. I. %	Modification	Plate [#] Name	Gel Idx/Pos [4700 Sample Name]	iTRAQ Ratio 115/114*	iTRAQ Ratio 116/114*	iTRAQ Ratio 117/114*	Rank	Result Type
1246.7505	1246.7312	-0.0193	-15	1685	1692	ELILDDLK	33	98.751	(N-term)_iTRAQ[0], Lysine(K)_iTRAQ[8]	[5] F4	288/280	0.647	0.803	1.038	1	Mascot

942	peripheral plasma membrane protein CASK [Rattus norvegicus]	gi|11559947	112431	1	32	0.669	1.403	1.168	0.000	0.000	0.000	1	1	1	98.353

Peptide Information

Calc. Mass	Obsrv. Mass	± da	± ppm	Start Seq.	End Seq.	Sequence	Ion Score	C. I. %	Modification	Plate [#] Name	Gel Idx/Pos [4700 Sample Name]	iTRAQ Ratio 115/114*	iTRAQ Ratio 116/114*	iTRAQ Ratio 117/114*	Rank	Result Type
1478.8451	1478.8325	-0.0126	-9	286	296	IHLPETVEQLR	32	98.353	(N-term)_iTRAQ[0]	[4] F7 and F10+11	304/296	0.669	1.403	1.168	1	Mascot

943	tyrosine hydroxylase [Rattus norvegicus]	gi|986947	12019.5	1	32	0.127	0.224	0.036	0.000	0.000	0.000	1	1	1	98.348

Protein Group

tyrosine 3-monooxygenase [Rattus norvegicus] gi|6981652 59423.5

Peptide Information

Calc. Mass	Obsrv. Mass	± da	± ppm	Start Seq.	End Seq.	Sequence	Ion Score	C. I. %	Modification	Plate [#] Name	Gel Idx/Pos [4700 Sample Name]	iTRAQ Ratio 115/114*	iTRAQ Ratio 116/114*	iTRAQ Ratio 117/114*	Rank	Result Type
1575.8978	1575.8717	-0.0261	-17	78	90	DGNAVLNLLFSLR	33	98.739	(N-term)_iTRAQ[0]	[1] F3 030912	555/547	0.127	0.224	0.036	1	Mascot

944	postsynaptic density protein [Rattus norvegicus]	gi|2745840	205104.3	1	32	0.869	0.719	0.782	0.000	0.000	0.000	1	1	1	98.331

Peptide Information

Calc. Mass	Obsrv. Mass	± da	± ppm	Start Seq.	End Seq.	Sequence	Ion Score	C. I. %	Modification	Plate [#] Name	Gel Idx/Pos [4700 Sample Name]	iTRAQ Ratio 115/114*	iTRAQ Ratio 116/114*	iTRAQ Ratio 117/114*	Rank	Result Type

Calc. Mass	Obsrv. Mass	± da	± ppm	Start Seq.	End Seq.	Sequence	Ion Score	C. I. %	Modification	Plate [#] Name	Gel Idx/Pos [4700 Sample Name]	iTRAQ Ratio 115/114*	iTRAQ Ratio 116/114*	iTRAQ Ratio 117/114*	Rank	Result Type
1544.8319	1544.8857	0.0538	35	1514	1524	GPPTYNEHITK	32	98.331	(N-term)_iTRAQ[0], Lysine(K)_iTRAQ[11]	[3] F6 and F9	1119/1111	0.869	0.719	0.782	1	Mascot

945	RecName: Full=Enoyl-CoA delta isomerase 1, mitochondrial; AltName: Full=3,2-trans-enoyl-CoA isomera	gi|118210	35352.1	1	32	1.688	1.366	1.381	0.000	0.000	0.000	1	1	1	98.307

Protein Group

Chain A, Crystal Structure Of Rat Mitochondrial 3,2-Enoyl-Coa	gi|56967286	32422.5
delta-3,delta-2-enoyl-CoA isomerase [Rattus norvegicus]	gi|203900	35156.8
delta3, delta2-enoyl-CoA isomerase precursor [Rattus norvegicus]	gi|220734	36135.4
enoyl-CoA delta isomerase 1, mitochondrial precursor [Rattus norvegicus]	gi|162287040	35362.2

Peptide Information

Calc. Mass	Obsrv. Mass	± da	± ppm	Start Seq.	End Seq.	Sequence	Ion Score	C. I. %	Modification	Plate [#] Name	Gel Idx/Pos [4700 Sample Name]	iTRAQ Ratio 115/114*	iTRAQ Ratio 116/114*	iTRAQ Ratio 117/114*	Rank	Result Type
1302.6715	1302.6923	0.0208	16	230	238	WFTIPDHSR	32	98.307	(N-term)_iTRAQ[0]	[3] F6 and F9	1255/1247	1.688	1.366	1.381	1	Mascot

946	ADP-ribosylation factor-like protein 3 [Rattus norvegicus]	gi|12083661	22743.2	1	32	0.908	0.966	0.531	0.000	0.000	0.000	1	1	1	98.307

Peptide Information

Calc. Mass	Obsrv. Mass	± da	± ppm	Start Seq.	End Seq.	Sequence	Ion Score	C. I. %	Modification	Plate [#] Name	Gel Idx/Pos [4700 Sample Name]	iTRAQ Ratio 115/114*	iTRAQ Ratio 116/114*	iTRAQ Ratio 117/114*	Rank	Result Type
1301.7086	1301.6423	-0.0663	-51	63	72	LNVWDIGGQR	32	98.307	(N-term)_iTRAQ[0]	[5] F4	234/226	0.908	0.966	0.531	1	Mascot

947	serine protease 23 precursor [Rattus norvegicus]	gi|56090287	48733.7	1	32	1.629	0.892	1.131	0.000	0.000	0.000	1	1	1	98.3

Peptide Information

Calc. Mass	Obsrv. Mass	± da	± ppm	Start Seq.	End Seq.	Sequence	Ion Score	C. I. %	Modification	Plate [#] Name	Gel Idx/Pos [4700 Sample Name]	iTRAQ Ratio 115/114*	iTRAQ Ratio 116/114*	iTRAQ Ratio 117/114*	Rank	Result Type
986.592	986.5673	-0.0247	-25	137	142	FSIFGK	32	98.3	(N-term)_iTRAQ[0], Lysine(K)_iTRAQ[6]	[3] F6 and F9	315/307	1.629	0.892	1.131	1	Mascot

948	Gart protein [Rattus norvegicus]	gi|56388736	50130.3	1	32	1.066	1.305	0.730	0.000	0.000	0.000	1	1	1	98.28

Peptide Information

Calc. Mass	Obsrv. Mass	± da	± ppm	Start Seq.	End Seq.	Sequence	Ion Score	C. I. %	Modification	Plate [#] Name	Gel Idx/Pos [4700 Sample Name]	iTRAQ Ratio 115/114*	iTRAQ Ratio 116/114*	iTRAQ Ratio 117/114*	Rank	Result Type
2272.134	2272.0408	-0.0932	-41	163	178	SKAEACEAVQEIMQEK	32	98.28	(N-term)_iTRAQ[0], Lysine(K)_iTRAQ[2,16], MMTS (C)[6]	[6] F8 110912	415/407	1.066	1.305	0.730	1	Mascot

949	RecName: Full=Nitric oxide synthase, brain; AltName: Full=BNOS; AltName: Full=Constitutive NOS; Alt	gi|266646	174675.1	1	32	0.600	0.797	0.914	0.000	0.000	0.000	1	1	1	98.264

Protein Group

Chain A, Crystal Structure Of Rat Neuronal Nitric-Oxide Synthase FadNADP+ DOMAIN AT 1.9A RESOLUTION	gi|16975326	53273.6
Chain A, Crystal Structure Of Rat Neuronal Nitric-Oxide Synthase Reductase Module At 2.3 A Resoluti	gi|52695822	84363.2
nitric oxide synthase, brain [Rattus norvegicus]	gi|16258811	178615.1

Peptide Information

Calc. Mass	Obsrv. Mass	± da	± ppm	Start Seq.	End Seq.	Sequence	Ion Score	C. I. %	Modification	Plate [#] Name	Gel Idx/Pos [4700 Sample Name]	iTRAQ Ratio 115/114*	iTRAQ Ratio 116/114*	iTRAQ Ratio 117/114*	Rank	Result Type
1469.7522	1469.7295	-0.0227	-15	1131	1139	GLQEYEEWK	32	98.264	(N-term)_iTRAQ[0], Lysine(K)_iTRAQ[9]	[3] F6 and F9	282/274	0.600	0.797	0.914	1	Mascot

950	ribosomal protein L19 [Rattus norvegicus]	gi|554467	5485.2	1	31	0.749	1.256	0.970	0.000	0.000	0.000	1	1	1	98.256

Protein Group

60S ribosomal protein L19 [Homo sapiens]	gi|4506609	28298.6	

Peptide Information

Calc. Mass	Obsrv. Mass	± da	± ppm	Start Seq.	End Seq.	Sequence	Ion Score	C. I. %	Modification	Plate [#] Name	Gel Idx/Pos [4700 Sample Name]	iTRAQ Ratio 115/114*	iTRAQ Ratio 116/114*	iTRAQ Ratio 117/114*	Rank	Result Type
1308.7708	1308.8251	0.0543	41	27	34	ILMEHIHK	31	98.256	(N-term)_iTRAQ[0], Lysine(K)_iTRAQ[8]	[8] F13-15 and F1+2	1193/1185	0.749	1.256	0.970	1	Mascot

951	secretogranin-3 precursor [Rattus norvegicus]	gi|16758722	60788.2	1	31	1.453	0.922	1.097	0.000	0.000	0.000	1	1	1	98.174

Protein Group

chromogranin/secretogranin-like vesicle protein precursor - rat	gi|111518	67763.7

Peptide Information

Calc. Mass	Obsrv. Mass	± da	± ppm	Start Seq.	End Seq.	Sequence	Ion Score	C. I. %	Modification	Plate [#] Name	Gel Idx/Pos [4700 Sample Name]	iTRAQ Ratio 115/114*	iTRAQ Ratio 116/114*	iTRAQ Ratio 117/114*	Rank	Result Type
2022.9978	2022.8679	-0.1299	-64	207	221	EANNYEEAPEKPTSR	31	98.174	(N-term)_iTRAQ[0], Lysine(K)_iTRAQ[11]	[6] F8 110912	124/116	1.453	0.922	1.097	1	Mascot

952	oxysterol-binding protein-related protein 1 [Rattus norvegicus]	gi|25282421	119509.9	1	31	0.919	1.101	0.895	0.000	0.000	0.000	1	1	1	98.105

Peptide Information

Calc. Mass	Obsrv. Mass	± da	± ppm	Start Seq.	End Seq.	Sequence	Ion Score	C. I. %	Modification	Plate [#] Name	Gel Idx/Pos [4700 Sample Name]	iTRAQ Ratio 115/114*	iTRAQ Ratio 116/114*	iTRAQ Ratio 117/114*	Rank	Result Type
1891.066	1891.0529	-0.0131	-7	752	763	ELHKVEGYIQDK	31	98.105	(N-term)_iTRAQ[0], Lysine(K)_iTRAQ[4,12]	[2] F12 040912	279/271	0.919	1.101	0.895	1	Mascot

953	cullin-5 [Rattus norvegicus]	gi|12083629	102244	1	31	1.355	1.291	1.345	0.000	0.000	0.000	1	1	1	98.092

Peptide Information

Calc. Mass	Obsrv. Mass	± da	± ppm	Start Seq.	End Seq.	Sequence	Ion Score	C. I. %	Modification	Plate [#] Name	Gel Idx/Pos [4700 Sample Name]	iTRAQ Ratio 115/114*	iTRAQ Ratio 116/114*	iTRAQ Ratio 117/114*	Rank	Result Type
2099.1094	2099.0015	-0.1079	-51	138	152	LMLDTWNESIFSNIK	31	98.092	(N-term)_iTRAQ[0], Lysine(K)_iTRAQ[15]	[5] F4	383/375	1.355	1.291	1.345	1	Mascot

954	neurexin III-alpha membrane-bound type 3 precursor - rat	gi|539981	174651.2	1	31	1.623	1.781	1.015	0.000	0.000	0.000	1	1	1	98.066

Protein Group

neurexin III-alpha [Rattus norvegicus]	gi|294597	165480.4
neurexin III-alpha [Rattus norvegicus]	gi|294600	174386.2
neurexin III-alpha [Rattus norvegicus]	gi|294598	162938.3
neurexin III-alpha [Rattus norvegicus]	gi|294602	169845.5
neurexin III-alpha precursor [Rattus norvegicus]	gi|294599	165199.5
neurexin III-alpha secreted type 1 precursor - rat	gi|539983	170137.5
neurexin-3-alpha precursor [Rattus norvegicus]	gi|168823431	187643.3

Peptide Information

Calc. Mass	Obsrv. Mass	± da	± ppm	Start Seq.	End Seq.	Sequence	Ion Score	C. I. %	Modification	Plate [#] Name	Gel Idx/Pos [4700 Sample Name]	iTRAQ Ratio 115/114*	iTRAQ Ratio 116/114*	iTRAQ Ratio 117/114*	Rank	Result Type
1950.0251	1949.968	-0.0571	-29	812	825	LEFHNIETGIMTEK	31	98.066	(N-term)_iTRAQ[0], Lysine(K)_iTRAQ[14]	[4] F7 and F10+11	344/336	1.623	1.781	1.015	1	Mascot

955	serine/threonine-protein kinase mTOR [Rattus norvegicus]	gi|9845251	309142.5	1	31	1.420	1.427	0.352	0.000	0.000	0.000	1	1	1	98.027

Peptide Information

Calc. Mass	Obsrv. Mass	± da	± ppm	Start Seq.	End Seq.	Sequence	Ion Score	C. I. %	Modification	Plate [#] Name	Gel Idx/Pos [4700 Sample Name]	iTRAQ Ratio 115/114*	iTRAQ Ratio 116/114*	iTRAQ Ratio 117/114*	Rank	Result Type
1276.6447	1276.6465	0.0018	1	1278	1285	DDWLEWLR	32	98.495	(N-term)_iTRAQ[0]	[7] F5 120912	461/453	1.420	1.427	0.352	1	Mascot

956 N-terminal EF-hand calcium-binding protein 2 [Rattus norvegicus] | gi\|19173782 | 42433.5 | 1 | 31 | 0.735 | 0.745 | 0.939 | 0.000 | 0.000 | 0.000 | 1 | 1 | 1 | 98.007

Peptide Information

Calc. Mass	Obsrv. Mass	± da	± ppm	Start Seq.	End Seq.	Sequence	Ion Score	C. I. %	Modification	Plate [#] Name	Gel Idx/Pos [4700 Sample Name]	iTRAQ Ratio 115/114*	iTRAQ Ratio 116/114*	iTRAQ Ratio 117/114*	Rank	Result Type
2255.061	2255.0105	-0.0505	-22	256	273	QEMAVCPEQLGEFLDSLR	31	98.007	(N-term)_iTRAQ[0], MMTS (C)[6]	[8] F13-15 and F1+2	246/238	0.735	0.745	0.939	1	Mascot

957 general vesicular transport factor p115 [Rattus norvegicus] | gi\|9507177 | 116200.4 | 1 | 31 | 1.074 | 1.391 | 0.867 | 0.000 | 0.000 | 0.000 | 1 | 1 | 1 | 97.984

Protein Group

RecName: Full=General vesicular transport factor p115; AltName: Full=Protein USO1 homolog; AltName: | gi\|1171952 | 116137.3

Peptide Information

Calc. Mass	Obsrv. Mass	± da	± ppm	Start Seq.	End Seq.	Sequence	Ion Score	C. I. %	Modification	Plate [#] Name	Gel Idx/Pos [4700 Sample Name]	iTRAQ Ratio 115/114*	iTRAQ Ratio 116/114*	iTRAQ Ratio 117/114*	Rank	Result Type
2109.0288	2108.9639	-0.0649	-31	261	276	AWFEVGDENPGWSAQK	31	97.984	(N-term)_iTRAQ[0], Lysine(K)_iTRAQ[16]	[5] F4	263/255	1.074	1.391	0.867	1	Mascot

958 synaptobrevin homolog YKT6 [Rattus norvegicus] | gi\|162138924 | 24798.9 | 1 | 31 | 0.867 | 0.794 | 0.667 | 0.000 | 0.000 | 0.000 | 1 | 1 | 1 | 97.903

Protein Group

prenylated SNARE protein Ykt6p [Rattus norvegicus] | gi\|2642348 | 24884.1

Peptide Information

Calc. Mass	Obsrv. Mass	± da	± ppm	Start Seq.	End Seq.	Sequence	Ion Score	C. I. %	Modification	Plate [#] Name	Gel Idx/Pos [4700 Sample Name]	iTRAQ Ratio 115/114*	iTRAQ Ratio 116/114*	iTRAQ Ratio 117/114*	Rank	Result Type
1668.8142	1668.7723	-0.0419	-25	21	33	AAYDVSSFSFFQR	31	97.903	(N-term)_iTRAQ[0]	[1] F3 030912	383/375	0.867	0.794	0.667	1	Mascot

959 fatty acid-binding protein [Rattus norvegicus] | gi\|6601500 | 16896.1 | 1 | 31 | 0.985 | 0.742 | 0.753 | 0.000 | 0.000 | 0.000 | 1 | 1 | 1 | 97.884

Protein Group

fatty acid-binding protein, heart [Rattus norvegicus] | gi\|13162363 | 16783

Peptide Information

Calc. Mass	Obsrv. Mass	± da	± ppm	Start Seq.	End Seq.	Sequence	Ion Score	C. I. %	Modification	Plate [#] Name	Gel Idx/Pos [4700 Sample Name]	iTRAQ Ratio 115/114*	iTRAQ Ratio 116/114*	iTRAQ Ratio 117/114*	Rank	Result Type
1350.6774	1350.5913	-0.0861	-64	98	107	WDGQETTLTR	31	97.884	(N-term)_iTRAQ[0]	[5] F4	128/120	0.985	0.742	0.753	1	Mascot

960 serine/Arginine-related protein 53 [Rattus norvegicus] | gi\|62079069 | 44023.1 | 1 | 31 | 1.056 | 0.941 | 0.967 | 0.000 | 0.000 | 0.000 | 1 | 1 | 1 | 97.874

Peptide Information

Calc. Mass	Obsrv. Mass	± da	± ppm	Start Seq.	End Seq.	Sequence	Ion Score	C. I. %	Modification	Plate [#] Name	Gel Idx/Pos [4700 Sample Name]	iTRAQ Ratio 115/114*	iTRAQ Ratio 116/114*	iTRAQ Ratio 117/114*	Rank	Result Type
934.5455	934.528	-0.0175	-19	197	202	ADEALK	31	97.874	(N-term)_iTRAQ[0], Lysine(K)_iTRAQ[6]	[3] F6 and F9	224/216	1.056	0.941	0.967	1	Mascot

961 glutathione S-transferase theta-2 [Rattus norvegicus] | gi\|6980992 | 29432.8 | 1 | 31 | 0.900 | 0.826 | 0.802 | 0.000 | 0.000 | 0.000 | 1 | 1 | 1 | 97.869

Peptide Information

Calc. Mass	Obsrv. Mass	± da	± ppm	Start Seq.	End Seq.	Sequence	Ion Score	C. I. %	Modification	Plate [#] Name	Gel Idx/Pos [4700 Sample Name]	iTRAQ Ratio 115/114*	iTRAQ Ratio 116/114*	iTRAQ Ratio 117/114*	Rank	Result Type
1099.6761	1099.6198	-0.0563	-51	16	22	AVYIFAK	31	97.869	(N-term)_iTRAQ[0], Lysine(K)_iTRAQ[7]	[7] F5 120912	230/222	0.900	0.826	0.802	1	Mascot

962 keratin, type II cytoskeletal cochleal [Rattus norvegicus] | gi\|114145409 | 62605.1 | 1 | 30 | 0.787 | 0.385 | 0.663 | 0.000 | 0.000 | 0.000 | 1 | 1 | 1 | 97.794

Protein Group

RecName: Full=Keratin, type II cytoskeletal 5; AltName: Full=Cytokeratin-5; Short=CK-5; AltName: Fu	gi|81170669	66248.7
keratin K5 [Rattus norvegicus]	gi|205055	10254.2
keratin, type II cytoskeletal 5 [Rattus norvegicus]	gi|50233797	66061.5
keratin, type II cytoskeletal 75 [Rattus norvegicus]	gi|57012352	66440.9

Peptide Information

Calc. Mass	Obsrv. Mass	± da	± ppm	Start Seq.	End Seq.	Sequence	Ion Score	C. I. %	Modification	Plate [#] Name	Gel Idx/Pos [4700 Sample Name]	iTRAQ Ratio 115/114*	iTRAQ Ratio 116/114*	iTRAQ Ratio 117/114*	Rank	Result Type
1590.92	1590.8463	-0.0737	-46	305	316	SLDLDSIIAEVK	30	97.794	(N-term)_iTRAQ[0], Lysine(K)_iTRAQ[12]	[5] F4	343/335	0.787	0.385	0.663	1	Mascot

963	antiquitin [Rattus sp.]	gi|9027561	27585.4	1	30	0.828	0.804	0.897	0.000	0.000	0.000	1	1	1	97.789

Peptide Information

Calc. Mass	Obsrv. Mass	± da	± ppm	Start Seq.	End Seq.	Sequence	Ion Score	C. I. %	Modification	Plate [#] Name	Gel Idx/Pos [4700 Sample Name]	iTRAQ Ratio 115/114*	iTRAQ Ratio 116/114*	iTRAQ Ratio 117/114*	Rank	Result Type
1615.9346	1615.8959	-0.0387	-24	116	126	ETFAPILYVFK	30	97.789	(N-term)_iTRAQ[0], Lysine(K)_iTRAQ[11]	[5] F4	388/380	0.828	0.804	0.897	1	Mascot

964	protein argonaute-2 [Rattus norvegicus]	gi|11024680	106390.3	1	30	1.055	0.756	0.856	0.000	0.000	0.000	1	1	1	97.769

Protein Group

RecName: Full=Protein argonaute-2; Short=Argonaute2; AltName: Full=Eukaryotic translation initiatio	gi|38372868	106049.1

Peptide Information

Calc. Mass	Obsrv. Mass	± da	± ppm	Start Seq.	End Seq.	Sequence	Ion Score	C. I. %	Modification	Plate [#] Name	Gel Idx/Pos [4700 Sample Name]	iTRAQ Ratio 115/114*	iTRAQ Ratio 116/114*	iTRAQ Ratio 117/114*	Rank	Result Type
1947.0393	1946.9551	-0.0842	-43	246	259	SIEEQQKPLTDSQR	30	97.769	(N-term)_iTRAQ[0], Lysine(K)_iTRAQ[7]	[4] F7 and F10+11	138/130	1.055	0.756	0.856	1	Mascot

965	sulfotransferase family 1D, member 1 [Rattus norvegicus]	gi|11120724	40437.6	1	30	1.938	1.194	1.866	0.000	0.000	0.000	1	1	1	97.748

Peptide Information

Calc. Mass	Obsrv. Mass	± da	± ppm	Start Seq.	End Seq.	Sequence	Ion Score	C. I. %	Modification	Plate [#] Name	Gel Idx/Pos [4700 Sample Name]	iTRAQ Ratio 115/114*	iTRAQ Ratio 116/114*	iTRAQ Ratio 117/114*	Rank	Result Type
2017.9939	2017.9199	-0.074	-37	148	161	MHPEPGTWEEFLEK	30	97.748	(N-term)_iTRAQ[0], Lysine(K)_iTRAQ[14]	[6] F8 110912	386/378	1.938	1.194	1.866	1	Mascot

966	Ab1-205 [Rattus norvegicus]	gi|33086478	88463.9	1	30	0.809	0.692	0.829	0.000	0.000	0.000	1	1	1	97.62

Peptide Information

Calc. Mass	Obsrv. Mass	± da	± ppm	Start Seq.	End Seq.	Sequence	Ion Score	C. I. %	Modification	Plate [#] Name	Gel Idx/Pos [4700 Sample Name]	iTRAQ Ratio 115/114*	iTRAQ Ratio 116/114*	iTRAQ Ratio 117/114*	Rank	Result Type
2172.0151	2171.999	-0.0161	-7	2	19	GQNDLMGTAEDFADQFLR	30	97.62	(N-term)_iTRAQ[0]	[8] F13-15 and F1+2	260/252	0.809	0.692	0.829	1	Mascot

967	olfactory receptor Olr936 [Rattus norvegicus]	gi|47777688	38034.8	1	30	1.074	0.282	2.026	0.166	0.091	0.091	2	2	2	97.615

Peptide Information

Calc. Mass	Obsrv. Mass	± da	± ppm	Start Seq.	End Seq.	Sequence	Ion Score	C. I. %	Modification	Plate [#] Name	Gel Idx/Pos [4700 Sample Name]	iTRAQ Ratio 115/114*	iTRAQ Ratio 116/114*	iTRAQ Ratio 117/114*	Rank	Result Type
1868.9899	1868.9553	-0.0346	-19	292	304	NQQVKQAFSDLMR	30	97.615	(N-term)_iTRAQ[0], Lysine(K)_iTRAQ[5], Oxidation (M)[12]	[3] F6 and F9	499/491	0.930	0.214	1.939	1	Mascot
1868.9899	1868.9833	-0.0066	-4	292	304	NQQVKQAFSDLMR	28	96.228	(N-term)_iTRAQ[0], Lysine(K)_iTRAQ[5], Oxidation (M)[12]	[4] F7 and F10+11	501/493	1.241	0.373	2.118	1	Mascot

968	long-chain-fatty-acid--CoA ligase 1 [Rattus norvegicus]	gi|25742739	86161.1	1	30	1.359	0.784	1.320	0.000	0.000	0.000	1	1	1	97.565

Peptide Information

Calc. Mass	Obsrv. Mass	± da	± ppm	Start Seq.	End Seq.	Sequence	Ion Score	C. I. %	Modification	Plate [#] Name	Gel Idx/Pos [4700 Sample Name]	iTRAQ Ratio	iTRAQ Ratio	iTRAQ Ratio	Rank	Result Type

Calc. Mass	Obsrv. Mass	± da	± ppm	Start Seq.	End Seq.	Sequence	Ion Score	C. I. %	Modification	Plate [#] Name	Gel Idx/Pos [4700 Sample Name]	iTRAQ Ratio 115/114*	iTRAQ Ratio 116/114*	iTRAQ Ratio 117/114*	Rank	Result Type
1462.656	1462.5972	-0.0588	-40	617	627	GFQGSFEELCR	30	97.565	(N-term)_iTRAQ[0], MMTS (C)[10]	[5] F4	312/304	1.359	0.784	1.320	1	Mascot

969	lactoylglutathione lyase [Rattus norvegicus]	gi|46485429	23538.1	1	30	2.261	1.691	0.989	0.000	0.000	0.000	1	1	1	97.559

Peptide Information

Calc. Mass	Obsrv. Mass	± da	± ppm	Start Seq.	End Seq.	Sequence	Ion Score	C. I. %	Modification	Plate [#] Name	Gel Idx/Pos [4700 Sample Name]	iTRAQ Ratio 115/114*	iTRAQ Ratio 116/114*	iTRAQ Ratio 117/114*	Rank	Result Type
1408.7743	1408.7343	-0.04	-28	29	38	DFLLQQTMLR	30	97.559	(N-term)_iTRAQ[0]	[1] F3 030912	361/353	2.261	1.691	0.989	1	Mascot

970	eukaryotic translation initiation factor 4B [Rattus norvegicus]	gi|56605726	74879.3	1	30	0.726	0.717	1.104	0.000	0.000	0.000	1	1	1	97.548

Peptide Information

Calc. Mass	Obsrv. Mass	± da	± ppm	Start Seq.	End Seq.	Sequence	Ion Score	C. I. %	Modification	Plate [#] Name	Gel Idx/Pos [4700 Sample Name]	iTRAQ Ratio 115/114*	iTRAQ Ratio 116/114*	iTRAQ Ratio 117/114*	Rank	Result Type
1820.0276	1820.0171	-0.0105	-6	357	372	AASIFGGAKPVDTAAR	30	97.548	(N-term)_iTRAQ[0], Lysine(K)_iTRAQ[9]	[4] F7 and F10+11	231/223	0.726	0.717	1.104	1	Mascot

971	RecName: Full=Transcription factor A, mitochondrial; Short=mtTFA; Flags: Precursor	gi|22654148	32964	1	30	0.968	0.560	0.468	0.000	0.000	0.000	1	1	1	97.491

Protein Group

transcription factor A [Rattus norvegicus]	gi|9963946	28421.8
transcription factor A, mitochondrial precursor [Rattus norvegicus]	gi|13786148	32918

Peptide Information

Calc. Mass	Obsrv. Mass	± da	± ppm	Start Seq.	End Seq.	Sequence	Ion Score	C. I. %	Modification	Plate [#] Name	Gel Idx/Pos [4700 Sample Name]	iTRAQ Ratio 115/114*	iTRAQ Ratio 116/114*	iTRAQ Ratio 117/114*	Rank	Result Type
1465.6865	1465.6014	-0.0851	-58	216	226	SWEEQMAEVGR	30	97.491	(N-term)_iTRAQ[0]	[5] F4	190/182	0.968	0.560	0.468	1	Mascot

972	glutathione peroxidase 1 [Rattus norvegicus]	gi|145275165	23871.3	1	30	0.990	1.113	0.671	0.000	0.000	0.000	1	1	1	97.485

Protein Group

Gpx1 protein [Rattus norvegicus]	gi|34849613	17913.3

Peptide Information

Calc. Mass	Obsrv. Mass	± da	± ppm	Start Seq.	End Seq.	Sequence	Ion Score	C. I. %	Modification	Plate [#] Name	Gel Idx/Pos [4700 Sample Name]	iTRAQ Ratio 115/114*	iTRAQ Ratio 116/114*	iTRAQ Ratio 117/114*	Rank	Result Type
1326.6804	1326.6375	-0.0429	-32	147	155	YIIWSPVCR	30	97.485	(N-term)_iTRAQ[0], MMTS (C)[8]	[1] F3 030912	419/411	0.990	1.113	0.671	1	Mascot

973	hypothetical protein LOC302247 [Rattus norvegicus]	gi|114145722	36921.9	1	30	0.415	0.666	0.596	0.000	0.000	0.000	1	1	1	97.479

Peptide Information

Calc. Mass	Obsrv. Mass	± da	± ppm	Start Seq.	End Seq.	Sequence	Ion Score	C. I. %	Modification	Plate [#] Name	Gel Idx/Pos [4700 Sample Name]	iTRAQ Ratio 115/114*	iTRAQ Ratio 116/114*	iTRAQ Ratio 117/114*	Rank	Result Type
1579.873	1579.8452	-0.0278	-18	61	70	NFIVWLEDQK	30	97.479	(N-term)_iTRAQ[0], Lysine(K)_iTRAQ[10]	[7] F5 120912	439/431	0.415	0.666	0.596	1	Mascot

974	JNK/SAPK-associated protein 1b [Rattus norvegicus]	gi|40714466	12456.2	1	30	1.006	1.000	1.065	0.000	0.000	0.000	1	1	1	97.444

Protein Group

JNK/SAPK-associated protein 1a [Rattus norvegicus]	gi|40714464	11373.6

Peptide Information

Calc. Mass	Obsrv. Mass	± da	± ppm	Start Seq.	End Seq.	Sequence	Ion Score	C. I. %	Modification	Plate [#] Name	Gel Idx/Pos [4700 Sample Name]	iTRAQ Ratio 115/114*	iTRAQ Ratio 116/114*	iTRAQ Ratio 117/114*	Rank	Result Type
1832.9044	1832.8344	-0.07	-38	13	29	MQQVGGGGQTESSLPGR	30	97.444	(N-term)_iTRAQ[0]	[1] F3 030912	167/159	1.006	1.000	1.065	1	Mascot

975	TEGT [Rattus norvegicus]	gi|456209	28647.2	1	30	1.080	0.974	0.833	0.000	0.000	0.000	1	1	1	97.402

Protein Group

Ac1-149 [Rattus norvegicus]	gi|32527701	47144.7
TEGT [Rattus norvegicus]	gi|456207	28778.3
bax inhibitor 1 [Rattus norvegicus]	gi|124248489	28883.3

Peptide Information

Calc. Mass	Obsrv. Mass	± da	± ppm	Start Seq.	End Seq.	Sequence	Ion Score	C. I. %	Modification	Plate [#] Name	Gel Idx/Pos [4700 Sample Name]	iTRAQ Ratio 115/114*	iTRAQ Ratio 116/114*	iTRAQ Ratio 117/114*	Rank	Result Type
1493.9427	1493.7589	-0.1838	-123	6	14	KINFDALLK	30	97.402	(N-term)_iTRAQ[0], Lysine(K)_iTRAQ[1,9]	[5] F4	363/355	1.080	0.974	0.833	1	Mascot

976	40S ribosomal protein S12 [Homo sapiens]	gi|14277700	17277.1	1	30	3.164	1.633	2.245	0.000	0.000	0.000	1	1	1	97.396

Protein Group

RecName: Full=40S ribosomal protein S12	gi|54039306	17241.2
ribosomal protein S12 [Rattus norvegicus]	gi|206741	16696.8

Peptide Information

Calc. Mass	Obsrv. Mass	± da	± ppm	Start Seq.	End Seq.	Sequence	Ion Score	C. I. %	Modification	Plate [#] Name	Gel Idx/Pos [4700 Sample Name]	iTRAQ Ratio 115/114*	iTRAQ Ratio 116/114*	iTRAQ Ratio 117/114*	Rank	Result Type
1330.714	1330.6913	-0.0227	-17	122	129	DVIEEYFK	30	97.396	(N-term)_iTRAQ[0], Lysine(K)_iTRAQ[8]	[7] F5 120912	307/299	3.164	1.633	2.245	1	Mascot

977	makorin ring finger protein 1 [Rattus norvegicus]	gi|51948434	38636.1	1	30	0.942	0.727	0.652	0.000	0.000	0.000	1	1	1	97.354

Peptide Information

Calc. Mass	Obsrv. Mass	± da	± ppm	Start Seq.	End Seq.	Sequence	Ion Score	C. I. %	Modification	Plate [#] Name	Gel Idx/Pos [4700 Sample Name]	iTRAQ Ratio 115/114*	iTRAQ Ratio 116/114*	iTRAQ Ratio 117/114*	Rank	Result Type
2068.0669	2067.9341	-0.1328	-64	198	211	EESEKEPTTVETEK	30	97.354	(N-term)_iTRAQ[0], Lysine(K)_iTRAQ[5,14]	[6] F8 110912	117/109	0.942	0.727	0.652	1	Mascot

978	leucine zipper putative tumor suppressor 1 [Rattus norvegicus]	gi|23618893	75050.6	1	30	1.232	1.443	0.776	0.000	0.000	0.000	1	1	1	97.348

Peptide Information

Calc. Mass	Obsrv. Mass	± da	± ppm	Start Seq.	End Seq.	Sequence	Ion Score	C. I. %	Modification	Plate [#] Name	Gel Idx/Pos [4700 Sample Name]	iTRAQ Ratio 115/114*	iTRAQ Ratio 116/114*	iTRAQ Ratio 117/114*	Rank	Result Type
1612.9395	1612.8942	-0.0453	-28	466	477	VNLLEQELLELR	30	97.348	(N-term)_iTRAQ[0]	[1] F3 030912	498/490	1.232	1.443	0.776	1	Mascot

979	carboxypeptidase E [Rattus norvegicus]	gi|203550	56792.3	1	29	0.773	0.809	0.813	0.000	0.000	0.000	1	1	1	97.139

Protein Group

Carboxypeptidase E [Rattus norvegicus]	gi|55249691	56739.5
RecName: Full=Carboxypeptidase E; Short=CPE; AltName: Full=Carboxypeptidase H; Short=CPH; AltName:	gi|115893	56767.5
carboxypeptidase E (EC 3.4.17.10) [Rattus norvegicus]	gi|203297	56781.5
carboxypeptidase E precursor [Rattus norvegicus]	gi|6978701	56815.5

Peptide Information

Calc. Mass	Obsrv. Mass	± da	± ppm	Start Seq.	End Seq.	Sequence	Ion Score	C. I. %	Modification	Plate [#] Name	Gel Idx/Pos [4700 Sample Name]	iTRAQ Ratio 115/114*	iTRAQ Ratio 116/114*	iTRAQ Ratio 117/114*	Rank	Result Type
1886.0092	1886.0094	0.0002	0	151	164	IHIMPSLNPDGFEK	29	97.139	(N-term)_iTRAQ[0], Lysine(K)_iTRAQ[14]	[4] F7 and F10+11	350/342	0.773	0.809	0.813	1	Mascot

980	cysteine and glycine-rich protein 1 [Rattus norvegicus]	gi|8393206	24748	1	29	1.214	0.549	0.812	0.000	0.000	0.000	1	1	1	97.112

Peptide Information

Calc. Mass	Obsrv. Mass	± da	± ppm	Start	End	Sequence	Ion	C. I. %	Modification	Plate [#] Name	Gel Idx/Pos [4700	iTRAQ	iTRAQ	iTRAQ	Rank	Result Type

				Seq.	Seq.		Score				Sample Name]	Ratio 115/114*	Ratio 116/114*	Ratio 117/114*		
1577.7832	1577.7352	-0.048	-30	179	193	GFGFGQGAGALVHSE	29	97.112	(N-term)_iTRAQ[0]	[1] F3 030912	298/290	1.214	0.549	0.812	1	Mascot

981	RecName: Full=Aspartoacylase; AltName: Full=Aminoacylase-2; Short=ACY-2	gi|122065121	38828.9	1	29	0.668	1.293	0.683	0.000	0.000	0.000	1	1	1	97.099

Peptide Information

Calc. Mass	Obsrv. Mass	± da	± ppm	Start Seq.	End Seq.	Sequence	Ion Score	C. I. %	Modification	Plate [#] Name	Gel Idx/Pos [4700 Sample Name]	iTRAQ Ratio 115/114*	iTRAQ Ratio 116/114*	iTRAQ Ratio 117/114*	Rank	Result Type
1862.0408	1861.9948	-0.046	-25	172	187	YPVGIEVGPQPHGVLR	29	97.099	(N-term)_iTRAQ[0]	[7] F5 120912	256/248	0.668	1.293	0.683	1	Mascot

982	oxidation resistance protein 1 isoform 3 [Rattus norvegicus]	gi|309384246	26975.9	1	29	0.833	0.943	1.384	0.000	0.000	0.000	1	1	1	97.092

Peptide Information

Calc. Mass	Obsrv. Mass	± da	± ppm	Start Seq.	End Seq.	Sequence	Ion Score	C. I. %	Modification	Plate [#] Name	Gel Idx/Pos [4700 Sample Name]	iTRAQ Ratio 115/114*	iTRAQ Ratio 116/114*	iTRAQ Ratio 117/114*	Rank	Result Type
1546.7974	1546.7721	-0.0253	-16	151	160	WTGDNMFFIK	29	97.092	(N-term)_iTRAQ[0], Lysine(K)_iTRAQ[10]	[7] F5 120912	376/368	0.833	0.943	1.384	1	Mascot

983	branched-chain alpha-keto acid dihydrolipoyl acyltransferase [Rattus norvegicus]	gi|12964662	23263.6	1	29	1.038	1.190	1.318	0.000	0.000	0.000	1	1	1	97.052

Peptide Information

Calc. Mass	Obsrv. Mass	± da	± ppm	Start Seq.	End Seq.	Sequence	Ion Score	C. I. %	Modification	Plate [#] Name	Gel Idx/Pos [4700 Sample Name]	iTRAQ Ratio 115/114*	iTRAQ Ratio 116/114*	iTRAQ Ratio 117/114*	Rank	Result Type
1907.1588	1907.0984	-0.0604	-32	125	136	ILKEDILNFLEK	29	97.052	(N-term)_iTRAQ[0], Lysine(K)_iTRAQ[3,12]	[6] F8 110912	501/493	1.038	1.190	1.318	1	Mascot

984	SNERG-1protein [Rattus norvegicus]	gi|8453205	71296.6	1	29	0.879	1.037	1.016	0.000	0.000	0.000	1	1	1	97.024

Protein Group

melanoma-associated antigen D1 [Rattus norvegicus]	gi|16758144	91555

Peptide Information

Calc. Mass	Obsrv. Mass	± da	± ppm	Start Seq.	End Seq.	Sequence	Ion Score	C. I. %	Modification	Plate [#] Name	Gel Idx/Pos [4700 Sample Name]	iTRAQ Ratio 115/114*	iTRAQ Ratio 116/114*	iTRAQ Ratio 117/114*	Rank	Result Type
1842.9919	1842.8588	-0.1331	-72	14	28	IPGADAQTQNVNQAK	29	97.024	(N-term)_iTRAQ[0], Lysine(K)_iTRAQ[15]	[5] F4	95/87	0.879	1.037	1.016	1	Mascot

985	Chain A, Rat Brain 3-Hydroxyacyl-Coa Dehydrogenase Binary Complex With Nadh And Estradiol	gi|14488724	29062.8	1	29	1.747	3.008	2.713	0.000	0.000	0.000	1	1	1	96.962

Protein Group

3-hydroxyacyl-CoA dehydrogenase type-2 [Rattus norvegicus]	gi|13994225	29243.8
Chain A, Rat Brain 3-Hydroxyacyl-Coa Dehydrogenase Binary Complex With Nadh And 3-Keto Butyrate	gi|14488714	29022.7
RecName: Full=3-hydroxyacyl-CoA dehydrogenase type-2; AltName: Full=17-beta-hydroxysteroid dehydrog	gi|7387724	29193.9

Peptide Information

Calc. Mass	Obsrv. Mass	± da	± ppm	Start Seq.	End Seq.	Sequence	Ion Score	C. I. %	Modification	Plate [#] Name	Gel Idx/Pos [4700 Sample Name]	iTRAQ Ratio 115/114*	iTRAQ Ratio 116/114*	iTRAQ Ratio 117/114*	Rank	Result Type
1530.7784	1530.6997	-0.0787	-51	105	115	NQVHTLEDFQR	29	96.962	(N-term)_iTRAQ[0]	[6] F8 110912	202/194	1.747	3.008	2.713	1	Mascot

986	ras-related protein R-Ras2 isoform a [Homo sapiens]	gi|21361416	25586.1	1	29	1.069	1.151	0.778	0.000	0.000	0.000	1	1	1	96.955

Peptide Information

Calc. Mass	Obsrv. Mass	± da	± ppm	Start Seq.	End Seq.	Sequence	Ion Score	C. I. %	Modification	Plate [#] Name	Gel Idx/Pos [4700 Sample Name]	iTRAQ Ratio 115/114*	iTRAQ Ratio 116/114*	iTRAQ Ratio 117/114*	Rank	Result Type
1260.6721	1260.6484	-0.0237	-19	99	106	GSFEEIYK	29	96.955	(N-term)_iTRAQ[0], Lysine(K)_iTRAQ[8]	[3] F6 and F9	227/219	1.069	1.151	0.778	1	Mascot

987	stathmin [Rattus norvegicus]	gi|8393696	20736.4	1	29	1.108	0.828	0.623	0.000	0.000	0.000	1	1	1	96.92

Peptide Information

Calc. Mass	Obsrv. Mass	± da	± ppm	Start Seq.	End Seq.	Sequence	Ion Score	C. I. %	Modification	Plate [#] Name	Gel Idx/Pos [4700 Sample Name]	iTRAQ Ratio 115/114*	iTRAQ Ratio 116/114*	iTRAQ Ratio 117/114*	Rank	Result Type
1532.8557	1532.8159	-0.0398	-26	15	27	ASGQAFELILSPR	29	96.92	(N-term)_iTRAQ[0]	[1] F3 030912	357/349	1.108	0.828	0.623	1	Mascot

988	fumarylacetoacetate hydrolase domain-containing protein 1 [Rattus norvegicus]	gi|68163417	27288.4	1	29	1.776	3.035	1.973	0.000	0.000	0.000	1	1	1	96.913	

Peptide Information

Calc. Mass	Obsrv. Mass	± da	± ppm	Start Seq.	End Seq.	Sequence	Ion Score	C. I. %	Modification	Plate [#] Name	Gel Idx/Pos [4700 Sample Name]	iTRAQ Ratio 115/114*	iTRAQ Ratio 116/114*	iTRAQ Ratio 117/114*	Rank	Result Type
1463.858	1463.8029	-0.0551	-38	135	144	EKIPDPHALR	29	96.913	(N-term)_iTRAQ[0], Lysine(K)_iTRAQ[2]	[2] F12 040912	183/175	1.776	3.035	1.973	1	Mascot

989	interleukin enhancer-binding factor 2 [Rattus norvegicus]	gi|114145744	54984.8	1	29	0.953	1.365	0.964	0.000	0.000	0.000	1	1	1	96.837

Peptide Information

Calc. Mass	Obsrv. Mass	± da	± ppm	Start Seq.	End Seq.	Sequence	Ion Score	C. I. %	Modification	Plate [#] Name	Gel Idx/Pos [4700 Sample Name]	iTRAQ Ratio 115/114*	iTRAQ Ratio 116/114*	iTRAQ Ratio 117/114*	Rank	Result Type
1753.0596	1753.0173	-0.0423	-24	259	269	KLDPELHLDIK	30	97.587	(N-term)_iTRAQ[0], Lysine(K)_iTRAQ[1,11]	[2] F12 040912	305/297	0.953	1.365	0.964	1	Mascot

990	monoacylglycerol lipase ABHD12 [Rattus norvegicus]	gi|66730294	48903.4	1	29	1.619	1.326	1.670	0.000	0.000	0.000	1	1	1	96.812

Peptide Information

Calc. Mass	Obsrv. Mass	± da	± ppm	Start Seq.	End Seq.	Sequence	Ion Score	C. I. %	Modification	Plate [#] Name	Gel Idx/Pos [4700 Sample Name]	iTRAQ Ratio 115/114*	iTRAQ Ratio 116/114*	iTRAQ Ratio 117/114*	Rank	Result Type
1984.0248	1984.0251	0.0003	0	219	232	GMTYDALHVFDWIK	29	96.812	(N-term)_iTRAQ[0], Lysine(K)_iTRAQ[14]	[4] F7 and F10+11	498/490	1.619	1.326	1.670	1	Mascot

991	solute carrier family 39 (zinc transporter), member 7 [Rattus norvegicus]	gi|57114294	51336.1	1	29	0.733	0.633	0.317	0.000	0.000	0.000	1	1	1	96.73

Peptide Information

Calc. Mass	Obsrv. Mass	± da	± ppm	Start Seq.	End Seq.	Sequence	Ion Score	C. I. %	Modification	Plate [#] Name	Gel Idx/Pos [4700 Sample Name]	iTRAQ Ratio 115/114*	iTRAQ Ratio 116/114*	iTRAQ Ratio 117/114*	Rank	Result Type
1913.0352	1913.0891	0.0539	28	297	309	DGPLKPQNPEEEK	29	96.73	(N-term)_iTRAQ[0], Lysine(K)_iTRAQ[5,13]	[3] F6 and F9	1106/1098	0.733	0.633	0.317	1	Mascot

992	calpastatin isoform b [Rattus norvegicus]	gi|76159288	85734.5	1	29	1.021	1.370	1.615	0.000	0.000	0.000	1	1	1	96.599

Peptide Information

Calc. Mass	Obsrv. Mass	± da	± ppm	Start Seq.	End Seq.	Sequence	Ion Score	C. I. %	Modification	Plate [#] Name	Gel Idx/Pos [4700 Sample Name]	iTRAQ Ratio 115/114*	iTRAQ Ratio 116/114*	iTRAQ Ratio 117/114*	Rank	Result Type
2010.0349	2009.9299	-0.105	-52	42	59	KGSDEVTASSAATGTSPR	29	96.599	(N-term)_iTRAQ[0], Lysine(K)_iTRAQ[1]	[6] F8 110912	103/95	1.021	1.370	1.615	1	Mascot

993	EG3RVC [Rattus norvegicus]	gi|32423788	47871.8	1	28	0.859	0.766	0.703	0.000	0.000	0.000	1	1	1	96.373

Protein Group

Sept4 protein [Rattus norvegicus]	gi|56971857	46310.1
septin 4 [Rattus norvegicus]	gi|58865356	39491.6

Peptide Information

Calc. Mass	Obsrv. Mass	± da	± ppm	Start Seq.	End Seq.	Sequence	Ion Score	C. I. %	Modification	Plate [#] Name	Gel Idx/Pos [4700 Sample Name]	iTRAQ Ratio 115/114*	iTRAQ Ratio 116/114*	iTRAQ Ratio 117/114*	Rank	Result Type
1350.7913	1350.7385	-0.0528	-39	83	91	IMQTVEITK	28	96.373	(N-term)_iTRAQ[0], Lysine(K)_iTRAQ[9]	[7] F5 120912	214/206	0.859	0.766	0.703	1	Mascot

994	RecName: Full=Hexaprenyldihydroxybenzoate methyltransferase, mitochondrial; AltName: Full=3,4-dihyd	gi|57015268	41743.4	1	28	0.662	0.929	0.921	0.000	0.000	0.000	1	1	1	96.314

Protein Group

Coq3 protein [Rattus norvegicus]	gi|51980294	41426.3

hexaprenyldihydroxybenzoate methyltransferase, mitochondrial [Rattus norvegicus]	gi|9506505	34926

Peptide Information

Calc. Mass	Obsrv. Mass	± da	± ppm	Start Seq.	End Seq.	Sequence	Ion Score	C. I. %	Modification	Plate [#] Name	Gel Idx/Pos [4700 Sample Name]	iTRAQ Ratio 115/114*	iTRAQ Ratio 116/114*	iTRAQ Ratio 117/114*	Rank	Result Type
2092.0498	2091.9924	-0.0574	-27	99	113	TFQALAHSWWDEQGK	28	96.314	(N-term)_iTRAQ[0], Lysine(K)_iTRAQ[15]	[6] F8 110912	413/405	0.662	0.929	0.921	1	Mascot

995	Psmd1 protein [Rattus norvegicus]	gi|56388811	102690.5	1	28	0.979	0.251	0.759	0.000	0.000	0.000	1	1	1	96.288

Protein Group

26S proteasome non-ATPase regulatory subunit 1 [Rattus norvegicus]	gi|14010879	116739.7

Peptide Information

Calc. Mass	Obsrv. Mass	± da	± ppm	Start Seq.	End Seq.	Sequence	Ion Score	C. I. %	Modification	Plate [#] Name	Gel Idx/Pos [4700 Sample Name]	iTRAQ Ratio 115/114*	iTRAQ Ratio 116/114*	iTRAQ Ratio 117/114*	Rank	Result Type
1669.77	1669.7444	-0.0256	-15	560	572	MEEADALIESLCR	28	96.288	(N-term)_iTRAQ[0], MMTS (C)[12]	[1] F3 030912	542/534	0.979	0.251	0.759	1	Mascot

996	RecName: Full=STE20-like serine/threonine-protein kinase; Short=STE20-like kinase; AltName: Full=ST	gi|81908254	153676.8	1	28	1.427	1.080	1.290	0.000	0.000	0.000	1	1	1	96.068

Peptide Information

Calc. Mass	Obsrv. Mass	± da	± ppm	Start Seq.	End Seq.	Sequence	Ion Score	C. I. %	Modification	Plate [#] Name	Gel Idx/Pos [4700 Sample Name]	iTRAQ Ratio 115/114*	iTRAQ Ratio 116/114*	iTRAQ Ratio 117/114*	Rank	Result Type
1728.0067	1728.0801	0.0734	42	1164	1173	KLQEQEVFFK	28	96.068	(N-term)_iTRAQ[0], Lysine(K)_iTRAQ[1,10]	[4] F7 and F10+11	1158/1150	1.427	1.080	1.290	1	Mascot

997	RecName: Full=Phosphatidylinositol-4-phosphate 5-kinase type-1 gamma; Short=PIP5K1-gamma; Short=Ptd	gi|78099090	81541.2	1	28	0.889	0.797	1.017	0.000	0.000	0.000	1	1	1	95.995

Protein Group

phosphatidylinositol-4-phosphate 5-kinase type-1 beta [Rattus norvegicus]	gi|60678264	66786.9

Peptide Information

Calc. Mass	Obsrv. Mass	± da	± ppm	Start Seq.	End Seq.	Sequence	Ion Score	C. I. %	Modification	Plate [#] Name	Gel Idx/Pos [4700 Sample Name]	iTRAQ Ratio 115/114*	iTRAQ Ratio 116/114*	iTRAQ Ratio 117/114*	Rank	Result Type
1836.9551	1836.9025	-0.0526	-29	201	214	LLPGYYMNLNQNPR	28	95.995	(N-term)_iTRAQ[0]	[1] F3 030912	344/336	0.889	0.797	1.017	1	Mascot

998	transmembrane emp24 domain-containing protein 10 precursor [Rattus norvegicus]	gi|16758214	27573.6	1	28	1.428	1.488	1.816	0.000	0.000	0.000	1	1	1	95.893

Protein Group

transmembrane protein [Rattus norvegicus]	gi|1360136	25993.8

Peptide Information

Calc. Mass	Obsrv. Mass	± da	± ppm	Start Seq.	End Seq.	Sequence	Ion Score	C. I. %	Modification	Plate [#] Name	Gel Idx/Pos [4700 Sample Name]	iTRAQ Ratio 115/114*	iTRAQ Ratio 116/114*	iTRAQ Ratio 117/114*	Rank	Result Type
2318.1948	2318.2129	0.0181	8	153	169	RLEDLSESIVNDFAYMK	28	95.893	(N-term)_iTRAQ[0], Lysine(K)_iTRAQ[17]	[4] F7 and F10+11	495/487	1.428	1.488	1.816	1	Mascot

999	catenin alpha-1 [Rattus norvegicus]	gi|55742755	111389	1	28	1.109	0.907	1.056	0.000	0.000	0.000	1	1	1	95.758

Peptide Information

Calc. Mass	Obsrv. Mass	± da	± ppm	Start Seq.	End Seq.	Sequence	Ion Score	C. I. %	Modification	Plate [#] Name	Gel Idx/Pos [4700 Sample Name]	iTRAQ Ratio 115/114*	iTRAQ Ratio 116/114*	iTRAQ Ratio 117/114*	Rank	Result Type
2236.207	2236.2471	0.0401	18	82	98	ESQFLKEELVAAVDDVR	28	95.758	(N-term)_iTRAQ[0], Lysine(K)_iTRAQ[6]	[4] F7 and F10+11	475/467	1.109	0.907	1.056	1	Mascot

1000	leucine-rich repeat-containing protein 7 [Rattus norvegicus]	gi|16924000	180972.7	1	28	0.718	0.537	1.418	0.000	0.000	0.000	1	1	1	95.758

Peptide Information

Calc. Mass	Obsrv. Mass	± da	± ppm	Start Seq.	End Seq.	Sequence	Ion Score	C. I. %	Modification	Plate [#] Name	Gel Idx/Pos [4700 Sample Name]	iTRAQ Ratio 115/114*	iTRAQ Ratio 116/114*	iTRAQ Ratio 117/114*	Rank	Result Type
1903.9971	1903.8436	-0.1535	-81	601	615	VENANPTANTEQTVK	28	95.758	(N-term)_iTRAQ[0], Lysine(K)_iTRAQ[15]	[5] F4	77/69	0.718	0.537	1.418	1	Mascot

1001 kinesin light chain 1 isoform C [Rattus norvegicus] gi|126517467 72287 1 28 2.528 1.461 0.980 0.000 0.000 0.000 1 1 1 95.758

Peptide Information

Calc. Mass	Obsrv. Mass	± da	± ppm	Start Seq.	End Seq.	Sequence	Ion Score	C. I. %	Modification	Plate [#] Name	Gel Idx/Pos [4700 Sample Name]	iTRAQ Ratio 115/114*	iTRAQ Ratio 116/114*	iTRAQ Ratio 117/114*	Rank	Result Type
1585.7295	1585.6436	-0.0859	-54	355	364	YEEVEYYYQR	28	95.758	(N-term)_iTRAQ[0]	[1] F3 030912	247/239	2.528	1.461	0.980	1	Mascot

1002 dipeptidyl peptidase 3 [Rattus norvegicus] gi|16758578 88352.4 1 28 1.253 1.060 1.206 0.000 0.000 0.000 1 1 1 95.738

Peptide Information

Calc. Mass	Obsrv. Mass	± da	± ppm	Start Seq.	End Seq.	Sequence	Ion Score	C. I. %	Modification	Plate [#] Name	Gel Idx/Pos [4700 Sample Name]	iTRAQ Ratio 115/114*	iTRAQ Ratio 116/114*	iTRAQ Ratio 117/114*	Rank	Result Type
1756.8527	1756.7595	-0.0932	-53	236	248	SYEFQGNHFQVTR	28	95.738	(N-term)_iTRAQ[0]	[6] F8 110912	238/230	1.253	1.060	1.206	1	Mascot

1003 long-chain specific acyl-CoA dehydrogenase, mitochondrial precursor [Rattus norvegicus] gi|6978431 52055.1 1 28 1.259 1.120 0.499 0.000 0.000 0.000 1 1 1 95.738

Peptide Information

Calc. Mass	Obsrv. Mass	± da	± ppm	Start Seq.	End Seq.	Sequence	Ion Score	C. I. %	Modification	Plate [#] Name	Gel Idx/Pos [4700 Sample Name]	iTRAQ Ratio 115/114*	iTRAQ Ratio 116/114*	iTRAQ Ratio 117/114*	Rank	Result Type
1684.8302	1684.7854	-0.0448	-27	255	267	AQDTAELFFEDVR	28	95.738	(N-term)_iTRAQ[0]	[1] F3 030912	383/375	1.259	1.120	0.499	1	Mascot

1004 ribosomal protein L14 [Rattus norvegicus] gi|114145740 45154.1 1 27 0.309 0.719 0.780 0.000 0.000 0.000 1 1 1 95.532

Protein Group

60S ribosomal protein L14 [Rattus norvegicus] gi|12621122 28891.9

Peptide Information

Calc. Mass	Obsrv. Mass	± da	± ppm	Start Seq.	End Seq.	Sequence	Ion Score	C. I. %	Modification	Plate [#] Name	Gel Idx/Pos [4700 Sample Name]	iTRAQ Ratio 115/114*	iTRAQ Ratio 116/114*	iTRAQ Ratio 117/114*	Rank	Result Type
1534.8628	1534.7023	-0.1605	-105	153	164	VAYISFGPHAGK	29	96.591	(N-term)_iTRAQ[0], Lysine(K)_iTRAQ[12]	[3] F6 and F9	1218/1210	0.309	0.719	0.780	1	Mascot

1005 40S ribosomal protein S4, X isoform X isoform [Homo sapiens] gi|4506725 33797.9 1 27 0.698 1.070 0.990 0.000 0.000 0.000 1 1 1 95.517

Protein Group

ribosomal protein S4 gi|227229 33882.9

Peptide Information

Calc. Mass	Obsrv. Mass	± da	± ppm	Start Seq.	End Seq.	Sequence	Ion Score	C. I. %	Modification	Plate [#] Name	Gel Idx/Pos [4700 Sample Name]	iTRAQ Ratio 115/114*	iTRAQ Ratio 116/114*	iTRAQ Ratio 117/114*	Rank	Result Type
1406.8005	1406.7797	-0.0208	-15	40	49	ECLPLIIFLR	27	95.517	(N-term)_iTRAQ[0], MMTS (C)[2]	[1] F3 030912	571/563	0.698	1.070	0.990	1	Mascot

1006 phosphatidylinositide phosphatase SAC1 [Rattus norvegicus] gi|16758640 72309.4 1 27 0.826 0.873 0.679 0.000 0.000 0.000 1 1 1 95.37

Peptide Information

Calc. Mass	Obsrv. Mass	± da	± ppm	Start Seq.	End Seq.	Sequence	Ion Score	C. I. %	Modification	Plate [#] Name	Gel Idx/Pos [4700 Sample Name]	iTRAQ Ratio 115/114*	iTRAQ Ratio 116/114*	iTRAQ Ratio 117/114*	Rank	Result Type
1594.8839	1594.9421	0.0582	36	291	301	HFDSQVIIYGK	27	95.37	(N-term)_iTRAQ[0], Lysine(K)_iTRAQ[11]	[3] F6 and F9	1211/1203	0.826	0.873	0.679	1	Mascot

1007 cAMP-dependent protein kinase type I regulatory subunit [Rattus norvegicus] gi|1661221 30147.7 1 27 1.020 0.874 1.006 0.000 0.000 0.000 1 1 1 95.36

Protein Group

cAMP-dependent protein kinase type I-alpha regulatory subunit [Rattus norvegicus] gi|6981396 46422.2

Peptide Information

Calc. Mass	Obsrv. Mass	± da	± ppm	Start Seq.	End Seq.	Sequence	Ion Score	C. I. %	Modification	Plate [#] Name	Gel Idx/Pos [4700 Sample Name]	iTRAQ Ratio 115/114*	iTRAQ Ratio 116/114*	iTRAQ Ratio 117/114*	Rank	Result Type
1762.9949	1762.9175	-0.0774	-44	122	133	VSILESLDKWER	27	95.36	(N-term)_iTRAQ[0], Lysine(K)_iTRAQ[9]	[6] F8 110912	425/417	1.020	0.874	1.006	1	Mascot

1008	myotubularin-related protein 9 [Rattus norvegicus]	gi|54114991	67852.7	1	27	0.621	0.832	0.601	0.000	0.000	0.000	1	1	1	95.13

Protein Group

myotubularin-related protein [Rattus norvegicus]	gi|22653222	61869.7

Peptide Information

Calc. Mass	Obsrv. Mass	± da	± ppm	Start Seq.	End Seq.	Sequence	Ion Score	C. I. %	Modification	Plate [#] Name	Gel Idx/Pos [4700 Sample Name]	iTRAQ Ratio 115/114*	iTRAQ Ratio 116/114*	iTRAQ Ratio 117/114*	Rank	Result Type
1805.8479	1805.7468	-0.1011	-56	249	262	GGGFEQEAHYPQWR	27	95.13	(N-term)_iTRAQ[0]	[6] F8 110912	253/245	0.621	0.832	0.601	1	Mascot

1009	ATP synthase-coupling factor 6, mitochondrial precursor [Rattus norvegicus]	gi|16758388	14215.8	1	27	1.112	1.319	1.070	0.000	0.000	0.000	1	1	1	95.005

Peptide Information

Calc. Mass	Obsrv. Mass	± da	± ppm	Start Seq.	End Seq.	Sequence	Ion Score	C. I. %	Modification	Plate [#] Name	Gel Idx/Pos [4700 Sample Name]	iTRAQ Ratio 115/114*	iTRAQ Ratio 116/114*	iTRAQ Ratio 117/114*	Rank	Result Type
2234.1174	2234.0681	-0.0493	-22	85	99	GEMDKFPTFNFEDPK	27	95.005	(N-term)_iTRAQ[0], Lysine(K)_iTRAQ[5,15]	[6] F8 110912	363/355	1.112	1.319	1.070	1	Mascot
